# Transcriptional co-expression and co-regulation of genes coding for components of the oxidative phosphorylation system

**DOI:** 10.1186/1471-2164-9-18

**Published:** 2008-01-14

**Authors:** Corina van Waveren, Carlos T Moraes

**Affiliations:** 1 Department of Cell Biology and Anatomy, University of Miami Miller School of Medicine, Miami, FL, USA; 2 Department of Neurology, University of Miami Miller School of Medicine, Miami, FL, USA

## Abstract

**Background:**

The mitochondrial oxidative phosphorylation (OXPHOS) is critical for energy (ATP) production in eukaryotic cells. It was previously shown that genes coding for mitochondrial proteins involved in energy production co-express at the RNA level. Because the OXPHOS enzymes are multimeric complexes, we tested the hypothesis that genes coding for components of specific complexes are also co-regulated at the transcriptional level, and share common regulatory elements in their promoters.

**Results:**

We observed for the first time that, not only OXPHOS genes as a group co-express, but there is a co-expression of genes within each of the five OXPHOS enzyme complexes, showing a higher degree of complexity in gene co-regulation. *In silico *analysis of homologous promoter sequences in mammals identified the likely core promoter elements for most genes encoding OXPHOS subunits/assembly factors. The results included a significant abundance of previously identified sites (e.g. NRF1, NRF2, ERRA and YY1), as well as several sites that had not been previously detected. Although we identified patterns that correlated to OXPHOS gene expression, we did not detect an OXPHOS complex-specific arrangement of transcription factor binding sites within the core promoter that could explain the tight co-expression of these functionally related genes.

**Conclusion:**

This study mapped the core promoters of most OXPHOS related genes and provided an example of gene expression regulation based on the final protein arrangement within a linear metabolic pathway.

## Background

**Figure 1:**
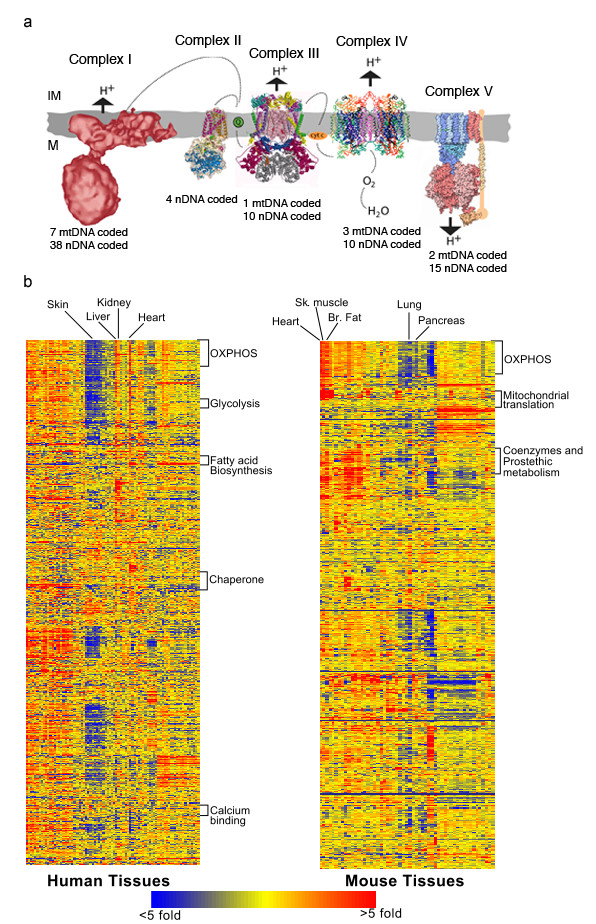
**Co-expression of nuclear-coded mitochondrial genes**. (a) Schematic diagram of the OXPHOS system showing the complexity of its subunit composition. IM: mitochondrial intermembrane space; M: mitochondrial matrix. (b) mRNA expression profiles for 1290 human and 1029 mouse mitochondrial compiled genes (rows) across 79 human (left) and 53 (right) mouse tissues (columns). Genes and tissues were hierarchically clustered and visualized using Genespring. Selected tissues are labeled (top). Clusters enriched in genes belonging to the same functional classification are labeled between brackets.

The oxidative phosphorylation system (OXPHOS) is responsible for 90% of adenosine triphosphate (ATP) production in a respiring cell. Five multi-subunit complexes, the respiratory chain complexes, and two additional electron carriers, coenzyme Q10 and cytochrome c, participate in OXPHOS to generate ATP [1]. The OXPHOS complexes consist of proteins encoded by both the nuclear (n = approximately 100 in humans) and the mitochondrial DNA (n = 13). Complex I is apparently made up of 38 nuclear coded and 7 mtDNA coded subunits, 4 nDNA coded subunits make up mature complex II, complex III contains 11 subunits one of which is mtDNA coded, complex IV contains 13 subunits, 3 are mtDNA coded and complex V is made up of 2 mtDNA coded and 15 nDNA coded subunits (Figure 1A). In addition, several nuclear-coded assembly factors that are not part of the mature complex and coded by the nDNA, have been shown to be necessary for the proper assembly and function of the OXPHOS system. Consequently, the correct function of the respiratory chain depends on an orchestrated crosstalk between the two genomes [2-4].

Microarray data have been widely used to study gene co-expression by studying genes with similar expression patterns across a set of samples. Over the past few years, several lines of evidence suggest that co-expressed genes (which display high correlation values (CV) amongst different expression experiments) are likely to encode proteins that participate in the same metabolic pathway, form a common structural complex, or might be regulated by the same mechanism [5,6].

In eukaryotes, the regulatory mechanisms underlying co-regulation of multiple genes is extremely complex [7]. At the mRNA level, co-expression of OXPHOS genes has become evident from recent studies of wide transcriptome analysis across and within different species. In humans, large-scale analysis across different tissues (Shyamsundar et al. 2005) and across multiple datasets and conditions [8] have revealed a co-expression cluster significantly enriched in OXPHOS genes. In mouse, co-expression of OXPHOS genes across different tissues has been also described [9]. Lastly, two macroevolutionary studies have also observed that several biological functional groups were repeatedly identified as co-expressed over large evolutionary distances and a wide variety of conditions. One of these clusters was significantly enriched in OXPHOS genes [10,11]. Hence, it became apparent that a significant number of genes involved in the energy generation pathway, and in particular OXPHOS, not only share the same metabolic pathway (ATP synthesis) and interact at a protein-level, but also share tight co-expression at the mRNA level within and across different conditions and organisms. This suggests that these genes might have a common regulatory mechanism that accounts for this striking pattern of co-expression.

Energetic demands vary substantially between different cells and tissues of an organism. For example, in mammals, the mitochondrial content of cardiac myocytes or brown adipose cells is very high compared to skeletal muscle fibers type IIb. In general, the energy demand of a specific tissue correlates with the level of expression of genes encoding components of the OXPHOS system [12]. The mechanisms controlling this nucleo-mitochondrial communication are just starting to emerge.

There are diverse regulatory mechanisms that may underlie co-expression. However, most studies have been devoted to the identification of proteins that regulate the transcription of nuclear-encoded mitochondrial genes as well as factors regulating mitochondrial transcription. Recent evidence points to both transcription factors (TFs) and transcriptional coactivators as important players in the regulation of mitochondrial biogenesis. DNA-binding TFs including nuclear-respiratory factor 1 and 2 (NRF1, NRF2) estrogen related receptor alpha (ERRA), Sp1, ying yang 1 (YY1), CREB and E-box factors have all been implicated in the expression of some nuclear-coded respiratory chain subunits, among other genes [13-15]. Transcriptional coactivator peroxisome proliferator-activated receptor gamma coactivator-1 (PGC-1α) and related family members (PGC-1β and PRC) are coactivators known to interact with NRF1, NRF2 and ERRA stimulating the expression of several OXPHOS genes [16-20]. PGC-1α was identified as a coactivator involved in mitochondrial gene regulation during adaptive thermogenesis in brown adipose tissue [21]. PGC-1α is mainly expressed in tissues with high energy demand and high mitochondrial content, such as heart, brain, kidney and brown fat, and is induced by fasting and exposure to cold [21-25]. Overexpression of PGC-1α induces the expression of a vast amount of genes participating in mitochondrial metabolism including the TFs ERRA and NRF2 [14,26]. On the other hand, PGC-1β was shown to regulate the expression of various OXPHOS genes as well as NRF1 and ERRA [27]. Therefore, PGC-1α and PGC-1β could play a role as master regulators of the respiratory capacity of a cell either by physically interacting with ERRA, NRF1 and NRF2 at the promoters of OXPHOS-related genes or by increasing the expression of these transcription factors. However, while these TFs and coactivators directly regulate OXPHOS genes, it is unclear how they are integrated in response to environmental cues.

The main conclusion we can draw from the above is that the expression of OXPHOS genes can be modulated at the transcriptional level. There is mounting evidence to show that TFs and coactivators, particularly PGC-1α, are able to cooperatively alter the expression of OXPHOS genes. Although OXPHOS gene expression can be regulated at different levels, gene expression is likely to be a major determinant for coordinated expression. Therefore, understanding the complete set of TFs and coactivators that regulate OXPHOS gene expression should yield insights into the control of OXPHOS gene expression regulation. Although significant progress has been made in this respect, a complete list of factors regulating the expression of all OXPHOS genes remains incomplete, and how these regulators are integrated under physiological conditions is poorly understood.

In this study, OXPHOS gene co-expression across different tissues in humans and mice was confirmed in an independent large microarray dataset. Detailed analyses showed that subunits of individual complexes co-express preferentially among each other than with subunits of other complexes. In addition, phylogenetic footprinting across human, mouse and rat, helped to define each OXPHOS gene core promoter, and resulted in a comprehensive list of factors that could participate in their transcriptional control.

## Results

### Most OXPHOS genes are co-expressed across humans and mouse normal tissues

From various genome wide expression studies, a significant group of OXPHOS genes appears to be co-expressed under various physiological conditions in several species. To identify which particular OXPHOS genes are co-expressed in different conditions/species, we obtained the complete microarray dataset of human and mouse from the Genomics Institute of the Novartis Research Foundation (GNF) tissue compendium. Using custom-designed whole-genome gene expression arrays from panels of mRNAs derived from 79 human and 61 mouse tissues and cell types performed in duplicates, this compendium evaluates the relative expression of 44,775 human and 36,182 mouse transcripts [28]. Analyzing all transcripts would incorporate significant noise to the analysis, thus data was subjected to global normalization and filtered for a compilation of mitochondria-related genes that contains all mitochondrial transcripts coding for mitochondrial proteins, as well as transcripts coding for OXPHOS related factors such as those involved in oxidative stress response, fatty acid biosynthesis and glycolysis (see methods).

In order to study co-expression of OXPHOS genes among all the human and mouse "mitochondrial genelists", an unsupervised hierarchical cluster analysis was performed. This approach was used to visualize relationships among the expression patterns of OXPHOS genes and other groups of genes. Figure 1b shows the result of the hierarchical clustering analysis using Pearson correlation similarity measure of the filtered genes. Genes are displayed on the horizontal axis and tissues on the vertical axis. The nodes that have genes that are enriched in a particular biological pathway are represented on the right side of each tree. The ordered list of genes based on Figure 1b is described on Additional file 1.

In both mice and humans, the hierarchical tree cluster analysis resulted in a cluster of genes enriched in OXPHOS genes. These results confirm previous observations that a significant number of OXPHOS genes share a common pattern of gene expression across different tissues and this co-expression is conserved across different species. Other gene groups that were significantly co-expressed among different tissues correspond to genes coding for fatty acid biosynthesis pathway, oxidative stress response, mitochondrial translation machinery, mitochondrial chaperone activity, glycolysis and calcium binding. Tissue clustering showed that tissues with high or low expression of OXPHOS genes clustered together. Tissues with known high energy consumption such as heart, skeletal muscle, kidney and liver displayed increased expression of OXPHOS genes compared to tissues with lower energy demand such as skin and lung. This pattern of expression was observed for other mitochondrial genes as well, which suggests that tissues with high energy demand have not only an increase in the expression of energy generating genes but also of other mitochondrial components. These proteins may be necessary to structurally and metabolically sustain increased energy production.

### Subunits of each complex tend to have tighter co-expression with subunits of the same complex than with subunits of other complexes

**Figure 2:**
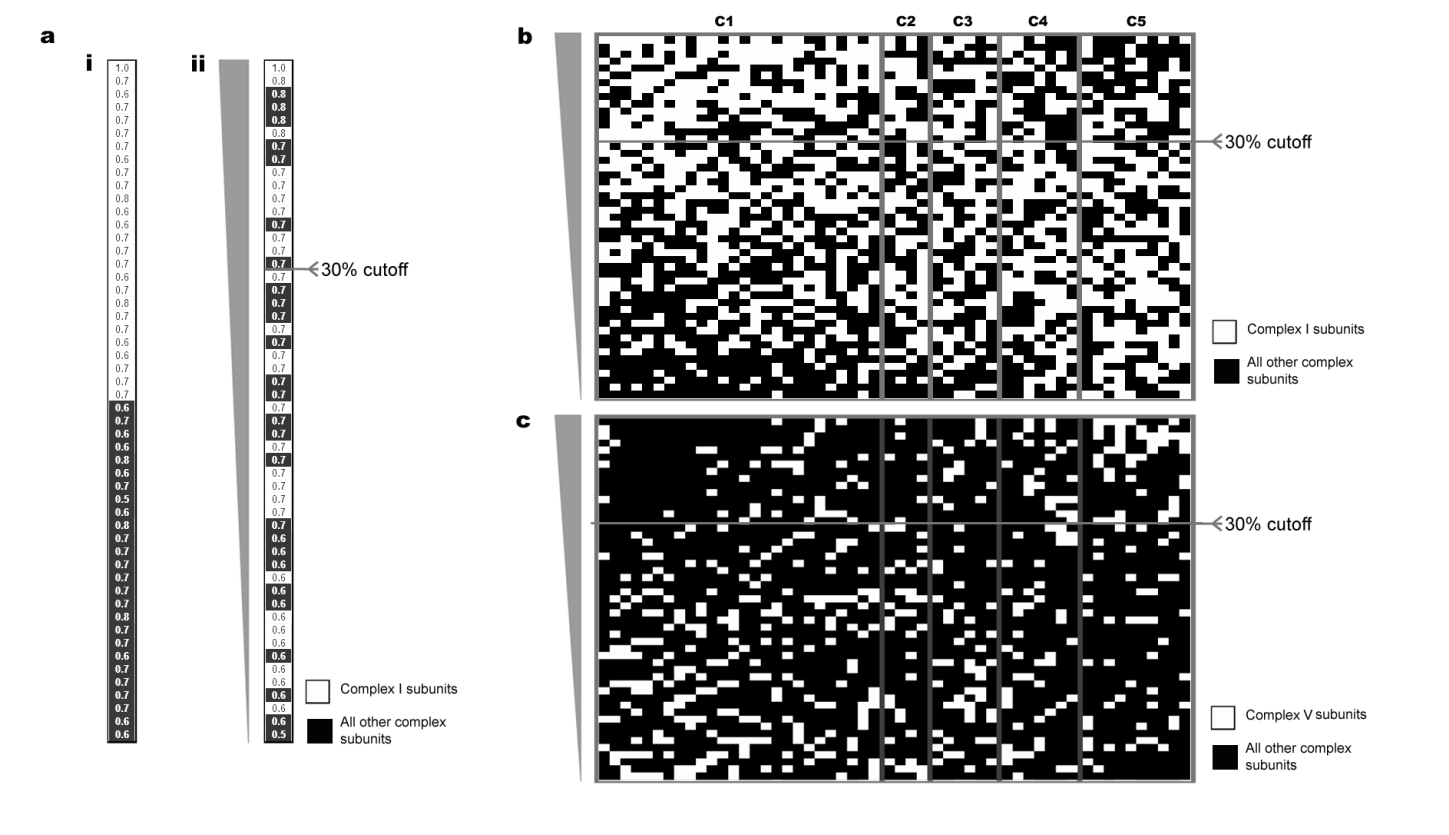
**Preferential co-expression of genes belonging to the same OXPHOS complex**. (a) (i) Pearson correlation values (PCV) were calculated between the expression patterns of each OXPHOS subunit against all the others. In this example, the PCV for complex I subunits are coded white and the rest in black. For simplicity, the PCV of one complex I subunit (vertical) against all the others OXPHOS subunits are shown. (ii) Next, for each subunit (on the vertical axis), the genes were vertically ordered by their PCV values. A 30% cutoff was selected to calculate the percentage of Complex I subunits (in this example) over all other subunits within this cutoff. In this case, 35% of genes in the 30% cutoff are complex I subunits. (b) Schematic representation of the vertically ordered PCV for all OXPHOS subunits coded for each complex I subunit (each vertical line). The same approach was repeated for all other OXPHOS subunits. Notice, the top left quadrant (30% most similar genes to complex I subunits) there is a higher likelihood of finding complex I subunits (white) than subunits belonging to any other complex (black). (c) The same analysis but in this case for complex V subunits. See table 1 for a statistical analysis of these diagrams. These diagrams were constructed using data from the mouse GNF tissue compendium. CI, complex I genes; CII, complex II genes; CIII, complex III genes; CIV, complex IV genes, CV, complex V genes.

The above study suggests that a significant number of OXPHOS subunits co-express among different tissues in both mouse and humans. Next, we studied if particular members of each respiratory complex co-express among each other preferentially over subunits/assembly factors of other OXPHOS complexes. To address this issue, an "OXPHOS correlation test" was designed (please refer to the methods section for a full explanation of this test). Briefly, a similarity matrix was created for all OXPHOS genes (Figure 2a). Next, the correlation values were ordered vertically and assigned to each complex (shaded in Figure 2aii). A cutoff of the 30% highest ranking genes was set. Next, for each gene (columns in Figure 2b), the percentage of factors belonging to each complex within the cutoff was calculated. Lastly, a Student's T-test was performed to test the null hypothesis that 'each gene has the same percentage of complex "X" factors within the cutoff'. Figure 2b shows a graphical view in which genes coding for complex I subunits (CI, top left), have more genes with high correlation values belonging to complex I (white squares) than any other complex. The same positive correlation can be seen for complex V subunits (Figure 2c, top right). This method is somehow similar to the "mitochondrial neighborhood" index used by Mootha and colleagues [29]. The similarity matrix is presented on Additional file 2.

Table 1 shows the summary of the results obtained for all the four datasets. Although the results for each dataset varied slightly, overall subunits of complex I and V co-express preferentially with themselves. For all complexes, the average of the percentage of subunits from a complex was higher within genes of the same complex than genes of other complexes. In addition, there seems to be a trend in which subunits of complex II and IV also behave in a similar manner (in most cases, the complex II or IV subunits had a higher average percentage of subunits from their own complex within the cutoff than in subunits from any other complex (asterisk on Table 1). However, these trends did not reach statistical significance because of the few genes representing these complexes (for example, complex II is only represented by 3–4 subunits). Therefore, to bypass this limitation, we grouped the results obtained from all the datasets and performed the statistical test on the combined data. In this way, we obtained significant evidence that genes coding for a particular OXPHOS complex co-express preferentially with genes of the same complex. Statistical significance was not reached for complex III, although a trend was observed.

**Table 1:** Co-expression of genes belonging to the same OXPHOS complex.

		**CI**	**CII**	**CIII**	**CIV**	**CV**	30% of genes	Total number of genes
Mouse GNF [28]	*T-test p-value*	**3.206E-08**	0.27071	0.17001	0.15739	**0.00218**		
	*Complex*	32%*	33%*	43%*	36%*	43%		
	*All the rest*	20%	20%	32%	27%	26%		
	*Number of genes*	26	3	6	7	10	22	74

Mouse U74A GNF [42]	*T-test p-value*	**0.04179**	0.13052	0.55088	**0.02878**	0.59082		
	*Complex*	33%	52%*	21%	42%*	25%		
	*All the rest*	29%	32%	23%	25%	23%		
	*Number of genes*	24	4	7	6	10	16	67

Human GNF [28]	*T-test p-value*	**0.01723**	0.30656	0.57973	0.71118	**0.00054**		
	*Complex*	27%	20%*	36%*	30%	58%		
	*All the rest*	24%	13%	33%	29%	37%		
	*Number of genes*	41	4	8	13	16	25	107

Human Stanford [43]	*T-test p-value*	0.07585	0.90503	0.24156	0.80409	**0.02637**		
	*Complex*	23%*	17%*	36%*	27%*	61%		
	*All the rest*	20%	15%	27%	25%	41%		
	*Number of genes*	30	3	8	6	10	17	74

All Datasets	*T-test p-value*	**1.980E-07**	**0.03427**	0.18684	**0.02169**	**1.01E-05**		
	*Complex*	28%	31%	34%	33%	48%		
	*All the rest*	23%	19%	29%	27%	33%		
	*Number of genes*	121	15	29	32	46	43	43

We observed a co-expression for genes within the same complex at a tissue level. The table shows the results of the OXPHOS correlation test for different mouse and human tissue microarray datasets. 'All datasets' represent the results obtained by grouping all the datasets. T-test p-values represent the probability that genes within each complex do not preferentially co-express with genes of its same complex. 'Complex' percentage values represent the average percentage of genes of each complex that have expression similarities within 30% cutoff to genes in the complex labeled above. 'ALL the rest' represent the average percentage of genes within each complex that have expression patterns within the first 30% similarity in genes of any other complex. The 'Number of genes' represent the number of genes representing that complex in each microarray dataset. Asterisks, show cases in which the statistical test did not reach the significant cutoff but showed a trend of higher average percentages in subunits of the same complex than all the rest.

This analysis showed that although OXPHOS subunits co-express among themselves within and across species, gene members coding for each multimeric complex also co-express preferentially with members of the same complex. This novel observation suggests that there is a higher degree of complexity in OXPHOS gene regulation than had been so far detected.

### Promoter Analysis of OXPHOS Genes

From the above study it is apparent that OXPHOS genes co-express at a tissue level. This raises the hypothesis that these genes have a common co-regulatory mechanism that allows co-expression among different tissues. Previous studies suggest that transcriptional initiation is the most common form of gene regulation of OXPHOS genes [30]. However, a comprehensive study of transcriptional regulation of OXPHOS genes has not been performed until now. Most studies concentrate on the characterization of one or a few number of OXPHOS gene promoters and are limited to only one species. Therefore, in this study, an exhaustive analysis of all OXPHOS gene promoters in human, mouse and rat was performed. Promoter analysis was performed for all known nuclear coded OXPHOS structural subunits and assembly factors, as well as 130 randomly selected mitochondrial genes. All available promoter sequences from human, mouse and rat were extracted for all genes analyzed. These species were chosen because their genomes are divergent enough for phylogenetic footprinting analysis to be performed, as they still display high sequence conservation and are the best annotated. An example of phylogenetic footprinting is described on the Additional file 3. For this study, core promoter regions were defined as DNA sequences 500 bp upstream and downstream of the transcription start site (TSS). Analyzing sequences that were larger than 500 bp around the TSS resulted in a significant amount of noise related to promoters of neighboring genes. In addition, previous studies suggest that TFBS which are close to the TSS (~300 bp), are more likely to participate in transcription initiation [31,32]. The complete phylogenetic footprinting alignments of the different OXPHOS polypeptides, highlighting the conserved regions, can be found in additional files (Core Promoter Additional files 4, 5, 6, 7, 8).

Respiration is a vital process in essentially every eukaryotic cell, thus OXPHOS genes are considered housekeeping genes and are expressed in all cell types. In vertebrates, CpG islands are usually associated with several housekeeping gene promoters. We tested for the presence of CpG islands in all human OXPHOS subunit promoters. Overall, CpG islands are present in the promoter regions of 83 out of the 101 human OXPHOS genes analyzed. Since during the course of evolution GC dinucleotides are usually lost, this analysis suggests that there is a high evolutionary pressure for sequence conservation of OXPHOS promoter sequences, and these conserved stretches of DNA probably reflect important transcriptional regulatory elements, and possible nucleosome positioning that would contribute to transcriptional regulation [33].

A hallmark of several OXPHOS promoters studied until now is the absence of a typical TATA box adjacent to the TSS. These promoters are called TATA-less [13]. The TATA box is recognized by the constitutive transcription factor TBP (TATA box binding protein). Therefore, in order to analyze all OXPHOS promoters for the presence of a TATA box, all homologous promoters were aligned using DiAlignTF (Genomatix) and searched for conserved TBP binding sites within 100 bp of the TSS. Only 23% of OXPHOS promoters have conserved TBP binding sites in this region. These results confirm and extend previous observations that nuclear coded OXPHOS gene promoters are mostly TATA-less.

### OXPHOS genes have common TF binding sites in their promoters

From our studies as well as others previously published, the fact that OXPHOS genes are coregulated is clear. Therefore, with the assumption that functionally important TFBS are conserved across evolution, searching for conserved TFBS in OXPHOS promoters would probably result in important information on the molecular basis of OXPHOS transcriptional regulation.

To search for conserved TFBS, phylogenetic footprinting was performed on human, mouse and rat orthologous promoters for all OXPHOS genes. Using DialignTF (Genomatix, DE), orthologue promoter sequences were aligned and strings of short-bp similarity (typically found in TFBS) were searched against a database of known TFBS. The most abundant TFBS families identified belong to TFs previously linked to transcription of some OXPHOS genes: ETSF (Human and murine ETS1 factors) which includes NRF2, SP1F (GC-Box factors SP1/GC), NRF1, EREF (Estrogen response elements) which includes ERRA TFBS, CREB (cAMP-responsive element binding proteins) and YY1F (Activator/repressor binding to transcription initiation site). We also identified TFs known to regulate housekeeping genes, such as EGRF (EGR/nerve growth factor induced protein C & related factors), ZBPF (Zinc binding protein factors) and EBOX (E-box binding factors) among others. Please refer to supplementary material (Additional file 9) for a complete list of results. ETSF belongs to the ETS family of transcription factors and participates in the transcriptional regulation of a myriad of genes [34]. NRF2 is a member of the ETS family, however to differentiate true NRF2 binding sites in the promoters of OXPHOS genes, NRF2 was analyzed also as an independent TFBS. NRF1 and ERRA, as previously described, have been implicated in the regulation of transcription of various mitochondrial genes among others. SP1 is a ubiquitous transcription factor expressed in almost every cell. It has been shown to be necessary for the transcription of some TATA-less promoters and is an important component of the eukaryotic cellular transcriptional machinery [35,36]. In human promoters, SP1 and EBOX transcription factor binding sites are recognized by the transcription factors NF-Y, SP1, and USF which are known to be constitutive TFs participating in basal gene transcription [31]. Our promoter analysis identified a high percentage of promoters with these TFBS conserved in OXPHOS genes. Since OXPHOS genes are housekeeping genes, these TFBS might have a role in basal transcription initiation. EGRF belongs to the family of TFs involved in the transcription of immediate-early gene products expressed in response to diverse stimuli [37]. YY1 is also a ubiquitous TF that has been implicated in the transcription of TATA-less promoters and regulates the transcriptional initiation of several mitochondrial genes [36]. The transcriptional repressor, ZBPF, binds elements found predominantly in genes that participate in lipid metabolism as well as several genes involved in processes related to energy metabolism and vascular disease [38]. CREB family of TFBS belongs to the cAMP response element (CRE) (recognized by CREB, ATF1, FOS/JUN and ATF2/JUN heterodimers), which are widely distributed TF families and known to regulate the expression of various unrelated genes. However, OXPHOS genes have been shown to be regulated by CREB under certain physiological conditions [30,39]. Therefore, an enrichment of this family of TFBS among OXPHOS subunits is not surprising.

NRF1, NRF2, YY1 and ERRA correspond to TFs that have been previously linked to the expression of some OXPHOS genes. To compare the data obtained by the *in silico *approach used in this study with experimentally verified TFBS, we searched for studies in which functional analysis of these TFBS were performed in OXPHOS promoters. In many cases, less stringent DialignTF parameters identified sites that showed a functional effect on transcription in the literature. For this reason, and only for this comparison, all promoters were analyzed using less stringent parameters ('SEL' mode in Additional file 9). 23 out of 27 experimentally verified sites found in the literature could be predicted by our *in *silico analysis showing a high degree of concordance between both methods (see Additional file 10). This analysis showed that the *in silico *approach used in this study is able to identify functional TFBS studied *in vivo*.

**Figure 3:**
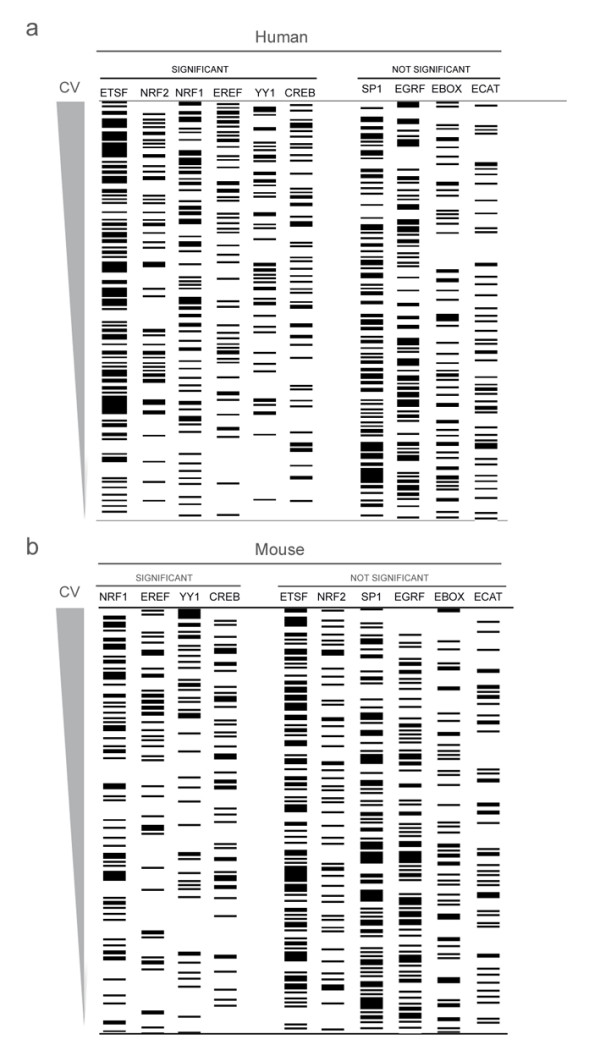
**TFBS enriched in OXPHOS gene promoters**. All genes, whose promoters were analyzed, were ordered by Pearson's correlation similarity to the expression pattern of an 'artificial gene' (the median of the expression pattern of OXPHOS genes) (see methods). Each gene was annotated for the presence of a particular TFBS in its promoter (black horizontal lines). A nonparametric statistics test was used to test if genes that contained a particular TFBS in their promoter ranked higher in the list. Statistical significance (p < 0.05) of each TFBS family is shown at the bottom (marked as significant or not significant). Statistical significance suggests an association between that TFBS and OXPHOS expression pattern. Human (top) and mouse (bottom) GNF datasets were used.

In order to test if the most abundant TFBS identified are enriched in promoters of OXPHOS genes over other promoters, we tested if the presence of TFBSs correlated with co-expressed OXPHOS genes. As a premise, we assumed that co-expression of genes should correlate with the presence of particular TFBS in their promoter. For this analysis, phylogenetic footprinting results from all OXPHOS subunits as well as other randomly selected mitochondrial genes participating in various metabolic processes were used. A nonparametric test was designed to check if genes with a particular conserved TFBS have a higher probability of having an 'OXPHOS expression pattern'. A p-value less than 0.05 would suggest an association between that particular TFBS and OXPHOS expression pattern (see methods). NRF1, ERRA, YY1 and CREB TFBSs showed a statistically significant enrichment in OXPHOS co-expressing gene promoters in both human and mouse tissues (Figure 3). A previous study, usinga different approach, identified ERRA and NRF2 as a regulator of OXPHOS genes through PGC-1α. In our study, NRF2 TFBS seemed to correlate with OXPHOS co-expressing genes in human tissues but did not reach statistical significance in mouse tissues using the datasets chosen (Figure 3). Mostly, the TFBSs identified in our study are associated to TFs that have previously been reported in promoters of some OXPHOS subunits [30]. Furthermore, these TFBS might play a role in the differential expression of OXPHOS genes across different tissues.

Overall, these results show that phylogenetic footprinting resulted in the detection of several TFBS that are enriched in OXPHOS gene promoters. Some TFBS that displayed a high abundance in OXPHOS gene promoters, like SP1F, did not show an association with OXPHOS gene expression, which suggests that they might participate in non-specialized basal transcriptional initiation of these genes. The vast majority of the OXPHOS promoters analyzed have not been characterized previously. Therefore, these results confirm that the unbiased computational approach chosen does lead to biologically relevant information.

### Conserved TFBS Show Positional Bias around the TSS

A fundamental question in a bioinformatics approach to promoter analysis is to determine which of the predicted TFBSs are biologically relevant. In the above study, the presence of a TFBS conserved across human, mouse and rat was taken as a guide to answer this question. In addition, the relative position of each conserved TFBS to the corresponding TSS can also be analyzed. Previous studies have relied on this approach, and found that TFBS that cluster relative to the TSS (i.e. with a positional bias) have a high likelihood of being biologically significant [31].

**Figure 4:**
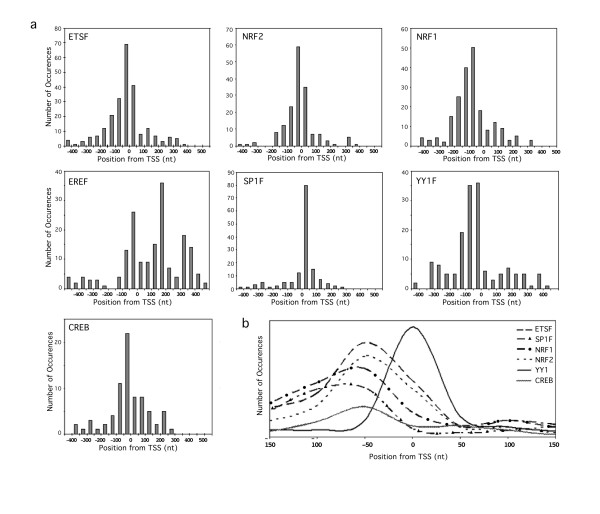
**TFBS display a clear positional bias within the TSS**. The relative position of each conserved TFBS from the TSS was determined. (a) Histograms of the most relevant TFBS families. These graphs represent the number of occurrences of each TFBS identified in a 50 bp bin relative to the TSS (position 0). (b) Overlapping Frequency Distribution Curves for each TFBS Relative to the TSS which show that each TFBS clusters in slightly different positions.

Following this idea, the positional bias of each TFBS relative to the TSS was recorded. Although the analysis covered 500 bp surrounding the TSS (500 bp upstream and downstream), the conserved TFBS identified by phylogenetic footprinting preferentially occur within ~150 bases of the TSS in all three organisms, consistent with the likelihood of these TFBS being involved in transcription initiation (Figure 4). Previous studies performed in a whole genome approach have shown that NRF1, ETS, NRF2, SP1, EBOX, ECAT and CREB TFBS cluster close to the promoter region in humans [31]. ERRA TFBS seemed to be present preferentially downstream of the TSS in OXPHOS gene promoters. In addition, they tend to cluster around 200 and 350 bp downstream of the TSS. Previous studies had not been able to detect a positional bias for ERRA TFBS [40]. Few conserved TFBS were observed outside 300 bp around the TSS, confirming that a 500 bp around the TSS has been a reasonable cutoff to obtain biologically relevant results with low noise.

Taking a closer look around the TSS it became apparent that YY1F TFBS are preferentially conserved at the TSS or around 10 bp of it, suggesting a strong role in transcription initiation of OXPHOS genes (Figure 4b). On the other hand, NRF1, NRF2 and ETSF and SP1F TFBSs cluster slightly upstream of the TSS (~50–70 bp upstream of the TSS). Each TFBS clusters in slightly different positions within 150 bp upstream of the TSS. This raises the possibility that these factors function together in transcriptional regulation.

In summary, there seems to be a clear positional bias around the TSS for the most abundant TFBS identified by phylogenetic footprinting. In particular, known OXPHOS related TFs, NRF1, NRF2 and YY1F (except ERRA) all cluster within ~150 bp of the TSS. This suggests that most conserved TFBS identified for OXPHOS promoters are probably transcriptionally functional in humans, mouse and rat, and raises the possibility that TFBS identified outside of this region might not be functional for the promoters of these ubiquitously expressed genes studied. These results further support the biological relevance of our *in silico *approach.

### TFBS Orientation on OXPHOS promoters

**Figure 5:**
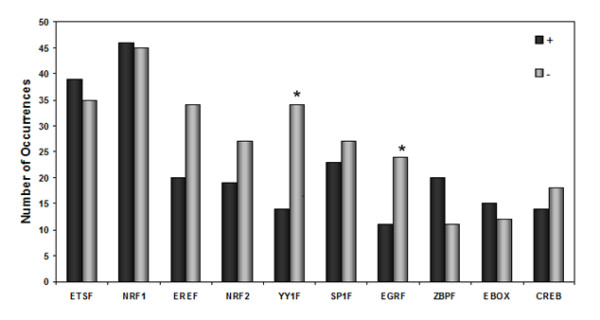
**Relative orientation of conserved TFBS**. Relative orientation of the conserved TFBS to the promoters analyzed using Genomatix library of PWM. "+" denotes binding to the positive strand, "-" denotes binding to the negative strand with respect to transcription. Asterisks denote statistically significant differences of TFBS orientation (p ≤ 0.05).

From previous studies, most TFs do not have a preferred orientation of binding in order to play a role in transcription initiation. To test this in OXPHOS promoters, TFBS orientation was recorded for all conserved TFBS detected in all three species. YY1F and EGRF families of TFBS showed a statistically significant (p < 0.05) bias towards one strand of DNA (Figure 5). Particularly, YY1F presented a clear bias to bind the negative strand. Since YY1F TFBS also presents a strong positional bias to the TSS (~50 nucleotides around the TSS), these results suggest that YY1F TFBSs might play an important role in TSS determination of these TATA-less promoters as well as in transcriptional initiation. ERRA and NRF2 TFBSs also seem to have a preferred orientation; however, they did not reach statistical significance. A strand bias for the two latter factors has previously been reported for mitochondrial genes [14].

## Discussion

The coordinate regulation of the expression of gene groups can occur at different levels, such as transcription [14] or translation [41,42]. In this study, we focused on the coordinated regulation of mRNA levels of genes related to OXPHOS function. Our results clearly show the power of *in silico *approach to define promoter regions and to study gene co-expression and co-regulation at a transcriptional level. Combining high-throughput gene expression data with promoter analysis has enabled the detection of sites that are recognized by unique or families of TFs which might play an important role in the co-expression of OXPHOS genes. Although previous studies have shown the co-expression and defined TFBS for genes coding for mitochondrial proteins [14,26], in this study we focused on OXPHOS-related genes.

From our gene expression studies, we were able to confirm co-expression of OXPHOS genes at the mRNA level. OXPHOS genes are known as housekeeping genes, and it would be expected that they are transcribed at similar levels in all cell types. However, there is a clear difference in relative mRNA abundance of a significant number of these genes, mostly related to the energy requirements of each tissue. Although differences in the number, size and protein content of mitochondria from different tissues is a recognized aspect in the field, differences in mRNA levels is a relatively recent discovery. These findings suggest that there must be an intricate mechanism of co-regulation of these genes at the mRNA level.

An unexpected finding was the significant tendency for genes within the OXPHOS complexes to co-express among each other than with genes of other complexes. This suggests that there is a coordinated control in the levels of mRNAs of genes coding for each complex. It is well established that there is a tendency for genes participating in the same metabolic process, or interacting physically, to co-express [43]. Therefore, these results suggest that, besides a master regulator of OXPHOS gene expression driving their co-expression at a tissue level, there is an underlying fine tuning mechanism regulating the expression of OXPHOS genes within each complex. In our studies, we did not detect a complex-specific pattern of TFBSs. Future studies will need to be designed to identify the mechanisms that are responsible for co-expression of genes within OXPHOS complexes. Although this may be due to the low power of statistical analyses (few conserved TFBS per gene promoter), it is possible that this "fine tuning" is orchestrated by cis elements relatively distant from the promoters or by mRNA degradation.

Previous studies have identified TFBS for NRF1, NRF2, ERRA, and YY1 in the promoters of some OXPHOS subunits [14,30]. Although only a few of OXPHOS genes have previously been characterized for the functional role of these TFBS (Additional file 10), these studies correlate extremely well with the predicted TFBS identified by our *in silico *approach. In this study, human, mouse and rat promoters of all known OXPHOS and accessory subunits were analyzed, whilst usually published data concentrates in the characterization of one promoter in one species. Therefore, this study constitutes the first comprehensive analysis of all OXPHOS gene promoters, making a good case for unbiased computational approach resulting in biologically relevant data.

Interestingly, statistical analysis of the most abundant TFBS identified showed that NRF1, EREF, YY1F and CREB correlate significantly with OXPHOS gene expression pattern. These factors may act independently or synergistically in the transcriptional regulation of OXPHOS genes. The presence of multiple factors may serve to integrate diverse signals into mitochondrial biogenesis. In the mitochondrial field, these TFs were assumed to be master regulators of mitochondrial gene transcription [30]. The results obtained in this study extend this concept to a broader level. Many of these TFs are not specific for OXPHOS-related genes (e.g. YY1F, CREB) and are also common in the promoters associated with other metabolic networks, but other are more commonly found in OXPHOS-related genes (e.g. NRF1, NRF2). Therefore, although the co-expression of genes coding for mitochondrial proteins can be explained by the presence of TFBS for TFs such as NRF1, NRF2 and ERRA, both the specific combination of TFs as well as other events controlling mRNA levels (e.g. coactivators, RNA degradation and long range cis effects) may ultimately explain the co-regulation.

From analyzing the positional bias around the TSS for the factors identified, it became apparent that most of the conserved TFBS identified cluster in specific positions close to the TSS. This suggests that the TFBS identified by phylogenetic footprinting are likely to be biologically functional if present within these clusters.

## Conclusion

We showed that the coordinated expression of known OXPHOS genes goes beyond a mitochondrial or even OXPHOS pattern, to the level of individual complexes. This finding implies that either common promoter elements or a feedback mechanism from the assembled complexes influences the levels of mRNAs Although the identification of the core promoters and their conserved TFBS of 98 OXPHOS genes provided the initial clues to understand this process, further work will be required to fully define the precise mechanisms responsible for this co-expression.

## Methods

### Microarray Data Handling

The human and mouse expression atlas was obtained from GNF [44,28]. This atlas contains custom-designed whole-genome gene expression arrays from mRNAs derived from 79 human and 61 mouse tissues and cell types performed in duplicates and evaluates the expression of 44,775 human and 36,182 mouse transcripts. Data was analyzed using Genespring microarray analysis software package (Agilent Technologies, Palo Alto, CA). To control for chip-wide variations in intensity, each array was normalized to the 50^th ^percentile of genes. In addition, to control for the differences in detection efficiency between spots, each probeset (the collection of probes designed to interrogate a given gene sequence) was normalized to its median across all chips or arrays. These normalization steps enabled comparisons of relative change in gene expression levels between experiments. Duplicate arrays were then averaged and considered as one.

Data was filtered for mitochondrial genes. A complete list of human mitochondrial genes was obtained from two online compilations, which contain data obtained by several proteomic approaches in an effort to obtain the complete list of mitochondrial genes. MitoRES [45] provided 808 genes and MitoProteome [46] which is a mitochondrial protein database generated from experimental evidence such as mass spectrometry and other public databases provided 869 genes [47]. Data was grouped excluding repetitions. For mouse, a complete list of mitochondrial genes was obtained from mitoRES (732 genes). The mitochondrial genes were compiled with genes belonging to "mitochondrial related pathways" which were not present in the lists such as glycolysis, fatty acid biosynthesis and oxidative stress genes. The resulting compilation contains 1290 probesets in the human and 1029 probesets in the mouse array datasets. Of these compilations, 147 represent human and 111 represent mouse OXPHOS genes, which code for both structural as well as accessory subunits of the respiratoy chain. Both human and mouse lists of mitochondrial probesets were used in expression profile analysis and were called "mitochondrial compilation".

Hierarchical clustering analysis was performed on the "mitochondrial compilation" genelists using Pearson correlation similarity. Genespring software package was used to create a graphical view of the data.

For the OXPHOS correlation test (explained bellow), two more datasets were used. The U74A mouse tissue compendium from GNF [48] and the human compilation of normal tissues available at the Stanford MicroArray Database [49,50].

### OXPHOS correlation test

To investigate if OXPHOS subunits co-express preferentially with subunits of the same complex than with subunits of other complexes, a "correlation similarity" test was designed. For each microarray dataset, a similarity matrix was made. Pearson's correlation was used to measure the pair-wise similarity between the expression profiles among all OXPHOS genes in the matrix (Figure 2). If one gene was represented by more than one probeset, the probeset with the highest average correlation values (CV) was included in the analysis and the rest was discarded. Next, for each OXPHOS gene (in a column), all other genes were rank ordered on the basis of their Pearson Correlation Value (CV). A cutoff of 30% of the highest ranking CV was set (Figure 2ii). The percentage of complex X subunits within the cutoff was calculated for each gene. Then, the null hypothesis that 'any gene has the same percentage of the different complexes subunits within the cutoff' was tested using the Student's t-Test. This analysis was performed for the two mouse and two human tissue atlases mentioned above.

Because some complexes have very few nuclear coded genes, the above analysis did not pass our statistical test for complexes with few subunits (complex II, complex III, and complex IV). To bypass this restriction, we grouped the results of the OXPHOS correlation test for all probesets from the four datasets before applying the statistical test.

### Promoter Sequence Extraction and Analysis

Genomatix [51] and the UCSC Genome Browser [52] human genome May 2004 build were used to extract 1000 bp DNA sequence around the transcriptional start site (TSS as annotated by RefSeq) for human, mouse and rat genomes (500 bp upstream and 500 bp downstream from the TSS). These 1000 bp were considered to contain the core promoters. In cases where more than one gene promoter was present for a given genome, the promoters conserved across the three species by ElDorado (Genomatix) were chosen for further analysis. For the sequences of the promoters analyzed in this study, see supplementary data (Additional files 4, 5, 6, 7, 8). In genomes where there was no annotation for a specific gene, BLAT search tool was used to locate its correspondent EST using an orthologous mRNA sequence for that gene. In most cases, this approach could identify the gene in the queried genome and enabled the promoter extraction. All OXPHOS genes and other arbitrarily selected mitochondrial genes (n = 235) were analyzed by phylogenetic footprinting.

The presence of CpG islands was assessed by the UCSC Genome Browser built-in application [53]. The algorithm considers segments of 200 bp of DNA and evaluates them for a 50% or greater content of GC. All human promoter sequences were visually inspected in the browser for the presence of CpG islands.

### Alignment of Orthologous Promoters

Orthologous promoter sequences from human, mouse and rat were aligned using DiAlignTF (Genomatix, Germany). This application is able to align and identify conserved TFBSs in orthologous promoters. DiAlignTF is a DNA alignment software that constructs alignments from gap-free pairs of similar segments of sequences. Therefore, the program is especially suited to detect short local similarities, characteristic of short TFBS, in otherwise completely unrelated sequences. DialignTF was used with its default parameters using the Genomatix Matrix Family Library Version 5.0 (January 2005) and the orthologous promoters for each gene. This alignment is often called phylogenetic footprinting. For this analysis, 98 OXPHOS orthologous promoters (human, mouse and rat) as well as 134 other gene promoters participating in several mitochondrial pathways were examined.

Because several transcription factors bind similar TFBSs, Genomatix groups these into TFBS families. For example, NRF2 binding site is similar to other ETS family members, and therefore, ETSF (ETS family) contains NRF2 binding site. Since NRF2 has been implicated in the expression of some OXPHOS subunits, for this analysis, promoters were analyzed for the presence of both ETSF (family) and NRF2 (single factor) binding sites. On the other hand, NRF1 has its unique TFBS and therefore is not part of any family of TFBS.

Conserved families of TFBSs in all orthologue promoters were retrieved. The presence of at least one copy of a conserved TFBS family in the aligned promoter sequences was examined. The presence of more than one copy of the same TFBS family was not recorded. Binding sites for the transcription factor families V$SF1F, V$RORA and V$ERER (Genomatix nomenclature) are very similar and therefore often overlap [54]. V$ERER family of transcription factors contains the ERRA binding site, thus only this family will be considered for this study since ERRA has previously been associated with OXPHOS gene transcription. All alignment results obtained are available in the supplementary material (Additional files 4, 5, 6, 7, 8).

Human, mouse and rat promoters were used to search all OXPHOS promoters for the presence of a TATA box. If a TATA box protein binding site (V$TBPF) was found to be conserved within 100 bp of the TSS of the aligned promoters, it was considered as a likely functional TATA box. If no V$TBPF binding site was present the genes were considered as being TATA-less [30].

### Statistical Analysis of transcription factor binding sites (TFBS)

To test if TFBSs identified by phylogenetic footprinting are enriched in OXPHOS gene promoters over random mitochondrial gene promoters, a statistical test was designed. This test was performed with all promoters analyzed by phylogenetic footprinting using DialignTF default parameters (n = 235). An ordered list of all genes was generated using the Pearson correlation to the expression pattern of the average expression pattern of OXPHOS genes (the median of the expression patterns of OXPHOS genes that clustered in the hierarchical gene tree (Figure 2)). This resulted in a similarity ordered list of genes where the most similar values correspond to genes whose expression pattern was most similar to OXPHOS subunits. Each gene was then annotated for the presence of each TFBS in its promoter (Additional file 11). The data was then subjected to a nonparametric Wilcoxon Rank-Sum test for two independent samples to assess, for each TFBS, whether genes that contain the TFBS tend to rank high on the list (i.e. has an expression pattern similar to OXPHOS genes). A p-value less than 0.05 was considered as a significant association. This analysis was performed for the human and the mouse GNF tissue atlas [28]

## Positional and Binding bias of each TFBS

To examine if conserved TFBSs cluster at a specific position from the TSS, the relative position to the TSS of each conserved TFBS identified by phylogenetic footprinting was documented for human, mouse and rat. All OXPHOS promoters and mitochondrial related promoters analyzed by phylogenetic footprinting were included in this analysis.

To study if there was a bias towards the TFBS orientation, the relative orientation (with respect to the direction of transcription) of each conserved TFBS analyzed above was determined. A nonparametric Sign test was used to determine if there was a preference of TF binding orientation. TFBS orientation depends on the Genomatix library of position weight matrices (PWM).

## Abbreviations

ATP: Adenosine-5'-triphosphate

ChIP: chromatin immunoprecipitation

COX: Cytochrome c Oxidase (a.k.a. Complex IV)

CREB: cAMP response element binding protein

CV: Pearson's correlation value

cyt c: Cytochrome c

ERRA: Estrogen related receptor alpha

ETC: electron transport chain

GNF: Genomics Institute of the Novartis Research Foundation

HIF: hypoxia inducible factor

KCN: potassium cyanide

MELAS: Mitochondrial myopathy Encephalopathy Lactic acidosis and Stroke-like episodes

miRNA: micro RNA

mRNA: messenger RNA

mtDNA: Mitochondrial DNA

nDNA: nuclear DNA

NRF1: Nuclear respiratory factor 1

NRF2: nuclear respiratory factor 2

OXPHOS: : Oxidative Phosphorylation System

PGC-1: Peroxisome-proliferator activated gamma coactivator-1

ROS : reactive oxygen species

RT-PCR: reverse transcriptase polymerase chain reaction

SDH: succinate dehydrogenase

TCA cycle: tricarboxylic acid cycle

TF: Transcription factor

TFAM: mitochondrial transcription factor A

TFBS: Transcription factor binding site

tRNA: transfer RNA

TSS: transcription start site

YY1: ying yang 1

OXPHOS: Oxidative Phosphorylation System

## Authors' contributions

CvW designed, executed, performed the statistical analysis and wrote the manuscript. CTM designed and wrote the manuscript. Both authors read and approved the final manuscript.

## Supplementary Material

Additional file 1 Ordered list of genes used for mRNA profile in figure 1b. Sheets 'Human' and 'Mouse' contain the ordered list of genes based on Figure 1b. Each row includes the corresponding GNF probe-set ID, the gene symbol, and its classification (based on enrichment of genes of a particular function). Click here for file

Additional file 2 OXPHOS mRNA correlation test. The accompanying tables correspond to the similarity matrix used to perform the OXPHOS correlation test. The Pearson's correlation value (CV) for each pair of genes is shown. The values shown are a measure of the pair-wise similarity between the expression profiles among each pair of OXPHOS gene in the matrix. Click here for file

Additional file 3 Example of phylogenetic footprinting. The figure shows how the phylogenetic footprinting approach is performed for the COX 6A gene. Click here for file

Additional file 4 Phylogenetic footprinting of Complex I core promoters. Core Promoters of Complex I subunits/related genes identified by phylogenetic footprinting. The conserved regions are shown in color codes, which correspond to specific Transcription Factor Binding Sites (TFBS). Click here for file

Additional file 5 Phylogenetic footprinting of Complex II core promoters. Core Promoters of Complex II subunits/related genes identified by phylogenetic footprinting. The conserved regions are shown in color codes, which correspond to specific Transcription Factor Binding Sites (TFBS). Click here for file

Additional file 6 Phylogenetic footprinting of Complex III core promoters. Core Promoters of Complex III subunits/related genes identified by phylogenetic footprinting. The conserved regions are shown in color codes, which correspond to specific Transcription Factor Binding Sites (TFBS). Click here for file

Additional file 7 Phylogenetic footprinting of Complex IV core promoters. Core Promoters of Complex IV subunits/related genes identified by phylogenetic footprinting. The conserved regions are shown in color codes, which correspond to specific Transcription Factor Binding Sites (TFBS). Click here for file

Additional file 8 Phylogenetic footprinting of Complex V core promoters. Core Promoters of Complex V subunits/related genes identified by phylogenetic footprinting. The conserved regions are shown in color codes, which correspond to specific Transcription Factor Binding Sites (TFBS). Click here for file

Additional file 9 Phylogenetic footprinting of promoters. Phylogenetic footprinting results of all promoters analyzed obtained by DialignTF (Genomatix, DE). Sheet "conserved TFBS" contains the list of all promoters analyzes with the conserved TFBS obtained using the default paramenters of DialignTF. Sheet "Summary" contains a Summary of the data obtained using default parameters of DialignTF. Sheet "conserved TFBS SEL" contains data obtained using DialignTF in default and SEL parameters. Sheet "SEL" indicates the TFBS selected and the parameters used for the analysis in SEL mode. Sheet "Matrix Family Library" contains all Genomatix Matrix families (TFBS families) used in this study. Click here for file

Additional file 10 Prediction of core promoters vs. experimentally identified. The table compares promoter elements identified experimentally (left column) with the predicted in this study (right column). Click here for file

Additional file 11 Data used to perform the statistical analysis of TFBS abundance. These spreadsheets show the data used to perform the statistical analysis of TFBS abundance. Human and Mouse GNF microarray data were used to obtain the Pearson correlation values (CV) of each gene to an OXPHOS average gene (the median of the expression patterns of OXPHOS genes that clustered in the hierarchical gene tree). Genes with a higher CV value are those whose expression pattern was most similar to OXPHOS subunits. Each gene is also annotated for the presence of each TFBS in its promoter. Click here for file
