# Supplementary figures and images for: Transcriptional co-expression and co-regulation of genes coding for components of the oxidative phosphorylation system
Source: BMC Genomics. 2008 Jan 14;9:18. doi: 10.1186/1471-2164-9-18 (PMC2268925; doi:10.1186/1471-2164-9-18)

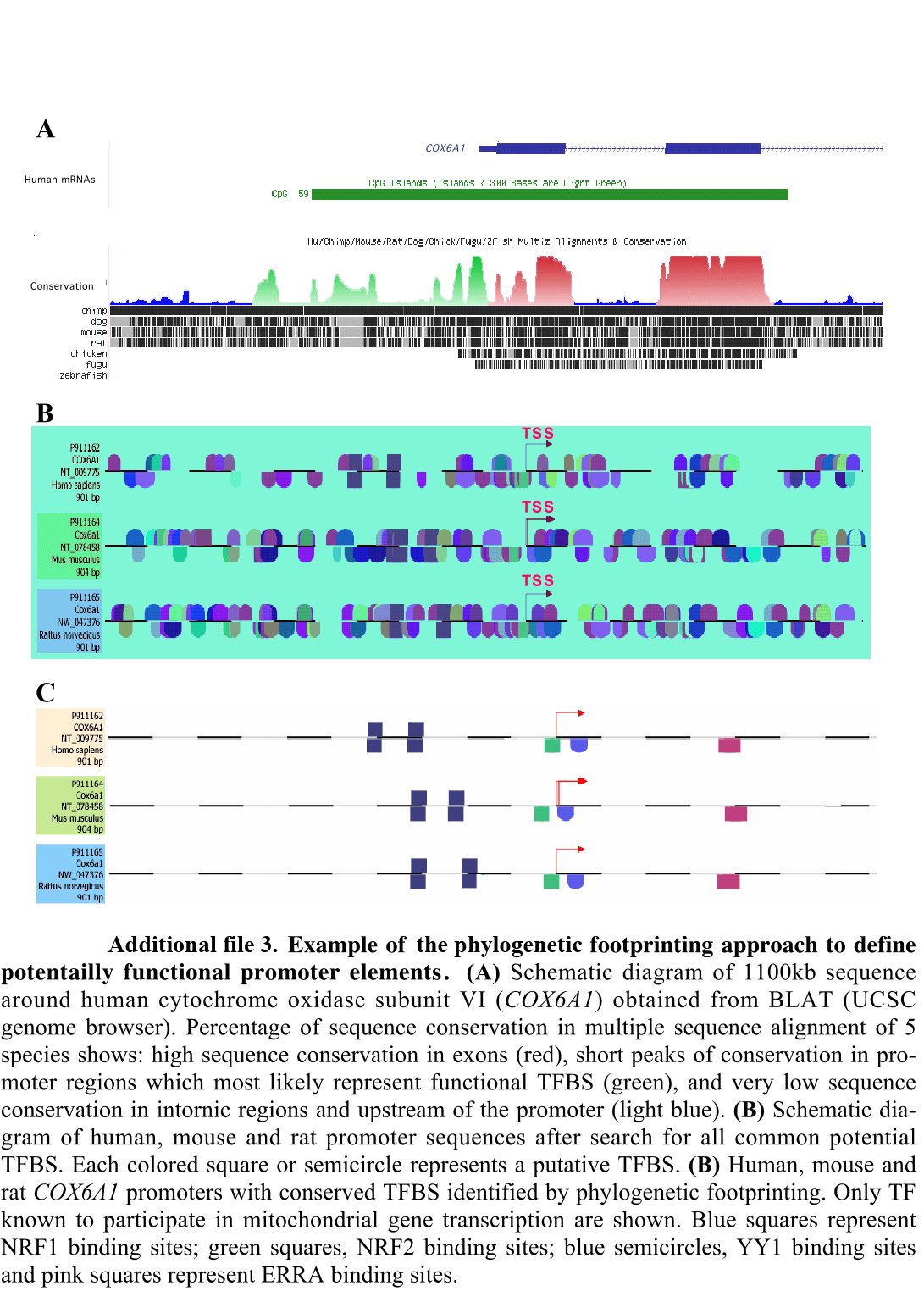

Supplement: Additional file 3 — Example of phylogenetic footprinting. The figure shows how the phylogenetic footprinting approach is performed for the COX 6A gene. [file 1471-2164-9-18-S3.TIFF]
