# Supplementary material for: Transcriptional co-expression and co-regulation of genes coding for components of the oxidative phosphorylation system
Source: BMC Genomics. 2008 Jan 14;9:18. doi: 10.1186/1471-2164-9-18 (PMC2268925; doi:10.1186/1471-2164-9-18)
Supplement: Additional file 4 — Phylogenetic footprinting of Complex I core promoters. Core Promoters of Complex I subunits/related genes identified by phylogenetic footprinting. The conserved regions are shown in color codes, which correspond to specific Transcription Factor Binding Sites (TFBS). [file 1471-2164-9-18-S4.pdf]

This Supplementary Material shows the Core Promoters of Complex I subunits/related genes identified by phylogenetic footprinting. The conserved regions are shown in color codes, which correspond to specific Transcription Factor Binding Sites (TFBS).

The description of the complete description of TFBS can be found in additional file 3 (in the “Matrix Family Library” Excell sheet).

GEMS Launcher Task: *DiAlign TF*: Multiple alignment plus TF sites
working on NDUFA1_HMR.seq (3 seq.)

[Alignment] [Pairwise similarities]

DiAlign professional TF Release 3.1 December 2004 **Mon Mar 14 20:25:14 2005**

Solution parameters:

**Sequence file:**	NDUFA1_HMR.seq (3 seq.)
**Type of sequences:**	nucleotide sequences
**Threshold T:**	0.00
**'*' signs below alignment denote:**	diagonal similarity (max. similarity is represented by 10 '*' signs)
**Alignment output:**	complete alignment is shown<br># nucleic acids per line: 50
**TF output:**	common TF matches located in aligned regions (common to 3 (100.0 %) sequences)
**Family matches:**	yes
**MatInspector library:**	Matrix Family Library Version 5.0 (February 2005)
**Selected groups** (core/matrix sim):	• ALL user_defined.lib (0.75/Optimized)<br>• ALL vertebrates.lib (0.75/Optimized)

Aligned Sequences:

No.	Sequence Name	Sequence Description	Length
1	P914042	P914042 sym=NDUFA1|loc=Loc4694|taxid=9606|spec=Homo sapiens|chr=X|ctg=NT_011786|str=(+)|start=3272907|end=3273632|len=726|tss=538|comm=NADH dehydrogenase (ubiquinone) 1 alpha subcomplex, 1, 7.5kDa; (NM_004541/538/bronze;)	726 bp
2	P914043	P914043 sym=Ndufa1|loc=Loc54405|taxid=10090|spec=Mus musculus|chr=X|ctg=NT_039702|str=(-)|start=4319612|end=4320330|len=719|tss=599,619|comm=NADH dehydrogenase (ubiquinone) 1 alpha subcomplex, 1; (AK007840/619/gold;NM_019443/599/bronze;)	719 bp
3	P914044	P914044 sym=na|loc=Loc363441|taxid=10116|spec=Rattus norvegicus|chr=X|ctg=NW_048033|str=(+)|start=100444|end=101164|len=721|tss=501|comm=similar to NADH dehydrogenase; (XM_343760/501/bronze;)	721 bp

Alignment (DiAlign format):

Please note that only **upper-case** letters are considered to be **aligned**. For more information, please have a look at the user guide.

V$VMYB V$SMAD V$FKHD V$EBOX V$HESF V$MYT1 V$PAX2 V$CREB V$ZBPF V$SP1F V$GLIF V$EGRF
V$HAML

```
alignment position 1.........   11.........   21.........   31.........   41.........
P914042     1  tct---TTCT  CTGTGTCCAG  CTCATAGACA  GCTGTCGCTC  CCATATCCTC
P914043     1  -------TCT  CAGTGTCCAG  TTCGTAGACG  GCGGTCGCCC  CCATATCCTC
P914044     1  cgttccTTCT  CAGTGTCCAG  TTCATAGACA  GCGGTCGCCC  CCATATCtTC
```

```
                               ****  **********  **********  **********  **********
                               ****  **********  **********  **********  **********
                               ****  **********  **********  **********  **********
                                ***  **********  **********  **********  **********
                                ***  **********  **********  **********  ******* **
                                ***  **********  **********  **********  ******* **
                                ***  **********  **********  **********  ******* **
                                ***  **********  **********  ******** *  ******  **
                                ***  **********  **********  ******** *  ******  **
                                                                      *  ******

alignment position       51........  61........  71........  81........  91........
P914042          48  TGGTCCCACG  GGTTTCGCCG  AACGGGTGGA  TTTATAAACC  ACGCCGAGAC
P914043          44  GGGCCCCACG  GGTTTTGCCG  AACGGGTAGA  CTTGTAGACG  ACGCCGAGAC
P914044          51  GGGCCCTACG  GGTTTTGCTG  AACGGGTAGA  CTTGTAGgtC  ACGCCAAGAC
                     **********  **********  **********  **********  **********
                     **********  **********  **********  **********  **********
                     **********  **********  **********  *******  *  **********
                     **********  **********  **********  *******     **********
                     **********  **********  **********  *******     **********
                     **********  **********  **********  *******     **********
                     **********  **********  ******* **  ******
                     **********  **********  *******
                     **********  **********  *******

alignment position       101.......  111.......  121.......  131.......  141.......
P914042          98  TCTCGGCTC   ATTTTCctct  tccTCTTCGC  TGCTCAAGTC  GCCGTAAGCC
P914043          94  CCTCCGGTTC  ATTCTCG---  ---TCCTCGC  TACTCAAGCC  GCCATAGGTT
P914044         101  CCTCAGGTTC  ATTCTCA---  ---TCCTCAC  TACTCAAACC  GCCGTAGTTT
                     **********  *******        *******  **********  **********
                     **********  *******        *******  **********  **********
                     **********  *******        *******  **********  ******
                     **********  ******         *******  ******
                     **********  ******         *******  ******
                     **********  ******

alignment position       151.......  161.......  171.......  181.......  191.......
P914042         148  GCCTTCTGTT  TACCACTGTC  ACGGGTCTTC  TGTATCATTG  GATTGTGGGT
P914043         138  GCCTTCTGTT  TAGCGCTGCC  ACGCGTCTTC  TGGATCATCG  GATTGTGGGT
P914044         145  GtCTTCTGTT  TAGCGCTGCC  ACGCGTCTTC  TGTATCATCG  GGTTGTGGGT
                     **********  **********  **********  **********  **********
                     **********  **********  **********  **********  **********
                       ********  **********  **********  **********  **********
                       ********  **********  **********  **********  **********
                       ********  **********  **********  **********  **********
                       ********  **********  **********  **********  * ********
                       ********  **********  **********  **********  * ********
                       ********  **   *****  **********  ******** *  *
                       ********  **   *****  **********  ******** *  *

alignment position       201.......  211.......  221.......  231.......  241.......
P914042         198  CACCCGCTTC  TTTTCCGGTC  GAACCACAGT  GCAGCCTTCG  TCGCTACTGC
P914043         188  CGCTTGCTTC  TTCTCCGGGC  GGACCACAGA  GCTGCCTTCA  TCACTGCTGC
P914044         195  AGCCTGCTTC  TTCTCGGGGC  GGACCACAGc  GCTGCCTTCG  TCACTGCTGC
                     **********  **********  **********  **********  **********
                     **********  **********  **********  **********  **********
                     **********  **********  *********   **********  **********
                     **********  **********  *********   **********  **********
                     **********  **********  *********   **********  **********
                     *    *****  **********  *********   **********  **********
                     *    *****  **********  *********   **********  *****
                                                         **********  *****

alignment position       251.......  261.......  271.......  281.......  291.......
P914042         248  TGCCGCTTTC  TCCGGGCTCT  GGGTCGCAGG  CCGGGCGCTT  TCTGCGTCCA
P914043         238  TGCAGCTGTC  CCCGGAGCCT  GCATCACAGA  GCCGGCGCTT  TCGGCGGCCC
P914044         245  TGCCGCTGTC  CCCGGACCCc  t CATCACAGA  GCGGGCGCTT  TCGGCGGCCA
```

```
                       ********** ********** ********** ********** **********
                       ********** *********   ********* ********** **********
                       ********** *********   ********* ********** **********
                       ********** *********   ********* ********** **********
                       ********** *****         *****    ********* **********
                       ********** *****

alignment position     301....... 311....... 321....... 331....... 341.......
P914042            298 GCAGCCCCTT TCCGCCCAGG CTTTTTGAAA AGGAAGGTGC ACACCTGAtc
P914043            288 GCCGCTCCTT TCCGCCCAGG CTTTTTGAAG AGGAACGTGC ACACCTGACG
P914044            295 GCCGtTCCTT TCCGGCCAGG CTTTTTGAAG AGAAAAGTGC ACACCTtACC
                       ********** ********** ********** ********** **********
                       **** ***** ********** ********** ********** **********
                       **** ***** ********** ********** ********** **********
                       **** ***** ********** ********** ********** ********
                       **   ***** ********** ********** ********** ****** *
                             **** ********** ********** ********** ****** *
                             **** ********** ********** ********** ****** *
                             **** ********** ********** ********** *****
                               ** ********** ********** ********** *****
                               ** ********** ********** ********** *****

alignment position     351....... 361....... 371....... 381....... 391.......
P914042            348 caccgccTTT CCTGGAGAAA GCTGCTCTGC CA-TTttAGA GTCCTGAGCT
P914043            338 GGTAGAATTC CCGGGAGAAA GTTGCTCCGC CAtTTAGAGA TCCCCAAGTT
P914044            345 GGTAGAATTC CCTGGAGAAA GTTGCTCCGC CA-TTAGAGA TCCCCAAGTT
                       ********** ********** ********** ** ******* **********
                       ********** ********** ********** ** ******* **********
                       ********** ********** ********** ** ******* **********
                              *** ********** ********** ** **  *** **********
                              *** ********** ********** ** **
                              *** ********** ********** **

alignment position     401....... 411....... 421....... 431....... 441.......
P914042            397 CCGAAACAGC CGTGGCTGCC TGGgttgcac ttgGCCCCTG CACGTCACTC
P914043            388 CTGAAACTGC CGCGACGTCC GGGACC---- ---GCTTCGC GTCGTCACAC
P914044            394 CTGAAACTGC TGTGACCTAC GGGACC---- ---GCTTCCG GTCGTCACAC
                       ********** ********** ******        ******* **********
                       **********    ******* ******        ******* **********
                       **********    ******* ***
                       **********

alignment position     451....... 461....... 471....... 481....... 491.......
P914042            447 CACGTTGCGC GCTCCGCCGg gagtcgaagc ---------- ----------
P914043            431 TCCGTGGTGG TCTCTGATGC AGCGGATCAG ATAGGAGGag taggacgaca
P914044            437 CTCTTGGTGG TCTCTGCTGC AGTGAATCGG ACAAGAGGgg gttaaa----
                       ********** ********** ********** ********
                           ****** ********** ********** ********

alignment position     501....... 511....... 521....... 531....... 541.......
P914042            477 -AAAAGACAC TGACGGAAGA GGACTGCGAG AGCCCGAgCG CCCGTCTCTC
P914043            481 gAAAGAATCC TGTCCGGGAA CGCTTGCTGG ACCGCGAACG CCAGTCACTT
P914044            483 -AAAATACAC CGCCGGGGAA TGCTTGCCAG ACCGCGAACG CCAGTTACTT
                        ********* ********** ********** ********** **********
                                       ***** ********** ********** **********
                                       ***** ********** ******* ** **********
                                                                ** ***** ****
                                                                ** ***** ****

alignment position     551....... 561....... 571....... 581....... 591.......
P914042            526 TTGTGCCCTA GCAGATTCCG TCGCTTCt-- -TCCGGAGCC GTACGTGGCA
P914043            531 TTGGGCCCCC ACAGCCACCG TCGCTTCCTC CTCCGGAGCC GTACGGGGCA
P914044            532 TTGGGCCCTC GCAGCCGCCG TCGCTTCCTA CT---GAGCG GTACGGGGCA
                       ********** ********** ********** ********** **********
                       ********** ********** ********** ********** **********
                       ********** ********** ********** ********** **********
                       ********** ********** *******     *   ***** **********
                       ********** ********** *******     *   ***** **********
                                                                   **********
                                                                   **********
```

```
                                                          **********
                                                          **********
alignment position  601.......  611.......  621.......  631.......  641.......
P914042        573  CCGCCCCGCT  CGCGGGCGGC  CGcggggctt  gctggGAAGA  GAGGCGAAGC
P914043        581  CCGCCCCTCC  CGCGTGCTGC  CG--------  -----GAAGA  GAGGTAAAGC
P914044        579  CCGCCCCTCT  CGCGTGCTGC  CG--------  -----GAAGA  GCGGTGAAGC
                    **********  **********  **               *****  **********
                    **********  **********  **               *****  **********
                    **********  **********  **               *****  *
                    **********  **********  **               *****  *
                    **********  **********  **               *****  *
                    **********  **********  **               *****  *
                    **********  **********  **
                    **********  **********  *
                    **********  **********  *

alignment position  651.......  661.......  671.......  681.......  691.......
P914042        623  CA--GGTCAC  CTttcaagga  cccagaagta  gggttttggc  ctag----GT
P914043        618  CG--GGTCAC  CTCTGAGGAG  CCGGT-----  -GACGGGTTG  GCGTGCGAGT
P914044        616  CcccGGTCAC  CTCTGAGGAG  CCGGTaacgg  tGACGGGTTG  GAGTGTGAGT
                    **  ******  **********  *****         ********  **********
                    **  ******  **********  *****         ********  **********
                        ******  **                        ********  **********
                                                                            **
                                                                            **
                                                                            **
                                                                            **
                                                                            **

alignment position  701.......  711.......  721.......  731.......  741.......
P914042        667  AACGGGGCAG  AGATGTGGTT  CGAGATTCTC  CCcGGACTCT  CCGTCATGGG
P914043        660  AACGGTGCGG  AGATGTGGTT  CGAGATTCTC  CCTGGCCTCG  CCATTATGGG
P914044        666  AACGGTGAGG  AGATGTGGTT  CGAGATTCTC  CCTGGCCTCG  CCATTATGGG
                    **********  **********  **********  **********  **********
                    **********  **********  **********  **********  **********
                    **********  **********  **********  **********  **********
                    **********  **********  **********  **********  **********
                    **********  **********  **********  **********  **********
                    **********  **********  **********  **  ******  **********
                    **********  **********  **********  **
                    **********  **********  **********  **
                                      ****  **********  **
                                      ****  **********  **

alignment position  751.......
P914042        717  CGTGTGCTTG
P914043        710  GGTGTGCTTG
P914044        716  GGTGTG----
                    **********
                    ******
                    ******
                    ******
                    ******
                    ******
```

Pairwise similarities:

For each pairwise alignment, the similarity (relative to the maximum similarity) and the number of identical nucleic acids (in % of shorter sequence) is given. Maximum values are underlined.

	P914043 (719 bp)	P914044 (721 bp)
**P914042 (726 bp)**	0.526<br>68 %	0.467<br>67 %

**P914043 (719 bp)**		1.000 83 %

Please note that the **similarity value 1.000** marks only the two **most similar** sequences, it does not necessarily mean that these sequences are identical.

**Extract alignment region**	
**Positions in alignment**	from: to:
**Output file**	dialign.seq

Extract aligned sequences

For questions or bug reports, please contact support@genomatix.de

© Genomatix Software GmbH 1998-2005 - All rights reserved. GEMS Launcher 4.1.0

Genomatix-Logo | Personal Personal | Password Password | Messages Messages | Logout Logout | GEMS-Logo

Genomatix Portal GEMS ElDorado Gene2Promoter MatBase
Frequently_asked_questions Your Results Your_Sequences Your Protocol Help

GEMS Launcher Task: *DiAlign TF*: Multiple alignment plus TF sites
working on ndufa2 (3 seq.)

[Alignment] [Pairwise similarities]

DiAlign professional TF Release 3.1.1 September 2005 **Fri Oct 20 20:30:09 2006**

Solution parameters:

**Sequence file:**	ndufa2 (3 seq.)
**Type of sequences:**	nucleotide sequences
**Threshold T:**	0.00
**'*' signs below alignment denote:**	diagonal similarity (max. similarity is represented by 10 '*' signs)
**Alignment output:**	complete alignment is shown<br># nucleic acids per line: 50
**TF output:**	common TF matches located in aligned regions (common to 3 (100.0 %) sequences)
**Family matches:**	yes
**MatInspector library:**	Matrix Family Library Version 6.1 (June 2006)
**Selected groups** (core/matrix sim)	• ALL user_defined.lib (0.75/Optimized)<br>• ALL vertebrates.lib (0.75/Optimized)

Aligned Sequences:

No.	Sequence Name	Sequence Description	Lengt[illegible]
1	GXP_123839	GXP_123839 loc=GXL_103357|sym=NDUFA2|geneid=4695|acc=GXP_123839|taxid=9606|spec=Homo sapiens|chr=5|ctg=NC_000005|str=(-)|start=140006924|end=140007924|len=1001|tss=501|descr=NADH dehydrogenase (ubiquinone) 1 alpha subcomplex, 2, 8kDa|comm=GXT_2834145/NM_002488/871/bronze	10[illegible]
2	GXP_83080	GXP_83080 loc=GXL_69065|sym=Ndufa2|geneid=17991|acc=GXP_83080|taxid=10090|spec=Mus musculus|chr=18|ctg=NC_000084|str=(-)|start=36869998|end=36871022|len=1025|tss=501,525|descr=NADH dehydrogenase (ubiquinone) 1 alpha subcomplex, 2|comm=GXT_13006391/AK018732/570/gold;GXT_13017074/AK003608/546/gold;GXT_13131667/NM_010885/546/bronze	10[illegible]
3	GXP_309531	GXP_309531 loc=GXL_260322|sym=Ndufa2_predicted|geneid=291660|acc=GXP_309531|taxid=10116|spec=Rattus norvegicus|chr=18|ctg=NC_005117|str=(-)|start=29443334|end=29444334|len=1001|tss=501|descr=NADH dehydrogenase (ubiquinone) 1 alpha subcomplex, 2 (predicted)|comm=GXT_21781541/XM_214570/566/bronze	10[illegible]

Alignment (DiAlign format):

Please note that only **upper-case** letters are considered to be **aligned**. For more information, please have a look at the user guide.

V$GATA V$STAF V$ZBPF V$SP1F V$YY1F V$PARF V$ZNFP V$CLOX V$HEAT TSS

```
alignment position             1.........  11........  21........  31........  41........
GXP_123839 (NDUFA2)          1 gcccatcatc acttggagta gg-------- -------AGT GAGGACTGGG
GXP_83080 (Ndufa2)           1 aaccacgtcc ctaaaagaga ggcacaagcc atggtttACT AAGGAAGGGA
GXP_309531
(Ndufa2_predicted)           1 ---------- ---------- ---------- ---------- ----------
                                                                       *** **********

alignment position             51........  61........  71........  81........  91........
GXP_123839 (NDUFA2)         36 GACGGAGTTT Ggagctg--- ---------- ---------- ----------
GXP_83080 (Ndufa2)          51 TAGGGAGGTA GAGCTGCctc aaaaaaTTCA AAACGCCGGT CCTCCACAAA
GXP_309531
(Ndufa2_predicted)           1 ----GAGGTG GGGCTGC--- ------TTCA AAACGCCGGT CCTCAACAAA
```

```
                                   ********** *******                 **** ********** **********
                                                                      **** ********** **********

alignment position                 101....... 111....... 121....... 131....... 141.......
GXP_123839 (NDUFA2)             53 ---------- ---------- -------TCG GCCTCACTTA GGACCCTGTG
GXP_83080 (Ndufa2)             101 aGAAATGAAA CTTGAGGGCA GCTCAGCTGG GCCTCTCCTA AGGACCAATG
GXP_309531 (Ndufa2_predicted)   38 -GAAACAAAA TTTGAGGGCA GTTCAGCAGG GCCTCTCCTA AAGGCCAATG
                                    ********* ********** ********** ********** **********
                                    ********* ********** ********** ********** *    ******
                                    ********* ********** ********** ********** *    ******

alignment position                 151....... 161....... 171....... 181....... 191.......
GXP_123839 (NDUFA2)             76 GCTTCTCCGG GCTCAagcgt cacacataca tacatacaca cacacacaca
GXP_83080 (Ndufa2)             151 GTTCGCGCGG CTCCA----- ---------- ---------- ----------
GXP_309531 (Ndufa2_predicted)   87 GTTCGCGCGC CTCCA----- ---------- ---------- ----------
                                   ********** *****
                                   ********** *****
                                   ********** *****

alignment position                 201....... 211....... 221....... 231....... 241.......
GXP_123839 (NDUFA2)            126 cacacacaca cACACACACA C---ACCCGG CTTAAGCTCA GAAgctacaa
GXP_83080 (Ndufa2)             166 ---------- -TCAGTCACA CgacACCTTG CTTAAATTCA GCAAACTCGA
GXP_309531 (Ndufa2_predicted)  102 ---------- -ACAGTCACA C---AACTGG CTTAAATTCA GCAAACTCAA
                                               ********* *   ****** ********** **********
                                               ********* *     **** ********** **********
                                               ********* *     **** ********** **********
                                               ********* *

alignment position                 251....... 261....... 271....... 281....... 291.......
GXP_123839 (NDUFA2)            173 taatGCGCCA AGTGCCCCAA GTGCCATGGG GAAGAAATAA CGGatggcct
GXP_83080 (Ndufa2)             205 C---GAGCCT AGCGTCTCTA GTCCCGCGGG CAGGAAAGAG TGGTcGACTC
GXP_309531 (Ndufa2_predicted)  138 C---AAGCCT AGTGTCTCTA GTCCCGCGGG CAAGAAAGAG TGGTtGACTC
                                   *   ****** ********** ********** ********** **** *****
                                   *   ****** ** ******* ********** ********** **** *****
                                   *   ****** ** ******* ********** ********** **** *****
                                         **** ** ******* ********** ********** ***

alignment position                 301....... 311....... 321....... 331....... 341.......
GXP_123839 (NDUFA2)            223 GAGCCTTACT ATCTCGCTCC GGCATTTTGt tatcgttctt tcgctgtcaC
GXP_83080 (Ndufa2)             252 GAGCCTTACT ATCTCGTTCT GGCATTTTGG CAGCGGCCTC TCAACG---T
GXP_309531 (Ndufa2_predicted)  185 TAGCCTTACT ATCTCGCTCC GGCATTTcGG CCGCGGTCTC TCAGCG---C
                                   ********** ********** ********** ********** ******   *
                                   ********** ********** ********** ********** ******   *
                                   ********** ********** *********
                                   ********** ********** ******* *
                                   ********** ********** ******* *
                                    ********* ********** *******
                                    ********* ********** *******

alignment position                 351....... 361....... 371....... 381....... 391.......
GXP_123839 (NDUFA2)            273 CAACAATCGa gtctccaa-- CAG--CAACA CCGCAGCGAC AAGCTCTTTC
GXP_83080 (Ndufa2)             299 CCGCAATCGC GAAATGTCCc cctaaCAAGA CAGCGGCGAC AAACTTTTAT
GXP_309531 (Ndufa2_predicted)  232 CAGCAATCGC GAAATATCCt CAA--CAAAA CAGCGGCGAC AAACTTTTAT
                                   ********** *********  ***  ***** ********** **********
                                   ********** *********  ***  ***** ********** **********
                                                              ***** ********** **********
                                                                  * ********** **********
                                                                  * ********** **********
                                                                  * ********** **********
                                                                  * ********** **********
                                                                  * ********** **********

alignment position                 401....... 411....... 421....... 431....... 441.......
GXP_123839 (NDUFA2)            319 CCACAGTGCA CCtcagaatc aacCCACACT GCTTTGCGTT CGCAGCCTTT
GXP_83080 (Ndufa2)             349 CCACAATGCA CCACGAAAac ctgt------ ---------- ----------
GXP_309531 (Ndufa2_predicted)  280 CCACAATGCA CCACGAAAgc ctgCGACAGA GCTTTGCCTT CCGTGCCTTC
```

* *
* * * * * * * * * * * * * * * * * *
* * * * * * * * * * * * * * * * * *
* * * * * * * * * * * * * * * * * *
* * * * * * * * * * * * * * * * * *
* * * * * * * * * * * *
* * * * * * * * * * * *
* * * * * * * * * * * *

alignment position 4 5 1 . . . . 4 6 1 . . . . 4 7 1 . . . . 4 8 1 . . . . 4 9 1 . . . .
GXP_123839 (NDUFA2) 369 CCGCTTCCTg tttttccctc cgaccaaccc cgagcgcaaa gaaattgacc
GXP_83080 (Ndufa2) 373 ---CTTCCTT CGCGTCACCA Cacgatgcct cctg------ ----------
GXP_309531 (Ndufa2_predicted) 330 CGGCTTCCTT CGCGTCACTA Ccattctgcc cctataaagg cctcatttaa
* *
* * * * * *

alignment position 5 0 1 . . . . 5 1 1 . . . . 5 2 1 . . . . 5 3 1 . . . . 5 4 1 . . . .
GXP_123839 (NDUFA2) 419 tcgcagcggt cctacaatac ttttataTCA TTGGCCAAGC TTTACCCCGC
GXP_83080 (Ndufa2) 404 ---------- ---------- -------TCA TTGGCTAAGA TTTATCCCGC
GXP_309531 (Ndufa2_predicted) 380 tt-------- ---------- ---------- ---GCTAAGA TTTGTCCCGC
* *
* *
* * * * * * * * * * * * * * * * *
* * * * * * * * * * * * * * * * *

alignment position 5 5 1 . . . . 5 6 1 . . . . 5 7 1 . . . . 5 8 1 . . . . 5 9 1 . . . .
GXP_123839 (NDUFA2) 469 CCCT--GCC- ---TCATGCA GCCTATGGGC TAGGCTTTAG GGTCCGCGGT
GXP_83080 (Ndufa2) 427 CCCTttCCCT GCGTCATCCA ATCGACGGCG AAGACTGGAG GGCCCGCTAT
GXP_309531 (Ndufa2_predicted) 399 CCCT--CCCC GCGTCATCCA ATCGATGACG GAGACTAGAG GGT-CGCTAT
* *
* *
* *
* * * *

alignment position 6 0 1 . . . . 6 1 1 . . . . 6 2 1 . . . . 6 3 1 . . . . 6 4 1 . . . .
GXP_123839 (NDUFA2) 513 TGGTCAGAcc ggagcacttg gcCTGAAGAC CTGGAATTGG CGACTTCGAT
GXP_83080 (Ndufa2) 477 TGGTCCGATT GAGGG----- ---TGAAAGG TCGTGATTGG GCGCTCCTTT
GXP_309531 (Ndufa2_predicted) 446 TGGTCCGATT GAGGGag--- --CTGAAAGG CCGTGATTGG GTGCTCCTTT
* *
* *
* *
* *

alignment position 6 5 1 . . . . 6 6 1 . . . . 6 7 1 . . . . 6 8 1 . . . . 6 9 1 . . . .
GXP_123839 (NDUFA2) 563 ATTAACAAGG ATGGCGGCGG CCGCAGCAAG TCGAGGAGTC GGGGCAAAGC
GXP_83080 (Ndufa2) 519 GCTAACAAAG ATGGCGGCTG CCGCTGCTAG CCGAGCGGTC GGCGCAAAGC
GXP_309531 (Ndufa2_predicted) 491 GCTAGCAAAG ATG------G CGGCCGCAAG CCGAGTAGTC GGTGCAAAGC
* *
* *
* *
* *
* *
* *
* * * * * * *

alignment position 7 0 1 . . . . 7 1 1 . . . . 7 2 1 . . . . 7 3 1 . . . . 7 4 1 . . . .
GXP_123839 (NDUFA2) 613 TGGGCCTGCG TGAGATTCGC ATCCACTTAT GTCAGCGCTC GCCCGGCAGC
GXP_83080 (Ndufa2) 569 TGGGGTTGCG TGAGATTCGC GTTCACTTAT GCCAGCGTTC CCCAGGCAGC
GXP_309531 (Ndufa2_predicted) 535 AGGTACTGCG TGAGATTCGC ATTCACTTAT GCCAGCGTTC CCCAGGCAGC
* *
* *
* *
* *
* *
* *
* *
* *
* *
* *

alignment position 7 5 1 . . . . 7 6 1 . . . . 7 7 1 . . . . 7 8 1 . . . . 7 9 1 . . . .

```
GXP_123839 (NDUFA2)           663 CAGGGCGTCA GGTGAGGCGC -GGCGTGGTG AGGCGGGCGG GCTTCGGGAc
GXP_83080 (Ndufa2)            619 CAGGGTGTGA GGTGAGGCGg aAGCGTGGGA AGGCGAGTGA GCTTCGGAA-
GXP_309531 (Ndufa2_predicted) 585 CAGGGTGTGA GGTGAGTCGT -AGCGTGGGA AGGCGAGTGA GCTTCGGAA-
                                  ********** **********  ********* ********** *********
                                  ********** **********  ********* ********** *********
                                  ********** *********   ********* ********** *********
                                  ********** *********   ********* ********** *********
                                  ********** ******      ********* ********** *******
                                  ********** ******       ******** ********** *******
                                  ********** ******
                                  *****  **** ******
                                  *****  **** ******
                                  *****

alignment position                801....... 811....... 821....... 831....... 841.......
GXP_123839 (NDUFA2)           712 GCCAGGGTCA CGACCTCGAC CTCCCTGAAG TTGAGGGaag cccccgccca
GXP_83080 (Ndufa2)            668 GCTGGAGCTA TGACCTTGAC CTCCGGGCAG GGGCGGGGGc ----------
GXP_309531 (Ndufa2_predicted) 633 GgTGGAGCTC TGACCTTGAC CTCCGGa--- -GGCGGGGGg tgtcagggtc
                                  ********** ********** ********** *********
                                  *  ******* ********** ******
                                  *           ********* ****
                                  *

alignment position                851....... 861....... 871....... 881....... 891.......
GXP_123839 (NDUFA2)           762 cagcacgc-- ---------- -GCGGCGCTC TCTCCg---- -------GCC
GXP_83080 (Ndufa2)            708 --TGTGTACC TTTGCCCGCT AGCGGCAGTC TCTCtcttcc ccccCCCCCC
GXP_309531 (Ndufa2_predicted) 679 agTGTGTACC TTTGCCCGCT AGCGACAGTC TCTCCt---- ----CCCGCC
                                    ******** ********** ********** *****          ******
                                    ******** ********** ********** ****           ******
                                    ******** ********** ********** ****              ***
                                                                                     ***
                                                                                      **
                                                                                      **

alignment position                901....... 911....... 921....... 931....... 941.......
GXP_123839 (NDUFA2)           788 CCGCAGGGAC TTCATTGAGA AACGCTACGT GGAGCTGAAG AAGGCGAATC
GXP_83080 (Ndufa2)            756 CCGCAGGGAT TTCATCGTGC AACGGTACGT GGAGCTGAAG AAGGCGCACC
GXP_309531 (Ndufa2_predicted) 721 CCGCAGAGAT TTCATCCAGC AACGGTACGT GGAGCTGAAG AAGGCGCACC
                                  ********** ********** ********** ********** **********
                                  ********** ********** ********** ********** **********
                                  ********** ********** ********** ********** **********
                                  ********** ********** ********** ********** **********
                                  ********** ********** ********** ********** **********
                                  ********** ******  ** ********** ********** **********
                                                     ** ********** ********** **********
                                                     ** ********** ********** **********
                                                     ** ********** ********** **********
                                                                          *** **********

alignment position                951....... 961....... 971....... 981....... 991.......
GXP_123839 (NDUFA2)           838 CCGACCTACC CATCCTAATC CGCGAATGCT CCGATGTGCA GCCCAAGCTC
GXP_83080 (Ndufa2)            806 CCAACCTGCC CATTCTGATC CGCGAATGCT CGGAGGTGCA GCCCAAGCTT
GXP_309531 (Ndufa2_predicted) 771 CCGACCTGCC CATTCTAATC CGCGAATGTT CAGAGGTGCA GCCCAAGCTC
                                  ********** ********** ********** ********** **********
                                  ********** ********** ********** ********** **********
                                  ********** ********** ********** ********** **********
                                  ********** ********** ********** ********** **********
                                  ********** ********** ********** ********** **********
                                  ********** ********** *******  ** ********** **********
                                  **  ****** ********** *******   * ********** **********
                                  **  ****** ********** *******   * ********** **********
                                  **                              * ********** **********
                                  **                                      *** **********

alignment position                1001...... 1011...... 1021...... 1031...... 1041......
GXP_123839 (NDUFA2)           888 TGGGCCCGCT ACGGTGAGTg cgGGACGCCA GGGGTCTGGG GCTCCGGTAG
GXP_83080 (Ndufa2)            856 TGGGCCCGCT ATGGTGAGTA CTGGTCGCCG GAGGTCAGGG GCCCGGGTTG
GXP_309531 (Ndufa2_predicted) 821 TGGGCCCGTT ATGGTGAGTA CTGGTCGttG GAGGTCAGGG GCCCGGGTTG
```

```
                                   ********** ********** ********** ********** **********
                                   ********** ********** *******  * ********** **********
                                   ********** ********** *******  * ********** **********
                                   ********** ********** *******  * ********** **********
                                   ********** *********    *****  * ********** **********
                                   ********** *********               ******** **********
                                   ********** *********
                                   ********** ********
                                   ********** ********
                                   ********** ********

alignment position                 1051...... 1061...... 1071...... 1081...... 1091......
GXP_123839 (NDUFA2)           938  GGGAACAAGG GTTTGAGAAG -AGAAGGGAC CGCCCAAGGT CGTGCAGAAA
GXP_83080 (Ndufa2)            906  CAGAATACGG GATGGAGAGA -TGAAGGGGC CTCCCAACGT CCTGGGGAAA
GXP_309531
(Ndufa2_predicted)            871  CAGAATACGG GATAGAAAAa cATAAGGAGC CTCCCCACGT CATGGGAAGA
                                   ********** ********** ********* ********** **********
                                   ********** *********     ******* ********** ******** *
                                   ********** ***                                       *
                                   ********** ***                                       *
                                   ********** ***
                                   ********** * *

alignment position                 1101...... 1111...... 1121...... 1131...... 1141......
GXP_123839 (NDUFA2)           987  Cccgtggaga aacta----- ---------- ---------- ----------
GXP_83080 (Ndufa2)            955  CGGGGCTGAA GGACCAAAAC TCGTACTGAT AAAGgAATTC ACATTTACTT
GXP_309531
(Ndufa2_predicted)            921  CGGGGCTGAA GGACCAAAAC TCCAACTGAT AAAGaAATTC ACATTTACTT
                                   ********** ********** ********** **** ***** **********
                                   ********** ********** ********** **** ***** **********
                                   ********** ********** ********** **** ***** **********
                                   *                                     ***** **********

alignment position                 1151...... 1161...... 1171...... 1181
GXP_123839 (NDUFA2)           1002 ---------- ---------- ---------- -
GXP_83080 (Ndufa2)            1005 AGTTGTGTGA TGCTTTTTAT T--------- -
GXP_309531
(Ndufa2_predicted)            971  AGTTGTGTGA TGCCTTTTAT Tcatggcaac a
                                   ********** ********** *
                                   ********** ********** *
                                   ********** ********** *
                                   ********** ********** *
```

Pairwise similarities:

For each pairwise alignment, the similarity (relative to the maximum similarity) and the number of identical nucleic acids (in % of shorter sequence) is given. Maximum values are underlined.

	GXP_83080 (1025 bp)	GXP_309531 (1001 bp)
GXP_123839 (1001 bp)	0.341<br>49 %	0.334<br>46 %
GXP_83080 (1025 bp)		<u>1.000</u><br><u>79 %</u>

Please note that the **similarity value 1.000** marks only the two **most similar** sequences, it does not necessarily mean that these sequences are identical.

Extract Alignment Region	
**Positions in alignment**	from: to:
**Output file**	dialign.seq

Extract Aligned Sequences

For comments, questions, or bug reports, please contact support@genomatix.de.

© Genomatix Software GmbH 1998-2006 - All rights reserved.

GEMS Launcher 4.3.0

License Agreement

Genomatix-Logo	Personal<br>Personal	Password<br>Password	Messages<br>Messages	Logout<br>Logout	GEMS-Logo

Genomatix Portal GEMS ElDorado Gene2Promoter MatBase
Frequently asked questions Your Results Your Sequences Your Protocol Help

GEMS Launcher Task: *DiAlign TF*: Multiple alignment plus TF sites
working on CVW-ndufa3 (3 seq.)

[Alignment] [Pairwise similarities]

DiAlign professional TF Release 3.1.1 September 2005 **Fri Oct 20 20:31:45 2006**

Solution parameters:

**Sequence file:**	CVW-ndufa3 (3 seq.)
**Type of sequences:**	nucleotide sequences
**Threshold T:**	0.00
**'*' signs below alignment denote:**	diagonal similarity (max. similarity is represented by 10 '*' signs)
**Alignment output:**	complete alignment is shown<br># nucleic acids per line: 50
**TF output:**	common TF matches located in aligned regions (common to 3 (100.0 %) sequences)
**Family matches:**	yes
**MatInspector library:**	Matrix Family Library Version 6.1 (June 2006)
**Selected groups** (core/matrix sim)	• ALL user_defined.lib (0.75/Optimized)<br>• ALL vertebrates.lib (0.75/Optimized)

Aligned Sequences:

No.	Sequence Name	Sequence Description	Length
1	P914067	P914067 sym=NDUFA3|loc=Loc4696|taxid=9606|spec=Homo sapiens|chr=19|ctg=NT_011109|str=(+)|start=26873850|end=26874850|len=1001|tss=501|comm=NADH dehydrogenase (ubiquinone) 1 alpha subcomplex, 3, 9kDa; (NM_004542/501/bronze;)	1001 bp
2	mNDUFA3	mNDUFA3 m6_dna range=chr7_random:9785301-9786471 5'pad=0 3'pad=0 revComp=FALSE strand=? repeatMasking=none	1171 bp
3	P914068	P914068 sym=na|loc=Loc361494|taxid=10116|spec=Rattus norvegicus|chr=1|ctg=NW_047555|str=(-)|start=9304776|end=9305776|len=1001|tss=501|comm=similar to NADH-ubiquinone oxidoreductase B9 subunit (Complex I-B9) (CI-B9); (XM_341773/501/bronze;)	1001 bp

Alignment (DiAlign format):

Please note that only **upper-case** letters are considered to be **aligned**. For more information, please have a look at the user guide.

V$STAT V$BRNF V$ZBPF V$YY1F V$NKXH V$EKLF V$RARF V$RORA V$MZF1 TSS

```
alignment position    1 . . . . . . . . .   1 1 . . . . . . . .   2 1 . . . . . . . .   3 1 . . . . . . . .   4 1 . . . . . . . .
P914067 (NDUFA3)    1 a g a g c c - - - -   - - - - - - - - - -   - - - - - - - - - -   - - - - - - - - - -   - - - - - - - - - -
mNDUFA3             1 t t a a t g - - - -   - - - - - - C C C T   G T C T C A A A A T   A A A A A G T A A T   G A A A G G G C T G A
P914068 (na)        1 a g a t a c t t a a   t g a g a C C C T A   T C T C A A A A T A   A A A A G C A A T G   A A A G G G T T G A
```

```
                                            ***** ********** ********** **********
                                            ***** ********** ********** **********
                                            ***** ********** ********** **********
alignment position    51........ 61........ 71........ 81........ 91........
P914067 (NDUFA3)    7 ---------- ---------- ---------- ---------- ----------
mNDUFA3            42 AAAATGTGGC TCAACAATAA AAGATATGCC TAAAATCCAC CAGCagggac
P914068 (na)       51 CAAATGTGGC TCAACAATAC AAAATATGCC TAGAATCCAC CAGCcggagc
                      ********** ********** ********** ********** ****
                      ********** ********** ********** ********** ****
                      ********** ********** ********** ********** ****
                          ****** ********** ********** ********** ****
alignment position    101....... 111....... 121....... 131....... 141.......
P914067 (NDUFA3)    7 ---------- ---------- ---------- ---------- ----------
mNDUFA3            92 ctgggatagt aGAATCTATC ACCAAGTGGA CGGAGGAGTG GCTCAATCgg
P914068 (na)      101 ctgggtcatg -GAATCTACC AGCAAGTGGA CCGAGGAGTG CCTCAGTCat
                                  ********* ********** ********** ********
                                  ********* ********** ********** ********
                                  ********* ********** ********** ********
alignment position    151....... 161....... 171....... 181....... 191.......
P914067 (NDUFA3)    7 ---------- ---------- ---------- ---------- ----------
mNDUFA3           142 AGATGGGTAG CCTAgcctgT CCTGGTTTCA GTTTTCAGCA CTAAGGGGCA
P914068 (na)      150 AGATGGGTAG CCTA-----T CCTGGTTTCA ATCTGCAACA CTGAGGGGCA
                      ********** ****     * ********** ********** **********
                                          * ********** ********** **********
alignment position    201....... 211....... 221....... 231....... 241.......
P914067 (NDUFA3)    7 ---------- ---------- ---------- ---------- --CGCAGAAA
mNDUFA3           192 GATGGAGGgA AAAGCCATGA TCTGCATGCT AGTAACACAA AGCACAGGCA
P914068 (na)      195 GAGGGTGGaA AAAACCATGA GCCGCATGCT AGTAACATAA AGCACAGACT
                      ******** * ********** ********** ********** **********
                      ******** * ********** ********** ********** *******
                               * ********** ********** ********** *******
alignment position    251....... 261....... 271....... 281....... 291.......
P914067 (NDUFA3)   15 GCTCCTAACC CATCTGGGAT GAgaccTAGC GCTTCCAGGA CGAGCCGATG
mNDUFA3           242 GTCCTTAATA TATTTGGGAT TAATT-TAGA ATTTCCAGAA TGAGCTTATG
P914068 (na)       245 GTTCTTAATC TATTTGGGAT CAATT-TAGA ATTTCTGGAA TGAGTTCATG
                      ********** ********** ***** **** ********** **********
                         ******* ********** ***** **** ********** ****   ***
                         ******* ********** ***** **** ********** ****   ***
                         ******* ********** ***** **** ********** ****
alignment position    301....... 311....... 321....... 331....... 341.......
P914067 (NDUFA3)   65 TTGAGCTGAG ACCTCGAa-- ---------- ---------- ----------
mNDUFA3           291 TCTAGATGTG ATCTCAATGT AAAGCTGACT TTTTTttttt taaggtttat
P914068 (na)      294 TCTAGACGCG ACCTCAATGT AATGAGGACT TTCTTgcaga agatcttgaa
```

```
                    * * * * * * * * * *  * * * * * * * * *  * * * * * * * * * *  * * * * *
                    * * * * * * * * * *  * * * * * * * * *  * * * * * * * * * *  * * * * *
                    * * * * * * * * * *  * * * * * * *

alignment position      3 5 1 . . . . . . .  3 6 1 . . . . . . .  3 7 1 . . . . . . .  3 8 1 . . . . . . .  3 9 1 . . . . . . .
P914067 (NDUFA3)    83  - - - - - - - - - -  - - - - - - - - - -  - - - - - - - - - -  - - - - - - - - - -  - - - - - - - - - -
mNDUFA3            341  t t a t g a g t a c  a c t g t t g c a g  t c t t c a g a c a  c a c c a g a a g a  g g g c g t c a g a
P914068 (na)       344  - - - - - - - - - -  - - - - - - - - - -  - - - - - - - - - -  - - - - - - - - - -  - - - - - - - - - -

alignment position      4 0 1 . . . . . . .  4 1 1 . . . . . . .  4 2 1 . . . . . . .  4 3 1 . . . . . . .  4 4 1 . . . . . . .
P914067 (NDUFA3)    83  - - - - - - - G G A  C A G G T T A G T C  A T T C A C C T T C  T c c c g g g c t c  a g t t t c t t c g
mNDUFA3            391  t c c c a t t G C A  G A T G G T T G T C  A G T C A C C A T G  T g g t t a c t g g  a a a t t a a a c c
P914068 (na)       344  - - - - - - - - - -  - - - - - - - - - -  - - - - - - - - - -  - - - - - - - - - -  - - - - - - - - - -
                                      * * *  * * * * * * * * * *  * * * * * * * * * *  *

alignment position      4 5 1 . . . . . . .  4 6 1 . . . . . . .  4 7 1 . . . . . . .  4 8 1 . . . . . . .  4 9 1 . . . . . . .
P914067 (NDUFA3)   126  t c t g t a a a a t  g g g c t t t c a t  a c - - - - - - - -  - - - - - - - - - -  - - - - - - - - - -
mNDUFA3            441  t t t g g a a g a g  c a g a c a g a g c  t c t g a g c c a t  c t c t c c a g c c  c a a a g c t g a c
P914068 (na)       344  - - - - - - - - - -  - - - - - - - - - -  - - - - - - - - - -  - - - - - - - - - -  - - - - - - - - - -

alignment position      5 0 1 . . . . . . .  5 1 1 . . . . . . .  5 2 1 . . . . . . .  5 3 1 . . . . . . .  5 4 1 . . . . . . .
P914067 (NDUFA3)   148  - - - - - - - - - -  - - - - - - - - - -  - - - - - - - - - -  - - - - - - - - - -  - - - - - - - - - A
mNDUFA3            491  t t t a t t a c a g  g a g a a c g t t a  t G T T A C T T C T  C C G A T G C A T T  T G C T G A G T T A
P914068 (na)       344  - - - - - - - - - -  - - - - - - - - - -  - G T T A C T T C T  C T G A C G C A T C  T T C T A A G G T A
                                                                    * * * * * * * * *  * * * * * * * * * *  * * * * * * * * * *
                                                                    * * * * * * * * *  * * * * * * * * * *  * * * * * * * * * *
                                                                                                                              *

alignment position      5 5 1 . . . . . . .  5 6 1 . . . . . . .  5 7 1 . . . . . . .  5 8 1 . . . . . . .  5 9 1 . . . . . . .
P914067 (NDUFA3)   149  T A A A C T A T A A  a a t g g g g a c t  a t t t t g t t c c  g c c t t a g g t g  g g t c g c a g c a
mNDUFA3            541  T A A A C T A T A A  G a a a a t - - - -  - - - - - - - - - -  - - - - - - - - - -  - - - - - - - - - -
P914068 (na)       373  T A A G C T A T A A  G a g a - - - - - -  - - - - - - - - - -  - - - - - - - - - -  - - - - - - - - - -
                        * * * * * * * * * *  *
                        * * * * * * * * * *  *
                        * * * * * * * * * *

alignment position      6 0 1 . . . . . . .  6 1 1 . . . . . . .  6 2 1 . . . . . . .  6 3 1 . . . . . . .  6 4 1 . . . . . . .
P914067 (NDUFA3)   199  g g a g g a c t a g  t c a c t c c g g a  g c g a c t t c t A  G G C T G A G A C T  A A G G A G A T T C
mNDUFA3            557  - - - - - - - - - -  - - - - - - - - - -  - - - - - - - - - -  - G G T G A G G C C  A G C A G G T G T T
P914068 (na)       387  - - - - - - - - - -  - - - - - - - - - -  - - - - - - - - - A  A G G T G A G A C C  A G G A G G T G T G
                                                                                     *  * * * * * * * * * *  * * * * * * * * * *
                                                                                           * * * * * * * * *  * * * * * * * * * *
                                                                                           * * * * * * * * *  * * * * * * * * * *

alignment position      6 5 1 . . . . . . .  6 6 1 . . . . . . .  6 7 1 . . . . . . .  6 8 1 . . . . . . .  6 9 1 . . . . . . .
P914067 (NDUFA3)   249  C A C G C A G G T c  c g c a a a g t c a  g g c t t g c g c t  t g c t c c t g a c  a c c a c t t c c t
mNDUFA3            576  C A A G G T G G T T  T A A A T C T T T C  a g t C C T C T T A  A G C - - - - - - -  - - - - - - - - - -
```

```
P914068        408 CAAGGTGGTT TAATTCTTTC tggCCTCCTA AGC------- ----------
(na)
                   ********** **********    ******* *** 
                   ********** **********    ******* *** 
                   ********** **********    ******* *** 
                                            ******* *** 

alignment           701....... 711....... 721....... 731....... 741.......
position
P914067        299 ttacctccac ggctccatct ttgttctgcg cgagtgcgca cgcgcaggct
(NDUFA3)
mNDUFA3        609 ---------- ---------- ---------- ---------- ----------
P914068        441 ---------- ---------- ---------- ---------- ----------
(na)

alignment           751....... 761....... 771....... 781....... 791.......
position
P914067        349 ccgaaagcgg gccgtCGCAC AGAGGGACCA CAACTCCCAG AGTGCTCCGC
(NDUFA3)
mNDUFA3        609 ---------- -----CTCAC TCCAGAACTA CAACTCCCGA AAGGGTCCAC
P914068        441 ---------- -----CTCAC TCTAGAACTA CAACTCCCGA AAGGCTTCAC
(na)
                                  ***** ********** ********** **********
                                  ***** ********** ********** **********
                                  ***** ********** ********** ****   ***
                                  ***** ********** ********** ****   ***
                                  ***** ********** ********** ****   ***
                                             ***** ********** ****

alignment           801....... 811....... 821....... 831....... 841.......
position
P914067        399 GTCCTTGCtt tcgcctctac ttgtgctcca gggcgcacgc gcagcCCTGG
(NDUFA3)
mNDUFA3        644 GTCCTCGC-- ---------- ---------- ---------- -----CCAAG
P914068        476 GTCCTTGC-- ---------- ---------- ---------- -----CCAAG
(na)
                   ********                                         *****
                   ********                                         *****
                   ********                                         *****
                   ********
                   *****

alignment           851....... 861....... 871....... 881....... 891.......
position
P914067        449 GAGCGGGTTC TCGCGCAtag ----GGACCA CAACTCCCAG GGTGCTCCGC
(NDUFA3)
mNDUFA3        657 GGGCGGGGCC TCACGCAAGG AGAAGGACCA CAACTCCCAG AGAGCTCCGC
P914068        489 GGGCGGGGCC TCATGCAAGG AAAAGAACCA CAACTCCCAG AGAGCTCCct
(na)
                   ********** ********** ********** ********** **********
                   ********** ***  ***** ********** ********** **********
                   ********** ***  ***** ********** ********** ********
                                             ****** ********** ********
                                             ****** ********** ********
                                               **** ********** ********

alignment           901....... 911....... 921....... 931....... 941.......
position
P914067        495 gtcctcgccg ctgtcgcCGC CGCGGAGACA AAGATGGCTG CGAGTAAGTG
(NDUFA3)
mNDUFA3        707 tccttgt--- -------CGC CGCCAAGAAC AAGATGGCCG GGAGTAAGTA
P914068        539 gctcttgt-- ---------- --CCAAGACC AAGATGGCTG CTAGTAAGTA
(na)
                                     *** ********** ********** **********
                                     *** ********** ********** **********
                                          ********* ********** **********
                                          ********* ********** **********
                                             ****** ********** **********
                                             ****** ********** **********
```

```
alignment position     951.......  961.......  971.......  981.......  991.......
P914067 (NDUFA3)  545  CAGGTTCCGG  TGGCGcaCGG  GGCTCGGGTA  GTTCTGGGAA  CCTCTGGGCG
mNDUFA3           747  CAGGTTtgga  GCTCG--CGG  GGATCAAGTA  GTTCCAGGAA  ACTGTGGGCT
P914068 (na)      575  CGCGTTCTGG  GCGCG--CGG  GGACCAAGTA  ATTCCAGGAA  ACCGTGGCTT
                       **********  *****  ***  **********  **********  **********
                       **********  *****  ***  **********  **********  **********
                       ******      *****  ***  **********  **********  *******  *
                       ******      *****  ***  **********  **********  *******  *
                       ******
                       ******

alignment position     1001......  1011......  1021......  1031......  1041......
P914067 (NDUFA3)  595  GTCCTGGGAC  TGAGGtgcgg  cagggcaggg  gtGGAAGCGA  TGGGGTCCGT
mNDUFA3           795  GATCCGTGGC  TGAGTCTCAG  TGGAGCCTTG  --GGGACCGG  Ta--ATCCGT
P914068 (na)      623  GACCCGTGGC  TGAGGCTCAG  TAGAGCCTTG  --GGGAGCGG  TAGGATCCGT
                       **********  **********  **********    ********  **********
                       **********  **********  **********    ********  *   ******
                       **********  **********  **********    ********  *
                       **********  **********  **********    ********  *

alignment position     1051......  1061......  1071......  1081......  1091......
P914067 (NDUFA3)  645  g-CTGGAGGG  GAACGCAGAA  GTCACGAGGG  GGctcctcCA  GGGCAGGGGT
mNDUFA3           841  CCCAGAATAG  GGGCGCGGCA  GTCACGAGGG  TG------CA  GAGCAGGGGT
P914068 (na)      671  CCCAGAAGAG  GGGCGCAGCA  GCCAATAGGG  TG------CA  GAGCAGGGGT
                       **********  **********  **********  **      **  **********
                       **********  **********  ****  ****  **      **  **********
                                      *******  ****  ****  **      **  **********
                                                     ****  **      **  **********
                                                     ****  **      **  **********
                                                     ****          **  **********
                                                                   **  **********
                                                                   **  **********
                                                                   **  **********
                                                                   **  **********

alignment position     1101......  1111......  1121......  1131......  1141......
P914067 (NDUFA3)  694  GGCACGAGAG  GGTTAGAGGT  CACCGggggc  agctacttgC  AGGGGTGACg
mNDUFA3           885  GATACGAGAG  TGTTAGAGGT  CACCGCAGTA  GTCCC----C  AAGGGTGACT
P914068 (na)      715  GATACGAGAG  TGTTAGAGGT  CACCGCAGCA  GTCCC----C  AAGGGCGACT
                       **********  **********  **********  *****    *  **********
                       **********  **********  **********  *****    *  **********
                       **********  **********  **********  *****    *  **********
                       **********  **********  *****
                       **********  **********  *****
                       **********  **********  *****
                       **********  **********  *****
                       **********  **********  *****
                       **********  **********  **
                       **********  **********  **

alignment position     1151......  1161......  1171......  1181......  1191......
P914067 (NDUFA3)  744  ctTCTTGCCA  CCCCTTCAGG  AGTCggCGCC  TTCCTCAAGA  ATGCCTGGGA
mNDUFA3           931  G-TCTTCTCA  CCTCTCTAGG  AATCTCTGCC  TTCCTCAAGA  ATGCCTGGGC
P914068 (na)      761  T-TTTTCTCA  CCTCT--AGG  AATCTCCGCC  TTCCTTAAGA  ATGCCTGGGC
```

```
                           * ******** ********** ********** ********** **********
                           * ******** *****  *** ********** ********** **********
                           * ******** *****  *** ********** ********** **********
                                             *** ********** ********** **********
                                                       **** ********** **********
                                                       **** ********** **********
                                                        *** ********** **********
                                                        *** ********** **********
                                                        *** ********** **********

alignment position         1201...... 1211...... 1221...... 1231...... 1241......
P914067 (NDUFA3)       794 CAAGGAGCCa GTGCTGGTCG TGTCCTTCGT CGTCGGGGGC CTCGGTGCGT
mNDUFA3                980 GAAGGAGCCG GTGCTGGTGG TGTCCTTCTC TGTCTGGGGC CTCGGTGAGT
P914068 (na)           808 AAAGGAGCCG GTGCTGGTGG TGTCCTTCAC TGTCTGGGGC CTCGGTGAGT
                           ********** ********** ********** ********** **********
                           ********** ********** ********** ********** **********
                           ********** ********** ********** ********** **********
                           ********** ********** ********** ********** **********
                           ********** ********** ********** ********** **********
                           *********  ********** ********** ********** *******
                           *********  ********** ********** ********** *******
                           *********  ********** ********** ********** *******
                           *********  ********** ********** ********** *******
                                  **

alignment position         1251...... 1261...... 1271...... 1281...... 1291......
P914067 (NDUFA3)       844 gagtgct--C CAGGCGCAAA CTTGCATCGT CCACCCccgt ccccctacat
mNDUFA3               1030 gccccctTCC CCGGCGCGAA AGTGCATCCT CCACCCGGAA GTCATCAgca
P914068 (na)           858 ac-----TCC CCGGAGCGAA CGTGTATCCT CCACCCTGAA GTCATCAata
                                  *** ********** ********** ********** *******
                                  *** ********** ********** ********** *******
                                  *** ********** ********** ********** *******
                                  *** ********** ********** ********** *******
                                    * ********** ********** ******

alignment position         1301...... 1311...... 1321...... 1331...... 1341......
P914067 (NDUFA3)       892 ccctccatct tgtaccccta aagccctatc gccgccctcg ggtcccctct
mNDUFA3               1080 ttccacggca cagtttcaTC TC-------- ---------- ----------
P914068 (na)           903 ttc------- --------TC TC-------- ---------- ----------
                                              ** **
                                              ** **
                                              ** **
                                              ** **

alignment position         1351...... 1361...... 1371...... 1381...... 1391......
P914067 (NDUFA3)       942 agtgtgtctg cacccccacg GCATCCCCTT atctatcccc atacccatta
mNDUFA3               1102 ---------- ---------- GCATCCCCTT GCCAGCCATA TCCTGACATt
P914068 (na)           910 ---------- ---------- GCATCCCCTT GCCAGCCATA TCCTGACATg
                                                 ********** ********** *********
                                                 ********** ********** *********
                                                 ********** ********** *********
                                                 ********** ********** *********

alignment position         1401...... 1411...... 1421...... 1431...... 1441......
P914067 (NDUFA3)       992 taacctctcc ---------- ---------- ---------- ----------
mNDUFA3               1132 ACTGTCGCTC TTCCCTCACC ACA------- -CACAAGAGG TGCACATT--
```

**P914068 (na)**	940	A C T G T C G C T C T T C C C T C A C C A C A t a c a c c t a C A C A A G A A G T G C A C A T T t a
		* * * * * * * * * * * * * * * * * * * * * * * * * * * * * * * * * * * * * * * *
		* * * * * * * * * * * * * * * * * * * * * * *
		* * * * * * * * * * * * * * * * * * * * * * *
alignment position		1 4 5 1 . . . . . . 1 4 6 1
**P914067 (NDUFA3)**	1002	- - - - - - - - - - - -
**mNDUFA3**	1172	- - - - - - - - - - - -
**P914068 (na)**	990	a c c c a g c t t g t a

Pairwise similarities:

For each pairwise alignment, the similarity (relative to the maximum similarity) and the number of identical nucleic acids (in % of shorter sequence) is given. Maximum values are underlined.

	**mNDUFA3 (1171 bp)**	**P914068 (1001 bp)**
**P914067 (1001 bp)**	0.227<br>36 %	0.193<br>36 %
**mNDUFA3 (1171 bp)**		1.000<br>79 %

Please note that the **similarity value 1.000** marks only the two **most similar** sequences, it does not necessarily mean that these sequences are identical.

**Extract Alignment Region**	
**Positions in alignment**	from: to:
**Output file**	dialign.seq

Extract Aligned Sequences

For comments, questions, or bug reports, please contact support@genomatix.de.

© Genomatix Software GmbH 1998-2006 - All rights reserved.

GEMS Launcher 4.3.0

License Agreement

Genomatix-Logo Personal Password Messages Logout GEMS-Logo
Personal Password Messages Logout

Genomatix PortalGEMSElDoradoGene2PromoterMatBase
Frequently asked questionsYour ResultsYour SequencesYour ProtocolHelp

GEMS Launcher Task: *DiAlign TF*: Multiple alignment plus TF sites
working on C1-NDUFA4 (3 seq.)

[Alignment] [Pairwise similarities]

DiAlign professional TF Release 3.1.1 September 2005 **Fri Oct 20 20:35:27 2006**

Solution parameters:

**Sequence file:**	C1-NDUFA4 (3 seq.)
**Type of sequences:**	nucleotide sequences
**Threshold T:**	0.00
**'*' signs below alignment denote**:	diagonal similarity (max. similarity is represented by 10 '*' signs)
**Alignment output:**	complete alignment is shown<br># nucleic acids per line: 50
**TF output:**	common TF matches located in aligned regions (common to 3 (100.0 %) sequences)
**Family matches:**	yes
**MatInspector library:**	Matrix Family Library Version 6.1 (June 2006)
**Selected groups** (core/matrix sim)	• ALL user_defined.lib (0.75/Optimized)<br>• ALL vertebrates.lib (0.75/Optimized)

Aligned Sequences:

No.	Sequence Name	Sequence Description
1	P876208	P876208 sym=NDUFA4|loc=Loc4697|taxid=9606|spec=Homo sapiens|chr=7|ctg=NT_007819|str=(-)|start=10274926|end=10275986|len=1061|tss=501,552,561|comm=NADH dehydrogenase (ubiquinone) 1 alpha subcomplex, 4, 9kDa; (AK024837/552/gold;AK130950/561/gold;NM_002489/501/silver;)
2	P876209	P876209 sym=Ndufa4|loc=Loc17992|taxid=10090|spec=Mus musculus|chr=6|ctg=NT_039340|str=(-)|start=8662756|end=8663771|len=1016|tss=501,505,514,516|comm=NADI dehydrogenase (ubiquinone) 1 alpha subcomplex, 4; (AK005084/533/gold;AK008357/546/gold;AK008506/548/gold;NM_010886/537/silver;)
3	rNDUFA4	rNDUFA4 n3_dna range=chr4:37251038-37252042 5'pad=0 3'pad=0 revComp=TRUE strand=? repeatMasking=none

Alignment (DiAlign format):

Please note that only **upper-case** letters are considered to be **aligned**. For more information, please have a look at the user guide.

V$P53F V$ZF5F V$AHRR V$HESF V$CLOX V$ETSF V$DMTF V$XBBF V$E2FF V$NRSF V$HEAT TSS

alignment position		1 . . . . 1 1 . . . . 2 1 . . . . 3 1 . . . . 4 1 . . . .
**P876208 (NDUFA4)**	1	c g t a a c a c a g g a a g c a g c t g c a g t c t t c t c a a a c c g c a g C C G C T G A C C T T
**P876209 (Ndufa4)**	1	- C C G T T G A C C C C

```
rNDUFA4          1 ---------- ---------- ---------- ---------C CGTTGACCCC
                                                            * **********
                                                            * **********
                                                            * **********
                                                            * **********
                                                            * **********

alignment position   51........ 61........ 71........ 81........ 91........
P876208 (NDUFA4)  51 AGCGGCAAGA GGCCCGACCT GCCCTCCAGg cgcccGGCGC TCGGCTCCCA
P876209 (Ndufa4)  12 GGCGGCTGGA GACTCGACCT GTCCTGCGGg atcTGGCGC TGAGCCTGCA
rNDUFA4           12 AGCGGCTGGA GACTCGACCT GTCCTGCGGg attTGGCGC TGCGCCTGCA
                     ********** ********** *********    ****** **********
                     ********** ********** *********    ****** **********
                     ********** ********** *********    ****** **********
                     ********** ********** *********      **** **********
                     ********** ********** *********      **** **********
                                                          **** **********

alignment position   101....... 111....... 121....... 131....... 141.......
P876208 (NDUFA4) 101 GAGGCGCCGC GCAGCTCCCT Cagcggcagt cacagctggC CAGCGCGCAC
P876209 (Ndufa4)  62 GAGGCGCCTG GCAGCTCCCT CcGGGGCCCT CCGGGCGGCG CGGCGCGCGC
rNDUFA4           62 GAGGCGCCTG GCAGCTCCCT CtGGGGCTCT CTGAGAGGCC CAGCGCGCGC
                     ********** ********** * ******** ********** **********
                     ********** ********** *                   * **********
                     ********** ********** *                        ********
                     ********** ********** *
                     ********** ********** *
                     ********** ********** *

alignment position   151....... 161....... 171....... 181....... 191.......
P876208 (NDUFA4) 151 CCGGCACGCG CGCCCCCA-G GGCCTCTGCG CGCGGACCCG GCTCTCAGGC
P876209 (Ndufa4) 112 CCCACACGCG CGCCCCCAAG CGCGTCCACG CGCGGCCCTG GCCGGGAGGC
rNDUFA4          112 ACCGCACGCG CGCCCCCAAG CGCGTCAGCG CGCGGCCCAG GCCTAGAGGC
                     ********** ********** ********** ********** **********
                     ********** ********** ********** ********** **********
                     ********** ********** ********** ********** ***  *****
                         ****** ********    ********* ********** ***  *****
                         ****** ********

alignment position   201....... 211....... 221....... 231....... 241.......
P876208 (NDUFA4) 200 GCCGCGGGAG Aggcccccta CTCACGGAGC GCGCGGCCCG TGCGCCCACG
P876209 (Ndufa4) 162 GCCGCTAAGG AAGCCGCG-- CCCGCCGAGC GCACTGCCAG CGGACCGCCG
rNDUFA4          162 GCCGCTAGGG AAGCCGCG-- CTCGCCAAGC GCACTtCCAG CCGACCGCCG
                     ********** ********   ********** ********** **********
                     ********** ********   ********** *****  **** **********
                     ********** ********   ********** *****  **** **********
                     ****

alignment position   251....... 261....... 271....... 281....... 291.......
P876208 (NDUFA4) 250 ctccgaccgc attcctcagt acgtggcacc CTTGGAAAGT GCCTGTCTGG
P876209 (Ndufa4) 210 GATGACCCTG GTCTATACGC ATGCcGT--- CCTGAAAAGT GCCTGTCTCA
rNDUFA4          210 GATGACCCTG ATCTACACGC ATGCtGT--- CCTGAAAATT GCCTGTCTTA
```

```
                          ********** ********** ****  **             ********** **********
                          ********** ********** ****  **             ********** **********
                          ********** ********** ****  **             ********** **********
                                                                     ********** **********

alignment position        301....... 311....... 321....... 331....... 341.......
P876208 (NDUFA4)     300  TCAATCGaag agtcctcgtt tcgac-C--- ------AAAA AAATTCCCCT
P876209 (Ndufa4)     257  CCAGTCGAGA CGCTCTGcct gctatgcaaa aaataaAAAA ATAAACCTAT
rNDUFA4              257  TCAGTCGAGA CGCTCTGatt tctgtgC--- ------AAAA ATAATCCTAT
                          ********** *******          *          **** **********
                          ********** *******                     **** **********
                          ********** *******                     **** **********
                          *******                                **** **********

alignment position        351....... 361....... 371....... 381....... 391.......
P876208 (NDUFA4)     340  CAGAAATATG CATGGCACGG CAATGAaggc atttcactat gaagtggaat
P876209 (Ndufa4)     307  CAAAAAGATG CATGGTGGAG AACTGAtgga tcatgAACTC AAAAATGCGA
rNDUFA4              298  CAAAAATATG CATAGTGGAG AACTaaagga tgtttAGCTT AACAATGGGA
                          ********** ********** ******          ***** **********
                          ********** ********** ****
                          ********** ********** ****
                          ********** ***

alignment position        401....... 411....... 421....... 431....... 441.......
P876208 (NDUFA4)     390  gatgatggtc cgaccagGTC AGGACGAACA TATGAGGGGT TAg-ATCTAA
P876209 (Ndufa4)     357  AGAAGTGGTC GGGACA-TTT AGGGCaatCG CGCGAGGGCT GACTACCTAT
rNDUFA4              348  AGAAGTGCTG GTACCA-GTT AGGGCGACCG CGCGAGGGCT GAACACCCAT
                          ********** ****** *** ********** ********** **********
                                                        ** ********** **********

alignment position        451....... 461....... 471....... 481....... 491.......
P876208 (NDUFA4)     439  GCAGGCACTA TTAAAGACAG TCGCATCCGG GCCCTCCTGC CGCAAGCTGC
P876209 (Ndufa4)     406  TCAGGCACCT TAGAGGACAG CCGCATCCGG GCTTTCCTGC TGCAGGCTGC
rNDUFA4              397  TCAGGCACCA TAGAGGACAG CCGCATCCGG GCTTTCCTAC TGCAGGCTGC
                          ********** ********** ********** ********** **********
                          ********** ********** ********** ********** **********
                           ********* ********** ********** ********** **********
                                         ****** ********** ********** **********
                                         ****** ********** ********** **********
                                          ***** ********** ********** **********
                                          ***** ********** **  ****** **********

alignment position        501....... 511....... 521....... 531....... 541.......
P876208 (NDUFA4)     489  CCTCCCAGCC AAGGGTCCTT CAGGTAGGAG GTCCTGGTGG ACTTTGGAAG
P876209 (Ndufa4)     456  CCTCagcGCC AGGGGTCCTT CAGGTAGGAG GTCCTGGTGG ACTTTGGACG
rNDUFA4              447  CCTCCCAGCC AGGGGTCCTT CAGGTAGGAG GTCCTGGTGG ACTTTGGACG
                          ********** ********** ********** ********** **********
                          ********** ********** ********** ********** **********
                          ****   *** ********** ********** ********** **********
                          ****   *** ********** ********** ********** **********
                          ****   *** ********** ********** ********** **********
                          ****   *** ********** ********** ********** **********
                          ****   *** ********** ********** ********** **********
                                     ****** ********** ********** *******
                                     ****** ********** ********** *******
```

```
                                                                  ****  *******
alignment position        551....... 561....... 571....... 581....... 591.......
P876208 (NDUFA4)     539  TCCGTAGTGT CTCATTGCAG ATAATTTTTA GCTTAGGGCC Tggtggctag
P876209 (Ndufa4)     506  TCCGCTCAGC CAGGTTGCAG AAGCGGCTTA GCGTGTGTCC TAATCTTCTC
rNDUFA4              497  TCCGCTCAGC CAGGTTGCAG AAGCGGTTTA GTGTGTGTCC TAATCTTCTC
                          ********** ********** ********** ********** **********
                          ********** ********** ****** *** ********** **********
                          ********** ********** ******
                          ********** ********** ******
                          ****
                          ****
                          ****

alignment position        601....... 611....... 621....... 631....... 641.......
P876208 (NDUFA4)     589  gtcggttctc tcctttccag tcggAGACCT CTGCCGCAAA CATGCTCCGC
P876209 (Ndufa4)     556  TCTgcGTGTA GGT------- ----AGGCCT GTGCCGCAAA CATGCTCCGC
rNDUFA4              547  TCTcgGTGTA GGT------- ----AGGCCT GTGCCGCAAA CATGCTCCGC
                          ***  ***** ***            ****** ********** **********
                          ***  ***** ***            ****** ********** **********
                               ***** ***            ****** ********** **********
                               ***** ***            ****** ********** **********
                                                    ****** ********** **********
                                                    ****** ********** **********
                                                    ****** ********** **********
                                                    ****** ********** **********

alignment position        651....... 661....... 671....... 681....... 691.......
P876208 (NDUFA4)     639  CAGATCATCG GTCAGGCCAA GAAGCATCCG AGCGTAAGTT Cgtgaccgct
P876209 (Ndufa4)     595  CAGATCCTCG GGCAAGCCAA GAAGCATCCC AGCGTAAGTT CACGgTCTTT
rNDUFA4              586  CAGATCCTCG GGCAAGCCAA GAAACATCCC AGCGTAAGTT CACGaTCTTT
                          ********** ********** ********** ********** **** *****
                          ********** ********** ********** ********** **** *****
                          ********** ********** ********** ********** ****
                          ********** ********** ********** ********** *
                          ********** ********** ********** ********** *
                          ********** **** ***** ********** ********** *
                          ****** *** **** ***** ********** ********** *
                          ******

alignment position        701....... 711....... 721....... 731....... 741.......
P876208 (NDUFA4)     689  ctcctaatgt tttcccttgt ccttcttgcg tcgGGGTGCG CCTCTTTCTG
P876209 (Ndufa4)     645  GCCAGGGTGG CCTCTTAGCT GT-------- ---GGTTGCT CCTGCCCGTG
rNDUFA4              636  GCCAGAGTGG GCTCTTAGCT GT-------- ---GATTGCG CCTTCCCGTG
                          ********** ********** **            ******* **********
                          ********** ********** **            ******* **********
                                                              ******* *** ******
                                                                 **** *** ******
                                                                          ******

alignment position        751....... 761....... 771....... 781....... 791.......
P876208 (NDUFA4)     739  TTCCCTGGGA ACGTCCTCAT TTacagccac ttaccgtccg tcagaccaga
P876209 (Ndufa4)     684  TCCCCTGGGG ACGTTCTCTT TTCTCTGTTc cttgggttta cattctgttG
rNDUFA4              675  TCCCCTGGGG ACGTTCTCTT TTCTCTGTTt ctgag----- ---------G
```

```
                        ********** ********** *********                     *
                        ********** ********** *********                     *
                        ********** ********** *********
                        ********** ********** *********
                        ********** ********** **

alignment position           801....... 811....... 821....... 831....... 841.......
P876208 (NDUFA4)        789  ccctgcctcg cctagtgttt gtcatcttag ttgtcccagg cctctgtgac
P876209 (Ndufa4)        734  TTGGGATGAC CTGATTGCTT GAAGCCTCTA CCTGGGGTGg ag--------
rNDUFA4                 711  TTGGCCTAAC CTGATAGCTC GAAGTCTCTA CTGGGGGTGg ggaagg----
                             ********** ********** ********** *********
                             ********** ********** ********** *********

alignment position           851....... 861....... 871....... 881....... 891.......
P876208 (NDUFA4)        839  cctctacgcc atgcattGTG GGGTTTTGTC TGACAGGAGG AAGGAGAcgg
P876209 (Ndufa4)        776  ---------- -------GTG AGGTTGCCTG AAGCAGAAGG TGGCAGAGAT
rNDUFA4                 757  ---------- ---------- -GGTTGCCTG AAGCCGAAGG TGTCAGACAT
                                               *** ********** ********** **********
                                                    ********* ********** **********

alignment position           901....... 911....... 921....... 931....... 941.......
P876208 (NDUFA4)        889  acactgctcg gaaaaaaaac ctagtttttt ctagtTAGTG AATATTGGAA
P876209 (Ndufa4)        809  CAGGGAgAAA GGTAAACA-- ---------- -----TTCTG AGTGTTGGAA
rNDUFA4                 786  CAGGGAaAAA GGTAAACA-- ---------- -----TTCTG AGTGTTGGAA
                             ****** *** ********                  ***** **********
                             ****** *** ********                  ***** **********
                                    *** ********                  ***** **********
                                    *** ********                  ***** **********

alignment position           951....... 961....... 971....... 981....... 991.......
P876208 (NDUFA4)        939  CAGTCTGGTG GCCGCTCTCT tttgcaggtt gtgcggtCCA GGAGGGATAG
P876209 (Ndufa4)        842  CACTGTGGC- -------TCT CAGATTACTT GGTCC--CAA GAGGGGTGAG
rNDUFA4                 819  CACTGTGGCC ACCGATCTCT CAGGTTACTT GGTCC--CAA GAAGGGTGAG
                             ********** ********** ********** *****  *** **********
                             *********         *** ********** *****  *** **********
                             *********         *** ********** *****  *** **********
                             *********

alignment position           1001...... 1011...... 1021...... 1031...... 1041......
P876208 (NDUFA4)        989  ATGTGCGCTG TGTgGGTCAG AGACTAGCAT AGGTCAGGTT CCCTATGtgt
P876209 (Ndufa4)        882  TGGTACGTTG AGT-GATTAG AATCCAAGAG AGGCCAAGTT TGTTATGCtg
rNDUFA4                 867  TGGTAtaTTG AGT-GATTAG AATCCAAGAG AGACCAAGTT TGTTATGCtg
                             ********** *** ****** ********** ********** ********
                             *****  *** *** ****** ********** ********** ********
                             *****  *** *** ****** ********** ********** ********
                                    *** *** ****** ********** ********** ********

alignment position           1051...... 1061...... 1071...... 1081...... 1091......
P876208 (NDUFA4)        1039 tataccatct ctggaggttc ttg------- ---------- ----------
P876209 (Ndufa4)        931  -CTCTTTGCG GCTTTTAGAG TCACTGcATT GCACCACCAA ACCTTCAGAA
rNDUFA4                 916  cCTTTTTGAG GCTATTATAT TCACCGgATT GCACCACCAA GCCTTCAGAA
```

```
                     ** ******* * *  *** ******* * *  *** * **      * * *  * * * * * * * * *  * * * * * * * * * *
                                                                    * * *  * * * * * * * * *  * * * * * * * * * *

alignment position   1101.......  1111.......  1121.......  1131.......
P876208 (NDUFA4)  1062 ----------  ----------  ----------  ----------
P876209 (Ndufa4)   980 GTGCAAGGGa  ---TCGTCAT  CGTGTAATCC  CCACTTTGCT
rNDUFA4            966 ATACAAGGGg  tcgTCGTCAT  CTTGTAATCA  TAACTTTGCT
                       *********      *******  **********  **********
                       *********
```

Pairwise similarities:

For each pairwise alignment, the similarity (relative to the maximum similarity) and the number of identical nucleic acids (in % of shorter sequence) is given. Maximum values are underlined.

	P876209 (1016 bp)	rNDUFA4 (1005 bp)
P876208 (1061 bp)	0.238<br>38 %	0.262<br>40 %
P876209 (1016 bp)		<u>1.000</u><br><u>85 %</u>

Please note that the **similarity value 1.000** marks only the two **most similar** sequences, it does not necessarily mean that these sequences are identical.

Extract Alignment Region	
**Positions in alignment**	from: to:
**Output file**	dialign.seq

Extract Aligned Sequences

For comments, questions, or bug reports, please contact support@genomatix.de.

© Genomatix Software GmbH 1998-2006 - All rights reserved.

GEMS Launcher 4.3.0

License Agreement

Genomatix-Logo Logout Personal Messages GEMS-Logo
Logout Personal Messages

Genomatix MainGEMSElDoradoGene2PromoterBiblioSphere
Frequently asked questionsYour ResultsYour SequencesYour ProtocolHelp

GEMS Launcher Task: *DiAlign TF*: Multiple alignment plus TF sites
working on promoters.seq (3 seq.)

[Alignment] [Pairwise similarities]

DiAlign professional TF Release 3.1 December 2004 **Mon Jun 6 23:08:24 2005**

Solution parameters:

**Sequence file:**	promoters.seq (3 seq.)
**Type of sequences:**	nucleotide sequences
**Threshold T:**	0.00
**'*' signs below alignment denote:**	diagonal similarity (max. similarity is represented by 10 '*' signs)
**Alignment output:**	complete alignment is shown<br># nucleic acids per line: 50
**TF output:**	common TF matches located in aligned regions (common to 3 (100.0 %) sequences)
**Family matches:**	yes
**MatInspector library:**	Matrix Family Library Version 5.0 (February 2005)
**Selected groups** (core/matrix sim):	• ALL user_defined.lib (0.75/Optimized)<br>• ALL vertebrates.lib (0.75/Optimized)

Aligned Sequences:

No.	Sequence Name	Sequence Description	Length
1	P921898	sym=NDUFA5|loc=Loc4698|taxid=9606|spec=Homo sapiens|chr=7| ctg=NT_007933|str=(-)|start=48380931|end=48382034|len=1104| tss=501,604| comm=NADH dehydrogenase (ubiquinone) 1 alpha subcomplex, 5, 13kDa; (AK022209/645/gold;NM_005000/542/bronze;)	1104 bp
2	P921899	sym=Ndufa5|loc=Loc68202|taxid=10090|spec=Mus musculus|chr=6| ctg=NT_039340|str=(-)|start=21360789|end=21361930|len=1142| tss=501,522,608,609,618,620,629,632,633,636,641,642| comm=NADH dehydrogenase (ubiquinone) 1 alpha subcomplex, 5; (AK011579/642/gold;AK019201/632/gold;AK008155/632/gold; AK008414/636/gold;AK011947/642/gold;AK008156/636/gold; AK009985/618/gold;AK019207/641/gold;AK019215/632/gold; AK010090/632/gold;AK019242/633/gold;AK010092/618/gold; AK019324/522/gold;AK019430/620/gold;AK009338/618/gold; AK008286/636/gold;AK019245/620/gold;AK009461/632/gold; AK019084/632/gold;AK009608/632/gold;AK009642/620/gold; AK019354/629/gold;AK019364/618/gold;AK019291/632/gold; AK009652/632/gold;AK009840/618/gold;AK019359/609/gold; AK009493/632/gold;AK009396/632/gold;AK019368/620/gold; AK010024/632/gold;AK009775/608/gold;NM_026614/501/bronze;)	1142 bp
3	P921900	sym=Ndufa5|loc=Loc25488|taxid=10116|spec=Rattus norvegicus| chr=4|ctg=NW_047689|str=(-)|start=21329033|end=21330033| len=1001|tss=501| comm=NADH dehydrogenase (ubiquinone) 1 alpha subcomplex 5; (NM_012985/508/bronze;)	1001 bp

Alignment (DiAlign format):

Please note that only **upper-case** letters are considered to be **aligned**. For more information, please have a look at the user guide.

V$NRF1 U$NRF1 V$SP1F V$HOXC V$RORA V$FKHD V$NF1F

```
alignment  position 1.........  11........  21........  31........  41........
P921898          1 gagg------ ---------- ---------- ---------- ----------
P921899          1 caaagaacaC ATGCATCTTT TATAAAGCCA TGATTCCACA TTCTAGAACA
P921900          1 ---------C ATGCATCTTT TATAAAGCCA TGATTCCACA TTCTAGAACA
                            * ********** ********** ********** **********
                            * ********** ********** ********** **********
                            * ********** ********** ********** **********
                            * ********** ********** ********** **********
                            * ********** ********** ********** *********
                            * ********** ********** ********** *********
                            * ********** ********** ********** *********

alignment  position 51........  61........  71........  81........  91........
P921898          5 ---------- ---------- ---------- ---------- ----------
P921899         51 CACTCAACAA TGACAGATGT TCTTGGGATC TGGAggGACA GAGAACAGGG
P921900         42 CACTTAACAA TGACAGATGT TCTTAGGACC TGGAaaGACG GAGAATAGAG
                   ********** ********** ********** ****  **** **********
                   ********** ********** ********** ****  **** **********
                   ********** ********** ********** ****  **** **********
                   ********** ********** ********** ****  **** **********

alignment  position 101.......  111.......  121.......  131.......  141.......
P921898          5 ---------- ---------- --TAGGAAAA AGTGGTATGG TCTGATATCA
P921899        101 ACTATGACCT CATAAACACC CAcTGGAACA AAGACTACGA CTTCATAACC
P921900         92 ACTATGACCT CATAAACTCC CATTGGAACA AAGACTATGA CTTCATAACC
                   ********** ********** ********** ********** **********
                   ********** ********** **  ****** ********** **********
                   ********** ********** **  ****** ********** **********
                   ********** ********** **  ****** ********** **********
                                            ******* ********** **********
                                            ******* ********** **********

alignment  position 151.......  161.......  171.......  181.......  191.......
P921898         33 TTTCATctgg aaa------- ---------- ---------- ----------
P921899        151 TTTCATTCAt CAGTCTCCTC AAGAGAAAAC GGATGCATCA GATGGTTAAT
P921900        142 TTTCATTCAc CAGCCTCCAC AGGAGCAAAC GGATATATCA GATGGTTAAT
                   ********** ********** ********** ********** **********
                   *********  ********** ********** ********** **********
                   *********  ********** ********** ********** **********
                   *********  ********** ********** ********** **********
                   *********
                   ******

alignment  position 201.......  211.......  221.......  231.......  241.......
P921898         46 ---------- ---------- TAAGATGATG TATTTCAGCC AGTGAAATCG
P921899        201 TCCACTTTAT AATTTCCACT TATGAGTATG CATTCACACA c-GTGGAGGG
P921900        192 TCCACTTTAT AGTTCCCATT TATGAATATG TATTCACGCA AGGGGGAGAG
                   ********** ********** ********** ********** **********
                   ********** ********** ********** **********
                   ********** ********** ********** **********
                   ********** ********** ********** **********

alignment  position 251.......  261.......  271.......  281.......  291.......
P921898         76 Aaaagagcga tggaaaatga cctcacactc catagggctt tggttcgtaa
P921899        250 AAAGCATTCT TCATGGTGCT C--------- ---------- ----------
P921900        242 AGCATTTTTT TCATGATGCT C--------- ---------- ----------
                   ********** ********** *

alignment  position 301.......  311.......  321.......  331.......  341.......
P921898        126 gacacagaga atactgatat cctgtgcga agaactaact tgctgttgag
P921899        271 ---------- ---------- ---------- ---------- ----------
P921900        263 ---------- ---------- ---------- ---------- ----------
```

```
alignment position 351.......  361.......  371.......  381.......  391.......
P921898   176      agatcaattc  agaggatgct  caAGCTCCGT  TATCACTTTC  ATTTTACTAA
P921899   271      ----------  ----------  --AGGcctgc  TTCCACCCTC  ATTTCCATAA
P921900   263      ----------  ----------  --AGGTCCGT  TTCCACCCTC  ATTTCAATCA
                                             ********  **********  **********
                                             ********  **********  **********
                                                       **********  **********
                                                       **********  **********
                                                          *******  **********

alignment position 401.......  411.......  421.......  431.......  441.......
P921898   226      ACTGAGAAAG  gaaggttgag  gaATGCATCA  ACGGTTAAGC  ACAGTGCAGG
P921899   299      CCTGAGGAAG  TCCGggTAAA  ATAGCAGTCG  AAGGCTGAGC  AAAATCAAAG
P921900   291      GCTGAGGAAG  TGCGcaTAAA  ACATCAATCA  AAGGCTGAGC  AAAATCAAAG
                   **********  ****  ****  **********  **********  **********
                   **********  ****  ****  **********  **********  **********
                   **********  ****  ****  **********  **********  **********
                   **********        ****  **********  **********  **********
                   **********                ********  **********  **********

alignment position 451.......  461.......  471.......  481.......  491.......
P921898   276      TTCCTACAGC  GACTgg----  --AGTTTTTT  GGTAGTTGGG  AGTGTTTCcc
P921899   349      TCCCTGCAGT  GACTACGTTG  CAAGTTTTTC  CGTAGCGTAG  CGCTTTTCTT
P921900   341      TCCCTG-AGT  GACTACATTG  AAAGTTTTTC  -GTAGCGCAG  CGTTTTTCTT
                   **********  **********  **********  **********  **********
                   ****** ***  **********  **********   *********  **********
                   ****** ***  ****                     *********  **********
                   ******
                   ******

alignment position 501.......  511.......  521.......  531.......  541.......
P921898   320      tcaatctttg  ggcaagccgt  cagcagtggc  aggaacagga  acagacagct
P921899   399      AACCTTTAGT  ACT--CAGAG  ACGTCAGTCG  GTCTCTACTA  CACCAAAGCC
P921900   389      AACCTTTAGT  CCTaaCAGAG  GCGTCAGTCT  GTCTCTGTTG  CGCCAAAACC
                   **********  ***  *****  **********  **********  **********
                   **********  ***  *****  **********  **********  **********
                   **********  ***  *****  **********  **********  **********

alignment position 551.......  561.......  571.......  581.......  591.......
P921898   370      gggaaaccct  ttctttcccc  aatcgtcttc  tagcttagct  ttaggcttgt
P921899   447      CTgCGTGGAG  CCGCTTGTGT  GAGCT-----  ----------  ----------
P921900   439      CTaCGTGGAG  CCACGCGTAT  GAGCT-----  ----------  ----------
                   ** *******  **********  *****
                   ** *******  **********  *****
                   ** *******  **********  *****
                      *******  **********  *****

alignment position 601.......  611.......  621.......  631.......  641.......
P921898   420      atgcccatcG  CGCATGCGTC  AATTTTTCTC  TTTTAAGATG  GGCGGGGCag
P921899   472      ---------G  CGCAGGCGCA  AATCTACCTC  TCCTAGGTGG  GCGGGAGCTG
P921900   464      ---------G  CGCAGGCGCA  AATCTCTCCC  TCCTAGGTGG  GCGGGAGTTA
                            *  **********  **********  **********  **********
                            *  **********  *****    *  **********  **********
                            *  **********  *****    *  **********  **********
                            *  **********  *****    *  **********  ********
                            *  **********  *****

alignment position 651.......  661.......  671.......  681.......  691.......
P921898   470      agtctttgct  cctttggcga  tCCTGAAGGG  GTGGAGCTAA  GCTGTTTCCA
P921899   513      CCCCTACCCa  gcccgagc--  --------GG  TTGGAGCCAA  GAGTGTTCTT
P921900   505      CCCCTAGCCc  cg--------  -CCTGAGAGG  TGGGAGCCAA  GTGCTTTCTT
                   *********                *********  **********  **********
                   *********                *********  **********  **********
                   *********                       **  **********  **********
                                                   **  **********  **********
                                                   **  **********  **********

alignment position 701.......  711.......  721.......  731.......  741.......
```

```
P921898        520 GGGTGACAGA GTGGCgacct cggtggTCGA TTGAGCAGGT CTGAGAATTG
P921899        553 AGGTGACTCA GGGGCAGCat cgACGCTCGA TTGAGCGGGC TTGGGAAAGG
P921900        546 GGGTGACACA GGAGCAGCtt ctACGTTCGA TTGAGCGGGC CTGGGAAAGG
                   ********** ********     ******** ********** **********
                   ********** ********     ******** ********** **********
                   ********** ********     ******** ********** **********
                   ********** *****        ******** ********** **********
                   ********** *            ******** ********** **********
                                                ****  ********** **********
                                                ****  ********** **********
                                                ****  ********** **********
                                                ****  ********** **********

alignment position 751....... 761....... 771....... 781....... 791.......
P921898        570 TTCCCAAAGG GTTGTGcgtc accgagtcgt tgGCGCTGTC ATGGCGGGTG
P921899        603 TTCCCAGAGG GCTGTGCGCT GGAGGCGCTG G-ACGCTGTC ATGGCGGGCT
P921900        596 TTCCCAGAAG GCcgcGCGCT GGAGTCTCTG A-GCGCAGTC ATGGCGGGCT
                   ********** ********** ********** * ******** **********
                   ********** ********** ********** * ******** **********
                   ********** ********** ********** * ******** **********
                   ********** **   *                  ******** **********
                   ********** **   *                  ******** **********
                   ********** **                      ******** **********
                   ********** **                       ******* **********
                   ********** *                        ******* **********
                   ********** *                        ******* **********

alignment position 801....... 811....... 821....... 831....... 841.......
P921898        620 TGCTGAAGAA GGTGAGacga aTGGAGGTCA CTGTtggaat ttagactgtg
P921899        652 TGCTGAAAAA GGTGAGCGTG -TGGAGGTCA CCGTGATGCT GcgaatgGGG
P921900        645 TGGTGAAGAA GGTGAGCGTC -TGGAGGTCA CCATGGTGCT Gaggac-GGG
                   ********** **********  ********* ********** *      ***
                   ********** **********  ********* ********** *      ***
                   ********** **********  ********* ********** *      ***
                   ********** **********  ********* ********** *
                   ********** ******
                   ** ******* ******
                   ** ******* ******
                   ** ******* ******
                      ******* ******

alignment position 851....... 861....... 871....... 881....... 891.......
P921898        670 ggggtagact cagtccgata gccctacttc ccgcgggtgt tgacccctac
P921899        701 ACCGTAGGCA GCCGCGTCTC CCTCATCGGT Gggcagagac -------GCT
P921900        693 GCGGTAGGCA GCCTTGTCTC CCTCATCCGT Gttaagagga tggagaaGCT
                   ********** ********** ********** *                ***
                   ********** ********** ********** *                ***
                   ********** ********** ********** *                ***
                                                                     ***
                                                                     ***
                                                                     ***

alignment position 901....... 911....... 921....... 931....... 941.......
P921898        720 tgtcgcgggc tcggcggtgc tttTCGGGGA CTCGTTCTGC ATGCCTTACC
P921899        744 AAGAGGAGGG G--------- ---TTGGGGG AGTGTTCCGC TGTCCTGGCA
P921900        743 AAGAGGACGG G--------- ---TTGGGGG AGTGTTCCGC TGTCCTGACA
                   ********** *             ******* ********** **********
                   ********** *             ******* ********** ******* **
                   ********** *             ******* ********** ******* **
                   ********** *             ******* ********** ******* **
                   ********** *             ******* ********** ******* **
                   ********** *             ******* ********** *******

alignment position 951....... 961....... 971....... 981....... 991.......
P921898        770 GGGTAGGGCA GGttagtggt tccgctccga gagtcaggaa aaaagctcca
P921899        782 GGGGGCGACA GGGTGCGTAG TTGTGTCGGG AGGGTCGGct aaccag----
P921900        781 GGGGGCGACA GGGTTCGTAG TTGTGTCTGG AAGTTCGGct aatcagcggg
```

```
                   ********** ********** ********** *********
                   ********** ********** ********** *********
                   ********** ********** ********** *********
                   ********** ********** ********** *********
                   ********** **

alignment position 1001...... 1011...... 1021...... 1031...... 1041......
P921898        820 aacaagcgag actgagagac cagtagctgg ctttagacca gttttaGCCG
P921899        828 ---------- ---------- ---------- ---------- ----------
P921900        831 ccaggactgg gg-------- ---------- ---------- ------GCCC
                                                                     ****

alignment position 1051...... 1061...... 1071...... 1081...... 1091......
P921898        870 GGGGATTCGT GGTTTTGTAT TTCCTTAGCG tgctggatgt atggaaaccg
P921899        828 ---------- ---------- ---------- ---------- -GGGATTACG
P921900        847 CGGGATGGGC GGGCTGGCTT TTCCTGCGCG ctgttacgcg tGGGATGACG
                   ********** ********** **********             *********
                                                                *********
                                                                *********
                                                                *********

alignment position 1101...... 1111...... 1121...... 1131...... 1141......
P921898        920 taGTAAACAG CAtgaaTCGT GTTTTGTTTT TGAACAGACC ACTGGCCTTG
P921899        837 --GTAAACAT CACGG-TCCT GTTTTGTCTC TGCACAGACA ACTGGCCTGG
P921900        897 --GTAAACAG CACGG-TCTT GTTTTGTTTC TGAACAGACT ACTGGCCTGG
                     ******** ***** **** ********** ********** **********
                     ******** ***** **** ********** ********** **********
                     ******** ***** **** ********** ********** **********
                     ******** ***** **** ********** ********** **********
                                    **** ********** ********** **********
                                    **** ********** ********** **********
                                    **** ******* ** ********** ******** *
                                    **** ******* ** ********** ******** *
                                    **** ******* ** ********** ******** *

alignment position 1151...... 1161...... 1171...... 1181...... 1191......
P921898        970 TGGGATTGGC TGTGTGCAAT ACTCCTCACG AGGTATGTAc ctttgttctt
P921899        884 TGGGGTTGGC GGTGTGCGAC ACTCCACACG AGGTAAGTGC GACTTTTTGT
P921900        944 TGGGATTGGC CGTTTGCGAC ACTCCACACG AGGTAAGTAC GGCTTTTTGT
                   ********** ********** ********** ********** **********
                   ********** ********** ********** ********** **********
                   ********** ********** ********** ********** **********
                   ********** ********** ********** ********** **********
                   ********** ********** ********** ********** **********
                   ********** ********** ********** *********
                   ********** *** ****** ********** *********
                   **** ***** *** ****** ********** *********
                   **** ***** *** ****** ********** ********
                                  ****** ********** ********

alignment position 1201...... 1211...... 1221...... 1231...... 1241......
P921898       1020 tcttcgttct tgaaTTCCCA AGGAAGACTa aattcctgtc actttgctta
P921899        934 GTCgtcc--- ----TTCTCA AGCAAGACTg tgatcgaccg aacttgccc-
P921900        994 GTCtttgc-- ---------- ---------- ---------- ----------
                   ***            ****** *********
                   ***
                   ***
                   ***
                   ***

alignment position 1251...... 1261...... 1271...... 1281...... 1291......
P921898       1070 TTGCAGGGTT AACGGGAtac agatgtttca agccc----- ----------
P921899        976 TTGCAAGATT AACGAGAccc ccgtgagctc tggcctccgg ggcagcgctg
P921900       1002 ---------- ---------- ---------- ---------- ----------
                   ********** *******

alignment position 1301...... 1311...... 1321...... 1331...... 1341......
P921898       1105 ---------- ---------- ---------- ---------- ----------
P921899       1026 agcttcgctg accccgtggc atcctttttg caatgcctcc ttttgccttc
```

```
P921900     1002 - - - - - - - - - -  - - - - - - - - - -  - - - - - - - - - -  - - - - - - - - - -  - - - - - - - - - -

alignment position 1 3 5 1 . . . . . .  1 3 6 1 . . . . . .  1 3 7 1 . . . . . .  1 3 8 1 . . . . . .  1 3 9 1 . . . . . .
P921898     1105 - - - - - - - - - -  - - - - - - - - - -  - - - - - - - - - -  - - - - - - - - - -  - - - - - - - - - -
P921899     1076 g c c g t g a c c t  c t t a a t t a a g  a c g c g t t t c t  t c a c g g c a t t  c t c t t a g c g t
P921900     1002 - - - - - - - - - -  - - - - - - - - - -  - - - - - - - - - -  - - - - - - - - - -  - - - - - - - - - -

alignment position 1 4 0 1 . . . . . .  1 4 1 1 . . .
P921898     1105 - - - - - - - - - -  - - - - - - -
P921899     1126 c a g t g g t g t g  g a g t a t t
P921900     1002 - - - - - - - - - -  - - - - - - -
```

Pairwise similarities:

For each pairwise alignment, the similarity (relative to the maximum similarity) and the number of identical nucleic acids (in % of shorter sequence) is given. Maximum values are underlined.

	P921899 (1142 bp)	P921900 (1001 bp)
P921898 (1104 bp)	0.158<br>31 %	0.177<br>35 %
P921899 (1142 bp)		1.000<br>77 %

Please note that the **similarity value 1.000** marks only the two **most similar** sequences, it does not necessarily mean that these sequences are identical.

Extract alignment region	
**Positions in alignment**	from: to:
**Output file**	dialign.seq

Extract aligned sequences

For comments, questions or bug reports, please contact support@genomatix.de

© Genomatix Software GmbH 1998-2005 - All rights reserved.

License Agreement

GEMS Launcher 4.1.1

Genomatix-Logo | Logout | Personal | Messages | GEMS-Logo
Logout | Personal | Messages

Genomatix Main GEMS ElDorado Gene2Promoter BiblioSphere
Frequently asked questions Your Results Your Sequences Your Protocol Help

GEMS Launcher Task: *DiAlign TF*: Multiple alignment plus TF sites
working on promoters.seq (3 seq.)

[Alignment] [Pairwise similarities]

DiAlign professional TF Release 3.1 December 2004 **Mon Jun 6 23:21:05 2005**

Solution parameters:

**Sequence file:**	promoters.seq (3 seq.)
**Type of sequences:**	nucleotide sequences
**Threshold T:**	0.00
**'*' signs below alignment denote:**	diagonal similarity (max. similarity is represented by 10 '*' signs)
**Alignment output:**	complete alignment is shown<br># nucleic acids per line: 50
**TF output:**	common TF matches located in aligned regions (common to 3 (100.0 %) sequences)
**Family matches:**	yes
**MatInspector library:**	Matrix Family Library Version 5.0 (February 2005)
**Selected groups** (core/matrix sim):	• ALL user_defined.lib (0.75/Optimized)<br>• ALL vertebrates.lib (0.75/Optimized)

Aligned Sequences:

No.	Sequence Name	Sequence Description	Length
1	P855595	sym=NDUFA6|loc=Loc4700|taxid=9606|spec=Homo sapiens|chr=22| ctg=NT_011520|str=(-)|start=21876764|end=21877764|len=1001| tss=501| comm=NADH dehydrogenase (ubiquinone) 1 alpha subcomplex, 6, 14kDa; (NM_002490/577/bronze;)	1001 bp
2	P855596	sym=Ndufa6|loc=Loc67130|taxid=10090|spec=Mus musculus| chr=15|ctg=NT_039621|str=(-)|start=43832732|end=43833737| len=1006|tss=501,506| comm=NADH dehydrogenase (ubiquinone) 1 alpha subcomplex, 6 (B14); (AK002749/558/gold;AK010988/558/gold;AK012328/558/gold; NM_025987/553/bronze;)	1006 bp
3	P855597	sym=na|loc=Loc315167|taxid=10116|spec=Rattus norvegicus| chr=7|ctg=NW_047781|str=(-)|start=1179476|end=1180476| len=1001|tss=501| comm=similar to NADH dehydrogenase (ubiquinone) 1 alpha subcomplex, 6 (B14); NADH dehydrogenase (ubiquinone) 1 alpha subcomplex, 6 (14kD, B14); (XM_235518/597/bronze;)	1001 bp

Alignment (DiAlign format):

Please note that only **upper-case** letters are considered to be **aligned**. For more information, please have a look at the user guide.

V$NRF1 U$NRF1 V$ECAT V$PCAT V$YY1F V$NEUR V$PLAG V$HICF V$E2TF V$NFKB V$CREB V$RORA
U$T3R U$T3Rbis V$E4FF V$STAT V$ETSF

```
alignment
position        1 . . . . . . . . .   1 1 . . . . . . . .   2 1 . . . . . . . .   3 1 . . . . . . . .   4 1 . . . . . . . .
P855595      1  a - - - - - - - - -   - - - - - - - - - -   - - - - - - - - - -   - - - - - - - - - -   - - - - - - - - - -
P855596      1  t c - - - - - - - -   - - - - - - - - - -   - - - - - - - - - -   - - - - - - - - - -   - - - - - - - - - -
```

```
P855597       1 acaagttcgg tggacacaaa cctgcagagg cagagaaatc actgcaagtt

alignment
position          51........ 61........ 71........ 81........ 91........
P855595       2 ---------- ---------- ---------- ---------- ----------
P855596       3 ---------- ---------- ---------- ---------- ----------
P855597      51 ctgaggccaa cctggctggt ctacataagt gagtttaagg tcagccgcaa

alignment
position          101....... 111....... 121....... 131....... 141.......
P855595       2 ---------- ---------- ---------- ---------- ----------
P855596       3 ---------- ----AAAACA ATGGCATGAA GTAAGTAAAC AAA-------
P855597     101 tcagacgaca acgaAAAACA ATGACAATAA GTAAACAAAC AAAcaatatg
                               ****** ********** ********** ***

alignment
position          151....... 161....... 171....... 181....... 191.......
P855595       2 ---------- ---------- ---------- ---------- ----------
P855596      32 ---------- --------CG GGGCTGAGAA ACGCCAAAAA GCTCAAGCCa
P855597     151 gagttttcat tataatccCG GGGCTGAGAA ACGGCACAAA GCTCAAGCCg
                                   ** ********** ********** *********
                                   ** ********** ********** *********

alignment
position          201....... 211....... 221....... 231....... 241.......
P855595       2 ---------- ---------- ---------- ---------- ----------
P855596      64 GAGGCGCTTT TGCGATCGAC ACCATGTCTA CAAGAAAACg gttttccgga
P855597     201 GAGGCGCTTT TGCGATCTAC ACCAAGTCTA GAAGAAAAC- ----------
                ********** ********** ********** *********
                ********** ********** ********** *********
                ********** ********** ********** *********

alignment
position          251....... 261....... 271....... 281....... 291.......
P855595       2 ---------- ---------- ---------- ---------- ----------
P855596     114 agcacataca caatatgctc agcagtacgc acTGTTGCTG CGGAAAGACT
P855597     240 ---------- ---------- ---------- --TGTTTCTG CGGAAAGACC
                                                    ******** **********

alignment
position          301....... 311....... 321....... 331....... 341.......
P855595       2 ---------- ---------- ---------- ---------- ----------
P855596     164 GCCATtgccg cctggcaccg ttatctatgc ccgaggtctt ctcctccgag
P855597     258 GCCATca--- ---------- ---------- ---------- ----------
                *****

alignment
position          351....... 361....... 371....... 381....... 391.......
P855595       2 ---------- --GCCGCCAG GCGGCAGGAc aa-------- ----------
P855596     214 cctgccaacc acGGCGCCTG GCTGAAGGAA CAAACttttg gtaagagcag
P855597     265 ---------- ------CCCG GCTGGAACAA CAAACcg--- ----------
                             ******** ********** *****

alignment
position          401....... 411....... 421....... 431....... 441.......
P855595      22 -------GCC CCACCGCAGC Ccaggcgcca ttatctgcct cagcagttaC
P855596     264 acagagcGCC TCACCGCTGC CTGAAG---- ---------- ---------C
P855597     286 ---------- --ACCGCTGC CGGAAG---- ---------- ---------C
                       *** ********** ******                       *
                             ******** ******                       *
                                                                   *

alignment
position          451....... 461....... 471....... 481....... 491.......
P855595      65 CTCGGGGTCC CGCCACGAGG AGGTTCTTCA AGGCCTGCGc gcccacggac
P855596     291 CTCGACGCTG CGCCTCCTGG TGGTTCCTCA GCGCCGCCGT TGAACGAG--
```

```
P855597    301 CTCGACGCTG CGCTCCCTGG TGGTTCTTCA GCGCCGCGGT CGAAGGAG--
               ********** ********** ********** ********** ********
               ********** ***  ***** ********** ********** ********
               ********** ***  ***** ********** ********** ********

alignment
position       501....... 511....... 521....... 531....... 541.......
P855595    115 actaccaagg agcgcggcgg gcagccggat agcaggacgc tgagggaaac
P855596    339 ---------- ---------- ---------- ---------- ----------
P855597    349 ---------- ---------- ---------- ---------- ----------

alignment
position       551....... 561....... 571....... 581....... 591.......
P855595    165 cccgcagtcg ctcggcgtcc ccggcgccag ctcacgaccc agcgacctcc
P855596    339 ---------- ---------- ---------- ---------- ----------
P855597    349 ---------- ---------- ---------- ---------- ----------

alignment
position       601....... 611....... 621....... 631....... 641.......
P855595    215 tgcggcccga gcgtcccctg gcggcaacgc cggctcgtta gagcgagcga
P855596    339 ---------- ---------- ---------- ---------- ----------
P855597    349 ---------- ---------- ---------- ---------- ----------

alignment
position       651....... 661....... 671....... 681....... 691.......
P855595    265 cagatggtgt agtCCAAAGG CCAAGCGGCG TTTCTGTAGA TGAGCATGCG
P855596    339 ---------- ---CCAACCc cggATCGGTG TTTCCGCGGT AGAGCATGCG
P855597    349 ---------- ---CCAACCA CCAATCGGTG TTTCCGCGGT AGAGCATGCG
                             ******* ********** ********** **********
                             ******     ******* ********** **********
                             ******     ******* ********** **********
                                        ******* ********** **********
                                        ******* ********** **********

alignment
position       701....... 711....... 721....... 731....... 741.......
P855595    315 CAgcacccac cgttcgctca gccagagaga ggcttcCCAG CCAATCCGAG
P855596    376 CATTGCAGAG CCCgaggctt ---------- ------CCAG CCAATCAGAG
P855597    386 CATGGCAGAG CCCgt----- ---------- ---------- CCAATCGGAG
               ********** ***                         **** **********
               ********** ***                              **********
               ********** ***
               ********** ***
               **

alignment
position       751....... 761....... 771....... 781....... 791.......
P855595    365 AGCCTCAGag tcatcctccc gcccacccag cataca---G GCGGGGCGTt
P855596    410 AGCCCTAGTC cagtcagtcc ttccctcTGC ACTGCAGGAG GCACGGCAGC
P855597    411 AGCCCCAGTC aa-------- -------TCC ACCGCAGAGG GCGTGGCGTC
               **********                   *** ********** **********
               ****

alignment
position       801....... 811....... 821....... 831....... 841.......
P855595    412 cctccttagc caatgggaaa agacattcgc ccgcggtccG CACGCGCTGc
P855596    460 GAGTCCCGTg gcaccgCGCG T--------- ---------G CGCACGCTGA
P855597    446 GCGTCCTGTa acgctaCGCG T--------- ---------G CGCGCGCTGA
               *********        **** *                  * **********
                                **** *                  * **********

alignment
position       851....... 861....... 871....... 881....... 891.......
P855595    462 ttgcaaaggg gtggggttgt GGAGTGGATG CTTTGGCAAG ATGGCGGgg-
P855596    492 AGG------- ---------- GGCGGGGCTT CCTCGGCAAG ATGGCGGCCG
```

```
P855597    478 GAG------- ---------- GGCGGGGCTT CCTTAGCAAG ATGGCGGCCG
               ***                   ********** ********** **********
               ***                   ********** ********** **********
                                     ********** ***  ***** **********
                                                     ***** *******

alignment
position       901....... 911....... 921....... 931....... 941.......

P855595    511 -----AGCGG CGTCCGCCAA GCTACTTCT- --ACCGCCAG CACCTTCGTG
P855596    525 CGGCCACCGG CCTGCGCCAA GCTGCTGCTg cgGCAGCCAG TACCTCGGTG
P855597    511 CGGCCAGCGG CCTGCGCCAG GCTGCTTCT- --GCAGCCAG TACCTCGGTG
               ********** ********** *********    ******** **********
               ********** ********** *********    ******** **********
               ********** ********** ******       ******** **********
                    ***** ********** ******       ******** **********
                    ***** ********** ******       ******** **********
                                                       ***** *****  ***
                                                       ***** *****  ***

alignment
position       951....... 961....... 971....... 981....... 991.......

P855595    553 AAGCCCATTT TCAGTCGGGA CATGAACGAG GCCAAGCGGA GGGTGCGCGA
P855596    575 AAGCCCATTT TCAGTCGCGA CCTGAACGAG GCCAAGCGGA GGGTGCGCGA
P855597    558 AAGCCCATTT TCAGTCGGGA CCTGAAAGAG GCCAAGCGGA GGGTGCGCGA
               ********** ********** ********** ********** **********
               ********** ********** ********** ********** **********
               ********** ********** ********** ********** **********
               ********** ********** ********** ********** **********
               ********** ******* ** ********** ********** **********
               ********** ******* ** ********** ********** **********
               ********** ******* ** * ******** ********** **********
                                  ** * ****  *** ********** **********
                                       ****  *** ********** **********
                                             *** ********** **********

alignment
position       1001...... 1011...... 1021...... 1031...... 1041......

P855595    603 GCTCTACCGC GCcTGGTATC GGGAGGTGCC GAACACTGGT GAGAGGTAGC
P855596    625 GCTCTACCGC GCTTGGTATC GGGAGGTGCC GAACACCGGT GAGAGGCAGG
P855597    608 GCTCTACCGC GCTTGGTACC GGGAGGTGCC AAACACTGGT GAGAGGCAGG
               ********** ********** ********** ********** **********
               ********** ********** ********** ********** **********
               ********** ********** ********** ********** **********
               ********** ** ******* ********** ********** **********
               ********** ** ******* ********** ********** **********
               ********** ** ******* ********** ********** **********
               ********** ** ******* ********** ********** **********
               ********** ** ******* ********** ********** ********
               ********** **
               ********** **

alignment
position       1051...... 1061...... 1071...... 1081...... 1091......

P855595    653 GGcttacgtG GGGACCCGGA GGCCGCCGCT CAAGGTcgta gccggcccgg
P855596    675 GGTGCATTCG GGGCGCCGGA GGCACCTGCC CAAGGTctc- ----------
P855597    658 GGTGCATTCA GGGCCCCGGA GTCACCAGTC CAAGGT---- ----------
               ********** ********** ********** ******
               ********** ********** ********** ******
               **       * ********** ********** ******
               **
               **
               **
               **

alignment
position       1101...... 1111...... 1121...... 1131...... 1141......

P855595    703 ccggggtttc tcaTGGGCCC AGCTGAGGTC ACCAACTTGG GCAGAGGCGA
P855596    714 ---------- ---TCGGCCG AGCTGAGGTC ACGTAGTGGG ACAGAAACGA
P855597    694 ---------- ------GCCG GGCTGAGGTC ACCTACTAGG ACAGAAAtGA
```

```
                                        * * * * * * *  * * * * * * * * * *  * * * * * * * * * *  * * * * * * * * * *
                                              * * * *  * * * * * * * * * *  * * * * * * * * * *  * * * * * * *   * *
                                              * * * *  * * * * * * * * * *  * * * * * * * * * *  * * * * * * *   * *
                                                                                                                  * *

alignment
position        1 1 5 1 . . . . .  1 1 6 1 . . . . .  1 1 7 1 . . . . .  1 1 8 1 . . . . .  1 1 9 1 . . . . . .

P855595    753  c t g c g g g t G C  T T C A G G G G A A  G A G G G T C A C C  C G A G C C t a c c  a t g g g c c g g a
P855596    751  G C C C T G G - G C  T T C G G G G G A A  G G G G G T C A C C  C G A G C C A G A G  T G C C C C A C A C
P855597    728  G C C C T G G - G C  T T C G G G G G A A  G G G G G T C A C A  C G g a C C A A A G  T G C C C C A C C C
                * * * * * *   * *  * * * * * * * * * *  * * * * * * * * * *  * * * * * * * * * *  * * * * * * * * * *
                * * * * * *   * *  * * * * * * * * * *  * * * * * * * * * *  * * * * * * * * * *  * * * * * * * * * *
                * * * * * *   * *  * * * * * * * * * *  * * * * * * * * * *  * *     * * * * * *  * * * * * * * * * *
                              * *  * * * * * * * * * *  * * * * * * * * *              * *
                              * *  * * * * * * * * * *  * * * * * * * * *

alignment
position        1 2 0 1 . . . . .  1 2 1 1 . . . . .  1 2 2 1 . . . . .  1 2 3 1 . . . . .  1 2 4 1 . . . . . .

P855595    803  c c t g g c g g c g  g c g c g g t g g t  c a g a c c g g c c  g t g c c t a g c a  t t c g t a c G T G
P855596    800  C G G G A C A G G G  C T T C C G A - - -  - - - - - - - - - -  - - - - - - - - - -  - - - - - - - G C G
P855597    777  C G G G A C A G G G  C T T C C G A - - -  - - - - - - - - - -  - - - - - - - - - -  - - - - - - - G A G
                * * * * * * * * * *  * * * * * * *                                                           * * *
                * * * * * * * * * *  * * * * * * *                                                           * * *
                * * * * * * * * * *  * * * * * * *                                                           * * *

alignment
position        1 2 5 1 . . . . .  1 2 6 1 . . . . .  1 2 7 1 . . . . .  1 2 8 1 . . . . .  1 2 9 1 . . . . . .

P855595    853  C T A T C G G A G C  T T T G G T C T C T  T T T G T C C T T c  g c a a t t g g c g  g g a g g a c g a t
P855596    820  C C C c A G T T G C  T T T G G T G G C C  T T T G T T C C T t  a - T T T C T T A T  G G A G C C C C T G
P855597    797  C C C T A G T C G C  T T T G G T G G C C  T T T G T T C C T a  t g T T T C T T A C  A G A G C C C C A G
                * * * * * * * * * *  * * * * * * * * * *  * * * * * * * * *      * * * * * * * *  * * * * * * * * * *
                * * *   * * * * * *  * * * * * * * * * *  * * * * * * * * *
                * * *

alignment
position        1 3 0 1 . . . . .  1 3 1 1 . . . . .  1 3 2 1 . . . . .  1 3 3 1 . . . . .  1 3 4 1 . . . . . .

P855595    903  g a c t g t c a g t  g a c a c a c t c a  c t t t g t g g g t  g a g g c a g c g g  a g g c a c c g a g
P855596    869  G G A G G G G C G -  - - - - - - - - - -  - - - - - - - - - -  - - - - - - - - - -  - - - - - - - - - -
P855597    847  G G A G G A G C G a  g c a g g g a c t a  t g a g a a a g - -  - - - - - - - - - -  - - - - - - - - - -
                * * * * * * * * *

alignment
position        1 3 5 1 . . . . .  1 3 6 1 . . . . .  1 3 7 1 . . . . .  1 3 8 1 . . . . .  1 3 9 1 . . . . . .

P855595    953  a g g t G A A G T G  A T G T G C C C A G  G G C C C A G G C C  G A G C C A C T G T  T G T a c c c a g -
P855596    878  - - - - - - - - - -  - - - - - T C C G C  G G T C C T G A G C  T A G C C C C G G G  T G C T T C T T G A
P855597    875  - - - - G A A T T G  A C G C G T C C G C  G G T C C C A C G C  T A G C C C C A G G  T G T T T C T G G A
                        * * * * * *  * * * * * * * * * *  * * * * * * * * * *  * * * * * * * * * *  * * * * * * * * * *
                                               * * * * *  * * * * * * * * * *  * * * * * * * * * *  * * * * * * * * * *

alignment
position        1 4 0 1 . . . . .  1 4 1 1 . . . . .  1 4 2 1 . . . . .  1 4 3 1 . . . . .  1 4 4 1 . . . . . .

P855595   1002  - - - - - - - - - -  - - - - - - - - - -  - - - - - - - - - -  - - - - - - - - - -  - - - - - - - - - -
P855596    913  A C C C a g C T T T  A C A G T T C C A G  A G A A G G T T C T  A G A T G C C C A T  T C G A t T T T G T
P855597    921  A C C C g a C T T T  A C A G T T C C A G  A A A A T G T T C C  A G A T G C C C A T  T A G A c T T T G T
                * * * *     * * * *  * * * * * * * * * *  * * * * * * * * * *  * * * * * * * * * *  * * * *   * * * * *
                * * * *     * * * *  * * * * * * * * * *  * * * * * * * * * *  * * * * * * * * * *  * * * *   * * * * *
                            * * * *  * * * * * * * * * *  * * * * * * * * * *  * * * * * * * * * *  * * * *   * * * * *

alignment
position        1 4 5 1 . . . . .  1 4 6 1 . . . . .  1 4 7 1 . . . . .  1 4 8 1 . . . . .  1 4 9 1

P855595   1002  - - - - - - - - - -  - - - - - - - - - -  - - - - - - - - - -  - - - - - - - - - -  - - - -
P855596    963  A A C T A A A G T G  G C G A A G G G C T  T T A G C A G T T A  T c t g t t a c t t  a g t c
P855597    971  A A C T A A A G T G  G C A A A G G G C T  T T A G C A G T T A  T - - - - - - - - -  - - - -
                * * * * * * * * * *  * * * * * * * * * *  * * * * * * * * * *  *
                * * * * * * * * * *  * * * * * * * * * *  * * * * * * * * * *  *
                * * * * * * * * * *  * * * * * * * * * *  * * * * * * * * * *  *
```

Pairwise similarities:

For each pairwise alignment, the similarity (relative to the maximum similarity) and the number of identical nucleic acids (in % of shorter sequence) is given. Maximum values are underlined.

	P855596 (1006 bp)	P855597 (1001 bp)
**P855595 (1001 bp)**	0.298<br>32 %	0.277<br>34 %
**P855596 (1006 bp)**		<u>1.000</u><br><u>71 %</u>

Please note that the **similarity value 1.000** marks only the two **most similar** sequences, it does not necessarily mean that these sequences are identical.

**Extract alignment region**	
**Positions in alignment**	from: to:
**Output file**	dialign.seq

Extract aligned sequences

For comments, questions or bug reports, please contact support@genomatix.de

© Genomatix Software GmbH 1998-2005 - All rights reserved. License Agreement GEMS Launcher 4.1.1

Logout Personal Messages

GEMS Launcher Task: *DiAlign TF*: Multiple alignment plus TF sites
working on promoters.seq (3 seq.)

[Alignment] [Pairwise similarities]

DiAlign professional TF Release 3.1 December 2004 **Mon Jun 6 23:42:11 2005**

Solution parameters:

**Sequence file:**	promoters.seq (3 seq.)
**Type of sequences:**	nucleotide sequences
**Threshold T:**	0.00
**'*' signs below alignment denote:**	diagonal similarity (max. similarity is represented by 10 '*' signs)
**Alignment output:**	complete alignment is shown<br># nucleic acids per line: 50
**TF output:**	common TF matches located in aligned regions (common to 3 (100.0 %) sequences)
**Family matches:**	yes
**MatInspector library:**	Matrix Family Library Version 5.0 (February 2005)
**Selected groups** (core/matrix sim):	• ALL user_defined.lib (0.75/Optimized)<br>• ALL vertebrates.lib (0.75/Optimized)

Aligned Sequences:

No.	Sequence Name	Sequence Description	Length
1	P878819	sym=NDUFA7|loc=Loc4701|taxid=9606|spec=Homo sapiens|chr=19| ctg=NT_077812|str=(-)|start=989762|end=990762|len=1001| tss=501| comm=NADH dehydrogenase (ubiquinone) 1 alpha subcomplex, 7, 14.5kDa; (NM_005001/517/bronze;)	1001 bp
2	P878820	sym=Ndufa7|loc=Loc66416|taxid=10090|spec=Mus musculus| chr=17|ctg=NT_039649|str=(+)|start=10247921|end=10248946| len=1026|tss=501,524,526| comm=NADH dehydrogenase (ubiquinone) 1 alpha subcomplex, 7 (B14.5a); (AK010299/526/gold;AK002476/501/gold;AK003468/501/gold; NM_023202/524/bronze;)	1026 bp
3	P878821	sym=na|loc=Loc299643|taxid=10116|spec=Rattus norvegicus| chr=7|ctg=NW_047773|str=(+)|start=16320701|end=16321701| len=1001|tss=501| comm=similar to NADH oxidoreductase; (XM_216859/524/bronze;)	1001 bp

Alignment (DiAlign format):

Please note that only **upper-case** letters are considered to be **aligned**. For more information, please have a look at the user guide.

V$AHRR V$HIFF V$P53F V$SP1F V$ECAT V$HESF V$MTF1 V$YY1F V$CREB V$EBOX V$TBPF V$STAF
V$WHZF V$NFKB

```
alignment
position        1 . . . . . . . . .  1 1 . . . . . . . .  2 1 . . . . . . . .  3 1 . . . . . . . .  4 1 . . . . . . . .
P878819       1 a g a t a t g a a t  t c a g a g a a c g  t g t c g g g g c c  a g a a t a c a g a  t c g t t a a a g g
P878820       1 n n n n n n n n n n  n n n n n n n n n n  n n n n n n n n n n  n n n n n n n A C C  A A A G C T T T C A
P878821       1 a a a a - - - - - -  - - - - - - - - - -  - - - - - - - - - -  - - - - - - - A C C  A A A G C T T T C A
```

```
                                                      *** *********
                                                      *** *********

alignment
position       51........ 61........ 71........ 81........ 91........

P878819    51  gaaccatcag aacccc---- ---------G TGATGAAATG AGAATCGGCT
P878820    51  AAGGCAGtct tactacggTA TCCGCGTTTC TTACAAAGTC ATATCCTc--
P878821    18  AAGACAGaca ttgtaaatTA TCCAGGTTTG TGACAAAGTT ATATCCTTCT
               *******          ** ********** ********** **********
               *******          ** ********** ********** *******

alignment
position       101....... 111....... 121....... 131....... 141.......

P878819    88  CCCGGATTCC GGCTGGCAGg ggttagggca gggtagaggt cacatcaggg
P878820    99  -CCTGAACCT AGCTACTAGA ATTAAACGTC ACCAAATCCA G-TTATAATC
P878821    68  TCCTAAACCT AGCAGTTAGG ATTAAACATC ACCAAACCCA GcTAATAAGC
               ********** ********** ********** ********** *  *******
                ********* ********** ********** ********** *

alignment
position       151....... 161....... 171....... 181....... 191.......

P878819   138  tttaggt--- ---------- ---------- --CGCCGGCA GTCGGCCAAA
P878820   147  CTGGTACTCG GGAGGA---- CTCCGCTCCG ACCCCAGGAA GTGGTCCGAA
P878821   118  CTGGTATTTA GGAGGAcccg CTCCGACCGG ACCCCAGGAA GTGGTCGGAA
               ********** ******     ********** ********** **********
                                     ********** ********** **********
                                     ********** ********** **********

alignment
position       201....... 211....... 221....... 231....... 241.......

P878819   163  TGCCCCCACC CGATTACCTG CGTGCACTGT CCCTGAGAAC CGGTCCTGCC
P878820   193  TGTTCCCATC a-CTCACCTG CGTGCACTGT CCCTGCGAAC CGGTCCTGCC
P878821   168  AGTTCCCATC CGCTCACCTG CGTGCACTGT CCCTGCGATC CGGTCCTGCC
               ********** ********** ********** ********** **********
               ********** ********** ********** ********** **********
               ********** ********** ********** ********** **********
                   ******   ******** ********** ********** **********
                   ******   ******** ********** ********** **********
                            ******** ********** ********** **********
                            ******** ********** ********** **********
                               ***** ********** ********** **********
                               ***** ********** ********  * **********
                               ***** ********** ********  * **********

alignment
position       251....... 261....... 271....... 281....... 291.......

P878819   213  CAGGACCTTG GTGACCTGGA GGAAAGAAGc tgggttcaga cccggcccgt
P878820   242  CAGCACTTTG GTTACCTACA GGAAATAAGG GAAAGTTAgg ctCGACATGT
P878821   218  CAGCACTTTA GTTACCTACA GGAAAGAAGA GACAGTTAaa ccCGACAGGT
               ********** ********** ********** ********     ********
               ********** ********** ********** ********     ********
               ********** ********** ********** ********     ********
               ********** ********** *********
               ********** ********** *********
               ********** ********** *********
               **
               **
               **
               **

alignment
position       301....... 311....... 321....... 331....... 341.......

P878819   263  ttggctcctc taatccctcc ccctgccccc aaattcgggc ccc-CACCTC
P878820   292  TCAGCTTTCT TCTCTCTTTA TTCAGGTTTC TAAACACCCG CAGTCGCCTC
P878821   268  CCAGCTTTCT TCTTTCTTTA TTCAGGCTTC TAAACACCCG TAGTCACCTC
               ********** ********** ********** ********** **********
               ********** ********** ********** ********** **********
               ********** ********** ********** ********** **********
                                                   ******** **********
                                                                ******
```

```
                                                                        ******
                                                                        ******
                                                                          ****
                                                                          ****
alignment
position        351.......  361.......  371.......  381.......  391.......
P878819    312  ACCCTGGCCA  GCTTGATAGG  CTGCACACGG  CTgGTGTCCA  TGATGGCGGC
P878820    342  ACCCTAGCCA  GCTTGATGGG  CTGCACGCGA  CTCGTGTCCA  TGATGGCGGC
P878821    318  ACCCTAGCCA  GCTTGATGGG  CTGCACGCGA  CTCGTGTCCA  TGATGGCGGA
                **********  **********  **********  **********  **********
                **********  **********  **********  **********  **********
                **********  **********  **********  **********  **********
                **********  **********  **********  **********  **********
                **********  **********  **********  **   *****  **********
                **********  **********  **********  **   *****  ********
                **********  **********  **********  **
                **********  **********  **********  **
                **********  **********  **********  **
alignment
position        401.......  411.......  421.......  431.......  441.......
P878819    362  Gcggcggcgg  TCTGGCGGAG  AGGAGTCACG  TGCTTCGGGG  AGAGCCTTTA
P878820    392  GA--------  TCTGGCGGAG  AGGAGTCACG  TGAGCAGGGG  ATGCCTTTTA
P878821    368  GA--------  TCTAGCGGAG  AGGAGTCACG  TGAGCAGGGG  ATGCCATTTA
                **          **********  **********  **********  **********
                **          **********  **********  **********  **********
                **          **********  **********  **********  **********
                **          **********  **********  **********  **********
                *           **********  **********  **********  **********
                            **********  **********  **********  **********
                                ******  **********  **
                                ******  **********  **
alignment
position        451.......  461.......  471.......  481.......  491.......
P878819    412  TAGGACGTTC  TAGCACCGCC  cac--TCCCG  TCTCGCGATA  CCacgccgcg
P878820    434  TAGTCGGCTC  TGACCACGCC  TCACTTTCGC  TCTCGCGAGA  CCGCACGCgG
P878821    410  TAGTCGGCTC  gGACCACGCC  TCACTTCCGC  TCTCGCGAGA  CCGCACGCaG
                **********  **********  **********  **********  ********  *
                **********   *********  **********  **********  ********  *
                **********   *********  **********  **********  ********  *
                **********   *********  **********  **********  ********
                **********
                ***
alignment
position        501.......  511.......  521.......  531.......  541.......
P878819    460  tctTTCCCAG  CAGCCCCAGC  GGTGGAGACG  TCACCGGCtg  cgcccttcag
P878820    484  ---TACCCAG  GAATCCTCGG  GACAGAGTCG  TCACCAGCGT  CCTTCGGA--
P878821    460  ---TACCCAG  GAACCCTCGG  GGCAGAATCG  TCACCGGCGT  CCTTCGGA--
                   *******  **********  **********  **********  ********
                   *******  **********  **********  **********  ********
                   *******  **********  **********  **********  ********
                   *******  **********  **********  ********       ******
                                                                   ******
alignment
position        551.......  561.......  571.......  581.......  591.......
P878819    510  tatcGCGGAC  GGAAGATGGC  GTCCGCCACC  CGTCTCATCC  AgcgGCTGCG
P878820    529  ----GCGGAA  GGAATATGGC  GTCCGCTACT  CGCGTTATCC  AAAAGCTGCG
P878821    505  ----GCGGAA  GGAATATGGC  GTCCGCTACT  CGCGTTATCC  AAAAGCTTCG
                    ******  **********  **********  **********  **********
                    ******  **********  **********  **********  **********
                    ******  **********  **********  **********  **********
                    ******  **********  **********  **********  **********
                    ******  **********  **********  **********  *   ******
                    ******  **********  **********  **********  *   ******
                    ******  **********  **********  **********  *   ******
                    ******  **********  **********  ********        ******
```

```
alignment
position       601....... 611....... 621....... 631....... 641.......
P878819   560  GAACTGGGCG TCCGGGTGC  Gcggggcgga cagggctgca ccatGGGGGG
P878820   575  GAACTGGGCG TCTGGGTGC  GTGTGCTGtT CCGATCCGGG CCG-GGAAGG
P878821   551  GAACTGGGCG TCTGGGTGC  GTGTACTG-T CCGACCCCGG CCG-GGAAGG
               ********** ********** ******** * ********** *** ******
               ********** ********** ******** * ********** *** ******
               ********** ********** ******** * ********** *** ******
               ********** ********** ********
               ********** ********** *
               ********** ********** *
               ********** ********** *
               ********** ********** *

alignment
position       651....... 661....... 671....... 681....... 691.......
P878819   610  CTTCGGGCTC CGTGTGTCCG GGAcccgctc ctgctcctca ccTCCCTCCT
P878820   624  ATTTGGGTTC TGTACGCCCT TGAATCCTA- ---------- --TCCCCCTT
P878821   599  CTTTGGGTTC TGTAAGCCCT CGAAACCTA- ---------- --TCCCTCTT
               ********** ********** *********             ********
               ********** **********  ********             ********
               ********** **********  ********             ********

alignment
position       701....... 711....... 721....... 731....... 741.......
P878819   660  AAgccgggac ccttcgctct ccccgaatct ccctgGGAAC CCCCAGCTAG
P878820   661  AACATCTCCT TAGTTCAGTG gataaTCCA- -----GGATC CCTGAATTTG
P878821   636  AACATCTGCT TAGTTCGGTG cggagTCCA- -----GGATC CCTGAATTTG
               ********** **********      ****       ***** **********
               ********** **********      ****       ***** **********
               ********** **********      ****       ***** **********

alignment
position       751....... 761....... 771....... 781....... 791.......
P878819   710  TCACCCCTTC AGCC---CGg gaccctctcc tggcctggga accactcagg
P878820   705  TGACTCCAGT AGTC---AGA CTCAAGGTCC TAAATGTTTT Tgg-CCGGGT
P878821   680  TGACTtccaa tagacagAGA CTCAATGTCC TAAATGTTTT TtgaCCGGAT
               ********** ****   *** ********** ********** *   ******
               *****             *** ********** ********** *   ******
               *****

alignment
position       801....... 811....... 821....... 831....... 841.......
P878819   757  c-CTGGGATT TCCccagtcc ccgaccttcT TAGCCTTGGA Tctgtgggcc
P878820   751  TACTGGGATT TCC------- ---------T TGGCCTTAGA TTGc------
P878821   730  TCTTGGAATT CCC------- ---------T TGGCTTTAGA TTGtcc----
               ********** ***                * ********** ***
               ********** ***                * ********** ***

alignment
position       851....... 861....... 871....... 881....... 891.......
P878819   806  cggactCTGG CTTTgctctt cacctgcagg acccacatcc gtttccccga
P878820   779  ------CTGG CTTTAGCTCC C--------- ---------- ----------
P878821   760  --------GG TTTTAGCTTC C--------- ---------- ----------
                     **** ********** *
                       ** ********** *

alignment
position       901....... 911....... 921....... 931....... 941.......
P878819   856  gtgttcatcc cctgccttcc cttaaaatgc gcatccccgg accatccctg
P878820   794  ---------- ---------- ---------- ---------- ----------
P878821   773  ---------- ---------- ---------- ---------- ----------

alignment
position       951....... 961....... 971....... 981....... 991.......
P878819   906  gTCCCTTGCC CTCTCCTTTC CTCTCTC--C tggggtgcct tgcaggccga
P878820   794  -CCCCGCCCC GCCCCTCTTC CTATCccttT ---------- ----------
```

```
P878821     773 - TCCCTCCCC TCCTCCTTTC CTATCTC-- T ---------- ----------
                  ********* ********** *******   *
                  ********* ********** *****     *
                                                 *

alignment
position          1001...... 1011...... 1021...... 1031...... 1041......

P878819     954 c CTCTCCTCT CCTCttccca cagcatgacc t--------- ----------
P878820     823 - CTCTCTTCG CCTCATTTGT CTCCCACGTg ------AGAC CAGGTGTTTC
P878821     800 - CTCTCCTTG CCTCATTTGT CTTCCACGTt ccactcAGAA CTGGTGTTTC
                  ********* ********** ********         **** **********
                  ********* ********** ********

alignment
position          1051...... 1061...... 1071...... 1081...... 1091......

P878819     985 ---------- ---------- ---------- ---------- ----------
P878820     866 ctcatcCCCC GTATCAGCTT GGAATCTTtg tttgtt---- ----------
P878821     849 tttct-CCCT GTATCAGCTT TGAATCTTgt gcccctattt tcggaaggtc
                      **** ********** ********
                      **** ********** ********

alignment
position          1101...... 1111...... 1121...... 1131...... 1141......

P878819     985 ---------- ---------- ---------- -----GCAGG GGAAGCTGca
P878820     902 ---------- ---------- ---------- ---------- ------TGTT
P878821     898 gaaggccagt ttttagattt tggaagaaaa cagagGCCGG GGAAGCTGTA
                                                     ***** **********

alignment
position          1151...... 1161...... 1171...... 1181...... 1191......

P878819    1000 gc-------- ---------- ---------- ---------- ----------
P878820     906 TGTTTTTGTT TTTGTTTTTC TTTTTCGAGA CAGGgtttct ctgtgtagcc
P878821     948 AATTTCTGTA TCTTAATTTA GGGTCTAAGA AAGGaagtat atctagagct
                ********** ********** ********** ****

alignment
position          1201...... 1211...... 1221...... 1231...... 1241......

P878819    1002 ---------- ---------- ---------- ---------- ----------
P878820     956 ctggctgtcc tggaactcac tttgtagacc aggctggcct tgaactcata
P878821     998 cagc------ ---------- ---------- ---------- ----------

alignment
position          1251...... 1261...... 1271

P878819    1002 ---------- ---------- -
P878820    1006 aatccgcctg cctctgcctc c
P878821    1002 ---------- ---------- -
```

Pairwise similarities:

For each pairwise alignment, the similarity (relative to the maximum similarity) and the number of identical nucleic acids (in % of shorter sequence) is given. Maximum values are underlined.

	P878820 (1026 bp)	P878821 (1001 bp)
**P878819 (1001 bp)**	0.253<br>33 %	0.248<br>32 %
**P878820 (1026 bp)**		<u>1.000</u><br><u>73 %</u>

Please note that the **similarity value 1.000** marks only the two **most similar** sequences, it does not necessarily mean that these sequences are identical.

Extract alignment region	
**Positions in alignment**	from: to:
**Output file**	dialign.seq

Extract aligned sequences

For comments, questions or bug reports, please contact support@genomatix.de

© Genomatix Software GmbH 1998-2005
- All rights reserved.

License Agreement

GEMS Launcher 4.1.1

Genomatix-Logo Logout Personal Messages GEMS-Logo
Logout Personal Messages

Genomatix MainGEMSElDoradoGene2PromoterBiblioSphere
Frequently asked questionsYour ResultsYour SequencesYour ProtocolHelp

GEMS Launcher Task: *DiAlign TF*: Multiple alignment plus TF sites
working on promoters.seq (3 seq.)

[Alignment] [Pairwise similarities]

DiAlign professional TF Release 3.1 December 2004 **Tue Jun 7 00:08:43 2005**

Solution parameters:

**Sequence file:**	promoters.seq (3 seq.)
**Type of sequences:**	nucleotide sequences
**Threshold T:**	0.00
**'*' signs below alignment denote:**	diagonal similarity (max. similarity is represented by 10 '*' signs)
**Alignment output:**	complete alignment is shown<br># nucleic acids per line: 50
**TF output:**	common TF matches located in aligned regions (common to 3 (100.0 %) sequences)
**Family matches:**	yes
**MatInspector library:**	Matrix Family Library Version 5.0 (February 2005)
**Selected groups** (core/matrix sim):	• ALL user_defined.lib (0.75/Optimized)<br>• ALL vertebrates.lib (0.75/Optimized)

Aligned Sequences:

No.	Sequence Name	Sequence Description	Length
1	P884734	sym=NDUFA8|loc=Loc4702|taxid=9606|spec=Homo sapiens|chr=9|ctg=NT_008470|str=(-)|start=32242802|end=32243802|len=1001| tss=501| comm=NADH dehydrogenase (ubiquinone) 1 alpha subcomplex, 8, 19kDa; (NM_014222/501/silver;)	1001 bp
2	P884735	sym=Ndufa8|loc=Loc68375|taxid=10090|spec=Mus musculus|chr=2|ctg=NT_039206|str=(-)|start=13477407|end=13478407|len=1001| tss=501| comm=NADH dehydrogenase (ubiquinone) 1 alpha subcomplex, 8; (AK002744/590/gold;NM_026703/590/bronze;)	1001 bp
3	P884736	sym=na|loc=Loc296658|taxid=10116|spec=Rattus norvegicus|chr=3|ctg=NW_047653|str=(-)|start=1194484|end=1195484| len=1001|tss=501| comm=similar to Aa2-258; (XM_216044/581/bronze;)	1001 bp

Alignment (DiAlign format):

Please note that only **upper-case** letters are considered to be **aligned**. For more information, please have a look at the user guide.

V$SORY V$BRNF V$SP1F V$ZBPF V$NRF1 U$NRF1 V$MINI V$GATA

```
alignment position 1 . . . . . . . . .  1 1 . . . . . . . .  2 1 . . . . . . . .  3 1 . . . . . . . .  4 1 . . . . . . . .
P884734        1 t a c a t c t c c a  a c t g g a t t a t  a a a c t c c a t a  a g a g c a a g g a  t c t t g a c a t t
P884735        1 c g c g c t c g - -  - - - - - - - - - -  - - - - - - - - - -  T - - - - - - - - -  - - - - - - - - - -
P884736        1 g g g a t a c a c g  c g c t c a t a c g  g a t a c a c a c a  T - - - - - - - - -  - - - - - - - - - -
                                                                                 *
                                                                                 *
                                                                                 *
```

```
alignment position 51........ 61........ 71........ 81........ 91........
P884734         51 ttaaatattc ttattcctat gtttaaaact ggcagatagc ACACACACAC
P884735         10 ---------- ---------- ---------- ---------- ACACACACAC
P884736         32 ---------- ---------- ---------- ---------- ACACACACAC
                                                               **********
                                                               **********
                                                               **********

alignment position 101....... 111....... 121....... 131....... 141.......
P884734        101 Agtcaatg-- ---------- ---------- ---CACACAC ACATataagt
P884735         20 ACACACACAC ACACACgaat gaatgaatga aagaat---- ----------
P884736         42 ACACACACAC ACACACacac acacacacac acaCACACAC ACATcacta-
                   ********** ******                   ******* ****
                   ********** ******
                   ********** ******

alignment position 151....... 161....... 171....... 181....... 191.......
P884734        126 atatatgGAA TGAATGCATC TTAGCTTTGC TGCTTATTAG ATATg-GTCC
P884735         56 -------GAA TGAGTGAATC CTACCTATGC GACTTATTAG GTATCTGTGC
P884736         91 -------GAA TGAATGAATC CCACCTATGC GGCTTATTAG GTATCTGTGT
                          *** ********** ********** ********** **********
                          *** ********** ********** ********** **********
                          *** ********** ********** ********** *******
                          *** ********** ********** ********** *******
                          *** ********** ********** ********** ****
                          *** ********** ********** ********** ****

alignment position 201....... 211....... 221....... 231....... 241.......
P884734        175 TTGAAAAAAT TACTCGGGCA AGTTTGTGAA AGTGCCTGGC ACAAGGTGTT
P884735         99 TTGAGCACTA TAGTTGAACA AGTTTTTGAA AGCATTTGGC TGAGGGTCCT
P884736        134 TTGAGCACGT TAGTTGAACA CGTTTTT-AA AGTGCTTGGC TTAGGGTCCT
                   ********** ********** ********** ********** **********
                   ********** ********** ******* ** ********** **********

alignment position 251....... 261....... 271....... 281....... 291.......
P884734        225 TCGCAGAAgc ----AAAAAG CCGTCTTTTT CCAGTCacag ctattcaagg
P884735        149 TGGCGGAGgc ttaaAAACAG TTGTCTTTCA CTAGCCtgaa AGTGGAGACA
P884736        183 AGTCTGAGag ---------- ---------- ---------- AGTGGGTACA
                   ********       ****** ********** ******     **********
                   ********                                    **********

alignment position 301....... 311....... 321....... 331....... 341.......
P884734        271 tatagtaagt gaatcaaatg agccCCTTAC CTCCCATCTA caTATTCCCC
P884735        199 TGGACGAATG GGG------- ----GCTTAC CTCCCGTTTA TGTATTCCCC
P884736        203 TGGACGAAAG GGG------- ----CCTTAC CTCCCATTTT TGTATGCCCC
                   ********** ***            ****** ********** **********
                   ********** ***            ****** ***** **** **********
                                             ****** ***** **** **********
                                                                *********
                                                                *********
                                                                *********

alignment position 351....... 361....... 371....... 381....... 391.......
P884734        321 GATATATTTG CTCCCTGTGT ACTCCATGgc gcctgttttt agcttccagc
P884735        238 GAAATACTGG CTCCCGGTGT ACTGCATGTT AACAGCTAGT GTCTAACAGA
P884736        242 GATATATTTG CTCCCGGTGT ACTGCATGTT AACAGCTAGA GTCTAACAGA
                   ********** ********** ********** ********** **********
                   ********** ********** ********** ********** **********
                   ********** ********** ********** ********** **********
                   ********** ********** ********** ********** **********
                   ********** ********** ********
                   ********** ********** ********
                   ********** ********** ********
                                            *****
                                            *****

alignment position 401....... 411....... 421....... 431....... 441.......
```

```
P884734  371 tgttaggaTC CGGAGTCTCA GTggatacGG CATCACTATG ACAACCTGGA
P884735  288 CACCAACTTC AGCtgtctt- --------GG CGTCACTATG ACAACCAGGA
P884736  292 CACCAACTTC AGCACTCTCA GT-------- -GTCACTATG ACAACTAGGA
             ********** ********** **      ** ********** **********
             ********** ***                ** ********** **********
             ********** ***                ** ********** **********
             ********** ***                    ********* **********
                                               ********* **********
                                                ******** **********
                                                ******** **********

alignment position 451....... 461....... 471....... 481....... 491.......
P884734  421 GGGGTGGGCG GAGCCgaatc GAAGCCCCAC CCCGCTGGAg gCTGAAAAGT
P884735  329 GATGTGGGCG GAGACCAGAT GAAGCCCCGC CCCTCGGGAC -CGCTAGACT
P884736  333 GATGTGGGCG GGGCCCATAT GAAGCCCCGC CCCGCGGCCC -CAGTAGACT
             ********** ********** ********** **********  *********
             ********** ********** ********** **********  *********
             ********** ********** ********** **********  *********
             ********** *****      ********** *****
             ********** *****
             ********** ***
             ********** ***

alignment position 501....... 511....... 521....... 531....... 541.......
P884734  471 TCTTGGTATT GCGCACGCTC CCTCGCTCgt ggttggg-CA GGGCAATACG
P884735  378 TCCTGGAATG GCTTACGCTT CGCCTCCTCC AGCGGCCCTA GAGCAGTGCG
P884736  382 TCCTGGAATG GCGTAAGCTC CGTCTTTCCG AGCGGCTCCA GAACAGTGCG
             ********** ********** ********** ********** **********
             ********** **    **** ********** ********** **********
             ********** **                            ** **********
                                                       * **********

alignment position 551....... 561....... 571....... 581....... 591.......
P884734  520 CCTGCGTGTT GCCGGATGCG CATGCGCAGg cgccgtgtgg cactcggcgg
P884735  428 CTTGCGCGTC GCTGGAGACG CATGCGCAGA TGGTACCGGG CAGTCTtggc
P884736  432 CATGCGCCTC GCCGGAGACG CATGCGCAGA TGGTATTGGG CAGTCTgaga
             ********** ********** ********** ********** ******
             ********** ********** ********** ********** ******
             ********** ********** ********** ********** ******
             ******  ** ********** *********
                     ** ********** *********
                     ** ********** *********

alignment position 601....... 611....... 621....... 631....... 641.......
P884734  570 tcg--AAAGG GGAGTTCAAG GAGACGGGgg CGACGCGGCT GAGGGCTTCT
P884735  478 cAGCCAAAGG GGACTTGAAG GAGACGGG-- CGACACGGCC GAGGGCACCT
P884736  482 aAGCCAAAGG GGAATTGAAG GAGACGGG-- CGACACTGCC GAGGGCACCT
              ********* ********** ********   ********** **********
              ********* ********** ********   ********** **********
              ********* ********** ********   ********** **********
                  ***** ********** ********   ****** *** **********
                  ***** ********** ********   ****** *** **********
                  ***** ********** ********   ****** *** **********
                                                     *** **********

alignment position 651....... 661....... 671....... 681....... 691.......
P884734  618 CGTCGGGGTC GGGGCTGCaG CCGTCATGCC GGGGATAGTG GAGCTGCCcA
P884735  526 GGTCAGGGTC GCGGCTACCG CCGTCATGCC GGGGATAGTG GAGCTGCCAA
P884736  530 GGTCAGGGTC GCGGCTGCCG CCGTCATGCC GGGGATAGTG GAGCTGCCAA
             ********** ********** ********** ********** **********
             ********** ********** ********** ********** **********
             ********** ********** ********** ********** **********
             ********** ********** ********** ********** **********
             ********** ******** * ********** ********** **********
             ********** ******** * ********** ********** *******  *
             ********** ******   * ********** ********** *******  *
                                 * ********** ********** *******  *
                                 * ********** ********** *******  *
                                 * ********** ********** *******
```

```
alignment position 701.......  711.......  721.......  731.......  741.......
P884734    668 CTCTAGAGGA GCTGAAAGTA GATGAGGTGA GGCTa-TCCG GAGGACAGGC
P884735    576 CTCTGGAAGA GCTGAAAGTG GAGGAGGTGA GGCTCTTCCT GAGGGCAGGC
P884736    580 CTCTGGAAGA GCTGAAAGTA GAGGAGGTGA GGCTTTACCT GAGGGCAGGC
               ********** ********** ********** ********** **********
               ********** ********** ********** ********** **********
               ********** ********** ********** ********** **********
               ********** ********** **********  ****
               ********** ********** **********  ****
               ********** ********** **********  ****
               ********** *******
               ********** *******

alignment position 751.......  761.......  771.......  781.......  791.......
P884734    717 aaGGGAGACA TGGGGCTGCG GCTTctcggg cctgggccta gcccccccttg
P884735    626 --GGGAAGCC TGGGATTGCA GCTTTCAGGC GGGCCcaggc aCGGTTCCGG
P884736    630 --GGG-AGCC TGGGATTGCA GCTGTCAGGC GGGCCatact -CGGTTCCGG
                 ******** ********** ********** *****       *********
                 *** **** ********** ********** *****       *********
                 *** **** ********** ********** *****       *********
                                                            *********

alignment position 801.......  811.......  821.......  831.......  841.......
P884734    767 ggctccgcgg atcctgggac ccgggccccc ctccccagtc ctccaacgca
P884735    674 ---------- ---------- ---------- ---------- ----------
P884736    676 ---------- ---------- ---------- ---------- ----------

alignment position 851.......  861.......  871.......  881.......  891.......
P884734    817 tatacaggtc gacccccgga ggtcccctcc gcagctttgg gagtcgcctc
P884735    674 ---------- ---------- ---------- ---------- ----------
P884736    676 ---------- ---------- ---------- ---------- ----------

alignment position 901.......  911.......  921.......  931.......  941.......
P884734    867 cCCTCTCTAC CCCACCCTGT CGCATCTTGC AGTGGCTCTA Gaatcctccc
P884735    674 -CCTCTGCGA CCTGCATGGT CACATCTCGC CTTGGCTCCA GCCATTCCTG
P884736    676 -CCTCTGCGA CCTGCACGGT CACATCCCGC CTt-GCTCCA GACATTCTTG
                ********* ********** ********** ********** **********
                ********* ********** ********** **   ***** **********
                ********* ********** ********** **   ***** **********
                ********* ********** ********** **

alignment position 951.......  961.......  971.......  981.......  991.......
P884734    917 cctttcgccc tcCACAGCGA CCTATTTCTC CTTCCGTGAA TaataGTGAT
P884735    723 TGCG------ --CACTGCGA CCGGCCTCTC TTCTTGTGAA TCGG-GTGAT
P884736    724 TGCG------ --CACCGAGA CCGACCTCTC CTCTTATGAA TCGG-GTGGT
               ****         ******** ********** ********** **** *****
               ****         ******** ********** ********* **** *****
               ****         ******** ********** ********* **** *****

alignment position 1001......  1011......  1021......  1031......  1041......
P884734    967 ACATTTTATT Aagcaccaac cagtcatttt actta----- ----------
P884735    764 ACTTTCCATT AGCATCACCT AGCCCCTTGC TCTCCCTCCC GGTGAGGCGA
P884736    765 ACTTTCCGTT AGTATCACCT AGCCCCTTGC TCTCCCTCCC CGTGAGGCGA
               ********** ********** ********** ********** **********
               ********** ********** ********** ********** **********
               ********** ********** ********** ********** **********
                                     ********** ********** **********

alignment position 1051......  1061......  1071......  1081......  1091......
P884734   1002 ---------- ---------- ---------- ---------- ----------
P884735    814 GCCTGCCTCC tcttggtgct caccttccCC TTCCAAGTCT GTCAAAATCT
P884736    815 GTCTGCCTCC ccaaatgctc a-------CC TTCCAAGTCT GTCAAAACCT
```

```
                         **********                                    ******************** **********
                         **********                                    ******************** **********
                         **********                                    ******************** **********
                         **********                                    ******************** **********

alignment position       1101...... 1111...... 1121...... 1131...... 1141......
P884734            1002  ---------- ---------- ---------- ---------- ----------
P884735             864  CGGTGCAGAG CAGGCCTGgg tGCCGCGGCA GCACACAGTA CGTGTTGGAC
P884736             858  CGGTGCAGAG CAGGCCTGga aGCCGCGGCA GCACACAGTG TGTGTTGGAC
                         ********** ********    ********* ********** **********
                         ********** ********    ********* ********** **********
                         ********** ********    ********* ********** **********
                         ********** ********    ********* ********** **********

alignment position       1151...... 1161...... 1171...... 1181...... 1191......
P884734            1002  ---------- ---------- ---------- ---------- ----------
P884735             914  AGTGAGTCTG GcaTGGGCCA GGGTGTTTTG GTAATGGGAC ACAAgTAAAG
P884736             908  AATGAGTCTG GtgTGGGCCA GGTTGTTTTG GTAATGGGAC ACAAtTAAAG
                         ********** *  ******* ********** ********** **** *****
                         ********** *  ******* ********** ********** **** *****
                         ********** *  ******* ********** ********** **** *****
                         ********** *                                     *****

alignment position       1201...... 1211...... 1221...... 1231...... 1241
P884734            1002  ---------- ---------- ---------- ---------- ----
P884735             964  CATTTACTTC TTCGAGAGAC ATTTAAGTAT TTgttatg-- ----
P884736             958  CATTTACTTC TTCAAGAGGC ATTTAAGTAT TTtgttatgt gcac
                         ********** ********** ********** **
                         ********** ********** ********** **
                         ********** ********** ********** **
                         ********** ********** ********** **
```

Pairwise similarities:

For each pairwise alignment, the similarity (relative to the maximum similarity) and the number of identical nucleic acids (in % of shorter sequence) is given. Maximum values are underlined.

	P884735 (1001 bp)	P884736 (1001 bp)
**P884734 (1001 bp)**	0.242<br>43 %	0.251<br>38 %
**P884735 (1001 bp)**		<u>1.000</u><br><u>80 %</u>

Please note that the **similarity value 1.000** marks only the two **most similar** sequences, it does not necessarily mean that these sequences are identical.

**Extract alignment region**	
**Positions in alignment**	from: to:
**Output file**	dialign.seq

Extract aligned sequences

For comments, questions or bug reports, please contact support@genomatix.de

---

© Genomatix Software GmbH 1998-2005 - All rights reserved. License Agreement GEMS Launcher 4.1.1

Genomatix-Logo Logout Personal Messages GEMS-Logo
Logout Personal Messages

Genomatix MainGEMSElDoradoGene2PromoterBiblioSphere
Frequently asked questionsYour ResultsYour SequencesYour ProtocolHelp

GEMS Launcher Task: *DiAlign TF*: Multiple alignment plus TF sites
working on promoters.seq (3 seq.)

[Alignment] [Pairwise similarities]

DiAlign professional TF Release 3.1 December 2004 **Tue Jun 7 00:50:41 2005**

Solution parameters:

**Sequence file:**	promoters.seq (3 seq.)
**Type of sequences:**	nucleotide sequences
**Threshold T:**	0.00
**'*' signs below alignment denote:**	diagonal similarity (max. similarity is represented by 10 '*' signs)
**Alignment output:**	complete alignment is shown<br># nucleic acids per line: 50
**TF output:**	common TF matches located in aligned regions (common to 3 (100.0 %) sequences)
**Family matches:**	yes
**MatInspector library:**	Matrix Family Library Version 5.0 (February 2005)
**Selected groups** (core/matrix sim):	• ALL user_defined.lib (0.75/Optimized)<br>• ALL vertebrates.lib (0.75/Optimized)

Aligned Sequences:

No.	Sequence Name	Sequence Description	Length
1	P921928	sym=NDUFA9|loc=Loc4704|taxid=9606|spec=Homo sapiens|chr=12| ctg=NT_009759|str=(+)|start=4612044|end=4613044|len=1001| tss=501| comm=NADH dehydrogenase (ubiquinone) 1 alpha subcomplex, 9, 39kDa; (NM_005002/503/bronze;)	1001 bp
2	P921929	sym=Ndufa9|loc=Loc66108|taxid=10090|spec=Mus musculus|chr=6| ctg=NT_039356|str=(-)|start=2341012|end=2342012|len=1001| tss=501| comm=NADH dehydrogenase (ubiquinone) 1 alpha subcomplex, 9; (NM_025358/501/bronze;)	1001 bp
3	P921930	sym=na|loc=Loc362440|taxid=10116|spec=Rattus norvegicus| chr=4|ctg=NW_047696|str=(-)|start=16340910|end=16341910| len=1001|tss=501| comm=similar to NADH dehydrogenase (ubiquinone) 1 alpha subcomplex, 9; (XM_342761/501/bronze;)	1001 bp

Alignment (DiAlign format):

Please note that only **upper-case** letters are considered to be **aligned**. For more information, please have a look at the user guide.

V$YY1F V$BRNF V$OCT1 V$AP1F V$SSTAT V$NRF1 U$NRF1 V$STAF V$BRAC V$CREB V$HIFF V$TEAF
V$VBPF

```
alignment position 1.........  11........  21........  31........  41........
P921928     1 ttggga----  ----------  ----------  ----------  ----------
P921929     1 ggatgttcct  ggcGGCGTTG  GCGT------  ---------G  CGAGGGGCTT
P921930     1 ttttgat---  ---GGCGTTG  GCGTgcactt  actttggcgG  CGAGGGGCTT
                             *******  ****                  *  **********
```

```
alignment position  51........ 61........ 71........ 81........ 91........
P921928          7  ---------- ---------- ---------- ---------- ----------
P921929         36  agGGTGGGGG CGCCTCTGCA TTTCTATTTA ACTCCCCCac ttgtacactg
P921930         45  gaGGTGGGGG CGCCTCTGCA TTTCTATTTA ACTCCCCCca ctgtaat---
                      ******** ********** ********** ********
                      ******** ********** ********** ********
                      ******** ********** ********** ********
                      ******** ********** ********** ********
                      ******** ********** ********** ********

alignment position  101....... 111....... 121....... 131....... 141.......
P921928          7  ----CACACC CTATTt---- TGGTTTCTCT TCTCCACTTt tcccctcgtt
P921929         86  tacGCAGACC CTATTACACC TTTTTCCTCc gTGCAACTCC TT--------
P921930         92  ---GCAGATC CTATTACACC TGTTTTCTCT TTGCAACTTC TT--------
                       ******* ********** ********** ********** **
                       ******* ********** *********   ********* **
                                                      ********* **

alignment position  151....... 161....... 171....... 181....... 191.......
P921928         49  cttgtccccc cttttctctc tcctgggccc tggatctcct gaGCGCGGAG
P921929        128  ---------- ---------- ---------- ---------- --GAGTGCAG
P921930        131  ---------- ---------- ---------- ---------- --GAGTGCAG
                                                                  ********
                                                                  ********
                                                                  ********
                                                                  ********

alignment position  201....... 211....... 221....... 231....... 241.......
P921928         99  GGCTACGCCT CCTTCTTCCT CCGGActcac cAGGGCTGCC AGCTTAGCTT
P921929        136  GGGCACCCTT CCTTCTTACC CCTGC----- -AGAGCTGTC TGACTCTTTT
P921930        139  GGACACCCTT CCTCCTTACC CCTGA----- -AGAGCTGTC AGACCCTTTC
                    ********** ********** *****       ********* **********
                    ********** ***  ***** *****       ********* **********
                    ********** ***  ***** *****       ********* **********
                    ********** ***

alignment position  251....... 261....... 271....... 281....... 291.......
P921928        149  ACATTTTCAG GGAACAGGAA Actgagagtc aggaaaggtg agctcttcta
P921929        180  TGGTAATTAG CCCATAGAAA AAGgGTCTTT GAAAG----- ----------
P921930        183  TGGcca-TCG CCCCTAGAAT GAGcGTCTTT GAAAG----- ----------
                    ********** ********** ***    ****** *****
                    ***                          ****** *****
                    ***                          ****** *****
                                                 ****** *****

alignment position  301....... 311....... 321....... 331....... 341.......
P921928        199  cctcgggtCT TGCCATTTTG CATGTCCT-G AGTCAGTCAC TGGTTAACTC
P921929        215  --------CT GGCCATTTTG CATGCCCTTG AGTCAGTCCT TGGTTAACTC
P921930        217  --------CT GGCCATTTTG CATGCCCTTt AGTCAGTCCC TGGTTAACTC
                            ** ********** ********** ********** **********
                            ** ********** ********** ********** **********
                            ** ********** ********** ********** **********
                            ** ********** *********  ********** **********
                            ** ********** ********   ********** **********
                            ** ********** ********   ********** **********
                                                     ********** **********
                                                     ********** **********
                                                     ********** **********
                                                     ********** **********

alignment position  351....... 361....... 371....... 381....... 391.......
P921928        248  TGAACTTCAG GTTCCTCGGc aattagagat tataaaTAGC CTATACAGCC
P921929        257  TGAAATGCAG ATTCCCCGGA AATATCAGG- ------TGGT CTTCACAACC
P921930        259  TGAAATGCAG ATTCCCCaGA AATATCAGA- ------CGGC CTTCACAACC
                    ********** ********** *********        * *** **********
                    ********** ********** *********        * *** **********
                    ********** ********** *********        * *** **********
                    ********** *******  *                     ** **********
```

```
                          ********** ******* *
                          ********** *******
                          ********** *******
                          ********** *****
                          ********** *****
                          ********** *****
alignment position        401....... 411....... 421....... 431....... 441.......
P921928              298  TATTCCAGAT CTGAGATTCC aacagccaaa gt------CT TTTCACTTCC
P921929              300  CATTCCAAAA CCGGGACTgt aTTTTCCCCT GCCTTCTCCT CCTCTTTTTC
P921930              302  CATTACAGAT CCCGGATTCT gTTTTCCCCT GCCTTCTCTC CCTCTCTTTT
                          ********** *********   ******** ********** **********
                          ********** *****       ******** ********** **********
                          ********** *****                        ** **********
                          ********** *****
alignment position        451....... 461....... 471....... 481....... 491.......
P921928              342  TTTCT--TCC CCCTCTCCTT CACTTTCCCA GCTTTCTTCT TAACCAACAA
P921929              350  TTTTT--CTC TCCTCTGCTT TTACTCCCAG GCTCTCTTAT CAACCATCAA
P921930              352  TCTTTtcTCT ACCTCTGCTT TCACTCCCAG TCTCTCTGAT CAACCtgggc
                          *****  *** ********** ********** ********** **********
                          *****        ******** ********** ********** *****
                          *****        ******** ********** ********** *****
                                                    ****** ********** *****
alignment position        501....... 511....... 521....... 531....... 541.......
P921928              390  TCTACCcgaa accgtcgtTT CTTGGTTAGC GCTTGCGCAG ACAGGcgaga
P921929              398  CCTGCCACTT CCG-----TT CCCAGTTGGC GTTTGCGCAG AGAGGTCCtt
P921930              402  cccGCCACTT CCG-----TT CTCAGTTGGC GTCTGCGCAG AGAGGTCCat
                          ********** ***      ** ********** ********** ********
                               ***** ***      ** ********** ********** ********
                               ***** ***      ** ********** ********** ********
                               ***** ***      ** ********** ********** ********
                               ***** ***      ** ********** ********** ********
                                              ** ********** ********** *****
alignment position        551....... 561....... 571....... 581....... 591.......
P921928              440  aAGGTAAGTT TTGACCCAGC TTAGCAGCCG TAGTCAGCCG ggagtgctgt
P921929              443  gAGATACCAT TTGACCCCGC GTTGTTGCCG TAGTTGGCGG AAGCACCGGC
P921930              447  gaga----TT TTGGCCCTGC GTAATTGCCG TAGTTGGCGG AAGCGCCGGC
                           ********* ********** ********** ********** **********
                                  ** ********** ********** ********** **********
                                          ****** ********** ********** **********
                                          ****** ********** ********** **********
                                          ****** ********** ***
alignment position        601....... 611....... 621....... 631....... 641.......
P921928              490  CGTGGGGGAT TGTGGGAAAA GATGGCGGCT GCCGcacaat ccCGGGTTGT
P921929              493  CGTGAGGCAT TGTGGGAGAA GATGGCGGCC GCCGTCCGCT TTCGGGTTGT
P921930              493  CGTGAGGCAT TGTGGGAGAA GATGGCGGCC GCCGTCCGCT TTCaGGTTGT
                          ********** ********** ********** ********** **********
                          ********** ********** ********** ********** ***  *****
                          ********** ********** ********** ********** ***  *****
                          ********** ********** ********** ********** ***  *****
                          ********** ********** ********** ********** ***  *****
                          ********** ********** ********** ********** ***  *****
                          ********** ********** ********** ****
                          ********** ********** ********** ****
                               ***** ********** ********** ****
                               ***** ********** ********** ****
                               ***** ********** ********** ****
alignment position        651....... 661....... 671....... 681....... 691.......
P921928              540  ccgggtcctg tCAATGTCAC GTAAGTGTTA CCGGGAACgg cGGCCCCTGG
P921929              543  TAGAGCGCTT CCAATGTCAC GTAAGTGTCA CCGGGGACCG -GGTCTTGGG
P921930              543  TAGAGCGCTT CCAATGTCAC GTAAGTGTCA CCGGGTACCG -GGTCTTGGA
                          ********** ********** ********** **********  *********
                          ********** ********** ********** **********  *********
                          ********** ********** ********** **********  *********
                          ********** ********** ********** ********
```

```
                  * * * * * * * * * *  * * * * * * * * * *  * * * * * * * * * *  * * * *     * *
                  * * * * * * * * * *  * * * * * * * * * *  * * * * * * * * * *  * * * *     * *
                                         * * * * * * * * *  * * * * * * * * * *  * * * *     * *
                                         * * * * * * * * *  * * * * * * * * * *  * * * *
                                         * * * * * * * * *  * * * * * * * * * *  * * * *
                                         * * * * * * * * *  * * * * * * * * * *  * * * *

alignment position 701.......  711.......  721.......  731.......  741.......
P921928       590 TCGGGATTCC GAGACGGGGA TTTA-AGGTT Ttggaagtgg caccgtgctg
P921929       592 GTTGCATTCG GCGACGGGAA CATG-ACCTT TCTTCCTCAT AC--------
P921930       592 GCTGCATTCC GCGACGGGAA CAaaaACCTT TCTTCCTCAT AC--------
                  * * * * * * * * * *  * * * * * * * * * *  * * * *   * * * * *  * * * * * * * * * *  * *
                  * * * * * * * * * *  * * * * * * * * * *  * *       * * * * *  * * * * * * * * * *  * *
                  * * * * * * * * * *  * * * * * * * * * *  * *       * * * * *  * * * * * * * * * *  * *
                            * * * * *  * * * * * * * *               * * * * *  * * * * * * * * * *  * *
                                                                     * * * * *  * * * * * * * * * *  * *

alignment position 751.......  761.......  771.......  781.......  791.......
P921928       639 cagcggtcac gccaacttcc taggcacact ctggtttctc taggtttgga
P921929       633 ---------- ---------- ---------- ---------- ----------
P921930       634 ---------- ---------- ---------- ---------- ----------

alignment position 801.......  811.......  821.......  831.......  841.......
P921928       689 gctgcctcag tctggtttct cctttcgctt tccgcttCCC CGGCTCACAT
P921929       633 ---------- ---------- ---------- -------TCT TGGCTCATGT
P921930       634 ---------- ---------- ---------- -------CCT TGGCGCATGT
                                                                   * * *  * * * * * * * * * *
                                                                   * * *  * * * * * * * * * *
                                                                   * * *  * * * * * * * * * *
                                                                   * * *  * * * * * * * * * *
                                                                   * * *  * * * * * * * * * *

alignment position 851.......  861.......  871.......  881.......  891.......
P921928       739 ATTCAATTGT GCACcacctt GGGAGGGGAC aaaAGATCTT AAGCAGCTGT
P921929       646 GTGAAACTG- -CACTCG--- GCAAGGGGAC GG-AGAGCTG GAGGAGCTGG
P921930       647 GTGAAACTGT GCACTCG--- GGGAGGAGAC GG-AGAGCTG GAGTAGTTGG
                  * * * * * * * * * *  * * * * * *        * * * * * * * * * *  * *   * * * * * * *  * * * * * * * * * *
                  * * * * * * * * *      * * * * * *      * * * * * * * * * *  * *   * * * * * * *  * * * * * * * * * *
                  * * * * * * * * *      * * * * * *      * * * * * * * * * *  * *   * * * * * * *  * * * * * * * * * *
                  * * * * * * * * *                                              * * * * * * *  * * * * * * * * * *
                  * * * * * * * * *

alignment position 901.......  911.......  921.......  931.......  941.......
P921928       789 AAGCGAAGAG GAGAATGCGG gg---GAC-A CGTTAGGGAT cgagagaatc
P921929       690 GAGGGACGAG GAGAACCCGG ACACTGAC-A GGTTCGAGAT ----------
P921930       693 GTGAGACGAG GAGGACCTGG ACACTtaaaA GGCTCGAGAT ----------
                  * * * * * * * * * *  * * * * * * * * * *  * * * * * * * *   *  * * * * * * * * * *
                  *     * * * * * *  * * * * * * * * * *                   *  * * * * * * * * * *
                  *                                                        *  * * * * * * * * * *
                  *

alignment position 951.......  961.......  971.......  981.......  991.......
P921928       835 agAATTCTGA TATGGTTGTA AGGGTTTAGG Gttat----- ----------
P921929       729 --AGTGCGTG GATTGTGGGG AAGGTTGCGG tccaagagag tacttatcca
P921930       733 --AATGCGGA GATTGTTGGG AAGGTTGAGG Gcaaaggaac tacttaggga
                      * * * * * * * *  * * * * * * * * * *  * * * * * * * * * *  *
                      * * * * * * * *  * * * * * * * * * *  * * * * * * * * * *
                      * * * * * * * *  * * * * * * * * * *  * * * * * * * * * *

alignment position 1001......  1011......  1021......  1031......  1041......
P921928       870 ---------- ---------- ---------- ---------- ----------
P921929       777 ggGGAAAGGG GGCTTGGACG GCAGGACATC GATTGACTTC ATGGTGGTGA
P921930       781 --GGAAAAGG GGTTTGGACA GCTGGACATC GATTGGCTTC ATGGTAGTAA
                      * * * * * * * *  * * * * * * * * * *  * * * * * * * * * *  * * * * * * * * * *  * * * * * * * * * *
                      * * * * * * * *  * * * * * * * * * *  * * * * * * * * * *  * * * * * * * * * *  * * * * * * * * * *
                      * * * * * * * *  * * * * * * * * * *  * * * * * * * * * *  * * * * * * * * * *  * * * * * * * * * *

alignment position 1051......  1061......  1071......  1081......  1091......
```

```
P921928      870 ---------- ---------- ---------- ---------- ----------
P921929      827 TCGCACTGGA AATCAGAATC CGCGCCACAG A-------TG TCAGGTTGTG
P921930      829 TTGCAATGGA AATCATAATC CGCTCCACGG AtggagtcTG TCAGGTTGTG
                 ********** ********** ********** *       ** **********
                 ********** ********** ********** *       ** **********
                 ********** ********** ********** *       ** **********
                                                          ** **********

alignment position 1101...... 1111...... 1121...... 1131...... 1141......
P921928      870 ---------- --------GA GAAATATTTG TATGGGGCGG GGGGAGAAAT
P921929      870 GCGGTAGGGA CTGCAGCAGC GAGAGATGGT TGTGGAGAGT CGGGAGCTGG
P921930      879 GCGCTAGGGT CTGCAGCAGA GAAAGATGTT TGTGGAGAAT TGGGAGCTGG
                 ********** ********** ********** ********** **********
                 ********** ********** ******** * ********** **********
                 ********** ********** ******** * ********** **********
                 ********** ********** ******** * ********** **********

alignment position 1151...... 1161...... 1171...... 1181...... 1191......
P921928      902 GTGTAAGGga gggaaaaaag gctagtggcg agaggttttc ttAGATTGAT
P921929      920 GTGGAAAGAA AAGTGAAGGa CCGCTTAGAA TTTGTCA--- --GCGTTGTT
P921930      929 GTGGAAAGAA AAGCGAAGG- CCGCTTAGAA TTTGTCA--- --ACATTGTT
                 ********** *********  ********** *******      ********
                 ********** *********  ********** *******      ********
                 ********** *********  ********** *******      ********
                 ********** *********  ********** *******      ********
                                       ********** *******      ********

alignment position 1201...... 1211...... 1221...... 1231...... 1241......
P921928      952 TTTATGACCT TGTGCAAACC CTTAATgggt ctcaattcat atgagcacaa
P921929      965 TATTTACTCT TCTGtcGTGA TTGAATGTGt tcacagc--- ----------
P921930      973 TATTTACTCT TCTGCTGTGC TTGAATCTG- ---------- ----------
                 ********** ********** *********
                 ********** ****
                 ********** ****
                 ********** ****
                 ********** ****
```

Pairwise similarities:

For each pairwise alignment, the similarity (relative to the maximum similarity) and the number of identical nucleic acids (in % of shorter sequence) is given. Maximum values are underlined.

	P921929 (1001 bp)	P921930 (1001 bp)
P921928 (1001 bp)	0.156<br>40 %	0.156<br>34 %
P921929 (1001 bp)		1.000<br>79 %

Please note that the **similarity value 1.000** marks only the two **most similar** sequences, it does not necessarily mean that these sequences are identical.

**Extract alignment region**	
**Positions in alignment**	from: to:
**Output file**	dialign.seq

Extract aligned sequences

For comments, questions or bug reports, please contact support@genomatix.de

© Genomatix Software GmbH 1998-2005
- All rights reserved.

License Agreement

GEMS Launcher 4.1.1

Genomatix-Logo Logout Personal Messages GEMS-Logo
Logout Personal Messages

Genomatix MainGEMSElDoradoGene2PromoterBiblioSphere
Frequently asked questionsYour ResultsYour SequencesYour ProtocolHelp

GEMS Launcher Task: *DiAlign TF*: Multiple alignment plus TF sites
working on promoters.seq (3 seq.)

[Alignment] [Pairwise similarities]

DiAlign professional TF Release 3.1 December 2004 **Tue Jun 7 01:08:06 2005**

Solution parameters:

**Sequence file:**	promoters.seq (3 seq.)
**Type of sequences:**	nucleotide sequences
**Threshold T:**	0.00
**'*' signs below alignment denote:**	diagonal similarity (max. similarity is represented by 10 '*' signs)
**Alignment output:**	complete alignment is shown<br># nucleic acids per line: 50
**TF output:**	common TF matches located in aligned regions (common to 3 (100.0 %) sequences)
**Family matches:**	yes
**MatInspector library:**	Matrix Family Library Version 5.0 (February 2005)
**Selected groups** (core/matrix sim):	• ALL user_defined.lib (0.75/Optimized)<br>• ALL vertebrates.lib (0.75/Optimized)

Aligned Sequences:

No.	Sequence Name	Sequence Description	Length
1	P982079	sym=NDUFA10|loc=Loc4705|taxid=9606|spec=Homo sapiens|chr=2| ctg=NT_005416|str=(-)|start=138902|end=139902|len=1001| tss=501| comm=NADH dehydrogenase (ubiquinone) 1 alpha subcomplex, 10, 42kDa; (NM_004544/501/silver;)	1001 bp
2	P982083	sym=Ndufa10|loc=Loc67273|taxid=10090|spec=Mus musculus| chr=1|ctg=NT_039173|str=(-)|start=6870406|end=6871447| len=1042|tss=501,506,542| comm=NADH dehydrogenase (ubiquinone) 1 alpha subcomplex 10; (AK013675/517/gold;AK005339/522/gold;NM_024197/558/silver;)	1042 bp
3	P982085	sym=Ndufa10|loc=Loc314071|taxid=10116| spec=Rattus norvegicus|chr=9|ctg=NW_047817|str=(-)| start=7820309|end=7821309|len=1001|tss=501| comm=NADH dehydrogenase (ubiquinone) 1 alpha subcomplex 10; (XM_234089/558/bronze;)	1001 bp

Alignment (DiAlign format):

Please note that only **upper-case** letters are considered to be **aligned**. For more information, please have a look at the user guide.

V$MAZF V$CREB V$E4FF V$HESF V$MOKF V$EREF V$SF1F V$REBV V$EBOX

```
alignment position 1.........  11.........  21.........  31.........  41.........
P982079      1 tggatgagac cacttagcct tcagctccgg aggcga---- ----------
P982083      1 ttctctcaga aacctaggca gggagaatgt gacgccccta gaactcagat
P982085      1 ---------- ---------- ---------- ---------- ----------
```

```
alignment position 51........ 61........ 71........ 81........ 91........
P982079        37 ---------- ---------- ---------- ---------- ----------
P982083        51 ggggaatgga ctctctcctc agcgaccaga aggGCGGGGA GCGGATCCTC
P982085         1 ---------- ---------- ---------- ---GCAGTGA GCAGATCCTC
                                                     ******* **********

alignment position 101....... 111....... 121....... 131....... 141.......
P982079        37 ---------- ---------- ---------- ---------- ----------
P982083       101 CCTACAGaac tcaaa----- ---------- CAGAAAGTGG GTCtacgttt
P982085        18 CCCTCAGact gggagtgggt ccatgcaacc CAGGAAGTGG ATCctcttgt
                  *******                          ********** ***

alignment position 151....... 161....... 171....... 181....... 191.......
P982079        37 ---------- ---------- ---------- ---------- ----------
P982083       136 gg-------- GCGTGGAGCA GGGAGACAGC A--------- --CAGATTCT
P982085        68 cagagctcga GCGTGGAGCA GAGAGGCAGC Aggttctcac tgCAGAATCT
                             ********** ********** *            ********
                                                                ********
                                                                ********
                                                                ********

alignment position 201....... 211....... 221....... 231....... 241.......
P982079        37 ---------- ---------- ---------- ---------- ----------
P982083       167 GGGGAGCGAG AGGATTCACC CCACAGAACA CCtgggagtg gggg------
P982085       118 GGGGAGCGAG CGGATCCACC CCACAGAATA CCcagagaga ggaggggtga
                  ********** ********** ********** **
                  ********** ********** ********** **
                  ********** ********** ********** **
                  ********** ********** ********** **

alignment position 251....... 261....... 271....... 281....... 291.......
P982079        37 ---------- ---------- ---------- ---------- ----------
P982083       211 GTCACACTTT TGTCACACAA CTCAAGCATC TGCGAGA-AC CCGAGCTCAA
P982085       168 GTCACACTTT TGTCACACAA CTCCAGCATC TGCGAGAaAC CCGAACTCAA
                  ********** ********** ********** ******  ** **********
                  ********** ********** ********** ******
                  ********** ********** ********** ******
                  ********** ********** ********** ******
                  ********** ********** ********** ******

alignment position 301....... 311....... 321....... 331....... 341.......
P982079        37 ---------- -------CCC A--------- ----GGCCGG CCTTgccagg
P982083       260 AGCTGGGAGC CTCaaggCCC A--------- ----GGCAGG ACTTCTAGCC
P982085       218 GACTGGCAGC CTCcgtgcaa ttcacaaggc tcagGGTGGG TCTTTTCGCC
                  ********** ***    *** *              ****** **********

alignment position 351....... 361....... 371....... 381....... 391.......
P982079        57 tccctctccc tgacaggcgc cccgcgacag gggtcccacc ccgtccctcC
P982083       297 CTTTGCCACT TTCATACTAT AacttgtctA ggatctagGC G--------C
P982085       268 CTTCCCCAGA CTTGTACTGT Atgggtcgag gaagta--GC A--------C
                  ********** ********** *                ** *        *
                                                         ** *        *
                                                                     *

alignment position 401....... 411....... 421....... 431....... 441.......
P982079       107 GCTCCTCGCC Ccctcttttt ccactttccc tccccagcga gaacgttctg
P982083       339 GCTCCTCGCC CAAACAATCT AAgctcaagc aactagagcg tcgacaa---
P982085       308 GCTCCCCGCC CAAACAATCT AAacccggcc ccagagccgc aatctaga--
                  ********** ********** **
                  ********** ********** **
                  ********** *

alignment position 451....... 461....... 471....... 481....... 491.......
P982079       157 agagctagga atgttctcta ggaggtcgct ttgacgtcca caccgcgcct
P982083       386 ---------- ---------- ---------- ---------- ----------
P982085       356 ---------- ---------- ---------- ---------- ----------
```

```
alignment position  501....... 511....... 521....... 531....... 541.......
P982079        207  ggggattct  gcgggagca  caggaccctg ggacgagacg cagaagtcgc
P982083        386  ---------- ---------- ---------- ---------- ----------
P982085        356  ---------- ---------- ---------- ---------- ----------

alignment position  551....... 561....... 571....... 581....... 591.......
P982079        257  tccacgccct gggcgGCCTC AGCTCCcgcg acaccgcagc aactcagaac
P982083        386  ---------- -----GCCTA AGCTCCGCCC CTTCCCTCT- ----------
P982085        356  ---------- ------CCTA AGCCCCGCCC CTTCCCTCT- ----------
                               ***** ********** *********
                                ****  ********** *********
                                ****  ********** *********

alignment position  601....... 611....... 621....... 631....... 641.......
P982079        307  ccaggcaggg gccgggcagc cagcatgctg cccccacgtt cacctccaac
P982083        410  ---------- ---------- ---------- ---------- ----------
P982085        379  ---------- ---------- ---------- ---------- ----------

alignment position  651....... 661....... 671....... 681....... 691.......
P982079        357  tcccacctct cggccctcgc cccaacccgc aggaccctgg cagcgcactg
P982083        410  ---------- ---------- ---------- ---------- ----------
P982085        379  ---------- ---------- ---------- ---------- ----------

alignment position  701....... 711....... 721....... 731....... 741.......
P982079        407  gctgtctccg cggaagcccc cgagggcctc ggcgcGGCCC CGCCCCctgg
P982083        410  ---------- ---------- ---------- -----GGCCC CGCCCCGTCc
P982085        379  ---------- ---------- ---------- -----GGCTC TGCCCCATCt
                                                          ***** **********
                                                          ***** **********
                                                          ***** **********
                                                          ***** *******

alignment position  751....... 761....... 771....... 781....... 791.......
P982079        457  aagg------ ---------- TTCCCGACAG GCGCCGCGGG CACCCCGgcc
P982083        425  cagttccgga atatttctTT TTTTCGACAG GCGCCGCGAA CACTCAGTG-
P982085        394  cgggtagt-- --------TT TTTCCGACAG GCGCCGCGGA CACTCGGTT-
                                       ** ********** ********** *********
                                       ** ********** ********** *********
                                       ** ********** ********** *********
                                       ** ********** ********** *********
                                          ********** ********** *******
                                          ********** ********** ***

alignment position  801....... 811....... 821....... 831....... 841.......
P982079        491  GTGACGTCAC GGCAGCGCGC CGgccgCGAG AGAGGGCCCC GTCGCGACCG
P982083        474  GTGACGTCAT GGCAGCGCGC CGCGGACGAG CTAGGACCCC GTCGCGTTCA
P982085        433  GTGACGTCAT GTCAGCGCGC CGCGGACAAG ATAGGACCCT GTCGCGTTCA
                    ********** ********** ********** ********** **********
                    ********** ********** ********** ********** **********
                    ********** ********** ********** ********** **********
                    *********    ******** ********** ********** **********
                    *********    ******** **    **** ********** **********
                    *********    ******** **
                    *********    ******** **

alignment position  851....... 861....... 871....... 881....... 891.......
P982079        541  CGTCCCcttg GGTCCTTGAT CCTGAGCTGA CCGGGTAGCC ATGGCCTTGc
P982083        524  TGTCCCTCT- GGTCCTTGAG CCGGCGCAGA CGGCGAAGTC ATGGCCTTGA
P982085        483  CGTCCCTCT- GGTCCTTGAG CCGGCGCAGG CAGCGAAGCA ATGGCCTTGA
                    *********  ********** ********** ********** **********
                    *********  ********** ********** ********** **********
                     ********  ********** ********** ********** **********
                     ********  ********** ********** ********** **********
                     *****     ********** ********** ********   **********
                               ********** ********** ********   *********
```

```
                              ********** ********** ********   *********
                                                               *********

alignment position 901....... 911....... 921....... 931....... 941.......
P982079        591 GGCTCCTGAA GCTGGCAGCG ACGTCCGCGT CCGCCCGGGt cgtgGCGGCG
P982083        573 GGTTGCTGAG ACTCGTCCCG GCGTCGGCTC CCGCGCGCGG CCTCGCGGCC
P982085        532 GGTTGCTGAG ACTTGTCCCG GCGTCGGCGT CCGCGCGCGG CCTCGCGGCC
                   ********** ********** ********** ********** **********
                   ********** ********** ********   ********** **********
                   ********** ********** ********   ********** **********
                   ********** ********** ********   ********** **********
                   ********** ********** ********   ********** **********
                        ***** ********** ********   ********** **********
                                                    *******        ******
                                                                   ******
                                                                   ******
                                                                   ******

alignment position 951....... 961....... 971....... 981....... 991.......
P982079        641 GGCGCCCAGC GCGTGGTGAG CGGgcggcgg ggtggcaggg tggcggggtg
P982083        623 GGAGCCCAGC GCGTGGTGAG CGGCGGGTGG CTCTCCTTGC TTGGATC-TC
P982085        582 GGAGCCCAGC GCGTGGTGAG CGGCGGGTGG CTTTCCTCGC TTGGATCcTC
                   ********** ********** ********** ********** ******* **
                   ********** ********** ********** ********** *******
                   ********** ********** ***
                   ********** ********** ***
                   ********** ********** ***
                   ********** ********** ***
                   ********** ********** ***
                   ********** **********
                   ********** **********
                   ********** **********

alignment position 1001...... 1011...... 1021...... 1031...... 1041......
P982079        691 gcagggtggc ---------- ---------- ---------- ----------
P982083        672 GGCCTGtcgg cTCCGAGCCC TGACGCTCCG GCggcgtctg TCAGACCCGT
P982085        632 GGCCTG---- -TCCGAGACC TGACACTCCG GCattgttga TCAGACCCTT
                   ******      ********* ********** **         **********
                                                               **********
                                                               **********

alignment position 1051...... 1061...... 1071...... 1081...... 1091......
P982079        701 ---------- ---------- ---------- ---------- ----------
P982083        722 GAGCCGCGAG CTCCTGAGGC CTCCgctcac agcacc-GGT GCAGCGGGAC
P982085        677 GAGCTACGAG CTCTTGAGGC CTCCacctac agctcagGGT GCAGCGGGAC
                   ********** ********** ****              *** **********
                   ********** ********** ****              *** **********
                   ********** ********** ****              *** **********

alignment position 1101...... 1111...... 1121...... 1131...... 1141......
P982079        701 ---------- ---------- ---------- ---------- ----------
P982083        771 TCACTTTCCC GGCTGCAtac GGAGAGCCGA GATGCCGGAG CtccGGAGCC
P982085        727 TCACATTCCC GGTTGCActt GGGGAACCGA GATGCCAGAG CcgtGGAGCC
                   ********** *******    ********** ********** *   ******
                   ********** *******                              ******
                   ********** *******                              ******

alignment position 1151...... 1161...... 1171...... 1181...... 1191......
P982079        701 ---------- ---------- ---------- ---------- ----------
P982083        821 TGGGCCCGCA TGCTCATCGG TTCAG----- ----AAACTG CTTTGTGGAA
P982085        777 TGGGCCCGCG TGCTCATCCG TTCAGtgatt ggtgAAGCTG CTTTGTCGAA
                   ********** ********** *****          ****** **********
                   ********** ********** *****          ****** **********
                   ********** ********** *****          ****** **********

alignment position 1201...... 1211...... 1221...... 1231...... 1241......
P982079        701 ---------- -----GGGGT GGCGGGGTGG GGAGCAGttc cacatctccc
P982083        862 CGCCTTGTAG ACTCTGGGGT GACGTGATGT GGAGCAGCAA CAGAGATTTG
P982085        827 CGCCTTGTAG ACTCGGGTGT --CGTGACGT GGAGCAGGAA CAGCGATTTG
```

```
                          * * * * * * * * * *  * * * * * * * * * *  * * * * * * * * * *  * * * * * * * * * *  * * * * * * * * * *
                          * * * * * * * * * *  * * * * * * * * * *      * * * * * * * *  * * * * * * * * * *  * * * * * * * * * *
                          * * * * * * * * * *  * * * * * * * * * *      * * * * * * * *  * * * * * * * * * *  * * * * * * * * * *
                                                         * * * * *      * * * * * * * *  * * * * * * * * * *  * * * * * * * * * *
                                                                        * * * * * * * *  * * * * * *

alignment position   1 2 5 1 . . . . . .  1 2 6 1 . . . . . .  1 2 7 1 . . . . . .  1 2 8 1 . . . . . .  1 2 9 1 . . . . . .
P982079         736  c c g g c c t c t g  g c g g c g g c t c  c c a g g t c g c t  c t c c g g g t g g  g t T T G G G G G A
P982083         912  G G G T A G A - - -  - - - - - - - - - -  - - - - - - - - - -  - - - - - - - - - -  - - T T G G G g a g
P982085         875  G G G T A G A - - -  - - - - - - - - - -  - - - - - - - - - -  - - - - - - - - - -  - - T T G G G G G A
                     * * * * * * *                                                                              * * * * * * * *
                     * * * * * * *                                                                              * * * * *
                     * * * * * * *                                                                              * * * * *
                     * * * * * * *                                                                              * * * * *

alignment position   1 3 0 1 . . . . . .  1 3 1 1 . . . . . .  1 3 2 1 . . . . . .  1 3 3 1 . . . . . .  1 3 4 1 . . . . . .
P982079         786  c c c c t c c t g c  c t c c g g a a t t  g t t t g g t c t t  t g g t c g t g g a  c a g a c c t c a g
P982083         927  g a t t A G G A C C  T T T T A G G G T G  C A T G C A G - - -  - - - - - - - - - -  - - - - - - - - - -
P982085         890  g g a g A G G A C T  T C C T A A G A T G  C A T G C A G - - -  - - - - - - - - - -  - - - - - - - - - -
                             * * * * * *  * * * * * * * * * *  * * * * * * *
                             * * * * * *  * * * * * * * * * *  * * * * * * *
                             * * * * * *  * * * * * * * * * *  * * * * * * *

alignment position   1 3 5 1 . . . . . .  1 3 6 1 . . . . . .  1 3 7 1 . . . . . .  1 3 8 1 . . . . . .  1 3 9 1 . . . . . .
P982079         836  g g g g c c a c g a  g c t t c t c c a g  g c c a t t t t a t  c t c a g c t t t a  a g c a A A A C G C
P982083         954  - - - - - - - - - -  - - - - - - - - - -  - - - - - - - - - -  - - - - - - - - - -  - - - - A A T A A T
P982085         917  - - - - - - - - - -  - - - - - - - - - -  - - - - - - - - - -  - - - - - - - - - -  - - - - A A T A G T
                                                                                                                    * * * * * *
                                                                                                                    * * * * * *
                                                                                                                    * * * * * *

alignment position   1 4 0 1 . . . . . .  1 4 1 1 . . . . . .  1 4 2 1 . . . . . .  1 4 3 1 . . . . . .  1 4 4 1 . . . . . .
P982079         886  T G G A G C G C G G  A G G T G A T C A G  G G C C C C G A G G  C T G c c c t g a t  g c g g g a g g c c
P982083         960  G G G T G A C A G T  A G G T G C T T A G  G G G A C C G T G G  C T G G A A A C A G  G A C A C G T c T T
P982085         923  G G G T G A T A G T  A A A T G C C T A G  G G G A C C G T G A  C T G G A A A C A G  G A C A C G T t T T
                     * * * * * * * * * *  * * * * * * * * * *  * * * * * * * * * *  * * * * * * * * * *  * * * * * *   * *
                     * * * * * * * * * *  *     * * * * * * *  * * * * * * * * * *  * * * * * * * * * *  * * * * * *   * *
                     * * * * * * * * * *  *     * * * * * * *  * * * * * * * * * *  * * * * * * * * * *  * * * * * *   * *
                                                * * * * * * *  * * * * * * * * * *  * * * * * * * * * *  * * * * * *

alignment position   1 4 5 1 . . . . . .  1 4 6 1 . . . . . .  1 4 7 1 . . . . . .  1 4 8 1 . . . . . .  1 4 9 1 . . . . . .
P982079         936  c g t g t g c a g g  t g g c a g g c c g  g g g g c a c a c g  c g c t t g t c a t  t a g a t a a t t c
P982083        1010  A T G A C G G C A T  C A T A A C T T C T  T T G C T T T a t a  g t a - - - - - - -  - - - - - - - - - -
P982085         973  A T G A C G A C A T  C A T A G C T T C T  T T G C T T T c t -  - - - - - - - - - -  - - - - - - - - - -
                     * * * * * * * * * *  * * * * * * * * * *  * * * * * * *
                     * * * * * * * * * *  * * * * * * * * * *  * * * * * * *
                     * * * * * * * * * *  * * * * * * * * * *  * * * * * * *

alignment position   1 5 0 1 . . . . . .  1 5 1 1 . .
P982079         986  a c g t a g c a g c  c c t g t c
P982083        1043  - - - - - - - - - -  - - - - - -
P982085        1002  - - - - - - - - - -  - - - - - -
```

Pairwise similarities:

For each pairwise alignment, the similarity (relative to the maximum similarity) and the number of identical nucleic acids (in % of shorter sequence) is given. Maximum values are underlined.

	P982083 (1042 bp)	P982085 (1001 bp)
P982079 (1001 bp)	0.150<br>22 %	0.136<br>25 %
P982083 (1042 bp)		<u>1.000</u><br><u>71 %</u>

Please note that the **similarity value 1.000** marks only the two **most similar** sequences, it does not necessarily mean that these sequences are identical.

**Extract alignment region**	
**Positions in alignment**	from: [ ] to: [ ]
**Output file**	dialign.seq

Extract aligned sequences

For comments, questions or bug reports, please contact support@genomatix.de

© Genomatix Software GmbH 1998-2005
- All rights reserved.

License Agreement

GEMS Launcher 4.1.1

Genomatix-Logo Logout Personal Messages GEMS-Logo
Logout Personal Messages

Genomatix_MainGEMSElDoradoGene2PromoterBiblioSphere
Frequently_asked_questionsYour_ResultsYour_SequencesYour_ProtocolHelp

GEMS Launcher Task: *DiAlign TF*: Multiple alignment plus TF sites
working on promoters.seq (3 seq.)

[Alignment] [Pairwise_similarities]

DiAlign professional TF Release 3.1 December 2004 **Tue Jun 7 02:18:19 2005**

Solution parameters:

**Sequence file:**	promoters.seq (3 seq.)
**Type of sequences:**	nucleotide sequences
**Threshold T:**	0.00
**'*' signs below alignment denote:**	diagonal similarity (max. similarity is represented by 10 '*' signs)
**Alignment output:**	complete alignment is shown<br># nucleic acids per line: 50
**TF output:**	common TF matches located in aligned regions (common to 3 (100.0 %) sequences)
**Family matches:**	yes
**MatInspector library:**	Matrix Family Library Version 5.0 (February 2005)
**Selected groups** (core/matrix sim):	• ALL user_defined.lib (0.75/Optimized)<br>• ALL vertebrates.lib (0.75/Optimized)

Aligned Sequences:

No.	Sequence Name	Sequence Description	Length
1	P865912	sym=NDUFAF1|loc=Loc51103|taxid=9606|spec=Homo sapiens|chr=15|ctg=NT_010194|str=(-)|start=12484677|end=12485677| len=1001|tss=501| comm=NADH dehydrogenase (ubiquinone) 1 alpha subcomplex, assembly factor 1; (NM_016013/501/bronze;)	1001 bp
2	P865914	sym=Ndufaf1|loc=Loc69702|taxid=10090|spec=Mus musculus|chr=2|ctg=NT_039207|str=(-)|start=60384597|end=60385597| len=1001|tss=501| comm=NADH dehydrogenase (ubiquinone) 1 alpha subcomplex, assembly factor 1; (AK010328/501/gold;NM_027175/501/bronze;)	1001 bp
3	P865917	sym=nalloc=Loc296086|taxid=10116|spec=Rattus norvegicus|chr=3|ctg=NW_047657|str=(-)|start=47582514|end=47583514| len=1001|tss=501| comm=similar to NADH dehydrogenase (ubiquinone) 1 alpha subcomplex, assembly factor 1; (XM_215814/501/bronze;)	1001 bp

Alignment (DiAlign format):

Please note that only **upper-case** letters are considered to be **aligned**. For more information, please have a look at the user guide.

Warning: No common TF matches found.

```
alignment position  1.........  11........  21........  31........  41........
P865912          1  ccgtctctac  taaaaataca  aaaattagtc  gggcatgatg  gcgcgcacct
P865914          1  gtgtgcacaa  gctctgttgt  gtggattaca  taGGGGCACG  ACTGAGGAAA
P865917          1  ggatt atgtg g---------  ----------  --GGGGCACG  ATTGAGGAAA
```

```
                                                                    ******** **********
                                                                    ******** **********
                                                                    ******** **********
                                                                    ******** **********
                                                                    ******** **********

alignment position  51........ 61........ 71........ 81........ 91........
P865912         51  gtaatcccag ctactcggga ggctgaggca ggagaatcgc ttgaatct--
P865914         51  GCCAGGATTG ATAGCTATCT AGGTGTACAG TGGCTCTCCA AAGGCAGTTA
P865917         30  GCCTGGATTG GTTGCTATCT AGGTGTACAG TGGCTCTCCA GAAGCAGTTA
                    ********** ********** ********** ********** **********
                    ********** ********** ********** ********** **********
                    ********** ********** ********** ********** **********
                    ********** ********** ********** ********** **********
                    ********** ********** ********** ********** **********
                                           ********* ********** **********

alignment position  101....... 111....... 121....... 131....... 141.......
P865912         99  ---------- ---------- ---------- ---------- ----------
P865914        101  AGTGGCTTTC TaAAGTCACG CAGCAGACAG TTCTCAAGGG ACTACAGGGA
P865917         80  AGTGGATTTC TgAAGTCACA CAGCAGACAG TTCTCAAGGG ACTACAGGGA
                    ********** * ******** ********** ********** **********
                    ********** * ******** ********** ********** **********
                    ********** * ******** ********** ********** **********
                    ********** * ******** ********** ********** **********
                    ********** * ******** ********** ********** **********
                    ********** * ******** ********** ********** **********
                                 ******** ********** ********** **********

alignment position  151....... 161....... 171....... 181....... 191.......
P865912         99  ---------- ---------- ---------- -GGGAGGCAG AGGTTGTAGT
P865914        151  AGaagaggcc CAAGCGGCCA GGCatctagA AGTGAAGAGG CGATAGCAGT
P865917        130  AGacaca--- CAAGTGGCCA GGC------A AGTGAAGAGG AGTTAGTAGT
                    **         ********** ***      * ********** **********
                    **                             * ********** **********
                    **                             * ********** **********
                    **                             * ********** **********
                    **
                    **
                    **

alignment position  201....... 211....... 221....... 231....... 241.......
P865912        118  GAGCCGAgat cgcgccactg cactccagcc tgggcgacag agcgagaatc
P865914        201  GCAGAGATCC TGTTTAAAAg ---------- ---------- ----------
P865917        171  GCGGAGATCC TGCTTGAAAa ataggggttg gggatttagc tcagtggtag
                    ********** *********
                    ********** *********
                    ********** *********
                    ********** *********

alignment position  251....... 261....... 271....... 281....... 291.......
P865912        168  cg-------- ---------- ---------- ---------- ----TCTCAA
P865914        221  ---------- ---------- ---------- ---------- ----------
P865917        221  agcgcttgcc taggaagcgc aaggccctgg gttcggtccc aagcTCCGAA
                                                                   ******
                                                                   ******

alignment position  301....... 311....... 321....... 331....... 341.......
P865912        176  AAAAAAAAAA AAAAAAAAAG Acgcagggat ggagtttgcc gcctgaaa-G
P865914        221  ---------- ---------- ---AGGATTG GTATTTACAT ACGGGGGCGG
P865917        271  AAAAAGAAAA AGGAAAAAAT AaaAGGATTG ATATTTACAT ATGAGGGCGG
                    ********** ********** *  ******* ********** **********
                    ********** ********** *  ******* ********** **********
                                             ******* ********** **********
                                             ******* ********** **********

alignment position  351....... 361....... 371....... 381....... 391.......
P865912        225  GATACAGGAA GGAGAGgcga aaaacttatc tgaattctca ctgcttcctt
P865914        248  GGTACAAGAA GCTGAGCGAA GAGCTGGCTC ACAAATGACA GGAcATTTCA
```

```
P865917      321 ATTACAAGAA GTAGAGCGAA GAGCTCGCTC ACAAAGGACA GGAaATTTCA
                 ********** ********** ********** ********** *** ******
                 ********** *  ******* ********** ********** *** ******
                 ********** *  ******* ********** ********** *** ******
                 ********** *  ******* ********** ********** *** ******
                                                                 ******

alignment position 401....... 411....... 421....... 431....... 441.......
P865912      275 acaactggcg accaagtgga ccaaaaacct CCAAAACCT CAGttggctg
P865914      298 GAAAGCGACC CCGCTGGGGA ATTTTGAggA CCTGAAGCCT CCGGTTGTCT
P865917      371 GAAAGCGAAC CCGCTGGGGA ACTTTGAatA CCAAAAGCCT CAGGTTGTCC
                 ********** ********** *******  * ********** **********
                 ********** ********** *******  * ********** **********
                 ********** ********** *******  * ********** **********
                 ********** ********** *******    ********** ***
                 ********** ********** *******

alignment position 451....... 461....... 471....... 481....... 491.......
P865912      325 g--GCTACGG GGTCCTTGCT GTCCTTCCTG GGCTtctgta ctcggccgga
P865914      348 TACGCTTTAG GATACTTTCC GTTACTCTTG AACTATCTGA AGCTCTTgtt
P865917      421 CATGCTTTAG GATACCTTtt GGTACTCTTG TACGACCTGA AACTCTA---
                 ********** ********** ********** ********** *******
                 ********** ********   ********** ********** *******
                 ********** ********

alignment position 501....... 511....... 521....... 531....... 541.......
P865912      373 actttggtgt tctgACGCCT TGTTTGGCAT CGGAAGGGAA AAGCAGATGG
P865914      398 tactgT---- ----ACACCT AGTTTGATGT CGGACAGGAA AAGTAGCGGG
P865917      468 -----T---- ----ACACCT AGTTTGGTGC CGGACGGGAA AAATAGATGG
                      *         ****** ********** ********** **********
                      *         ****** ********** ********** **********
                      *         ****** ********** ********** **********
                      *         ****** ********** ********** **********
                                ****** ********** ********** **********
                                ****** ********** ********** **********
                                ****** ********** ********** **********
                                ****** ********** ********** **********

alignment position 551....... 561....... 571....... 581....... 591.......
P865912      423 ACCTatacgg gtaaagtggc tt-------- ---------- -----CTGGG
P865914      440 ACCacaACCG GAAGCGTGGC GAACCGGGCG AGAAAAGTAG AAGGACtatt
P865917      505 ACCTgcACCG GAAGCGTGGA GAACCGGGCG GAGAAAACAG AAGGACTGGC
                 ****  **** ********** ********** ********** **********
                 ****  **** ********** ********** ********** ******
                 ****  **** ********** ********** ********** ******
                 ***   **** ********** ********** ********** ******
                 ***
                 ***
                 ***
                 ***

alignment position 601....... 611....... 621....... 631....... 641.......
P865912      450 C---GGAAGg tac------- ---------- ---------- ----------
P865914      490 cgggGGAAGC CGAATGTG-- GGGTCTGAGA AGGTGAGCGG AACCTTCTCA
P865917      555 C---GGAAGC CATATGTGgg GGGTCTGAGA AGGTAAGCTG ACCCTTCTCC
                 *   ****** ********   ********** ********** **********
                                       ********** ********** **********
                                       ********** ********** **********
                                       ********** ********** **********
                                       ********** ********** **********

alignment position 651....... 661....... 671....... 681....... 691.......
P865912      460 ---------A CTATAGGCTC GGGGAGGTaa gcggcggcag gccggcggtt
P865914      538 TAGGTGGGAA CTGCAGGGAC GAGGAGGTCG GGA------- ----------
P865917      602 TAGATGGGAA actCAGGGAC AAGGAGGCGG GGA------- ----------
                 ********** ********** ********** ***
                 **********    ******* ********** ***
                 **********    ******* ********** ***
                 **********    ******* ********** ***
```

```
                     **********
alignment position   701....... 711....... 721....... 731....... 741.......
P865912          501 ggtgtgtccc gggtgtgggg aggcgacAGA GCCCTGGC-- ---ACTTGAG
P865914          571 ---------- ---------- -------AGA GTCCTGGCgg gtgAGGTGAG
P865917          635 ---------- ---------- -------AGA GTCCTGGC-- ---AGGTGAG
                                                   *** ********     *******
                                                   *** ********     *******
                                                   *** ********     *******
                                                   *** ********     *******
                                                   *** ********        ****
                                                   *** ********
alignment position   751....... 761....... 771....... 781....... 791.......
P865912          546 GGTTGAGGGG GCCTCcccag cgcggcgaac CGTCTAGCCT CCGGAGGCCA
P865914          594 GGTCGAGGGG CtgcCACTCC TCGGGAG--- CGCCTAGCTT CAGGAGGCTG
P865917          653 GGTTGAGGGG CCGTCACTCC TCGGGAG--- CGCCTAGCTT CCGGAGGCTG
                     ********** ********** *******    ********** **********
                     ********** ********** *******    ********** **********
                     ********** ********** *******    ********** **********
                     ********** *   ****** *******    ********** **********
                     **********     ****** *******    ********** **********
                                    ****** *******    ********** **********
                                    *                 ********** **********
                                    *                 ********** ********
alignment position   801....... 811....... 821....... 831....... 841.......
P865912          596 GGCCGTGagt g--------- ----CGGGAG GTATACGCCA AGGCGGAAGA
P865914          641 GGACGTGacc aaccccgCGC CTGCCGAGAG GTGAACGCCT GGTCTTCAGA
P865917          700 GGgacgtggt taactc-CGC CAGCCGAGAG GTGAACGCCT GGTCTTCAGA
                     *******           *** ********** ********** **********
                     *******           *** ********** ********** **********
                     **                *** ********** ********** **********
                     **                *** ********** ********** **********
                     **                *** ********** ********** **********
                     **                *** ********** ********** **********
                     **
alignment position   851....... 861....... 871....... 881....... 891.......
P865912          633 ATtttgccac tcactaccTG TGTGACCTCG GGTAAattag ccttggaacg
P865914          691 GTGAgTTTGG TCTCTGG-TG TGTGACCTCG AATAAttATT TAATTGTGGA
P865917          749 GTGAcTTTTG TCTCTGG-TT TGTGATCTCG AATA---ATT TAATTGTGGA
                     **** ***** ******* ** ********** *****  *** **********
                     **** ***** ******* ** ********** ****   *** **********
                     **** ***** ******* ** ********** ****   *** **********
                     ****
                     ****
                     ****
alignment position   901....... 911....... 921....... 931....... 941.......
P865912          683 tcag----TT TCTTCGTCTC TATAATTGAA ATAATAATAG tacctctc--
P865914          740 ATCTCCTTTT TTTTAGTCTT TGTAATTGGA ATAATACTAG AAGT-ACCTG
P865917          795 GTCTCCTTTT cgTTAGTCTT TGTCATTGGA ATAATACTAG AAGTtACCCG
                     ********** ********** ********** ********** **** *****
                     ********** ********** ********** ********** **** *****
                     **********   ******** ********** ********** **** *****
                             **   ******** ********** ********** **** *****
                             **   ******** ********** ********** **** *****
                                  ******** ********** **********
                                  ******** ********** *********
alignment position   951....... 961....... 971....... 981....... 991.......
P865912          727 --------TC AGGATTGTTG TGAGCCGTCA GTGAA---AC ACTTAGAGCA
P865914          789 TCTCAGGGTC AGGATTATCG TGAGAAGTCA GTAAAAACAC ACGGAGAATA
P865917          845 TCTCAGGATC AGGATTATTG TGAGAAATCA ATAAAAACAC ACGGAGAATA
                     ********** ********** ********** ********** **********
                     ********** ********** ********** ********** **********
                     ********** ********** ********** ********** **********
                     ********** ********** ********** ********** **********
```

```
                              ********** ********** ********** ********** **********
                                      ** ********** ********** ********** **********
                                      ** ********** ********** *****   ** **********
                                                                       ** **********
                                                                       ** **********
                                                                        * **********

alignment position 1001...... 1011...... 1021...... 1031...... 1041......
P865912        766 GTTTCTGGCA CATGGTAAGG CATGGATAGc tttactaatt ctactcactt
P865914        839 GTTGCTGGCA CATGGGAAGC CCTAGGTTGA ACAGTCGTTT CTGTACTCAG
P865917        895 GTTTCTGGCA CATGGTAAGC CCTAGATt-A ACAGTCGTTT CTGTACATAG
                   ********** ********** ********** ********** **********
                   ********** ********** ********** ********** **********
                   ********** ********** *******  * ********** **********
                   ********** ********** *******  * ********** **********
                   ********** ********** *******  * ********** **********
                   ********** ********** *****
                   ********** ********** *****
                   ********** ********** *****
                   ********** ********** *****
                   ********** ********** *****

alignment position 1051...... 1061...... 1071...... 1081...... 1091......
P865912        816 ct-------- ---------- -AGCTGCGTG GATTGTGTTG TCAAAACCCA
P865914        889 TAGTACAGTC ATActgCTAG GAGCGGTGTG AATATCTTAT TAAAAACTCA
P865917        944 TAGTACAGTC ATAgtaCTAG GAGCGATGTG AACACCCTAT TAAAAACTCA
                   ********** ***   **** ********** ********** **********
                   ********** ***   **** ********** ********** **********
                   ********** ***   **** ********** ********** **********
                   ********** ***   **** ********** ********** **********
                   ********** ***   **** ********** ********** **********

alignment position 1101...... 1111...... 1121...... 1131...... 1141......
P865912        847 CCGTCAACCT Cagggcggta ttgtccagtt aatagtttag atttttctgtt
P865914        939 CAGTTAGCTT Cggaccgacg tcacccggtt actgagtttg accgagtttc
P865917        994 CCGTTAgc-- ---------- ---------- ---------- ----------
                   ********** *
                   ******
                   ******
                   ******
                   ******

alignment position 1151...... 1161...... 1171...... 1181...... 1191......
P865912        897 tactggtaga aaaaaaaaaa tgaaacaaag catttataat taaagttata
P865914        989 ttgtaagaaa tta------- ---------- ---------- ----------
P865917       1002 ---------- ---------- ---------- ---------- ----------

alignment position 1201...... 1211...... 1221...... 1231...... 1241......
P865912        947 ttattagaga agtaacacta cggagggttt ggaaaacatt ttatgagcta
P865914       1002 ---------- ---------- ---------- ---------- ----------
P865917       1002 ---------- ---------- ---------- ---------- ----------

alignment position 1251.
P865912        997 tcatt
P865914       1002 -----
P865917       1002 -----
```

Pairwise similarities:

For each pairwise alignment, the similarity (relative to the maximum similarity) and the number of identical nucleic acids (in % of shorter sequence) is given. Maximum values are underlined.

	**P865914**	**P865917**

	(1001 bp)	(1001 bp)
**P865912 (1001 bp)**	0.097<br>28 %	0.143<br>28 %
**P865914 (1001 bp)**		1.000<br>74 %

Please note that the **similarity value 1.000** marks only the two **most similar** sequences, it does not necessarily mean that these sequences are identical.

**Extract alignment region**	
**Positions in alignment**	from: to:
**Output file**	dialign.seq

Extract aligned sequences

For comments, questions or bug reports, please contact support@genomatix.de

© Genomatix Software GmbH 1998-2005 - All rights reserved.

License Agreement

GEMS Launcher 4.1.1

Genomatix-Logo	Logout<br>Logout	Personal<br>Personal	Messages<br>Messages	GEMS-Logo

Genomatix MainGEMSElDoradoGene2PromoterBiblioSphere
Frequently asked questionsYour ResultsYour SequencesYour ProtocolHelp

GEMS Launcher Task: *DiAlign TF*: Multiple alignment plus TF sites
working on C1-NDUFB1 (3 seq.)

[Alignment] [Pairwise similarities]

DiAlign professional TF Release 3.1 December 2004 **Tue Jun 7 14:05:14 2005**

Solution parameters:

**Sequence file:**	C1-NDUFB1 (3 seq.)
**Type of sequences:**	nucleotide sequences
**Threshold T:**	0.00
**'*' signs below alignment denote:**	diagonal similarity (max. similarity is represented by 10 '*' signs)
**Alignment output:**	complete alignment is shown<br># nucleic acids per line: 50
**TF output:**	common TF matches located in aligned regions (common to 3 (100.0 %) sequences)
**Family matches:**	yes
**MatInspector library:**	Matrix Family Library Version 5.0 (February 2005)
**Selected groups** (core/matrix sim):	• ALL user_defined.lib (0.75/Optimized)<br>• ALL vertebrates.lib (0.75/Optimized)

Aligned Sequences:

No.	Sequence Name	Sequence Description	Length
1	P303789	P303789 sym=NDUFB1|loc=Loc4707|taxid=9606|spec=Homo sapiens|chr=14|ctg=NT_026437|str=(-)|start=73587406|end=73588406|len=1001|tss=501|comm=NADH dehydrogenase (ubiquinone) 1 beta subcomplex, 1, 7kDa; (NM_004545/501/bronze;)	1001 bp
2	mNDUFB1	mNDUFB1 m5_dna range=chr12:96654325-96656324 5'pad=0 3'pad=0 revComp=TRUE strand=? repeatMasking=none	2000 bp
3	rNDUFB1	rNDUFB1 n3_dna range=chr6:126249139-126250606 5'pad=0 3'pad=0 revComp=TRUE strand=? repeatMasking=none	1468 bp

Alignment (DiAlign format):

Please note that only **upper-case** letters are considered to be **aligned**. For more information, please have a look at the user guide.

V$STAT V$IKRS V$YY1F V$NRF1 V$EREF V$RREB

```
alignment position 1.........  11........  21........  31........  41........
P303789          1 gtca------  ----------  ----------  ----------  ----------
mNDUFB1          1 gaaactagGA  ATGGGATCTT  GGGAAacgtg  GTGATTGTGG  tagcgccgag
rNDUFB1          1 --------GA  ATGGGATCTT  GGGAAtca--  GTGATTGTGG  aggcactaag
                           **  **********  *****       **********
                           **  **********  *****

alignment position 51........  61........  71........  81........  91........
P303789          5 ----------  ----------  ----------  ----------  ----------
```

```
mNDUFB1             51 a--GAAGTTA ATTCTATCAA GGCGTGGCAT gagCCTCTCA AGGGTCTTTG
rNDUFB1             41 cgcGACGTTA ATTCTAGCAA GGCGTGGCAT aaaCCTCTCA AGGGTCTTTG
                          ******* ********** **********    ******* **********
                          ******* ********** **********    ******* **********
                          ******* ********** **********    ******* **********
                                                           ******* **********

alignment position     101....... 111....... 121....... 131....... 141.......
P303789              5 ---------- ---------- ---------- ---------- ----------
mNDUFB1             99 GAATTAAGGA GCTACGAAGG CACctgCTGG CCTCGTGTTC TCTGAGCCCA
rNDUFB1             91 AAGTTAAGGA GCTAGGAGGG CAC---CTGG CCTCGTGGTC TCTGAACCCA
                       ********** ********** ***   **** ********** **********
                       ********** ********** ***   **** ********** **********
                       ********** ********** ***   **** ********** **********
                       ********** ********** ***   **** ********** **********
                                                   **** ********** **********

alignment position     151....... 161....... 171....... 181....... 191.......
P303789              5 ---------- ---------- ---------- ---------- ----------
mNDUFB1            149 AGATGGGAAA GGCAgAGATG GGGAGGCTTG GGGAGTACGG CGGTACAAGG
rNDUFB1            138 AGATGGGAAA GGCAcAGATG GGGAGGCTTG GGGAGTAAAG CGGCAACAGG
                       ********** **** ***** ********** ********** **********
                       ********** **** ***** ********** ********** **********
                       ********** **** ***** ********** ********** **********
                       ********** ****
                       ********** ****

alignment position     201....... 211....... 221....... 231....... 241.......
P303789              5 ---------- ---------- ---------- ---------- ----------
mNDUFB1            199 GGAAaGAGCC CAGGCTGGAC GCGGATTAAC TTTTAGCTCC AGctGATGAA
rNDUFB1            188 GGAA-GAGCC CAAGCAGGAC TCCGATTAAC TTTTAGTTCC AGagGATGAA
                       **** ***** ********** ********** ********** **  ******
                       **** ***** ********** ********** ********** **  ******
                       **** ***** ********** ********** ********** **

alignment position     251....... 261....... 271....... 281....... 291.......
P303789              5 ---------- ---------- ---------- ---------- ----------
mNDUFB1            249 AGCTCAGAGG AACTAGACAG GAGGgagggc -------AAG GAGGAAGCAT
rNDUFB1            237 AGATCAGAGG AAATGGACAG AAGGagggca aggggcaAAG GAGGAACCAT
                       ********** ********** ****              *** **********
                       ********** ********** ****              *** **********

alignment position     301....... 311....... 321....... 331....... 341.......
P303789              5 ---------- ---------- ---------- -CTTCTCTT- ----------
mNDUFB1            292 AGGCGCGCCC C-AACACCCC GAGGTTTCAG CCcggggaaa agtcacttct
rNDUFB1            287 AGGAGCGCTC CaAACACCCC GAGGCTTCAG CCTTCTCTT- ----------
                       ********** *  ******** ********** *********
                       ********** *  ******** ********** *********
                                                          *

alignment position     351....... 361....... 371....... 381....... 391.......
P303789             13 ----TCCCTC GCTGACCACT TCTGCCGGGA ACGACCGGGA CAGTCGCAGC
mNDUFB1            341 cttcCCCCTT GCGGAACGCT TGTTCCAGGA ACCACGGAGA CTGTCACAAC
rNDUFB1            326 ----CCCCTT GCGGAACGCT TGTTCCAGGA ACCACGGAGA CGGACACAAC
                           ****** ********** ********** ********** **********
                           ****** ********** ********** ********** **********
                           ****** ********** ********** ********** **********
                           ****** ********** ********** ********** *   ******
                           ****** ********** ********** ********** *   ******
                           ****** ********** ********** ********** *   ******
                            ***** ********** ********** **  ****  ***  ******
                                                                       ******

alignment position     401....... 411....... 421....... 431....... 441.......
P303789             59 CGAAGCGGCG GGGGCGAGTC CtcGGGGGAG ACCGGGTCGG AGTCGGGGCC
mNDUFB1            391 CGAAACCGCG GGAGCGCGTC C--GGGCGAC ACCGGGCCGA CGCGGGAACC
rNDUFB1            372 CGAAACCGCG GGAGCGCGTC C--GGGCGAC ACCGGGCCGa tGCGGGAACC
```

```
                        **********  **********  *  *******  **********   *********
                        **********  **********  *  *******  ******       *********
                        **********  **********  *  *******  ******       *********
                        **********  **********  *  *******  ******       *********
                        **********  **********  *  *******  ******       *********
                        **********  **********  *  *******  ******
                        **********  **********  *
                        **********  **********  *
                        **********  **********  *

alignment position      451.......  461.......  471.......  481.......  491.......
P303789           109   CcgtGCTCCC  GGAGGGCCaa  tgacgccgcC  TTCCCCAGCC  CG--AGGCTC
mNDUFB1           439   CA--TCCCCT  TGAGGGCCT-  ---------C  CTCCCAACCC  GA--AGGCTC
rNDUFB1           420   CA--GCTCCT  TGAGGGCCT-  ---------C  CTCCCAACCC  GAgaAGGCTC
                        **  ******  *********            *  **********  **  ******
                        **  ******  *********            *  **********  **  ******
                        **  ******  *********            *  **********  **
                        **  ******  *********            *  **********  **
                        **  ******  *********            *  **********  **
                                                         *  **********  **

alignment position      501.......  511.......  521.......  531.......  541.......
P303789           157   GCTCCTTTCT  CCCGCCCTCA  CTTGCCTCGT  CAGgcCTCCC  CCAACG-CCG
mNDUFB1           475   CTTTCCGTCT  CCCGTCCTCA  CCTGCCAGGC  CAAT-CTCCC  CCcggcaCCG
rNDUFB1           458   CTTTC-TTCT  CCCATCCTCA  CCTGCCAGGT  CAAT-CTCCC  CCGGTA-CCG
                        **********  **********  **********  **** *****  ****** ***
                        **********  **********  **********  **** *****  ****** ***
                               ***  **********  **********  **** *****  ****** ***
                               ***  **********  **********  **** *****  **     ***
                               ***  **********  **********  ***  *****  **     ***
                               ***  **********  *                *****  **

alignment position      551.......  561.......  571.......  581.......  591.......
P303789           206   TCCACAGCCC  TTCCCAACCC  CTAGGCGAAT  CCAGGTTCAA  GACGTAGAAA
mNDUFB1           524   TCCGCCGCCC  TTCCCAACCC  CTAGTGGAAC  GCAGGTCGGA  GAGCGAGCAG
rNDUFB1           505   ACCGCTGCCC  TTCCCAACCC  CTAGTGGAAC  GCAAGTCGGA  GAGCGAGTAG
                        **********  **********  **********  **********  **********
                        **********  **********  **********  **********  ******
                        **********  **********  **********  **********  ******
                        **********  **********  **********  **********  ******
                        **********  **********  **********  **********  ******
                              ****  **********  **********  **********  ******
                              ****  **********  **********  ******
                              ****  **********  **********  ******
                              ****  **********  ****
                              ****  **********  ****

alignment position      601.......  611.......  621.......  631.......  641.......
P303789           256   AGCCGATGCT  GGGTCAGCAG  CGccagggac  taccgGCCGG  CAGAAGCCCC
mNDUFB1           574   AGCTAGCGCG  CGCTCAGCGG  CGGAGAGCA-  -----GCCGG  CAGGAGCCCC
rNDUFB1           555   ACCTAGCGCG  CGCTCAGCGG  CGGAGAACA-  -----GCCGG  CAGGAGCCTC
                        **********  **********  *********        *****  **********
                          ********  **********  *********        *****  **********
                          ********  **********  *********        *****  **********
                          ********  **********  *********        *****  **********
                          ********  **********  *********        *****  ******** *
                          ********  **********  *********        *****  ******** *
                                                                 *****  ******** *
                                                                 *****  ******** *
                                                                 *****  ******** *

alignment position      651.......  661.......  671.......  681.......  691.......
P303789           306   ACAGTGGCAG  CCGCCATTTT  GGaGCTGGAG  ACTAGCGGCG  GAGACGAGGA
mNDUFB1           618   TGTGTGGTGG  CCGCCATCTT  GG-GCCTGAG  CAGAGCGGCT  GAGGCGGGGA
rNDUFB1           599   TGTGTGGTGG  CCGCCATCTT  GG-GCCTGAG  CAGCGCGGCT  GAGGCGGGCA
                        **********  **********  ** *******  **********  **********
                        **********  **********  ** *******  *** ******  **********
                        **********  **********  ** *******  *** ******  **********
                        **********  **********  ** *******  *** ******  **********
                        **********  **********  ** *******  *** ******  **********
```

```
                         **********  **********  **              ******  **********
                         **********  **********  **
                         **********  **********  **
                         **********  **********  **
alignment position      701.......  711.......  721.......  731.......  741.......
P303789           356   GGAaCCAGGG  CGAGACCATC  TCCGGGGGgt  gggcacggtg  ctgtc--GGA
mNDUFB1           667   CGA-CCCGGG  CGACACCATC  TGTAGGGGTG  GTGACGAACC  GGGTCAGGGA
rNDUFB1           648   CGA-CCCGGG  CGACACCATC  TGTAGGGGTG  GCGAGGAACC  GGTTCAGGGA
                         *** ******  **********  **********  **********  **********
                         *** ******  **********  **********  **********  **********
                         *** ******  **********  ********
                         *** ******  **********  *****
                         *** ******  **********  *****
                         *** ******  **********  *****
                             ******  **********  *****
alignment position      751.......  761.......  771.......  781.......  791.......
P303789           404   AGGCGACGGC  Ggtcacgcct  GCACGCCTCG  TCTGTCTGGA  GCGAAATTTT
mNDUFB1           716   AGAGGACAGC  GCaaAAGCTC  GCAAACCTTG  TCAGCCTGGA  TCCACGTCTT
rNDUFB1           697   AAGTGACGGC  GCc-AAGCTC  GCAAACCTTG  TCAGCTTGGA  TCCAAGTTTT
                         **********  **  ******  **********  **********  **********
                         **********  **  ******  **********  **********  **********
                                         ******  **********  **********  **********
                                         ******  **********  **********  **********
                                                 **********  **********  **********
                                                 **********  **********  **********
alignment position      801.......  811.......  821.......  831.......  841.......
P303789           454   ATTCAGAAat  tattggtaat  gtttggaagc  taaaaaaaaa  AAAAATGAGA
mNDUFB1           766   ATTCAaaacg  tgtcgcgatc  ATTCACAGCT  T---------  AAGATGTACG
rNDUFB1           746   ATCCAGAAcc  tatcacgat-  ATTTAAAGCT  C---------  AAAATGTAGA
                         ********                **********  *           **********
                         ********
                         **
                         **
                         **
                         **
alignment position      851.......  861.......  871.......  881.......  891.......
P303789           504   GGAGTCAACC  CTGAGGAGGA  AAAagtagta  tgatttgctg  gcgt--CA-C
mNDUFB1           807   GGATTCGCGC  GTGAGAAGGG  AGAGAGAAGC  GCCGGTTGCC  CGACGCCAcC
rNDUFB1           786   GGACTCACTC  GTGAGAAGGG  AGAGAGAGGC  GTCGATTGGC  CATCGCCA-C
                         **********  **********  **********  **********  ******** *
                                 *  **********  **********  **********  ******** *
                                 *  **********  **********  **********  ******** *
                                 *  **********  ***                           ** *
                                                                               ** *
                                                                               ** *
                                                                                  *
                                                                                  *
                                                                                  *
                                                                                  *
alignment position      901.......  911.......  921.......  931.......  941.......
P303789           551   CCCTCTGCTC  CGTGCGGGCG  CGGCGAATGG  CAGGTCCcgA  GGTCGCAGCT
mNDUFB1           857   CCCTCTCATC  AGGGCTGGCG  CGGCGAGTGG  CAGCTCCTTA  GGTCACGGCT
rNDUFB1           835   CCCTCTCCTC  AGGGCGGGCG  CGGCGAGTGG  CAGCTCCTTg  GGTCACGGCT
                         **********  **********  **********  **********  **********
                         **********  **********  **********  *********   **********
                         **********  **********  **********  *********
                         **********  **********  **********  *********
                         **********  **********  **********  *********
                         **********  **********  **********  *******
                         **********  **********  **********  *******
                         **********  **********  **********  *******
                         **********  **********  **********  *******
                         **********  **********  **********  *******
alignment position      951.......  961.......  971.......  981.......  991.......
```

```
P303789      601 TCCACTGGCG CGGGTTGagt tcc------C TGTTGCCCTT GGTCTCGGGG
mNDUFB1      907 TCCGCCGGCG CCAGTGGCCT AGCTCCCTGC GGCTGTCCTT GGTCGCCTTG
rNDUFB1      885 TCCGCCGTCG CCAGTGGCCT AGCTCCCTGC GGCTGTCCTT GGTCGCGctt
                 ********** ********** ********** ********** **********
                 *******  ** ********* ********** ********** ******
                          ** ********* ********** ********** ******
                          ** ********* ********** ********** ******
                          ** ********* ********** ********** ******
                          ** ********* ********** ********** ******
                          ** *******

alignment position 1001...... 1011...... 1021...... 1031...... 1041......
P303789      645 T-CGCtgtag gcgctgAGGC TGCAGGTGGG TTTGGGGAGC CCGGggggcc
mNDUFB1      957 T-CACC---- ------AGGC TGAAGGTGGG TCTGGGGACC CCGGCGCCAC
rNDUFB1      935 gaCACC---- ------GGGC TGAAGGTGGG TCTGGGGACC CCAGTGCCAC
                 * ****           **** ********** ********** **********
                   ****           **** ********** ********** **********
                   ****           **** ********** ********** **********
                   ****           **** ********** ********** **********
                   ****           **** ********** ********** **********
                                  **** ********** ********** ****
                                  **** ********** ********** ****
                                   *** ********** ********** **

alignment position 1051...... 1061...... 1071...... 1081...... 1091......
P303789      694 gccgcgacga ggatcacggc gacggtggcg GGGAGGTTCT AGGCAAgcc
mNDUFB1      996 CC---GTGGT GGTGatgcgG GC-------- GGGAGGTTCT GGGGAACGC
rNDUFB1      975 CCtctGTGGT GGTGca---G GC-------- GGGAGGTTCT GGGGAACGC
                 **   ***** ****     * **        ********** **********
                 **                  * **        ********** **********
                 **                  * **        ********** **********
                 **                  * **        ********** **********
                 **                  * **        ********** **********
                                     * **        ********** **********

alignment position 1101...... 1111...... 1121...... 1131...... 1141......
P303789      744 ctcggtgctg tgcatgtgtg gaggggtggt ctgggcttcc ccggggcttc
mNDUFB1     1035 TCGCAT---- ---------- ---------- ---------- ----------
rNDUFB1     1014 TCTCAT---- ---------- ---------- ---------- ----------
                 ******
                 ******
                 ******
                 ******
                 ******
                 ******

alignment position 1151...... 1161...... 1171...... 1181...... 1191......
P303789      794 cgaacgaagg tgacgGGAAA GGAAAATGGG GTTTTCTTCG GGTGgaggat
mNDUFB1     1041 ---------- -----GGAGG GGAAAca-GG GTCGCCTTCG G-TGACCCCT
rNDUFB1     1020 ---------- -----GGAGG GGAAATCGGG GTCGCCTTCG GGTGACCTCT
                                 ***** ********** ********** **********
                                 ***** *****                  ********
                                 ***** *****                  **
                                 ***** *****
                                 ***** *****
                                 ***** *****

alignment position 1201...... 1211...... 1221...... 1231...... 1241......
P303789      844 gg--GATTTC CGGGCCGGAG CTTTTGGAAA GGGTT----- ----------
mNDUFB1     1074 GCGCGCTGCC CGCCCCACGC CTTTTACAAA GACTG----- ----------
rNDUFB1     1055 GCGCGCTGTC AGCTTCACAC CTTTCACAGA GAattcgcta tgctagctta
                 ********** ********** ********** *****
                 ********** ********** ********** **

alignment position 1251...... 1261...... 1271...... 1281...... 1291......
P303789      877 ---------- TTCTGAGGct gtcataacag gaaaacgtcc gtagagatgt
mNDUFB1     1109 ---------- CTCTGGGGAC ---------- ---------- ----------
rNDUFB1     1105 atcaaagcgt CTTTGGGGAC ---------- ---------- ----------
```

```
                                   **********
                                   **********
                                   **********
                                   **********

alignment position   1301......  1311......  1321......  1331......  1341......
P303789          917 GACCCAGTGA  CCGCCCGCTA  ACCGGACTGG  AGAGaagacc  ggccc-----
mNDUFB1         1119 GATCTGATGG  CCTGGCTCTC  GGGGGACTcG  GAAGGGTTCT  CAAGGATCCG
rNDUFB1         1125 GATCCGATGG  CCTCGCTCTC  GAGGGACTAG  GAAGGGCTCT  CAAGGATCCC
                     **********  **********  **********  **********  **********
                     **********  **********  ********  *  **********  **********
                     **********  **********  ********  *  **********  **********
                     **********  **********  ********

alignment position   1351......  1361......  1371......  1381......  1391......
P303789          962 ----------  ----------  GGATCCCACG  CCTGGCCTGA  AGCAGGGAAg
mNDUFB1         1169 ACTCTTGAGC  CCACAaTAGG  GAATGCAGCT  CCTATCCAGA  AGCGGGGcgc
rNDUFB1         1175 ATTCTTGAAC  CCACAgTAGG  GAATGCAGCT  CCTATCCAGA  AGCAGGGAAc
                     **********  ***** ****  **********  **********  *********
                     **********  ***** ****  **********  **********  *******
                     **********  ***** ****  **********  **********  *******
                                       ****  **********  **********  *******

alignment position   1401......  1411......  1421......  1431......  1441......
P303789          992 acgctgcctc  ----------  ----------  ----------  ----------
mNDUFB1         1219 ggTCGCCCAG  GCGTCTGTCT  CCTCGTCCAA  CTCTCCTgat  tGTGAAACCC
rNDUFB1         1225 atTCGCCCAG  GCATCCGCCT  CCTGGTTCAA  CTCTCCTaaa  cGTGAAACCC
                       ********  **********  **********  *******      *********
                       ********  **********  **********  *******      *********
                       ********  **********  **********  *******      *********
                                                                      *********
                                                                      *********

alignment position   1451......  1461......  1471......  1481......  1491......
P303789         1002 ----------  ----------  ----------  ----------  ----------
mNDUFB1         1269 GCACGTAGCC  ATTTGCCTTT  TAAAAACAaG  GCTTTCTGCT  TGGTTTCCAG
rNDUFB1         1275 GCACTTACCC  ATTTGCCTTT  TAAAAACAtG  GCTTTCTGCG  TGGTTTCCAG
                     **********  **********  ********  *  **********  **********
                     **********  **********  ********  *  **********  **********
                     **********  **********  ********
                     **********  **********  ********
                     **********  **********  ********

alignment position   1501......  1511......  1521......  1531......  1541......
P303789         1002 ----------  ----------  ----------  ----------  ----------
mNDUFB1         1319 tccg----TG  TTATGATCTC  AACGGTTTCg  gttttgctcc  atcca-----
rNDUFB1         1325 cgtgtgtaTG  TCGTGTTCGC  AAAGGTTTCa  tagctccatg  cattcacttg
                             **  **********  *********

alignment position   1551......  1561......  1571......  1581......  1591......
P303789         1002 ----------  ----------  ----------  ----------  ----------
mNDUFB1         1360 --TTCCATAT  CTTAGGTGAC  ACTTTGTAAT  AATT-ATAAA  GAACTGTGGG
rNDUFB1         1375 ttTTCCTTGG  CTTAGGTGAT  ACTTTGTAAT  ACTTaATAAG  GAACTGTGGG
                       ********  **********  **********  ****  *****  **********
                       ********  **********  **********  ****  *****  **********
                                                               *****  **********
                                                               *****  **********

alignment position   1601......  1611......  1621......  1631......  1641......
P303789         1002 ----------  ----------  ----------  ----------  ----------
mNDUFB1         1407 GTCCAGTCCA  ACACAAGGTA  TTatCTCTTA  AAAAGTCTAC  ATGTgcctgc
rNDUFB1         1425 GTCCAGTCGG  ACACAAGGTA  TTgcCTCTTA  AAAAGTCTAC  ATGT------
                     **********  **********  **    ******  **********  ****
                     **********  **********  **    ******  **********  ****
                     **********  **********  **
                     **********  **********  **

alignment position   1651......  1661......  1671......  1681......  1691......
P303789         1002 ----------  ----------  ----------  ----------  ----------
```

```
mNDUFB1      1457 agtggtggtg gcgcatgcct ttaatcccag cacttggaaa gcagaggcag
rNDUFB1      1469 ---------- ---------- ---------- ---------- ----------

alignment position 1701...... 1711...... 1721...... 1731...... 1741......
P303789      1002 ---------- ---------- ---------- ---------- ----------
mNDUFB1      1507 gtggatttct gagttcaagg ccaggctggt ctacagagtg agttccaggg
rNDUFB1      1469 ---------- ---------- ---------- ---------- ----------

alignment position 1751...... 1761...... 1771...... 1781...... 1791......
P303789      1002 ---------- ---------- ---------- ---------- ----------
mNDUFB1      1557 cagccagggc tttaaagaga acccctgtct tgaaaaacaa aacaacaaaa
rNDUFB1      1469 ---------- ---------- ---------- ---------- ----------

alignment position 1801...... 1811...... 1821...... 1831...... 1841......
P303789      1002 ---------- ---------- ---------- ---------- ----------
mNDUFB1      1607 aaagtctaca tgtttccagc atgaatccag atagaagact cagcaaaata
rNDUFB1      1469 ---------- ---------- ---------- ---------- ----------

alignment position 1851...... 1861...... 1871...... 1881...... 1891......
P303789      1002 ---------- ---------- ---------- ---------- ----------
mNDUFB1      1657 agactcacag cagtgtctgt tattcttgaa aagggaaaga aactttaatc
rNDUFB1      1469 ---------- ---------- ---------- ---------- ----------

alignment position 1901...... 1911...... 1921...... 1931...... 1941......
P303789      1002 ---------- ---------- ---------- ---------- ----------
mNDUFB1      1707 ccagcacttg gaaagcagag gcaggtggta tttaagggaa ataccttgtg
rNDUFB1      1469 ---------- ---------- ---------- ---------- ----------

alignment position 1951...... 1961...... 1971...... 1981...... 1991......
P303789      1002 ---------- ---------- ---------- ---------- ----------
mNDUFB1      1757 ttagagaaaa atattaagaa atatgtggga tgtggggtta gttgtttttt
rNDUFB1      1469 ---------- ---------- ---------- ---------- ----------

alignment position 2001...... 2011...... 2021...... 2031...... 2041......
P303789      1002 ---------- ---------- ---------- ---------- ----------
mNDUFB1      1807 gtttttaaaa agtctcacac acacacacat ggagagagag agagagagag
rNDUFB1      1469 ---------- ---------- ---------- ---------- ----------

alignment position 2051...... 2061...... 2071...... 2081...... 2091......
P303789      1002 ---------- ---------- ---------- ---------- ----------
mNDUFB1      1857 agagagagag agagagagag agagagagag gctctgcaaa gaagaaaaga
rNDUFB1      1469 ---------- ---------- ---------- ---------- ----------

alignment position 2101...... 2111...... 2121...... 2131...... 2141......
P303789      1002 ---------- ---------- ---------- ---------- ----------
mNDUFB1      1907 gaggaggagt tatttaactg tttacctccg tacacaggag agagagtaga
rNDUFB1      1469 ---------- ---------- ---------- ---------- ----------

alignment position 2151...... 2161...... 2171...... 2181...... 2191
P303789      1002 ---------- ---------- ---------- ---------- ----
mNDUFB1      1957 acaaaagagc ttaagcctga tcaggataca gattgtctgg ggac
rNDUFB1      1469 ---------- ---------- ---------- ---------- ----
```

Pairwise similarities:

For each pairwise alignment, the similarity (relative to the maximum similarity) and the number of identical nucleic acids (in % of shorter sequence) is given. Maximum values are underlined.

	mNDUFB1 (2000 bp)	rNDUFB1 (1468 bp)
**P303789 (1001 bp)**	0.123<br>42 %	0.134<br>44 %
**mNDUFB1 (2000 bp)**		1.000<br>80 %

Please note that the **similarity value 1.000** marks only the two **most similar** sequences, it does not necessarily mean that these sequences are identical.

**Extract alignment region**	
**Positions in alignment**	from: to:
**Output file**	dialign.seq

Extract aligned sequences

For comments, questions or bug reports, please contact support@genomatix.de

© Genomatix Software GmbH 1998-2005 - All rights reserved.

License Agreement

GEMS Launcher 4.1.1

Genomatix-Logo Logout Personal Messages GEMS-Logo
Logout Personal Messages

Genomatix_MainGEMSElDoradoGene2PromoterBiblioSphere
Frequently_asked_questionsYour_ResultsYour_SequencesYour_ProtocolHelp

GEMS Launcher Task: *DiAlign TF*: Multiple alignment plus TF sites
working on promoters.seq (3 seq.)

[Alignment] [Pairwise_similarities]

DiAlign professional TF Release 3.1 December 2004 **Tue Jun 7 14:28:13 2005**

Solution parameters:

**Sequence file:**	promoters.seq (3 seq.)
**Type of sequences:**	nucleotide sequences
**Threshold T:**	0.00
**'*' signs below alignment denote:**	diagonal similarity (max. similarity is represented by 10 '*' signs)
**Alignment output:**	complete alignment is shown<br># nucleic acids per line: 50
**TF output:**	common TF matches located in aligned regions (common to 3 (100.0 %) sequences)
**Family matches:**	yes
**MatInspector library:**	Matrix Family Library Version 5.0 (February 2005)
**Selected groups** (core/matrix sim):	• ALL user_defined.lib (0.75/Optimized)<br>• ALL vertebrates.lib (0.75/Optimized)

Aligned Sequences:

No.	Sequence Name	Sequence Description	Length
1	P914132	sym=NDUFB2|loc=Loc4708|taxid=9606|spec=Homo sapiens|chr=7|ctg=NT_007914|str=(+)|start=987533|end=988533|len=1001| tss=501| comm=NADH dehydrogenase (ubiquinone) 1 beta subcomplex, 2, 8kDa; (NM_004546/501/bronze;)	1001 bp
2	P914133	sym=Ndufb2|loc=Loc68198|taxid=10090|spec=Mus musculus|chr=6|ctg=NT_039341|str=(+)|start=8838279|end=8839314|len=1036| tss=501,503,510,536| comm=NADH dehydrogenase (ubiquinone) 1 beta subcomplex, 2; (AK028334/501/gold;AK007495/536/gold;AK010642/503/gold; NM_026612/510/bronze;)	1036 bp
3	P914135	sym=na|loc=Loc362344|taxid=10116|spec=Rattus norvegicus|chr=4|ctg=NW_047690|str=(+)|start=309516|end=310516| len=1001|tss=501| comm=similar to NADH dehydrogenase (ubiquinone) 1 beta subcomplex, 2; (XM_342664/532/bronze;)	1001 bp

Alignment (DiAlign format):

Please note that only **upper-case** letters are considered to be **aligned**. For more information, please have a look at the user guide.

V$CREB V$E4FF V$ETSF V$EGRF V$WHZF V$SF1F V$STAF V$P53F

```
alignment
position          1 . . . . . . . . .   1 1 . . . . . . . .   2 1 . . . . . . . .   3 1 . . . . . . . .   4 1 . . . . . . . .
P914132        1  c c t g a g c c t a   t g c c a a c c c a   g c c t t a a a g c   c a g t g g a t c g   a a t c a a g c c a
P914133        1  a C C T C G A A T T   G G C T A A G G C T   C G A G G C G T T A   G G A A C T T T c c   c c C A T G T T C C
P914135        1  - C C C C G A A T G   G T C T A A G G A T   T A A G G C G T T A   G A A A C C T T t a   c t C A T A T T C C
```

```
                 * * * * * * * * *  * * * * * * * * * *  * * * * * * * * * *  * * * * * * *          * * * * * * * *
                 * * * * * * * * *  * * * * * * * * * *  * * * * * * * * * *  * * * * * * *          * * * * * * * *

alignment
position         5 1 . . . . . . .  6 1 . . . . . . . .  7 1 . . . . . . . .  8 1 . . . . . . . .  9 1 . . . . . . . .

P914132      51  t t t g t g g c a t  t a t c t - - - - -  - - - - - - - - - -  - - - - - - - - - -  - - - - - - - - - -
P914133      51  T G G C C G A C T T  A T T C C G A G t g  t G C C G G G T T G  G G T T C C T C A G  G T G A T T C c c g
P914135      50  T G G C C G C C T T  A T T C C A A G g a  g G C A G G G T T G  G G T T C C T C A G  G T G A C T C t g a
                 * * * * * * * * * *  * * * * * * * *        * * * * * * * * *  * * * * * * * * * *  * * * * * * *
                 * * * * * * * * * *  * * * * * * * *        * * * * * * * * *  * * * * * * * * * *  * * * * * * *

alignment
position         1 0 1 . . . . . .  1 1 1 . . . . . .  1 2 1 . . . . . .  1 3 1 . . . . . .  1 4 1 . . . . . .

P914132      66  - - - - - - - - - -  - - - - - - - - - -  - - - - - - - - - -  - - - - - - - - - -  - - - - - - - - - -
P914133     101  T G C C A C C A G T  C C A G G T T C T G  A A A T T T A A C A  C C c t g g c t g a  t t a g t c a - - -
P914135     100  T G C C A G C G G T  C C A C G T T C T G  A G A T T T A A C A  C C t g g c t g c t  g g t g a a t c a t
                 * * * * * * * * * *  * * * * * * * * * *  * * * * * * * * * *  * *
                 * * * * * * * * * *  * * * * * * * * * *  * * * * * * * * * *  * *

alignment
position         1 5 1 . . . . . .  1 6 1 . . . . . .  1 7 1 . . . . . .  1 8 1 . . . . . .  1 9 1 . . . . . .

P914132      66  - - - - - - - - - -  - - - - - - - - - -  - - - - - - - - - -  - - - - - - - - - -  - - - - - - - - - -
P914133     148  - - - - - T T C A G  A A C A A T G C T G  A A A C A A C G A t  g c t a t a a a a t  a a a c a t t g c g
P914135     150  c a g g t T T C A G  A A C A G T A C T G  G A A C A A C G A -  - - - - - - - - - -  - - - - - - - - - -
                           * * * * *  * * * * * * * * * *  * * * * * * * * *
                           * * * * *  * * * * * * * * * *  * * * * * * * * *

alignment
position         2 0 1 . . . . . .  2 1 1 . . . . . .  2 2 1 . . . . . .  2 3 1 . . . . . .  2 4 1 . . . . . .

P914132      66  - - - - - - - - - -  - - - - - - - - - -  - A T T G C T G C G  T A C G A A C T T A  - G C C T G G C A C
P914133     193  a T G T T A T A A A  A T A A A C A T T G  C G T G G T T C A G  A A A G G G C T T A  g G C C T T T A A C
P914135     179  - T G T T A T A A A  C T A A A C A T G G  C A T G G T T C A G  A A A G G A C T T A  - G C C T T T A A C
                   * * * * * * * * *  * * * * * * * * * *  * * * * * * * * * *  * * * * * * * * * *    * * * * * * * * *
                   * * * * * * * * *  * * * * * * * * * *  * * * * * * * * * *  * * * * * * * * * *    * * * * * * * * *
                   * * * * * * * * *  * * * * * * * * * *  * * * * * * * * * *  * * * * * * * * * *    * * * * * * * * *
                                                            * * * * * * * * *  * * * * * * * * * *    * * * * * * * * *

alignment
position         2 5 1 . . . . . .  2 6 1 . . . . . .  2 7 1 . . . . . .  2 8 1 . . . . . .  2 9 1 . . . . . .

P914132      94  A A T G C C t g t a  a t c c c a g c t a  c t c g g g a a g t  t g a g g c a g g a  g a a t c g c t t g
P914133     243  A A G G G C C C A C  A G T G G C A - - -  - - - - - - - - - -  - - - - - - - - - -  - - - - - - - - - -
P914135     227  A A G G G C C C A C  A G T G G C A - - -  - - - - - - - - - -  - - - - - - - - - -  - - - - - - - - - -
                 * * * * * * * * * *  * * * * * * *
                 * * * * * * * * * *  * * * * * * *
                 * * * * * * * * * *  * * * * * * *
                 * * * * * * * * * *  * * * * * * *

alignment
position         3 0 1 . . . . . .  3 1 1 . . . . . .  3 2 1 . . . . . .  3 3 1 . . . . . .  3 4 1 . . . . . .

P914132     144  a g c c t g g g a g  g c a g a g g t t g  c a g t g a g c c g  a g a t g g t g t c  a c t g c a c t c c
P914133     260  - - - - - - - - - -  - - - - - - - - - -  - - - - - - - - - -  - - - - - - - - - -  - - - - - - - - - -
P914135     244  - - - - - - - - - -  - - - - - - - - - -  - - - - - - - - - -  - - - - - - - - - -  - - - - - - - - - -

alignment
position         3 5 1 . . . . . .  3 6 1 . . . . . .  3 7 1 . . . . . .  3 8 1 . . . . . .  3 9 1 . . . . . .

P914132     194  a g c c t g g g t g  a c a g a g c a a g  g c c t t g t c t c  a a a a a g a a a a  a a a a a a a a a a
P914133     260  - - - - - - - - - -  - - - - - - - - - -  - - - - - - - - - -  - - - - - - - - - -  - - - - - - - - - -
P914135     244  - - - - - - - - - -  - - - - - - - - - -  - - - - - - - - - -  - - - - - - - - - -  - - - - - - - - - -

alignment
position         4 0 1 . . . . . .  4 1 1 . . . . . .  4 2 1 . . . . . .  4 3 1 . . . . . .  4 4 1 . . . . . .

P914132     244  a a a a g a a t t a  t a c a c c a c t c  a g t t t t a g g a  g g g g c A G A A C  A C G g g g a g a g
P914133     260  - - - - - - - - - -  - - - - - - - - - -  - - - - - - - - - -  - - - - - A G A A C  A t G C A T G A A A
P914135     244  - - - - - - - - - -  - - - - - - - - - -  - - - - - - - - - -  - - - - - A G A A C  A C G C A C G A A A
```

```
                                                *****  **********
                                                *****  *  *******
                                                *****  *
                                                *****  *

alignment position   451.......  461.......  471.......  481.......  491.......
P914132   294  ccctacagga  tcagaggggc  aaccaacaag  ctgtttcagA  AGGCATGGGG
P914133   275  GCACCGTCTA  TATGAGCC--  ----------  ---------A  AAGCAGGGAG
P914135   259  GCACCGTTCA  TATAAGCC--  ----------  ---------G  AAGCAa----
               **********  ********                        *  **********
               **********  ********                        *  *****

alignment position   501.......  511.......  521.......  531.......  541.......
P914132   344  CAGCtcagca  accataagtt  atgcgtgggc  tttgctctcg  ggagagaggt
P914133   304  CAGCagTAAG  GTCAG-----  ----------  ----------  ----------
P914135   284  ------TAAG  GTCAG-----  ----------  ----------  ----------
               ****  ****  *****
                     ****  *****
                     ****  *****

alignment position   551.......  561.......  571.......  581.......  591.......
P914132   394  ttcctgcagc  CGAAGGACTC  ACAGCTAATC  TGGGTCAGGA  GCGGCTGCCG
P914133   319  ----------  CCAAGAGCAC  CTTGCCAGGC  TGTGCCACGA  GGCAGCCAGG
P914135   293  ----------  CTAAGAGCAC  CTTGCCACGC  TGCGTCACGA  GACAGCCAGA
                           **********  **********  **********  **********
                           **********  ******* **  **********  **********
                           **********  ******* **  **********  **********

alignment position   601.......  611.......  621.......  631.......  641.......
P914132   444  GCTCCCCCac  tcctg-----  ----------  ----------  ----------
P914133   359  GCTCCTCCTG  AAGGACGAcC  TATCTGCCCG  TTAACCTGGA  GCCTCTAAGG
P914135   333  GCTCCTCCCA  AAGGACGAtC  TATCTGCCCG  TTAACCCGGA  GCCTCTAAGG
               **********  ******** *  **********  **********  **********
               **********  ******** *  **********  **********  **********
               **********  ******** *  **********  **********  **********
                                    *  **********  **********  **********
                                    *  **********  **********  **********

alignment position   651.......  661.......  671.......  681.......  691.......
P914132   459  ----------  ----------  ----------  ----------  ----------
P914133   409  TCCGCCAATg  gcttgaaCTT  GTCCCCTGTG  GCCTACCATC  AGCCCGGGTA
P914135   383  TCCGCCAATa  gtctgagCTT  GTCCGCTGTG  GCCTACCATC  AGCCCCGATA
               *********          ***  **********  **********  **********
               *********          ***  **********  **********  **********
               *********          ***  **********  **********  **********
               *********          ***  **********  **********  **********
               *********

alignment position   701.......  711.......  721.......  731.......  741.......
P914132   459  GAGATGA---  GCGACTCTGT  GACGTCACGG  Gaggcgcggg  cggagcgcCG
P914133   459  GAGACGCagt  GCGACGCAGT  GACGTCACGG  GCCGGGACT-  --------CG
P914135   433  GAGACGC---  GCGACGCAGT  GACGTCACGt  tCCAGGACT-  --------CG
               *******     **********  **********  ********            **
               *******     **********  *********     *******           **
               *******     **********  *********     *******           **
               *******     **********  *********     *******           **
               *******

alignment position   751.......  761.......  771.......  781.......  791.......
P914132   506  GGGAAGCGAA  GTAGGCAGGG  GCGAGGCGGC  Tggggaccgc  ggggcggacg
P914133   500  GGGAAGTGAC  GCATCAGGGG  TGGGAACTGC  agtctt----  ----------
P914135   471  GGGAAGTGAC  GCATCAGGGG  TGGGGACTGC  Tctccc----  ----------
```

```
                    ********** ********** ********** *
                    ********** ********** **********
                    ********** ********** **********
                    ********** ********** **********

alignment
position            801....... 811....... 821....... 831....... 841.......

P914132       556   ggaGCGAGTA TGTCCGCTCT GACTCGGCTG GCGTCTTTCG cTCGCGTTGG
P914133       536   ---GCTAGTA TGTCTGCGCT CACCCGTCTG GTACCTTTCG GcCGCGTCGG
P914135       507   ---GCTAGGA TGTCTGCGCT GGCCCGCCTG GCACCTTTCG GTCGCGTTGG
                       ******* ********** ********** ********** **********
                       ******* ********** ********** ********** **********
                       ******* ********** ********** ********** *  ********
                       ******* ********** ********** **********    ********
                       ******* ********** ********** **********    ********
                                                                   ********
                                                                   ********

alignment
position            851....... 861....... 871....... 881....... 891.......

P914132       606   AGGCCGCCTT TTCAGAAGCG GCTGCGCACG GactGCTGGA GATGGTGGAG
P914133       583   AGGCCGCCTG CTCCGGGGCT GCCGCGCGCG GGcgGCTGGA GACAGTGGAG
P914135       554   AGGCCGCCTG CTTCGGGGCT GCCGCGCGCG GGcaGCTGGA GACGGTGGAG
                    ********** ********** ********** **  ****** **********
                    ********** ********** ********** **  ****** **********
                    ********** ********** ********** **  ****** **********
                    ********** ********** ********** **  ****** **********
                    ********** ********** ********** *   ****** **********
                    ********** ********** ********** *   ****** **********
                    ********** ********** ********** *   ****** **********
                                                         ****** **********
                                                         ****** **********
                                                         ****** **********

alignment
position            901....... 911....... 921....... 931....... 941.......

P914132       656   TCCGTCAGTG AGTGTGGGGC TGGGgatgGG GGCCTCGCCG GCCTGTAGCG
P914133       633   TCCGTCAGTG AGTGTCGGGC GGGGgc--GA GGGTTCGGCC GGCTTGAACA
P914135       604   TCCGTCAGTG AGTGTCGGGT GGGGacc--A GGGCTCGGCC GGCTCGAACA
                    ********** ********** ****    ** ********** **********
                    ********** ********** ****     * ********** **********
                    ********** ********** ****     * ********** **********
                    ********** ********** ****     * ********** **********
                    ********** ********** ****
                    ********** ********** ****
                    ********** ********** ****
                    ********** *********
                    ********** *********
                    ********** *********

alignment
position            951....... 961....... 971....... 981....... 991.......

P914132       706   GACAGCGCGG gtccctgGGA CGCTCTCACC TTGACTGGGG CGCCTGAaga
P914133       681   GGCAGCGCTG CCATCC-GGA CGCTGTCACC TTGAAGCAGT CGCCCGGGAG
P914135       652   GGCAGCGCTG CCATCCc-GA CGCTGTCACC TTGAAGCAGT CGCCCGAGCG
                    ********** ******  *** ********** ********** **********
                    ********** ******   ** ********** ********** **********
                    ********** ******   ** ********** ********** **********
                    ********** ******   ** ********** ********** **********
                                        ** ********** ****

alignment
position            1001...... 1011...... 1021...... 1031...... 1041......

P914132       756   g--------- -----GCCGC GTCCCGAGGC ATGCTGGGAC CTGTAGTCC-
P914133       730   GGTGCGTGaa actccGCTGC CGCCCGAGGC ATGCTGGGTC TTGTAGTttt
P914135       701   AGTGCATGac actttGCTGC CGCCTGAGGC ATGCTGGGTC TTGTAGTCG-
                    ********         ***** ********** ********** *********
                    ********         ***** ********** ********** *********
                    ********         ***** ********** ********** *******
                    ********         ***** ********** ********** *******
```

```
                                            ***** ********** ********** *******
                                                  ********** ********** *******
                                                  ********** ********** *******

alignment
position        1051...... 1061...... 1071...... 1081...... 1091......

P914132     791 GCGTGtctcc tgaagccgcg actcgaggag ---------- ----------
P914133     780 GCCTGGACCG AGAC--TGGC CTTTTAGTCG GCTTGGTGGG CTAGAGGTga
P914135     750 GCCTGGACGG AGACtgTGGC TTTTTGGTCG GATTGGTGGG GTAGAGGTt-
                ********** ****  **** ********** ********** ********
                *****            **** ********** ********** ********

alignment
position        1101...... 1111...... 1121...... 1131...... 1141......

P914132     821 AGGGACCCCG CTGGCCGGAG GGAGAGGGag agacttcgct gggcagatgg
P914133     828 AGGGATGGCG CAGGGTTGCG GGAGAGAGGC GTTAACTAAG ACccgagtga
P914135     799 --GGATGGCA CAGGGTTAGG GGAGAGAGGC GTTAACTAGG ACttgagtgc
                ********** ********** ********** ********** **
                  ******** ********** ********** ********** **
                  ******** ********** ********** ********** **
                  ******** ********** ********** ********** **

alignment
position        1151...... 1161...... 1171...... 1181...... 1191......

P914132     871 gacaccgtgt gcagggccgt catggtagta ggggccgcct gccctcggga
P914133     878 tAAGAATCCA GTCCCGCACA GTCTCC---- ---------- ----------
P914135     847 aAAGAATCCA GCCGTGCACA GTCTCC---- ---------- ----------
                 ********* ********** ******
                 ********* ********** ******
                 ********* ********** ******

alignment
position        1201...... 1211...... 1221...... 1231...... 1241......

P914132     921 gacgcacgcg tgaatgcgga gCGGGCTGAG CCagtcggcg ccggcgc---
P914133     904 ---------- ---------- -CGGACTGAG CCTTCCAAGC AGCTGGCTAG
P914135     873 ---------- ---------- -GGGCCTGAA CCTTCCAAGT CGCTGGCTAG
                                       ********* ********** **********
                                       ********* ********** **********
                                       ********* ********** **********

alignment
position        1251...... 1261...... 1271...... 1281...... 1291......

P914132     968 ---------- ---------- ---------- ---------- --------GG
P914133     933 TGGAGGTCCC GAGCGCATGG AGACggGTGG AGATGGGCTC AGAGACAAGA
P914135     902 TGTAGGTCCG GAGCGCAAGG AGACcaGTGG AGATGGGCTC TGAGACCAGA
                ********** ********** ****  **** ********** **********
                ********** ********** ****  **** ********** **********
                ********** ********** ****  **** ********** **********

alignment
position        1301...... 1311...... 1321...... 1331...... 1341......

P914132     970 GCGGTCCAGG CGGAGGAAGC agcgtgcgcg gc-------- ----------
P914133     983 CGGGTCCAGG CAGGGcagac acggagacct tgagtcagta aggtagtata
P914135     952 GTGGGCCAGG CAGGGAATGC cttggaggac tgaggatttt tccatcagtc
                ********** **********
                ********** *****
                ********** *****

alignment
position        1351

P914132    1002 ----
P914133    1033 gtct
P914135    1002 ----
```

Pairwise similarities:

For each pairwise alignment, the similarity (relative to the maximum similarity) and the number of identical nucleic acids (in % of shorter sequence) is given. Maximum values are underlined.

	P914133 (1036 bp)	P914135 (1001 bp)
P914132 (1001 bp)	0.133<br>25 %	0.139<br>27 %
P914133 (1036 bp)		1.000<br>79 %

Please note that the **similarity value 1.000** marks only the two **most similar** sequences, it does not necessarily mean that these sequences are identical.

**Extract alignment region**	
**Positions in alignment**	from: to:
**Output file**	dialign.seq

Extract aligned sequences

For comments, questions or bug reports, please contact support@genomatix.de

© Genomatix Software GmbH 1998-2005 - All rights reserved. License Agreement GEMS Launcher 4.1.1

Genomatix-Logo Logout Personal Messages GEMS-Logo

Logout Personal Messages

Genomatix Main GEMS ElDorado Gene2Promoter BiblioSphere
Frequently asked questions Your Results Your Sequences Your Protocol Help

GEMS Launcher Task: *DiAlign TF*: Multiple alignment plus TF sites
working on promoters.seq (3 seq.)

[Alignment] [Pairwise similarities]

DiAlign professional TF Release 3.1 December 2004 **Tue Jun 7 14:48:40 2005**

Solution parameters:

**Sequence file:**	promoters.seq (3 seq.)
**Type of sequences:**	nucleotide sequences
**Threshold T:**	0.00
**'*' signs below alignment denote:**	diagonal similarity (max. similarity is represented by 10 '*' signs)
**Alignment output:**	complete alignment is shown<br># nucleic acids per line: 50
**TF output:**	common TF matches located in aligned regions (common to 3 (100.0 %) sequences)
**Family matches:**	yes
**MatInspector library:**	Matrix Family Library Version 5.0 (February 2005)
**Selected groups** (core/matrix sim):	• ALL user_defined.lib (0.75/Optimized)<br>• ALL vertebrates.lib (0.75/Optimized)

Aligned Sequences:

No.	Sequence Name	Sequence Description	Length
1	P876211	sym=NDUFB3|loc=Loc4709|taxid=9606|spec=Homo sapiens|chr=2| ctg=NT_005403|str=(+)|start=52145450|end=52146450|len=1001| tss=501| comm=NADH dehydrogenase (ubiquinone) 1 beta subcomplex, 3, 12kDa; (NM_002491/572/bronze;)	1001 bp
2	P876212	sym=Ndufb3|loc=Loc66495|taxid=10090|spec=Mus musculus|chr=1| ctg=NT_039170|str=(+)|start=36282125|end=36283164|len=1040| tss=501,506,518,540| comm=NADH dehydrogenase (ubiquinone) 1 beta subcomplex 3; (AK013608/761/gold;AK009008/783/gold;AK007930/749/gold; NM_025597/744/bronze;)	1040 bp
3	P876213	sym=na|loc=Loc301427|taxid=10116|spec=Rattus norvegicus| chr=9|ctg=NW_047816|str=(+)|start=681579|end=682579| len=1001|tss=501| comm=similar to NADH dehydrogenase (ubiquinone) 1 beta subcomplex 3; (XM_217400/645/bronze;)	1001 bp

Alignment (DiAlign format):

Please note that only **upper-case** letters are considered to be **aligned**. For more information, please have a look at the user guide.

V$HOXH V$TALE V$CMYB V$EGRF V$NF1F V$NEUR V$ECAT V$PCAT V$AREB V$ETSF U$NRF2 V$RORA
V$CREB V$HOXF

```
alignment position 1 . . . . . . . . .  1 1 . . . . . . . .  2 1 . . . . . . . .  3 1 . . . . . . . .  4 1 . . . . . . . .
P876211          1 c t t g c t c c c g  c c t c t c c t c c  c t c t c t g c t g  g c g c c a c c g c  a g c c c c c a c g
P876212          1 - - - - - - - - - -  - - - - - - - - - -  - - - - - - - - - -  - - - - - - - - - -  - - - - - - - - - -
P876213          1 c - - - - - - - - -  - - - - - - - - - -  - - - - - - - - - -  - - - - - - - - - -  - - - - - - - - - -
```

```
alignment position  51........ 61........ 71........ 81........ 91........
P876211         51  ccctaggtgg gcccccacgt cgccccacaa gGCTCGGCTT CCACCCGCCT
P876212          1  ---------- ---------- ---------- ---------- ----------
P876213          2  ---------- ---------- ---------- -GCTCATCCT CAAGCCACCT
                                                      ********* **********

alignment position  101....... 111....... 121....... 131....... 141.......
P876211        101  CGGCTGCCGC CTTctcctcC TCACGACCCT CCCAAGatgc tacctggttt
P876212          1  ---------- ---------- ---------- ---------- ----------
P876213         21  CAGCAGTCGC CTT------C TCACGACCCT CCCGAGcttg ctctcaggtc
                    ********** ***      * ********** ******

alignment position  151....... 161....... 171....... 181....... 191.......
P876211        151  acgagaGGTT CCGGGACCAC CAACACACCG GCTACCGCCT TTCCAgacCA
P876212          1  ---------- ---------- ---------- ---------- -------TCA
P876213         65  tgcag-GGTT TCGGATCCGC CAACATTCCT GCTGCCGCCA CCCCAccTCA
                          **** ********** ********** ********** *****  ***
                                                                       ***
                                                                        **

alignment position  201....... 211....... 221....... 231....... 241.......
P876211        201  CCCTCGCCAG CTCCCGTCTG TAGTTTCCGC GGCCGAAGGA GAAgaaaacC
P876212          4  CCTTCGTCAG CTCCCGACTA CGGCGTCCGA AGCCGGAGGA GAAAT----T
P876213        114  CCTTCGTCAG CTCCCGGCTA CCATGTCCGC AGCCGGAGAA GAAAA----C
                    ********** ********** ********** ********** *****    *
                    ********** ********** ********** ********** *****    *
                    ********** ********** *    ***** ********** ***
                       ******* ********** *    ***** ****

alignment position  251....... 261....... 271....... 281....... 291.......
P876211        251  CCTCTTCATC T--AGCACTT TGCct----- ---------- CCCCCAAAGA
P876212         50  CCTCTTCACC C--GGCACCG TGCGcccccc cccccgcaac CCCCCCAAGA
P876213        160  CCTCTTCACC CcgGGCACCG TGCGttgtcg cccttccta- CCCCCCAAGA
                    ********** *  ******* ****                  **********
                    ********** *                                **********
                                                                **********
                                                                **********
                                                                **********
                                                                **********
                                                                **********
                                                                **********

alignment position  301....... 311....... 321....... 331....... 341.......
P876211        284  CAGCACCGCC GCCTACCTGT CACCCTGCtg tCTCCGGCTT CCTACCGCCG
P876212         98  CAGCACCGCC GCCTACCTGT CACCCCGCAG CCTCAGGCTT CCTACCGCCA
P876213        209  CAGCACCGCC GCCTACCTGT CACCCCGCAG CCTCAGGCTT CCTACCGCCA
                    ********** ********** ********** ********** **********
                    ********** ********** ********** ********** **********
                    ********** ********** ********** ********** **********
                    ********** ********** ********** ********** **********
                    ********** ********** ********    ********* **********
                    ********** ********** ********    ********* **********
                    ********** ********** ********    ********* **********
                    ********** ********** ********    ********* *********
                                    ***** ********
                                    ***** ********

alignment position  351....... 361....... 371....... 381....... 391.......
P876211        334  ---------- --CCGCCTCC CGCAGCAGCC TGCCAACTGC AGCGCAACAA
P876212        148  ccgccaccgc caCCGCCTCC CGCAGCAGCC TGCCAACTGC AGCATAACAA
P876213        259  ---------- --CCGCCTCC CGCAGCAGCC TGCCAACTGC AGCATAACAA
                                ******** ********** ********** **********
                                ******** ********** ********** **********
                                ******** ********** ********** **********
                                ******** ********** ********** **********
                                ******** ********** ********** **********
                                ******** ********** ********** **********
```

```
                                        *** ********** ********** ***    *****
                                        *** ********** ********** ***
                                        *** ********** ********** ***
                                        *** ********** ***

alignment position  401.......  411.......  421.......  431.......  441.......
P876211        372  CCAACCCTCT  CATTGGGTGa  gaaCACGCTC  TCAACAACAG  CCTCCCTTAT
P876212        198  CCAGCCTTCT  CATTGAGTGG  GCCCACGCGC  TTAATAACAA  CCGCTCTCAT
P876213        297  CCAGCCTTCT  CATTGAGTGG  GTCCACGTGC  TTAATAACAA  CCGCTCTCAT
                    **********  **********  **********  **********  **********
                    **********  **********  **********  **********  **********
                    **********  *********       ******  **********  **********
                    **********  *********       ******  **********  **********
                    **********  *********       ******  **********  **********
                    *******                                    ***  **********
                    *******

alignment position  451.......  461.......  471.......  481.......  491.......
P876211        422  TGGCTGCAGT  ATGccggcag  ccccgccctg  tGTGCGGTGT  CTAAGGATTC
P876212        248  TGGCTGCGCT  ATGACTTTGG  CCCGCCTcTC  GGTGCTCCGC  CTAAAGATTC
P876213        347  TGGCTGCGCT  ATGGCTTTGG  CCCGCCTtTC  GGTGCACCTC  TTACAGATTC
                    **********  **********  *******  **  **********  **********
                    **********  **********  *******  **  **********  **********
                    **********  **********  *******       *********  **********
                    **********  **********  *******                       *****
                    **********  ***
                    **********  ***

alignment position  501.......  511.......  521.......  531.......  541.......
P876211        472  CGTCCCCAAG  CAAGGACAAC  Atattatctt  ccccgccctt  ctCTTTTCTA
P876212        298  CGTCCTGGAG  CAAGACAAAC  ACGGCTTTAT  CGTC------  --CTTCTCCT
P876213        397  CGTCCGGGAG  CAAGGAGAAC  AAGGCTTCAT  CGTC------  --CTCCTCCT
                    **********  **********  **********  ****          ********
                    **********  **********  **********  ****          ********
                    **********  ****   ***  *                         ********
                    **********  ****   ***  *

alignment position  551.......  561.......  571.......  581.......  591.......
P876211        522  CAGTATCTGT  TACCTCAGTC  AGATTGAGGA  TGCAcatata  ctgggAAAAT
P876212        340  CAATATCTTC  TACCTCAAGC  AGACTCCAGC  TGCAAAGTGT  GGGA-AATAA
P876213        439  CCGTGTCTCC  TACCGCAGTC  AAACTCCAGC  TGCAAAGAGT  AGAA-AACAA
                    **********  **********  **********  **********  ****  *****
                    **********  ****           *******  **********  ****  *****
                    **********  ****           *******  ****              *****

alignment position  601.......  611.......  621.......  631.......  641.......
P876211        572  AATCGACTTC  CAGCGTCCCT  CATGTAGTCT  CTACctgatt  caggaatctc
P876212        389  TCTCTACTTC  CAGGCTCCTT  CATGTCATCT  CTACGTTGTC  TGGGAGCCTT
P876213        488  TATCGACTTC  CAGGCTTTTT  CATGTCATCT  CTGTCTTGTC  TGGGAGCCTT
                    **********  **********  **********  **********  **********
                    **********  **********  **********  **********  **********
                    **********  ***         **  **********  **********  **********
                      ********  ***         **  **********  ****
                                            **  **********  **

alignment position  651.......  661.......  671.......  681.......  691.......
P876211        622  taacCCAGTA  TCGGAACGTT  AAGCGGCTTC  TCCGCTTTCC  TGCCGTgAGA
P876212        439  GCG-CCAGTA  CCGGAACGTT  TACCATCTCC  CCCATTTTCC  TGTCGTAAGA
P876213        538  GCG-CTAGTC  CCGGAACGTT  TACTATCTCC  TCCACTTTCC  TGTCGTAAGA
                    ***  ******  **********  **********  **********  **********
                    ***  ******  **********  **********  **********  **********
                    ***  *****   **********  **********  **********  **********
                         *****   **********  **********  **********  **********
                           ***   **********  **********  **********  ****    ***
                                                                             ***
                                                                             ***
                                                                             ***
                                                                             ***

alignment position  701.......  711.......  721.......  731.......  741.......
```

```
P876211         672 AACTCGAGGT GACAACCTCC GTTTCCGGTT GGCTCCGgtt gcagag----
P876212         488 AACTAGAGGA AACAACGTCC GTTTCCGGTT GTCTCCGGCT GTAAGGTTGA
P876213         587 AACTAGAGGA AACAACGTCC GTTTCCGGTT GGCTCCaGCT GGAAGGTTGA
                    ********** ********** ********** ********** **********
                    ********** ********** ********** ********** **********
                    ********** ********** ********** ********** **********
                    ********** ********** ********** ******
                    ********** ********** ********** ******
                    ********** ********** ********** ******
                    ********** ********** ********** ******
                    ********** ********** ********** ******
                    ********** ********** ********** ******

alignment position  751....... 761....... 771....... 781....... 791.......
P876211         718 ---TTGAGTG TCCTGAGAGG TCAGATTGCT GTCAGGTAAA TTAGGGAGTT
P876212         538 ATATTGAGTG TCCCGGGAGG TCAGATTGCT GTCAGGTAAT TTAGGGAGCT
P876213         637 ATATTGAGTG TCCTGGGAGG TCACATTGCT GTCAGGTAAT TTAGGGAGTT
                    ********** ********** ********** ********** **********
                    ********** ********** ********** ********** **********
                    ********** ********** ********** ********** **********
                       ******* ********** ********** ********** **********
                       ******* ********** ********** ********** **********
                       ******* ********** ********** ********** **********
                       ******* ********** *** ****** *********  **********
                                              ****** *********
                                                 *** *********

alignment position  801....... 811....... 821....... 831....... 841.......
P876211         765 GGTGGGAGGC CCGTTGATTA GGATTAAgag ctagactctA TTCTGGGGAG
P876212         588 GGTGGGAGAC TTCTGGATTA CGCATAAtct aaactttt-- ATCCAGGGAA
P876213         687 GATGGGAGAC TTCTGGAta- ---------- ---------A ATCTGGGAAA
                    ********** ********** *******             * **********
                    ********** *******                          **********
                    ********** *******                           *********
                    ********** *******
                    ********** *******
                    ********
                    ********

alignment position  851....... 861....... 871....... 881....... 891.......
P876211         815 TGCAGAAAAG GAGAGActaa atggagaagg atgggaggct gGGAGGGAGA
P876212         635 TGCAGAAGTG GAGAGGGCTG AAGGGAtccg ataAGACC-- -GGGAGAAGA
P876213         717 TGCAGAAGTG GAGAGTGTTG AGAGGAcgag ctcAGACC-- -GGGAGGAGA
                    ********** ********** ******        *****    *********
                    ********** ********** ******        *****    *********
                    ********** *****                    *****    *********

alignment position  901....... 911....... 921....... 931....... 941.......
P876211         865 TCAAAGAACT AGCAGGCGAG AACTAATGaa gtcCTAGTTT AGAGTTGGGG
P876212         682 TCCGGGATCT AGCTTGGGAG CGCCGCtg-- ---CAAGGTT CTTGCTGGGC
P876213         764 TCCGGGACCT AGCTAGGGAG CCCTGCTGcc tagtacagct ggacgaggg-
                    ********** ********** ********      ******* **********
                    ********** ********** ******
                    ********** ********** ******

alignment position  951....... 961....... 971....... 981....... 991.......
P876211         915 TAGGAAGAGG TGGTTGGAGG ATTCAGTGAT CATTACTGTA GTTGTTAAAT
P876212         727 TGTGTTGAGG CGGATGAAGG ATgtctTGAG AATTACGATG GTTGTTAAAT
P876213         813 ------GAGG CGGATGAAGG ATACTGTGAG AATTACTGTG GTGGTTAAAT
                    ********** ********** ********** ********** **********
                          **** ********** ********** ********** **********
                                   ****** **    **** ********** **********
                                                **** ********** **********
                                                **** ********** **********

alignment position  1001...... 1011...... 1021...... 1031...... 1041......
P876211         965 CCA---GGTG CTTGTTCCCG CTTCTGCCAC ACGCAAaatt ----------
P876212         777 CCCAGAGATG TTTTAAACCG CCGCTACCAG ATGCAATGTA CTCCTCGCCT
P876213         857 CCCAGAGATG TTTTAACACG CCGCTGCCAG ATGCAATTTA CTCCTCGCCT
```

```
                         ********** ********** ********** ********** **********
                         ********** ******  ** ********** ********** **********
                         ********** ******  ** ********** ********** **********
                         ***              ** ********** ********** **********
                         **

alignment position       1051...... 1061...... 1071...... 1081...... 1091......
P876211           1002   ---------- ---------- ---------- ---------- ----------
P876212            827   CCTGGCTTTT TGGGTTGTAT TTGAATAGGG TAAAGAACAG CTCatacttt
P876213            907   CCTGGCTTTT GGGGTTGTGT TTGAATAGGG TAAAGAAGAA CTCtgtcccg
                         ********** ********** ********** ********** ***
                         ********** ********** ********** ********** ***
                         ********
                         ********

alignment position       1101...... 1111...... 1121...... 1131...... 1141......
P876211           1002   ---------- ---------- ---------- ---------- ----------
P876212            877   tAAATACTTA AAACTAGAAG AAATAATTGC AACGGTTGct tcacaagctt
P876213            957   aAAATACTTA AAACTAGAAG AAATAATTGC AACGGTTGtt tcaca-----
                          ********* ********** ********** ********
                          ********* ********** ********** ********
                          ********* ********** ********** ********
                          ********* ********** ********** ********

alignment position       1151...... 1161...... 1171...... 1181...... 1191......
P876211           1002   ---------- ---------- ---------- ---------- ----------
P876212            927   gtattttaag tacggtttcc caaatataag tgtttacgac acagtgctta
P876213           1002   ---------- ---------- ---------- ---------- ----------

alignment position       1201...... 1211...... 1221...... 1231...... 1241......
P876211           1002   ---------- ---------- ---------- ---------- ----------
P876212            977   agtgaaaaag agcaagtcac aaaatcaaga ctactgcata atcctaggat
P876213           1002   ---------- ---------- ---------- ---------- ----------

alignment position       1251...... 1261
P876211           1002   ---------- ----
P876212           1027   taaggattgg atac
P876213           1002   ---------- ----
```

Pairwise similarities:

For each pairwise alignment, the similarity (relative to the maximum similarity) and the number of identical nucleic acids (in % of shorter sequence) is given. Maximum values are underlined.

	P876212 (1040 bp)	P876213 (1001 bp)
P876211 (1001 bp)	0.364<br>48 %	0.419<br>52 %
P876212 (1040 bp)		1.000<br>73 %

Please note that the **similarity value 1.000** marks only the two **most similar** sequences, it does not necessarily mean that these sequences are identical.

**Extract alignment region**	
**Positions in alignment**	from: to:

Output file	dialign.seq

Extract aligned sequences

For comments, questions or bug reports, please contact support@genomatix.de

© Genomatix Software GmbH 1998-2005
- All rights reserved.

License Agreement

GEMS Launcher 4.1.1

Genomatix-Logo | Logout Logout | Personal Personal | Messages Messages | GEMS-Logo

Genomatix_MainGEMSElDoradoGene2PromoterBiblioSphere
Frequently_asked_questionsYour_ResultsYour_SequencesYour_ProtocolHelp

GEMS Launcher Task: *DiAlign TF*: Multiple alignment plus TF sites
working on promoters.seq (3 seq.)

[Alignment] [Pairwise_similarities]

DiAlign professional TF Release 3.1 December 2004 **Tue Jun 7 15:09:12 2005**

Solution parameters:

**Sequence file:**	promoters.seq (3 seq.)
**Type of sequences:**	nucleotide sequences
**Threshold T:**	0.00
**'*' signs below alignment denote:**	diagonal similarity (max. similarity is represented by 10 '*' signs)
**Alignment output:**	complete alignment is shown<br># nucleic acids per line: 50
**TF output:**	common TF matches located in aligned regions (common to 3 (100.0 %) sequences)
**Family matches:**	yes
**MatInspector library:**	Matrix Family Library Version 5.0 (February 2005)
**Selected groups** (core/matrix sim):	• ALL user_defined.lib (0.75/Optimized)<br>• ALL vertebrates.lib (0.75/Optimized)

Aligned Sequences:

No.	Sequence Name	Sequence Description	Length
1	P914161	sym=NDUFB4|loc=Loc4710|taxid=9606|spec=Homo sapiens|chr=3|ctg=NT_005612|str=(+)|start=26809774|end=26810774|len=1001| tss=501| comm=NADH dehydrogenase (ubiquinone) 1 beta subcomplex, 4, 15kDa; (NM_004547/501/silver;)	1001 bp
2	P914163	sym=Ndufb4|loc=Loc68194|taxid=10090|spec=Mus musculus|chr=16|ctg=NT_096987|str=(-)|start=2776611|end=2777611| len=1001|tss=501| comm=NADH dehydrogenase (ubiquinone) 1 beta subcomplex 4; (AK009807/571/gold;NM_026610/571/bronze;)	1001 bp
3	P914164	sym=na|loc=Loc288088|taxid=10116|spec=Rattus norvegicus|chr=11|ctg=NW_047356|str=(+)|start=18247379|end=18248379| len=1001|tss=501| comm=similar to NADH:ubiquinone oxidoreductase B15 subunit; (XM_213619/569/silver;)	1001 bp

Alignment (DiAlign format):

Please note that only **upper-case** letters are considered to be **aligned**. For more information, please have a look at the user guide.

V$IRFF V$NRF1 U$NRF1 V$ETSF U$NRF2 V$EREF U$EREF V$RORA V$TBPF

```
alignment position 1.........  11........  21........  31........  41........
P914161         1 ttaatgttgt atgaatctag catggtaaag tacagaaaag cttattctct
P914163         1 a--------- ---------- ---------- ---------- ----------
P914164         1 tg-------- ---------- ---------- ---------- ----------

alignment position 51........  61........  71........  81........  91........
```

```
P914161      51 tcttcttggg tagggaattc tgtTTTGCAA AGCACTTTgt gaTCTATTTG
P914163       2 ---------- ---------- --------AA AGCAATTTCA T-TGTTTTCA
P914164       3 ---------- ---------- ---TTTGGAA AGCAATTTCA T-TGTTTTCG
                                         ******* ********** * ********
                                              ** ********** * ********
                                              ** ********** * ********
                                              ** ********

alignment position 101....... 111....... 121....... 131....... 141.......
P914161     101 TTAGCTTATT TAAtcCTCAC AACAACTTTG CTAGGTGGAC AGgagtcatt
P914163      23 ATGTCTCATT TAA--TTCC- AACAACCTTG CTAGGAGTGC AGAGTCAAGG
P914164      29 TTGTCTCATT TAA--CTCCC AACAACTTTG CTAGGAGTGC GGGGTCAAAG
                ********** ***  ***** ********** ********** **********
                ********** ***  ***** ********** ********** **********
                ********** ***  ****  ********** ********** **********
                                ****  ********** ********** **
                                ****  ********** *****

alignment position 151....... 161....... 171....... 181....... 191.......
P914161     151 a----TCTCC AGGATAAGGA AAAGGAAAct ca-GAGACTT TAAGTGACTT
P914163      70 TCATTTCTCC AGGGTAAGAA AAAGGAAATT TGGGAGACAC TAATGACCTT
P914164      77 TCATTTCTCC AGGATAAGAA AAAGGAAATT TGGGAGACAT TAATGACCTT
                ********** ********** ********** ********** **********
                ********** ********** ********** ********** **********
                ********** ********** ********** ********** **********
                     ***** ********** ********** ********** **********
                     ***** ********** ********      ******* **********
                     ***** ********** ********
                           ********** ********

alignment position 201....... 211....... 221....... 231....... 241.......
P914161     196 AT------CC AAGGTGACAC AGcacggtta gACCCACAAT GAAAACCCGG
P914163     120 gactaatgAC AAGGTCACCC AGGTTGGAAG AACCCACCCT GGACTGTAGG
P914164     127 CT------AC AAGGTCACCC ACTTTGGAAG AACGCACCCT GAACTGTAGA
                **      ** ********** ********** ********** **********
                        ** ********** ********** ********** ********
                        ** ********** ********** ********** ********
                        ** ********** *           ********* ********

alignment position 251....... 261....... 271....... 281....... 291.......
P914161     240 ACTTCTGACT CCACAGCCAg tcccacaca- ---------- ------GCTC
P914163     170 ACTTCAGGAG TCACAGACAA CCCAGTTTCC CAGTCTCcTC TAGTCTACTC
P914164     171 CCTTCAGGAG CCACAGACAA CCCAGTTTCC CAGTCTCgTC TAGTCTGCTC
                ********** ********** ********** *******  ** **********
                 ********* ********** ********** *******  ** **********
                 ********* ********** ********** *******
                 ********* ********** ********** *******

alignment position 301....... 311....... 321....... 331....... 341.......
P914161     273 AACTTTACAT AATAAGccct ggttatgtca gtagagacaa ggCTGCTAGG
P914163     220 AACTTCAGGA AATCAGTT-- ---------- ---------- --CTGCAAAC
P914164     221 AACTTCAGGA AAcCAGTT-- ---------- ---------- --CTGCAAGC
                ********** ********                           ********
                ********** **  *****                          ********
                              *****                           ********

alignment position 351....... 361....... 371....... 381....... 391.......
P914161     323 AAGAAGCAGC GCGGTGACAG A-TGAGtagg tggggagcgg cggtggg---
P914163     246 AAGTAGTAGC ACAGAGAGAA AaGGAGTTTA CCACCAGACA AGAGGAGGCA
P914164     247 AAGCAGTAGC ACAGAAAGAA A-GGAGCTTA ACACCAAACA AAAGGAGACA
                ********** ********** *  ******* ********** **********
                ********** ********** *  ******* ********** **********
                ********** ********** *

alignment position 401....... 411....... 421....... 431....... 441.......
P914161     369 ---------G CAGGGCGCAC TGCGAGAAGA gcacttcgcg catgcgcata
P914163     296 AAacGAGAAG CAGTGCGCAC GCGCAGAATA CG-------- ----------
P914164     296 AAcaGAGAAA CAGTACGCGT GCGCAGACTA CG-------- ----------
```

```
                       **  ****** ********** ********** **
                       **  ****** ********** ********** **

alignment position 451....... 461....... 471....... 481....... 491.......
P914161        410 tcaccgagcg aaCGCGCCGT GTTCGG--GA AGGtaggaga aagaggcg--
P914163        328 ---------- --CGGGACGA GGGCGT--GA AGGAGGATTG GCAAAGGCTC
P914164        328 ---------- --CATGACGA GaggcttgGA GGGAGCCTGG GTAAAGGCTC
                                ******** ******  ** ********** **********
                                ******** *

alignment position 501....... 511....... 521....... 531....... 541.......
P914161        456 ---------- ---------- ---------- ---------- ---GAGCTCC
P914163        364 CACCCaGCCT CGAACCCCAC CCCTCTCCCG CCCCTTCCTG CCAGAGCGCC
P914164        366 CGCCCcGCCT CGAACTCTAC CTCTTTTCCA CCTCTTCCTG CCAGAGcact
                   ***** **** ********** ********** ********** **********
                         **** ********** ********** ********** ******

alignment position 551....... 561....... 571....... 581....... 591.......
P914161        463 TTGTtaacga catccccaac ctctgctaaC GCGCAGGCGC ACTTCCTGAA
P914163        414 TTGTGCG--- ---------- ---------C GCGCAGGCGC ACTTCCTGAG
P914164        416 cTGTGCG--- ---------- ---------C GCGCAGGCGC ACTTCCTGAG
                   *******                        * ********** **********
                    ******                        * ********** **********
                    ******                        * ********** **********
                    ******                        * ********** **********
                                                  * ********** **********
                                                  * ********** **********
                                                  * ********** **********
                                                  * ********** **********
                                                  * ********** **********

alignment position 601....... 611....... 621....... 631....... 641.......
P914161        513 GCTGAAGGTC AGcatagaca aag--GGCCT CAGAATCGCG CAGGCGCAAT
P914163        442 CCTGAAGGTC AGactcgAGA GGGCAGCCCT CATATCCGCG CAGGCGCAGA
P914164        444 TCTGAAGGTC AaactaaAGA GGGCAGCCCT TATATCCGCG CAGGCGCAAA
                   ********** **     *** ********** ********** **********
                   ********** **     *** ********** ********** **********
                   ********** **     *** ********** ********** ******** *
                   ********** *               ***** ********** ******** *
                   ********** *                 *** ********** ********
                   ********** *
                   ********** *
                   ********** *
                   ********** *

alignment position 651....... 661....... 671....... 681....... 691.......
P914161        561 TGTGCCCTGG ttcGCCAAGA TGTCGTTCCC AAAGTATAAG CCGTCGAGCC
P914163        492 TGTGCGCCGG GA-GTCAAGA TGTCGGGTTC CAAGTATAAG CCTGCGCCCC
P914164        494 TGTTCGCCGG GA-GCCAAGA TGTCGTTTTC CAAGTATAAG CCTGCACCCC
                   ********** ** ******* ********** ********** **********
                   ********** ** ******* ********** ********** **********
                   **********    ******* ********** ********** **********
                   **********    ******* *****  *** ********** **********
                                 ******* *****  *** ********** **********
                                                *** ********** **      **
                                                *** ********** **

alignment position 701....... 711....... 721....... 731....... 741.......
P914161        611 TGCGCACTCT GCCTGAGACC CTCGACCCAG CCGAATACAA CATATCTCCG
P914163        541 TGGCCACTCT CCCCAgTACC CTCGACCCTG CCGAGTATGA CGTGTCTCCG
P914164        543 TGGCCGCTCT CCCCAATACC CTCGACCCCG CCGAGTATGA CGTGTCTCCG
                   ********** ********** ********** ********** **********
                   ********** ********** ********** ********** **********
                   ********** ***** **** ********** ********** **********
                   ********** ***** **** ********** ********** **********
                   ********** ***** **** ********** ********** **********
                   **               **** ********** ********** **********
                                     *** ********** *******  * **********
                                     *** ********** *******  * **********
```

```
alignment position 751.......  761.......  771.......  781.......  791.......
P914161        661 GAAACCCGGC GGGCGCAAGC CGAGCGGTTG GCCATAAGAG CCCAGCTGAA
P914163        591 GAGACCaGAA GGGCGCAGGT CGAGCGCTTG AGCATAAGGG CCCGGCTTAA
P914164        593 GAGACCCGAA AGGCGCAGGT CGAGCGCATG AGCATAAGGG CCCGGCTTAA
                   ********** ********** ********** ********** **********
                   ********** ********** ********** ********** **********
                   ****** *** ********** ********** ********** **********
                   ****** *** ********** ********** ********** **********
                   ****** *** ********** ********** ********** **********
                   ******     ********** ********** ********** **********
                   ******     ********** *******      ******** **********
                   ******                             ******** **********
                                                                       **
                                                                       **

alignment position 801.......  811.......  821.......  831.......  841.......
P914161        711 ACGAGAGTAC CTGCTTCAGT ACAACGATCC CAACCGCCGA GGGCTCATCG
P914163        641 ACGGGAGTAT CTGCTTCAGT ACAACGACCC CAAACGCgtg tCGCACATCG
P914164        643 ACGGGAGTAT CTGCTTCAGT ACAACGACCC CAAACGCCAA ACGCACATCG
                   ********** ********** ********** ********** **********
                   ********** ********** ********** ********** **********
                   ********** ********** ********** *******     *********
                   ********** ********** ********** *******     *********
                   ********** ********** ********** *******     *********
                   ********** ********** ********** *******     *********
                   ********** ********** ********** *******
                   ********** ********** ********** *******
                   ********** ********** **

alignment position 851.......  861.......  871.......  881.......  891.......
P914161        761 TGAGTGTGGg gcctcCCAGG CGGGAATAGG GCCCGAGTCc tgggagagac
P914163        691 TGAGTGGGG- -----CCGGG CGGGACTGTG GGCCGGGGCA GCCGCATCTC
P914164        693 TGAGTGGGG- -----CCGGG CGGGACTGTG GGCCGGGGCA GCCGCATCTC
                   *********       ***** ********** ********** **********
                   *********       ***** ********** ********** **********
                   *********       ***** ********** ********** **********
                   *********       ***** ********** ********** **********
                   *********       ***** ********** *******
                   *********
                   *********

alignment position 901.......  911.......  921.......  931.......  941.......
P914161        811 cgagag---- ---------- ----AGA-CC ACGCAAAGGG TCGGCCACCG
P914163        735 GCGTGGGCAC ACGCGGGGCC GCCCAGC-TC CCTCCGTGTG CTGGACACCG
P914164        737 GCGCGGGCAC ACGCGGGGTC ACCCAGCtCC AATCCGTGTG CTGGACACCG
                   ********** ********** ******* ** ********** **********
                   ********** ********** *******      ******** **********
                   ********** ********** *******      ******** **********
                   ********** ********** *******      ******** **********

alignment position 951.......  961.......  971.......  981.......  991.......
P914161        842 CTCCCGATCA GTAtctcaga cgcaggtcct cttccaggcc tagccaactc
P914163        784 GCCCCGAGCC GCA------- ---------- ---------- ----------
P914164        787 GCCCCTAGCT GCA------- ---------- ---------- ----------
                   ********** ***
                   ********** ***
                   ********** ***
                   ********** ***

alignment position 1001......  1011......  1021......  1031......  1041......
P914161        892 actacccttt tacctttgcC AGAGCTTGGC CCTcgagagg acc-------
P914163        797 ---------- ---------C AGCCCTTGGC CCTTCAACTT CGGCCTTCGG
P914164        800 ---------- ---------C AGCCCTTGGT CCTTCAACTT TAGCCTTATG
                            * ********** ********** **********
                            * ********   ********** **********
                            * ********   ********** **********
                            * ********
```

```
alignment position 1051......  1061......  1071......  1081......  1091......
P914161       935 ----------  ----------  GGCTCTGGAC  TCAGACCTGG  CTGGGTTTGA
P914163       828 CTCAGTGGTC  AAAGGGATcg  gacggcttcc  cccttgg---  ----------
P914164       831 CTCAGTGGTC  AAAGGGATtg  GGCTCCGGCT  GCAGACCCAG  CTGGCCTTGC
                  **********  ********    **********  **********  **********
                  **********  ********    **********  **********  **********
                  **********  ********

alignment position 1101......  1111......  1121......  1131......  1141......
P914161       965 ATCCTGGCTt  gatcacttcc  tgcctgcgtg  accttaa---  ----------
P914163       865 ----------  ----------  ----TTTAGA  GAAACTTGTT  ccACTCTGCA
P914164       881 GTCCTGGCTa  gaccgttccc  ccccaTTTGGA GAAAGTTGTT  aaACTCTGCG
                  *********                     ******  **********    ********
                  *********                                           ********
                                                                      ********
                                                                      ********

alignment position 1151......  1161......  1171......  1181......  1191......
P914161      1002 ----------  ----------  ----------  ----------  ----------
P914163       891 TAGTCTCGGC  TTTCTTGTCT  GAAACATGGC  CAtaATATGG  TCCTTCATAT
P914164       931 TAGTCTCGGC  TTTCTTATCT  GAAACATGGC  CAtgATATTG  TCCTTCATAT
                  **********  **********  **********  **  ******  **********
                  **********  **********  **********  **  ******  **********
                  **********  **********  **********  **  ******  **********
                  **********  **********  **********  **

alignment position 1201......  1211......  1221......  1231......  1241......
P914161      1002 ----------  ----------  ----------  ----------  ----------
P914163       941 CTTTTGAGGC  AGATTAacac  atgactgact  aatctagcac  agtatcttcc
P914164       981 CTTCTGAAGC  AGATTAcacg  t---------  ----------  ----------
                  **********  ******
                  **********  ******
                  **********  ******

alignment position 1251......  1261
P914161      1002 ----------  -
P914163       991 tcgaaaaggg  c
P914164      1002 ----------  -
```

Pairwise similarities:

For each pairwise alignment, the similarity (relative to the maximum similarity) and the number of identical nucleic acids (in % of shorter sequence) is given. Maximum values are underlined.

	P914163 (1001 bp)	P914164 (1001 bp)
**P914161 (1001 bp)**	0.188<br>39 %	0.233<br>40 %
**P914163 (1001 bp)**		<u>1.000</u><br><u>81 %</u>

Please note that the **similarity value 1.000** marks only the two **most similar** sequences, it does not necessarily mean that these sequences are identical.

**Extract alignment region**	
**Positions in alignment**	from: to:
**Output file**	dialign.seq

Extract aligned sequences

For comments, questions or bug reports, please contact support@genomatix.de

© Genomatix Software GmbH 1998-2005
- All rights reserved.

License Agreement

GEMS Launcher 4.1.1

GEMS Launcher Task: *DiAlign TF*: Multiple alignment plus TF sites
working on promoters.seq (3 seq.)

[Alignment] [Pairwise similarities]

DiAlign professional TF Release 3.1 December 2004 **Tue Jun 7 15:22:31 2005**

Solution parameters:

**Sequence file:**	promoters.seq (3 seq.)
**Type of sequences:**	nucleotide sequences
**Threshold T:**	0.00
**'*' signs below alignment denote:**	diagonal similarity (max. similarity is represented by 10 '*' signs)
**Alignment output:**	complete alignment is shown<br># nucleic acids per line: 50
**TF output:**	common TF matches located in aligned regions (common to 3 (100.0 %) sequences)
**Family matches:**	yes
**MatInspector library:**	Matrix Family Library Version 5.0 (February 2005)
**Selected groups** (core/matrix sim):	• ALL user_defined.lib (0.75/Optimized)<br>• ALL vertebrates.lib (0.75/Optimized)

Aligned Sequences:

No.	Sequence Name	Sequence Description	Length
1	P862298	sym=NDUFB5lloc=Loc4711ltaxid=9606lspec=Homo sapienslchr=3l ctg=NT_005612lstr=(+)lstart=85817233lend=85818233llen=1001l tss=501l comm=NADH dehydrogenase (ubiquinone) 1 beta subcomplex, 5, 16kDa; (NM_002492/553/bronze;)	1001 bp
2	P862299	sym=Ndufb5lloc=Loc66046ltaxid=10090lspec=Mus musculuslchr=3l ctg=NT_039228lstr=(+)lstart=3823115lend=3824123llen=1009l tss=501,503,508,509l comm=NADH dehydrogenase (ubiquinone) 1 beta subcomplex, 5; (AK013223/553/gold;AK088061/546/gold;AK002279/553/gold; AK005521/553/gold;AK018562/548/gold;NM_025316/554/bronze;)	1009 bp
3	P862300	sym=nalloc=Loc294964ltaxid=10116lspec=Rattus norvegicusl chr=2lctg=NW_047625lstr=(+)lstart=5303108lend=5304108l len=1001ltss=501l comm=similar to NADH dehydrogenase (ubiquinone) 1 beta subcomplex, 5; NADH dehydrogenase (ubiquinone) 1 beta subcomplex 5; (XM_215544/501/bronze;)	1001 bp

Alignment (DiAlign format):

Please note that only **upper-case** letters are considered to be **aligned**. For more information, please have a look at the user guide.

V$CREB V$YY1F V$PCAT V$SMAD V$RARF V$RORA V$EREF U$EREF V$SF1F

```
alignment position 1 . . . . . . . . .   1 1 . . . . . . . .   2 1 . . . . . . . .   3 1 . . . . . . . .   4 1 . . . . . . . .
P862298          1 c a t  c t a c a a a   c c c c a t a t a c   t g t c t t g t c c   a c a g c a g g c a   g c a a c a a a a t
P862299          1 - - - - - - - - - -   - - - - - - - - - -   - - - - - - - - - -   - - - - - - - - - -   - - - - - - - - - -
P862300          1 t a g a g g a a g a   a c a g g g t g c g   c c a g g g t a g a   c g a - - - - - - -   - - - - - - - - - -
```

```
alignment position   51........ 61........ 71........ 81........ 91........
P862298           51 gaggaaggaa tagaaatgtc ggaggtgagc gacttCTCCA AGGTCACTCA
P862299            1 ---------- ---------- ---------- ---------- ----------
P862300           34 ---------- ---------- ---------- -----CTCCA AGGTCACGCT
                                                           ***** **********

alignment position   101....... 111....... 121....... 131....... 141.......
P862298          101 CTCctcacca aagtgggctc cggctctgcc atccaggcgg ccgttccatt
P862299            1 -----CCTCC GCGGTGGCCT GTctagagCT CTGTTGCATC CTGCAAGAAG
P862300           49 CTCtgCCTCC CTGGTGGTCT AT------CT CTATTGCACA CTGCAAGAAG
                     ***  ***** ********** **      ** ********** **********
                                                   ** ********** **********
                                                   ** ********** **********

alignment position   151....... 161....... 171....... 181....... 191.......
P862298          151 cttg------ ---------- -AGTTGTTCT Cggcatctcg gcatctcggc
P862299           46 CGCCCAAAGG CGGaGGCAGT CATTTCTTCT CT-------- ----------
P862300           93 CTCCCAAAGG CGGcGGCAGT CAGTTCTTCT CT-------- ----------
                     ********** *** ****** ********** **
                     ********** *** ****** ********** **
                     ********** *** ****** ********** **
                                    ****** ********** **
                                    ****** ********** **

alignment position   201....... 211....... 221....... 231....... 241.......
P862298          184 aaccaaacaa gatccaaagt tggagaggtg ggcaaaccta ctttataCAT
P862299           78 ---------- ---------- ---------- ---------- -------CAC
P862300          125 ---------- ---------- ---------- ---------- -------CAT
                                                                      ***
                                                                      ***
                                                                      ***
                                                                      ***
                                                                      ***
                                                                      ***

alignment position   251....... 261....... 271....... 281....... 291.......
P862298          234 CACTTACCCT GTGCAGGCAG GGACCTGAGG AGTTATTaac gatcgggaag
P862299           81 CACTTACCCC GAGTGAGCAG CGTCCTTGGG ATTTACTAGG GAACAAGCTG
P862300          128 CACTTACCCG GAGTGAGCAG GGGTCTTGGG ATTTACTAGG GAACAAGCAG
                     ********** ********** ********** ********** **********
                     ********** ********** ********** ********** **********
                     ********** *********      ****** ********** **********
                     ********** *********      ****** ********** **********
                     ********** *********      ****** *******
                     ********** *********      ****** *****
                     ********** *********

alignment position   301....... 311....... 321....... 331....... 341.......
P862298          284 atttcagggc ggatgaaaCT CTCCTACAAA GAAGGGCCAA ACCGGCCGCA
P862299          131 CCTTcagGAA CGCTGAGGCT CTTCTACAAA TACCCACTAG ACTGGTCGCA
P862300          178 CCTTtaaGAA CGCTGAGGCT CTTCTACAAA TACCCACTAG ACTGGTGGCA
                     ****   *** ********** ********** ********** **********
                     ****   *** ********** ********** ********** **********
                     ****   *** ********** ********** ********** **********
                     ****   *** ********** ********** ********** ****** ***
                            *** ********** ********** ********** ****** ***
                            *** ********** ********** ********** ******
                                        ** ********** ********** ******
                                        ** ********** ********** ******
                                        ** ********** ********** ******

alignment position   351....... 361....... 371....... 381....... 391.......
P862298          334 GCCATGTTtt cgcataactg gcaaaa---- ---------- ----------
P862299          181 GCCATGTTTC TAATGgAGAC CCACAGTGTC GGTGTCCACG AGGTCACTTC
P862300          228 GCCATGTTTC TAATGcAGAC ACACTGTGCC GGTGTCCACT AGGTCACTTC
```

```
                          *********  *****   ****  **********  **********  **********
                          *********  *****   ****  **********  **********  **********
                          *******            ****  **********  **********  **********
                          *******            ****  **********  **********  **********
                          *******

alignment position  401.......  411.......  421.......  431.......  441.......
P862298        360  ----------  ----------  ----------  ----------  ----------
P862299        231  CGCtggcggc  GTGAGGCAAG  TGAAGCAAAC  AGGAACAGGC  TTTGCCCTTA
P862300        278  CGC-------  GTGAGACGAA  TGAAGCAAAC  AAGAACAGGC  TTTGCCCTTA
                    ***         **********  **********  **********  **********
                    ***         **********  **********  **********  **********
                    ***                                     ****  **********
                    ***                                     ****  **********
                                                            ****  **********
                                                            ****  **********

alignment position  451.......  461.......  471.......  481.......  491.......
P862298        360  ----------  ----------  ----------  ----------  ----------
P862299        281  CTAAACATGG  CCGCTCCCAC  GGCTCTcgct  tcaatctccc  aa--------
P862300        321  CTAAACATGG  TCGCTCCCAC  GGCTCTttcc  ctcactccct  cccccctcc
                    *********   **********  ******
                    *********   **********  ******
                    *********   **********  ******
                    *********   **********  ******
                    *********   **********  ******
                    *********   **********  ******

alignment position  501.......  511.......  521.......  531.......  541.......
P862298        360  ----------  ----------  ----------  CGCGTTT-CC  GCCAACGATA
P862299        323  -GTCTGCCAT  AGAGTTTACA  GGGCTCTCTT  TGGGCgccCC  ACCTACAATG
P862300        371  cGTATGCTAT  AGAGTTTACG  CGGCTCTCTT  CGCGCAC-CC  ACCTACAATG
                     ********   **********  **********  *******  **  **********
                     ********   **********  **********  *****      *  **********
                                                                   *  **********
                                                                   *  **********
                                                                   *  **********

alignment position  551.......  561.......  571.......  581.......  591.......
P862298        379  GCGTCACTTC  CGGCGTCTCA  AGCCGGCggt  gaggagaata  gaaaact---
P862299        372  TCGTCACTTT  TGTCCGCCAG  CGCCTGCCTg  TGAAGCTCGC  AGGAACGGGC
P862300        420  TCGTCACTGT  TTTCCGCCAG  CGCCTGCCTa  TGAAGCTAGC  AAGCACCCGT
                    *********   **********  *********   **********  **********
                    *********   **********  *********   **********  **********
                    *********   **********  *********   **********  **********
                    *********   **********  *********
                    *********   **********  *********

alignment position  601.......  611.......  621.......  631.......  641.......
P862298        426  ---TGCCCTC  ACTTAAGATG  GCCACATCCT  GCCTTTGTCC  TCATTGGTAA
P862299        422  CGCTGCCCTC  ACTCAATATG  GCCTCCTCTA  GCGTTCGCCC  TCACTGGTGG
P862300        470  CGCTGCCCTC  ACTCAACATG  GCCttcccg-  GCATTCGCCT  TCACTGGTGG
                    *********   **********  **********  **********  **********
                    *********   **********  **********  **********  **********
                    *********   **********  ***         **********  **********
                       ******   **********              **********  **********
                       ******   **********              **********  **********
                       ******   **********              **********  **********
                                                        **********  ***

alignment position  651.......  661.......  671.......  681.......  691.......
P862298        473  ATTGGCTCTC  TCAGCTTCCG  TGACCCGCCT  CCCTtcttcc  tccTGCCCGT
P862299        472  ATTGTCCCTC  CCGGCTCCCT  TAGCCCTCCC  CACTTCCTTC  CG-TGGCCGG
P862300        519  ATTGTCCCTC  CCGGCTCCCG  TtgcctaCCC  TACTTCCTTC  CG-TGGCTGT
                    *********   **********  **********  ************   *******
                    *********   **********  *       ***  ************   *******
                    *********   ********            ***  ************   *******
                    *********   ********            ***  ************   *******
                    *********   ********                                *******
                    *********   ********                                *******
```

Pairwise similarities:

For each pairwise alignment, the similarity (relative to the maximum similarity) and the number of identical nucleic acids (in % of shorter sequence) is given. Maximum values are underlined.

	P862299 (1009 bp)	P862300 (1001 bp)
**P862298 (1001 bp)**	0.162<br>35 %	0.176<br>34 %
**P862299 (1009 bp)**		1.000<br>78 %

Please note that the **similarity value 1.000** marks only the two **most similar** sequences, it does not necessarily mean that these sequences are identical.

**Extract alignment region**	
**Positions in alignment**	from: to:
**Output file**	dialign.seq

Extract aligned sequences

For comments, questions or bug reports, please contact support@genomatix.de

© Genomatix Software GmbH 1998-2005 - All rights reserved. License Agreement GEMS Launcher 4.1.1

Genomatix-Logo Logout Personal Messages GEMS-Logo
Logout Personal Messages

Genomatix_MainGEMSElDoradoGene2PromoterBiblioSphere
Frequently_asked_questionsYour_ResultsYour_SequencesYour_ProtocolHelp

GEMS Launcher Task: *DiAlign TF*: Multiple alignment plus TF sites
working on C1-NDUFB6 (3 seq.)

[Alignment] [Pairwise_similarities]

DiAlign professional TF Release 3.1 December 2004 **Tue Jun 7 15:44:43 2005**

Solution parameters:

**Sequence file:**	C1-NDUFB6 (3 seq.)
**Type of sequences:**	nucleotide sequences
**Threshold T:**	0.00
**'*' signs below alignment denote:**	diagonal similarity (max. similarity is represented by 10 '*' signs)
**Alignment output:**	complete alignment is shown<br># nucleic acids per line: 50
**TF output:**	common TF matches located in aligned regions (common to 3 (100.0 %) sequences)
**Family matches:**	yes
**MatInspector library:**	Matrix Family Library Version 5.0 (February 2005)
**Selected groups** (core/matrix sim):	• ALL user_defined.lib (0.75/Optimized)<br>• ALL vertebrates.lib (0.75/Optimized)

Aligned Sequences:

No.	Sequence Name	Sequence Description	Len[illegible]
1	P855688	P855688 sym=NDUFB6|loc=Loc4712|taxid=9606|spec=Homo sapiens|chr=9|ctg=NT_008413|str=(-)|start=32562682|end=32563682|len=1001|tss=501|comm=NADH dehydrogenase (ubiquinone) 1 beta subcomplex, 6, 17kDa; (NM_002493/501/silver;NM_182739/501/silver;)	1[illegible]
2	mNDUFB6	mNDUFB6 m6_dna range=chr4:40417544-40418994 5'pad=0 3'pad=0 revComp=TRUE strand=? repeatMasking=none	1[illegible]
3	P855691	P855691 sym=na|loc=Loc297990|taxid=10116|spec=Rattus norvegicus|chr=5|ctg=NW_047711|str=(-)|start=31999505|end=32000505|len=1001|tss=501|comm=similar to NADH dehydrogenase; (XM_216378/501/bronze;)	1[illegible]

Alignment (DiAlign format):

Please note that only **upper-case** letters are considered to be **aligned**. For more information, please have a look at the user guide.

V$CREB V$CDEF V$RORA V$EVI1 V$HEN1 V$AP4R V$MYOD V$BEL1 V$EGRF V$BRNF

```
alignment
position      1.........  11........  21........  31........  41........
P855688     1 ggtgaaagca  tga-------  --ATTCATCT ATGCAATTTT GCTAGATATT
mNDUFB6     1 catacttggt  gtagttggtt  ttATTTACTT ATTTATTTTT TTAACTTATC
P855691     1 acca------  ----------  ---------- ---------- ----------
                                        ********  ********** **********
```

```
alignment
position        51........ 61........ 71........ 81........ 91........
P855688      42 AAgctact-- ---------- ---------- ---------- ----------
mNDUFB6      51 AAtagattgt agaagttact ccagttatga atagaaggct catttatggc
P855691       5 ---------- ---------- ---------- ---------- ----------
                **

alignment
position        101....... 111....... 121....... 131....... 141.......
P855688      50 ---------- ---------- ---------- ---------- ----------
mNDUFB6     101 agtagcatac caatgcttta cagtactata agctttccca ctaatcctct
P855691       5 ---------- ---------- ---------- ---------- ----------

alignment
position        151....... 161....... 171....... 181....... 191.......
P855688      50 ---------- ---------- ---------- ---------- ----------
mNDUFB6     151 attctgaaat ctgggaggtg atttttcaat gttttgctat tacatatcgt
P855691       5 ---------- ---------- ---------- ---------- ----------

alignment
position        201....... 211....... 221....... 231....... 241.......
P855688      50 ---------- ---------- ---------- ---------- ----------
mNDUFB6     201 gttgtgacga atattcgtgc acacccctac aaatttttac tgCCATACCT
P855691       5 ---------- ---------- ---------- ---------- --CCATACCT
                                                              ********
                                                              ********

alignment
position        251....... 261....... 271....... 281....... 291.......
P855688      50 ---------- ---------- ---------- ---------- ----------
mNDUFB6     251 TTGGGATGTC TTACAAAGTG GGcCGGCAGA ATCAAAGAGT GAGCGAGCCT
P855691      13 CTGGGATATC TTACAAAGTG GGaCGGCAGG ATCAAAGAGT GAGCCAGTCC
                ********** ********** ** ******* ********** **********
                ********** ********** ** ******* ********** **********

alignment
position        301....... 311....... 321....... 331....... 341.......
P855688      50 ---------- ---------- ---CTTTTCA CTATTCCTGT ACAGATGGAG
mNDUFB6     301 ATGGAAGCCT cCCAAATAAG GTGCTCTTTA TTTCTGCAGT ACAGAGaggg
P855691      63 ATGTAAGCCT -CCAAATAAG GTGCTCTTTA TTTCTGCAGT ACAGAGGAGG
                **********  ********* ********** ********** **********
                **********  ********* ********** ********** ******
                            ********* ********** ********** ******
                            ********* ********** ********** ******

alignment
position        351....... 361....... 371....... 381....... 391.......
P855688      77 GGACAGAtg- AGACAGCTGC CCAgggcact tcAGCAGTTA CTGGCATAGC
mNDUFB6     351 aagaAAACCG AGATAGGCGC TCAAGATTAC TtAGCGGGTT ACGAAAGAGG
P855691     112 GAAGAAACCC AGATAGCTGC TCAAAGTCAC Tt---GAGTT ACTAAAGGGC
                ********** ********** ********** * ******** **********

alignment
position        401....... 411....... 421....... 431....... 441.......
P855688     126 TGGGATTTGA ACTGGGATGT AActatttc atacacTCTC TAGGTCAttc
mNDUFB6     401 TGGGAGCCAA ATTCGGACGC AAGTG----- ------TCTC TCGGTCAAG-
P855691     159 TGGGAGCTGA ACTTGAATGC AATTG----- ------TCTC TAGGTCAAG-
                ********** ********** *****            **** *********
                                   ** *****            **** *********

alignment
position        451....... 461....... 471....... 481....... 491.......
P855688     176 cAAGGTGGGA gaggcaagca aataattttg aatctggcca gcatattcac
mNDUFB6     439 -AAGa---GA ATTCAAGGGG GCGAGGCAGA GGTTCGCT-- ----------
P855691     197 -AAGGTGGGA ATTCAAGCGG GCGGGGCAGA GGTTCCCT-- ----------
```

```
                *********  ********** ********** ********
                ***    **  ********** ********** ********
                       **  ********** ********** ********

alignment
position       501....... 511....... 521....... 531....... 541.......

P855688    226 tgAAACATTT ATGCTTATCC TTCCTCCTCC Cagca-TACA AGTTCTTCCT
mNDUFB6    473 --AAACCATC GTCCTTGTTC TTCCGGTCCC CTCCCCTACA AGTTCTTTCT
P855691    234 --AAACCCTT GTccttg--- ---CGGTCCC CTCCCCGACA AGTTCTTTCT
                 ******** ********** ********** ********** **********
                 ******** **            ******* ********** **********
                 ******** **            ******* ********** **********
                                                      **** **********

alignment
position       551....... 561....... 571....... 581....... 591.......

P855688    275 Tagcaaaacc tccagtcctc taatttagca tactttcgtc taaTCTGTCA
mNDUFB6    521 CTGAACCCTA GCC--CCAGG ACT------- ---------- ---TCTCACA
P855691    276 TTGAACTCTA GCCccCCGGG ACT------- ---------- ---TCTCACA
               ********** ***  ***** ***                     *******
               ********** ***  ***** ***                     *******
               ********** ***  ***** ***                     *******
               *

alignment
position       601....... 611....... 621....... 631....... 641.......

P855688    325 CTTTCCtcag gccccacaac tacgagtact ccttgtcccc acccggtcct
mNDUFB6    549 CTTTCCAACA CCGCCCACTT AgtTCTTAAC TCAGGGACCT TCCTGAGCTC
P855691    306 CTTTCTAACA CAGACCACTT AaaTCATAAC TAAAAGACCT TCCAGAGCTT
               ********** ********** *  ******* ********** **********
               ********** ********** *
               ********** ********** *

alignment
position       651....... 661....... 671....... 681....... 691.......

P855688    375 aatttggtcg ggtatagcgg aggtgggcgt agagagaact ggaggtgGAG
mNDUFB6    599 aTACTTAACC CAGGTTGTTA AAACCCAGGG TCCAAGA--- -------GAG
P855691    356 gTACTTAATC CGGGTTCTTA AAACCCGGGG TCAGAGA--- -------GAG
                ********* ********** ********** *******           ***
                ********* ********** ********** *******           ***
                ********* ********** ********** *******           ***

alignment
position       701....... 711....... 721....... 731....... 741.......

P855688    425 ACCTTTGTGC ATGCCCACAC ACTGCTTTCC TCGcgaggaa tctggggccc
mNDUFB6    639 GGCTTTGCGC ATGAGCAGAG CCTGCTTTCC GCGAAGGAGT CggcccgccC
P855691    396 GGCTTTGCGC ATGAGCAGAA CCTGCTTTCG GCGAAGGAAT CagcttgctC
               ********** ********** ********** ********** *        *
               ********** ********** ********** ********** *        *
               ********** ********** ********** ********** *        *
                ********* ********** ********** ***
                 ******** ********** *********

alignment
position       751....... 761....... 771....... 781....... 791.......

P855688    475 ttgCAGAGGC GGaaccagGA TGTGACGTAA TAACCGCGCG CGGCGC-TCG
mNDUFB6    689 C--CAGAGGC GGGGCT--GG TGTGGCGTCA TCACTCCGCG CCGCttcCAG
P855691    446 C--CAGAGGC TGGGCT--GC TGTGACGCCA TCACATCGCG CCGTTG-CAG
               *  ******* ******  ** ********** ********** ****** ***
               *  ******* ******  ** ********** ********** ***    ***
               *  ******* ******  ** ********** ********** ***    ***
                                                                 ***

alignment
position       801....... 811....... 821....... 831....... 841.......

P855688    524 GCGttcCCGC AAGGTCGCTT TGCAGAGCGG GAGCGCGC-T TAAGTaacta
mNDUFB6    735 GCG---CCTC AAGGTCGCTT TGCGGAGCGG GAGgggcagG TCAGTGACCG
P855691    491 GCG---CCTC AAGGTCGCTT TGCGGAGCGG GATCGCGC-G TAAGTGACCG
```

```
                   ***   **** ********** ********** ******** * **********
                   ***   **** ********** ********** ******** * *****
                   ***   **** ********** ********** ******** * *****
                   ***   **** ********** ********** ***
                         **** ********** ********** **
                         **** ********** ********** **
                         **** ********** ********** **
                         **** ********** ********** **

alignment
position           851....... 861....... 871....... 881....... 891.......

P855688      573   gtccgtagtt cgaGGGTGCG CCGTGTCctt ttgcgttggt accagCGGCG
mNDUFB6      782   GTCCtgt--- ---------- ---------- -----GTAGG CTCGTCGGCA
P855691      537   GCCCcaggtt c--GGGTGCG CGCTGTCact ctctgGTAAG CTCGCCGGCG
                   ****          ******* *******         ***** **********
                                                         ***** **********
                                                                    *****
                                                                    *****
                                                                    *****
                                                                    *****

alignment
position           901....... 911....... 921....... 931....... 941.......

P855688      623   ACATGACGGG GTACACTCCG GATGAGAAAC TGCGGCTGCA GCAGCTGCGA
mNDUFB6      804   ACATGTCAGG GTACACGCCG GATGAGAAGC TGCGGCTGCA GCAGCTGCGG
P855691      585   ACATGTCGGG GTACACGCCC GATGAGAAGC TGCGGCTGCA GCAGCTGCGG
                   ********** ********** ********** ********** **********
                   ********** ********** ********** ********** **********
                   ********** ********** ********** ********** **********
                   ********** ********** ********** ********** **********
                   ********** ********** ********** ********** **********
                   ********** *********  ********** ********** **********
                   ********** *********  ********** ********** **********
                                         ********** ********** **********
                                         ********** ********** **********
                                                         ***** **********

alignment
position           951....... 961....... 971....... 981....... 991.......

P855688      673   GAGCTGAGAA GGCGATGGCT GAAGGACCAG GAGCTGAGCC CTCGGGAGCC
mNDUFB6      854   GAGCTAAGGA GACGATGGCT GAAGGACCAG GAGCTGAGTC CCCGGGAGCC
P855691      635   GAGCTGAGAA GGCGATGGCT GAAGGACCAA GAGCTGAGCC CCCGGGAGCC
                   ********** ********** ********** ********** **********
                   ********** ********** ********** ********** **********
                   ********** ********** ********** ********** **********
                   ********** ********** ********** ********** **********
                   ********** ********** ********** ********** **********
                   ********** ********** ********** ******** * **********
                   ********** ********** ********** ******** * **********
                   ***** **** ********** *****               * **********
                   ***** **** ********** *****               * **********
                   *****                                     * **********

alignment
position           1001...... 1011...... 1021...... 1031...... 1041......

P855688      723   GGTGCTGCCC CCacagaaGA TGGGGCCTAT GGAGAAATTC TGGAATAAAT
mNDUFB6      904   CGTGTTGCCC CCGCGCAGGA TGTGGCCCCT GGAGCGATTC TGGGATAACT
P855691      685   CGTGTTGCCC CCGCGCAGGA TGTGGCCCCT GGAGCGATTC TGGAATAACT
                   ********** ********** ********** ********** **********
                   ********** ********** ********** ********** **********
                   ********** ********** ********** ********** **********
                   ********** ********** ********** ********** **********
                   ********** ********** ********** ********** **********
                   ********** **      ** ********** ********** **********
                   ********** **      ** *********
                   ********** **      ** *********
                   ********** **
                   ********** **
```

```
alignment
position          1051......  1061......  1071......  1081......  1091......

P855688      773  TTTTGgagaa  taaatCCCCT  TGGAGGAAAA  TGGTGAGTGA  CAGACCTCTC
mNDUFB6      954  TTTTGCGGGA  CgGGGCCGTG  TGGAAGAACA  TGGTGAGTGA  TGAGCCACAC
P855691      735  TTTTGCGGGA  CcGGGCCCTG  TGGAAGTACA  TGGTGAGTGA  TGTGCCACAC
                  **********  * ********  **********  **********  **********
                  **********  * ********  **********  **********  **********
                  **********  * ********  **********  **********  **********
                  *****            *****  **********  **********  **********
                  *****
                  *****

alignment
position          1101......  1111......  1121......  1131......  1141......

P855688      823  CGG--TCccc  ----------  ----------  -----CCCAA  CA---CATCC
mNDUFB6     1004  gggtgTCCGG  GTTCCCACCC  CGGGTCGCAG  CCTGGCCAAG  CCtccCAGCC
P855691      785  AGA--TCCGG  GTTCCCACTC  CGGGTCGCAA  CCCGGCCAAC  CC---CTTCC
                  ***  *****  **********  **********  **********  **   *****
                       *****  **********  **********  **********  **      **
                       *****  **********  **********  **********  **      **

alignment
position          1151......  1161......  1171......  1181......  1191......

P855688      843  CACTGCTGCC  GGCAGCCTGA  ACACTGAACg  tccgctccct  gcagccacac
mNDUFB6     1054  CACTCCTCTC  AGTTCCATCA  AGGCCCAACC  CTGTCCAGAG  CCCGCATACC
P855691      830  CACTCCTCTC  AGTTCCATTA  AGGCCCGACC  CTCTCCAGAG  CCCGCACACC
                  **********  **********  **********  **********  **********
                  **********  **********  **********  **********  **********
                  **********  **********  **********  **********  **********
                                                              **  **********

alignment
position          1201......  1211......  1221......  1231......  1241......

P855688      893  caaggactga  taacgctcag  tgct------  ----------  ----CTGGGA
mNDUFB6     1104  CGGGAGGGCC  CCAAGGGACA  ATCAGTCCAG  GGGCTCAGGT  TTAGCGGAGA
P855691      880  CGGGAGGGCC  CCAAGGGACA  ATCAGTCCAG  GCCCTCAGAT  TTAGCGGAGA
                  **********  **********  **********  **********  **********
                  **********  **********  **********  **********  **********
                  **********  **********  **********  **********  **********
                  **********  **********  ********

alignment
position          1251......  1261......  1271......  1281......  1291......

P855688      923  CACTGGCCAG  TCCCGAGAGT  CCtcctggag  tctCGGGAGG  CAGGAGTCCA
mNDUFB6     1154  CGCTGTCAGG  TCGCTATTGT  TCAGGG----  ---CTGGGGT  CTGGAGTCCA
P855691      930  TGCTGTCACG  TCGCTAGAGT  TCAGGG----  ---CCGGGGT  CTGGAGTCCT
                  **********  **********  ******         *******  **********
                  **********  ******  **  ******         *******  **********
                  **********  ******  **  ******         *******  **********

alignment
position          1301......  1311......  1321......  1331......  1341......

P855688      973  GGAATATGTa  TGGCTTTGAG  AGAATCATg-  ----------  ----------
mNDUFB6     1197  GGAGCAGGTg  gctgtcaggt  gtactgagaa  gtcgggtctt  aggttttaca
P855691      973  GGAGCAGGT-  TGGCTTCGGG  AGGATGATcc  ----------  ----------
                  *********   **********  ********
                  *********
                  *********

alignment
position          1351......  1361......  1371......  1381......  1391......

P855688     1002  ----------  ----------  ----------  ----------  ----------
mNDUFB6     1247  agacgctcgc  cgcacatgca  gttctttcga  gtgtagatac  cttttctcgg
P855691     1002  ----------  ----------  ----------  ----------  ----------

alignment
position          1401......  1411......  1421......  1431......  1441......
```

```
P855688    1002 - - - - - - - - - -  - - - - - - - - - -  - - - - - - - - - -  - - - - - - - - - -  - - - - - - - - - -
mNDUFB6    1297 g t t a a a c t g g  t c a g a a t t a a  c t g a g a t c a c  a g g c t g t g a t  t t c a g t a t t t
P855691    1002 - - - - - - - - - -  - - - - - - - - - -  - - - - - - - - - -  - - - - - - - - - -  - - - - - - - - - -

alignment
position        1 4 5 1 . . . . . .  1 4 6 1 . . . . . .  1 4 7 1 . . . . . .  1 4 8 1 . . . . . .  1 4 9 1 . . . . . .
P855688    1002 - - - - - - - - - -  - - - - - - - - - -  - - - - - - - - - -  - - - - - - - - - -  - - - - - - - - - -
mNDUFB6    1347 g a g a a c a c t t  a c g c g g t g g g  t a g g g t t g a g  g t g c c a g a g a  c t g g c c a g c t
P855691    1002 - - - - - - - - - -  - - - - - - - - - -  - - - - - - - - - -  - - - - - - - - - -  - - - - - - - - - -

alignment
position        1 5 0 1 . . . . . .  1 5 1 1 . . . . . .  1 5 2 1 . . . . . .  1 5 3 1 . . . . . .  1 5 4 1 . . . . . .
P855688    1002 - - - - - - - - - -  - - - - - - - - - -  - - - - - - - - - -  - - - - - - - - - -  - - - - - - - - - -
mNDUFB6    1397 g g t a t a g t a c  a g c c c c a t g g  g g t c g c a a c a  g a a g a c a g g g  t t c t a t t t g g
P855691    1002 - - - - - - - - - -  - - - - - - - - - -  - - - - - - - - - -  - - - - - - - - - -  - - - - - - - - - -

alignment
position        1 5 5 1 .
P855688    1002 - - - - -
mNDUFB6    1447 t a t g c
P855691    1002 - - - - -
```

Pairwise similarities:

For each pairwise alignment, the similarity (relative to the maximum similarity) and the number of identical nucleic acids (in % of shorter sequence) is given. Maximum values are underlined.

	mNDUFB6 (1451 bp)	P855691 (1001 bp)
P855688 (1001 bp)	0.208 37 %	0.260 40 %
mNDUFB6 (1451 bp)		1.000 80 %

Please note that the **similarity value 1.000** marks only the two **most similar** sequences, it does not necessarily mean that these sequences are identical.

Extract alignment region	
**Positions in alignment**	from: to:
**Output file**	dialign.seq

Extract aligned sequences

For comments, questions or bug reports, please contact support@genomatix.de

© Genomatix Software GmbH 1998-2005 - All rights reserved. License Agreement GEMS Launcher 4.1.1

Genomatix-Logo | Logout | Personal | Messages | GEMS-Logo
Logout | Personal | Messages

Genomatix Main GEMS ElDorado Gene2Promoter BiblioSphere
Frequently asked questions Your Results Your Sequences Your Protocol Help

GEMS Launcher Task: *DiAlign TF*: Multiple alignment plus TF sites
working on promoters.seq (3 seq.)

[Alignment] [Pairwise similarities]

DiAlign professional TF Release 3.1 December 2004 **Tue Jun 7 15:59:00 2005**

Solution parameters:

**Sequence file:**	promoters.seq (3 seq.)
**Type of sequences:**	nucleotide sequences
**Threshold T:**	0.00
**'*' signs below alignment denote:**	diagonal similarity (max. similarity is represented by 10 '*' signs)
**Alignment output:**	complete alignment is shown<br># nucleic acids per line: 50
**TF output:**	common TF matches located in aligned regions (common to 3 (100.0 %) sequences)
**Family matches:**	yes
**MatInspector library:**	Matrix Family Library Version 5.0 (February 2005)
**Selected groups** (core/matrix sim):	• ALL user_defined.lib (0.75/Optimized)<br>• ALL vertebrates.lib (0.75/Optimized)

Aligned Sequences:

No.	Sequence Name	Sequence Description	Length
1	P906353	sym=NDUFB7|loc=Loc4713|taxid=9606|spec=Homo sapiens|chr=19|ctg=NT_011295|str=(-)|start=5945188|end=5946188|len=1001| tss=501| comm=NADH dehydrogenase (ubiquinone) 1 beta subcomplex, 7, 18kDa; (NM_004146/501/bronze;)	1001 bp
2	P906354	sym=Ndufb7|loc=Loc66916|taxid=10090|spec=Mus musculus|chr=8|ctg=NT_078575|str=(+)|start=8532757|end=8533760|len=1004| tss=501,504| comm=NADH dehydrogenase (ubiquinone) 1 beta subcomplex, 7; (AK002678/514/gold;NM_025843/511/bronze;)	1004 bp
3	P906355	sym=na|loc=Loc361385|taxid=10116|spec=Rattus norvegicus|chr=19|ctg=NW_047534|str=(-)|start=11544622|end=11545622| len=1001|tss=501| comm=similar to NADH dehydrogenase (ubiquinone) 1 beta subcomplex, 7; (XM_341664/507/bronze;)	1001 bp

Alignment (DiAlign format):

Please note that only **upper-case** letters are considered to be **aligned**. For more information, please have a look at the user guide.

V$ETSF U$NRF2 V$OAZF V$HEN1 V$MINI V$CP2F V$E2FF V$NRF1 U$NRF1

```
alignment position  1 . . . . . . . . .   1 1 . . . . . . . .   2 1 . . . . . . . .   3 1 . . . . . . . .   4 1 . . . . . . . .
P906353       1 - - - - - - - - - -   - - - - - - - - - -   - - - - - - - - - -   - - - - - - - G G C   A A G A G A A T T T
P906354       1 c a c a c c t a c g   t a t g t g c a c t   C A C A C C G A C A   A A C C T A G G G A   A A G T G T T T G G
P906355       1 a c a t g - - - - -   - - - - - - - - - -   C A C A C C G A C A   A A C C T A A G G A   A A G T G T T T G G
```

```
                                                  ********** ********** **********
                                                  ********** ********** **********
                                                  ********** ********** **********
                                                  ********** ********** **********
alignment position 51........ 61........ 71........ 81........ 91........
P906353         14 GAGCCCAGGA GGTGGAGGCT GCAGTGagcc gagatcgcgc caatacactc
P906354         51 AAGTTCGAGT GTTGGAAATG GTAGTGTATA TCTGTGGGAA AGCACTTATC
P906355         36 AGGTTGGAGa GTTGGAAGTG acAGTATATA TGCGTAGGAG AGCACTTATC
                   ********** ********** ********** ********** **********
                   *********               ******** ********** **********
                   *********
                   *********
alignment position 101....... 111....... 121....... 131....... 141.......
P906353         64 cagtctgggc gacaggtact gtgtct---- ----CCAAAA ACAAAAAATT
P906354        101 TACCATATGC TAggcctggG GGTCTATCCT AAAACCAGAA AAAAAAATTA
P906355         86 TGCCATGTGC TAt------G TGTCTATCCC AACGCCAttt tattattatt
                   ********** **       * ********** ********** **********
                   ********** **
alignment position 151....... 161....... 171....... 181....... 191.......
P906353        106 CTTAAAGtga ctttatgcaa aattacatac attaacgata taatagatat
P906354        151 ATTTAAGaaa aaaa------ ---------- ---------- ----------
P906355        130 attattatta ttattattat tat------- ---------- ----------
                   *******
alignment position 201....... 211....... 221....... 231....... 241.......
P906353        156 cgtgccTTTT TTAAGAGAAA TTTGAATGTT TTCAAAGAAA aaactt----
P906354        165 ---------- ---------- ---------- ----AAAAAA AGAGGAGGAG
P906355        153 ------TATT ATTATATTAT TATTATTATT TTAGAAGAAA GGAGGGGGAG
                         **** ********** ********** ********** **********
                                                        ****** **********
alignment position 251....... 261....... 271....... 281....... 291.......
P906353        202 ---------- ---------- ------TTTG GAAAATGata tgaaaaataa
P906354        181 GAAGAGGAAC GCGGAAGATG GTTC------ ---------- ----------
P906355        197 GGGAAGGAAC GTGGAATATG GTTCagTTTG TAAAATGcct gccatgcgat
                   ********** ********** ****  **** *******
                   ********** ********** ****
alignment position 301....... 311....... 321....... 331....... 341.......
P906353        226 ttttaatatg tgctttagcc tcgggctacc agaagagccc cagatagGGT
P906354        205 ---------- ---------- ---------- ---------- -------GGT
P906355        247 cgtgctagtc a--------- ---------- ---------- ----------
                                                                  ***
alignment position 351....... 361....... 371....... 381....... 391.......
P906353        276 CGAGGGAGGC CTCAGTCCCa caccgtcccc ttctgccccg cccagatgga
P906354        208 CCAGGGTGTC CACAGTACCC ATGTAtacac atgtatACAG CAGGCTTGGT
P906355        258 -----GTGTC CACAGTACCC ATGTAa---- ------ACAG CAGGCTTGGT
                   ********** ********** ******           **** **********
                        ***** ********** ******           **** **********
                                                          **** **********
alignment position 401....... 411....... 421....... 431....... 441.......
P906353        326 tttccag--- ---------- ---------- --GCTCAGGG AAGTCC----
P906354        258 GGCACACATT TaTAATCCTA GCACTGGGCA GAGCACAGGG GACcctgcca
P906355        293 GGCACACATT TgTAATCCTA GGACTGGGTA GAGCATAGGG AACGCC----
                   ********** * ******** ********** ********** ******
                   ********** * ******** ********** ********** ***
                   ********** *
alignment position 451....... 461....... 471....... 481....... 491.......
P906353        347 ---------- ---------- -------CCT CTCGGGTCCA ACCATATCCT
P906354        308 cctccccgct tccacacccg cactcctCCC TGCAACTCCC TGGCCAGCCT
P906355        339 ---------- ---------- -------CCC TGCAGATCCC TGGCCAGCCT
```

```
                                                  *** ********** **********
                                                  *** ********** **********
                                                  *** ********** **********
                                                  *** ********** **********

alignment position  501....... 511....... 521....... 531....... 541.......
P906353         370 Gcgaagagac aca------- ------GCAC CTGTCCCGCC TCCGCCTGTC
P906354         358 GGCCTTCTAG CCAACTGGCG TTCGCCGCAC CCGCTCTGTC TCCTCCACAC
P906355         362 GGCTTTCTAG CCAACTGGCG TTCGCCGCAG CCGCCCTGTC TCCTCCACGC
                    ********** ********** ********** ********** **********
                    ********** ********** ********** ********** **********
                    ********** ********** ********** ********** **********
                    ********** *******          **** ********** **********

alignment position  551....... 561....... 571....... 581....... 591.......
P906353         407 CGCGAGGTGA CGCCAGgcgg accggagcga tcctctggag tccTTCATCT
P906354         408 CGCCAGGGGG TGTCAGCGAG CCAGGAAGCG TTCCATCG-- ---TTCATCT
P906355         412 CACCAGGAGG TGCCAGCGGG CCAGGAAGTC TTCCACCG-- ---TTCgTCT
                    ********** ********** ********** ********      *******
                    * ******** ********** ********** ********      *******
                    * ******** ********** ********** ********      *******
                    *                                                 *** ***
                                                                  *** ***
                                                                         ***
                                                                         ***
                                                                         ***

alignment position  601....... 611....... 621....... 631....... 641.......
P906353         457 CTATGATCTC CGCAGCTGCC TAAATGACCC GGAAGctgtg tctgACTGAG
P906354         453 CTATGACCCC CGCCGCAGCC TAGATGACCC GGAAGaC--- ----ACTGAA
P906355         457 CTATGACCCC CGCTGCGGGC TAGATGACCC GGAAGtC--- ----ACTGAA
                    ********** ********** ********** ***** *        ******
                    ********** ********** ********** ***** *        ******
                    ********** ********** ********** ***** *        ******
                    ********** ********** ********** ***** *        ******
                    ********** ********** ********** ***** *        ******
                    ********** ********** ********** *****          ******
                    ********** ********** ********** ***             ******
                    ********** ********** ********** ***             ******
                                                                    ******
                                                                    ******

alignment position  651....... 661....... 671....... 681....... 691.......
P906353         507 GGGTCAGTGG TTCCGGGTAG GAGCTAGGTG ACCCtCGGCT GCTgcaggga
P906354         496 GGGGCAGTGC TTCCAGGTGG GAGCGAGGTG ACCC-CGGCT ACTAAAGAAG
P906355         500 GGGGCAGTGC TTCCAGGTGG GAGCGAGGTG ACCC-CGGCT GCTAAAGAAA
                    ********** ********** ********** **** ***** **********
                    ********** ********** ********** **** ***** **********
                    ********** ********** ********** **** ***** **********
                    ********** ********** ********** **** ***** **********
                    ********** ********** ********** ****
                    ********** ********** ********** ****
                    ********** ********** ********** ****
                    ********** ********** ********** ****
                    ********** ********** ********** ****
                    ********** ********** ********** ***

alignment position  701....... 711....... 721....... 731....... 741.......
P906353         557 tctgcagcga ctgcAGCCAT GGGGGCCCAC CTGGTCCGGC GCTACCTGGG
P906354         545 GGTAAGGCAG AGT-AGCCAT GGGGGCGCAC CTGACCCGGC GCTATCTGTG
P906355         549 GGTAGGGCAG AGT-AGCCAT GGGGGCGCAC CTGACCCGGC GCTATCTGTG
                    ********** *** ****** ********** ********** **********
                    ********** *** ****** ********** ********** **********
                    ********** *** ****** ********** ********** **********
                    ********** *** ****** ********** ********** **********
                                   ****** ********** ********** **********
                                   ****** ********** ********** **********
                                   ****** ********** ********** **********
                                               ***** ********** **********
                                               ***** ***  ***** **********
```

```
                                                        ***** **********
alignment position 751.......  761.......  771.......  781.......  791.......
P906353       607  CGATGCCTCG GTGGAGCCCG ACCCCCTGCA GATGCCAACC TTCCCGCCAG
P906354       594  GGATGCCTCG GTGGAGCCTG ACCCCGAGAA GATACCCAGC TTCCCGCCGG
P906355       598  GGATGCCTCG GTGGAGCCTG ACCCTGAGAA GATGCCCAGC TTCCCGCCGA
                   ********** ********** ********** ********** **********
                   ********** ********** ********** ********** **********
                   ********** ********** ********** ********** **********
                   ********** ********** ********** ********** **********
                   ********** ********** ********** ********** **********
                   ********** ********** ****     * ********** **********
                   ********** ********** ****     * ********** **********
                   ********** ********** ****
                   ********** *****
                   ********** *****

alignment position 801.......  811.......  821.......  831.......  841.......
P906353       657  ACTACGGCTT CCCCGAACGC AAGGAGCGCG GTGAGgccCA GTGCGCAGGC
P906354       644  ACCTCGGCTT TCCGGAGCGC AAGGAGCGAG GTGAGCT-CA GTGCGCAGGC
P906355       648  ACTACGGtcT TCCGGAGCGC AAGGAGCGAG GTGAGCT--- -TGCGCAGAC
                   ********** ********** ********** ******* ** **********
                   ********** ********** ********** *******     *********
                   *******  * ********** ********** *******     *********
                   *******  * ********** ********** *****       *********
                   **       * ********** ********** ****
                   **          ********* ********** ****
                   **

alignment position 851.......  861.......  871.......  881.......  891.......
P906353       707  GCagcc---- ---------- ---------- --AG------ ------AGGG
P906354       693  GCGACCGCAG ATCCCGCGGA ACTCAGCTGC ctgcttgcgg agggccGGGT
P906355       694  GCGACCGCCG ATCCCGCGGA ACTCGGCTGC agAG------ ------GGGT
                   ********** ********** **********   **             ****
                   ********** ********** **********
                   ********** ********** **********
                   ********** ********** **********

alignment position 901.......  911.......  921.......  931.......  941.......
P906353       719  CGTGGGGCGC GGGTGCCTGA acacctggca ccccagggct ggcaccccag
P906354       743  CGAGTCGGGA GGGCGacctc cttg------ ---------- ----------
P906355       732  CGGATCGCGA GGGCGCCTGA g--------- ---------- ----------
                   ********** **********

alignment position 951.......  961.......  971.......  981.......  991.......
P906353       769  ggctgggctc taacctcagg tgttgtatct gggtttgggt ctagactagg
P906354       767  ---------- ---------- ---------- ---------- ----------
P906355       753  ---------- ---------- ---------- ---------- ----------

alignment position 1001......  1011......  1021......  1031......  1041......
P906353       819  agtccacagg acgtgcatga ccccacctcA GTGTGGAGGC gttccagccc
P906354       767  ---------- ---------- ---------A GTGTGGAGGC tgtgc-----
P906355       753  ---------- ---------- ---------- ---------- ----------
                                                  * **********

alignment position 1051......  1061......  1071......  1081......  1091......
P906353       869  aaGGGCACGG CTTGAGCAAA AGCCAGGGAg caaaagtgag gcgacaa---
P906354       783  --GGGGATGG CTAAAGCAAA AGGCTGGGAC GAGACAGCAG AGAACGGGaa
P906355       753  ---------- -------AAA AGGCTGGGAC AAGACAGCAG AGAACGGGga
                     ******** ********** ********** ********** ********
                                     *** ********** ********** ********
                                     *** ********** ********** ********
                                     *** *********

alignment position 1101......  1111......  1121......  1131......  1141......
P906353       916  ---------- ---------- ---------- ---------- ----------
P906354       831  gat-GAGACT GCCCCGGTGC ACTTCGTCCT GGGCACAGCG CGGAgccta-
P906355       786  agacGAGACT GCCCCGGTGC ACTTCGTCCT GGACACAGCG CGGAgcctag
```

```
                            ****** ********** ********** ********** ****
                            ****** ********** ********** ********** ****
                            ****** ********** ********** ********** ****
                            ****** ********** ********** ********** ****
                            ****** ********** ********** ********** ****

alignment position      1151...... 1161...... 1171...... 1181...... 1191......
P906353             916 ---------- ---------- ---------- ---------- ----------
P906354             879 GGACATGTTC TTTGCATCTC CATACATTGT CACTagca-T TTGGTTACGT
P906355             836 GGACATGTTC TTTGCATCTC CATACAGTGT CACTcgtatT TTGGTTATGT
                        ********** ********** ********** ****     * **********
                        ********** ********** ********** ****     * **********
                        ********** ********** ********** ****     * **********
                        ********** ********** ********** ****

alignment position      1201...... 1211...... 1221...... 1231...... 1241......
P906353             916 ---------- -------GTG GGGGTTCGGG CAGGAATGCG ACGCGCagag
P906354             928 ACAATAATAC AAGGTTGGGG GGc--TGGAG CGATCATGCt c----CTTGT
P906355             886 ACAATAATAC AAGGTCGGCG GGGGATGGGG CGGTCCTGCA GTGCACTTGT
                        ********** ********** ********** ********** **********
                        ********** ********** **                         *****
                        ********** ********** **                         *****
                                                                         *****

alignment position      1251...... 1261...... 1271...... 1281...... 1291......
P906353             949 ctgggtcgag tctttttttt ttttcttttg agacggagtc tcgcttttc
P906354             972 CCTGTCTAGT GTGATGGTGC TGGGGGAGGA ACC------- ----------
P906355             936 CCTGTCTAGT GTGATGGTGC TGGGGATGGA ACCcaaggcc tgtgtgtact
                        ********** ********** ********** ***
                        ********** ********** ********** ***
                        ********** ********** ********** ***
                        ********** ********** ********** ***

alignment position      1301...... 1311..
P906353             999 ttt------- ------
P906354            1005 ---------- ------
P906355             986 cgcacggttc ttgttc
```

Pairwise similarities:

For each pairwise alignment, the similarity (relative to the maximum similarity) and the number of identical nucleic acids (in % of shorter sequence) is given. Maximum values are underlined.

	P906354 (1004 bp)	P906355 (1001 bp)
**P906353 (1001 bp)**	0.217<br>31 %	0.177<br>28 %
**P906354 (1004 bp)**		<u>1.000</u><br><u>72 %</u>

Please note that the **similarity value 1.000** marks only the two **most similar** sequences, it does not necessarily mean that these sequences are identical.

Extract alignment region	
**Positions in alignment**	from: to:
**Output file**	dialign.seq

Extract aligned sequences

For comments, questions or bug reports, please contact support@genomatix.de

© Genomatix Software GmbH 1998-2005 - All rights reserved. License Agreement GEMS Launcher 4.1.1

Logout Personal Messages

GEMS Launcher Task: *DiAlign TF*: Multiple alignment plus TF sites
working on promoters.seq (3 seq.)

[Alignment] [Pairwise similarities]

DiAlign professional TF Release 3.1 December 2004 **Tue Jun 7 16:10:54 2005**

Solution parameters:

**Sequence file:**	promoters.seq (3 seq.)
**Type of sequences:**	nucleotide sequences
**Threshold T:**	0.00
**'*' signs below alignment denote:**	diagonal similarity (max. similarity is represented by 10 '*' signs)
**Alignment output:**	complete alignment is shown<br># nucleic acids per line: 50
**TF output:**	common TF matches located in aligned regions (common to 3 (100.0 %) sequences)
**Family matches:**	yes
**MatInspector library:**	Matrix Family Library Version 5.0 (February 2005)
**Selected groups** (core/matrix sim):	• ALL user_defined.lib (0.75/Optimized)<br>• ALL vertebrates.lib (0.75/Optimized)

Aligned Sequences:

No.	Sequence Name	Sequence Description	Length
1	P927722	sym=NDUFB8|loc=Loc4714|taxid=9606|spec=Homo sapiens|chr=10| ctg=NT_030059|str=(-)|start=21037647|end=21038647|len=1001| tss=501| comm=NADH dehydrogenase (ubiquinone) 1 beta subcomplex, 8, 19kDa; (NM_005004/510/bronze;)	1001 bp
2	P927731	sym=Ndufb8|loc=Loc67264|taxid=10090|spec=Mus musculus| chr=19|ctg=NT_039692|str=(-)|start=4936006|end=4937027| len=1022|tss=501,512,522| comm=NADH dehydrogenase (ubiquinone) 1 beta subcomplex 8; (AK028070/501/gold;AK010177/522/gold;NM_026061/512/bronze;)	1022 bp
3	P927735	sym=na|loc=Loc293991|taxid=10116|spec=Rattus norvegicus| chr=1|ctg=NW_047567|str=(-)|start=293982|end=294982| len=1001|tss=501| comm=similar to NADH dehydrogenase (ubiquinone) 1 beta subcomplex 8; (XM_215269/529/bronze;)	1001 bp

Alignment (DiAlign format):

Please note that only **upper-case** letters are considered to be **aligned**. For more information, please have a look at the user guide.

V$MEF2 V$TBPF V$NRF1 U$NRF1 V$EVI1 V$YY1F V$RBPF V$AP1R V$EREF U$EREF V$ZF35 V$NKXH V$EBOX

```
alignment position  1.........  11........  21........  31........  41........
P927722           1 cag-------  ----------  ----------  ----------  ----------
P927731           1 tttttttttt  cctgaaaaat  gGGAGGTTTG  GGGCTGGGTC  AGGCGAGCCG
P927735           1 at--------  ----------  -GGGGGTTTG  GGGCTGTGTC  AGGAGAGCCG
```

```
                                          ********* ********** **********
                                          ********* ********** **********
                                          ********* ********** **********

alignment position 51........ 61........ 71........ 81........ 91........
P927722          4 ---------- --GGCCGAAT TGTGTGTTGC ACTGGCGTCT AAGTGA----
P927731         51 CCTCCAGTAA TcGGCTTGGT TTTTGTATTT GTTGGGTt-G AGGTGATTGA
P927735         32 CCTCCAGTAA TaGGCTTGGT TTTGTATTTC GTTGGATGCG AGGTGACTGA
                   ********** * ******** ********** ********** **********
                   ********** *                              * **********
                   ********** *                              * **********

alignment position 101....... 111....... 121....... 131....... 141.......
P927722         38 ---------- ---------- ---------- ---------- ----------
P927731        100 GACATTTAGA TGAACTGAGA ---------C AACTTTGAGT ATATAAGGAA
P927735         82 GACATTTAGA TGAACCGAGA ctaaggagcC AACTTTGAGT ATATAAGGAA
                   ********** **********          * ********** **********
                   ********** **********          * ********** **********
                   ********** **********          * ********** **********

alignment position 151....... 161....... 171....... 181....... 191.......
P927722         38 --AGATCAAC CCATTTGGAG aacAGAACAA CAAGCAAGAC CAACAGGTGA
P927731        141 AGAG------ --ATTTGGAG ---AAAGGAT CAAACAAAAC CAAGAGCTAA
P927735        132 AGAGGTGAGC CTATTTGGAG ---AGAGGAT CAAACAAAAC CAAGAGCTAA
                   ********** **********    ******* ********** **********
                   ****         ********    ******* ********** **********
                   ****         ********    ******* ********** **********
                                ********    ******* ********** **********
                                            ******* ********** **********
                                                        ****** **********

alignment position 201....... 211....... 221....... 231....... 241.......
P927722         86 ATTTGAAGAA TGATCATGTt tggggatgga aggaagtgcc cgtgaaggag
P927731        180 ATGTGAAAAA GGACTGTGT- ---------- ---------- ----------
P927735        179 ATGTGAAAAA TGAGTGCGT- ---------- ---------- ----------
                   ********** *********
                   ********** *********
                   ********** *********
                   ********** ***
                   *****
                   *****

alignment position 251....... 261....... 271....... 281....... 291.......
P927722        136 aggaaagcaa gcagccagag aaGAGAGTAA GACAGGCTCA TTTCACTagt
P927731        199 ---------- ---------- --GTGAGGAC AGAAGGCTGG TTTTACTTTG
P927735        198 ---------- ---------- --GTGAGGAC AGACGCCTGA TTTTACTTTG
                                           ******** ********** **********
                                           ******** ********** **********
                                                                       **

alignment position 301....... 311....... 321....... 331....... 341.......
P927722        186 acagctcctg ggggaggtgg ttaAGCTGTC Tacctgagtt gctcaggttc
P927731        227 CAC------- ---------- ---AGCGGTC TGAGATATTC GGCTGGCG--
P927735        226 CTC------- ---------- ---AGCTGTC TGAGATATTC GGCGGGAG--
                   ***                      ******* ********** ********
                   ***                      ******* ********** ********
                   ***                      ******* ********** ********

alignment position 351....... 361....... 371....... 381....... 391.......
P927722        236 cgagttccca tCATCCCTGG ATGGGTCTAA GTCAGCTCAG GGAGAAAAAG
P927731        255 ---------- -CAGCCCCGA AAGGGCCTCA GTCGGTTGAA GGCGAAAAGG
P927735        254 ---------- -CAGCCCTGA A--------- ---------- ----AAAAGA
                               ********* ********** ********** **********
                               ********* *                         ****
                               ********* *

alignment position 401....... 411....... 421....... 431....... 441.......
P927722        286 GACACAACAT TCTGAAATGT CAGAgacct- ---GCCTGGT TTTCCCTTCT
P927731        294 AATACAGTAA CTTGCAATAT CAAGTAAGcA GTAGCTTTGT TTTCTCTCCT
```

```
P927735      270 AACACTATAA CTCGAAATAT CAAATGAGtA GTAGCTTTGT TTTCTCTCCT
                 ********** ********** *******  * ********** **********
                                                * ********** **********
                                                * ********** **********

alignment position 451....... 461....... 471....... 481....... 491.......
P927722      332 TCCCGCTTAT CCTTCccagc ctctatccaa cccttggctt ------CCCA
P927731      344 TGCTGTCTGT CCTggcacc- ---TCATCAG CCGCTCACCG TCCAGGCTCT
P927735      320 TGCTGGCTGT CTATCttggc actTCACCAG CCACTCACTG CGCAGGCTCT
                 ********** *****         ******* ********** **********
                 ********** *             ******* ********** **********
                 ********** *                                      ****

alignment position 501....... 511....... 521....... 531....... 541.......
P927722      376 ACTGCGTATT TAA----ACC CCGCCCCTGT TGAACCCttc ggcttttCTT
P927731      390 ACAGCATATT TAAataaAGC CCGCCCAGCT TGATGCCGGG A------CTT
P927735      370 ACAGCGTATT TAA----AGC CCGTCCAGCT TGACACCGGG A------CTT
                 ********** ***    *** ********** ********** *      ***
                 ********** ***    *** ********** ********** *      ***
                 ********** ***    *** ********** *******           ***

alignment position 551....... 561....... 571....... 581....... 591.......
P927722      422 CACTCCCGGC TTCCTCCGTC A-CGTTTGGC CCGGCTCAGC CGCCGTGACA
P927731      434 CTGCGCTGGC TTCggagatc gcCGTTTGGC CCGACTCAGC CACCGTGACC
P927735      410 CAGCGCTAGC TTCAACGATC G-CGTTTGTC CCcGCTCAGC CACCGTGACC
                 ********** ********** * ******** ********** **********
                 ********** ***          ******** ********** **********
                 ********** ***                      ******* **********
                                                     ******* **********
                                                      ****** **********
                                                      ****** **********
                                                      ****** *********

alignment position 601....... 611....... 621....... 631....... 641.......
P927722      471 GGCCTTTGCG CATGTGCAGA aggGAAACGT GAAGAAGGTG AAGATGGCGG
P927731      484 CGCTTTTGCG CATGTGCAGA gt-GAACCGC GGAGAAGGTG AAGATGGCTG
P927735      459 CGCCTTTGCG CATGTGCAcg gcgagcgt-C GGAGAAGGTG AAGATGGCGG
                 ********** **********    ******* ********** **********
                 ********** ********      ******* ********** **********
                 ********** ********            * ********** **********
                 ********** ********              ********   ********
                 ********** ********
                 ********** ********

alignment position 651....... 661....... 671....... 681....... 691.......
P927722      521 TGGCCAGGGC CGGGGTCTTG GGAGTCCAGT GGCTGCAAAG GGCATCCCGG
P927731      533 CCGCCCGGGC TGCGGCCCTG GGAGTCCGAT GGCTGCAGAG GACAACCCGG
P927735      508 CGTCCCGGGC TGCGGCCCTG GGAGTCCGGT GGCTGCAAAG GACATCCCGc
                 ********** ********** ********** ********** **********
                 ********** ********** ********** ********** **********
                 ********** ********** ********** ********** *********
                    ******* ********** ********** ********** *********
                    ******* ********** ********** ********** ***
                    *******    **   ** ********** ********** ***
                                    ** ********** ********** ***

alignment position 701....... 711....... 721....... 731....... 741.......
P927722      571 aaCGTGATGC CGCTGGGCGC ACGGACAGGT CCGCgagaac gggcgagacc
P927731      583 gGCGTGGTGC CACTGGAGGC ACGGAGAGGT CTGCACG--- ----------
P927735      558 tGCGTGGTGC CACTTGAGGC ACGGAGAGGT CTGCACG--- ----------
                  ********* ********** ********** *******
                  ********* ********** ********** *******
                  ********* ********** ********** *******
                  ********* ********** ********** *******
                   ******** ********** ********** ****
                   ******** ********** ********** ****

alignment position 751....... 761....... 771....... 781....... 791.......
P927722      621 tcaGTGTGGG AAGGGGGTAC CTGAAATTTG GTGTATCGGG AGGggggatc
```

```
P927731       620 ---GTGTGGG AAGCGGGTGC CTGAAGTTTA CTGCTTCGGG ATGGAGGCAT
P927735       595 ---GTGTGGG AAGCGGGTGC CTGATGTTTG CTGCTTCGGG AGGGAGGCAT
                     ******* ********** ********** ********** **********
                     ******* ********** ********** ********** **********
                     ******* ********** ********** ********** *******
                     ******* ********** ********** ********** ***
                     ******* ********** ********** ********** ***
                     ******* ********** ********** ********** *
                     ******* ********** ********** ********** *

alignment position 801....... 811....... 821....... 831....... 841.......
P927722       671 gtagatgccg cggcggcagc ctccgggtag GGAGTGGAGG TGGCGTTGAT
P927731       667 CCTT------ ---------- ---------- GGGACGGTGG CGGAGGTGTC
P927735       642 CCTT------ ---------- ---------- GGAATGGTGG CGTCGGTGTT
                  ****                             ********** **********
                  ****                             ********** **********

alignment position 851....... 861....... 871....... 881....... 891.......
P927722       721 ATATCCAGAA A-ATGGGGTG GAGGATCGCA CTGatcct-- ----------
P927731       691 CTGGCTGGGG gtAAGGGGTG GGAGGTCGCG GTGGATTCGT GTGCCCAAAG
P927735       666 CTGCCTGGGG C-TGGGGGTG Gtacagt--- -TGAATTACT GAGTCCAAAG
                  ********** *  ******* ********** ********** **********
                  **********                        ********* **********

alignment position 901....... 911....... 921....... 931....... 941.......
P927722       758 ---------- ---------- ------GCGA AGTCCCGGGC TCGGACCTCT
P927731       741 AAGGGATACA GGGTTGCTgt aGTCCGGCAA GGTGCGAAAC gaCAGCCTCT
P927735       711 AAGGAATACA AGGTTGCTat -GTTCGGCCA GGTGCGGAAC AGCAACCTCT
                  ********** ********   ********* ********** **********
                  ********** ********                          ********
                                                               ********

alignment position 951....... 961....... 971....... 981....... 991.......
P927722       782 GCAGCCCCTG CGACTTGAAG CCTT-CGGag cggctggaga gcgggct---
P927731       791 GCAGTCACTG CGGCTTAGTC CTTT-CTGGG TGGGCGCCTA AAGCACCGTG
P927735       760 GCAGTTACTG CAGCTTAGTC CTTTtCTGGG TGGGTGTCTA AAGAACCGTA
                  ********** ********** **** ***** ********** **********
                  ********** ********** **** ***** ********** **********
                  ********** ********** **** ***

alignment position 1001...... 1011...... 1021...... 1031...... 1041......
P927722       828 CGCTTCTGAC CTGTTCTCTT CTGCGtTCTA GCCTCCCACA TGACCAAGGA
P927731       840 TCCATCTGAC CTGCATCCCT CTGCG-TCCA GCCTTCCATA TGACCAAGGA
P927735       810 CCCATCTAAC CTGCTTCCCT CTGTG-TCCA GCCTTCCACA TGACCAAGGA
                  ********** ********** ***** **** ********** **********
                  ********** ****  ***** ***** **** ********** **********
                                   ***** ***** **** ********** **********
                                   ***** ***** **** ********** **********
                                               **** ********** **********
                                               **** ********** **********
                                                  * ********** **********
                                                  * ********** **********

alignment position 1051...... 1061...... 1071...... 1081...... 1091......
P927722       878 CATGTTCCCG GGGcccTATC CTAGGACCCC AGAAGAACGG GCCGCcGCCG
P927731       889 CATGTTGCCG GGGTCATATC CTAGGACCCC AGAGGAACGC GCCGCGGCCG
P927735       859 CATGATGCCG GGGTCATATC CTAGGACCCC AGAAGAACGG GCCGCGGCCG
                  ********** ********** ********** ********** **********
                  ********** ********** ********** ********** **********
                  ********** ********** ********** ********** **********
                  ********** ***   **** ********** ********** *****  ****
                  ********** ***   **** ********** ********** *****  ****
                  ****  ***** ***   **** ********** ***  ****** *****  ****
                  ****  ***** ***   **** ********** ***  ****** *****  ****
                  ****                                    ****** *****  ****
                                                                        ****
                                                                        ****

alignment position 1101...... 1111...... 1121...... 1131...... 1141......
```

```
P927722      928 CCAAGAAGTA TAATATGCGT GTGGAAGACT ACGAaCCTTA CCCGGATGAT
P927731      939 CCAAGAAGTA TAACATGCGA GTGGAAGACT ACGAGCCATA CCCCGATGAT
P927735      909 CCAAGAAGTA TAATATGCGT GTGGAAGACT ACGAGCCGTA CCCAGATGAT
                 ********** ********** ********** ********** **********
                 ********** ********** ********** ********** **********
                 ********** ********** ********** ********** **********
                 ********** ********** ********** ********** **********
                 ********** ********** ********** **** ***** **********
                 ********** ********** ********** **** ***** **********
                 ********** ********** ********** **** ***** **********
                 ********** ********** ********** ****
                 ********** ********** ********** ****
                 ********** ********** ********** ****

alignment position 1151...... 1161...... 1171...... 1181...... 1191
P927722      978 GGCATGGGGT GAGAcagaga gggt------ ---------- ---
P927731      989 GGCATGGGGT GAGtg--AGG TAGAGGACTC GGGTCA---- ---
P927735      959 GGCATGGGGT GAGAtgaAGG AAGTCTACTC GCATCAgggg cag
                 ********** ****   *** ********** ******
                 ********** ****
                 ********** ***
                 ********** ***
                 ********** ***
                 ********** ***
                 ********** ***
```

Pairwise similarities:

For each pairwise alignment, the similarity (relative to the maximum similarity) and the number of identical nucleic acids (in % of shorter sequence) is given. Maximum values are underlined.

	P927731 (1022 bp)	P927735 (1001 bp)
**P927722 (1001 bp)**	0.340<br>50 %	0.384<br>50 %
**P927731 (1022 bp)**		1.000<br>78 %

Please note that the **similarity value 1.000** marks only the two **most similar** sequences, it does not necessarily mean that these sequences are identical.

Extract alignment region	
**Positions in alignment**	from: to:
**Output file**	dialign.seq

Extract aligned sequences

For comments, questions or bug reports, please contact support@genomatix.de

---

© Genomatix Software GmbH 1998-2005 - All rights reserved. License Agreement GEMS Launcher 4.1.1

Genomatix-Logo Logout Personal Messages GEMS-Logo
Logout Personal Messages

Genomatix_MainGEMSElDoradoGene2PromoterBiblioSphere
Frequently_asked_questionsYour_ResultsYour_SequencesYour_ProtocolHelp

GEMS Launcher Task: *DiAlign TF*: Multiple alignment plus TF sites
working on promoters.seq (3 seq.)

[Alignment] [Pairwise_similarities]

DiAlign professional TF Release 3.1 December 2004 **Tue Jun 7 17:05:45 2005**

Solution parameters:

**Sequence file:**	promoters.seq (3 seq.)
**Type of sequences:**	nucleotide sequences
**Threshold T:**	0.00
**'*' signs below alignment denote:**	diagonal similarity (max. similarity is represented by 10 '*' signs)
**Alignment output:**	complete alignment is shown<br># nucleic acids per line: 50
**TF output:**	common TF matches located in aligned regions (common to 3 (100.0 %) sequences)
**Family matches:**	yes
**MatInspector library:**	Matrix Family Library Version 5.0 (February 2005)
**Selected groups** (core/matrix sim):	• ALL user_defined.lib (0.75/Optimized)<br>• ALL vertebrates.lib (0.75/Optimized)

Aligned Sequences:

No.	Sequence Name	Sequence Description	Length
1	P922003	sym=NDUFB9|loc=Loc4715|taxid=9606|spec=Homo sapiens|chr=8|ctg=NT_008046|str=(+)|start=38769028|end=38770040|len=1013| tss=501,513| comm=NADH dehydrogenase (ubiquinone) 1 beta subcomplex, 9, 22kDa; (AK026538/513/gold;NM_005005/501/silver;)	1013 bp
2	P922004	sym=Ndufb9|loc=Loc66218|taxid=10090|spec=Mus musculus|chr=15|ctg=NT_039621|str=(+)|start=20271682|end=20272682| len=1001|tss=501| comm=NADH dehydrogenase (ubiquinone) 1 beta subcomplex, 9; (AK003540/601/gold;NM_023172/601/bronze;)	1001 bp
3	P922005	sym=na|loc=Loc299954|taxid=10116|spec=Rattus norvegicus|chr=7|ctg=NW_047779|str=(-)|start=13034523|end=13035523| len=1001|tss=501| comm=similar to NADH dehydrogenase (ubiquinone) 1 beta subcomplex, 9, 22kDa; NADH dehydrogenase (ubiquinone) 1 beta subcomplex, 9 (22kD, B22); (XM_216929/577/bronze;)	1001 bp

Alignment (DiAlign format):

Please note that only **upper-case** letters are considered to be **aligned**. For more information, please have a look at the user guide.

V$ECAT V$ETSF U$NRF2 V$STAF V$TBPF V$ZF5F V$OCT1

```
alignment position  1 . . . . . . . . .  1 1 . . . . . . . .  2 1 . . . . . . . .  3 1 . . . . . . . .  4 1 . . . . . . . .
P922003           1 g a t a t g a a c g  a a t g a a t g c t  t g a g t g a a t t  g g t c c c c a g a  a g c g g t c a g c
P922004           1 t a g a - - - - - -  - - - - - - - - - -  - - - - - - - - - -  - - - - - - - - - -  - - - - - - - - - -
P922005           1 - - - - - - - - - -  - - - - - - - - - -  - - - - - - - - - -  - - - - - - - - - -  - - - - - - - - - -
```

```
alignment position  51........ 61........ 71........ 81........ 91........
P922003          51 ctctccgcca aggcagaggG GAAGGGCCGG GAGGTGCACG tgggatgaat
P922004           5 ---------- ---------- ---------- AGAGTTCACG AAAGACA---
P922005           1 ---------- ---------G GGAGGGAGGG AGGGTTCACG AAATACC---
                               * ********** ********** *******
                                                       ********** *******

alignment position  101....... 111....... 121....... 131....... 141.......
P922003         101 gGAAACATAG TGATTGGCct ttttggag-- -------TTT TCTTCTTaaa
P922004          22 -GAAGAAAAG TGATTGGCtT GGGGGGCGGT GCTTTttgag aga-------
P922005          29 -GGAGAATAG TGATTAGC-T GGGGGGCGGT GCTTTctTTT TCTTTTTtgg
                     ********* ********   ********** *****  *** *******
                     ********* ******** * ********** *****

alignment position  151....... 161....... 171....... 181....... 191.......
P922003         142 aaactgaata cattacagat gt-------- ---------- ----------
P922004          64 ----TGGGAG GACTCGGGGA CTAGCCCATG CTAGGCgatc AGTCTACGGC
P922005          77 gtggTGGGAG GACTCGGGGA TTAGCCCATG CTAGGC---- AGTCTACCGC
                        ****** ********** ********** ******     **********
                        ****** ********** ********** ******
                        ****** ********** ********** ******
                        ****** ********** ********** ******

alignment position  201....... 211....... 221....... 231....... 241.......
P922003         164 ---------- ---------- ---------- ---------- ----------
P922004         110 TGaaGCACAC CCCACACCTA TTCCTCTCTT TCCAGTTGAA TTaaacACAC
P922005         123 TGtcGCACAT CCCACACCTA TACCTCTATT TTCAGTTGAA TT----ACAC
                    **  ****** ********** ********** ********** **    ****
                        ****** ********** ********** ********** **    ****
                        ****** ********** ********** ********** **

alignment position  251....... 261....... 271....... 281....... 291.......
P922003         164 -TGAAAACAG CACAAaaatc ataactgtac gGCTTGTTAA TTTTTACAAA
P922004         160 ACAAAAAAAG CACAAGTCac aatgattcat TGCCCGGGAA TGCTCATAAA
P922005         169 ATAAAAAATG CACAAGTCca atta------ TGCTCGTGAA TTCTCATAAA
                    ********** ********             ********** **********
                    ********** ********             ********** **********
                                                     ********* **********

alignment position  301....... 311....... 321....... 331....... 341.......
P922003         213 TAAATACACC CTCGTGAATC CAgaaCCTAG ATCAAGAACC AGAGCA----
P922004         210 TGAACATGCA CTAGGGAAAC CAT--CCTCC AGCAAGAACC AGATCAGTCA
P922005         213 TCAATATGCA TTCGGGAAct CAT--CCTCG AGCAAAAACC AGATCAATCA
                    ********** ********** ***  ***** ********** **********
                    ********** ********   ***  ***** ********** **********
                    ********** ********   ***  ***** ********** **********
                                          **   ***** ********** ******

alignment position  351....... 361....... 371....... 381....... 391.......
P922003         259 TTACCAGCAC ccagaaacct ccccgcgccc tcctccactg agcggcCCTT
P922004         258 TTGCCAGCAC gCGAGGCCTC CGGGCAT--- ---------- ------CTTC
P922005         261 TCGCCAGCAC tCGAGGCCTC CGGGAAT--- ---------- ------CTTC
                    **********  ********* *******                    ****
                    **********  ********* *******                    ****
                    **********  ********* *******                    ****

alignment position  401....... 411....... 421....... 431....... 441.......
P922003         309 TCAACCCTTA CAATGCCGGA GGGCGCAAtt cctgggtcct gtgaccttgg
P922004         289 CGTGCCCTGG AAAgGCCATG CCGCGAAAAG GG-------- ----------
P922005         292 TGTACCCTGG AAAAGCCAAG CCGCGAAAAG GG-------- ----------
                    ********** ********** ********** **
                    ********** *** ****** ********** **
                    ********** *** ****** ********** **

alignment position  451....... 461....... 471....... 481....... 491.......
P922003         359 tgaccgacGC CCGGGACAAG TCAGAAAGAC CCAAGGGCGT GGAAAACgCT
P922004         321 --------GA CCCGGGACTG TCAGCGAGGC TCACGACCAA GACAAAC-CT
```

```
P922005  324 --------GG CCCGGGACAG CCAGCAAAAC CCATGGCCAA GGAAAtC-CT
                     ** ********** ********** ********** ******* **
                     ** ********** *****                       * **
                     ** ********** *****                       * **
                                                               * **
                                                               * **
                                                                 **
                                                                 **

alignment position 501....... 511....... 521....... 531....... 541.......
P922003  409 CCTCTTACCG ATAAACTTGA AGCGACTCAT GACTGCGCAt ggaggacctc
P922004  362 GCCCTTACCC ACGAACTTGA AGAGACTCAT GACGGCGCGG CAGGCTCCTC
P922005  365 GCCCTTACCC ACGAACTTGA AGAGACTCAT GATAGCGCAG CAGACTCCTC
             ********** ********** ********** ********** **********
             ********** ********** ********** ********** **********
             ********** ********** ********** ********** **********
             ********** ********** ********** *********
             ********** ********** ********** *********
             ********** ********** ********** **  ****
             ********** ********** ********** **  ****
                ******* ********** ********** **
                ******* ********** ********** **

alignment position 551....... 561....... 571....... 581....... 591.......
P922003  459 cccagcggAA GCGGAAGTGG CCGCCGGCAA CTCCGCCCTT CCGGCTggcc
P922004  412 CCAGGGAAAA GCGGAAGTGG CTCAGAGCAC GCTCGTCCTT CCGGTTCCGC
P922005  415 CCAGGAAAAG GCGGAAATGG CGTAGAGCAC GCTCATTCTT CCGGCTCCGT
             ********** ********** ********** ********** **********
             ********** ********** *  ******* ********** **********
             ********** ********** *  ******* ********** **********
                     ** ********** *  ******* ********** ***

alignment position 601....... 611....... 621....... 631....... 641.......
P922003  509 ccgctcagtc acccgCAGCA GGCGTGCAGT TTCCCGGCTC TCCGCGCGGC
P922004  462 CTCACAA--- -----CCGCC CGCTTCCGGG TTCCCGGCAG CCCCCGCGGC
P922005  465 CTCGCAA--- -----CCGCC TGCTTCCGGG TTCCCGGCAG CCCCCGCGGC
             *******         ***** ********** ********** **********
             *******         *****  ********* ********** **********
             *******         *****  ********* ********** **********
                             *****  ********* ********** **********
                                    ********* ********** **********
                                    ********* ********** **********

alignment position 651....... 661....... 671....... 681....... 691.......
P922003  559 CGgg---GAA GGTCAGCgcc gta--ATGGC GTTCTtggcg tcgggacCCT
P922004  504 GGCCGCTGAA GGTGAGCCGG ACGCTATGGC GTTCTGCGCT CCCCCGGCCT
P922005  507 AGCCGCTGAA GGTGAGCCGG GCGCTATGGC GTTCTGTGCT CCCCCGGCCT
             ********** ********** ********** ********** **********
             ********** ********** ********** ********** **********
             ********** ********** ********** ********** **********
             ********** ********** ********** ********** **********
             ********** ********** ********** ********** **********
             **                                                ***
                                                               ***
                                                               ***
                                                               ***
                                                               ***

alignment position 701....... 711....... 721....... 731....... 741.......
P922003  604 ACCTGACCCA TCAGCAAAAG GTGTTGCGGC TTTATAAGCG GGCGCTACGC
P922004  554 ACCTGACCCA CCAGCAGAAG GTGCTGCGGC TGTATAAGCG CGCGCTGCGC
P922005  557 ACTTAACCCA CCGGCAGAAG GTGCTGCGGC TGTATAAGCG CGCGCTGCGC
             ********** ********** ********** ********** **********
             ********** ********** ********** ********** **********
             ********** ********** ********** ********** **********
             ********** ********** ********** ********** **********
             ********** ********** ********** ********** **********
             ********** ********** ********** ********** **********
             *    ***** ********** ********** ********** **********
             *    ***** ********** ********** ********** **********
```

```
                        *    ***** ********** ********** *******     *********
                        *    ***** ********** ********** *******     *********
alignment position      751....... 761....... 771....... 781....... 791.......
P922003             654 CACCTCGAGT CGTGGTGCGT CCAGAGGTAa gggatGGGGA CCCAGGACTC
P922004             604 CACCTCGAGT CATGGTGTAT CCACAGGTGG GGAGAGGGGA CCCGGGGCTC
P922005             607 CACCTCGAGT CATGGTGTGT CCACAGGTAG GGAGAggaga -CCTGGTCTC
                        ********** ********** ********** ********** **********
                        ********** ********** ********** ********** **********
                        ********** ********** ********** *****       *********
                        ********** ********** ********** *****       *********
                        ********** ********** ********** *****       *********
                        ********** ********** *********              *********
                        ********** ********** *********
                        ********** ********** *********
                        ********** ********** *******
                        ********** ********** *******
alignment position      801....... 811....... 821....... 831....... 841.......
P922003             704 GGGGAGGTGA CCCTCGGGGC CCCATGgagg tggagaggcc cctgggtaCC
P922004             654 GGGGACGGAA GCCGAGGGGC CCCGTGGG-- ---------- --------CC
P922005             656 GGGGACGGAA GCCGAGGGGC CCTGTGGGtt gat------- ----------
                        ********** ********** ********                    **
                        ********** ********** ********
                        ********** ********** ********
                        ********** ********** ********
                        ********** ********** ***
                        ********** ********** ***
alignment position      851....... 861....... 871....... 881....... 891.......
P922003             754 TGGAGGTTCA AGGACTTC-G GGAacggaat tggaaggtgg cctctcgctt
P922004             684 AGGCTGATCA AGGACTTggG GGACACAGTG AAGGGAGCCC GCCCTTGA--
P922005             689 ---CTGATCA AGGACTTA-G GGACACAGTG AAGGGAGCCC GCCCTTGA--
                        ********** *******  * ********** ********** ********
                               *** ******   * ********** ********** ********
                                            * ********** ********** ********
                                            * ********** ********** ********
                                            * ***
alignment position      901....... 911....... 921....... 931....... 941.......
P922003             803 cacagaatcc tatggtagAC CCGGGTTCAA ATCCAGGCTT CGCCA-----
P922004             732 ---------- --------AC CCGGGTCCCG ATGCAGTCCT CTCTG-----
P922005             733 ---------- --------AC CTGGGTCCTG ATGCAGTCCT CTCTcttgcc
                                          ** ********** ********** *****
                                          ** * ******** ********** ****
                                          ** *
                                          ** *
                                          ** *
alignment position      951....... 961....... 971....... 981....... 991.......
P922003             848 -CTTatggcc gggaGACCTT GTAGAGTTGC TTGTCGGGtc tgggcgtcag
P922004             759 -CTT------ ----GATCTG GCGGAGATGA TTGTGTGGGG CGca------
P922005             765 tCTT------ ----TATCTG GCAGAGATGA TTATGTGGAG CG--------
                         ***           ****** ********** ********** **
                         ***           ****** ********** ********** **
                         ***
alignment position      1001...... 1011...... 1021...... 1031...... 1041......
P922003             897 ctttttgttt agtaaaacgg acGCACGACC AGCGCAGAGG GTCGCAGGAa
P922004             792 ---------- ---------- -------ACC AGGACCCAGC GTAGAAAGTT
P922005             797 ---------- ---------- --GCGCGACC AGGACACAGC GTAGAAGGAT
                                                ******** ********** **********
                                                     *** ********** **********
                                                     *** ********** **********
                                                     *** ********** **********
                                                     *** ********** *********
alignment position      1051...... 1061...... 1071...... 1081...... 1091......
P922003             947 aattaagtga aataaaggAA AGTGCCTAAA ATTGTAATTG TCATGGGTCC
```

```
P922004         815 GCATGAAGAA ATAA----AA AGTACCTAGG ACTGTCATTG TCATGGATCT
P922005         825 GCATGAAGAA ATAA----AA AATACCCAGG ACTGTCATTG TCATGAATCT
                    ********** ****    ** ********** ********** **********
                    ********** ****    ** ********** ********** **********
                    ********** ****    ** *      *** ********** **********
                    ********** ****    ** *      *** ********** **********
                                       ** *      *** ********** **********
                                       ** *      *** ********** **********

alignment position  1101...... 1111...... 1121...... 1131...... 1141......
P922003         997 AGCGCTcgct caatcgt--- ---------- ---------- ----------
P922004         861 AAAGCTAACG TTTTTATgac tctgtcGTCC CTAACCTTAA CAGTAATTTG
P922005         871 AACGCTAATG TTTTTATaac tctt--GTCC CTAACCTTAA CAGTAATTTG
                    ********** *******          **** ********** **********
                    ********** *******          **** ********** **********
                    ********** *******          **** ********** **********
                    ********** *******          **** ********** **********
                    ******
                    ******

alignment position  1151...... 1161...... 1171...... 1181...... 1191......
P922003        1014 ---------- ---------- ---------- ---------- ----------
P922004         911 TCCATtATCT TTAGTTTGCA GAGTAACTCA GatatTAGTG ATTATTTTAG
P922005         919 TCCATgATCT TTAGTTTGCA GATTAACTGA GgaacTAGTG ATTATTTTAG
                    ***** **** ********** ********** *    ***** **********
                    ***** **** ********** ********** *    ***** **********
                    *****                                 ***** **********
                    *****

alignment position  1201...... 1211...... 1221...... 1231...... 1241
P922003        1014 ---------- ---------- ---------- ---------- -
P922004         961 CCTGATTTTA CAGATAAGCa ggctcattct attccacctc c
P922005         969 CCTCATTTTA TAGGTAAGCt caggattgac ttg------- -
                    ********** *********
                    ********** *********
                    ********** *********
```

Pairwise similarities:

For each pairwise alignment, the similarity (relative to the maximum similarity) and the number of identical nucleic acids (in % of shorter sequence) is given. Maximum values are underlined.

	P922004 (1001 bp)	P922005 (1001 bp)
P922003 (1013 bp)	0.188<br>38 %	0.161<br>38 %
P922004 (1001 bp)		1.000<br>80 %

Please note that the **similarity value 1.000** marks only the two **most similar** sequences, it does not necessarily mean that these sequences are identical.

Extract alignment region	
**Positions in alignment**	from: to:
**Output file**	dialign.seq

Extract aligned sequences

For comments, questions or bug reports, please contact support@genomatix.de

© Genomatix Software GmbH 1998-2005
- All rights reserved.

License Agreement

GEMS Launcher 4.1.1

Genomatix-Logo Logout Personal Messages GEMS-Logo
Logout Personal Messages

Genomatix_MainGEMSElDoradoGene2PromoterBiblioSphere
Frequently_asked_questionsYour_ResultsYour_SequencesYour_ProtocolHelp

GEMS Launcher Task: *DiAlign TF*: Multiple alignment plus TF sites
working on promoters.seq (3 seq.)

[Alignment] [Pairwise_similarities]

DiAlign professional TF Release 3.1 December 2004 **Tue Jun 7 17:18:57 2005**

Solution parameters:

**Sequence file:**	promoters.seq (3 seq.)
**Type of sequences:**	nucleotide sequences
**Threshold T:**	0.00
**'*' signs below alignment denote:**	diagonal similarity (max. similarity is represented by 10 '*' signs)
**Alignment output:**	complete alignment is shown<br># nucleic acids per line: 50
**TF output:**	common TF matches located in aligned regions (common to 3 (100.0 %) sequences)
**Family matches:**	yes
**MatInspector library:**	Matrix Family Library Version 5.0 (February 2005)
**Selected groups** (core/matrix sim):	• ALL user_defined.lib (0.75/Optimized)<br>• ALL vertebrates.lib (0.75/Optimized)

Aligned Sequences:

No.	Sequence Name	Sequence Description	Length
1	P914197	sym=NDUFB10|loc=Loc4716|taxid=9606|spec=Homo sapiens|chr=16|ctg=NT_037887|str=(+)|start=1949020|end=1950020|len=1001| tss=501| comm=NADH dehydrogenase (ubiquinone) 1 beta subcomplex, 10, 22kDa; (NM_004548/526/silver;)	1001 bp
2	P914198	sym=Ndufb10|loc=Loc68342|taxid=10090|spec=Mus musculus|chr=17|ctg=NT_039649|str=(-)|start=1254142|end=1255239| len=1098|tss=501,598| comm=NADH dehydrogenase (ubiquinone) 1 beta subcomplex, 10; (AK002517/598/gold;XM_128594/501/bronze;)	1098 bp
3	P914199	sym=na|loc=Loc287121|taxid=10116|spec=Rattus norvegicus|chr=10|ctg=NW_047334|str=(-)|start=802694|end=803694| len=1001|tss=501| comm=similar to NADH dehydrogenase (ubiquinone) 1 beta subcomplex, 10, 22kDa; NADH dehydrogenase (ubiquinone) 1 beta subcomplex, 10 (22kD, PDSW); (XM_213242/534/bronze;)	1001 bp

Alignment (DiAlign format):

Please note that only **upper-case** letters are considered to be **aligned**. For more information, please have a look at the user guide.

V$OAZF V$STAF V$NRF1 U$NRF1 V$PAX5 V$TEAF V$EBOX V$HESF V$HIFF V$PAX1

```
alignment position 1 . . . . . . . . .  1 1 . . . . . . . .  2 1 . . . . . . . .  3 1 . . . . . . . .  4 1 . . . . . . . .
P914197          1 c c t c c c c - - -  - - - - - - - - - -  - - - - - - - - - -  - - - - - - - - - -  - - - - - - - - - -
P914198          1 g c t g t t t c t t  a g c c c c t a a c  t c t g g c a g g a  g g t g a c c a g c  a c c t a g a t g a
P914199          1 - - - - - - - - - -  - - - - - - - - - -  - - - - - - - - - -  - - - - - - - - - -  - - - - - - - - - -
```

```
alignment position 51........ 61........ 71........ 81........ 91........
P914197        8 ---------- ---------- ---------- ---------- ----------
P914198       51 cacataagtg aggatttcca ggttgcaaaa ccaggtttta aacacataaa
P914199        1 ---------- ---------- ---------- ---------- ----------

alignment position 101....... 111....... 121....... 131....... 141.......
P914197        8 ---------- ---------- ---------- ---------- ----------
P914198      101 ctcaagagtt ttggcagtgc agtcctggac agTGAACAAT TCAGGACACA
P914199        1 ---------- ---------- ---------- --TGGGCAAC TCGGGGCACA
                                                    ******** **********

alignment position 151....... 161....... 171....... 181....... 191.......
P914197        8 ---------- ---------- ---------- ---------- ----------
P914198      151 AgtGCTCAGG ACCAGAACCA CcagaaGCAG GGCGAAGCAC TGTG--CCTA
P914199       19 A--GCTCAGG ACCAGAACCA Ctctt-GCAG AGCAAAGCAC TGTGtgCCTA
                 *  ******* ********** *     **** ********** ****  ****
                    ******* ********** *

alignment position 201....... 211....... 221....... 231....... 241.......
P914197        8 ---------- ---------- ---------- ---------- ---CAGACAG
P914198      199 GGGTGGCGGA CCCTTTtcct ccaaggattg catc------ -TTCAGAGAA
P914199       66 GGGTAGGAAA TCCTTTatgg acacgagaca agccctgcct aTTCAGAGAA
                 ********** ******                           *********
                                                             *********
                                                             *********
                                                             *********

alignment position 251....... 261....... 271....... 281....... 291.......
P914197       15 GCTCCCAAGT TAACTGGCAC CAGCCAGTCC atgaggggcc cttgtagaGA
P914198      242 GTCCCGAGGT TCACTGGGAC CCACCAGCCC Cag-GGAACC T-------AG
P914199      116 GTCCCGAGGT TTACAGGGAC CCACCAGCCC CcaaGGGACC T-------GA
                 ********** ********** ********** *   ****** *       **
                 ********** ********** ********** *   ****** *       **
                 ********** ********** ********** *
                 ********** ********** ********** *

alignment position 301....... 311....... 321....... 331....... 341.......
P914197       65 GCGGGGCCCA GAGAAGTctg tagggactag gcaatccagg aaagcttcct
P914198      284 GCAGAGTCTA GAGCAGTTGT TGACTggtg- ---------- ----------
P914199      159 GCAGAGTCTA GAGCAGTTGT TGACTtatat gtactcttt- ----------
                 ********** ********** *****
                 ********** ********** *****

alignment position 351....... 361....... 371....... 381....... 391.......
P914197      115 ggaggagtag ggtgtttgga atcgcgtgcT ATTTGTTCAC TTTGTCGtgt
P914198      313 ---------- ---------- ---------- ---------- ----------
P914199      198 ---------- ---------- ---------T ATTTATTTTT TTTTTCGgag
                                                * ********** *******

alignment position 401....... 411....... 421....... 431....... 441.......
P914197      165 gcgctctaac ggcctgtgtt ctccatcaca ---------- ----------
P914198      313 ---------- ---------- ---------- ---------- ----------
P914199      219 ctgggaaccg aacccagggc cttgccaagc gcactctacc actgagctaa

alignment position 451....... 461....... 471....... 481....... 491.......
P914197      195 ---------- ---------- ---------- ---------G TTTATCTTAT
P914198      313 ---------- --------TG TGTACTCTT- CCAAGCCATG TTCACCATAT
P914199      269 atccccaacc ccgactctTG TGTACTCTTg CCAAGCCATG TTCACtgcAT
                                    ** *********  ********** **********
                                                                     **
                                                                     **
                                                                     **
                                                                     **

alignment position 501....... 511....... 521....... 531....... 541.......
```

```
P914197      206 TCCCTGCTgc actcagcaca gtgcctggcg cgcagtggga accgaatgac
P914198      344 TCCCTGCTAT CTTAGCTGGT AGGTCAGGGC AAAGCATTtc cagccatt--
P914199      319 TCCCTGCTAT CTTAGCTGGT AGGTCAGGGC AAAGCATTtc cgtgcacttg
                 ********** ********** ********** ********
                 ********** ********** ********** ********
                 ********** ********** ********** ********
                 ********** ********** ********** ********
                 ********

alignment position 551....... 561....... 571....... 581....... 591.......
P914197      256 cccctcctat tccttatgtc agttgccttt tgtctgtttc tttcatcgta
P914198      392 -TGGTGATCT TTGTCCAACT ---------- ---------- ----------
P914199      369 gTGATGATCT CTGTCCAACT ---------- ---------- ----------
                  ********* **********

alignment position 601....... 611....... 621....... 631....... 641.......
P914197      306 agcaggaatc tgtccggttc gcggctgctt cccaggcgcc cagaccagcg
P914198      411 ---------- ---------- ---------- ---------- ----------
P914199      389 ---------- ---------- ---------- ---------- ----------

alignment position 651....... 661....... 671....... 681....... 691.......
P914197      356 accagcaccg cgtaggcgct cggcacAGAC CGAGAACGGA ----------
P914198      411 ---------- ---------- ------AAAa acaggatgcc tcagagcgct
P914199      389 ---------- ---------- ------AAAC CAGGTGCGCG ----------
                                             **** **********

alignment position 701....... 711....... 721....... 731....... 741.......
P914197      396 -GGCCCCACG CGGGGGCGCC Ctgcggcgag gcgggtcccg gccgcgcccg
P914198      435 gGGGACCACA AGGGGGTGCT CGAGA----- ---------- ----------
P914199      403 -GAAACCACA AGGGGGTGCC aGAGA----- ---------- ----------
                  ********* ********** *****
                                        ****
                                        ****

alignment position 751....... 761....... 771....... 781....... 791.......
P914197      445 gcggagccGG GGAACTACAA gTCCCATGGT GCATCGCGGC -G-CCAGCGC
P914198      460 --------GG CGAACCACTA -TCCCATGGT GCCTCGCGGC TGcCCACTAC
P914199      427 --------GG CGAACCACAA -TCCCATGGT ACCTCGCGGC TG-CCACTAC
                         ** **********  ********* ********** **  *******
                         ** **********  ********* ********** **  *******
                         ** **********  ********* ********** **
                                        ********* **********  *

alignment position 801....... 811....... 821....... 831....... 841.......
P914197      493 GCAGACGCAG CCGCCCTCGg cgtcctctg- ---------- ---------T
P914198      501 GCAGGCGCAG CTGtcacCTT TCTGCGCAGG CGTAGAGGTC CCGCGTCCTT
P914199      467 GCAGGCGCAG CTGCCGTCTT TCTGCGCAGG CGTAGAGGTC CCGCGTCCTT
                 ********** ********** ********** ********** **********
                 ********** ***    *** ********** ********** **********
                                   *** ********** ********** **********
                                   *** ********** ********** **********

alignment position 851....... 861....... 871....... 881....... 891.......
P914197      523 AGCGGGCgac ctaggccgcg ggacccggac ggaggtagag gccagggCAG
P914198      551 AGCGGGCC-- ---------- ---------- ---------- -------CAG
P914199      517 AGCaGGCC-- ---------- ---------- ---------- -------GAG
                 ********                                           ***
                 ***
                 ***
                 ***

alignment position 901....... 911....... 921....... 931....... 941.......
P914197      573 CGCGTCCGGG AGCGGAGtcc gcgcccgccg cc-------- ----------
P914198      562 CGGATCCGGA CGTGGAGCGA GTAGAAGGTC GGtggggaga GTTTGCTTAA
P914199      528 CCGACCCAGA CACAGAACGA GTGGAAGGTC CGcaggg--- GTTCGCTTAA
```

```
                     ********** ********** ********** **         *********
                                                                  *********
                                                                  *********

alignment position   951....... 961....... 971....... 981....... 991.......
P914197          605 GCCATGCCGG ACagcTGGGA CAAGGATGTG TACCCTGAGC CCCCGCGCCG
P914198          612 GCCATGCCAG ATTCTTGGGA CAAGGATGTG TACCCGGAGC CCCCGAGCCG
P914199          575 ACCATGCCGG ACTCTTGGGA CAAGGATGTG TACCCGGAGC CCCCGCGCCG
                     ********** ********** ********** ********** **********
                     ********** ********** ********** ********** **********
                     ********** ********** ********** ********** **********
                                     ***** ********** ********** **********
                                     ***** ********** ********** **********
                                     ***** ********** ********** **********
                                     ***** ********** ********** **********
                                     ***** ********** ********** **********
                                     ***** ********** ********** **********
                                                      ********** **********

alignment position   1001...... 1011...... 1021...... 1031...... 1041......
P914197          655 CACGCcggtg cag------- ---------- -CCCAATCCC ATCGTCTACA
P914198          662 CACGCCTGCT CCCTCGCCGC AGACCTCGCT CCCTAACCCC ATCACCTACT
P914199          625 CACGCCTGCT CCCTCGCCGC AGACCTCGAT CCCTAACCCC ATCACCTACT
                     ********** ********** ********** ********** **********
                     ********** ********** ********** ********** **********
                     ********** ********** ********** ********** **********
                     ********** ********** ********** ********** **********
                     *****                             ********* **********
                     *****                             ********* **********
                     *****
                     *****
                     *****
                     *****

alignment position   1051...... 1061...... 1071...... 1081...... 1091......
P914197          687 TGATGAAAGC GTTCGACCTC aTCGTGGACC GACCCGTGAC CCTCGTGAGA
P914198          712 TGACGAAGGC CTACGACCTC GTCGTGGACT GGCCCGTGAC CCTCGTGAGA
P914199          675 TGACGAAGGC CTACGACCTC GTCGTGGACT GGCCCGTGAC CCTCGTGAGA
                     ********** ********** ********** ********** **********
                     ********** ********** ********** ********** **********
                     ********** ********** ********** ********** **********
                     ********** ********** ********** ********** **********
                     ********** **********  ********* ********** **********
                     ********** **********  ********* ********** **********
                                            ********* ********** **********
                                            ********* ********** **********
                                            ********* ********** **********

alignment position   1101...... 1111...... 1121...... 1131...... 1141......
P914197          737 GGTACgaagc cccagcccgg ggctccctcg ccggcctctg ggga--CCCC
P914198          762 GGTACAGGGT CTAGAATTTC GCCCGCTTAG TGGAGATCCC cccTACCCCG
P914199          725 GGTACAGGGT CTAGAATTGC TCCTGCTTAG TGGAGATCCC gg-TACCCCA
                     ********** ********** ********** **********     ******
                     ********** ********** ********** **********     ******
                     ********** ********** ********** **********     ******
                     *****
                     *****
                     *****
                     *****
                     *****

alignment position   1151...... 1161...... 1171...... 1181...... 1191......
P914197          785 TGGATCCCAC ACCCtgcctg gatcctccaa tgcctccggg gtcccgtctg
P914198          812 CGGATCCCAC ATCCAGTTTC CGCCC----- ---------- ----------
P914199          774 CGGATCCCAC AGCCAGGTTC CGCCC----- ---------- ----------
                     ********** ********** *****
                     ********** ********** *****
                     ********** ********** *****

alignment position   1201...... 1211...... 1221...... 1231...... 1241......
```

```
P914197        835 CCTGAGACCG CCCCCCGCTG CACCCCGggg acaa-CTCCC CACCCCCGGA
P914198        837 CCTGAAACCA CCACCCGCTG CATGCCGCTG TCCCGCACCC TGAATC----
P914199        799 CCTagaa--- -------CTG CATGCCGCTG TCCTGCACCC AGACTCCGGA
                   ********** ********** ********** ********** **********
                   ***               *** ********** ********** ******
                   ***               *** *******

alignment position 1251...... 1261...... 1271...... 1281...... 1291......
P914197        884 GACCtccgag ctccgtcgcc tcctttggcc tcccactg-- ----------
P914198        883 GTCTCAATCT CC-----CCC AGTGCATCTA AGATACCCTC TACCTTTTAT
P914199        839 GTCCAAATCT CCttaatCCC AGTGCATCTA GGACTCTTTC TACCTTATAT
                   ********** **     *** ********** ********** **********
                                     *** ********** ********** **********

alignment position 1301...... 1311...... 1321...... 1331...... 1341......
P914197        922 ---------- ---------- --CACCCCGG A--------- --CCTCACCT
P914198        928 CTGAGAcgcC CCGGTGCTGT CACTCCagat cacttcccac gcCCTCCCGT
P914199        889 CCGAGAaa-C CCGGAGCTGT CACCCCCAGG A--------- --CCTCCCAT
                   ******   * ********** ********** *            ********
                   ******                                         ********
                                                                  ********

alignment position 1351...... 1361...... 1371...... 1381...... 1391......
P914197        939 Cccaggatcc cttactctcc cCTGCACCCC GGGATGCCCC GCTTCTTCCC
P914198        978 CTACCCTG-- ---------- -CTGCACCGC AGGATAGTCC ACTTCTTTCC
P914199        927 CTACCCTG-- ---------- -CTGCACCGC GGGATGGTCC ACTTCTTCCC
                   ********               ********* ********** **********
                   ********               ********* ********** **********
                   ********               ********* ********** **********
                                          ********* ********** *****   **
                                          ********* ********** *****   **
                                          ********* ********** *****

alignment position 1401...... 1411...... 1421...... 1431...... 1441......
P914197        989 AGGACTcttc cct------- ---------- ---------- ----------
P914198       1015 GGGACTACCC TGTCTATAAT CCTTTCTGTC TTCAGCAgaa ccccaactaa
P914199        964 AGGACTAACC TGTCTATAAT TCTTTCTGCC TTCAGCAa-- ----------
                   ********** ********** ********** *******
                   ********** ********** ********** *******
                   ********** ********** ********** *******
                   ******
                   ******

alignment position 1451...... 1461...... 1471...... 1481
P914197       1002 ---------- ---------- ---------- ----
P914198       1065 cctttcgtcg accccggtcg ttcttttaag cggt
P914199       1002 ---------- ---------- ---------- ----
```

Pairwise similarities:

For each pairwise alignment, the similarity (relative to the maximum similarity) and the number of identical nucleic acids (in % of shorter sequence) is given. Maximum values are underlined.

	P914198 (1098 bp)	P914199 (1001 bp)
**P914197 (1001 bp)**	0.204<br>32 %	0.221<br>31 %
**P914198 (1098 bp)**		<u>1.000</u><br><u>72 %</u>

Please note that the **similarity value 1.000** marks only the two **most similar** sequences, it does not necessarily mean that these sequences are identical.

Extract alignment region	
**Positions in alignment**	from: to:
**Output file**	dialign.seq

Extract aligned sequences

For comments, questions or bug reports, please contact support@genomatix.de

© Genomatix Software GmbH 1998-2005
- All rights reserved. License Agreement GEMS Launcher 4.1.1

Genomatix-Logo | Logout | Personal | Messages | GEMS-Logo
Logout | Personal | Messages

Genomatix MainGEMSElDoradoGene2PromoterBiblioSphere
Frequently asked questionsYour ResultsYour SequencesYour ProtocolHelp

GEMS Launcher Task: *DiAlign TF*: Multiple alignment plus TF sites
working on promoters.seq (3 seq.)

[Alignment] [Pairwise similarities]

DiAlign professional TF Release 3.1 December 2004 **Tue Jun 7 17:53:39 2005**

Solution parameters:

**Sequence file:**	promoters.seq (3 seq.)
**Type of sequences:**	nucleotide sequences
**Threshold T:**	0.00
**'*' signs below alignment denote:**	diagonal similarity (max. similarity is represented by 10 '*' signs)
**Alignment output:**	complete alignment is shown<br># nucleic acids per line: 50
**TF output:**	common TF matches located in aligned regions (common to 3 (100.0 %) sequences)
**Family matches:**	yes
**MatInspector library:**	Matrix Family Library Version 5.0 (February 2005)
**Selected groups** (core/matrix sim):	• ALL user_defined.lib (0.75/Optimized)<br>• ALL vertebrates.lib (0.75/Optimized)

Aligned Sequences:

No.	Sequence Name	Sequence Description	Length
1	P961889	sym=P17.3|loc=Loc54539|taxid=9606|spec=Homo sapiens|chr=X|ctg=NT_079573|str=(-)|start=9853400|end=9854746|len=1347| tss=501,847| comm=neuronal protein 17.3; (AK000501/847/gold;NM_019056/501/silver;)	1347 bp
2	P961890	sym=Np15|loc=Loc104130|taxid=10090|spec=Mus musculus|chr=X|ctg=NT_039700|str=(-)|start=13827557|end=13828828|len=1272| tss=501,772| comm=nuclear protein 15.6; (AK028121/815/gold;NM_019435/544/silver;)	1272 bp
3	P961891	sym=na|loc=Loc299310|taxid=10116|spec=Rattus norvegicus|chr=X|ctg=NW_048034|str=(+)|start=486187|end=487187| len=1001|tss=501| comm=similar to neuronal protein 15.6; (XM_216785/541/bronze;)	1001 bp

Alignment (DiAlign format):

Please note that only **upper-case** letters are considered to be **aligned**. For more information, please have a look at the user guide.

V$TALE V$AIRE V$CDEF V$PAX3 V$ECAT V$PCAT V$COMP V$PBXC V$GLIF V$TBPF V$GKLF V$LEFF
V$IRFF V$SNAP V$SP1F V$E2FF V$ZBPF V$EGRF V$CREB

```
alignment
position        1 . . . . . . . . .   1 1 . . . . . . . .   2 1 . . . . . . . .   3 1 . . . . . . . .   4 1 . . . . . . . .
P961889      1  g t c c c c g c c c   c g g g c c c c t t   a c c t c c g c c c   c c a c g a c c a g   c g t c g a C G C T
P961890      1  - - - - - - - - - -   - - - - - - - - - -   - - - - - - - - - -   - - - - - - - - - -   - - - - - - - - - -
P961891      1  g - - - - - - - - -   - - - - - - - - - -   - - - - - - - - - -   - - - - - - - - - -   - - - - - - C G C T
```

```
                                                                 * * * *
                                                                 * * * *
                                                                 * * * *
alignment
position         51........  61........  71........  81........  91........

P961889     51   CACCTTCTCG  GCCCGGGCTG  TCAAGGAAGG  GCTCCCAGAC  ATCACCTCTG
P961890      1   CACCTTCTCG  GCCCGGGCTG  TCAAGGGAGG  GCTCCCAGGC  ATCACCTCCG
P961891      6   CACCTTCTCG  GCCCGGGCTG  TCAAGGGAGG  GCTCCCAGGC  ATCACCTCCG
                 **********  **********  **********  **********  **********
                 **********  **********  **********  **********  **********
                 **********  **********  **********  **********  **********
                 **********  **********  **********  **********  **********
                 **********  **********  **********  **********  **********
                 **********  **********  **********  **********  **********
                 **********  **********  **********  **********  **********
                 **********  **********  **********  ******** *  **********
                 **********  **********  ******  ***  ********
                 **********  **********  ******  ***  ********

alignment
position         101.......  111.......  121.......  131.......  141.......

P961889    101   CCCGCCGCCA  CCTCCTCCAa  ctctcccagc  tcagcc-GGA  GCCGGAGCGC
P961890     51   CCCGCCACCA  CCTCCTTCAG  AACTGTTAGT  tcaaatGGGA  GCCAATGCAT
P961891     56   CCCGCCACTA  CCTCCTTCAG  AACTGTTAGT  tcgac-GGGA  GCCAATGCAT
                 **********  **********  **********        ****  **********
                 **********  **********  **********        ****  **********
                 **********  **********  **********        ****  **********
                 **********  **********  **********        ****  **********
                 **********  *********                      ***  **********
                 **********  *********                      ***  **********
                 **********  ******                         ***  **********
                 **********  ******

alignment
position         151.......  161.......  171.......  181.......  191.......

P961889    150   GCAACAACCA  AGTCCGAGAC  TGGAGGC---  ----AGATCG  GGGGGAGGGG
P961890    101   GCAACTACCA  AGTCCAAGAC  TGGgaGCAAA  AAGGAGGAAA  GGGGGAGGGG
P961891    105   GCAACTACCA  AGTCCAAGAC  TGGAGGCAAA  AGGGAGGACA  GGGGGAAGGG
                 **********  **********  **********  **********  **********
                 **********  **********  **********  **********  **********
                 **********  **********  ***  **         ******  **********
                 **********  **********  ***  **
                 **********  **********  ***
                 **********  **********  ***
                 **********  **********  ***

alignment
position         201.......  211.......  221.......  231.......  241.......

P961889    193   AGAAGCGCCA  AgCGCGCTGC  CCTCCCAGGG  AAACTCACTG  CCGCCTACTC
P961890    151   AGACGCGACC  gACGCGTTGC  CCTCCCAAGG  AAACTCGGTG  CCACCTCCTC
P961891    155   AGACGCGCCC  AACGCGTTGC  CCTCCCAAGG  AAACTCAGTG  CCGCCTCCTC
                 **********  **********  **********  **********  **********
                 **********   *********  **********  **********  **********
                 **********   *********  **********  **********  **********
                              *********  **********  **********  **********
                               ********  **********  **********  **********
                               ********  **********  **********  ****** ***
                               ********  **********  **********  ****** ***
                                                                        ***
                                                                        ***

alignment
position         251.......  261.......  271.......  281.......  291.......

P961889    243   CCAGCCGGCC  ACAGTCACCA  GCTCAAAATG  GCGACGAcga  GAAGGGAGTC
P961890    201   CtAGCCGGCC  ACAGTCACCA  ACACAAAATG  GCGACGA---  GAAGAGAGCC
P961891    205   CCAGCCGGCC  ACAGTCACCA  ACACAAAATG  GCGACGA---  GAAGAGAGCC
```

```
                 ********** ********** ********** *******    **********
                 ********** ********** ********** *******    **********
                 ********** ********** ********** *******    **********
                 ********** ********** ********** *******    **********
                 *  ******* ********** ********** *******    **********
                 *  ******* ********** ********** *******    **********
                 *  ******* ********** ********** *******    **********
                 *  ******* ********** ********** *******    **********
                 *  ******* ********** ********** *******    **********
                    ******* ********** ********** *******    **********

alignment
position         301....... 311....... 321....... 331....... 341.......

P961889      293 GGCGCTCCGA CCACCATCCA CCTACTAAGG aAGCGCGCTC TGGCCGGCCC
P961890      248 GGCGATCCGA CCACCATCCA CCTACTAAGG GAGCGCGCTC TGGCCGGCCT
P961891      252 GGCGCTCCGA CCACCATCCA CCTACTAAGG GAGCGCGCTC TGGCCGGCCT
                 ********** ********** ********** ********** **********
                 ********** ********** ********** ********** **********
                 ********** ********** ********** ********** **********
                 ********** ********** ********** ********** **********
                 ********** ********** **********  ********* **********
                 ********** ********** **********  ********* **********
                 ********** ********** **********  ********* **********
                 ********** ********** **********
                 ********** ********** **********
                 ********** ********** **********

alignment
position         351....... 361....... 371....... 381....... 391.......

P961889      343 CGGCGATTGG TCACCGcCCG CTAGGGGACA GCCCTGGCCT CCTCTGATTG
P961890      298 TGACGATTGG TTGCCGGTCA CCATtGGCTG GCCCTGGCAT TCTCTGATTG
P961891      302 TGACGATTGG TTGCCGGCCA CTATGGGCTG GCCCTGGCAT TCTCTGATTG
                 ********** ********** ********** ********** **********
                 ********** ********** ********** ********** **********
                 ********** ********** **** ***** ********** **********
                 ********** ********** **** ***** ********** **********
                 ********** ******  *** **** ***** ********** **********
                 ********** ******          ***** ********** **********
                 ********** ******                ********** **********

alignment
position         401....... 411....... 421....... 431....... 441.......

P961889      393 GCAAGCgctg GCCACCTCCC CACACCCCTT GCGAACGctc ccctagtgga
P961890      348 GCAAGCATAT GCTACCTCCC CATTCCCTGT GAGAACGGCC CAGGAGTCCa
P961891      352 GCAAGCATAT GCTACCTCCC CATTCCCCGT GAGAATGGCC CAGGAGTCCt
                 ********** ********** ********** ********** *********
                 ********** ********** ********** ********** *********
                 ********** ********** ********** ********** *********
                 ********** ********** ********** *******
                 ********** *****
                 ******     *****
                 ******

alignment
position         451....... 461....... 471....... 481....... 491.......

P961889      443 g-AAAAGGAG TAGCTATTAG Ccaattcggc agggcCC-GC TTTTTAGAAG
P961890      398 GAAAAAAGAG TAGCTATTGG CTACCCCTAA GAGG-CCcGC TCTTTAGAAG
P961891      402 GAAAAAAGAG TAGCTATTGG CTATCCCTAT GAGG-CC-GC TTTTTAGAAG
                 ********** ********** ********** ****  ** ** **********
                 ********** ********** ********** ****  ** ** **********
                 ********** ********** ********** ****  ** ** **********
                   ******** ********** *               ** ** **********
                                                       ** ** **********
                                                       ** ** **********
                                                          ** **********
                                                          ** **********

alignment
position         501....... 511....... 521....... 531....... 541.......

P961889      491 CTTGATTTCC TTTGAAGATG AAAGACTAGC GGAAGCTCTG CCTCTTTCCC
```

```
P961890    447 CTTGCTGTCC TTTGAAGTTG AAAGACAACT AGAAGCTCCA CCTCTTTCCC
P961891    450 CTTGCTGTCC TTTGAAGTTG AAAGACAAgT AGAAGCTCCA CCTCTTTCCC
               ********** ********** ********** ********** **********
               ********** ********** ********** ********** **********
               ********** ********** ******** * ********** **********
               ********** ********** ******** * ********** **********
               ********** ********** ********    ********* **********
               ********** ********** ******
               ********** ********** ******
               ********** ******* ** ******

alignment
position       551....... 561....... 571....... 581....... 591.......

P961889    541 CAGTGGGCGa gggaactcgg ggcgattggc tgggaactgt atccacccaa
P961890    497 TACAGGGCca TCTAG----- ---------- ---------- ----------
P961891    500 TACAGGGCGg TCTAG----- ---------- ---------- ----------
               *********  *****
               ********   *****
               ********   *****
               ********   *****
               ********

alignment
position       601....... 611....... 621....... 631....... 641.......

P961889    591 ATGTCACCGA TTTCTTCCTA TGCAGGAAAT GAGCAGACCc aTCAATAAGA
P961890    512 ATGTCACCGC TTTCTTCTTA CGAAGAAAAT GAACAGACCT CTCAAAAACA
P961891    515 ATGTCACCGC TTTCTTCTTA TGAAGAAAAT GAACAGACCT CTTAAAAACA
               ********** ********** ********** ********** **********
               ********** ********** ********** ********** **********
               ********** ********** ********** *********
               ********** ********** ********** *********
               ********** ********** ********** *********
               ********** ********** ********** *********
               ********** ********** ********** *****
               ********** ********** ********** *****

alignment
position       651....... 661....... 671....... 681....... 691.......

P961889    641 AATTTCTCag cctggccgaa aatggttggc cccacgaagc cacgacaact
P961890    562 ACTCTCTCCC AGACTAACAG AAAtgATAAC GTTGATTCGG TAGCGACACG
P961891    565 AATTTCTTCC AGCCTAACAG AAAcaATAAA GCTAATTCGA TTGCGACGCG
               ********** ********** ***  ***** ********** **********
               ********** ********** ***  ***** ********** **********

alignment
position       701....... 711....... 721....... 731....... 741.......

P961889    691 -----GGAGG CAAAgagggt tgcTCAACGC CCCGCCTCAT TGGAAAACCA
P961890    612 GCGTGGGAGG CAAAATAGAG ---TCCGCAC CTCGCCGAAC TCGTAAACAA
P961891    615 GCGTGGAAGG tAAAATAGAG ---TCCACAC CTCGCCACAC TCGAAAACAA
               ********** **********    ******* ********** **********
               **********  *********    ******* ********** **********
                           *********    ******* ********** **********
                                        ******* ********** **********

alignment
position       751....... 761....... 771....... 781....... 791.......

P961889    736 AATCAGatct GGGACCTATA TAGCGTGGCG GAGGCGGGGC GAtgattgTC
P961890    659 ACTTAGgCCG GGGATCTATT TAGCAAGGTA AGGGCGGAGC GACAAAAaTC
P961891    662 ACTTAaaCCC GGGATCTATT TAGCGAGGTA AGGGCGGAGT GACAAAAtTC
               ****** *** ********** ********** ********** ******* **
               ****   *** ********** ********** ********** ******* **
               ****   *** ********** ********** ********** ******* **
               ****       ********** ********** ********** **      **
                                                                   **

alignment
position       801....... 811....... 821....... 831....... 841.......

P961889    786 GCGCTCGCAC CCACTGCAGC TGCGCACAgt cgcatttctt t------CCC
P961890    709 GCGCTGGGAT CAACCGCAGC TGCGCACATT GTATTTCCgt CTCCTCCCCC
```

```
P961891      712  GCGCTGGGAT CAACCGCAGT TGCGCACTTC GTATTTCCgg CTCCCCACCC
                  ********** ********** ********** ********   **********
                  ********** ********** ********** ********   **********
                  ********** ********** ********** ********
                  ********** ********** ********
                  ********** ********** *******

alignment
position          851....... 861....... 871....... 881....... 891.......
P961889      830  CGCCCCtga- -----GACCC TGCAGCACCA TCTGTCATGG CGGCTGGGCT
P961890      759  CCCCCCGCCC CCCAGGACTC CGCAGTACAA GCTGTCATGG CGGCCCGACT
P961891      762  CGCCCCCACC CCCTGGACTC CTCAGTACAA GCGGTCATGG CGGCCCGGCT
                  ********** ********** ********** ********** **********
                  ********** ********** ********** ********** **********
                                  ***** ********** ********** **********
                                  ***** ********** ********** **********
                                                 * ********** **********

alignment
position          901....... 911....... 921....... 931....... 941.......
P961889      874  GTTtgGTTTG AGCGCTCGCC GTCTTTTGGC GGCAGCGGCG ACGCGAGGGC
P961890      809  GTTAAGCTTG TATGGTCGCT GCCTGTCAGC AGCGGGGGCG ATGAGAGGGC
P961891      812  GTTAAGCTTG TATGCTCGCC GCCTGTCAGT AGCGGCGGCG ATACGAGAGG
                  ********** ********** ********** ********** **********
                  ********** *********  ********** ********** **********
                  ********** *********  ********** ********** **********
                  ***  ***** *********                   **** **********
                  ***                                    **** *   ******
                                                                  ******

alignment
position          951....... 961....... 971....... 981....... 991.......
P961889      924  TCCCGGCCGC CCGCGTCCGC TGGGAATCTa gcttcTCCAG GACTGTGGTC
P961890      859  TCCCGGCTGC TCGCGTTCGC TGGGAGTCC- -----TCCAG GGCTGTAATC
P961891      862  TCCCGGCTGC CCGTGTTCGC TGGGAATCT- -----TCCAG GGCTGTAATC
                  ********** ********** *********       ***** **********
                  ********** ********** *********       ***** **********
                  ********** ********** *********       ***** **********
                  ********** ********** *********       ***** **********
                  **********  ********* *********       ***** **********
                  **********  ********* *****           ***** **********
                              ********* *****

alignment
position          1001...... 1011...... 1021...... 1031...... 1041......
P961889      974  GCCCCGTCCG CTGTGGCGGG AAAGCGGCCC CCAGAACCGA CCACACCGTG
P961890      903  GCCCCATCCG GTGTGGAGAA AAAGCGGCAA CGAGAACCGA CCATGCAGTG
P961891      906  GCCCCGTCCG GTGTGGAGAG AAAGCGGCAA AGAGAACCGA CCATGgtaTG
                  ********** ********** ********** ********** **********
                  ********** ********** ********** ********** **********
                  ********** ********** ********** ********** *****   **
                  *****  *** ********** ********** ********** *****   **
                  *****  *** ********** ********** ********** *****   **
                  *****  *** ******     *****                         **
                         *** ******     *****                         **
                                                                      **

alignment
position          1051...... 1061...... 1071...... 1081...... 1091......
P961889     1024  GCAAGAGGAC CCAGAACCCG AGGACGAAAA CTTGTATGAG AAGGTGAGAG
P961890      953  GCAGGAGGAC CCAGAACCCG AGGACGAAAA CGTCTACGCG AAGGTGAGAG
P961891      956  GCAGGAGGAC CCAGAACCCG AGGACGAAAA CCTCTACGCG AAGGTG----
                  ********** ********** ********** ********** **********
                  ********** ********** ********** ********** **********
                  ********** ********** ********** ********** **********
                  ********** ********** ********** ********
                  ********** ********** ********** ********
                  ********** ********** ********** ********
                  ********** ********** ********** ********
                  ********** ********** ********** *
```

```
                        **********  **********  *
alignment position      1101......  1111......  1121......  1131......  1141......
P961889   1074          gcgggggggg  gggggggggt  agtggggacg  gaaccccgaa  ggggtcagtc
P961890   1003          ggagggg---  ----------  ----------  ----------  ----------
P961891   1002          ----------  ----------  ----------  ----------  ----------

alignment position      1151......  1161......  1171......  1181......  1191......
P961889   1124          cgggatcGGC  AGATGGGGCG  CTGTAGAGGA  CGTTCTtagg  cagta-----
P961890   1010          -------GGA  AAACGGGACG  CTGTAGGGGC  CAATCTggac  cagggaaacc
P961891   1002          ----------  ----------  ----------  ----------  ----------
                               ***  **********  **********  ******

alignment position      1201......  1211......  1221......  1231......  1241......
P961889   1169          ----------  ----------  -------GAG  GACTGCGCGG  CCGGCGAAAG
P961890   1053          agtgtcaaag  cggacattct  ccggcccGAG  ATTTGAGCAG  TGGACAAAAT
P961891   1002          ----------  ----------  ----------  ----------  ----------
                                                       ***  **********  **********

alignment position      1251......  1261......  1271......  1281......  1291......
P961889   1192          CCTAGAAGAC  TtgggAATTT  TGCCTACCTT  GTGTTCCTCC  TTTGTctctc
P961890   1103          CTTCGAATAT  TcagaAATTT  TGCCTAGCTT  GTTTTTCTCT  TTCGTtgcct
P961891   1002          ----------  ----------  ----------  ----------  ----------
                        **********  *    *****  **********  **********  *****

alignment position      1301......  1311......  1321......  1331......  1341......
P961889   1242          ggtatcttcg  ttccagggcc  agaggcagga  ttggttttat  TTTGCAGACA
P961890   1153          ta--------  ----------  ----------  ----------  TTTGCAGTCA
P961891   1002          ----------  ----------  ----------  ----------  ----------
                                                                        **********

alignment position      1351......  1361......  1371......  1381......  1391......
P961889   1292          GAAATCACAC  ctcAAGTGGA  CAGCGCTCAA  AACACGTTAG  AAGCAGTATA
P961890   1165          GAAATCACAC  tgaAAGAGGG  CAGCACCCAA  AGCATGTTAG  AAACTTTATA
P961891   1002          ----------  ----------  ----------  ----------  ----------
                        **********     *******  **********  **********  **********

alignment position      1401......  1411......  1421......  1431......  1441......
P961889   1342          GTacaa----  ----------  ----------  ----------  ----------
P961890   1215          ATtagggtaa  tactaaactc  taaggtaaca  cgtgcataat  gccaggctgt
P961891   1002          ----------  ----------  ----------  ----------  ----------
                        **

alignment position      1451....
P961889   1348          --------
P961890   1265          gctagaca
P961891   1002          --------
```

Pairwise similarities:

For each pairwise alignment, the similarity (relative to the maximum similarity) and the number of identical nucleic acids (in % of shorter sequence) is given. Maximum values are underlined.

	P961890 (1272 bp)	P961891 (1001 bp)

**P961889 (1347 bp)**	0.488 59 %	0.481 67 %
**P961890 (1272 bp)**		1.000 89 %

Please note that the **similarity value 1.000** marks only the two **most similar** sequences, it does not necessarily mean that these sequences are identical.

**Extract alignment region**	
**Positions in alignment**	from: to:
**Output file**	dialign.seq

Extract aligned sequences

For comments, questions or bug reports, please contact support@genomatix.de

© Genomatix Software GmbH 1998-2005 - All rights reserved.

License Agreement

GEMS Launcher 4.1.1

Logout Personal Messages

GEMS Launcher Task: *DiAlign TF*: Multiple alignment plus TF sites
working on C1-NDUFC1 (3 seq.)

[Alignment] [Pairwise similarities]

DiAlign professional TF Release 3.1 December 2004 **Tue Jun 7 19:15:08 2005**

Solution parameters:

**Sequence file:**	C1-NDUFC1 (3 seq.)
**Type of sequences:**	nucleotide sequences
**Threshold T:**	0.00
**'*' signs below alignment denote**:	diagonal similarity (max. similarity is represented by 10 '*' signs)
**Alignment output:**	complete alignment is shown<br># nucleic acids per line: 50
**TF output:**	common TF matches located in aligned regions (common to 3 (100.0 %) sequences)
**Family matches:**	yes
**MatInspector library:**	Matrix Family Library Version 5.0 (February 2005)
**Selected groups** (core/matrix sim):	• ALL user_defined.lib (0.75/Optimized)<br>• ALL vertebrates.lib (0.75/Optimized)

Aligned Sequences:

No.	Sequence Name	Sequence Description	Length
1	P855801	P855801 sym=NDUFC1|loc=Loc4717|taxid=9606|spec=Homo sapiens|chr=4|ctg=NT_016354|str=(-)|start=64711588|end=64712588|len=1001|tss=501|comm=NADH dehydrogenase (ubiquinone) 1, subcomplex unknown, 1, 6kDa; (NM_002494/501/bronze;)	1001 bp
2	mNDUFC1	mNDUFC1 m6_dna range=chr3:51040462-51041402 5'pad=0 3'pad=0 revComp=TRUE strand=? repeatMasking=none	941 bp
3	rNDUFC1	rNDUFC1 n3_dna range=chr2:140251319-140252373 5'pad=0 3'pad=0 revComp=TRUE strand=? repeatMasking=none	1055 bp

Alignment (DiAlign format):

Please note that only **upper-case** letters are considered to be **aligned**. For more information, please have a look at the user guide.

V$MOKF V$NRSF V$RORA V$EREF U$EREF V$LEFF

```
alignment position 1.........  11........  21........  31........  41........
P855801          1 aaa------- ---------- ---------- ---------- ----------
mNDUFC1          1 caccccgaTG AATAACAGAA CCTACCTCAT AGGACTGAGA GGAATCaatg
rNDUFC1          1 ctccccggTG AATAACAGAA CCTATCTCAC AGGACTGAGA GGAATCtatg
                           ** ********** ********** ********** ******
                           ** ********** ********** ********** ******
                           ** ********** ********** ********** ******
                           ** ********** ********** ********** ******

alignment position 51........  61........  71........  81........  91........
```

```
P855801        4 - - - - - - - - - -  - - - - - - - - - -  - - - - - - - - - -  - - - - - - - - - -  - - - - - - A T A T
mNDUFC1       51 t g a t t T T G A G  T A T A A G T G G A  T T T T A G G A A C  T G T T A A G A G C  C T T T G C A A A T
rNDUFC1       51 t g g t c T T G A G  T A T A A G T G G A  T T T T A G G A A C  T G T T A A G A G G  C T T T G C A A A T
                           * * * * *  * * * * * * * * * *  * * * * * * * * * *  * * * * * * * * * *  * * * * * * * * * *
                           * * * * *  * * * * * * * * * *  * * * * * * * * * *  * * * * * * * * * *  * * * * * * * * * *
                           * * * * *  * * * * * * * * * *  * * * * * * * * * *  * * * * * * * * * *  * * * * * * * * * *
                           * * * * *  * * * * * * * * * *  * * * * * * * * * *  * * * * * * * * * *  * * * * *
                           * * * * *  * * * * * * * * * *  * * * * * * * * * *  * * * * * * * * * *  * * * * *

alignment position 1 0 1 . . . . . . .  1 1 1 . . . . . . .  1 2 1 . . . . . . .  1 3 1 . . . . . . .  1 4 1 . . . . . . .
P855801        8 A A T A T G T C A C  C T T G G A T G A G  T A G C T g t c t t  c t t t a a g c t t  - - - - - - - - - -
mNDUFC1      101 A C A A A G T T C C  C T T C G T C A A C  A A G T T T A T C T  T T G a - - - - - -  - - - - - - - - - -
rNDUFC1      101 A C A A A G T T C C  C T T A G T C G A C  A A G T T T G T C T  T T G g g c a c a c  c t c t t t t c t t
                 * * * * * * * * * *  * * * * * * * * * *  * * * * * * * * * *  * * *
                 * * * * * * * * * *  * * * * * * * * * *  * * * * * * * * * *  * * *
                 * * * * * * * * * *  * * * * * * * * * *  * * * * * * * * * *  * * *

alignment position 1 5 1 . . . . . . .  1 6 1 . . . . . . .  1 7 1 . . . . . . .  1 8 1 . . . . . . .  1 9 1 . . . . . . .
P855801       48 - - - - - - - - - -  - - - - - - - - - -  - - - - - - - - - -  - - - - - - - - - -  - - - - - - - - - -
mNDUFC1      135 - - - - - - - - - -  - - - - - - - - - -  - - - - - - - - - -  - - - - - - - - - -  - - - - - - - - - -
rNDUFC1      151 t t t t t t t t t t  t t t t t t t t t g  g t t c t t t t t t  t c g g a g c t g g  g g a c c g a a c c

alignment position 2 0 1 . . . . . . .  2 1 1 . . . . . . .  2 2 1 . . . . . . .  2 3 1 . . . . . . .  2 4 1 . . . . . . .
P855801       48 - - - - - - - - - -  - - - - - - - - - -  - - - - - - - - - -  - - - - - - - - - -  - - - - - - - - - -
mNDUFC1      135 - - - - - - - - - -  - - - - - - - - - -  - - - - - - - - - -  - - - - - - - - - -  - - - - - - - - - -
rNDUFC1      201 c a g g g c c t t g  c g c t t c c t a g  g t a a g c g c t c  t a c c a c t g a g  c t a a a t c c c c

alignment position 2 5 1 . . . . . . .  2 6 1 . . . . . . .  2 7 1 . . . . . . .  2 8 1 . . . . . . .  2 9 1 . . . . . . .
P855801       48 - - - - - - - - - -  - - C A T C T G T A  A A T T G A G G C T  T A T A A C C A C G  G C A T g a t a t g
mNDUFC1      135 - - - - - - - - - G  C A C A T C T C T T  A G T A G A T G C T  A A T A A T C A C A  G C A C a t c c t a
rNDUFC1      251 a g c c c c t g g G  C A C A C C T C T T  A A T T G T T G C T  A A T A A T C A C A  - - - - - - - - - -
                                   *  * * * * * * * * * *  * * * * * * * * * *  * * * * * * * * * *  * * * *
                                   *  * * * * * * * * * *  * * * * * * * * * *  * * * * * * * * * *
                                          * * * * * * * *  * * * * * * * * * *  * * * * * * * * * *

alignment position 3 0 1 . . . . . . .  3 1 1 . . . . . . .  3 2 1 . . . . . . .  3 3 1 . . . . . . .  3 4 1 . . . . . . .
P855801       86 t g c t c g t t t t  g a a g a t g a a a  t a a g a c a a t g  a a t g t a a a c c  a t a t g a t a c a
mNDUFC1      176 c a g t c a c t g t  a a t g a c t c g t  g t a a a g c a c a  c g - - - - - - - -  - - - - - - - - - -
rNDUFC1      291 - - - - - - - - - -  - - - - - - - - - -  - - - - - - - - - -  - - - - - - - - - -  - - - - - - - - - -

alignment position 3 5 1 . . . . . . .  3 6 1 . . . . . . .  3 7 1 . . . . . . .  3 8 1 . . . . . . .  3 9 1 . . . . . . .
P855801      136 t t a a t g c a c c  g t g c c t g g a c  c a c a g a a t t c  a a t c c a a a t g  c t a g t t a t t a
mNDUFC1      208 - - - - - - - - - -  - - - - - - - - - -  - - - - - - - - - -  - - - - - - - - - -  - - - - - - - - - -
rNDUFC1      291 - - - - - - - - - -  - - - - - - - - - -  - - - - - - - - - -  - - - - - - - - - -  - - - - - - - - - -

alignment position 4 0 1 . . . . . . .  4 1 1 . . . . . . .  4 2 1 . . . . . . .  4 3 1 . . . . . . .  4 4 1 . . . . . . .
P855801      186 t t c a g c a c g a  c a c t t t t C T T  T C T T C T G G C T  C A T G T A G G A A  A A T T T T A A T A
mNDUFC1      208 - - - - - - - - - -  - - - - - - - - - A  G T T T T T G G C T  G A C A A A C G A A  C T C T G T A G T A
rNDUFC1      291 - - - - - - - - - -  - - - - - - - C T A  G T T T T T G A C T  G A C A G A C G A A  C A C T G G A G T A
                                                    * * *  * * * * * * * * * *  * * * * * * * * * *  * * * * * * * * * *
                                                        *  * * * * * * * * * *  * * * * * * * * * *  * * * * * * * * * *
                                                        *  * * * * * * * * * *  * * * * * * * * * *  * * * * * * * * * *

alignment position 4 5 1 . . . . . . .  4 6 1 . . . . . . .  4 7 1 . . . . . . .  4 8 1 . . . . . . .  4 9 1 . . . . . . .
P855801      236 A A C C T c t t g a  t g a a t a g c c g  t a g a g a t g g g  g t g a a a a a c c  c c c a g a g g a c
mNDUFC1      239 A A C C T G A C c A  G C A - - - - - - -  - - - - - - - - - -  - - - - - - - - - -  - - - - - - - - - -
rNDUFC1      324 A A C C T G A C a A  G C A - - - - - - -  - - - - - - - - - -  - - - - - - - - - -  - - - - - - - - - -
                 * * * * * * * *   *  * * *
                 * * * * * * * *   *  * * *
                 * * * * * * * *   *  * * *

alignment position 5 0 1 . . . . . . .  5 1 1 . . . . . . .  5 2 1 . . . . . . .  5 3 1 . . . . . . .  5 4 1 . . . . . . .
P855801      286 a g a g a a t a g g  t a a a c t c t t t  t t t c t t t t a a  t t a t t t t c t t  c c t a a t T C C A
mNDUFC1      252 - - - - - - - - - -  - - - - - - - - - -  - - - - - - - - - -  - - - - - - - - - -  - - - - - - C C C T
```

```
rNDUFC1     337 ---------- ---------- ---------- ---------- ------TCCT
                                                                  ****
                                                                  ****
                                                                  ****

alignment position 551....... 561....... 571....... 581....... 591.......
P855801     336 CTGTATAGAC AGAAGGCTg- ---------- ---------- ----------
mNDUFC1     256 GGAGATGGAC AAACTGCTTT CCCAGCCCca a--------- ----------
rNDUFC1     341 GGGGATAGAC AAACTGCTTT CCCAGCCCaa gcccagtag agccccagcc
                ********** ********** ********
                ********** ********** ********
                ********** ********** ********

alignment position 601....... 611....... 621....... 631....... 641.......
P855801     355 ---------- ---------- ---------- ---------- ------AGGC
mNDUFC1     287 ---------- CCCTCTTCGT ACCCTTTGTT TTATTCACAG CCCACTAAGT
rNDUFC1     391 ccagccccag CCCTCTTTGC ACCTTGTGTT TTATGCACAG ACTCCTGAGT
                           ********** ********** ********** **********
                           ********** ********** ********** **********

alignment position 651....... 661....... 671....... 681....... 691.......
P855801     359 CGGGGTGCTA GAGAACCTTG CCGTGCTGGA At tcacgttg acgcccccac
mNDUFC1     327 CCGGGTTCTT GGGAACCTTG TACTGAATGA Ag gtaggagT CTGGCCCACC
rNDUFC1     441 ---------- --GAACCTTG TCCTGGt agct ac gac----T CTGGCCCACC
                ********** ********** ********** *         * **********
                                                           * **********
                                                           * **********
                                                           * **********
                                                           * **********

alignment position 701....... 711....... 721....... 731....... 741.......
P855801     409 ggagctctgc tgcagagccc tCGAGTCCCg cacTTCCGGC CGCCAGGTGG
mNDUFC1     377 TG-------- ---------- -CGAGTCCCA ---GTTCTGC TGCCAGGGGG
rNDUFC1     475 TG-------- ---------- -CGAGTCCCA ---TTTCTGC TGCCAGGGGG
                **                      *********    ******* **********
                **                      *********    ******* **********
                **                      *********    ******* **********
                **                      *********    ******* **********
                **                      *********    ******* **********

alignment position 751....... 761....... 771....... 781....... 791.......
P855801     459 CGCTGgttct ---------- ---------- ---------- --------GT
mNDUFC1     405 CGCTGCGCTC TGCGCCACCA ACCGGAGATG CGCGCGCAGG gaGAAGGCGG
rNDUFC1     503 CGCTGCGCTC TGCGGTACCT GCCGGAGATG CGCGCGCAGG agGAAGGCGG
                ********** ********** ********** **********   ********
                ********** ********** ********** **********   ********
                ********** ********** ********** **********   ********
                *****
                *

alignment position 801....... 811....... 821....... 831....... 841.......
P855801     471 TGCCAACTCG GAGAGACtga gctgggcca- ---------- -------CGC
mNDUFC1     455 TGCCAGCTCG GCCAAACGAG CAAccCGAGA AGAGGCGGAG CCTGCGGAAC
rNDUFC1     553 TGCCCGCTCG GCAAAACGAG CAAagCGAGA AGAGGAGGAG CCTGAAGCGC
                ********** ********** ***  ***** ********** **********
                ********** ********** ***  ***** ********** **********
                ********** ********** ***                          ***
                                                                     *

alignment position 851....... 861....... 871....... 881....... 891.......
P855801     503 AAGATGGCGC CGTCCGCCTT GCTGCGTCCC CTTTCcCGGC TGCTGGCCCC
mNDUFC1     505 AAGATGGCGC CGTCCGTAGT GCTGCGCTCG TTTTCGCGGC TGCTGGCCCC
rNDUFC1     603 AACATGGCGC CGCCCGTAGT ACTGCGCTCG TTTTCGCGGC TGCTGGCCCC
                ********** ********** ********** ********** **********
                ********** ********** ********** ********** **********
                ********** ********** ********** ********** **********
                ********** **   ***** ********** ********** **********
                                ***** ********** ********** **********
                                ***** ********** *****  **** **********
```

```
                              *** **** ********** *****  **** **********
                                                         **** **********
                                                         **** **********
                                                         **** **********

alignment position 901.......  911.......  921.......  931.......  941.......
P855801      553 CGCCAGGCTC CCGAGCGGCC GTGAGTatcc tcctcgTGCA G-GCCGACTC
mNDUFC1      555 CGCCCGGCTG CCGAGCTGCT GTGAGTGTCT T-----TTCA GaGCCCATTC
rNDUFC1      653 CGCCAGGCTG CCGAGCTGCT GTGAGTGTCT T-----TTCA G-GCCCATTC
                 ********** ********** ********** *     **** *  ********
                 ********** ********** ********** *     **** *  ********
                 ********** ********** ********** *     **** *
                 ********** ********** ********** *     **** *
                 ********** ********** ******
                 ********** ********** ******
                 ***   ***** ********** ******
                 ***   ***** ********** ******
                 ***   ***** ********** ******
                 ***   ***** ********** ******

alignment position 951.......  961.......  971.......  981.......  991.......
P855801      602 GACCCTCAGA TTACCCCTCc taaggcggac ccagcc---- ---CACGGGT
mNDUFC1      600 GGCCCTCAGC CTAGCCCTCA TCCAACACGA GCCACCCTGG AATCTCCGGA
rNDUFC1      697 GGCCTTTAGC CTAt-CCTCA CCCAGCATGA GCCACACTGG AATCTCTGTA
                 ********** ********** ********** ********** **********
                 ********** ***   **** ********** ********** **********
                                  ****                           ******

alignment position 1001......  1011......  1021......  1031......  1041......
P855801      645 GCAGGAGGCG GCAGCGGCCC GGGttCGTCC TGCAGTGACC TTCGCCGGCa
mNDUFC1      650 GGAGGAGGAG GTCGCGGTCC AGGC-CGACC TGCAGTGACC TTCACTGGCC
rNDUFC1      746 GTAGGAGGAG GTCGCGGTCC AGGC-CGTCC TGCAGTGACC TTCACCGt CC
                 ********** ********** **** ***** ********** **********
                 ********** ********** **** ***** ********** ******   **
                 ********** ********** **** ***** ********** ******   **
                       ***** ********** **** ***** ********** *****    **
                       ***** ********** **** ***** ********** *****    *
                                             ***** ********** *****
                                             ***** ********** *****

alignment position 1051......  1061......  1071......  1081......  1091......
P855801      695 gacgacccaa ggcccagccc acgcctgtcc cctacGCAGG GCTCTCTGCT
mNDUFC1      699 GCGA------ ---------- ---------- -----GCCGG GTTCTCCACT
rNDUFC1      795 GCGA------ ---------- ---------- -----GCCGG GTTCTCCACT
                 ****                                   ***** **********
                 ****                                   ***** **********
                 ****                                   ***** **********
                 ****                                   ***** **********

alignment position 1101......  1111......  1121......  1131......  1141......
P855801      745 CCCCATCACC CGCCtctagc acttatccaa gttcaacctg accACTCTTG
mNDUFC1      718 CATCATTAGC CGTCAG---- CTTTCCTCCA GGCTA----- ---AACCCTG
rNDUFC1      814 CCTCATCAGC CGTCAGtcag CTTTCCTCCA AACTA----- ---ACCCCTG
                 ********** ******     ********** *****         *******
                 ********** ******     ********** *****         *******
                 ********** ******     ********** *****         *******
                 ********** ******                                 ****

alignment position 1151......  1161......  1171......  1181......  1191......
P855801      795 GCCTTTGTAA ATGGACTTTG GGTCCTTGcc ttccccacct ctgtacgtta
mNDUFC1      756 ACCTCTGCGA ATGGATTTTG AGTCTTTTtG TCCCAGGCTT TTCAATGTCT
rNDUFC1      856 ACCTCTACGA ATGAATTTTG GGTCTTTT-G TCCCAGGCTC TTAATTGCCT
                 ********** ********** ******** * ********** **********
                 ********** ********** ********
                 ********** ********
                 ********** ********

alignment position 1201......  1211......  1221......  1231......  1241......
P855801      845 ggtcccgt-- AAACCGGGAG AggagacACC CTCCCCGGGG CGCCCCTTAC
```

```
mNDUFC1      806 GGGAG- - - - -  - - - - - - - - - -  - - - - - - - ACC CTTCCGGGGC TGCCTCCCAA
rNDUFC1      905 CGGAGa c a a c  AAACAGGGAG At - - - - - - - C CTTCCGGGGC TGCCTCCCAA
                 * * * * *           * * * * * * * * * *  *             * * *  * * * * * * * * * *  * * * * * * * * * *
                                                                          *  * * * * * * * * * *  * * * * * * * * * *
                                                                          *  * * * * * * * * * *  * * * * * * * * * *
                                                                          *  * * * * * * * * * *  * * * * * * * * * *
                                                                          *  * * * * * * * * * *  * * * * * * * * * *

alignment position 1251......  1261......  1271......  1281......  1291......
P855801      893 ATGACCACTC CCGAGTCgTC TGAGGGTCCG CAAGCCCTTA AACGAGGCTT
mNDUFC1      834 CCTCCCACTC CAGAGCC-TA TGAGGGCCGA CGAGGCTTTA ACCCAAGCTT
rNDUFC1      948 CCTCCCACTC TAGAGCC-Tt TGAGGGCCGA CGAGACTTTA ACCTAAGCTT
                 * * * * * * * * * *  * * * * * * *   * *  * * * * * * * * * *  * * * * * * * * * *  * * * * * * * * * *
                 * * * * * * * * * *  * * * * * * *   *    * * * * * * * * * *  * * * * * * * * * *  * * * * * * * * * *
                 * * * * * * * * * *  * * * * * * *   *    * * * * * * * * * *  * * * * * * * * * *  * * * * * * * * * *
                 * * * * * * * * * *  * * * * * * *   *    * * * * * * * * * *  * * * * * * * * * *  * * * * * * * * * *
                 * * * * * * * * * *  * * * * *       *    * * * * * * * * * *  * * * * * * * * * *  * * * * * * * * * *

alignment position 1301......  1311......  1321......  1331......  1341......
P855801      943 TGAAAGCctc ggacaggaca ggtgcgtcct gtctaactcg ccagtggccc
mNDUFC1      883 TGAAGGCGCC ctgagtaCCA AGGACGACCT TCTGTCTCTG CTGGTGTCCC
rNDUFC1      997 TGAAGACGCC atgagccCCA AGGACGACCT TCTGTCTCTG CTGGTGTCCC
                 * * * * * * * * * *               * * *  * * * * * * * * * *  * * * * * * * * * *  * * * * * * * * * *
                 * * * * * * * * * *               * * *  * * * * * * * * * *  * * * * * * * * * *  * * * * * * * * * *
                 * * * * * * * * * *               * * *  * * * * * * * * * *  * * * * * * * * * *  * * * * * * * * * *
                 * * * * * * * * * *               * * *  * * * * * * * * * *  * * * * * * * * * *  * * * * * * * * * *
                 * * * * *                         * * *  * * * * * * * * * *  * * * * * * * * * *  * * * * * * * * * *

alignment position 1351.....
P855801      993 tgggcgagg
mNDUFC1      933 CGAACaaat
rNDUFC1     1047 CGAACgaat
                 * * * * *
                 * * * * *
                 * * * * *
                 * * * * *
                 * * * * *
```

Pairwise similarities:

For each pairwise alignment, the similarity (relative to the maximum similarity) and the number of identical nucleic acids (in % of shorter sequence) is given. Maximum values are underlined.

	mNDUFC1 (941 bp)	rNDUFC1 (1055 bp)
**P855801 (1001 bp)**	0.151<br>34 %	0.140<br>32 %
**mNDUFC1 (941 bp)**		1.000<br>79 %

Please note that the **similarity value 1.000** marks only the two **most similar** sequences, it does not necessarily mean that these sequences are identical.

Extract alignment region	
**Positions in alignment**	from: to:
**Output file**	dialign.seq

Extract aligned sequences

For comments, questions or bug reports, please contact support@genomatix.de

© Genomatix Software GmbH 1998-2005
- All rights reserved.

License Agreement

GEMS Launcher 4.1.1

Logout Personal Messages

GEMS Launcher Task: *DiAlign TF*: Multiple alignment plus TF sites
working on promoters.seq (3 seq.)

[Alignment] [Pairwise similarities]

DiAlign professional TF Release 3.1 December 2004 **Tue Jun 7 19:27:17 2005**

Solution parameters:

**Sequence file:**	promoters.seq (3 seq.)
**Type of sequences:**	nucleotide sequences
**Threshold T:**	0.00
**'*' signs below alignment denote:**	diagonal similarity (max. similarity is represented by 10 '*' signs)
**Alignment output:**	complete alignment is shown<br># nucleic acids per line: 50
**TF output:**	common TF matches located in aligned regions (common to 3 (100.0 %) sequences)
**Family matches:**	yes
**MatInspector library:**	Matrix Family Library Version 5.0 (February 2005)
**Selected groups** (core/matrix sim):	• ALL user_defined.lib (0.75/Optimized)<br>• ALL vertebrates.lib (0.75/Optimized)

Aligned Sequences:

No.	Sequence Name	Sequence Description	Length
1	P914220	sym=NDUFC2|loc=Loc4718|taxid=9606|spec=Homo sapiens|chr=11| ctg=NT_033927|str=(-)|start=8013089|end=8014180|len=1092| tss=501,526,545,592| comm=NADH dehydrogenase (ubiquinone) 1, subcomplex unknown, 2, 14.5kDa; (AK098173/545/gold;AK023013/526/gold;AK022785/592/gold; NM_004549/501/silver;)	1092 bp
2	P914221	sym=Ndufc2|loc=Loc68197|taxid=10090|spec=Mus musculus|chr=7| ctg=NT_039433|str=(+)|start=15232116|end=15233139|len=1024| tss=501,516,520,524| comm=NADH dehydrogenase (ubiquinone) 1, subcomplex unknown, 2; (AK002373/536/gold;AK008889/536/gold;AK019002/517/gold; AK003380/540/gold;NM_024220/532/bronze;)	1024 bp
3	P914222	sym=na|loc=Loc293130|taxid=10116|spec=Rattus norvegicus| chr=1|ctg=NW_047561|str=(+)|start=13521471|end=13522471| len=1001|tss=501| comm=similar to RIKEN cDNA 1810004I06; (XM_214989/545/bronze;)	1001 bp

Alignment (DiAlign format):

Please note that only **upper-case** letters are considered to be **aligned**. For more information, please have a look at the user guide.

V$TBPF V$ETSF V$EGRF V$INSM V$SP1F V$PBXC

```
alignment position 1.........  11........  21........  31........  41........
P914220         1  gtgaaacatt  ttagggtgct  gcctccgaat  ctgaatttca  acaatagact
P914221         1  aggggT----  ----------  ----------  ----------  ----------
P914222         1  t---T-----  ----------  ----------  ----------  ----------
```

```
                        *
alignment position  51........ 61........ 71........ 81........ 91........
P914220         51 aAAACGATCC AGTCCTGGGC TTCTCTAAAT tcTTTTGTTG CTGGCCAGAC
P914221          6 -AAATGATTC GCTCCTGACC TCCgaatacc c----TGGTG CTGACCTTAC
P914222          3 -AAAGGATCT GCTCCTGGCC TTCTCTGAAT a-TTCTGCTA CTGACCTTCC
                    ********* ********** **********   ******** **********
                                                      ******** **********
                                                           ***** **********
                                                                 **********
                                                                 **********

alignment position 101........ 111........ 121........ 131........ 141........
P914220        101 CTTAATTTTA GGCTTTGGAG G--------- --GAGTGTCT ATGCGAATGA
P914221         51 CTTCATTTTA GGCTTTGGGG GTGGGGga-- -----AGTAA CTGCAAACGA
P914222         51 CTTAATTTTA GGCATTGGGG GTGGGGcgtg aaGGGAGTAA CTGCAAATGA
                   ********** ********** ******       ******** **********
                   ********** ********** ******            ***** **********
                   ********** ********** ******            ***** **********
                   ********** ********** *
                   ********** ********** *

alignment position 151........ 161........ 171........ 181........ 191........
P914220        140 ACGACTGAAA ACTCAGAAAa cgaaaaCTAG CTTTTATGGA ATTTCAATTA
P914221         94 ACTGCTGGAA CCCCAGAAAG ------CTTG CTTTTATAGG CTTTAGATAA
P914222        101 ACTGTCGGAA CCCCAGAAAG ------CTAG CTTTTATGCG TTTTCAACAA
                   ********** **********       **** ********** **********
                   ********** **********       **** *                  **
                   ********** **********       **** *                  **
                                               **** *

alignment position 201........ 211........ 221........ 231........ 241........
P914220        190 ATTacgttta tcaaAACTAC GCATCATGCC CATTTGTGGG TATTGGATGa
P914221        138 ATG------- ----AACTAG CCATTATGAC CACTTGTAGA TGTTGa-TTT
P914222        145 ATG------- ----AACTAC CTATCACGAC CACCTGTAGG TGTTGGGTTT
                   ***            ****** ********** ********** **********
                   ***            ****** ********** ********** *****   ***
                   **             ****** ********** ********           ***

alignment position 251........ 261........ 271........ 281........ 291........
P914220        240 aaagata--- ----AATCTT TCCAGAA--- ---------- -GGGGCAGGG
P914221        176 TTGGATGCTA AGATAATGTT TTCTGAAGAG CCATaggcct gGGGGCAAGT
P914222        184 TTGGATGCTA AAATAATCTT TTCTGAACAG TCATgaggct -GGGGCAAGT
                   ********** ********** ********** ****        *********
                   ********** ********** ********** ****        *********
                   ********** ********** ********** ****

alignment position 301........ 311........ 321........ 331........ 341........
P914220        269 GAGGGAGAGG CAATTCTGGT TGGGGAagtt gggagaaaag a---------
P914221        226 AAGTGAGAGA CAATTTCGAT TGTGGTAACT GGACTACATA CATACATACA
P914222        233 GAGTGAGAa- -AATTTCGTA TGCGACAACT GAACTAAATA AACAAATAAA
                   ********** ********** ********** ********** **********
                   ********     ******** ******

alignment position 351........ 361........ 371........ 381........ 391........
P914220        310 ---------- ---------A AATCAGTATC TCCTAGTCTT CGCCCAGTTT
P914221        276 Tacatacata catacatacA TACCAATGTT TCCTAGCCTT TGCCCACTGT
P914222        281 Taaatag--- ---------- ---CAACGTT TCCTAGTCTT TGCCCACTGT
                   *                  * ********** ********** **********
                                           ******* ********** **********
                                           ******* ********** **********
                                           ******* ********** **********
                                                   ********** **********

alignment position 401........ 411........ 421........ 431........ 441........
P914220        341 GGCTCGTTTG GGGTACTCCT cgacccgcgt t-TACCAGGC CCgtgaatCC
P914221        326 GGGTTATTTA TTataacccc ccccacacac cacacacaca ca------CA
P914222        315 GGGTTATTTA TTTTACCCCT ggactggctt agTACCTGGC CCccagcacc
```

```
                    ********** **********              ******** **      **
                    ********** **
                    ********** **
                    ****

alignment position  451....... 461....... 471....... 481....... 491.......
P914220         390 CACTCCCACC AGAGAATTAG CGCGGGCgga cgagcaaagt gaa--ACTTA
P914221         370 CACACACACT ACAATGTCAG CGCAAGCCTT CACGCGTTGT GGGCCACTTA
P914222         365 ctcat----T ACAACATCAG CCCAATCCAG CAGGCGTTGT CGGCCACTTA
                    ********** ********** ********** ********** **********
                             * ********** ********** ********** **********
                                                                      ****
                                                                      ****

alignment position  501....... 511....... 521....... 531....... 541.......
P914220         438 GTAGCCCGGA ACTTCGCGTG CCGGGTTCCC GCAGcaaca- CGTACCGGGA
P914221         420 GAAGCCCGGA ACCTCGCGTG CAGAGGTTGC TAAGGCTGAG CGCACAGGGA
P914222         411 ATATCCCGGA ACCTTGCATG CAGAGGTTCC TAAGGCTGCG CGCgCAGGGA
                    ********** ********** ********** ********** **********
                    ********** ********** ********** ********** **********
                        ****** ********** ********** ********** *** ******
                        ****** ********** *                     *** ******
                        ****** ********** *                           ****
                                                                      ****

alignment position  551....... 561....... 571....... 581....... 591.......
P914220         487 AGCGTTTGGG CGCTGCACCG CGCTGCGCCG TGCCCGTGAG TCCGGCGCGC
P914221         470 AGCTTTTGGG TGCTGCACCG CCGTACGCAA TGCCCTTCAG CCTAGAGCGC
P914222         461 AGCTTTTGGG TGCTGCACCG CCCTACGCtt TGCCCTTCAG CCCGGAGCTC
                    ********** ********** ********** ********** **********
                    ********** ********** ********   ********** **********
                    ********** ********** ********   ********** **********
                    ********** ********** ********
                    ********** ********** ********
                    ********** ********** *

alignment position  601....... 611....... 621....... 631....... 641.......
P914220         537 AGAGGAGGAG Gagaaagctg accgctTAGG CCGGGGTAGT GGTCGTCGTG
P914221         520 GGAGGAGGAC GGTAGC---- ------TcGG TCACGGCGTT TGGAGTCGTT
P914222         511 GGAAGAGGAG GGTAGC---- ------TAGG TCGCGGGGTT TGTAGTCGTG
                    ********** ******           **** ********** **********
                    ********** ******           **** ********** **********
                    ********** *                * ** ********** **********

alignment position  651....... 661....... 671....... 681....... 691.......
P914220         587 GTTTTCCTTG TAGTTCGTGg tctGAGACCA GGCCTCAAGT GGAAACGGCG
P914221         560 GTTTTTCCTA TAGTTGGAGT C--GAGGTCA AACCTCTCGT GGAACCTGCC
P914222         551 GTTTTTCTTA TAGTTGGAGT C--GAGGTCA GACCTCACGT GGAACCCGCA
                    ********** ********** *  ******* ********** **********
                    ********** ********** *  ******* ********** **********
                    ******* ** ********** *  ******* ********** *********
                            ** *****         ******* ********** *********

alignment position  701....... 711....... 721....... 731....... 741.......
P914220         637 TCACCATGAT ---CGCACGG CGGAACCCAG AACCCTTACG GTTTCTGCCG
P914221         608 TCAAGATGAT GAACGGCCGG CCGGGCCATG AGCCCTTAAA ATTCTTGCCG
P914222         599 TCAATATGAT GAACGGCCGG CCGGGCCATG AGCCCTTAAG ATTCTTGCCG
                    ********** ********** ********** ********** **********
                    ********** ********** ********** *********  **********
                    ********** ********** ********** *********  **********
                    ********** ********** ********** *********       *****
                    **********                                       *****
                                                                     *****

alignment position  751....... 761....... 771....... 781....... 791.......
P914220         684 GATGAGGCCC GGAGCCTGCC CCCGCCCAAG CTGACCGACC CGCGGCTCCT
P914221         658 GACGAGGCCC GGAGCCTGCC CCCGCCCAAG CTGAACGACC CGCGGCTTGT
P914222         649 GATGAGGCCC GGAGACTGCC CCCGCCCAAG CTGAACGACC CGCGGCTTGT
```

```
                   ********** ********** ********** ********** **********
                   ********** ********** ********** ********** **********
                   ********** ********** ********** ********** **********
                   ********** ********** ********** ********** **********
                   ********** ********** ********** ********** **********
                   ********** ********** ********** ********** **********
                          *** ********** ********** ********** **********
                          *** ********** ********** **** ***** *******  *
                          *** ********** ********** **** ***** *******  *
                                           ******** **** ***** *******

alignment position 801....... 811....... 821....... 831....... 841.......
P914220        734 CTACATCGGC TTCTTGGGCT ACTGCTCCGG CCTGATTGAT AACCTAATCC
P914221        708 CTACATGGGC TTGTTGGGCT ACTGCACGGG CCTGATGGAC AACATGcTGC
P914222        699 CTACATCGGC TTCCTGGGCT ACTGCACGGG CCTGATGGAC AACATGATGC
                   ********** ********** ********** ********** **********
                   ********** ********** ********** ********** ****** ***
                   ********** ********** ********** ********** ****** ***
                   ********** ********** ********** ********** ******   *
                   ********** ********** ********** *********
                   ********** **  ****** *****   ** *********
                   ********** **  ****** *****
                   ********** **  ****** *****
                   ********** **

alignment position 851....... 861....... 871....... 881....... 891.......
P914220        784 GGCGGAGGCC GATCGCGACG GCTGGTGAGT GCTGCGCGGC CGggatcggc
P914221        758 GGATGCGACC GGTGATGAGA GCAGGTGAGG GCTGCGggcg atcccgct-C
P914222        749 GAATGAGACC TGTGATGAAA GCAGGTGAGG GCTGCGAGGC CGtcctaccC
                   ********** ********** ********** ********** **       *
                   ********** ********** ********** ******              *
                   ********** ********** ********** ******
                   **********            ********** ******

alignment position 901....... 911....... 921....... 931....... 941.......
P914220        834 tgggaggaga aggGGTCGTA GGGCTGCAGA CTGGCCCGgc cgtaggcccc
P914221        807 ---------- ---GGACGAG GAGCTGCAGG GTTGCCCGCT GAGGTCAt--
P914222        799 ---------- ---GGAGGAG GAGCTACAGG GTTGTCTACT AAGGTCAgcc
                                 ******* ********** ********** *******
                                 ******* ********** ********** *******

alignment position 951....... 961....... 971....... 981....... 991.......
P914220        884 cctgaggtgC TCTGCTTTTC CGTGCCTTTT TGCCTAGCTG GCCCCATCTC
P914221        842 ---------C TGTGCTCTAC GAGGCCTTCC TGCCTAGCTG GGGGCGCCTG
P914222        836 tcccggatcc g-TGCTTTTC Ccgg------ -GCCTAGCTG GGAGCGCCTG
                            * ********** ********** ********** **********
                                                     ********* **********
                                                     ********* **********
                                                     ********* *

alignment position 1001...... 1011...... 1021...... 1031...... 1041......
P914220        934 GCCTTTCCGG GGAGTCCGCG CCCCATTAGC TCTTTAGaat caagcagagc
P914221        883 GCTCTCCCAG GGCGTGCATC CCCTCTCGGG TTCTCAGCGC caagagtAGC
P914222        878 GCTTTCCCAG GTCGTGCACC CCGTCTAGAG TTTTCAGCGC g------AGC
                   ********** ********** ********** **********        ***
                   ********** ********                                ***
                   ********** ********

alignment position 1051...... 1061...... 1071...... 1081...... 1091......
P914220        984 cagagtgtca tttggcacTT GAAGAAACTG AGGTGCAGTG AAGCAGGAAC
P914221        933 CACA------ --------TT GAAGAAAACC AAGTGCAGTG CAATGGGGAC
P914222        922 CACA------ --------TT AAAGAAACCC AAGTGCGCCG CAACTGGGAA
                   ****               ** ********** ********** **********
                   ****               ** ********** ******   * **********

alignment position 1101...... 1111...... 1121...... 1131...... 1141......
P914220       1034 Tg-------- --------AG TCTGTGAAGG ACCCGAAAct a-CTCTCCTC
P914221        969 TTCTAAACCC CACGGTtaAG TCTGCGAAGG ACTCAAGAAG GGCTCCTCAC
P914222        958 TTCTAAACCT CCCAGTt-AG TCCGTAAAGG ACTCAAGAGG GGCTC-----
```

```
                         **** ***** ** ***** **     ** ********** **** ***** ** **********
                                                    ** ********** **
alignment position 1151 . . . . . .  1161 . . . . . .  1171 . .
P914220    1067  AGTT CGt g t t  g t g c t g t g t g  c g c a a a
P914221    1019  AGTT CG- - - -  - - - - - - - - - -  - - - - - -
P914222    1002  - - - - - - - - - -  - - - - - - - - - -  - - - - - -
                 **** **
```

Pairwise similarities:

For each pairwise alignment, the similarity (relative to the maximum similarity) and the number of identical nucleic acids (in % of shorter sequence) is given. Maximum values are underlined.

	**P914221 (1024 bp)**	**P914222 (1001 bp)**
**P914220 (1092 bp)**	0.340<br>51 %	0.354<br>53 %
**P914221 (1024 bp)**		<u>1.000</u><br><u>74 %</u>

Please note that the **similarity value 1.000** marks only the two **most similar** sequences, it does not necessarily mean that these sequences are identical.

**Extract alignment region**	
**Positions in alignment**	from: to:
**Output file**	dialign.seq

Extract aligned sequences

For comments, questions or bug reports, please contact support@genomatix.de

© Genomatix Software GmbH 1998-2005 - All rights reserved. License Agreement GEMS Launcher 4.1.1

Genomatix-Logo Logout Personal Messages GEMS-Logo
Logout Personal Messages

Genomatix MainGEMSElDoradoGene2PromoterBiblioSphere
Frequently asked questionsYour ResultsYour SequencesYour ProtocolHelp

GEMS Launcher Task: *DiAlign TF*: Multiple alignment plus TF sites
working on promoters.seq (3 seq.)

[Alignment] [Pairwise similarities]

DiAlign professional TF Release 3.1 December 2004 **Tue Jun 7 19:41:50 2005**

Solution parameters:

**Sequence file:**	promoters.seq (3 seq.)
**Type of sequences:**	nucleotide sequences
**Threshold T:**	0.00
**'*' signs below alignment denote:**	diagonal similarity (max. similarity is represented by 10 '*' signs)
**Alignment output:**	complete alignment is shown<br># nucleic acids per line: 50
**TF output:**	common TF matches located in aligned regions (common to 3 (100.0 %) sequences)
**Family matches:**	yes
**MatInspector library:**	Matrix Family Library Version 5.0 (February 2005)
**Selected groups** (core/matrix sim):	• ALL user_defined.lib (0.75/Optimized)<br>• ALL vertebrates.lib (0.75/Optimized)

Aligned Sequences:

No.	Sequence Name	Sequence Description	Length
1	P922028	sym=NDUFS1|loc=Loc4719|taxid=9606|spec=Homo sapiens|chr=2|ctg=NT_005403|str=(-)|start=57233028|end=57234103|len=1076| tss=501,576| comm=NADH dehydrogenase (ubiquinone) Fe-S protein 1, 75kDa (NADH-coenzyme Q reductase);(AK098395/637/gold;NM_005006/562/bronze;)	1076 bp
2	P922029	sym=Ndufs1|loc=Loc227197|taxid=10090|spec=Mus musculus|chr=1|ctg=NT_039170|str=(-)|start=40865305|end=40866347| len=1043|tss=501,525,543| comm=NADH dehydrogenase (ubiquinone) Fe-S protein 1;(AK078590/619/gold;AK036926/601/gold;NM_145518/577/bronze;)	1043 bp
3	P922030	sym=nalloc=Loc301458|taxid=10116|spec=Rattus norvegicus|chr=9|ctg=NW_047816|str=(-)|start=5260820|end=5261820| len=1001|tss=501| comm=similar to RIKEN cDNA 9930026A05; (XM_217417/501/silver;)	1001 bp

Alignment (DiAlign format):

Please note that only **upper-case** letters are considered to be **aligned**. For more information, please have a look at the user guide.

V$MOKF V$ETSF V$TBPF V$SRFF V$PERO V$HNF4 V$COUP V$EREF U$EREF V$GKLF V$RORA V$BRNF
V$HNF1 V$LHXF V$RBIT V$HOMF V$HOXF V$SP1F V$DEAF V$NFKB V$SORY V$YY1F V$HNF6 V$SF1F
V$EGRF

```
alignment
position       1 . . . . . . . . .  1 1 . . . . . . . .  2 1 . . . . . . . .  3 1 . . . . . . . .  4 1 . . . . . . . .
P922028     1  - - - - - - - - - -  - - - - - - - - - -  - - - - - - - - - -  - - - - - - - - - -  - - - - - - - - - -
```

```
P922029        1 cgaagg----  ----------  ----------  ----------  ----------
P922030        1 cccctcaatg  tagctcttgt  ccgccaggta  atcgttgagc  acctggaggc

alignment
position          51........  61........  71........  81........  91........

P922028        1 ----------  ----------  ----------  ----------  -------GAG
P922029        7 ----------  ----------  ----------  ---------C  TGGAACGGGG
P922030       51 cggcgggggt  tttcaggtct  ccgaaaccca  tggcgtcgaC  TGTAACCGGG
                                                               *  **********

alignment
position          101.......  111.......  121.......  131.......  141.......

P922028        4 AGCTGGGGAG  CAGCAGAAAG  AGAGCGCGCA  AATTGTGGGC  GCCCCACGCT
P922029       18 AGCTGTGCAA  AAGCGGAGGA  CGAGAGCGCC  GGGAGGCCGC  GGCCAGCGCT
P922030      101 AGCGGAGGAA  AAG-------  ----AGCGCT  AGAAGCCCGC  GGCCAGCGCT
                 **********  **********  **********  **********  **********
                                                ******  **********  **********
                                                ******  **********  **********

alignment
position          151.......  161.......  171.......  181.......  191.......

P922028       54 GAAGagagGA  AAAAGGACCC  GAAGAGACCG  GAAAAtTCCT  TATATAGAGA
P922029       68 GAGGGA--AA  AAAAGGACCC  AGAGCGTCCG  GAAAACTCCT  TATATAGAGA
P922030      140 GAGGGA--GA  AAAAGGGCCG  GGACCGGCCG  GAAAAGTCCT  TATATAGAGA
                 ******  **  **********  **********  **********  **********
                 ******  **  **********  **********  **********  **********
                 ******  **  ******             ***  **********  **********
                          *  ******             ***  **********  **********
                                                ***  *****  ****  **********
                                                           ****  **********
                                                           ****  **********
                                                           ****  **********
                                                           ****  **********
                                                           ****  **********

alignment
position          201.......  211.......  221.......  231.......  241.......

P922028      104 CGGAGGCGTT  TCCCTCCACC  GCGCCGgacc  gaAGGTTAAA  GGTCAAAGTA
P922029      116 CGGAGGCGTT  TCCCTCCGCC  GCCCCGGCTA  G-AGGTTAAA  GGTCAAGGTC
P922030      188 CGGAGGCGTT  TCCCTCCACT  GCACCGGCTA  G-AGGTTAAA  GGTCAAAGTA
                 **********  **********  **********  *  ********  **********
                 **********  **********  **********  *  ********  **********
                 **********  **********  **********  *  ********  **********
                 **********  **********  ******         ********  **********
                 **********  **********  ******         ********  **********
                 **********  **********  ******
                 **********  **********  ******
                 **********  *******        ***
                 **********  *******        ***
                 **********  *******        ***

alignment
position          251.......  261.......  271.......  281.......  291.......

P922028      154 CTTTTGCATT  CCCACTTCTC  CACGTAcgaa  ttgggggccc  TGAATTAAAT
P922029      165 CGTTCCTAGT  CTCGGTCCTC  CGCGTACCAC  TGCGACTTG-  GTAACTAAAG
P922030      237 CGTTACTAGT  CTCGTTCCTT  CACGTAAAAC  TGCGTCTTG-  TGAATTAGAC
                 **********  **********  **********  *********  **********
                 **********  ****  *****  *                      **********
                 **********  ****
                 **********  ****
                 *

alignment
position          301.......  311.......  321.......  331.......  341.......

P922028      204 GGCCCACAAA  TTGTCCATGG  ACCTTTCGTT  GCGTTTGGAA  GTAAATTAAT
P922029      214 GGCCCACAGG  TCGTCCGCCG  GCCTTCCCTT  GTCTTTGGAA  GAAAATTAAT
P922030      286 GACCCACAGG  TCGTCCGCGG  GCTTTTCCTT  GTGTTTGGAA  GGAAATTAAT
```

```
                 **********  **********  **********  **********  **********
                 *  *******  **********  **********  **********  **********
                    *******  **********  **********  **********  **********
                    *******  ******        ********  **********  **********
                                           ********  **********  *  *******
                                             ******  **********  *  *******
                                             ******  **********  *

alignment
position         351.......  361.......  371.......  381.......  391.......

P922028      254 AAAATAGGCA  TgtcTTAAGT  TCGTAC-CTC  CACGGCTCCC  GAAGATGACG
P922029      264 AAAGGAGGCA  TTT-TAAAAT  GCCTtcgCTC  CAAGGACTCC  GAAACGAAGG
P922030      336 AAAAGATGCA  TTT-TAAAAC  GCCTAC-CTC  CAAGGATTCT  AAAACTAAAG
                 **********  ***  ******  ******  ***  **********  **********
                 **********  ***  ******  ****    ***  **********  **********
                 **********  *
                 **********  *
                 **********  *
                 **********  *

alignment
position         401.......  411.......  421.......  431.......  441.......

P922028      303 GGAACcagCC  CTAGAAGCGC  CGGAGATGAC  CGAAGACGCC  CGGCCTCGAG
P922029      313 GAAACGC-CC  tcTGAGGAGC  CGGAGTTGAA  CGAAAGCGCT  CGGACGCAAG
P922030      384 GAAACGC-CC  CTTGAGGAGC  CGGAGTTGAA  CGAAAGCATT  CGGCCTCAAG
                 *******  **  **********  **********  **********  **********
                 *******  **     ********  **********  **********  **********
                          **     ********  **********  *******           ****
                                  *******  **********  *******           ****
                                                                         ****
                                                                         *

alignment
position         451.......  461.......  471.......  481.......  491.......

P922028      353 GCtggGGCCA  CTTCCGGCGG  AACTGCGGGT  CCAAGTTGTC  ct--------
P922029      362 GCCTCGGCTA  CTTCCGGCGG  AACTGCAGGT  CCAAGTCGTC  TTCTCCGCTT
P922030      433 GCCTCGGCTA  CTTCCGGCGG  AACTGCAGGT  CCAAGTTGTC  TTCTCCGCTT
                 **********  **********  **********  **********  **********
                 **********  **********  **********  **********  **********
                 **********  **********  **********  **********
                 **********  **********  **********  **********
                 **********  **********  **********  **********
                      *****  **********  **********  ******  ***
                      *****  **********  **********  ******
                      *****  **********  **********  ******
                      *****  **********  **********  ******

alignment
position         501.......  511.......  521.......  531.......  541.......

P922028      395 --TCCCGCCT  CCgggctggg  ccgacctcag  ctccgccctc  tggtcgGCTG
P922029      412 CCTCCCGGgc  tc--------  ----------  ----------  ------GCTC
P922030      483 TCTCCCGGCT  CCctctcgt-  ----------  ----------  ------GCTG
                 **********  **                                        ****
                 *******                                               ****

alignment
position         551.......  561.......  571.......  581.......  591.......

P922028      443 ACGGAAATTG  CCGAcaaac-  ----------  ----TT---G  TCGAATgcaa
P922029      428 TCGGAAATTG  CCGAACAGGT  CCGAAGACGG  GgaaaaacaG  TCGAATCTGT
P922030      506 ACGGAAATTG  CCGAACAGGT  CCAAAGAGGG  GagaTT---G  TCGAATCTGG
                 **********  **********  **********  *   **   *  **********
                 **********  **********  **********  *
                  *********  ****
                  *********  ****

alignment
position         601.......  611.......  621.......  631.......  641.......

P922028      475 ggaTTCTAGT  CCCTCCGGGC  TTTCCGTTCT  CCAGGcccGG  CTGACAGAGT
P922029      478 GT-TTCTAGT  CCCTCCTGGC  TTTCCGTCCT  CCAGGTCGGG  CTGACAGAGG
P922030      553 CT-TTGTTGT  CCCTCCTGGC  TTTCCGTCCG  CCAGGTCAGG  CTGACGGAGT
```

```
                  **   ******* ********** ********** ********** **********
                       ******* ********** ********** ********** **********
                       ******* ********** ********** ********** **********
                       ******* ********** ********** ********** **********
                            ** ********** ********** *****   ** **********
                            ** ********** ********** *****   ** ********
                            ** ********** ********** *****   ** ********
                                                             ** *******

alignment
position          651....... 661....... 671....... 681....... 691.......

P922028     525   TAGCCGAGGC CGCCATATTG AATAAGCGac ccggcctcct agGGGGTCGT
P922029     527   TTGCCGTGGC CGCCATATTG ACTTAGCGGC caGGGTTTTG A-GGGGTCAG
P922030     602   TAGCCGTGGC CGCCATATTG ACTTAGCGGC t-GGGTTTTG A-GGGGTCAG
                  ********** ********** **********   ******** *  *******
                  ********** ********** **********   ******** *  *******
                  ********** ********** **********   ******** *  *******
                  ********** ********** ********
                  ********** ********** ********
                    ******** ********** ********
                    ******** ********** ********
                    ******** ********** ********

alignment
position          701....... 711....... 721....... 731....... 741.......

P922028     575   CGTGGTCCAG ACAGTTTAGC AGAACAGCCT ccgcGGCTCC GGGGAGAAGG
P922029     576   GGAGACCCGG ACAGGTCCGC GGAACtACTC GGTGGGCTCC GGAGGAAAGG
P922030     650   GGGGATCCGG ACAAATCAGC GGGACAACTT GGTGAGCTCT AGAGGAAAGG
                  ********** ********** ********** ********** **********
                  ********** ********** **    **** ********** **********
                  ********** ********** **    **** ********** **********
                                                                  ****

alignment
position          751....... 761....... 771....... 781....... 791.......

P922028     625   TGAGGTCTTG TATGGATGGG AAGGGTGAGG TGCGTCGGCC AGAGGCTTAT
P922029     626   TGAGGACGCG TTGAAGTGGG CAAGTTGAAG CGCTTCGAGT GGTGTTTTCT
P922030     700   TGAGGACGCG TTTAAGAGAG AAAGTTGAAG TGCTTCGAGT GGTGTTTTCT
                  ********** ********** ********** ********** **********
                  ********** ******      ********* ********** **********
                  ********** ******      ********* ********** **********
                  ********** *           ********* ********** **********

alignment
position          801....... 811....... 821....... 831....... 841.......

P922028     675   TTATTGACGG GACTGTTTCC TTTGGCCCAc gcgacgtagT CTTCTGTTGT
P922029     676   TTTTTGGCAG Gtctgcatcc caTAGGTCAG ACAAGCCGTT CTCCTCTTGC
P922030     750   TTCTTAGCAG GACTGTGTTC TTTAGGTCAA ACAAGTCATT CTCCTCTTGT
                  ********** ********** ********** ********** **********
                  ********** *            ******** ********** **********
                  ********** *                                      ****
                  ********** *                                      ***
                                                                    ***

alignment
position          851....... 861....... 871....... 881....... 891.......

P922028     725   CCTTGACTGG GCGCCGCCTC CCGCCCCGCC GCCTCGGAAG CCCCTGAGAG
P922029     726   CCTTGACTGG GTACCAGCTC CTGCCCCGCC ACCTCTGGAA CCCGGGAGAG
P922030     800   CCTTGACTGG GCACCACCTC CTGCCCCGCC ACCTCcaGAA CCCCGGAGAA
                  ********** ********** ********** ********** **********
                  ********** ********** ********** ********** **********
                  ********** ********** ********** *****  *** **********
                  ********** ********** ********** *****  *** **********
                  ********** ********** ********** *****
                  ********** *      *** ********** *****
                                    *** ********** *****

alignment
position          901....... 911....... 921....... 931....... 941.......

P922028     775   CCGGCAGATG ggGGGCTTTT AGCCACGGTC TCTGGTCTGA TGAAGCAGAA
```

```
P922029     776 CCGGCAGGTG A-GGGTTATG AGCCTTGGTT TCTGGTCTGA TGGAGCCTAA
P922030     850 CCGGCAGGTG A-AGGTTAcT AGCCATAGTT TCTGGTCTGA TGGAGCATAA
                ********** * ******** ********** ********** **********
                ********** * ****** * ********** ********** **********
                *******      ****** * ********** ********** **********
                *******               ********** ********** ******

alignment
position        951....... 961....... 971....... 981....... 991.......

P922028     825 aagaagATGA GGATCAGGGG GTCCTCGGGA acgctgtcac gaatgtaGGC
P922029     825 GCc------- -GATCAGGAG ACCTTTGGGA GTCCG----- -------GGC
P922030     899 GC----AAGA TGATCAGGAA ACCTTTGGGA GTTCC----- -------GGC
                **    **** ********** ********** *****             ***
                **          ********* ********** *****             ***
                            ********* ********** *****             ***
                                                                   ***

alignment
position        1001...... 1011...... 1021...... 1031...... 1041......

P922028     875 TTGCAGGTGA CCTTCCCTCT GCAGGGAAAa ttCAGGACAT TTCCCTCTCA
P922029     855 TTGCGAGCTA CTCTCTCTTT GCAGGGGAAG A-CAGGAGAT GGCCCTGTCA
P922030     933 TTGCTAGCTA CTTTCTCTCT GCAGGGGAAG A-CAGGAGAT GGCCCTGTCt
                ********** ********** ********** * ******** **********
                ********** ********** ********** * ******** *********
                ********** **  ******* ********** * ******** *********
                ********** **  ******* *********
                               ******* *********

alignment
position        1051...... 1061...... 1071...... 1081...... 1091......

P922028     925 gaGGAGGAGG CCGTTTTATT CCCCGGGaga ggaccagtgt ccgatgactt
P922029     904 tc--AGGAGA CCGGTTCATC TCCCAGGgtc acgagagact taaacctcag
P922030     982 --GGAGGAGA CCGGGTCATT TC-------- ---------- ----------
                  ******** ********** *******

alignment
position        1101...... 1111...... 1121...... 1131...... 1141......

P922028     975 aggccttaag actaagcctt tccccccaga tttttacacg cctgatacag
P922029     952 gcatcg---- ---------- ---------- ---------- ----------
P922030    1002 ---------- ---------- ---------- ---------- ----------

alignment
position        1151...... 1161...... 1171...... 1181...... 1191......

P922028    1025 tgcatgattG TTTGGTCGCT GTTGTGttag caatggctCA CGCTTCTGTT
P922029     958 ---------G TTTAGTTGCT GTCTTGaacc tgtc----CA CGCTACTGTT
P922030    1002 ---------- ---------- ---------- ---------- ----------
                         * ********** ******             ** **********

alignment
position        1201...... 1211...... 1221...... 1231...... 1241.....

P922028    1075 TG-------- ---------- ---------- ---------- ---------
P922029     995 TGtttttgtc tagcccggtg atcatgttgg gtagtatttt attcattcc
P922030    1002 ---------- ---------- ---------- ---------- ---------
                **
```

Pairwise similarities:

For each pairwise alignment, the similarity (relative to the maximum similarity) and the number of identical nucleic acids (in % of shorter sequence) is given. Maximum values are underlined.

	P922029 (1043 bp)	P922030 (1001 bp)
**P922028 (1076 bp)**	0.396<br>54 %	0.442<br>55 %

**P922029 (1043 bp)**		1.000<br>73 %

Please note that the **similarity value 1.000** marks only the two **most similar** sequences, it does not necessarily mean that these sequences are identical.

**Extract alignment region**	
**Positions in alignment**	from: to:
**Output file**	dialign.seq

Extract aligned sequences

For comments, questions or bug reports, please contact support@genomatix.de

© Genomatix Software GmbH 1998-2005 - All rights reserved. License Agreement GEMS Launcher 4.1.1

Genomatix-Logo Logout Personal Messages GEMS-Logo
Logout Personal Messages

Genomatix_MainGEMSElDoradoGene2PromoterBiblioSphere
Frequently_asked_questionsYour_ResultsYour_SequencesYour_ProtocolHelp

GEMS Launcher Task: *DiAlign TF*: Multiple alignment plus TF sites
working on C1-NDUFS2 (3 seq.)

[Alignment] [Pairwise_similarities]

DiAlign professional TF Release 3.1 December 2004 **Tue Jun 7 20:32:24 2005**

Solution parameters:

**Sequence file:**	C1-NDUFS2 (3 seq.)
**Type of sequences:**	nucleotide sequences
**Threshold T:**	0.00
**'*' signs below alignment denote:**	diagonal similarity (max. similarity is represented by 10 '*' signs)
**Alignment output:**	complete alignment is shown<br># nucleic acids per line: 50
**TF output:**	common TF matches located in aligned regions (common to 3 (100.0 %) sequences)
**Family matches:**	yes
**MatInspector library:**	Matrix Family Library Version 5.0 (February 2005)
**Selected groups** (core/matrix sim):	• ALL user_defined.lib (0.75/Optimized)<br>• ALL vertebrates.lib (0.75/Optimized)

Aligned Sequences:

No.	Sequence Name	Sequence Description	Lengt
1	hNDUFS2	hNDUFS2 g17_dna range=chr1:157983624-157985875 5'pad=0 3'pad=0 revComp=FALSE strand=? repeatMasking=none	225: b
2	P914253	P914253 sym=Ndufs2|loc=Loc226646|taxid=10090|spec=Mus musculus|chr=1|ctg=NT_039185|str=(-)|start=15158394|end=15159394|len=1001|tss=501|comm=NADH dehydrogenase (ubiquinone) Fe-S protein 2; (NM_153064/501/bronze;)	100 b
3	P914254	P914254 sym=na|loc=Loc289218|taxid=10116|spec=Rattus norvegicus|chr=13|ctg=NW_047399|str=(-)|start=4601374|end=4602374|len=1001|tss=501|comm=similar to NADH dehydrogenase (ubiquinone) Fe-S protein 2; (XM_213940/524/bronze;)	100 b

Alignment (DiAlign format):

Please note that only **upper-case** letters are considered to be **aligned**. For more information, please have a look at the user guide.

V$ETSF V$DMTF V$YY1F V$MYOD

```
alignment position 1 . . . . . . . . .  1 1 . . . . . . . .  2 1 . . . . . . . .  3 1 . . . . . . . .  4 1 . . . . . . . .
hNDUFS2          1 c c a g a g a a c t  c t a c t g c c c c  t t t c c a c t c c  c c t a t t t g a g  a t g a g g a c a a
P914253          1 a c t t g a g t c -  - - - - - - - - - -  - - - - - - - - - -  - - - - - - - - - -  - - - - - - - - - -
P914254          1 n n n n n n n n n n  n n n n n n n n n n  n n n n n n n n n n  n n n n n n n n n n  n n n n n n n n n n

alignment position 5 1 . . . . . . . .  6 1 . . . . . . . .  7 1 . . . . . . . .  8 1 . . . . . . . .  9 1 . . . . . . . .
hNDUFS2         51 a g g g g g t g a c  t a a g c a a t g a  a a g a c a a a g g  g c t t t a g a g t  g g c t g c a g c a
```

```
P914253        10 - - - - - - - - - -  - - - - - - - - - -  - - - - - - - - - -  - - - - - - - - - -  - - - - - - - - - -
P914254        51 n n n n n n n n n n  n n n n n n n n n n  n n n n n n n n n n  n n n n n n n n n n  n n n n n n n n n n

alignment position 1 0 1 . . . . . . .  1 1 1 . . . . . . .  1 2 1 . . . . . . .  1 3 1 . . . . . . .  1 4 1 . . . . . . .
hNDUFS2       101 g t g t g t g t g g  g g g t g t t g g c  a g c a g g g a g a  a g g c a c a a a a  g c t g c a g t g t
P914253        10 - - - - - - - - - -  - - - - - - - - - -  - - - - - - - - - -  - - - - - - - - - -  - - - - - - - - - -
P914254       101 n n n n n n n n n n  n n n n n n n n n n  n n n n n n n n n n  n n n n n n n n n n  n n n n n n n n n n

alignment position 1 5 1 . . . . . . .  1 6 1 . . . . . . .  1 7 1 . . . . . . .  1 8 1 . . . . . . .  1 9 1 . . . . . . .
hNDUFS2       151 c c t a g g c a g c  c c c t c a g g a g  c c c c c c a c c c  c a g t c c c a g c  t c a a g c t g c c
P914253        10 - - - - - - - - - -  - - - - - - - - - -  - - - - - - - - - -  - - - - - - - - - -  - - - - - - - - - -
P914254       151 n n n n n n n n n n  n n n n n n n n n n  n n n n n n n n n n  n n n n n n n n n n  n n n n n n n n n n

alignment position 2 0 1 . . . . . . .  2 1 1 . . . . . . .  2 2 1 . . . . . . .  2 3 1 . . . . . . .  2 4 1 . . . . . . .
hNDUFS2       201 t c c a g g g c c a  a g c a g c c c a a  a g t c t a t c c t  g a c t t c t c c c  a g g c t g g a t c
P914253        10 - - - - - - - - - -  - - - - - - - - - -  - - - - - - - - - -  - - - - - - - - - -  - - - - - - - - - -
P914254       201 n n n n n n n n n n  n n n n n n n n n n  n n n n n n n n n n  n n n n n n n n n n  n n n n n n n n n n

alignment position 2 5 1 . . . . . . .  2 6 1 . . . . . . .  2 7 1 . . . . . . .  2 8 1 . . . . . . .  2 9 1 . . . . . . .
hNDUFS2       251 a a g g c a a a t a  a a t g c a g t c c  t t c c c a t c c c  c c a c c a c c a t  g g c c a c a t t t
P914253        10 - - - - - - - - - -  - - - - - - - - - -  - - - - - - - - - -  - - - - - - - - - -  - - - - - - - - - -
P914254       251 n n n n n n n n n n  n n n n n n n n n n  n n n n n n n n n n  n n n n n n n n n a  t - - - - - - - - -

alignment position 3 0 1 . . . . . . .  3 1 1 . . . . . . .  3 2 1 . . . . . . .  3 3 1 . . . . . . .  3 4 1 . . . . . . .
hNDUFS2       301 g c t g t g t t t c  a a c a a t a g a a  a g a a g g c c c a  g g g a a a g a g c  a a g g a a t g a g
P914253        10 - - - - - - - - - -  - - - - - - - - - -  - - - - - - - - - -  - - - - - - - - - -  - - - - - - - - - -
P914254       292 - - - - - - - - - -  - - - - - - - - - -  - - - - - - - - - -  - - - - - - - - - -  - - - - - - - - - -

alignment position 3 5 1 . . . . . . .  3 6 1 . . . . . . .  3 7 1 . . . . . . .  3 8 1 . . . . . . .  3 9 1 . . . . . . .
hNDUFS2       351 g a c a a c a g a g  g g a c c a a a c c  t c t g c c c t g g  c t c t g t g g c a  g c t g c c t t g t
P914253        10 - - - - - - - - - -  - - - - - - - - - -  - - - - - - - - - -  - - - - - - - - - -  - - - - - - - - - -
P914254       292 - - - - - - - - - -  - - - - - - - - - -  - - - - - - - - - -  - - - - - - - - - -  - - - - - - - - - -

alignment position 4 0 1 . . . . . . .  4 1 1 . . . . . . .  4 2 1 . . . . . . .  4 3 1 . . . . . . .  4 4 1 . . . . . . .
hNDUFS2       401 c t a g c c c a g g  t c c t g g t c a g  t t c c c a c a g c  t a a g g a t c t g  g c c c c a c c c t
P914253        10 - - - - - - - - - -  - - - - - - - - - -  - - - - - - - - - -  - - - - - - - - - -  - - - - - - - - - -
P914254       292 - - - - - - - - - -  - - - - - - - - - -  - - - - - - - - - -  - - - - - - - - - -  - - - - - - - - - -

alignment position 4 5 1 . . . . . . .  4 6 1 . . . . . . .  4 7 1 . . . . . . .  4 8 1 . . . . . . .  4 9 1 . . . . . . .
hNDUFS2       451 t t g g t a c a g t  t c c c a t t a g g  t c a a t t c c c a  g g c c a g a a g t  c t g g c t t t c c
P914253        10 - - - - - - - - - -  - - - - - - - - - -  - - - - - - - - - -  - - - - - - - - - -  - - - - - - - - - -
P914254       292 - - - - - - - - - -  - - - - - - - - - -  - - - - - - - - - -  - - - - - - - - - -  - - - - - - - - - -

alignment position 5 0 1 . . . . . . .  5 1 1 . . . . . . .  5 2 1 . . . . . . .  5 3 1 . . . . . . .  5 4 1 . . . . . . .
hNDUFS2       501 c c t c t t t c t c  t g c a g t g a a g  a a t t c c a c c a  t c t c t g c t t c  c t g g t c t g t c
P914253        10 - - - - - - - - - -  - - - - - - - - - -  - - - - - - - - - -  - - - - - - - - - -  - - - - - - - - - -
P914254       292 - - - - - - - - - -  - - - - - - - - - -  - - - - - - - - - -  - - - - - - - - - -  - - - - - - - - - -

alignment position 5 5 1 . . . . . . .  5 6 1 . . . . . . .  5 7 1 . . . . . . .  5 8 1 . . . . . . .  5 9 1 . . . . . . .
hNDUFS2       551 t g c c t c a c c c  t g c t g c g c t g  c t g g t g g g c t  g t g t c c c t g g  g a t c c t g t c t
P914253        10 - - - - - - - - - -  - - - - - - - - - -  - - - - - - - - - -  - - - - - - - - - -  - - - - - - - - - -
P914254       292 - - - - - - - - - -  - - - - - - - - - -  - - - - - - - - - -  - - - - - - - - - -  - - - - - - - - - -

alignment position 6 0 1 . . . . . . .  6 1 1 . . . . . . .  6 2 1 . . . . . . .  6 3 1 . . . . . . .  6 4 1 . . . . . . .
hNDUFS2       601 a g g g g a t a t c  t g g g g a a g g c  c t c t t t c c a g  g c t c a g a g t t  g g a g a g t t g t
P914253        10 - - - - - - - - - -  - - - - - - - - - -  - - - - - - - - - -  - - - - - - - - - -  - - - - - - - - - -
```

```
P914254             292 ---------- ---------- ---------- ---------- ----------

alignment position  651 ......     661 ......  671 ......  681 ......  691 ......
hNDUFS2             651 acaaactcta cgggtgggtg gcccagctgg agttgggctt agaggcagag
P914253              10 ---------- ---------- ---------- ---------- ----------
P914254             292 ---------- ---------- ---------- ---------- ----------

alignment position  701 ......     711 ......  721 ......  731 ......  741 ......
hNDUFS2             701 ggagaaaaca ggaactagcc tctaaggctg gaagaatctt tgtccataca
P914253              10 ---------- ---------- ---------- ---------- ----------
P914254             292 ---------- ---------- ---------- ---------- ----------

alignment position  751 ......     761 ......  771 ......  781 ......  791 ......
hNDUFS2             751 gaattgtgat cagactcctg gtgctactac atttccgtgc tgttggccat
P914253              10 ---------- ---------- ---------- ---------- ----------
P914254             292 ---------- ---------- ---------- ---------- ----------

alignment position  801 ......     811 ......  821 ......  831 ......  841 ......
hNDUFS2             801 tacccaggag ggctatagca gggtcagaaa gacgttaggg aaaagaggct
P914253              10 ---------- ---------- ---------- ---------- ----------
P914254             292 ---------- ---------- ---------- ---------- ----------

alignment position  851 ......     861 ......  871 ......  881 ......  891 ......
hNDUFS2             851 gacgcaagac ggaagtgggg tgtgaggcat ctctaagggc gcttcctggg
P914253              10 ---------- ---------- ---------- ---------- ----------
P914254             292 ---------- ---------- ---------- ---------- ----------

alignment position  901 ......     911 ......  921 ......  931 ......  941 ......
hNDUFS2             901 tctgtgattc tgtaggttcg ggaagcctgg aggatgtcag ggtcacccac
P914253              10 ---------- ---------- ---------- ---------- ----------
P914254             292 ---------- ---------- ---------- ---------- ----------

alignment position  951 ......     961 ......  971 ......  981 ......  991 ......
hNDUFS2             951 agaggacctc cactccccca tctgggatga ggtggctcct acacaccccg
P914253              10 ---------- ---------- ---------- ---------- ----------
P914254             292 ---------- ---------- ---------- ---------- ----------

alignment position  1001 ......    1011 ......  1021 ......  1031 ......  1041 ......
hNDUFS2            1001 acactcccct ccgcaccacc aatgccggcg gccttggcat cctattcttt
P914253              10 ---------- ---------- ---------- ---------- ----------
P914254             292 ---------- ---------- ---------- ---------- ----------

alignment position  1051 ......    1061 ......  1071 ......  1081 ......  1091 ......
hNDUFS2            1051 tttgttttgg tttggcggga gctctcagac ccgcccagcc ccacttaggc
P914253              10 ---------- ---------- ---------- ---------- ----------
P914254             292 ---------- ---------- ---------- ---------- ----------

alignment position  1101 ......    1111 ......  1121 ......  1131 ......  1141 ......
hNDUFS2            1101 tCCTTTCTCC AATCTCAATT TATGACATCT GGAAATTAGC tggctttccc
P914253              10 -CCTTCACAC AAGCCCGTTT CCGGACCTCT GGAAATGAAC a---------
P914254             292 ---------- ---------- ---------- ---------- ----------
                         ********* ********** ********** **********
alignment position  1151 ......    1161 ......  1171 ......  1181 ......  1191 ......
hNDUFS2            1151 aactcctgtC TTTTGGATTC AGTGATGGGA AAGTAATTGG CAAAGCCTGg
P914253              50 ---------C TTTTGGATTT CGTAGTAAAG AGATAGTCAT CAAAACTTGg
P914254             292 ---------- ---------- ---------- ---------- ----------
```

```
                        *  ********** ********** ********** *********
alignment position 1201...... 1211...... 1221...... 1231...... 1241......
hNDUFS2       1201 ggctacccta taagggcagg gctcagatac aaTCCGAGAG CAGGACTAAA
P914253         91 gggcttctg- ---------- ---------- --TCGGAAAG CAGGACTACC
P914254        292 ---------- ---------- ---------- ---------- ----------
                                                     ******* *********
alignment position 1251...... 1261...... 1271...... 1281...... 1291......
hNDUFS2       1251 GCATGAGGGC GGCCAAGGCG Gaagggagta gggaaggaag cgccgcgcgt
P914253        118 GCAGGAGACT GACCAAGATG Gtaagatagt caaggaacgc ccatacagag
P914254        292 ---------- ---------- ---------- ---------- ----------
                   ********** ********** *
alignment position 1301...... 1311...... 1321...... 1331...... 1341......
hNDUFS2       1301 ttccaagata cgcaggcggg ctcggc---- ---------- ----------
P914253        168 agtgcgtagg ggacctagag ggagcgcagt ttctaaggaa agttcgagtg
P914254        292 ---------- ---------- ---------- ---------- ----------

alignment position 1351...... 1361...... 1371...... 1381...... 1391......
hNDUFS2       1327 ---------- ---------- ---------- ---------- ----------
P914253        218 cagagcgtgg acctagcggg aagtgctgga ttctgcgcga agttgcctca
P914254        292 ---------- ---------- ---------- ---------- ----------

alignment position 1401...... 1411...... 1421...... 1431...... 1441......
hNDUFS2       1327 -GAGAGAGCA CGAAGTATCT GCCCCACGCA GGAACGGcaa ttttcccttg
P914253        268 gGAGAGAGCA GTAAATCTCT CACGCCCAGA GAAGCCGtgg gaggg-----
P914254        292 ---------- ---------- ---------- ---------- ----------
                    ********* ********** ********** *******
alignment position 1451...... 1461...... 1471...... 1481...... 1491......
hNDUFS2       1376 ctcgctccta gaaaagccag ccaggagctg tgggaggaaA CGCCCTCAGT
P914253        313 ---------- ---------- ---------- ---------A CGCCCTTACT
P914254        292 ---------- ---------- ---------- ---------- -GCCCTAACT
                                                              * *********
                                                              * *********
                                                                *********
                                                                *********
                                                                *********
                                                                *********
                                                                *********

alignment position 1501...... 1511...... 1521...... 1531...... 1541......
hNDUFS2       1426 AAAGATGACC GCGGTCACTG TTATCTAAAC GCAAGTGAAG CCGAGTCACA
P914253        324 AAAGATGACG GCAGTCGTCG TTATTGAAAC GCAAGTGAAG CTGAGAAGCG
P914254        301 AAAGATGACA GCAGTCGTTG TTATTTAAGC GtgAGTGAAG CTGAGAAGCA
                   ********** ********** ********** ********** **********
                   ********** ********** ********** ********** **********
                   ********** ********** ********** ********** **********
                   ********** ********** ********** *   ****** **********
                   ********** ********** ********** *   ****** **********
                   ********** ********** ****   **** *   ****** **********
                   ********** ********** ****   **** *   ****** **********
                                                     ****** **********

alignment position 1551...... 1561...... 1571...... 1581...... 1591......
hNDUFS2       1476 GGACCCGGAT GTTGTcagtt cgacggtaaa cgaCCCTGCC AGCTTCCAAG
P914253        374 GAATCCGGAT GTTGTTCCGT TCGCAATTGA CCTCCCTGCC AGCTTCCAAG
P914254        351 GGATCCGGAT GTTGTTCCGT TCGCAATTGA CCTTCCTGCC AGCTTCCAAG
                   ********** ********** ********** ********** **********
                   ********** ********** ********** ********** **********
                   ********** ********** ********** ********** **********
                   ********** ********** ********** ********** **********
                   ********** ********** ***            ****** **********
                   ********** *****                     ****** **********
                   ********** *****                      ***** **********
```

```
                        **********  *****                                   ******  **********
                                                                            ******  **********

alignment  position   1601......  1611......  1621......  1631......  1641......
hNDUFS2        1526   AGGGCGGCTT  CACTGTGCGA  AT-AGgtgag  aagccaagAA  GGAGGCGCGC
P914253         424   ATGGGGACTC  CGTTGTGgcg  ataAGAAGGG  AAGCCCACCA  GGGGGCGCGC
P914254         401   ATGGTGACTC  CATTGTGCCA  AT-AGAGGGG  AAGTCCATAA  GGAGGCGCGC
                      **********  **********  ** *******  **********  **********
                      **********  **********  ** *******  **********  **********
                      **********  **********  ** **               **  **********
                      **********  *******        **               **  **********
                      **********  *******                         **  **********
                      **********  *                                **  **********
                      **********  *                                *  **********
                      **********  *                                *  **********
                      **********  *                                *  **********

alignment  position   1651......  1661......  1671......  1681......  1691......
hNDUFS2        1575   TGGAGTTACT  TCCGCCCGGT  TCTCCTTCCc  gCAGTCTGCA  GCCGGAGTAA
P914253         474   GGGAGCGACT  TCCGCCCGGC  TCTCCTTCCT  ACAGTCTGCA  GCCCGGTAAA
P914254         450   GTGAGTCACT  TCCGCCCGGT  TCTCCTTCCT  AAGGTCTGCA  GCCCAGTAAA
                      **********  **********  **********  **********  **********
                      **********  **********  **********  **********  **********
                      **********  **********  **********  **********  **********
                      **********  **********  **********  **********  **********
                      **********  **********  *********    *********  **** *****
                      **********  **********  *********    *********  **** *****
                      **********  **********  *********      *******  ****  *****
                      *****  ***  **********  ********       *******  ****
                      *****  ***  **********  ********
                             ***  **********  ********

alignment  position   1701......  1711......  1721......  1731......  1741......
hNDUFS2        1625   GATGGCGGCG  CTGAGGGCTt  TGTGCGGCTT  CCGGGGCGTC  GCGGCCCAGG
P914253         524   GATGGCGGCC  CTGAGGGCGC  TGCGCTGCCT  CCGTGGGGTC  GGAGCCCCGG
P914254         500   GATGGCGGCC  CTCAGGGCTG  TGTGCAGCCT  CCGCGGTGTC  GGGGCCCAGG
                      **********  **********  **********  **********  **********
                      **********  **********  **********  **********  **********
                      **********  **********  **********  **********  **********
                      **********  *********   **********  **********  **********
                      **********  *********   **********  **********  **********
                      **********  ********    **********  *** ******  **********
                      **********  ********    **********  *** ******  **********
                                              **********  *** ******  **********

alignment  position   1751......  1761......  1771......  1781......  1791......
hNDUFS2        1675   TGCTGCGGCC  tGGGGCTGGA  GTCCGATTGC  CGATTCAGCC  CAGCAGGTGA
P914253         574   TGCTGCGGCC  CGGGTCTGGG  ATCCGCTTGC  CAAGTCAGCC  CAGCAGGTGA
P914254         550   TGCTGCGGGC  CGGGTCTGGG  ATTCGTCTGC  CGAGTCAGCC  CAGCAGGTGA
                      **********  **********  **********  **********  **********
                      **********  **********  **********  **********  **********
                      **********  **********  **********  **********  **********
                      **********  **********  **********  **********  **********
                      **********   *********  **********  **********  **********
                      ********     *********  **     ***  **********  **********
                      ********     *********  **     ***  **********  **********
                      ********     *********  **     ***  **********  **********
                                                     ***  **********  **********

alignment  position   1801......  1811......  1821......  1831......  1841......
hNDUFS2        1725   Gatcgagggc  agctctcgac  acacTTTCTC  CAAGGCTAGG  GTTTCTCAGG
P914253         624   GGAGGAGGCG  GtcCCTACAT  GCGGTCTCTG  CAGAACTGCA  ATGTCTCcac
P914254         600   GGAGGAGGCG  GctCCTACAT  GCGTTCTCTT  CAAAACTGGA  GTTTCTCGAG
                      **********  *  *******  **********  **********  **********
                      **********  *  *******  **********  **********  *******
                      **********  *
                      **********  *
                      *
                      *
                      *
```

```
                  *
                  *
alignment position 1851...... 1861...... 1871...... 1881...... 1891......
hNDUFS2      1775 Ttggggacgc tttact---- --------CC CCCAGAAAAT AATAACCCAA
P914253       674 TCTGAGGACA TTTCAAGGGC TCTGTGACCC CTGAGGAGAG AATGACCCAG
P914254       650 TCTGAGGACA TTTCAAGGGC TCTGTGACCC CTGAGGAAAG AATGACCCAG
                  ********** ********** ********** ********** **********
                  ********** ********** ********** ********** **********
                  ********** ********** ********** ******* ** **********
                  ********** ********** ********** ******* ** **********
                  ********** ********** ********** ******* ** **********
                  *                             ** ******* ** **********
                                                ** *******

alignment position 1901...... 1911...... 1921...... 1931...... 1941......
hNDUFS2      1813 GCCTGTGACC Ccattgtcca tc-CCTGAT- --TTCTTGGG CTACGGGATG
P914253       724 GCCTGTAACC CTCCTCACGC AGACCAGAtt tcTTCCTAGG TTCCTAGGTG
P914254       700 GCCTATAACC CTCCTTACGC TGACCAGAT- --TTCTTAGG TTCCTAGATG
                  ********** ********** *********     ******** **********
                  ********** ********** ********      ******** **********
                  ********** ********** ********      ******** **********
                  ********** ********** ********      ******** **********
                  ********** *             *****
                  ********** *

alignment position 1951...... 1961...... 1971...... 1981...... 1991......
hNDUFS2      1859 CAGtggcGCC CACTCTGGCG AGGGAGGGGA GAgggtctag agttcgttcg
P914253       774 CAGCCA-GCC TCCTGTGGCA AAGGATGGGA AATAAGGGAG ATGAATTTtt
P914254       747 CAGTCA-GCT TCCTGTGGCG GAGGATGGGA AGTAAGGGAG ATGATTTTat
                  ****** *** ********** ********** ********** ********
                  ****** *** *********   ********* ********** ********
                  ****** *** *********   ********* ********** ********
                  ***

alignment position 2001...... 2011...... 2021...... 2031...... 2041......
hNDUFS2      1909 tg-------- ---CCGAGGT GAAATCAGCG GAGTGGGAGA CTCTGCTTTT
P914253       823 at---TTTTT TGTCAGAAGC GAAATATGTG GGATGAAATA TCCTACCCTT
P914254       796 ttttaTTTTT TGTCAGAAGC GAAATATGTA GCGTGAAAGA CCCTACCCTT
                       ***** ********** ********** ********** **********
                       ***** ********** *********     ******* **********
                       ***** ********** *********

alignment position 2051...... 2061...... 2071...... 2081...... 2091......
hNDUFS2      1948 TAGcttcagT AAT----TTG AATGCACCTC TCCTGTCata cctgagtaga
P914253       870 CAGA-----T GAatggcTAG AGAGTACCTC CCATATCCGA AGGAGACCCC
P914254       846 CAAA-----T GAT----TAG AGCGTACCTC CCATATCCGA AGGAGACCCC
                  ****     * ***    *** ********** ********** **********
                  ****     * **     *** ********** ********** **********
                                    *** ********** ********** **********
                                    *** ********** ********** **********

alignment position 2101...... 2111...... 2121...... 2131...... 2141......
hNDUFS2      1994 gctcagccaa agagccaga- ---------- ---------- ----------
P914253       915 -CTCTTCTCT GAGCCCTCTT TCCCCAGTTC Cct------C TTTTTTGCTC
P914254       887 tCTCTTTTCT GAGCCCTCCT TCCCCAGTTC CtcttcttcC TCTTTCGCTC
                   ********* ********** ********** *        * **********
                   ********* ********** ********** *
                   ********* ********** ********** *

alignment position 2151...... 2161...... 2171...... 2181...... 2191......
hNDUFS2      2013 ----ATGCT- -------CGC TGTCTCTCTg tcgttgacct tgaacaaaca
P914253       958 GCCTATGCT- -------CTT TCCCTCTCTC CTCCCGCCTT CCATACTCCC
P914254       937 GCATATGCga ggttgcaCTT TCCCTCTTTC CTCCCGCCTT CCCTACTCCC
                  *********         *** ********** ********** **********
                                    *** ********** ********** **********
                                    *** ********** ********** **********
                                    *** ********** ********** **********

alignment position 2201...... 2211...... 2221...... 2231...... 2241......
```

```
hNDUFS2    2051 c t t t t ct c ac  g t t c a t a t t c  a c c t g g t t g a  t a a t a t c a a t  a t g a t g a a a a
P914253    1000 t g - - - - - - - -  - - - - - - - - - -  - - - - - - - - - -  - - - - - - - - - -  - - - - - - - - - -
P914254     987 c c c g c c c c c a  t c t c t - - - - -  - - - - - - - - - -  - - - - - - - - - -  - - - - - - - - - -

alignment  position  2 2 5 1 . . . . . .  2 2 6 1 . . . . . .  2 2 7 1 . . . . . .  2 2 8 1 . . . . . .  2 2 9 1 . . . . . .
hNDUFS2    2101 t a t  g a a g a a g  c t a g g a t c c c  a a c c t t g c a t  g c c c a g g g g t  g a t  g g g t  g g a
P914253    1002 - - - - - - - - - -  - - - - - - - - - -  - - - - - - - - - -  - - - - - - - - - -  - - - - - - - - - -
P914254    1002 - - - - - - - - - -  - - - - - - - - - -  - - - - - - - - - -  - - - - - - - - - -  - - - - - - - - - -

alignment  position  2 3 0 1 . . . . . .  2 3 1 1 . . . . . .  2 3 2 1 . . . . . .  2 3 3 1 . . . . . .  2 3 4 1 . . . . . .
hNDUFS2    2151 g t t  a t  t c c a g  g a a t a a t t t g  t t a g t a g g a a  t g g a a c c t c t  a g a g t t c c a g
P914253    1002 - - - - - - - - - -  - - - - - - - - - -  - - - - - - - - - -  - - - - - - - - - -  - - - - - - - - - -
P914254    1002 - - - - - - - - - -  - - - - - - - - - -  - - - - - - - - - -  - - - - - - - - - -  - - - - - - - - - -

alignment  position  2 3 5 1 . . . . . .  2 3 6 1 . . . . . .  2 3 7 1 . . . . . .  2 3 8 1 . . . . . .  2 3 9 1 . . . . . .
hNDUFS2    2201 c t t  g t  t t t  g c  c g a a g c t  a c a  t t a a c t t  c a g  a t c a g t g a c t  t g g c  a t t  a a a
P914253    1002 - - - - - - - - - -  - - - - - - - - - -  - - - - - - - - - -  - - - - - - - - - -  - - - - - - - - - -
P914254    1002 - - - - - - - - - -  - - - - - - - - - -  - - - - - - - - - -  - - - - - - - - - -  - - - - - - - - - -

alignment  position  2 4 0 1
hNDUFS2    2251 a a
P914253    1002 - -
P914254    1002 - -
```

Pairwise similarities:

For each pairwise alignment, the similarity (relative to the maximum similarity) and the number of identical nucleic acids (in % of shorter sequence) is given. Maximum values are underlined.

	P914253 (1001 bp)	P914254 (1001 bp)
hNDUFS2 (2252 bp)	0.307<br>39 %	0.324<br>32 %
P914253 (1001 bp)		1.000<br>57 %

Please note that the **similarity value 1.000** marks only the two **most similar** sequences, it does not necessarily mean that these sequences are identical.

Extract alignment region	
**Positions in alignment**	from: to:
**Output file**	dialign.seq

Extract aligned sequences

For comments, questions or bug reports, please contact support@genomatix.de

© Genomatix Software GmbH 1998-2005 - All rights reserved. License Agreement GEMS Launcher 4.1.1

Genomatix-Logo Logout Personal Messages GEMS-Logo
Logout Personal Messages

Genomatix Main GEMS ElDorado Gene2Promoter BiblioSphere
Frequently asked questions Your Results Your Sequences Your Protocol Help

GEMS Launcher Task: *DiAlign TF*: Multiple alignment plus TF sites
working on promoters.seq (3 seq.)

[Alignment] [Pairwise similarities]

DiAlign professional TF Release 3.1 December 2004 **Tue Jun 7 20:56:18 2005**

Solution parameters:

**Sequence file:**	promoters.seq (3 seq.)
**Type of sequences:**	nucleotide sequences
**Threshold T:**	0.00
**'*' signs below alignment denote**:	diagonal similarity (max. similarity is represented by 10 '*' signs)
**Alignment output:**	complete alignment is shown<br># nucleic acids per line: 50
**TF output:**	common TF matches located in aligned regions (common to 3 (100.0 %) sequences)
**Family matches:**	yes
**MatInspector library:**	Matrix Family Library Version 5.0 (February 2005)
**Selected groups** (core/matrix sim):	• ALL vertebrates.lib (0.75/Optimized)

Aligned Sequences:

No.	Sequence Name	Sequence Description	Length
1	P878333	sym=NDUFS3|loc=Loc4722|taxid=9606|spec=Homo sapiens|chr=11| ctg=NT_009237|str=(+)|start=46387373|end=46388373|len=1001| tss=501| comm=NADH dehydrogenase (ubiquinone) Fe-S protein 3, 30kDa (NADH-coenzyme Q reductase); (NM_004551/560/silver;)	1001 bp
2	P878334	sym=na|loc=Loc433454|taxid=10090|spec=Mus musculus|chr=2| ctg=NT_039207|str=(-)|start=31738929|end=31740003|len=1075| tss=501,530,575| comm=similar to NADH-ubiquinone oxidoreductase 30 kDa subunit, mitochondrial precursor (Complex I-30KD) (CI-30KD); (AK088337/501/gold;AK002501/530/gold;XM_485037/575/bronze;)	1075 bp
3	P878335	sym=na|loc=Loc295923|taxid=10116|spec=Rattus norvegicus| chr=3|ctg=NW_047657|str=(-)|start=16664085|end=16665085| len=1001|tss=501| comm=similar to NADH-ubiquinone oxidoreductase 30 kDa subunit, mitochondrial precursor (Complex I-30KD) (CI-30KD); (XM_215776/550/bronze;)	1001 bp

Alignment (DiAlign format):

Please note that only **upper-case** letters are considered to be **aligned**. For more information, please have a look at the user guide.

V$XBBF V$VMYB V$AREB V$SP1F V$RREB V$ZBPF V$EGRF V$GABF V$ETSF V$ZF35 V$EREF V$MOKF

```
alignment
position         1 . . . . . . . . .   1 1 . . . . . . . .   2 1 . . . . . . . .   3 1 . . . . . . . .   4 1 . . . . . . . .
P878333    1  - - - - - - - - - -   - - - - - - - - - -   - - - - - - - - - -   - - - - - - - - - -   - - - - - - - - - -
P878334    1  c c a a t a c t g a   g g c t t g g g c c   t g g a c t t c c a   c t t t t a c t c t   t t t c t a a g c T
P878335    1  - - - - - - - - - -   - - - - - - - - - -   - - - - - - - - - -   - - - - - - - - - -   - - - - - - - - - T
```

```
                                                                    *
                                                                    *
alignment
position       51........ 61........ 71........ 81........ 91........

P878333     1  ---------- ---------C TCGCTCGAAA GACTCCCTAT GGCTGCGTcc
P878334    51  GCTGTTTCTT ACTTACCT-C TCGCTCCAGT GAGTTCTTAA AGAAGCCctt
P878335     2  GCTGTTTCTT ACTTACCTaC TCGCTCCAAT GAGTTCTTAA AGAAGCCTga
               ********** ******** * ********** ********** ********
               ********** ******** * ********** ********** *******
                                   * ********** ********** *******

alignment
position       101....... 111....... 121....... 131....... 141.......

P878333    32  cagaacttGA GTCCATCCGC CCCCAGGCCG GGGCACTGGG AGGCCCATCA
P878334   100  GA------GA GCCCAGCCGT CCCCGGCCGG GGGCACAAGG AAGTCCATAA
P878335    52  GA------GA GCCCAGCCGT CCCCGGCCGG GGGCACAAGG AAGTCCATAA
               **      ** ********** ********** ********** **********
               **      ** ********** ********** ********** **********
               **      ** ********** ********** ********** **********
               **      ** ********** ********** ********** **********
               **      ** ********** ********** ********** **********
                                     ********** ********** **********
                                     ********** *****   ** **********
                                                        ** ******

alignment
position       151....... 161....... 171....... 181....... 191.......

P878333    82  CCCTGTGCAA TGTCCCCGCT CCACCTTGAG TCGTCTTGGA AACGGTCGCC
P878334   144  CCCTGTGCAA TCTGACCGTG CCACCTGGAG TCGCCTAGGA AACGGTCTCC
P878335    96  CCCTGTGCAA TCTGACCGCG CCACCTAGAG TCGCCTAGGA AACGGTCTCC
               ********** ********** ********** ********** **********
               ********** ********** ********** ********** **********
               ********** ********** ********** ********** **********
               ********** ********** ********** ********** **********
               ********** ********** ********** ********** **********
               **********            ********** ********** **********
               **********            ********** ********** **********
                                     ****** *** ********** **********
                                     ****** *** ********** **********
                                                     ***** **********

alignment
position       201....... 211....... 221....... 231....... 241.......

P878333   132  AGGGAAACCG TTCTTCccgc tcaggcccg- --CCCGCCCT CGTCAAAA--
P878334   194  AAGGAAACCG TCCATCGTCT CCCCCcccca CCCCGCCCCC CATTAAAAaa
P878335   146  AAGGAAACCG TCCATCGACT CCCCCac--- CCCCGCCCCC CATTAAAA--
               ********** ********** *****      ********** ********
               ********** ********** *****      ********** ********
               ********** ********** *****
               ********** ********** *****
               ********** ******
               ********** ******
               **********
               **********
               **********

alignment
position       251....... 261....... 271....... 281....... 291.......

P878333   177  GCTTGGCTCA GaGAGCGGGG GAGGGGTGTG TAGCGTAGAA CCCAAAACGC
P878334   244  GCTTAGCTCA A-GAGCCGGc --GCGGTGTA GAACTTAGAA CCCAGAACGC
P878335   191  GCTTAGCTCA G-GAGCCAGA GAGCGGTGTA GAACTTAGAA CCCAGAACGC
               ********** * ******** ********** ********** **********
               ********** * *******    ******** ********** **********
                                       ******** ********** **********
                                       ******** ********** **********
                                       ******** ********** **********
                                                     ***** **********
                                                     ***** **********
                                                                *****
```

```
alignment
position        301.......  311.......  321.......  331.......  341.......

P878333    227  CCGCCCCCTC  CCCTCCTCTC  ACCCGCCTGG  TTCTTcagcg  tCCTCGCCTT
P878334    291  CCACCCTTTC  CCTTTCTCTC  ACCGGTGCGG  TTCGTTG---  -CGTCTTCTT
P878335    240  CCACCCTTTC  CCTTTCTCTC  ACCCGTCCGA  TTCTTTG---  -CGTCTTCTT
                **********  **********  **********  ******       *********
                **********  **********  **********  ******       *********
                **********  **********  **********  *****              ***
                **********  **********  **********  *****              ***
                **********  **********  ***
                **********  **
                **********  **
                **********  **

alignment
position        351.......  361.......  371.......  381.......  391.......

P878333    277  CTCTCTAGGC  TCTGCCACAA  GGAGCTAGGA  CCACGCTC-A  CCTCACGATT
P878334    337  CTCTTCGGCC  TCCGCTGTAC  CGAGATAGGG  CTACGTAC-A  CCTCACGGTT
P878335    286  CTCTTCGGCC  TCCGCCGTAC  CAAAATAGGG  CTACGTAgaA  CCTCACGGTT
                **********  **********  **********  ******** *  **********
                **********  **********  **********  ******** *  **********
                **********  **********  **********  *******  *  **********
                **********  **********  **********  *******  *  **********
                                         *********  *******  *  **********
                                                             *  **********
                                                             *  **********

alignment
position        401.......  411.......  421.......  431.......  441.......

P878333    326  TCCCTACCTG  CctgtctcgC  TTTCTCACCT  GCGTTCCCTC  CTTTCATCCC
P878334    386  TCCCCACCTG  CGGGT----C  TTTCTTACCT  GCGATACCTC  CTTTCATGCC
P878335    336  TCCCCACCTG  CGGGT----C  TTTCTTACCT  GCGTTACCTC  CTTTCATGCC
                **********  *****    *  **********  **********  **********
                **********  *****    *  **********  **********  **********
                **********  *****    *  **********  **********  **********
                **********  *****    *  **********  **********  **********
                **********  *****    *  **********  *** ******  **********
                **********  *        *  **********  ***
                **********  *        *  **********  ***
                                     *  **********  ***

alignment
position        451.......  461.......  471.......  481.......  491.......

P878333    376  GGAGC-----  -CCGGAACCT  CCGCTTCCGG  CTCCACGTCC  GCCCGGAAGA
P878334    432  AGGGCTCAGA  TCCGGAACCT  CTGCTTCCCG  CCCCGCGTCC  GCCCGGAAGA
P878335    382  AatgtTCAGA  TCCGGAACCT  CTGCTTCCCG  CCCCGTGTCC  GCCCGGAAGA
                **********  **********  **********  **********  **********
                **********  **********  **********  **********  **********
                *    *****  **********  **********  **********  **********
                             *********  **********  **********  **********
                             *********  **********  **********  **********
                             *********  **********  **********  **********
                             *********  **********  ***** ****  **********
                             *********  **********  ***** ****  **********
                             *********  **********  ***** ****  **********

alignment
position        501.......  511.......  521.......  531.......  541.......

P878333    420  AgatctgCTG  CACACTTCCG  TTTCCGGTCC  GTGCCCTTGG  GGCTCCGTgt
P878334    482  AGAGTCACTG  CACATTTCCA  CTTCCGGTCC  GTGCCCTTGG  GGCTCCGTGG
P878335    432  AGATTCATTG  CACATTTCCA  CTTCCGGTCC  GTGCCCTTGA  GGCTCCGTGG
                **********  **********  **********  **********  **********
                **********  **********  **********  **********  **********
                **********  **********  **********  **********  **********
                **********  **********  **********  **********  ********
                *      ***  **********  **********  **********  ********
                *      ***  **********  **********  **********  ********
                *      ***  **********  **********  **********  *******
                *        **  **********  **********  **********  *******
                *        **  **********  **********  **********  *******
```

```
                    **  *********  ******

alignment
position        551.......  561.......  571.......  581.......  591.......

P878333     470 cctgc TGTCT TTCCGTCCGC TGCCTAGTCT GCAt-CTGAG TAACATGGCG
P878334     532 CCTG- CGTGT GTTCTCCGGC TACTTAGTCG GCACACTAAG GAACATGGCG
P878335     482 CCTG- TGTGT GTTTTCCGGC TACTTAGTCG GCACCCTAAG GAACATGGCG
                ****  ***** ********** ********** ********** **********
                ****  ***** ***  ***** ********** ********** **********
                ****  ***** ***  ***** ********** ********** **********
                                 ***** ********** ********** **********
                                                       ***** **********

alignment
position        601.......  611.......  621.......  631.......  641.......

P878333     519 GCGGCGGCgg tAGCCAGGCT GTGGTGGCGC GGGATCTTGG GGGCCtcggc
P878334     581 GCGGCTGC-- -AGCCAGGGT CTGGTGTCGT GGGCTCTTGG GGGCCGCTTC
P878335     531 TCTGCTGC-- -AGCCAGGGT CTGGTGCCGT GGGCTCTTGG GGGCtGCTTC
                *******     ******** ********** ********** **********
                   *****    ******** ********** ********** **********
                   *****    ******** ********** ********** ****  *****
                   *****    ******** ********** ********** ****
                   **       ******** ********** ********** ****
                            ******** ********** ********** ****
                            ******** ********** ********** ****

alignment
position        651.......  661.......  671.......  681.......  691.......

P878333     569 gctgaCCAGG GGTGAGCACG Gg-CAGCCA- --GCTGAGAC CGGGGTCAGG
P878334     628 CGTAGGCAGG GGTGAGCAAG GAACAGCAGc agGTTGAGAC CACGGGCAGC
P878335     578 CGTAGCCAGG GGTGAGCAAA GAACAGCAG- --GCCGAGAC CACGGGCAGC
                ********** ********** *********    ******* **********
                ********** ********** *********        ***** **********
                ********** ********** *********
                      **** ********        ******

alignment
position        701.......  711.......  721.......  731.......  741.......

P878333     615 CGCAGCGGcg tgcccagtgC AGAGAGCTCC TCAGGGCTCA tCCCGCGCGT
P878334     678 a--------- ---------C GGAGACCTCC CCGAGTCTCA -CCCGCGCCT
P878335     625 GGCAGAGGca cacctt gcaC ATAGACCTCC TCGGGTCTCA -CCCGCGCCT
                *******            * ********** **********  *********
                                     ******** **********  *********
                                     ******** **********  *********
                                     ******** **********  *********
                                                              *********
                                                              *********
                                                              *********
                                                              *********
                                                              *********

alignment
position        751.......  761.......  771.......  781.......  791.......

P878333     665 CTGTGCCTTT TATCTCCCTG TGCAGGGaCT GGGCGACCCT CCGTTCTGTT
P878334     709 TTGTGCCTTT TCTCTCCCTG TGCAGGGGCT GGGCGACCCT CCGTGCTGTG
P878335     674 TTGTGCCTTT TATCTTCCTG TGCAGGGGCT GGGCGACCCT CAGTGCTGTT
                ********** ********** ********** ********** **********
                ********** ********** ********** ********** **********
                ********** ********** ********** ********** **********
                ********** ********** ********** ********** **********
                ********** ********** *******  ** ********** **********
                ********** ********** *******  ** ********** **********
                ********** *      **** *******
                ********** *      **** *******
                ********** *      **** *******

alignment
position        801.......  811.......  821.......  831.......  841.......

P878333     715 GCTGCCG--- GTGAGGCGGG AGAGCGCCGG GGCCGACACG CGCCGTGAGT
P878334     759 GCAGCAC--- GTAAGAAGGG AGAGCGCGGC GGCTGACAAG CGCCGTGAGT
```

```
P878335       724 GCAGCAgcgt GTGAGAAGGG AGAGCGCGGC GGCTGACAAG CGCCGTGAGT
                  *******    ********** ********** ********** **********
                  *******    ********** ********** ********** **********
                  ******        ******* ********** ********** **********
                  ******        ******* ********** ********** **********
                  ******        ******* ********** ********** **********
                  **            **  *** ********** ********** **********
                  **            **  *** ********** ********** **********
                                    *** ********** ********** **********
                                                       ****** **********
                                                       ****** **********

alignment
position              851.......  861.......  871.......  881.......  891.......

P878333       762 ATGTGCGGGC AGCGCTCTTC TCTGAACTAT CGGCGGGGCC CCTT------
P878334       806 ATGTGCGGAT GGCGCTTTTT CCCCCAACGT TAGCTGGGGC CTTTcgTTGT
P878335       774 ATGTGCGGAT AGCGCTCTTC TCTTCAACGT TAGCAGGGGT CTTccaTTGT
                  ********** ********** ********** ********** ****  ****
                  ********** ********** ********** ********** ***   ****
                  ********** ********** ***                         ****
                  ********** ********** ***
                  ********** ********** ***
                  *******
                  *******
                  *******
                  ***
                  ***

alignment
position              901.......  911.......  921.......  931.......  941.......

P878333       806 -------AGA TGCCTGTCCT TGCCCTAGGA TGCCCTTCCC TacgtcctCC
P878334       856 TTCTGTCAGA TGTTTGTCCT TGCCTTGGAA TGTCCTTCCC T-------CC
P878335       824 TTCTGCCAGA TGTTTGTCCT TGCCCTGGAA TGTCCTTCCC T-------TC
                  ********** ********** ********** ********** *       **
                  ********** ********** ********** ********** *       **
                  ********** ********** ********** ********** *       **
                         *** ********** ****  ***** ********** *       **
                         *** ********** ****  ***** ********** *
                                             ***** ********

alignment
position              951.......  961.......  971.......  981.......  991.......

P878333       849 TGGCAAATCT tggagatctg ac-----TTG CAGTGGTCTT CACCCTGCAT
P878334       899 TGGCAAATCT CTGCTTGtCC TTGGAGATTG CTATGGTCTC CACTTTGCTG
P878335       867 TGGCAAATCT CTGCTTGcCC TTGGAGATTG CTATGGTCTC CACCTTGCTG
                  ********** ******* ** ********** ********** **********
                  ********** ******* ** ********** ********** **********
                  ********** ******* ** ********** ********** **********
                  ********** ******* ** ********** ********** **********
                                                      ******* **********

alignment
position              1001......  1011......  1021......  1031......  1041......

P878333       894 GCAGAATCGA GCCCTCCCTG TGCCCTtagg cctgttatgg c------CCT
P878334       949 TTAGAATCGA GCACTTCCTG TGCCCTAGAT ATTTGTTCCC TTCTGATCTT
P878335       917 TTAGAAACGA GTACTTCCTG TGCCCTAGAT ATTTGTTCCC TTCTGATCTT
                  ********** ********** ********** ********** **********
                  ********** ********** ********** ********** **********
                  ********** ********** ********** ********** *******
                  ****** *** ********** ********** ********** *******
                  ****** *** ********** ******
                    **** *** ********** ******
                         *** ********** ***

alignment
position              1051......  1061......  1071......  1081......  1091......

P878333       938 GATTGTTCCT TGTCCATTTC AATCATCCCC ---------T TTTTGTCTGA
P878334       999 GATCGCCTTT TGCTCGTTAT CAggatttga tttttttttT TGTCCTCCGA
P878335       967 GATCGCCCTT TGCTCGTTTT CACAATCTTC ---------T ATTT------
```

```
                   * * * * * * * * * *  * * * * * * * * * *  * * * * * * * * * *              *  * * * * * * * * * *
                   * * * * * * * * * *  * * * * * * * * * *  * *

alignment
position           1 1 0 1 . . . . . .  1 1 1 1 . . . . . .  1 1 2 1 . . .

P878333      979   T T A A A C T G T C  a g c t  c t  t t g a  a a g - - - -
P878334     1049   T G A A A T T G T C  t t t g a g c g c a  g g t a a c a
P878335     1002   - - - - - - - - - -  - - - - - - - - - -  - - - - - - -
                   * * * * * * * * * *
```

Pairwise similarities:

For each pairwise alignment, the similarity (relative to the maximum similarity) and the number of identical nucleic acids (in % of shorter sequence) is given. Maximum values are underlined.

	P878334 (1075 bp)	P878335 (1001 bp)
P878333 (1001 bp)	0.356<br>60 %	0.366<br>64 %
P878334 (1075 bp)		1.000<br>88 %

Please note that the **similarity value 1.000** marks only the two **most similar** sequences, it does not necessarily mean that these sequences are identical.

**Extract alignment region**	
**Positions in alignment**	from: to:
**Output file**	dialign.seq

Extract aligned sequences

For comments, questions or bug reports, please contact support@genomatix.de

© Genomatix Software GmbH 1998-2005 - All rights reserved. License Agreement GEMS Launcher 4.1.1

Genomatix-Logo Logout Personal Messages GEMS-Logo
Logout Personal Messages

Genomatix Main GEMS ElDorado Gene2Promoter BiblioSphere
Frequently asked questions Your Results Your Sequences Your Protocol Help

GEMS Launcher Task: *DiAlign TF*: Multiple alignment plus TF sites
working on C1-NDUFS4 (3 seq.)

[Alignment] [Pairwise similarities]

DiAlign professional TF Release 3.1 December 2004 **Tue Jun 7 22:10:52 2005**

Solution parameters:

**Sequence file:**	C1-NDUFS4 (3 seq.)
**Type of sequences:**	nucleotide sequences
**Threshold T:**	0.00
**'*' signs below alignment denote:**	diagonal similarity (max. similarity is represented by 10 '*' signs)
**Alignment output:**	complete alignment is shown<br># nucleic acids per line: 50
**TF output:**	common TF matches located in aligned regions (common to 3 (100.0 %) sequences)
**Family matches:**	yes
**MatInspector library:**	Matrix Family Library Version 5.0 (February 2005)
**Selected groups** (core/matrix sim):	• ALL user_defined.lib (0.75/Optimized)<br>• ALL vertebrates.lib (0.75/Optimized)

Aligned Sequences:

No.	Sequence Name	Sequence Description
1	P855897	P855897 sym=NDUFS4|loc=Loc4724|taxid=9606|spec=Homo sapiens|chr=5|ctg=NT_006713|str=(+)|start=3450344|end=3451344|len=1001|tss=501|comm=NADH dehydrogenase (ubiquinone) Fe-S protein 4, 18kDa (NADH-coenzyme Q reductase); (NM_002495/516/bronze;)
2	P855898	P855898 sym=Ndufs4|loc=Loc17993|taxid=10090|spec=Mus musculus|chr=13|ctg=NT_039590|str=(-)|start=22613656|end=22614693|len=1038|tss=501,533,538|comm=NADH dehydrogenase (ubiquinone) Fe-S protein 4; (AK011124/501/gold;AK020552/533/gold;NM_010887/538/bronze;)
3	rNDUFS4	rNDUFS4 n3_dna range=chr2:46407330-46408355 5'pad=0 3'pad=0 revComp=TRUE strand=? repeatMasking=none

Alignment (DiAlign format):

Please note that only **upper-case** letters are considered to be **aligned**. For more information, please have a look at the user guide.

V$NFAT V$AP4R V$BRNF V$NKXH V$MYOD V$FKHD V$ETSF V$YY1F V$NEUR V$TALE V$WHZF V$RORA
V$SP1F

```
alignment
position        1 . . . . . . . . .  1 1 . . . . . . . .  2 1 . . . . . . . .  3 1 . . . . . . . .  4 1 . . . . . . . .
P855897     1   t t t c g t t - - -  - - - - - - - - - -  - - - - - - - - - -  - - - - - - - T G G  A A A A T A C G A A
P855898     1   c - - - T T T T T T  T T T T T T T G C G  A G G C T A G C T T  T T T A G T G T G G  A A A A T A T G A T
rNDUFS4     1   t t t t T T T T T T  A T C T T T G G C G  A G G C C A G A T T  T T T A G T G T G G  A A A A T A T G A T
```

```
                                 ****** ********** ********** ********** **********
                                 ****** ********** ********** ********** **********
                                                                     *** **********
                                                                     *** **********
                                                                     *** **********
                                                                     *** **********
                                                                      ** **********
                                                                      ** **********
                                                                      ** **********

alignment position     51........ 61........ 71........ 81........ 91........
P855897            21 CATTACTACT ACAGAATATC AAACTGAata cagcCCTGTC CTGAGGGCTT
P855898            48 TATTATAACT ACAGACCATC AAACTGAGTG CAG-CCTGTT CTGAAGGCTT
rNDUFS4            51 TATTATAACT ACAGACCATC AAACTGAGTG CAG-CCTGTT ATGAAGGCTT
                      ********** ********** ********** ***    ****** **********
                      ********** ********** ********** ***    ****** **********
                      ********** ********** ********** ***    ****** **********
                      ********** ********** ********** ***    ****** **********
                      ********** ********** ********** ***    ****** **********
                      ********** ********** *******           ****** **********
                      ********** ********** *******           ****** **********
                      ********** ********** *******           ****** **********
                      ********** ********** *******

alignment position     101....... 111....... 121....... 131....... 141.......
P855897            71 GCAAAGTGAA TCTGGCAAtA CTTTGGAAAA GTACTACTGC AGGTTCTCAA
P855898            97 GCAAACGGAA TCTGGCAACA CTTTGGGAAA GTACTACTTT AGTTTCTCAA
rNDUFS4           100 GCAAACGGAA TCTGGCAACA CTTTGGGAAA GTACTACTTT AGTTTCTCAA
                      ********** ********** ********** ********** **********
                      ********** ********** ********** ********** **********
                      ********** ********** ********** ********** **********
                      ********** ********** ********** ********** **********
                      ********** ********** ********** ********** **********
                      ********** ******** * ********** ********** **********
                      ********** ******** * ********** ********** **********
                      ********** ******** * ********** ********** **********
                                          * ********** ********** **********

alignment position     151....... 161....... 171....... 181....... 191.......
P855897           121 GGACCCAAaG AGCTCTCCAG CTTTTCCAGT GGGTTCAGCC CCAAATATcC
P855898           147 AGACTCAAGa AGCCCTCCGG CTCTTCCAGT GGGCCCAGCC TCAGATATTC
rNDUFS4           150 AGACTCAAGG AGCCCTCCGG CTCTTCCAGT GGGCTCAGTC TCAGATATTC
                      ********** ********** ********** ********** **********
                      ********** ********** ********** ********** **********
                      *********  ********** ********** ********** **********
                      *********  ********** ********** ********** **********
                      *********  ********** ********** ********** ******** *
                      ********   ********** ********** ********** ******** *
                      ********   ********** ********** ********** ********
                      ********   ********** ********** ********** ********
                      ********

alignment position     201....... 211....... 221....... 231....... 241.......
P855897           171 TAATTTCTTC AATAAATTAC GAGTTGCAGG TGTTTAA-T atgcaag---
P855898           197 cGAGTTCTTT GAAAAATTAT GAGTTACAGG TGTTTAcatT TTTCGAAGCG
rNDUFS4           200 TGAGTTCTTT GAAAAATTAT GAGTTACAGG TGTTTATA-T TTTTGAAGTG
                      ********** ********** ********** ******** * **********
                      ********** ********** ********** ******** * **********
                       ********* ********** ********** ******   * **********
                       ********* ********** ********** ******   *
                       ********* ********** ********** ******   *
                       ********* ********** ********** ******
                          ****** ********** ********** ******

alignment position     251....... 261....... 271....... 281....... 291.......
P855897           217 -TATATTTTG GTCTTTTCGT TATCATTGTA AAACACCAgt cctaacaagc
```

```
P855898    247 TTTTCTTTTG GGCTTCTCAG CCTCAATGTA AATGACCAAC CTTACTAAC-
rNDUFS4    249 TTTTCTTTGG GGCTTCTCAG CCTCAGTGTA AATGACCAAC CTTACTAAC-
               ********** ********** ********** ********** *********
               ********** ********** ********** ********** *********
               ********** ********** *****  *** ********** *********
                ********* ********** *****  *** ********** *********
                                           **** ********

alignment
position       301....... 311....... 321....... 331....... 341.......

P855897    266 agtAAAGATT ACACTTTATG GTGGTAATAT TTACAGTTAA TGcacttctg
P855898    296 ---AAGGATT ACTTTCTATA GAGGCATCAT TTGCATTCAC TGagtcc-TT
rNDUFS4    298 ---AAGTATT ACTTctc--- ---------- ---CATTCAC TaaattttTT
                  ******* ********** ********** ********** **      **
                  ******* ****                                     **
                  ******* ****
                  ******* ****

alignment
position       351....... 361....... 371....... 381....... 391.......

P855897    316 catttcttcc cgcaCATTTT TCTTCTTACT TATTTAAACT AAGCGTTTcc
P855898    342 TCATC----- ----CATTTC CCTCTTTAGT TCTTTCAGCT AGAACTTTAA
rNDUFS4    329 TCATC----- ----CATTTT CCTCCTTATT TCCTTTAACT AGAtCTTTTA
               *****          ****** ********** ********** **********
               *****          ****** ********** ********** ***
                              ****** ********** ********** *

alignment
position       401....... 411....... 421....... 431....... 441.......

P855897    366 cCTCTTTTTT TAAGCCCCAG TATTAAAAAG AAAATAAAat cccaaagaat
P855898    383 TTTCTTTTCT AGGTCCTCAA TCGCAAAAGG AAAACAAACA GAACATGcct
rNDUFS4    370 TCTCTTTTCT AGACTCTCAA TATCAAAAAG AAAACAAACA CAACATGatt
               ********** ********** ********** ********** *******
                ********* ********** ********** ********** *******
                               ***** ********** ********
                               ***** ********** ********

alignment
position       451....... 461....... 471....... 481....... 491.......

P855897    416 ttcgcaacat tgcgattGCA CTGGCTTGAG AACGAAGGAA GTTAGTCCCT
P855898    433 ctgtttcacT GCGGCC-GCA TTGCTTTGAG AACGGAGGAA GTGAGTCCCT
rNDUFS4    420 tgtgtttt-T GCGGCC-GCA CTGCTTTGAG AACGAAGGAA GTGAGTCCCT
                        * ****** *** ********** ********** **********
                        * ****** *** ********** ********** **********
                        * ****** *** ********** ********** **********
                        * ****** *** ********** ********** **********
                                 *** ********** ********** **********
                                 *** ********** ********** **********
                                          ***** ********** **********
                                          ***** ********** **********
                                          ***** ********** **********

alignment
position       501....... 511....... 521....... 531....... 541.......

P855897    466 TGCGGTGATC CGTCCTTTCA TCCTGGCGTT TGCCTGCAGC AAGATGGCGG
P855898    482 AGCGGGGATC CGTACTCGCA TCCTGGCGTT TGCCTACAGG AAGATGGCGG
rNDUFS4    468 AGCGGGGATC CGTACTCCCA TCCTGGCGTT TGCCCACAGG AAGATGGCGG
               ********** ********** ********** ********** **********
               ********** ********** ********** ********** **********
               ********** ********** ********** ********** **********
               ********** ********** ********** ********** **********
               ********** ********** ********** ********** **********
               ********** ***     ** ********** ********** **********
                ********* ***     ** ********** ****  **** **********
                ********* ***     ** ********** ****  **** **********
                                                      **** **********

alignment
position       551....... 561....... 571....... 581....... 591.......
```

```
P855897     516 CGGTGTCAAT GTCAGTGGTA CTGAGGCAGA CGTTGTGGCG GAGAAGGGCA
P855898     532 CGGTCTCAAT GTCAGTGTCG CTGAGACAGG CGATGTTGGG GCGAAGGGCA
rNDUFS4     518 CGGTCTCAAT GTCAGTGTCG CTGAGACAGG CGTTGTTGAG GCAAAGGGCA
                ********** ********** ********** ********** **********
                ********** ********** ********** ********** **********
                ********** ********** ********** ********** **********
                ********** ********** ********** ********** **********
                ********** ********** ********** ********** * ********
                ********** ********** **********              ********
                ********** ********** ***** ****
                **** ***** ********** ***** ****
                **** ***** ********** *****
                ****

alignment
position        601....... 611....... 621....... 631....... 641.......
P855897     566 GTGGCTGTAG CTGCCCTTTC CGTTTCCAGG GTTCCGACCA GGTAATAGAA
P855898     582 ATGGCTACAG CTGCCGTTTC CGTCTGTAGA GTTCCATCCA GGTAACATAA
rNDUFS4     568 GTAGCTACAG CTGCGGTTTC CGTCTGCAGA GTTCCATCCA GGTAACATAA
                ********** ********** ********** ********** **********
                ********** ********** ********** ********** **********
                ********** ********** ********** ********** **********
                ********** ********** ********** ********** **********
                ********** ****  **** ********** ********** **********
                ********** ****  **** ********** ********** **********
                                      * **

alignment
position        651....... 661....... 671....... 681....... 691.......
P855897     616 TTTTCACACT TTTC------ ----TTCAAG CTTCTTGGGT CCCTCCATTT
P855898     632 CTGTCAGCCT TGTCGAGCTA TTCATTCAGG GTTCCCAGGT CGCTCCACTT
rNDUFS4     618 TTGTCAGCCT aGTCGAGCTA TTCATTCAGG ATTCCCAGGT CGCTCCACTT
                ********** ********** ********** ********** **********
                **********  ********* ********** ********** **********
                **********  ********* ********** ********** **********
                **********  ********* ********** ********** **********
                **********  ********* ********** ********** **********
                ******                    ****** ********** **********

alignment
position        701....... 711....... 721....... 731....... 741.......
P855897     656 TTGCGGAGTC TctGCTGGCT GAGTTCCGGA GGAGGCCTcg GGGAAGGCGT
P855898     682 TGGGGGAGGC TGGGCCGGCT GGGCCCCGGA GGCGGCCTT- GGGAACGCGT
rNDUFS4     668 TGGCGGGGGC CGGGCCTGCT GGGCCCTGGA GGCGGTCTT- GGGAACGCGT
                ********** ********** ********** *********  **********
                ********** ********** ********** *********  **********
                ********** ********** ********** *********  **********
                *********                                    *********
                *                                             ********
                *                                             ********

alignment
position        751....... 761....... 771....... 781....... 791.......
P855897     706 CCTCTGTTGA CCCTT----- ---------- ---------- ----------
P855898     731 CCTCCCTTGA CCCTTCCTCG GGATGCGGGG ACcGACGCTG GTTTCCGGCG
rNDUFS4     717 CCTCCTCTGA CCCTTCCTCG GGATGCGGGG ACgGACTCTG GTCTCCCGCA
                ********** ********** ********** **  ****** **********
                ********** ********** ********** **  ****** **********
                ********** ********** ********** **
                ********** ********** ********** **
                ********** *****
                ********** *****

alignment
position        801....... 811....... 821....... 831....... 841.......
P855897     721 ---------- ---------- -TTCTCGGGA Gaagtcgggc ggactaggga
P855898     781 AGGCGCCTGC GGGGCCTGGG GTT------- ---------- ----------
rNDUFS4     767 AGGCGCCTAT GGAGCCTCGG GTTCTCGGGA Gccc------ ----------
```

```
                   ********** ********** ********** *
                   ********** ********** ***

alignment
position           851....... 861....... 871....... 881....... 891.......
P855897      750   ctgtctgcta tcaatgggcg ttggccaggc gagtgagggc gtgagtgcga
P855898      804   ---------- ---------- ---------- ---------- ----------
rNDUFS4      801   ---------- ---------- ---------- ---------- ----------

alignment
position           901....... 911....... 921....... 931....... 941.......
P855897      800   ggccggggag gaggacctgt ggttttctgg aagctttcgt atTCGGCTTG
P855898      804   ---------- ---------- ---------- ---------- -------CTG
rNDUFS4      801   ---------- ---------- ---------- ---------- --TGGGTCTG
                                                                 ********

alignment
position           951....... 961....... 971....... 981....... 991.......
P855897      850   TCGCTGAGGG A----GAGGG TCCGTGgagg ttttgcctcc GGAGCTGCAA
P855898      807   CAGCTGCGGa cgggcGAGGG TCTGTGCGGA ---------- GGAGCTGTGA
rNDUFS4      809   CAGCTGCGGG T----GAGGG TCTATGCGGA ---------- GGAGCTGTGA
                   ********** *    ***** **********            **********
                                   ***** **********            **********
                                   ***** **********            **********
                                   ***** *****

alignment
position           1001...... 1011...... 1021...... 1031...... 1041......
P855897      896   GCTGAACCTT AGCCCtcaGA GAAGGGGAGG AGTCTcactg atttctgttt
P855898      847   ACTGGATCGT GGCTCCT-GA GAAGGGGAGG AGTCTGGGCG ACCTCTCTCC
rNDUFS4      845   ACCGGACTGT GGCTCCT-GA GAAGGGGAGG AGTCTGGGCG ACTTCTCTCC
                   ********** *******  ** ********** ********** **********
                   ********** *******  ** ********** ********** **********
                   ********** *******  ** ********** ********** **********
                                   **  ** ********** ********** **********
                                   **  ** ********** ********** **********
                                       ** ********** *****
                                       ** ********** *****

alignment
position           1051...... 1061...... 1071...... 1081...... 1091......
P855897      946   ccactttC-T GCATCCCTTC CATTTGTCAC CTGTTTGCCC accactccag
P855898      896   CAATGTcC-T ACATCCCTTC CGCTTGTCAC CTGCTTGCCC CATTCCAGCC
rNDUFS4      894   CAATGTcctT GCATCCCTTC CGTTTGTCAC CTGTTTGCCC CATTCCAGCC
                   ******  * * ********** ********** ********** **********
                   ******  * * ********** ********** ********** **********
                   ******    * ********** ********** ********** **********
                   ******    * ********** ********** ********** **********
                   ******    * ********** ********** **********
                                ********* ********** **********
                                ********* ********** **********
                                ********* ********** **********
                                ********* ********** **********

alignment
position           1101...... 1111...... 1121...... 1131...... 1141......
P855897      995   tccttac--- ---------- ---------- ---------- ----------
P855898      945   TGAAAGGGTA AATCCCTGCT ATCCTTTCCC TGGCTTTAGT TaataTCGGC
rNDUFS4      944   TGAAAGGGTA AATCCCTGCT TTCCTTTCCC TGGCATTAGT TactgTCGAC
                   ********** ********** ********** ********** *    *****
                   ********** ********** ********** ********** *    *****
                   ********** ********** ********** ********** *    *****
                   ********** ********** ********** ********** *

alignment
position           1151...... 1161...... 1171...... 1181...... 1191
P855897     1002   ---------- ---------- ---------- ---------- ----
P855898      995   TGGGCTTCCA AACAGAAAGG TTGTTGTATT CATcattgtg cata
```

```
rNDUFS4      994 TGAGCTTCTG AACAGAAAGG ATGTTGTATT CAT------- ----
                 ********** ********** ********** ***
                 ********** ********** ********** ***
                 ********** ********** ********** ***
```

Pairwise similarities:

For each pairwise alignment, the similarity (relative to the maximum similarity) and the number of identical nucleic acids (in % of shorter sequence) is given. Maximum values are underlined.

	P855898 (1038 bp)	rNDUFS4 (1026 bp)
**P855897 (1001 bp)**	0.320 54 %	0.335 54 %
**P855898 (1038 bp)**		1.000 87 %

Please note that the **similarity value 1.000** marks only the two **most similar** sequences, it does not necessarily mean that these sequences are identical.

Extract alignment region	
**Positions in alignment**	from: to:
**Output file**	dialign.seq

Extract aligned sequences

For comments, questions or bug reports, please contact support@genomatix.de

© Genomatix Software GmbH 1998-2005 - All rights reserved. License Agreement GEMS Launcher 4.1.1

Genomatix-Logo Logout Personal Messages GEMS-Logo
Logout Personal Messages

Genomatix MainGEMSElDoradoGene2PromoterBiblioSphere
Frequently asked questionsYour ResultsYour SequencesYour ProtocolHelp

GEMS Launcher Task: *DiAlign TF*: Multiple alignment plus TF sites
working on C1-NDUFS5 (3 seq.)

[Alignment] [Pairwise similarities]

DiAlign professional TF Release 3.1 December 2004 **Tue Jun 7 22:52:13 2005**

Solution parameters:

**Sequence file:**	C1-NDUFS5 (3 seq.)
**Type of sequences:**	nucleotide sequences
**Threshold T:**	0.00
**'*' signs below alignment denote:**	diagonal similarity (max. similarity is represented by 10 '*' signs)
**Alignment output:**	complete alignment is shown<br># nucleic acids per line: 50
**TF output:**	common TF matches located in aligned regions (common to 3 (100.0 %) sequences)
**Family matches:**	yes
**MatInspector library:**	Matrix Family Library Version 5.0 (February 2005)
**Selected groups** (core/matrix sim):	• ALL vertebrates.lib (0.75/Optimized)

Aligned Sequences:

No.	Sequence Name	Sequence Description	Length
1	P914276	P914276 sym=NDUFS5|loc=Loc4725|taxid=9606|spec=Homo sapiens|chr=1|ctg=NT_004511|str=(+)|start=9463424|end=9464424|len=1001|tss=501|comm=NADH dehydrogenase (ubiquinone) Fe-S protein 5, 15kDa (NADH-coenzyme Q reductase); (NM_004552/501/bronze;)	1001 bp
2	mNDUFS5	mNDUFS5 m6_dna range=chr4:122744619-122745729 5'pad=0 3'pad=0 revComp=TRUE strand=? repeatMasking=none	1111 bp
3	P914277	P914277 sym=nalloc=Loc362588|taxid=10116|spec=Rattus norvegicus|chr=5|ctg=NW_047719|str=(-)|start=4185309|end=4186309|len=1001|tss=501|comm=similar to NADH dehydrogenase (ubiquinone) Fe-S protein 5; (XM_342907/501/bronze;)	1001 bp

Alignment (DiAlign format):

Please note that only **upper-case** letters are considered to be **aligned**. For more information, please have a look at the user guide.

V$FKHD V$HOXF V$EGRF V$ETSF

```
alignment position 1 . . . . . . . . .   1 1 . . . . . . . .   2 1 . . . . . . . .   3 1 . . . . . . . .   4 1 . . . . . . . .
P914276          1 t g g c t a g g c c   t t c c c t g t g t   c t c t g t a a t t   g a t g g g t t t g   g g t t t t t t t t
mNDUFS5          1 T T A A A C C G A -   - - - - - - - - - -   - - - - - - - - - -   - - - - - - - - - -   - - - - - - - - - -
P914277          1 T T A A A C C G G -   - - - - - - - - - -   - - - - - - - - - -   - - - - - - - - - -   - - - - - - - - - -
                   * * * * * * * * *
                   * * * * * * * * *
                   * * * * * * * * *

alignment position 5 1 . . . . . . . .   6 1 . . . . . . . .   7 1 . . . . . . . .   8 1 . . . . . . . .   9 1 . . . . . . . .
```

```
P914276     51 cctttctctc tctctacctc ctctctctct tcttcatcct ctctctctca
mNDUFS5     10 ---------- ---------- ---------- ---------- ----------
P914277     10 ---------- ---------- ---------- ---------- ----------

alignment position 101....... 111....... 121....... 131....... 141.......
P914276    101 ctctcccct gagataaggt cttgctctgt tgccagggct ggaatgcaat
mNDUFS5     10 ---------- ---------- ---------- ---------- ----------
P914277     10 ---------- ---------- ---------- ---------- ----------

alignment position 151....... 161....... 171....... 181....... 191.......
P914276    151 ggaatggtgt gatcatggct cactacagcc tcaatctcct gggctcaaGG
mNDUFS5     10 ---------- ---------- ---------- ---------- --------GG
P914277     10 ---------- ---------- ---------- ---------- --------GG
                                                                   **
                                                                   **
                                                                   **

alignment position 201....... 211....... 221....... 231....... 241.......
P914276    201 GATCCTCCCA CCTCAACctc ctgagtagct ggaaccacag gcatgcacca
mNDUFS5     12 ACCTCTCCCA GATCAACAaA TGCTCTTAAC CTCTGAGCTG TCTCTCCA--
P914277     12 GCCTCTCCCA GAGCAACAcA TGCTCTTAAC CTCTGAGCTG TCTCTTCA--
               ********** ********  * ********* ********** ******* *
               ********** ********  * ********* ********** ******* *
               ********** ********  * ********* ********** ******* *
                                    * ********* ********** ******* *

alignment position 251....... 261....... 271....... 281....... 291.......
P914276    251 cctcGCCCGG CTAATTTTTT ATTTtttgta gaga------ -----GGGGA
mNDUFS5     60 ----GCCCGG CTATTATGTT TATTACCAAA AAGGGAAACT GCCACcgtga
P914277     60 ----GCCa-G CCATTATGTT TATTATCAGA AAAGAAAACA GTCACGGAGA
                   ****** ********** ********** ********** **********
                   ***   * ********** ********** ********** *****
                   ***
                   ***

alignment position 301....... 311....... 321....... 331....... 341.......
P914276    290 GTTTCGCCA- -TGTTGCCCA GGCTGTtttc gaactcctgg gctgaa----
mNDUFS5    106 aactgggaaa cAGTCACACA AGTTTTGGGG TCACGGCACA CGAGCTTTAA
P914277    105 GATTGGGAG- -AGTCACCCA AGTTTTGGGG TCACGGCCCA CGAGCTTTAA
               *********   ********* ********** ********** **********
                           ********* ********** ********** **********
                           ********* ********** ********** **********
                           ********* ********** ********** **********
                           ********* ********** ********** **********

alignment position 351....... 361....... 371....... 381....... 391.......
P914276    334 ---------- ---------- ---------- ---------- ------GTGA
mNDUFS5    156 tcaccacatt tcCTTGGGGT GAGGGAAGCA CTACTCTTTC TCGAGTGTGA
P914277    153 ccactacgtt ttCTTAGGGC GAGGGAAGCA ATACTCTTTC TCAAGTGTGA
                            ******** ********** ********** **********
                            ******** ********** ********** **********
                            ******** ********** ********** **********
                            ******** ********** ********** **********

alignment position 401....... 411....... 421....... 431....... 441.......
P914276    338 TCTTCCTCcc ---------- ---------- ---------- ----------
mNDUFS5    206 CCTTGCTCAA GTAGCACAAC AATCAACGGT CACGACAGTG CTgaaaggga
P914277    203 CCTTGCTCAA GTAGCACAAC AATCAACTGT CATGACAGTG CTg-------
               ********** ********** ********** ********** **
               ********** ********** ********** ********** **
               ********** ********** ********** ********** **
               ********** ********** ********** ********** **
                  ******* ********** ********** ********** **

alignment position 451....... 461....... 471....... 481....... 491.......
P914276    348 ---------- ---------- ---------- -----TCAGC CTTCCA----
```

```
mNDUFS5     256 gaactcacta cCGGGCGCAG TAGTATG-GG GAAAGTCGGT TTATCagctg
P914277     246 ---------- -CTGGCACAG TAGCACGaGG AAAAGTCAGT TTATCC----
                 ********* ******* ** ********** ******

alignment position 501....... 511....... 521....... 531....... 541.......
P914276     359 ---------- -------AGG TGCTGGGATT GCAGACGTCA G-CCACcgta
mNDUFS5     305 tcagcgtctg gaggctgGGC TGCTTGCTGC GCAGGCGCAg acCCGCTGT-
P914277     281 ---------- -------GGC CGCTGGCTGC GCAGGCGCAA A-CCGCTCT-
                                  *** ********* ********* *  ****** **
                                  *** ********* ********

alignment position 551....... 561....... 571....... 581....... 591.......
P914276     391 ccaggccaac ggGCTCGGTT TCTATGGTTt tgtggggaga cggctgaggg
mNDUFS5     354 ---------- --GCTTGGTT TCTACAGTTc ctc------- --GGCCGGT-
P914277     312 ---------- --GCTTGGTT TCCCTGGTTg ccgcagggac aaGGCCGGC-
                            ******** ********               ******
                            ******** **                     ******
                                                            ******

alignment position 601....... 611....... 621....... 631....... 641.......
P914276     441 aaGGTCGGCT TCCGGCGTCG CGCTGC--AA ACGTCTCGCT GAGGTCATCC
mNDUFS5     382 --GGTTGATT TACGGCGTCG CTCTGG--AG ATGTCGtGCT GCGGGCGTCC
P914277     349 --GGTCGATT TACGGCGTCG CGCTctgtAG ATGTCGCACC GCGGGCGTCC
                  ******** ********** ******   ** ********* **********
                  ******** ********** ****          ******* **********
                  ******** ********** *             ******* **********
                  ******** ********** *             ****  *** **********
                                                         *** **********
                                                             **********
                                                             **********
                                                             **********
                                                             **********
                                                             **********
                                                             **********

alignment position 651....... 661....... 671....... 681....... 691.......
P914276     489 TTTACGGCAG GCGTCCGCGT Cgctagcta- --GTCGTTCT GAAGCGGCGG
mNDUFS5     428 TTTACGGCAG GCGTCTGCGT CCTCCcgcAG CCGGCGGTCG GGAATTGCa-
P914277     397 TTTACGGCAG GCGTCTGCGT CCTCCttaAG CCGGCGGTCG TGAGCTGCGC
                ********** ********** *****   ** ********** **********
                ********** ********** *****          ******* ******* *
                ********** ********** *****
                ********** ********** *****
                ********** ********** *****
                ********** ********** *****
                ********** ********** *
                ********** ********** *
                ********** ********** *
                ********** ********** *
                ********** ********** *

alignment position 701....... 711....... 721....... 731....... 741.......
P914276     536 CCAGAGAAGA GTCAAGGGCA CgaGCATCGG GTAGGTAGGG GCTGCTTATG
mNDUFS5     477 CCAGGGACCT GACAAGGGCA CT-GCAGAGG TGCGGTAGGC GC-TGCTGTG
P914277     447 CCAGGGACCT GAGAAGGGCA TT-GCTGAGG TGCGGTAGGC GCTTGCTGTG
                ********** ********** ** ******* ********** **********
                ********** ********** ** ******* ********** *  *******
                ********** ********** ** ******* ********** *  *******
                ********** ********** ** ******* ********** *
                ********** ********** ** ******* ********** *

alignment position 751....... 761....... 771....... 781....... 791.......
P914276     586 TCCGCGCGGC agcttagctt aaggaccttc cttccgaggt CTCTTTAACA
mNDUFS5     525 TCCGCACAGC CAAGAAGTTT CGTTCAGGAT TaG------- CTCCTTGACA
P914277     496 TCCGGTCAGC CAAGAAGGTT CGCTAGGGAT T-G------- CTCCTAGACA
                ********** ********** ********** *  *       **********
                ********** ********** ********** *  *       **********
                ********** ********** ********** *          **********

alignment position 801....... 811....... 821....... 831....... 841.......
```

```
P914276             636 GATTTCCTCT T---Ggcttg ctgtgtgtcc ccttctccca gacctgaacT
mNDUFS5             568 CATTTCCTCT CtctGTGGGT GGCCCAGG-- ---------- ---------T
P914277             538 AATTTCCTCT C---GTGGGT GACCCAGG-- ---------- ---------T
                        ********** *   ****** ********                       *
                        ********** *   ****** ********                       *
                        **********     ****** ********                       *
                                       ****** ********                       *
                                       ****** ********                       *
                                       *

alignment position      851....... 861....... 871....... 881....... 891.......
P914276             683 CAGGGCGTGT TCCCTGCCCT CCCaggccgc t--------- ----TCACCT
mNDUFS5             597 CTGGGCGTGT CCTCCTGCCT TCCCTTCACC CCGTAACTAG GTCgTCACCC
P914277             564 CCGGGCGTGT CCTCCTGCCT TCCCTTCATC CCACAACTCG GTCc------
                        ********** ********** ********** ********** *** ******
                        ********** ********** *****
                        ********** ********** *****
                        ********** ********** *****
                        ********** ********** *****

alignment position      901....... 911....... 921....... 931....... 941.......
P914276             720 CCACagctaa tggcaccgaa gcctggagat tgccgctctc tccactttcc
mNDUFS5             647 CCACCCCCGG CCCTCCGGAG CAAATCCACC G--------- ----------
P914277             608 ---CCTCCAG CCCTCCAGAG TGATTCCGCC C--------- ----------
                        ********** ********** ********** *
                           ******* ********** ********** *

alignment position      951....... 961....... 971....... 981....... 991.......
P914276             770 acatttgttt aggttTTTGC CGGACTAATG TTTTGGCTTT CTgtttctta
mNDUFS5             678 ---------- -----TCTGC CGGACTCCTG CCTTGGCCTC CTTCCTACCC
P914277             636 ---------- -----TCTGC CGGACTCCTG CCTTGGCCTC CTTCCTACCC
                                        ***** ********** ********** **********
                                        ***** ********** ********** **********
                                        ***** ********** ********** **********
                                                   ***** ********** **********
                                                   ***** ********** **********
                                                   ***** ********** **********
                                                   ***** ********** **********
                                                   ***** ********** **

alignment position      1001...... 1011...... 1021...... 1031...... 1041......
P914276             820 acctaaagtc tacgatcctt atcccagctt tccaggctct gcgtgatttc
mNDUFS5             713 TGACATCCCG CTCCTttttg TTTTTGTTTT GAGACAGGGT TTCTCTGTGT
P914277             671 TGACATCCCG CTCCT----- TTTTTGTTTT GAGACATGGT TTTTCTGTGT
                        ********** *****      ********** ********** **********
                        ********** *****      ********** ********** **********
                        ********** *****      ********** ********** **********
                        ********** *****      ********** ********** **********
                        ********** *****
                        ********** *****
                        ********** *****

alignment position      1051...... 1061...... 1071...... 1081...... 1091......
P914276             870 ggccc----- ---------- ---------- ---------- ----------
mNDUFS5             763 AG-----GCT GGAATTCACT CTGTAGACCA AGCTGGCCTT GAACTCAcag
P914277             716 AGccctgGCT GAAATTCATT GTATAGACCA AGCTGGCCTT GAACTCAacg
                        **     *** ********** ********** ********** *******
                        **     *** ********** ********** ********** *******
                        **     *** ********** ********** ********** *******
                        **     *** ********** ********** ********** *******

alignment position      1101...... 1111...... 1121...... 1131...... 1141......
P914276             875 ---------- ---------- ---------- ---------- ----------
mNDUFS5             808 -AGAAGCATC TGCCTCTGTC TTCCAAGTGT TGAGATTAAA GGCGTGCACC
P914277             766 tAGAAGCACC TGCCTCTGTC TCCCAAGGGT TGAGATTAAA GGCGTGCGCT
                         ********* ********** ********** ********** **********
                         ********* ********** ********** ********** **********
                         ********* ********** ********** ********** *
                         ********* ********** ********** ********** *
```

```
                        ******* ***  ******** **  ********* *********  *

alignment position 1151.......  1161......  1171......  1181......  1191.......
P914276       875 ----------  ------CGCC  GGCCTCCTGA  GCTCATTTCA  TTCTTtt cac
mNDUFS5       857 ACATCCTGAT  TTTGGTCCCC  Agagccctct  a-------CT  TTCTTCCTG-
P914277       816 CCATACTGAT  TTTGATCCCC  AATCTCCTGA  GACCTCTGCT  TTCTTCCTG-
                  ******* ***  ******** **  ********* *********  ******* **
                  ******* ***  ******** **  *                  **  ******* **
                                                               **  ******* **
                                                               **  ******* **
                                                               **  ******* **

alignment position 1201.......  1211......  1221......  1231......  1241.......
P914276       909 cgtcatgcac  gctgcgctcc  aTTGTCCCCC  AAACATAGCC  AGCACTTCCG
mNDUFS5       899 ----------  ----------  -TTGTGCACT  TGGCTCAGCA  GCCGCTCCAG
P914277       865 ----------  ----------  -TTGTGCACC  TGACTGAGCA  GCCGCTCCAG
                                             ********  *********  *******  ***
                                             ********  *********  *******  ***
                                             ********  *********  *******  ***
                                             ********  *********  *******  ***
                                             ********  *********  *******  ***

alignment position 1251.......  1261......  1271......  1281......  1291.......
P914276       959 GCTGT-----  ----------  -----AACTC  Cgggctgttg  cccaactgga
mNDUFS5       928 agcctctgtc  cgtcctcatt  gtcatAACTT  CTGTTGTCta  tgtttGTGTG
P914277       894 ATTGG-----  ----------  -----AACTT  CTGTTGTCat  tgcccGTGTG
                  *****                          ***** ********           ** ***
                                                                          ** ***

alignment position 1301.......  1311......  1321......  1331......  1341.......
P914276       989 acactctgcc  cac-------  ----------  ----------  ----------
mNDUFS5       978 TGTGTGTGTG  TGTGTGTGTG  TGTGTGTGTG  TGtgtgtgtg  agagagagag
P914277       924 CGCGTGTGTG  CGTTTGCGTG  CGTGTGTGCG  TGctcgcgcg  cgcaattgtg
                  ******* ***  ******** **  ********* **
                  ******* ***  ******** **  ********* **

alignment position 1351.......  1361......  1371......  1381......  1391.......
P914276      1002 ----------  ----------  ----------  ----------  ----------
mNDUFS5      1028 agagagagag  agagagagag  agagagagag  agagcgagag  agagagcgat
P914277       974 tgggt-----  ----------  ----------  ----------  ----------

alignment position 1401.......  1411......  1421......  1431...
P914276      1002 ----------  ----------  ----------  -------
mNDUFS5      1078 gtagatcctc  cTCTGGGACT  TTTtctATTC  TCCC---
P914277       979 ----------  -TCTGGGACT  TTT---ATTC  TCCCgag
                               ******** **  ***     **** ****
```

Pairwise similarities:

For each pairwise alignment, the similarity (relative to the maximum similarity) and the number of identical nucleic acids (in % of shorter sequence) is given. Maximum values are underlined.

	mNDUFS5 (1111 bp)	P914277 (1001 bp)
P914276 (1001 bp)	0.068<br>21 %	0.069<br>26 %
mNDUFS5 (1111 bp)		1.000<br>76 %

Please note that the **similarity value 1.000** marks only the two **most similar** sequences, it does not necessarily mean that these sequences are identical.

Extract alignment region	
**Positions in alignment**	from: to:
**Output file**	dialign.seq

Extract aligned sequences

For comments, questions or bug reports, please contact support@genomatix.de

© Genomatix Software GmbH 1998-2005 - All rights reserved.

License Agreement

GEMS Launcher 4.1.1

Genomatix-Logo Logout Personal Messages GEMS-Logo
Logout Personal Messages

Genomatix Main GEMS ElDorado Gene2Promoter BiblioSphere
Frequently asked questions Your Results Your Sequences Your Protocol Help

GEMS Launcher Task: *DiAlign TF*: Multiple alignment plus TF sites
working on C1-NDUFS6 (3 seq.)

[Alignment] [Pairwise similarities]

DiAlign professional TF Release 3.1 December 2004 **Tue Jun 7 23:36:06 2005**

Solution parameters:

**Sequence file:**	C1-NDUFS6 (3 seq.)
**Type of sequences:**	nucleotide sequences
**Threshold T:**	0.00
**'*' signs below alignment denote:**	diagonal similarity (max. similarity is represented by 10 '*' signs)
**Alignment output:**	complete alignment is shown<br># nucleic acids per line: 50
**TF output:**	common TF matches located in aligned regions (common to 3 (100.0 %) sequences)
**Family matches:**	yes
**MatInspector library:**	Matrix Family Library Version 5.0 (February 2005)
**Selected groups** (core/matrix sim):	• ALL user_defined.lib (0.75/Optimized)<br>• ALL vertebrates.lib (0.75/Optimized)

Aligned Sequences:

No.	Sequence Name	Sequence Description	Length
1	P878337	P878337 sym=NDUFS6|loc=Loc4726|taxid=9606|spec=Homo sapiens|chr=5|ctg=NT_006576|str=(+)|start=1791009|end=1792014|len=1006|tss=501,506|comm=NADH dehydrogenase (ubiquinone) Fe-S protein 6, 13kDa (NADH-coenzyme Q reductase); (AK022503/506/gold;NM_004553/501/silver;)	1006 bp
2	mNDUFS6	mNDUFS6 sym=mNDUFS6|chr=13|start=69383481|end=69384469|tss=519	989 bp
3	rNDUFS6	rNDUFS6 sym=rNDUFS6|chr=17|start=44230|end=45095|tss=420	866 bp

Alignment (DiAlign format):

Please note that only **upper-case** letters are considered to be **aligned**. For more information, please have a look at the user guide.

V$NRF1 U$NRF1 V$YY1F V$RORA V$CMYB V$VMYB

```
alignment position 1 . . . . . . . . .   1 1 . . . . . . . .   2 1 . . . . . . . .   3 1 . . . . . . . .   4 1 . . . . . . . .
P878337          1 - - - - - - - - - -   - - - - - - - - - -   - - - - - - - - - -   - - - - - - - - - -   - - - - - - - - - -
mNDUFS6          1 t t c c g g a c a g   c t t t c c a A T G   C T G G G T A A A C   T T C C T T G C A G   T C T A A C T G G A
rNDUFS6          1 t t t t c c - - - -   - - - - - - - A T A   C T G G G A A A A C   T C C C T T G C A G   T C T A A C T G G A
                                                       * * *   * * * * * * * * * *   * * * * * * * * * *   * * * * * * * * * *
                                                       * * *   * * * * * * * * * *   * * * * * * * * * *   * * * * * * * * * *
                                                       * * *   * * * * * * * * * *   * * * * * * * * * *   * * * * * * * * * *

alignment position 5 1 . . . . . . . .   6 1 . . . . . . . .   7 1 . . . . . . . .   8 1 . . . . . . . .   9 1 . . . . . . . .
P878337          1 - - - - - - - - - -   - - - - - - - - - -   - - - - - - - - - -   - - - - - - - - - -   - - - - - - - - - -
```

```
mNDUFS6            51 AACTgaACTA CTGCAGCGTT GCAAAAATGC TCAGAAATCT aaCTTTCCTA
rNDUFS6            39 AACTttACTA CCGCTGCATT GCAAAAATGC TCTTAAGTCT tcCTTTCCCA
                      ****  **** ********** ********** **********   ********
                      ****  **** ********** ********** **********   ********
                      ****

alignment position    101....... 111....... 121....... 131....... 141.......
P878337             1 ---------- ---------- ---------T GCACTTCCAG TCTCGTCTGC
mNDUFS6           101 CACCAACATA CAGGACCAGG AGAGTTGACT GCacaaactG TTTCTTCTGC
rNDUFS6            89 CCCCAACATA CATAGCTAGG AAAGTTGACT GCATGTACTG GTTCTTCTAT
                      ********** ********** ********** ********** **********
                      ********** ********** ********** **          *********
                                                                   *********

alignment position    151....... 161....... 171....... 181....... 191.......
P878337            22 TAAGCAGTTG ACTCCTTCAC CCAAtaattt tgatatttaa atgtctcatg
mNDUFS6           151 TGAACAACTT ACTTCTTTAC TCAAAACAAA acagaaaaca aaaaacaaac
rNDUFS6           139 TGAACAAGGT ATTTCTTTAC TGAAAACAAA taaaatct-- ----------
                      ********** ********** **********
                      ********** ********** **********
                      ********** ********** **********

alignment position    201....... 211....... 221....... 231....... 241.......
P878337            72 a--------- ---------- --TTATCGGT TACCATAAGA ATATAAACAA
mNDUFS6           201 caacccacGG AAAATCTGCA TTTTCTCCAT TGGCATAAAA GCACACACAG
rNDUFS6           177 --------GG GAAATCTGCA TTTTCTCCAT TGCTATAAAA GTGCACAC--
                              ** ********** ********** ********** **********
                              ** ********** ********** ********** ********
                              ** ********** ********** ********** ********
                                                     * ********** ********

alignment position    251....... 261....... 271....... 281....... 291.......
P878337           101 TGTATTTTCA GTTTACATCC t-----CCCC TGCCTgtccg ctaaataaga
mNDUFS6           251 CGCATCTTCA GTTTCCGTCC ccccacCCCC TGCCTTTGTC ACACTAGTAA
rNDUFS6           217 TGCATCTTCA GTTTTCATCC ---------- ---CTTTGTC ACGTTAGTAA
                      ********** **********       **** ********** **********
                      ********** **********               ******* **********
                                                          ******* **********

alignment position    301....... 311....... 321....... 331....... 341.......
P878337           146 accactgggt actcctaatc gctgaaggtt tcgcacacca ttgcctgcct
mNDUFS6           301 CCTTTGAACA CT-------- ---------- ---------- ----------
rNDUFS6           254 ACTTTGAACA CG-------- ---------- ---------- ----------
                      ********** **
                      ********** **
                      ********** **

alignment position    351....... 361....... 371....... 381....... 391.......
P878337           196 catcagggcc ctagctctag cctgcggctg caactgcatg aggctgcacc
mNDUFS6           313 ---------- ---------- ---------- ---------- ----------
rNDUFS6           266 ---------- ---------- ---------- ---------- ----------

alignment position    401....... 411....... 421....... 431....... 441.......
P878337           246 tgccggcgca acggggccgc ttaactttta ggccattgat gaaaagtcaa
mNDUFS6           313 ---------- ---------- ---------- ---------- ----------
rNDUFS6           266 ---------- ---------- ---------- ---------- ----------

alignment position    451....... 461....... 471....... 481....... 491.......
P878337           296 acaaGCGCAC AGTactcggt gtaatcaggg gcatttttag cgagatacct
mNDUFS6           313 ----GCGCAC AGTTAAGCC- ---------- ---------- ----------
rNDUFS6           266 ----GCGCAC AGTTAAGCT- ---------- ---------- ----------
                          ****** *********
                          ****** *****
                          ****** *****

alignment position    501....... 511....... 521....... 531....... 541.......
```

```
P878337     346 gggatgaaaa cgggtgacca cctgaatcag agaGCAGCTC CGgcg-----
mNDUFS6     328 ---------- ---------- ---------- ---GCAGCTC CGCATCCTAT
rNDUFS6     281 ---------- ---------- ---------- ---GGAGCTC AGTATCCCCG
                                                    ******* **********

alignment position 551....... 561....... 571....... 581....... 591.......
P878337     391 -------CAC CCTCTGCCCC Gac-----CC GCGCTCCCCG CCCCCAGGGC
mNDUFS6     345 CCAGTTCCAC CACCAGCCCC G--------C CAGCCGCTCA GGCATAGGAC
rNDUFS6     298 TCAGGTCTAG CACCTACCCg gccagccgCC CAGCCGCTCA GCCACAGTAC
                ********** ********** *       ** ********** **********
                                                * ********** **********
                                                * ********** **********
                                                * ********** **********

alignment position 601....... 611....... 621....... 631....... 641.......
P878337     429 GAAATAAATA CCgggtgttt ggCGCCGCCA GAAGCCGTGC GCATGCGTTA
mNDUFS6     387 GAAAAAAGTT CCACAAGtAC T-CCCCGCCG ACTGCTGTGC GCCTGCGCCG
rNDUFS6     348 GAAAAAAGTT CCACAAGcAC T-CCCGGCCG AATGCTGTGC GCCTGCGCCG
                ********** *******  ** *  ******** ********** **********
                ********** *******  ** *  ******** ********** **********
                ********** *******  ** *  ******** ********** **********
                ********** *******            ******** ********** **********

alignment position 651....... 661....... 671....... 681....... 691.......
P878337     479 GCGCTaagCG GGACGTTGCG CCGGGTCAAA GGCCAGCGgc GCAAAATGGC
mNDUFS6     436 GCACTt---G GTACGACGCG TGGGGTCAAG GGTCACCG-- GCAAGATGGC
rNDUFS6     397 GCGCT---CT GTACGACGCG TGGGGTTAAG GGTCGTCG-- GCAAGATGGC
                *****   ** ********** ********** ********   **********
                *****      ********** ********** ****  **   **********
                *****      ********** ********** ****  **   **********
                *****                                  **   **********
                                                       **   **********
                                                            **********
                                                            **********
                                                            **********
                                                            **********
                                                            **********

alignment position 701....... 711....... 721....... 731....... 741.......
P878337     529 GGCGGCGATG ACCTTCTGCC GGCTGCTGAa ccggtgtggc gaggcggcgc
mNDUFS6     481 GGCGGTGCTG ACCTTCCGCC GGCTGTTGAC TCTC------ ----------
rNDUFS6     442 GGCGGCGCTG ACCTTCCGCC GGCTGTTGgC TCTC------ ----------
                ********** ********** ********** ****
                ********** ********** ********** ****
                ********** ********** ********** ****
                ********** ********** ********
                ********** ********** ********
                ********** ********** ********
                ********** ********** ********
                ********** ********** ********
                ********** ********** ********
                ********** ********** ********

alignment position 751....... 761....... 771....... 781....... 791.......
P878337     579 ggagcctgCC CCTGGGCGCC AGGTGTTTCG GGGTGCGGGT CTCGCCGAcC
mNDUFS6     515 --------CC CAGGGCTGCA CGGGGTTTCG GGGTTCAAGT GTCGCCGAGC
rNDUFS6     476 --------CC TAGGGCTGCC CGGGGCTTCG GGGTGCGAGT GTCGCGGAGC
                        ** ********** ********** ********** **********
                        ** ********** ********** ********** **********
                        ** ********** ********** ********** ***   ****
                        ** ********** ********** ********** ***   ****
                                  *** ********** ********** ***   ****
                                                                  **  *
                                                                  **  *
                                                                      *
                                                                      *
                                                                      *

alignment position 801....... 811....... 821....... 831....... 841.......
```

P878337 629 GGGGAGAAGG TCACGCACAC TGGCCAGGTA ACGGCCGCtg gg--------
mNDUFS6 557 GGGGAAAAGA TCACGCATAC CGGCCAGGTA ACGGCCGCGC CCGCCATGCC
rNDUFS6 518 GGGGAGAAGA TCACGCATAC CGGCCAGGTA ACGGCCGtGC CCGCCATGCC

```
                **********  **********  **********  **********  **********
                **********  **********  **********  **********  **********
                **********  **********  **********  **********  **********
                **********  **********  **********  *******  **  **********
                **********  **********  **********  *******  **  **********
                **********  **********  **********  *******
                **********  **********  **********  ******
                **********  **********  **********  ******
                **********  **********  **********  ******
                **********  **********  **********  ******
```

alignment position 851....... 861....... 871....... 881....... 891.......

P878337 671 ---------- ---------- ---------- ---------- ----------
mNDUFS6 607 TGGAACCCAA GACTGACGGT AGCAGGGAgc gcaGCAAGGT GTGCTTCTGC
rNDUFS6 568 TGGAACCCGA GACTGACGGT AGCAGGGAgc gtgGCAAGGT GTGCTTCTGC

```
                **********  **********  ********       *******  **********
                **********  **********  ********       *******  **********
                **********  **********  ********       *******  **********
                **********  **********  ********       *******  **********
                **********  **********  ********
```

alignment position 901....... 911....... 921....... 931....... 941.......

P878337 671 ---------- --------TA CAGGATGCAC CTTC---CTC CAGCCGCA--
mNDUFS6 657 CAGGCTAGCG CGCGGCaCTA CTGGGCTCAC CTTG---TGC AACCCGCT--
rNDUFS6 618 CAGGCTAGCG CGCGGCcCTA CTGGACTCAC CTTGgggTGC AGCCCGCTgc

```
                **********  ******  ***  **********  ****   ***  ********
                **********  ******
                **********  ******
                **********  ******
```

alignment position 951....... 961....... 971....... 981....... 991.......

P878337 698 ---CCTCGGG cg----ACTG GTGGCagtgg gctcgccggg catccgcggc
mNDUFS6 702 ---GCTCCGG TTTCTAGCTG GTGGCTTTcg gtT------- ----------
rNDUFS6 668 ccaGCTCTGA TTTCTAACTG GTGGCTTTgc c-T------- ----------

```
                   *******  **********  ********       *
                   *******  **********  ********       *
                                                       *
```

alignment position 1001...... 1011...... 1021...... 1031...... 1041......

P878337 741 cctggttctg cgccggcagc gcaggtcgtg gtaaggttgt gctgtcgctc
mNDUFS6 732 ---------- ---------- ---------- ---------- ----------
rNDUFS6 700 ---------- ---------- ---------- ---------- ----------

alignment position 1051...... 1061...... 1071...... 1081...... 1091......

P878337 791 acgcgccggg gaggacgccc gcgagtcggg acggtggtgg cagcctgtag
mNDUFS6 732 ---------- ---------- ---------- ---------- ----------
rNDUFS6 700 ---------- ---------- ---------- ---------- ----------

alignment position 1101...... 1111...... 1121...... 1131...... 1141......

P878337 841 cggtgggtct cgtccttcca gggcctgcgg gctctggaac gggaagtctc
mNDUFS6 732 ---------- ---------- ---------- ---------- ----------
rNDUFS6 700 ---------- ---------- ---------- ---------- ----------

alignment position 1151...... 1161...... 1171...... 1181...... 1191......

P878337 891 cccagcgggc agcacttgct gtggactggc tgcccatagG CGATTTGTGG
mNDUFS6 732 ---------- ---------- ---------- ---------G GGACTTGGAT
rNDUFS6 700 ---------- ---------- ---------- ---------G GGACTTGGAT

```
                                                         *  **********
                                                         *  **********
                                                         *  **********
```

alignment position 1201...... 1211...... 1221...... 1231...... 1241......

```
P878337             941 CAGCCGATTA TTTGGGTCAG AACGTTtaga atttgtgtaa tattcaATAA
mNDUFS6             743 CTCCTCATTC TTTAGGCCAT ATCTTT---- ---------- ------ATAT
rNDUFS6             711 CTGCTCATTC TTTAGGTCAT AACTTT---- ---------- ------ATAA
                        ********** ********** ******                     ****
                        ********** ****** *** ******                     ****
                        ********** ****** *** ******

alignment position      1251...... 1261...... 1271...... 1281...... 1291......
P878337             991 TAAATTttag taacct---- ---------- ---------- ----------
mNDUFS6             773 TAAGTTCAAT GTTTTAACAA atgattgtTG TGTTTTGGAC CCGGGACaga
rNDUFS6             741 TAAGTTCCAA GTTTTAAGAA tggttg--TG TTTTTAGACC CCGGGACtgg
                        ********** **********         ** ********** *******
                        ********** **********

alignment position      1301...... 1311...... 1321...... 1331...... 1341......
P878337            1007 ---------- ---------- ---------- ---------- ----------
mNDUFS6             823 gGACTGAGGT GCATATTTTA CATATCAAAG GAGACCcGGC ATGCAGGTTC
rNDUFS6             789 aGACTGAGGT GCATATTTTA CATATCAAAG CAGACCtGGC ATCCAGGTTC
                         ********* ********** ********** ****** *** **********
                         ********* ********** ********** ****** *** **********
                         ********* ********** ********** ****** *** **********
                         ********* ********** ********** ****** *** **********

alignment position      1351...... 1361...... 1371...... 1381...... 1391......
P878337            1007 ---------- ---------- ---------- ---------- ----------
mNDUFS6             873 TCCCAGCATT CCTCAGTCCC TACCTGGcgt tgcagcccag gatcctccct
rNDUFS6             839 TCCCAGCATC CCTCAGTCCC TACCTGGc-- ---------- ----------
                        ********** ********** *******
                        ********** ********** *******
                        ********** ********** *******
                        ********** ********** *******

alignment position      1401...... 1411...... 1421...... 1431...... 1441......
P878337            1007 ---------- ---------- ---------- ---------- ----------
mNDUFS6             923 atataatcca ggcattttgg tttctcccac cctgtctctt tctcctctcc
rNDUFS6             867 ---------- ---------- ---------- ---------- ----------

alignment position      1451...... 1461...
P878337            1007 ---------- -------
mNDUFS6             973 ttgaacacac tcccttt
rNDUFS6             867 ---------- -------
```

Pairwise similarities:

For each pairwise alignment, the similarity (relative to the maximum similarity) and the number of identical nucleic acids (in % of shorter sequence) is given. Maximum values are underlined.

	mNDUFS6 (989 bp)	rNDUFS6 (866 bp)
**P878337 (1006 bp)**	0.150<br>31 %	0.155<br>30 %
**mNDUFS6 (989 bp)**		1.000<br>81 %

Please note that the **similarity value 1.000** marks only the two **most similar** sequences, it does not necessarily mean that these sequences are identical.

**Extract alignment region**	
**Positions in alignment**	from: to:

Output file	dialign.seq

Extract aligned sequences

For comments, questions or bug reports, please contact support@genomatix.de

© Genomatix Software GmbH 1998-2005
- All rights reserved.

License Agreement

GEMS Launcher 4.1.1

Genomatix-Logo | Logout | Personal | Messages | GEMS-Logo
Logout | Personal | Messages

Genomatix_MainGEMSElDoradoGene2PromoterBiblioSphere
Frequently_asked_questionsYour_ResultsYour_SequencesYour_ProtocolHelp

GEMS Launcher Task: *DiAlign TF*: Multiple alignment plus TF sites
working on promoters.seq (3 seq.)

[Alignment] [Pairwise_similarities]

DiAlign professional TF Release 3.1 December 2004 **Tue Jun 7 23:49:38 2005**

Solution parameters:

**Sequence file:**	promoters.seq (3 seq.)
**Type of sequences:**	nucleotide sequences
**Threshold T:**	0.00
**'*' signs below alignment denote**:	diagonal similarity (max. similarity is represented by 10 '*' signs)
**Alignment output:**	complete alignment is shown<br># nucleic acids per line: 50
**TF output:**	common TF matches located in aligned regions (common to 3 (100.0 %) sequences)
**Family matches:**	yes
**MatInspector library:**	Matrix Family Library Version 5.0 (February 2005)
**Selected groups** (core/matrix sim):	• ALL user_defined.lib (0.75/Optimized)<br>• ALL vertebrates.lib (0.75/Optimized)

Aligned Sequences:

No.	Sequence Name	Sequence Description	Length
1	P968931	sym=NDUFS7lloc=Loc374291ltaxid=9606lspec=Homo sapiensl chr=19lctg=NT_011255lstr=(+)lstart=1323383lend=1324406l len=1024ltss=501,507,521,524l comm=NADH dehydrogenase (ubiquinone) Fe-S protein 7, 20kDa (NADH-coenzyme Q reductase); (AK092169/501/gold;AK091623/521/gold;AK128713/507/gold; NM_024407/524/bronze;)	1024 bp
2	P968933	sym=Ndufs7lloc=Loc75406ltaxid=10090lspec=Mus musculusl chr=10lctg=NT_039496lstr=(+)lstart=6105465lend=6106465l len=1001ltss=501l comm=NADH dehydrogenase (ubiquinone) Fe-S protein 7; (NM_029272/517/bronze;)	1001 bp
3	P968934	sym=nalloc=Loc362837ltaxid=10116lspec=Rattus norvegicusl chr=7lctg=NW_047773lstr=(-)lstart=10970711lend=10971711l len=1001ltss=501l comm=similar to NADH dehydrogenase (ubiquinone) Fe-S protein 7; (XM_343165/511/bronze;)	1001 bp

Alignment (DiAlign format):

Please note that only **upper-case** letters are considered to be **aligned**. For more information, please have a look at the user guide.

V$GATA V$VMYB V$RORA V$NRF1 U$NRF1 V$TEAF V$DEAF V$LTUP V$FKHD V$IRFF V$ETSF V$BARB
V$PCAT V$HAML V$MINI V$EKLF V$GLIF V$YY1F

```
alignment position 1.........  11........  21........  31........  41........
P968931      1 ----------  ----------  ----------  ----------  --AGGGACCT
P968933      1 gtt aaaaaca ct--------  TGCCGAAACC  CGGGATCGAA  CCAGGGACCT
P968934      1 gtc gatggga taaaaacctc  TGCCGAAACC  CGGGATCGAA  CCAGGGACCT
```

```
                                                      **********  **********  **********
                                                      **********  **********  **********
                                                      **********  **********  **********
                                                      **********  **********  **********
                                                                                ********
                                                                                ********
                                                                                ********
                                                                                ********
                                                                                ********
                                                                                ********
alignment position  51........  61........  71........  81........  91........
P968931          9  TTAGATCTTC  AGTCTAACGC  TCTCCCAACT  GAGCTATTTC  GGCTGggcgg
P968933         43  TTAGATCTTC  AGTCTAACGC  TCTCCCAACT  GAGCTATTTC  GGCgacgcgc
P968934         51  TTAGATCTTC  AGTCTAACGC  TCTCCCAACT  GAGCTATTTC  GGCTGcgtga
                    **********  **********  **********  **********  *****
                    **********  **********  **********  **********  ***
                    **********  **********  **********  **********  ***
                    **********  **********  **********  **********  ***
                    **********  **********  **********  **
                    **********  **********  **********  **
                    **********  **********  **********  **
                    **********  **********  **********
                    **********  **********  **********
                    **********  **********  **********
alignment position  101.......  111.......  121.......  131.......  141.......
P968931         59  cagagtcgct  ctcgccg-GG  ACTCCGTCCC  GCGCTGCGCC  TTCCCTGCGC
P968933         93  tgtttgtcgg  taacaccctc  cCTCCGCCCC  GTGCCATTGC  TTTAGCGCGC
P968934        101  aggacgtcag  cactgctcGG  TCTCCGCCCC  GTGCCATTGC  TCCTGTGCGC
                                        **  **********  **********  **********
                                             *********  **********  **********
                                             *********  **********  **********
alignment position  151.......  161.......  171.......  181.......  191.......
P968931        108  AGGCGCAGTc  gtttcgcgtc  agcaggcccc  acatcctttg  taaggtctcc
P968933        143  AAGCGCACTT  Cacaatttgt  ctGTCAAACC  TTGTAGGGTT  ATACTATAAG
P968934        151  AAGCGCACTT  Cacacttcat  gaGTTAAACC  TCGTAGGTTT  ATACTATAGA
                    **********  *             ********  **********  **********
                    **********  *
                    **********  *
alignment position  201.......  211.......  221.......  231.......  241.......
P968931        158  cagggcgccg  gagcgcagTG  TCGTCTCTGT  GGCGCAATCG  GTTAGCGCGT
P968933        193  CAGCTgactA  A-------TG  TCGTCTCTGT  GGCGCAATCG  GTTAGCGCGT
P968934        201  AAGCTtcacA  A-------TG  TCGTCTCTGT  GGCGCAATCG  GTTAGCGCGT
                    *****    *  *       **  **********  **********  **********
                             *  *       **  **********  **********  **********
                             *  *       **  **********  **********  **********
                             *  *       **  **********  **********  **********
                                        **  **********  **********  **********
                                        **  **********  **********  **********
                                        **  **********  **********  **********
                                        **  **********  **********  **********
                                        **  **********  **********  **********
                                        **  **********  **********  **********
alignment position  251.......  261.......  271.......  281.......  291.......
P968931        208  TCGGCTGTTA  ACCGAAAGGT  TGGTGGTTCG  AGCCCACCCA  GGGACGcgtg
P968933        236  TCGGCTGTTA  ACCGAAAGGT  TGGTGGTTCG  AGCCCACCCA  GGGACGacca
P968934        244  TCGGCTGTTA  ACCGAAAGGT  TGGTGGTTCG  AGCCCACCCA  GGGACGacta
                    **********  **********  **********  **********  ******
                    **********  **********  **********  **********  ******
                    **********  **********  **********  **********  ******
                    **********  **********  **********  **********  ******
                    **********  **********  **********  **********  ******
                    **********  **********  **********  **********  ******
                    **********  **********  **********  **********  ******
                    **********  **********  **********  **********  ******
                    **********  **********  **********  **********  ******
```

```
                    ********** ********** ********** ********** ******
alignment position  301....... 311....... 321....... 331....... 341.......
P968931         258 --GTGA-CTT TTGCCCTCCT GGAATAACAA GAAAATGAGT GTcgcctctg
P968933         286 caGCGA-CTT TTTCATTCCC TAATACTGAA TAAAGTGGAA GTTCTTTA--
P968934         294 tgattcgCCT TTTTGTCCCC TAATt-TGAG TAAAATAGAA GTTTTTTC--
                      **** *** ********** ********** ********** ********

alignment position  351....... 361....... 371....... 381....... 391.......
P968931         305 tTTTTGTCGg tccgccgcag acccgcggcc cccacgt--G CGCACTCcga
P968933         333 -TTTTGTCGc TGTTTCCTGT TTGTCACAGG ACGACCGTGG CGCACTCTGC
P968934         341 -TTTTGTC-- TGTTTCCTGT TTGTCACAGG AGGACGGTTG TGTACACTGC
                     ********  ********** ********** ********** **********
                               ********** ********** ********** **********

alignment position  401....... 411....... 421....... 431....... 441.......
P968931         353 tgacctttcc gcctcgggtg atctgggggt gcacggcgca ACACGACCAC
P968933         382 gCGG------ ---------- ---------- ---------- ACGCGACCAC
P968934         388 tCGG------ ---------- ---------- ---------- GCGCGATCAC
                     ***                                        **********
                     ***                                        **********

alignment position  451....... 461....... 471....... 481....... 491.......
P968931         403 ggaggccgAA AGCGCAGCGG AACCTCCCGG CTCCAGGTTC CGGTTTCCgc
P968933         396 AGGTAT--AA AGCGGGTCAG AACCACTCGG CTTGTGGTGC GGGTTTCCGC
P968934         402 AGGAAA--AA AGCGGGTCGG AACCGCTCAG TTTGTGGTTC CGGTTTCCGC
                    ******  ** ********** ********** ********** **********
                    ******  ** ********** ********** ********** **********
                            ** ********** ********    ********* **********
                                                      ********* ********
                                                      ********* ********

alignment position  501....... 511....... 521....... 531....... 541.......
P968931         453 atgcgctccg agccgcTTTG GGCGGCGGCC CCGggaacCC GGAGCGCTAA
P968933         444 TGGTGTCTA- ------TTTG GGCGTTGGac tAGAA---CC GGAACTCTTT
P968934         450 TGGTGTCTA- ------TTTG GGCGTTGGAC CAGAA---CC GGAACTCTTT
                    *********        **** ********** *****   ** **********
                    *********        **** ********    ****   ** **********
                    *********        **** ********    ****   ** **********
                                                            ** **********
                                                            ** **********

alignment position  551....... 561....... 571....... 581....... 591.......
P968931         503 GGAGAACGGA CCTCAGAGGT TGTCTGAAGG CCGAGGCCAA GATGGCGGTG
P968933         484 GGAGGACGGA CCGCAGAGGT TGTCTGAAGG CCGAGGCCAA GATGGCGGCG
P968934         490 GGAAGACGGA CCGCAGAGGT TGTCTGAAGG CCGAGGCCAA GATGGCGGCG
                    ********** ********** ********** ********** **********
                    ********** ********** ********** ********** **********
                    ********** ********** ********** ********** **********
                    ********** ********** ********** ********** **********
                    ********** ********** ********** ********** **********
                                            ******** ********** **********
                                            ******** ********** **********
                                            ******** ********** **********

alignment position  601....... 611....... 621....... 631....... 641.......
P968931         553 CTGTCAGGTG AGcgcggcac cggcggcggg tgtggggccg cgcgggtctg
P968933         534 CTGGCGGGTG AGTGaagccc tTGCGGTC-- ---------- ----------
P968934         540 CTGGCGGGTG AGTGaagatc -TGAGATC-- ---------- ----------
                    ********** ****        *******
                    ********** ****        *******
                    ********** ****
                    ********** ****
                    ********** **
                    ********** **
                    ********** **
                    ********** **

alignment position  651....... 661....... 671....... 681....... 691.......
```

```
P968931     603 gggccgtggg agcctcgGGT GTCGTGGTGG CGTCGGGGGt cggtgccggc
P968933     562 ---------- -------GGG AGAGTGGCGT AGTCCTGGG- ----------
P968934     567 ---------- -------GGG GTAGTGGCGC AGTCCTGGG- ----------
                                  *** ********** *********
                                  *** ********** *********

alignment position 701....... 711....... 721....... 731....... 741.......
P968931     653 gtcgtggccg cggtcctctc cgggcttCTC CGAGCCGGCc gct------C
P968933     584 ---------- ---------- -------CTC CGAGCTGGCg ctgCGGGGAC
P968934     589 ---------- ---------- -------CCT CGAGCTGGgc tt-CGGGGAC
                                             *** *********     *******
                                             *** ********      *******
                                                               *******

alignment position 751....... 761....... 771....... 781....... 791.......
P968931     697 CTCGGGCTCC CCGCCCGGct tgcgatgaac ggtcgccgtt attgcgt---
P968933     607 CTTGGAGCCC AAGCCTAGTG TTGAGAGGCG TTGggaccgt --AGAGACCT
P968934     611 CTTGGAGCCC AAGCCCGGTA TTGACAGGCG TTGggatcgc agAGAGACCT
                ********** ********** ********** ***          ********
                ********** ********** ********** ***          ********
                ********** ********** ********** ***          ********

alignment position 801....... 811....... 821....... 831....... 841.......
P968931     744 ---------- ---------- ---------- ---------- ----------
P968933     655 ATAGGTCAGA TCCCAGCCCT CGGTAATGTC GGtgTCTAGC ATTAGGCCGA
P968934     661 ATAGGTCAGA ACCCAGCCCG CGATAGTGTC GGgaTCTAGG ATTGGGCCGA
                ********** ********** ********** **   ***** **********
                ********** ********** ********** **   ***** **********
                ********** ********** ********** **

alignment position 851....... 861....... 871....... 881....... 891.......
P968931     744 ---------- ---------- ---------- ---------- ----------
P968933     705 AGCATGTTTG AATTCACgtt ACACAGTTCT GCATGGTCTA CATAGCAAGT
P968934     711 AGTGTGTTTG AATCCACttc ACACAGTTAT GTCTGGTCTA CCTAGCAAGT
                ********** *******    ********** ********** **********
                ********** *******    ********** ********** **********

alignment position 901....... 911....... 921....... 931....... 941.......
P968931     744 ---------- ---------- -CCAGAGTAC AGTCGGGGAA ACCGAGGCCC
P968933     755 TGCAGGCCTC ctgctatcgt cCCTGATTAT AGTCGGGAAC GCTGAGGCCT
P968934     761 TCCAGGCCTC ccattactgc agCAGATTGT AGTTGTGGAC GCTGAGGCCT
                **********              ******** ********** **********
                **********                     * ********** **********

alignment position 951....... 961....... 971....... 981....... 991.......
P968931     773 Ggtaggtcat gacgcaagcc tccagcctca ggaaggcgCT CGGGGTGGAG
P968933     805 GTGTATCTTC CAAC------ ---------- --------CT CAGgaagCTG
P968934     811 GTATATCTCT CAAT------ ---------- --------CT CAGGAGACTG
                ********** ****                         ** **********
                ********** ****                         ** ***    ***

alignment position 1001...... 1011...... 1021...... 1031...... 1041......
P968931     823 GCTGGAGGGG CCCCATTCAC GTCCCCGAga ccagggcacg gcccgcgagg
P968933     831 ACTGCAGAAA CCCGGCTGAT GTCCCCGAAG CACAGAgtgg gaca------
P968934     837 ATTGCAGGAG CCCAGCTAAT GACCCTGAAG CTCAGAcag- ----------
                ********** ********** ********** ******
                ********** ********** ********** ******

alignment position 1051...... 1061...... 1071...... 1081...... 1091......
P968931     873 ctgctcttga gatgccctgg agagagcggg gcctgcctga caggctggga
P968933     875 ---------- ---------- ---------- ---------- ----------
P968934     876 ---------- ---------- ---------- ---------- ----------

alignment position 1101...... 1111...... 1121...... 1131...... 1141......
P968931     923 aactgaggcc cattgagggt cttcgaggtc acagggccgg gccTGGGAAG
P968933     875 ---------- ---------- ---------- ---------- ---------G
```

```
P968934        876 ---------- ---------- ---------- ---------- ---TGGGACG
                                                                  *******
                                                                        *

alignment position 1151...... 1161...... 1171...... 1181...... 1191......
P968931        973 GAACAGGACC CAGGACATCC ACGCCACCCT GCTGGACCCT Ccacctccct
P968933        876 GGGAGAGGCC CAGGACACTT AGGCCACCCA CCCGGTGGCT CTTTCCATCT
P968934        883 GGAAGAGGCC CAGGACACTC AGGGCACTCA CCCtGTGGCT CTTTCACCTT
                   ********** ********** ********** ********** **********
                   ********** ********** ********** ***  ****** **********
                         **** ********** *********

alignment position 1201...... 1211...... 1221...... 1231...... 1241......
P968931       1023 cc-------- ---------- ---------- ---------- ----------
P968933        926 CATCCCCAAC CTGGACAGCT AGcaagggcc tcTAGAGCAG ATGTCTTAGT
P968934        933 CATCACCGCC CTGGACAGCT AGggcaat-- --TAGAGCAG ATGCCTCAAT
                   ********** ********** **           ******** **********
                   ********** ********** **           ******** **********

alignment position 1251...... 1261...... 1271..
P968931       1025 ---------- ---------- -------
P968933        976 TTCCCTGCCA AAGCTTTGGT GTctttt
P968934        979 TTCCCTGCCG AAGTTATGGT GTc---
                   ********** ********** **
                   ********** ********** **
```

Pairwise similarities:

For each pairwise alignment, the similarity (relative to the maximum similarity) and the number of identical nucleic acids (in % of shorter sequence) is given. Maximum values are underlined.

	P968933 (1001 bp)	P968934 (1001 bp)
**P968931 (1024 bp)**	0.348<br>38 %	0.345<br>36 %
**P968933 (1001 bp)**		1.000<br>76 %

Please note that the **similarity value 1.000** marks only the two **most similar** sequences, it does not necessarily mean that these sequences are identical.

Extract alignment region	
**Positions in alignment**	from: to:
**Output file**	dialign.seq

Extract aligned sequences

For comments, questions or bug reports, please contact support@genomatix.de

© Genomatix Software GmbH 1998-2005 - All rights reserved.

License Agreement

GEMS Launcher 4.1.1

Genomatix-Logo Logout Personal Messages GEMS-Logo
Logout Personal Messages

Genomatix MainGEMSElDoradoGene2PromoterBiblioSphere
Frequently asked questionsYour ResultsYour SequencesYour ProtocolHelp

GEMS Launcher Task: *DiAlign TF*: Multiple alignment plus TF sites
working on promoters.seq (3 seq.)

[Alignment] [Pairwise similarities]

DiAlign professional TF Release 3.1 December 2004 **Wed Jun 8 00:24:29 2005**

Solution parameters:

**Sequence file:**	promoters.seq (3 seq.)
**Type of sequences:**	nucleotide sequences
**Threshold T:**	0.00
**'*' signs below alignment denote:**	diagonal similarity (max. similarity is represented by 10 '*' signs)
**Alignment output:**	complete alignment is shown<br># nucleic acids per line: 50
**TF output:**	common TF matches located in aligned regions (common to 3 (100.0 %) sequences)
**Family matches:**	yes
**MatInspector library:**	Matrix Family Library Version 5.0 (February 2005)
**Selected groups** (core/matrix sim):	• ALL user_defined.lib (0.75/Optimized)<br>• ALL vertebrates.lib (0.75/Optimized)

Aligned Sequences:

No.	Sequence Name	Sequence Description	Length
1	P855978	sym=NDUFS8|loc=Loc4728|taxid=9606|spec=Homo sapiens|chr=11|ctg=NT_033903|str=(+)|start=13103404|end=13104475|len=1072| tss=501,572| comm=NADH dehydrogenase (ubiquinone) Fe-S protein 8, 23kDa (NADH-coenzyme Q reductase); (AK002110/572/gold;NM_002496/501/silver;)	1072 bp
2	P855979	sym=Ndufs8|loc=Loc225887|taxid=10090|spec=Mus musculus|chr=19|ctg=NT_082868|str=(-)|start=700730|end=701730| len=1001|tss=501| comm=NADH dehydrogenase (ubiquinone) Fe-S protein 8; (NM_144870/536/bronze;)	1001 bp
3	P855980	sym=na|loc=Loc293652|taxid=10116|spec=Rattus norvegicus|chr=1|ctg=NW_047563|str=(-)|start=14967775|end=14968775| len=1001|tss=501| comm=similar to NADH dehydrogenase:ubiquinone Fe-S protein 8; (XM_215197/501/bronze;)	1001 bp

Alignment (DiAlign format):

Please note that only **upper-case** letters are considered to be **aligned**. For more information, please have a look at the user guide.

V$ETSF V$SP1F V$NRSF V$YY1F V$MYT1 V$ECAT V$PCAT V$SF1F V$NRF1 U$NRF1 V$HOXF V$HOMF V$NKXH

```
alignment position 1 . . . . . . . . .  1 1 . . . . . . . .  2 1 . . . . . . . .  3 1 . . . . . . . .  4 1 . . . . . . . .
P855978          1 a a a t t c g c t g  g t a g g a a a c a  t a g g c t g g t t  g a g t a c g g g a  g t - - - - - - - -
P855979          1 c a t g t c c t t c  c t g g c c t t c c  a g a t t c t a c a  c c g c c a g c t t  a g a c A C T A C T
P855980          1 - - - - - - - - - -  - - - - - - - - - -  - - - - - - - - - -  - - - - - - - - - -  - - - - A C T A T T
```

```
                                                                   ******

alignment position 51........ 61........ 71........ 81........ 91........
P855978        43  ---------- ---------- --------CA TACAGATCAG GTGGATcagg
P855979        51  TCCTGAAGGA AGCATTTC-T GATCACTCCA TACAAGGCAT GCAGAT----
P855980         7  CCCTAAGGGA AGCCTTCCcT GATCACTCCA TACAAGACAT GCGGAT----
                   ********** ******** * ********** ********** ******
                                       * ********** ********** ******
                                       * ********** ********** ******
                                       * ********** ********** ******
                                       * ********** ********** ******

alignment position 101....... 111....... 121....... 131....... 141.......
P855978        65  cggccgcaca ccgcagctct acggcccggg aacgttacta aatactccca
P855979        96  ---------- ---------- ---------- ---------- ----------
P855980        53  ---------- ---------- ---------- ---------- ----------

alignment position 151....... 161....... 171....... 181....... 191.......
P855978       115  TGCCTCAGCT TCCCGCCTTA ACGTGGTAAC ACGTTACtgt agtttctCCA
P855979        96  TGCCTCATGG TTCtCTAGTC CTAGGGGCAG ACTTGACAAA AgTCCTGCTA
P855980        53  TGCCTCATGG TTCCCTAGTC CCAGGGGCAG ACTTGACAAA AaTCCTGCTA
                   ********** ********** ********** ********** * ********
                   ********** ***  ***** ********** ********** * ********
                   ********** ***  ***** ********** ********** * ********
                   ********** ***                                ********
                   ********** ***                                ********

alignment position 201....... 211....... 221....... 231....... 241.......
P855978       165  CGCAGAGCTG CTGTGAGGAT TAAAAGAGGC CGCGTgagtt gagcatc---
P855979       146  CCCGCGGCCG TTACAAGGAC CAGtg----- ------GTTA AAGCGCTTGT
P855980       103  CCCGCGGCCG TTACAAGGAC CAGAAGAAGC CAAGTaGTTA AAGAGCTTAT
                   ********** ********** ********** ***** **** **********
                   ********** ********** ***              **** **********
                   ********** ********** ***              **** **********
                   ********** ********** ***              **** **********
                   ********** ********** ***

alignment position 251....... 261....... 271....... 281....... 291.......
P855978       212  ------TCGT CGCCATTACC GTCATCACAC GCCCCCCACC CAACCCggcg
P855979       185  ACGCTGTCGC CATCTTTGCA GTATTCAAAT GAGCCCTACC CCTCCCGCCT
P855980       153  GAGCTGTCGC CATCTTTGCA GTATTTAAAT GAGCCCCACC ATTCCCTCCC
                   ********** ********** ********** ********** **********
                   ********** ********** ***** **** ********** ******
                   ********** ********** *****
                   ********** ********** *****

alignment position 301....... 311....... 321....... 331....... 341.......
P855978       256  ggcccaccct ctgcacccac ggcgaaagca ctctttCTAA ACCACGCCCC
P855979       235  CCCcggactg tag--CAGCG TC-------- ------CCAA ACCGAGGTCA
P855980       203  CTCccggaaa tgtacCAGCG TC-------- ------CCAA ACCGAGCTCA
                   ***             ***** **               **** **********
                                   ***** **               **** **********
                                   ***** **               **** **********
                                   ***** **               **** **********
                                   ***** **               **** **********
                                                          **** **********
                                                            ** **********
                                                            ** **********

alignment position 351....... 361....... 371....... 381....... 391.......
P855978       306  GGATGTCCCG CCTCCAGCGG CTCGGAggtc ttcactTATT GGCCTCCCCA
P855979       269  GGAAGTCCCG CCTTCAGGAT CTCGGAAGTC AGGCG-CATT AACCTCTCTG
P855980       239  GGAAGTCCCG CCTTCAGCAT CTCGGAAGTC AGGCG-TATT AACCTCTCCG
                   ********** ********** ********** ***** **** **********
                   ********** ********** ********** ***** **** **********
                   ********** ********** ********** ***** **** **********
                   ********** *******  ** ********** ***** **** **********
                   ********** *******  ** ********** ***** **** **********
```

```
                   ********** *******   ** ******          **** **********
                   ********** *******   ** ******           *** **********
                   ********** *******

alignment position 401.......  411.......  421.......  431.......  441.......
P855978        356 AACCAACATG  GCGGCCCCCT  ACAAA-CTTC  AAGCGCCGGT  CTCTCCTAGC
P855979        318 ATACAACATG  GCAGCTCCCT  ACAAAACTTC  AAGCATCTGC  TCCCATCAGC
P855980        288 ATACAATATG  GCAGCTCCCT  ACAAAACTTC  AAGCATCTGC  TCCCATCAGC
                   **********  **********  **********  **********  **********
                   **********  **********  **********  **********  **********
                   **********  **********  **********  **********  **********
                   ******  *** **********  **********  **********  **********
                   ******  *** **********  **********  **********  **********
                   ******  *** **********  **********  **********  **********
                   ******  *** **********  *****                         ****
                           *** **********  *****
                           *** **********  *****

alignment position 451.......  461.......  471.......  481.......  491.......
P855978        405 CAATCAAAAG  AAAgatattt  ctgaatcccg  cctCTTTCAG  CAGCCTTGCT
P855979        368 CAATTAGAGA  AAAATATAGT  CCTGGGCCCG  ---CCTCCTa  caggaccctA
P855980        338 CAATTAGAGA  AAAATATAGT  CCTGGGCCCG  ---CCTCCTG  CAGCACCGCA
                   **********  **********  **********     *******  **********
                   **********  **********  **********     *******  **********
                   **********  **********  **********     ******            *
                   **********  **********  **********     ******            *
                   **********  **********  **********     ******            *
                   **********  **********  **********     ******
                   **********  ***                        ******

alignment position 501.......  511.......  521.......  531.......  541.......
P855978        455 GGCCAATCAG  ACCACACCAT  GAAGCATCAA  TAcaG-AAAA  GGGGGAACCC
P855979        415 GGCCAATCAG  ACCAAAGAAG  GAAAGGGCAC  TATGGaAAAA  GGCGTGAGAA
P855980        385 GGCCAATCAG  ACCAAAGAAG  GAAAGGGAAC  TCTGG-AAAA  GGCGTGAGAA
                   **********  **********  **********  ***** ****  **********
                   **********  **********  **********  ***** ****  **********
                   **********  **********  **********  ***** ****  **********
                   **********  **********  **********  ***** ****  **********
                   **********  **********  **********  **   * ****  **********
                   **********  **********  ***               ****  **********

alignment position 551.......  561.......  571.......  581.......  591.......
P855978        504 GCTGGCCCAA  TGGCAGCGTC  CTacAGTGTA  GCCTCCGCCT  CCCGATTGAC
P855979        465 GCTCGCCCAA  TGGCAGCATC  CTG-AGCTTA  GGCTCCGCCT  TCCGGTTGAA
P855980        434 GCTCGTCCAA  TGGCAGCATC  CcG-AACTTA  GGCTCCGCCT  TCCGGTTGAC
                   **********  **********  *** ******  **********  **********
                   **********  **********  *** ******  **********  **********
                   **********  **********  * * ******  **********  **********
                   **********  **********  * * ******  **********  **********
                   **********  **********  *       **  **********  **********
                   **********  **********  *
                   **********  **********  *

alignment position 601.......  611.......  621.......  631.......  641.......
P855978        554 TGGCCTgCTT  GGCAAGGCAA  GTAGCGGCGG  CGCTTCAAGG  TGGGTGTtca
P855979        514 CCGCAT-CTG  GGCCAGGGAA  GGAGCCGCAG  CACTTCAAGG  TGGGTGgCCA
P855980        483 CCGCAc-CTG  GGCCAGGGAC  GGAGCCGCTG  CACTTCAAGG  TGGGTGTCCA
                   ******  *** **********  **********  **********  **********
                   *****   *** **********  **********  **********  **********
                   *****   *** **********  **********  **********  *******
                   *****   *** **********  **********  **********  ******
                           *** **********  **********  **********  ******
                           *** **********  **********  **********  ******
                           *** **********  **********  **********  ******
                           *** **********  **********  **********  ******
                           *** **********  **********  **********  ******

alignment position 651.......  661.......  671.......  681.......  691.......
P855978        604 cgttttggcc  gtgaaagtc-  GGGCTTGGGC  AGCGTGGCGT  CGGGGGCGAG
P855979        563 GGGCAAGGCG  GCGAAGACAG  GGtc-TGGGT  GACGCAACGA  CAGGGTTGAA
```

```
P855980             532 AGGCAAGGCA GCGAAGACAG GGGTTTGGGT GACGCAACGA CAGGGTCGga
                        ********** ********** ********** ********** **********
                        ********** ********** **   ***** ********** ******
                                                   ***** ********** ******

alignment  position     701....... 711....... 721....... 731....... 741.......
P855978             653 TGTGGTGTCC CCGTTGGCGC CGCCAGCTCT gcgcctgggc ttcccctcgg
P855979             612 CGGGGTGTCA CCGCTGACCT TATGAAGTCT GGGATCTAGC GctTCGG---
P855980             582 c-GGGTGTCA CCACTGACCT CGTGAAGTCT TGTATCTAGC GggTCGG---
                        ********** ********** ********** ********** *  ****
                          ******** ********** ********** ********** *  ****
                          ******** ********** ********** ********** *
                          ******** *****

alignment  position     751....... 761....... 771....... 781....... 791.......
P855978             703 ctcaCTCAGG ATCCGCGGCC TCCGTGTCCT TCA-GGTGCA GCGGggagcc
P855979             659 ----CTCGGG ACCCGCGGCT TGTGCGTCCT TCC-CGAGCT GCGGACCggA
P855980             628 ----CTCCGG ACCTGCGGCT TCTGTGCCCT TtcgCGAACT GCGGGCCa-A
                            ****** ********** ********** *** ****** *******  *
                            ****** ********** ********** *** ****** ****     *
                            ****** ********** ********** *                   *
                            ****** ********** ********** *                   *

alignment  position     801....... 811....... 821....... 831....... 841.......
P855978             752 ggctctca-- ----GGGCGC ATGCGCCGCC cgctctgcgc caccggccta
P855979             704 AAGCCTGCAC CCTCAGGCGC ATGCGCTGCC TCGCCGGcTG CCTTCTGCGT
P855980             673 ACGCCCGCAC CCTCGGGCGC ATGCGCTGCC TCGCCGGaTG CCTTCTGCGT
                        ********** ********** ********** ******* ** **********
                        ********** ********** ********** ******* ** **********
                        ********** ********** ********** ******* ** **********
                        ********** ********** ********** ******* ** **********
                                       ****** **********         ** **********
                                                                 ** **********

alignment  position     851....... 861....... 871....... 881....... 891.......
P855978             796 gt-TAGGGTC GGAGATCACT GGGAAGGTg- --GGGAAGGT GCTGGGTTGG
P855979             754 TCATAGGGTC AGAGGTCAAG GGGAAGGTCT GAGAGAAAGT GGTAGGTTGG
P855980             723 TCATAGGGTC AGAGGTCAAG GGGAAGGTCT GAGAGAAAGT GGTAGGTTGG
                        ********** ********** ********** ********** **********
                        ********** ********** ********** ********** **********
                        ********** ********** ********** ********** **********
                        ********** ********** ********
                        ********** ********** ********
                        ********** ********** ********
                           ******* ********** ********
                           ******* ********** ********

alignment  position     901....... 911....... 921....... 931....... 941.......
P855978             842 -GTCCctgga actgcagtca gtgatccaaa ggagtgggcc tgttaggggg
P855979             804 -ATCCAGGAT TGCTTGGGGT CAAGAATTGG CCGACCC-CT ACAGGTCAAA
P855980             773 gATCCAGAAT TGCCTTGGGT CAAGAATTAG CCGACCCgCT ACAGGTCAAA
                         ********* ********** ********** ******* ** **********
                         ********* ********** ********** ******* ** **********
                         ********* ********** ********** ******* ** **********
                         ****                                    ** **********
                                                                 ** **********
                                                                 ** **********

alignment  position     951....... 961....... 971....... 981....... 991.......
P855978             891 ttcacaggtc gtgggATGAG CTCTTATGAG CGCCGGCTAA GTGCCGGGCT
P855979             852 GGTCAC---- -----AAGAG GGCGCTAGAG CGTTGGTGAT CTGCCAGACT
P855980             823 GGTCAC---- -----AAGAG GGCGCTAGAG CGTTGGTGAT GTGCCAGACT
                        ******          ***** ********** ********** **********
                        ******          ***** ********** ********** **********
                        ******          ***** ********** *******
                        ******          ***** ********** *******
                        ******          ***** ********** *******
                                                     *** *******
```

```
alignment position 1001...... 1011...... 1021...... 1031...... 1041......
P855978        941 CTGGGgtgcc cttggcatgt acgggctc-- CCCTAATTCC CAAGACCaac
P855979        893 CTTGGTCAAA ATCCCCTTGt TTGCATACAT CCCTAATTTT CGGGACC---
P855980        864 CTTGTTCAAC ATCCCCTTGg TTGCATACAT CCCTAATTTT CGGGACC---
                   ********** ********** ********** ********** *******
                   ********** ********** ********** ********** *******
                   ********** ********** ********** ********** *******
                                         ********** ********** *******
                                         ********** ********** *******
                                         ********** ********** *******

alignment position 1051...... 1061...... 1071...... 1081...... 1091......
P855978        989 cgcCCCCAAC CCACTTCATA GGTGAGAAgA CGGAGGTCAG GTCACATCAT
P855979        940 ---CCTCTTC CTACATtgta atCCAGAA-A CTGAGGCCAG GGGACATCAT
P855980        911 ---CCTCTTC CTACATCGCA GGCCAGAA-A CTGAGACCAA GGGACATCAT
                      ******* ********** ******** * ********** **********
                      ******* ******       ****** * ********** **********
                      ******* ******       ****** * ********** **********
                      ******* ******       ****** * ********** **********
                      ******* ******       ****** * ********** **********
                      ******* ******            * ********** **********
                                                * ********** **********
                                                                *******
                                                                *******
                                                                *******

alignment position 1101...... 1111...... 1121...... 1131...... 1141.
P855978       1039 CTGCCCAAGG TCACGCGGCA GGCTGGGGga cagt------ -----
P855979        986 TTGCCCAAGG TCACtc---- ---------- ---------- -----
P855980        957 TTGCCCAAGG TCACTCCGCA GGCTGGGGag agttccagga ggtcc
                   ********** ********** ********
                   ********** ********** ********
                   ********** ********** ********
                   ********** ********** ********
                   ********** ****
                   ********** ****
                   ********** ***
                   ********** ***
                   ********** ***
                   ********** ***
```

Pairwise similarities:

For each pairwise alignment, the similarity (relative to the maximum similarity) and the number of identical nucleic acids (in % of shorter sequence) is given. Maximum values are underlined.

	P855979 (1001 bp)	P855980 (1001 bp)
P855978 (1072 bp)	0.147<br>42 %	0.153<br>45 %
P855979 (1001 bp)		<u>1.000</u><br><u>84 %</u>

Please note that the **similarity value 1.000** marks only the two **most similar** sequences, it does not necessarily mean that these sequences are identical.

**Extract alignment region**	
**Positions in alignment**	from: to:
**Output file**	dialign.seq

Extract aligned sequences

For comments, questions or bug reports, please contact support@genomatix.de

© Genomatix Software GmbH 1998-2005
- All rights reserved.

License Agreement

GEMS Launcher 4.1.1

Genomatix-Logo Logout Personal Messages GEMS-Logo
Logout Personal Messages

Genomatix Main GEMS ElDorado Gene2Promoter BiblioSphere
Frequently asked questions Your Results Your Sequences Your Protocol Help

GEMS Launcher Task: *DiAlign TF*: Multiple alignment plus TF sites
working on promoters.seq (3 seq.)

[Alignment] [Pairwise similarities]

DiAlign professional TF Release 3.1 December 2004 **Wed Jun 8 00:54:44 2005**

Solution parameters:

**Sequence file:**	promoters.seq (3 seq.)
**Type of sequences:**	nucleotide sequences
**Threshold T:**	0.00
**'*' signs below alignment denote:**	diagonal similarity (max. similarity is represented by 10 '*' signs)
**Alignment output:**	complete alignment is shown<br># nucleic acids per line: 50
**TF output:**	common TF matches located in aligned regions (common to 3 (100.0 %) sequences)
**Family matches:**	yes
**MatInspector library:**	Matrix Family Library Version 5.0 (February 2005)
**Selected groups** (core/matrix sim):	• ALL user_defined.lib (0.75/Optimized)<br>• ALL vertebrates.lib (0.75/Optimized)

Aligned Sequences:

No.	Sequence Name	Sequence Description	Length
1	P937020	sym=NDUFV1|loc=Loc4723|taxid=9606|spec=Homo sapiens|chr=11| ctg=NT_033903|str=(+)|start=12679702|end=12680702|len=1001| tss=501| comm=NADH dehydrogenase (ubiquinone) flavoprotein 1, 51kDa; (NM_007103/537/bronze;)	1001 bp
2	P937022	sym=Ndufv1|loc=Loc17995|taxid=10090|spec=Mus musculus| chr=19|ctg=NT_082868|str=(-)|start=800669|end=801695| len=1027|tss=501,522,527| comm=NADH dehydrogenase (ubiquinone) flavoprotein 1; (AK075692/527/gold;AK005021/522/gold;NM_133666/501/bronze;)	1027 bp
3	P937024	sym=na|loc=Loc293655|taxid=10116|spec=Rattus norvegicus| chr=1|ctg=NW_047563|str=(-)|start=15125430|end=15126430| len=1001|tss=501| comm=similar to NADH dehydrogenase (ubiquinone) flavoprotein 1; (XM_215176/531/bronze;)	1001 bp

Alignment (DiAlign format):

Please note that only **upper-case** letters are considered to be **aligned**. For more information, please have a look at the user guide.

V$HOMF V$NKXH V$TEAF V$CREB V$ETSF V$E2FF V$HOXH V$TALE V$HMTB

```
alignment position 1.........  11.........  21.........  31.........  41.........
P937020          1 ga--------  ----------  ----------  ----------  ----------
P937022          1 aaggtttaca  gacacacagc  cctgagaagt  cctatgcaaa  ggttctgggg
P937024          1 ----------  ----------  ----------  ----------  ----------
```

```
alignment position 51........ 61........ 71........ 81........ 91........
P937020      3 ---------- ---------- ---------- ---------- ----------
P937022     51 cAGCTTTGTG GGAGGTGAGg gacctcaccg gttctt---- ----------
P937024      1 -AGCTTTGTG GAAGGTGTGt gtggggatgg agtgggggag gcagcaatgg
                ********* *********

alignment position 101....... 111....... 121....... 131....... 141.......
P937020      3 ---------- ---------- --------GC ACACGGACGC CCGGGGtggg
P937022     87 -----TTGTT AATGAGTACT TCTCCAAGGC ACACGGATGC TCAGGGggag
P937024     50 ttctgTTGTT AATAAATACT TCCCCAGGGC ACACGGATGC TCAagagg--
                    ***** ********** ********** ********** ******
                    ***** ********** ********** ********** ***
                    ***** ********** ********** ********** ***

alignment position 151....... 161....... 171....... 181....... 191.......
P937020     25 gaagcccacA GTCTGGCAGG GCTCGCACTG GGCAGAATAA TTGCAACgta
P937022    132 cgctT----A GTTCAGCAGG GCTCGGCTAG TTCAGCGTAA TTACACCAGA
P937024     98 ----T----A GTTCAGCAGG GCTTGGCTAG GTCAGCGTAA TTACACCAGA
                   *     * ********** ********** ********** **********
                   *     * ********** *********    ******** **********
                         * ********** *********    ******** **********
                                                   ******** **********
                                                   ******** **********

alignment position 201....... 211....... 221....... 231....... 241.......
P937020     75 gTCATGAGGA TCGGGCTATG ACCTGCCTGA CTTTtcactc ctcccatgat
P937022    178 -TCAGGACGA TTCCGCTGTA GTCCAACTAA TTTTCTCACC AGCTTCCATT
P937024    140 -TCAGGACGA TCCCGCTGTA GCCCAACTAA TTCTCTCCCC AGCATCTATT
                ********* ********** ********** ********** **********
                ********* ********** *  ******* ********** **********
                ********* ********** *
                ********* ********** *
                ********* ********** *

alignment position 251....... 261....... 271....... 281....... 291.......
P937020    125 acaccgatgc ataccGCAGG TGCACACTAA ACGCCGAATC TATAGAAAGT
P937022    227 CGTgcatata ta---GCTGG TGTCAACTAG ACGCAAAGTC TGTAGGAAGT
P937024    189 TGTac----- ------CTGG TGCCCACTAA ACACAGAGCC TGTAGGAAGT
               ***             ***** ********** ********** **********
               ***             ***** ********** ********** **********
                                **** ********** ********** **********
                                  ** ********** ********** **********
                                  ** ********** ********** **********

alignment position 301....... 311....... 321....... 331....... 341.......
P937020    175 GGAACACacc aGGACCCAGC AGAAGGCGAC ATGCGAGGTT TGTTTACTtg
P937022    274 GGAAtacttg -GGATCCAGC AGAGGGCGAC ACGTGAGCCT GGTTTACTAG
P937024    228 GAAAAACtta -----CCAGC AGAGGGCGAC ATGTGAGCCT TGTTTTCTAG
               *******       ********* ********** ********** **********
               *******       ********* ********** ********** **********
               ****              ***** ********** ********** **********
               ****              ***** ********** ********** ********
               ****              ***** ********** ********** ********
                                 ***** ********** ********** ********

alignment position 351....... 361....... 371....... 381....... 391.......
P937020    225 tggcatcaaa ggctgggagc cggcgagcta aggagggagc gaaagagcga
P937022    323 GAAAA----- ---------- ---------- ---------- ----------
P937024    273 AGAAAggaaa ctg------- ---------- ---------- ----------
               *****
               *****
               *****

alignment position 401....... 411....... 421....... 431....... 441.......
P937020    275 gcggtgGGGA CAGCCACCGC TAAATGCCAG CCCGGtggag ggaggaaggc
P937022    328 ------GGAA GAGTCTCCGA GAAAGGAGAG TCTGGgcaAG C---------
P937024    286 ---------- --GTCTCCAA GAAAGGAGAG TCTGGctcAG C---------
```

```
                              **** ********** ********** *****     ** *
                                      ******** ********** *****     ** *

alignment position 451.......  461.......  471.......  481.......  491.......
P937020       325 ggacccaggc cggtGGGAC CACATCTCCC AGAAGCCCcg gcgccaaggc
P937022       363 ---------- -----TGGAC TACATCTCCC AGAAGGCCtA GCACGAGAGT
P937024       315 ---------- -----GGGAC TACATCTCCC AGAAGaaccA GTACCAGAAT
                                 ***** ********** *******  * **********
                                 ***** ********** *******  * **********
                                 ***** ********** *****    * **********
                                  **** ********** *****

alignment position 501.......  511.......  521.......  531.......  541.......
P937020       375 ac-------- --------TGGC CTCTCAGTCC TCCCCCGAGG AGGCGGGGCC
P937022       398 AAATTTCCGG TCTCTGTGCC CGCGCACCtt t------AGT GGGCGAGGCC
P937024       350 AAATTTCCGG TCTCTGTGCC CTCGCACCCC TTAGTGAGGG GGGTGGGGgg
                  ********** ********** ********** ********** **********
                  ********** ********** ********
                  ********** ********** ********

alignment position 551.......  561.......  571.......  581.......  591.......
P937020       411 GCTGGCG--- -------AAG TTGAAGGGgC GCAGCGATAC CGGTGCGCGG
P937022       442 GAGGACA--- -------GAG CTCAAAGG-A ACGGAGGTCC CTGGACACGC
P937024       400 aaggagcgcg gagcgtgGAG CGCGAAGG-C GCGGAGGCAT CTGGACACGC
                  *******            *** ******** * ********** **********
                                     *** ******** * ********** **********
                                                                      ***

alignment position 601.......  611.......  621.......  631.......  641.......
P937020       451 CGCTGGCGTC -GGGCGCGTG ACGTCTTTCC TGCGCGCCAC Cta------G
P937022       481 CACTGGCTGC -GGATGTGTG ACGCACTTCC TGTGCGCTCC CCAcCCCGTG
P937024       449 CTCTGGCTgc gGGACGTGTG ACGCACTTCC TGTGCGCCCC CCA-CCCGTG
                  *********  ********* ********** ********** ***  ******
                  ********   ********* ********** ********** ***  ******
                  ********   ********* ********** ********** ***  ******
                             ********* ********** ********** *         *
                                ****** ********** ******

alignment position 651.......  661.......  671.......  681.......  691.......
P937020       494 CGTCTCTATC GCGCCAGTTC CTCAGcctca gtgctaTGAA GGTGACAGCG
P937022       530 TGTTTCTGTC GCGCCGGTCC CGCGGTTCGG AGTTG-TGAA GGTGACAGCC
P937024       498 TGTTTCTGTC GCGCCGGTCC CTCGGTTCGG AGTTG-TGAA GGTGACAGCC
                  ********** ********** ********** *****  **** **********
                  ********** ********** *  ******* *****  **** **********
                  ********** ********** *  ******* *****  **** **********
                  ********** ********** *  ******* *****  **** **********
                                           ******* *****  **** **********
                                                          **** **********
                                                          **** **********
                                                          **** **********

alignment position 701.......  711.......  721.......  731.......  741.......
P937020       544 TGAGGTGACC CATCTGGCcc gCCGCGATGC TGGCAACACG GCGGCTGCTC
P937022       579 GGAGGTGACC CGTCTGGCTC -CCGCGATGC TAGCGGCACG GCATTTTCTC
P937024       547 GGAGGTGACt ggtccGGCTT -CCGCGATGC TAGCAGCACG GCATTTTCTC
                  ********** **********  ********* ********** **********
                  ********** **********  ********* ********** **********
                  ********** **********  ********* ********** **********
                  *********       *****  ********* ********** **********
                  *********       ***    ********* ********** **********
                  *********       ***    ********* ********** **********
                  *********              ********* ********** **********
                  *********

alignment position 751.......  761.......  771.......  781.......  791.......
P937020       594 GGCTGGTCGC TTCCCGCGCG GGTATCTGTG CGTTTCAGCG GCGACACGGT
P937022       628 GGCGGGTTGG TTCCCGTGCG GGTATCTGTG CGTTTCAGCA GCGGCACGGT
P937024       596 GGCGGGTCGG TTCCCGTGCG GGTATCTGTG CGTCTCAGCA GCGGCACGGT
```

```
                     ********** ********** ********** ********** **********
                     ********** ********** ********** ********** **********
                     ********** ********** ********** ********** **********
                     ******  ** ********** ********** ********** **********
                     ******  ** ********** ********** ********** **********
                     ******  ** ********** ********** ********** ********
                     ******  *  ********** ********** ********** ********
                             *  ********** ********** ********** ********
                                 ********* ********** ********** *** ****
                                 ********* ********** ********** *** ****

alignment position   801....... 811....... 821....... 831....... 841.......
P937020          644 GAGGCCCAGC CTGGCTGGGG CCACGGGTGT TTGGGGCCGG GTGtcgcggc
P937022          678 Ga-GCCCGCC CTGATTCAGG CCCACGCCTA TGGCGGAGGA GTGgcgaGCG
P937024          646 GAGGCCCACC CTGATTCTGG CCTGGGGTGT ATGc------ -------GTG
                     ********** ********** ********** ********** ***    ***
                     ********** ********** ********** ***
                     *  ******* ********** **
                     *
                     *

alignment position   851....... 861....... 871....... 881....... 891.......
P937020          694 cgcgcccgtg cacgagcagt ccctgGGGTC TGTAGTGGCC TCTGGAGAGC
P937022          727 GCGAGC---- ---------- -----GGGAT TGTGGTCCCC TCTGCAGAGT
P937024          683 GCGAGC---- ---------- -----GGGGT TTGGTTCCCC cggtagaag-
                     ******                     ***** ********** **********
                                                ***** **********

alignment position   901....... 911....... 921....... 931....... 941.......
P937020          744 CCTtctcccc aggccaggcg ggaaggcccc cgctCCGGCC TGGTTGAAgt
P937022          758 CCTCTCCTCT CCGAGCTACC AC-------- ----CCGGTC TGGATAAAAA
P937024          713 CCTCTCCTCT CCGGGCTACT AC-------- ----CCGGCC TGGATTAAAA
                     ********** ********** **             ****** **********
                     ********** ********** **             ****** **********
                     ********** ********** **             ****** **********
                     ***

alignment position   951....... 961....... 971....... 981....... 991.......
P937020          794 aggggaacgG GTCCCAACGC GGGGCCGACT CCCTtgaccc tctgacttgc
P937022          796 ct-AAC---G GTCCCAACTC CAGGCTGACG ATGTCCAGtC CA--------
P937024          751 atgAGC---G GTTCCAACTC GGGACTGATG CTCTCCGG-C CA--------
                        ***   * ********** ********** ******** * **
                                                               * **
                                                               * **

alignment position   1001...... 1011...... 1021...... 1031...... 1041......
P937020          844 tgGCTTCCCT TGTTTTACAG TCCTCGGCCT GGCGG-AAGT TTTGCAGGTT
P937022          834 --GCTACTCT TGTTTTAACA ACCCCGTCTT GGGGTgAGGG TTTGCAGGTG
P937024          789 --GCCTCTCT TGTTTTAACA ACCTCGTCCT AGGGT-AAGG TTTGCAGGTT
                       ******** ********** ********** ***** **** **********
                       ******** ********** ********** ***** **** **********
                       ******** ********** ********** *****   ** **********
                             ** ********** ********** *****   ** *********

alignment position   1051...... 1061...... 1071...... 1081...... 1091......
P937020          893 GTGAAGACCC CTAGtctttc a-TTCTCTCC TTGTCACAGC CTTGTAGGGC
P937022          882 TTGAGGCGTC TTAGATAATC TGTTATTTCT TCCTCGCAGC CCTACCGGGT
P937024          836 TTGAGGCATC TTAGCTAGTC TGTgttnctn ctncgcagnc tttcngnnnn
                     ********** ********** ********** ********** **********
                     ********** ********** ***
                     ********** ********** ***

alignment position   1101...... 1111...... 1121...... 1131...... 1141......
P937020          942 GTGAagGGGT CATTGCCCCA TTTagg---- ---------- ----------
P937022          932 ATCA--GGGT CTTCGCCCCA TTTcaaagtt cagaaaacag ttttgaaAAA
P937024          886 ncggnnntnn ccccnnntgn ngtnncagaa nncanntnng n------AAA
                     ****  **** ********** ***                          ***

alignment position   1151...... 1161...... 1171...... 1181...... 1191......
```

```
P937020    968 GATGCGCACC TCCTGAT- TT  CCCGTCCACT gctct ----- ----------
P937022    980 GATGTGTATC TCCTCAA- CC  CCCCGACAGT ATTTTGCTTC CAGTAGTGa-
P937024    930 AATGTGTATC CCCTCAnn CC  CCCCGACAGT GTTCTGGTTC CTGTAGTGgc
               ********** ******   **  ********** ********** ********
                                   **  ********** ********** ********

alignment position 1201....... 1211....... 1221
P937020   1002 ---------- ---------- --
P937022   1028 ---------- ---------- --
P937024    980 cagaaagcgc ttttgtgatc cc
```

Pairwise similarities:

For each pairwise alignment, the similarity (relative to the maximum similarity) and the number of identical nucleic acids (in % of shorter sequence) is given. Maximum values are underlined.

	P937022 (1027 bp)	P937024 (1001 bp)
**P937020 (1001 bp)**	0.270<br>45 %	0.281<br>42 %
**P937022 (1027 bp)**		<u>1.000</u><br><u>67 %</u>

Please note that the **similarity value 1.000** marks only the two **most similar** sequences, it does not necessarily mean that these sequences are identical.

**Extract alignment region**	
**Positions in alignment**	from: to:
**Output file**	dialign.seq

Extract aligned sequences

For comments, questions or bug reports, please contact support@genomatix.de

© Genomatix Software GmbH 1998-2005 - All rights reserved. License Agreement GEMS Launcher 4.1.1

Genomatix-Logo Logout Personal Messages GEMS-Logo
Logout Personal Messages

Genomatix MainGEMSElDoradoGene2PromoterBiblioSphere
Frequently asked questionsYour ResultsYour SequencesYour ProtocolHelp

GEMS Launcher Task: *DiAlign TF*: Multiple alignment plus TF sites
working on promoters.seq (3 seq.)

[Alignment] [Pairwise similarities]

DiAlign professional TF Release 3.1 December 2004 **Wed Jun 8 04:29:52 2005**

Solution parameters:

**Sequence file:**	promoters.seq (3 seq.)
**Type of sequences:**	nucleotide sequences
**Threshold T:**	0.00
**'*' signs below alignment denote:**	diagonal similarity (max. similarity is represented by 10 '*' signs)
**Alignment output:**	complete alignment is shown<br># nucleic acids per line: 50
**TF output:**	common TF matches located in aligned regions (common to 3 (100.0 %) sequences)
**Family matches:**	yes
**MatInspector library:**	Matrix Family Library Version 5.0 (February 2005)
**Selected groups** (core/matrix sim):	• ALL user_defined.lib (0.75/Optimized)<br>• ALL vertebrates.lib (0.75/Optimized)

Aligned Sequences:

No.	Sequence Name	Sequence Description	Length
1	P964855	sym=NDUFV2|loc=Loc4729|taxid=9606|spec=Homo sapiens|chr=18| ctg=NT_010859|str=(+)|start=9092225|end=9093225|len=1001| tss=501| comm=NADH dehydrogenase (ubiquinone) flavoprotein 2, 24kDa; (NM_021074/522/silver;)	1001 bp
2	P964858	sym=Ndufv2|loc=Loc72900|taxid=10090|spec=Mus musculus| chr=17|ctg=NT_039657|str=(-)|start=9606486|end=9607493| len=1008|tss=501,508| comm=NADH dehydrogenase (ubiquinone) flavoprotein 2; (AK078351/508/gold;AK013511/501/gold;)	1008 bp
3	P964862	sym=Ndufv2|loc=Loc81728|taxid=10116|spec=Rattus norvegicus| chr=9|ctg=NW_047818|str=(-)|start=8001731|end=8002731| len=1001|tss=501| comm=24-kDa subunit of mitochondrial NADH dehydrogenase; (XM_343638/508/silver;)	1001 bp

Alignment (DiAlign format):

Please note that only **upper-case** letters are considered to be **aligned**. For more information, please have a look at the user guide.

V$STAF V$NRF1 U$NRF1 V$ZF5F V$P53F V$GREF V$HNF4 V$EGRF

```
alignment position  1 . . . . . . . . .   1 1 . . . . . . . .   2 1 . . . . . . . .   3 1 . . . . . . . .   4 1 . . . . . . . .
P964855          1  - - - - - - - - - -   - - - - - - - - - -   - - - - - - - - - -   - - - - - - - - - -   - - - - - - - - - -
P964858          1  - - - - - - - - - -   - - - - - - - - A T   C A T T T C T A C C   T C C C A C T C A C   T G G C A T C C C G
P964862          1  c g c g t a g c a t   c g a c a g t c A T   C A T T T C T A C C   T C C C A C T T C C   T G G C A T T C C G
```

```
                                   ** ********** ********** ** *******
                                   ** ********** ********** ** *******
                                   ** ********** ********** ** *******
                                   ** ********** ********** ** *******

alignment position 51........  61........  71........  81........  91........
P964855       1 ----------  ----------  ----------  ----------  ----------
P964858      33 AAGTCAATtc  cagttTAGCC  TGTCGTTTTA  CAGACTCTAA  cgca-AAGGC
P964862      51 AAGTCAATtc  cagc-TAGCC  TATCCTTTTA  CAGACTCTAA  aagcgAAGTC
                ********        *****  **********  **********       *****
                ********        *****  **********  **********       *****
                ********                                             *****
                ********

alignment position 101.......  111.......  121.......  131.......  141.......
P964855       1 ---GGAACCA  AGACATTTGG  TGTCTATGGC  tagcaCACTG  GATAGCAGCC
P964858      82 TTGGGAGCCA  AGGCACTTGC  TGTCCAAGTC  -----CACTA  GGTGGCAGCC
P964862     100 TTGGAAGACA  AGGCACTTGC  TGTTCAGGTC  -----CACTA  AGTGGCAGCC
                **********  **********  **********       *****  **********
                **********  **********  **********       *****   *********
                **********  **********  **********       *****   *********
                   *******  **********  **********       *****   *********

alignment position 151.......  161.......  171.......  181.......  191.......
P964855      48 CTCTGTGTct  tgaattcgta  acatgtagaa  taagatttat  agtgggttgc
P964858     127 CTTTAAATAC  GATCGTATGT  AAGAAAAGAC  TCAACCAAAC  GAATTTTGAA
P964862     145 CTTTAAGTAC  GATCGGGTGT  AAGATAAGTC  TCAGCCGACC  GGATTGTGCA
                **********  **********  **********  **********  **********
                **********  **********  **********  **********  **********
                **********  **********  **********  *
                **

alignment position 201.......  211.......  221.......  231.......  241.......
P964855      98 aacccgactt  tattttAAC  TAACAGCAAT  ATAAGAGGAG  GTGGctaatc
P964858     177 AG--------  -------AAA  TAACTGCAAT  ACCAGAGGAG  GTGTTTGACA
P964862     195 GG--------  -------AAG  TAACTGCAAT  ACCAGAAGAG  GTGTTTGACA
                **                 ***  **********  **********  **********
                **                 ***  **********  **********  **********
                                   ***  **********  *****  ***  **********
                                   ***  **********  *****  ***  **********
                                                           ***  ****
                                                           ***  ***

alignment position 251.......  261.......  271.......  281.......  291.......
P964855     148 ct-----CAC  AAGGCTCTCC  ATG-------  ----------  ----------
P964858     212 GAACAAACAC  AAGATCCCCC  AGaaggtacg  cctcgtcctc  aacaaacaca
P964862     230 GAACAAACAC  AAGATCCTCC  AGG-------  ----------  ----------
                **********  **********  ***
                **********  **********  **
                **********  **********  **
                **********  **********  **
                       ***  **********  **

alignment position 301.......  311.......  321.......  331.......  341.......
P964855     166 ----------  --AGGGAAGC  Tcacccctat  aaatccaggc  gggcgcttcg
P964858     262 agagcctcga  gaAGGCGAGC  CTCGTCC---  ----------  ----------
P964862     253 ----------  --AGGCCCGC  TTCGTCC---  ----------  ----------
                              ********  *******
                              ********  *******
                              ********  *******
                              ********  *

alignment position 351.......  361.......  371.......  381.......  391.......
P964855     204 ttacccagga  gcccaaggtg  cagctccggg  acgcttcttt  TCAAGGCCGG
P964858     289 ----------  ----------  ----------  ----------  TCAGGGCGGG
P964862     268 ----------  ----------  ----------  ----------  TCAGGGCCGG
```

```
                                                                                             ** *** **** **
                                                                                             ** *** **** **
                                                                                             ** *** **** **
                                                                                             ** *** **** **

alignment position 401.......   411.......   421.......   431.......   441.......
P964855        254 GACccctgca  gcggcccgta  ggacacctcC  CTTTCCGAGC  ACGGACTGGT
P964858        299 GACTCAGCTG  GCCCAaccgg  GCCGG----C  CTTTTAGGGA  CTGGGCTGTT
P964862        278 GACTCAGCTT  GCCCA-----  GCCGG----C  CTTTTAGGGA  TTGGGCTGTT
                   *** *** *** *****       *****    *  *********  ** *** ****
                   *** *** *** *****       *****    *  *********  ** *** ****
                   *** *** *** *****       *****    *  *********  ** *** ****
                   ***

alignment position 451.......   461.......   471.......   481.......   491.......
P964855        304 CCCCGCCCCT  CCCCCGGgaa  gtctccgccc  acagggcCCC  AGCAGCCCCC
P964858        345 TCGGGCCCGC  CCACCGGt--  ----------  -------CCC  AGCAGCCCTC
P964862        319 ----------  ----------  ----------  --------CC  AGCAGGCCAC
                   *** *** ***  *******                      ***  ** *** ****
                                                             ***  ** *** ****
                                                              **  ** *** ****
                                                              **  ** *** ****

alignment position 501.......   511.......   521.......   531.......   541.......
P964855        354 GCGCGCCCTC  GCtccctccc  gtcagccccc  gccCCTCGGC  GAAGGGAGCG
P964858        376 GCGCGCCgct  ----------  ----------  --------GC  GTCAGGAACG
P964862        331 GCGCGCCCTC  GCgcg-----  ----------  ---CCCGGGC  GCCTGGAGCT
                   *** *** ***  **                           *******  ** *** ****
                   *** *** ***  **                                **  ** *** ****
                   *** ****                                                ******
                   *** ****                                                ******

alignment position 551.......   561.......   571.......   581.......   591.......
P964855        404 GCGTGCCGTC  CGGGTCGCCT  AGG-------  ----------  ----------
P964858        398 GCGTACCGTT  CGGGACGCCC  AGG-------  ----------  ----------
P964862        363 GCGCACTCTT  CCAGACGCCC  AGGctcgggc  tcgtgagcgt  gcgccgtgcg
                   *** *** ***  *********  ***
                   *** *** ***  *********  ***
                   *** *** ***  *********  ***
                   *** *** ***  *********  ***

alignment position 601.......   611.......   621.......   631.......   641.......
P964855        427 -CCTGGGGTC  GGGA--GCGC  GCACGCTGTG  CGCCCTGGGC  GCGctcggga
P964858        421 -CCAGGGCTC  GTGAgcGCGC  GCGCGCCGTG  CGTCCTGGGC  GCGGGGCCGC
P964862        413 tCCCGGGCTC  GTGA--GCGC  GCGCGCCGTG  CGTCCTGGGC  TCGGGGCCGC
                    ** *** ***  ****    ****  *********  *********  ** *** ****
                    ** *** ***  ****    ****  *********  *********  ** *** ****
                    ** *** ***  ****    ****  *********  *********  ** *** ****
                    ** *** ***  *       ****  *********  *********  ** *** ****
                    ** *** ***  *       ****  *********  *********   * *** ****
                                        ****  *********  *********   **
                                        ****  *********  *********   **
                                                                     **
                                                                     **

alignment position 651.......   661.......   671.......   681.......   691.......
P964855        474 ttctcgcctg  gc--------  ----------  ----------  ---GCGGCTG
P964858        470 GCGCGCCGGG  CCTCCGGGCT  CGTTCGTTCC  CaGTCAGCCT  AGAGCGGCCG
P964862        461 GCCCGCCGGG  CCTCCAGGCT  CGTTCGTTCC  CtGTCAGCCT  AGAGCGGCCA
                   *** *** ***  *********  *********  *  *******  ** *** ****
                   *** *** ***  *********  *********  *  *******  ** *** ****
                   *** *** ***  *********  *********  *  *******  ** *** ****
                   *** *** ***  *********  *********  *                ** ****
                   *** *** ***  *********  *********  *                ** ****
                                                                        ** ****

alignment position 701.......   711.......   721.......   731.......   741.......
P964855        493 GGGAAGGTGA  ACAGTGTGGC  CCGCCATGTT  CTtctccgcG  GCGCTCCGGG
P964858        520 GGGTCGGAGC  ATCGCGTACC  CCGCCATGTT  CTCCTT---G  GCGCTGCGGG
```

```
P964862             511 GGGTCGGAGA ACCGCGTACC CCGCCATGTT CTCCTT---G GCGCTGCGGG
                        ********** ********** ********** ******   * **********
                        ********** ********** ********** ******   * **********
                        ********** ********** ********** ******   * **********
                        ********** ********** ********** ******   * **********
                        ********** ********** ********** ******   * **********
                        ********** ********** ********** ******   * **********
                                        ***** ********** **       * **********
                                        ***** ********** **       * **********
                                        ***** ********** **       * **********
                                                                  * **********

alignment position      751....... 761....... 771....... 781....... 791.......
P964855             543 CCCGGGCGGC TGGCCTCACC GCCCACTGGg taAGGAGGCT CAAGCTGAG-
P964858             567 CCAGGGCGAC CGGCCTCGCT GCTCAGTGGG T-AAGAGGCT CGGGGTGAAc
P964862             558 CCAGGGCGTC CGGCCTCACC GCTCAGTGGG T-AAGAGGCT CGGGGTGAA-
                        ********** ********** ********** * ******** *********
                        ********** ********** ********** * ******** *********
                        ********** ********** ********** * ******** *********
                        ********** ********** ********** * ******** *********
                        ********** ********** ********** * ******** *********
                        ******** * ********** *********
                        ******** * ********** *********
                        ******** * ********** *********
                        ******** * ********** *********
                        ********

alignment position      801....... 811....... 821....... 831....... 841.......
P964855             592 -CCCGGCGCT GCCGCCGCcc tTCTCTTGCT CTCCGTGTCT CCCTTAGTTT
P964858             616 cCCGGGCACT GTCGCCACAG -TCTCCCGCT CTTCGTGCCT CCCTTAGCTT
P964862             606 -GCGGGCACT GTCGCCATAG -TCg-CCGCT CTTTATGTTT CTCTTAGCTT
                         ********* **********  ********* ********** **********
                          ******** **********  ********* ********** **********
                                               **  ***** ********** **********
                                                      *** ********** **********

alignment position      851....... 861....... 871....... 881....... 891.......
P964855             641 TG-GGCGCAG GAGAGCCCTG GGCTCTGCAC GGGCCTGCAG GCGGCGGCAA
P964858             665 TTaGTCCTCG GAGACCCCTA GGCGCTGGAC AGGTCAGCTG GCGGCGGCGG
P964862             653 TG-GTACCCG GAGACCCCAG GGTGCTGGAT AGGTCAGCTG GCGATGGCGG
                        ** ******* ********** ********** ********** **********
                        ** ******* ********** ********** ********** **********
                        *  ******* ********** ********** ********** **********
                        *  ******* ********** ********** ********** ***  *****
                                 * ********** ********** ********** ***  *****
                                 * ********                    **** ***  ***
                                                                         ***

alignment position      901....... 911....... 921....... 931....... 941.......
P964855             690 GGACACTGCG GCCTTAGCCC TCttagggag gccgctgcag ccccaCCGTG
P964858             715 GGACGCCGCG GCCTTCGTCC TCAGTGCCTC CGGGAtgctc cg-------C
P964862             702 AGACGCCGCG GCCTTCGTCC TGGGTGCCTC CGGGAtgc-- -----CCCTC
                        ********** ********** ********** *****           *****
                        ********** ********** ********** *****               *
                        ********** ********** ********** *****               *
                        ********** ********** ********** *****               *
                        ********** ********** **                             *
                        ********** ********** *

alignment position      951....... 961....... 971....... 981....... 991.......
P964855             740 AGAAGCCCCG AGGGAGGGTT GCCata---- ---------- CTGGGAAGTG
P964858             758 AGCTGCGGTG GCGGCGGGTG GCGGGCCGCC CGCACCGGGc CGGGGAAAGT
P964862             745 AGCTGCGGCG GCGGCGGGTG GCCGGCCGCC CGCACCGGGc cc-GGAAAGT
                        ********** ********** ********** *********  **********
                        ********** ********** ********** *********
                        ********** ********** ********** *********
                        ********** ********** ********** *********
                        ********** ********** ********** *********

alignment position      1001...... 1011...... 1021...... 1031...... 1041......
```

```
P964855     776 TACTCCTAGT TTGGTCGAGG aggaca---- ---------- ----------
P964858     808 CACCCCTAAC TTAGTCAAGG TCAGGAAGGC GaTTGTCATT TATCCTGGTG
P964862     794 CA-CCCTAAC TTAGTCAAGG TCAGGAAGGC GgTTGTCATT TGTCCCGGTG
                *** ****** ********** ********** * ******** **********
                    ****** ********** ********** * ******** **********
                    ****** ********** ********** * ******** **********
                    ****** ********** ********** *

alignment position 1051...... 1061...... 1071...... 1081...... 1091......
P964855     802 ---------- ---------- ---------- ---------- ----------
P964858     858 CGGAAGCTAG TGggggcCC TGGCGGCCCG CTGCTTCCAG GATCCTAGCA
P964862     843 CGGAACCTAG TGagat--CC TGGCGGCCTG CTGCTTCCAG GAACCCGGCA
                ********** **      ** ********** ********** **********
                ********** **      ** ********** ********** **********
                ********** **      ** ********** ********** **********
                                   ** ********** ********** **********

alignment position 1101...... 1111...... 1121...... 1131...... 1141......
P964855     802 --GAAAAGAG GGGCTGCTTG gtgatatata tatatatgtt tgtttgtttt
P964858     908 GCGAAGcttt GGGCTGCTTG TC-------- ---------- ----------
P964862     891 GCGAAGAAAG GGGCTTCTTG TC-------- ---------- ----------
                ********** ********** **
                ******     ********** **
                ******     ********** **
                ******

alignment position 1151...... 1161...... 1171...... 1181...... 1191......
P964855     850 tgctgttttg ctgtagcgca gaatctaggc ctgctctggc cagatcagtt
P964858     930 ---------- ---------- ---------- ---------- ----------
P964862     913 ---------- ---------- ---------- ---------- ----------

alignment position 1201...... 1211...... 1221...... 1231...... 1241......
P964855     900 tcgaagaccg tcgctccgaa ggaggcacct ctcgTTTCAA GCCTAGTGAC
P964858     930 ---------- ---------- ---------- ----TGTCAG GCATAGCGGC
P964862     913 ---------- ---------- ---------- ----TGTCAG ACTTAGCGGC
                                                     ****** **********
                                                     ****** **********
                                                     ****** **********

alignment position 1251...... 1261...... 1271...... 1281...... 1291......
P964855     950 CTCGATGCTT ttaggttgc- AGCATACTGG AGAg--CTCT GGCTTGCTTC
P964858     946 TGCGCTGCAT cggatttaaa AGCATGCTGG AGAAAACGCT GATAGGCTTG
P964862     929 AACGCTGCAa cagaattaaa gGCATGCTGG AGAAAACGCT GATGTGCTTG
                **********            ********** ********** **********
                ********                ******** ********** **********
                ********                ******** ********** **********
                                        ******** ********** **********
                                        ******** ***

alignment position 1301...... 1311...... 1321
P964855     997 GTGAA----- ---------- ---
P964858     996 GTAAAGGCTT Gcc------- ---
P964862     979 GTGAAGGCTT Gccgagttcc ttc
                ********** *
                ********** *
                ********** *
                ********** *
```

Pairwise similarities:

For each pairwise alignment, the similarity (relative to the maximum similarity) and the number of identical nucleic acids (in % of shorter sequence) is given. Maximum values are underlined.

	P964858	P964862

	(1008 bp)	(1001 bp)
**P964855 (1001 bp)**	0.194<br>40 %	0.166<br>34 %
**P964858 (1008 bp)**		1.000<br>79 %

Please note that the **similarity value 1.000** marks only the two **most similar** sequences, it does not necessarily mean that these sequences are identical.

**Extract alignment region**	
**Positions in alignment**	from: to:
**Output file**	dialign.seq

Extract aligned sequences

For comments, questions or bug reports, please contact support@genomatix.de

© Genomatix Software GmbH 1998-2005 - All rights reserved. License Agreement GEMS Launcher 4.1.1

Genomatix-Logo Logout Personal Messages GEMS-Logo
Logout Personal Messages

Genomatix_MainGEMSElDoradoGene2PromoterBiblioSphere
Frequently_asked_questionsYour_ResultsYour_SequencesYour_ProtocolHelp

GEMS Launcher Task: *DiAlign TF*: Multiple alignment plus TF sites
working on C1-NDUFV3.seq (3 seq.)

[Alignment] [Pairwise_similarities]

DiAlign professional TF Release 3.1 December 2004 **Wed Jun 8 05:00:37 2005**

Solution parameters:

**Sequence file:**	C1-NDUFV3.seq (3 seq.)
**Type of sequences:**	nucleotide sequences
**Threshold T:**	0.00
**'*' signs below alignment denote**:	diagonal similarity (max. similarity is represented by 10 '*' signs)
**Alignment output:**	complete alignment is shown<br># nucleic acids per line: 50
**TF output:**	common TF matches located in aligned regions (common to 3 (100.0 %) sequences)
**Family matches:**	yes
**MatInspector library:**	Matrix Family Library Version 5.0 (February 2005)
**Selected groups** (core/matrix sim):	• ALL user_defined.lib (0.75/Optimized)<br>• ALL vertebrates.lib (0.75/Optimized)

Aligned Sequences:

No.	Sequence Name	Sequence Description	Length
1	hNDUFV3	hNDUFV3 sym=NDUFV3|loc=Loc4731|taxid=9606|spec=Homo sapiens|chr=21|ctg=NT_030188|tss=761	1547 bp
2	mNDUFV3	mNDUFV3 sym=1500032D16Rik|loc=Loc78330|taxid=10090|spec=Mus musculus|chr=17|ctg=NT_039649|str=(+)|tss=455	1471 bp
3	rNDUFV3	rNDUFV3 sym=Mipp65|loc=Loc64539|taxid=10116|spec=Rattus norvegicus|chr=20|ctg=NW_047598|str=(+)|tss=420	966 bp

Alignment (DiAlign format):

Please note that only **upper-case** letters are considered to be **aligned**. For more information, please have a look at the user guide.

V$PCAT V$ZBPF V$ZF5F

```
alignment position  1.........  11........  21........  31........  41........
hNDUFV3          1  ggaatcccta  cacaatccat  ttaaatgact  gtggcagggc  ctcttcccct
mNDUFV3          1  tttgaactag  c---------  ----------  ----------  ----------
rNDUFV3          1  ctcatt----  ----------  ----------  ----------  ----------

alignment position  51........  61........  71........  81........  91........
hNDUFV3         51  gtggaagctg  aaggtggcac  gtccatctcc  gatgaaatgc  gaatggggct
mNDUFV3         12  ----------  ----------  ----------  ----------  ----------
rNDUFV3          7  ----------  ----------  ----------  ----------  ----------
```

```
alignment  position 1 0 1 . . . . . . .  1 1 1 . . . . . . .  1 2 1 . . . . . . .  1 3 1 . . . . . . .  1 4 1 . . . . . . .
hNDUFV3        101 c t g g g c a c g c  g g c g g a a g g c  a g a g a a g a g g  c a g a g A A A G A  A G C T A A T A A C
mNDUFV3         12 - - - - - - - - - -  - - - - - - - - - -  - - - - - - - - - -  - - - - - - - - - -  - - - - - - - - - -
rNDUFV3          7 - - - - - - - - - -  - - - - - - - - - -  - - - - - - - - - -  - - - - - A A T G A  A T T T A T C T A C
                                                                                          * * * * *  * * * * * * * * * *

alignment  position 1 5 1 . . . . . . .  1 6 1 . . . . . . .  1 7 1 . . . . . . .  1 8 1 . . . . . . .  1 9 1 . . . . . . .
hNDUFV3        151 T A A T T A C A C C  g a c t c c a c a a  t t a c g a c t a a  c g t t g g g g a c  g a a c t c t t t g
mNDUFV3         12 - - - - - - - - - -  - - - - - - - - - -  - - - - - - - - - -  - - - - - - - - - -  - - - - - - - - - -
rNDUFV3         22 T G A T T A A T C C  t t a c a - - - - -  - - - - - - - - - -  - - - - - - - - - -  - - - - - - - - - -
                   * * * * * * * * * *

alignment  position 2 0 1 . . . . . . .  2 1 1 . . . . . . .  2 2 1 . . . . . . .  2 3 1 . . . . . . .  2 4 1 . . . . . . .
hNDUFV3        201 c t a a a t a a a c  a t t t a g g t g a  a a c a a a g c t t  c c a t t t c c a a  t c g g c c t a a c
mNDUFV3         12 - - - - - - - - - -  - - - - - - - - - -  - - - - - - - - - -  - - - - - - - - - -  - - - - - - - - - -
rNDUFV3         37 - - - - - - - - - -  - - - - - - - - - -  - - - - - - - - - -  - - - - - - - - - -  - - - - - - - - - -

alignment  position 2 5 1 . . . . . . .  2 6 1 . . . . . . .  2 7 1 . . . . . . .  2 8 1 . . . . . . .  2 9 1 . . . . . . .
hNDUFV3        251 t c t g g t a t t a  g c g t t g g c a g  g g g c c a t g c a  g g a g g g g c a g  t g a g c a g t g g
mNDUFV3         12 - - - - - - - - - -  - - - - - - - - - -  - - - - - - - - - -  - - - - - - - - - -  - - - - - - - - - -
rNDUFV3         37 - - - - - - - - - -  - - - - - - - - - -  - - - - - - - - - -  - - - - - - - - - -  - - - - - - - - - -

alignment  position 3 0 1 . . . . . . .  3 1 1 . . . . . . .  3 2 1 . . . . . . .  3 3 1 . . . . . . .  3 4 1 . . . . . . .
hNDUFV3        301 t g g c c g c G T T  G C C G A C A G A G  G G T G A G G G A G  C T G G G C G C G T  g g t g t g g g c g
mNDUFV3         12 - - - - - - - - - -  - C C T A C T G C T  G G T A T G G T A G  G T G G G C A C C T  C C A t g C C C A G
rNDUFV3         37 - - - - - - - G T T  G C C T A G T G C T  G G T A A G G T A G  G T G G G C A C C A  C C A c t C C C A G
                                 * * *  * * * * * * * * * *  * * * * * * * * * *  * * * * * * * * * *  * * *     * * * * *
                                          * * * * * * * * *  * * * * * * * * * *  * * * * * * * * * *  * * *     * * * * *
                                          * * * * * * * * *  * * * * * * * * * *  * * * * * * * * * *  * * *     * * * * *
                                          * * * * * * * * *  * * * * * * * * * *  * * * * * *                     * * * * *

alignment  position 3 5 1 . . . . . . .  3 6 1 . . . . . . .  3 7 1 . . . . . . .  3 8 1 . . . . . . .  3 9 1 . . . . . . .
hNDUFV3        351 c c g a g g t c c c  g a g c t g a a g a  g c t t t - - - - -  - - - - - - - - - -  - - - - - - - - - -
mNDUFV3         51 C T T C T G G T T T  G T T T G T T C A A  G A C C A C G C C C  C T A c t g G G C A  A G C G C A C T A C
rNDUFV3         80 C T T C C G G T T T  G T T T G T G C A A  G G C C A C G C C C  C T A t t a G G C A  A A C G C C C T A C
                   * * * * * * * * * *  * * * * * * * * * *  * * * * * * * * * *  * * *       * * * *  * * * * * * * * * *
                   * * * * * * * * * *  * * * * * * * * * *  * * * * * * * * * *  * * *       * * * *  * * * * * * * * * *
                   * * * * * * * * * *  * * * * * * * * * *  * * * * * * * * * *  * * *       * * * *  * * * * * * * * * *
                   * * * * * * * * * *  * * * * * * * * * *  * * * * * * * * * *  * * *

alignment  position 4 0 1 . . . . . . .  4 1 1 . . . . . . .  4 2 1 . . . . . . .  4 3 1 . . . . . . .  4 4 1 . . . . . . .
hNDUFV3        376 - - - - - - - - - -  - - - - - - - - - -  - - - - - - - - - -  - - - - - - A A A A  C C T C T C A A A A
mNDUFV3        101 C A C C G A G T T G  T A T C T C C A A G  A A A C A A C A A A  C T C A C A A A G A  T C T C A C C A A A
rNDUFV3        130 C A C C T A G T T G  T A T C T C C A A G  A A A C A A C A A A  C T C A C A A A A A  T C T C A C C A A A
                   * * * * * * * * * *  * * * * * * * * * *  * * * * * * * * * *  * * * * * * * * * *  * * * * * * * * * *
                   * * * * * * * * * *  * * * * * * * * * *  * * * * * * * * * *  * * * * * * * * * *  * * * * * * * * * *
                   * * * * * * * * * *  * * * * * * * * * *  * * * * * * * * * *  * * * * * * * * * *  * * * * * * * * * *
                                                      * *  * * * * * * * * * *  * * * * * * * * * *  * * * * * * * * * *
                                                      * *  * * * * * * * * * *  * * * * * * * * * *  * * * * * * * * * *
                                                      * *  * * * * * * * * * *  * * * * * * * * * *  * * * * * * * * * *
                                                                                             * * * *  * * * * * * * * * *

alignment  position 4 5 1 . . . . . . .  4 6 1 . . . . . . .  4 7 1 . . . . . . .  4 8 1 . . . . . . .  4 9 1 . . . . . . .
hNDUFV3        390 C A C A G A A C T A  T C a a T C A A G T  A G G A C C A G A c  t g t g c c c c c a  g g g a a g g t c g
mNDUFV3        151 C A C A G A A G T C  T C - - T C A A G T  C G A A C C A G A A  A C G C A A A G G A  C A T G A T T C T A
rNDUFV3        180 C A C A G A A G a C  T C - - T C A A G A  C G A A C C A G A A  A C G C A A A G G A  C A C G A T C C T A
                   * * * * * * * * * *  * *     * * * * * *  * * * * * * * * * *  * * * * * * * * * *  * * * * * * * * * *
                   * * * * * * * *   *  * *     * * * * * *  * * * * * * * * * *  * * * * * * * * * *  * * * * * * * * * *
                   * * * * * * * *   *  * *     * * * * * *  * * * * * * * * * *  * * * * * * * * * *  * * * * * * * * * *
                   * * * * * * * *   *  * *     * * * * * *  * * * * * * * * * *  * * * * * * * * * *  * * * * * * * * * *
                   * * * * * * * *   *  * *     * * * * * *  * * * * * * * * * *  * * * * * * * * * *  * * * * * * * * * *
                   * * * * * * * *
                   * * * * * * *

alignment  position 5 0 1 . . . . . . .  5 1 1 . . . . . . .  5 2 1 . . . . . . .  5 3 1 . . . . . . .  5 4 1 . . . . . . .
```

```
hNDUFV3    440 ---------- ---------- -------CAG ATTTAAGGCT GCGAggacaa
mNDUFV3    199 GctgtgCAAT TTTGGTAGGT TACAGACCAG AGTTAAGGGC GAGATCAGGG
rNDUFV3    228 GttgcaCAAC TTTAGTAGGT TGCAGACTAG AGTTAAGGGT GAGATCAGGG
               *     **** ********** ********** ********** **********
               *     **** ********** ********** ********** **********
               *     **** ********** ********** ********** **********
               *     **** ********** ********** ********** **********
               *

alignment position 551....... 561....... 571....... 581....... 591.......
hNDUFV3    463 gggcaaagaa gtgcgaagcg ttcgGGTGGA CATGCaagcc ggcccagcgc
mNDUFV3    249 CAA------- ---------- ----GGTGGG CAGGCCCAAA TCGGAGGG--
rNDUFV3    278 CAA------- ---------- ----GGTGGA CAGGCCCAGA TCGCAGGG--
               ***                       ****** ********** ********
               ***                       ****** ********** ********
               ***                       ****** ********** ********
               ***                       ****** ********** ********

alignment position 601....... 611....... 621....... 631....... 641.......
hNDUFV3    513 agacgcctga gaggggtgcc cagcagcccc cGCAGGTCTC CGCCGCGCCC
mNDUFV3    276 ---------- ---------- ---------- -GCGGGACTT tcagcgggat
rNDUFV3    305 ---------- ---------- ---------- -GCAGGACTT CCACCCAGAC
                                                 ********* **********
                                                 *********
                                                 *********
                                                 *********

alignment position 651....... 661....... 671....... 681....... 691.......
hNDUFV3    563 CCGCCACAGC GCCcaggcgc ccgcagggca caggggatcgg caccagccgg
mNDUFV3    295 cta-CCCAGG GGAGGACCCC ACGCTTTCCC gaA------- ----------
rNDUFV3    324 CTGCCCAAGG GGCGGGCACC AAGCACTCTC --A------- ----------
               ********** ********** **********   *
                                                  *
                                                  *
                                                  *

alignment position 701....... 711....... 721....... 731....... 741.......
hNDUFV3    613 tgacgtgtcc ctcacgctgc tgCCTGTGAT TGGTGGTCCC GATCCGGGGC
mNDUFV3    327 ---------- ---------- --CCCGGAAT TGGTGGACTC AGTTAGGGGC
rNDUFV3    355 ---------- ---------- --CCCGGGAT TAGTGGACTC AGTCAGAGGC
                                       ******** ********** **********
                                       ******** ********** **********
                                       ******** ********** **********
                                       ******** ********** **********
                                       ******** ********** **********

alignment position 751....... 761....... 771....... 781....... 791.......
hNDUFV3    663 GTGGCCCCAA GGgaggcact tagccctact ggggatgcgc gcgcagaccc
mNDUFV3    355 GTGGCCTTAA GGAGCCCTCC CAGAA----- ---------- ----------
rNDUFV3    383 GTGGCCTctg GGAGCCCGCC CAGAA----- ---------- ----------
               ********** ********** *****
               ********** ********** *****
               *******    ********** *****
               *******    ********** *****
               *******    **

alignment position 801....... 811....... 821....... 831....... 841.......
hNDUFV3    713 acggcctctg ctccgccccc agctggcctg taattggcgc agtcggccca
mNDUFV3    380 ---------- ---------- ---------- ---------- ----------
rNDUFV3    408 ---------- ---------- ---------- ---------- ----------

alignment position 851....... 861....... 871....... 881....... 891.......
hNDUFV3    763 aggggcggct gttcaggcgc gggTGCGCGC GCAGCTGCTG TGGCCCTGCT
mNDUFV3    380 ---------- ---------- ---TGCGCGC GCAAACGCTT GGGTCCACCA
rNDUFV3    408 ---------- ---------- ---TGCGCGC GCAAGCGCTT GGCCCCACCC
```

```
                                              *******  **********  **********
                                              *******  **********  **********
                                              *******  **********  **  ******
                                              *******  **********  **  ******
                                              *******  **********  **
                                              *******  **********  **

alignment position  901.......  911.......  921.......  931.......  941.......
hNDUFV3        813  TGGTGCGCcc  gctgtcacc-  ----------  ----------  ----------
mNDUFV3        407  TGGTGTGCGA  TTGGTGAACG  TGgACGCGGG  GCGGAGCCTG  GGGTGGTGCG
rNDUFV3        435  TGGTGTACGA  TTGGCGAACG  TG-ACGCGGG  GCCGAGCCTC  GGGTTGTGCG
                    **********  **********  ** *******  **********  **********
                    **********  **********  ** *******  **********  **********
                    ********                   *******  **********  **********
                    *****

alignment position  951.......  961.......  971.......  981.......  991.......
hNDUFV3        832  ----------  ----------  ----------  ----------  ----------
mNDUFV3        457  TGTGCACaag  tAGGTCACGT  ACCTAAGTGC  GACCTCCGCT  CTTGGCATTT
rNDUFV3        484  GGTGCACgct  cAGGTTACGT  ACCTAGGTGC  GACCTCGGCG  CTGGGCGTCT
                    *******      *********  **********  **********  **********
                    *******      *********  **********  **********  **********
                    *******      *********  **********  **********  **********

alignment position  1001......  1011......  1021......  1031......  1041......
hNDUFV3        832  --GCCATGGC  tgccccgtgt  tTGCTGCGGC  AAGGACGAGC  CGGGGCGCTG
mNDUFV3        507  GtGCCATGGC  GGTCTCTCTC  CTCCTGCGGG  GAGGACGGAT  CCGGGCGCTG
rNDUFV3        534  GtaCCATGGC  GGTCTCTCTC  CTGCTGCGGG  GAGGACGAAT  CCGGGCGCTG
                    * ********  **********  **********  **********  **********
                    *  *******  **********  **********  **********  **********
                    *  *******  **********  **********  **********  **********
                       *******  **********  **********  **********  **********
                       *******  **********  **********  **********  **********
                                             *********  **********  **********
                                             *********  **********  **********
                                             *********  **********  **********
                                               *******  **********  ***
                                               *******  **********  ***

alignment position  1051......  1061......  1071......  1081......  1091......
hNDUFV3        880  AAGGTAAAGG  aggagccagc  ctgggctggc  tgcgcggccc  cgagcctacg
mNDUFV3        557  AAGGTAAAGG  GCTAACGGGC  GGGA------  ----------  ----------
rNDUFV3        584  AAGGTAAAAA  GCGAACGGGA  GAGA------  ----------  ----------
                    **********  **********  ****
                    **********  **********  ****
                    **********  **********  ****
                    **********
                    **********
                    ********
                    ********
                    ********

alignment position  1101......  1111......  1121......  1131......  1141......
hNDUFV3        930  taggggatgg  ggtgaggggg  cgcggtgctg  gtcaggtccg  ggcctgtccg
mNDUFV3        581  ----------  ----------  ----------  ----------  ----------
rNDUFV3        608  ----------  ----------  ----------  ----------  ----------

alignment position  1151......  1161......  1171......  1181......  1191......
hNDUFV3        980  cgacctctag  ccgtcctagt  tgggccgctg  accccgctcc  ctgggcccag
mNDUFV3        581  ----------  ----------  ----------  ----------  ----------
rNDUFV3        608  ----------  ----------  ----------  ----------  ----------

alignment position  1201......  1211......  1221......  1231......  1241......
hNDUFV3       1030  gactgaccgc  cacggggcca  ggcccggatg  tggccagcga  gcgtcccgcg
mNDUFV3        581  ----------  ----------  ----------  ----------  ----------
rNDUFV3        608  ----------  ----------  ----------  ----------  ----------
```

```
alignment position 1251...... 1261...... 1271...... 1281...... 1291......
hNDUFV3      1080 gttcccCCGA GCCGGTCCgc cgccgccgct gcccgaggcc caggccgagg
mNDUFV3       581 ------CATA CCGGGcCCTT AGCCTGACCC TTA------- ----------
rNDUFV3       608 ------CCTA CCCGGTCCTT AGTCTGGCCC TTA------- ----------
                        **** ********** ********** ***
                        **** ***** **** ********** ***
                        **** ***** **** ********** ***
                                   **** ********** ***

alignment position 1301...... 1311...... 1321...... 1331...... 1341......
hNDUFV3      1130 ctggcgtgcc ccgtgccgac gggcgctccc gaggGCTGCA TGCCTGGGCg
mNDUFV3       608 ---------- ---------- ---------- ----ACTGCA CCCGTGGGCC
rNDUFV3       635 ---------- ---------- ---------- ----GCTGCA CGCGTGGGCT
                                                      ****** **********
                                                      ****** **********
                                                      ****** **********
                                                      ****** **********

alignment position 1351...... 1361...... 1371...... 1381...... 1391......
hNDUFV3      1180 gcagactcgg gtggacgttc ccaggtgatg ctgccgcgcg gctggggtca
mNDUFV3       624 CGGTGCGgag -AGGGGTCCT AGGGCCTGTG CTCACCTGAC CCggacAGAA
rNDUFV3       651 CGGTGCGgta aAGAGGTCCC AGGGCCTGTG CTCACCTGAC CCcaagAGAA
                  *******     ********* ********** ********** **    ****
                  *******     ********* ********** ********** **    ****
                  *******     ********* ********** ********** **
                  *******     ********* ********** ********** **

alignment position 1401...... 1411...... 1421...... 1431...... 1441......
hNDUFV3      1230 gccgctgtgc ggctcccgca cGGCTTCTCT GAAGGAcagt gacttctcca
mNDUFV3       673 GG-------- ---------- -GTTTTCTCT GCAGGAGTGA AACCGAGtca
rNDUFV3       701 GT-------- ---------- -GGTTTCTCT GCAGGAGCGA AGCCAAGgct
                  **                     ********* ********** ******
                  **                     ********* ********** ******

alignment position 1451...... 1461...... 1471...... 1481...... 1491......
hNDUFV3      1280 tttagccaag agaggaaatg agggaacttt cagggacctg gttaacttgg
mNDUFV3       704 ctgaGGCTGT TTTTAAAACG AAACCTGGCT TTCTCTGCAA AActAGTATT
rNDUFV3       732 ccttGGCTGT TTAAAAAGCA AAACCCGACT TTCTCTGCAA AAtcAGTGTT
                      ****** ********** ********** ********** **  ******
                      ****** ********** ********** ********** **  ******
                      ****** ********** ********** ********** **  ******
                                                                  ******
                                                                  ******

alignment position 1501...... 1511...... 1521...... 1531...... 1541......
hNDUFV3      1330 taacttactg acagccgttg a--------G AAGCCAGCTG ACATGACCTG
mNDUFV3       754 GGGTTTTGTT TGGGTTGGGT TCGGTGGATG GAACCTAGGG CCCTGCGCTT
rNDUFV3       782 GGGGTTTGTT TGGGTTGGGT TCGGTGGATG AAACCTAGGG CCCTGCGCTT
                  ********** ********** ********** ********** **********
                  ********** ********** ********** ********** **********
                  ********** ********** ********** ********** **********
                  ********** ********** ********** ********** **********
                  ********** ********** ********** ********** **********
                                                      ****** **********
                                                      ****** **********

alignment position 1551...... 1561...... 1571...... 1581...... 1591......
hNDUFV3      1372 ACCTGTGAAA AGgag----- ---------- --AAGAAGGG CTTTCTTTTC
mNDUFV3       804 GCTTGTCAAG AGCTGTCATT GAACtGGCCC CAAAGCAGGA AATGCTCACA
rNDUFV3       832 GCTTGTCAAG AGCTGTCATT GAACcGGCCC CAAAGTAGGA AATGCTCAAA
                  ********** ********** **** ***** ********** **********
                  ********** ********** **** ***** ********** **********
                  ********** ********** **** ***** ********** **********
                  ********** ********** **** ***** ********** **********
                  ********** ********** **** ***** ********** **********
                  ********** ********** ****
                  ********** ********** ****

alignment position 1601...... 1611...... 1621...... 1631...... 1641......
```

```
hNDUFV3            1405 GGCAAATAAA AAGCTAAGGC CAaggccggg cgcggacact ttgggatttt
mNDUFV3             854 GCAATATAAA ATACAggTGA CAGAGGGCTC CCGTCGTCAA ATGGGCACTg
rNDUFV3             882 GCAAATAAAA ATACAGATGA CAGAGGGCCG CCATCGTCAG ATGAACACTa
                        ********** ********** ********** ********** *********
                        ********** *****  *** ********** ********** *********
                        ********** *****
                        ********** *****
                        ********** *****

alignment position      1651...... 1661...... 1671...... 1681...... 1691......
hNDUFV3            1455 gcctgt---- ---------- ---------- ---------- ----------
mNDUFV3             904 tttaaTTATT CCTTCAGGTA TAttgCTCCC AGCGATTTTT Ttccagcgtg
rNDUFV3             932 g----TTACT CCTTCAGGTA TAa--CTTCC AGCGATTTTT T---------
                             ***** ********** **   ***** ********** *

alignment position      1701...... 1711...... 1721...... 1731...... 1741......
hNDUFV3            1461 ---------- ---------- ---------- ---------- ----------
mNDUFV3             954 tatcggtaca cataatttgt acacacacgg agaatcatca gtataaatag
rNDUFV3             967 ---------- ---------- ---------- ---------- ----------

alignment position      1751...... 1761...... 1771...... 1781...... 1791......
hNDUFV3            1461 ---------- ---------- ---------- ---------- ----------
mNDUFV3            1004 aagttttccg gttcttttgc tccacccccc aaccccctcc ctagcatgaa
rNDUFV3             967 ---------- ---------- ---------- ---------- ----------

alignment position      1801...... 1811...... 1821...... 1831...... 1841......
hNDUFV3            1461 ---------- ---------- ---------- ---------- ----------
mNDUFV3            1054 gcatcctagg atctaacaca taccttagcg gttgccggct gaattacaat
rNDUFV3             967 ---------- ---------- ---------- ---------- ----------

alignment position      1851...... 1861...... 1871...... 1881...... 1891......
hNDUFV3            1461 ---------- ---------- ---------- ---------- ----------
mNDUFV3            1104 aagattcttc tcaaattgta acacttctga agcaattatc tacatcctat
rNDUFV3             967 ---------- ---------- ---------- ---------- ----------

alignment position      1901...... 1911...... 1921...... 1931...... 1941......
hNDUFV3            1461 ---------- ---------- ---------- ---------- ----------
mNDUFV3            1154 ttactagtag atagtgtttc tgcaacttta ctaactgtag ggagataatt
rNDUFV3             967 ---------- ---------- ---------- ---------- ----------

alignment position      1951...... 1961...... 1971...... 1981...... 1991......
hNDUFV3            1461 ---------- AATCCCAGTA CTTTGGAAGG ccgagccagg cggatcgctt
mNDUFV3            1204 ttgcaaaaag AAACATAGTA TTTTGTAAAG taacaaaata gccagt----
rNDUFV3             967 ---------- ---------- ---------- ---------- ----------
                                   ********** **********

alignment position      2001...... 2011...... 2021...... 2031...... 2041......
hNDUFV3            1501 gagttcagga gttcgAGACT AGCCTGGCCA ACATGTCGAA ACCCCgt---
mNDUFV3            1250 ---------- -----AGAGT AAACTGGCTG CCCTGTATTA AGACCcagga
rNDUFV3             967 ---------- ---------- ---------- ---------- ----------
                                        ***** ********** ********** *****

alignment position      2051...... 2061...... 2071...... 2081...... 2091......
hNDUFV3            1548 ---------- ---------- ---------- ---------- ----------
mNDUFV3            1285 gtcttgggct gagagccaac tgggccttgc atccaggatc caggcctgag
rNDUFV3             967 ---------- ---------- ---------- ---------- ----------

alignment position      2101...... 2111...... 2121...... 2131...... 2141......
hNDUFV3            1548 ---------- ---------- ---------- ---------- ----------
mNDUFV3            1335 tgttacagcc ggcatgatgg ctgccatcca gaggccagca cttccctccc
rNDUFV3             967 ---------- ---------- ---------- ---------- ----------
```

```
alignment position 2151.......  2161.......  2171.......  2181.......  2191.......
hNDUFV3    1548 ----------  ----------  ----------  ----------  ----------
mNDUFV3    1385 ccctccccca  tgctaacaca  ggctcccccc  ctccccccaa  cacaggtctg
rNDUFV3     967 ----------  ----------  ----------  ----------  ----------

alignment position 2201.......  2211.......  2221.......  2231...
hNDUFV3    1548 ----------  ----------  ----------  --------
mNDUFV3    1435 tct aactgag  gctttgcagg  acacgtgaca  gcaagag
rNDUFV3     967 ----------  ----------  ----------  --------
```

Pairwise similarities:

For each pairwise alignment, the similarity (relative to the maximum similarity) and the number of identical nucleic acids (in % of shorter sequence) is given. Maximum values are underlined.

	mNDUFV3 (1471 bp)	rNDUFV3 (966 bp)
hNDUFV3 (1547 bp)	0.063<br>14 %	0.074<br>24 %
mNDUFV3 (1471 bp)		1.000<br>80 %

Please note that the **similarity value 1.000** marks only the two **most similar** sequences, it does not necessarily mean that these sequences are identical.

Extract alignment region	
**Positions in alignment**	from: to:
**Output file**	dialign.seq

Extract aligned sequences

For comments, questions or bug reports, please contact support@genomatix.de

© Genomatix Software GmbH 1998-2005 - All rights reserved. License Agreement GEMS Launcher 4.1.1
