# Supplementary material for: Transcriptional co-expression and co-regulation of genes coding for components of the oxidative phosphorylation system
Source: BMC Genomics. 2008 Jan 14;9:18. doi: 10.1186/1471-2164-9-18 (PMC2268925; doi:10.1186/1471-2164-9-18)
Supplement: Additional file 5 — Phylogenetic footprinting of Complex II core promoters. Core Promoters of Complex II subunits/related genes identified by phylogenetic footprinting. The conserved regions are shown in color codes, which correspond to specific Transcription Factor Binding Sites (TFBS). [file 1471-2164-9-18-S5.pdf]

This Supplementary Material shows the Core Promoters of Complex II subunits/related genes identified by phylogenetic footprinting. The conserved regions are shown in color codes, which correspond to specific Transcription Factor Binding Sites (TFBS).

The description of the complete description of TFBS can be found in additional file 3 (in the “Matrix Family Library” Excell sheet).

Genomatix-Logo Logout Personal Messages GEMS-Logo
Logout Personal Messages

Genomatix_MainGEMSElDoradoGene2PromoterBiblioSphere
Frequently_asked_questionsYour_ResultsYour_SequencesYour_ProtocolHelp

**GEMS Launcher Task: *DiAlign TF*: Multiple alignment plus TF sites**
**working on promoters.seq (3 seq.)**

[Alignment] [Pairwise_similarities]

**DiAlign professional TF Release 3.1 December 2004** **Wed Jun 8 21:31:13 2005**

# Solution parameters:

| | |
|---|---|
| **Sequence file:** | promoters.seq (3 seq.) |
| **Type of sequences:** | nucleotide sequences |
| **Threshold T:** | 0.00 |
| **'*' signs below alignment denote:** | diagonal similarity (max. similarity is represented by 10 '*' signs) |
| **Alignment output:** | complete alignment is shown<br># nucleic acids per line: 50 |
| **TF output:** | common TF matches located in aligned regions (common to 3 (100.0 %) sequences) |
| **Family matches:** | yes |
| **MatInspector library:** | Matrix Family Library Version 5.0 (February 2005) |
| **Selected groups** (core/matrix sim): | • ALL user_defined.lib (0.75/Optimized)<br>• ALL vertebrates.lib (0.75/Optimized) |

# Aligned Sequences:

| No. | Sequence Name | Sequence Description | Length |
|---|---|---|---|
| 1 | P878032 | sym=SDHA\|loc=Loc6389\|taxid=9606\|spec=Homo sapiens\|chr=5\|ctg=NT_006576\|str=(+)\|start=207947\|end=208947\|len=1001\| tss=501\| comm=succinate dehydrogenase complex, subunit A, flavoprotein (Fp); (NM_004168/501/silver;) | 1001 bp |
| 2 | P878035 | sym=Sdha\|loc=Loc66945\|taxid=10090\|spec=Mus musculus\|chr=13\|ctg=NT_078713\|str=(-)\|start=5811894\|end=5812912\|len=1019\| tss=501,502,514,519\| comm=succinate dehydrogenase complex, subunit A, flavoprotein (Fp); (AK029520/510/gold;AK010561/522/gold;AK050475/522/gold; AK049590/522/gold;AK075990/527/gold;XM_127445/509/bronze;) | 1019 bp |
| 3 | P878037 | sym=Sdha\|loc=Loc157074\|taxid=10116\|spec=Rattus norvegicus\|chr=1\|ctg=NW_047549\|str=(+)\|start=6979031\|end=6980031\| len=1001\|tss=501\| comm=succinate dehydrogenase complex, subunit A, flavoprotein (Fp); (NM_130428/501/bronze;) | 1001 bp |

# Alignment (DiAlign format):

Please note that only **upper-case** letters are considered to be **aligned**. For more information, please have a look at the user guide.

V$GLIF V$NRF1 U$NRF1 V$EGRF V$P53F V$INSM V$EREF U$EREF V$RORA

```
alignment position 1.........  11.........  21.........  31.........  41.........
P878032    1 cacttaagt ttgttacat gtgacctcc- ---------- ---ACAAACA
P878035    1 ---------- ---------C CTCCAGCTTC CCAGCTGCTG -TAACGAAGA
P878037    1 aggatgacaa caggccggtC CCCGAGCTTT CCAGCAGCTG tTAACGAAGA
```

```
                                            * ********** **********  *********
                                                                     *********
                                                                     *********
alignment position 51........ 61........ 71........ 81........ 91........
P878032         37 CACACAATTA CAAAATAGTT Tagttaat-- ---------- ----------
P878035         31 CGAAAGCTTT GGATATAGTT TCTAGTTCAG TATCCTGGCT TTTACGTAGC
P878037         51 TGAAAGCGAT GGATATAcTT TCTAGTTCAG TATTCTGGAT TCTGCGTAGC
                   ********** ********** ********** ********** **********
                   ********** *******  ** ********** ********** **********
                   ********** *******  ** ********** ********** **********
                                      ** ********** ********** **********
                                      ** ********** ********** **********
alignment position 101....... 111....... 121....... 131....... 141.......
P878032         65 ---------- ---------- ---------- --CAAGGGAT gtataataaa
P878035         81 CCAGTGCagc tAGCTGCTCC TTGTCCAGCC ACCAAGacAT CATTCCTACC
P878037        101 TCAGTGC--- -AGCTGCTCC TTGTCCAGCC ACCAAGGGAT CATTCCTACC
                   *******     ********* ********** ********** **********
                   *******     ********* ********** ******  ** **********
                   *******     ********* ********** ******  ** **********
                   *******     ********* ********** ******
                   *******     ********* ********** ******
alignment position 151....... 161....... 171....... 181....... 191.......
P878032         83 caagaggtcg gaggccccgg gccgctcccg cccacctccg cggacgagcg
P878035        131 TACTCTAGAA GTCCAGGGCA GTAtgta--- ---------- ----------
P878037        147 CACAACAGAA GTTCAGGGCG GTAg------ ---------- ----------
                   ********** ********** ***
                   ********** ********** ***
                   ********** ********** ***
alignment position 201....... 211....... 221....... 231....... 241.......
P878032        133 CCCCCCTCCG ACCCCATTCC CTGCGGTCTG GGCGTTCAgg cccttcggtc
P878035        158 CCCCCCCCCC CCCCCATTAG TTGAGATTCa AGCGCCCAAC AAAACGGGCT
P878037        171 --------CC CGCCCATTTC TTGAGATTCT AGCGCCCAAC AAAATGGGTT
                   ********** ********** ********** ********** **********
                           ** *******             ********** **********
                                                  ********** **********
                                                  ********** **********
                                                  ********** **********
                                                  ********** **********
alignment position 251....... 261....... 271....... 281....... 291.......
P878032        183 tgggcgatcc cgg------- ---------- ---------- ----------
P878035        208 CTTATGGAGC TTGCGATCtc cgcccc-AGG ACCCTACTTC CATCTCTCCA
P878037        213 CTTATGGAGC TTGCGTTCgc ttccactAGG ACCCTACTTT CATCTGTCCG
                   ********** ********         *** ********** **********
                   ********** ********         *** ********** **********
                   ********** ********         *** ********** **********
                   ********** ********
                   ********** ********
                   ********** ********
alignment position 301....... 311....... 321....... 331....... 341.......
P878032        196 ---------- ---------- ---------- -AGA---ACC ACCCAcgggg
P878035        257 GATCATCCGA CCCtctcTGT TTTGGCAAAC AAGA---ACC ACCCGAGAAG
P878037        263 GATCCTCGGG CCCggagTGT CTTGGCAAAC AAGAaaaACC ACCCAAGAAG
                   ********** ***    *** ********** ****   *** **********
                   ********** ***    *** ********** ****   *** **********
                   ********** ***                          *** **********
                                                           *** **********
alignment position 351....... 361....... 371....... 381....... 391.......
P878032        212 --CTTTAAAA ATGTTGGTGC CCACCACCTC CCCGGAACAG GGC-------
P878035        304 CACTTTAAAG ACATTGGCAC CTCACTTACC CCTGGAGCAG AGCCCATCAG
P878037        313 CACTTTAAGG ACATTGGTAC CTCACTTACC CCTGGAGCAA AGCCCATCAG
```

```
                        ********** ********** ********** ********** **********
                        ********** ******   ** ********** ********** **********
                        ********** ******   ** ********** ********** **********
                        ********** ******   ** ********** ********** **********
                           ******* ******   ** ********** ********** **********
                                            ** ********** ********** **********

alignment position      401....... 411....... 421....... 431....... 441.......
P878032          253    --------CC GCTCTACCTC GGTCGGGgag cgcgggacct cagcgttccc
P878035          354    CgttGCTGCC ACCCTACCTC AGACGTGTTG ---------- ----------
P878037          363    CtaaGCTGAC AGCCTATCTC ACAGGTAATG ---------- ----------
                        *   ****** ********** **********
                        *   ****** ********** **********
                        *   ****** ********** **********
                        *   ****** ********** **********
                        *
                        *

alignment position      451....... 461....... 471....... 481....... 491.......
P878032          295    ttaacgccaC CGTCCGCGGG TCCGCTTTGC GCAGGCGCGG CGCCCCcact
P878035          384    ---------C CACCGCCGCG TTTaCAGGGC GCAGGCGCAC CACCACTCAC
P878037          393    ---------C CACCGCCGCG TTTGCAGAGC GCAGGCGCAC CACCACTCAG
                                 * ********** ********** ********** **********
                                 * ********** ***  ***** ********** **********
                                 * ********** ***  ***** ********** **********
                                 * ********** ***  ***** ********** **********
                                                   ***** ********** **********
                                                   ***** ********** **********
                                                   ***** ********** **********

alignment position      501....... 511....... 521....... 531....... 541.......
P878032          345    cagtacccgc tccgggcgtg gcatggtgcg caggcgcgat gtcccccact
P878035          425    GCGAGCCAGG AGG------- ---------- ---------- ----------
P878037          434    GCGAGCCAGG AGGa------ ---------- ---------- ----------
                        ********** ***
                        ********** ***
                        ********** ***
                        ********** ***
                        ********** ***
                        ********** ***
                        ********** ***

alignment position      551....... 561....... 571....... 581....... 591.......
P878032          395    gcagccccgc tcgactccGG CGTGGTGCGC AGGCGCGGTA TCccccctcc
P878035          438    ---------- --------GG CGTGTTGCGC AGGCGCAGAA TCTTCCAAGC
P878037          448    ---------- ---------G CGTGTTGCGC AGGCGCAGAG CCTTCCAAGC
                                          ** ********** ********** **********
                                          ** ********** ********** **********
                                           * ********** ********** **********
                                           * ********** ********** **********
                                           * ********** ********** **********
                                           * ********** ********** **
                                           * ********** ********** **
                                           * ********** ******

alignment position      601....... 611....... 621....... 631....... 641.......
P878032          445    cccgccagct cgaccccggt gtGGTGCGCA GGCGCAGTCT G-CGCAGGGA
P878035          470    CCGACACCcG CGCGACTC-- --GGTGCGCA AGCGCAGTCT GTCGCTGGGG
P878037          479    GCCACACCaG CGCGGCTC-- --GGTGCGCA AGCGCAGTCT ATCGCTGAGG
                        ********  * ********    ******** ********** **********
                        ********  * ********    ******** ********** **********
                        ********  * ********    ******** ********** **********
                        ********  * ********    ******** ********** **********
                        ********  * ********    ******** ********** **********
                                  * ********    ******** ********** **********
                                                ******** ********** *
                                                ******** ********** *
                                                ******** **********

alignment position      651....... 661....... 671....... 681....... 691.......
```

```
P878032     494 CTGGCGGGAc tgcgcggcgg caacaGCAGA CATGTCGGGG GTCCGGGGCC
P878035     516 CTTGCGGGAA G--------- -----GCAGA CATGGCCGGG GTTGGCGCAG
P878037     525 CTTGCGGGAA G--------- -----GCAAA CATGGCCGGG GTTGGCGCAG
                ********** *          ***** ********** **********
                ********** *          ***** ********** **********
                ********** *          ***** ********** **********
                ********** *          ***** ********** **********
                ********** *          ***** ********** **********
                ********** *          ***** ********** **********
                   ******* *          ***** ********** **********
                   ******* *          ***** ********** **********
                                      ***** ********** **********

alignment position 701.......  711.......  721.......  731.......  741.......
P878032     544 TGTCGCGGCT GCTgagcgct CGGCGCCTGG CGCTGGCCAA GGCGGTGAGT
P878035     552 TTTCGAGGCT TCTTCGCGGG CGGCGCTTGG CGTTAACTGG GGCGGTGAGT
P878037     561 TTTCGAGACT TCTTCGCGGG CGGCGCTTGG CTCTAGCTGG GGCGGTGAGT
                ********** ********** ********** ********** **********
                ********** ********** ********** ********** **********
                *******  ** ********** ********** ********** **********
                *******  ** ********** ********** ********** **********
                *******  ** ********** ********** ********** **********
                *******  ** ********** ********** ********** **********
                *******  ** ***        ********** ********** **********
                *******  **            ********** ********** **********
                *******                ********** ********** ********
                                       ********** ********** ********

alignment position 751.......  761.......  771.......  781.......  791.......
P878032     594 CCGTGCCGCG gaccggggcg gggcaggcgg gggccgaggc GGCGGTAGGA
P878035     602 TAGTGCCACG AAATGGCA-- ---------- ---------- GGCGCTAGGA
P878037     611 TAGTGCCACG GTATGGCG-- ---------- ---------- GGCACCAGGA
                ********** ******* **                       **********
                ********** ******* **                       **********
                ********** ******* **                       **********
                **********
                **********
                **********
                **********
                **********

alignment position 801.......  811.......  821.......  831.......  841.......
P878032     644 Gcgggacggt ccccagcggg tccgagcgga gcgggcgccg ggtccctgcg
P878035     630 GTGCGcca-- -----CGATT CCACCCGAGT ---------- ----------
P878037     639 ATGCGtctaa tcctgCGATC CCACCCGAGT ---------- ----------
                *****           ***** **********
                *****           ***** **********
                *****           ***** **********
                                ***** **********

alignment position 851.......  861.......  871.......  881.......  891.......
P878032     694 ccctcTGTCC CGGGATCGGG AAGGGGCTGA GAGAGccctg ggccggtgcg
P878035     653 -----TGGCC TGAGTCTGGG AAGGTGCAGT GGGCCGAAGC C---------
P878037     669 -----TGAAT TGGATCTGGG AAGGTGCAGC GGGCGGAAGC G---------
                     ***** ********** ********** ********** *
                     ***** ********** *********  ********** *
                     ***** ********** *********  ********** *
                     ***** ********** *********  ********** *

alignment position 901.......  911.......  921.......  931.......  941.......
P878032     744 aggggaagcc gcggggcgga ctcggggacc cggggagctc ggtccttagt
P878035     689 ---------- ---------- ---------- ---------- ----------
P878037     705 ---------- ---------- ---------- ---------- ----------

alignment position 951.......  961.......  971.......  981.......  991.......
P878032     794 agataGTCCG CGTCCGGGTG AAGGTCACAA CCCCGCGGGC ttgctgggcg
P878035     689 -----GACTG CTTTCCGGCG AAGGTCACGA CCCCGAGGTC GCGGGCGTGG
P878037     705 -----GACAC CGTTCCGGCG AAGGTCATGA CCCCGAGGTC GCGGGCATGG
```

```
                        ***** ********** ********** ********** **********
                        ***** ********** ********** ********** **********
                        ***** ********** ********** ********** **********
                        ***** ********** ********** ********** **********
                        ***** ********** ********** ********** **********
                        ***** ********** ********** ********** **********
                              ********** ********** ********** **********
                                                        ******
                                                        ***

alignment position 1001...... 1011...... 1021...... 1031...... 1041......
P878032        844 tcc----CCT CCGCCGCCTT GGTCcgggcc tggggtcctg ggaccccgcg
P878035        734 AGTCTGGCCT TCCCCGTCTT GGTC-TAGCG AAGTTCTCCA AGGTTAAAGG
P878037        750 AGTCTGGCCT TCCCTGTCTT GGTCtTAGCA AAGTTCTCCA AGGTTAACGG
                   ********** ********** **** ***** ********** **********
                   ********** ********** **** ***** ********** **********
                   ********** ********** **** ***** ********** **********
                   ********** ********** **** ***** ********** **********
                   ********** ********** **** ***** ********** **********
                   ********** ********** **** ***** ********** **********
                   ********** ********** ****

alignment position 1051...... 1061...... 1071...... 1081...... 1091......
P878032        890 ggctgaggta gcccctcgcc tcagtgCCTG GCAGGTGGAC TCGGGGAGGA
P878035        783 GAGGGA---- ---------- ------CCGG CCTGTTGCAT TCTGGATGCG
P878037        800 GAGGGA---- ---------- ------CCAG CCTGTgGCAC TCTGGATGTG
                   ******                      **** ********** **********
                   ******                      **** ***** **** **********
                   ******                      **** *****
                   ******                      **** *****
                   ******                      **** *****
                   ******                      **** *****

alignment position 1101...... 1111...... 1121...... 1131...... 1141......
P878032        940 GTCGTGTCTG Cccaaggtca cccgggcgga tagcggccgg tggccgccct
P878035        813 GACGGGTCTG CGAgtaATCC GTTGGTAGTC TGGGTGTCGA CACTAAgact
P878037        830 GGCAGATCCG CGA---ATCC GTTGGTAGCC TGGGTGTGGA CACTAA----
                   ********** ***   **** ********** ********** ******
                   ********** ***   **** ********** ********** ******
                                    **** ********** ********** ******
                                    **** ********** ********** ******

alignment position 1151...... 1161...... 1171...... 1181...... 1191......
P878032        990 ggctgggctg gg-------- ---------- ---------- ----------
P878035        863 tataagtgat gagcttacaG GTTTTGTGTA GTGGCCTTAG CTACTGTTTT
P878037        873 ---------- ---------G GTTTTGTGTA GTAGTCTTAG CTACTGTTTT
                                       * ********** ********** **********
                                       * ********** ********** **********
                                       * ********** ********** **********
                                       * ********** ********** **********
                                       * ********** ********** **********
                                       * ********** ********** **********
                                       * ********** ********** **********

alignment position 1201...... 1211...... 1221...... 1231...... 1241......
P878032       1002 ---------- ---------- ---------- ---------- ----------
P878035        913 TAAATATCAa AGAGATATTT GAAGATACCA GGTGAGACTT CTGCACcGTG
P878037        904 TAAATATCAg AGAGAGATTT GAAAATATCA GGTGAGACTT CTGCACtGTG
                   *********  ********** ********** ********** ****** ***
                   *********  ********** ********** ********** ****** ***
                   *********  ********** ********** ********** ******
                   *********  ********** ********** ********** ******
                   *********  ********** ********** ********** ******
                   *********
                   *********

alignment position 1251...... 1261...... 1271...... 1281...... 1291......
P878032       1002 ---------- ---------- ---------- ---------- ----------
P878035        963 TTCATCCAGT CTTGCGACTT CT-TTTTGCA TTTGAAGAGG TACACAGtga
P878037        954 TTCATCCAAT TTTGCGGCTT CTgTTTTGCA TTGGAAGAGA GTCACAGa--
```

```
                    ********** ****** **** **  * * * * * * ********** * ******* 
                    ********** ****** **** **
alignment position  1301....
P878032       1002  --------
P878035       1012  ctcagggt
P878037       1002  --------
```

---

## Pairwise similarities:

For each pairwise alignment, the similarity (relative to the maximum similarity) and the number of identical nucleic acids (in % of shorter sequence) is given. Maximum values are underlined.

| | P878035 (1019 bp) | P878037 (1001 bp) |
|---|---|---|
| **P878032 (1001 bp)** | 0.086<br>29 % | 0.075<br>28 % |
| **P878035 (1019 bp)** | | <u>1.000</u><br><u>83 %</u> |

Please note that the **similarity value 1.000** marks only the two **most similar** sequences, it does not necessarily mean that these sequences are identical.

---

| **Extract alignment region** | |
|---|---|
| **Positions in alignment** | from: to: |
| **Output file** | dialign.seq |

Extract aligned sequences

---

For comments, questions or bug reports, please contact support@genomatix.de

---

© Genomatix Software GmbH 1998-2005 - All rights reserved.

License Agreement

GEMS Launcher 4.1.1

Genomatix-Logo Logout Personal Messages GEMS-Logo
Logout Personal Messages

Genomatix_MainGEMSElDoradoGene2PromoterBiblioSphere
Frequently_asked_questionsYour_ResultsYour_SequencesYour_ProtocolHelp

**GEMS Launcher Task: *DiAlign TF*: Multiple alignment plus TF sites**
**working on promoters_2.seq (3 seq.)**

[Alignment] [Pairwise_similarities]

**DiAlign professional TF Release 3.1 December 2004** **Wed Jun 8 21:35:28 2005**

# Solution parameters:

| | |
|---|---|
| **Sequence file:** | promoters_2.seq (3 seq.) |
| **Type of sequences:** | nucleotide sequences |
| **Threshold T:** | 0.00 |
| **'*' signs below alignment denote:** | diagonal similarity (max. similarity is represented by 10 '*' signs) |
| **Alignment output:** | complete alignment is shown<br># nucleic acids per line: 50 |
| **TF output:** | common TF matches located in aligned regions (common to 3 (100.0 %) sequences) |
| **Family matches:** | yes |
| **MatInspector library:** | Matrix Family Library Version 5.0 (February 2005) |
| **Selected groups** (core/matrix sim): | • ALL user_defined.lib (0.75/Optimized)<br>• ALL vertebrates.lib (0.75/Optimized) |

# Aligned Sequences:

| No. | Sequence Name | Sequence Description | Length |
|---|---|---|---|
| 1 | P876195 | sym=SDHB\|loc=Loc6390\|taxid=9606\|spec=Homo sapiens\|chr=1\|ctg=NT_004610\|str=(-)\|start=204488\|end=205488\|len=1001\| tss=501\| comm=succinate dehydrogenase complex, subunit B, iron sulfur (Ip); (NM_003000/501/bronze;) | 1001 bp |
| 2 | P876196 | sym=Sdhb\|loc=Loc67680\|taxid=10090\|spec=Mus musculus\|chr=4\|ctg=NT_039267\|str=(+)\|start=8395078\|end=8396196\|len=1119\| tss=501,608,609,619\|comm=succinate dehydrogenase complex, subunit B, iron sulfur (Ip); (AK003052/608/gold;AK009660/609/gold;AK003533/619/gold; NM_023374/501/bronze;) | 1119 bp |
| 3 | P876197 | sym=na\|loc=Loc298596\|taxid=10116\|spec=Rattus norvegicus\|chr=5\|ctg=NW_047726\|str=(+)\|start=3781027\|end=3782027\| len=1001\|tss=501\| comm=similar to succinate dehydrogenase Ip subunit; (XM_216558/533/bronze;) | 1001 bp |

# Alignment (DiAlign format):

Please note that only **upper-case** letters are considered to be **aligned**. For more information, please have a look at the user guide.

V$ZBPF V$SP1F V$NRF1 U$NRF1 V$EBOX V$P53F V$ETSF U$NRF2 V$CDEF V$EKLF V$MAZF V$CREB
V$E4FF V$YY1F V$MYOD V$EREF U$EREF V$RORA

```
alignment
position          1 . . . . . . . . .   1 1 . . . . . . . .   2 1 . . . . . . . .   3 1 . . . . . . . .   4 1 . . . . . . . .

P876195       1   g t  a a t t c c a g   c a c t t  t g g g a   g g c  c a  a g g t  g   g g c  g g a t c a c   g a g g t  c a g g a
P876196       1   c t  g g c a c c a g   c c a c a t a c t  a   g g t  a c c c t t t     c t g g c t  g c t t    t g g t t  t t g g t
P876197       1   g c  - - - - - - - -   - - - - - - - - - -   - - - - - - - - - -     - - - - - - - - - -     - - - - - - - - - -
```

```
alignment
position          51........ 61........ 71........ 81........ 91........

P876195      51 gttcaagacc agtctggcca agatgatgaa accccgtctc tactaaaaat
P876196      51 tttgtgtgtt t--------- ---------- ---------- ----------
P876197       3 ---------- ---------- ---------- ---------- ----------

alignment
position          101....... 111....... 121....... 131....... 141.......

P876195     101 acaaaaaatt agccgggtgt gatggtgtgc gcctgtaatc ccagctactc
P876196      62 ---------- ---------- ---------- ---------- ----------
P876197       3 ---------- ---------- ---------- ---------- ----------

alignment
position          151....... 161....... 171....... 181....... 191.......

P876195     151 gggaggcTGA GGCAGGagaa tcgcgtgaac ccgggaggcg gaggttgcag
P876196      62 -------TGA GGCAGGgtCT CAAGTATCCC AGAT------ ----------
P876197       3 ---------- --------CT CAAGTATCCC AGAT------ ----------
                       *** ******   ** ********** ****
                                    ** ********** ****
                                    ** ********** ****

alignment
position          201....... 211....... 221....... 231....... 241.......

P876195     201 tgagccaaga tcgcgccatt gcactccagc ccgggaaaca gtgcgAGACT
P876196      89 ---------- ---------- ---------- ---------- -----GGACT
P876197      19 ---------- ---------- ---------- ---------- -----AGCCT
                                                                 *****
                                                                 *****
                                                                 *****

alignment
position          251....... 261....... 271....... 281....... 291.......

P876195     251 CCATCTCAaa caaacaaaca aaa------- ---------- ----------
P876196      94 CGACCTCATT AAGTAGCggA GGGTGAGCTT GGTTCTCCTA CTGTCTCCAT
P876197      24 CCACCTCACT AAGTAGCcaA GGATGAGCTT GATTCTCCTA CTGCCTCCAC
                ********** *******  * ********** ********** **********
                ********** *******  * ********** ********** **********
                ********** *******

alignment
position          301....... 311....... 321....... 331....... 341.......

P876195     274 ---------- -----ATTCC AGGAAT---- ---------- ----------
P876196     144 ATGCCg---- ---------- -GGCATGCAA CTTCTGTACc gacg------
P876197      74 ATGCCaattt ctgggATTCC AGGCATGCAA CTTACGTACa tatttatgaa
                *****           ***** ********** *********
                *****

alignment
position          351....... 361....... 371....... 381....... 391.......

P876195     285 ---------- ---------- ---------- ---------- ----------
P876196     173 ---------- ---------- -TGACGTTCA TCTTTtgatc acttcagtca
P876197     124 taaatgaatg agcgacacct tTGACATTCA TTTTTgtgac cgcctcaatc
                                       ********* *****

alignment
position          401....... 411....... 421....... 431....... 441.......

P876195     285 ---------- ---------- ---------- ---------- ----------
P876196     202 cttaa-CAAG TGTCCCAATC AGGTAGGGAC TGACAGGTTC TCTAGattta
P876197     174 gctcgcCAAC TGTCTCGCTC AGAGAGGGAC TGACGGGTCC TCTAG-----
                      **** ********** ********** ********** *****
                      **** ********** ********** ********** *****

alignment
position          451....... 461....... 471....... 481....... 491.......
```

```
P876195     285 ---------- ---------- ---------- ---------- ----------
P876196     251 gCACAAAGCT GACCAGACAA GAGTCACAGG TGATTGACAG ACACCACTGC
P876197     219 -CACAAAACT GATCAGACAA GAGTCACAGG TGATTGACAG ACACGACTGC
                 ********* ********** ********** ********** **********
                                    * ********** ********** **********
                                    * ********** ********** **********
                                    * ********** ********** **********

alignment
position        501....... 511....... 521....... 531....... 541.......

P876195     285 ------AAA- GAGGTTGAAA TCGGGGCAGG GACCCGGATA GGATGGCTCC
P876196     301 AAGTAGAGAa GGGTTAAAAA TCACAGCCAC CAGCAGAATA GGAAAGCCCC
P876197     268 AAGTAGAGA- GGGATTAAAA TCACAGCCTT CAGCAAACTA GGAAAGACCC
                *********  ********** ********** ********** **********
                *********  ********** ********** ********** **********
                *********
                *********

alignment
position        551....... 561....... 571....... 581....... 591.......

P876195     328 G-CCCcatct aaGTCTTAGC CCCACCCCct gaaagtcgc- ---------C
P876196     351 G-CCCTCCTG --GCCTCTGC CCCGCCCCTC CACGCTACCC TTCC-CTCTC
P876197     317 gcCCCTCCTG --GCCTTTGC CCCACCCCTC CAGGCTACCT TCCCtCTTTC
                *  ********   ******** ********** ********** ****  *****
                   ********   ******** ********** ********** ****      *
                   ********   ******** ********** ********** ****

alignment
position        601....... 611....... 621....... 631....... 641.......

P876195     367 CTGCCTCTCA GACTCCtccc ctttctgaga aggtcacggg GGAAGCCAAA
P876196     397 CCGCCCCTCA GACTCCAGAG GTCACCTTAG GAACATA--- GGAAGCCCAG
P876197     365 CCGCTGCTCC GACTCCAGAG GTCACCTTAG GAACATA--- GGAAGCCCAG
                ********** ********** ********** *******    **********
                ********** ******        ******* *******    **********
                                         ******* *******    **********
                                         ******* *******    **********
                                                            **********
                                                            **********

alignment
position        651....... 661....... 671....... 681....... 691.......

P876195     417 TGGGCATGCG CCgCTACTGC GCTATTGCGC ACGCTCGCTG TGCTTGCCCc
P876196     444 TGGGCATGCG CC-CTACGGG CCGCTGCTGC ACACACGCAC TTCCTGTACA
P876197     412 TGGGCATGCG CC-CTACTGG CCGCTACGGC ACACTCGCAC TTCCTGTCCG
                ********** **  ****** ********** ********** **********
                ********** **  ****  ** ******** ********** **********
                ********** **  ****
                ********** **  ****
                ********** **
                ********** **

alignment
position        701....... 711....... 721....... 731....... 741.......

P876195     467 gccttccctc cgcccACCCG G-GAAACCGG AAGCCGCCTC CCACTTGGTT
P876196     493 TTG------- -----GCTCG gaGAAACCGG AAGCGGCCTT CCACTCGTTG
P876197     461 TTT------- -----ACTCG G-GATACCGG AAGCTGCCTT CCACTCGTTG
                ***             ***** *  ******** ********** **********
                ***             ***** *  ******** ********** **********
                                *****    ******** ********** **********
                                         ******** ********** **********
                                         ******** ********** **********

alignment
position        751....... 761....... 771....... 781....... 791.......

P876195     516 GCTCGTACGC GGctagtgGG TCCTCAGtgg atgta---GG CTGGGCGCCG
P876196     531 GCGCTTAGGC GGCTAGC-GG TCCTCAGGGT GAGAGGCCGG CTTCCCACCG
P876197     498 GCTCTTAGGC TGCTAGC-GG ACCTCAAGGG GAGAGGCCGG CTTCCCACCG
```

```
                  ********** ******* ** ********** ********** **********
                  ********** ******* ** ********** ********** **********
                  ********** **                         ***** **********
                  ** *                                  ***** **********
                  **                                       ** **********
                                                           ** **********

alignment
position          801....... 811....... 821....... 831....... 841.......

P876195      563  CGATGTTCGA CGGGACACCG GCGGagagcg acctcggggt TAAGGGGTGG
P876196      580  CGGTGTTCGA CGGGATGCCG GAGGGg---- ---------- -AAGGGGTGG
P876197      547  CGGTGTTCGA CGGGATGCCG GAGGG----- ---------- TAAGGGGTGG
                  ********** ********** *****                **********
                  ********** ********** *****                **********
                  ********** ********** *****                **********
                  ********** ********** *****                **********
                  ********** ********** ****                  *********
                  ********** ********** ****                  *********
                                                              *********
                                                              *********
                                                              *********
                                                              *********

alignment
position          851....... 861....... 871....... 881....... 891.......

P876195      613  GGCTGACGTC AGGAGCCAAG ATGGCGGCGG TGGTCGCCCT CTCCTTGAGG
P876196      615  GGCTGACGTC AGGAGCCAAA ATGGCGGCGa cGGTCGGGGT CTCCTTGAAG
P876197      582  GGCTGACGTC AGGAGCCAAA ATGGCGGCGG TGGTCGGGGT CTCCTTGAAG
                  ********** ********** ********** ********** **********
                  ********** ********** **********  ********* **********
                  ********** ********** **********  ********* **********
                  ********** ********** **********  ********* **********
                  ********** ********** *********
                  ********** ********** *********
                  ********** ********** *********
                  ********** ********** *********
                  ********** ********** *********
                  ********** ********** *********

alignment
position          901....... 911....... 921....... 931....... 941.......

P876195      663  CGCCGGTTGC CGGCCACaac cCTTGGCGGA GCCTGCCTGC AGGTGAGTCC
P876196      665  CGCGGCTTCC CGGCTGCCGT TCTCGGCAGA GTCGGCCTGC AGGTGAGCCC
P876197      632  CGCGGCTTTT CGGCTACGGC TCTCGGCAGA GTCGGCCTGC AGGTGAGCCC
                  ********** ********** ********** ********** **********
                  ********** ********** ********** ********** **********
                  ********   ********** ********** ********** **********
                  ********   *******     ********* ********** **********
                             ****        ********* ********** **********
                                         ********* ********** **********

alignment
position          951....... 961....... 971....... 981....... 991.......

P876195      713  TGGAGCCtca gagagggaaa agtcaggaaa gc-CACAGAG ACTGCCTGGa
P876196      715  GGGAGCCGTG GTTCAGTGGG GAGCCGTGCC cGTCACCGAG GCTGCCTGGG
P876197      682  CGGAGCCGTA GTTCAGTGGG GAACCGTGCC gGTCACCGAG GCTGCCTGGG
                  ********** ********** **********  ********* **********
                  ********** ********** **********  ********* **********
                  *******                           ********* **********
                   ******
                   ******

alignment
position          1001...... 1011...... 1021...... 1031...... 1041......

P876195      762  gctgatGGGG CTGCGGAGAG ACTGAGGAAG CATAGGCAA GGCCTCAGgg
P876196      765  TAGA--GAGG CCGCGGAGAG GCGGAAGGAG CCCGGGTCCA GGCCTCGGGG
P876197      732  TGGA--GAGT CCGCGGAGAA GCGGAAAGAG CCCGGGTCCA GGCCTCAGGA
```

```
                    ****  **** ********** ********** ********** **********
                    ****  **** ********** ********** ********** **********
                    ****  **** *********  ********** ********** **********
                          **** *********  ********** ********** ********

alignment
position            1051...... 1061...... 1071...... 1081...... 1091......

P876195       812   agatggagga cctcctcccc ctggtgcagt gctttggttt ggcttcGAGC
P876196       813   GAGGACTTAa cccggcatcc tgctctT--- ---------- ------GAGC
P876197       780   GAGGACTTAc cagctccctg cccc--T---- --------- ------GAGC
                    *********              *                        ****
                    *********              *                        ****
                    *********              *                        ****

alignment
position            1101...... 1111...... 1121...... 1131...... 1141......

P876195       862   ATCCctgtca agttatcttt CTGCGCCTCG CAGCAGCTT- ---GTGAGTT
P876196       844   ATCCTTTCAG ACAGCCATT- CCGCGCTTCT CCatcaccgg agaGAGAGGC
P876197       809   ATCCTTTCAG ATAGCCAAC- CCGCGCTTCT CCCAAGCTG- ---GAGAGTC
                    ********** *********  ********** *********     *******
                    ********** *********  ********** **            *******
                    ********** *********  ********** **

alignment
position            1151...... 1161...... 1171...... 1181...... 1191......

P876195       908   AGGGCACGGg caatttttta cagctaaggg tgcggggggg ggggacGTTC
P876196       893   GCGGCCGGGA GGAGGTTGGA AgAGAGAACA GAAAGACAGA ------GGTC
P876197       854   GCGGCCGGGA GGAGGTTGGA AaAGATAACG GAAAGGCAGA ------GGTC
                    ********** ********** * ******** **********       ****
                    ********** ********** * ******** **********       ****

alignment
position            1201...... 1211...... 1221...... 1231...... 1241......

P876195       958   AGAAAGGG-G ACTTGAATTG CTCAAGGTCA CATGGCAAGC GAatg-----
P876196       937   TGGGAGGAgG ACAGGGAGAG GTCAAGGTCA TAGGATGAGC GACCTGTGGT
P876197       898   AGGGAGGA-C AAGCGAATTG GTCAAGGTCA TATGATGGGC GACCTGTTGT
                    ******** * ********** ********** ********** **********
                    ********           * ********** ********** **********
                                       * ********** ********** **********

alignment
position            1251...... 1261...... 1271...... 1281...... 1291......

P876195      1002   ---------- ---------- ---------- ---------- ----------
P876196       987   CGGGACCCAG AATCAATCCC TGCTAGTTGT ACTCCGCCAG GCTTGTGTga
P876197       947   TGGAACCCAG AATCAATCCC TGATATTTGC ACTCGACCAA CCTTGTGTaa
                    ********** ********** ********** ********** ********
                    ********** ********** ********** ********** ********
                    *********

alignment
position            1301...... 1311...... 1321...... 1331...... 1341......

P876195      1002   ---------- ---------- ---------- ---------- ----------
P876196      1037   aagggaggcc tgcggggtgt ctggggtggg acccgggagg ccaggtcgca
P876197       997   aaggg----- ---------- ---------- ---------- ----------

alignment
position            1351...... 1361...... 1371...... 1381

P876195      1002   ---------- ---------- ---------- ---
P876196      1087   cctccaggtt aggctggtct ggatcggtca ggt
P876197      1002   ---------- ---------- ---------- ---
```

---

## Pairwise similarities:

For each pairwise alignment, the similarity (relative to the maximum similarity) and the number of identical nucleic acids (in %

of shorter sequence) is given. Maximum values are underlined.

| | P876196 (1119 bp) | P876197 (1001 bp) |
|---|---|---|
| P876195 (1001 bp) | 0.200 34 % | 0.209 38 % |
| P876196 (1119 bp) | | 1.000 79 % |

Please note that the **similarity value 1.000** marks only the two **most similar** sequences, it does not necessarily mean that these sequences are identical.

| Extract alignment region | |
|---|---|
| **Positions in alignment** | from: to: |
| **Output file** | dialign.seq |

Extract aligned sequences

For comments, questions or bug reports, please contact support@genomatix.de

© Genomatix Software GmbH 1998-2005 - All rights reserved.

License Agreement

GEMS Launcher 4.1.1

| Genomatix-Logo | Logout<br>Logout | Personal<br>Personal | Messages<br>Messages | GEMS-Logo |
|---|---|---|---|---|

Genomatix_MainGEMSElDoradoGene2PromoterBiblioSphere
Frequently_asked_questionsYour_ResultsYour_SequencesYour_ProtocolHelp

**GEMS Launcher Task: *DiAlign TF*: Multiple alignment plus TF sites**
**working on promoters_2.seq (3 seq.)**

[Alignment] [Pairwise_similarities]

**DiAlign professional TF Release 3.1 December 2004** **Wed Jun 8 21:41:39 2005**

# Solution parameters:

| | |
|---|---|
| **Sequence file:** | promoters_2.seq (3 seq.) |
| **Type of sequences:** | nucleotide sequences |
| **Threshold T:** | 0.00 |
| **'*' signs below alignment denote:** | diagonal similarity (max. similarity is represented by 10 '*' signs) |
| **Alignment output:** | complete alignment is shown<br># nucleic acids per line: 50 |
| **TF output:** | common TF matches located in aligned regions (common to 3 (100.0 %) sequences) |
| **Family matches:** | yes |
| **MatInspector library:** | Matrix Family Library Version 5.0 (February 2005) |
| **Selected groups** (core/matrix sim): | • ALL user_defined.lib (0.75/Optimized)<br>• ALL vertebrates.lib (0.75/Optimized) |

# Aligned Sequences:

| No. | Sequence Name | Sequence Description | Length |
|---|---|---|---|
| 1 | P876246 | sym=SDHC\|loc=Loc6391\|taxid=9606\|spec=Homo sapiens\|chr=1\|ctg=NT_004487\|str=(+)\|start=11774025\|end=11775026\|len=1002\| tss=501,502\| comm=succinate dehydrogenase complex, subunit C, integral membrane protein, 15kDa; (AK131051/502/gold;NM_003001/501/bronze;) | 1002 bp |
| 2 | P876247 | sym=Sdhc\|loc=Loc66052\|taxid=10090\|spec=Mus musculus\|chr=1\|ctg=NT_039185\|str=(-)\|start=15061885\|end=15062886\|len=1002\| tss=501,502\| comm=succinate dehydrogenase complex, subunit C, integral membrane protein; (AK032458/501/gold;AK012818/501/gold;NM_025321/502/bronze;) | 1002 bp |
| 3 | P876248 | sym=na\|loc=Loc289217\|taxid=10116\|spec=Rattus norvegicus\|chr=13\|ctg=NW_047399\|str=(-)\|start=4499141\|end=4500141\| len=1001\|tss=501\| comm=similar to integral membrane protein CII-3; (XM_213936/501/bronze;) | 1001 bp |

# Alignment (DiAlign format):

Please note that only **upper-case** letters are considered to be **aligned**. For more information, please have a look at the user guide.

V$SP1F V$CREB V$ETSF V$YY1F V$EBOX V$E4FF V$MOKF

```
alignment position 1.........  11.........  21.........  31.........  41.........
P876246     1 tctcgaggcc cctctacaca ctccccaact taggtcacaa tTTATGTATC
P876247     1 -----GCTTG CC-------- ---------- ---------- -TAAcacg--
P876248     1 acagtGCTTG CC-------- ---------- ---------- -TAATATCTC
                   ** *** **                              ** *******
```

```
alignment position  51........ 61........ 71........ 81........ 91........
P876246          51 TGTCTGGTTA AAGCTTCGTa ttagaaagg- -------GAG GGAACCCAAA
P876247          15 ---------- ---------- -GGAGGCACC AAGTTCAGAA GAAGGCCAAA
P876248          22 TATAGGGCTG AAGAATCGTg aGGAGGCCCC GAGTTCAGAG GAAAGCCAAA
                    ********** *********   ********* ********** **********
                                           ********* ********** **********
                                                            *** **********

alignment position  101....... 111....... 121....... 131....... 141.......
P876246          93 GTATC-TTTT CTCTTCGGGG TCTCAACCGG CCAACCACca cg-TTTTTAG
P876247          44 CTAGCaTTTT TCCTTGGGTG TCTCAGCCAG CTAACTAGgg ga-TTTTTAA
P876248          72 GGAGC-TTTT TCCTTGGGTG TCTCAGCCAG CTAACTcagg attTTTTTAG
                    ***** **** ********** ********** ********      *******
                    ***** **** ********** ********** ******        *******
                    ***** **** ********** ********** ******        *******
                          **** ********** ********** ******
                          **** ********** *******
                          **** ********** *******

alignment position  151....... 161....... 171....... 181....... 191.......
P876246         141 GCCGACCTGG GAGGCAGGGG GaacgcaacA GACTGCGAAG TCCTTAAGGA
P876247          93 GACAACTTGG AAGGAGGGGG GTGAAGTG-A GCCTCTGAAA TCCTCAGGGA
P876248         121 GACAACTTGG AAGGAGGGGC tTGAAGTG-A GCCTCTGAAA TCCTCCGGGA
                    ********** ********** ******** * ********** **********
                    ********** *********   ******* * ********** **********
                    ********** *********   ******* * ********** **********
                                           ******* * ********** **********

alignment position  201....... 211....... 221....... 231....... 241.......
P876246         191 AGAA----TC taaacaagtc cgccgtttgC TGTTTGGGAG TGCGATACAA
P876247         142 ATAAataaGC CGGAGAAGtg gctgc----C TGGCAGAGCG AGCCCTACAA
P876248         170 ATAA----GC CGGCGAAGcc aactgctatC TGGTAGAGCG AGCCCTACAA
                    ****    ** ********            * ********** **********
                    ****                           * ********** **********
                    ****                           * ********** **********
                    ****                                    *** **********

alignment position  251....... 261....... 271....... 281....... 291.......
P876246         237 AATGAATATG TACGCAGGTg gaacgtagac tgcagatttt gagccagcgg
P876247         188 AATAAACACT CGGGCAGGTA AACGCACTAA CggTAACCAC TCGGCGA---
P876248         216 AATAAACACG CAGGCAGGTA AATGCACTAA C--TAATCAC TCGGCGA---
                    ********** ********** ********** *  ******* *******
                    *********    *******                ******* *******
                    *********                           ******* *******
                    *********                           ******* *******

alignment position  301....... 311....... 321....... 331....... 341.......
P876246         287 cgTTTCTGCC GAAATCCTGG CAAATTCC-- --GGTAGAAg tcggctgcag
P876247         235 --TTTTGGCC AAAACTTTGA CAAACGACgc taGCTAGAAA CTGGCGCGTG
P876248         261 --TTTTTGTC AAAACTTAGA CAAACGAC-- --GCTAGAAA CTGGAGCGAG
                      ******** ********** ********     ******** **********
                      ******** ********** ********     ******** **********
                      ******** ********** ********     ******** **********
                      ******** ********** ********     ******** **********

alignment position  351....... 361....... 371....... 381....... 391.......
P876246         333 gaggcggagg aggagactaa ---------- ---------- ----------
P876247         283 AGAGGAAAAC CGAAGAACTG AAaagcaaag caaaacaaaa aactaaaacc
P876248         305 AGAGGAAAAC CGAAGAGCCG AA-------- ---------- ----------
                    ********** ********** **
                    ********** ********** **
                    ********** ********** **
                    ********** ********** **

alignment position  401....... 411....... 421....... 431....... 441.......
P876246         353 ---------- ---------- -AAAACCAGA AAACAACCAG CAAACCAGCT
P876247         333 AAAAAAAACC AAAAAGAACC CAAAACAAAA AAAGTACCAT CCACCAAGCC
P876248         327 AAACAAAATC AAAGGAACAA GCAAACAAAA AGAGGAACcg tctacaaaca
```

```
                         ********** ********** ********** ********** **********

alignment position       451....... 461....... 471....... 481....... 491.......
P876246            382   AGGCAgagct gagcggagaa gctccagagc cttttaaaga gactctcGTC
P876247            383   CGGAAcacaa --------AG GAGGTTCCAG CCTAAAAATT CTGAGGTGTC
P876248            377   gcgaactgaa taaccaagAG GAGGTTCCAG CCTAGAAATT CTAAGGTGTC
                         *****              ** ********** ********** **********
                                            ** ********** ********** **********
                                            ** ********** ********** **********
                                            ** ********** ********** **********

alignment position       501....... 511....... 521....... 531....... 541.......
P876246            432   ACATGACACC CCCaaccccg acccccagcc ggcgCGCCTC CGCCCtcg--
P876247            425   ATATGATAGT CCC------- ---------- ----CGCCTC CGCCCCGCCG
P876248            427   ACATGACAgc gtcccccgc- ---------- ---------- --CCCGGCCC
                         ********** ***                         ****** **********
                         ******
                         ******
                         ******

alignment position       551....... 561....... 571....... 581....... 591.......
P876246            480   ---------- ---------- --------GG TGGCGGGGCc gcctgGCGTC
P876247            454   GCTCCGCC-C CCAAAGGCAG GGCCACACGG GGGGAGgagc gaT--GCGTC
P876248            454   GCTCCGCCgC CCAAAGGGAG GGTTACGCGG GGGCGGGGCg t-T--GGGTC
                         ******** * ********** ********** *********    *  *****
                                                                      *  *****
                                                                      *  *****
                                                                      *  *****
                                                                         *****
                                                                         *****
                                                                         *****
                                                                         *****
                                                                           ***

alignment position       601....... 611....... 621....... 631....... 641.......
P876246            502   ACTTCCGTCC AGACCGGAAC CCAAGATGGC TGCGCTGTTG CTGAGGTGAC
P876247            501   ATTTCCGTCC AGGCCGGAAC TCAAGATGGC TGCGtTCTTG CTGAGGTGAC
P876248            501   ACTTCCGTCC AGGCCGGAAC TCAAGATGGC TGCGCTCTTG CTGAGGTGAC
                         ********** ********** ********** ********** **********
                         ********** ********** ********** ********** **********
                         ********** ********** ********** ********** **********
                         ********** ********** ********** **** ***** **********
                         ********** ********** ********** **** ***** **********
                         ********** ********** ********** **** ***** **********
                         ********** ********** ********** **** ***** **********
                         ********** ********** ********** ****   *** **********
                         ********** **********  ********* ****
                                                *********  ****

alignment position       651....... 661....... 671....... 681....... 691.......
P876246            552   TTCAG--TGG GACTGGGAGT TGGTGCCTGC GGCCCTCCGG AGATC-TGAA
P876247            551   TTTAGCCTGG GGCTAGGAGT CCACGCCCGC GACCGTTGGG GGAACCTGAA
P876248            551   TTTAGCCTGG GGCTGGGAGT TCGTTCCcgt tactctt-GG GGAGCCTGAA
                         ********** ********** ********** ********** **********
                         ********** **********                    ** **********
                         ********** **********                    ** ***** ****
                         ********** **********
                         *****  *** **********
                         *****
                         *****
                         *****

alignment position       701....... 711....... 721....... 731....... 741.......
P876246            599   CTGGCCCCTC Acgttttgct gataactgtt tatcctgTGC CTGGGCAGGG
P876247            601   TTAGTCCCTG AGGGGGTGAC AGCGGtGTGT GCCT---TGC GGGGACAGGG
P876248            600   TTGGTCCCTC AGTGGGTAAC AGTGGgGTGT GCCC---TGC GGGAACAGGG
                         ********** ********** ***** **** ****   *** **********
                         ********** ********** ***** **** ****   *** **********
                         ********** *                **** ****   *** **********
                                                                 *** **********
```

```
                                                            *** **********
alignment position 751....... 761....... 771....... 781....... 791.......
P876246        649 AAAGGACCCA TGGGTGTGGA GGCCaagcgC TCGGGATCC TAGAGACCCC
P876247        648 AAAGGACCTG CGGGTGTGCA GGCCGCGC-C CCTGGGATCT TAGCACCCCC
P876248        647 AAAGGACCTG TGG-TGTGCA GGCCGGGT-C CCTGGGATCT TAGCACCCTC
                   ********** ********** ******** * ********** **********
                   ********** ********** ******** * ********** **********
                   ********** *** ****** ******** * ********** **********
                   ********** *** ****** ******** * ********** **********
                   ********** *** ****** ****
                                  ****** ****

alignment position 801....... 811....... 821....... 831....... 841.......
P876246        699 TTTTC-CCgt cccccccagc cgctccggtg cGCTCCGTAG GGCTTCGGGG
P876247        697 TCTTCtCCCC CGGCGGAGAG ---------- -GCTCGGAAG GGCTTAGAGG
P876248        695 TCTTC-CCCC CGGCAGAGAG ---------- -CCTCGGAAA GGCTTAGAGG
                   ***** **** **********             ********* **********
                   ***** **** **********             ********* **********
                   ***** **** **********             ********* **********
                   ***** **** **********             ********* **********

alignment position 851....... 861....... 871....... 881....... 891.......
P876246        748 TCACTgactt cgtatcgagg ggccCTCGGC TCTCGCCCCT TCTGTTTTCC
P876247        736 TCACACTgtg tgtgGCCGAG TGG-CGCGGC TGTCATTGTT TCTGCCTCGC
P876248        733 TCACTCTgtg ta--GCCGAG TGG-CGCGGC TGTCATTGTT TCTGTCTCGC
                   *******        ****** *** ****** ********** **********
                   *******        ****** *** ****** ********** **********
                   *******        ****** *** ****** ********** **********
                   *******        ****** *** ****** ********** **********
                                  ****** *** ****** ********** **********

alignment position 901....... 911....... 921....... 931....... 941.......
P876246        798 CCACCTCctc tagtacttct aaCGCAAATT GCTCTCGGGC CTGCACCCAG
P876247        785 CCACCTCTAA AGCCTCTT-- --CGAAAACT GCTCTTGTGG CCCCAaaaag
P876248        780 CCACGTCTAA AGCCTCTT-- --CGCAAACT GCTCTTGTGG CCCCACCCAG
                   ********** ********     ******** ********** **********
                   **** ***** ********     ******** ********** **********
                   **** ***** ********     ******** ********** ******
                   **** ***** ********     ******** ********** ******
                   **** **                 ******** ********** ******

alignment position 951....... 961....... 971....... 981....... 991.......
P876246        848 AGCCGAGCtc tgagaaaaTA ACTTCAAGGT CAGCCACCCA TGGGTCCTAC
P876247        831 ctgctg---- --------TA ACTTCGAGAT CAGCGGGCCC CTAGGCCTGC
P876248        826 GTCCGAGCct caat------ ---------- ---------C CTAGGCCTAC
                   ********           ** ********** ********** **********
                   ********                                  * **********

alignment position 1001...... 1011...... 1021...... 1031...... 1041......
P876246        898 GATTTCGAGA GCGtatttag acatttagct tc-GGAGAGA GAGATCTTCG
P876247        869 AGTTTTGGGG Ggac------ TTAGGGATTG TAGAGGGAGG GGCTCCTTGG
P876248        851 AATTTTGGGG GAGggggat  TTAGCGATTG TAGGGGGAGG GGCTCCTTGG
                   ********** ***        ********** ********** **********
                   ********** *          ********** ********** **********
                                         ********** ********** **********
                                         ********** ********** **********

alignment position 1051...... 1061...... 1071...... 1081...... 1091......
P876246        947 TGTATTTTGC CTTTGCTTCG AAtgccttca gacactagct gctcccaccg
P876247        913 AGCACCTAct gggtgc-TTT AACTCCTGGC Aa---TCCTG CTAGCAACCG
P876248        901 AGCACCTAGT CTGTGCTTTT AACTCCTGGC AgctcTACGG CTGGCAGCCT
                   ********** ********** ********** *    ***** **********
                   ********
                   ********
                   ********

alignment position 1101...... 1111...... 1121...... 1131...... 1141......
P876246        997 tgagtg---- ---------- ---------- ---------- ----------
```

```
P876247     959  GTGG AAA CTG  TC g GGCAT CC  TAAT GCA T C C  C T C C C A A G CC  AAG G- - - - - -
P876248     951  GTGG AGA CTG  TC a GGCT T CC  TAAT GCA T C C  C T C C C A T G CC  AAG Gt c a a g a
                 **** *** ***  **   ****** **  ******** * * *  * * * * * * * * **  *** *
                                    ****** **  ******** * * *  * * * * * * * * **  *** *
                                    ****** **  ******** * * *  * * * * * * * * **  *** *

alignment position 1151
P876246    1003 -
P876247    1003 -
P876248    1001 a
```

---

## Pairwise similarities:

For each pairwise alignment, the similarity (relative to the maximum similarity) and the number of identical nucleic acids (in % of shorter sequence) is given. Maximum values are underlined.

| | **P876247 (1002 bp)** | **P876248 (1001 bp)** |
|---|---|---|
| **P876246 (1002 bp)** | 0.164<br>35 % | 0.186<br>41 % |
| **P876247 (1002 bp)** | | 1.000<br>73 % |

Please note that the **similarity value 1.000** marks only the two **most similar** sequences, it does not necessarily mean that these sequences are identical.

---

| **Extract alignment region** | |
|---|---|
| **Positions in alignment** | from: to: |
| **Output file** | dialign.seq |

Extract aligned sequences

---

For comments, questions or bug reports, please contact support@genomatix.de

---

© Genomatix Software GmbH 1998-2005 - All rights reserved. License Agreement GEMS Launcher 4.1.1

Genomatix-Logo Logout Personal Messages GEMS-Logo
Logout Personal Messages

Genomatix_MainGEMSElDoradoGene2PromoterBiblioSphere
Frequently_asked_questionsYour_ResultsYour_SequencesYour_ProtocolHelp

**GEMS Launcher Task: *DiAlign TF*: Multiple alignment plus TF sites**
**working on promoters_2.seq (3 seq.)**

[Alignment] [Pairwise_similarities]

**DiAlign professional TF Release 3.1 December 2004** **Wed Jun 8 21:46:18 2005**

# Solution parameters:

| | |
|---|---|
| **Sequence file:** | promoters_2.seq (3 seq.) |
| **Type of sequences:** | nucleotide sequences |
| **Threshold T:** | 0.00 |
| **'*' signs below alignment denote:** | diagonal similarity (max. similarity is represented by 10 '*' signs) |
| **Alignment output:** | complete alignment is shown<br># nucleic acids per line: 50 |
| **TF output:** | common TF matches located in aligned regions (common to 3 (100.0 %) sequences) |
| **Family matches:** | yes |
| **MatInspector library:** | Matrix Family Library Version 5.0 (February 2005) |
| **Selected groups** (core/matrix sim): | • ALL user_defined.lib (0.75/Optimized)<br>• ALL vertebrates.lib (0.75/Optimized) |

# Aligned Sequences:

| No. | Sequence Name | Sequence Description | Length |
|---|---|---|---|
| 1 | P876742 | sym=SDHD\|loc=Loc6392\|taxid=9606\|spec=Homo sapiens\|chr=11\|ctg=NT_033899\|str=(+)\|start=15519538\|end=15520538\|len=1001\| tss=501\| comm=succinate dehydrogenase complex, subunit D, integral membrane protein; (AK075360/520/gold;NM_003002/520/bronze;) | 1001 bp |
| 2 | P876743 | sym=Sdhd\|loc=Loc66925\|taxid=10090\|spec=Mus musculus\|chr=9\|ctg=NT_039473\|str=(-)\|start=10108845\|end=10109850\|len=1006\| tss=501,506\| comm=succinate dehydrogenase complex, subunit D, integral membrane protein; (AK013503/507/gold;AK013962/502/gold;NM_025848/502/bronze;) | 1006 bp |
| 3 | P876744 | sym=Sdhd\|loc=Loc363061\|taxid=10116\|spec=Rattus norvegicus\|chr=8\|ctg=NW_047799\|str=(-)\|start=23593559\|end=23594559\| len=1001\|tss=501\| comm=succinate dehydrogenase complex, subunit D, integral membrane protein; (XM_343388/519/bronze;) | 1001 bp |

# Alignment (DiAlign format):

Please note that only **upper-case** letters are considered to be **aligned**. For more information, please have a look at the user guide.

V$AREB V$EBOX V$ETSF U$NRF2 V$RORA V$EREF V$HNF4 U$EREF V$SF1F V$YY1F V$FKHD

```
alignment position 1.........   11.........   21.........   31.........   41.........
P876742        1  gc--------  ----------  ----------  ----------  ----------
P876743        1  cctgtGCTCC  AAGGTCGGAA  CCCAGAGATT  CCGAGTGCCA  TTcccgACCA
P876744        1  -----GCTCC  AAGGTCAGAA  CCCAGAGATC  CCGATTGCTA  TTtcgaACCA
```

```
                                 ***** ********** ********** ********** **    ****
                                 ***** ********** ********** ********** **
                                 ***** ********** ********** ********** **

alignment position            51........ 61........ 71........ 81........ 91........
P876742                3      ---------- ---------- ---------G TCGCCCAAAT CTTCCCTGTT
P876743               51      GCGACCAGCT AAT--AACCG CATGCTCCTG TGGCCCAAAC CTTTCCGCTG
P876744               46      GCGACCGGCT AATatAACCG CATGCTTCTG TTGCCCAAAT CTTTCTGCTG
                              ********** ***  ***** ********** ********** **********
                                              ***** ********** ********** **********
                                              ***** ********** ********** **********

alignment position            101....... 111....... 121....... 131....... 141.......
P876742               24      TTacaccttt tcttttctCC ACGCTACGCT TATATACCTG GCACcTGAGC
P876743               99      TTTTCgAACC CTTTCCTCCC ACGCAGCGCT TGCCTCCCGG GCAC-TGAGC
P876744               96      CTTTCcAACC CTTTGATCCC ACACAGCGCT TGCCTCACGG GCggtcgcta
                              ***** **** ********** ********** ********** **** *****
                              ***** **** ********** ********** ********** **
                              ***** **** ********** ********** ********** **

alignment position            151....... 161....... 171....... 181....... 191.......
P876742               74      ACTACCGGTC ACCAGGGaca ggagagccat aactttgtct ttcgtgaggg
P876743              148      ACTGTAGATC ACTGGGGCGG GAGGC----- ---------- ----------
P876744              146      ---------- ----GGGTGG GAGGC----- ---------- ----------
                              ********** ********** *****
                                             ****** *****
                                             ****** *****

alignment position            201....... 211....... 221....... 231....... 241.......
P876742              124      gaatgggatG CAGCCGGGAT CGAGcaccag tgagc--CGC CAGTGTACAG
P876743              173      ---------G CAGCCCGGAT CGAGTGGGGC CGCTGTGCcG CAGTGTCCAG
P876744              157      ---------G CAGCCGGGAT CGGGTGGGCC CGCTCTGCTG CAGTGTCCAG
                                       * ********** ********** ********** **********
                                       * ********** ********** ******** * **********
                                       * ********** ********** ******** * **********
                                       * ********** **                * * **********
                                                                          **********

alignment position            251....... 261....... 271....... 281....... 291.......
P876742              172      ACCTCCGAGC GTGCCCAGGA CCaccaagga aggtgaaact TCCTTTCCCT
P876743              214      AGCTCCGTGA GTGCCCGGGA TCTGCGcCTC ACCC------ TCCTTCCCCT
P876744              198      GGCTCTGTGA GTGCCCGGGA TCTGCGtCTC ACCC------ TCCTTTCCCT
                              ********** ********** ****** *** ****       **********
                              ********** ********** ****** *** ****       **********
                              ********** ********** ******                **********
                              ********** ********** ******
                              ********** **********

alignment position            301....... 311....... 321....... 331....... 341.......
P876742              222      TCACCCTCCC CGTCCCCGCA CCTGTGCAGT AAACTGCGCC TTCTGCTGCT
P876743              258      GTTCCTTCCT CATCCTCGTA CCTGCGCAGT GAATTGCGCC TTCTGCTGTT
P876744              242      GTTCt----T CATCCTCGTA CCTGCGCAGT GAACTGTGCC TTCTGCTGTT
                              ********** ********** ********** ********** **********
                              ********** ********** ********** ********** **********
                              ****     * ********** ********** ********** **********
                                       * ********** ********** ********** **********
                                           ******** **********  ********* **********
                                           ******** **********  **    *** **********
                                                                **    *** **********
                                                                      *** **********
                                                                      *** ******** *
                                                                      *** ******** *

alignment position            351....... 361....... 371....... 381....... 391.......
P876742              272      CGGCGGCCAC CAGGCGCTGC AACTCCGCTT CATCGGCTTC GCCCAGCTCC
P876743              308      CGGCGGCCAC CAGGCGTTGT AACTCCGCTT CGTCCGCTTC ACCAAGCTCG
P876744              288      CGGCGGCCAC CAGGCGTTGT AACTCCGCTT CGTCCGCTTC ACCAAGCTCG
```

```
                              ********** ********** ********** ********** **********
                              ********** ********** ********** ********** **********
                              ********** ********** ********** ********** **********
                              ********** ********** ********** ********** **********
                              ********** ********** ********** ********** **********
                              ********** ********** ********** ********** **********
                              ********** ********** ********** *********
                              ********** ********** ********** *********
                              ********** *********  *******
                              ********** *********  *******

alignment position            401....... 411....... 421....... 431....... 441.......
P876742                  322  GCCATTGTTC GCCTCAGGCT CGCCACcttc cgacagctgt gtttgcgcat
P876743                  358  GCCATTGTCG GAGTCAggcg cttcgCTGGG GTGCGCAC-- ----------
P876744                  338  GCCATGGTCA GCGTCAATCT GTTCACTGGA GTACGCAC-- ----------
                              ********** ********** ********** ********
                              ********** ******          ***** ********
                              ********** ******          ***** ********
                              ********** ******
                              ********
                              ********

alignment position            451....... 461....... 471....... 481....... 491.......
P876742                  372  GCGCGACGGG TGTGCACCGC CTCTCGACTT CCGGTTCACc cagcatttcc
P876743                  396  GCGCGGTGGC AGCGCACCGT CTCCTGCCTT CCGGTTCACG CTTCAGGTTG
P876744                  376  GCGCGGTGGC AGAGCACCAC CTCCTGCCTT CCGGTTCACT CTTGAGGTTG
                              ********** ********** ********** ********** **********
                              ********** ********** ********** ********** **********
                              ********** ********** ********** ********** **********
                              ********** ********** ********** *********
                              ********** ********** ********** *********
                                                           *** *********

alignment position            501....... 511....... 521....... 531....... 541.......
P876742                  422  tcttccctgt tttctttcgt cgTCGTGGGT GGGAATTGTC GCCTAAGTGG
P876743                  446  CCTCACCGTT CTTTTACgc- -----GGGGC GGGACTTACT ACATAACTGT
P876744                  426  CTTCGCCGTT CTTTTAC--- --TCGGGGGC GGGACTTACG ACATAACTGC
                              ********** ********     ******** ********** **********
                              ********** ********        ***** ********** **********
                              ********** ********        ***** ********** **********
                                                          **** ********** **********

alignment position            551....... 561....... 571....... 581....... 591.......
P876742                  472  TTCCGGGTTG GtGGATGACC TTGAGCCCTC AGGAACGAGA TGGCGGTTCT
P876743                  490  TTCCGGGTTG TAGAGTGACC TTGAGCCCTC GAAAGCGACA TGGCGGTTCT
P876744                  471  TTCCGGGccG TAGGGTGACC TTGAGCCCTC AAAAGCGAAA TGGCGGTTCT
                              ********** ********** ********** ********** **********
                              *******  * ********** ********** ********** **********
                              *******  * ********** ********** ********** **********
                              *******  * * ******** ********** ********** **********
                                           ******** ********** ********** **********
                                           ******** ********** ********** **********
                                              ***** ********** ********** **********
                                              ***** ********** ********** **********
                                                                                 ***

alignment position            601....... 611....... 621....... 631....... 641.......
P876742                  522  CTggaggctg agtgccgttT GCGGTGCCCT AGGAGGCCGA GGTGAGGGGT
P876743                  540  CTTAAAGCTG GGCGTTCTCT GCAGTGGCCA AGGAGCTCGA GGTGAGGTGT
P876744                  521  CTTAAAGCTG GGCGTTCTCT GCAGTGGCCA AGGAGCTCGA GGTGAGGTGT
                              ********** ********** ********** ********** **********
                              ********** ********** ********** ********** **********
                              ********** ********** ********** ********** **********
                              ********** ********** ********** ********** **********
                              ********** ********** ********** ********** **********
                              **                  * ********** ********** **********
                              **                  * ********** ********** **********
                              **                  * ********** ********** **********
                              **

alignment position            651....... 661....... 671....... 681....... 691.......
```

```
P876742         572 CTTcccaccc tgAGGTGCTT AGCGTAGCCT CCAGCCAGGG AAGGGGATGg
P876743         590 CTTGGCCATC AAAGGTTCTT GCTGCAGGCT GAAGCAGGGG AGAGGAAGG-
P876744         571 CTTGGCCATC AAAGGTTCTT GCTGCAGCCT GGAGCAGGGG AAAGGAAGG-
                    ********** ********** ********** ********** *********
                    ********** ********** *******      ******** *********
                    ********** ********** *******      ******** *********
                    ********** ********** *******
                    ********** ********** *******
                    ***
                    ***
                    ***
alignment position  701....... 711....... 721....... 731....... 741.......
P876742         622 aAGTGAGGAC TCATCTGCCG GGTGG-GAGA TCTCTTGAGG AGAAGAAAAT
P876743         639 -AGTTAGAGC TCGCCCGCTG GGTGGTGGGA CGTCTTGAGA GGAGGGAACT
P876744         620 -AGTGAGGGC TCGACTGCCG GGTaGTGGGA CGTCTTGAGA GGAAGAAACT
                     ********* ********** ********** ********** **********
                     ********* ********** *** ****** ********** **********
                     ********* ********** *** ****** ********** **********
                                                ****  ********** **********
alignment position  751....... 761....... 771....... 781....... 791.......
P876742         671 ACCgaaat-- ---------- ---------- ----CACAGC AATGACCACT
P876743         688 TGCTCTTGTG CCCTcgaggc acCACCTATG CGGACGCAAC GTTGACCGCT
P876744         669 TCCTCTGGTG CCCTcgggg- --CACCTCTG CGGACGCAGC GGTGACCGCG
                    ********** ****         ******** ********** **********
                    ********** ****         ******** ********** **********
                    ********** ****                        **** **********
                    ***
alignment position  801....... 811....... 821....... 831....... 841.......
P876742         695 GTAGTCTAGG GGTCCAGATG TTTACccgaa ggtatatttc a---------
P876743         738 ATAGCCTAAA GGCCCAAGTG TTTACgtgCT GGCTGGTGTC CCCAGCTCGT
P876744         716 GTAGCCCAG- GGCCCAAGTG TTTACaacCT GGCTGGTCTC CCCTGCTCGT
                    ********** ********** *****   ** ********** **********
                    ******     ********** *****   ** ********** **********
                    ******                *****   ** ********** **********
alignment position  851....... 861....... 871....... 881....... 891.......
P876742         736 ---------- ---------- ---------- ---------- ----------
P876743         788 ACTTTCCGCT TCTGCACTGT GGTTGTGACC CCGGGCTAAT ATGAAAACAA
P876744         765 ACTTTTCGCT TCTGGACTGT GGTTGTGACA CCGGGCTAAT GTGAAAACAA
                    ********** ********** ********** ********** **********
                    ********** ********** ********** ********** **********
                    ********** ********** ********** ********** **********
                                       ** ********** ********** **********
alignment position  901....... 911....... 921....... 931....... 941.......
P876742         736 ---------- ---------- ---------- ---------- ----------
P876743         838 ACTCCTATtt gaGTGTGGAT GGAAGGAGTC CGTTTGATAa tTTGTTCTGA
P876744         815 ACTCCTATcc gtGTGTGGAT GGAAGGAATC TGTTTGATAg -TTGTTTTTA
                    ********     ******** ********** *********   *********
                    ********     ******** ********** *********
                    ********
                    ********
alignment position  951....... 961....... 971....... 981....... 991.......
P876742         736 ----CTTGCT G--TGagctg acgagttgag ggaataatca gaaagagagc
P876743         888 GTGGCTTGCT G--TGT---- ---------- ---------- ----------
P876744         864 GTGGCTTGCT ataTGT---- ---------- ---------- ----------
                    ********** *  ***
alignment position  1001...... 1011...... 1021...... 1031...... 1041......
P876742         780 tccctctgga agtcgcagtc ctgatgaggc taatccacat agcAGTTCTG
P876743         902 ---------- ---------- ---------- ---------- ---AGTGCTG
P876744         880 ---------- ---------- ---------- ---------- ---AGTTCTG
                                                                   *******
alignment position  1051...... 1061...... 1071...... 1081...... 1091......
```

```
P876742      830 TT- TTCt ccc  cgttcactgt  ccctagaatg  ctccccactc  gctcccaccc
P876743      909 TC- TTCCTCG  ATCTAGCCAT  GTAGACAAGA  CTAACCTA--  ----------
P876744      887 gct TTCCTCG  ATCTAGCCAT  GTAGACAAGA  CTAACCTA--  ----------
                 **  *******  **********  **********  ********
                     *******  **********  **********  ********
                     *******  **********  **********  ********
                     *******  **********  **********  ********
                     *******  **********  **********  ********

alignment position 1101......  1111......  1121......  1131......  1141......
P876742      879 tgagtcggga  aagagggtta  gGAGCTTGCC  CATTTCTTCT  GGAGTTGGTg
P876743      946 ----------  ----------  -GAGCTTGCC  GGGAGCCCCG  TGACTGc-TC
P876744      925 ----------  ----------  -GAGCTAGCC  GGTAGCTCCC  TGACTGGTTC
                                          *********  **********  **********
                                          *****                         **
                                          *****
                                          *****
                                          *****

alignment position 1151......  1161......  1171......  1181......  1191......
P876742      929 tgtttggatg  tgggagtgga  gggggggatca gttcgaaaat  cattaacct
P876743      974 CCGAGGGCTG  CCATAAAAAC  CGTGCACCCT  CAC-------  ----------
P876744      954 CCGAGTGCTG  GCATAACAAC  CATGCACCAT  CACtcagggc  ttcgataa--
                 **********  **********  **********  ***
                 **********  **********  **********  ***

alignment position 1201......  1211......  1221
P876742      979 gggcatttgt  gttacctcag  gta
P876743     1007 ----------  ----------  ---
P876744     1002 ----------  ----------  ---
```

---

## Pairwise similarities:

For each pairwise alignment, the similarity (relative to the maximum similarity) and the number of identical nucleic acids (in % of shorter sequence) is given. Maximum values are underlined.

| | P876743 (1006 bp) | P876744 (1001 bp) |
|---|---|---|
| P876742 (1001 bp) | 0.215 39 % | 0.213 39 % |
| P876743 (1006 bp) | | 1.000 83 % |

Please note that the **similarity value 1.000** marks only the two **most similar** sequences, it does not necessarily mean that these sequences are identical.

---

| **Extract alignment region** | |
|---|---|
| **Positions in alignment** | from: to: |
| **Output file** | dialign.seq |

Extract aligned sequences

---

For comments, questions or bug reports, please contact support@genomatix.de

© Genomatix Software GmbH 1998-2005 - All rights reserved.

License Agreement

GEMS Launcher 4.1.1
