# Supplementary material for: Transcriptional co-expression and co-regulation of genes coding for components of the oxidative phosphorylation system
Source: BMC Genomics. 2008 Jan 14;9:18. doi: 10.1186/1471-2164-9-18 (PMC2268925; doi:10.1186/1471-2164-9-18)
Supplement: Additional file 6 — Phylogenetic footprinting of Complex III core promoters. Core Promoters of Complex III subunits/related genes identified by phylogenetic footprinting. The conserved regions are shown in color codes, which correspond to specific Transcription Factor Binding Sites (TFBS). [file 1471-2164-9-18-S6.pdf]

This Supplementary Material shows the Core Promoters of Complex III subunits/related genes identified by phylogenetic footprinting. The conserved regions are shown in color codes, which correspond to specific Transcription Factor Binding Sites (TFBS).

The description of the complete description of TFBS can be found in additional file 3 (in the “Matrix Family Library” Excell sheet).

Genomatix-Logo	Logout	Personal	Messages	GEMS-Logo
	Logout	Personal	Messages	

Genomatix_MainGEMSElDoradoGene2PromoterBiblioSphere
Frequently_asked_questionsYour_ResultsYour_SequencesYour_ProtocolHelp

GEMS Launcher Task: *DiAlign TF*: Multiple alignment plus TF sites
working on promoters.seq (3 seq.)

[Alignment] [Pairwise_similarities]

DiAlign professional TF Release 3.1 December 2004 **Thu May 19 15:54:04 2005**

Solution parameters:

**Sequence file:**	promoters.seq (3 seq.)
**Type of sequences:**	nucleotide sequences
**Threshold T:**	0.00
**'*' signs below alignment denote**:	diagonal similarity (max. similarity is represented by 10 '*' signs)
**Alignment output:**	complete alignment is shown<br># nucleic acids per line: 50
**TF output:**	common TF matches located in aligned regions (common to 3 (100.0 %) sequences)
**Family matches:**	yes
**MatInspector library:**	Matrix Family Library Version 5.0 (February 2005)
**Selected groups** (core/matrix sim):	• ALL user_defined.lib (0.75/Optimized)<br>• ALL vertebrates.lib (0.75/Optimized)

Aligned Sequences:

No.	Sequence Name	Sequence Description	Length
1	P986561	sym=CYC1|loc=Loc1537|taxid=9606|spec=Homo sapiens|chr=8| ctg=NT_023684|str=(+)|start=1115192|end=1116211|len=1020| tss=501,520| comm=cytochrome c-1; (AK026633/517/gold;NM_001916/536/silver;)	1020 bp
2	P986563	sym=Cyc1|loc=Loc66445|taxid=10090|spec=Mus musculus|chr=15| ctg=NT_039621|str=(+)|start=37804647|end=37805670|len=1024| tss=501,524| comm=cytochrome c-1; (AK002815/524/gold;NM_025567/501/bronze;)	1024 bp
3	P986564	sym=na|loc=Loc300047|taxid=10116|spec=Rattus norvegicus| chr=7|ctg=NW_047780|str=(+)|start=8996190|end=8997190| len=1001|tss=501| comm=similar to cytochrome c-1; (XM_216944/535/silver;)	1001 bp

Alignment (DiAlign format):

Please note that only **upper-case** letters are considered to be **aligned**. For more information, please have a look at the user guide.

V$STAF V$YY1F V$AP2F V$AREB V$EREF U$EREF V$RORA V$RARF V$ZBPF V$MZF1 V$HESF V$SF1F

```
alignment
position       1.........  11........  21........  31........  41........

P986561     1  gcatcacgcg  tcgggccatg  ----------  ----------  ----------
P986563     1  ggcagcacca  agtgccacag  tgatcagtcc  cttacatcca  gagggagctg
P986564     1  ----------  ----------  ----------  ----------  ----------
```

```
alignment position   51........ 61........ 71........ 81........ 91........

P986561   21 ---------- ---------- ---------- ---------- ----------
P986563   51 cagcttatcT TTGTAATGGA CGGGTACCCC CACTTAGATA TGCTCTCAtt
P986564    1 ---------T TTGTGATGGA CAGGTACCCC CACTTAGTTA TGTTCTCAgt
                      * ********** ********** ********** ********
                      * ********** ********** ********** ********
                      * ********** ********** ********** ********

alignment position   101....... 111....... 121....... 131....... 141.......

P986561   21 ---------- ---------- ---------- ---------- ------AGTC
P986563  101 aAGAAAATTG AATTATGGAc tTTATATGAG GGTTTAAAAG AAAGTTAGTC
P986564   42 gAGAAAATTG AATTATAGAa cTTATATGAG GGTTTAAAAG AAATTTAGTT
              ********* *********   ********* ********** **********
                                    ********* ********** **********
                                    ********* ********** **********

alignment position   151....... 161....... 171....... 181....... 191.......

P986561   25 AAGGGAAtga aggagaacag ggtgctcagc ctgggggccc agcctcccgc
P986563  151 AGGGGAAGTT TCTATGCTGG GTCGCCT--- ---------- ----------
P986564   92 AGGGaggGTT TCTATGCCCA GTCGCCT--- ---------- ----------
             ********** ********** *******
             ****   *** ********** *******
             ****

alignment position   201....... 211....... 221....... 231....... 241.......

P986561   75 tgtcacgcgc tgcctgcggc cgagacccct gcccgcgccc tctgccgggc
P986563  178 ---------- ---------- ---------- ---------- ----------
P986564  119 ---------- ---------- ---------- ---------- ----------

alignment position   251....... 261....... 271....... 281....... 291.......

P986561  125 tgccctccaa gccgcccttt ctctggaggt cctcagcctg caggggcacc
P986563  178 ---------- ---------- ---------- ---------- ----------
P986564  119 ---------- ---------- ---------- ---------- ----------

alignment position   301....... 311....... 321....... 331....... 341.......

P986561  175 ctccacccgg ccatcgcgca gcctgggaag GTGGAGAAAA GGAGCGtcgg
P986563  178 ---------- ---------- ---------- GTGGAGCAGG GGAGAGct--
P986564  119 ---------- ---------- ---------- GTAGAGCAGG GGAGctacat
                                             ********** ******
                                             ********** ****

alignment position   351....... 361....... 371....... 381....... 391.......

P986561  225 ggtctcgga- ---------- ---------- ---------- ----------
P986563  196 -----GCTGT TAATCCCACA CAGGcCATGG ATGAGCAAGC tc--------
P986564  139 agagaGCCGC TTACCCCTCA CTGG-CATGG ATGGGCAAGC cactcttaac
                  ***** ********** **** ***** **********

alignment position   401....... 411....... 421....... 431....... 441.......

P986561  234 ---------- ---------- -------GGC GGCGTGGGAA ACGCCGGGCG
P986563  233 -TCAGAAGAC TATCCAGTGG CTCAACTGGA -GGGCGTGAT TCGCGGGGCC
P986564  188 tTCAGAAGAC TGTCCAGTAG CTTAACTGGA CGAGCGGGAT ACGCGGGGCC
              ********* ********** ********** ********** **********
              ********* ********** **********             *********
                                                          *********
                                                          *********

alignment position   451....... 461....... 471....... 481....... 491.......
```

```
P986561   257 GAGcgtggcg ctgtcacggc aacagagaga cgcgacgggg ccccgcccca
P986563   281 GAGGTGAAGG AACTGGGGAT ATAGTAGAGA AgccagaAAA ACAAACAAAC
P986564   238 AAGGTGAAGG AACTGGGAAT ATAGTAGAGA Agcag--AAA AAAAGAAAAA
              ********** ********** ********** *      *** **********
              ********** ********** ********** *
              ********** ********** ********** *
              ********** ********** ********** *

alignment
position          501.......  511.......  521.......  531.......  541.......
P986561   307 ---------- ---------- ---------- ---------- ----------
P986563   331 AAACAAAGCA ga-------- ---------- ------TGTT GTCATTGTAA
P986564   286 AAACAAAACA ataagacaaa aacaaaaaaa tccaggTGTT ATTATTGTAA
              **********                             **** **********
                                                     **** **********
                                                     **** **********

alignment
position          551.......  561.......  571.......  581.......  591.......
P986561   307 ---------C CGCCAGTTTC CACGACAACC CGAAGAGCGT GGGGAGGCAg
P986563   357 TTAGAGTTGC GGCCAGGCTC AACCCGGACT AGAGGAGCCG GGCAAGGGAC
P986564   336 TTAGAATTGC AGCCAGACTC AACCt----- ---------G GGGAAGGTAC
              ********** ********** ********** ********** **********
              ********** ********** ****                * **********
              ********** ********** ****                * **********
                                                        * **********

alignment
position          601.......  611.......  621.......  631.......  641.......
P986561   348 gcggtgcccc ggcccctgac tgacgcgacc gggaccagcg cgcttcgtcc
P986563   407 AGTCCTGGTC CACTGATGGA CAG------- ---------- ----------
P986564   372 AGCCCTGGTC CACTGATGGA CAG------- ---------- ----------
              ********** ********** ***
              ********** ********** ***
              ********** ********** ***
              ********** ********** ***

alignment
position          651.......  661.......  671.......  681.......  691.......
P986561   398 ccgcccaccc gacaggcCCC GCCCCCGAGC CCGGCCCCGC CCCGCGCTCc
P986563   430 ---------- -------CTC CCTACCCAGC TCCGCCTAGG TCCGCCCACT
P986564   395 ---------- -------CTC CCTATCCAGC TGGGCGTAGG CCCGCCCATT
                                *** ********** ********** **********
                                *** ***
                                *** ***
                                *** ***

alignment
position          701.......  711.......  721.......  731.......  741.......
P986561   448 ccg--GCTTT CGCGAGGTtt tgaCTCTCGT GGCGCCCCAG GGGCCGACGG
P986563   463 gtGTTGCTCT CGCGTGGTCT GAGCTCTCGT CGGCCCGCAG GGGCCGACGG
P986564   428 ggGTTGCTCT CGCGTGGTCT GAGTTCTCGT CGGCCCGCAA GGGCCGACGG
                ******** ********** ********** ********** **********
                ******** ********** ********** ********** **********
                ******** ********** ********** ********** **********
                ******** ********** ********** ********** **********
                                       ******* *********  **********
                                       ******* *********  **********
                                        ****** *********  **********
                                        ****** *********  **********

alignment
position          751.......  761.......  771.......  781.......  791.......
P986561   496 GAGTGGCGGC CGCgcgg-AG GAGGCCAAGA T---GGCGGC AGCTGCGGCT
P986563   513 GAACGGCGGC CGCACTAAAG GGGGCCGACA T---GGCGGC GGCGGCGGCT
P986564   478 GAACGGCGGC CGCACTAAAG AGGGCCGACA tggcGGCGGC GGCGGCGGCT
```

```
                     ********** ********** ********** *   ****** **********
                     ********** ********** ********** *   ****** **********
                     ********** ********** ********** *   ****** **********
                     ********** ********** **********     ****** **********
                     ********** ***     ** **********     ****** **********
                     ********** ***     ** **********     ****** **********
                     ********** ***     ** **********
                     ********** ***

alignment position   801....... 811....... 821....... 831....... 841.......
P986561          542 TCGCTTCGCg gggtaGTGTT GGGCCCGCGG GGCGCGGGGC TCCCGGGCGC
P986563          560 TCGCTTCGCC GgaCGGTACT GGGCCCGCGG GGCGTGGGGC TTCCAGGTGC
P986564          528 TCGCTTCGCC GatCGGTGCT AGGCCCGCGG GGCATGGGGC TTCCAGGTGC
                     ********** *  ******* ********** ********** **********
                     ********** *  ******* ********** ********** **********
                     ********** *  ******* ********** ********** **********
                     *********       ***** ********** ********** **********
                     *********           * ********** ********** **********
                     *********           * ********** ********** **********
                                                                          *

alignment position   851....... 861....... 871....... 881....... 891.......
P986561          592 gcGTGCCCGG GGTCTGCTGT GCAGCGCGCG TCCCGGGCAG CTcccgcTAC
P986563          610 AAGTGCTCCG GGTCTGCTGG GCGGCGCGCG GTCCCGGCAG CTTCCATTGC
P986564          578 AAGTGCTCCG GGCCTGCTGG GCGGCGCGCG GCCCCGGCAT CTTCCATTAC
                     ********** ********** ********** ********** **********
                     ********** ********** ********** ********** **********
                     ********** ********** ********** ********** **********
                     ********** ********** ********** ********** **     ***
                       ******** ********** ********** *********  **     ***
                       ******** ********** ********** *********  **       *
                       ******** ********** ********** *********  **

alignment position   901....... 911....... 921....... 931....... 941.......
P986561          642 GGACACCTCA GGTGAGCGCT GGGCCGGGCC CCGGCCTCCG CGCGGCCCcg
P986563          660 GGACACCGCA GGTGAGTCCT GGCGCGGGCC CCGACCATCG CGCGCCCCTC
P986564          628 GGACACCGCA GGTGAGTACT GGCACGGGa- CGGTCCATCG CGCGCCCCTC
                     ********** ********** ********** ********** **********
                     ********** ********** ********** ********** **********
                     ********** ********** ********       ****** **********
                     ********** ********** ********       ****** **********
                     ********** ********** ********       ****** **********
                     ********** ********** ********       **
                                                          **

alignment position   951....... 961....... 971....... 981....... 991.......
P986561          692 catctCCGTG AAGGTCACGG CGGGGAGGCT GCGGGCGCGG GCCtgggcag
P986563          710 AG---CCCTG AAGGTCACGG CGGGGAGGCT CCGGGCGCGC GCCGGCATCG
P986564          677 AG---CCCTG AAGGTCACGG CGGGGAGGCT CCGGGCGCGC GCCAGCATCG
                     **   ***** ********** ********** ********** **********
                     **   ***** ********** ********** ********** **********
                     **   ***** ********** ********** ********** **********
                     **   ***** ********** ********** ********** ***
                     **   ***** ********** ********** ********** ***
                          ***** ********** ********** ********** ***
                          ***** ********** ********** ********** ***
                          ***** ********** ********** ********** ***
                          ***** ********** ********** ********** ***
                          ***** ********** *******

alignment position   1001...... 1011...... 1021...... 1031...... 1041......
P986561          742 cgcggaagcg gtaccggcca cccagcgtcc ccGGTCCCAG CTGCCTGCCg
P986563          757 GTCTCCTGCG TTCCTCCTAC AACTCGACGG --GGTCCCCG CCGCCGGGCC
P986564          724 GTCACCTACA TTCCTCCTAC GACTCGGCAG --GGTCCCCG CTGACCGGCC
```

```
              ********** ********** **********   ******** **********
              ********** ********** **********   ******** **********
              ********** *******

alignment
position      1051...... 1061...... 1071...... 1081...... 1091......
P986561   792 accttgagcg tgtGGGATCA GGGCTGGGCG CCCACCTCTC CGAACGGCAG
P986563   805 GGCACACcca ga---AAGCC GGTCTGGGTG CCCACGCCTT GGAGTGGCAG
P986564   772 GGCACACtcg ---GGAAGCC AGTCTGGGTG CCCACGCCTT GGAGCCATAG
              *******       ******* ********** ********** **********
              *******          ***** ********** ********** ****
                                ***** ********** ********** ****

alignment
position      1101...... 1111...... 1121...... 1131...... 1141......
P986561   842 AGagcccgtc cccagcgtgg gggttgg--- ---------- -CGGGACGGG
P986563   852 ggcatTGTTG CTCTGAGCCC AGGGTCCCAC TGTGaTGGGG TCGGGACGGG
P986564   819 GGca-TGTTG CTCTGAGCCC AGGGTCCGAC TGTGgTGGGG TCGGGACGGG
              **   ***** ********** ********** **** ***** **********
                   ***** ********** ********** **** ***** **********
                   ***** ********** ********** **** ***** **********
                                                    ***** **********

alignment
position      1151...... 1161...... 1171...... 1181...... 1191......
P986561   878 ctagctgccg tggcggggct ggggctttcc CGAATGGCGC GCCCAGGACG
P986563   902 AGATTGCAG- ---------- ---------- CGAAGGGCTC GCCCGGAGCG
P986564   868 AGATTCCTG- ---------- ---------- CGAAGGGCTC GCCCAGAACG
              *********                        ********** **********
              *********                        ********** **********
              *********                        ********** **** *****
              *********                        ********** **** *****
                                               ********** ****

alignment
position      1201...... 1211...... 1221...... 1231...... 1241......
P986561   928 GCTCTTGCGG CTGGCTGTCC aaactgggcc cgcGTCCTGA AGTGACCCCA
P986563   931 ACTGCCGAAG CCGGCTGTCC GGACCTGGCG GATacCCTGC AGTGACCCCA
P986564   897 ACTCCCGAAG CCGGCTGTCT TGACCTGGCC GATGTCCTGA AGTGACCCCA
              ********** ********** ********** ********** **********
              ********** ********** ********** ********** **********
              ********** ********** ********** ********** **********
              ********** *********                  ***** **********
                                                    ***** **********
                                                          **********
                                                          **********

alignment
position      1251...... 1261...... 1271...... 1281...... 1291......
P986561   978 GCCTGATCTC GGCCAGCTGC TTGTGACCTT GGCCTGTCCC AGc-------
P986563   981 GCCTGACCTC AGCGGCCTGC AGGTGACCTT GGTCCGCTGC AGTA------
P986564   947 GCCTGACCTC AGCGGGCTGC GGGTGACCTT GGTCTGCTGC AGTActcttg
              ********** ********** ********** ********** ****
              ********** ********** ********** ********** ****
              ********** ********** ********** ********** **
              ********** ***** **** **********
              ********** ***** **** **********
              ********** ***
              ********** ***

alignment
position      1301.
P986561  1021 -----
P986563  1025 -----
P986564   997 gtctt
```

Pairwise similarities:

For each pairwise alignment, the similarity (relative to the maximum similarity) and the number of identical nucleic acids (in % of shorter sequence) is given. Maximum values are underlined.

	P986563 (1024 bp)	P986564 (1001 bp)
**P986561 (1020 bp)**	0.247<br>38 %	0.227<br>36 %
**P986563 (1024 bp)**		1.000<br>79 %

Please note that the **similarity value 1.000** marks only the two **most similar** sequences, it does not necessarily mean that these sequences are identical.

**Extract alignment region**	
**Positions in alignment**	from: ____ to: ____
**Output file**	dialign.seq

Extract aligned sequences

For comments, questions or bug reports, please contact support@genomatix.de

© Genomatix Software GmbH 1998-2005 - All rights reserved. License Agreement GEMS Launcher 4.1.1

Genomatix-Logo Logout Personal Messages GEMS-Logo

Genomatix_MainGEMSElDoradoGene2PromoterBiblioSphere
Frequently_asked_questionsYour_ResultsYour_SequencesYour_ProtocolHelp

GEMS Launcher Task: *DiAlign TF*: Multiple alignment plus TF sites
working on C3-HSPC051-HMR (3 seq.)

[Alignment] [Pairwise_similarities]

DiAlign professional TF Release 3.1 December 2004 **Mon May 2 23:33:24 2005**

Solution parameters:

**Sequence file:**	C3-HSPC051-HMR (3 seq.)
**Type of sequences:**	nucleotide sequences
**Threshold T:**	0.00
**'*' signs below alignment denote**:	diagonal similarity (max. similarity is represented by 10 '*' signs)
**Alignment output:**	complete alignment is shown<br># nucleic acids per line: 50
**TF output:**	common TF matches located in aligned regions (common to 3 (100.0 %) sequences)
**Family matches:**	yes
**MatInspector library:**	Matrix Family Library Version 5.0 (February 2005)
**Selected groups** (core/matrix sim):	• ALL user_defined.lib (0.75/Optimized)<br>• ALL vertebrates.lib (0.75/Optimized)

Aligned Sequences:

No.	Sequence Name	Sequence Description
1	P884387	P884387 sym=HSPC051|loc=Loc29796|taxid=9606|spec=Homo sapiens|chr=22|ctg=NT_011520|str=(+)|start=9553427|end=9554427|len=1001|tss=501|comm=ubiquinol-cytochr[illegible] c reductase complex (7.2 kD); (NM_013387/661/bronze;)
2	P884388	P884388 sym=1110020P15Rik|loc=Loc66152|taxid=10090|spec=Mus musculus|chr=11|ctg=NT_039515|str=(-)|start=1598616|end=1599628|len=1013|tss=501,505,508,513|comm=RIK[illegible] cDNA 1110020P15 gene; (AK003881/658/gold;AK002227/665/gold;AK012489/662/gold;NM_197979/670/bronze;)
3	rn3_dna	rn3_dna range=chr14:85372862-85373441 5'pad=0 3'pad=0 revComp=TRUE strand=? repeatMasking=none

Alignment (DiAlign format):

Please note that only **upper-case** letters are considered to be **aligned**. For more information, please have a look at the user guide.

V$GATA V$XBBF V$OCT1 V$NRF1 U$NRF1 V$ZBPF V$PAX5 V$RORA V$EREF V$HNF4 U$EREF V$SF1F
V$COUP V$SP1F

```
alignment position 1 . . . . . . . . .   1 1 . . . . . . . .   2 1 . . . . . . . .   3 1 . . . . . . . .   4 1 . . . . . . . .
P884387          1 c a c a g a g a a g   g a a g t g t t c a   a g t c t t c c a g   t g c g g a - - - -   - - - - - - - - - -
P884388          1 g g t t c c c t g c   c t c t g t a t t c   a c t c c c c a t g   c c a g a a a a a g   c g g a t a g c g c
rn3_dna          1 - - - - - - - - - -   - - - - - - - - - -   - - - - - - - - - -   - - - - - - - - - -   - - - - - - - - - -
```

```
alignment position 51........ 61........ 71........ 81........ 91........
P884387         37 ---------- ---------- ---------- ----GAAAAG AGACTAGGAC
P884388         51 ggacttagaa tgaaatgcct actcttctga ttggGAAAAG AGCCTAAAAC
rn3_dna          1 ---------- ---------- ---------- ---------- ----------
                                                      ****** **********

alignment position 101....... 111....... 121....... 131....... 141.......
P884387         53 TCGCCCCTCG ACGTCTCGCG GAAGgtacct ggctccccgg tggctgcagc
P884388        101 TTGGACGCGG ATGTCTCACC AAAGatacct ggt------- ----------
rn3_dna          1 ---------- ---------- ---------- ---------- ----------
                   ********** ********** ****

alignment position 151....... 161....... 171....... 181....... 191.......
P884387        103 TCCGGGCTCC TCCTCCTGCG TCCTCGCGTC GCGTACTGCT TGCTGGGATT
P884388        134 TCCGGGCTCC TCCTCTTGAG TCCTCGCGTT GCGCTCTGCT CGCTGGGATT
rn3_dna          1 ---------- ------TGTG TCCTCGTGTC GCGCTCTGCT CGCTGGGATT
                   ********** ********** ********** ********** **********
                   ********** ********** ********** ********** **********
                   ********** ********** ********** ********** **********
                                    **** ********** ********** **********
                                    **** ********** ********** **********
                                       * ********** ***  ***** **********
                                       * ********** ***

alignment position 201....... 211....... 221....... 231....... 241.......
P884387        153 CGTAGTCtcg ccttggccat cagttcgccc ttcccagaga gctttGGGAA
P884388        184 TGTAGTCCTT CCTAAGGTAC CGAGAAGGC- ---------- -----GGAAT
rn3_dna         35 CGTAGTCCTT CCTAAGGTAC CGAGAAAGC- ---------- -----GGAAT
                   ********** ********** *********                 *****
                   ********** ********** *********                 *****
                   ********** ********** *********                 *****
                   *******
                    ******
                    ******
                    ******

alignment position 251....... 261....... 271....... 281....... 291.......
P884387        203 TCTAAGCCGG GACTGGGCGG AGGGCGGGGA GAAGGtgatg gcgcgcgaGG
P884388        218 ACGAAGGCGA GACAGCACTG GGGAAGATGG GTAAGCtAAG --------GG
rn3_dna         69 GCTAAGGCAA GACAGCGCTG GGGAAGATGG GTAAGCcAAG --------GG
                   ********** ********** ********** ****** ***         **
                      ******* ********** ********** ****** ***         **
                      ******* ********** ********** ****** ***         **
                                                           ***         **
                                                                       **
                                                                       **

alignment position 301....... 311....... 321....... 331....... 341.......
P884387        253 GCTTCTGGGA GTTGTAGTTT GACATATTCT AACAgccagg aattctgGAG
P884388        260 GCTTTCGGGA CTTGTAGTTC AAAATAGCCA AACgcaggAA ATGGTT-GAG
rn3_dna        111 GCTTTCGGGA GTTGTAGTTC AAAATAGTCA AACAcaatAA ACGGTT-GAG
                   ********** ********** ********** ****    ** ****** ***
                   ********** ********** ********** ****    ** ****** ***
                   ********** ********** ********** ***     ** ****** ***
                   ********** ********** ********** ***               ***
                   ********** ********** ********** ***               ***
                   ********** ********** ********** ***               ***

alignment position 351....... 361....... 371....... 381....... 391.......
P884387        303 AAGAAAAATT GGGTAATAAA TCTTATCAGA TTatctggg- ----------
P884388        309 GAGAAAAACT GAGAAATAAG TCTTATCAAA TTTTGGTTGT GTGGAATATG
rn3_dna        160 GAGAAAAACT GGAAAATAAA TCTTATCAAa cTTTAGCTGT CTGGAATATG
                   ********** ********** ********** ********** **********
                   ********** ********** ********** ********** **********
                   ********** ********** *********   ********* **********
                   ********** ********** *********   *
                   ********** ********** *********
                   ********** ********** ********
```

```
alignment position 401....... 411....... 421....... 431....... 441.......
P884387        342 ---------- ----CTGTCC TGATATGAAC CTTTTGAGTT TTTTTcctca
P884388        359 AACATTGTAA TAAACTGTTA CCCTGTGCAC ATTATCAGTT TCTTTAA---
rn3_dna        210 GACATTGTAA TAATCTGTTA TCCTGTGCAC ATTGTCAGTT TCTTTAA---
                   ********** ********** ********** ********** *******
                   ********** **********  ********* ********** *******
                   ********** **********  ********* ********** *******
                                          ********* ********** *******

alignment position 451....... 461....... 471....... 481....... 491.......
P884387        378 ttcccaggat aggaatacct ctgTGGTTCA AAAC-ACGAa ggCTGAGATT
P884388        406 ---------- ---------- ---TGGTTCA AAAtgTCGAC T-CTGAGATA
rn3_dna        257 ---------- ---------- ---TGGTTCA AAAC-TCGAC T-CTGAGATA
                                             ******* **** ***** * ********
                                             ******* ***  ***** * ********
                                             ******* ***  ***** * ********
                                             ******* ***  ****      ********
                                                                    ********
                                                                    ********
                                                                    ********

alignment position 501....... 511....... 521....... 531....... 541.......
P884387        427 CAGAGGAACT ACAAATCCCA TAAAGtgttg cacgtctagg gccttggggg
P884388        432 CAGAGAAACT ACAATTCCCA TAAAGCAGGG CGCGTTGAAA CTTTTGGAGc
rn3_dna        282 CCGAGAAACT ACAATTCCTA TAAAGCAGTG CGCGTTGAAA CTTTTGGAGt
                   ********** ********** ********** ********** *********
                   ********** ********** ********** ********** *********
                   ********** ********** ********** ********** *********
                   ********** ********** *****
                   ********** ******** * *****
                   ********** ******** * *****
                   ********** ********

alignment position 551....... 561....... 571....... 581....... 591.......
P884387        477 aacGAGGTCG GCCCCAGCGC AGGCGCGGTG GCGCGAGTTG GACTGTGAAG
P884388        482 aA-GACATTG GGTTCTACGC AAGCGCAGTG GCGCGAGTTC GGCAGTGAGG
rn3_dna        332 gA-GAAGTTG GTCGCTACGC AGGCGCAGTG ACGCCAGTTC GTCAGCGAGG
                    * ******* ********** ********** ********** **********
                    * ******* ********** ********** ********** **********
                      ******* ********** ********** ********** **********
                                     *** ********** ********** *  ********
                                     *** ********** ********** *  ********
                                                                 *

alignment position 601....... 611....... 621....... 631....... 641.......
P884387        527 AAACATGGCG GCCGCGACGT TGACTTCGAA ATTGTACTCC CTGCTGTTCC
P884388        531 CAACATGTCG TCGCCGACGA TCCCTTCGCG CCTGTACTCC TTGCTGTTCC
rn3_dna        381 CAACATGGCG TCGCCGACGG TCACTTCGCG TTTGTACTCC TTGCTGTTCC
                   ********** ********** ********** ********** **********
                   ********** ********** ********** ********** **********
                   ********** ********** ********** ********** **********
                   ********** ********** **********   ******** **********
                   *******        ****** **********   ******** **********
                    ******        ****** **********   ********  *********
                                                                *********
                                                                *********

alignment position 651....... 661....... 671....... 681....... 691.......
P884387        577 GCAGGACCTC CACCTTCGCC CTCACCATCA TCGTGGGCGt caTGTTCTTC
P884388        581 GCAGAACTTC CACCTTTGCC CTCACCATCG CAGTGGGCGC CCTGTTCTTC
rn3_dna        431 GCAGGACTTC TACCTTCGCC CTCACCATCG CAGTGAGCGC CCTGTTCTTC
                   ********** ********** ********** ********** **********
                   ********** ********** ********** ********** **********
                   ********** ********** ********** ********** **********
                   ********** ********** ********** ********** **********
                   ********** ********** ********** *********    ********
                   ********** ****** *** ********** *********    ********
                   **** ***** ****** *** *********               ********
                   **** ***** ****** *** *********               ********
```

```
                                      ***  *********              ********
alignment position  701.......  711.......  721.......  731.......  741.......
P884387        627  GAGCGCGCCT  TCGATCAAGG  CGCGGACGCt  ATCTACGACC  ACATCAACGA
P884388        631  GAGCGAGCCT  TCGATCAGGG  CGCAGACGCG  ATCTACGAGC  ACATCAACGA
rn3_dna        481  GAGCGCGCCT  TCGATCAGGG  TGCAGACGCG  GTCTACGATT  ACATCAACGA
                    **********  **********  **********  **********  **********
                    **********  **********  **********  **********  **********
                    **********  **********  **********  **********  **********
                    **********  **********  *********   **********  **********
                    **********  **********  *********   **********  **********
                    **********  **********  *********   **********  **********
                    ***** ****  **********  *********    *********  **********
                    ***** ****  **********  *********    *******    **********
                    *****                                *******

alignment position  751.......  761.......  771.......  781.......  791.......
P884387        677  GGGGGTGAGG  GCCTGTGCCA  Tc CCTGACCT TGGACCCGCC  TGAGGGGTGA
P884388        681  GGGGGTGAGG  GCCTGGGCCA  T- CCTGACCT TGGGCCCGCC  TGAAGGGTGA
rn3_dna        531  GGGGGTGAGG  GACTGGGCCA  T- CCTGACCT TGGGCCCGCC  TGAAGGGTGA
                    **********  **********  * ********  **********  **********
                    **********  **********  * ********  **********  **********
                    **********  **********  * ********  **********  **********
                    **********  **********  * ********  **********  **********
                    **********  **********  * ********  **********  **********
                    **********  **********  * ********  **********  **********
                    **********  **********  * ********  **********  **********
                    **********  * ********    ********  **********  **********
                                  ********
                                  ********

alignment position  801.......  811.......  821.......  831.......  841.......
P884387        727  Cagtggagta  GAGGTGGGAT  GGGACTCAGT  ATACTTTTAC  CAGGAAGGGG
P884388        730  Ctgggagg--  GAGGTAGGAC  GGAACTCAAT  CAAGTCTTAC  CAGGTCTGGG
rn3_dna        580  C---------  ----------  ----------  ----------  ----------
                    *           **********  **********  **********  **********
                    *
                    *
                    *
                    *
                    *
                    *
                    *

alignment position  851.......  861.......  871.......  881.......  891.......
P884387        777  gtaaggggta  aaccgtcggc  gtcttctgtc  tcgagtccgc  cgtctgTCCC
P884388        778  gtgaactgtg  cccgttaata  catctat---  ----------  ------TTCT
rn3_dna        581  ----------  ----------  ----------  ----------  ----------
                                                                          ****

alignment position  901.......  911.......  921.......  931.......  941.......
P884387        827  CTTGGCTTGA  TGTGAATTCT  AAaacgttaa  gcgaggttaa  tatatttcat
P884388        809  CTTGACATGG  TGCGAGGCCT  AAttggtatg  tcctgtc---  ----------
rn3_dna        581  ----------  ----------  ----------  ----------  ----------
                    **********  **********  **

alignment position  951.......  961.......  971.......  981.......  991.......
P884387        877  tttgcagggg  ttgtaagtat  tgctgtgtgc  caccctacat  ACCCACTTGC
P884388        846  ----------  ----------  ----------  ----------  ACACAGTTCC
rn3_dna        581  ----------  ----------  ----------  ----------  ----------
                                                                    **********

alignment position  1001......  1011......  1021......  1031......  1041......
P884387        927  CCATTATCAG  AAGCAAAAAC  TAAaggctag  t------GAG  AGCAAAGGAC
P884388        856  ACATATTCAT  TTGCGAAAGC  TAActcctga  ctcctgaGAC  ATCAGAGGAC
rn3_dna        581  ----------  ----------  ----------  ----------  ----------
                    **********  **********  ***                ***  **********

alignment position  1051......  1061......  1071......  1081......  1091......
```

```
P884387      971 TTACtagctg cacaggaagc caggggcaag t--------- ----------
P884388      906 TTACgggtct gaaccactcc attaagtcgg tggtgagttg gaactccgac
rn3_dna      581 ---------- ---------- ---------- ---------- ----------
                 ****

alignment position 1101...... 1111...... 1121...... 1131...... 1141......
P884387     1002 ---------- ---------- ---------- ---------- ----------
P884388      956 ccctgccgtc cttgctagtt tcaagggcag atttcagcaa ggcagtcgtt
rn3_dna      581 ---------- ---------- ---------- ---------- ----------

alignment position 1151....
P884387     1002 --------
P884388     1006 aaaagtag
rn3_dna      581 --------
```

Pairwise similarities:

For each pairwise alignment, the similarity (relative to the maximum similarity) and the number of identical nucleic acids (in % of shorter sequence) is given. Maximum values are underlined.

	P884388 (1013 bp)	rn3_dna (580 bp)
**P884387 (1001 bp)**	0.511<br>48 %	0.478<br>63 %
**P884388 (1013 bp)**		1.000<br>89 %

Please note that the **similarity value 1.000** marks only the two **most similar** sequences, it does not necessarily mean that these sequences are identical.

**Extract alignment region**	
**Positions in alignment**	from: to:
**Output file**	dialign.seq

Extract aligned sequences

For comments, questions or bug reports, please contact support@genomatix.de

© Genomatix Software GmbH 1998-2005 - All rights reserved. License Agreement GEMS Launcher 4.1.1

Genomatix-Logo Logout Personal Messages GEMS-Logo
Logout Personal Messages

Genomatix_MainGEMSElDoradoGene2PromoterBiblioSphere
Frequently_asked_questionsYour_ResultsYour_SequencesYour_ProtocolHelp

GEMS Launcher Task: *DiAlign TF*: Multiple alignment plus TF sites
working on C3-UQCR (3 seq.)

[Alignment] [Pairwise_similarities]

DiAlign professional TF Release 3.1 December 2004 **Wed Jun 8 22:07:49 2005**

Solution parameters:

**Sequence file:**	C3-UQCR (3 seq.)
**Type of sequences:**	nucleotide sequences
**Threshold T:**	0.00
**'*' signs below alignment denote:**	diagonal similarity (max. similarity is represented by 10 '*' signs)
**Alignment output:**	complete alignment is shown<br># nucleic acids per line: 50
**TF output:**	common TF matches located in aligned regions (common to 3 (100.0 %) sequences)
**Family matches:**	yes
**MatInspector library:**	Matrix Family Library Version 5.0 (February 2005)
**Selected groups** (core/matrix sim):	• ALL user_defined.lib (0.75/Optimized)<br>• ALL vertebrates.lib (0.75/Optimized)

Aligned Sequences:

No.	Sequence Name	Sequence Description
1	P936092	P936092 sym=UQCR|loc=Loc10975|taxid=9606|spec=Homo sapiens|chr=19|ctg=NT_011255|str=(-)|start=1544931|end=1545931|len=1001|tss=501|comm=ubiquinol-cytochror reductase (6.4kD) subunit; (NM_006830/560/silver;)
2	P936093	P936093 sym=Uqcr|loc=Loc66594|taxid=10090|spec=Mus musculus|chr=10|ctg=NT_039496|str=(-)|start=6269155|end=6270219|len=1065|tss=501,531,565|comm=ubiquin c reductase (6.4kD) subunit; (AK003443/531/gold;AK013513/565/gold;NM_025650/501/bronze;)
3	rUQCR	rUQCR sym=Uqcr|spec=RATUS|chr=7|str=(+)|tss=343

Alignment (DiAlign format):

Please note that only **upper-case** letters are considered to be **aligned**. For more information, please have a look at the user guide.

V$ETSF U$NRF2 V$NRF1 U$NRF1

```
alignment position 1 . . . . . . . . .  1 1 . . . . . . . .  2 1 . . . . . . . .  3 1 . . . . . . . .  4 1 . . . . . . . .
P936092          1 c t g c a a a g a g  a t t t c c g a c a  g g g c c t c c a a  g c a a c g g g c a  a c g c t g a g g c
P936093          1 - - - - - - - - - -  - - - - - - - - - -  - - - - - - - - - -  - - - - - - - - - -  - - - - - - - - - -
rUQCR            1 - - - - - - - - - -  - - - - - - - - - -  - - - - - - - - - -  - - - - - - - - - -  - - - - - - - - - -

alignment position 5 1 . . . . . . . .  6 1 . . . . . . . .  7 1 . . . . . . . .  8 1 . . . . . . . .  9 1 . . . . . . . .
P936092         51 t c a g g g a a g c  a c t g c c g c t c  g a c c a a a g t c  c t a c a G C G A A  T C A G G A A C A A
P936093          1 - - - - - - - - - -  - - - - - - - - - -  - - - - - - - - - -  - - - - - G C G A C  T C A C C G A C A A
```

```
rUQCR                 1 ---------- ---------- ---------- ---------- ----------
                                                                ***** **********

alignment position      101....... 111....... 121....... 131....... 141.......
P936092             101 CGCCTGACGC CTCCAAAAGT ---------- ---------- ----------
P936093              16 CTTCAAATGT ATTCAAAGGT gtggcccaac tcgagccttt ccccttcagg
rUQCR                 1 ---------- ---------- ---------- ---------- ----------
                        ********** **********

alignment position      151....... 161....... 171....... 181....... 191.......
P936092             121 ---------- ---------- ---------- ---------- ----------
P936093              66 gtctgtgctc aagttcctct ggtttaaatt gatcaaagat agggctgtgg
rUQCR                 1 ---------- ---------- ---------- ---------- ----------

alignment position      201....... 211....... 221....... 231....... 241.......
P936092             121 ---------- ---------- ---------- ---------- GGGCTCCAAT
P936093             116 ctcagtggga cgaacgctcc tctagcgtgt ccaaggctct GGGGTCCAGT
rUQCR                 1 ---------- ---------- ---------- ---------- ----------
                                                                    **********

alignment position      251....... 261....... 271....... 281....... 291.......
P936092             131 CCCAGCTCTT CCCCTCTgtg t--------- ---------- ----------
P936093             166 CCCAGCACTC CCTGTCTacc ccAGGCTTAC ACTTTAGGAG GTGGAGGCAG
rUQCR                 1 ---------- ---------- --AGGCTTAC ACTTTAGGAG GTGGAGGCAG
                        ********** *******      ******** ********** **********
                                                ******** ********** **********
                                                ******** ********** **********
                                                ******** ********** **********
                                                ******** ********** **********

alignment position      301....... 311....... 321....... 331....... 341.......
P936092             152 GACTCTGAGC AAgacccttc cccgctggac ctcagtttcc ttcattgtaa
P936093             216 GAGTCTGAGG AAttcacGGC CAGCCCTGAA TATAGGAGAC CCCCATATCA
rUQCR                29 GAGCGTGAGG AAatcaaGGC CAGCCCTGGA TATACGAGAC CCCCGTCTCG
                        ********** **       *** ********** ********** **********
                        ********** **       *** ********** ********** **********
                        ********** **       *** ********** ********** **********
                        ********** **       *** ********** ********** **********
                        ********** **

alignment position      351....... 361....... 371....... 381....... 391.......
P936092             202 ataacgttct ggcaca---- ---------- ---------- ----TACTAG
P936093             266 AAAACACcta agtATGTGTA TTATTAAAGT TATTATcagA TTAGTACTAC
rUQCR                79 AAAACACca- ---ATGTGTA TTATTAAAGT TATTAT---A TTAGTACTAG
                        *******       ******* ********** ******   * **********
                        *******       ******* ********** ******         ******
                        *******       ******* ********** ******
                        *******

alignment position      401....... 411....... 421....... 431....... 441.......
P936092             224 GCGCACAAAA TCACTGGcag gtagccccgc ggagcccaga agcacaatcg
P936093             316 GTGGTCAAAG TCACa----- ---------- ---------- ----------
rUQCR               122 GTGCTCAGAG TCCCGGG--- ---------- ---------- ----------
                        ********** *******
                        ********** ****

alignment position      451....... 461....... 471....... 481....... 491.......
P936092             274 gccgccagac cacgtggcca gaaagcaccg ccctaacgcC GCCCACTCCG
P936093             331 ---------- ---------- ---------- ---------C ACACTCTCCC
rUQCR               139 ---------- ---------- ---------- ---------- ----------
                                                                 * **********

alignment position      501....... 511....... 521....... 531....... 541.......
P936092             324 GACCACTTCT CCGCGGTcgt ccaa------ ---------- ----------
P936093             342 CACCCCATCC CCGGTGTtcc agtGTGCATG GAGCTACTGC TGGGATCCAC
rUQCR               139 ---------- ---------- ---GTGCATG GAGCTACCGC CGGGATCCAC
```

```
                       **********  *******             *******  **********  **********
                                                       *******  **********  **********
                                                       *******  **********  **********
                                                       *******  **********  **********

alignment  position    551.......  561.......  571.......  581.......  591.......
P936092         348    ----------  ----------  ----------  ----------  ----------
P936093         392    TGCAACC---  ----------  ----------  ----------  ----------
rUQCR           166    TGCAACCtca  ggcacagaat  ctcagagcgc  ccagccggct  agaagttccg
                       *******
                       *******
                       *******
                       *******

alignment  position    601.......  611.......  621.......  631.......  641.......
P936092         348    ----------  ----------  ----------  ----------  --CCCTCGCC
P936093         399    --ACAGAACC  AGGACAGAAG  CGGATC-AGT  AACGTCTTTC  TCTCCTCCCC
rUQCR           216    ccACTGAAGC  AAGACAGAGG  CGGGTCtAGT  AAGGTCTGTC  TCCCCTCCCC
                         ********  **********  ******  ***  **********  **********
                                                       ***  **********  **********
                                                       ***  **********  **********
                                                       ***  **********  **********
                                                                           *******

alignment  position    651.......  661.......  671.......  681.......  691.......
P936092         356    GGAAGTTACT  GTAAGTGGCT  TCCGGGTCtg  cgcgcacagt  ggcggatgat
P936093         446    GGAAGTAGAT  GTCAATGTCT  TCGGCGCCAC  CCCCGGAAAA  GGTGGAGCga
rUQCR           266    GGAAGAAGAT  GTCAACGTCC  TCTGTGCCAC  CACCGGAAGA  GGTGGTGCcg
                       **********  **********  **********  **********  ********
                       **********  **********  **********  **********  ********
                       **********  *****  ****  ********
                       **********  *****
                       **********  *****

alignment  position    701.......  711.......  721.......  731.......  741.......
P936092         406    gttttttttt  cctccccccc  ccccgccct  cCCCGCGCGC  GTGCGCGCCG
P936093         496    tcg-------  ----------  ----------  -CCTTCACGC  ATGCGCTATA
rUQCR           316    ttgct-----  ----------  ----------  ---TTCACGC  ATGCGCTGTA
                                                             *********  **********
                                                                ******  **********
                                                                ******  **********
                                                                ******  **********
                                                                ******  **********
                                                                ******  **********
                                                                  ****  **********

alignment  position    751.......  761.......  771.......  781.......  791.......
P936092         456    CGGCCGCGCC  TGCGCAGTGt  agCCGGGTCA  GCTGGACAGG  GTCATCCTGA
P936093         518    CGTCTGCACA  TGCGTAGTGC  T-CCAGGGCA  GCGGAACGGG  GTGACCCTGA
rUQCR           338    CGTCTGCACA  TGCGTAGTGC  T-CCAGGGCG  GCGGAACAGG  GTGACCCTGA
                       **********  **********  *  ********  **********  **********
                       **********  **********  *  ********  **********  **********
                       **********  **********  *  ********  **********  **********
                       **********  **********  *  ********  **********  **********
                       **********  **********  *  ********  **********  **********
                       **********  **********  *  ********  **********  **********
                       **********  *********      **

alignment  position    801.......  811.......  821.......  831.......  841.......
P936092         506    Ggg-TGCGAC  TCCGCCGCGA  TGGTGACCCG  GTTCCTGGGC  CCACGCTACC
P936093         567    GTATTGAGAC  CCTGCAGCGA  TGCTGAGCAG  GTTTCTAGGC  CCGCGCTACC
rUQCR           387    GTATTGAGAC  CCCGCAGCGA  TGCTCAGCAG  ATTTCTAGGC  CCGCGCTACC
                       **********  **********  **********  **********  **********
                       **********  **********  **********  **********  **********
                       **********  **  *******  **********  **********  **********
                       **********  **  *******  **********  **********  **********
                       **********  **  *******  **********  **********  **********
                       *    ******  **  *******  **********  **********  **********
                             *****  **  *******  **********  **********  **********
                                                                   ***  **********
```

```
                                                             ***  **  *******
                                                                      *******
alignment position 851....... 861....... 871....... 881....... 891.......
P936092        555 GGGAGCTGGT CAAGAACTGG TGAGGACGCC Gacccgcggc cccatccgcg
P936093        617 GGGAACTGGC CAGAAACTGG TGAGGAGGTC ACCCGCCatt cca-----CC
rUQCR          437 GAGAACTGGC CAAAAACTGG TGAGGAGGCC GCCCGCCcgc cattccacCC
                   ********** ********** ********** *******            **
                   ********** ********** ********** *******            **
                   ********** ********** ********** *******            **
                   ********** ********** ********** *******
                   ********** ********** ********** *
                   ********** ********** ********** *
                   * ******** ********** ******
                   * ******** ********** ******
                   * ******** ********** ******
                   *
alignment position 901....... 911....... 921....... 931....... 941.......
P936092        605 ctcccgcctc ggcccagccc cgcgccgctg cgttccgtat ttgtgcgcgt
P936093        662 GTGTGCTCTG GGACT----- ---------- ---------- ----------
rUQCR          487 GTGTGCTCTG GGACT----- ---------- ---------- ----------
                   ********** ******
                   ********** ******
                   ********** ******
alignment position 951....... 961....... 971....... 981....... 991.......
P936092        655 ttccgggggc cgggccgggc cgggcccagg tcccttgttg ttcccggtga
P936093        678 ---------- ---------- ---------- ---------- ----------
rUQCR          503 ---------- ---------- ---------- ---------- ----------

alignment position 1001...... 1011...... 1021...... 1031...... 1041......
P936092        705 cagaGGGGGA AACTGAGGCc gagaggcgga gcggcgcgtc ccggcgtgaa
P936093        678 ----GGGGAA ACCTGAGGCT GTAACAGGCA GAGTATTgc- GGGACACAGT
rUQCR          503 ----GGGGGA AACTGAGGCT GTGACAGGCA AAGTATTcgg GGGACACAGT
                       ****** ********** ********** *******    **********
                       ****** ********** ********** *******    **********
                       **** * *********                        **********
                       **** * *********                        **********
alignment position 1051...... 1061...... 1071...... 1081...... 1091......
P936092        755 cgGGACGTGA CGGACCCAAG GCCAAACccc agcatgcccg gctccgcggc
P936093        723 --GCAGGTGG AAGCTCCGAG GTtaagCTGC AATCGCCTTC GTTCGGTGTC
rUQCR          549 --GCAGGTGG AAGATCCGAG GTCCAACTGT AATCACCTTC GTTCAGTGTC
                     ******** ********** ********** ********** **********
                     ******** ********** **    **** ********** **********
                     ******** ********** **    **** ********** **********
                     ******** ********** **    **** ********** **********
alignment position 1101...... 1111...... 1121...... 1131...... 1141......
P936092        805 tagGCCGCTC CAAGCCTCAG TTTCCCCATC TCTACCGGGG GATCCgaatg
P936093        771 TGAGGTGCTC CAAGCCTGGG TTTCCCCAGC TTGACCTGGG TAGCCTTGTG
rUQCR          597 TGAGCTGCTC CGAGCCTCAG TTTCCCCAGC CTGACCTGGG TAGCCTTGTG
                   ********** ********** ********** ********** **********
                   ********** ********** ********** ********** **********
                   ********** ********** ********** ********** **********
                   ********** ********** ********** ********** **********
                      ******* ******   * ********** ********** *****
                      ******* ******   * ********** ********** *****
                         **** ******   * ********** **********
                                       * ********** **********
alignment position 1151...... 1161...... 1171...... 1181...... 1191......
P936092        855 gtgaccccct ggacggaa-- --GCGCCCAG AAGGTGCCGA GTCCCGATCC
P936093        821 tgcggctctg gGTGGGTACC CTGGACTCCT ACGGCTCGGA ACTCCGACgt
rUQCR          647 cggccccga- -GTGGGTACC CTGGGCTCCT ACGGCTCGGA ACTCCGACTT
```

```
                                ********* ********** ********** **********
                                ********* ********** ********** ********
                                ********* ********** ********** ********
                                ********* ********** ********** ********
                                ********* ********** ********** ********

alignment position  1201...... 1211...... 1221...... 1231...... 1241......
P936092         901 CCAGCCCAGC Actcgcggca tcttcggcct ttGCCACTAT TTTGGTTTTT
P936093         871 gCAGCCTGGC GACTT-GCA- ---------- --GCCTTTGG CTTTGATTTT
rUQCR           695 TCAGCCTAGC AACTTgGCG- ---------- --GCCGTTGG CTTTGATTTT
                    ********** ***** ***               ******** **********
                                     ***               ******** **********
                                     ***               ******** **********

alignment position  1251...... 1261...... 1271...... 1281...... 1291......
P936092         951 ATGATTTTTA Acaaggagcg tgaaagcttc agctgcgcct gagcccacgt
P936093         907 GCAGTCTTTC GTGTACTTcg ttttttcgtt taaacag-TG TTTTTGTAAc
rUQCR           732 GCGGTCTTTA ATGTCCTTta aaaaaaaaaa aaaaaacctG TTTCTGTAAt
                    ********** ********                      ** *********
                    ********** ********
                    ********** ********

alignment position  1301...... 1311...... 1321...... 1331...... 1341......
P936092        1001 g--------- ---------- ---------- ---------- ----------
P936093         956 cCCAAGTTGG TCTGGAACTC CCACCAATCA TCCTGCCTCa ca---CTTCT
rUQCR           782 -CCAAGTTGG TCTGGAACTC CCACCAATCA TCCTGCCTCc tcacgCTTGT
                     ********* ********** ********** *********       *****
                     ********* ********** ********** *********       *****
                     ********* ********** ********** *********       *****
                     ********* ********** ********** *********       *****
                     ********* ********** ********** *********
                     ********* ********** ********** *********

alignment position  1351...... 1361...... 1371...... 1381...... 1391......
P936092        1002 ---------- ---------- ---------- ---------- ----------
P936093        1003 TGAACGCTAG AATAGCGGGC ACCCACCCTC ACCTGaatgt tttctttgca
rUQCR           831 TGAACGGTGG AATAGCGGGC ACCCACCATC ACCTGa---- ----------
                    ********** ********** ********** *****
                    ********** ********** ********** *****
                    ********** ********** ********** *****
                    ********** ********** ********** *****

alignment position  1401...... 1411
P936092        1002 ---------- ---
P936093        1053 tgcgtgtctg tgt
rUQCR           867 ---------- ---
```

Pairwise similarities:

For each pairwise alignment, the similarity (relative to the maximum similarity) and the number of identical nucleic acids (in % of shorter sequence) is given. Maximum values are underlined.

	P936093 (1065 bp)	rUQCR (866 bp)
**P936092 (1001 bp)**	0.129<br>29 %	0.126<br>31 %
**P936093 (1065 bp)**		1.000<br>78 %

Please note that the **similarity value 1.000** marks only the two **most similar** sequences, it does not necessarily mean that these sequences are identical.

Extract alignment region

**Positions in alignment**	from: to:
**Output file**	dialign.seq

Extract aligned sequences

For comments, questions or bug reports, please contact support@genomatix.de

© Genomatix Software GmbH 1998-2005 - All rights reserved.

License Agreement

GEMS Launcher 4.1.1

Genomatix-Logo | Logout | Personal | Messages | GEMS-Logo

Genomatix_MainGEMSElDoradoGene2PromoterBiblioSphere
Frequently_asked_questionsYour_ResultsYour_SequencesYour_ProtocolHelp

GEMS Launcher Task: *DiAlign TF*: Multiple alignment plus TF sites
working on promoters.seq (3 seq.)

[Alignment] [Pairwise_similarities]

DiAlign professional TF Release 3.1 December 2004 **Fri Apr 29 23:46:55 2005**

Solution parameters:

**Sequence file:**	promoters.seq (3 seq.)
**Type of sequences:**	nucleotide sequences
**Threshold T:**	0.00
**'*' signs below alignment denote**:	diagonal similarity (max. similarity is represented by 10 '*' signs)
**Alignment output:**	complete alignment is shown<br># nucleic acids per line: 50
**TF output:**	common TF matches located in aligned regions (common to 3 (100.0 %) sequences)
**Family matches:**	yes
**MatInspector library:**	Matrix Family Library Version 5.0 (February 2005)
**Selected groups** (core/matrix sim):	• ALL user_defined.lib (0.75/Optimized)<br>• ALL vertebrates.lib (0.75/Optimized)

Aligned Sequences:

No.	Sequence Name	Sequence Description	Length
1	P879713	sym=UQCRH|loc=Loc7388|taxid=9606|spec=Homo sapiens|chr=1| ctg=NT_032977|str=(+)|start=588249|end=588932|len=684| tss=552| comm=ubiquinol-cytochrome c reductase hinge protein; (NM_006004/552/silver;)	684 bp
2	P879718	sym=Uqcrh|loc=Loc66576|taxid=10090|spec=Mus musculus|chr=4| ctg=NT_039264|str=(-)|start=16198124|end=16198804|len=681| tss=501,514,515,518,537| comm=ubiquinol-cytochrome c reductase hinge protein; (AK054005/514/gold;AK019085/518/gold;AK088185/515/gold; AK012552/501/gold;AK003539/537/gold;AK003266/515/gold; NM_025641/501/silver;)	681 bp
3	P879720	sym=na|loc=Loc366448|taxid=10116|spec=Rattus norvegicus| chr=5|ctg=NW_047718|str=(+)|start=7693061|end=7693742| len=682|tss=582| comm=similar to Ubiquinol-cytochrome C reductase complex 11 kDa protein, mitochondrial precursor (Mitochondrial hinge protein) (Cytochrome C1, nonheme 11 kDa protein) (Complex III subunit VIII); (XM_345568/582/silver;)	682 bp

Alignment (DiAlign format):

Please note that only **upper-case** letters are considered to be **aligned**. For more information, please have a look at the user guide.

V$MAZF V$ZBPF V$RREB V$NRF1 U$NRF1 V$NOLF V$PLAG V$CREB V$HIFF V$HESF V$XBBF V$E4FF
V$ZF5F V$YY1F V$GLIF V$AP2F V$HOXH V$RORA V$CLOX V$ETSF U$NRF2 V$DMTF

alignment position 1 . . . . 11 . . . . 21 . . . . 31 . . . . 41 . . . .

```
P879713      1 --CAGGGCGG GGGGAGCGTT GGGGCCCGAG GCCGAGCTCT TCGCTGGCGC
P879718      1 aACAGGGCGG GGGGAGCGCT GGGGCCCGAG GCCGAGCTCT TCGCTGGCGC
P879720      1 -ACAGGGCGG GGGGAGCGCT GGGGCCCGAG GCCGAGCTCT TCGCTGGCGC
                *********  ********** ********** ********** **********
                *********  ********** ********** ********** **********
                *********  ********** ********** ********** **********
                *********  ********** ********** ********** **********
                 ********  ********** ********** ********** **********
                 ********  ********** ********** ********** **********
                 ********  ********** ********** ********** **********
                 ********  ********** ********** ********** **********
                 ********  ********** ********** ********** **********
                 ********  ********** ********** ********** **********

alignment
position       51........ 61........ 71........ 81........ 91........

P879713     49 CGCCTCCCGG GACGTGGCCT CCATGGTCGT TGccGCCGCT ACCTCACAGA
P879718     51 CGCCTCCCGG GACGTGGCCT CCATGGTCGT TGCTGCCGCT ACCTCACAGA
P879720     50 CGCCTCCCGG GACGTGGCCT CCATGGTCGT TGCTGCCGCT ACCTCACAGA
               ********** ********** ********** ********** **********
               ********** ********** ********** ********** **********
               ********** ********** ********** ********** **********
               ********** ********** ********** *** ****** **********
               ********** ********** ********** **  ****** **********
               ********** ********** ********** **  ****** **********
               ********** ********** ********** **  ****** **********
               ********** ********** ********** **  ****** **********
               ********** ********** ********** **  ****** **********
               ********** ********** ********** **  ****** **********

alignment
position       101....... 111....... 121....... 131....... 141.......

P879713     99 ACCAGCAACT CCGGGCGCGC CAGGCCTCGG GCGCCGCCAT CTTGGGGAGG
P879718    101 ACCAGCAACT CCGGGCGCGC CAGGCCTCGG GCGCCGCCAT CTTGGGGAGG
P879720    100 ACCAGCAACT CCGGGCGCGC CAGGCCTCGG GCGCCGCCAT CTTGGGGAGG
               ********** ********** ********** ********** **********
               ********** ********** ********** ********** **********
               ********** ********** ********** ********** **********
               ********** ********** ********** ********** **********
               ********** ********** ********** ********** **********
               ********** ********** ********** ********** **********
               ********** ********** ********** ********** **********
               ********** ********** ********** ********** **********
               ********** ********** ********** ********** **********
               ********** ********** ********** ********** **********

alignment
position       151....... 161....... 171....... 181....... 191.......

P879713    149 TGCGCGAGCC CGAGagtgtc gCCCGCGGAC CGCCATCTTG AAAAGGTCAG
P879718    151 TGCGCGAGCC CGAGAATGAC ACCCGCGGGC CGCCATCTTG AAGAGGTCAG
P879720    150 TGCGCGAGCC CGAGAATGAC ACCCGCAGAC CGCCATCTTG AAGAGGTCAG
               ********** ********** ********** ********** **********
               ********** ********** ********** ********** **********
               ********** ********** ********** ********** **********
               ********** ****        ********* ********** **********
               ********** ****        ********* ********** **********
               ********** ****        ********* ********** **********
               ********** ****
               ********** ****
               ********** ****
               ********** ****

alignment
position       201....... 211....... 221....... 231....... 241.......

P879713    199 CagTTAGGAC GGCTCCATAA GCGATATGGG GAAGAAtgtG AATTATTCTA
P879718    201 CTTTTAGGAC CGCTCCATAA GGGATATGGG GAAGAACAGa AACAACATTA
P879720    200 CTTTTAGGGC CGCTCCATAA GTGATATGGG TAAGAACAGG AACTATATCA
```

```
                 **********  **********  **********  **********  **********
                 **********  **********  **********  *********   **********
                 **********  **********  **********  *********
                 *  *******  **********  **********  ******
                 *  *******  **********  **********  ******
                 *  *******  **********  **********  ******

alignment
position         251.......  261.......  271.......  281.......  291.......

P879713     249  AAGAAATTTC  GAAGAAATTA  GCACACTTTc  CAAGTCTCTT  AGGAGCTACT
P879718     251  AAAGATCGTC  AAACAGAGGA  GCAGATgact  CAAGTATCTT  AGGGAATACT
P879720     250  AAAGACTATC  AAACAAATTA  GCAGATATTt  tAAATATCTT  AGGAAACACT
                 **********  **********  *********   **********  **********
                 **********  **********  ******       *********  **********

alignment
position         301.......  311.......  321.......  331.......  341.......

P879713     299  ATTTTAGATA  AACTCCTAGA  TCAATtatac  ttgggtgtgg  tatgactaag
P879718     301  ACTTGGAAAA  ataCTGTAGA  TCAACACATT  TGGGCAAGAA  GTGACCGAAA
P879720     300  ACTTGAAAAA  CTTCTGTAGA  TCAATACGTT  TGGGCAGAAA  GTGACCAAAA
                 **********  **********  **********  **********  **********
                 **********     *******  **********  **********  **********

alignment
position         351.......  361.......  371.......  381.......  391.......

P879713     349  gGGATGTTTC  TTAGCAAAGC  CTCCTCGGGT  GCAAACATCA  GCTACCCCTA
P879718     351  -GGATGTTTC  TTAGCAAATT  CTTTGCAGGC  GTATATAGCA  GCTCCCCCTA
P879720     350  agg-TGCTTC  TTAGCAAATT  CTTTGCAGGC  ATATATACCA  GCTCCCCCTA
                  *********  **********  **********  **********  **********
                     ******  **********  **********  ******* **  **********
                     ******  **********  **********  ******* **  **********
                        ***  ********                        **  *******

alignment
position         401.......  411.......  421.......  431.......  441.......

P879713     399  acctctgcct  gcggctgGTA  GTACATGCCA  ATCTGAGCAT  GTGTTCGCGA
P879718     400  CGTCTCGTAG  CTAGCGAGTA  GTACAGCCCA  ATTTGTGCGT  GTGTTCGGGA
P879720     399  CGTCTTGCAG  CTAGCGAGTA  GTACAGGCCA  ATTTGTGGGT  GTGTTCGAGA
                 **********  **********  **********  **********  **********
                 **********  **********  **********  **********  **********
                 **********  **********  **********  **********  **********
                                    ***  ****** ***  **********  **********
                                    ***  ****** ***  **********  **********

alignment
position         451.......  461.......  471.......  481.......  491.......

P879713     449  CCACGATGAG  TGTGCCGCAC  TTCCGGCCAG  ATCGCCGGAT  TTCCGctGAG
P879718     450  CTACGAgGAG  TATGCCGCAC  TTCCGGCCAG  GTCGCCGGAT  ATCCGTCGAG
P879720     449  CTACGATGAG  TATGCCGCAC  TTCCGGCCAG  GTCGCCGGAT  ATCCGTCGAG
                 **********  **********  **********  **********  **********
                 **********  **********  **********  **********  **********
                 **********  **********  **********  **********  **********
                 ****** ***  **********  **********  **********  **********
                 ****** ***  **********  **********  **********  *****  ***
                      * ***  **********  **********  **********  *****  ***
                        ***  **********  **********  **********  *****  ***
                        ***  **********  **********  **********  *****

alignment
position         501.......  511.......  521.......  531.......  541.......

P879713     499  TGACCCTTAC  AAGTCCTTCT  TGATCCTGAA  CTGGgtTAGG  TGCCGCTGTT
P879718     500  TGACCCTTAC  TAGTCCTTCT  TAAACCTGAA  GTGGAGTact  a----CTCTG
P879720     499  TGACCCTTAC  AAGTCCTTCT  TAAACCTCAA  GTGGAGTAGG  TACCGTTGTT
                 **********  **********  **********  **********  **********
                 **********  **********  **********  **********  **********
                 **********  **********  **********  *******
                 **********  **********  **********  ****  *
                 **********  **********  **********  ****
                 **********  **********  **********  ****
                 **********  **********  **********  ****
```

Pairwise similarities:

For each pairwise alignment, the similarity (relative to the maximum similarity) and the number of identical nucleic acids (in %

of shorter sequence) is given. Maximum values are underlined.

	P879718 (681 bp)	P879720 (682 bp)
P879713 (684 bp)	0.649 67 %	0.648 69 %
P879718 (681 bp)		1.000 88 %

Please note that the **similarity value 1.000** marks only the two **most similar** sequences, it does not necessarily mean that these sequences are identical.

Extract alignment region	
**Positions in alignment**	from: to:
**Output file**	dialign.seq

Extract aligned sequences

For comments, questions or bug reports, please contact support@genomatix.de

© Genomatix Software GmbH 1998-2005 - All rights reserved. License Agreement GEMS Launcher 4.1.1

Genomatix-Logo Logout Personal Messages GEMS-Logo

Genomatix_MainGEMSElDoradoGene2PromoterBiblioSphere
Frequently_asked_questionsYour_ResultsYour_SequencesYour_ProtocolHelp

GEMS Launcher Task: *DiAlign TF*: Multiple alignment plus TF sites
working on van_waveren3_4.seq (3 seq.)

[Alignment] [Pairwise_similarities]

DiAlign professional TF Release 3.1 December 2004 **Tue May 3 00:08:58 2005**

Solution parameters:

**Sequence file:**	van_waveren3_4.seq (3 seq.)
**Type of sequences:**	nucleotide sequences
**Threshold T:**	0.00
**'*' signs below alignment denote:**	diagonal similarity (max. similarity is represented by 10 '*' signs)
**Alignment output:**	complete alignment is shown<br># nucleic acids per line: 50
**TF output:**	common TF matches located in aligned regions (common to 3 (100.0 %) sequences)
**Family matches:**	yes
**MatInspector library:**	Matrix Family Library Version 5.0 (February 2005)
**Selected groups** (core/matrix sim):	• ALL user_defined.lib (0.75/Optimized)<br>• ALL vertebrates.lib (0.75/Optimized)

Aligned Sequences:

No.	Sequence Name	Sequence Description
1	P884800	P884800 sym=QP-C|loc=Loc27089|taxid=9606|spec=Homo sapiens|chr=5|ctg=NT_034772|str=(+)|start=34616870|end=34617870|len=1001|tss=501|comm=low molecular ma ubiquinone-binding protein (9.5kD); (NM_014402/501/bronze;)
2	P884801	P884801 sym=1500040F11Rik|loc=Loc22272|taxid=10090|spec=Mus musculus|chr=11|ctg=NT_096135|str=(-)|start=18541060|end=18542096|len=1037|tss=501,514,515,517,537|comm cDNA 1500040F11 gene; (AK005374/515/gold;AK007982/537/gold;AK002389/517/gold;AK003160/501/gold;NM_025352/514/bronze;)
3	rQP-C	rQP-C rn3_dna range=chr10:38898600-38899450 5'pad=0 3'pad=0 revComp=TRUE strand=? repeatMasking=none

Alignment (DiAlign format):

Please note that only **upper-case** letters are considered to be **aligned**. For more information, please have a look at the user guide.

V$SP1F V$NFKB V$STAT V$ETSF V$CREB V$RBPF V$IKRS V$EBOX V$HESF

```
alignment position 1.........  11.........  21.........  31.........  41.........
P884800        1 tactggccaa ggccaaatga aacctc---- ---------- ----------
P884801        1 tatgcaaagg cctggtactg agcggaaagG ACCGCCTCTT GGAACACCCA
rQP-C          1 ---------- ---------- ---------G ACAGCCTCTT GGAACGGCCA
                                                 * ********** **********
                                                 * ********** **********

alignment position 51.........  61.........  71.........  81.........  91.........
```

```
P884800         27 -GTGCTCCTc tgctgtgggg cggcccggcc tcggggctca gaggcgctct
P884801         51 GGTGCTCCTA AGCCaCGCCC ATCACCCTAA TC-------- ----------
rQP-C           22 GAAGCTCCTA AGCCcCGCCC ATCATCCTAA TC-------- ----------
                   ********** **** ***** ********** **
                   ********** **** ***** ********** **
                                   ***** ********** **

alignment position 101....... 111....... 121....... 131....... 141.......
P884800         76 gtctacagct gggaagactc gCAGGCCCCA AGCTCCTGGC CAGCGCTGCC
P884801         83 ---------- ---------- -CGCGCCCCA TGCACCTATA -GGCCCCGCC
rQP-C           54 ---------- ---------- -CACGCCCCA TGCACCTATA AGGCACCGCC
                                           ********* ********** **********
                                           ********* **********
                                           ********* **********
                                           ********* **********

alignment position 151....... 161....... 171....... 181....... 191.......
P884800        126 CCGGAGgtcg gcaggcccct tcctcgtcac ctttttgttc cctcccccgc
P884801        111 CCGTGCACCT CTCGACgggG GACCTTACGG ACGTATCCAC CTAAGATCCC
rQP-C           83 CCTGCGCCCT CTAGACaa-G GACCTTACGA GCGTATCCAA CTAAGATCCC
                   ********** ******   * ********** ********** **********
                                       * ********** ********** **********
                                       * ********** ********** **********

alignment position 201....... 211....... 221....... 231....... 241.......
P884800        176 ctcccgcatt cggccgcttc ctgactggga ttccacagaa aagccgaggg
P884801        161 CAGGGtggct aaTAAGCCCC GCCCACTTGC ---------- ----------
rQP-C          132 TAGGGgct-- --TAGGCCCC GCCCACTTGC ---------- ----------
                   *****        ******** **********
                   *****
                   *****

alignment position 251....... 261....... 271....... 281....... 291.......
P884800        226 ctgaggagaa gtgtgagcgc ctccgcctgt ccactgTCCC CCAAAGTCAG
P884801        191 ---------- ---------- ---------- ------Ttgg cacccct---
rQP-C          158 ---------- ---------- ---------- ------TCCC AAAAGCTCAG
                                                         **** **********
                                                         *

alignment position 301....... 311....... 321....... 331....... 341.......
P884800        276 TTCAATCCCC GACgtcctcc gc-------- ---------- ----------
P884801        202 --------CC CACCAAGCCA CCATTCCTCT TCAAGCCCCG CCCCTTgGCA
rQP-C          172 TACATTCACC CACCAGGCCC CCATTCCTCT TCAAGCTCCG CCCCTTaGCA
                   ********** ********** ********** ********** ****** ***
                           ** ********** ********** ********** ******
                           ** ********** ********** ********** ******

alignment position 351....... 361....... 371....... 381....... 391.......
P884800        298 ---------- ---------- ---------- ---------- ----------
P884801        244 ACATCAACCT CTCCcgtgcg ttcactactt accg-TAAGC CCACAAGCAC
rQP-C          222 ACAACACCCT CTCCttgagg tccaccacgc tccctTAAGC CCACAGGCAC
                   ********** ****                       ***** **********
                                                         ***** **********
                                                         ***** **********

alignment position 401....... 411....... 421....... 431....... 441.......
P884800        298 -----TAGGC TCCACCCCAC CGGCCCGGGC AGGGCCTCCA AGgcacctcc
P884801        293 CTCTATAAGC CCCACCCACG TGTCGCTAGA AGCGCCTCTA AGctccgcc-
rQP-C          272 CTCTGTAGGC CCCACCCACA TGTCtctcct tgggtcagtt ccgcacccgc
                   ********** ********** ********** ********** **
                   ********** ********** ****
                   ********** ********** ****

alignment position 451....... 461....... 471....... 481....... 491.......
P884800        343 cacctacggg tcacccagtC AGCCCACTTC TTTCTGGgac aaaggcgtca
P884801        342 ---------- ---------C AGCCCTCTTG GTACTGGtcc tttccggcta
rQP-C          322 g--------- ---------- ---------- ---------- ----------
```

```
                                              *  *********  *******

alignment position   501.......  511.......  521.......  531.......  541.......
P884800         393  tccctttagag acagt-----  ----------  ----------  ----------
P884801         373  cacGAAGGTC  AGGCCACGCC  CACTCAGGTC  AACTgacaat  TTTTTTTTTG
rQP-C           323  ---GAAGGTC  AGGCCGCGCC  CACCGAGGTC  AACTcgca--  TTTCTTTTTG
                        *******  **********  **********  ****        **********
                        *******  **********  **********  ****

alignment position   551.......  561.......  571.......  581.......  591.......
P884800         408  ----------  ----------  AGGAAAATGG  TATCTCCCGG  AAGTTACCTC
P884801         423  AGTCTCCCCC  TTGAGATCGA  AaGAAAATGG  TAGTTCCCGG  AAATGACTTC
rQP-C           368  CATCTCCTCC  TCAAGATAGA  AGAAAAATGG  TAGCTCCCGG  AAATGACTTC
                     **********  **********  **********  **********  **********
                                             **********  **********  **********
                                             **********  **********  **********
                                               ********  **********  **********
                                                *******  **********  **********
                                                *******  **********  **********
                                                *******  **********  **********

alignment position   601.......  611.......  621.......  631.......  641.......
P884800         438  ACGACCTCCA  agagCGGCTT  CCAACCTTGC  CGGAAATGAC  Gaacgagtca
P884801         473  ACGTTCTCTA  GGcTCGGCTT  CCGCTCCTCC  CGGAAGTGAC  Gggcgaaagc
rQP-C           418  ACGATCTCCA  GGgTCGGCTT  CCGCTCCTCC  CGGAAGTGAC  Gtgcaaagcc
                     **********  **  ******  **********  **********  *
                     **********  **  ******  **********  **********  *
                     **********  **  ******  **********  **********  *
                     **********      ******  **********  **********  *
                     **********
                     **********
                     ********

alignment position   651.......  661.......  671.......  681.......  691.......
P884800         488  accggatcgg  tgaCTGTGGA  GGGCGAGCTG  AGCcctgtgc  gtgagtgggg
P884801         523  cGGCTCAGAG  ---CTCTGGA  GGTAGAGGTG  ACCgatcctg  ggggtc----
rQP-C           468  gGGCTCAGCG  ---CTGTGGA  GGTAGAGGTG  ACtgggtaca  gagggtc---
                      *********     *******  **********  ***
                      *********     *******  **********  **
                                    *******  **********  *

alignment position   701.......  711.......  721.......  731.......  741.......
P884800         538  tctggttgtg  caGTGTTCGT  GGACCCTGGG  A-Ggctaggg  gcgccccgct
P884801         566  ----------  ----GGTGTT  AGGCCGTGGG  AgGTTT----  ----------
rQP-C           512  ----------  --GTGGTCGT  GGGCAGTGGG  A-GTTT----  ----------
                                   ********  **********  *  ****
                                                            ****
                                                            ****
                                                            *

alignment position   751.......  761.......  771.......  781.......  791.......
P884800         587  gggctgggaa  aggataagga  gtgcaggggc  aggagtctgg  ggttggggat
P884801         588  ----------  ----------  ----------  ----------  ----------
rQP-C           535  ----------  ----------  ----------  ----------  ----------

alignment position   801.......  811.......  821.......  831.......  841.......
P884800         637  ggacccccgc  ggggactgcg  gcgcTTCGCG  AAAGCGAGCC  AAGCGCCTGT
P884801         588  ----------  ----------  ----TTCGCG  AGACTGAGCC  ACGCGTCTAT
rQP-C           535  ----------  ----------  ----TTTGCG  AGACTGAGCC  ACGCACCTAT
                                                 ******  **********  **********
                                                 ******  **********  **********
                                                 ******  **********  **********
                                                 ******  **********  **********

alignment position   851.......  861.......  871.......  881.......  891.......
P884800         687  Ccaccctcgg  tcctgCAGGG  CCGCCGCCAC  AATGGGCCGC  GAGTTTGGGA
P884801         614  CTTCTGTCC-  -----CAGGG  CCGCCGTCAC  CATGGGCCGC  GAGTTTGGGA
rQP-C           561  CTTCTGTCCt  -----CAGGG  CCGCCGCCAC  CATGGGCCGC  GAGTTTGGGA
```

```
                   *********         *****  **********  **********  **********
                   *********         *****  **********  **********  **********
                   *********         *****  **********  **********  **********
                   *                 *****  **********  **********  **********
                                     *****  **********  **********  **********
                                     *****  **********  **********  **********
                                     *****  **********  **********  **********
                                     *****  **********  **********  **********
                                     *****  **********  **********  **********
                                     *****  **********  **********  **********

alignment position 901.......  911.......  921.......  931.......  941.......
P884800        737 AtCTGACGCG  GATGCGGCAT  GTGATCagCT  ACAGCTTGTC  ACCGTTCGAG
P884801        658 ACCTGGCGCG  GATACGGCAC  GTGATCTCCT  ACAGCTTGTC  GCCCTTTGAG
rQP-C          606 ACCTGACGCG  GATACGGCAT  GTGATCTCCT  ACAGCTTGTC  GCCCTTTGAG
                   **********  **********  **********  **********  **********
                   **********  **********  **********  **********  **********
                   **********  **********  **********  **********  **********
                   ***** ****  **********  **********  **********  **********
                   * *** ****  **********  ******  **  **********  **********
                   * *** ****  **********  ******  **  **********  **********
                   * *** ****  **********  ******  **  **********  **********
                   *                               **  **********  **********
                   *                               **  **********  ******
                   *

alignment position 951.......  961.......  971.......  981.......  991.......
P884800        787 CAGCGCGCCT  ATCCGCACgt  cttcactAAA  GGAATCCCCA  ATGTTCTGCG
P884801        708 CAGCGCGCCT  TCCCAAGCTA  TTTCAGCAAA  GGCATCCCCA  ACGTGCTGCG
rQP-C          656 CAGCGCGCCT  TCCCGCACTA  TTTCAGCAAA  GGCATCCCCA  ACGTGCTGCG
                   **********  **********  **********  **********  **********
                   **********  **********  **********  **********  **********
                   **********  **********  **********  **********  **********
                   **********  **********  **********  **********  **********
                   **********  ********           ***  **********  **********
                   **********  ********           ***  **********  **********
                   **********                     ***  **********  **********
                   **********                                ****  **********
                                                             ****  **********

alignment position 1001......  1011......  1021......  1031......  1041......
P884800        837 CCGCATTCGG  GAGtcttcct  ttCGCGTGGT  GCCGCGTGAG  TGCctgggc
P884801        758 CCGCACTCGC  GAGCGCATCC  TGCGCGTGGC  GCCGCGTGAG  TGCGG-----
rQP-C          706 CCGCACTCGC  GAGCGCATCC  TGCGCGTGGC  GCCGCGTGAG  TGCGG-----
                   **********  **********  **********  **********  *****
                   **********  **********  **********  **********  *****
                   **********  **********  **********  **********  *****
                   **********  **********  **********  **********  *****
                   **********  **********  **********  **********  ***
                   **********  ***            *******  **********  ***
                   **********  ***            ****
                   **********  ***
                   **********  ***

alignment position 1051......  1061......  1071......  1081......  1091......
P884800        887 ccgcgggagc  gggaggctgg  acccagcag   cagcagcagt  cactgcgcct
P884801        803 ----------  ----------  ----------  ----------  ----------
rQP-C          751 ----------  ----------  ----------  ----------  ----------

alignment position 1101......  1111......  1121......  1131......  1141......
P884800        937 cccctctgag  cgctGGCGGC  CGGGcaggcg  ctctgtaact  tGCCGTACAC
P884801        803 ----------  ----GGCGGG  CGGGTACA--  ----------  -GCCGCG--C
rQP-C          751 ----------  ----GGCGGG  TGGGTACG--  ----------  -GCCGCGCGC
                                   ******  *********               *********
                                   ******  *********               ******
                                   ******  *********               ******
                                   ******  *********               ******

alignment position 1151......  1161......  1171......  1181......  1191......
```

```
P884800       987 ACCTTTCTca ttgaa----- ---------- ---------- ----------
P884801       825 ACCTCTCCTT CCGACGCCCT CGCGGGGCAg acgtggaGGA GTTCTTATCA
rQP-C         775 ACCTTTCTTA CCAACATCTT CGACAGACAt -------GGA GTTCTTATCG
                  ********** ********** *********        *** **********
                                                         *** **********
                                                         *** **********
                                                         *** **********

alignment position 1201...... 1211...... 1221...... 1231...... 1241......
P884800      1002 ---------- ---------- ---------- ---------- ----------
P884801       875 TATCGAAATG ACTGGCCTAA GGTCACTtgg gtgactccaa cggctccctc
rQP-C         818 TATCGAAATG ACTGGCCTAA GGTCACTtgg gtga------ ----------
                  ********** ********** *******
                  ********** ********** *******
                  ********** ********** *******
                  ********** ********** *******

alignment position 1251...... 1261...... 1271...... 1281...... 1291......
P884800      1002 ---------- ---------- ---------- ---------- ----------
P884801       925 tgaggtcatc gctggccact atctgtgatg aaaggctgat gcaatcagtg
rQP-C         852 ---------- ---------- ---------- ---------- ----------

alignment position 1301...... 1311...... 1321...... 1331...... 1341......
P884800      1002 ---------- ---------- ---------- ---------- ----------
P884801       975 tcttgtgtta gatgtgcggc cgattttgct gtcggtttca tggcattgtg
rQP-C         852 ---------- ---------- ---------- ---------- ----------

alignment position 1351...... 1361
P884800      1002 ---------- ---
P884801      1025 aatatccgtg aag
rQP-C         852 ---------- ---
```

Pairwise similarities:

For each pairwise alignment, the similarity (relative to the maximum similarity) and the number of identical nucleic acids (in % of shorter sequence) is given. Maximum values are underlined.

	P884801 (1037 bp)	rQP-C (851 bp)
**P884800 (1001 bp)**	0.237<br>30 %	0.295<br>38 %
**P884801 (1037 bp)**		1.000<br>77 %

Please note that the **similarity value 1.000** marks only the two **most similar** sequences, it does not necessarily mean that these sequences are identical.

**Extract alignment region**	
**Positions in alignment**	from: to:
**Output file**	dialign.seq

Extract aligned sequences

For comments, questions or bug reports, please contact support@genomatix.de

© Genomatix Software GmbH 1998-2005
- All rights reserved.

License Agreement

GEMS Launcher 4.1.1

Genomatix-Logo Logout Personal Messages GEMS-Logo

Genomatix_MainGEMSElDoradoGene2PromoterBiblioSphere
Frequently_asked_questionsYour_ResultsYour_SequencesYour_ProtocolHelp

GEMS Launcher Task: *DiAlign TF*: Multiple alignment plus TF sites
working on promoters.seq (3 seq.)

[Alignment] [Pairwise_similarities]

DiAlign professional TF Release 3.1 December 2004 **Tue May 3 03:50:06 2005**

Solution parameters:

**Sequence file:**	promoters.seq (3 seq.)
**Type of sequences:**	nucleotide sequences
**Threshold T:**	0.00
**'*' signs below alignment denote:**	diagonal similarity (max. similarity is represented by 10 '*' signs)
**Alignment output:**	complete alignment is shown<br># nucleic acids per line: 50
**TF output:**	common TF matches located in aligned regions (common to 3 (100.0 %) sequences)
**Family matches:**	yes
**MatInspector library:**	Matrix Family Library Version 5.0 (February 2005)
**Selected groups** (core/matrix sim):	• ALL user_defined.lib (0.75/Optimized)<br>• ALL vertebrates.lib (0.75/Optimized)

Aligned Sequences:

No.	Sequence Name	Sequence Description	Length
1	P910348	sym=BCS1L|loc=Loc617|taxid=9606|spec=Homo sapiens|chr=2| ctg=NT_005403|str=(+)|start=69733410|end=69734410|len=1001| tss=501|comm=BCS1-like (yeast); (AK096210/501/gold;)	1001 bp
2	P910350	sym=Bcs1l|loc=Loc66821|taxid=10090|spec=Mus musculus|chr=1| ctg=NT_039170|str=(+)|start=52342932|end=52343944|len=1013| tss=501,505,513| comm=BCS1-like (yeast); (AK012324/513/gold;AK079385/505/gold;AK078925/501/gold; NM_025784/505/bronze;)	1013 bp
3	P910353	sym=na|loc=Loc301514|taxid=10116|spec=Rattus norvegicus| chr=9|ctg=NW_047816|str=(+)|start=17367336|end=17368336| len=1001|comm=similar to BCS1-like; (CompGen promoter)	1001 bp

Alignment (DiAlign format):

Please note that only **upper-case** letters are considered to be **aligned**. For more information, please have a look at the user guide.

V$MYT1 V$GABF V$EKLF V$ETSF V$ZBPF V$SP1F V$PBXC

```
alignment position 1 . . . . . . . . .  1 1 . . . . . . . .  2 1 . . . . . . . .  3 1 . . . . . . . .  4 1 . . . . . . . .
P910348        1 - - - - - - - - - -  - - - - - - - - - -  - - - - - - - - - -  - - - - - - - - - -  - - - - - - - - - -
P910350        1 - - - - - - - - - -  - - - - - - - - - -  - - - - - - - - - -  - - - - - - - - - -  - - - - - - - - - -
P910353        1 g g t a g g a g t g  a g c c a g a a a a  a a g t a a c g t g  c t t t c t t t g a  c a c c g g a g a a
```

```
alignment position 51........ 61........ 71........ 81........ 91........
P910348         1 ---------- ---------- ---------- ---------- ----------
P910350         1 ---------- ---------- ---------- ---------- ----------
P910353        51 aaaaaggaac acagaaggcg cccctgagta agcacctcag ttcccacgcg

alignment position 101....... 111....... 121....... 131....... 141.......
P910348         1 ---------- ---------- ---------- ---------- ----------
P910350         1 ---------- ---------- ---------- ---------- ----------
P910353       101 cctctgggct tggactcacc gtgtggcctt gaacagatgc acttgcctcg

alignment position 151....... 161....... 171....... 181....... 191.......
P910348         1 ---------- ---------- ---------- ---------- ----------
P910350         1 ---------- ---------- ---------- ---------- ----------
P910353       151 cttctctggg cctcagcttt cctatctgta aagtggaggc ggcgacagcc

alignment position 201....... 211....... 221....... 231....... 241.......
P910348         1 ---------- ---------- ---------- ---------- ----------
P910350         1 ---------- ---------- ---------- ---------- ----------
P910353       201 aagtcacgag atccagcgga aaagaaaggc gtttgaaacc ctgcaaggct

alignment position 251....... 261....... 271....... 281....... 291.......
P910348         1 ---------- ---------- ---------- ---------- ----------
P910350         1 ---------- ---------- ---------- ---------- ----------
P910353       251 tgggatgatg catcgtcacg attatggtcc tgaggagagg cagcggatac

alignment position 301....... 311....... 321....... 331....... 341.......
P910348         1 ---------- ---------- ---------- ---------- ----------
P910350         1 ---------- ---------- ---------- ---------- ----------
P910353       301 ccgggcgggg cccggagctg tgacgcgact gaggcgccca agtgcagcag

alignment position 351....... 361....... 371....... 381....... 391.......
P910348         1 ---------- ---------- ---------- ---------- --------CC
P910350         1 ---------- ---------- ---------- ----CCGCAG CACCTCCTCC
P910353       351 agatgcgccc gagccccgct catcgacgcg ctccCCTCAG CACCTCCTCC
                                                      ****** **********
                                                      ****** **********

alignment position 401....... 411....... 421....... 431....... 441.......
P910348         3 CAGGCCCACC CTGATCCCGG GGCTcga--- ---------- ---AAAACTT
P910350        17 CGCGGgct-C CACCTCCAGG GCCCACTCAA GCTGGAGCTA GAGAAACTTT
P910353       401 CACGGCTTCC CACCTCCAAG GCCTATTCAA GCTGGGGCTA GAGAAACTTT
                  ********** ********** ********** ********** **********
                  *****    * ********** ********** ********** **********
                                                          ** **********
                                                          ** **********
                                                          ** **********

alignment position 451....... 461....... 471....... 481....... 491.......
P910348        37 TGCCTCCAGC TCGCTCCACC CGCATTCCCt tcccaGTTCC GCCCCGCAGC
P910350        66 TACCTTCAGC TCACAGCGCC CGCACTTCCC CTG--GTTCC CGCCGACAGC
P910353       451 TACCTCCAGC TCGCAGCGTC CGCACTTCCC CTG--GTTCC CGCCGACAGC
                  ********** ********** ********** ***  ***** **********
                  ********** ********** ********** ***  ***** **********
                  ********** ********** ********** ***  ***** **********
                  ********** ********** ********** ***  ***** **********
                  ********** ********** ********** ***  ***** **********
                    ******** ********** ********** ***  ***** **********
                                                        ***** **********
                                                        ***** **********

alignment position 501....... 511....... 521....... 531....... 541.......
```

```
P910348         87 CGCTCACTCA CCTCCagaCC CAGAGCCGGC GGGAGCCGCA TCTCTATGGT
P910350        114 CGCTCTCTCA CCTCCTACCC gtGGGCTGGC GGGAGCTGCA TCTCTATGGT
P910353        499 CGCTCTCTCA CCTTCTACCC CAGGGCTGGC GGGAGCTGCA TCTCTATGGT
                   ********** ********** ********** ********** **********
                   ********** ********** ********** ********** **********
                   ********** ********** ********** ********** **********
                   ********** **********   ******** ********** **********
                   ********** **********   ******** ********** **********
                   ********** **********   ******** ********** **********
                   ********** *****   **   ******** ********** **********
                   ********** ***     **   ******** ********** **********
                                             ****** ********** **********
                                             ****** ********** **********

alignment position 551....... 561....... 571....... 581....... 591.......
P910348        137 C-GGCCGCGG CTGGAACGGC CCCCGGGGCC CGCTTtgTGC GACCGCCTCC
P910350        164 CCGACTGGGA CTGGAGTGGC TCCTCGGGGG CCTTTGATGT GGCCGCCCCC
P910353        549 CCGACTGGaA CTGGCGTGGC TCCTCAGGGG CCTTTGATGC GACCGCCCCC
                   ********** ********** ********** ********** **********
                   ******** * ********** ********** ********** **********
                   ******** * ********** ********** ********** **********
                   ********                                ***  **********
                   ********                                **     ********
                   ********                                **     ********
                   *                                       **     ********
                   *                                              ********
                   *                                              ********
                   *                                              ********

alignment position 601....... 611....... 621....... 631....... 641.......
P910348        186 GGCTCAGCCG CCTCTAGGAt attggggaTG TGGAAGCCAc tctCTCTTCC
P910350        214 GGCCCAGGCG CCTCTAGGAA TCTGACG-TG TGGCAGCCA- ---CTCTGTC
P910353        599 GGCCCAGGCG CCTCTGGGAA TCTGACG-TG TGGcggtCA- ---CTCTGTC
                   ********** ********** ******* ** *********     *******
                   ********** ********** ******* ** ***    **     *******
                   ********** ********** ******* ** ***    **     *******
                   ********** ********** ******* ** ***           *******
                   ********** ********** ******* ** ***
                   ********** ********** ******* ** ***
                   ********** *********
                   ********** *********
                   ********** *********
                   ********** *********

alignment position 651....... 661....... 671....... 681....... 691.......
P910348        236 TCTTGCCGGC TGCCGGGGTA CTCTATGTTT Tgtctctgtg gtttttcccg
P910350        259 TCTCTTCAAG GCCCGTGCTA CTCTATGGTT TCCCCTCTAT GTCCTTGAGT
P910353        644 TCTCTTCGAG GCCCGTGCCA CTCTATGGTT TCCCCTCTGT GTCCTTGAGT
                   ********** ********** ********** ********** **********
                   ********** ******** * ********** ********** **********
                   ********** ******** * ********** ********** **********
                   ********** ******** * ********** ********** **********
                                       * ********** ********** **********
                                       * ********** *

alignment position 701....... 711....... 721....... 731....... 741.......
P910348        286 ccctccaccc ccACTCCAGC CCTGAAGCCg gagaa--GCA CCATCGAGAG
P910350        309 ---------- --ACCCCcct tCTGAAGCCC CAAAGCGGAA CCATCGAGAA
P910353        694 ---------- --ACCCCAGC GCTGAAGCGC CAAAGCGGAA CCATCGAGAA
                                ******** ********** ********** **********
                                *****     ********* ********** **********
                                *****     ********* ********** **********
                                *****     ********* ********** **********
                                *****                      *** **********
                                                             * **********

alignment position 751....... 761....... 771....... 781....... 791.......
P910348        334 CTCTGGGGTA GAGTGGCGTG GGCTTCtctG TGGGTGTGGC GGGAAGTAGA
P910350        347 TTGCAGGGAA GAGTGGCAAG GACTCCGTGG TGGGTGTGGC CGGAAGTAGA
P910353        732 TTTCAGGGAA GAGTGGCAAG GACCTCGTGG TGGGTGTGGC AGGAAGTAGA
```

```
                      ********** ********** ********** ********** **********
                      ********** ********** ********** ********** **********
                      **  ****** ********** ********** ********** **********
                      **  ****** ********** ********** ********** **********
                      **  ****** ********** ********** ********** **********
                      **  ****** ********** ******   * **********  *********
                                                     * **********  *********
                                                     * **********
                                                     * **********

alignment position    801....... 811....... 821....... 831....... 841.......
P910348          384  TGAAAGGACC CGGGGCGGGg ccgaacgcag cttccccaag gtgcAGGCGC
P910350          397  TGCAGCAGTC CGGGGCGGGA CTAGCGGCGC Tgcctcttcg t---AGGCCC
P910353          782  TTCTGCATTC GGGGGCGGGC CTAGAGGCGC Ttattccgag tacggact--
                      ********** ********** ********** *              ******
                      ********** ********** ********** *              ******
                      ********** ********** ********** *
                      ********** *********
                      ********** *********
                      ********** *********
                      *

alignment position    851....... 861....... 871....... 881....... 891.......
P910348          434  GGTGAAACCA TCGAGACGGA GGGCCAGAGA GTCACGGCGG TGAGAGGGCT
P910350          444  GGCGAAACCA TCGAGTGTGA GGGCCAGAGA GTCATGGAGG GAAGGGGGCG
P910353          830  GGCGAAACCA TCGAGTCTGA GGGCCAGAGA GTCATGaaag tAAGGGGGCG
                      ********** ********** ********** ********** **********
                      ********** ********** ********** ********** **********
                      ********** ********** ********** ********** **********
                      ********** ********** ********** ******      *********
                      ********** ********** ********** ******      *********
                      ********** ********** ********** ******      *********
                      ********** ********** ********** ******       ********
                      ********** ********** ********** ****
                      ********** *****   ** ********** ****
                                         ** ********** ****

alignment position    901....... 911....... 921....... 931....... 941.......
P910348          484  GAGTGACGGG TTACCCCAAA CCATGTGGCT GGAGGCGCGA GCTGGGGTGT
P910350          494  GAGTGACAGA TTTCTCtctc CCACGTTCCT GGAAGCGCGA GCAGGTCTGT
P910353          880  GAGTGACAGA TTTCTCCCGA CCACGTTCCT GGGAGCGCGG GCTGGGTTGT
                      ********** ********** ********** ********** **********
                      ********** ********** ********** ********** **********
                      ********** *******    ********** ********** **********
                      ********** *******    ********** **    ****** **********
                      ********** *******
                      *******
                      *******

alignment position    951....... 961....... 971....... 981....... 991.......
P910348          534  G--GGCGAGG TTCAGGAGGG GGTTCCTGGG TGTCGCGGa- ---GGGGCCG
P910350          544  GtaTAGGGTG TCCGGGAGTC AGCTGCTGTG TGTCAGGGTt cggGAGCCGG
P910353          930  G--TAGGGTG TCCGGGAGTC AGCCGCTGTG TGTCAGGGTc agt-AGCCGG
                      *  ******* ********** ********** *********    *******
                      *  ******* ********** ********** *********     ******
                      *  ******* ********** ********** *********     ******
                      *  ******* ********** ********** *********     ******
                         ******* ********** ********** *********
                         ******* *********

alignment position    1001...... 1011...... 1021...... 1031...... 1041......
P910348          578  GGGACGGGAG AGCTTTGTCT GTGGCctTCC ATGCATAGGT GTTTTGGAAT
P910350          594  GGGCCAGGAG ATCTTCGTCA GCGGCtcTCC ATGCAAAGGT GCTTTGGAAT
P910353          977  AGGCCAGGAG ATCTTCGTCA GTGGC----- ---------- ----------
                      ********** ********** *****  *** ********** **********
                      ********** ********** *****  *** ********** **********
                      ********** ********** *****  *** ********** **********
                      ********** ********** *****  *** ********** **********
                       ********* ********** *****

alignment position    1051...... 1061...... 1071...... 1081...... 1091......
```

```
P910348             628 TCTGGATCCC AGtTCTGAGG CTCAGCCGAG GTCACTGCTG TCAGCTCACC
P910350             644 TCTGGATCAC AGcTCCGAGC CTTAGCGGAG GTCACGGCTG TCAATTCACC
P910353            1002 ---------- ---------- ---------- ---------- ----------
                        ********** ** ******* ********** ********** **********
                        ********** ** ******* ********** ********** **********
                        ********** **
                        ********** **

alignment position      1101...... 1111...... 1121...... 1131...... 1141......
P910348             678 tc----CCTG CATGCCAGGC GCAGGgcaaA GTGGCCGGGG TTCGGGGTTG
P910350             694 agtgggCCGG TGTGCCAGGC AGAGG----A GTGGCAGTGG AGAAGTGTTG
P910353            1002 ---------- ---------- ---------- ---------- ----------
                              **** ********** *****    * ********** **********

alignment position      1151...... 1161...... 1171...... 1181...... 1191......
P910348             724 g-AGGAGAGG TGTACTAGAA ACAACTTTGC TCTTGTCCCt caggatctta
P910350             740 caAGGAGAGG TGTTCTGGAA ATAATTGCCA TTTTGTCCCc ttaggcattg
P910353            1002 ---------- ---------- ---------- ---------- ----------
                          ******** ********** ********** *********
                          ******** ********** ********** *********

alignment position      1201...... 1211...... 1221...... 1231...... 1241......
P910348             773 ttgtctacga tgtgacga-- ---------- ---------- ----------
P910350             790 tcttaagtta ggcttagtca catcttaatt gggttgaatt tgaaaaaggc
P910353            1002 ---------- ---------- ---------- ---------- ----------

alignment position      1251...... 1261...... 1271...... 1281...... 1291......
P910348             791 ---------- ---------- ---------- ---TCCGTGG AGAAAGTAGc
P910350             840 tccaaaaaaa aaaaaaaaga aaaagaaaaa ggcTCCGTGG AAGAAGTAGa
P910353            1002 ---------- ---------- ---------- ---------- ----------
                                                            ******* *********

alignment position      1301...... 1311...... 1321...... 1331...... 1341......
P910348             808 agatgggcag attttcattt ggggagaatg agggagaaaa gaacggg-CA
P910350             890 ctatatgatc tcaatgaaaa agaatgccta gaataaaggg gtgggggtCA
P910353            1002 ---------- ---------- ---------- ---------- ----------
                                                                          **

alignment position      1351...... 1361...... 1371...... 1381...... 1391......
P910348             857 TTCCGAGCCA AGAGCACTGC ATGAGCAAGG AGTtgggagg ttgcttactt
P910350             940 TTCGGTACCA ACAGAACTGA GAGAGCTAGA AGTaggtagt g---------
P910353            1002 ---------- ---------- ---------- ---------- ----------
                        ********** ********** ********** ***

alignment position      1401...... 1411...... 1421...... 1431...... 1441......
P910348             907 gttgaacata tttgttgcgt atttgcatAT TTGCTAaaca ggtagtgtta
P910350             981 ---------- ---------- --------AT TTGCTAgtaa aggtacgcat
P910353            1002 ---------- ---------- ---------- ---------- ----------
                                                      ** ******

alignment position      1451...... 1461...... 1471...... 1481...... 1491.
P910348             957 ggagctaatc catgaaaggt aggtgggcgc cgtattgtag agagc
P910350            1003 tctaatgtag g--------- ---------- ---------- -----
P910353            1002 ---------- ---------- ---------- ---------- -----
```

Pairwise similarities:

For each pairwise alignment, the similarity (relative to the maximum similarity) and the number of identical nucleic acids (in % of shorter sequence) is given. Maximum values are underlined.

	P910350 (1013 bp)	P910353 (1001 bp)

**P910348 (1001 bp)**	0.404 50 %	0.279 32 %
**P910350 (1013 bp)**		1.000 53 %

Please note that the **similarity value 1.000** marks only the two **most similar** sequences, it does not necessarily mean that these sequences are identical.

**Extract alignment region**	
**Positions in alignment**	from: to:
**Output file**	dialign.seq

Extract aligned sequences

For comments, questions or bug reports, please contact support@genomatix.de

© Genomatix Software GmbH 1998-2005 - All rights reserved. License Agreement GEMS Launcher 4.1.1

Genomatix-Logo Personal Password Messages Logout GEMS-Logo
Personal Password Messages Logout

Genomatix PortalGEMSElDoradoGene2PromoterMatBase
Frequently_asked_questionsYour ProjectsYour ProtocolHelp

GEMS Launcher Task: *DiAlign TF*: Multiple alignment plus TF sites
working on UQCRB.seq (3 seq.)

[Alignment] [Pairwise similarities]

DiAlign professional TF Release 3.1.2 January 2007 **Fri Feb 16 20:24:21 2007**

Solution parameters:

**Sequence file:**	UQCRB.seq (3 seq.)
**Type of sequences:**	nucleotide sequences
**Threshold T:**	0.00
**'*' signs below alignment denote**:	diagonal similarity (max. similarity is represented by 10 '*' signs)
**Alignment output:**	complete alignment is shown<br># nucleic acids per line: 50
**TF output:**	common TF matches located in aligned regions (common to 3 (100.0 %) sequences)
**Family matches:**	yes
**MatInspector library:**	Matrix Family Library Version 6.2 (October 2006)
**Selected groups** (core/matrix sim)	• ALL user_defined.lib (0.75/Optimized)<br>• ALL vertebrates.lib (0.75/Optimized)

Aligned Sequences:

No.	Sequence Name	Sequence Description	I
1	GXP_207133	GXP_207133 loc=GXL_172620|sym=UQCRB|geneid=7381|acc=GXP_207133|taxid=9606|spec=Homo sapiens|chr=8|ctg=NC_000008|str=(-)|start=97316487|end=97317487|len=1001|tss=501|descr=ubiquinol-cytochrome c reductase binding protein|comm=GXT_2834825/NM_006294/533/bronze	
2	sym=mmUQCRB|chr13:67340605-673	sym=mmUQCRB|chr13:67340605-67341558 5'pad=0 3'pad=0 revComp=TRUE strand=? repeatMasking=none	9
3	GXP_52345	GXP_52345 loc=GXL_43082|sym=Uqcrb_predicted|geneid=362897|acc=GXP_52345|taxid=10116|spec=Rattus norvegicus|chr=7|ctg=NC_005106|str=(-)|start=67963262|end=67964262|len=1001|tss=501|descr=ubiquinol-cytochrome c reductase binding protein (predicted)|comm=GXT_21790265/XM_343225/529/bronze	

Alignment (DiAlign format):

Please note that only **upper-case** letters are considered to be **aligned**. For more information, please have a look at the user guide.

V$HOMF V$NKXH V$HOXF V$NRF1 V$STAF V$EREF V$NR2F V$YY1F TSS

```
alignment position                               1......... 11........ 21........ 31........ 41........
GXP_207133 (UQCRB)                           1 cgggtactcc ag-------- ---------- ---------- -A-GGAATCC
sym=mmUQCRB|chr13:67340605-673 (mmUQCRB)     1 ---------- ------GTGC TTACTTTATA TTCTGTATTA AA-GTAATCC
GXP_52345 (Uqcrb_predicted)                  1 taacaattat tgtgtaGTGC TTACTTTATA TCCTGTATTA AAaGTAATCT
                                                                ****  ********** ********** ** *******
                                                                ****  ********** ********** ** *******
                                                                ****  ********** ********** **

alignment position                               51........ 61........ 71........ 81........ 91........
GXP_207133 (UQCRB)                          21 TGgaggaagc tttatggctt gctgcatg-- ---------- ----------
sym=mmUQCRB|chr13:67340605-673 (mmUQCRB)    34 TGAGGTGATT TAAAGAATGC AATAGTGT-- ATACATTATA TTAAAACACC
GXP_52345 (Uqcrb_predicted)                 51 AGAGATGCTT TAAAGAAAGC AAGAGTGTgc ATAGATTATA CTAAAACACC
                                                 ********** ********** ********   ********** **********
                                                 ********** ********** ********   ********** **********
                                                                                  ********** **********
                                                                                  ********** **********
                                                                                  ********** **********

alignment position                               101....... 111....... 121....... 131....... 141.......
GXP_207133 (UQCRB)                          49 ---------- -CAGGAAGAG ATAGGTCATC AAACC--TTT TCTgaaacta
sym=mmUQCRB|chr13:67340605-673 (mmUQCRB)    82 GCACCATTTG ACATAAAGAA TTTGAGCATC AAGCG--TCC TCTAGCGTCC
GXP_52345 (Uqcrb_predicted)                101 GCACCATTTG ACATAAAGcA TTTGACCATC AAGCttaTCC TCTAGTGTCC
                                                 ********** ********** ********** *****  *** **********
                                                 ********** ********                     *** **********
                                                 ********** ********                     *** **********
                                                 ********** ********
                                                 ********** ********

alignment position                               151....... 161....... 171....... 181....... 191.......
```

```
GXP_207133 (UQCRB)                                   86 caatttctcc aatattttca actcgaaata atcaatgtac caattccata
sym=mmUQCRB|chr13:67340605-673 (mmUQCRB)            130 TAGCATCCCC ---------- ---------- ---------- ----------
GXP_52345 (Uqcrb_predicted)                         151 TAGAATCCCC ---------- ---------- ---------- ----------
                                                        **********
                                                        **********
                                                        **********

alignment position                                      201....... 211....... 221....... 231....... 241.......
GXP_207133 (UQCRB)                                  136 tatttttggg atggcacatc catcagTCCT TCAATAGAAA GTAAGACGAT
sym=mmUQCRB|chr13:67340605-673 (mmUQCRB)            140 ---------- ---------- ------TCCA TGCATTTTGA GatgcgGTTT
GXP_52345 (Uqcrb_predicted)                         161 ---------- ---------- ------TCCA TCCATACTGA GGAAGTGTGT
                                                                                    **** ********** **********
                                                                                    **** ********** *     ****
                                                                                    **** ********** *
                                                                                    **** ********** *

alignment position                                      251....... 261....... 271....... 281....... 291.......
GXP_207133 (UQCRB)                                  186 GCCCTTAAAA CATTTAGCT- -----TCCTG CCTGGCACAA AGAgctgaat
sym=mmUQCRB|chr13:67340605-673 (mmUQCRB)            164 GCCTGAAAAT CATTTACTA- -----CATTA CCTGTCTCCA AGA-----CT
GXP_52345 (Uqcrb_predicted)                         185 GCCTGAAAAT CATCTACTAt ctattCATTA CCTGTCTCCA AGcaaaggCT
                                                        ********** *********       ***** ********** ***     **
                                                        ********** *********       ***** ********** **

alignment position                                      301....... 311....... 321....... 331....... 341.......
GXP_207133 (UQCRB)                                  230 taatactgtc cacgctccca tagctattaa cagcaaATAA CAGAacccag
sym=mmUQCRB|chr13:67340605-673 (mmUQCRB)            203 CCATTTATTC CGCAAAGgTT CTTTCCT--- ------ATAA CAGAGCATTG
GXP_52345 (Uqcrb_predicted)                         235 CCATCTATAC CATAAAG-TT CTTTCCT--- ------GTAA CAGGGCATGG
                                                        ********** *******  ** *******         **** **********
                                                                           ** *******         **** **********
                                                                           ** *******         **** **********

alignment position                                      351....... 361....... 371....... 381....... 391.......
GXP_207133 (UQCRB)                                  280 ctttaagcct tgGTCTCCTA ATTcctgacc tgtgtTCTTC CCATCACATC
sym=mmUQCRB|chr13:67340605-673 (mmUQCRB)            244 AATAACACT- --GTCTCCTA ATTTCGCAGT AC---TTTCC CCATTGTGTC
GXP_52345 (Uqcrb_predicted)                         275 AAGAACACC- --GTCCCCTA ATTTCGTACT AC---TTTTC CCATTGTGTG
                                                        *********    ******** ********** **   ***** **********
                                                        *********    ******** ********** **   ***** **********
                                                        *********    ******** ********** **   ***** **********
                                                                     ******** ***

alignment position                                      401....... 411....... 421....... 431....... 441.......
GXP_207133 (UQCRB)                                  330 ACAACCAATA CTTCTTAGAA TGTAGCACGC AAAATGCCCC GCCTACTcca
sym=mmUQCRB|chr13:67340605-673 (mmUQCRB)            288 GCGAAGAATG TTTTTTGTAA CAAGGCACAC AAAAAGATCA GCCTAATGTG
GXP_52345 (Uqcrb_predicted)                         319 ACGAAAAATG TTTCTT-TAA CAAGGCGCAC AAAAAGATCA GGCTAATGTG
                                                        ********** ********** ********** ********** **********
                                                        ********** ***    *** ********** ********** **********
                                                        ********** ***    *** ********** ********** **********
                                                              **** ***    *** ********** ********** **********
                                                                          *** ********** ********** **********

alignment position                                      451....... 461....... 471....... 481....... 491.......
GXP_207133 (UQCRB)                                  380 ttggcctcac cctggccgag aggtgcttac gcaggtgctg aacggcagtc
sym=mmUQCRB|chr13:67340605-673 (mmUQCRB)            338 TGGAGTCcaT TCAGGTAGGA AAgagt---A AATGCCTTCT GCTGGCG---
GXP_52345 (Uqcrb_predicted)                         368 TGGAGTCcgT TCAGATAAGA AAatttaaaA AACGCCTTCC ACGGGCC---
                                                        *******  * ********** **       * ********** *******
                                                        *******                        * ********** *******
                                                        *******
                                                        *******
                                                        *******

alignment position                                      501....... 511....... 521....... 531....... 541.......
GXP_207133 (UQCRB)                                  430 GTCAGAACTG CGCCTGCGCA AGCGGCCTtt ctctgttcgc gatgtgacgt
sym=mmUQCRB|chr13:67340605-673 (mmUQCRB)            382 GTCAGAGCAG CGCCTGCccc tc-------- ---------- ------CCTT
GXP_52345 (Uqcrb_predicted)                         415 ATCAGAGCTG CGCCTGTGCA GGCGCCCTca gcactccctc ttccctCCCT
                                                        ********** ********** ********                  ****
                                                        ********** ********** ********                  ****
                                                         ********* ******                               ****
                                                         ********* ******

alignment position                                      551....... 561....... 571....... 581....... 591.......
GXP_207133 (UQCRB)                                  480 aacg------ ---------- --CGCCTGCG gactggGCCC AGCTTGTCCT
sym=mmUQCRB|chr13:67340605-673 (mmUQCRB)            408 CCCCTTCCTA AAGTATCCAG GACGCCTGCG tagGA-GCCC GGCGTGTCCT
GXP_52345 (Uqcrb_predicted)                         465 CCCCTTCCGA AAGTATGCAG GACGCCTGCG cgtGA-CCCC GGCGCGTCCT
                                                        ********** ********** **********    ** **** **********
                                                        ********** ********** **********    ** **** **********
                                                        ********** ********** **********    ** **** **********
                                                                                            ** **** **********
                                                                                            ** **** **********
                                                                                               **** **********
                                                                                               **** **********
                                                                                               **** **********
                                                                                                *** **********
```

```
                                                                                ********** 
                                                                            *** **********
alignment position                  601....... 611....... 621....... 631....... 641.......
GXP_207133 (UQCRB)              512 CTATGACTTA CCCAGAAGGC AACGCTTCTC TTTCTGGTCA AAATGGCTGG
sym=mmUQCRB|chr13:67340605-673  457 CTAGGACTTA CCCAGAAGGC AGCGCTTCAC TCTCAGGTCA AAATGGCGGG
(mmUQCRB)
GXP_52345 (Uqcrb_predicted)     514 CTAGGACTTA CCCAGAAGGC AGCTCTctAC TCTTAGGTCA AAATGGCGGG
                                    ********** ********** ********** ********** **********
                                    ********** ********** ********** ********** **********
                                    ********** ********** ******  ** ********** **********
                                    ********** ********** ******  ** ********** **********
                                    ********** ********** ******  ** ********** **********
                                    ********** ********** ***          ******** **********
                                    ********** ********** ***
                                    ********** ********** * *
                                    ********** ********** * *
                                    ********** ********** *

alignment position                  651....... 661....... 671....... 681....... 691.......
GXP_207133 (UQCRB)              562 TAAGCAGGCC GGTAAGTAAC TgggggtCTT CTTGGCCGGC ATCGTCGCGC
sym=mmUQCRB|chr13:67340605-673  507 CCGATCTGCT GGTGAGTAAT Tactc--CTC CTTTGTGGGT ATCGTGGAGC
(mmUQCRB)
GXP_52345 (Uqcrb_predicted)     564 CCGACCTGCA GGTAAGTgag ctcatc-CTC TTTTGTGGGT ATCGTGGGGC
                                    ********** ********** *      *** ********** **********
                                    ********** *******               ********* **********
                                    ********** *******               ********* **********
                                    ********** *******               ********* **********
                                    ********** *******               ********* **********

alignment position                  701....... 711....... 721....... 731....... 741.......
GXP_207133 (UQCRB)              612 ttcaccgttt at-------- ---------T TTTGCTGCAG CTtgctccgt
sym=mmUQCRB|chr13:67340605-673  555 GGTTGTATTT TCTCACGAAc tTGTTCTCCT TTTGCGTTAG CTAAAtagcT
(mmUQCRB)
GXP_52345 (Uqcrb_predicted)     613 GGTTGTATTT TCCCACGAAa -TGCTCTCAT TTTGCTTTAG CTAAA----T
                                    ********** *********   ********* ********** *****    *
                                    ********** *********   ********* ********** *****    *
                                    ********** *********                                 *
                                    ********** *********
                                    ********** *********

alignment position                  751....... 761....... 771....... 781....... 791.......
GXP_207133 (UQCRB)              645 tttctctctt ctttgtaa-- GTGGCCAAGT TTGTCGCGGA AGGGAAAttg
sym=mmUQCRB|chr13:67340605-673  605 AGTTTGTGAT AAGAGACGTG GCGACCCAGC ----AGCTCA AAGGACAGGT
(mmUQCRB)
GXP_52345 (Uqcrb_predicted)     658 AGTTTGCGAT AAGAGACGTG GTGGCCCAGC CTGTAGCTCA AAGGACAGGT
                                    ********** ********** ********** ********** **********
                                    ********** ********** **********     ****** **********
                                    ********** ********** **********     ****** **********

alignment position                  801....... 811....... 821....... 831....... 841.......
GXP_207133 (UQCRB)              693 gctgcttcag tccagtgaaa aggatgtgct tccccactcg tttgctgtcc
sym=mmUQCRB|chr13:67340605-673  651 TTCGCTgag- ---------- ---------- ---------- ----------
(mmUQCRB)
GXP_52345 (Uqcrb_predicted)     708 TTCGCTtaa- ---------- ---------- ---------- ----------
                                    ******
                                    ******
                                    ******

alignment position                  851....... 861....... 871....... 881....... 891.......
GXP_207133 (UQCRB)              743 CCAGGGCGGG CTTGCCTGta tagggttggg atgttaaccc gctgtttgtG
sym=mmUQCRB|chr13:67340605-673  660 CCAGGTCGGG CCTGCCTGAG GGTTAGAACA GGGACCTCTG CCG------G
(mmUQCRB)
GXP_52345 (Uqcrb_predicted)     717 ---------- CCTGCCTGAG GATTAGAACA GGGACCTCCG CCG------G
                                    ********** ********** ********** ********** ***      *
                                               ********** ********** ********** ***      *
                                               ********** ********** ********** ***      *
                                               ********** ********** ********** ***      *
                                               ********** ********** ********** ***      *
                                               ********                                  *

alignment position                  901....... 911....... 921....... 931....... 941.......
GXP_207133 (UQCRB)              793 AAGGTGCCCG CGGTGGCTct gacagccctg gagttccagc tgcccgtGCT
sym=mmUQCRB|chr13:67340605-673  704 AAGGAGCCCA GCGGGGCTga c--CG----- ---------- -------GGT
(mmUQCRB)
GXP_52345 (Uqcrb_predicted)     751 AAGGAGCCCG CTGTGGCaga cagCG----- ---------- -------GGA
                                    ********** ********      **                     ***
                                    ******                                          ***
                                    ******
                                    ******
                                    ******
                                    ******

alignment position                  951....... 961....... 971....... 981....... 991.......
GXP_207133 (UQCRB)              843 TGGAAGTCCT TCCC-CTTGG CCTTAGAAAA GTTAAAGGGC Acaagaggat
sym=mmUQCRB|chr13:67340605-673  730 TCGGCCTCCT TCCC-CTCCG CCTTGGGACA GCTTAGGGag gTGAGGAGGT
(mmUQCRB)
GXP_52345 (Uqcrb_predicted)     779 TCGGCCTCCT CCCttCTCCG CCTTATGACA GCTTAGGAAC ATGGGGAGGT
                                    ********** **** ***** ********** ********** **********
                                    ********** ***  ***** ********** *******     *********
                                                                                 *********

alignment position                  1001...... 1011...... 1021...... 1031...... 1041......
```

```
GXP_207133 (UQCRB)                                892 aactagtgga tgactttcaa cccgaatgag ctcagtgctt tctatgaccc
sym=mmUQCRB|chr13:67340605-673 (mmUQCRB)          779 CAGCAGACGA C--------- ---------- ---------- ----------
GXP_52345 (Uqcrb_predicted)                       829 CAGCAGAAGA C--------- ---------- ---------- ----------
                                                      ********** *
                                                      ********** *
                                                      ********** *

alignment position                                    1051...... 1061...... 1071...... 1081...... 1091......
GXP_207133 (UQCRB)                                942 aagtgctcta tcgtgtgcat tggccctata aattttctTG CCATTGCGAC
sym=mmUQCRB|chr13:67340605-673 (mmUQCRB)          790 ---------- ---------- ---------- --------AG CCACAGCGAG
GXP_52345 (Uqcrb_predicted)                       840 ---------- ---------- ---------- --------TG CCACAGCGAG
                                                                                               ** **********
                                                                                               ** **********
                                                                                               ** **********

alignment position                                    1101...... 1111...... 1121...... 1131...... 1141......
GXP_207133 (UQCRB)                                992 G---AGGGAG ata------- ---------- ---------- ----------
sym=mmUQCRB|chr13:67340605-673 (mmUQCRB)          802 TagtGGGGAG CATTTGGAGT CAGGCGCAGG CCACTTCAGT GACAGTGATC
GXP_52345 (Uqcrb_predicted)                       852 T---GGGGAG CATTTAGAGT CAGACGCAGG CCTTTTCAGT GACAGTGATC
                                                      *   ****** ********** ********** ********** **********
                                                      *   ****** ********** ********** ********** **********
                                                      *   ****** ********** ********** ********** **********
                                                          ****** ********** ********** ********** ****

alignment position                                    1151...... 1161...... 1171...... 1181...... 1191......
GXP_207133 (UQCRB)                               1002 ---------- ---------- ---------- ---------- ----------
sym=mmUQCRB|chr13:67340605-673 (mmUQCRB)          852 GGAGCAAACA GCGCCCGAGC CCGCATGACG CTCCtaagac ttgtcagaAG
GXP_52345 (Uqcrb_predicted)                       899 TGAGCAAATA GCTCTGGAGT CCGGATGCCG CTCCtcgagc ttgtc---AG
                                                      ********** ********** ********** ****               **
                                                      ********** ********** ********** ****               **
                                                      ********** ********** ********** ****

alignment position                                    1201...... 1211...... 1221...... 1231...... 1241......
GXP_207133 (UQCRB)                               1002 ---------- ---------- ---------- ---------- ----------
sym=mmUQCRB|chr13:67340605-673 (mmUQCRB)          902 AATTAAAAGG AAGTAAGATA GGgttatAGT GAGGTTTCAG GTTAACCTTG
GXP_52345 (Uqcrb_predicted)                       946 AATTAAAGGG AGGTAAGATA GGtcac-AGT GAGGTTTCAG GTTAATCTTG
                                                      ********** ********** **     *** ********** **********
                                                      ********** ********** **     *** ********** **********
                                                                                   *** ********** **********

alignment position                                    1251...
GXP_207133 (UQCRB)                               1002 -------
sym=mmUQCRB|chr13:67340605-673 (mmUQCRB)          952 GCa----
GXP_52345 (Uqcrb_predicted)                       995 GCctatc
                                                      **
                                                      **
                                                      **
```

Pairwise similarities:

For each pairwise alignment, the similarity (relative to the maximum similarity) and the number of identical nucleic acids (in % of shorter sequence) is given. Maximum values are underlined.

	sym=mmUQCRB|chr13:67340605-673 (954 bp)	GXP_52345 (1001 bp)
GXP_207133 (1001 bp)	0.112<br>27 %	0.090<br>25 %
sym=mmUQCRB|chr13:67340605-673 (954 bp)		1.000<br>79 %

Please note that the **similarity value 1.000** marks only the two **most similar** sequences, it does not necessarily mean that these sequences are identical.

Extract Alignment Region	
**Positions in alignment**	from: to:
**Output file**	dialign.seq

Extract Aligned Sequences

For comments, questions, or bug reports, please contact support@genomatix.de.

© Genomatix Software GmbH 1998-2007 - All rights reserved. GEMS Launcher 4.4.0 License Agreement

Genomatix-Logo Logout Personal Messages GEMS-Logo
Logout Personal Messages

Genomatix_MainGEMSElDoradoGene2PromoterBiblioSphere
Frequently_asked_questionsYour_ResultsYour_SequencesYour_ProtocolHelp

GEMS Launcher Task: *DiAlign TF*: Multiple alignment plus TF sites
working on promoters_3.seq (3 seq.)

[Alignment] [Pairwise_similarities]

DiAlign professional TF Release 3.1 December 2004 **Wed Jun 8 22:14:47 2005**

Solution parameters:

**Sequence file:**	promoters_3.seq (3 seq.)
**Type of sequences:**	nucleotide sequences
**Threshold T:**	0.00
**'*' signs below alignment denote:**	diagonal similarity (max. similarity is represented by 10 '*' signs)
**Alignment output:**	complete alignment is shown<br># nucleic acids per line: 50
**TF output:**	common TF matches located in aligned regions (common to 3 (100.0 %) sequences)
**Family matches:**	yes
**MatInspector library:**	Matrix Family Library Version 5.0 (February 2005)
**Selected groups** (core/matrix sim):	• ALL user_defined.lib (0.75/Optimized)<br>• ALL vertebrates.lib (0.75/Optimized)

Aligned Sequences:

No.	Sequence Name	Sequence Description	Length
1	P889591	sym=UQCRC1|loc=Loc7384|taxid=9606|spec=Homo sapiens|chr=3|ctg=NT_022517|str=(-)|start=48586578|end=48587602|len=1025| tss=501,525|comm=ubiquinol-cytochrome c reductase core protein I;(AK129903/556/gold;NM_003365/532/silver;)	1025 bp
2	P889594	sym=Uqcrc1|loc=Loc22273|taxid=10090|spec=Mus musculus|chr=9|ctg=NT_039478|str=(+)|start=24319|end=25379|len=1061| tss=501,554,560,561|comm=ubiquinol-cytochrome c reductase core protein 1;(AK004031/554/gold;AK013128/561/gold;AK010553/561/gold;AK002292/560/gold;NM_025407/501/bronze;)	1061 bp
3	P889596	sym=na|loc=Loc301011|taxid=10116|spec=Rattus norvegicus|chr=8|ctg=NW_047802|str=(+)|start=399100|end=400100| len=1001|tss=501| comm=similar to ubiquinol-cytochrome c reductase core protein 1; (XM_217267/552/bronze;)	1001 bp

Alignment (DiAlign format):

Please note that only **upper-case** letters are considered to be **aligned**. For more information, please have a look at the user guide.

V$EGRF V$NRF1 U$NRF1 V$EVI1 V$YY1F V$NEUR V$MZF1 V$RBPF V$IKRS V$RORA V$SP1F V$MAZF V$CMYB V$VMYB

alignment position 1 . . . . 11 . . . . 21 . . . . 31 . . . . 41 . . . .

P889591 1 ggcaggagaa tcgcttgaag ccgggaggcg gaggttgcag tgagccgaga

```
P889594      1 cg-------- ---------- ---------- ---------- ----------
P889596      1 ggagtcctat ttcacaaatt acctctacac ggttccttct tttcacacac

alignment
position       51........ 61........ 71........ 81........ 91........
P889591     51 tcgcgccatt gcactccagc ctgggcgaca gagcGAGACC CTGTCTCAGA
P889594      3 ---------- -------TTT TG-------- ----GAGACA CTGTCTTACA
P889596     51 actttttttt tcttacaTTT TG-------- ----GAGACA CTGTCTTACA
                                 *** **             ****** **********
                                 *** **             ****** **********

alignment
position       101....... 111....... 121....... 131....... 141.......
P889591    101 AAAtaaaaaa taaataaaga gaaaaaaata ctgactttca gcaccaactt
P889594     24 CAA------- ---------- ---------- ---------- ----------
P889596     89 tag------- ---------- ---------- ---------- ----------
               ***

alignment
position       151....... 161....... 171....... 181....... 191.......
P889591    151 gtggtCCCAG GTAAGTTTc- ---------- ---------- ----------
P889594     27 -----CCCAG GGTAGTTTTG AACTCCTTAC ACAAGAGAGG AggaTGTCCT
P889596     92 -----CCCAG GATAGTTTTG AACTCCTTAC ACAGGAGAGG A---TGTCCT
                    ***** ********** ********** ********** *   ******
                    ***** ********** ********** ********** *   ******
                    ***** ********** ********** ********** *

alignment
position       201....... 211....... 221....... 231....... 241.......
P889591    170 ---------- ---------- ---------- ---------- ----------
P889594     72 TGAACTCCTG ATTCTTCTGT GTCTCCATTT CCCAagtgct atatatttgg
P889596    134 TGAACTCCTT ATTCTTTTTT GTCTCCTCTT CCCA------ ----------
               ********** ********** ********** ****
               ********** ********** ********** ****

alignment
position       251....... 261....... 271....... 281....... 291.......
P889591    170 ---------- ---------- ---------- ---------- ----------
P889594    122 caatatatta ttaaacgtct ttcaagtcaa ccatttaagc atattttatt
P889596    168 ---------- ---------- ---------- ---------- ----------

alignment
position       301....... 311....... 321....... 331....... 341.......
P889591    170 ---------- ---------- ---------- ---------- -------CAC
P889594    172 cttttaaact aactttagtg GACACCTTCT t--------- GCACAGACAG
P889596    168 ---------- ---------- GACACCTTCT agcacagaga GCACAGACAG
                                     **********            **********
                                                           **********

alignment
position       351....... 361....... 371....... 381....... 391.......
P889591    173 GCTGGTACTT TAGCCCTGGG ctcgaacctg cggacacgct gtggctgcaa
P889594    213 GCAAGTGCTT TAGCCCTTGG GCTCAATtgt gcct-AGC-- ----------
P889596    198 AAAAGTGCTT CAGCTTTTGG GCTTAATgtg tgctgAGC-- ----------
               ********** ********** *******          ***
               ********** ********** *******          ***

alignment
position       401....... 411....... 421....... 431....... 441.......
P889591    223 ctCCCCGCCC GCCAGACCTC AGtacgcagc gcggctggtg agaaacataa
P889594    250 --CACTGCCC TCCAGGTTTC AGAGCTGCTG CTGGCGTACa atgcATCTCC
P889596    236 --CACTGTCC TCCAGGGCTC TGAGCTGCTG CTGGCTTAC- ----ATCTCC
                 ******** ********** ********** *********      ******
                 ******** ********** ********** *********      ******
                                                               ******
```

```
alignment
position       451....... 461....... 471....... 481....... 491.......

P889591   273  ------TGCA CTCTGACTTC CACGCGTGGA ATGGGGAGAT GGActgcacg
P889594   298  CACAGTTGCA TACCGCCTCC CTCAGGTGGA AGAAGGTGAT GAATAGAACC
P889596   279  TGCAGTTGCA CACCGCCTCC CTCAGGTGGA AGAAGGCGAC GAATGGGACC
               ********** ********** ********** ********** **********
               ********** ********** ********** ****
               ********** ********** ********** ****

alignment
position       501....... 511....... 521....... 531....... 541.......

P889591   317  gcgggcggac ctgctcgggc tgatggacg- ------GCAG GTGG------
P889594   348  tgtctcagat gggaccagat gAAGGGCCCC TCCCTCGCAG GTTgataaat
P889596   329  gtct------ ---------- -AAGGGCCAG CCAGTCGCAG GTTC------
                                      ********* ********** ****

alignment
position       551....... 561....... 571....... 581....... 591.......

P889591   354  ---------- ACTGATGTGC GCAGGGACtg gcggcagcgc ggtcagagcc
P889594   398  gtcccgaaga TCCTAGAGGC ACACGGACCA AATGACTACA TGCGATcAAT
P889596   356  ---------- TCTTAGAGGC ACAAGGACCA AACGACAACA TGCGATtAAT
                          ********** ********** ********** ****** ***
                          ********** ********** ********** ****** ***

alignment
position       601....... 611....... 621....... 631....... 641.......

P889591   394  agtcagccaa agccaggcca gcacaataga ctgtcccggt tcccgccagg
P889594   448  TCTTGCTAAC GTACTTGCTG CCCAACAGGG ---------- ----------
P889596   396  TCTTGCCAAT GGACTTGATG CCCAGCAGGG ---------- ----------
               ********** ********** **********
               ********** ********** **********

alignment
position       651....... 661....... 671....... 681....... 691.......

P889591   444  aGGCGGCCGA GCACCAACTG TACGGTACTG CGCCTGCGCC gcgaccgcca
P889594   478  -GGCGACTGA GCACCAAGTC TTCCTTACAG CGCTTGCGCC TCAGACT---
P889596   426  -GGCGATCGA GCACCAACTC TTCCTTACAG CGCCTGCGCC TCATACT---
                ********* ********** ********** ********** *******
                ********* ********** ********** ********** *******
                ********* ********** ********** ********** *******
                *****   ** ********** ********** **********
                *****   ** ********** ********** **********

alignment
position       701....... 711....... 721....... 731....... 741.......

P889591   494  acgcgcCCAG TCTACGCTTG CGCGGCGCaa cagg--GCCG ACTGCAGCTG
P889594   524  ------Ccgc TCTGCGCATG CGCAGCGCGG CTCTACACTG ACGGAAGTTA
P889596   472  ------CCAC CCTGCGCATG CGTAGCGCGG CTTTACGCTG ACGGAAGTTA
                     **** ********** ********** ********** **********
                     *      ******** ********** ********** **********
                     *      ******** ********** ********** **********
                                                      **** **********
                                                         * **********
                                                                    *
                                                                    *

alignment
position       751....... 761....... 771....... 781....... 791.......

P889591   542  GAAGATGGCG GCGTCCGTGG TCTGTCGGGC CGCTACCGCC GGGGCACAAG
P889594   568  GAAGATGGCG GCGTCCGCAG TCTGCCGAGC GGCCTGCTCc GGGACGCAAG
P889596   516  GAAGATGGCG GCGTCCGCAG TCTGCCGAGC GGCCTGCTCT GGGACGCAAG
               ********** ********** ********** ********** **********
               ********** ********** ********** *********  **********
               ********** ********** ********** *********  **********
               ********** ********** ********** *********        ****
               ********** ********** *******                     ****
               ********** ********** ****
               ********** ********** ****
```

```
alignment
position        801.......  811.......  821.......  831.......  841.......

P889591     592 TGCTATTGCG  CGCCCGCCGC  TCGGTGAGGT  GGTGGCGAGA  gcgcggggct
P889594     618 TGCTACTTCG  CACCCGCCGC  TCGGTGAGGT  GGCAGTGAGA  CGTTGGGCTG
P889596     566 CGCTACTTCG  CACCTGCCGC  TCGGTGAGGT  GGCAGCGAGA  CGTTGGGCTG
                **********  **********  **********  **********  **********
                **********  **********  **********  **********  **********
                **********  **********  **********  **********  **********
                **********  **********  **********  **********
                **********  **********  **********  **
                 *********  **********  **********  **
                 *********  **********  **********  **

alignment
position        851.......  861.......  871.......  881.......  891.......

P889591     642 tc-GGGACAG  GGCTTCGGGA  AGCGGAc-AG  GTGTGGGCTG  Tt--AGCAGT
P889594     668 CAGGGCCCAG  GGCTTTcgtA  CACAGATGAG  GAGCAGGCTG  TCTCAGCAGT
P889596     616 CAGGGCTCAG  GGCTTTGGCA  CGCAGATGAG  GAGTAGGCTG  TCTCAGCAGT
                **********  **********  **********  **********  **********
                **********  ******   *  **********  **********  **********
                **********  ******   *  **********  **********  **********
                                                                    ******
                                                                    ******

alignment
position        901.......  911.......  921.......  931.......  941.......

P889591     688 GGCAGGTCGA  GGTGGGGAGG  ACGC-GGGGT  CAGGGCCGTG  GGAAGGTCAC
P889594     718 GGCGGGTCGG  AGTGGGGAGG  TCGCGGGGGT  CAGGGCCGTG  GGAAGGTCAT
P889596     666 GGCGGGTCTG  AGTGGGGAGG  TCGCGGGGGT  CAGGGCCGTG  GGAAGGTCAT
                **********  **********  **********  **********  **********
                **********  **********  **********  **********  **********
                **********  **********  **********  **********  **********
                ******** *  **********  **********  **********  **********
                ******** *  **********  **** *****  **********  **********
                         *  **********  ****  ****  **********  **********
                                             *****  **********  **********
                                             *****  **********  **********
                                             *****  **********  **********
                                             *****  **********  *********

alignment
position        951.......  961.......  971.......  981.......  991.......

P889591     737 GGCGGAGGGC  GGGGAaaggt  atctc-----  ----------  ----GCCGCT
P889594     768 GGCGGAGGGC  GGGGATGGTT  ATCTGCTCTG  GCTTGGgttT  GAGAGCCGAG
P889596     716 GGCGGAGGGC  GGGGATGGGT  ATCTTCTCGG  GCTTGGatgT  GAGAGTCGAG
                **********  **********  **********  ******   *  **********
                **********  **********  **********  ******   *  **********
                **********  **********  **********  ******   *  **********
                **********  *****
                **********  *****
                **********  *****
                **********  *****
                **********  *****
                **********  *****

alignment
position        1001......  1011......  1021......  1031......  1041......

P889591     768 TGCCTAGGGG  CGGGGTTCGG  GGGTCCTCCT  GAGGACctgg  gaggtgggga
P889594     818 GTCCCAGAGG  CTTGGCGAGC  AGTTCTTGCA  ATGGTCGCCT  TCTCCCTTT-
P889596     766 GTCCCAGAGG  CTTGGAGAGC  AGTTCTTACA  GTGGTCACCT  TCTTCCTTT-
                **********  **********  **********  **********  *********
                **********  **********  ******* **  **********  *********
                **********  **********  ******* **  **********  *********

alignment
position        1051......  1061......  1071......  1081......  1091......

P889591     818 gcgatcccct  gaccgagtcc  tcactcgcgg  ctgttccctt  ccacAGCCGG
P889594     867 ----------  ----------  ----------  ----------  ----AGCCGG
P889596     815 ----------  ----------  ----------  ----------  ----AGCCGG
```

```
                                                              ******
                                                              ******
                                                              ******
                                                              ******
                                                              ******
                                                              ******

alignment
position         1101...... 1111...... 1121...... 1131...... 1141......

P889591      868 CCCTGCTGCG GACGCCAGCC TTGCGGAGTA CgGCAACCTT CGCTCAGGCG
P889594      873 CCCTGCTGAG GTTACCTGCC TTGCGGGGTA CCGCAACCTT CGCCCAGGCC
P889596      821 CCCTGCTGAG GCTACCTGCC TTGCGGGGTA CCGCAACCTT CGTCCAGGCC
                 ********** ********** ********** ********** **********
                 ********** ********** ********** ********** **********
                 ********** ********** ********** ********** **********
                 ********** * ******** ********** * ******** **********
                 ********** * ******** ********** * ******** ** *******
                 ********** * ******** ********** * ******** ** *******
                                                    ******** ** *******

alignment
position         1151...... 1161...... 1171...... 1181...... 1191......

P889591      918 CTCCAGTTCG TGCCGGAGAC gCAGGTTAGC CTGCTGGACA ACGGCCTGCG
P889594      923 CTCCAGAGCG TGCCGGAGAC CCAGGTCAGC ATCTTGGACA ACGGGCTGCG
P889596      871 CTCCAGAGCG TGCCGGAGAC ACAGGTCAGC GTATTGGACA ACGGGCTGCG
                 ********** ********** ********** ********** **********
                 ********** ********** ********** ********** **********
                 ********** ********** ********** ********** **********
                 ********** **********  ********* ********** **********
                 ********** **********  *********    ******* **********
                 ********** **********  *********    ******* **********
                 ********** **********  *********    ******* **********
                                                     ******* **********

alignment
position         1201...... 1211...... 1221...... 1231...... 1241......

P889591      968 TGTGGCCTCc GAGCAGTCCT CTCAGCCCAC TTGCACGGTG AGTtggggac
P889594      973 TGTGGCCTCG GAGCAGTCCT CGCATGCTAC CTGCACGGTA AGTGTgcGCC
P889596      921 TGTGGCCTCG GAGCAGTCCT CGCATCCTAC CTGCACGGTG AGTGTttGCC
                 ********** ********** ********** ********** *****  ***
                 ********** ********** ********** ********** ***    ***
                 ********** ********** ********** ********** ***
                 ********** ********** ********** ********** ***
                 *********  ********** ***
                 *********  ********** ***
                 *********  ********** ***
                 *********

alignment
position         1251...... 1261...... 1271...... 1281.....

P889591     1018 gcttttgg-- ---------- ---------- ---------
P889594     1023 AGTCCTCTAG GTGAGGCCCG CCGTGTTGTC Ccaggttgg
P889596      971 AGTCCTCCAG ATGAGGCGCG CCGTGTTGTC C--------
                 ********** ********** ********** *
                 ********** ********** ********** *
```

Pairwise similarities:

For each pairwise alignment, the similarity (relative to the maximum similarity) and the number of identical nucleic acids (in % of shorter sequence) is given. Maximum values are underlined.

	P889594 (1061 bp)	P889596 (1001 bp)
**P889591 (1025 bp)**	0.286<br>40 %	0.280<br>41 %

**P889594 (1061 bp)**		1.000 81 %

Please note that the **similarity value 1.000** marks only the two **most similar** sequences, it does not necessarily mean that these sequences are identical.

**Extract alignment region**	
**Positions in alignment**	from: to:
**Output file**	dialign.seq

Extract aligned sequences

For comments, questions or bug reports, please contact support@genomatix.de

© Genomatix Software GmbH 1998-2005 - All rights reserved. License Agreement GEMS Launcher 4.1.1

Logout Personal Messages

GEMS Launcher Task: *DiAlign TF*: Multiple alignment plus TF sites
working on promoters_4.seq (3 seq.)

[Alignment] [Pairwise_similarities]

DiAlign professional TF Release 3.1 December 2004 **Mon Jun 13 19:20:05 2005**

Solution parameters:

**Sequence file:**	promoters_4.seq (3 seq.)
**Type of sequences:**	nucleotide sequences
**Threshold T:**	0.00
**'*' signs below alignment denote:**	diagonal similarity (max. similarity is represented by 10 '*' signs)
**Alignment output:**	complete alignment is shown<br># nucleic acids per line: 50
**TF output:**	common TF matches located in aligned regions (common to 3 (100.0 %) sequences)
**Family matches:**	yes
**MatInspector library:**	Matrix Family Library Version 5.0 (February 2005)
**Selected groups** (core/matrix sim):	• ALL user_defined.lib (0.75/Optimized)<br>• ALL vertebrates.lib (0.75/Optimized)

Aligned Sequences:

No.	Sequence Name	Sequence Description	Length
1	P877126	sym=UQCRC2|loc=Loc7385|taxid=9606|spec=Homo sapiens|chr=16|ctg=NT_010393|str=(+)|start=13276560|end=13277560|len=1001| tss=501|comm=ubiquinol-cytochrome c reductase core protein II; (AK094006/501/gold;)	1001 bp
2	P877133	sym=Uqcrc2|loc=Loc67003|taxid=10090|spec=Mus musculus|chr=7|ctg=NT_039433|str=(+)|start=38538143|end=38539182|len=1040| tss=501,510,530,537,540|comm=ubiquinol cytochrome c reductase core protein 2; (AK088103/530/gold;AK004492/510/gold;AK075856/540/gold;AK077583/537/gold;AK003975/510/gold;NM_025899/501/bronze;)	1040 bp
3	P877136	sym=na|loc=Loc293448|taxid=10116|spec=Rattus norvegicus|chr=1|ctg=NW_047562|str=(+)|start=22129644|end=22130644| len=1001|tss=501| comm=similar to Ubiquinol-cytochrome C reductase complex core protein 2, mitochondrial precursor (Complex III subunit II); (XM_215042/506/bronze;)	1001 bp

Alignment (DiAlign format):

Please note that only **upper-case** letters are considered to be **aligned**. For more information, please have a look at the user guide.

Warning: No common TF matches found.

```
alignment position  1.........  11........  21........  31........  41........
P877126          1  ttgggttgag  ggcgtcccca  gcaggcacag  aaattgggtt  ggtgccaggt
P877133          1  a---------  ----------  ----------  ----------  ----------
P877136          1  tggttcccga  ----------  ----------  ----------  ----------
```

```
alignment position 51........ 61........ 71........ 81........ 91........
P877126         51 tcttaatgcc cagatcttaa ttagagagcc caagtccgtt gtaggcctgg
P877133          2 ---------- ---------- ---------- ---------- ----------
P877136         11 ---------- ---------- ---------- ---------- ----------

alignment position 101....... 111....... 121....... 131....... 141.......
P877126        101 agcaattcag acacaaggca aaaagaaagt acATTTGCCT ATGCCTTGTA
P877133          2 ---------- ---------- ---------- ---------- ----------
P877136         11 ---------- ---------- ---------- --ATTAGCCT CAACAATGTA
                                                      ******** **********
alignment position 151....... 161....... 171....... 181....... 191.......
P877126        151 ACgtctacac caccagcctt tctgaaacta ttgtgcatac gaagtcatcc
P877133          2 ---------- -------GAC TATAT----- ---------- ----------
P877136         29 ACacattaat gatttgtGAC TATGT----- ---------- ----------
                   **                ***  *****
                                     *** *****
                                     *** *****
                                     *** *****
                                     *** *****
alignment position 201....... 211....... 221....... 231....... 241.......
P877126        201 caggatcttg taaaaacacc gattcggatc caatatggga cctggagttc
P877133         10 ---------- ---------- ---------- ---------- ----------
P877136         54 ---------- ---------- ---------- ---------- ----------

alignment position 251....... 261....... 271....... 281....... 291.......
P877126        251 atacatttct aatcaactcc taaatgatat tgatgctgat actgtcggtt
P877133         10 ---------- ---------- ---------- ---------- ----------
P877136         54 ---------- ---------- ---------- ---------- ----------

alignment position 301....... 311....... 321....... 331....... 341.......
P877126        301 tcccaaccaa gcaacgtagc aaggtacgaa actctaaagt ccctaaaggg
P877133         10 ---------- ---------- ---------- ---------- ----------
P877136         54 ---------- ---------- ---------- ---------- ----------

alignment position 351....... 361....... 371....... 381....... 391.......
P877126        351 cagggaccac gcctctcgcg ttggcatagt gcctagctca aggcactttc
P877133         10 ---------- ---------- ---------- ---------- ----------
P877136         54 ---------- ---------- ---------- ---------- ----------

alignment position 401....... 411....... 421....... 431....... 441.......
P877126        401 tgggttaaca ctcgcTGAGG ACCGGAGTGA ACGCCCCCGC CCCAAGTCG-
P877133         10 ---------- -----TAAGT CCCTGATTAT ACACACACGC GCAAATTTGc
P877136         54 ---------- -----TAAGT CCCTGGTTCT ACACACACAC ACAcacacac
                                   ***** ********** ********** *********
                                   ***** ********** ********** ***
                                   ***** ********** ********** ***
                                   ***** ********** ********** ***
                                   ***** ********** ********** ***
alignment position 451....... 461....... 471....... 481....... 491.......
P877126        450 ---------- -TCACGTGAC TGACTttctc caa------- ----------
P877133         45 gcgagaaaat tTCACGAGAT TGAGTccttg ctgGTGACGA TCTCCTTGCg
P877136         89 acacacgagc a--------- ---------- ---GTGACGA TCTCCTTGCc
                               ********* *****         ******* *********
                                                       ******* *********
                                                       ******* *********
alignment position 501....... 511....... 521....... 531....... 541.......
P877126        472 ---------- ---------- ---------- ---------- -------CTG
```

```
P877133         95 gcaaggagac ctacaaGTGT GTCGATCTGT TCGACGAGAC ATTCGTCCTG
P877136        117 agcccaccta tag---GTGT GTCGACCTGT TCGACGAGAC ACTCGTCCTG
                         **** ********** ********** **********
                         **** ********** ********** **********
                         **** ********** ********** **********
                         **** ********** ********** **********
                         **** ********** ********** **********
                         **** ********** ********** **********
                         **** ********** ********** **********

alignment position 551....... 561....... 571....... 581....... 591.......
P877126        475 CCGGTTTCGG ctcacataac acacctacta tccaaaacat ttctattttc
P877133        145 CCAGTTTCtg tg-AACTCGG TCA------- ---------- ----------
P877136        164 CCGGTCTCGG tgaAACTCGG TCA------- ---------- ----------
                   **********    ******* ***
                   *****
                   *****
                   *****
                   *****
                   *****
                   *****

alignment position 601....... 611....... 621....... 631....... 641.......
P877126        525 ctaacacaaa aactatagct aagttttat  attctatgta gccccagcct
P877133        167 ---------- ---------- ---------- ---------- ----------
P877136        187 ---------- ---------- ---------- ---------- ----------

alignment position 651....... 661....... 671....... 681....... 691.......
P877126        575 aggacgtaaa ttccacgaga ccaaaagcct tgtgatgatt ccccgaggtt
P877133        167 ---------- ---------- ---------- ---------- ----------
P877136        187 ---------- ---------- ---------- ---------- ----------

alignment position 701....... 711....... 721....... 731....... 741.......
P877126        625 gctgaccctc ccggtgtgtc ccgggcctgc tctggggac  acagcggcga
P877133        167 ---------- ---------- ---------- ---------- ----------
P877136        187 ---------- ---------- ---------- ---------- ----------

alignment position 751....... 761....... 771....... 781....... 791.......
P877126        675 atacgttgaa cgaggcccct gtttcacgga gctaaagatc cagtaaggac
P877133        167 ---------- ---------- ---------- ---------- ----------
P877136        187 ---------- ---------- ---------- ---------- ----------

alignment position 801....... 811....... 821....... 831....... 841.......
P877126        725 ttcacccagc gagaggcaac tacgccgggc ttcgataccc aatacctctg
P877133        167 ---------- ---------- ---------- ---------- ----------
P877136        187 ---------- ---------- ---------- ---------- ----------

alignment position 851....... 861....... 871....... 881....... 891.......
P877126        775 cagaaaggtg gagtggcagg aatctcctga agcctccaac gtCC-AGGAA
P877133        167 ---------- ---------- ---------- ---------- --CC-AAGTT
P877136        187 ---------- ---------- ---------- ---------- --CagAAATT
                                                                 ** *****

alignment position 901....... 911....... 921....... 931....... 941.......
P877126        824 CGCGGAAACC CAGGGCCTCC AGTGCAGGTG TCatcccg-- ----------
P877133        174 CGCGGTACCG GGAAGACTAC AGTAAAAGTG GCGAGGGACG ACCTCCGAGG
P877136        195 CTCGGTACCA CTTTTACTAC AGAACAAGTG GCGAGGGATG ACCTCCCGGG
                   ********** ********** ********** ********** **********
                                                 ** ********** **********
                                                 ** ********** **********
                                                 ** ********** **********
                                                 ** ********** **********
```

```
alignment position  951....... 961....... 971....... 981....... 991.......
P877126         862 ---------- ---------- ---------- --GAGGGCGC ATGCGGAGTC
P877133         224 AACAGGGAGT GCCGACGAct cccgg-ACTT GCGAGGACTC ACGGGCAGGC
P877136         245 TATAGGAAGT TCCGACGAgt ctgggaACTG GCGCGGACTC ACGAGCAGGT
                    ********** ********         **** ********** **********
                    ********** ********         **** ********** **********
                    ********** ********         **** ********** **********
                    ********** ********         **** ********** **********
                    ********** ********

alignment position  1001...... 1011...... 1021...... 1031...... 1041......
P877126         880 CCAAGAgagg cgcgtcccat tgGACAATGC TGAGCGGAAG AGCCCAAGAT
P877133         273 CCAAGACggc tgg------- --------GG TGACTGTGAC GGCTGCAGAC
P877136         295 CCAAGACtag agttgggcgc --GGCTAAGG TGACCGTGAC GGCTGCAGAC
                    *******                 ******** ********** **********
                    *******                       ** ********** **********
                    *******                       ** ********** **********
                    *******                       ** ********** **********
                                                  ** ********** **********
                                                  ** ********** **********

alignment position  1051...... 1061...... 1071...... 1081...... 1091......
P877126         930 AGCGGGGGGA GTtgtgACAG GC-------- ---TGCTGCA GACGTACTTA
P877133         308 GCAGCTGGGA TT----ACAG GCtgcatgtc tttTGCAGAA AAAGAACTCA
P877136         343 GAAGTTGGGA TT----ACAG GC-------- ---TGCAGAA AAAGAACTCA
                    ********** **    **** **            ******* **********
                    ********** **    **** **            ******* **********
                    ********** **    **** **            ******* **********
                    ********** **    **** **            ******* **********
                    ********** **    **** **            ******* **********
                    ********** **    **** **            ******* **********
                    ********** **    **** **            ******* **********
                                     **** **            ******* **********
                                     **** **            ******* **********

alignment position  1101...... 1111...... 1121...... 1131...... 1141......
P877126         969 GAAAAGgggt ---------- ---------- ---------- ----------
P877133         354 GAAAGGATAA CCTacaaaag ctgcaatcta gtccgggaga cctctagcct
P877136         378 GAAAGGATAA CCTtaacatg ctgcgtgctc gcaagactca tccg------
                    ********** ***
                    ********** ***
                    ********** ***
                    ********** ***
                    ********** ***
                    ********** ***
                    ********** ***
                    ****
                    ****

alignment position  1151...... 1161...... 1171...... 1181...... 1191......
P877126         979 ---------- ---------- ---------- ---------- ----------
P877133         404 ctccccagga gctgggctcg tcccGCCTAC CGGAGCAGTA GAGCACTGCA
P877136         422 ---------- ---------- ----GCCTTC CGGAGCAGTA GAGCACAGAG
                                              ****** ********** **********
                                              ****** ********** **********
                                              ****** ********** **********
                                              ****** ********** **********
                                              ****** ********** **********
                                              ****** ********** **********

alignment position  1201...... 1211...... 1221...... 1231...... 1241......
P877126         979 ---------- ---------- ---------- ---------- ----------
P877133         454 TGCGTAGAGG GCCGccggta CAAGGGGGCG TGGGCAAGCT CGCAGTGCAC
P877136         448 TGCGTAGAAG GCCGctcggc CAAGGGGGCG TGGGCAAGCT CGCAGTGCAC
                    ********** ****       ********** ********** **********
                    ********** ****       ********** ********** **********
                    ********** ****       ********** ********** **********
                    ********** ****       ********** ********** **********
```

```
                         ********** ****       ********** ********** **********
                         ********** ****       ********** ********** **********
                                               ********** ********** **********
                                               ********** ********** **********
                                               ********** ********** **********
alignment position       1251...... 1261...... 1271...... 1281...... 1291......
P877126          979     ----GCATAC TGGATATTCA GGCtagg--- ---------- ----------
P877133          504     ACCGGGAAAC tcGGTCCTCC GACCCCATCT TGCTTTGCTG TCTGCAGCgg
P877136          498     GCCGGGAAAC TTGGTCTTCC GGCACCATCT TGCTTTGCTG TCTGCAGCtt
                         ********** ********** ********** ********** ********
                         **********   ******** ********** ********** ********
                         **********   ******** ********** ********** ********
                         **********   ******** ********** ********** ********
                         **********   ******** ********** ********** ********
                         **********   ******** ********** ********** ********
                         **********
                         **********
                         **********
alignment position       1301...... 1311...... 1321...... 1331...... 1341......
P877126         1002     ---------- ---------- ---------- ---------- ----------
P877133          554     TGACCGTGGG AAAGGGCAAC TGCTAGAGCC ATGAAGCTCC TCAGCAGGGC
P877136          548     TGACCGTAGG AAAGGGCAAC TGCTAGAGCC ATGAAGCTCC TCAGCAGGGC
                         ********** ********** ********** ********** **********
                         ********** ********** ********** ********** **********
                         ********** ********** ********** ********** **********
                         ********** ********** ********** ********** **********
                         ********** ********** ********** ********** **********
                         ********** ********** ********** ********** **********
                         ********** ********** ********** ********** **********
                                                              ****** **********
                                                              ****** **********
                                                              ****** **********
alignment position       1351...... 1361...... 1371...... 1381...... 1391......
P877126         1002     ---------- ---------- ---------- ---------- ----------
P877133          604     CGGGTCCTTC TCGGTGAGCA TGGGaCGTGC GTGGGGCAGG GCTGGGGACG
P877136          598     CGGGTCCTTC TCGGTGAGCA TGGGgCGCGC GCGGGTCAGG GCTGGGGACA
                         ********** ********** **** ***** ********** **********
                         ********** ********** **** ***** ********** **********
                         ********** ********** **** ***** ********** **********
                         ********** ********** **** ***** ********** **********
                         ********** ********** ****
                         ********** ********** ****
                         ********** ********** ****
                         ********** ********** ****
                         ********** ********** ****
                         ********** ********** ****
alignment position       1401...... 1411...... 1421...... 1431...... 1441......
P877126         1002     ---------- ---------- ---------- ---------- ----------
P877133          654     CTTGTCgGCC ACTCAGGAGC TGGGTGCGGA GTGTGCGGCC tTGGCCTCAG
P877136          648     CTTGTCaGCC ACTCAGGGGC TGGCAGCGGA GTGTGCGGCC cTGGCCTCAA
                         ****** *** ********** ********** **********  *********
                         ****** *** ********** ********** **********  *********
                         ****** *** ********** ********** **********  *********
                         ****** *** ********** ********** **********  *********
                                *** ********** ********** **********  *********
                                                                      *********
                                                                      *********
alignment position       1451...... 1461...... 1471...... 1481...... 1491......
P877126         1002     ---------- ---------- ---------- ---------- ----------
P877133          704     GCCTGGGTCT GCCCTTCAGG TAAACCCcGC GGAGAAGGGG GCTGCCAGCG
P877136          698     GCCTGGGTCT GCCCTTCAGG TTAACCCgGC GGACAAGCGT GCTGCCAGCA
                         ********** ********** ******* ** ********** **********
                         ********** ********** ******* ** ********** **********
                         ********** ********** ******* ** ********** **********
                         ********** ********** ******* ** ********** **********
```

```
                            ********** ********** ******* ** ********** **********
                            ********** ********** *******
                            ********** ********** *******

alignment position          1501...... 1511...... 1521...... 1531...... 1541......
P877126             1002    ---------- ---------- ---------- ---------- ----------
P877133              754    AGCCGAGGCC TGGGAgGCTC GGCGGGTCCT GTGAGCAGcc gagagctaac
P877136              748    AGCTGAGGCC TGGGA-GCTC GGCGGGTTCT GTGAGCAGga agagaactga
                            ********** ***** **** ********** ********
                            ********** ***** **** ********** ********
                            ********** ***** **** ********** ********
                            ********** *****
                            ********** *****

alignment position          1551...... 1561...... 1571...... 1581...... 1591......
P877126             1002    ---------- ---------- ---------- ---------- ----------
P877133              804    gcgg-GGAAA GCGGAAGAGG AGGGTGGCCT GGAGAGTGGC GGAGAagCGG
P877136              797    ctcccGGAAA GCCGGAGAGA AGGGTGGCCT TGGGAGTGGC GGAGAgtCGG
                                 ***** ********** ********** ********** *****  ***
                                 ***** ********** ********** ********** *****  ***
                                 ***** ********** ********** ********** *****  ***
                                 ***** ********** ********** ********** *****  ***
                                 ***** ********** ********** ********** *****  ***
                                 ***** ********** ********** ********** *****  ***
                                                                               ***

alignment position          1601...... 1611...... 1621...... 1631...... 1641......
P877126             1002    ---------- ---------- ---------- ---------- ----------
P877133              853    CTCTCGGGAG ATTCCTGAAG GTCACTCGAG AAGCaGGTAG GGTGTAGGCC
P877136              847    GTCTCAGGAG ACTCCTGAAG GTCACTCGAG AAGCtGGTAG GGAGAAGGCC
                            ********** ********** ********** **** ***** **********
                            ********** ********** ********** **** ***** **********
                            ********** ********** ********** **** ***** **********
                            ********** ********** ********** **** ***** **********
                            ********** ********** ********** ****
                            ********** ********** ********** ****
                            ********** ********** ********** ****

alignment position          1651...... 1661...... 1671...... 1681...... 1691......
P877126             1002    ---------- ---------- ---------- ---------- ----------
P877133              903    AACGCCACCC GGGCCGCCTG CTTcccctac ctacgacCTC TGCCCCTGGG
P877136              897    AACGACACCC GAGCCTCTTA CTTttccagc agagat-CTC TGCTCCTGGG
                            ********** ********** ***               *** **********
                            ********** ********** ***               *** **********
                            ********** ********** ***
                            ********** ********** ***

alignment position          1701...... 1711...... 1721...... 1731...... 1741......
P877126             1002    ---------- ---------- ---------- ---------- ----------
P877133              953    TTGATCCcga GGCGTCGGTT TCCCCAATGC GTGCGAGTTT ATTAaattca
P877136              946    TTTATCCtcg GGCATCAGTT TCCCCAGTGC CTGCAAGTAT AATAtaataa
                            *******    ********** ********** ********** ****
                            *******    ********** ********** ********** ****
                                       ********** ********** ********** ****

alignment position          1751...... 1761...... 1771...... 1781....
P877126             1002    ---------- ---------- ---------- --------
P877133             1003    caaacgcggt gcgctgctta tctcagtgcc ggcacctt
P877136              996    atgcac---- ---------- ---------- --------
```

Pairwise similarities:

For each pairwise alignment, the similarity (relative to the maximum similarity) and the number of identical nucleic acids (in % of shorter sequence) is given. Maximum values are underlined.

	P877133 (1040 bp)	P877136 (1001 bp)
**P877126 (1001 bp)**	0.011<br>8 %	0.015<br>9 %
**P877133 (1040 bp)**		1.000<br>71 %

Please note that the **similarity value 1.000** marks only the two **most similar** sequences, it does not necessarily mean that these sequences are identical.

**Extract alignment region**	
**Positions in alignment**	from: to:
**Output file**	dialign.seq

Extract aligned sequences

For comments, questions or bug reports, please contact support@genomatix.de

© Genomatix Software GmbH 1998-2005 - All rights reserved.

License Agreement

GEMS Launcher 4.1.1

Genomatix-Logo Logout Personal Messages GEMS-Logo

Logout Personal Messages

Genomatix_MainGEMSElDoradoGene2PromoterBiblioSphere
Frequently_asked_questionsYour_ResultsYour_SequencesYour_ProtocolHelp

GEMS Launcher Task: *DiAlign TF*: Multiple alignment plus TF sites
working on promoters_4.seq (3 seq.)

[Alignment] [Pairwise_similarities]

DiAlign professional TF Release 3.1 December 2004 **Mon Jun 13 21:20:39 2005**

Solution parameters:

**Sequence file:**	promoters_4.seq (3 seq.)
**Type of sequences:**	nucleotide sequences
**Threshold T:**	0.00
**'*' signs below alignment denote:**	diagonal similarity (max. similarity is represented by 10 '*' signs)
**Alignment output:**	complete alignment is shown<br># nucleic acids per line: 50
**TF output:**	common TF matches located in aligned regions (common to 3 (100.0 %) sequences)
**Family matches:**	yes
**MatInspector library:**	Matrix Family Library Version 5.0 (February 2005)
**Selected groups** (core/matrix sim):	• ALL user_defined.lib (0.75/Optimized)<br>• ALL vertebrates.lib (0.75/Optimized)

Aligned Sequences:

No.	Sequence Name	Sequence Description	Length
1	P932494	sym=UQCRFS1|loc=Loc7386|taxid=9606|spec=Homo sapiens|chr=19| ctg=NT_011109|str=(-)|start=1971832|end=1972832|len=1001| tss=501| comm=ubiquinol-cytochrome c reductase, Rieske iron-sulfur polypeptide 1; (NM_006003/502/silver;)	1001 bp
2	P932495	sym=Uqcrfs1|loc=Loc66694|taxid=10090|spec=Mus musculus| chr=13|ctg=NT_039578|str=(-)|start=17240566|end=17241620| len=1055|tss=501,505,543,555| comm=ubiquinol-cytochrome c reductase, Rieske iron-sulfur polypeptide 1; (AK014470/539/gold;AK012180/589/gold;AK003966/577/gold; NM_025710/535/silver;)	1055 bp
3	P932496	sym=na|loc=Loc291103|taxid=10116|spec=Rattus norvegicus| chr=17|ctg=NW_047491|str=(+)|start=14745436|end=14746436| len=1001|tss=501| comm=similar to RIKEN cDNA 4430402G14; (XM_214457/508/silver;)	1001 bp

Alignment (DiAlign format):

Please note that only **upper-case** letters are considered to be **aligned**. For more information, please have a look at the user guide.

V$SNAP V$EGRF V$ETSF V$CREB V$RORA V$VMYB V$E2FF V$DEAF V$REBV V$EBOX V$NOLF V$EKLF
V$MINI V$NRF1 U$NRF1 V$ZF5F V$HICF V$LEFF

```
alignment
position         1.........  11.........  21.........  31.........  41.........

P932494       1  a---------  ----------  ------ACAAA  TGAAagaacg  taggtataat
P932495       1  ----------  ----------  --------AAA  TGAACAACGA  AGGAAACTAC
P932496       1  gtgttatttc  ctcaagatcg  taattACAAA  TGAACAATGA  AGGAAACTAC
```

```
                                          ***** ********** **********
                                            *** ********** **********
                                            *** ********** **********

alignment
position      51........ 61........ 71........ 81........ 91........
P932494    27 aaaacgcaAA ACCAAATACA CAGTGAATAC TTAATAAACA TAATCCAtta
P932495    24 C-------AA ATCAAAAGCG TAGTAGATCA TGAATAATCG TAAGCCACcg
P932496    51 C-------AA ATCCAAAGCG TGGTGTATGA TGAAAAATTG TAAGCCACg-
              *       ** ********** ********** ********** ********
              *       ** ********** ****
              *       ** ********** ****
                      ** ********** ****

alignment
position      101....... 111....... 121....... 131....... 141.......
P932494    77 gc-------- ---------- -------ATT ATCGAGCTTT GCACAGATGC
P932495    67 ttttgttttg ttttgttttg ttttGTTTTT GTCAAACCTT GCATATAGGT
P932496    93 ---------- ---------- ----GATATT GTCAAATTTT GTATAAAGGT
                                        ****** ********** **********
                                            ** ********** **********
                                                            ********
                                                            ********

alignment
position      151....... 161....... 171....... 181....... 191.......
P932494   102 CTCTGGCCTT TTTGTTGTtg ttgttgttag atgAAGATAA TAAAAATCAT
P932495   117 TTCTGGCTCT TTTGTTGT-- ---------- ---AAGAAGA TCAAAATCGT
P932496   119 TTCTGGCCTT TTTGTTTT-- ---------- ---AAGATGA TAAAATTTGT
              ********** ********               ******* **********
              ********** ********               ******* *****   **
              ********** ********               ******* *****   **
              ********** ******

alignment
position      201....... 211....... 221....... 231....... 241.......
P932494   152 CATAGTAAAC ac---ATAGC GATTACTATG TGCTGGACTT CGTCGTAGTT
P932495   152 CTTGGCAAAA GACAAAAAGC GGTTGGTATT TATTCTAGGT GATAGGCGTT
P932496   154 CATAGCAAAA GACAGAAAGT GGCTGGTATT TATTCTACAT GATAGGCGTT
              ********** ********** ********** ********** **********
              ********** ********** ********** *******  * **********
              ********** ********** ********** *******  * **********

alignment
position      251....... 261....... 271....... 281....... 291.......
P932494   199 GAGATATATT ACCATCctaa gttaaactat aatcgT---T TATTGTTATC
P932495   202 AGGAAGTAGT AGTAGCCTGT TGTTTtt--- -----T---T TTTTCTCATG
P932496   204 AGGAAGTAGT AATAGCCTGT TCTTTaactc tgtaataacT TTTTCTCATG
              ********** ********** *****           *   * **********
              ********** ********** *****               * **********
              ********** ********** *****               * **********
                                                        * **********

alignment
position      301....... 311....... 321....... 331....... 341.......
P932494   246 TCTAGGTCTT CCTAGATGAA ATTTTTa-CC CTAAAGGAtc gtaaagtcgg
P932495   241 CCTCGGTCGT CCCCGATTTA AGGTTTtGCC CTAAATGAAG GATCTCAATC
P932496   254 CCTAGGTCGT CCCTGATTTA AGTTTTcGCC GTAAACGAAG GATCTCAATC
              ********** ********** ****** *** ********** **********
              ********** ********** ****** *** ********** **********
              ********** ********** ****** *** ********** **********
              ********** ********** ******

alignment
position      351....... 361....... 371....... 381....... 391.......
P932494   295 cccac----- ---------- ---------- ---------- ----------
P932495   291 ACGACCACAA GAAatccctc ccGACTTCGG TTTTCCTGGG GCAGGGCGAC
P932496   304 ACGACCACAA GAAgttgcta ctGACTTCTG TTTTCCTGGG GCAAAGCGAC
```

```
                 ********** ***          ******** ********** **********
                 ********** ***          ******** ********** **********
                 ********** ***          ******** ********** **********

alignment
position         401....... 411....... 421....... 431....... 441.......

P932494      300 -----CGGAC GTCAGTGGGG TCCC-CTGTT CAGCCCTCTC GTTCttctgg
P932495      341 CGAGTCAGAC ACCAGAGTGT CTCC-AGGCC CTGCCCTTTC CTGCCC----
P932496      354 CAAGTCAGAC cccagcgtca gcgttAGGTA CTTCCCTTTC CCCTCC----
                 ********** ********** ****  ***** ********** ******
                 **********
                 **********

alignment
position         451....... 461....... 471....... 481....... 491.......

P932494      344 ggatctctcg ggacactgac gaggtggcac cgggacctac aggtGGGCCG
P932495      386 ---------- ---------- ---------- ---------- ----GGGCCC
P932496      400 ---------- ---------- ---------- ---------- ----CGCCCG
                                                                 ******
                                                                 ******

alignment
position         501....... 511....... 521....... 531....... 541.......

P932494      394 CAAGCCCCGC CCCCAGCGAC Ggcttgtagc cccgtCCCCC GACGCAGCGC
P932495      392 CAACCTGCGC CCGAAGCCAC G--------- -----CCCCC AACGCGGCGT
P932496      406 CAATTTGCGC CCGTAGCCAC G--------- -----CCTCC AACGCGGCAA
                 ********** ********** *               ***** **********
                 ********** ***   ****** *               ***** **********
                                  ****** *               ***** **********

alignment
position         551....... 561....... 571....... 581....... 591.......

P932494      444 CTCTCCAAGT TTGCCCCGCC CTGCAggact gcagAATTTC CTTTCCGGCG
P932495      428 CCCTCCCGAT TCGCTCAACC TTGCGA---- ----AACGCC CTTTCCGGCG
P932496      442 TCCTCCGGAT TCGCTTAGCC TTACGA---- ----AATTCC CTTTCCGGCG
                 ********** ********** ******         ****** **********
                 ********** *****   ** ******         ****** **********
                 ********** *****   ** ******         ****** **********
                                    ** *****          ****** **********
                                                      ****** **********
                                                           * **********

alignment
position         601....... 611....... 621....... 631....... 641.......

P932494      494 GCGCGCTGTC GTCAC----- ----GCGTCC CGCGCCTCCT CGCGCCTGCG
P932495      470 TCGCGCTGAC GTCACAaacC CTGCGCCTCC CGCTCCTCTT CGGGCCgtCG
P932496      484 TCGCGCTTAC GTCACAagtC CTGCGCCTCC CGCTCCTCTT CGGGCCTGCG
                 ********** ******   * ********** ********** **********
                 ********** ******   * ********** ********** ******  **
                 ********** ******   * ********** ********** ******  **
                 ********** *****        ****** ********** ******  **
                 ********** *****
                 ********** ****

alignment
position         651....... 661....... 671....... 681....... 691.......

P932494      535 ccgtggttGG AAGGTCGTCC CTGTGACCGG CTTGGCGGTT GGAGCGGCTG
P932495      520 TTCGAAC-GA AAGGTCGTCC CTGTGACCGA CGAGGTGGTT TGAGCAGCTG
P932496      534 TTCGAAC-GG AAGGTCGTCC CTGTGACCGA CTAGGTaGTT GGAGCAGCTG
                 *******  ** ********** ********** ********** **********
                 *******  ** ********** ********** ********** **********
                 *******  ** ********** ********** ********** **********
                          ** ********** ********** ******  *** **********
                          ** ********** ********** ******  *** **********
                             ********** *********     **   *** **********
                                                      **   *** **********

alignment
position         701....... 711....... 721....... 731....... 741.......

P932494      585 TCGCCATGTT GTCGGTAGCa tCCCGCTCAG GCCCGTTCGC GCCCGTCCTG
```

```
P932495   569 TCGCCATGTT GTCGGTCGCc GCCCGCTCGG GCCCGTTCGC GCCCGTCCTA
P932496   583 TCGCCATGTT GTCGGTCGCt GCCCGCTCGG GCCCGTTCGC GCCCGTCCTA
              ********** *********  ********** ********** **********
              ********** *********  ********** ********** **********
              ********** *********  ********** ********** **********
              ********** *********  ********** ********** **********
              ********** *********   ********* ********** **********
              ********** *********   ********* ********** **********
              ********** *********   ********* ********** **********
                                     ********* ********** **********
                                     ********* ********** **********
                                     ********* ********** **********

alignment
position          751.......  761.......  771.......  781.......  791.......

P932494   635 TCGGCCACGT CCCGCGGGGT GGCGGGCGCG CTGCGGCCCT TGGTGCAggc
P932495   619 TCGGCCACtT CCCGCGGGGT GGCGGGCGCG CTGCGTCCTC TGCTGCAAgG
P932496   633 TCGGCCACGT CCCGCGGGGT GGCGGGAGCG CTGCGGCCCC TGCTGCAAaG
              ********** ********** ********** ********** ******** *
              ********** ********** ********** ********** ******** *
              ********** ********** ********** ********** ******** *
              ********** ********** ********** ********** *******
              ******** * ********** ********** ********** *******
              ******** * ********** ********** ********** *******
              ******** * ********** ********** ********** *******
              ******** * ********** ********** ********** *******
              ******** * *
              ********

alignment
position          801.......  811.......  821.......  831.......  841.......

P932494   685 caCGGTGCCC GCCACCCCGG AGCAGCCTGT GTTGGACCTG AAGCGGCCCT
P932495   669 CGCGGTGCCC GCCGCCTCGG AGCCACCTGT TCTGGATGTG AAGCGACCCT
P932496   683 CGCGGTGCCC GCCACCTCGG AACCACCGGT TCTGGACGTG AAGCGACCCT
              ********** ********** ********** ********** **********
              ********** ********** ********** ********** **********
              ********** ********** ********** ********** **********
                ******** ********** ********** ****** *** **********
                ******** ********** ********** ****** *** **********
                ******** ********** ********** ****** *** **********
                                                      *** **********
                                                            ********

alignment
position          851.......  861.......  871.......  881.......  891.......

P932494   735 TCCTCAGCCG GGAGTCGCTG AGCGGCCAGG CCgtgcgCCG GCCTTTGGTC
P932495   719 TCCTGTGCCG CGAGTCCCTG AGTGGCCAGG CCGCAGCCCG GCCTTTGGTG
P932496   733 TCCTGTGCCG TGAGTCGCTG AGTGGCCAGG CCGCGACCCG TCCTTTGGTG
              ********** ********** ********** ********** **********
              ********** ********** ********** ********** **********
              ********** ********** ********** ********** **********
              ********** ********** ********** **     *** **********
              ********** ********** ********** **     *** **********
              ********** ********** ********** **     *** **********
              ********** ********** ********** **     *** **********
              ********** ********** *******           *** **********

alignment
position          901.......  911.......  921.......  931.......  941.......

P932494   785 GCCTCCGTGG GCCTCAATGG TGAGCCG--- ---------- ----------
P932495   769 GCCACGGTGG GCCTGAACGG TGAGCCGGCT TCGGAGTACC CCGATCTCGT
P932496   783 GCCACAGTGG GCCTGAATGG TGAGCCGGCT TCAGGGTGCC CCGATCTCGT
              ********** ********** ********** ********** **********
              ********** ********** ********** ********** **********
              ********** ********** ********** ********** **********
              ********** ********** *******
              ********** ********** *******
              ********** ******* ** *******
              ********** ******* ** *******
              ********** *******
```

```
alignment
position           951.......  961.......  971.......  981.......  991.......

P932494      812   ----------  ----------  --------GG  GCGAGGGCCG  CGAGCAGGGC
P932495      819   TCCCt-----  ----------  ---GCGGCGG  CGGAGGGCGG  TGTGCTGGGA
P932496      833   TTCCctgcgg  ctccaggtcc  gggGCGGCGG  CAGAGGGCGG  TGCGCTGGGA
                   ****                       *******  **********  **********
                   ****                       *******  **********  **********
                   ****                       *******  **********  **********

alignment
position           1001......  1011......  1021......  1031......  1041......

P932494      834   TGGGGgtctc  tcggtg----  ----------  ----------  ----------
P932495      851   TGTGGTGGgc  cGGCCGCGGC  TACCTTGGCA  GGCCGAGCGC  tccccgGAGC
P932496      883   TGTGGTGGac  tGGCCGCAGC  GACCTTGGCA  GGCCCAGCGC  ctcttcGAGC
                   ********     *********  **********  **********        ****
                   ********     *********  **********  **********        ****
                   ********

alignment
position           1051......  1061......  1071......  1081......  1091......

P932494      850   ----------  ----------  ----------  -------CCC  GCTCCTCCGG
P932495      901   AGGCGGGCTC  ACGGTGAC--  ---------G  GGATGCTCCC  GTAGCTGCAT
P932496      933   AGGCGGGCTC  ACGGTGACcg  gatgccgctG  GGATGCTCCA  GGGGCCGCAT
                   **********  ********             *  **********  **********
                   **********  ********             *  **********  **********
                                                              ***  **********

alignment
position           1101......  1111......  1121......  1131......  1141......

P932494      863   CCCCCAGCAG  GGAGCGGCCG  cgggcgcgcg  agcggagcct  gaatCAGGTC
P932495      940   CCCCCAGGAG  TCAGGAGCCG  gtctg-----  ----------  ----CACGTC
P932496      983   CCCCCAGGCG  TCAGGAACC-  ----------  ----------  ----------
                   **********  **********                              ******
                   **********  *********
                   **********  *********

alignment
position           1151......  1161......  1171......  1181......  1191......

P932494      913   GGGCTGGGCG  GCCTCCGCAa  ctctgggcct  ggccccgggg  ctcggggcgt
P932495      971   AGTCTGGGTT  CCCTCCTCAg  gcttcacttg  actttcttga  ttaagatcgg
P932496     1002   ----------  ----------  ----------  ----------  ----------
                   **********  *********

alignment
position           1201......  1211......  1221......  1231.....

P932494      963   ctttctggtg  gccggcctgg  actcgcgtcc  gccggcttc
P932495     1021   cctccctgta  tcatggctaa  gatactgtta  gttac----
P932496     1002   ----------  ----------  ----------  ---------
```

Pairwise similarities:

For each pairwise alignment, the similarity (relative to the maximum similarity) and the number of identical nucleic acids (in % of shorter sequence) is given. Maximum values are underlined.

	P932495 (1055 bp)	P932496 (1001 bp)
P932494 (1001 bp)	0.342<br>49 %	0.362<br>43 %
P932495 (1055 bp)		<u>1.000</u><br><u>78 %</u>

Please note that the **similarity value 1.000** marks only the two **most similar** sequences, it does not necessarily mean that these sequences are identical.

Extract alignment region	
**Positions in alignment**	from: to:
**Output file**	dialign.seq

Extract aligned sequences

For comments, questions or bug reports, please contact support@genomatix.de

© Genomatix Software GmbH 1998-2005 - All rights reserved.

License Agreement

GEMS Launcher 4.1.1
