# Supplementary material for: Transcriptional co-expression and co-regulation of genes coding for components of the oxidative phosphorylation system
Source: BMC Genomics. 2008 Jan 14;9:18. doi: 10.1186/1471-2164-9-18 (PMC2268925; doi:10.1186/1471-2164-9-18)
Supplement: Additional file 7 — Phylogenetic footprinting of Complex IV core promoters. Core Promoters of Complex IV subunits/related genes identified by phylogenetic footprinting. The conserved regions are shown in color codes, which correspond to specific Transcription Factor Binding Sites (TFBS). [file 1471-2164-9-18-S7.pdf]

This Supplementary Material shows the Core Promoters of Complex IV subunits/related genes identified by phylogenetic footprinting. The conserved regions are shown in color codes, which correspond to specific Transcription Factor Binding Sites (TFBS).

The description of the complete description of TFBS can be found in additional file 3 (in the "Matrix Family Library" Excell sheet).

Genomatix-Logo Logout Personal Messages GEMS-Logo

Genomatix_MainGEMSElDoradoGene2PromoterBiblioSphere
Frequently_asked_questionsYour_ResultsYour_SequencesYour_ProtocolHelp

GEMS Launcher Task: *DiAlign TF*: Multiple alignment plus TF sites
working on promoters.seq (3 seq.)

[Alignment] [Pairwise_similarities]

DiAlign professional TF Release 3.1 December 2004 **Thu May 5 21:22:57 2005**

Solution parameters:

**Sequence file:**	promoters.seq (3 seq.)
**Type of sequences:**	nucleotide sequences
**Threshold T:**	0.00
**'*' signs below alignment denote:**	diagonal similarity (max. similarity is represented by 10 '*' signs)
**Alignment output:**	complete alignment is shown<br># nucleic acids per line: 50
**TF output:**	common TF matches located in aligned regions (common to 3 (100.0 %) sequences)
**Family matches:**	yes
**MatInspector library:**	Matrix Family Library Version 5.0 (February 2005)
**Selected groups** (core/matrix sim):	• ALL user_defined.lib (0.75/Optimized)<br>• ALL vertebrates.lib (0.75/Optimized)

Aligned Sequences:

No.	Sequence Name	Sequence Description	Length
1	P977959	sym=COX4I2|loc=Loc84701|taxid=9606|spec=Homo sapiens|chr=20|ctg=NT_028392|str=(+)|start=421283|end=422483|len=1201| tss=501| comm=cytochrome c oxidase subunit IV isoform 2 (lung); (NM_032609/501/silver;)	1201 bp
2	P977960	sym=Cox4i2|loc=Loc84682|taxid=10090|spec=Mus musculus|chr=2|ctg=NT_039210|str=(+)|start=3150756|end=3151956|len=1201| tss=501| comm=cytochrome c oxidase subunit IV isoform 2; (NM_053091/501/bronze;)	1201 bp
3	P977961	sym=Cox4b|loc=Loc84683|taxid=10116|spec=Rattus norvegicus|chr=3|ctg=NW_047658|str=(+)|start=33280248|end=33281448| len=1201|tss=501|comm=cytochrome c oxidase, subunit 4b; (NM_053472/530/bronze;)	1201 bp

Alignment (DiAlign format):

Please note that only **upper-case** letters are considered to be **aligned**. For more information, please have a look at the user guide.

V$TEAF V$ETSF V$NFKB V$ZBPF V$RORA V$TBPF V$HIFF V$HOXF V$NKXH V$HOMF

```
alignment position 1 . . . . . . . . .   1 1 . . . . . . . .   2 1 . . . . . . . .   3 1 . . . . . . . .   4 1 . . . . . . . .
P977959     1 a t g a g c c a c c   g c g c c c g c c c   a a t t t t t c t a   t t t t t a - - - -   - - - - - - - - - -
P977960     1 a g g c a g a a g g   a g g c t t t c c c   C T C T C C C C T T   A C T G C A C C C C   T C T T A G G G T G
P977961     1 - - - - - - - - - -   - - - - - - - - - -   C T C T C C C C T T   A A C G C A T C T C   T C T T A G G G T G
```

```
                                                         ********** ********** **********
                                                         ********** ********** **********
                                                         ********** ********** **********
                                                         ********** ********** **********

alignment position 51........  61........  71........  81........  91........
P977959        37 ---------- ---------- ---------- ---------- ----------
P977960        51 GAGGGCCCAG gaGAGCCCAC AGTGGTACac aAAGGCTGCC TGGGGCGGAA
P977961        31 GAGGGCCCAG g-GAGCCCAC GATGGTACct gAAGGCTACC TGGGGCAGGA
                  **********   ******** *******     ********* **********
                  **********                        ********* **********
                  **********                        ********* **********
                  **********

alignment position 101.......  111.......  121.......  131.......  141.......
P977959        37 ---------- ---------- ---------- ---------- ----------
P977960       101 GGCTGGATGA CTGAtgggct ctggcgtcac acctgtAATC TCAGCGCCCT
P977961        80 GGCTGGAGGG CTGAgacatt ggaatcatct gta---AATC TCAGTGCCCT
                  ********** ****                        **** **********
                  ********** ****                        **** **********
                  ********** ****                        **** **********

alignment position 151.......  161.......  171.......  181.......  191.......
P977959        37 ---------- ---------- ---------- ---------- ATAGAGAAGG
P977960       151 GAAAGTGGCG GCAAGAGGTA GCTCAGGGCC AGTCTCTGCT ATATAGTAAG
P977961       127 GGAAGTAAAG TCAAGAAGTA GCTCAGGGCC AGAGTCCGTT ATATAGTGAG
                  ********** ********** ********** ********** **********
                  ********** ********** ********** ********** **********
                  ********** ********** ********** ********** **********

alignment position 201.......  211.......  221.......  231.......  241.......
P977959        47 TGTTTCGCCA TGCTGGGCAG GCTGGTCTC- -----Aaact cctgacctca
P977960       201 TTGCAGGCCA TCCTGGACtc cagaaaaccc tgactAACCA AACC------
P977961       177 TTCCAGGCCA GCCTGGAAAC CCCCGACTC- -----AACAA AACC------
                  ********** ********** *********       ***** ****
                  ********** ******                     ***** ****
                  ********** ******                     ***** ****

alignment position 251.......  261.......  271.......  281.......  291.......
P977959        91 ggtgatcctc ccgcctcggc ctcccaaagt gctaggatta caggcgtgag
P977960       245 ---------- ---------- ---------- ---------- ----------
P977961       215 ---------- ---------- ---------- ---------- ----------

alignment position 301.......  311.......  321.......  331.......  341.......
P977959       141 ccactgctgg cctaaaagat gcttctggag taggaggcat tgggcctggc
P977960       245 ---------- ---------- ---------- ---------- ----------
P977961       215 ---------- ---------- ---------- ---------- ----------

alignment position 351.......  361.......  371.......  381.......  391.......
P977959       191 tggaaggttt gcgtctgcaa aggcctgaAG AGAACATCCC AGGTGGGAGA
P977960       245 ---------- ---------- --------AG ACAGCATTCC TGGTGGGATG
P977961       215 ---------- ---------- --------AG AAAGCATTCC TGGTGc--TG
                                                 ** ********** **********
                                                 ** ********** *****   **
                                                 ** ********** *****

alignment position 401.......  411.......  421.......  431.......  441.......
P977959       241 AACAGCAAGG AGGGGAGGtg tggaggaggc agggagaccc tgttcttggg
P977960       267 GCAAGGAGAA AGGTGTGGCa ggg------T TCTCCTGTT- ----------
P977961       235 GCACGGAGAA AGGTGTGGCg tcaaggttcT TCTCCAGTT- ----------
                  ********** *********          * *********
                  ********** *********          * *********
                                                * *********
                                                * *********

alignment position 451.......  461.......  471.......  481.......  491.......
```

```
P977959     291 aCTACTGTCT GCAGAGGGTC TCCAGAGGca gg-ACCCCGG TAGGGACact
P977960     300 -CTTCTGTCG GCTGGGGGTC TGCTGAGGTC CctactctGG TGAGGACCCA
P977961     274 -CCTCTGTCA GCTGGGGGTC TGCGGAGGTC CccACACCGG TGAGGACCGG
                 ********* ********** ********** *  ******* **********
                 ********* ********** ********** *       *** **********
                 ********* ********** ********** *       *** **********
                 ********* ********** ********** *
                 ********* ********** ********
                    ****** ********** ********

alignment position 501....... 511....... 521....... 531....... 541.......
P977959     340 gatgcaggaG GCGCTCTGCA GTGCCTCCCC CCAATCTGTG GGCCGGGGtg
P977960     349 GC-------G GGAAACCGCA GTCCTTTCTC TTTTCCTGTG CCTCTGGGGA
P977961     323 GT-------G GCAAGCTGCA GTCCTTTCTC TTTTCCTGCG CCCCTGGGGA
                **       * ********** ********** ********** **********
                **       * ********** ********** ********** **********
                **       * ********** ********** ********** **********

alignment position 551....... 561....... 571....... 581....... 591.......
P977959     390 cccccaggga cgcaggctgt gcgctcccac gccctgccGA AGCAGGACGT
P977960     392 CATGGCTCTG CAAAAGCGAC Cgagg----- -------CGA GGCGGGATGT
P977961     366 CGTGGCCCAG CACAAGCGAC Ca-------- ---------A GGCGGGATGT
                ********** ********** *                 ** * **********
                ********** ********** *                 ** * **********
                ********** ********** *                    * **********
                                                              *********

alignment position 601....... 611....... 621....... 631....... 641.......
P977959     440 TCCCACGC-T GGGGCGGCcc cgagaaaggc tgtgggcggg gccgggggcg
P977960     430 TCCCACGT-G GGGGTGGC-- ---------- ---------- ----------
P977961     399 TCCCACGCgG GGGGTGGC-- ---------- ---------- ----------
                ********  * ********
                ********  * ********
                *******   * ********
                *******   * ********
                          * ********
                          * ********

alignment position 651....... 661....... 671....... 681....... 691.......
P977959     489 ggcgcccgca ctcaggtccc tccgcagcgg gttctcagtt gctcgctggg
P977960     447 ---------- ---------- ---------- ---------- ----------
P977961     417 ---------- ---------- ---------- ---------- ----------

alignment position 701....... 711....... 721....... 731....... 741.......
P977959     539 cagacccagg tcgcgctccc actgccgagc ccgcgaggtg agtgagttgg
P977960     447 ---------- ---------- ---------- ---------- ----------
P977961     417 ---------- ---------- ---------- ---------- ----------

alignment position 751....... 761....... 771....... 781....... 791.......
P977959     589 gggaggaccc cgccacccaa gcccgcgcgG GTCAGGGGCG CGGCTCGGTC
P977960     447 ---------- ---------- ---------G GTCGGAGGCG GGGCGTGTTC
P977961     417 ---------- ---------- ---------G GGCGGAGGCG GAGCGTGCTC
                                               * ********** **********
                                               * ********** **********
                                               * ********** **********
                                               * ********** **********

alignment position 801....... 811....... 821....... 831....... 841.......
P977959     639 Tcctgccttc gccgctgggt cacgccccag tggtccctgt cttagagggt
P977960     468 TGGTCC---- ---------- ---------- ---------- ----------
P977961     438 TGGTCC---- ---------- ---------- ---------- ----------
                ******
                ******
                ******
                ******

alignment position 851....... 861....... 871....... 881....... 891.......
```

```
P977959      689 tggggggctt cttgtaggtt agagttgaag actcctgggg agtctgcata
P977960      474 ---------- ---------- ---------- ---------- ----------
P977961      444 ---------- ---------- ---------- ---------- ----------

alignment position 901....... 911....... 921....... 931....... 941.......
P977959      739 tgtgtgccat ctctttcccc aaatatagat tacatcctCC CTCTCTTGAT
P977960      474 ---------- ---------- ---------- --------CC CTCTCCGGGT
P977961      444 ---------- ---------- ---------- --------CC tcagCCTGGT
                                                          ** **********
                                                          **         ***
                                                          **         ***
                                                          **         ***
                                                          **         ***
                                                                     ***
                                                                     ***

alignment position 951....... 961....... 971....... 981....... 991.......
P977959      789 ATGTGGAGCT CTCCCACCCA CTAATCAtaa tcactggccg gagc------
P977960      486 CTGCGGTTCC CTGCCAGTCA CTCAGCAGGG CAGCTCTgga tagtTCCGCC
P977961      456 CTGCGGTTCC CTGCCAGTCA CTCAGCAGGG CAGTTCTggg taccTCCGCC
                 ********** ********** ********** *******        ******
                 ********** ********** ********** *******        ******
                 ********** ********** ********** *******        ******
                 ********** ********** ********** *******        ******
                 ********** ********** ********** *******        ******
                 ********** ********** ********** *******
                 ********** ********** *******

alignment position 1001...... 1011...... 1021...... 1031...... 1041......
P977959      833 ---------- ---------- ---------- ---------- ----------
P977960      536 GCCTCCAGCT TGCAATTGTG AGTCCCACCT GAGGgGAAGG GTCGCGGGGA
P977961      506 GTCTTCAGCT TGCAACTGTG AGTCCCACCT GAGG-GAAGG GTCTTGGGGG
                 ********** ********** ********** **** ***** **********
                 ********** ********** ********** **** ***** **********
                 ********** ********** ********** **** ***** **********
                 ********** ********** ********** ****
                 ********** ********** ********** ****

alignment position 1051...... 1061...... 1071...... 1081...... 1091......
P977959      833 ---------- ---------- ---------- ---------- ---------C
P977960      586 TGGGATGTTC GTGGCGGC-- ---------- ---------C ACCACACAGC
P977961      555 TGGGGTGTTC GTGGTGGCca ccctaagcct ttgctccatC ACCACACAGC
                 ********** ********                     * **********
                 ********** ********                     * **********
                 ********** ********                     * **********
                                                         * **********

alignment position 1101...... 1111...... 1121...... 1131...... 1141......
P977959      834 CCTCTTTTCC AGAAGTgtgg gccgcccccct cccacttatc acctgtagtg
P977960      615 CCTCTTTTGC TGAGGTTT-- ---------- ---------- ----------
P977961      605 CCTCTTTTGC TGAGa-TT-- ---------- ---------- ----------
                 ********** ********
                 ********** ****
                 ********** ****
                 ********** ****

alignment position 1151...... 1161...... 1171...... 1181...... 1191......
P977959      884 tgccCCCTCC CTCACTC--- ---------- -TATTTGTTG GGTGCCCCTC
P977960      633 ----CCCCCA CTGACTA--- ---------- -TCCTTGTCC CGGGCATCAC
P977961      622 ----CCCCCA CTGACccgtt ttaatgctgg gTCCTAGTCC CGGGCATCGT
                     ****** *******               ********* **********
                                                  ********* **********

alignment position 1201...... 1211...... 1221...... 1231...... 1241......
P977959      920 TCtctcccta gatgtgggac tcccct---- ---------- ---TCTTTTC
P977960      665 ACCGGGCCCC AAAAGTGAGG tCTCTTTTCA GAGGATGGGC TGGTCCTTTT
P977961      668 CCCCGGCCCC AGAAATGGGG cCTCTTTCTA GAGGGTGGGG TGGCCCTTTT
```

```
                           ********** ********** ********** ********** **********
                           ********** ********** ********** ********** **********
                                                  ********* ********** **********

alignment position         1251...... 1261...... 1271...... 1281...... 1291......
P977959               953  CCCAGATGTG tgtctccct- --CCCTCTGA CCCAGTTAAG TCCCCATCCT
P977960               715  CCTAGAAGTG CTGCCTCTCC TACCCGCTGA CCCAGTTTGG TCCTTAGCCT
P977961               718  CCTAGAAATG CTGCCTCTCC CACCCGCTGA CCCAGTTTGG TCCTTGGCCT
                           ********** ********** ********** ********** **********
                           ********** ********** ********** ********** **********
                           ********** ********** ********** ********** **********
                                      ********** ********** ********** **********
                                      ********** ********** ********** **********
                                                   ******** ********** **********
                                                   ******** ********** **********
                                                   ******** ********** **********
                                                   ******** ********** **********
                                                   ******** ********** **********

alignment position         1301...... 1311...... 1321...... 1331...... 1341......
P977959              1000  CTAAAGCCCC Tccccaccac catGGATGCC AAACCCTGAG TCTTCCctgg
P977960               765  CTAAAGCCCC TG-------- ---GGATACC AGACTCTGCA TGTCCCGCGT
P977961               768  CTAAAGCCCC TG-------- ---GGATGTC AGACTCTGCA CGTCCCGCGT
                           ********** **            ******* ********** **********
                           ********** **            ******* ********** **********
                           ********** **            ******* ********** **********
                           ********** **            ******* ********** **********
                           ********** **            ******* ********** **********
                           ********** *
                           ********** *
                           ********** *
                           ********** *
                           ********** *

alignment position         1351...... 1361...... 1371...... 1381...... 1391......
P977959              1050  gggtgggaga ctgggctcag ccagactggc taAGCCCAGT CCCCACCCCA
P977960               804  C-----CCGA GTCCGGAGTG ATGGT----- --AGGGCACA GCCTGCCCCC
P977961               807  CccgttCCGA GTCCGGAGTG ATGGT----- --AGGGCACA GCCTGCCCCC
                           *     **** ********** *****        ******** **********
                           *     **** ********** *****        ******** **********
                           *     **** ********** *****        ******** **********
                           *     **** ********** *****        ******** **********
                           *     **** ********** *****        ******** **********
                                 **** ********** *****        ******** **********
                                 **** ********** *****        ******** **********

alignment position         1401...... 1411...... 1421...... 1431...... 1441......
P977959              1100  ACCAGATCCA GCAGAGCAGG CctgtgtGCA CGTAGGCAGC CAATTAGGGg
P977960               842  TCCTCCTGCA GCGGAGCAGG CAGAGC-GCA CGTAGGCAGT CAATTAAGGA
P977961               850  TCC---TGCA GCGGAGCAGG CAGAGC-GCA CGTAGGCAGT CAATTAAGGA
                           ********** ********** ****** *** ********** **********
                           ***   **** ********** ****** *** ********** **********
                           ***   **** ********** ****** *** ********** *********
                           ***   **** ********** ****** *** ********** *********
                           ***   **** ********** ****** *** ********** *********
                           ***   **** ********** ****** *** ********** *******
                           ***   **** ********** ****** *** ********** *******
                                                        *** ********** *******
                                                        *** ********** *******

alignment position         1451...... 1461...... 1471...... 1481...... 1491......
P977959              1150  ggaaggggTG GGGGCCAG-- ----ACCGTC TGtc------ ----------
P977960               891  GACC----TT GGAGCCAG-- ----AGCTTC TGCTTAGAGG TCTGGGCTAA
P977961               896  GACC----TT GGAGCCAGgc gcagAGCATC TGCTTAGAGG TCTGGGCTAA
                           ****    ** ********      ****** ********** **********
                           ****    ** ********      ****** ********** **********
                                                    ****** ********** **********
                                                    ****** ********** **********

alignment position         1501...... 1511...... 1521...... 1531...... 1541......
```

```
P977959    1178 ---------- ---------- ---------- ---------- ----------
P977960     931 CAGCCCTGTG GgaAGGAGGC TCAGGACTTT TCtctgggga agacaattAG
P977961     942 CCGCCCTGCG GtgAGGAGGC TCAGGATTTT TCctctgg-- --------AG
                ********** *  ******* ********** **                 **
                ********** *  ******* ********** **
                ********** *
                ********** *

alignment position 1551...... 1561...... 1571...... 1581...... 1591......
P977959    1178 ---------- ---------- ---------- ---------- ----------
P977960     981 TGGAGCTTCC CAACT---CT GATGGGAGca catcaCATCA GAGCACAGGG
P977961     982 TGGGGCTTCC CAACTtggCT GATGGGAGgc tgg--CATCA GAGAACGGGG
                ********** *****   ** ********        ***** **********
                                                      ***** **********
                                                      ***** **********
                                                      ***** **********
                                                      ***** **********

alignment position 1601...... 1611...... 1621...... 1631...... 1641......
P977959    1178 ---------- ---------- ---------- ---------- ----------
P977960    1028 CTCCCTGTCC TAAACTGTTC ACATCttACC CAGTAGCTTG ACCTCTTCCC
P977961    1030 CTCCCTGTCC TAAACTGTTC AGATCtcACC CAGTGGCTAG ACCTCTTCCC
                ********** ********** *****  *** ********** **********
                ********** ********** *****  *** ********** **********
                ********** ********** *****  *** ********** **********
                ********** ********** *****
                ********** ********** *****

alignment position 1651...... 1661...... 1671...... 1681...... 1691......
P977959    1178 ---------- ---------- ---------- ---------- ----------
P977960    1078 ACTTTCCAAA TGATGAAtgg agaacccaag agggcCCCAA AGATCTAGGT
P977961    1080 ACTTCTCAAA CGACGAAtga aaa------- -----CCCAA AGGTCCAGGG
                ********** *******                 ***** **********
                ********** *******                 ***** **********
                ********** *******                 ***** **********
                                                   ***** **********

alignment position 1701...... 1711...... 1721...... 1731...... 1741......
P977959    1178 ---------- ---------- ---------- -------TGT TCTGCCGGTG
P977960    1128 CAGCGGTTCC CAACCTGGGA Gggggagttgg ggggcgtTGA ACTACTTTTA
P977961    1118 CAGCGGTTCT CAACCTGGGA Gttgaacgac t--------- ----CTTTTA
                ********** ********** *                 *** **********
                ********** ********** *                         ******
                ********** ********** *                         ******
                ********** ********** *

alignment position 1751...... 1761...... 1771...... 1781...... 1791...
P977959    1191 CAGGGGCCTC t--------- ---------- ---------- -------
P977960    1178 CAGGGGTCTC CTAAGACCGT TGGC------ ---------- -------
P977961    1155 CAGGGGTCTC CTAAAACCAC TGGCgtatcg ggtatttaca gtaggat
                ********** ********** ****
                ********** ********** ****
                ********** ********** ****
```

Pairwise similarities:

For each pairwise alignment, the similarity (relative to the maximum similarity) and the number of identical nucleic acids (in % of shorter sequence) is given. Maximum values are underlined.

	P977960 (1201 bp)	P977961 (1201 bp)
**P977959 (1201 bp)**	0.079<br>23 %	0.066<br>17 %
**P977960 (1201 bp)**		<u>1.000</u><br><u>77 %</u>

Please note that the **similarity value 1.000** marks only the two **most similar** sequences, it does not necessarily mean that these sequences are identical.

Extract alignment region	
**Positions in alignment**	from: to:
**Output file**	dialign.seq

Extract aligned sequences

For comments, questions or bug reports, please contact support@genomatix.de

© Genomatix Software GmbH 1998-2005 - All rights reserved.

License Agreement

GEMS Launcher 4.1.1

GEMS Launcher Task: *DiAlign TF*: Multiple alignment plus TF sites
working on promoters.seq (3 seq.)

[Alignment] [Pairwise_similarities]

DiAlign professional TF Release 3.1 December 2004 **Tue May 3 22:33:50 2005**

Solution parameters:

**Sequence file:**	promoters.seq (3 seq.)
**Type of sequences:**	nucleotide sequences
**Threshold T:**	0.00
**'*' signs below alignment denote:**	diagonal similarity (max. similarity is represented by 10 '*' signs)
**Alignment output:**	complete alignment is shown<br># nucleic acids per line: 50
**TF output:**	common TF matches located in aligned regions (common to 3 (100.0 %) sequences)
**Family matches:**	yes
**MatInspector library:**	Matrix Family Library Version 5.0 (February 2005)
**Selected groups** (core/matrix sim):	• ALL user_defined.lib (0.75/Optimized)<br>• ALL vertebrates.lib (0.75/Optimized)

Aligned Sequences:

No.	Sequence Name	Sequence Description	Length
1	P875598	sym=COX4I1|loc=Loc1327|taxid=9606|spec=Homo sapiens|chr=16|ctg=NT_010498|str=(+)|start=39446895|end=39448095|len=1201| tss=501| comm=cytochrome c oxidase subunit IV isoform 1; (NM_001861/501/silver;)	1201 bp
2	P875602	sym=Cox4i1|loc=Loc12857|taxid=10090|spec=Mus musculus|chr=8|ctg=NT_078575|str=(+)|start=45821111|end=45822366|len=1256| tss=501,517,556| comm=cytochrome c oxidase subunit IV isoform 1; (AK012583/524/gold;AK019276/579/gold;NM_009941/540/silver;)	1256 bp
3	P875604	sym=Cox4a|loc=Loc29445|taxid=10116|spec=Rattus norvegicus|chr=19|ctg=NW_047536|str=(+)|start=13459368|end=13460568| len=1201|tss=501|comm=cytochrome c oxidase, subunit 4a; (NM_017202/501/bronze;)	1201 bp

Alignment (DiAlign format):

Please note that only **upper-case** letters are considered to be **aligned**. For more information, please have a look at the user guide.

V$ETSF V$ZF35 V$TEAF V$AP1R V$CREB V$DEAF U$NRF2 V$DICE V$CMYB V$MYOD V$RORA V$EREF
U$EREF V$SF1F V$COUP V$ZF5F V$E4FF

```
alignment
position         1 .........  1 1 .........  2 1 .........  3 1 .........  4 1 .........
P875598        1 ggggcgagg-  ----------  ----------  ----------  ----------
P875602        1 ----------  ----------  ----------  ----------  ----------
```

```
P875604      1 gccgtggccc cgagcttact agggtgagcg ccacctccag catgggcgtc

alignment
position         51........ 61........ 71........ 81........ 91........

P875598     10 ---GCCAGGG TGCCGTGGAA GAGGGGGATG CAGTCCACGA AGAgggtgtg
P875602      1 --AGCCAGCG TGCCGTGGAA GAGCGGGATG CAGTCCACGA AGAGCGTGTG
P875604     51 agAGCCAGCG TGCCGTGGAA GAGCGGGATG CAGTCCACGA AGAGAGTGTG
                 ******** ********** ********** ********** **********
                 ******** ********** ********** ********** **********
                 ******** ********** ********** ********** ***
                 ******** ********** ********** ********** ***
                  ******* ********** ********** ********** ***
                  ******* ********** ********** ********** ***
                  ******* ********** ********** ********** ***
                  ******* ********** ********** ********** **
                  ******* ********** ********** ********** **
                  ******* ********** ********** ********** **

alignment
position         101....... 111....... 121....... 131....... 141.......

P875598     57 gtgg---GCG CCGGGgccgc ccaggggag gTGCTCCTTA CGCGGCTTCT
P875602     49 GCTGCCCGCT CCGGGAGGA- ---------- -TGCTCCTTG CGCGGCCTCT
P875604    101 GTTGCCCGCG CCGGGAGGA- ---------- -TGTTCCTTG CGCGGCCTCT
               ********** *********             ********* **********
               ********** *********             ********* **********
                                                ********* **********
                                                ********* **********
                                                ** ****** **********
                                                ** ****** **********
                                                   ****** **********

alignment
position         151....... 161....... 171....... 181....... 191.......

P875598    104 GCTTCTCGGC CACCAGGAGC CCGTTGACGG CGCAGTGCGG GTACTTGGCG
P875602     87 GCCTCTCGGC CACCAGGAGC CCGTTGACGG CGCAGTGCGG GTACTTGGCG
P875604    139 GCCTCTCTGC CACCAGaAGC CCGTTGACGG CGCAGTGCGG GTACTTGGCG
               ********** ********** ********** ********** **********
               ********** ********** ********** ********** **********
               ********** ********** ********** ********** **********
               ********** ******  ** ********** ********** **********
               ********** ******  ** ********** ********** **********
               ********** ******  ** ********** ********** **********
               ********** ******  ** ********** ********** **********
                                 *** ********** ********** **********
                                 *** ********** ********** **********
                                 *** ********** ********** **********

alignment
position         201....... 211....... 221....... 231....... 241.......

P875598    154 CCGTGCAGCA CCATCTTGCA GTAGGCCTGG GTGGTCAGTT TCACCCCGGG
P875602    137 CCGTGGAGCA CCATCTTGCA GTAGGCCTGC GTAGTCAGCT TCACGCCGGG
P875604    189 CCGTGtAGCA CCATCTTGCA GTAGGCCTGG GTGGTCAGCT TCACGCCAGG
               ********** ********** ********** ********** **********
               ********** ********** ********** ********** **********
               ********** ********** ********** ********** **********
               ********** ********** ********** ********** ******
               ***** **** ********** ********** ********** ******
               ***** **** ********** ********** ********** ******
               ***** **** ********** ********** ********
               ***** **** ********** ********** ********
               ***** **** *
               *****

alignment
position         251....... 261....... 271....... 281....... 291.......

P875598    204 CATGCTGACC Cgggagggc- ---------- ---------- ----------
P875602    187 Ca-------- ---------- ---TCGCGAC CCACGCACCG CGGTcctccg
P875604    239 CATGCTGAGC Ctagctggtc gcgTCGCGAC CAACGCACCA CAGTacgaaa
```

```
             ********** *          ******* ********** ****
             *
             *

alignment
position         301....... 311....... 321....... 331....... 341.......

P875598  223 ---------- ---------- CCCGGAGGCC CCTGGGCGCG CGGCTGAGGC
P875602  216 ccacgaccCT CCGCTGCACG CCGTGACGCT CTCGCACCTC CGGCCGCGGC
P875604  289 cacga---CC CCACTGCACG CTAAGATGAG CTCCCACCAC CAGCtacaGC
                     ** ********** ********** ********** **********
                                                                 **

alignment
position         351....... 361....... 371....... 381....... 391.......

P875598  253 CTggaccgc  tgcctggccg cgcggCGCCT CAGCCGagaa gcgggacgag
P875602  266 CTCC------ ---------- -----CGCCT CAGCCGGT-- ----------
P875604  336 CTCC------ ---------- -----CGCCT CAGTCCGC-- ----------
             ****                       ***** ********
             ****                       ***** ********

alignment
position         401....... 411....... 421....... 431....... 441.......

P875598  303 gcggcggcga ttgatggcgc ggccgcgggc tggcggggga ccctcaggc
P875602  283 ---------- ---------- ---------- ---------- ----------
P875604  353 ---------- ---------- ---------- ---------- ----------

alignment
position         451....... 461....... 471....... 481....... 491.......

P875598  353 ccggcccgt  ttgggcctcg gctcctggaa aagcgactcg cgcctctggg
P875602  283 ---------- ---------- ---------- ---------- ----------
P875604  353 ---------- ---------- ---------- ---------- ----------

alignment
position         501....... 511....... 521....... 531....... 541.......

P875598  403 aagccgcagc cccagactcc agtcgcgctt ctcgcccggc gCCGCCGGAA
P875602  283 ---------- ---------- ---------- ---------- -CGGCCGGAA
P875604  353 ---------- ---------- ---------- ---------- -CCGCCGGAA
                                                       *********
                                                       *********

alignment
position         551....... 561....... 571....... 581....... 591.......

P875598  453 agcagcctct ccAA------ ---------- CGCCTGCCGG AAAGCAGCCC
P875602  292 ---------- --AA------ ---------- CGTCTGCCGG AAAGCAGCtg
P875604  362 ---------- --Agcgtagt cgctagctag CGTCTGCCAG AAAGCAGCGC
                          **                ********** **********
                          *                 ********** *********
                                            ********** *********

alignment
position         601....... 611....... 621....... 631....... 641.......

P875598  487 GGCC-----C GGCATTTTAC GACGTTCG-- ---------- ----------
P875602  314 ctacccGCTC GGCCTTTCGC GACAGttacc gccccgcccc gcctcgtcct
P875604  400 GGCCt-GCTC GGCCTTTCGC GACAGCTG-- ---------- ----------
             ****  **** ********** ********
                   **** ********** *****

alignment
position         651....... 661....... 671....... 681....... 691.......

P875598  510 ---------- ---------- ---------- --------CA GCGCTACCCT
P875602  364 gcccgccct  gccccgagcc gcgccgcccc gccccgccCC GCCCCGCGCT
P875604  427 ---------- ---------- ---------- --------CC GCACCGCGAT
                                                    ** **********

alignment
position         701....... 711....... 721....... 731....... 741.......
```

```
P875598      522 TTTCCGCTcc acggtgacct ccgtgcggcc gggtgcgggc ggagtcTTCC
P875602      414 CTGAAACGGA CGGGCTTGGG GCGGGgct-- ---------- ------TCCC
P875604      439 CTGAAGCTGA TGGACGTGGG CGGGGcttct tc-------- ----------
                 ********** ********** *****                      ****

alignment
position         751....... 761....... 771....... 781....... 791.......

P875598      572 TCGATCCCGT GGTGCTCCGC GGCGCGGCCT TGCTCT---- ----------
P875602      446 TGATTCCCGC GATGCTTCGC GGCTCCGTCT TGGTCTTCCG GtTGCGGGCC
P875604      471 -GATTCCCGC GATGCTTCGC GGCACCGTCT TGCTCTTCCG G-TGCGGGAC
                 ********** ********** ********** ********** *  ********
                  ********* ********** ********** ********** *  ********
                  ********* ********** ********** ********** *
                  ********* ********** ********** ******
                     ******* ********** ********** ******

alignment
position         801....... 811....... 821....... 831....... 841.......

P875598      608 -----CTTCC GGTCGCGgga cacc--GGGT GTAGAGGGCG GTCGCGGCGG
P875602      496 CCGTTCTTCC GGTCGCGAGC ACCCcaGGGT GTAGAGGGCG GTCGCGGCGG
P875604      519 CCGCTCTTCC GGTCGCGAGC ACCCg-GGGT GTAGAGGGCG GTCGCGGCGG
                 ********** ********** ****  **** ********** **********
                 ********** ********** ****  **** ********** **********
                      ***** *******          **** ********** **********
                                             **** ********** **********
                                             **** ********** **********
                                             **** ********** *********
                                             **** ********** *********

alignment
position         851....... 861....... 871....... 881....... 891.......

P875598      651 --------GC AGTGGCGGCA GGTGAgacag gag------- ----------
P875602      546 TCGCCTGGGC AGCGGTGGCA GGTGACGGCC TTGGACGGCG GGTGAGGGTG
P875604      568 TCGCCTGGGC AGCAGTGGCA GGTGACGGCC TTTGACAGCG GGTGAGGGCG
                 ********** ********** ********** ********** **********
                 ********** *** ****** ********** ********** **********
                 ********** *** ****** ********** ********** **********
                 ********** ***

alignment
position         901....... 911....... 921....... 931....... 941.......

P875598      676 ---------- ---------- ---------- --GTGGCCGG -TGCGGCGCC
P875602      596 GCacggtcgg gGGCCCGAGG ACCTGGAGCG CCGGGGCCGG gAGCGGTGCC
P875604      618 GCgcggccat -GGGCCGAGA TCCTGGAGCG CCGGGGCCGG -AGCGGTGCC
                 **          ********* ********** ********** *********
                 **          ********* ********** ********** *********
                 **                                          *********
                                                             *********

alignment
position         951....... 961....... 971....... 981....... 991.......

P875598      693 GCCAGGCCGG GCCGGGTCGG GGGGCcggga gccatcaagt ctgcacgtcc
P875602      646 TCCGAGACGC CCCCGGACCC GGGACTTGAG CgGCTCCG-- ----------
P875604      666 TCCGAGGCGC CCCCGGACTC GGGACTTGAG CaGCTCCG-- ----------
                 ********** ********** ********** *  ******
                 ********** ********** ********** *  ******
                 ********** ********** ********** *
                 ********** ********** ***

alignment
position         1001...... 1011...... 1021...... 1031...... 1041......

P875598      743 gcagcctggc cgctcggctt cagcaggaag caccgaatgg gcctcggagc
P875602      684 ---------- ---------- ---------- ---------- ----------
P875604      704 ---------- ---------- ---------- ---------- ----------

alignment
position         1051...... 1061...... 1071...... 1081...... 1091......

P875598      793 caggtgacat tgaggccggc ccgtggggac tccgggctcg gtggctccag
```

```
P875602    684 ---------- ---------- ---------- ---------- ----------
P875604    704 ---------- ---------- ---------- ---------- ----------

alignment
position       1101...... 1111...... 1121...... 1131...... 1141......
P875598    843 gctcgggggg ccgccccgaa gtgcccggtc catcttaccc ggtctcgcag
P875602    684 ---------- ---------- ---------- ---------- ----------
P875604    704 ---------- ---------- ---------- ---------- ----------

alignment
position       1151...... 1161...... 1171...... 1181...... 1191......
P875598    893 cggctgcgga ccggcctcgg gcacTGACCT TGGAGCGCCC GCTggccga-
P875602    684 ---------- ---------- ----TGACCT TGGGGCGCCC GCTTCCCcga
P875604    704 ---------- ---------- ----TGACCT TGGGGCGCCC GCTTCCCgg-
                                      ****** ********** *******
                                      ****** ********** *******
                                      ****** ********** ***
                                      ****** ********** ***

alignment
position       1201...... 1211...... 1221...... 1231...... 1241......
P875598    942 ---------- ---------- ---------- -----GGGCT CAGGCTGCGG
P875602    710 GCGGGTCTGT TGTCTCGGGT CCCCgc---- --------TC CGGCCTGCCG
P875604    729 GCGGATCTGT TGTCTTGGGT CCCCatttgc cgttcGGTTC CGGCCTGCCC
               ********** ********** ****            ***** **********
                                                        ** **********

alignment
position       1251...... 1261...... 1271...... 1281...... 1291......
P875598    957 GGAGGCGGGC CCGCGCTCTG TGCCtgcagc ctccgccctt gctccttcaa
P875602    748 GGCACCGGGC GTTCCCGCTG TGCC------ ---------- ----------
P875604    779 AGCATCGGGC GTTCCCGCTC CGCC------ ---------- ----------
               ********** ********** ****
               ********** ********    ***

alignment
position       1301...... 1311...... 1321...... 1331...... 1341......
P875598   1007 aaggtccctg tgcaacccct cccgggtttt gcggggctcc cgggcggcgc
P875602    772 ---------- ---------- ---------- ---------- ----------
P875604    803 ---------- ---------- ---------- ---------- ----------

alignment
position       1351...... 1361...... 1371...... 1381...... 1391......
P875598   1057 tcggctttcc agcctggaag gcgcctattg tttgctcacg caagggctAG
P875602    772 ---------- ---------- ---------- ---------- --------AG
P875604    803 ---------- ---------- ---------- ---------- --------AG
                                                                   **
                                                                   **

alignment
position       1401...... 1411...... 1421...... 1431...... 1441......
P875598   1107 GCCCAAGGag agaagctaga cgcgggcgtt cAGCCCTGAC GTCATGCTtg
P875602    774 GCCCGAGGGA CAGCGACCTG ---------- -AGCCCTGAC GTCACGCTAC
P875604    805 GCCCGAGGGA CAGCGAGCTG ---------- -AGTCCTGAC GTCACGCTAC
               ********** **********             ********* **********
               ********** **********             ** ****** **********
                                                 ** ****** **********
                                                    ****** **********

alignment
position       1451...... 1461...... 1471...... 1481...... 1491......
P875598   1157 tttaTTCTGC CCCAAGGAGG GTAT------ TTcccccacat cactctcacc
P875602    813 GGT-TTCTGC CGCCCCGGGC GTTGattttt TTTTTCCCGG GCTCGGACGT
P875604    844 GGT-TTCTGC CGCCACGGGC GTTG------ TTTTTTGCGG TCTCGGGCGT
```

```
                  *** ****** ********** ****            ********** **********
                  *** ****** ********** ****            ********** **********
                  *** ****** ********** ****
                  *** ****** ********** ****

alignment
position          1501...... 1511...... 1521...... 1531...... 1541......

P875598     1201  a--------- ---------- ---------- ---------- ----------
P875602      862  TCTTTCTCGG GGATCGAAtc tc--AGGGGT TGTGCTACAG TGAGGCGTGG
P875604      887  TCGCTCTCGG GGAGCGAAat caagAGGGGT TGTGTTAAAG TGAGGCGTGG
                  ********** ********       ****** ********** **********
                  ********** ********       ****** ********** **********
                                            ****** ********** **********

alignment
position          1551...... 1561...... 1571...... 1581...... 1591......

P875598     1202  ---------- ---------- ---------- ---------- ----------
P875602      910  GGCTCGGGTC GCTGGGGCGG GTGACAGGGT CATCCAGGTC GCCTGCGGGA
P875604      937  AGCTCGGGTC GCTGGGGCGG GTGACAGGGT CATCCAGGTC GCCTGCGGGC
                  ********** ********** ********** ********** **********
                  ********** ********** ********** ********** **********
                  ********** ********** ********** ********** **********
                                 ****** ********** ********** **********

alignment
position          1601...... 1611...... 1621...... 1631...... 1641......

P875598     1202  ---------- ---------- ---------- ---------- ----------
P875602      960  CGCAggaACG GGAAGCGGGC CAGGGCCTGA GCCTGGGCGG CCTCCTCccg
P875604      987  CGCAgggACG GGAAGCGCGC CAGGGCTAGG GCTTGGGCGG CCTCCTCcgc
                  ****   *** ********** ********** ********** *******
                  ****   *** ********** ********** ********** *******
                  ****
                  ****

alignment
position          1651...... 1661...... 1671...... 1681...... 1691......

P875598     1202  ---------- ---------- ---------- ---------- ----------
P875602     1010  cttgcCCTTC GCAGTCTCTG TACAAGTCAA TTTTTAAAAT AAAACtcagt
P875604     1037  ttgt-CCTTC GCAGTCTCTG CGCAAGTCGA TTTTTAAAAT AAAACcaagt
                       ***** ********** ********** ********** *****
                       ***** ********** ********** ********** *****
                       ***** ********** ********** ********** *****

alignment
position          1701...... 1711...... 1721...... 1731...... 1741......

P875598     1202  ---------- ---------- ---------- ---------- ----------
P875602     1060  -CGCAGGAGG TGtgctaccg tgctatctat ctatctatct atctatctat
P875604     1086  cCGCAGGCGG TGctcccacc gccacagaga ggaacagctc gtggtggcac
                   ********* **

alignment
position          1751...... 1761...... 1771...... 1781...... 1791......

P875598     1202  ---------- ---------- ---------- ---------- ----------
P875602     1109  cta--TATAT CTAGTTCTAG CAGTTTCAGT CATtTTTGTC TTTCTAGTTA
P875604     1136  cgtgcTATAC CGAGTTCTAG CAGTTTCAGT CAT-TCTGGC CTTCTAGTTA
                       ***** ********** ********** ***  ****** **********
                       ***** ********** ********** ***

alignment
position          1801...... 1811...... 1821...... 1831...... 1841......

P875598     1202  ---------- ---------- ---------- ---------- ----------
P875602     1157  ATTTTTTAAG Aagccggatt tatagtcatt ggccaaagcc atgtatatta
P875604     1185  ATCTTTAAAA Agctgcg--- ---------- ---------- ----------
                  ********** *

alignment
position          1851...... 1861...... 1871...... 1881...... 1891......

P875598     1202  ---------- ---------- ---------- ---------- ----------
P875602     1207  cagcccttgg cttgatgtaa ggaagttcac cctgtgcctt cgcatcagta
```

P875604 1202 -

Pairwise similarities:

For each pairwise alignment, the similarity (relative to the maximum similarity) and the number of identical nucleic acids (in % of shorter sequence) is given. Maximum values are underlined.

	**P875602 (1256 bp)**	**P875604 (1201 bp)**
**P875598 (1201 bp)**	0.300 32 %	0.294 30 %
**P875602 (1256 bp)**		1.000 74 %

Please note that the **similarity value 1.000** marks only the two **most similar** sequences, it does not necessarily mean that these sequences are identical.

**Extract alignment region**	
**Positions in alignment**	from: to:
**Output file**	dialign.seq

Extract aligned sequences

For comments, questions or bug reports, please contact support@genomatix.de

© Genomatix Software GmbH 1998-2005 - All rights reserved. License Agreement GEMS Launcher 4.1.1

Genomatix-Logo Logout Personal Messages GEMS-Logo

Genomatix_MainGEMSElDoradoGene2PromoterBiblioSphere
Frequently_asked_questionsYour_ResultsYour_SequencesYour_ProtocolHelp

GEMS Launcher Task: *DiAlign TF*: Multiple alignment plus TF sites
working on promoters.seq (3 seq.)

[Alignment] [Pairwise_similarities]

DiAlign professional TF Release 3.1 December 2004 **Thu May 5 21:49:15 2005**

Solution parameters:

**Sequence file:**	promoters.seq (3 seq.)
**Type of sequences:**	nucleotide sequences
**Threshold T:**	0.00
**'*' signs below alignment denote:**	diagonal similarity (max. similarity is represented by 10 '*' signs)
**Alignment output:**	complete alignment is shown<br># nucleic acids per line: 50
**TF output:**	common TF matches located in aligned regions (common to 3 (100.0 %) sequences)
**Family matches:**	yes
**MatInspector library:**	Matrix Family Library Version 5.0 (February 2005)
**Selected groups** (core/matrix sim):	• ALL user_defined.lib (0.75/Optimized)<br>• ALL vertebrates.lib (0.75/Optimized)

Aligned Sequences:

No.	Sequence Name	Sequence Description	Length
1	P878122	sym=COX5A|loc=Loc9377|taxid=9606|spec=Homo sapiens|chr=15|ctg=NT_010194|str=(-)|start=46020090|end=46021207|len=1118| tss=501,505,518| comm=cytochrome c oxidase subunit Va; (AK026623/548/gold;AK026615/552/gold;NM_004255/565/silver;)	1118 bp
2	P878123	sym=Cox5a|loc=Loc12858|taxid=10090|spec=Mus musculus|chr=9|ctg=NT_039474|str=(+)|start=3799809|end=3800917|len=1109| tss=501,509| comm=cytochrome c oxidase, subunit Va; (AK018723/552/gold;NM_007747/560/silver;)	1109 bp
3	P878124	sym=Cox5a|loc=Loc252934|taxid=10116|spec=Rattus norvegicus|chr=8|ctg=NW_047799|str=(+)|start=30893983|end=30895083| len=1101|tss=501| comm=cytochrome c oxidase, subunit Va; (NM_145783/525/bronze;)	1101 bp

Alignment (DiAlign format):

Please note that only **upper-case** letters are considered to be **aligned**. For more information, please have a look at the user guide.

V$EGRF V$SP1F V$MTF1 V$MOKF V$EREF U$EREF V$RARF V$MINI V$EBOX V$AP2F V$ETSF U$NRF2 V$MYOD V$NEUR V$GREF

```
alignment
position        1 . . . . . . . . .  1 1 . . . . . . . .  2 1 . . . . . . . .  3 1 . . . . . . . .  4 1 . . . . . . . .
P878122      1  g - - - - - - - - -  - - - - - - - - - -  - - - - - - - - - -  - - - - C C T G G C  T A A T T T T G T A
P878123      1  c c a a a a t g t t  g a t t a t t g t c  t t g A G T C A C T  A C T C C C A G T A  T G A T T T T T T T
```

```
P878124        1 ---------- ---------- ---AGACGCC ACTCCCAGTA TGAaaaaaaa
                                           ******* ********** **********

alignment
position           51........ 61........ 71........ 81........ 91........

P878122       18 T---TTTTAG TAGAGACGGG ggttcctcca tgttgcccag gctggtcttg
P878123       51 T---TTTTCT GAGACTCAGG TTGCATAAGA GAGCCTGAAA TTCCtaataC
P878124       28 ttccTTTTCT GAGACTCAGG ATGCATAGGA GAGTTTGAAA TTCCtaatcC
                 *    ****** ********** ********** ********** ****     *
                     ****** ********** ********** ********** ****     *
                     ****** ********** ********** ********** ****     *
                                                                      *

alignment
position           101....... 111....... 121....... 131....... 141.......

P878122       65 aactcccgac cgcaggtgat ccgcccgcct cggcctccca AAAGTGCTGG
P878123       98 TTAGCCTGT- ---------- ---------- ---------- AGAGGGCTAG
P878124       78 TTAGCCTGT- ---------- ---------- ---------- AGAGGGCTGG
                 *********                                   **********
                 *********                                   **********
                 *********                                   **********
                 *********                                   **********

alignment
position           151....... 161....... 171....... 181....... 191.......

P878122      115 GATTACAGgc atgagccaCA GCGCCCGGGC GCAAAGtacG TATTTTTTAT
P878123      117 AATTATAGCA TACAC---CA ACGCGCGGGG GCAAAGCG-G TTATTCTTAT
P878124       97 GATTATAGCA TACAC---CA Atacct-GGG GAAAAGCG-G TTATTCTTAT
                 ********** *****   ** ********** ********  * **********
                 ********** *****   ** *      *** ********  * **********
                 ********** *****   ** *      *** ********  * **********
                 ********** *****   ** *      *** ******    * **********
                                                               *******

alignment
position           201....... 211....... 221....... 231....... 241.......

P878122      165 TATAGTTTGT TGTTATTGCT GTCAttccta ttttgaggtg attaagtgcC
P878123      163 TATAGTTTGC CGTTATTAtc GCTACAATTC CAAATTGTGA TAGTCT---C
P878124      142 TACAGTTTAT CGTTATTATT GCCACATTTC GATTTCGTGA TAGTCT---C
                 ********** ********** ********** ********** ******   *
                 ********** ********   ********** ********** ******   *
                 ********** ********   ****
                 ********** ********
                 ********** *******

alignment
position           251....... 261....... 271....... 281....... 291.......

P878122      215 CTCCTGGGCC TGACTCGAAC AAGATCACGG TCGGTTGCT- ----------
P878123      210 CTCTGTTGGC Ga--TTCCAA ATCCACATTG TCCATTGCTC ACCCTGCCTT
P878124      189 CTCCGTTGGC GGATTTCAAA ATCCACATTG TCCATTGCTC GCCCTGACTT
                 ********** ********** ********** ********** **********
                 ********** *    ****** ********** ********** **********
                                ****** ********** ********** **********
                                ****** ********** ********** **********

alignment
position           301....... 311....... 321....... 331....... 341.......

P878122      254 -CCTCCCTCG Cctccattcc tacttacagg atggcagctg CACGTCACCT
P878123      258 TCCTCCCACG CAGGAATGTT CACTACGGTC ACC------- CGGGTCATCG
P878124      239 TCCTCCCACG CAGGAATGTT CACTACAGCC AGC------- CAGGTCATCG
                 ********** ********** ********** ***        **********
                 ********** ********** ********** ***        **********
                 ********** ********** ********** ***        **********
                 ****

alignment
position           351....... 361....... 371....... 381....... 391.......

P878122      303 GACCAGagac aaggcgggac gcgaggctaa caGTTTGTCC TCAGCGCGCC
P878123      301 GACCAGCTTT GGCGCGGCGC CGACGTG--- --GTTTGTCC TCCACG-GCC
```

```
P878124    282 GACCAGCACT GACTCGGCGC AGACGTG--- --GTTTGTCC TCGACGCGCC
               ********** ********** *******      ******** **********
               ********** ********** *******      ******** **********
               ********** ********** *******      ******** ******  ***
                                                  ******** ******  ***
                                                                   ***

alignment
position       401....... 411....... 421....... 431....... 441.......

P878122    353 GCCGTAGCCC CCGCCGccgc cggccgcgcg gtggctcctc ccgcccACGC
P878123    345 GCCGTAGCCG CCGGCGACCG CATGGCGCCC CACCCGCTTC CGT---AGGC
P878124    327 GCCGTAGTGA CCGGCGACCG CATGGTGCCC CACCCGCTTC CGT---AGGC
               ********** ********** ********** ********** ***   ****
               ********** ********** ********** ********** ***   ****
               ********** ********** ********** ********** ***   ****
               *******                             ******* ***   ****
               *******                                           ****

alignment
position       451....... 461....... 471....... 481....... 491.......

P878122    403 GCCAGAGTCG CAGTGGGCGG GCCTacgTGC TCCGCCCgct gtgagcct--
P878123    392 GCGAGCTCCG CAGTGGGCCG GCCTTA-TGC TCCGCCCAGC GTGCGTCGCT
P878124    374 GCGAGCTCTG CAGAGGGCCG GCCTTA-TGC TCCGCCCAGC GTGCGTCGCT
               ********** ********** ******  *** ********** **********
               ********** ********** ******  *** ********** **********
               ********** ********** ******  *** ********** **********
               ********** ********** ******  *** ********** **********
               ********** ********** ***     *** ********** **********

alignment
position       501....... 511....... 521....... 531....... 541.......

P878122    451 ------GTCC GGCCCCCGCc cgctccggag caaCCCGCGA GCTTACACCG
P878123    441 GCGTGAGTCC GGCCCCCGCG AACTGCTGTA CCGCCCGCGA GCTCACACCG
P878124    423 GCGTGAGTCC GGCCCCCaaG AACTGCTGTG CCGCCCGCGA GCTCACACCG
               ********** ********** ********** ********** **********
               ********** *******  * ********** ********** **********
               ********** *******  * ********** ********** **********
               ********** *******  * ********** ********** **********
               ********** *******                  ******* **********
                     **** *******                  ******* **********
                                                   ******* **********
                                                   ******* **********

alignment
position       551....... 561....... 571....... 581....... 591.......

P878122    495 GCTTCTCTct gtCCTCAGCC CGCGCGCCGC CATCGCCGTC ATGCTGGGCG
P878123    491 GCTTCTCgTC AGCCTCAGCC AGTGCGCTGT GGTCGCCGTC ATGCTCGCCG
P878124    473 GCTTCTCTTC AGCCCCAGCC AGTGCGCTGT GGTCGCCGTC ATGCTCGCCG
               ********** ********** ********** ********** **********
               ********** ********** ********** ********** **********
               *******  ** ********** ********** ********** **********
               *******  ** ********** ********** ********** **********
               *******       ******** ********** ********** **********
               *******                            ******** **********
               *******                                      *********
               *******                                      ****

alignment
position       601....... 611....... 621....... 631....... 641.......

P878122    545 CCGCTCTCCG CCGCTGCGCT GTGGCCGCaa ccacccgggc cgacccctCGA
P878123    541 CTGCCCTCCG TCGCTGTACC GCAGCCGCGG CtGCC----- -------CGA
P878124    523 CTGCCCTCCG TCGCTGTACC GCAGCCGCGG CcGCC----- -------CGA
               ********** ********** ********** *  ***             ***
               ********** ********** ********** *  ***             ***
               ********** ********** ********** *  ***             ***
               ********** ********** ********** *  ***             ***
               ********** ********** ********** *                  ***
               ********** ********** ********                      ***
               ********** ******                                   ***
                                                                   ***
```

```
alignment
position         651.......  661.......  671.......  681.......  691.......

P878122     595  GGCCTCCTGC  ACTCCGCCCG  GACCCCCGGC  CCCGCCGtgG  GTAAGGCCCC
P878123     579  GGCCTCCTGC  ACCCCGCCTC  GGCTCCCAGC  CCCGCCGCCG  GTAAGGTCCG
P878124     561  GGCCTTCTGC  ACCCCGTCTC  GGCTCCCAGC  CCCGCt GCCG  GTAAGGCCCG
                 **********  **********  **********  **********  **********
                 **********  **********  **********  **********  **********
                 **********  **********  **********  **********  **********
                 **********  **********  **********  ***** *  *  **********
                 **********  **********  **********  ****  *
                 **********  **********  **********  ****
                 **********  **********  **********  ****
                 **********  **********  **********  ****

alignment
position         701.......  711.......  721.......  731.......  741.......

P878122     645  TGCTTGCCAC  TGCCCGCGCC  CTCGTGTCCT  TGCGGTGACC  TGGGTCCCCG
P878123     629  CGCCCGCCAC  AGCCCTCGCT  CGCGTGTCCT  TGCTGTGACC  TGGGCTCCCG
P878124     611  CTCCCGCCAC  AGCCCGCGCC  CGCGTGTCCT  TGCTGTGACC  TGGGCTCCCG
                 **********  **********  **********  **********  **********
                 **********  **********  **********  **********  **********
                 **********  **********  **********  **********  **********
                 **********  **********  **********  **********  **********
                      *****  **********  **********  **********  **********
                      *****  *********   **********  **********  **********
                      *****  *********   **********  **********  **********
                                         **********  **********  ****  ****
                                         **********  **********  ****
                                         **********  **********  ****

alignment
position         751.......  761.......  771.......  781.......  791.......

P878122     695  CTGCCACTCC  GGGGCGGGCT  Ctccc-CGGC  CTAGCCCTTG  CCCTacccctg
P878123     679  CCGCCACTCG  AGGGCAGGCT  CCCTGGCGGC  CTAGCCTTTG  CCCTGCgAGC
P878124     661  CCGCCACTCG  AGGGCAGGCT  CCCTGGCGGC  CTAGCCTTTG  CCCTGCaAGC
                 **********  **********  **********  **********  ****** ***
                 **********  **********  **********  **********  ****** ***
                 **********  **********  **********  **********  ******
                 **********  **********  **********  **********  ******
                 **********  **********  *     ****  **********  ****
                 **********  *********         ****  **********  ****
                 **********  *********
                 **********

alignment
position         801.......  811.......  821.......  831.......  841.......

P878122     744  gttact--GA  ACCCGGTGAC  CGCGGGCCGC  CCtgcCCGCC  GCCTCGAGTA
P878123     729  CGGCATTGGA  GCCCGGTGAC  CGCAGTGCGT  CCgcg--GCC  GTCTGGAGTC
P878124     711  CGGCAGTGGA  GTCCGGTGAC  CGCAGGgtat  cca--CTGCC  GTCTGGAGTT
                 **********  **********  **********  **   *****  **********
                 **********  **********  *****              ***  **********
                         **  **********  *****              ***  **********

alignment
position         851.......  861.......  871.......  881.......  891.......

P878122     792  GTGGAGGAcc  tgaaagcgcg  cCCCCGGGGC  CTCCCCCCGC  GCAACTTCCT
P878123     777  GTGTAGGAGA  CT--------  -CCTCCGGGC  CTCCCTCCCC  GCCACTTCCT
P878124     759  ATGTGGGAGA  CT--------  -CCCCGGGGC  CTCCCT-CCC  GCCACTTCCT
                 **********  **           *********  **********  **********
                 **********  **           *********  **********  **********
                 **********  **           *********  ****** ***  **********
                                          *********  ******
                                          *********  *****

alignment
position         901.......  911.......  921.......  931.......  941.......

P878122     842  Ggctgtgcca  CCGCGGACAG  gtcaga-TGT  GGACCTGCTG  TGGGCGCCGA
P878123     818  GATGCCGaac  CCGCGGCCAG  TCAGGTGTGT  GGACTTGCCG  GGGTCGCCGA
P878124     799  GATGCCGgat  acaCGACCGG  TCAGGTGTAT  GGACCTGCCC  GGGTCGCTAA
```

```
                  *******    ********** ********** ********** **********
                  *******       ******* ********** ********** **********
                  *                 *** ********** *******

alignment
position          951.......  961.......  971.......  981.......  991.......

P878122      891  GGAGGGGACG CAGCGCTCCC CTTCGGTTCC CGGGCCATGA CCCTGCCCca
P878123      868  GGAcga-GCT CAGCACTTCC TTTCAGTCTC TGGGCCGTGA CCGTTCCCTG
P878124      849  CGACAGGGCT CATCACTTCC TTTCAGTCTC TGGGCCGTGA CCGTTCCCTG
                  ********** ********** ********** ********** **********
                  ***    *** ********** ********** ********** **********
                         *** ********** ********** ********** **********
                         *** ********** ********** ********** **********
                          ** ********** ********** ********** **********
                                                                 *****
                                                                 *****

alignment
position          1001......  1011......  1021......  1031......  1041......

P878122      941  gcgccGACCC TGCGGCCACC TGACCGAGAG TGGCCGCCGC CGGCCtgcaG
P878123      917  CGCT-GACCC CGCAGCCACC TGACCGGGAG TGGCCCCCGC CGGCCTGCGG
P878124      899  CGCT-GACCC CGCAGCCACC TGACCGGGAG TGGCTCCCGC CGGCCTACGG
                  **** ***** ********** ********** ********** **********
                  **** ***** ********** ********** ********** **********
                  **** ***** ********** ********** ********** **********
                  **** ***** ********** ********** ********** **********
                  **** ***** ********** ********** ********** *****    *
                       ***** ********** ********** ********** *****    *
                       ***** ********** ********** **** ***** *****    *
                       ***** ********** ********** **** ***** *****
                       ***** ********** ********** **** ***** *****
                       ***** ********** ********** ****

alignment
position          1051......  1061......  1071......  1081......  1091......

P878122      991  CGCCTCGGCG AAGGTGACAT TGAGCCTCCG GGCACGCCGC ttgggg----
P878123      966  CATCTTGGCG AAGGTGACAT TGAGCCTCCG GGCGCGCCGC CCGCCTCTGC
P878124      948  CGTCTTGGCG AAGGTGACAT TGAGCCTCCG GGCGCGACGC CCGCCTCTGC
                  ********** ********** ********** ********** **********
                  ********** ********** ********** ********** **********
                  ********** ********** ********** ********** **********
                  ********** ********** ********** ********** **********
                  ********** ********** ********** ********** **********
                  ********** ********** ********** **********
                  ********** ********** ********** **********
                     ******* ********** ********** **********
                     ******* ********** ********** ***
                     ******* ********** ********** ***

alignment
position          1101......  1111......  1121......  1131......  1141......

P878122     1037  ---TCCGAGG ACACAATATA CTTCCgta-T GGGACACAcg attatacttt
P878123     1016  GTTTCCTAGA ACACAATTTG ATTCCCCAGT GGGACACAAT ACCCTTTTAG
P878124      998  GTTTCCTAGA ACACAATTCG ATTCCCCAGT GGGACACAAT ACCCTTTTAG
                  ********** ********** ********** ********** **********
                  ********** ********** ********** ********** **********
                  ********** ********** ********** ********** **********
                  ********** ********** ********** ********** **********
                  ********** *****

alignment
position          1151......  1161......  1171......  1181......  1191......

P878122     1083  tc--AATCAG GCATTTCTCG GTTAGCGATT -AGTGTGtc- ----------
P878123     1066  CCCTgATCAG GCAATTCTCT GCTGGCATTT tTGTGTCACC TTCA------
P878124     1048  TCCTAATCAG GCAATTCTCT GCTGGCATTC -TGTGTCACC TTCAtaacgg
                  ********** ********** **********  ********* ****
                  **** ***** ********** *********
                  **** ***** ********** *********
                  **** ***** ********** *********
```

alignment position		1 2 0 1 .
**P878122**	1119	- - - - -
**P878123**	1110	- - - - -
**P878124**	1097	g t a c g

Pairwise similarities:

For each pairwise alignment, the similarity (relative to the maximum similarity) and the number of identical nucleic acids (in % of shorter sequence) is given. Maximum values are underlined.

	**P878123 (1109 bp)**	**P878124 (1101 bp)**
**P878122 (1118 bp)**	0.267<br>52 %	0.238<br>52 %
**P878123 (1109 bp)**		1.000<br>87 %

Please note that the **similarity value 1.000** marks only the two **most similar** sequences, it does not necessarily mean that these sequences are identical.

**Extract alignment region**	
**Positions in alignment**	from: to:
**Output file**	dialign.seq

Extract aligned sequences

For comments, questions or bug reports, please contact support@genomatix.de

© Genomatix Software GmbH 1998-2005 - All rights reserved.

License Agreement

GEMS Launcher 4.1.1

Genomatix-Logo Logout Personal Messages GEMS-Logo

Genomatix_MainGEMSElDoradoGene2PromoterBiblioSphere
Frequently_asked_questionsYour_ResultsYour_SequencesYour_ProtocolHelp

GEMS Launcher Task: *DiAlign TF*: Multiple alignment plus TF sites
working on promoters.seq (3 seq.)

[Alignment] [Pairwise_similarities]

DiAlign professional TF Release 3.1 December 2004 **Thu May 5 22:14:45 2005**

Solution parameters:

**Sequence file:**	promoters.seq (3 seq.)
**Type of sequences:**	nucleotide sequences
**Threshold T:**	0.00
**'*' signs below alignment denote**:	diagonal similarity (max. similarity is represented by 10 '*' signs)
**Alignment output:**	complete alignment is shown<br># nucleic acids per line: 50
**TF output:**	common TF matches located in aligned regions (common to 3 (100.0 %) sequences)
**Family matches:**	yes
**MatInspector library:**	Matrix Family Library Version 5.0 (February 2005)
**Selected groups** (core/matrix sim):	• ALL user_defined.lib (0.75/Optimized)<br>• ALL vertebrates.lib (0.75/Optimized)

Aligned Sequences:

No.	Sequence Name	Sequence Description	Length
1	P875606	sym=COX5B|loc=Loc1329|taxid=9606|spec=Homo sapiens|chr=2|ctg=NT_022171|str=(+)|start=328045|end=329345|len=1301| tss=501| comm=cytochrome c oxidase subunit Vb; (NM_001862/524/bronze;)	1301 bp
2	P875607	sym=Cox5b|loc=Loc12859|taxid=10090|spec=Mus musculus|chr=1|ctg=NT_039170|str=(+)|start=14310302|end=14311631|len=1330| tss=501,530| comm=cytochrome c oxidase, subunit Vb; (AK008339/530/gold;NM_009942/501/bronze;)	1330 bp
3	P875608	sym=Cox5b|loc=Loc94194|taxid=10116|spec=Rattus norvegicus|chr=9|ctg=NW_047814|str=(+)|start=1737477|end=1738777| len=1301|tss=501| comm=cytochrome c oxidase subunit Vb; (NM_053586/531/bronze;)	1301 bp

Alignment (DiAlign format):

Please note that only **upper-case** letters are considered to be **aligned**. For more information, please have a look at the user guide.

V$EGRF V$NRF1 U$NRF1 V$ETSF V$NFKB V$SP1F V$STAT U$NRF2 V$XBBF V$SF1F V$MYT1 V$RARF
V$RORA V$EREF U$EREF

```
alignment
position        1 . . . . . . . . .  11 . . . . . . . . .  21 . . . . . . . . .  31 . . . . . . . . .  41 . . . . . . . . .

P875606       1 c g a a a c t g - -  - - - - - - - - - -  - - - - - - - - - -  - - - - - - - - - -  - G G C C A G G A C
P875607       1 t t a a a a a a c a  a a a c a a A A C A  G A G T G G A G G a  - - G G G A A T T G  A G G C T A A G A C
P875608       1 - - - - - - - - - -  - - - - - - A A G A  G A G T G G A G G c  g c G G G G A T T G  A G G C T A A G A C
```

```
                                  ****  *********      ******** **********
                                                       ******** **********
                                                                 *********

alignment
position            51........ 61........ 71........ 81........ 91........

P875606         18  TGCAGGTAG- -------GTC CCGTCCAATG Cgacacgctc gcgacggccc
P875607         49  TGTGtgtgtg ggggggtGTA CTGTCAATTT CTGACggAGT GGGCCATTGC
P875608         35  TGTGGGGAG- -------GTC CTGTCAAATT CTGACccAGT GGGCCATTGC
                    *********         *** ********** *****  *** **********
                    ****              *** ********** *      *** **********
                    ****                                    *** **********

alignment
position            101....... 111....... 121....... 131....... 141.......

P875606         60  acgcactgc- ---------- ---------- -CAGAACCAG CCCTGCCACT
P875607         99  AACAATTTGG TAACAACTAA AGCT--GTCT GCAGTCTCGA ACCCGTGGCT
P875608         77  AACAAGTTGG TAATGGCTAA AGCTctGTCT GCAGACACGA ACCTGCAGCT
                    ********** ********** ****  **** ********** **********
                    ********** ********** ****  **** ********** **********
                    ********** ********** ****  **** ********** **********

alignment
position            151....... 161....... 171....... 181....... 191.......

P875606         88  AACCAGCCTA GTAAggaacg cagtgatCTT TAGGAGCCCA CTTCTAAATT
P875607        147  AACAGGCATA ACAAACaCAA CAGG---CTT CCTGAGCTCA CGCCCAAGGT
P875608        127  CACAGGCATA ACAAACtCAA CAGT---CTT CCCGAGCTCA CGCCCAAGGT
                    ********** ****** *** ****   *** ********** **********
                    ********** ****** *** ****   *** ********** **********
                    ********** ****** *** ****   *** ********** **********
                                      *** ****   *** ********** **********

alignment
position            201....... 211....... 221....... 231....... 241.......

P875606        138  CAAATCATTT GGGGTTCAgt aagtctcttc acagacgggg acctaatagc
P875607        194  TGTATGATTC GTGGTggaag cccttgactg cggga-ACTT CGGCTTCCAC
P875608        174  TGTACCATTC GTGCTTCAaa tccctaactg agagggACTT CGGCTTCCAC
                    ********** ********                    **** **********
                    **********                             **** **********
                    **********                             **** **********
                    **********                             **** **********

alignment
position            251....... 261....... 271....... 281....... 291.......

P875606        188  ttccattagc agcctccacg tttgctgaac acgactacgg ggccagacCC
P875607        243  AGCACCTCCA ---------- ---------- ---------- --------CT
P875608        224  AGCACCTCCA ---------- ---------- ---------- --------CT
                    **********                                         **
                    **********                                         **
                    **********                                         **
                    **********                                         **

alignment
position            301....... 311....... 321....... 331....... 341.......

P875606        238  ACAGGAACTC CAATGTGGTT CAGCCGCTca actccaggga ccaaaaagac
P875607        255  ACAAGAACTA CTATGTGGCT GGGACACTGG GCGCGAACAC TCAAGCGGAc
P875608        236  AC-------- -TAAGTGGCT TCGACTCTGG GGCGCGAGCA GCAAGCGGAa
                    ********** ********** ********** ********** *********
                    **
                    **
                    **

alignment
position            351....... 361....... 371....... 381....... 391.......

P875606        288  tttcccacct cccagcgtta ttaaaggcaa ccc------- ------CGCC
P875607        305  aggtcatgaa ccCGAAAGCC GCTAGCTGTC AGGGTGACTG ACGCCACGCC
P875608        277  cagtca---- --CGATAGCC ACAAGGTATC ACGGTAACTG ACGCTACGCC
                                 ******** ********** ********** **********
                                 ******** ********** ********** **********
```

```
alignment position
            401....... 411....... 421....... 431....... 441.......
P875606 325 CCCAAGcctc agcca--CTA CGCGGTGCAG AAAGAGGCAG AGCTGGGCAT
P875607 355 CCggAGGCGG CGGGAGTCTG CGCGGCCCAC GAAGAGGCAG AACTGCGCAT
P875608 321 CCCAAGGCGG CGGGAGTCTG CGCGACCGGC GAAGAGTCAG AGCTGCGCAT
            ********** ********** ********** ********** **********
            **  ****** ********** ********** ********** **********
                ****** ********** ********** ********** **********
                              *** ********** ********** **********
                              *** ********** ********** **********
                                              ********* **********

alignment position
            451....... 461....... 471....... 481....... 491.......
P875606 373 GCGCAAtttc cgcccccgcc acagtcttgg gagtgggcgg gctcgagggc
P875607 405 GTGCGGCGTC TACTTTTAGC GCTGCTTCGG CTGGtc-GG- ----------
P875608 371 GTGCAACATC TACCTTTAGC GCTTCTTCGG CTGGccgGG-  ----------
            ********** ********** ********** ****   **
            ********** ********** ********** ****   **
            ********** ********** ********** ****   **
            ******                                  **
            ******
            ****

alignment position
            501....... 511....... 521....... 531....... 541.......
P875606 423 tGGGAGGAAG CCGAAAGCTG ACCGAGAGga gaaAGAAGCC CGCCCCCGGA
P875607 443 -GGCAGGAAG CGGAAGACTG ACCGGGCGTT GTTAGACTCC CACCAACGGA
P875608 410 -GGCAGGAAG CGGAAGGCTG ACTGGGCGTT GTTAGACaCC TACCAACGGA
             ********* ********** ********** ********** **********
             ********* ********** ********** ******* ** **********
             ********* ********** ********** ******* ** **********
             ********* ********** ********** *******          ****
             ********* ********** ********      ****
             ********* ********** ********

alignment position
            551....... 561....... 571....... 581....... 591.......
P875606 473 AGTCCCTCCT GTCTCtg-CA GCTTGTTCCC GGAAGTTTTG CTGCTAGTCG
P875607 492 AGTCCCGCCC ATCTTGCTCA GCCTGTTCCC GGAAGTGCAT CTGCTTGTCT
P875608 459 AGTCCCGCCT GTCTTGCTCt gtCTGTTCCC GGAAGTGCAT CTGCTTGTCT
            ********** ********** ********** ********** **********
            ********** ********** ********** ********** **********
            ********** *********    ******** ********** **********
            ********** ****    *    ******** ********** **********
                                  ******** ********** **********
                                  ******** ********** **********
                                  ******** ********** **********
                                   ******* ********** **********

alignment position
            601....... 611....... 621....... 631....... 641.......
P875606 522 CGGACGcaAT GGCTTCAAGG TTACTTCGCG GAGctggaac gcTGGCCGCG
P875607 542 CGGGCGAGAT GGCTTCAAGG TTACTTCGCG GAGTGGGCGC TTTGGCGGCG
P875608 509 CGAGCGAGAT GGCTTCAAGG TTACTTCGCG GAGTAGGCGC TTTGGCGGCT
            ********** ********** ********** ********** **********
            ********** ********** ********** ********** **********
            ********** ********** ********** ********** **********
            ********** ********** ********** ********** **********
            ********** ********** ********** ********** *********
            ******  ** ********** ********** ***          *******
            ******  ** ********** ********** ***          *******
            **      ** ********** ********** ***          *******
                    ** ********** ********** ***          *******
                    ** ********** ********** ***

alignment position
            651....... 661....... 671....... 681....... 691.......
P875606 572 CAGGCCCTGA GGGCTCGCGG CCCCaGTGGC GCGGCCGCGA TGCGCTCCAT
```

```
P875607     592 CAGGCCCTGA GGGCCCACGG CCCCCGTGGC GCGGCCGTGA CCCGCTCCAT
P875608     559 CAGGCCCTGA GGGCCCACGG ACCCCGTGGC GTGGCCGCTA CCCGCTCCAT
                ********** ********** ********** ********** **********
                ********** ********** ********** ********** **********
                ********** ********** ********** ********** **********
                ********** ********** ********** ********** **********
                ********** ********** **** ***** ********** **********
                ********** ********** **** ***** ********** **********
                ********** ********** **** ***** *******  * **********
                ********** ********** **** ***** *******  * **********
                                           ***** *******  * **********
                                           ***** *******  * **********

alignment
position            701.......  711.......  721.......  731.......  741.......

P875606     622 GGCATCTGGA GGTACtcggg tctCCGGGCG TGCCAGGgac cagagtgttg
P875607     642 GGCTTCTGGA GGTACGTGAC CAGCTGGGCG GGACAGGC-- ----------
P875608     609 GGCTTCTGGA GGTAGGTGAC TAGCTGGGTG CGCCAGGT-- ----------
                ********** ********** ********** ********
                ********** ********** ********      *****
                ********** ********** ********      *****
                ********** ********** ********
                ********** *****
                ********** *****
                ********** *****
                ********** ****
                ********** ****

alignment
position            751.......  761.......  771.......  781.......  791.......

P875606     672 ccctcccagg gtggtcccag ggcggcaaag cggcgcggct cgtgcagctt
P875607     680 ---------- ---------- ---------- ---------- ----------
P875608     647 ---------- ---------- ---------- ---------- ----------

alignment
position            801.......  811.......  821.......  831.......  841.......

P875606     722 ctcgaggtcc cagtggccgc tttaCGGTCC CCAGTGCCTC AGGCtctgca
P875607     680 ---------- ---------- ----CGGTGT CCATGGCCTC CGGCGTGGTT
P875608     647 ---------- ---------- ----CGGTGT CCTTGGCCTC CGACGTGGTT
                                          ****** ********** **********
                                          ****** ********** **********
                                          ****** ********** **********

alignment
position            851.......  861.......  871.......  881.......  891.......

P875606     772 ggca------ ---------- ---------- ---------- ----------
P875607     706 GGTgATGGGC GGTCGAGCGG GGTCGGCCAT TAGcgttctC TCGAGCCCTG
P875608     673 GGTaATGGGC GGTCGAGCGG GGTCGGCCAT TAGagctccC TCGAGCCCTG
                *** ****** ********** ********** ***      * **********
                *** ****** ********** ********** ***      * **********
                *** ****** ********** ********** ***      * **********
                    ****** ********** ********** ***      * **********
                                                          * **********

alignment
position            901.......  911.......  921.......  931.......  941.......

P875606     776 ---------- ---------- ---------- ---------T CTCCCTGTAA
P875607     756 CTGACCACCT TAGGCTCCGG GAGCCGGCGc tctgtCCGGT CTTGCTGTAA
P875608     723 CTGACCACCT TAGTCTCCGG GAGCCGGCGc cccgcCCGGT CTTGCTGTAA
                ********** ********** *********      ***** **********
                ********** ********** *********      ***** **********
                ********** ********** *********      ***** **********
                ********** ********** *********      ***** **********
                ********** ********** *********

alignment
position            951.......  961.......  971.......  981.......  991.......

P875606     787 TTctggaccg ctgctcCTGC CGCTCCCCGA ACTCACTCCG CTGCGAAAGT
```

```
P875607     806 TTT------- ------CGGC CGCCCACCCG ATCGAACCCG CTTTCAAAGT
P875608     773 TTT------- ------CGGC CGCTCTCCCG ATTGACCTCG CTTTCAAAAT
                ***              **** ********** ********** **********
                ***              **** ********** *****   ** **********
                ***              **** ********** *****   ** **********
                ***              **** ********** *****   ** **********
                                                         ** **********

alignment
position        1001...... 1011...... 1021...... 1031...... 1041......

P875606     837 ATCCTAAacg g-AGGTGCCG GGTGACCTTG GGAGggaccg gggct-----
P875607     843 GCCCTCACTG GAAGGTGCCG GGTGACCTTG GAACACAAGG ACTTGGAGGG
P875608     810 GCCCTAACTG GAAGGTGCCG GGTGACCTTG GGAGATAGGG ACTTGGAGGG
                ********** ********** ********** ********** **********
                ********** ********** ********** ********** **********
                ********** ********** ********** ********** **********
                ********** ********** ********** ****
                ********** ********** ********** ****
                             ******** ********** *
                             ******** *******
                             ******** *******

alignment
position        1051...... 1061...... 1071...... 1081...... 1091......

P875606     881 ---------- ---------- ---------- ---------- ----------
P875607     893 CTGAAAAAGC GCTGGACCaA CATTCAAATC CCTTGCCCGC CACCAGCCAG
P875608     860 AAGAGAAAGC GCTGGACCgA CATTCAAATC ACTTGCCCGC CTCCAGCCAA
                ********** ******** * ********** ********** **********
                ********** ******** * ********** ********** **********
                ********** ******** * ********** ********** **********
                                    * ********** ********** **********

alignment
position        1101...... 1111...... 1121...... 1131...... 1141......

P875606     881 ---------- ---------- ---------- ---------- ----------
P875607     943 TATACTCTCa -TGACTTGAT TGTAGGGAAA GTCGACAACC AAGCTTCCGC
P875608     910 TATACTGTCa tTGACTTGAT TTTAGGGAAA GTCGAAAACC AAGCTTCCGC
                *********   ********* ********** ********** **********
                *********   ********* ********** ********** **********
                *********   ********* ********** ********** **********
                *********   ********* ********** ********** **********
                            ********* ********** ********** **********

alignment
position        1151...... 1161...... 1171...... 1181...... 1191......

P875606     881 ---------- ---------- ---------- ---------- ----------
P875607     992 GgtgGGAAGG GAAAAACGGT ATGGCTTGGG AGTCATGCCT CAGTGGGCTT
P875608     960 GataGGAAGG GAAAACCGGT TCGGTTTAGG AGTCATGCCT CAGTGGGCTT
                *   ****** ********** ********** ********** **********
                *   ****** ********** ********** ********** **********
                *   ****** ********** ********** ********** **********
                *                                        * **********
                *                                        * **********

alignment
position        1201...... 1211...... 1221...... 1231...... 1241......

P875606     881 ---------- ---------- ---------- ---------- ----------
P875607    1042 AGGGGACTGT GGGAATGGGT AGCTTGGGAa CCCACCAAAG AAGCGGATAA
P875608    1010 AGGGGACTGT GGAAATGGGT AGCTTGGGAg CCCACCAAAG AACCGTATAA
                ********** ********** *********  ********** **********
                ********** ********** *********  ********** **********
                ********** ********** *********  ********** **********
                ********** ********** *********  ********** **********
                ********** ********** *********

alignment
position        1251...... 1261...... 1271...... 1281...... 1291......

P875606     881 ---------- ---------- ---------- ---------- ----------
P875607    1092 CGTCACCTTT CCCGTGTTGA TCTTTTTATC GTGTATCCAT CGTGGTCATA
```

```
P875608   1060 TGTCACCTTT CCCGTGTTGA TCTTTTTAGC GCGTATTCAT CTTGGTCATA
               ********** ********** ********** ********** **********
               ********** ********** ********** ********** **********
               ********** ********** ********** ********** **********
               ********** **********

alignment
position       1301...... 1311...... 1321...... 1331...... 1341......
P875606    881 ---------- ---------- ---------- ---------- ----------
P875607   1142 TCAattgccg tagcTCATTT TTCTTCTTCT AGGTGGTGTC CCcACTGATG
P875608   1110 TCAggtccca taatTCATTT TTCTTCTTCT AGGTGGTGTC CCtACTGATG
               ***            ****** ********** ********** ** *******
               ***            ****** ********** ********** ** *******
               ***            ****** ********** ********** ** *******
                              ****** ********** ********** ** *******
                                                             *******

alignment
position       1351...... 1361...... 1371...... 1381...... 1391......
P875606    881 --------GC CACCGGGATG GGGAGGGgtc cggcctccct tcaaacctgc
P875607   1192 AGGAGCAGGC TACTGGGCTG GAGAGGGAGA TCATGATAGC AGCACAGAAG
P875608   1160 AGGAGCAGGC TACCGGGCTG GAGAGGGAGA TCATGATAGC AGCACAGAGG
               ********** ********** ********** ********** **********
               ********** ********** ********** ********** **********
               ********** ********** ********** ********** **********
               ********** ********** ********** ***
               ********** ********** ********** ***
                       ** ********** *******

alignment
position       1401...... 1411...... 1421...... 1431...... 1441......
P875606    923 gcccacctca agcagagtgg gttctacatg cttttagaca aatgtcgaca
P875607   1242 GGACTGGTAA ---------- ---------- ---------- ----------
P875608   1210 GGACTGGTGA ---------- ---------- ---------- ----------
               **********
               **********
               **********

alignment
position       1451...... 1461...... 1471...... 1481...... 1491......
P875606    973 aatttgcctc ggtggttgga gaaagaaaag ctcataggcc gggcgcggtg
P875607   1252 ---------- ---------- ---------- ---------- ----------
P875608   1220 ---------- ---------- ---------- ---------- ----------

alignment
position       1501...... 1511...... 1521...... 1531...... 1541......
P875606   1023 gctcacaact gtaatcccag cactttggga ggccgaggcG GACAGATCCC
P875607   1252 ---------- ---------- ---------- ---------G GAGAAATGCT
P875608   1220 ---------- ---------- ---------- ---------G AAGct---CT
                                                        * *********
                                                        * ***     **
                                                        * ***     **
                                                                  **

alignment
position       1551...... 1561...... 1571...... 1581...... 1591......
P875606   1073 CTGAGGTCAG GAGCTCaaga ccagcctggc caacatggta aaaccccgtt
P875607   1263 GTGTCCTCTG GAGCCCTGGG GCTTGGATGG GATTA----- ----------
P875608   1228 GTGTCCTCTG GAGACCTTGG GCCTGGATGG GATCA----- ----------
               ********** ********** ********** *****
               ********** ********** ********** *****
               ********** ********** ********** *****
               ********** ********** ********** *****

alignment
position       1601...... 1611...... 1621...... 1631...... 1641......
P875606   1123 tctactaaaa atacaaaaat tagccgggcg tggtggcgcg cgcctgtagt
P875607   1298 ---------- ---------- ---------- ---------- ----------
```

```
P875608   1263 ---------- ---------- ---------- ---------- ----------

alignment
position       1651...... 1661...... 1671...... 1681...... 1691......
P875606   1173 cccagctact cgggaggctg aggcaggaga atcgcttaaa cccgggagGC
P875607   1298 ---------- ---------- ---------- ---------- --------GA
P875608   1263 ---------- ---------- ---------- ---------- --------GA
                                                                   **
                                                                   **
                                                                   **
                                                                   **

alignment
position       1701...... 1711...... 1721...... 1731...... 1741......
P875606   1223 GGAGGTTCCC GTGAGCCAAC ATCGCGCCAT TGCActccag cctgggcaac
P875607   1300 Gga-GTTCCT GTCAGCCAGC GTCTGGTCCT GA-------- ----------
P875608   1265 GGAGTCTCCT TTCAGCCAGC TTTTGGTCCT GACAact--- ----------
               ********** ********** ********** ****
               *     **** ********** ********** **
               *
               *

alignment
position       1751...... 1761...... 1771.....
P875606   1273 aagagcaaaa ctccgtctca aaaaagaaa
P875607   1331 ---------- ---------- ---------
P875608   1302 ---------- ---------- ---------
```

Pairwise similarities:

For each pairwise alignment, the similarity (relative to the maximum similarity) and the number of identical nucleic acids (in % of shorter sequence) is given. Maximum values are underlined.

	P875607 (1330 bp)	P875608 (1301 bp)
**P875606 (1301 bp)**	0.148<br>29 %	0.142<br>27 %
**P875607 (1330 bp)**		<u>1.000</u><br><u>83 %</u>

Please note that the **similarity value 1.000** marks only the two **most similar** sequences, it does not necessarily mean that these sequences are identical.

**Extract alignment region**	
**Positions in alignment**	from: to:
**Output file**	dialign.seq

Extract aligned sequences

For comments, questions or bug reports, please contact support@genomatix.de

© Genomatix Software GmbH 1998-2005 - All rights reserved. License Agreement GEMS Launcher 4.1.1

Genomatix-Logo Logout Personal Messages GEMS-Logo

Genomatix_MainGEMSElDoradoGene2PromoterBiblioSphere
Frequently_asked_questionsYour_ResultsYour_SequencesYour_ProtocolHelp

GEMS Launcher Task: *DiAlign TF*: Multiple alignment plus TF sites
working on promoters.seq (3 seq.)

[Alignment] [Pairwise_similarities]

DiAlign professional TF Release 3.1 December 2004 **Thu May 5 23:50:03 2005**

Solution parameters:

**Sequence file:**	promoters.seq (3 seq.)
**Type of sequences:**	nucleotide sequences
**Threshold T:**	0.00
**'*' signs below alignment denote:**	diagonal similarity (max. similarity is represented by 10 '*' signs)
**Alignment output:**	complete alignment is shown<br># nucleic acids per line: 50
**TF output:**	common TF matches located in aligned regions (common to 3 (100.0 %) sequences)
**Family matches:**	yes
**MatInspector library:**	Matrix Family Library Version 5.0 (February 2005)
**Selected groups** (core/matrix sim):	• ALL user_defined.lib (0.75/Optimized)<br>• ALL vertebrates.lib (0.75/Optimized)

Aligned Sequences:

No.	Sequence Name	Sequence Description	Length
1	P879022	sym=COX6A2|loc=Loc1339|taxid=9606|spec=Homo sapiens|chr=16| ctg=NT_010393|str=(-)|start=22752500|end=22753300|len=801| tss=501| comm=cytochrome c oxidase subunit VIa polypeptide 2; (NM_005205/501/bronze;)	801 bp
2	P879023	sym=Cox6a2|loc=Loc12862|taxid=10090|spec=Mus musculus|chr=7| ctg=NT_039433|str=(-)|start=46288613|end=46289449|len=837| tss=501,537| comm=cytochrome c oxidase, subunit VI a, polypeptide 2; (AK002506/556/gold;NM_009943/520/bronze;)	837 bp
3	P879024	sym=Cox6a2|loc=Loc25278|taxid=10116|spec=Rattus norvegicus| chr=1|ctg=NW_047562|str=(-)|start=30145948|end=30146748| len=801|tss=501| comm=cytochrome c oxidase, subunit VIa, polypeptide 2; (NM_012812/584/bronze;)	801 bp

Alignment (DiAlign format):

Please note that only **upper-case** letters are considered to be **aligned**. For more information, please have a look at the user guide.

V$MYOD V$AP4R V$MEF2 V$AREB V$COMP V$ECAT V$PCAT V$MZF1 V$ETSF V$NKXH

```
alignment position 1 . . . . . . . . .   11 . . . . . . . .   21 . . . . . . . .   31 . . . . . . . .   41 . . . . . . . .
P879022          1 t g c c a a a a t c   c a g g t t g a c a   c a t t c c t a c c   t t a c a t a a a c   t t c a t a a a a a
P879023          1 g c c c a g g a t a   g c t t t g a c c t   t g g t a c g t a g   t c c a a g a t g g   c c t t g a a c t c
P879024          1 - - - - - - - - - -   - - - - - - - - - -   - - - - - - - - - -   - - - - - - - - - -   - - - - - - - - - -
```

```
alignment  position  5 1 . . . . . . . .  6 1 . . . . . . . .  7 1 . . . . . . . .  8 1 . . . . . . . .  9 1 . . . . . . . .
P879022        51  t g a t g t a a g g  g c c g t g t a g t  g g c t c a t g c c  t g t a a t c c c a  g c a c t g t g g g
P879023        51  c t g a c c c t t c  t g c c t c c a t g  t c c c a a g t g c  t g g c t g g c a t  t a t a a a t a a g
P879024         1  - - - - - - - - - -  - - - - - - - - - -  - - - - - - - - - -  - - - - - - - - - -  - - - - - - - - - -

alignment  position  1 0 1 . . . . . . .  1 1 1 . . . . . . .  1 2 1 . . . . . . .  1 3 1 . . . . . . .  1 4 1 . . . . . . .
P879022       101  a g g c t g a g g c  g g g a g g a t c a  c g t g a g c t c a  a g a g t t c c a c  a c c a g c c t g g
P879023       101  t g c a C C - - - -  - - - - - - - - - -  - - - - - - - - - -  - - - - - - - - - -  - - - - - - - - - -
P879024         1  - - - - C C - - - -  - - - - - - - - - -  - - - - - - - - - -  - - - - - - - - - -  - - - - - - - - - -
                           * *
                           * *
                           * *
                           * *

alignment  position  1 5 1 . . . . . . .  1 6 1 . . . . . . .  1 7 1 . . . . . . .  1 8 1 . . . . . . .  1 9 1 . . . . . . .
P879022       151  a c a a t a t a g t  g a g a t c c c c g  t c t c c a g t a t  t t a a a a a a t c  t t t t a a g g t a
P879023       107  - - - - - - - - - -  - - - - - - - - - -  - - - - - - - - - -  - - - - - - - - - -  - - - - - - - - - -
P879024         3  - - - - - - - - - -  - - - - - - - - - -  - - - - - - - - - -  - - - - - - - - - -  - - - - - - - - - -

alignment  position  2 0 1 . . . . . . .  2 1 1 . . . . . . .  2 2 1 . . . . . . .  2 3 1 . . . . . . .  2 4 1 . . . . . . .
P879022       201  c g a a t t a t t a  t a c a t t t t a c  a g c t g a a a A C  A C T G A G A c t c  a a a a a - - - - -
P879023       107  - - - - - - - - - -  - - - - - - - - - -  - - - - - - - - A C  A C T G A G A T T G  T G T A G G A A T A
P879024         3  - - - - - - - - - -  - - - - - - - - - -  - - - - - - - - A C  A C T G G G C T T G  T G T A G C A G T A
                                                                     * *  * * * * * * * * * *  * * * * * * * * * *
                                                                     * *  * * * * * * * * * *  * * * * * * * * * *
                                                                     * *  * * * * * * * * * *  * * * * * * * * * *
                                                                     * *  * * * * * * * * * *  * * * * * * * * * *

alignment  position  2 5 1 . . . . . . .  2 6 1 . . . . . . .  2 7 1 . . . . . . .  2 8 1 . . . . . . .  2 9 1 . . . . . . .
P879022       246  - - - - - - - - - -  - - - - - - - - - -  - - - - - - - - - -  - - - - - - - - - -  - - - - - - - - - -
P879023       129  G G G A A A G A A C  T C A G G a C A T T  A T G G G T A C T T  G G T A G G C A G A  C A C T G C A G G C
P879024        25  G G G A A A G A A C  T C A G G g C A T T  A T G G G T A C T A  A G T A G G C A G A  C A C T C C A G G C
                   * * * * * * * * * *  * * * * *   * * * *  * * * * * * * * * *  * * * * * * * * * *  * * * * * * * * * *
                   * * * * * * * * * *  * * * * *   * * * *  * * * * * * * * * *  * * * * * * * * * *  * * * * * * * * * *
                   * * * * * * * * * *  * * * * *   * * * *  * * * * * * * * * *  * * * * * * * * * *  * * * * * * * * * *
                   * * * * * * * * * *  * * * * *

alignment  position  3 0 1 . . . . . . .  3 1 1 . . . . . . .  3 2 1 . . . . . . .  3 3 1 . . . . . . .  3 4 1 . . . . . . .
P879022       246  - - - - - - - - - -  - - - - - - - - - -  - - - - - - - - - -  G A C A C T T A A T  T C A T T C T A C A
P879023       179  A g t c T A C C A A  C T G A G C T A C A  C T C C C A C C C C  C A C C C C A C C C  C a c t g t - - A A
P879024        75  A t t t T A C C A A  C C G A A C T A C A  C T C C C A C C C C  G A C C C C C A C C  C C A T T T T A A A
                   *       * * * * * *  * * * * * * * * * *  * * * * * * * * * *  * * * * * * * * * *  * * * * * * * * * *
                   *       * * * * * *  * * * * * * * * * *  * * * * * * * * * *  * * * * * * * * * *  *               * *
                   *       * * * * * *  * * * * * * * * * *  * * * * * * * * * *  * * * * * * * * * *  *               * *
                                                                                                                        * *

alignment  position  3 5 1 . . . . . . .  3 6 1 . . . . . . .  3 7 1 . . . . . . .  3 8 1 . . . . . . .  3 9 1 . . . . . . .
P879022       266  A A T A T T T A T T  A A A G C C C A a c  t g t g t g c c a g  c c a c c a g g c a  t a g a a t a g g g
P879023       227  A A T T T G T T T T  A A A G A C A A G A  T C T T A G G T A G  T T C A A G C T G T  G A T C A A A C T C
P879024       125  A T T T T G T T T T  A A A G A T G G G A  T C T T A G G T T G  T T C A A G C T G C  G C T C A A C T T C
                   * * * * * * * * * *  * * * * * * * * * *  * * * * * * * * * *  * * * * * * * * * *  * * * * * * * * * *
                   * * * * * * * * * *  * * * * * * * * * *  * * * * * * * * * *  * * * * * * * *
                   * * * * * * * * * *  * * * * * * * * * *  * * * * * * * * * *  * * * * * * * *
                   * * * * * * * * * *  * * * *

alignment  position  4 0 1 . . . . . . .  4 1 1 . . . . . . .  4 2 1 . . . . . . .  4 3 1 . . . . . . .  4 4 1 . . . . . . .
P879022       316  a a c a a a a t a g  t t t c c c t g c a  a c t a t a t a a a  a a g a a t g g c a  t a t t g t a c - -
P879023       277  A T T A A G T T G A  T A A C A G T G G C  C T T G A A C T C T  G A T C C T C C A G  C T T A C A T A T T
P879024       175  A C T A A C T T G A  C C A C A G T G G C  C T T G A A C T C T  G A T C T T C C T G  C C T A C C T G T T
                   * * * * * * * * * *  * * * * * * * * * *  * * * * * * * * * *  * * * * * * * * * *  * * * * * * * * * *
                                              * * * *  * * * * * * * * * *  * * * * * * * * * *  * * * * * * * * * *
                                              * * * *  * * * * * * * * * *  * * * * * * * * * *  * * * * * * * * * *

alignment  position  4 5 1 . . . . . . .  4 6 1 . . . . . . .  4 7 1 . . . . . . .  4 8 1 . . . . . . .  4 9 1 . . . . . . .
P879022       364  - - - - - - - - - -  - - - - - - - - - -  - - - - - - - - - -  - - - - - - - - - -  - - - - - - - - - -
P879023       327  T C A A G T t t g t  a - - - - - - - - -  - - - - - - - - - -  - - - - - - - - - -  - - - - - - - T C A
```

```
P879024      225 TCAAGTgccg ggccgacagg cttgtatggc caagccaggg gcgtattTCA
                 ******                                           ***
                 ******
                 ******

alignment position 501....... 511....... 521....... 531....... 541.......
P879022      364 --TTAAATGC CTCCTTGCCA AAATAAGAGG CACAGGACAA CAGCTGTCTG
P879023      341 CTTTAAATCT TCtCTTGCCA AAATAAAAGG CACATTGCAT CAGCTGCCTG
P879024      275 CTTTAAATGT TCCCTTGCCA AAATAAAAGG CACATTGCAT CAGCTGCCAG
                 ********** ********** ********** ********** **********
                   ******** ********** ********** ********** **********
                   ******** ********** ********** ********** **********
                               ******* ********** ********** **********
                               ******* ********** ********** ******** *
                               ******* ********** ********** ******** *
                               ******* ********** ********** ******** *
                               ******* ********** ****   *** ******** *
                               ******* ********** ****

alignment position 551....... 561....... 571....... 581....... 591.......
P879022      412 GAGATGACCT CAAGCCAGTC AGGCCCCATT TTAAATATAG AAA-CCCCTA
P879023      391 AAGAGGATCT CCTGCCAGTC AAGACCCACT TTAGATAGAG AAAGCCCCTA
P879024      325 AAGAGGATCT CCTTCCAGTC AAGACCCACT TTAGtcAGAG AAAGTCCCTA
                 ********** ********** ********** ********** **********
                 ********** ********** ********** ********** **********
                 ********** ********** ********** ****  **** **********
                 ********** ********** ********** ****  **** **********
                 ********** ********** ********** ****  **** ***  *****
                 ***  ***** ********** ********** ****  **** ***
                 ***  ***** ********** *******
                 ***

alignment position 601....... 611....... 621....... 631....... 641.......
P879022      461 AGAATAGCCg ccagtgctcc agaCTCAACA GGTGATTGGC CCAGAGAGGG
P879023      441 AAAATAGCCA TCCATGTGCg GG-CTCAACA GGTGATTGGC TCTGAGAGGG
P879024      375 AAAATAGCCA TCCATGTGCc GG-CTCAACA GGTGATTGGC TGCGAGTGGG
                 ********** *********  ** ******* ********** **********
                 ********** *********  ** ******* ********** **********
                 ********** *********  ** ******* ********** **********
                 ********** *********  ** ******* ********** **********
                 *********                ******* ********** **********
                                          ******* ********** **********
                                          ******* ********** **********
                                          ******* ********** **********
                                          ******* ********** **********

alignment position 651....... 661....... 671....... 681....... 691.......
P879022      511 GAGGTGACCC Caggccccag gaaagggagc g-----AGGA CAGCGCTGGT
P879023      490 GAGGAGAGCC tctcGACTGG GTGAAGGAGA CAGAGAAGGA CAGTGCCATT
P879024      424 GAGGAGATCC CctaGACTGA GTGAAGGAGA CAGAGAAGGA CAGTGCCATT
                 ********** *   ****** ********** ********** **********
                 ********** *   ****** ********** ********** **********
                 ********** *   ****** ********** ********** **********
                 **********     ****** ********** ********** **********
                 **********     ****** ********** ********** **********
                 **********
                 **********
                 **********
                 **********

alignment position 701....... 711....... 721....... 731....... 741.......
P879022      556 TCCCGGCTCC CCGCACCAtc ATGGCTTTGC CTCTGAGGCC CCTGACCCGG
P879023      540 CCTAGCCTCC CTTTGACA-- ATGGCTCTGC CTCTAAAGGT CCTGAGCCGG
P879024      474 CCTAGCCTCC CTTCAACA-- ATGGCTCTGC CTCTTAAGGT CCTCAGTCGG
                 ********** ********   ********** ********** **********
                 ********** ********   ********** ********** **********
                 ********** ********   ********** ********** **********
                 ********** ********   ********** ********** **********
                 ****                  ********** ********** ******  ***
                                       ********** ********** ******  ***
```

```
                                        ********** ********** ******     ***
                                                                          ***
alignment position 751....... 761....... 771....... 781....... 791.......
P879022        606 GGCTTGGCCA gcgctGCCAA AGGAGGCCAC GGAGGAGCAG GAGGTGAGTG
P879023        588 AGCATGGCCA GCGCAGCCAA AGGAGACCAT GGAGGGGCAG GAGGTAAGTG
P879024        522 AGCATGGCCA GCGCATCCAA AGGAGACCAC GGAGGCGCAG GAGGTAAGTG
                   ********** ********** ********** ********** **********
                   ********** ********** ********** ********** **********
                   ********** ********** ********** ********** **********
                   ********** ********** ********** ********** **********
                   **********      ***** ********** ********** **********
                   **********      ***** ********** ********** **********
                   **********      ***** ********** ********** **********
                   **********       **** ********** *****  *** **********
                                    **** ********** *****  *** **********
                                    **** ********** *****  *** **********
alignment position 801....... 811....... 821....... 831....... 841.......
P879022        656 GGGaacgggc ggatccggg- ---------- ---------- ----------
P879023        638 GGGCCAGGCC TGActctGAT TTAGTAGCCT AGCCGATCTG TTCTCTTTGC
P879024        572 GGGCCAGGCC TGAgggaGAT TTAGTAGCCT AGCCCATTTG TTTTCTTGGC
                   ********** ***    *** ********** ********** **********
                   ********** ***    *** ********** ********** **********
                   ********** ***    *** ********** ********** **********
                   ********** ***
                   ***
                   ***
                   ***
                   ***
                   ***
                   ***
alignment position 851....... 861....... 871....... 881....... 891.......
P879022        675 ---------- ---------- -------GGC TCCCCTACCC TGccc-----
P879023        688 CCg-GCTTCC CCCTCCTTCT GTCTCCTGGC TCCTCTCTCC TGATCTACCA
P879024        622 CCctGCCTTC CCCTCCCTCC CTTTCCTGGC TCCCCTCTCC TGATCTACCA
                   **  ****** ********** ********** ********** **********
                   **                           *** *** ****** **********
                   **                                   ****** **********
                                                        ****** **********
                                                        ****** **********
alignment position 901....... 911....... 921....... 931....... 941.......
P879022        693 ---------- ---------- ---------- ---------- ---------A
P879023        737 GGCACTGATC TTTCAGACCT CTAGGCCCAG GGATAGTTTT TGCCTATGAA
P879024        672 GGCACCGATC TTTCAGACCT CTAGGCCCAG TGATAACTTT TACCTGTGAA
                   ********** ********** ********** ********** **********
                   ********** ********** ********** ********** **********
                   ********** ********** ****
                   ********** ********** ****
                   ********** ********** ****
alignment position 951....... 961....... 971....... 981....... 991.......
P879022        694 CCTGTTCACA GGCCCGCCgc cccaggaccg ccgcgctcac cccgctccgt
P879023        787 TCTACTCACA C-CCCACCTT TGCTCCTCCA GCCAAC---- ----------
P879024        722 TCTGCTCTCA CTCCCACCTT TGCTCTCCCA GCCAAC---- ----------
                   ********** ********** ********** ******
                   ********** * ******** ********** ******
                                ******** ********** ******
                                ******** ********** ******
alignment position 1001...... 1011...... 1021...... 1031...... 1041......
P879022        744 ccgcagctcg tACCTGGCGT CTGCTGACCT TCGTGCTGGC GCTGCCCAGC
P879023        822 ---------- -ACCTGGCGC CTCCTGA--- ---------- ----------
P879024        758 ---------- -ACTTGGCGC CTCCTGACCT TTGTGCTGGC TCTCCCCAGC
                               ********* ********** ********** **********
                               ********* ********** ********** **********
                               ********* ********** ********** **********
                               ********* *******
```

```
                                  ****** *******
                                  ****** *******
                                  ****** *******
alignment position 1051. . . .
P879022      794 GTg gccc t
P879023      838 - - - ------ -
P879024      797 GTa gc-- -
                 **
                 **
                 **
```

Pairwise similarities:

For each pairwise alignment, the similarity (relative to the maximum similarity) and the number of identical nucleic acids (in % of shorter sequence) is given. Maximum values are underlined.

	P879023 (837 bp)	P879024 (801 bp)
**P879022 (801 bp)**	0.214 30 %	0.229 34 %
**P879023 (837 bp)**		1.000 79 %

Please note that the **similarity value 1.000** marks only the two **most similar** sequences, it does not necessarily mean that these sequences are identical.

**Extract alignment region**	
**Positions in alignment**	from: to:
**Output file**	dialign.seq

Extract aligned sequences

For comments, questions or bug reports, please contact support@genomatix.de

© Genomatix Software GmbH 1998-2005 - All rights reserved. License Agreement GEMS Launcher 4.1.1

Genomatix-Logo Logout Personal Messages GEMS-Logo

Genomatix_MainGEMSElDoradoGene2PromoterBiblioSphere
Frequently_asked_questionsYour_ResultsYour_SequencesYour_ProtocolHelp

GEMS Launcher Task: *DiAlign TF*: Multiple alignment plus TF sites
working on promoters.seq (3 seq.)

[Alignment] [Pairwise_similarities]

DiAlign professional TF Release 3.1 December 2004 **Fri May 6 22:06:49 2005**

Solution parameters:

**Sequence file:**	promoters.seq (3 seq.)
**Type of sequences:**	nucleotide sequences
**Threshold T:**	0.00
**'*' signs below alignment denote:**	diagonal similarity (max. similarity is represented by 10 '*' signs)
**Alignment output:**	complete alignment is shown<br># nucleic acids per line: 50
**TF output:**	common TF matches located in aligned regions (common to 3 (100.0 %) sequences)
**Family matches:**	yes
**MatInspector library:**	Matrix Family Library Version 5.0 (February 2005)
**Selected groups** (core/matrix sim):	• ALL user_defined.lib (0.75/Optimized)<br>• ALL vertebrates.lib (0.75/Optimized)

Aligned Sequences:

No.	Sequence Name	Sequence Description	Length
1	P911188	sym=COX6C|loc=Loc1345|taxid=9606|spec=Homo sapiens|chr=8| ctg=NT_008046|str=(-)|start=14123568|end=14126068|len=2501| tss=2001| comm=cytochrome c oxidase subunit VIc; (NM_004374/508/bronze;)	2501 bp
2	P911190	sym=Cox6c|loc=Loc12864|taxid=10090|spec=Mus musculus|chr=15| ctg=NT_039618|str=(-)|start=27052693|end=27055194|len=2502| tss=2001,2002| comm=cytochrome c oxidase, subunit VIc; (AK013459/502/gold;NM_053071/501/bronze;)	2502 bp
3	P911192	sym=Cox6c|loc=Loc54322|taxid=10116|spec=Rattus norvegicus| chr=7|ctg=NW_047778|str=(-)|start=3775762|end=3778262| len=2501|tss=2001| comm=cytochrome oxidase subunit VIc; (NM_019360/501/bronze;)	2501 bp

Alignment (DiAlign format):

Please note that only **upper-case** letters are considered to be **aligned**. For more information, please have a look at the user guide.

V$TBPF V$LHXF V$NRF1 U$NRF1 V$IRFF V$ETSF V$EREF U$EREF V$RORA V$SF1F

```
alignment position 1 . . . . . . . . .  1 1 . . . . . . . .  2 1 . . . . . . . .  3 1 . . . . . . . .  4 1 . . . . . . . .
P911188          1 a a a t c a c a c t  a t g c c c g g c t  a a g t t t t a a a  g t t t t t t a t g  - - - - - - - - - -
P911190          1 g a a c t c a a a t  t a a c t a a c a g  g c c a g t a c c t  a g c a t c a g t t  t c c a g a t t a c
P911192          1 c a t c c a - - - -  - - - - - - - - - -  - - - - - - - - - -  - - - - - - - - - -  - - - - - - - - - -

alignment position 5 1 . . . . . . . .  6 1 . . . . . . . .  7 1 . . . . . . . .  8 1 . . . . . . . .  9 1 . . . . . . . .
```

```
P911188       41 ---------- ---------- ---------- ---------- ----------
P911190       51 actcaacaac aagatcttcc actaacacca gtttccaggt tcctgcccct
P911192        7 ---------- ---------- ---------- ---------- ----------

alignment position 101....... 111....... 121....... 131....... 141.......
P911188       41 ---------- ---------- ---------- ---------- ----------
P911190      101 gacacttcac ttcctaacaa ccactgatcc ggaggaaagc agaagttacg
P911192        7 ---------- ---------- ---------- ---------- ----------

alignment position 151....... 161....... 171....... 181....... 191.......
P911188       41 ---------- ---------- ---------- ---------- ----------
P911190      151 tttgtggttt ggctctgaac aattatgtta aaggccataa tggcccccta
P911192        7 ---------- ---------- ---------- ---------- ----------

alignment position 201....... 211....... 221....... 231....... 241.......
P911188       41 ---------- ---------- ---------- ---------- ----------
P911190      201 gccaggtgtg taccaagcta gataaacccc cccccttttg cctctataaa
P911192        7 ---------- ---------- ---------- ---------- ----------

alignment position 251....... 261....... 271....... 281....... 291.......
P911188       41 ---------- ---------- ---------- ---------- --------GA
P911190      251 tgcttgcctg aaactgggct ttgggccccc tcccattctc ctgcatcaGA
P911192        7 ---------- ---------- ---------- ---------- ----------
                                                                   **
alignment position 301....... 311....... 321....... 331....... 341.......
P911188       43 GATGGTGGTC TATGTTGCTC AGGTTggtct ccaacacctg ggctcaagta
P911190      301 GAAGGTGGTA TGTATAGCCC AAGCTcaagc ttgaataaag ggaccttaat
P911192        7 ---------- ---------- ---------- ---------- ----------
                 ********** ********** *****
alignment position 351....... 361....... 371....... 381....... 391.......
P911188       93 atctgccttc cttggcctcc caaagtgct- ---------- ----------
P911190      351 gtaattgcat cggaatcagc tccttggtgg tcttttgggg gttcatgaat
P911192        7 ---------- ---------- ---------- ---------- ----------

alignment position 401....... 411....... 421....... 431....... 441.......
P911188      122 ---------- ---------- ---------- ------GGGA TTACAGGTGT
P911190      401 gattcctggc acagcagaac cttaccttca ttgttaGGGA CTAAAGGTGT
P911192        7 ---------- ---------- ---------- ---------- ----------
                                                       **** **********
alignment position 451....... 461....... 471....... 481....... 491.......
P911188      136 gagccaccac acccagt--- ---------- ---------- ----------
P911190      451 tcactaaggg cgagtctgtt tttttccagc caaatcatac tgtaatctag
P911192        7 ---------- ---------- ---------- ---------- ----------

alignment position 501....... 511....... 521....... 531....... 541.......
P911188      153 ---------- ---------- ---------- ---------- ------CTGG
P911190      501 ggtgtctaca tttggctgga tcatatacgt ctcagtctcc ttgttgCTGG
P911192        7 ---------- ---------- ---------- ---------- ----------
                                                                 ****
alignment position 551....... 561....... 571....... 581....... 591.......
P911188      157 AATAAGATTt gactgttgta gaatcacatg gctagaagtg atgaaggatt
P911190      551 ATTAAAATTc ccctacat-- ---------- ---------- ----------
P911192        7 ---------- ---------- ---------- ---------- ----------
                 *********
alignment position 601....... 611....... 621....... 631....... 641.......
```

```
P911188              207 gattaagcac tgctactaaa agaaagattg aaggaatgca ggacaggcaa
P911190              569 ---------- ---------- ---------- ---------- ----------
P911192                7 ---------- ---------- ---------- ---------- ----------

alignment position       651....... 661....... 671....... 681....... 691.......
P911188              257 aagaacacag ggaggaatgc atattcagaa tgtctgtttg tcaagcactg
P911190              569 ---------- ---------- ---------- ---------- ----------
P911192                7 ---------- ---------- ---------- ---------- ----------

alignment position       701....... 711....... 721....... 731....... 741.......
P911188              307 tataggttga acacccttaa tctgaaaatc caaaatccaa aacgctccaa
P911190              569 ---------- ---------- ---------- ---------- ----------
P911192                7 ---------- ---------- ---------- ---------- ----------

alignment position       751....... 761....... 771....... 781....... 791.......
P911188              357 aatccaaaac ttttgagcac tgatgtgatg gctcaaggaa atgctcagtg
P911190              569 ---------- ---------- ---------- ---------- ----------
P911192                7 ---------- ---------- ---------- ---------- ----------

alignment position       801....... 811....... 821....... 831....... 841.......
P911188              407 gagcattttg gatttcagat tttcagattg ggatgctcaa ctggcatgta
P911190              569 ---------- ---------- ---------- ---------- ----------
P911192                7 ---------- ---------- ---------- ---------- ----------

alignment position       851....... 861....... 871....... 881....... 891.......
P911188              457 ttctgcaaat actcaaaaat ctgaaaaaat ccaaaatctg aaacacttct
P911190              569 ---------- ---------- ---------- ---------- ----------
P911192                7 ---------- ---------- ---------- ---------- ----------

alignment position       901....... 911....... 921....... 931....... 941.......
P911188              507 ggtctcaaat atttcagata agggatactc aagctgcata tacattatcc
P911190              569 ---------- ---------- ---------- ---------- ----------
P911192                7 ---------- ---------- ---------- ---------- ----------

alignment position       951....... 961....... 971....... 981....... 991.......
P911188              557 catttaattt acatcacaac ccaacaaaat gggtactatt tttagaggag
P911190              569 ---------- ---------- ---------- ---------- ----------
P911192                7 ---------- ---------- ---------- ---------- ----------

alignment position       1001...... 1011...... 1021...... 1031...... 1041......
P911188              607 aaagcagact tagaaagatg aagaaatttg ccgctacaag ttgatgagct
P911190              569 ---------- ---------- ---------- ---------- ----------
P911192                7 ---------- ---------- ---------- ---------- ----------

alignment position       1051...... 1061...... 1071...... 1081...... 1091......
P911188              657 gggactcaaa tccaggtctg ttcggctcta gaacagaacc tatatgtaac
P911190              569 ---------- ---------- ---------- ---------- ----------
P911192                7 ---------- ---------- ---------- ---------- ----------

alignment position       1101...... 1111...... 1121...... 1131...... 1141......
P911188              707 aTGTCTCTCT TCATCTATCG CTCAGgtttt attcacgtgt caccctcagt
P911190              569 -TGTATCAGT TCAAAGAACG CTCAGactgt tcactgtcat ataaaatctg
P911192                7 ---------- ---------- ---------- ---------- ----------
                          ********* ********** *****
alignment position       1151...... 1161...... 1171...... 1181...... 1191......
P911188              757 cattcactat ggttcact-- ---------- ---------- ----------
```

```
P911190      618 cttgaaggct aaagttgtat tctaagttta aattactggg cttaagagat
P911192        7 ---------- ---------- ---------- ---------- ----------

alignment position 1201...... 1211...... 1221...... 1231...... 1241......
P911188      775 ---------- ---------- ---------- ---------- ----------
P911190      668 ctatcaatca aacatgcctc taacctgtaa gcctcacttg cccaagggca
P911192        7 ---------- ---------- ---------- ---------- ----------

alignment position 1251...... 1261...... 1271...... 1281...... 1291......
P911188      775 ---------- ---------- ---------- ---------- ----------
P911190      718 gataatctct ggagtgctgg gggctgttac ttatgtaaga tagtgaacta
P911192        7 ---------- ---------- ---------- ---------- ----------

alignment position 1301...... 1311...... 1321...... 1331...... 1341......
P911188      775 ---------- ---------- ---------- ---------- ----------
P911190      768 tggtgttttc tcttctgtaa acaacctcac ttgccccaac tttacaggat
P911192        7 ---------- ---------- ---------- ---------- ----------

alignment position 1351...... 1361...... 1371...... 1381...... 1391......
P911188      775 ---------- ---------- ---------- ---------- ----------
P911190      818 aagcttgatc acacacaggt ggaggtaggc tggaggttca taaggaaata
P911192        7 ---------- ---------- ---------- ---------- ----------

alignment position 1401...... 1411...... 1421...... 1431...... 1441......
P911188      775 ---------- ---------- ---------- ---------- ----------
P911190      868 cattgggatc taagcgtgcc cctgattgga cgaaggtggg aagtactgac
P911192        7 ---------- ---------- ---------- ---------- ----------

alignment position 1451...... 1461...... 1471...... 1481...... 1491......
P911188      775 ---------- ---------- ---------- ---------- ----------
P911190      918 cctttggggg ttgcctttat aagcctctga ctgagttgta atgtggcgat
P911192        7 ---------- ---------- ---------- ---------- ----------

alignment position 1501...... 1511...... 1521...... 1531...... 1541......
P911188      775 --------GG ATAGCTAGGG AAGCACAGAA CAaaca---- ----------
P911190      968 gtattttgGG ATTCCTAGGT ATGCACCTGA CAgtgtccac tgaactggcc
P911192        7 ---------- ---------- ---------- ---------- ----------
                         ** ********** ********** **
alignment position 1551...... 1561...... 1571...... 1581...... 1591......
P911188      803 ---------- ---------- ---------- ---------- ----------
P911190     1018 aatgcttaat aaagcttgtt ttaaatttgg ttcaaaactt acagtagccg
P911192        7 ---------- ---------- ---------- ---------- ----------

alignment position 1601...... 1611...... 1621...... 1631...... 1641......
P911188      803 ---------- ---------- ---------- ---------- ----------
P911190     1068 ttttcactat tctcaccggg tgggattaac cgTAGCCCTG GTACTCTGAT
P911192        7 ---------- ---------- ---------- --TAGCCCTG GTACTCTGAT
                                                   ******** **********
                                                   ******** **********
                                                   ******** **********
alignment position 1651...... 1661...... 1671...... 1681...... 1691......
P911188      803 ---------- ---------- ---------- --------CT CAGTATTTCA
P911190     1118 CCCg-TGAAC CTTTCTTTGT AACCATGGGt tcagttcag- ----------
P911192       25 CCCcaTGAAC CTTTCTTTCT AATCATAGGg tggtccagCT CAGTAGCTCA
                 ***  ***** ********** *********            ** **********
                 ***  ***** ********** *********
                 ***
```

```
alignment position 1701......    1711......    1721......    1731......    1741......
P911188        815 CTCAGgtcag    gctctgtgcc    ctgagtgtgc    ctgtctttgc    catgttctcc
P911190       1156 ----------    ----------    ----------    ----------    ----------
P911192         75 CTCAG-----    ----------    ----------    ----------    ----------
                   *****

alignment position 1751......    1761......    1771......    1781......    1791......
P911188        865 cagccaatgt    tttctctcca    atttctccag    ttttccaggc    tttccattag
P911190       1156 ----------    ----------    ----------    ----------    ----------
P911192         80 ----------    ----------    ----------    ----------    ----------

alignment position 1801......    1811......    1821......    1831......    1841......
P911188        915 actgaaactc    agctctgctt    ccatctcttc    cacgtattgt    ctgaagcttt
P911190       1156 ----------    ----------    ----------    ----------    ----------
P911192         80 ----------    ----------    ----------    ----------    ----------

alignment position 1851......    1861......    1871......    1881......    1891......
P911188        965 ccctgaTGGT    TTCACAtaca    ctaatccctg    tgaactctta    agttcaccac
P911190       1156 ------TGGT    TTCACAcctt    cagtta----    ----------    ----------
P911192         80 ----------    ----------    ----------    ----------    ----------
                         ****    ******

alignment position 1901......    1911......    1921......    1931......    1941......
P911188       1015 ttcttctctg    ggggtgggga    tggaaagaga    atagaaacaa    attactctct
P911190       1176 ----------    ----------    ----------    ----------    ----------
P911192         80 ----------    ----------    ----------    ----------    ----------

alignment position 1951......    1961......    1971......    1981......    1991......
P911188       1065 ggacaaattc    catgcaaaga    ttttcagctg    ggtagaccct    tgataagagg
P911190       1176 ----------    ----------    ----------    ----------    ----------
P911192         80 ----------    ----------    ----------    ----------    ----------

alignment position 2001......    2011......    2021......    2031......    2041......
P911188       1115 cctgaaaaac    gttggtaaag    tccaaaccta    cttttgaacc    tcaattgagc
P911190       1176 ----------    ----------    ----------    ----------    ----------
P911192         80 ----------    ----------    ----------    ----------    ----------

alignment position 2051......    2061......    2071......    2081......    2091......
P911188       1165 accttttctt    gtggccaggt    TTCACCCTCC    AGAGTGTTGG    TTAATAGGTt
P911190       1176 ----------    ----------    TTCTCCATAT    AGTGTGATGG    TGCATGTGTc
P911192         80 ----------    ----------    ----------    ----------    ----------
                                               **********    **********    ******** **

alignment position 2101......    2111......    2121......    2131......    2141......
P911188       1215 gaggacaaag    actccctgat    cagaatgaga    ctccccgaag    atctgtcctc
P911190       1206 actgctcaag    cagggttcct    aagccctctc    aaaccctgca    tatatataaa
P911192         80 ----------    ----------    ----------    ----------    ----------

alignment position 2151......    2161......    2171......    2181......    2191......
P911188       1265 ttgtttagca    gaatctggca    catagtaggt    actcagtgta    tatatcgact
P911190       1256 gta-------    ----------    ----------    ----------    ----------
P911192         80 ----------    ----------    ----------    ----------    ----------

alignment position 2201......    2211......    2221......    2231......    2241......
P911188       1315 caatatcatg    catctgttgt    gtcccagcta    ccttccacag    GGCTTGATGG
P911190       1259 ----------    ----------    ----------    ----------    GGCTTACTCA
P911192         80 ----------    ----------    ----------    ----------    ----------
                                                                            ******* ***

alignment position 2251......    2261......    2271......    2281......    2291......
```

```
P911188         1365 GTTGACTTAA ATAATGCTTT TAAAattcag gttatttcaa tacaaacagc
P911190         1269 GCTGACTTAA GTAACACCTG TGAATCTAGT TGATGTCAct gAAGTAAGAT
P911192           80 --TGATTTAA GTAGCCCCTG TGAATCTAGT TGATGTCAtt aAAGTAAGAC
                     ********** ********** ********** ********    *********
                       ******** ********** ********** ********    *********
                       ******** ********** ********** ********
                       ******** ********** ********** ********

alignment position   2301...... 2311...... 2321...... 2331...... 2341......
P911188         1415 ctggcatcat taaggcacaa gagcctcacc cttctacacc aCTTTCCACG
P911190         1319 AAACGTCCCA AGGCAcgaaa agtcaTCTTT AGTCGG---- -CTTTCCAAA
P911192          128 AAGCGTCCCA AGGCAaaagt catttTCTTT AGTCAG---- -CTTACCCAA
                     ********** *****           ***** ******      *********
                     ********** *****           ***** ******      *********
                                                ***** ******      *********
                                                ***** ******      *********
                                                ***** ******      *********

alignment position   2351...... 2361...... 2371...... 2381...... 2391......
P911188         1465 AACTTCCagt tgccttcc-T TCAAAATCCT TGTTTTCTTA AGAAACCCAC
P911190         1364 AACATCCCTG TTGTTCCCgT TCACACGTTA TTTTTTATTA AGAACCCGAC
P911192          173 AACATCCCTG TTGTTCCCaT TCAGAAGTTA TCTCTTATTA AGAACCCAGC
                     ********** ********  * ********** ********** **********
                     ********** ********  * ********** ********** **********
                     ********** ********  * ********** ********** **********
                     ********** ********  * ********** ********** **********
                     ********** ********                 ******** **********

alignment position   2401...... 2411...... 2421...... 2431...... 2441......
P911188         1514 AAAAATACCg ccccctctatc aaaacgTCCT CAGCTTTGCC AGGTAGAGAA
P911190         1414 ACAAATACCc attacga--- ---------- ----TTTGTT AAGCAGATCA
P911192          223 ACAAACACCt aataggatt- ------TGCT CATGTTTGTT AAGCAGATTA
                     *********                   **** ********** **********
                     *********                          ****** **********
                     *********
                     *********
                     *********

alignment position   2451...... 2461...... 2471...... 2481...... 2491......
P911188         1564 TTGCTTCTGC AGTAGct--- --GGGCCACG TCTCTgtggt agtgcCCACT
P911190         1447 TTGAGTc--T GGCAGTGGAG TGGGATCACA TCTCTAGTTT GTT--CCACA
P911192          266 TTGGGTTTGT GGCAGTGGAG TGGGGTCACA TCTCTAGTTT GTT--CCACg
                     ********** ********** ********** ********** ***  *****
                     ******   * ********** ********** ********** ***  ****
                              * ********** ********** ********** ***  ****
                              * ********** ********** ********** ***  ****
                              * ********** ********** ********** ***  ****
                              * ********** ********** ********** ***  ****

alignment position   2501...... 2511...... 2521...... 2531...... 2541......
P911188         1609 GCATGGGATC GGTATTAATC T--GTTTGTC TTTttcatct tctgggctgt
P911190         1493 GCATCGGTTC TAAAATTACA T--GTTAAAC TCTGAAATTG GCCtttctcg
P911192          314 GCATGTGTAC TAAAATTACA TtgGTTAAGC TCTAAAATTG GCCatttccc
                     ********** ********** *  ******* ********** ***

alignment position   2551...... 2561...... 2571...... 2581...... 2591......
P911188         1657 gagctcctag aagtcagctt tttCACTTTT ACCCAAGCCA AACGGTTATT
P911190         1541 ---------- ---------- ---CTCTTTG CCAAAAACCA AGCGTTTATT
P911192          364 tc-------- ---------- -------TTT ACCAAAACCA GGCATTTATT
                                               ******* ********** **********
                                                          ******* **********
                                                          ******* **********
                                                          ******* **********

alignment position   2601...... 2611...... 2621...... 2631...... 2641......
P911188         1707 CGCGTAAGGT TGGTtaTAAT TAAAATGtcg gtTTGTGCAC ATGGTTTGGG
P911190         1568 GTGCCGAGGT AGTTG-TAAT GAAAATGC-- --TTATTCAT GTGGTTTGGG
P911192          389 GTGCCAAGGT AGCTG-TAAT AAAAATGC-- --TTGTGCAT GTGGTTTGGG
```

```
                            ********** ***** **** ********   ******** **********
                            ********** ***** ****  *******   ******** **********
                            ********** ***** ****  *******   ******** **********
                            ********** ***** ****  *******   ******** **********
                                               *******   ******** **********

alignment position          2651...... 2661...... 2671...... 2681...... 2691......
P911188             1757    actctgtgta ---------- ---------- ---------- ----------
P911190             1613    CAATCCTTTG ACacaaatgt catgtccccc AAGATATGCC TTTTCTCAAG
P911192              434    CAATCCTTTG ACgtgaggcc cct------- AAAATATGCC TTTTCACAAG
                            ********** **                    ********** **********
                            ********** **                    ********** **********
                            ********** **                    ********** **********
                            ********** **                    ********** **********
                            ********** **                    ********** **********

alignment position          2701...... 2711...... 2721...... 2731...... 2741......
P911188             1767    ---------- ---------- ---------- ---------- ----------
P911190             1663    GTTGGGCATT TAGAAAAAAa aagctctcag atgatgtagt gcc-------
P911192              477    GTTGGGCATT TAGAAAAAAg ccctcagact taaagttcct gctcgctaaa
                            ********** *********
                            ********** *********
                            ********** *********
                            ********** *********
                            ********** *********

alignment position          2751...... 2761...... 2771...... 2781...... 2791......
P911188             1767    ---------- ---------- ---------- ---------- ----------
P911190             1706    ---------- ---------- ---------- ---------- ----------
P911192              527    aggaaccatt ctctttttct ctggcaaaag ggaagtgtag ctcttttgtt

alignment position          2801...... 2811...... 2821...... 2831...... 2841......
P911188             1767    ---------- ---------- ---------- ---------- ----------
P911190             1706    ---------- ---------- ---------- ---------- ----------
P911192              577    aacatattac tgtgctgcct attagcgtgt tggagtcgag gttcaggtcg

alignment position          2851...... 2861...... 2871...... 2881...... 2891......
P911188             1767    ---------- ---------- ---------- ---------- ----------
P911190             1706    ---------- ---------- ---------- ---------- ----------
P911192              627    attaccagct tctaggcctc cttgctctag ctcctaggct gcttttctcc

alignment position          2901...... 2911...... 2921...... 2931...... 2941......
P911188             1767    ---------- ---------- ---------- ---------- ----------
P911190             1706    ---------- ---------- ---------- ---------- ----------
P911192              677    caatacattt gacccaggct aaccaccctc accaccaatc ccctacacag

alignment position          2951...... 2961...... 2971...... 2981...... 2991......
P911188             1767    ---------- ---------- ---------- ---------- ----------
P911190             1706    ---------- ---------- ---------- ---------- ----------
P911192              727    cttctctctg ctttcccagt agacagcctt gacactttgg aactttcccc

alignment position          3001...... 3011...... 3021...... 3031...... 3041......
P911188             1767    ---------- ---------- ---------- ---------- ----------
P911190             1706    ---------- ---------- ---------- ---------- ----------
P911192              777    ttttcaccca cgcagaggct aatttggttt ctttactgtg cctatttttt

alignment position          3051...... 3061...... 3071...... 3081...... 3091......
P911188             1767    ---------- ---------- ---------- ---------- ----------
P911190             1706    ---------- ---------- -TTTATAGAG TATATTATTT TTtccccaCA
P911192              827    cattgagtat attattatac tTTTATAGAG TATATTATTT TTccc---CA
```

```
                                          *********  ********** **         **
                                          *********  ********** **         **
                                          *********  ********** **         **
                                                                           **
                                                                           **
alignment position 3101...... 3111...... 3121...... 3131...... 3141......
P911188       1767 ---------- ---------- ---------- ---------T GAGTACGACT
P911190       1735 TGTGAAATAT ATACGAATTC CTTAATAAGT TCACAACAcc ggaCAAGATT
P911192        874 TGTGAAATAT ATATGAATTC CTTAATAAGT CCTCAACAtT GGGCAAGATT
                   ********** ********** ********** *******  * **********
                   ********** ********** ********** *******      *******
                   ********** ********** ********** *******      *******
                   ********** ********** ********** *******
                   ********** ********** ********** *******

alignment position 3151...... 3161...... 3171...... 3181...... 3191......
P911188       1778 AATATCTTCA ATTTACTAAg aagataaaga ggtcgataaa taAGGAGGTC
P911190       1785 AATATTTGTA ATTTACTGAA GAAACCTTTG T--------- --AGGTAGTC
P911192        924 AATATTTGTA ATTTATTTAA GGAATCTTTG T--------- --AGGTAGTC
                   ********** ********** ********** *            ********
                   ********** *****   ** ********** *            ********
                   ********** *****   ** ********** *            ********
                                      ** ********** *            ********
                                      ** ********** *            ********

alignment position 3201...... 3211...... 3221...... 3231...... 3241......
P911188       1828 AAGCCCGTTT TGCAAGGTCA CACAG----- ---------G AAAGAAgcag
P911190       1824 AGGACTCGTT TTCAAGGTCc tgtttaggga aaaaaaaaaA AAAAAAAGAC
P911192        963 AGAACTCGTT TTCAAGGTTC CACTT----- ---------A GAAGAAAAGC
                   ********** ********** *****               * **********
                   ********** ********
                   ********** ********
                   ********** ********
                   ********** ********

alignment position 3251...... 3261...... 3271...... 3281...... 3291......
P911188       1864 accacgaa-- ----ATGAAC TTCGGCTGTC ACCTGCGGTG GGCGCACTAG
P911190       1874 ATAGTGTAAC TGGCATAAAT TTCAGCTGTG ACCGGCTGGA TGCGCGCTTG
P911192        999 ATAGTCCAGC TAATATAggT TTCAGTTGTG ACCAACTGGA TGCGCGTTTG
                   ********** ********** ********** ********** **********
                                       * ********** ********** **********
                                       * ********** ********** **********
                                       * ********** ********** **********
                                       * ********** ********** **********

alignment position 3301...... 3311...... 3321...... 3331...... 3341......
P911188       1908 AggtCTTTTG AACTCCTTCG GCTACCGTCG CCGCGTTCTC gctgtgcact
P911190       1924 AATACTTTTG GACTCCTTAG GCCACCGTCG CCGGGTCCTC CATCGACTCt
P911192       1049 TATACTTTTG AACTCCTTAG GCCACCGTCG CCGATTCCTC CATCGACTC-
                   ********** ********** ********** ********** *********
                   ********** ********** ********** ********** *********
                   ********** ********** ********** ********** *********
                   ********** ********** ********** ********** *********
                   *   ****** ********** ********** ********** *********
                       ****** ********** ********** **********
                       *****   ********* ********** **********
                       *****   ********* ********** **********
                       *****   ********* ********** ***
                               ********* ********** ***

alignment position 3351...... 3361...... 3371...... 3381...... 3391......
P911188       1958 cttattCTGC GCCTGCGCGC GGCTACAGCA CGGTTCGTTT TTCCtttagt
P911190       1974 -------TGC GCATGCGTGC TGCTGGAAGG CTCTCCGTTT CTCCGAGAAC
P911192       1098 ------CTGC GCATGCGTGC TGCTAGAAGG CTCTCCGTTT CTCCGAGAAC
                         **** ********** ********** ********** **********
                          *** ********** ********** ********** **********
                          *** ********** ********** ********** **********
                          *** ********** ********** ********** **********
                          *** ********** ********** ********** **********
```

```
                           *** ********** ********** ********** **********
                           *** ********** ********** ********** ****

alignment position  3401...... 3411...... 3421...... 3431...... 3441......
P911188       2008  caGGAAGGAC GTTGGTGTTG AGGTGAGtcc ggtcccttt t gcatccctac
P911190       2017  CGGGAAGGAC GTTGGTGTAG AGGTGAGAAT GGTGGAG--- -AGAGAGAGG
P911192       1142  CGGGAAGGAC GTTGGTATAG AGGTGAGAAT GGTGGAGaga gAGAGAGAGG
                    ********** ********** ********** *******     *********
                    ********** ********** ********** *******     *********
                    ********** ********** ********** *******     *********
                    ********** ********** ********** *******     *********
                    ********** ********** ********** *******     *********
                    ********** ********** ********** *******
                      ******** ********** *******
                      ******** ********** *******
                      ******** ********** *******
                      ******** ********** *******

alignment position  3451...... 3461...... 3471...... 3481...... 3491......
P911188       2058  cccgac---- --------AC TGCGGGTTgt cacaacggca ccctcccgct
P911190       2063  AGATTCTGGG GTTTGACAAC TGCGGGTTTC tcGGCTTCTC GTgGGGGCGG
P911192       1192  AGATCCTGGG GTTTGACAAC TGTGGTTTTC --GGCTTCTC GT-GGGGCGG
                    ********** ********** **********   ******** ** *******
                    ********** ********** **********               *******
                    ********** ********** **********               *******
                    ********** ********** **********
                    ********** ********** **********

alignment position  3501...... 3511...... 3521...... 3531...... 3541......
P911188       2096  ttctctctgc ctcggattta gtcgtgactg tgtgtctccg ccgtgGTGCA
P911190       2113  CGAACTAGGA TGTCTTGCAC GTCACAGCCc A--------- -----GTGGA
P911192       1239  CGAACTAGGA TTTGTTCCAT GTCCCAGCCt A--------- -----GTGGA
                    ********** ********** *********  *               *****
                    ********** ********** *********  *               *****
                    ********** ********** *********  *               *****
                                                     *               *****

alignment position  3551...... 3561...... 3571...... 3581...... 3591......
P911188       2146  GCTTCAGGCC Tctcccgcat ctactctctc acgcttccgc tg--------
P911190       2149  GCCTCGGGCC TGCCTTCCTC GTGGGCGCG- TTGCCCCCGC CGCGTCCTCA
P911192       1275  GCCGCGGGCC TACCTTCCTC GTGGCCGCGc TTGTCCCCTC CGGGTCCTCA
                    ********** ********** *********  ********** **********
                    ********** ********** *********  ********** **********
                    ********** ********** *********  ********** **********
                    ********** ********** *********  ********** **********

alignment position  3601...... 3611...... 3621...... 3631...... 3641......
P911188       2188  ---------- --------CG GCCT--GAGG GAGGGCGGCg ggcggaccac
P911190       2198  GGAGGCTGCG GGCCTGacCG GCCT--GAAC TCGGGCGGCC CCGCCATCTC
P911192       1325  GGAGGCTGCG GGCCTGgtgg gcctgaGAGC TCGGGCGGCC CCGCTGTTTA
                    ********** ******  ** ****  **** ********** **********
                    ********** ******           **** ********** **********
                    ********** ******           **** ********** **********
                    ********** ******

alignment position  3651...... 3661...... 3671...... 3681...... 3691......
P911188       2218  ggaccggggt gggttgcgac ggccccaccg aagccgggtg ggctgcggga
P911190       2246  CCCGCGTGCG CTcttagggc tccGGAGGAG CTCTGCGGAG CCTCAGGGTC
P911192       1375  CCCGAGTGCG CTatcggggc cttGGAGGAG CTCTGCGGAG CCTCTGGGCC
                    ********** **            ******* ********** **********
                    ********** **            ******* ********** **********
                    ********** **            ******* ********** **********
                                             ******* ********** **********

alignment position  3701...... 3711...... 3721...... 3731...... 3741......
P911188       2268  ccctcgagaa cccagctggc ttcggagtga gctgGCCCGG GGCTGGTACC
P911190       2296  TCGCTGgTGG CC-------- ---------- ----GGCCGG GCCCGTTTCC
P911192       1425  TCGCTGaTGG CC-------- ---------- ----GGCCGG GCCCGTTACC
```

```
                        ****** *** **                                   ****** **********
                        ****** *** **                                   ****** **********
                        ****** *** **                                   ****** **********
                        ****** *** **                                   ****** **********

alignment position      3751...... 3761...... 3771...... 3781...... 3791......
P911188           2318  CGCTGCGCTT TTTCACAGCC ATTGTGACCT TGGTGAGGTC ATTTGACCTA
P911190           2324  CCTTGTATTT TGCCGCGGCC AGGTTGACCT TGATTGGGTC GTTTGACCTG
P911192           1453  CGTTGTATTT TGCtGCGGCC AGGGTGACCT TGATTGGGTC GTTTGACTTG
                        ********** ********** ********** ********** **********
                        ********** ********** ********** ********** **********
                        ********** *** ****** ********** ********** **********
                        ********** *** ****** ********** ********** **********
                                ** *** ****** ********** ********** **********
                                       ****** ********** ********** **********
                                          *** ********** ********** ********

alignment position      3801...... 3811...... 3821...... 3831...... 3841......
P911188           2368  TCTGGCCccg gTGTTTCTCC CGTGAGAAAA GGGGTGgtgg tttctaatgc
P911190           2374  cGGGGGC--- -TGGTGCTCC AGTTGGATAA GGGGTGCGAC CTTGGAGGGG
P911192           1503  TGGGGCC--- -TGGTGCTCC ACTTGGATAA GGGCTGCGAT CTTGGAGGGG
                        *******     ********* ********** ********** **********
                        *******     ********* ********** ********** **********
                         ******     ********* ********** ********** **********
                         ******     ********* ********** ********** **********
                         ******     ********* ********** ******
                         ******

alignment position      3851...... 3861...... 3871...... 3881...... 3891......
P911188           2418  agctggtcgt ga-------- -GGCGCAAGG GTTAGTGTAG GCGAAGCAGC
P911190           2420  ACTCAGGCAC CGAGGGGCCC CGGTGGTCGG CCTGGTGTGG GAGTAGAATT
P911192           1549  ACGGAGGCAC CCAGGGGCCC TGGTGGTCGG CCTaGTGTGG AAGTAGAGCT
                        ********** ********** ********** ********** **********
                        ********** ********** ********** *** ****** **********
                        ********** ********** ********** *** ****** **********
                        ********** ********** ********** *** ****** **********

alignment position      3901...... 3911...... 3921...... 3931...... 3941......
P911188           2459  TGGCAGCgtc gctcgctgtg tggc------ ---------- ----------
P911190           2470  GGGAAGCTGG AGGGTCCCCG A----TCTGT CGCGCCT--- ----------
P911192           1599  GGGAAGCTGG AGGGTCCCCG AcatgTCTGT CGCGCCTgct ccaccggccc
                        ********** ********** *    ***** *******
                        ********** ********** *
                        ********** ********** *
                        ********** ********** *

alignment position      3951...... 3961...... 3971...... 3981...... 3991......
P911188           2483  ---------- ---------- ---------- ---------- ----------
P911190           2503  ---------- ---------- ---------- ---------- ----------
P911192           1649  tcggggcgcc tgctgggccc cagttccgca gtctcagtga cctgagttct

alignment position      4001...... 4011...... 4021...... 4031...... 4041......
P911188           2483  ---------- ---------- ---------- ---------- ----------
P911190           2503  ---------- ---------- ---------- ---------- ----------
P911192           1699  taccgaagac ttttcttaag ggtaaattct gaaaaggcta cagctaggtc

alignment position      4051...... 4061...... 4071...... 4081...... 4091......
P911188           2483  ---------- ---------- ---------- ---------- ----------
P911190           2503  ---------- ---------- ---------- ---------- ----------
P911192           1749  gcatctgcct ttctgtagat ggggtatgac actactgtcg tagtttttgc

alignment position      4101...... 4111...... 4121...... 4131...... 4141......
P911188           2483  ---------- ---------- ---------- ---------- ----------
P911190           2503  ---------- ---------- ---------- ---------- ----------
P911192           1799  tcatcggttg tgccagttac cgccttgttg ggggtagtgg aactgtatag
```

```
alignment  position  4151......  4161......  4171......  4181......  4191......
P911188        2483  ----------  ----------  ----------  ----------  ----------
P911190        2503  ----------  ----------  ----------  ----------  ----------
P911192        1849  gagagaagcg  gaaccggcaa  agatgaagct  gcctaagtca  ctgaaacctt

alignment  position  4201......  4211......  4221......  4231......  4241......
P911188        2483  ----------  ----------  ----------  ----------  ----------
P911190        2503  ----------  ----------  ----------  ----------  ----------
P911192        1899  gcagaagtca  gtccgcagtt  actaaaatac  gatgtgcacg  tagagttcat

alignment  position  4251......  4261......  4271......  4281......  4291......
P911188        2483  ----------  ----------  ----------  ----------  ----------
P911190        2503  ----------  ----------  ----------  ----------  ----------
P911192        1949  tcaatcactt  accccattct  caggcttttt  tgtttgtttg  tttgttttac

alignment  position  4301......  4311......  4321......  4331......  4341......
P911188        2483  ----------  ----------  ----------  ----------  ----------
P911190        2503  ----------  ----------  ----------  ----------  ----------
P911192        1999  ttgcaaagga  cattggctac  catgagttcc  ggtgctctgt  tgccgaaacc

alignment  position  4351......  4361......  4371......  4381......  4391......
P911188        2483  ----------  ----------  ----------  ----------  ----------
P911190        2503  ----------  ----------  ----------  ----------  ----------
P911192        2049  acagatgcgt  ggtcttctgg  ccaagcgtct  gcgggttcat  attgttggcg

alignment  position  4401......  4411......  4421......  4431......  4441......
P911188        2483  ----------  ----------  ----------  ----------  ----------
P911190        2503  ----------  ----------  ----------  ----------  ----------
P911192        2099  cattcgttgt  ggccctagga  gttgctgctg  cctataaggt  atgtgcttgc

alignment  position  4451......  4461......  4471......  4481......  4491......
P911188        2483  ----------  ----------  ----------  ----------  AGCGTTTTAA
P911190        2503  ----------  ----------  ----------  ----------  ----------
P911192        2149  ttgtgtgtgc  tttaggttac  aaggtttaaa  cctagttaag  AGCTTTTTAA
                                                                     **********

alignment  position  4501......  4511......  4521......  4531......  4541......
P911188        2493  ATTAGAgct-  ----------  ----------  ----------  ----------
P911190        2503  ----------  ----------  ----------  ----------  ----------
P911192        2199  AAAAGAaact  cccatcttta  agatattccc  ctcttaacct  tccataagc
                     ******

alignment  position  4551......  4561......  4571......  4581......  4591......
P911188        2502  ----------  ----------  ----------  ----------  ----------
P911190        2503  ----------  ----------  ----------  ----------  ----------
P911192        2249  ttcagcgtgc  caggagccca  tgtagttgac  cctatctaca  aatgaagctg

alignment  position  4601......  4611......  4621......  4631......  4641......
P911188        2502  ----------  ----------  ----------  ----------  ----------
P911190        2503  ----------  ----------  ----------  ----------  ----------
P911192        2299  tgtttgctat  atcaaagaac  caggccgagt  ggtttaaatg  cagaaatttg

alignment  position  4651......  4661......  4671......  4681......  4691......
P911188        2502  ----------  ----------  ----------  ----------  ----------
P911190        2503  ----------  ----------  ----------  ----------  ----------
P911192        2349  ctttcttaca  gggctgtttt  tgagataggt  cttactatat  aaccctgcct
```

```
alignment  position  4 7 0 1 . . . . . .   4 7 1 1 . . . . . .   4 7 2 1 . . . . . .   4 7 3 1 . . . . . .   4 7 4 1 . . . . . .
P911188     2502 - - - - - - - - - -   - - - - - - - - - -   - - - - - - - - - -   - - - - - - - - - -   - - - - - - - - - -
P911190     2503 - - - - - - - - - -   - - - - - - - - - -   - - - - - - - - - -   - - - - - - - - - -   - - - - - - - - - -
P911192     2399 a g g c a a g g c t   g g c c a g g a g c   t c a t g t g a t t   t g c c t g c c t c   t a c c c c a c a a

alignment  position  4 7 5 1 . . . . . .   4 7 6 1 . . . . . .   4 7 7 1 . . . . . .   4 7 8 1 . . . . . .   4 7 9 1 . . . . . .
P911188     2502 - - - - - - - - - -   - - - - - - - - - -   - - - - - - - - - -   - - - - - - - - - -   - - - - - - - - - -
P911190     2503 - - - - - - - - - -   - - - - - - - - - -   - - - - - - - - - -   - - - - - - - - - -   - - - - - - - - - -
P911192     2449 t g c t g g g a t c   a a a a g a t g t g   c a c c a c c a t a   c t c c c a g t t t   t t a a g a t c a a

alignment  position  4 8 0 1
P911188     2502 - - -
P911190     2503 - - -
P911192     2499 g g t
```

Pairwise similarities:

For each pairwise alignment, the similarity (relative to the maximum similarity) and the number of identical nucleic acids (in % of shorter sequence) is given. Maximum values are underlined.

	P911190 (2502 bp)	P911192 (2501 bp)
**P911188 (2501 bp)**	0.087<br>19 %	0.076<br>12 %
**P911190 (2502 bp)**		1.000<br>40 %

Please note that the **similarity value 1.000** marks only the two **most similar** sequences, it does not necessarily mean that these sequences are identical.

**Extract alignment region**	
**Positions in alignment**	from: to:
**Output file**	dialign.seq

Extract aligned sequences

For comments, questions or bug reports, please contact support@genomatix.de

© Genomatix Software GmbH 1998-2005 - All rights reserved. License Agreement GEMS Launcher 4.1.1

Genomatix-Logo	Logout	Personal	Messages	GEMS-Logo

Genomatix_MainGEMSElDoradoGene2PromoterBiblioSphere
Frequently_asked_questionsYour_ResultsYour_SequencesYour_ProtocolHelp

GEMS Launcher Task: *DiAlign TF*: Multiple alignment plus TF sites
working on C4-COX6B2-HMR (3 seq.)

[Alignment] [Pairwise_similarities]

DiAlign professional TF Release 3.1 December 2004 **Fri May 6 01:19:06 2005**

Solution parameters:

**Sequence file:**	C4-COX6B2-HMR (3 seq.)
**Type of sequences:**	nucleotide sequences
**Threshold T:**	0.00
**'*' signs below alignment denote:**	diagonal similarity (max. similarity is represented by 10 '*' signs)
**Alignment output:**	complete alignment is shown<br># nucleic acids per line: 50
**TF output:**	common TF matches located in aligned regions (common to 3 (100.0 %) sequences)
**Family matches:**	yes
**MatInspector library:**	Matrix Family Library Version 5.0 (February 2005)
**Selected groups** (core/matrix sim):	• ALL user_defined.lib (0.75/Optimized)<br>• ALL vertebrates.lib (0.75/Optimized)

Aligned Sequences:

No.	Sequence Name	Sequence Description
1	P984975	P984975 sym=COX6B2|loc=Loc125965|taxid=9606|spec=Homo sapiens|chr=19|ctg=NT_011109|str=(-)|start=28133854|end=28134769|len=916|tss=501,516|comm=cytochrome c oxidase subunit VIb polypeptide 2 (testis); (AK057427/501/gold;NM_144613/516/silver;)
2	mCOX6B2	mCOX6B2 mm6_dna range=chr7:3998838-3999737 5'pad=0 3'pad=0 revComp=TRUE strand=? repeatMasking=none
3	rn3_dna	rn3_dna range=chr1:67888264-67889158 5'pad=0 3'pad=0 revComp=FALSE strand=? repeatMasking=none

Alignment (DiAlign format):

Please note that only **upper-case** letters are considered to be **aligned**. For more information, please have a look at the user guide.

V$NFKB V$TBPF V$ZBPF V$EGRF V$SP1F V$MAZF V$PAX2 V$ECAT V$PCAT V$NF1F V$AP2F V$HNF4
V$ETSF V$VMYB V$CMYB

```
alignment
position        1.........  11........  21........  31........  41........

P984975       1 aggggggcga  ggga------  -GGAGGGTGG  GGGGGGcggg  taacggaggg
mCOX6B2       1 GAGAACACCG  AGTGTGGTCG  CGGAGGGTGG  AGGGGGAT--  ----------
rn3_dna       1 GAGAACACCG  AGTGTGGTCG  CGGAGGG-GG  AGGGAGAT--  ----------
                **********  **********  **********  *********
                **********  **********  *******  **  *********
                **********  **********  *******
```

```
alignment position
               51........ 61........ 71........ 81........ 91........
P984975     44 gggaggacga ggggggcggg ggggttccca ggagcccgga ccccaccgct
mCOX6B2     39 ---------- ---------- ---------- ---------- ----------
rn3_dna     38 ---------- ---------- ---------- ---------- ----------

alignment position
               101....... 111....... 121....... 131....... 141.......
P984975     94 gctctttcct cttaacccca caggccccga gggttctgtg cgcaccgact
mCOX6B2     39 ---------- ---------- ---------- ---------- ----------
rn3_dna     38 ---------- ---------- ---------- ---------- ----------

alignment position
               151....... 161....... 171....... 181....... 191.......
P984975    144 gtaggaagcg gaatgttgaa agccgcttgg gGGGATTTGC CCCCTCTGGT
mCOX6B2     39 ---------- ---------- ---------- -GGGATCCTC CTTCTCCCAT
rn3_dna     38 ---------- ---------- ---------- -GGGATCCTC CTCCTCCCAT
                                                *********  **********
                                                *********  ** *******
                                                *********  ** *******
                                                              *******

alignment position
               201....... 211....... 221....... 231....... 241.......
P984975    194 AGATACCAGA ACTAtggata tgccttTATA TATTGGGGGG CGGGGGGGGG
mCOX6B2     58 AGAGACCAGA GCTAGATATA AG----TTTA T-----TGGT GAGGATGGGG
rn3_dna     57 AGAGACCAGA GCTAGATATA AA----TTTA TCTTACTGGG GAGGATGGGG
               ********** ********** **    **** ********** **********
               ********** ********** **    **** *     **** **********
               ********** ********** **    **** *
               ********** ****             **** *

alignment position
               251....... 261....... 271....... 281....... 291.......
P984975    244 CGGGGGCGAG cccgt-GGAG CCGCCCAACT TCGCAGAGAT TAAAgcttg-
mCOX6B2     99 CGGGGCAGAG ------GGAG AAGCTCAACT TTGTAGGGAT TAAAAGTCCT
rn3_dna    103 CGGGGCTGAt ggtcaaGGAG AAGCTCAATT TTGTAGGGAT TAAAAGTCCT
               **********       **** ********** ********** **********
               *********        **** ********** ********** **********
                                **** ********** ********** **********
                                **** ********** ********** **********
                                **** ********** ********** ****

alignment position
               301....... 311....... 321....... 331....... 341.......
P984975    292 AGCCTGAGAC TCAgcgagtc gcacctcggt gcctgtgtgc ac--------
mCOX6B2    143 AGTCTGAGAC TCAAAGCCCT GTCTTCGTGC TGGTAAGTGT ACGCGcTCTT
rn3_dna    153 AGTCCGAGAC TCAAAGCCCT GTTTCTGTGC TGGTAAGTGT AAGCGtTCTT
               ********** ********** ********** ********** ***** ****
               **** ***** ********** ********** ********** ***** ****
               **** ***** ********** ********** ********** ***** ****
               ****

alignment position
               351....... 361....... 371....... 381....... 391.......
P984975    334 --GCGCAGGC CCCCGAGGGG Gcgtcgggg- --GAGCAAGG GGTGGGGTGG
mCOX6B2    193 GTGCGCGGTT CTCACAAGGG GACTGCCTAC AAGAACCAAG GGGGAGGGGC
rn3_dna    203 GTGCGCGGGT CCCCCCAGGG GACTGCCTAC AAGAACta-- GGGGGAGGGC
               ********** ********** ********** ********** **********
               ********** ********** ********** *******    **********
               ********** ********** ********** *******    **********
                                                           **********

alignment position
               401....... 411....... 421....... 431....... 441.......
P984975    379 GGGCGCCCCT ACCAGCCCCG CGGTGCAcGA CGGCTCCCAT TGGCTGGGGC
```

```
mCOX6B2  243 CGCCACTGAT CCCACCCCCG CATTACA-GA CAGCTCCTAT TGGCTGGGAC
rn3_dna  251 CTCCACTGAT CCCACCCCCG CATTACA-GA CGGCTCCTAT TGGCTGGAAC
             ********** ********** ******* ** ********** **********
             ********** ********** ******* ** ********** **********
             ********** ********** ******* ** * ******** **********
             ********** ********** ******* ** * ******** **********
                                          ** *

alignment
position     451....... 461....... 471....... 481....... 491.......

P984975  429 TCgggcgtc c t-AGCCAATC CGGCCGCGGG GTgcg----T TTCTCCtgac
mCOX6B2  292 TCCCTGGGaG CCAGCCAATC CGGCCTGAGG GTCGTATTTT TTCTCCc--G
rn3_dna  300 TCCCTGGGgG CCAGCCAATC AGGCCTGGGG GTCGTATTTT TTTTCtcgtG
             ******** * ********** ********** ********** ******   *
             ******** * ********** ********** ********** *****    *
             **       * ********** ********** ********** *****    *
             **           ******** ********** **                  *

alignment
position     501....... 511....... 521....... 531....... 541.......

P984975  474 ccgggtggga ccgcaccccg cggactcaga agcgagcggc accccGGGAC
mCOX6B2  340 TGCTCTT--- ---------- ---------- ---------- -----GGGAC
rn3_dna  350 TGCTCTT--- ---------- ---------- ---------- -----GGGAC
             *******                                        *****
             *******                                        *****
             *******                                        *****
             *******                                        *****

alignment
position     551....... 561....... 571....... 581....... 591.......

P984975  524 CATCCCACAg cagatccagt ggccgc---- ---------- ----------
mCOX6B2  352 CAGCCCTCAT TCTCAAACTA AAGGCACgGG TGGATTCCGC AGCCCTGGTG
rn3_dna  362 CAGCCCTCAT TCTAAAGCTA AAGGCACtGG TGGACTCCGC AACTGTGGTG
             ********** ********** *******  ** ********** **********
             ********** ********** *******  ** ********** **********
             ********** ********** *******
             ********** ********** *******

alignment
position     601....... 611....... 621....... 631....... 641.......

P984975  550 ---------- ------CAAC GTCAGGCTGG TGAGGGCACG ACGGGTGGGG
mCOX6B2  402 TTCCAGCTAC TTTGGctCAC TTCAGTCTGG TAAGGACAGG GTGGGTGGTG
rn3_dna  412 TTCCAGCTAC GTTGGtCCAC TTCAGTCTGG TGAGGACGGG GTGGGTGGTG
             ********** ***** **** ********** ********** **********
             ********** *****  *** ********** ********** **********
                               *** ********** ********** **********
                               *** ********** ********** **********
                               *** ********** ********** **********
                                ** ********** ********** **********

alignment
position     651....... 661....... 671....... 681....... 691.......

P984975  584 TTCAGGGgcg gGGGTCAGGA TGGGGAGACG Ggtcggagcg aGGGCGCCCC
mCOX6B2  452 TTTAGAGt-- -GGGACAGGA GCTGGGGAAG GTGGAAGCAC GAGGCGCCCT
rn3_dna  462 TTTAGAGt-- -GGGTTAGGA GCTGGGGAAG GTGGAAGCAC GGGGCGGCCC
             *******     ********* ********** ********** **********
             *******          **** ********** ********** **********
             *******          **** ********** ********** **********
             *******                                    *********
             *******                                     ********
             *****

alignment
position     701....... 711....... 721....... 731....... 741.......

P984975  634 CTCGGCCCCA CGGAGTCCcc gtccgtCCC GTCTCGCAGG AGTTGcctcC
mCOX6B2  499 CTAGGCCCCA CGGAGCCCTG CAC----CCC TTCCTGCAGG AATTG----C
rn3_dna  509 CTAGTCTCCA CGGAGCTCTG CAC----CCT TTCTTGCAGG AATTT----C
```

```
                 ********** ********** ***    *** ********** *****    *
                 ********** ********** ***    *** ********** *****    *
                 ****   *** ********** ***    *** ********** *****    *
                 ****   *** ********                                  *
                 ****   *** ********

alignment
position         751....... 761....... 771....... 781....... 791.......
P984975      684 TTCGTGGATG TTGGATGTGG AAGCCCAGGA GCCCCCcaag gggaaatggt
mCOX6B2      541 TACtTGGATG TTGGGTGTTC AAGCCCAGAA GCCCCCTCCG GGCCAATGGA
rn3_dna      551 TACCTGGATG TTGGGTGTTC AAGCCCAGAT GCCTGCTCCA GGCCAATGGA
                 ********** ********** ********** ********** **********
                 ********** ********** ********** ********** **********
                 *** ****** ********** ********** ********** **********
                 *** ****** ********** ********** ******         ******
                     ****** ********** ********** ******
                     ****** ********** ********** ***

alignment
position         801....... 811....... 821....... 831....... 841.......
P984975      734 cgACGCCGCC CTTCGACCCG CGCTTCCCCA GCCAGAACCA GAtcCGTAAC
mCOX6B2      591 CAACGCCGCC CTTTGATCCG CGCTTCCCTA ACCAGAACCA GACGCGTAAC
rn3_dna      601 CAACGCCGCC CTTCGACCCG CGCTTCCCTA ACCAAAACCA GACGCGTAAC
                 ********** ********** ********** ********** **********
                 ********** ********** ********** ********** **********
                 ********** ********** ********** ********** **********
                 ********** ********** ********** ********** **********
                   ******** ********** ********** ********** **********
                   ******** ********** ********** ********** **   ******
                   ******** ********** ********** ****  ***** **   ******
                   ******** ********** ********** ****  ***** **   ******
                   ******** ********** ********** ****  ***** **   ******
                   ******** ********** ********** ****  ***** **   ******

alignment
position         851....... 861....... 871....... 881....... 891.......
P984975      784 TGCTACCAGA ACTTCCTGGG TGAGACCtGC TCAGCCAGGT TATGGGAGGA
mCOX6B2      641 TGCTACCAGA ATTTTCTGGG TGAGACCGGT TGAGCCAAAT TAGAGGGGAG
rn3_dna      651 TGCTACCAGA ATTTTCTGGG TGAGACCGGT TGGGCCAAAT TAGAGGGGAG
                 ********** ********** ********** ********** **********
                 ********** ********** ********** ********** **********
                 ********** ********** ********** ********** **********
                 ********** ********** ********** ********** **********
                 ********** ********** *******
                 ********** ********** *******
                 ********** ********** *******
                 ********** ********** *******
                 ********** ********** *******
                 ********** ********** *****

alignment
position         901....... 911....... 921....... 931....... 941.......
P984975      834 GCagggtgtg acaagggacg gcagggaggg gcactCCCAC CCTAAGTCCC
mCOX6B2      691 GCC------- ---------- ---------- -----CCCAC CTGGAGaCCG
rn3_dna      701 GCC------- ---------- ---------- -----CCCAC CTGGAGGCCG
                 ***                                   ***** **********
                 ***                                   ***** **********
                 ***                                   ***** ****** ***
                 ***                                   ***** ****** ***
                                                       ***** ******

alignment
position         951....... 961....... 971....... 981....... 991.......
P984975      884 GCGGAGACCG TTAGGGTGGG AGGCCGGGCg ggg------- ----------
mCOX6B2      709 TAGGAGACCG TTGGAATGGG AGGCTGGGCC GCGGCCTGGA GGGGTGGGGC
rn3_dna      719 TCGGAGACCG TTGGAATGGG AGGCTGcaCC GTGGCCTAGA GGGGTGGGGC
                 ********** ********** ********** ********** **********
                 ********** ********** ******  ** ********** **********
                 ********** ********** ******  *
                 ********** ********** ******
```

```
                  ******** ********** ****

alignment
position          1001...... 1011...... 1021...... 1031...... 1041......
P984975       917 ---------- ---------- ---------- ---------- ----------
mCOX6B2       759 CTGTACCAAa gtgggTGGGG CTGAGCCATG GAGGGGCGGG GCCTGGAATG
rn3_dna       769 CTGCACAAAg cgga-TGGGG CTGAACCATG GAGGGACCGA CCCTGGAATG
                  *********       ***** ********** ********** **********
                  *********       ***** ********** ********** **********
                                  ***** ********** ********** **********

alignment
position          1051...... 1061...... 1071...... 1081...... 1091......
P984975       917 ---------- ---------- ---------- ---------- ----------
mCOX6B2       809 GAGGGAGGCA GCTGGTCTGA AGGGATTAAG TTTGCCGCGT TGGagtgaaa
rn3_dna       818 GAGGGAGGCA GCTGGTCTGG AGGGAATAAG ATTGCCACGT TGGg------
                  ********** ********** ********** ********** ***
                  ********** ********** ********** ********** ***
                  ********** ********** ********** ********** ***

alignment
position          1101...... 1111...... 1121...... 1131...... 1141
P984975       917 ---------- ---------- ---------- ---------- --
mCOX6B2       859 tgtATTGAGC CAGAAGGGAT GGAGCCTTGT CTGTAAAggg ca
rn3_dna       862 ---ATTGAGC CAGAAAGGAT GGAGCCTTGT CTGTAAA--- --
                     ******* ********** ********** *******
                     ******* ********** ********** *******
                     ******* ********** ********** *******
```

Pairwise similarities:

For each pairwise alignment, the similarity (relative to the maximum similarity) and the number of identical nucleic acids (in % of shorter sequence) is given. Maximum values are underlined.

	mCOX6B2 (900 bp)	rn3_dna (895 bp)
P984975 (916 bp)	0.205<br>40 %	0.189<br>42 %
mCOX6B2 (900 bp)		1.000<br>88 %

Please note that the **similarity value 1.000** marks only the two **most similar** sequences, it does not necessarily mean that these sequences are identical.

Extract alignment region	
**Positions in alignment**	from: to:
**Output file**	dialign.seq

Extract aligned sequences

For comments, questions or bug reports, please contact support@genomatix.de

© Genomatix Software GmbH 1998-2005 - All rights reserved.

License Agreement

GEMS Launcher 4.1.1

Genomatix-Logo | Logout Logout | Personal Personal | Messages Messages | GEMS-Logo

Genomatix_MainGEMSElDoradoGene2PromoterBiblioSphere
Frequently_asked_questionsYour_ResultsYour_SequencesYour_ProtocolHelp

GEMS Launcher Task: *DiAlign TF*: Multiple alignment plus TF sites
working on C4-COX6A1 (3 seq.)

[Alignment] [Pairwise_similarities]

DiAlign professional TF Release 3.1 December 2004 **Wed Aug 10 18:02:04 2005**

Solution parameters:

**Sequence file:**	C4-COX6A1 (3 seq.)
**Type of sequences:**	nucleotide sequences
**Threshold T:**	0.00
**'*' signs below alignment denote:**	diagonal similarity (max. similarity is represented by 10 '*' signs)
**Alignment output:**	complete alignment is shown<br># nucleic acids per line: 50
**TF output:**	common TF matches located in aligned regions (common to 3 (100.0 %) sequences)
**Family matches:**	yes
**MatInspector library:**	Matrix Family Library Version 5.0 (February 2005)
**Selected groups** (core/matrix sim):	• ALL vertebrates.lib (0.75/Optimized)

Aligned Sequences:

No.	Sequence Name	Sequence Description	Length
1	hCOX6A1	hCOX6A1 sym=hCOX6A1|spec=Homo sapiens|chr=12|str=(+)|start=119338137|end=119339250|len=1114|tss=493	1114 bp
2	mCOX6A1	mCOX6A1 sym=mCOX6A1|spec=Mus musculus|chr=5|str=(-)|start=114458935|end=114460045|len=1111|tss=522	1111 bp
3	rCOX6A1	rCOX6A1 sym=rCOX6A1|spec=Rattus norvegicus|chr=12|str=(+)|start=42392988|end=42394045|len=1058|tss=532	1058 bp

Alignment (DiAlign format):

Please note that only **upper-case** letters are considered to be **aligned**. For more information, please have a look at the user guide.

V$PLZF V$NRF1 V$SP1F V$ZBPF V$EGRF V$ETSF V$YY1F V$CP2F V$HESF V$CHRE V$VMYB

```
alignment position 1.........  11........ 21........ 31........ 41........
hCOX6A1      1 actcttggta tcaca----- ---------- ---------- ----------
mCOX6A1      1 tgccatgaaa GATTTCGGGT AACTCTCGGG ATGCAGCGCT GGCAAAAaTC
rCOX6A1      1 ---------- GATTTCGGGT AACTCTGGGA ATGCAGCGCT GGCAAAA-TC
                          ********** ********** ********** ******* **
                          ********** ********** ********** ******* **
                          ********** ********** ********** *******

alignment position 51........  61........ 71........ 81........ 91........
hCOX6A1     16 ---------- -GACTTTATA TTCTAGTGGG GGAGACAGAC AAAATAATAA
mCOX6A1     51 TAAGCACTGC GGACTTTACA GTCTATGTAG GGCACAACGG AAGAGAATAA
```

```
rCOX6A1      40 TAAGCACCAC GGACTTTACA GTCCATATAG GGCGTCACAG AAGAGAATAA
                ********** ********** ********** ********** **********
                ********** ********** ********** ***    **** **********
                            ********* ********** ***    **** **********
                                                       **** **********

alignment position 101....... 111....... 121....... 131....... 141.......
hCOX6A1      55 t--------- ---------- ---------- ATAACGACga aggtgcaatg
mCOX6A1     101 CAACGGTCTT TAAGAGCGAT agggacaatg ATAACGACaa ggacagggcT
rCOX6A1      90 CAACGGTCTT TAAGAGTGAT gacaaggg-- ---------- ---------T
                ********** **********            ********           *
                ********** **********                               *
                ********** **********                               *

alignment position 151....... 161....... 171....... 181....... 191.......
hCOX6A1      76 ttatgtgcta tgagaaaacg cagtcggagg atg------G CGAGGAAGGG
mCOX6A1     151 GTCGAGGGCC TGGGAAATCG CGGAATTAGA AGGCAGCGAG CGAGGAAAGA
rCOX6A1     119 GTCGGGTGCC TTGGAAATCG CGGAATTAGA AGTCAGCGAg ---GAAAGGA
                ********** ********** ********** ********** **********
                ********** ********** ********** *********       *****
                ********** ********** ********** *********       *****

alignment position 201....... 211....... 221....... 231....... 241.......
hCOX6A1     120 GAACGGAATT TTAGATAGGG CGGTCTGGGA TCagaacctc cagatgCCCG
mCOX6A1     201 GAATGGCGCC TCCGCTAACG TGACTTGTTC TCcGCACTTC AGAT--CCTG
rCOX6A1     166 GAATGGCGCT TCCGATAACG TCATTTGTTC TCgGCATTTC AGAT--CCTG
                ********** ********** ********** **  ******* ****  ****
                ********** ********** ********** **  ******* ****  ****
                ******                              ******* ****  ****
                                                    ******* ****  ****
                                                                  ****
                                                                  ****
                                                                  ****

alignment position 251....... 261....... 271....... 281....... 291.......
hCOX6A1     170 TCCATGGACT CCAACGAAGG GAGCGATTcc aggtaccagg gcaccctgca
mCOX6A1     249 TCCAGGGACT CCAAAGAAGG GAGCGATTAA AACCAGAAAA g--------C
rCOX6A1     214 TCCAGGGACT CCAAAGAAGG TAGCagTTAA AAACAAAACA aaaaaatatC
                ********** ********** ********** **********          *
                ********** ********** *******                        *
                ********** ********** ****
                ********** ********** ****
                ********** ********** ****
                ********** ********** ****
                ********** ********** ****

alignment position 301....... 311....... 321....... 331....... 341.......
hCOX6A1     220 ca----AACC TCTCTTGAGC ttccgcctcg ccc------- ----------
mCOX6A1     291 TCGCAAAAGT TCAGTTCCGC GGACATCCTC ACCTAGgcgc tgacaAGCAG
rCOX6A1     264 TCACAGAAGC TCTATTCAGC GAACATCCTC ACCCAGcagc ag---AGGAG
                ********** ********** ********** ******          *****
                ********** ********** ********** ******

alignment position 351....... 361....... 371....... 381....... 391.......
hCOX6A1     249 --AGGTGGGG acggtaggga gaccggaggg cacacgcatg cgcacgaagg
mCOX6A1     341 GGAGATGGGG CAC------- ---------- ---------- ----------
rCOX6A1     311 GGAGGTGGGG CAC------- ---------- ---------- ----------
                ********** ***

alignment position 401....... 411....... 421....... 431....... 441.......
hCOX6A1     297 aaacggtaaa gcctgaaggg AGGTGCAGAG CGCATGCTCT CTCTTGCCCG
mCOX6A1     354 ---------- ---------- AGGAACGGGG CGCATGCGCG CTCTCGCTCC
rCOX6A1     324 ---------- ---------- AGGTATGAAG CGCATGCGCA ATCTCACTCG
                                      ********** ********** **********
                                      ***      * ********** **********

alignment position 451....... 461....... 471....... 481....... 491.......
hCOX6A1     347 AGatg--CCG AGGATTTTGA CAAGGACtcc gtcgtcccgg atgatAGTGC
mCOX6A1     384 AGCCGCCCCa g--------- ---GGACGGA TAA------- -----AGTGC
```

```
rCOX6A1             354 TGCCGCCCCG AGGGTTTGGA CCAGGACCGA AAA------- -----TGTGC
                        ********** ********** ********** ***             *****
                        *********                 ****** ***             *****
                                                 ****

alignment position      501....... 511....... 521....... 531....... 541.......
hCOX6A1             395 TCAGGttaat ---------G CCAG---TGG GAGGGCGGCG CCCAATAGTA
mCOX6A1             410 GCAGGCGCTG GAGTTCCTAG CCAGgtcTGA GAAGGCGTTG CCCAATAGGA
rCOX6A1             392 GCAGGCGCTA CAATTCCTGG CCAG---TGA GAAGGCGTTG CCCAATga-A
                        ********** ********** ****   *** ********** **********
                        ********** ********** ****   *** ********** ******   *
                                           * ****   *** ********** ******   *

alignment position      551....... 561....... 571....... 581....... 591.......
hCOX6A1             433 ACTTCCTTTG GAGgttgTAG TACCGCCCCC AGAGCCaatt ttCCACTTCC
mCOX6A1             460 ACGTCGTTCG GCGTCT-TTG TCCCGCCCCC TTCGCCcgtC C-CCATTTCC
rCOX6A1             438 ACTTCGTTTG GCGTCT-CTG TCCCGCCCCC TTCGgggacC C-CCATTTCC
                        ********** ****** *** ********** ******   * * ********
                        ********** ****** *** ********** ****     * * ********
                        ********** ****** *** ********** ****     * * ********
                                                                 * * ********
                                                                       ********

alignment position      601....... 611....... 621....... 631....... 641.......
hCOX6A1             483 GCTTCCGGCG CTGCGGcagt ccAGATCAAA AATGGCGGTA GTTGGTGTGT
mCOX6A1             508 GCTTCCGGCG CGCCGGTGT- --AGTTCCAT GATGGCGTCG GCGGTGCTGT
rCOX6A1             486 GCTTCCGGCG CGCCGGGGT- --AGTTCCAA GATGGCGTCG GCGGTGCTGA
                        ********** *********    ******** ********** **********
                        ********** *********    *******  ********** **********
                        ********** *********    *******  ********** **********
                        ********** *********    *******
                        ********** ******

alignment position      651....... 661....... 671....... 681....... 691.......
hCOX6A1             533 CCTCG----- -GTTTCTCGG CTGCTGGGTC GGTCCCGCCC ACAGCTGGGG
mCOX6A1             555 CAGCGTCTCG GGTCTCTCGG CCGCTGGGCC GGGCCCTCCC CGGGTTGAGG
rCOX6A1             533 GTGCGTCTCG AGTCTCTGGG CTGCTGGGCC GGGCCCTCCC ACGGGTGGGG
                        ********** ********** ********** ********** **********
                        ********** ********** ********** ********** **********
                        ********** ********** ********** ********** **********
                                    ****** ** ********** ********** **********
                                       *** ** ********** ********** **   *****
                                                                       *****
                                                                       *****

alignment position      701....... 711....... 721....... 731....... 741.......
hCOX6A1             577 CGGCCTATGT CGAGTGGCGC CCATGGCGAA GAGGGCTCAG GTACTGGGG-
mCOX6A1             605 CGGCCCATGT CGAGTGGCGC CCACGGCGAG GAGGGTTCAG GTACCcgggg
rCOX6A1             583 CGGCCCATGT CGAGTGGCGC CCACGGCGAG GAGGGTTCAG GTACCGGGG-
                        ********** ********** ********** ********** *********
                        ********** ********** ********** ********** *****
                        ********** ********** ********** ********** *****
                        ********** ********** ********** ********** *****
                        ********** ********** ********** ********** *****
                        **** ***** ********** ********** ********** ****
                        **** ***** ********** ********** ********** ****
                             ***** ********** ********** ********** ****
                              **** ********** ********** *****
                              **** ********** ********** *****

alignment position      751....... 761....... 771....... 781....... 791.......
hCOX6A1             626 ---CCGGGGT CGACGGGtcg agcctcagcc ccactcgggc GAGACAGGGA
mCOX6A1             655 gctCTCGGAT GGGCGTGGGG tctcggcggg tggcgtgcg- GCGGCGTGGC
rCOX6A1             632 ---CTCGGGT GGGCGTGGGG gcctcagtgg ctggcac--- GCGGCCTGGC
                           ******* **********                       **********
                           ******* *******                                 ***
                                                                          ***
                                                                          ***

alignment position      801....... 811....... 821....... 831....... 841.......
```

```
hCOX6A1     673 GGGACTGTGA CCTTGGCCCG AGGCCTT-GC GGGAGGGAaa gtgagacccg
mCOX6A1     704 GGGGCCGGAG CCCGGGGCGG TGACCTTgGC GGGAGGGAGC GGCCGAGGCT
rCOX6A1     676 GGGGCCGTGA CCTTGGCGGG AGGCCTT-GC GGGAGGGAGC GGCCGAGGCT
                ********** ********** *******  ** ********** **********
                ********** ********** *******  ** ********** **********
                ********** ********** *******  ** ********** **********
                ********** ********** *******  ** ********
                                              ** ********
                                              ** ********

alignment position 851.......  861.......  871.......  881.......  891.......
hCOX6A1     722 ggcccgcccc ataccggcgc tgaacgtttg TGGCTTC-TC CGCAGCTCGC
mCOX6A1     754 GACGTTG--- ---------- ---------- TGGCCCCcGC CGCAGCTCGG
rCOX6A1     725 GACATTC--- ---------- ---------- TGGCCCC-GC TGCAGCTCGT
                *******                          *******  ** **********
                *******                          *******  ** **********
                *******                          *******  ** **********
                                                 *******  ** **********
                                                 *******   * **********
                                                           * **********

alignment position 901.......  911.......  921.......  931.......  941.......
hCOX6A1     771 ATGTGGAAGA CTCTCACCTT CTTCGTCGCG CTCCCCGGGG TGGCAGTCAG
mCOX6A1     781 ATGTGGAAGG CCCTCACCTA CTTCGTGGCG CTGCCCGGGG TGGGAGTGAG
rCOX6A1     751 ATTTGGAAGG CCCTCACCTA CTTCGTGGCG CTGCCCGGGG TGGGAGTGAG
                ********** ********** ********** ********** **********
                ********** ********** ********** ********** **********
                ********** ********** ********** ********** **********
                ********** ********** ********** ********** **********
                ********** ********** ********** ********** **********
                ********** *********  ********** ********** **********
                                      ********** ********** **********
                                        ****  *** ********** **********
                                        ****  *** ********** **********

alignment position 951.......  961.......  971.......  981.......  991.......
hCOX6A1     821 CATGCTGAAT GTGTaCCTGA AGTCGCACCA CGGAGAGCAC GAGAGACCCG
mCOX6A1     831 CATGCTCAAC GTGTTCCTCA AGTCGCGGCA CGAAGAGCAC GAGAGACCCC
rCOX6A1     801 CATGCTCAAC GTtTTCCTGA AGTCGCGACA CGAAGAGCAC GAGAGACCCG
                ********** ********** ********** ********** **********
                ********** ********** ********** ********** **********
                ********** ** ******* ********** ********** **********
                ********** ** * ***** ********** ********** **********
                ********** ** * ***** ********** ********** **********
                ********** **   ***** ********** ********** **********
                ********** **   ***** ********** ********** *********
                ********        ***** ********** ********** *********
                ********

alignment position 1001......  1011......  1021......  1031......  1041......
hCOX6A1     871 AGTTCaTCGC CTACCCCCAT CTCCGCATCA GGACCAAGGT ACGCCCTtgt
mCOX6A1     881 CGTTCGTTGC CTACCCTCAC CTGCGCATCA GGACCAAGGT AGGCGCTGCG
rCOX6A1     851 AGTTCGTCGC CTACCCCCAT CTCCGCATCA GGACTAAGGT ACGCGCTGCG
                ********** ********** ********** ********** **********
                ********** ********** ********** ********** **********
                ***** **** ********** ********** ********** *******
                ***** **** ********** ********** ********** *******
                ***** **** ********** ********** ********** ****
                ***** **** ********** ********** ********** ****
                 ****   ** ********** ********** **** ***** ****
                        ** ********** ********** **** ***** ****

alignment position 1051......  1061......  1071......  1081......  1091......
hCOX6A1     921 acatct---- -CTTCAAGCG ---------- ------TCCG TTctcttttc
mCOX6A1     931 CCTTTACTGT CCGTCtgtcc gtccgtcctc caggcaTCCG TTATTGCGGG
rCOX6A1     901 CCTCTGCTGT CCTTCAAGCG ---------- ------TCCG TTATTTCTGG
                ********** **********                  **** **********
                ********** *****                       **** **********
                            ****                       **** **********
```

```
                                                                    **** **
alignment position 1101...... 1111...... 1121...... 1131...... 1141......
hCOX6A1        950 gttatgtgtg ccttagtgca agttcttcat tctctgaagg catgggtgcc
mCOX6A1        981 GTTGTCCACT CCCCTGGCCC AGCCCTtggg ggagtttTAG GATCTTGG--
rCOX6A1        935 GTTGTCCACT CCCCAGTCCC AGCCCTtggt gcgc---TAG GATCTCGG--
                   ********** ********** ******            *** ********
                   ********** ********** ******
                   ********** ********** ******

alignment position 1151...... 1161...... 1171...... 1181...... 1191......
hCOX6A1       1000 aggcgtgtaC AGCTTGTTTA TCCTCACAAA CAGAAAATGT ATTTTCTTC-
mCOX6A1       1029 ---------C TGCCTGTCCG TCCGTGCAGA TAGAATATTT TATTTACTCT
rCOX6A1        980 ---------C TGCCTGTCTA TCCGCGCAGA CTGAACATTT TATTTACTCT
                            * ********** ********** ********** **********
                            * ********   ********** ********** **********

alignment position 1201...... 1211...... 1221...... 1231...... 1241......
hCOX6A1       1049 ---------- ---------- CATTTTGTGG atggacagct gacacttggg
mCOX6A1       1070 tCGTTTGACG GAAGGGAAGC CAGTTTGTAG GTCGTGGCTc gg--------
rCOX6A1       1021 -CGTTTTACA TGAGGGAAGC CAGTTTGTGG GTCGCGGCT- ----------
                    ********* ********** ********** *********
                    ********* ********** ********** *********

alignment position 1251...... 1261...... 1271...... 1281..
hCOX6A1       1079 attacgtctc aattctcttc ttcaaggtca tacaat
mCOX6A1       1112 ---------- ---------- ---------- ------
rCOX6A1       1059 ---------- ---------- ---------- ------
```

Pairwise similarities:

For each pairwise alignment, the similarity (relative to the maximum similarity) and the number of identical nucleic acids (in % of shorter sequence) is given. Maximum values are underlined.

	mCOX6A1 (1111 bp)	rCOX6A1 (1058 bp)
**hCOX6A1 (1114 bp)**	0.293<br>37 %	0.371<br>47 %
**mCOX6A1 (1111 bp)**		1.000<br>80 %

Please note that the **similarity value 1.000** marks only the two **most similar** sequences, it does not necessarily mean that these sequences are identical.

**Extract alignment region**	
**Positions in alignment**	from: to:
**Output file**	dialign.seq

licensed users only Extract aligned sequences

For comments, questions or bug reports, please contact support@genomatix.de

---

© Genomatix Software GmbH 1998-2005 - All rights reserved. License Agreement GEMS Launcher 4.1.1

Genomatix-Logo Logout Personal Messages GEMS-Logo

Genomatix_MainGEMSElDoradoGene2PromoterBiblioSphere
Frequently_asked_questionsYour_ResultsYour_SequencesYour_ProtocolHelp

GEMS Launcher Task: *DiAlign TF*: Multiple alignment plus TF sites
working on C4-COX6B1-HMR (3 seq.)

[Alignment] [Pairwise_similarities]

DiAlign professional TF Release 3.1 December 2004 **Fri May 6 00:46:04 2005**

Solution parameters:

**Sequence file:**	C4-COX6B1-HMR (3 seq.)
**Type of sequences:**	nucleotide sequences
**Threshold T:**	0.00
**'*' signs below alignment denote**:	diagonal similarity (max. similarity is represented by 10 '*' signs)
**Alignment output:**	complete alignment is shown<br># nucleic acids per line: 50
**TF output:**	common TF matches located in aligned regions (common to 3 (100.0 %) sequences)
**Family matches:**	yes
**MatInspector library:**	Matrix Family Library Version 5.0 (February 2005)
**Selected groups** (core/matrix sim):	• ALL user_defined.lib (0.75/Optimized)<br>• ALL vertebrates.lib (0.75/Optimized)

Aligned Sequences:

No.	Sequence Name	Sequence Description
1	P875609	P875609 sym=COX6B1|loc=Loc1340|taxid=9606|spec=Homo sapiens|chr=19|ctg=NT_011109|str=(+)|start=8406873|end=8407773|len=901|tss=501|comm=cytochrome c oxidase subunit Vib polypeptide 1 (ubiquitous); (NM_001863/501/bronze;)
2	P875610	P875610 sym=Cox6b|loc=Loc110323|taxid=10090|spec=Mus musculus|chr=7|ctg=NT_039413|str=(-)|start=3670224|end=3671190|len=967|tss=501,538,567|comm=cytochrom c oxidase, subunit VIb; (AK012686/538/gold;AK018721/567/gold;NM_025628/501/bronze;)
3	rCOX6B1	rCOX6B1 rn3_dna range=chr1_random:1613878-1614820 5'pad=0 3'pad=0 revComp=TRUE strand=? repeatMasking=none

Alignment (DiAlign format):

Please note that only **upper-case** letters are considered to be **aligned**. For more information, please have a look at the user guide.

V$ECAT V$ETSF U$NRF2 V$EREF U$EREF V$CREB V$EBOR

```
alignment position  1 . . . . . . . . .  1 1 . . . . . . . .  2 1 . . . . . . . .  3 1 . . . . . . . .  4 1 . . . . . . . .
P875609          1  a - - - - - - - - -  - - - - - - - - - -  - - - - - - - - - -  - - - - - - - - AG  AC C CC A T C T C
P875610          1  c a a a c a a a c a  a a c a a a c a a a  c a a a c a AAG A  GTT G C C GT AG  AC A T G A T GT C
rCOX6B1          1  - - - - - - - - - -  - - - - - - - - - -  - - - - - - - AAG A  GTT G C C GT GG  T C A T G T T GT C
```

```
                                                       **** ********** **********
                                                       **** ********** **********
                                                       **** ********** **********
                                                       **** ********** **********
alignment position 51........ 61........ 71........ 81........ 91........
P875609        14 TATAAAATGC Atatatat-- ---------- ---------- ----------
P875610        51 TCTTCACTGC AATAAAACAC AAACTAAGAT TGTCTATCCC GAAAACCCAA
rCOX6B1        25 TCTTCACAGC AATAAAACAC AAACTAAGAT ACCTTATCTC GAAAATCCAA
                  ********** ********** ********** ********** **********
                  ********** ********** ********** ********** **********
                  ********** ********** ********** ********** **********
                  ********** ******
alignment position 101....... 111....... 121....... 131....... 141.......
P875609        32 ---------- ---------- -----ATCCA GGAGTAGCGG TGCGCATCTG
P875610       101 ATAAAtAAAT ATAAATAACT TTCGAATCAA GGCACAGGGG TAAACGCTTG
rCOX6B1        75 ATAAAcAAAC ATAAATAACT TTTGAATCAA AGCATAGGGG TAAAAGCTTG
                  ***** **** ********** ********** ********** **********
                  ***** **** ********** ********** ********** **** *****
                  ***** **** ********** ********** ********** **** *****
                        **** ********** ********** ********** ****
alignment position 151....... 161....... 171....... 181....... 191.......
P875609        57 TAATCCCAGC TACTcgggag gatgaggcgg gagaatcgct tgaacccagg
P875610       151 TTATCCTAAC ACCT------ ---------- ---------- ----------
rCOX6B1       125 TTATCCTAGC ACTC------ ---------- ---------- ----------
                  ********** ****
                  ********** ****
                  ********** ****
alignment position 201....... 211....... 221....... 231....... 241.......
P875609       107 aggcggaggt tgcAGTGAGC TGAGATCgcg ctactgcact CCAGCCTGGG
P875610       165 ---------- ---AGTAATC TGTGATCAAA At-TTCGAGG CCAGTCTGAG
rCOX6B1       139 ---------- ---AGGAATC TGTGATCAAA AagTTCGAGG CCAGTCTGTG
                                ******* ********** *  ******* **********
                                ******* ********** *  ******* **********
                                ******* ********** *  ******* **********
                                                              **********
alignment position 251....... 261....... 271....... 281....... 291.......
P875609       157 CCATAGAGTA AGACCCTGTC TCAAAAattt aaaaaaaaga aaaaaaaaat
P875610       201 ATATACAGCG AAGTCCTATT TCAAAAcaCA GATTAT---- ----------
rCOX6B1       176 CTGTATAGCA AAGTCCTGTC TCAAAA--CA GATTAT---- ----------
                  ********** ********** ******  ** ******
                  ********** ********** ******
                  ********** ********** ***
                  ********** ********** ***
alignment position 301....... 311....... 321....... 331....... 341.......
P875609       207 ttaaagaaaa gaaatacctg acacagaaaa agcgttcaat aaatatttct
P875610       237 ---------- ---------- ---------- ---------- ----------
rCOX6B1       210 ---------- ---------- ---------- ---------- ----------

alignment position 351....... 361....... 371....... 381....... 391.......
P875609       257 tgcatgaata aaagtacttt cgCTGGGACA TTTTTCTCCA CACCTACCCT
P875610       237 ---------- ---------- --CTGTGACA CTTTTC--CA TTCCCACTCT
rCOX6B1       210 ---------- ---------- --CTGgt--A TATTTTCCCA TTCCCACTCT
                                          ******** ********** **********
                                                           ** **********
alignment position 401....... 411....... 421....... 431....... 441.......
P875609       307 CCAGCCAGCC CCgggatcta taacactctc tccgagtgAA GACACAGAGG
P875610       263 GAAGACAGTC Ttatg----C TTTTCCCTCT GGGA----AG GACAGGCAGG
rCOX6B1       236 GCAGACAGCC TCactctgtC TTTTTCATCT GGGA----AG GACTGGCAGG
```

```
                      ********** **       * ********** ****    ** **********
                      ********** *        * ********** ****    ** **********
                                          * ********** ****    ** **********
                                          * ********** ****    ** **********

alignment position    451....... 461....... 471....... 481....... 491.......
P875609          357  TGATGAGATC GGGTAAccca cgggcgcccg agcccc---A GCCGCGCAAG
P875610          305  TGATAATAAC CCATAAtTGA ACGTCAGGTG CCTTTTAAGA GCAAAGTAAA
rCOX6B1          282  TGACAATAGC CCAtagcTGA ACGTCAGGAG CCTTTTAAGA GCAAAGCAAA
                      ********** ****** *** ********** ********** **********
                      ********** ***    *** ********** ********** **********
                      ********** ***    *** ********** ********** **********
                      ********** ***    *** ********** ********** **********

alignment position    501....... 511....... 521....... 531....... 541.......
P875609          404  CTCACGATTG GGCCAGAAGT GAGGATGAAC AAGCATTTAG CCCAATAGAA
P875610          355  ATCTCGATTG GCCCAGCAAC AATAATAAGC AGAAATTTAG CTCAATAGGA
rCOX6B1          332  CTGCTGATTG GACCAGCAAC AATAATAAAC AGGAATTTAG CTCAATAGGA
                      ********** ********** ********** ********** **********
                           ***** ********** ********** ********** **********
                           ***** ********** ********** ********** **********
                           ***** ********** ********** ********** **********
                           ***** ********** ********** ********** **********
                                                ****** ********** **********

alignment position    551....... 561....... 571....... 581....... 591.......
P875609          454  AGTCGTAGTT Ctcttgc--T GGGCGTGGCT TGAATGACTT CAGTGGCCTC
P875610          405  GGTCGGGCTT CTTCGGAAGT GGGCGTGGCC AAAGCGGTTT CTGGGCACGC
rCOX6B1          382  GGTCGGGCCT CTTTGGAAGT GGGAGTGGCC AGAGCGACTT CTGGGCACGC
                      ********** ********** ********** ********** **********
                      ********** ********** ********** ********** **********
                      ********** ********** ********** *****   ** **********
                      ********** ********** ********** *****   ** **********
                      ********** *        * ********** *****   ** **********
                      *****

alignment position    601....... 611....... 621....... 631....... 641.......
P875609          502  CTCCTGGGag ggagCTGAAG CCGCTCGCAA GACTCCCGTA GTCCCCACCT
P875610          455  CTCCCGAGGC CA--ATCTAG CAGCCCGGAG GACTCAGCTC GGCTCCTCCT
rCOX6B1          432  CTCCCGAAGC CA--CTCTAG CAGCTCGGAG GACTCAATTC GGCTTCTCCT
                      ********** **  ****** ********** ********** **********
                      ********** **  ****** **** ***** ********** **********
                      ********** **  ****** **** ***** ********** **********
                      ********** **  ****** **** ***** ********** **********
                      ****                                            ******
                                                                      ******
                                                                      ******

alignment position    651....... 661....... 671....... 681....... 691.......
P875609          552  CTCTCAGCTT CCGGCTGGTA GTAGTTCCGC TTCCTGTCCG ACTGTGGTGT
P875610          503  CTCGCAGCTT CCGGCCAGTC GGAATTCCGC TTCCCGTCTG ACATTGGCGT
rCOX6B1          480  CTTGCAGCTT CCGGCCAGTC GGAATTCCGC TTCCCGTCTG ACATTGGCGT
                      ********** ********** ********** ********** **********
                      ********** ********** ********** ********** **********
                      ********** ********** ********** ********** **********
                      ********** ********** ********** ********** **********
                      ********** ********** ********** ********** **********
                      ********** ********** ********** ********** **********
                      **  ****** ********** ********** ********** **********
                          ****** ********** ********** ********** **********
                                      ***** ********** **** ***** **  ******
                                      ***** ********** **** ***** **  ******

alignment position    701....... 711....... 721....... 731....... 741.......
P875609          602  CTTTGCTGAG GGTCACATTG Agctgcaggt tgaatccggg gtgcCTTTAG
P875610          553  CTTTGCTAAG GGTCACATTG AAGTAACGCT ACTCCGGGAC AaT-CTTTAG
rCOX6B1          530  CTTTGCAAAG GGTCACATTG AAGTAACGCT GCTCCGGGAC AgT-CTTTAG
```

```
                      ********** ********** ********** ********** * * ******
                      ********** ********** ********** ********** * * ******
                      ********** ********** ********** ********** * * ******
                      ********** ********** ********** ********** * * ******
                      ********** ********** *                         ******
                      *****  *** ********** *                         ******
                      *****  *** ********** *                         ******
                      *****  *** ********** *
                      *****
                      *****

alignment position    751....... 761....... 771....... 781....... 791.......
P875609          652  GTGAGTGTGG AGGGTtCTGT AACCTGGGAC -CCCAGTCTA GCGGGCTGAG
P875610          602  GTGAGTGTGA AGGGT-CGGG ATCCTGGGGG -AACCTTGTC CGTTGCAGAG
rCOX6B1          579  GTGAGTGTAA AGGGT-CAGG ATCCTGGGGG gAACCTTGTC TGTGGTAGAG
                      ********** ***** **** **********  ********* **********
                      ********** ***** **** **********  ********* **********
                      ********** ***** **** **********  ********* **********
                      ********** ***** **** **********  ********* **********
                      ********** *****
                      ********** *****
                      ********** *****

alignment position    801....... 811....... 821....... 831....... 841.......
P875609          701  GAGCcggacc ccaGCTTCCC TGAGAACGGG TTGAGGCTTC CGGCTGGCgg
P875610          650  AAGCGCCCCG GG-GCTTCCC TGACGCCAAG CAGAAACTTC CGGCGGCCAC
rCOX6B1          628  AAGCGCCCCG GG-GCTTCCC TGAAGCCGGG AAGAAACTTC CGGCTACCCT
                      ********** ** ******* ********** ********** **********
                      ********** ** ******* ********** ********** ********
                      ********** ** ******* ********** ********** *****
                      ********** ** ******* **
                                    ******* **
                                    ******* **

alignment position    851....... 861....... 871....... 881....... 891.......
P875609          751  cgtccggcct cccctggacc CGGGGCCCTG ttctgttcat cgtaaGAGCT
P875610          699  GGGTc----- ---------- CGGTGTCCTG ---------- -----GAGCT
rCOX6B1          677  GGGCg----- ---------- CGGGGTCCTG ---------- -----GAGCT
                      ****                  **********                 *****
                                            **********                 *****
                                            **********                 *****
                                                                       *****

alignment position    901....... 911....... 921....... 931....... 941.......
P875609          801  AACAATGATA CGGTTCTTCC TTGATGC--- -CAGGCAACA TCC-CAAACA
P875610          719  AACCATTATA GCCGCCTTTC TTCATGAtgg cTAGGTCGCT TtctAAGCAG
rCOX6B1          697  CACCATTATA TCCGCCTTCA T-GATGT--- -TAGGTCGCT TGA-AAGCAG
                      ********** ********** *******     ********* *** ******
                      ********** ********
                      ********** ********
                      ********** ********

alignment position    951....... 961....... 971....... 981....... 991.......
P875609          846  ACTTTAATTA ATTTATTTaa ctctcccaCC AGCCCTATGA GATAGGGCCT
P875610          769  TCcc-TATTA CTCCATCCT- --------TC AGCCCTAcaa g---------
rCOX6B1          741  TCTCTTATTA CTCCATTCT- --------CC AGCCCTATGA ACTAGGACCT
                      ********** *********          ** ********** **********
                           ***** *********          ** ********** **********
                                                    ** *******

alignment position    1001...... 1011...... 1021...... 1031...... 1041......
P875609          896  GCGATT---- ---------- ---------- ---------- ----------
P875610          800  -------TCC CCTTTGATAG CGTTGGGGCT GGAGTGTGAA GCGCCTTacc
rCOX6B1          782  GCCATTaTCC CCTTTAACAG AGCTGGGGCT GGAGTGTGAA GCGCCTTagc
                      ****** *** ********** ********** ********** *******
                      ****** *** ********** ********** ********** *******
                             *** ********** ********** ********** *******
                             *** ********** ********** ********** *******

alignment position    1051...... 1061...... 1071...... 1081...... 1091......
```

```
P875609     902 ---------- ---------- ---------- ---------- ----------
P875610     843 cgtGGCTATA AGGCAATTCG GATTTAGACC CAGTAAATTG GACTGTGGAG
rCOX6B1     832 agcGGCTGTA TGGCAATTAG GATTTGGACC CAGTAAATTG AACTGTGGTG
                   ******* ********** ********** ********** **********
                   ******* ********** ********** ********** **********
                   ******* ********** ********** ********** **********
                   ******* ********** ********** ********** **********

alignment position 1101...... 1111...... 1121...... 1131...... 1141......
P875609     902 ---------- ---------- ---------- ---------- ----------
P875610     893 CTTTTAGTTG CGAGTTCACA GGCGTGGCCA CCCgcgttcc AAGGCCGAAG
rCOX6B1     882 CTTTTGGTCG CGGGCTCACA GGCGTGGCCA CCCg------ AAGGCCGAAG
                ********** ********** ********** ***        **********
                ********** ********** ********** ***        **********
                ********** ********** ********** ***        **********
                ********** ********** ********** ***

alignment position 1151...... 1161...... 1171.
P875609     902 ---------- ---------- -----
P875610     943 CCTGTCGCCT TGACTCGTgt ctttc
rCOX6B1     926 TCTGTCGCCT TGACTCGT-- -----
                ********** ********
                ********** ********
                ********** ********
```

Pairwise similarities:

For each pairwise alignment, the similarity (relative to the maximum similarity) and the number of identical nucleic acids (in % of shorter sequence) is given. Maximum values are underlined.

	P875610 (967 bp)	rCOX6B1 (943 bp)
P875609 (901 bp)	0.126 37 %	0.167 36 %
P875610 (967 bp)		1.000 82 %

Please note that the **similarity value 1.000** marks only the two **most similar** sequences, it does not necessarily mean that these sequences are identical.

Extract alignment region	
**Positions in alignment**	from: to:
**Output file**	dialign.seq

Extract aligned sequences

For comments, questions or bug reports, please contact support@genomatix.de

© Genomatix Software GmbH 1998-2005 - All rights reserved. License Agreement GEMS Launcher 4.1.1

Genomatix-Logo Logout Personal Messages GEMS-Logo

Genomatix_MainGEMSElDoradoGene2PromoterBiblioSphere
Frequently_asked_questionsYour_ResultsYour_SequencesYour_ProtocolHelp

GEMS Launcher Task: *DiAlign TF*: Multiple alignment plus TF sites
working on C4-COX7C (3 seq.)

[Alignment] [Pairwise_similarities]

DiAlign professional TF Release 3.1 December 2004 **Wed May 11 16:45:21 2005**

Solution parameters:

**Sequence file:**	C4-COX7C (3 seq.)
**Type of sequences:**	nucleotide sequences
**Threshold T:**	0.00
**'*' signs below alignment denote:**	diagonal similarity (max. similarity is represented by 10 '*' signs)
**Alignment output:**	complete alignment is shown<br># nucleic acids per line: 50
**TF output:**	common TF matches located in aligned regions (common to 3 (100.0 %) sequences)
**Family matches:**	yes
**MatInspector library:**	Matrix Family Library Version 5.0 (February 2005)
**Selected groups** (core/matrix sim):	• ALL user_defined.lib (0.75/Optimized)<br>• ALL vertebrates.lib (0.75/Optimized)

Aligned Sequences:

No.	Sequence Name	Sequence Description
1	P985294	P985294 sym=COX7C|loc=Loc1350|taxid=9606|spec=Homo sapiens|chr=5|ctg=NT_006713|str=(+)|start=36507518|end=36508642|len=1125|tss=601,625|comm=cytochrome c oxidase subunit VIIc; (AK026505/501/gold;NM_001867/525/bronze;)
2	P985295	P985295 sym=Cox7c|loc=Loc12867|taxid=10090|spec=Mus musculus|chr=13|ctg=NT_039589|str=(-)|start=9490489|end=9491609|len=1121|tss=601,606,621|comm=cytochrc c oxidase, subunit VIIc; (AK002494/526/gold;AK027989/531/gold;NM_007749/546/bronze;)
3	rCOX7C	rCOX7C rn3_dna range=chr2:15175947-15176956 5'pad=0 3'pad=0 revComp=TRUE strand=? repeatMasking=non

Alignment (DiAlign format):

Please note that only **upper-case** letters are considered to be **aligned**. For more information, please have a look at the user guide.

V$MYOF V$STAT V$RBPF V$STAF V$GKLF V$RORA V$ETSF V$PLAG

```
alignment position 1 . . . . . . . . .  11 . . . . . . . .  21 . . . . . . . .  31 . . . . . . . .  41 . . . . . . . .
P985294          1 t t c a t c a g g c  g g a c a c c a g t  t c c t a g t c c a  g g c t a g c t c c  a g g t G T C A G A
P985295          1 a a - - - - - - - -  - - - - - - - - - -  - - - - - - - - - -  - - - - - - - - - -  - - - - G T C A G A
rCOX7C           1 - - - - - - - - - -  - - - - - - - - - -  - - - - - - - - - -  - - - - - - - - - -  - - - - G T C A G A
                                                                                                                 * * * * * *
                                                                                                                 * * * * * *
                                                                                                                 * * * * * *
```

```
alignment position 51........ 61........ 71........ 81........ 91........
P985294         51 GGCCACTTTT CCCTTCTACT CCTCACACAT GCTGtactca taccagactt
P985295          9 AGGTTCTTGT GTCTTCTCCA ACCTAATCAT GCTGcgcata ggagtttcTA
rCOX7C           7 AGGTACTCAT CTCTTCCATA ATCTAATCAT GCTtcacata tcgtt---TA
                   ********** ********** ********** ****              **
                   ********** ********** ********** ***               **
                   ********** ********** ********** ***

alignment position 101....... 111....... 121....... 131....... 141.......
P985294        101 gagttttccc tgccctttgt tcttcctTCT TTTTTATTCG ATTCCCATTA
P985295         59 TC-------- ---------- -------TGT ATGTCCTCTT ATATCCAATT
rCOX7C          54 TC-------- ---------- -------TGT ATGTTCTTAA ATATCCATTT
                   **                           *** ********** **********
                   **                           *** ********** **********

alignment position 151....... 161....... 171....... 181....... 191.......
P985294        151 TCTTTAGTg- -------ATC ATTCCCTTAG CATTTCAGTt ttctctctga
P985295         84 TCTATACCTT CAAaaaaa-C CAGGTCTTAG CACTTCAGTC CAGTTTTCAC
rCOX7C          79 TCTGTATTTT CAAaaacATC CAGCTCTTGG CACTTCAGTC CAGTTTTCAC
                   ********** ***    *** ********** ********** **********
                   ********** ***      * ********** ********** **********
                                       * ********** ********** **********
                                       * ********** ********** **********

alignment position 201....... 211....... 221....... 231....... 241.......
P985294        193 ag-----TCC TTTGTGCTGC ACGTTGTTTG ATTACTTGtg tttatgcTTT
P985295        133 TACGGCATCC TTTGTGCTAC ATACTGTTTG ATCAGTTGCT -------TGT
rCOX7C         129 CATGGCATCc -TTGTGCTAC ATACTGTTTG ATTAGTTGCC -------TGT
                   ********** ********** ********** **********        ***
                   ********** ********** ********** **********        ***
                   *********   ********* ********** **********        ***
                   *********   ********* ********** **********        ***
                          **   ********* ********** ********
                               ********* ********** ********

alignment position 251....... 261....... 271....... 281....... 291.......
P985294        238 GTTTCTTCAG CTAGGATCAT TAgcaCATAT CTCTCAGCAT TGGCAAAAgt
P985295        176 GTTCCCTCAG ACAGGACTAT AATG-CACAT CTTTCAACCT TGGCAAAGAA
rCOX7C         171 GTTCCCctc- ---GGAATAG AATG-CACAT CTTTCAATGT TGGCAAAAAA
                   ********** ********** **** ***** ********** **********
                   ******        ******* **** ***** *******  * **********
                   ******                     ***** *******  * **********
                   ******                                    * *******

alignment position 301....... 311....... 321....... 331....... 341.......
P985294        288 cc----TCGA AATCATTGAC ACCT------ AGCATATACT TTATTTTTaa
P985295        225 CCATAATCAA AATGACTGAT Atactcacaa GAAATAATTT TTATTTCTTG
rCOX7C         216 TCATAATCAA AATGAGTGAT ACAT------ GAAATAATTT TTATTTCTTG
                   ********** ********** ****       ********** **********
                   ********** ********** *          ********** **********
                   ********** ********** *          ********** **********
                                                    ********** **********
                                                    ********** **********

alignment position 351....... 361....... 371....... 381....... 391.......
P985294        328 aaaagaaaga aagaagagaa aagggatggg tttattgtcc ttTTCAACAG
P985295        275 ---------- ---------- ---------- ---------- --TTCCATTA
rCOX7C         260 ---------- ---------- ---------- ---------- --TTCCATAA
                                                                 ********
                                                                 ********
                                                                 ********
                                                                 ********
                                                                 ********

alignment position 401....... 411....... 421....... 431....... 441.......
P985294        378 ACTAGAGTAT ACGGGGTGAA ACTGCTTCAC TTGATTCAAT aAAATCGTTT
P985295        283 ACTTGAACAC ACATGTTAAA GCAGCTTTAG TTTCTTCAAT -AAATTGTTT
rCOX7C         268 ACTTGAACAC ACACGGTTAA ACAACTTTAG TTACCTCAAT -AAGTTGATT
```

```
                          **********  **********  **********  **********   **********
                          **********  **                **********  **********   **********
                          **********  **                **********  **********   **********
                          **********  **                                        **********
                          **********  **                                        **********
                          **********  **

alignment position 451.......  461.......  471.......  481.......  491.......
P985294      428 CCGGTAACAG  GCCCCAGGAA  TCCTAGACCT  aagc------  ----------
P985295      332 CTGGAAACAA  ATCCCAAGAA  TCCTAGATCT  GAACTCATAG  GACAGCTCAG
rCOX7C       317 ACGGAAACAA  ATCCCAAGAA  TCCGAGATCT  GAACTCAGAA  AACAGCTCAG
                 **********  **********  **********  **********  **********
                 **********  **********  **********  **********  **********
                 **********  **********  **********  **********  **********
                 **********  **********  **********  **********  **********
                 **********  **********  **********
                                ********  **********

alignment position 501.......  511.......  521.......  531.......  541.......
P985294      462 ----------  ----------  ----------  ----------  ----------
P985295      382 GTcccattga  atcactaaag  gtaAGTTATC  ACTTGGTGCT  GGTGCGCGCT
rCOX7C       367 GTtctaaaca  cagatgataa  gctAGCTATC  ACTTGGTGGG  GGAGCGCGCT
                 **                          *******  **********  **********
                 **                          *******  **********  **********
                 **
                 **

alignment position 551.......  561.......  571.......  581.......  591.......
P985294      462 ----CTGGCG  CGAAACTACA  TTTCCCACAA  TCCTTCGGgg  gctgataagg
P985295      432 CATTCTAGAG  CCAGACTACA  TTTCCCACAA  TCCATCGGAG  CCGATATGGc
rCOX7C       417 CATT------  -CAGACTACA  TTTCCCAAAA  TCCACCGGAA  CCGATATGGg
                 **********  **********  **********  **********  *********
                 **********  **********  **********  **********  *********
                               *********  **********  **********  *********
                               *********  **********  **********  *********
                               *********  **********  ********
                               *********  **********  ********
                                   ******  **********  ********

alignment position 601.......  611.......  621.......  631.......  641.......
P985294      508 ctCCGCAATG  GTCTGAACTA  CAATTCCCAC  AATCCaggg c  gatttccgct
P985295      482 GCCCGGTATT  ATCTAAACTA  CAAAGCCCAC  AAACCTCAGA  GCCCGCCGGT
rCOX7C       460 GCCAGGTATT  ATCTAAACTA  CAAATCCCAC  AATCCTCGGA  GCCCGCCAGT
                 **********  **********  **********  **********  **********
                 **********  **********  **********  **********  **********
                    *******  **********  **********  *****
                               *********  ****  *****  *****
                               *********  ****  *****  *****

alignment position 651.......  661.......  671.......  681.......  691.......
P985294      558 ttgtcgCGTT  TCCTCAAGGC  TCCGCCCCAT  TTCCCATCTT  TCTTTTCAGT
P985295      532 TGAGTACGTT  TCAGGAAGTT  CCTGTTGTAA  CTCCCATCTT  CCCTTTCGGT
rCOX7C       510 TGATTGCGTc  -CAGGAAGTC  CGTGCTGTAG  TTCCCATCTT  CCCTTTCGGT
                 **********  **********  **********  **********  **********
                 *********     ********  **********  **********  **********
                               *********  **********  **********  **********
                                                 **  **********  **********
                                                                      *******

alignment position 701.......  711.......  721.......  731.......  741.......
P985294      608 CCTTGCGCAC  CGGGGAACAA  GGTCGTGAAA  AAAaggtct   TGGTGAGGTG
P985295      582 CTTCGCGCTC  TGCCGAGCAA  GGTCGTGTAG  AAAGGGAG-   Tt AGGTGGTA
rCOX7C       559 CTTCTCGTTC  CGGATAGCAA  GGTCGTGAAG  AAAGTAGGG-  TGAGGTGGTA
                 **********  **********  **********  *********   **********
                 **********  **********  **********  *********   *  ********
                 **********  **********  **********  ***            ********
                 **********  ***                                    ********
                 *                                                     ******

alignment position 751.......  761.......  771.......  781.......  791.......
```

```
P985294     658 CCGCCATTTC ATCTGTCCTC ATTCTCTGCG CCTTTCGCAG AGCTTCCAGC
P985295     631 CGGCCATTTC TTCCGCCTTC CGTGTCTGCG gcccTCGCAG AACTTCCAGC
rCOX7C      608 CGGTCATTTC TTCTGCCTTC CGTCTCTGCG ACTCTCGGAG AACTTCCAGC
                ********** ********** ********** ********** **********
                ********** ********** ********** ********** **********
                ********** ********** **********    ******* **********
                ********** ********** **********    ******* **********
                ********** **********   ********    ******* **********
                                        ********     ****** **********
                                                     ****** **********
                                                     ****** **********

alignment position 801.......  811.......  821.......  831.......  841.......
P985294     708 AGCGGTATGT TGGGCCAGAG CATCCGGAGG TTCACAACCT CTGGTGGTCCG
P985295     681 AGCGACATGT TGGGCCAGAG TATCCGGAGG TTCACGACCT CCGTGGTCCG
rCOX7C      658 AGCAGCATGT TGGGCCAGAG TATCCGGAGG TTCACCACCT CCGTGGTGCG
                ********** ********** ********** ********** **********
                ********** ********** ********** ********** **********
                ********** ********** ********** ********** **********
                ********** ********** ********** ********** **********
                ********** ********** ********** ********** **********
                ********** ********** ********** ********** **********
                ********** ********** ********** ********** **********
                ***   **** ********** ********** ********** ******  **
                      **** **********  ********* ********** ******  **
                      **** **********  ********* ********** ******  **

alignment position 851.......  861.......  871.......  881.......  891.......
P985294     758 TAGGAGCCAC TATGAGGAGG GCCCTGGGAA GGTTAGTGtg taaggGGCAC
P985295     731 TCGCAGCCAC TATGAGGAGG GTCCGGGGAA GGTGAGTGA- -----GGCTC
rCOX7C      708 TCGCAGCCAC TATGAGGAGG GTCCGGGGAA GGTGAGTGA- -----GGCCT
                ********** ********** ********** *********       *****
                ********** ********** ********** *********       *****
                ********** ********** ********** *********       *****
                ********** ********** ********** *********       *****
                ********** ********** ********** *********       *****
                ********** ********** ********** ********
                ********** ********** ********** ********
                *   ****** ********** ********** ********
                *   ****** ********** ********** ********
                *   ****** ********** ********** ********

alignment position 901.......  911.......  921.......  931.......  941.......
P985294     808 GGCTTCGTTG GGGGAGGGGG cgcttggctg tgactCGCGC ACCTGCAAGG
P985295     775 GGCTGCCTAG GGGCAAGGTG acgctcggc- ---------- -------GGC
rCOX7C      752 GGCTGCtgag aatcgccacc ---------- -----CGCCT ATCTCAGGGC
                ********** **********                 ***** **********
                ******                                             ***
                ******                                             ***
                ******                                             ***
                ******

alignment position 951.......  961.......  971.......  981.......  991.......
P985294     858 CCGCCTCCGG GCTGTGGCGT GGGAGATGAt agccagaaaC CAGGCTGAGA
P985295     807 CGGCCTGGAG CGTGTGGGGA GGGAAGTGAC TGT------C CAGGAGCTCG
rCOX7C      787 CGGCCTAGAG CGTGTGGGGA GGGAAGTGCC TAG------C CAGcc-----
                ********** ********** ********** ***      * **********
                ********** ********** ********** ***      * ***
                ********** ********** ********** ***      * ***
                ********** ********** ********** ***      * ***

alignment position 1001......  1011......  1021......  1031......  1041......
P985294     908 CGCAGACTAG CATTCCACTT AGCCCAagga ccagtGAGGA AGCTGGGCAT
P985295     851 GGGAGACACT CAGGCCCCTC AGTCCACACC CCG--GGGag AACTGGGAAG
rCOX7C      826 ----GACGCG CAGGCCCTCT GGTCCACATC CCC--GTGAA GGCTGGGAAG
                ********** ********** ********** ***  ***** **********
                                                      ***   **********
                                                              ********
                                                              ********
```

```
alignment position 1051...... 1061...... 1071...... 1081...... 1091......
P985294     958 CCTAGCGCGT AGCCGCTaaa ggaatgggca ggtagatccg gaAGCCCTGC
P985295     899 CACGGCGCCT AGCCGCTGCC TC-------- ---------- --CACGCCGC
rCOX7C      870 CACAGCGCCT AGCTGCTGCC TC-------- ---------- --AGCACTGC
                ********** ********** **                      ********
                ********** ********** **                      ********
                ********** ********** **                      ********
                ********** *******

alignment position 1101...... 1111...... 1121...... 1131...... 1141......
P985294    1008 CTccatcagc cacctgacGC CCCCTCCCCC GCCCCGCAGA AAGCCCTGAG
P985295     929 CTgtcagccg ---------- ---------- ----CAGAGC GAGGCCTGGA
rCOX7C      900 CT-------- --------GC CTGCCACCTC TTCCCAGAGC CAGGCCTGGA
                **                 ** ********** ********** **********
                **                                   ****** **********
                **                                   ****** **********
                                                     ****** **********

alignment position 1151...... 1161...... 1171...... 1181...... 1191......
P985294    1058 ATGgctccgg gaggccacgg ctgtaggtgt gttggttaaa tccgagcTGG
P985295     955 AGGTTCGGTG GGAGCTGGGC GGCGtcggag at-------- -------TGA
rCOX7C      934 AGGTTAGGTT GGACCTGGGC GGCGtcagag atgagc---- --------GA
                ********** ********** ****                       ***
                ********** ********** ****                        **
                ********** ********** ****                        **
                ********** ********** ****                        **

alignment position 1201...... 1211...... 1221...... 1231...... 1241......
P985294    1108 AGGTCATCGG ACCCGAAA-- ---------- ---------- ----------
P985295     990 AGGTCATTGG AAACAAAAGA AGAGGGCCTG TGGCTGgtta ttagcgcttt
rCOX7C      972 AGGTCATTGG AAACAAAAGA AAAGGGCTTG AGGCTGtgg- ----------
                ********** ********** ********** ******
                ********** ********** ********** ******
                ********** ********** ********** ******
                ********** ********** ********** ******

alignment position 1251...... 1261...... 1271...... 1281...... 1291......
P985294    1126 ---------- ---------- ---------- ---------- ----------
P985295    1040 aaaccagcaa gccacgtttg aagcggcgat cctcggttag gcccggttta
rCOX7C     1011 ---------- ---------- ---------- ---------- ----------

alignment position 1301...... 1311...... 1321...... 1331
P985294    1126 ---------- ---------- ---------- --
P985295    1090 gccccccagtg taagtaaacg gagtacttag tg
rCOX7C     1011 ---------- ---------- ---------- --
```

Pairwise similarities:

For each pairwise alignment, the similarity (relative to the maximum similarity) and the number of identical nucleic acids (in % of shorter sequence) is given. Maximum values are underlined.

	P985295 (1121 bp)	rCOX7C (1010 bp)
P985294 (1125 bp)	0.272<br>45 %	0.246<br>43 %
P985295 (1121 bp)		<u>1.000</u><br><u>77 %</u>

Please note that the **similarity value 1.000** marks only the two **most similar** sequences, it does not necessarily mean that these sequences are identical.

Extract alignment region	
**Positions in alignment**	from: to:
**Output file**	dialign.seq

Extract aligned sequences

For comments, questions or bug reports, please contact support@genomatix.de

© Genomatix Software GmbH 1998-2005
- All rights reserved. License Agreement GEMS Launcher 4.1.1

GEMS Launcher Task: *DiAlign TF*: Multiple alignment plus TF sites
working on C4-COX7B (3 seq.)

[Alignment] [Pairwise_similarities]

DiAlign professional TF Release 3.1 December 2004 **Sat May 7 01:36:43 2005**

Solution parameters:

**Sequence file:**	C4-COX7B (3 seq.)
**Type of sequences:**	nucleotide sequences
**Threshold T:**	0.00
**'*' signs below alignment denote:**	diagonal similarity (max. similarity is represented by 10 '*' signs)
**Alignment output:**	complete alignment is shown<br># nucleic acids per line: 50
**TF output:**	common TF matches located in aligned regions (common to 3 (100.0 %) sequences)
**Family matches:**	yes
**MatInspector library:**	Matrix Family Library Version 5.0 (February 2005)
**Selected groups** (core/matrix sim):	• ALL user_defined.lib (0.75/Optimized)<br>• ALL vertebrates.lib (0.75/Optimized)

Aligned Sequences:

No.	Sequence Name	Sequence Description
1	P985229	P985229 sym=COX7B|loc=Loc1349|taxid=9606|spec=Homo sapiens|chr=X|ctg=NT_011651|str=(+)|start=450269|end=452269|len=2001|tss=1001|comm=cytochrome c oxidase subunit VIIb; (NM_001866/501/bronze;)
2	P985231	P985231 sym=Cox7b|loc=Loc66142|taxid=10090|spec=Mus musculus|chr=X|ctg=NT_039711|str=(+)|start=11047340|end=11049343|len=2004|tss=1001,1004|comm=cytochro c oxidase subunit VIIb; (AK002593/514/gold;NM_025379/511/bronze;)
3	rCOX7B	rCOX7B rn3_dna range=chrX:94210088-94212438 5'pad=0 3'pad=0 revComp=FALSE strand=? repeatMasking=nc

Alignment (DiAlign format):

Please note that only **upper-case** letters are considered to be **aligned**. For more information, please have a look at the user guide.

V$RBPF V$IKRS V$OCT1 V$CLOX V$E2FF V$SF1F V$GATA V$STAT V$LEFF V$TCFF

```
alignment position 1 . . . . . . . . .   1 1 . . . . . . . .   2 1 . . . . . . . .   3 1 . . . . . . . .   4 1 . . . . . . . .
P985229      1  gcgattctgc  cgcctcagtc  tacagagtag  ctgggattac  aggcacatgc
P985231      1  acTATTACTG  AATGGGAcaT  GCTAAGATCC  TATACCTTTT  GATAGGATGC
rCOX7B       1  at TATTACTG  AATGGGA--T  GCTAAGGTCC  TATACCTTCT  GATAGGATGC
```

```
                              * * * * * * * *  * * * * * * *    *  * * * * * * * * * *  * * * * * * * * * *  * * * * * * * * * *
                                                                 *  * * * * * * * * * *  * * * * * * * * * *  * * * * * * * * * *
                                                                 *  * * * * * * * * * *  * * * * * * * * * *  * * * * * * * * * *
                                                                 *  * * * * * * * * * *  * * * * * * * * * *  * * * * * * * * * *

alignment position  5 1 . . . . . . .  6 1 . . . . . . .  7 1 . . . . . . .  8 1 . . . . . . .  9 1 . . . . . . .
P985229         51  c a c c a c g c c c  a g c t a a t t g t  t g g t a t t t t t  a g t a g a g a t g  g g g - - - - - - -
P985231         51  T C T A A A A A G c  a g A G T T T G A T  T T G T A C A T T A  T T T A T C T A A T  C A T G A C A C T a
rCOX7B          49  T C T A A G A A G c  - - A G T T T G A T  T T G T A C G T T A  T T T C T C T A A T  C A T G A C A C T g
                    * * * * * * * * *       * * * * * * * *  * * * * * * * * * *  * * * * * * * * * *  * * * * * * * * *
                    * * * * * * * * *       * * * * * * * *  * * * * * * * * * *  * * * * * * * * * *  * * * * * * * * *
                    * * * * * * * * *       * * * * * * * *  * * * * * * * * * *  * * * * * * * * * *  * * * * * * * * *
                    * * * * * * * * *       * * * * * * * *  * * * * * * * * * *  * * * * * * * * * *  * * * * * * * * *

alignment position  1 0 1 . . . . . . .  1 1 1 . . . . . . .  1 2 1 . . . . . . .  1 3 1 . . . . . . .  1 4 1 . . . . . . .
P985229         94  - - - - - - - - - -  - - - - - - - - - -  T T T C A C C A T G  T T G G C C A G G A  T G G T C T C G A T
P985231        101  G A G A A G C T A A  G G C A A A A G A A  T T G C A A A T T C  T A G G C C A G T C  T G G G C T A C A T
rCOX7B          97  G A G A A G C T A A  G G C A G A A A A A  T C G C A A A T T C  C A G G C C A G T C  T G G G C T A C A T
                    * * * * * * * * * *  * * * * * * * * * *  * * * * * * * * * *  * * * * * * * * * *  * * * * * * * * * *
                    * * * * * * * * * *  * * * * * * * * * *  * * * * * * * * * *  * * * * * * * * * *  * * * * * * * * * *
                    * * * * * * * * * *  * * * * * * * * * *  * * * * * * * * * *  * * * * * * * * * *  * * * * * * * * * *
                    * * * * * * * * * *  * * * * * * * * * *  * * * * * * * * * *  * * * * * * * * * *  * * * * * * * * * *

alignment position  1 5 1 . . . . . . .  1 6 1 . . . . . . .  1 7 1 . . . . . . .  1 8 1 . . . . . . .  1 9 1 . . . . . . .
P985229        124  c t c c t g a c c t  c g t g a t c c g c  c c a c c t c g g c  c t c c c a a a g t  g c t g g g a t t a
P985231        151  A G T A A G T T C T  A G C C A G T C T G  G G T T A C A T A G  g g a g a c C T G G  C T T A C A C A G A
rCOX7B         147  A G T G A G T T C T  A G C C A G T T T A  G G T T A C A T A G  t a a a c t C T G T  C T T A C A C A G A
                    * * * * * * * * * *  * * * * * * * * * *  * * * * * * * * * *              * * * *  * * * * * * * * * *
                    * * * * * * * * * *  * * * * * * * * * *  * * * * * * * * * *              * * * *  * * * * * * * * * *
                    * * * * * * * * * *  * * * * * * * * * *  * * * * * * * * * *              * * * *  * * * * * * * * * *
                    * * * * * * * * * *  * * * * * * * * * *  * * * * * * * * * *

alignment position  2 0 1 . . . . . . .  2 1 1 . . . . . . .  2 2 1 . . . . . . .  2 3 1 . . . . . . .  2 4 1 . . . . . . .
P985229        174  c a g g c g t g a g  c c a c c g c g c c  a g g c c t a a c a  g c t t t c t T T T  C T T T C T T T C T
P985231        201  G - - - - - - - - -  - - - - - - - - - -  - - - - - - - - - -  - - - - - - - T T T  C T T T T T T T T T
rCOX7B         197  G - - - - - - - - -  - - - - - - - - - -  - - - - - - - - - -  - - - - - - - T T T  C T T T C T T T C T
                    *                                                                        * * *  * * * * * * * * * *
                    *                                                                        * * *  * * * * * * * * * *
                    *                                                                        * * *  * * * * * * * * * *
                                                                                             * * *  * * * * * * * * * *

alignment position  2 5 1 . . . . . . .  2 6 1 . . . . . . .  2 7 1 . . . . . . .  2 8 1 . . . . . . .  2 9 1 . . . . . . .
P985229        224  T T T T g a g a c g  t a g t c t c g c t  c t g t c g c c c a  g g c t g g a g t c  c a g t g g c a c g
P985231        215  T T T T T - - - - -  - - - - - - - - - -  - - - - - - - - - -  - - - - - - - - - -  - - - - - - - - - -
rCOX7B         211  T T T T T - - - - -  - - - - - - - - - -  - - - - - - - - - -  - - - - - - - - - -  - - - - - - - - - -
                    * * * * *
                    * * * * *
                    * * * * *
                    * * * *

alignment position  3 0 1 . . . . . . .  3 1 1 . . . . . . .  3 2 1 . . . . . . .  3 3 1 . . . . . . .  3 4 1 . . . . . . .
P985229        274  t t c t c g g c t c  a c t g c a a c c t  c c g c c t c c c g  g g t t c a a g t g  a t t a t t c t g c
P985231        220  - - - - - - - - - -  - - - - - - - - - -  - - - - - - - - - -  - - - - - - - - - -  - - - - - - - - - -
rCOX7B         216  - - - - - - - - - -  - - - - - - - - - -  - - - - - - - - - -  - - - - - - - - - -  - - - - - - - - - -

alignment position  3 5 1 . . . . . . .  3 6 1 . . . . . . .  3 7 1 . . . . . . .  3 8 1 . . . . . . .  3 9 1 . . . . . . .
P985229        324  c t c a g c c t c c  t g a g t a g c t g  g g a t g a c a g g  c g c g c g c c a c  c a c g c c g g c t
P985231        220  - - - - - - - - - -  - - - - - - - - - -  - - - - - - - - - -  - - - - - - - - - -  - - - - - - - - - -
rCOX7B         216  - - - - - - - - - -  - - - - - - - - - -  - - - - - - - - - -  - - - - - - - - - -  - - - - - - - - - -

alignment position  4 0 1 . . . . . . .  4 1 1 . . . . . . .  4 2 1 . . . . . . .  4 3 1 . . . . . . .  4 4 1 . . . . . . .
P985229        374  a a T T T T T G T A  T T T t t a g t a g  a g a c g g g g t t  t c a c c a t c t t  g g c c a g g c t g
P985231        220  - - T T T T T T T A  T T T - - - - - - -  - - - - - - - - - -  - - - - - - - - - -  - - - - - - - - - -
rCOX7B         216  - - T a - - - - - -  - - - - - - - - - -  - - - - - - - - - -  - - - - - - - - - -  - - - - - - - - - -
```

```
                           * * *** **** * **
                           *
                           *
alignment  position 451.......  461.......  471.......  481.......  491.......
P985229        424  gtctcgaact  ccgacctcgt  gatccgccca  ccttggcctc  tcaaagtgct
P985231        231  ----------  ----------  ----------  ----------  ----------
rCOX7B         218  ----------  ----------  ----------  ----------  ----------

alignment  position 501.......  511.......  521.......  531.......  541.......
P985229        474  gggattacag  gcgtgagcca  ccgcgcccgg  ccaatagctt  tccAAAGTTA
P985231        231  ----------  ----------  ----------  ----------  -------ATT
rCOX7B         218  ----------  ----------  ----------  ----------  ---AAACATT
                                                                       *******
alignment  position 551.......  561.......  571.......  581.......  591.......
P985229        524  TTTTATCACG  TATTTATCTA  c--ATATTAC  AGAAGTTTTT  ACTGTTCTTG
P985231        234  ATTTATTATA  Tgtaagtaca  ctgtagctgt  cttcagacac  accagaagag
rCOX7B         225  TTTTATTATA  TATTTCTTTA  tttACATTTC  AAAGGTTATT  ACCCTTCCTG
                    **********  **********     *******  **********  **********
alignment  position 601.......  611.......  621.......  631.......  641.......
P985229        572  caactttct   ctaaacttga  cgttatttga  aa--------  ----------
P985231        284  ggcatcagat  ctcattaagg  atggttgtga  gccaccatgt  ggttgctggg
rCOX7B         275  gtttcctgtc  cataagcccc  catcccctcc  ccctccctca  tatgggtatt

alignment  position 651.......  661.......  671.......  681.......  691.......
P985229        604  ----------  ----------  ----------  ----------  ----------
P985231        334  atttgaactc  tggacctttg  gaagagcagt  cgggtgctct  tacccactga
rCOX7B         325  ccccccttact gccccccccc  ccatattccc  ttgcactggg  gggtccaacc

alignment  position 701.......  711.......  721.......  731.......  741.......
P985229        604  ----------  ----------  ----------  ----------  ----------
P985231        384  gccatctcac  cag-------  ----------  ----------  ----------
rCOX7B         375  ttggcaagac  ctagggcttt  cccttccact  ggtgccccaa  caaagctatt

alignment  position 751.......  761.......  771.......  781.......  791.......
P985229        604  ----------  ----------  ----------  ----------  ----------
P985231        397  ----------  ----------  ----CCCTAC  ACAGAGTTTc  ----------
rCOX7B         425  ctctgctaca  tatgcagttg  gagcCCTTAC  ACAGAGTTTa  agaagataca
                                                ******  *********
alignment  position 801.......  811.......  821.......  831.......  841.......
P985229        604  ----------  ----------  ----------  ----------  ----------
P985231        413  ----------  ----------  ----------  ----------  ----------
rCOX7B         475  tttgnnnnnn  nnnnnnnnnn  nnnnnnnnnn  nnnnnnnnnn  nnnnnnnnnn

alignment  position 851.......  861.......  871.......  881.......  891.......
P985229        604  ----------  ----------  ----------  ----------  ----------
P985231        413  ----------  ----------  ----------  ----------  ----------
rCOX7B         525  nnnnatatct  gaagcatgtg  aagatgtcac  ctacatgccc  aggcgttcac

alignment  position 901.......  911.......  921.......  931.......  941.......
P985229        604  ----------  ----------  ----------  ----------  ----------
P985231        413  ----------  ----------  ----------  ----------  ----------
rCOX7B         575  acagatgcct  ctgggaagaa  aacttcaggc  aatacaaatc  gcaagacagg

alignment  position 951.......  961.......  971.......  981.......  991.......
P985229        604  ----------  ----------  ----------  ----------  ----------
P985231        413  ----------  ----------  ----------  ----------  ----------
```

```
rCOX7B       625 agtgaagctt aaacacagct gtctgcttag ccttggctga tcagtcagga

alignment position 1001...... 1011...... 1021...... 1031...... 1041......
P985229      604 ---------- ---------- ---------- ---------- ----------
P985231      413 ---------- ---------- ---------- ---------- ----------
rCOX7B       675 cagcaggaca ctgcttccct ctaggcaaat actaaactga acccacagtt

alignment position 1051...... 1061...... 1071...... 1081...... 1091......
P985229      604 ---------- ---------- ---------- ---------- ----------
P985231      413 ---------- ---------- ---------- ---------- ----------
rCOX7B       725 tgttccgtct gctctaacca agagtaggaa agagatgaga aaagcaccta

alignment position 1101...... 1111...... 1121...... 1131...... 1141......
P985229      604 ---------- ---------- ---------- ---------- ----------
P985231      413 ---------- ---------- ---------- ---------- ----------
rCOX7B       775 gagattccta tttggaaaag agaagccaaa tgcacatgaa tactttttaca

alignment position 1151...... 1161...... 1171...... 1181...... 1191......
P985229      604 ---------- ---------- ---------- ---------- ----------
P985231      413 ---------- ---------- ---------- ---------- ----------
rCOX7B       825 tttcaaaggt tattaccctt cctggtttcc tgtccataag cccccatccc

alignment position 1201...... 1211...... 1221...... 1231...... 1241......
P985229      604 ---------- ---------- ---------- ---------- ----------
P985231      413 ---------- ---------- ---------- ---------- ----------
rCOX7B       875 ctccccctcc ctcatatggg tattccccct tactgccccc ccccccatat

alignment position 1251...... 1261...... 1271...... 1281...... 1291......
P985229      604 ---------- ---------- ---------- ---------- ----------
P985231      413 ---------- ---------- ---------- ---------- ----------
rCOX7B       925 tcccttgcac tggggggtcc aaccttggca agacctaggg ctttcccttc

alignment position 1301...... 1311...... 1321...... 1331...... 1341......
P985229      604 ---------- ---------- ---------- ---------- ----------
P985231      413 ---------- ---------- ---------- ---------- ----------
rCOX7B       975 cactggtgcc ccaacaaagc tattctctgc tacatatgca gttggagccc

alignment position 1351...... 1361...... 1371...... 1381...... 1391......
P985229      604 ---------- ---------- ---------- ---------- ----------
P985231      413 ---------- -TTAAGAAGA TACA----TA TATAGTATTC TTGCCAGATG
rCOX7B      1025 ttacacagag tTTAAGAAGA TACAtttgTA TATAGTATCA TTGCCAGATG
                             ********* ****    ** ********** **********
                                               ** ********** **********
alignment position 1401...... 1411...... 1421...... 1431...... 1441......
P985229      604 ---------- ---------- ---------- ---------- ----------
P985231      448 CACATgaac- ---TGGAATC CTTAGGGGAA AAAggtc-AA TTGATCTCCC
rCOX7B      1075 TACATacact gaaTGGAATC CCTAGGGAAA AAAagactAA TTGATCTCCC
                 *****         ******* ********** ***     ** **********
                 *****
alignment position 1451...... 1461...... 1471...... 1481...... 1491......
P985229      604 ---------- ---------- ---------- ---------- ----------
P985231      493 AGcacttggg aggcaaaggc aggacttttt ctgtgagttc caggccagct
rCOX7B      1125 AG-------- ---------- ---------- ---------- ----------
                 **
alignment position 1501...... 1511...... 1521...... 1531...... 1541......
P985229      604 ---------- ---------- ---------- ---------- ----------
```

```
P985231        543 tggtctacat agagagttcc tggtcagcca ggactaaata gagaggccgt
rCOX7B        1127 ---------- ---------- ---------- ---------- ----------

alignment position 1551...... 1561...... 1571...... 1581...... 1591......
P985229        604 ---------- ---------- ---------- --------AC TAAAAGCTAA
P985231        593 gtctcaactc ccccacaaaa attccaaaaa aaaaaaccAA TAAAGGTAAA
rCOX7B        1127 ---------- ---------- ---------- ---------- ----------
                                                            ** **********

alignment position 1601...... 1611...... 1621...... 1631...... 1641......
P985229        616 GCCAATTAGT ATAGtaGTAA TAGCAGACAA AAaaaaTGAG AGAAGTAAAG
P985231        643 ACCAATTAAT GTAG--GTAA TAGGAGACAG AAtg--TGAA AAATTTGAAG
rCOX7B        1127 ---------- ---------- ---------- ---------- ----------
                   ********** ****  **** ********** **    **** **********

alignment position 1651...... 1661...... 1671...... 1681...... 1691......
P985229        666 GGAGTTTTGG GGGAACAGAA AGAagaaaga gctagtgtgg agatactaaa
P985231        689 GGACTGTAAT TGGAAGTGAG ACActagatc caat------ ----------
rCOX7B        1127 ---------- ---------- ---------- ---------- ----------
                   ********** ********** ***

alignment position 1701...... 1711...... 1721...... 1731...... 1741......
P985229        716 ccgaagCTTG TGGAACTTGA ACGCCATTTT GAAGCTAGTT CCTTAgacga
P985231        723 ------CCTC TGGAGTTTTA GCACCATTTT GATGCTGGTG CCTTATGT--
rCOX7B        1127 ---------- ---------- ----CATCTT GAAGCTGGTG CCTTCTGT--
                         **** ********** ********** ********** ********
                                             ****** ********** ********
                                                 ** ********** ********
                                                 ** ********** *****
                                                 ** ********** ****

alignment position 1751...... 1761...... 1771...... 1781...... 1791......
P985229        766 ttcggtcgAA GATCTGTCTC TCGGCTGCCC TCTATAGAGC TCTAAACCTG
P985231        765 --------AA GGTCGGTGTC TCAGCTCTCC TTTGTAGAGT CCGAAACCTT
rCOX7B        1151 --------AA GGTTGGTGTC TCAGaTCTCC TTTGTAGAGT CCTAAACCTT
                           ** ********** ********** ********** **********
                           ** ********** **** ***** ********** **********
                           ** ********** **** ***** ********** **********
                           ** ********** **** ***** ********** **********
                                              ***** ********** **********
                                              ***** ********** **********
                                                 ** ********** ********

alignment position 1801...... 1811...... 1821...... 1831...... 1841......
P985229        816 GCTTCAAGGA GTTCT-TTCa ttcttcatta gataccaccc accatacgtc
P985231        807 TGTCCAAAGA GTTAacCTCC CTGCTTAGTA AATACTACTT TATGGaAGCC
rCOX7B        1193 TGTCCAAAGA GTTAT-CTCC CTGTTTAGTA AATACTAGTC AATGGcAGCC
                   ********** ***** **** ********** ********** ***** ****
                   ********** ****  **** ********** ********** ***** ****
                   ********** ****  ***                              ****
                   ********** ****                                   ****
                   ********** ****
                   ********** ****

alignment position 1851...... 1861...... 1871...... 1881...... 1891......
P985229        865 attttATAAA GCCGGAAACT ACATTTCCCA AAaggccttt aTT----TTT
P985231        857 -----ATCAA GCTTGAAACT ACATTTCCCA AAGACC---- -TTcctaTTT
rCOX7B        1242 -----ATCAA GTTTGAAACT ACATTTCCCA AAGGCC---- -TT----TTT
                        ***** ********** ********** ******      **    ***
                        ***** ********** ********** ******      **    ***
                        ***** ********** ********** ******      **    ***
                        ***** ********** ********** ******      **    ***
                        ***** ********** ********** **          **    ***
                                  ****** ********** **          **    ***
                                  ****** ********** **                ***
                                                                      ***

alignment position 1901...... 1911...... 1921...... 1931...... 1941......
```

```
P985229       911  CCTCGCGGGA AAAGGGATTG CAATTACTAT AGGTtttaca gg-----TAT
P985231       897  CCTCGCAGGA ACTAGGATGA CAATTTCTAT AGGTCAAc-- CCCTAAATCT
rCOX7B       1278  TCTCGCAGGA AATAGGATTA CAATTTCTAT AGGTCAAttt CCCCAAATCT
                   ********** ********** ********** *******    **********
                   ********** ********** ********** *******    **********
                   ********** ********** ********** *******           ***
                   ********** ********** ********** *******           ***
                   ********** ********** ********** ****
                   ********** ********** ********** ****
                   ********** ********** ********** ****
                   ********** ********** ********** ****

alignment position 1951...... 1961...... 1971...... 1981...... 1991......
P985229       956  CGCGAGATTT CGTCAAATCT CATTACGGAT CCCGGCTGAA AGCCATTTTG
P985231       945  CGCGACATCT CACCAATTCT CATTTGGGCT CCCTGCTGAA AGCCATTTTG
rCOX7B       1328  CGCGATATGT TACCAAATCT CATTAGGAAT CGCCGCaGAA AGCCATTTTG
                   ********** ********** ********** ********** **********
                   ********** ********** ********** ********** **********
                   ********** ********** ********** ********** **********
                   ********** ********** ****  **** ******  ** **********
                                              *            *** **********
                                              *            *** **********
                                                           *** **********
                                                           *** **********

alignment position 2001...... 2011...... 2021...... 2031...... 2041......
P985229      1006  TTTTTCAGCT CAC--TTCAA GGGTACCTGA AGCGAATTGG CACCAAAGCA
P985231       995  TTTTTCAGGA CgcttTGCAA GGGTAGCTGG GGTGAATTTG CACCAAGGCA
rCOX7B       1378  TTTTTCAGGA CTC--TGCAA GGGTAGCTGG GGTGAATTTG CACCCAGGTA
                   ********** ***  ***** ********** ********** **********
                   ********** ***  ***** ********** ********** **********
                   ********** ***  ***** ********** ********** **********
                   ********** *    ***** ********** ********** **********
                   ********** *    ***** ********** ********** **********
                   ********** *    ***** ********** ********** **********
                   ********        ***** ********** ********** **********
                   ********          *** *********
                                     *** *********

alignment position 2051...... 2061...... 2071...... 2081...... 2091......
P985229      1054  GCAGCTGTAt t-GCCGCAGT TCTAGCTTCA CCTTCACGAT GTTTCCCTTG
P985231      1045  GCAGCa-TAG TCGCCGCAGT TCCATCTTCG TCTCCGCGAT GTTGCCCTTA
rCOX7B       1426  GCAGCTGTAG TCACCGCAGT TCCATCCTTG TCTCCGCGAT GTTGCCCTTG
                   ********** ********** ********** ********** **********
                   *****  *** ********** ********** ********** **********
                   *****  *** ********** ********** ********** **********
                   *****  **    ******** ********** ********** **********
                   *****        ******** ********** ********** **********
                   *****                                         ********
                   *****                                         ********
                                                                    *****
                                                                    ****
                                                                    ****

alignment position 2101...... 2111...... 2121...... 2131...... 2141......
P985229      1103  GTCAAAAGCG CACTAAATCG TCTCCAAGGT GAGCAaaaat tatgacaaat
P985231      1094  GCCAAAAACG CACTAAGCCG TCTCCAAGGT GAGCAGATAA TGTGGGCGAG
rCOX7B       1476  GCCAAAAACG CACTAAGTCG TCTCCAAGGT GAGCAGATAA TGTGGGCGAG
                   ********** ********** ********** ********** **********
                   ********** ********** ********** ********** **********
                   ********** ********** ********** ********** **********
                   ********** ********** ********** ********** **********
                   ********** ********** ********** ********** **********
                   ********** ********** ********** *****
                   ********** ********** ********** *****
                   ********** ********** ********** *****
                     ******** ********** ********** *****
                     ******** ********** ********** *****

alignment position 2151...... 2161...... 2171...... 2181...... 2191......
```

```
P985229      1153 ca-TTTACAA CAACCttatA TCAATGTGTC CTCGCGGCCT Ctcgtgtgct
P985231      1144 TTCTTTACAA CACCCGGG-A TCCggctgtC CTCGTGCTCT TCTGCCCTCT
rCOX7B       1526 CCCTTTACAA CACCCGGG-A TCCATCTGCC CTCGTGCTCT CATGCCTTTT
                  ********** ******** * ********** ********** **********
                  ********** ******** * ***      * ********** **********
                  ********** ******** * ***      * ********** **********
                  ********** ******** * ***
                  ********** ******** * ***

alignment position 2201...... 2211...... 2221...... 2231...... 2241......
P985229      1202 ttccttttac gaacatctcg gccttctaat cttgaatgct gtctGTCTCC
P985231      1193 TCCTCCGCCC CAAATCCTGc ggGTTTTAAT TCTAGGTGCA TG--GTCCCC
rCOX7B       1575 TCCTCTGCCC TAAAGCCTGc a-GTTTTAAT TCTAGGTGCA CG--GTtCCC
                  ********** *********    ******** ********** **  ******
                  ********** *********    ******** ********** **  ** ***
                  ********** *********                               ***
                                                                     ***

alignment position 2251...... 2261...... 2271...... 2281...... 2291......
P985229      1252 CATCTCGTCC CAAGTCATTG GGATGATgtc cttag----- ----------
P985231      1241 TGCGTGGTCT CCAGTCACTG GGAAGggaag ctagccataa cagaGGTAGC
rCOX7B       1622 TGCGTGGTCT CCAGTCACTG GGAAGATagc aatgacagt- ----GGTAGC
                  ********** ********** *******                  ******
                  ********** ********** *****                    ******
                  ********** ********** *****                    ******
                  ********** ********** *****                    ******
                                                                 ******
                                                                 ******

alignment position 2301...... 2311...... 2321...... 2331...... 2341......
P985229      1287 -----CATTG GCTTTATCAC CCAGTATTTC TGTTtcggtt tccagctctc
P985231      1291 TGTGACAGTG GCTTTATCAT CCTGTCCTTG TGTTCCTAAC CTCGTT----
rCOX7B       1667 TGTGACAGTG GCTTTATCAT CCTGTCCTTG TGTTCCTAGC CTAGTT----
                  ********** ********** ********** ********** ******
                  ********** ********** ********** ********** ******
                  ********** ********** ********** ********** ******
                  ********** ********** ********** ********** ******
                  ********** ********** ********** ****
                  ********** ********** ********** ****
                       ***** ********** ********** ****

alignment position 2351...... 2361...... 2371...... 2381...... 2391......
P985229      1332 tttctgttat cggagtgata gtctagccCT GCTTTTTAAT TCTTCAGTAA
P985231      1337 ---------- ---------- --------AT GCATTCTCTT TCTTCAGAGA
rCOX7B       1713 ---------- ---------- --------CT GCATTCTCTT TCTTCAGAGA
                                                ** ********** **********
                                                ** ********** **********
                                                ** ********** **********
                                                ** ********** **********
                                                ** ********** **********
                                                 * ********** **********

alignment position 2401...... 2411...... 2421...... 2431...... 2441......
P985229      1382 CCTTTGATTT CAGGGAAGga cattcttggt aGACACCTCG TTCCTTAAGT
P985231      1359 CCTATGATTT CCAGGAAGAA CGCTTTGGCG -GACACCTTG CTCTTTTAGC
rCOX7B       1735 CCTATCATTT CCAGGAAGAC CGTTTTGGCG -GGCACCTTG CTCTTTTAGC
                  ********** ********** **********  ********* **********
                  ********** ********** **********  ********* **********
                  ********** ********** **********  ********* **********
                  ***** **** ********** **********  ********* **********
                  ***** **** ********                ********* **********
                  ***** **** ********                   ******* **********
                                                        ******* *******

alignment position 2451...... 2461...... 2471...... 2481...... 2491......
P985229      1432 GATGTCCTAC ATTTTAGCTT TGGggatatg tttactccta accctGTATA
P985231      1408 GATGATCTAA ATTTTAGCTC TGGGAGTGTA TTTAc----- -----GTAGA
rCOX7B       1784 CATGTTCTAA ATTGTAGCTT TGGGACTGTA TTTAtat--- ----------
```

```
                        ********** ********** ********** ****              *****
                        ********** ********** ********** ****
                        ********** ********** ********** ****
                        ********** ********** ***
                        ********** *********
                        ********** *********

alignment position 2501...... 2511...... 2521...... 2531...... 2541......
P985229       1482 GAAAAATATC TTCTCCAATt cctattactg taatagacgg aaactgtaaa
P985231       1448 AGAACTGATC TCCTACAATa a---CGGTGG TAGGAAACTA AATCTA----
rCOX7B        1821 AGAACTGATC TCCTAtggtg gtagCGGTGG TAGGAAACTA AATCTA----
                   ********** *********       ****** ********** ******
                                              ****** ********** ******
                                              ****** ********** ******
                                              ****** ********** ******

alignment position 2551...... 2561...... 2571...... 2581...... 2591......
P985229       1532 tctagctCTT CACCTGGTTT AATTGCAGGG AAACAACCCA AAtggtatcg
P985231       1491 -------CCG CATCTGGATT CATAGCAAGG ggttgaaaac cttaagcCTG
rCOX7B        1867 -------CCT CACCTGCATT CATAGCGAGG GGGAAAAGCA AAatt--CTG
                          *** ********** ********** ********** **     ***
                          *** ********** *****                        ***
                          *** ********** *****                        ***
                          *** ********** *****                        ***
                                                                      ***

alignment position 2601...... 2611...... 2621...... 2631...... 2641......
P985229       1582 atGCAGCTTT GAAGttattt gtggattaac aatgttagct ggccaggcgc
P985231       1534 --GCAGCTTT GAAGAAGTCA TCTGTAGATT ATTAACGG-- ----------
rCOX7B        1908 --GCAGCTTT GAAGAAGTCG TCTGTAGATT ATTAACGG-- ----------
                     ******** ********** ********** ********
                     ******** ********** ********** ********
                     ******** ********** ********** ********
                     ******** ********** ********** ********
                     ******** ********** ********** ********
                     ******** ****

alignment position 2651...... 2661...... 2671...... 2681...... 2691......
P985229       1632 ggtggctcac acttgtaatc ccagcacttt gggaggccaa ggcgggcgga
P985231       1570 ---------- ---------- ---------- ---------- ----------
rCOX7B        1944 ---------- ---------- ---------- ---------- ----------

alignment position 2701...... 2711...... 2721...... 2731...... 2741......
P985229       1682 tcccctgagg tcaggagttc gagaccagcc tggccaacat agtgaaacgc
P985231       1570 ---------- ---------- ---------- ---------- ----------
rCOX7B        1944 ---------- ---------- ---------- ---------- ----------

alignment position 2751...... 2761...... 2771...... 2781...... 2791......
P985229       1732 cgtccctact aaaaatacaa aaattagccg ggcgtggtgg tgggcgcctg
P985231       1570 ---------- ---------- ---------- ---------- ----------
rCOX7B        1944 ---------- ---------- ---------- ---------- ----------

alignment position 2801...... 2811...... 2821...... 2831...... 2841......
P985229       1782 taatcccagc tacttgggag gctgaggcaa gagaatcgct tgtacccggg
P985231       1570 ---------- ---------- ---------- ---------- ----------
rCOX7B        1944 ---------- ---------- ---------- ---------- ----------

alignment position 2851...... 2861...... 2871...... 2881...... 2891......
P985229       1832 aggcggaggt tgcagtgagc caagactgct actgcactcc agcctgggca
P985231       1570 ---------- ---------- ---------- ---------- ----------
rCOX7B        1944 ---------- ---------- ---------- ---------- ----------

alignment position 2901...... 2911...... 2921...... 2931...... 2941......
```

```
P985229   1882 acaagagcgg gactccgtct cgaaaacaaa aacaaaacAA TGTTAGCTGT
P985231   1570 ---------- ---------- ---------- --------AA TATTAACTAT
rCOX7B    1944 ---------- ---------- ---------- --------AA TATTAATTAT
                                                       ** **********
                                                       ** **********
                                                       ** **********
                                                       ** **********
                                                       ** **********
                                                       *
                                                       *

alignment position 2951...... 2961...... 2971...... 2981...... 2991......
P985229   1932 CCTGAAGGCA ATCGTGGTCA TTTTTTTctt atcc--AAAG GTTGGGCAGG
P985231   1582 AGCGAAAACA ATCATGGTCA TTTTTTTTTC CTGCTTTAAA GTTAGGCAAG
rCOX7B    1956 CGCGAAGACA ATCATGGTCA TTTg---TTC CTGCTTAAAA GTTAGGCAAG
               ********** ********** ********** ********** **********
               ********** ********** ********** ********** **********
               ********** ********** ***    *** ********** **********
               ********** ********** ***    *** ********** **********
               ********** ********** ***    *** ********** **********
                                                      **** **********

alignment position 3001...... 3011...... 3021...... 3031...... 3041......
P985229   1980 TACAGGGAGT TTTGAGACCA GC-------- ---------- ----------
P985231   1632 TACGTGGAAA CTTGAGACTA GCAGTATATA TGGAAAATAC ACTGGACTGG
rCOX7B    2003 TACGTGGAAA TTTGAGACTA GCATTATATA TGGAGAAAAC ATTAGACTGG
               ********** ********** ********** ********** **********
               ********** ********** ********** ********** **********
               ********** ********** ********** ********** **********
               ********** ********** **
               ********** ********** **
               ********** *******

alignment position 3051...... 3061...... 3071...... 3081...... 3091......
P985229   2002 ---------- ---------- ---------- ---------- ----------
P985231   1682 AAAGaaAAAA ATTCATTTTT GTAATTGCTT TACCcTGAAG GTGACCTTCA
rCOX7B    2053 AAAG--AAAA ACTCATTTTC GTAATTGCTT TCCCgTGAAG GTGACCTTCA
               ****  **** ********** ********** ****  ***** **********
               ****  **** ********** ********** ****  ***** **********
               ****                                   ***** **********
                                                      ***** **********
                                                      ***** **********

alignment position 3101...... 3111...... 3121...... 3131...... 3141......
P985229   2002 ---------- ---------- ---------- ---------- ----------
P985231   1732 GTAATGTGAA TGAAGGTGAC CTTACacaat actta----C TTGCTTTTCT
rCOX7B    2101 GTAATGTGAA TAAAGGTGAC CTTACgcaaa tacctttagC TTGCTTCTCT
               ********** ********** *****               * **********
               ********** ********** *****               * **********
               ********** ********** *****               * **********
               ********** ********** *****
               ********** ********** *****

alignment position 3151...... 3161...... 3171...... 3181...... 3191......
P985229   2002 ---------- ---------- ---------- ---------- ----------
P985231   1778 AGCCATGGAT TTAGTTTTAT TTTACcttta gatcacatg- ----CTGTGT
rCOX7B    2151 AGTCATGGAT TTATTTTTAT TTTACagaaa tgttaggagt atcaCTGTGT
               ********** ********** *****                     ******
               ********** ********** *****                     ******
               ********** ********** *****                     ******
                                                               ******
                                                               ******

alignment position 3201...... 3211...... 3221...... 3231...... 3241......
P985229   2002 ---------- ---------- ---------- ---------- ----------
P985231   1823 TCTAAGTACT GAAGTACTTT GGGAATAATT GAAAaaaaaa aaaaagaaAG
rCOX7B    2201 TCTAAGCACT GAAGTACTTT GGGAATAATT GAAAaaat-- --------AG
```

```
                         ********** ********** ********** ****          **
                         ********** ********** ********** ****          **
                         ********** ********** ********** ****          **
                         ********** ********** ********** ****
                         ********** ********** ********** ****

alignment position       3251...... 3261...... 3271...... 3281...... 3291......
P985229            2002  ---------- ---------- ---------- ---------- ----------
P985231            1873  AAGCCCAACT GATCTTTCAT CTGCTACAAG CTGTGTTtat taTTTCTTTC
rCOX7B             2241  GAGCCCAATT GATGTTTCAT CTGCCACAGG CTGTGTTc-- --TTTCTTTC
                         ********** ********** ********** *******     ********
                         ********** ********** ********** *******     ********
                         ********** ********** ********** *******     ********
                                                                      ********
                                                                      ********

alignment position       3301...... 3311...... 3321...... 3331...... 3341......
P985229            2002  ---------- ---------- ---------- ---------- ----------
P985231            1923  ACTTTGGACA AATGATTTAG CGTTATAAAc TTGTTTTTGT AAAACAGAGG
rCOX7B             2287  ACTTTGGACA AATGATTTAG CTTTATAAAt TTGTTTTTGT AAAACAAAGG
                         ********** ********** *********  ********** **********
                         ********** ********** *********  ********** **********
                         ********** ********** *********  ********** **********
                         ********** ********** *********  ********** **********
                         ********** ********** *********

alignment position       3351...... 3361...... 3371...... 3381
P985229            2002  ---------- ---------- ---------- --
P985231            1973  TTAGTAAGTA ATACCtatgt caaagaccat ta
rCOX7B             2337  TTAGTAAGTA ATACC----- ---------- --
                         ********** *****
                         ********** *****
                         ********** *****
                         ********** *****
```

Pairwise similarities:

For each pairwise alignment, the similarity (relative to the maximum similarity) and the number of identical nucleic acids (in % of shorter sequence) is given. Maximum values are underlined.

	P985231 (2004 bp)	rCOX7B (2351 bp)
P985229 (2001 bp)	0.175<br>28 %	0.171<br>26 %
P985231 (2004 bp)		1.000<br>65 %

Please note that the **similarity value 1.000** marks only the two **most similar** sequences, it does not necessarily mean that these sequences are identical.

Extract alignment region	
**Positions in alignment**	from: to:
**Output file**	dialign.seq

Extract aligned sequences

For comments, questions or bug reports, please contact support@genomatix.de

© Genomatix Software GmbH 1998-2005 License Agreement GEMS Launcher 4.1.1

- All rights reserved.

Genomatix-Logo Logout Personal Messages GEMS-Logo

Genomatix_MainGEMSElDoradoGene2PromoterBiblioSphere
Frequently_asked_questionsYour_ResultsYour_SequencesYour_ProtocolHelp

GEMS Launcher Task: *DiAlign TF*: Multiple alignment plus TF sites
working on promoters.seq (3 seq.)

[Alignment] [Pairwise_similarities]

DiAlign professional TF Release 3.1 December 2004 **Fri May 6 23:46:47 2005**

Solution parameters:

**Sequence file:**	promoters.seq (3 seq.)
**Type of sequences:**	nucleotide sequences
**Threshold T:**	0.00
**'*' signs below alignment denote**:	diagonal similarity (max. similarity is represented by 10 '*' signs)
**Alignment output:**	complete alignment is shown<br># nucleic acids per line: 50
**TF output:**	common TF matches located in aligned regions (common to 3 (100.0 %) sequences)
**Family matches:**	yes
**MatInspector library:**	Matrix Family Library Version 5.0 (February 2005)
**Selected groups** (core/matrix sim):	• ALL user_defined.lib (0.75/Optimized)<br>• ALL vertebrates.lib (0.75/Optimized)

Aligned Sequences:

No.	Sequence Name	Sequence Description	Length
1	P871935	sym=COX7A2|loc=Loc1347|taxid=9606|spec=Homo sapiens|chr=6|ctg=NT_007299|str=(-)|start=13773297|end=13774697|len=1401| tss=1001| comm=cytochrome c oxidase subunit VIIa polypeptide 2 (liver); (NM_001865/501/bronze;)	1401 bp
2	P871938	sym=Cox7a2|loc=Loc12866|taxid=10090|spec=Mus musculus|chr=9|ctg=NT_039474|str=(-)|start=26236564|end=26237987|len=1424| tss=1001,1017,1024| comm=cytochrome c oxidase, subunit VIIa 2; (AK019210/530/gold;AK011269/523/gold;NM_009945/507/silver;)	1424 bp
3	P871940	sym=Cox7a3|loc=Loc29507|taxid=10116|spec=Rattus norvegicus|chr=8|ctg=NW_047800|str=(-)|start=1094113|end=1095513| len=1401|tss=1001| comm=cytochrome c oxidase, subunit 7a 3; (NM_022503/529/bronze;)	1401 bp

Alignment (DiAlign format):

Please note that only **upper-case** letters are considered to be **aligned**. For more information, please have a look at the user guide.

V$OCT1 V$ETSF V$CMYB V$AP1F V$SF1F V$RORA

```
alignment position 1.........  11........  21........  31........  41........
P871935          1 tgggccatt   gacataCAGT  AGATTGTGAA  GAAGAAAac-  CCCAAGGATT
P871938          1 ----------  ------CAGT  AGGTTATGAA  GAAAAAAGTA  CCCCAAGATT
P871940          1 ----------  ----------  ----------  ---AAAAGTA  CCCCAAGAGT
```

```
                               ****      ********** ********** **********
                                                       ******* **********
                                                       ******* **********
                                                       ******* **********
                                                       ******* **********
                                                       ******* **********

alignment position 51........ 61........ 71........ 81........ 91........
P871935         50 ATAAGGAATa gttttaatAT TACTAATTTT TATcctttgc tgaaactatg
P871938         35 TGAAGGAATT AAGC----AT TACCAGTTTT TATTCACGCA ACACTGTGGA
P871940         18 TGAAGGAATT AGGC----AT TACCAGTttc tcTTCACTCA GCACTGTTGA
                   ********** ****    ** ********** ********** **********
                   ********** ****    ** *******      ********* **********
                   ********** ****    ** *******      ********* **********
                   ********** ****    ** *******      ********* **********
                   ********** ****    ** *******      ********* **********
                   ********** ****    ** *******

alignment position 101....... 111....... 121....... 131....... 141.......
P871935        100 ggtttc---- ---------T TATTTGAAAT GGCAATCAAA TAATTTGTAA
P871938         81 ACCCGTTTAT CATTAGCTTT TACCTGAAAT GGCAATGAAG TCGTTTCTGA
P871940         64 ACCCGTTTAT CATTAGCTCT TAc-TGAGAT GGCAATGAGG TCGTTTCTAA
                   ********** ********** ********** ********** **********
                   ********** ********** **  ****** ********** **********
                   ********** ********** **      ** ********** **********
                   ********** ********** **      ** ********** **********
                   ********** ********** **      ** ********** **********
                                                 ** ********** ******

alignment position 151....... 161....... 171....... 181....... 191.......
P871935        137 Gagtttgtag aaaGTGGAAA GA---TTATT GTAATACAGG GTAGA-TATT
P871938        131 GTTTGCAAAA GG-GTGGAAA GA---CATTT GTATTGTTGA GTAGATTATT
P871940        113 GTTTACAAAA GG-GTGGAAA GAaatTTGTT GTAGTGTTGA GTAGATTATT
                   ********** ** ******* **   ***** ********** **********
                   ********** ** ******        ** ********** **********
                   ********** ** ******        ** ********** **********
                   ********** ** ******        ** ********** **********
                   ********** ** ******                            ****

alignment position 201....... 211....... 221....... 231....... 241.......
P871935        183 TGCATTTGTA CATACTTGAG GGTAAGTACA AGAAataaa- CACTTGTAAT
P871938        177 TTTACTTGTA CATta----- -GTGTGCAGA AGAATAAAGG CAGTTGTAAG
P871940        162 TTTAATTGCA CATACATTAG GGTGTGCAGA AGAATAAAGG CAGTTGTAAG
                   ********** ********** ********** ********** **********
                   ********** ********** ********** ********** **********
                   ********** ***         ********* ********** **********
                   ********** ***         ********* ********** **********
                   ********** ***         ********* ********** **********
                                          ********* ********** **********
                                          ********* ********** **********
                                          ********* ****       **********
                                                               **********

alignment position 251....... 261....... 271....... 281....... 291.......
P871935        232 GGGGATTACT TAAGCAAAAT GGATG----A CACCGACAGC CTTTCTGTAC
P871938        221 GAGGAATAGT TATGCAAGAC TGATGCAAAA TGCTAACAGC CTATCTGTAA
P871940        212 GAGGAATAGT TAAGCAAGAC TGATGCAAAA CACAAACATC CTATCTGTAA
                   ********** ********** ********** ********** **********
                   ********** ********** ********** ********** **********
                   ********** *  ******* ********** ********** **********
                   ********** *  ******* ********** ********** **********
                   ********** *  ******* *****           ***** **********
                   ********** *  ****
                   ********** *
                   ********** *
                   ********** *

alignment position 301....... 311....... 321....... 331....... 341.......
P871935        278 AACAaataat tgctgtgctt ctCTACTCCA GTAATGAATG TAGTGCCCTG
P871938        271 AATACGCGCA CTTCTGA--- --CAGCTCCA TCCAGGAGTG AAATGTGCTG
```

```
P871940             262 AATACGCGCA CCTCTGA--- --CAACTCCA TCAAGGAGTG AAATGTACAG
                        ********** *******     ******** ********** **********
                        ********** *******     ******** ********** *******
                        ********** *******     ******** ********** *******
                        ********** *******     ******** ********** *******
                        ********** *******     ******** ********** *******
                         *

alignment  position     351....... 361....... 371....... 381....... 391.......
P871935             328 CTGGATCTGG GACAGCAAGA Cttttgagtc cagattcgcc atcccatttc
P871938             316 C-GGATCTGA GAAAGCACGA CACAGTCGAT ATTTGTGTTG AACgtggatT
P871940             307 CT-GATCTGA GAAAGCACGA CGCAGTCGTT ATTTGTGTTG AACatgga-T
                        ********** ********** ********** ********** ***      *
                           ******* ********** ********** ********** ***      *
                           ******* ********** ********** ********** ***      *
                           ******* ********** ********** ********** ***      *
                           ******* ********** ********** ********** ***      *
                           ******* ********** ********** ********** ***      *
                                                                             *

alignment  position     401....... 411....... 421....... 431....... 441.......
P871935             378 ttgttccct- ---------- ---AACGTTT CCTCAGAagc gagcgagcct
P871938             365 TAAAAATGTA TTCCCCTCTT TTCAGCGTTT TCCCAGACTC AATAACGAAG
P871940             355 TAAAAATGTA TTCCCCTCTT TTCAGCGTTT TCCCAGACTC AATAACGAAG
                        ********** ********** ********** ********** **********
                        ********** ********** ********** ********** **********
                        ********** ********** ********** ********** **********
                        ********** ********** ********** ********** **********
                        ********** ********** ********** ********** **********
                        ********** ********** ********** ********** **********
                        ********** ********** ********** *********

alignment  position     451....... 461....... 471....... 481....... 491.......
P871935             414 cttcca---- -CAATTTGGG GACACtgtGG AAGAACTTCT TGCTTGGGGA
P871938             415 TCAGCCAGTT CCAGTCTGGG GACACA--GG AAGAGCTTCC AGCACAAGGA
P871940             405 CCAGCCAGTT CCAGTCTGGG GACACA--GG ACGAGCTTCC AGCACAAGAG
                        ********** ********** ******  ** ********** **********
                        ********** ********** ******  ** * ******** **********
                        ********** ********** ******  ** * ******** **********
                        ********** ********** ******  ** * ******** **********
                        ********** ********** ******  ** *
                        ********** ********** ******  ** *
                                                      ** *

alignment  position     501....... 511....... 521....... 531....... 541.......
P871935             459 GTTCTGCAGC AGTcgaaggt acaagaatat aaaaactggt atctcgacac
P871938             463 GTTTAGCAGA AGAtccacag gttggcATAC CTGTGGATGC CAACATCGGT
P871940             453 GTTTAGCAGA AGgtgctag- ------ATAC CTGTGGATGC CAACACCGGT
                        ********** ***              **** ********** **********
                        ********** **               **** ********** **********
                        ********** **               **** ********** **********
                        ********** **

alignment  position     551....... 561....... 571....... 581....... 591.......
P871935             509 agctcttaca gcatatTCTC AACACAAGGA acagtaatgt gccgcagCAA
P871938             513 CgTTCCTATA TGA---TCTC AGCACAAAGA CTAACGCCTC C------CAC
P871940             496 CtTTCCTATA TGA---TCTC AGCACAAAGA CTAACGCCTC C------CAG
                        * ******** ***   **** ********** ********** *      ***
                        * ******** ***   **** ********** ********** *      **
                        * ******** ***   **** ********** ********** *      **
                          ******** ***   **** ********** ********** *      **
                          ******** ***   **** ********** ********** *      **
                          ******** ***   **** ********** ********** *      **
                          ******** ***   **** ********** ********** *      **

alignment  position     601....... 611....... 621....... 631....... 641.......
P871935             559 TTCCTTCGGT GGAAAAAAct ---------- -GACTGTATC CGcg-TAAGA
P871938             554 TTTCATCTGT GGAAGACAAA CCCTAAAACT GGACTCTATC CGTATTAAGA
P871940             537 TTTCATCTGT GGAAAACAAA CCCCCAAACT GAACTCTATC CGTGTTAAGA
```

```
                        ********** ********** ********** ********** **********
                        ********** ********** ********** ********** **********
                        ********** ********** ********** ********** **********
                        ********** ********** ********** ********** **********
                        ********** ********** ********** **********      *****

alignment position      651....... 661....... 671....... 681....... 691.......
P871935             597 GGAGTAATTC TTTTTTGAAT TTTATGAGGC TTcagagctg tatttcggcc
P871938             604 GGAGCTTTTG AATATTTAAG CTTGTACGAC GTAGCTTTGA C---------
P871940             587 GGAGCTTTTG TTTATTTAAG CTTGCACGAa GTAGCTTTGA C---------
                        ********** ********** ********** ********** *
                        ********** ********** *********  ********** *
                        ********** ********** *********  ********** *
                        ********** ********** *********  ********** *
                        ********** ********** ***        ********** *
                                                         ********** *

alignment position      701....... 711....... 721....... 731....... 741.......
P871935             647 aACCCAGTCT TTGTTCAGTT TCTAgcggct tcaaggttgc ccagttttac
P871938             645 -ATCCATTGT TCCTTCAGAC TGTAAACAAT ggCTCAAAGC CTT-------
P871940             628 -AGCCATTGT TCCTTCAGAC TGTAAACAAT gaCTCAAAGC CTT-------
                         ********* ********** **********   ******** ***
                         ********* ********** **********   ******** ***
                         ********* ********** **********
                         ********* ********** **********
                         ********* ********** **********

alignment position      751....... 761....... 771....... 781....... 791.......
P871935             697 gccttctcgC TCAAAACAGA CACTA-GTTt tctttGATAG CCTGGTCTCA
P871938             687 ---------C TCACAAGACA AGCTAcTTTC CG---GGTAG TCTGGTTGTC
P871940             670 ---------C TCAAAAGACA AGCTA-TTTC CC---GATAG TCTGGTTGCC
                                 * ********** *****  **** **   ***** **********
                                 * ********** *****  **** **   ***** **********
                                 * ********** *****  **** **   ***** **********
                                                     **** **   ***** **********
                                                               ***** **********
                                                                 *** **********

alignment position      801....... 811....... 821....... 831....... 841.......
P871935             746 CATTGACTTC ATCTAAGCTT ATtacattcg tctcataaat ----------
P871938             725 CACTGACTTC ACCAGAGCGT ATGAAGAGGG CTGCAaGACT TCACGCctCT
P871940             707 CACTGACTTC AGCAGA-CGT ACGAAGAGGG CTGCA-GACA TCACGC--CT
                        ********** ********** ********** *****  **** ******  **
                        ********** ******  *** ********** *****              **
                        ********** ******                                    **
                        ********** ******                                    **
                        ********** ******
                        ********** **

alignment position      851....... 861....... 871....... 881....... 891.......
P871935             786 ---------- ---CGTTTGC TTTttcccca gccttagctc cagggaccag
P871938             775 CCATTGCGGG TGGCGTTTGC TTTCCATTGT GATTCTgGGT CCGGCGGCAG
P871940             753 CCATTGCGGG CGGCATTTGC TTTCAGTTGT GATTCTaGGT CCCACTGCGG
                        ********** ********** ********** ******  *** **********
                        ********** ********** ********** ******
                        ********** ********** ********** ******
                        ********** ********** ********** ******

alignment position      901....... 911....... 921....... 931....... 941.......
P871935             823 cctactgaac atcgaaaagg tcaggaacga gatcacggga ctgagacggc
P871938             825 GCGCGGCAGG GCG------- ---------- ---------- ----------
P871940             803 GTGCCACAGG GCC------- ---------- ---------- ----------
                        ********** ***

alignment position      951....... 961....... 971....... 981....... 991.......
P871935             873 GAGCCGGCTA GAGCGGATAG AGCCGTACTG CCGctctagt ttttctttt-
P871938             838 GAGCCAAGTG GTAAGGATAG CTTCGGAGGT CCGGAGGCTA GACGGGCCGC
P871940             816 GAGACAGGAG GTAcagatgg aTTCGGAGGT CCGGAGGGTA GACCGGCCGC
```

```
                   ********** ********** ********** ********** **********
                                           ********* ********** **********
                                           ********* ********** **********
                                           ********* ********** **********
                                           ********* ********** **********

alignment position 1001...... 1011...... 1021...... 1031...... 1041......
P871935        922 ---------- ---------- --------GC TCTCTATGGT TAGGAGCGCA
P871938        888 TGCGAGAAGT CGCTCTGGCT CGCCTTCCGC TCTCTATGGT CGGGAGCGCG
P871940        866 TTCGAGGAGT CGCTCTGGCT CTCCCTCCGC TCTCTATGGT CGGGAGCGCG
                   ********** ********** ********** ********** **********
                   ********** ********** ********** ********** **********
                   ********** ********** ********** ********** **********
                   ********** ********** ********** ********** **********
                   ********** ********** ********** ********** **********
                               ********* ********** ********** **********
                                                 ** ********** **********
                                                 ** ********** **********
                                                 ** ********** **********
                                                 ** ********** **********

alignment position 1051...... 1061...... 1071...... 1081...... 1091......
P871935        944 AGCCTCTCTT CGGCCCGGaa agatttaagt tcgtgaatgc atacgcaaga
P871938        938 AGCCCCTCCT CTTCCCGGAA CGAGCCCTCT TGCTCGACGC gCGCGCAGCG
P871940        916 AGCCCCTCCT CTTCCGGGAA CGAGCACTCT TCCTTGACGC aCGCGCAGCG
                   ********** ********** ********** **********  *********
                   ********** ********** ********** **********  *********
                   ********** ********** ********** **********  *********
                   ********** ********** ********** **********  *********
                   ********** ********                          *********
                   ********** ********                          *********
                   ********** ********
                   ********** ********
                   ********** ********
                   *

alignment position 1101...... 1111...... 1121...... 1131...... 1141......
P871935        994 CTCGGAGGTA GTTCCGGTTC CGGCGTGGCC ATTTTCGTTg g-TGGTGTTC
P871938        988 CTAGGAGGGA GTTCCGTTTC CGGTCTGGTG GCTTTCGATT TTTGGCGTTC
P871940        966 CCAGGAGGGA GTTCCGTTTC CGGTCTGGTG GTTTTCGATT TTTGGCGTTC
                   ********** ********** ********** ********** **********
                   ********** ********** ********** ********** **********
                   ********** ********** ********** ********** **********
                   ********** ********** ********** * ******** **********
                   ********** ********** ********** * ******** **********
                   ********** ********** ********** * ******** **********
                   ********** ********** ********** * ******** **********
                   ********** ********** ********** * *******    ********
                       ****** ********** ********** * *****      ********
                                                      *****

alignment position 1151...... 1161...... 1171...... 1181...... 1191......
P871935       1043 AGTTGTGGCG GTTGCTGGtc agtaac--AG CCAAGATGCT GCGGAATCTG
P871938       1038 AGTAGTCGCG GTTGGTGGGT AACAACCGAG CCAAGATGTT GCGGAATCTG
P871940       1016 AGTAGTCGCG GTTGGTGGGT AACAACTGAG CTAAGATGCT GCGGAATGTG
                   ********** ********** ********** ********** **********
                   ********** ********** ********** ********** **********
                   ********** ********** ********** ********** **********
                   ********** ********** ********** ********** **********
                   ********** ********** **      ** ********** **********
                   ********** ********** **      ** ********** **********
                   ********** ********** **      ** ********** **********
                   ********** ********           ** ********** **********
                   ********** ********           ** ********** **********
                                                 ** ********** **********

alignment position 1201...... 1211...... 1221...... 1231...... 1241......
P871935       1091 CTGGTGAGGA GCTTctcatc ttgcattcat gatgggggcg aggggagtGGA
P871938       1088 CTGGTAGGTA GCTTTGGGGG TTTGGCTGGT ACTGGAGTGT C------GGG
P871940       1066 CTGGTAGGTA tCTTTGGGGA TTTTACTGGT ACTGGAGTGT C------GGG
```

```
                        ********** ********** ********** ********** *          ***
                        ********** ********** ********** ********** *          ***
                        ********** ********** ********** ********** *          ***
                        ********** ********** ********** ********** *          ***
                        **********   ***                                        ***
                        **********   ***
                        **********   ***
                        *****        ***
                        *****
                        *****

alignment  position    1251...... 1261...... 1271...... 1281...... 1291......
P871935        1141    GAGGAGGCTG AGGCACCACG ACAGAAAACC CTcacCTGGG AGGGGTGCCG
P871938        1132    GCGGAGGGTC AGGCATCCTG ACAGAAAGGC GTG--CTGGA AATGTGGCAG
P871940        1110    GCCGAGAGTG AGGTACCTTG ACAATAAGCC GTG--CTGGA AATGGGACCG
                        ********** ********** ********** ***  ***** **********
                        ********** ********** ********** ***  ***** **********
                        ******             ** ********** ***  ***** **********
                        ******             ** ********** ***  ***** **********
                        ******             ** *******

alignment  position    1301...... 1311...... 1321...... 1331...... 1341......
P871935        1191    AAGGCGGCTA ATGAAGTCAC CTTGATTCGT GTCCTTTTTC CCTGACCTTA
P871938        1180    AAGACAGCAG CCGGAGTCAC CTTGGCCGCG CGTCCTTTTC CCTGACCTTC
P871940        1158    AAGACAGCAG -CGGAGTCAC CTTGGCCGGG CGTCCTTTTC CCTGACCTTC
                        ********** ********** ********** ********** **********
                        **********  ********* ********** ********** **********
                        **********  ********* ********** ********** **********
                        **********  ********* ********** ********** **********
                                    ********* ********** ********** **********
                                    ********* ********** ********** **********
                                    ********* ********** ********** **********
                                     ******** ****            ***** *********

alignment  position    1351...... 1361...... 1371...... 1381...... 1391......
P871935        1241    Ctttcccttc cgtctatTCC TCTCGCCCCA GGCCGTTACT GGGTAGATGT
P871938        1230    Cg-------- -------TCC ACTCGCCCCT GGCTGTTTCT GGGTAGTCGT
P871940        1207    Cga------- --------CC ACTCTCCCCT GGCTATTTCT GGGTAGTt-T
                        *                *** ********** ********** **********
                        *                *** ********** ********** **********
                        *                 ** ********** ********** *******   *
                        *                 ** ********** ********** *******   *
                        *                 ** ********** ********** *******   *
                        *                 ** ********** ********** *****
                        *

alignment  position    1401...... 1411...... 1421...... 1431...... 1441......
P871935        1291    Aaggagtgga gaggctgggg aagtgaccag agactcgtgg gttagacccg
P871938        1265    AGAGGGAAGA GCAGGATCGC A--------- ---------- ----------
P871940        1241    AGAGGGAAGA ACACGATCAC A--------- ---------- ----------
                        ********** ********** *
                        ********** ********** *
                        ********** ********** *
                        *
                        *

alignment  position    1451...... 1461...... 1471...... 1481...... 1491......
P871935        1341    tcTTGGGTTC TTAGTCACCA CCTCTAACCc tgtcggtcat tgatgaggtt
P871938        1286    --TTTGGTTC TTGGTCACTA CAGCTTACCG TTGCTTATgg ctatgact--
P871940        1262    --TTTGGTTC CTAGTCACTA CTGCTTACTG TTGCTTATtg a---------
                          ******** ********** ********** *******
                          ******** ********** ********** *******
                          ********     ******* *********
                          ********     ******* *********

alignment  position    1501...... 1511...... 1521...... 1531...... 1541......
P871935        1391    aGTAGCTCCG A--------- ---------- ---------- ----------
P871938        1332    -GTAGCTCTG ACGTAGGCTC CAGTGGGGGg ATCTTGGACA CCTGGAAAAC
P871940        1301    --TAGCTCTG ATGTAGGCTC TAGTGGGGG- ATCTCGAACA CCTGGAAACA
```

```
                          ********* ********** ********** ********** **********
                           ******** ********** ********** ********** **********
                           ******** ********** **********
alignment position        1551...... 1561...... 1571...... 1581...... 1591......
P871935              1402 ---------- ---------- ---------- ---------- ----------
P871938              1381 CTAGGTGGaa gGTGGCTTTT TGTTGTCATT TTGTTTtgta tcat------
P871940              1348 CTAGGCGGga aGTGGCTTTT TGTTGTCATT TTGTTTgtat catagtgaaa
                          ********    ********* ********** ******
                          ********    ********* ********** ******
                                      ********* ********** ******
                                      ********* ********** ******

alignment position        1601
P871935              1402 ----
P871938              1425 ----
P871940              1398 aaaa
```

Pairwise similarities:

For each pairwise alignment, the similarity (relative to the maximum similarity) and the number of identical nucleic acids (in % of shorter sequence) is given. Maximum values are underlined.

	P871938 (1424 bp)	P871940 (1401 bp)
**P871935 (1401 bp)**	0.131<br>36 %	0.103<br>35 %
**P871938 (1424 bp)**		1.000<br>84 %

Please note that the **similarity value 1.000** marks only the two **most similar** sequences, it does not necessarily mean that these sequences are identical.

**Extract alignment region**	
**Positions in alignment**	from: to:
**Output file**	dialign.seq

Extract aligned sequences

For comments, questions or bug reports, please contact support@genomatix.de

© Genomatix Software GmbH 1998-2005 - All rights reserved. License Agreement GEMS Launcher 4.1.1

Genomatix-Logo Logout Personal Messages GEMS-Logo

Genomatix_MainGEMSElDoradoGene2PromoterBiblioSphere
Frequently_asked_questionsYour_ResultsYour_SequencesYour_ProtocolHelp

GEMS Launcher Task: *DiAlign TF*: Multiple alignment plus TF sites
working on C4-COX8A (3 seq.)

[Alignment] [Pairwise_similarities]

DiAlign professional TF Release 3.1 December 2004 **Wed May 11 23:58:14 2005**

Solution parameters:

**Sequence file:**	C4-COX8A (3 seq.)
**Type of sequences:**	nucleotide sequences
**Threshold T:**	0.00
**'*' signs below alignment denote:**	diagonal similarity (max. similarity is represented by 10 '*' signs)
**Alignment output:**	complete alignment is shown<br># nucleic acids per line: 50
**TF output:**	common TF matches located in aligned regions (common to 3 (100.0 %) sequences)
**Family matches:**	yes
**MatInspector library:**	Matrix Family Library Version 5.0 (February 2005)
**Selected groups** (core/matrix sim):	• ALL user_defined.lib (0.75/Optimized)<br>• ALL vertebrates.lib (0.75/Optimized)

Aligned Sequences:

No.	Sequence Name	Sequence Description
1	P905108	P905108 sym=COX8A|loc=Loc1351|taxid=9606|spec=Homo sapiens|chr=11|ctg=NT_033903|str=(+)|start=9047274|end=9048374|len=1101|tss=601|comm=cytochrome c oxid subunit 8A (ubiquitous); (NM_004074/501/bronze;)
2	P905109	P905109 sym=Cox8a|loc=Loc12868|taxid=10090|spec=Mus musculus|chr=19|ctg=NT_082892|str=(-)|start=403413|end=404557|len=1145|tss=601,610,641,645|comm=cytoch c oxidase, subunit VIIIa; (AK008594/537/gold;AK002524/572/gold;AK002218/528/gold;NM_007750/568/bron
3	rCOX8A	rCOX8A n3_dna range=chr1:210046403-210047502 5'pad=0 3'pad=0 revComp=TRUE strand=? repeatMasking=n

Alignment (DiAlign format):

Please note that only **upper-case** letters are considered to be **aligned**. For more information, please have a look at the user guide.

V$SORY V$PCAT V$BCL6 V$CREB V$TCFF V$ETSF V$YY1F V$RORA V$EREF U$EREF V$SF1F V$AP2F

```
alignment
position          1 . . . . . . . . .  1 1 . . . . . . . .  2 1 . . . . . . . .  3 1 . . . . . . . .  4 1 . . . . . . . .
P905108         1 c t g g - - - - - -  - - - - - - - - - -  - - - - - - - - - -  - - - - - - - - - -  - - - - - - - - - -
P905109         1 g a A C A G T T A C  C A G T T A T G T G  G A A A C G G G T t  T C T G A G A G G A  T A A C A G T A A A
rCOX8A          1 g g A C A G T T A A  C A G T T A T G T G  G A A A C G G G T g  T C T G A G A G G A  A A A T A G T A A A
```

```
                   ******** ********** *********  ********** **********
                   ******** ********** *********  ********** **********
                   ******** ********** *********  ********** **********

alignment
position         51........ 61........ 71........ 81........ 91........

P905108      5   ---------- ---------- ---------- ---------- ----------
P905109     51   TGACAGGAGT CTCggGGCTG GAGAGAGAGC TCAGCGCTTA GAAGCACTc-
rCOX8A      51   TGACTGGAGT CTCa-GGCTG GAGAGATAGC TCAGTAGCTA AAAACACTgg
                 ********** ***  ***** ********** ********** ********
                 ********** ***  ***** ********** ********** ********
                 ********** ***

alignment
position         101....... 111....... 121....... 131....... 141.......

P905108      5   ---------- ---------- ---------- ---------- ----CAAGAC
P905109    100   GCTGCTCTTG CAGAGGTCCA GTTCAGTTCA GTTCCCAGCA tccaCAAGAT
rCOX8A     100   GCTGCTCTTA CAGAGGAACA GTTCAGTTCA GTTCCCAGCA cctacaggtc
                 ********** ********** ********** **********     ******
                 ********** ********** ********** **********
                 ********** ********** ********** **********
                 ********** ********** ********** **********

alignment
position         151....... 161....... 171....... 181....... 191.......

P905108     11   TGGTCTCAAA CTCCTGACCT Cagatgatct accggccccg gcctcccaaa
P905109    150   TACTCACTAT TGCCTGAACT Cca-GTTCCA GAGGATCCAA CACCCTCTTT
rCOX8A     150   t----ACTAT TGCTTGtgat tcctGTTCCA GAGGATCCAA CACCCTCTTT
                 ********** ********** *   ****** ********** **********
                                           ****** ********** **********
                                           ****** ********** **********
                                           ****** ********** **********
                                           ****** ********** **********

alignment
position         201....... 211....... 221....... 231....... 241.......

P905108     61   gtgttgggat tacatgcgtg agccactgcg cccggccaat ttttgtattt
P905109    199   TGGCCTCTCT GCGTtctgca ta-------- -CACAGATGA A---------
rCOX8A     196   TGGTCTCTCT GCGTactgca tgcaagtcgt gCATAGATGC A---------
                 ********** ****                   ********* *
                 ********** ****                   ********* *
                 ********** ****
                 ********** ****
                 ********** ****

alignment
position         251....... 261....... 271....... 281....... 291.......

P905108    111   ttcgtagaga tggggtttca cCATGCTGGC AAGACtggtg tcaaactcct
P905109    231   ---------- ---------- -CATGCTGAC AAAACactcA TAAAGTAAAA
rCOX8A     237   ---------- ---------- -CATGCAGAC AAAAC----A TAAAGTAAAA
                                        ********* *****    * **********
                                        ********* *****    * **********
                                                           * **********
                                                           * **********

alignment
position         301....... 311....... 321....... 331....... 341.......

P905108    161   gacctcagat gatctacccg ccccggcctc ccaaagtgct gggatTAGAG
P905109    260   CTATGTTTTT AAAAC----- ---------- ---------- -----TAGAG
rCOX8A     262   CAATGTTCTT AAAAC----- ---------- ---------- -----TAGAG
                 ********** *****                                *****
                 ********** *****                                *****
                 ********** *****                                *****
                 ********** *****                                *****

alignment
position         351....... 361....... 371....... 381....... 391.......

P905108    211   GCGTgagcca acgcgcccag cccgtacttt ttttttttTA GTACAGacgg
P905109    280   GCTTACTGgt ggcgAGCTGG AGTCAC---- --------TA GTACAGTGCT
```

```
rCOX8A      282 GCGTACTGt t  at gaAGCTGG AGTCAC----  ---------TA GTTGAGTGCT
                ********         ****** ******            ** **********
                ********         ****** ******            ** **********
                ********         ****** ******            ** **********
                ********         ****** ******            ** **********
                                 ****** ******            ** **********

alignment
position            401.......  411.......  421.......  431.......  441.......

P905108     261 cct cccaaaa gt tctgagat tacgggcgtg agccaccacg cccagcctga
P905109     318 GGC-------  ----------  ----------  ----------  ----------
rCOX8A      320 GGC-------  ----------  ----------  ----------  ----------
                ***
                ***
                ***
                ***
                ***

alignment
position            451.......  461.......  471.......  481.......  491.......

P905108     311 atAGAGAGAG at ttaacag-  -TATGAATGA AGGGAGGAGT CAAGAACAGT
P905109     321 --AGAGAGAG GATCCTAGTT  AAATGAATGG AGGGAGGAGC CAAGGAGTCA
rCOX8A      323 --AGAGAGAG GATCCTAGTT  ATATGAATGG AGGGAGGAGT TAAGAACTCA
                  ********  **********  **********  **********  **********
                  ********  **********  **********  **********  **********
                  ********  **********  **********  **********  **********
                  ********  *****         *********  **********  **********
                  ********  *****         *********  **********  **********
                                           *********  **********  **********
                                            ********  **********  ****

alignment
position            501.......  511.......  521.......  531.......  541.......

P905108     359 TTAGGCTTCT CGCGTACATG ATTGGGTGGC CCCAGATGTC ATTCACAAGT
P905109     369 ATAGACTTGT AGCTTGTGTG ATTGGGTGGC TATGCGTGTC ACTCTCGAag
rCOX8A      371 ATAGACTTCT CgGTTGTGTG ATTGGGTGGT TATACGTGTC ACTCTCGAGG
                ********** **********  **********  **********  **********
                ********** *   *******  **********  **********  ********
                ********** *   *******  **********  **********  ********
                *****          *******  **********  **********  ********
                *****              **  **********  **********  *
                *****

alignment
position            551.......  561.......  571.......  581.......  591.......

P905108     409 --Ataagcct  TGGAGGCGGA A-CATAActc gaagaagagc cctttt gt gC
P905109     419 aaAG------  TGGAGGTGGG AgCAAAGGTG TCTCATTT--  ---------C
rCOX8A      421 --AG------  TGGAGGCGGG A-CAAAAGTG TCTCATTT--  ---------C
                  **        ********** *  ********  ********            *
                                          ********  ********            *
                                          ********  ********            *
                                          ********  ********            *

alignment
position            601.......  611.......  621.......  631.......  641.......

P905108     456 TCCGCCCATa gc-G-TAGGA AGGTGTCaaT TGGCTGTTTC GAGGAACGCG
P905109     452 TCCTCCCATC TGTG-TAGGA TGATGTCGGT TGGTTGTTTC GAGGAGCGCG
rCOX8A      451 TCCTCCCATC TGTGgTAGGA TAAAGTTGGT TGGTAGTTTC AAGGAGCGCG
                ********** ****  *****  **********  **********  **********
                ********** ****                   *  **********  **********
                ********** ****                   *  **********  **********
                ********** ****                   *  **********  **********
                                                  *  **********  **********
                                                  *  **********    ********
                                                                   ********
                                                                   ********

alignment
position            651.......  661.......  671.......  681.......  691.......

P905108     504 CCAAAAACTG CCAAGGgctg tgggaggtg-  TGTTCTTGCG TCATTTCCGA
```

```
P905109   501 CCAAAAACAG CCCAGGCGGG CGAGGGggCT TGTCCCGACG TCATTTCCGA
rCOX8A    501 CCATAAACAG CCCAGGCGGG CCAGGGa-CT TGTCCTGACG TCATTTCCGA
              ********** ********** ******  ** ********** **********
              ********** ********** ******  ** ********** **********
              ********** ********** ******  ** ********** **********
              ********** ********** ******  ** ********** **********
              ********** ******                ********** **********
              ********** ******                        ** **********
              ********** ******                        ** **********
              ********** ******

alignment
position          701....... 711....... 721....... 731....... 741.......
P905108   553 GAGACTTCCG CGCcgcagtt tccctgcttc ccCAGCTCCA GAACTTCCGG
P905109   551 AATACTTCCG Gctcg-GGCG TCCGGCAACT T-CAGCGCCG GAACTTCCGG
rCOX8A    550 AATACTTCCG GGCtgaGGCG TCTATCAACT T-CAGCGCCG GAACTTCCGG
              ********** ***   **** ********** *  ******** **********
              ********** ***   **** ********** *  ******** **********
              ********** *     **** ********** *  ******** **********
              ********** *                        ******** **********
              ********** *                                 **********
              **********                                   **********
              **********

alignment
position          751....... 761....... 771....... 781....... 791.......
P905108   603 CCAGCGCAGC CATTTTGGCT TCCTGACCTT GGGCTACGGC TGaccgtttt
P905109   599 CTGGTTCGGC CATCTTGACT CCCTGACCTT GGa------C TGGTCCTCGT
rCOX8A    599 CTattGCGGC CATCTTGACT TCCTGACCTT GGGCTTCGGC TGGTCCTTGT
              ********** ********** ********** ********** **********
              ********** ********** ********** ********** **********
              **   ***** ********** ********** ********** **********
              **   ***** ********** ********** **       * **
              **   ***** ********** ********** **       * **
              *     **** ********** ********** **       * **
                    **** ********** **********

alignment
position          801....... 811....... 821....... 831....... 841.......
P905108   653 ttgtggtgtA CTCCGTGCCA TCATGTCCGT CCTGACGCCG CTGCTGCTGc
P905109   643 CCGTGGTC-G TTCCGCGCCG TCATGTCTGT CCTGACGCCA CTGCTGCTGA
rCOX8A    649 CCTTGGTC-A CTCCGCGCCG TCATGTCTTC CCTGACGCCG CTGCTGCTGA
              ******** * ********** ********** ********** **********
              ******** * ********** ********** ********** **********
              ******** * ********** ********** ********** **********
                       * ********** ********** ********** **********
                       * ********** ********** ********** **********
                       * ********** ********** ********** *********
                          ********* ********   ********** *********
                          ********* ********   ********** *********
                          ********* ********   ********** *********
                                               ********** *********

alignment
position          851....... 861....... 871....... 881....... 891.......
P905108   703 GGGGCTTGAC AGGCTCGGCC CGGCGGCTCC CAGTGCCGCG cgccaagaTC
P905109   692 GGAGCCTGAC CGGCTCGGCC CGGCGGCTCA TGGTGCCGCG GGCTCAGGTC
rCOX8A    698 GGAGCCTGAC CGGCCCGGCC CGGCGGCTTA TGGTGCCGCG GGCTCAGGTC
              ********** ********** ********** ********** **********
              ********** ********** ********** ********** **********
              ********** ********** ********** ********** **********
              ********** ********** ********** ********** **********
              ********** ********** ********** ********** **********
              ********** ********** ********** **********         **
              ********** ********** ********** **********         **
              ********** ********** ********** **********
              ********** ********** ********** **********
                                    ********** **********

alignment
position          901....... 911....... 921....... 931....... 941.......
```

```
P905108      753 CATTCGTTGC CGCCGGAGGG GAAGCTTGGG ATCATGGT-- GAGGAACGGG
P905109      742 CACTCGAAGC CGGCGCGGGA GCAGCTCGGG GTCCTGGTGA GCGGGACGGA
rCOX8A       748 CACTCGAAGC CGCCGCGGGA GCAGCTCGGG GTCCTGGTGA GCGGGACGGA
                 ********** ********** ********** ********** **********
                 ********** ********** ********** ********** **********
                 ********** ********** ********** ********** **********
                 **********    ******* ********** ********** **********
                 **********    ******* ********** ********** **********
                 **********    ******* ********** ********
                 **********    ******* ********** ********
                               ******* ********** ********

alignment
position             951....... 961....... 971....... 981....... 991.......

P905108      801 CCTGGAAGAG CGCGGGAGGC GCCGTGGGCT gaccccttct gcctagctga
P905109      792 CCGGGAGCGG GGAGGTGGGC GAACTGTGGT AGCCTGCGAC ATT-------
rCOX8A       798 GCGGGAGCGG GGAGGTGGGC GAACTGGGGT AGCCTGCGAC ATT-------
                 ********** ********** ********** ********** ***
                  ********* ********** ********** ********** ***
                  ********* ********** ********** ********** ***
                  ********* ********** ********** ********** *
                  ********* ********** ********** ********** *

alignment
position             1001...... 1011...... 1021...... 1031...... 1041......

P905108      851 cctcaggtgg tcctgagaat gcctcgcgcc ccgcagcgta gcggggagca
P905109      835 ---------- ---------- ---------- ---------- ----------
rCOX8A       841 ---------- ---------- ---------- ---------- ----------

alignment
position             1051...... 1061...... 1071...... 1081...... 1091......

P905108      901 gcagtgccGG TTTGGGCATG TCTGGAGGGC GCGAACGAcg gggacagtgc
P905109      835 --------GG CTTAGGCCTG TCCTCAAGGC ATGGATGA-- ----------
rCOX8A       841 --------GG CTTGGGCCTG TCTGCAAGGC GTGGATGA-- ----------
                         ** ********** ********** ********
                         ** ********** ********** ********
                         ** ********** ********** ********
                         ** ********** ********** *****

alignment
position             1101...... 1111...... 1121...... 1131...... 1141......

P905108      951 ccgggcGACA GTCGgggctg tcg------- GCGTTAGGTC TTTCCCCACA
P905109      865 ------GACA GTCGctcgg- CTGCTCCTAA GCCCTAGGTA TTTTTCCCAA
rCOX8A       871 ------GACA agttgctcga CTGCTCCTAA ACCCTAGTTA TTTTTCCCAA
                       **** ****       ********** ********** **********
                       ****            ********** ********** **********
                       ****            ********** ********** **********
                                       ********** ********** **********
                                                  ********** **********

alignment
position             1151...... 1161...... 1171...... 1181...... 1191......

P905108      994 GCCCCAGGAT CTCGGGTCTG gtcctgtcag gcc------- ----------
P905109      908 GTTCCTGGAT GTCAGGTCAG CCCGCTAGTT TGTTTACT-- CTGTTCGGCC
rCOX8A       915 GTTCCTGGca -------CTG CCAGCCGGTC AGTTTACTcg CTGTTCGGCC
                 ********** ********** ********** ********   **********
                 ********                                    **********
                 ********                                    **********
                 ********
                 ********

alignment
position             1201...... 1211...... 1221...... 1231...... 1241......

P905108     1027 ---------- ---CTCCCTC TCCTGTCGCC TCCTCGCTGC GCCGCctcct
P905109      956 TCTCTAAACC CGTCTCTCCT TCCATTCCCC CCCCCCCCCC ACCGCagGGG
rCOX8A       958 TCTCTAAACC GGTCTCTCTT Tttaattttt tttgcgt--- -------GGG
```

```
                   ********** ********** ********** ********** ******-***
                   ********** ********** *
                   ********** ********** *

alignment
position           1251...... 1261...... 1271...... 1281...... 1291......

P905108     1064   gccgacttct ttagccgctc tgagcccatt ttctcgtt-- ----------
P905109     1006   AGGGCATGCC TGGTAACTGG CAGCgacgtc atggttgtta ctgctacatt
rCOX8A       998   AGAAAATGCC TAGTAATTGG CAGGg----- ---------- ----------
                   ********** ********** ****

alignment
position           1301...... 1311...... 1321...... 1331...... 1341......

P905108     1102   ---------- ---------- ---------- ---------- ----------
P905109     1056   ttattattaa ggagggcTGC AGAGTt--CA CCAGGTCTac a---TTCCCA
rCOX8A      1023   ---------- -------TGC AGAGTaccCA CCAGGTCTtt ggggTTCCCA
                          *** *****   ** ********     ******

alignment
position           1351...... 1361...... 1371...... 1381...... 1391.

P905108     1102   ---------- ---------- ---------- ---------- -----
P905109     1101   GAgATGTAGG AGCATTTAGA TACTAGGACA ATTTTTACTT Ttaaa
rCOX8A      1056   GAaATGTAAG ACCATTTAGA TATTAGGACA ATTTTTACTT Tcaaa
                   ** ******* ********** ********** ********** *
                      ******* ********** ********** ********** *
                      ******* ********** ********** ********** *
                      ******* ********** ********** ********** *
```

Pairwise similarities:

For each pairwise alignment, the similarity (relative to the maximum similarity) and the number of identical nucleic acids (in % of shorter sequence) is given. Maximum values are underlined.

	P905109 (1145 bp)	rCOX8A (1100 bp)
**P905108 (1101 bp)**	0.184<br>34 %	0.204<br>34 %
**P905109 (1145 bp)**		<u>1.000</u><br><u>80 %</u>

Please note that the **similarity value 1.000** marks only the two **most similar** sequences, it does not necessarily mean that these sequences are identical.

Extract alignment region	
**Positions in alignment**	from: to:
**Output file**	dialign.seq

Extract aligned sequences

For comments, questions or bug reports, please contact support@genomatix.de

© Genomatix Software GmbH 1998-2005 - All rights reserved. License Agreement GEMS Launcher 4.1.1

Genomatix-Logo Logout Personal Messages GEMS-Logo

Genomatix_MainGEMSElDoradoGene2PromoterBiblioSphere
Frequently_asked_questionsYour_ResultsYour_SequencesYour_ProtocolHelp

GEMS Launcher Task: *DiAlign TF*: Multiple alignment plus TF sites
working on C4-COX8C (3 seq.)

[Alignment] [Pairwise_similarities]

DiAlign professional TF Release 3.1 December 2004 **Thu May 12 17:46:32 2005**

Solution parameters:

**Sequence file:**	C4-COX8C (3 seq.)
**Type of sequences:**	nucleotide sequences
**Threshold T:**	0.00
**'*' signs below alignment denote:**	diagonal similarity (max. similarity is represented by 10 '*' signs)
**Alignment output:**	complete alignment is shown<br># nucleic acids per line: 50
**TF output:**	common TF matches located in aligned regions (common to 3 (100.0 %) sequences)
**Family matches:**	yes
**MatInspector library:**	Matrix Family Library Version 5.0 (February 2005)
**Selected groups** (core/matrix sim):	• ALL user_defined.lib (0.75/Optimized)<br>• ALL vertebrates.lib (0.75/Optimized)

Aligned Sequences:

No.	Sequence Name	Sequence Description	Len[illegible]
1	P304066	P304066 sym=COX8C|loc=Loc341947|taxid=9606|spec=Homo sapiens|chr=14|ctg=NT_026437|str=(+)|start=74812790|end=74813790|len=1001|tss=501|comm=cytochrome c oxidase subunit 8C; (NM_182971/501/bronze;)	1[illegible]
2	mCOX8C	mCOX8C m6_dna range=chr12:98345092-98345877 5'pad=0 3'pad=0 revComp=FALSE strand=? repeatMasking=none	786
3	rCOX8C	rCOX8C n3_dna range=chr6:127164794-127165676 5'pad=0 3'pad=0 revComp=FALSE strand=? repeatMasking=none	883

Alignment (DiAlign format):

Please note that only **upper-case** letters are considered to be **aligned**. For more information, please have a look at the user guide.

V$NRF1 U$NRF1 V$CP2F V$AP4R V$EKLF

```
alignment position  1 . . . . . . . . .   11 . . . . . . . .   21 . . . . . . . .   31 . . . . . . . .   41 . . . . . . . .
P304066          1  gccaatgcca  gggcatgtta  agaggtatat  aaagtatagg  tagagaggaa
mCOX8C           1  GTAAAAATGG  TATGATATCT  GCTATTGACT  A---------  ----------
rCOX8C           1  GTAAAAATGG  TATGATATCT  GCTATTAACT  G---------  ----------
                    **********  **********  **********  *
                    **********  **********  **********  *
                    **********  **********  **********  *
                    **********  **********  **********  *
                    **********  **********  **********  *
```

```
alignment position 51........ 61........ 71........ 81........ 91........
P304066       51 gacAAAGGAA AGGAGTAggg gtgccaa--- ---------- ----------
mCOX8C        32 ---AAAGCAA CGGAATACTG AGCAATAGCT ATAGATGTTG GCAGCCAAT-
rCOX8C        32 ---AAAttag tGGGATACTG AGCAACACCT ATAGATGTTG GCAGCCAATa
                    ******* ********** ********** ********** *********
                    ***      ********* ********** ********** *********
                    ***      ********* ********** ********** *********
                    ***      ********* ********** ********** *********
                    ***      ********* ********** ********** *********

alignment position 101....... 111....... 121....... 131....... 141.......
P304066       78 -GTCCCTCAC CTTACCctgc agaaaag--- -----AGATA CTTAGTGTGa
mCOX8C        78 GGTCCCTCAC CTTgtgTTAG GAGAAGAAAA TCAGAAAATC CTGAGTGTGG
rCOX8C        79 GGTCCTTCAC CTTACCTTAG GAGAAGAAAA TCAGAAGACC CTGAGTGTGG
                 ********** ********** ********** ********** **********
                  ********* ***   **** ********** ********** **********
                                  **** ********** ********** **********
                                  **** ********** ********** **********
                                  **** ********** ********** **********
                                  **** ********** ********** **********

alignment position 151....... 161....... 171....... 181....... 191.......
P304066      119 ggtggggTTG GGGCTCAGGG GAGGGTGGCA GGAGAGTTTA GGAGAGGATA
mCOX8C       128 C------TTT GGgact-GAA TAGGTTTGGC AAAGAGTTTG GGAGAGGGCA
rCOX8C       129 C------TTT GGGGGCCGAA TAGGTTTGGT AAAGACTTTG GGAGAGGGCA
                 *      *** ********** ********** ********** **********
                 *      *** **      *** ********** ********** **********
                 *      *** **      *** ********** ********** **********
                 *      *** **      *** ********** ********** **********
                 *      *** **      *** ********** ********** **********
                 *      *** **      *** ********** ********** **********
                        *** **           ********* ********** **********

alignment position 201....... 211....... 221....... 231....... 241.......
P304066      169 GGAc-----A CGTGGGTGGA AAGTGAGGTG AATGAGGgct gggaggttgc
mCOX8C       171 GGAAGCTGGA CTTGTGAGGA CATAGGTGGG AAGAAGGTAG ----------
rCOX8C       173 GGTAGCTGGA CTTGTGGGGA CAGGGGCGGG AAGCATGTAG ----------
                 ********** ********** ********** **********
                 ********** ********** ********** **********
                 ********** ********** ********** **********
                 *******
                 *******
                 *******
                 ***

alignment position 251....... 261....... 271....... 281....... 291.......
P304066      214 agggagccct gagtggaaaa ggcggggcag agcagggggt actgaactga
mCOX8C       211 ---------- ---------- ---------- ---------- ----------
rCOX8C       213 ---------- ---------- ---------- ---------- ----------

alignment position 301....... 311....... 321....... 331....... 341.......
P304066      264 agagagtgga gcagagcagg ggtgtgctgg gtggaagggg tggtgcagag
mCOX8C       211 ---------- ---------- ---------- ---------- ----------
rCOX8C       213 ---------- ---------- ---------- ---------- ----------

alignment position 351....... 361....... 371....... 381....... 391.......
P304066      314 caagtggtgc taagcAGAGG GGGTGGGGCA AAGCACGGAC TGCTGAGtgg
mCOX8C       211 ---------- -----AGAGT GGCAGGGCCA AAACACATAC AGCGGAGAA-
rCOX8C       213 ---------- -----AGTGT Ga-TAGGGCG CAGCATTTAC AGCTGAGGA-
                                 ***** ********** ********** *********
                                 ***** *   ******* ********** *********
                                 ***** *                 *** *********
                                 ***** *                 *** *********
                                                         *** *********
                                                         *** *******

alignment position 401....... 411....... 421....... 431....... 441.......
```

```
P304066   364 aaagggcagt acagagaaag gagtggtgag cgGAAGAGGC GGGGCAGAGC
mCOX8C    245 ---------- ---------- ---------- --GCAGGGTC GGGTAAGAGC
rCOX8C    246 ---------- ---------- ---------- --GCAGGGTG GGGTAAGAGC
                                                ******** **********
                                                ******** **********
                                                ******** **********
                                                ******** **********
                                                ******** **********

alignment position 451....... 461....... 471....... 481....... 491.......
P304066   414 aggaggtgct gggcagaagg ggtggggtag ggggcgaggg cagagcgccC
mCOX8C    263 TCAAATTCCA tgCAAAGGG- ---------- ---------- ---------C
rCOX8C    264 GCAAATTCCA ttCAAAGGG- ---------- ---------- ---------C
              **********   *******                                *
              **********   *******                                *
              **********   *******                                *
              **********   *******                                *
              **********   *******                                *
                           *******                                *
                                                                  *
                                                                  *

alignment position 501....... 511....... 521....... 531....... 541.......
P304066   464 CCTCGTGGCT GGGGCGCCTG CGCAAACCAG CTGCCtcacg agcactggag
mCOX8C    283 TTGAGTGGAT GGTGCGCATG CTCAAACCAG CTGCCCGCC- ----------
rCOX8C    284 CCGAGTGGTT GGTGCGCATG CTCAAACCAG CTGCTCGCC- ----------
              ********** ********** ********** *********
              ********** ********** ********** *********
              ********** ********** ********** *********
              ********** ********** ********** *********
              ********** ********** ********** *********
              ********** ********** ********** *****
              ********** ********** ********** *****
              ********** ********** ********** *****
                  ****** ********** ********** ****
                  ****** ********** ********** ****

alignment position 551....... 561....... 571....... 581....... 591.......
P304066   514 cttgCGTTAC TTGGCCTCAC CTCACCtgtg ct-------- ----------
mCOX8C    322 ----CTTTCC TCGCGCACAC TTTACCAACG GCCTTtgact cCGACACTGG
rCOX8C    323 ----CTCTCC TCGAGCACAC TTTACCAACG GCCTT----- -CGACTCTGG
                  ****** ********** ********** *****       *********
                  ****** ********** ********** *****       *********
                  ****** ********** ********** *****       *********
                  ****** ********** ********** *****
                  ****** ********** ********** *****

alignment position 601....... 611....... 621....... 631....... 641.......
P304066   546 --GTCCACGC CTGGCTTTGT CTCAcctgac gcgatatgc- ---------C
mCOX8C    368 TGGTCTCCGG TTTGCTCTGT CTCAGTGGCC CTTTTCTGAC CTGCGATGTC
rCOX8C    363 TGGTCTCCCA TTTGCTCTTC CTCgGTGGCC CTTTTCTGAC CTGTGATGTC
              ********** ********** ********** ********** **********
              ********** ********** *** ****** ********** **********
              ********** ********** *** ****** ********** **********
                                        ****** ********** **********
                                        ****** ********** **********
                                        ****** ********** **********

alignment position 651....... 661....... 671....... 681....... 691.......
P304066   584 TCTCCTGCGT GGGCGCTGTC CTGCCCGCCG CCACTACCGc cgcttggccc
mCOX8C    418 TCGCCTGCTG CTACTCTGCT CTTCGCTCCT CCGCCACCGT GCAGTCCTGT
rCOX8C    413 TCGCTTGCTG CAGTTCTGCT CTTCCCTCCT CCGACACCGT GTAGTCCTGT
              ********** ********** ********** ********** **********
              ********** *   ****** ********** ********** **********
              ********** *   ****** ********** ********** **********
              ********** *   ****** ********** ********** **********
              ********** *   ****** ********** ********** **********
              ********** *   ****** ********** ********** **********

alignment position 701....... 711....... 721....... 731....... 741.......
```

```
P304066     634 tgctcggcct gcagcccgct ccccgcttcG CCCACTCGGG GCCCCCGCGC
mCOX8C      468 TCTCGAAGCC T--------- ---------G GCCACCCAGG CCGCCTCAGC
rCOX8C      463 TCTCGAAGCC T--------- ---------G GCCACTCAGG CCGCCTCAGC
                ********** *                   * ********** **********
                ********** *                   * ********** **********
                ********** *                   * ********** **********
                ********** *                   * ********** **********
                ********** *                   * ********** **********
                ********** *                   * ********** **********
                    ****** *                   * ********** **********

alignment position 751....... 761....... 771....... 781....... 791.......
P304066     684 CAgcggcc-- ---------- ------CCTG TCTGCCGCGG TGAGTTGAGG
mCOX8C      500 CACTCAGAAA GCCCACAAAA AAAAATCCTG TCGCCCACGG TGAGGTAGAA
rCOX8C      495 CACTCAGAAA GCCCACAAAA CCAAGTCCTG ACACCCACGG TGAGGTAGAg
                ********** ********** ********** ********** **********
                ********** ********** ********** ********** *********
                ********** ********** ********** ********** *********
                ********** ********** ********** ********** *********
                ********** **
                ********** **
                ********** **

alignment position 801....... 811....... 821....... 831....... 841.......
P304066     716 CCCAgccatc atggtgggcg ggaagcgcgt ggccctggcg gggcGCCCCG
mCOX8C      550 CCCActTAAC G--------- ---------- ---------- ----GTCCAA
rCOX8C      545 tccgccTAAC G--------- ---------- ---------- ----GTCCAA
                ****  **** *                                   ******
                      **** *                                   ******
                      **** *                                   ******
                      **** *                                   ******
                      **** *                                   ******

alignment position 851....... 861....... 871....... 881....... 891.......
P304066     766 ACGGGTGGGG AGAAGGGAGG ACACGGCGT- ---------G caggcctcgc
mCOX8C      567 ATGGGTGTGG AGGGGGGAGT GTGTGGTGT- ---------G T---------
rCOX8C      562 GTGGGTGGGG ATTGGGGAGT GTGTGGTGTg tggtgtgtgG T---------
                ********** ********** *********           * *
                ********** ********** *********
                ********** ********** *********
                ********** ********** *********
                ********** ********** *********

alignment position 901....... 911....... 921....... 931....... 941.......
P304066     806 GTGGGAGGCT CTTGTGGCTT GGTCGCCGTT GGGGGAGGTt cctgtggc--
mCOX8C      598 GTGTGTGgtt --------GT GTGTGTGGTT GTGTGTGTTG GCAGCTTCAG
rCOX8C      603 GTGTGTGTTA GGTGTGGCGT GTGTGTTGTA GTGTGTGTTG GCAACTTCAG
                ********** ********** ********** ********** **********
                                   ** ********** ********** **********
                                   ** ********** ********** **********
                                   ** ********** ********** **********
                                   ** ********** ********** **********

alignment position 951....... 961....... 971....... 981....... 991.......
P304066     854 ---TTGGTCG CCTTTGGGGG AGGTTCCTGG GAGAGGGTcg cggtgaggat
mCOX8C      640 TTGTGGGgtc ACTTTGGATG GCGGTTCAGG GCGAGGATga GAGTCCCAAG
rCOX8C      653 TTGTGGGTTT ACTTTGGATG GGGGCTCAGA GTGAGGGT-- GAGTCCCAGG
                ********** ********** ********** ********   **********
                *******    ********** ********** ******     **********
                *******                                     **********
                *******                                     **********
                *******

alignment position 1001...... 1011...... 1021...... 1031...... 1041......
P304066     901 gccgtcgtcg ---------- ---------- GGCACAGGGg cggaaagccc
mCOX8C      690 TAGGACCTGG CTCTCCATTA TG------GA GGCACAGGG- ----------
rCOX8C      701 TAGGACCTGG CCCTCCTTTA TGagcatgGA GGTGCAGGA- ----------
```

```
                       ********** ********** **               ** *********
                       ********** ********** **               ** *********
                       ********** ********** **
                       ********** ********** **

alignment position     1051...... 1061...... 1071...... 1081...... 1091......
P304066           931  gggaccctga ggaacgcgcg gggattgggc cTCCTTCGTT GTTACCCTTT
mCOX8C            723  ---------- ---------- ---------- -TCCTCGGTT CACTCCAGGG
rCOX8C            740  ---------- ---------- ---------- -TCCTTGGTT CGTTCCAGGG
                                                         ********* **********
                                                         ********* **********

alignment position     1101...... 1111...... 1121...... 1131...... 1141......
P304066           981  ACCGGCACCT GGCTCGGGGG C--------- ---------- ----------
mCOX8C            742  TAATcTACCT GCCTGGTGGC CAAGACATAG CATTTTCTCT AAAAA-----
rCOX8C            759  TCATTTACCT GTCTTGTGGC CGAGCCATAG CATTTTCTCT AAAAAacgat
                       ********** ********** ********** ********** *****
                       ****  ***** ********** ********** ********** *****
                            ***** ********** ********** ********** *****
                            ***** ********** ********** ********** *****
                            ***** ********** ********** ********** *****

alignment position     1151...... 1161...... 1171...... 1181...... 1191......
P304066          1002  ---------- ---------- ---------- ---------- ----------
mCOX8C            787  ---------- ---------- ---------- ---------- ----------
rCOX8C            809  cgggacacta gacatcttcg agctaccaga atctagacgt cagaaattgg

alignment position     1201...... 1211...... 1221.
P304066          1002  ---------- ---------- -----
mCOX8C            787  ---------- ---------- -----
rCOX8C            859  caactattga aagaaattga gtttt
```

Pairwise similarities:

For each pairwise alignment, the similarity (relative to the maximum similarity) and the number of identical nucleic acids (in % of shorter sequence) is given. Maximum values are underlined.

	**mCOX8C (786 bp)**	**rCOX8C (883 bp)**
**P304066 (1001 bp)**	0.079<br>34 %	0.053<br>27 %
**mCOX8C (786 bp)**		<u>1.000</u><br><u>83 %</u>

Please note that the **similarity value 1.000** marks only the two **most similar** sequences, it does not necessarily mean that these sequences are identical.

**Extract alignment region**	
**Positions in alignment**	from: to:
**Output file**	dialign.seq

Extract aligned sequences

For comments, questions or bug reports, please contact support@genomatix.de

© Genomatix Software GmbH 1998-2005 License Agreement GEMS Launcher 4.1.1

- All rights reserved.

Genomatix-Logo Logout Personal Messages GEMS-Logo

Genomatix_MainGEMSElDoradoGene2PromoterBiblioSphere
Frequently_asked_questionsYour_ResultsYour_SequencesYour_ProtocolHelp

GEMS Launcher Task: *DiAlign TF*: Multiple alignment plus TF sites
working on promoters.seq (2 seq.)

[Alignment] [Pairwise_similarities]

DiAlign professional TF Release 3.1 December 2004 **Thu May 12 18:22:09 2005**

Solution parameters:

**Sequence file:**	promoters.seq (2 seq.)
**Type of sequences:**	nucleotide sequences
**Threshold T:**	0.00
**'*' signs below alignment denote**:	diagonal similarity (max. similarity is represented by 10 '*' signs)
**Alignment output:**	complete alignment is shown<br># nucleic acids per line: 50
**TF output:**	common TF matches located in aligned regions (common to 2 (100.0 %) sequences)
**Family matches:**	yes
**MatInspector library:**	Matrix Family Library Version 5.0 (February 2005)
**Selected groups** (core/matrix sim):	• ALL user_defined.lib (0.75/Optimized)<br>• ALL vertebrates.lib (0.75/Optimized)

Aligned Sequences:

No.	Sequence Name	Sequence Description	Length
1	P862017	sym=COX10|loc=Loc1352|taxid=9606|spec=Homo sapiens|chr=17| ctg=NT_010718|str=(+)|start=13569068|end=13570568|len=1501| tss=1001| comm=COX10 homolog, cytochrome c oxidase assembly protein, heme A; (NM_001303/501/silver;)	1501 bp
2	P862018	sym=Cox10|loc=Loc70383|taxid=10090|spec=Mus musculus|chr=11| ctg=NT_096135|str=(-)|start=29162492|end=29164044|len=1553| tss=1001,1053| comm=COX10 homolog, cytochrome c oxidase assembly protein, heme A; (AK010385/501/gold;NM_178379/553/bronze;)	1553 bp

Alignment (DiAlign format):

Please note that only **upper-case** letters are considered to be **aligned**. For more information, please have a look at the user guide.

V$ETSF U$NRF2 V$YY1F V$NEUR V$ZF5F V$STAF V$SORY V$EBOX V$RORA V$COUP

```
alignment position  1 . . . . . . . . .   11 . . . . . . . .   21 . . . . . . . .   31 . . . . . . . .   41 . . . . . . . .
P862017          1  a - - - - - - - - -   - - - - - - - - - -   - - - - - - - - - -   - - - - - - - - - -   - - - - - - - - - -
P862018          1  a a g g g c t a a c   t t a t t t a t g a   t g a t c a g a g t   t c c a g g a a c a   g t g g g t c t a a

alignment position  51 . . . . . . . .   61 . . . . . . . .   71 . . . . . . . .   81 . . . . . . . .   91 . . . . . . . .
P862017          2  - - - - - - - - - -   - - - - - - - - - -   - - - - - - - - - -   - - - - - - - - - -   - - - - - - - - - -
P862018         51  t t a a c c a a g a   t a g c t g t a a a   g c a g a a g g c c   t c c c c t a a g c   a g c a c t c a c t
```

```
alignment position 101....... 111....... 121....... 131....... 141.......
P862017          2 ---------- ---------- ---TCTACTT TTTACTTGTG GAaccccaat
P862018        101 gttggtgtga ttagatccca aatTGTGCTT TTCATTTGTT GAtttaagg-
                                             ******* ********** **

alignment position 151....... 161....... 171....... 181....... 191.......
P862017         29 ctgaaaagaa gatcgagggg ttgggggatg ctgagatcta ccctggtaat
P862018        150 ---------- ---------- ---------- ---------- ----------

alignment position 201....... 211....... 221....... 231....... 241.......
P862017         79 cacaacactt atgataacac aggtaataat ctattagtgc ctgtacctag
P862018        150 ---------- ---------- ---------- ---------- ----------

alignment position 251....... 261....... 271....... 281....... 291.......
P862017        129 taggtgtctg gctacccatg aaacacattt atctaattct ctcaacctct
P862018        150 ---------- ---------- ---------- ---------- ----------

alignment position 301....... 311....... 321....... 331....... 341.......
P862017        179 tataaaagat gtaccattac tactccattg tattgctaga taactgaggt
P862018        150 ---------- ---------- ---------- ---------- ----------

alignment position 351....... 361....... 371....... 381....... 391.......
P862017        229 tcagaggact taggtaattt gctccaacaa ggtcaaaagg ccagaactag
P862018        150 ---------- ---------- ---------- ---------- ----------

alignment position 401....... 411....... 421....... 431....... 441.......
P862017        279 aatacggatt tcactccaga ccaccaaatc tgaacctcta catgcaatct
P862018        150 ---------- ---------- ---------- ---------- ----------

alignment position 451....... 461....... 471....... 481....... 491.......
P862017        329 ggagatatct ggatgattca gttcctttag tgtggctcag aaacaagtaa
P862018        150 ---------- ---------- ---------- ---------- ----------

alignment position 501....... 511....... 521....... 531....... 541.......
P862017        379 cccggagttc ctctgggctc ccatttgata gagactacgg taaagtgtaa
P862018        150 ---------- ---------- ---------- ---------- ----------

alignment position 551....... 561....... 571....... 581....... 591.......
P862017        429 ttgattagac gtggaggtgg aagcatcccc acgagaacca tttcctcatg
P862018        150 ---------- ---------- ---------- ---------- ----------

alignment position 601....... 611....... 621....... 631....... 641.......
P862017        479 atgctcctgt caccgggaag tgttctaatt ttaactttaT TTTTATTTTA
P862018        150 ---------- ---------- ---------- ---------T TTGTACTTTG
                                                            * **********

alignment position 651....... 661....... 671....... 681....... 691.......
P862017        529 AAATTTATTT TTAGAGACAG GGTCTCActg tgtcgcccag actgcagtgc
P862018        161 TAATATTTCA CTTAACACAG CATTTCAaag tgctcgaaaa acagtggatt
                   ********** ********** *******

alignment position 701....... 711....... 721....... 731....... 741.......
P862017        579 agtggcgcaa tcaccgcaac ctcgacctcc caggctcaag cgatccTCCC
P862018        211 gaacagttgt taacttttca gttattcacc agaaa----- ------TCCA
                                                                  ****

alignment position 751....... 761....... 771....... 781....... 791.......
P862017        629 GCCTCAGCCT CCTGAGTagc tgagaccaca ggcacgcgcc accacacctg
```

```
P862018       250 TCCTCTTCTT CCTTAGTcta agattaattt gatggggaca accattatcc
                  ********** *******

alignment position 801....... 811....... 821....... 831....... 841.......
P862017       679 gctaatttta cattttttgt agaggcgggg tctcgctttg ttgcccaggc
P862018       300 taggtccaca gtgatgaact gtgatgggcc tcagaaaatc ccattctctg

alignment position 851....... 861....... 871....... 881....... 891.......
P862017       729 tggtctcgag ttcccaggat caagcgatc- ---------- ----------
P862018       350 ctctgtcttc ctggtaaact ggaaaactct cacagttcct tctactgagc

alignment position 901....... 911....... 921....... 931....... 941.......
P862017       758 ---------- ---------- ---------- ---------- ----------
P862018       400 agcaagttgg ctcttcaaag cctttcttta gcctacttca cagaaatgct

alignment position 951....... 961....... 971....... 981....... 991.......
P862017       758 ---------- ---------- ---------- ---------- ----------
P862018       450 aacctcattc catagcgctt tctaccttat ccctatcggg atgacgctca

alignment position 1001...... 1011...... 1021...... 1031...... 1041......
P862017       758 ---------- ---------- ---------- ---------- --------CT
P862018       500 ccatgaccag ttcttgctgc agagacacag tgcgtgctct tacacaaaCT
                                                                      **
alignment position 1051...... 1061...... 1071...... 1081...... 1091......
P862017       760 CCCGCCTCGG TCTCCCtaag cactgggatt gcaggcGTGA GCCACAGcgc
P862018       550 CCTGTCTCAG TGGCCCctga tcagcacctg ag----GTGA ACCACAGaga
                  ********** ******                      **** *******
alignment position 1101...... 1111...... 1121...... 1131...... 1141......
P862017       810 ccggccgctg taactttaaa ttgcccggga ggactctgca gcccaaccct
P862018       596 tctcacctca tcgctgaaag caaacacaaa ctgcccttgt aagtcatgtt

alignment position 1151...... 1161...... 1171...... 1181...... 1191......
P862017       860 gcccacctca cccggtcacc ggaggatgc- ---------- ----------
P862018       646 ttgctctttt aatgagtttc tttcttcctc gtttttttac ttaccaaact

alignment position 1201...... 1211...... 1221...... 1231...... 1241......
P862017       889 ---------- ---------- ---------- ---------- ----------
P862018       696 ccacgagaag ccaacagcct gcgcaagatc agcctgggtg acaactgagg

alignment position 1251...... 1261...... 1271...... 1281...... 1291......
P862017       889 ---------- --CTGTCAAC TTCTGGGAGC TGCAAGCGCG GGCCGGCCag
P862018       746 aggcattcaa ttCTTTCAAG CTCAGGGGAC TGCCGGGCCG GGCCGGACtc
                               ******** ********** ********** ********
                               ******** ********** ********** ********
                               ******** ********** ********** ********
alignment position 1301...... 1311...... 1321...... 1331...... 1341......
P862017       927 tcgtccgcgc gcggcttttt cttcaggaca gcggaggcac accgtgcttt
P862018       796 ---------- ---------- ---------- ---------- ----------

alignment position 1351...... 1361...... 1371...... 1381...... 1391......
P862017       977 gcggttcccg gaagtccttc aggcctCAGA CCTGTCAGCC CGCCACactc
P862018       796 ---------- ---------- ------CAGA GCCCTAACCC CGCCACgact
                                                **** ********** ******
alignment position 1401...... 1411...... 1421...... 1431...... 1441......
P862017      1027 cagccagtcc cttccgatca cctccagaca ccacgctctc ctttcggagc
P862018       820 cgccttccgc ccgcgggctc cgacgatctg tccctcccgg gcagagagct
```

```
alignment  position  1451......  1461......  1471......  1481......  1491......
P862017        1077  ccgccc----  ----------  ----------  ----------  ----------
P862018         870  cacctagtct  acatctggag  cgtctgaggg  gaaagcgaaa  gctctaccat

alignment  position  1501......  1511......  1521......  1531......  1541......
P862017        1083  ----------  ----------  ----------  ----------  -------GCC
P862018         920  ccgatcttta  taagctccgc  tagcctagct  ttcagagagc  gctgggaGCC
                                                                              ***

alignment  position  1551......  1561......  1571......  1581......  1591......
P862017        1086  GGAAGTGgcg  gcccggaact  actcccacag  gggggcgggg  a--AGGAAGA
P862018         970  GGAAGTGaca  agaggaagtc  ccgcctcact  cagagggcgg  gctAGCAAGA
                     *******                                            *******
                                                                        *******
                                                                        *******
                                                                        *******
                                                                        *******
                                                                        *******
                                                                        *******
                                                                        *******
                                                                        *******
                                                                        *******

alignment  position  1601......  1611......  1621......  1631......  1641......
P862017        1134  TGGCGGCGCC  CAGCGTCCCG  TGAGGAGAGA  GGacaca-GG  GATCCCGGGG
P862018        1020  TGGCGGCGCC  CAGGAGCCGG  TGAGGTGAGA  GGcgtcctGG  GGTCCTGGGG
                     **********  **********  **********  **      **  **********
                     **********  **********  **********  **
                     **********  **********  **********  **
                     **********  **********  **********  **
                     **********  **********  **********  **
                     **********  **********  **********  **
                     **********  **********  **********  **
                     **********  **********  **********  **
                     **********  **********  **********  **
                     **********  **********  **********  **

alignment  position  1651......  1661......  1671......  1681......  1691......
P862017        1183  Agcggcccca  gactcGTAAA  TTATGGCCGC  ATCTCCGCAC  ACtctcTCCT
P862018        1070  Actgctgcac  ca---GTACG  TTATGGCCGC  GTCCCCGCAC  ACcattTCCT
                     *                *****  **********  **********  **    ****
                                      *****  **********  **********  **    ****
                                      *****  **********  **********  **    ****
                                      *****  **********  **********  **    ****
                                      *****  **********  **********  **    ****
                                                                           ****
                                                                           ****
                                                                           ****
                                                                           ****
                                                                           ****

alignment  position  1701......  1711......  1721......  1731......  1741......
P862017        1233  CACGCCTCCT  GACAGGTACT  GTACCCGccT  TGGGCACGAC  CTTGGGGGAA
P862018        1117  CGCGCCTCCT  CACAGGTATT  GTACCCGg-T  TGGGAATGAT  CGTGGACGAA
                     **********  **********  *******  *  **********  **********
                     **********  **********  *******
                     **********  **********  *******
                     **********  **********  *******
                     **********  **********  *******
                     **********  **********  *******
                     **********  **********  *******
                     **********  **********  *******
                     **********  **********  *******
                     **********  **********  *******

alignment  position  1751......  1761......  1771......  1781......  1791......
P862017        1283  ATTCTTCTCT  TATTaCCACG  TGCGAGGCCG  TGGTTCCGGC  TGGCTGCAAC
P862018        1166  TTGTTTTCTT  TTTTgCCATG  TGCGGTACCG  TGTTGCCGGC  CGCCTGCAAC
```

```
                       * * * * * * * * * *  * * * *    * * * * *  * * * * * * * * * *  * * * * * * * * * *  * * * * * * * * * *
                                                       * * * * *  * * * * * * * * * *  * * * * * * * * * *  * * * * * * * * * *
                                                       * * * * *  * * * * * * * * * *  * * * * * * * * * *  * * * * * * * * * *
                                                       * * * * *  * * * * * * * * * *  * * * * * * * * * *  * * * * * * * * * *
                                                       * * * * *  * * * * * * * * * *  * * * * * * * * * *  * * * * * * * * * *
                                                       * * * * *  * * * * * * * * * *  * * * * * * * * * *  * * * * * * * * * *
                                                       * * * * *  * * * * * * * * * *  * * * * * * * * * *  * * * * * * * * * *
                                                       * * * * *  * * * * * * * * * *  * * * * * * * * * *  * * * * * * * * * *

alignment position     1801......  1811......  1821......  1831......  1841......
P862017        1333    CTTGGggAGC  CCTTGGCgag  gttggccggc  caccggtgtg  gtggGGGAAA
P862018        1216    CTTGGa-AGC  CCTTGGCttg  tcacttggca  ----------  ----GGGAGG
                       *****  ***  *******                                 ******
                       *****                                               ******
                       *****                                               ******
                       *****
                       *****
                       *****
                       *****
                       *****

alignment position     1851......  1861......  1871......  1881......  1891......
P862017        1383    TGACCTCTGG  AGTAGCTTGG  AGTGAAGAGG  TCACggagct  agGGTCCAGT
P862018        1251    TGACCTTTAG  GGTCACCTGA  AGGGACGAGG  CCAAgtattg  t-GGACCAGC
                       **********  **********  **********  ****          ********
                       **********  **********  **********  ****          ********
                       **********  **********  **********  ****

alignment position     1901......  1911......  1921......  1931......  1941......
P862017        1433    CTTGCACTGT  GGCTTTAGTG  GTCCacacag  cct-------  ----------
P862018        1300    TTTGCACCGT  AGGCCAGGTG  GTCCctaagg  cttgatatcc  cgtgctcttt
                       **********  **********  ****
                       **********  **********  ****

alignment position     1951......  1961......  1971......  1981......  1991......
P862017        1466    ----------  ----------  ----------  ----------  ----------
P862018        1350    ggagtttcag  acagtgcaag  atggagggac  aggataaacc  atggggctcc

alignment position     2001......  2011......  2021......  2031......  2041......
P862017        1466    ----------  ----------  ----------  ----------  ----------
P862018        1400    acaagcctgg  cacatgagag  ccgcggttcc  tggccaccgc  cttttaatga

alignment position     2051......  2061......  2071......  2081......  2091......
P862017        1466    ----------  -----CCTCC  ATCCGGCTTC  TGCGGAAGAG  CGGTGTCTTC
P862018        1450    caagaagcgc  acctcCCCCC  CCCCCCCCCC  CCCGTTAAGG  AGCTGTCTTC
                                        *****  **********  **********  **********

alignment position     2101......  2111......  2121......  2131......  2141......
P862017        1501    C---------  ----------  ----------  ----------  ----------
P862018        1500    Cagcttaaag  tgttacctta  atcaccatga  taatgataac  tgccaactga
                       *

alignment position     2151
P862017        1502    ----
P862018        1550    atcc
```

Pairwise similarities:

For each pairwise alignment, the similarity (relative to the maximum similarity) and the number of identical nucleic acids (in % of shorter sequence) is given. Maximum values are underlined.

	**P862018 (1553 bp)**

**P862017 (1501 bp)**	1.000 22 %

Please note that the **similarity value 1.000** marks only the two **most similar** sequences, it does not necessarily mean that these sequences are identical.

**Extract alignment region**	
**Positions in alignment**	from: to:
**Output file**	dialign.seq

Extract aligned sequences

For comments, questions or bug reports, please contact support@genomatix.de

© Genomatix Software GmbH 1998-2005 - All rights reserved. License Agreement GEMS Launcher 4.1.1

Genomatix-Logo Logout Personal Messages GEMS-Logo

Genomatix_MainGEMSElDoradoGene2PromoterBiblioSphere
Frequently_asked_questionsYour_ResultsYour_SequencesYour_ProtocolHelp

GEMS Launcher Task: *DiAlign TF*: Multiple alignment plus TF sites
working on promoters.seq (3 seq.)

[Alignment] [Pairwise_similarities]

DiAlign professional TF Release 3.1 December 2004 **Thu May 12 18:51:16 2005**

Solution parameters:

**Sequence file:**	promoters.seq (3 seq.)
**Type of sequences:**	nucleotide sequences
**Threshold T:**	0.00
**'*' signs below alignment denote:**	diagonal similarity (max. similarity is represented by 10 '*' signs)
**Alignment output:**	complete alignment is shown<br># nucleic acids per line: 50
**TF output:**	common TF matches located in aligned regions (common to 3 (100.0 %) sequences)
**Family matches:**	yes
**MatInspector library:**	Matrix Family Library Version 5.0 (February 2005)
**Selected groups** (core/matrix sim):	• ALL user_defined.lib (0.75/Optimized)<br>• ALL vertebrates.lib (0.75/Optimized)

Aligned Sequences:

No.	Sequence Name	Sequence Description	Length
1	P911216	sym=COX11|loc=Loc1353|taxid=9606|spec=Homo sapiens|chr=17| ctg=NT_010783|str=(-)|start=11698832|end=11699832|len=1001| tss=501| comm=COX11 homolog, cytochrome c oxidase assembly protein (yeast); (NM_004375/502/bronze;)	1001 bp
2	P911219	sym=Cox11|loc=Loc69802|taxid=10090|spec=Mus musculus|chr=11| ctg=NT_039521|str=(+)|start=2038150|end=2039150|len=1001| tss=501| comm=COX11 homolog, cytochrome c oxidase assembly protein (yeast); (AK008096/529/gold;NM_199008/529/bronze;)	1001 bp
3	P911220	sym=na|loc=Loc287621|taxid=10116|spec=Rattus norvegicus| chr=10|ctg=NW_047336|str=(+)|start=20039176|end=20040176| len=1001|tss=501| comm=similar to COX11 homolog; cytochrome c oxidase subunit 11; cytochrome c oxidase assembly protein COX11; (XM_213433/540/silver;)	1001 bp

Alignment (DiAlign format):

Please note that only **upper-case** letters are considered to be **aligned**. For more information, please have a look at the user guide.

V$SMAD V$AHRR V$MYOD V$EGRF V$DMTF V$BNCF V$HIFF V$STAF V$P53F V$GATA V$ZFIA V$PAX6 V$SRFF

```
alignment
position         1.........  11.........  21.........  31.........  41.........
P911216      1   gcaagttgta  agggcatcag  aGCAGGGGA  AGCCGACTGC  CCAc-GGGCT
P911219      1   ----------  ----------  --GCAGTGGG  AGACATCTGC  CCATGGGGCT
```

```
P911220      1 ---------- ---------- ---------- ------CTGC CCTTGGGGCT
                                      ******** ********** **********
                                                     ****  **********
                                                                 *****

alignment
position         51.......  61.......  71.......  81.......  91.......

P911216     50 GAGAAAATGC AGGTGTCGTG -GGtgcccat GAGGGCTGCG GTTAACACAG
P911219     29 GAGAAGATGC ACAGGTCGGG -GTGTTGGA- GAGAAAGGCG GTTCTCATAC
P911220     15 GAGAAGACGC ACAAGTggag aGTGTTGGA- GAGAAATGCG GTTCTCACAG
               ********** **********  ********  ********** **********
               ********** ******      ********  ********** *******
               ********** ******      **               *** *******

alignment
position        101.......  111.......  121.......  131.......  141.......

P911216     99 C-AAGGCTTC CTGAGAGAGG tga------- TACTTGAACT AAGTCCAGGG
P911219     77 T-AAGTGTTC CAGAAAGAGG GACGTTggtt TGCCTGCGCT CAGTCAGCGG
P911220     64 taAAGTGTTC CTGAAAGAGG CAAGTTtgcc c----GAGCT CGGTCAGAGG
               * ******** ********** ******     ********** **********
                 ******** **********                         ********

alignment
position        151.......  161.......  171.......  181.......  191.......

P911216    141 CAGTGAGCAC AGGAGCcagc ggccaaggga CCCCAGGAGA AAAGACCGCG
P911219    126 CGGAAAGCAA CGGGGCTGAG CGA------A CCCCAAGTGA GAGCCCCGCG
P911220    110 CAGTAAGCGA GGGGGTTTAG TGA------A CCCCAAGTGG AAGCCCCGCG
               ********** ********** ***      * ********** **********
               ********** ********** ***      * ********    *********
                                             * ********    *********
                                                            *********
                                                            *********

alignment
position        201.......  211.......  221.......  231.......  241.......

P911216    191 GGAGAGCgtc gcc--GGGGC TGCCGGGTCG AGACAAAGGC TGGTGGAGTT
P911219    170 GCAGAGCTGC CGCTGGGGGC CGCTAGGTCG AGACAAAGGC TGGCGAAGTT
P911220    154 GCAGAGCTGC CGCTGGGGGC CGCTAGGTCA AGACAAAGAC TGGCGGAGTT
               ********** ********** ********** ********** **********
               ********** ********** ********** ********** **********
               ********** ********** ********** ********** **********
               ********** ********** ********** ********** **********
               ********** ********** *********  ********** **********
                               ***** *********  ********** **********
                               ***** *********
                               ***** *********

alignment
position        251.......  261.......  271.......  281.......  291.......

P911216    239 Taaataaaga tcacaggcgC AACCCCAAGT GAtggcaAGC TTGCACGCGG
P911219    220 TGGGGAGATG GGGATA---C AAGCCCCAGT GAGG---AGC TTGCACGCCG
P911220    204 TGGGAAGATG AGAGTA---C ActctccgGT GAGG---AGT TTGCACGCCG
               ********** ******   * ********** ****   *** **********
               ********** ******   * *       ** ****   *** **********
               *                           ** ****   *** **********
               *                           ** ****   *** **********
               *                                     *** **********
               *                                     *** **********
                                                     *** **********

alignment
position        301.......  311.......  321.......  331.......  341.......

P911216    289 ACGGGCGAAA CGCATTTTCC TAAATGTtaa gagacgttct gg---CGAGA
P911219    264 AAGGCCCAAA TGCATTTTCt gCAAAGTAAA CTAAAGAAGC ATCCCAAGGA
P911220    248 AAGGCCCAAA TGCATTTTCC TCAAAGTAAA TTAAAGAAGC ATCATCAGGA
               ********** ********** ********** ********** **********
               ********** ********** ********** ********** **********
               ********** *********   ******                  *****
               ********** *********   ******
```

```
                  ********** *********
                  ********** *********
                  ********** *********

alignment
position          351....... 361....... 371....... 381....... 391.......

P911216       336 AGC-GAAGTG GGAGTAACAG TCACAAAGC- --AAAACGCA AACTTACCCg
P911219       314 AACaAAAGTG CGAGGCACAG TCACTTAGCA AGAAAACGCG AACTTACCCT
P911220       298 AAC-AAAGTG CGAGGCACAG TCGCCTAGCA AAAAAACGCG AACTTACCCT
                  *** ****** ********** ********** ********** **********
                  *** ****** ********** ********** ********** **********
                  *** ****** ********** ********** ********** **********
                                                   ******** **********
                                                        *** **********
                                                        *** *********

alignment
position          401....... 411....... 421....... 431....... 441.......

P911216       382 taaTCTGGCA ACCCGGCCTC CGTAGCCCGA CTGCaagcgc tgccACCTGA
P911219       364 CT-TCGGGCC ACCCGGCCTC CGTAGCGCCA CTGCT----- ----AACTGT
P911220       347 CT-TCGGGCC ACCCGGCCTC CGTAGCGCGA CTGCT----- ----ACCTGT
                  ** ******* ********** ********** *****          ******
                  ** ******* ********** ********** *****          ******
                  ** ******* ********** ********** *****          ******
                  ** ******* ********** ********** *****          ******
                  ** ******* ********** ******** * ****           ******
                     ******* ********** ******** * ****             ****
                     ******* ********** ******** * ****
                     ******* ********** ******** * ****

alignment
position          451....... 461....... 471....... 481....... 491.......

P911216       432 GGAGCCTGGT AGCCAAGGCA GCGGC--AGG CTCAACCCAG ATCTCGCGAG
P911219       404 GGAATCTGGT AGTCAAGGCA GCAGCGGAGG CTCAAGCCGG GTACCGCGAG
P911220       387 GGAATCTGGT AGTCAAGGCA GCAGCGGA-G CTCAAGCCGG AGACCGCGAG
                  ********** ********** ********** ********** **********
                  ********** ********** ********              ********
                  ********** ********** ********              ********
                  ********** ********** ********
                  ********** ********** *****  *
                  ********** ********** **

alignment
position          501....... 511....... 521....... 531....... 541.......

P911216       480 ACggagcacg cCTCGCGAGA TTTGACCTCT CGTCCCTGAG AGGCGGGtgg
P911219       454 ACTCGCGAGG TCTCGCGAGA TCTCGCC-AG CGGCCCCGCC AGGCGGGCAT
P911220       436 ACTCCGGAAG TGTCGCGAGA TCTCGCCTAG CGGCCCTGAC AGGCGGGCGT
                  ********** ********** ********** ********** **********
                  ********** ********** ********** ********** **********
                  ********** ********** ******* ** ********** *******
                               ******** *******

alignment
position          551....... 561....... 571....... 581....... 591.......

P911216       530 gtgttagttc agaGGGTTAT GGGAGGGCTC TGGCGTCCTG GATGGAGGTg
P911219       503 TCTTTCCGGG ---GGGTTAT GGGAGGGCTC TGGTGTCCGG GATGGAGGTT
P911220       486 TCTTTCCGGG ---GGGTTAT GGGAGGGCTC TGGTGTCCGG GATGGAGGTT
                  **********    ******* ********** ********** **********
                  **********    ******* ********** ********** **********
                         ***    ******* ********** ********** **********
                         ***    ******* ********** ********** **********
                         ***    ******* ********** ********** **********
                                ******* ********** ********** *********
                                ******* ********** ********** *********
                                ******* ********** ********** *********
                                ******* ********** ********** *********
                                ******* ********** ********** *********

alignment
position          601....... 611....... 621....... 631....... 641.......
```

```
P911216   580 cGTTCCTTTC TGTGGCTGGC GCTGGATCCA CCCTGGGTct ccaaccaggg
P911219   550 gGTGGCTTCG TGTGGCCGGG GCTGGAGGCA GCCCGGGTGG TCAGGgCGGA
P911220   533 catcaCTTTG TGTGGCCGGG GCTGGAGCCA GCCCCGGTGG TCGGGcCGGA
               ********* ********** ********** ********** ***** ****
                   ***** ********** ********** ********** ***** ****
                   ***** ********** ********** ********** ***** ****
                   ***** ********** ********** ********
                   ***** ********** ********** ********

alignment
position          651.......661.......671.......681.......691.......

P911216   630 ctgcagagag ggtAGAGCCG TTTCTTAGGC CAGAGTGGAG TGGGACAGGA
P911219   600 CTGTGGTGAA CGCAGAGCTG GTGCTTAGGC CCGGGTGGGA TGGGCTCGGA
P911220   583 CGGTGGTGAA CGCAGAGCGG GCTCTTAGGC CGGGATGGAA TGGGGTCGGA
              ********** ********** ********** ********** **********
              ********** ********** ********** ********** **********
              ********** ********** ********** **** ***** **********
                            ******* ********** **** ***** **********
                                                    ***** **********

alignment
position          701.......711.......721.......731.......741.......

P911216   680 GGtGCCGAGA GAGGACTGAG GTGGCTTGGG ACATGGAAGC GCtgcagcct
P911219   650 GGCGCGGAGC GGGGTCTGAG ACGCCTCGGG ACATGGAAGC GCCCGTGCGG
P911220   633 GGCGCGGAGC AGGGACTGAG ACGCCTCGGG ACATGGAAGC GCCCGAGCGG
              ********** ********** ********** ********** **********
              ********** ********** ********** ********** **********
              ********** ********** ********** ********** **********
              ** ******* ********** ********** ********** **
              ** ******* ********** ********** ********** **
                 ******* ********** ********** ********** **

alignment
position          751.......761.......771.......781.......791.......

P911216   730 tcgagcccgg catccagcat tGCAGCCGCC GCGGCGGCCT AAGAGCTCGA
P911219   700 GGTCCGGGGG CCCGcgaC-- -GCAGCCGCC ACGGCGGCCG AGGAGCAGCA
P911220   683 GGCCCGGGGT CCCGtcgC-- -GCAGCCGCC ACGGCGGCCG AAGAGCAGCA
              ********** ****   *    ********* ********** **********
              ********** ****   *    ********* ********** **********
              ********** ****   *    ********* ********** **********
                                *    ********* ********** **********
                                     ********* ********** **********
                                     ********* ********** **********
                                     ********* ********** **********
                                     ********* ********** **********

alignment
position          801.......811.......821.......831.......841.......

P911216   780 ACCCTTTCac aCGCGCGCAG GAGGAGGAGC GGCGGCGGCA GAACAAGACG
P911219   747 ACCCTTTCCA gCGCGCGCAG GAGGACGAGT GGCGGCGGCG GAACAAGACG
P911220   730 ACCCTTTCCA gCGGGCGCAG GAAGACGAGT GGCGGCGGCG GAACAAGACG
              *********   ********* ********** ********** **********
              *********   ********* ********** ********** **********
              *********   ********* ********** ********** **********
              *********   ********* ********** ********** **********
              *******     ********* ********** ********** **********
              *******     ********* ********** ********** **********
              *******        ****** ********** ********** **********
              *******        ****** ********** ********** **********
                             ****** ********** ********** **********

alignment
position          851.......861.......871.......881.......891.......

P911216   830 accCTCACTT ACGTGGCCGC TGTCGCCGTG GGCATGCTGG GGGcgTCCTA
P911219   797 GTGCTCACCT ACGTGGCCGC GGCCGCCGTG GGCATGCTGG GGGCCTCCTA
P911220   780 GTGCTCACGT ACGTGGCCGC GGCCGCCGTG GGCATGCTGG GGGCCTCgTA
              ********** ********** ********** ********** **********
              ********** ********** ********** ********** **********
              ********** ********** ********** ********** **********
```

```
               ********** ********** ********** ********** ******* **
                  ******* ********** ********** ********** ******* **
                  ******* ********** ********** ********** ***  ** **
                  *     * ********** ********** ********** ***  ** **
                  *     * ********** ********** ********** ***     **
                  *     * ********** ********** ********** ***     **
                  *     * ********** ********** ********** ***

alignment
position       901....... 911....... 921....... 931....... 941.......

P911216   880  CGCTGCCGTA CCCCTTTATC GGCTCTATTG CCAGGTAGgg gccggcgccg
P911219   847  CGCCGCGGTG CCCCTCTATC GGCTCTACTG CCAGGTA--- ----------
P911220   830  CGCCGCGGTG CCCCTCTATC GGCTCTACTG CCAGGTAGgg a---------
               ********** ********** ********** ********
               ********** ********** ********** ********
               ********** ********** ********** ********
               ********** ********** ********** *******
               ********** ********** ********** *******
               ********** ********** ********** *******
               ********** ********** ********** *******
               ********** ********** ********** *****
               ********** ********** ********** *****

alignment
position       951....... 961....... 971....... 981....... 991.......

P911216   930  cccgcAGGGT GGCGCAGACG CGAGaggtgg gggtgcaaga caaatcccgg
P911219   884  -----AGGGA GGGACGAAGC CGCGGCACGG ATCCCACCTG ----------
P911220   871  ---------- -GGGACGAGG CGCGGCACGG ATCCCACCTG ----------
                    ***** ********** ********** **********
                           ********* ********** **********
                           ********* ********** **********
                           ********* ********** **********

alignment
position       1001...... 1011...... 1021...... 1031...... 1041......

P911216   980  gacaAGCCCT CGCTCTGCGG Gt-------- ---------- ----------
P911219   919  ----AGCCAT CCCCCTGCCG GGGAGGCCGA GGTCACCCGC Cggagggggg
P911220   900  ----AGCCAT CACTCTGTCC CAGAGGCCGA GGTTACCCAC Ccgtg-----
                   ****** ********** ********** ********** *
                   ****** *
                   ****** *
                   ****** *

alignment
position       1051...... 1061...... 1071...... 1081...... 1091......

P911216   1002 ---------- ---------- ---------- ---------- ----------
P911219   965  aggggggcGT CGCCTCGGAT CCAGACCGGG AAGGACG--- ----------
P911220   941  --------GT CGCCTCGGTT CCAGACCGGG AAGGACGtac ccaaggtcac
                       ** ********** ********** *******
                       ** ********** ********** *******
                       ** ********** ********** *******

alignment
position       1101...... 1111.....

P911216   1002 ---------- ---------
P911219   1002 ---------- ---------
P911220   983  taaagaaaac tgcatgggg
```

Pairwise similarities:

For each pairwise alignment, the similarity (relative to the maximum similarity) and the number of identical nucleic acids (in % of shorter sequence) is given. Maximum values are underlined.

	**P911219 (1001 bp)**	**P911220 (1001 bp)**

**P911216 (1001 bp)**	0.370 58 %	0.374 56 %
**P911219 (1001 bp)**		1.000 83 %

Please note that the **similarity value 1.000** marks only the two **most similar** sequences, it does not necessarily mean that these sequences are identical.

**Extract alignment region**	
**Positions in alignment**	from: to:
**Output file**	dialign.seq

Extract aligned sequences

For comments, questions or bug reports, please contact support@genomatix.de

© Genomatix Software GmbH 1998-2005 - All rights reserved.

License Agreement

GEMS Launcher 4.1.1

Genomatix-Logo Logout Personal Messages GEMS-Logo

Genomatix_MainGEMSElDoradoGene2PromoterBiblioSphere
Frequently_asked_questionsYour_ResultsYour_SequencesYour_ProtocolHelp

GEMS Launcher Task: *DiAlign TF*: Multiple alignment plus TF sites
working on promoters.seq (3 seq.)

[Alignment] [Pairwise_similarities]

DiAlign professional TF Release 3.1 December 2004 **Thu May 12 21:13:29 2005**

Solution parameters:

**Sequence file:**	promoters.seq (3 seq.)
**Type of sequences:**	nucleotide sequences
**Threshold T:**	0.00
**'*' signs below alignment denote:**	diagonal similarity (max. similarity is represented by 10 '*' signs)
**Alignment output:**	complete alignment is shown<br># nucleic acids per line: 50
**TF output:**	common TF matches located in aligned regions (common to 3 (100.0 %) sequences)
**Family matches:**	yes
**MatInspector library:**	Matrix Family Library Version 5.0 (February 2005)
**Selected groups** (core/matrix sim):	• ALL user_defined.lib (0.75/Optimized)<br>• ALL vertebrates.lib (0.75/Optimized)

Aligned Sequences:

No.	Sequence Name	Sequence Description	Length
1	P939218	sym=COX15|loc=Loc1355|taxid=9606|spec=Homo sapiens|chr=10|ctg=NT_030059|str=(-)|start=20239926|end=20240926|len=1001| tss=501| comm=COX15 homolog, cytochrome c oxidase assembly protein (yeast); (NM_078470/501/bronze;NM_004376/501/bronze;)	1001 bp
2	P939219	sym=Cox15|loc=Loc226139|taxid=10090|spec=Mus musculus|chr=19|ctg=NT_039692|str=(-)|start=4133505|end=4134589| len=1085|tss=501,585| comm=COX15 homolog, cytochrome c oxidase assembly protein (yeast); (AK051792/603/gold;NM_144874/519/silver;)	1085 bp
3	P939220	sym=na|loc=Loc309391|taxid=10116|spec=Rattus norvegicus|chr=1|ctg=NW_047566|str=(+)|start=38313|end=39313|len=1001| tss=501| comm=similar to COX15 gene product; (XM_219882/587/silver;)	1001 bp

Alignment (DiAlign format):

Please note that only **upper-case** letters are considered to be **aligned**. For more information, please have a look at the user guide.

V$ZBPF V$OAZF V$ETSF V$E2FF U$NRF2 V$AREB V$IRFF V$OCTP V$EBOX

```
alignment position  1.........  11........  21........  31........  41........
P939218        1 aggggacccc gggactcgaa ccttggctcc acagctgagc cattctcgct
P939219        1 ctaggttgta gag------- ---------- ---------- ----------
P939220        1 ggcgagtg-- ---------- ---------- ---------- ----------
```

```
alignment position 51........ 61........ 71........ 81........ 91........
P939218        51 acctgcccct cgtcacgccc tCCGTTTCCA CACCTTTAAC GCCTCAAAGA
P939219        14 ---------- ---------- -CTGTCACCA CACTTTTATT ATCTACCACA
P939220         9 ---------- ---------- ---------- ---------- ----------
                                         ********* ********** **********

alignment position 101....... 111....... 121....... 131....... 141.......
P939218       101 TAGAagg--- ---------- ---------T GCCGCCCAGG GGCTGGAAGG
P939219        43 GAGAcaggga ggtGGTATGG TGCCCTCTTG TGGCCCTAGG GGCGGCGGGA
P939220         9 ---------- ---GGTATGG TGCCCTCTTG TGGCCCAAGG GGCTGCGGGA
                  ****          ******* ********** ********** **********
                                ******* ********** ********** **********
                                ******* ********** ********** **********
                                ******* ********** ********** **********
                                                ** ********** **********
                                                     ******** **********

alignment position 151....... 161....... 171....... 181....... 191.......
P939218       130 AGCTGAGGAA ACgactccag aagaaatcac cactgactac gactcccg--
P939219        93 CGCTGAGGGC ACTTGCGAGA CTGGCGGGGA TCCAGGAGcA GCTGAGGGAC
P939220        46 CGCTGAGGGA ACTTGTGTGA CTGGCCGACA CCCACGAGgA GCTGACGGAA
                  ********** ********** ********** *******  * **********
                  ********** ********** ********** *******  * **********
                  ********** **                             * **********
                  ***                                       * **********
                  ***
                  ***

alignment position 201....... 211....... 221....... 231....... 241.......
P939218       178 ---------- ---------- ---------- ---------- ----------
P939219       143 TGCGTGACTG CCCGGGACTC CGGAGCActg aGAAACTCGA CCGCGGGAAA
P939220        96 TGCGTGACTG CCCGGGACCC CGGAGCAgag -GACACTCGA CCGCGGGAAA
                  ********** ********** *******     ********* **********
                  ********** ********** *******     ********* **********
                  ********** ********** *******     ********* **********
                  ********** ********** *******

alignment position 251....... 261....... 271....... 281....... 291.......
P939218       178 ---------- -CCGGCCCGC CCCGGGGAGC CTTCGGCCGA CCGt-CCCCT
P939219       193 GCCTGGCAAG CCGGGACCCA CCCCGGGGTC CACCGGCCGA CCGCCCCCCT
P939220       145 GCATGACAGG CCGGGAgCCA CCCCGGGGTC CACCGGCCGA CCACCCCCt-
                  ********** ********** ********** ********** **********
                  ********** ******  *** ********** ********** **********
                  ********** ******  *** ********** ********** **********
                                    *** ********** ********** ***    ***
                                                                     ***
                                                                     ***

alignment position 301....... 311....... 321....... 331....... 341.......
P939218       216 CCCCCGCCAC CTTCCGCACC GGCCTTCCCG GACGGTATCC GCGCTCGTTT
P939219       243 GCCCCGCCAC CTTCCGCACC CGCCTTCCCC GACGATATCC ACGCCTGTTT
P939220       194 -----GCCCC CTTCCGCACC CGCCTTCCCC GACGATATCC ACGCCTGTTT
                  ********** ********** ********** ********** **********
                  ********** ********** ********** ********** **********
                  ********** ********** ********** ********** **********
                       ***** ********** ********** ********** **********
                             ********** ********** ********** **********
                             ********** ********** ********** **********
                                           ******* ********** **********
                                           ******* ********** **********
                                           ******  ********** **********
                                           ******

alignment position 351....... 361....... 371....... 381....... 391.......
P939218       266 TCGCTCAGAG GAGGCCCCCT gccttTTCAT GCTCCACGCG TTCCTCCCTC
P939219       293 CCGCTCGCAA GAAGCCCCTT TC---TTCAT GCTTCCCGCG TTCCTCCCTC
P939220       239 CCGCTCGCAA GAAatCCCTT TC---TTCAT GCTTCCCGCG TTCCTTCCTC
```

```
                            ********** ********** **   ***** ********** **********
                            ********** ********** **   ***** ********** **********
                            ********** ***  ***** **   ***** ********** **********
                            ********** ***  ***** **   ***** ********** **********
                            ********** ***  ***** **   ***** ********** **********
                            ********** ***  *****      ***** ********** **********
                                                       ***** ********** **********
                                                       ***** ********** **********
                                                       ***** ********** **********

alignment position  401....... 411....... 421....... 431....... 441.......
P939218        316  GTGCGCCTGC AGTTTccact tggaatttgg gctccggcgc gcaccagcta
P939219        340  GCTCGCCTTC GGTTTCCACT GAGTCCCCGC G--------- ----------
P939220        286  GCTCGCCTTC GGTTTCCACT GAGTCCCCGC G--------- ----------
                    ********** ********** ********** *
                    ********** ********** ********** *
                    ********** ********** ********** *
                    ********** ********** ********** *
                    ********** *****
                    ********** *****
                    ********** *****
                    ********** *****
                    ********

alignment position  451....... 461....... 471....... 481....... 491.......
P939218        366  agaagcgcgt caacaGCTGC GCGCGCCcgt gc----GCGC GTCCCCGACA
P939219        371  ---------- -----GCCGC GCGAGCgACG GAGGAGGCGC GTCCCCGACG
P939220        317  ---------- -----GCTGC GCGAGCCACC GAGAAGGCGC GGCCCCGATG
                               ***** ********** ********** **********
                               ***** ****** *** ********** **********
                               ***** ****** *** ********** **********
                               ***** ******

alignment position  501....... 511....... 521....... 531....... 541.......
P939218        412  CCTACGCCCC aGCAGCC-CC ---------- --CGCGAAAG CGGAGTCGCA
P939219        406  CCCGTGCCCG -GCAGCCtCC CAGGGAGTCG TGCGTGCACG CGCAGTGGCC
P939220        352  CCGGTGCCCC -GCAGCC-CC CGCGGAGTCA TATGCGCAAG CGCAGTGGCC
                    **********  ****** ** ********** ********** **********
                    **********  ****** ** ********** ********** **********
                    **********  ******

alignment position  551....... 561....... 571....... 581....... 591.......
P939218        449  ACGCAGGCGC acttctgttc gctccggtcc ccagagaagg cggggctccc
P939219        455  AACCTAGCGC gaggggggcgc gcacgac--- ---------- ----------
P939220        400  AGCCTcacta gcag------ ---------- ---------- ----------
                    **********
                    *****

alignment position  601....... 611....... 621....... 631....... 641.......
P939218        499  gctGCCCGAC CCGGAAGTGC TTCTCTTTTC CTTGGCGGAG GAGggaGACC
P939219        482  ---GCCTGAC CCGGAAGTGC TTGTTCTTTC GGGAGCGGAG AAGCG-GACC
P939220        414  ---GCCTGAC CCGGAAGTGT TTGTCCTTTT GGta---GAG AAGCG-GACC
                       ******* ********** ********** ********** ***** ****
                       ******* ********** ********** ********** ***** ****
                       ******* ********** ********** **     *** ***
                       ******* ********** ********** **     *** ***
                       ******* ********** *********

alignment position  651....... 661....... 671....... 681....... 691.......
P939218        549  ACAGAGCCCT GGGTTGTGGA AGAGGTGGCt gttccctgtc aTCAGTATGC
P939219        528  GCAGCTCGCT GGGGCGCTCC GGCAGTGGC- GCTGTACTG- -TCAGCATGC
P939220        457  GCAGATCGCT GGCTGGTGcg ttccgcagag GCTGTACTG- -TCAGTATGC
                    ********** ********** *********  *********   *********
                    ********** **                    *********   *********
                                                     *********   *********
                                                                 *********
                                                                 *********

alignment position  701....... 711....... 721....... 731....... 741.......
P939218        599  AGCGATTGCT CTTTCCGCCG TTGAGGGCCT TGAAGGGGAG gcagtatctg
```

```
P939219      575 AACGGTTGCT CCTTCCGTCC CTGAGGGCCT TGACGGGGAG CCGCAATGTC
P939220      505 AACGGTTGTT CCTCCCGCCC CTCAAGGCCT TGATGGGGAG CCGCAATGTC
                 ********** ********** ********** ********** **********
                 ********** ********** ********** ********** **********
                 ********** ********** ********** ********** **********
                 ********** ********** ********** ********** **********
                 ********** ********** **   ***** ********** **********
                   ******** ********** **   ***** **********
                   ******** ********** **   ***** **********
                                            ***** *

alignment position 751....... 761....... 771....... 781....... 791.......
P939218      649 ccGCTCCTGG CTCCTAGGGC AGCGCCTAGA GCACAGGTAC CgCACTGCAG
P939219      625 GGGCTCCTGG TCCCTAGGGT GGCGTCCAGA ACACAGGTAC C-CACTCCTG
P939220      555 GGGCTCCTGG TCCCTAAGGC GGCATCTAGA GCACAGGTAC C-CACTACAG
                 ********** ********** ********** ********** * ********
                 ********** ********** ********** ********** * ********
                 ********** ********** ********** ********** * ********
                 ********** ********** ********** ********** *
                 ********** ********** ********** ********** *
                   ******** *****  *** ********** ********** *
                   ******** *****  *** ********** ********** *
                   ******** *****
                   ******** *****

alignment position 801....... 811....... 821....... 831....... 841.......
P939218      699 CGGACTcCGA GTCGCGGGAG CGGGGATGGa gtg-AAAGG- ---GCCGGGA
P939219      674 CTGGCT-CGG ATCACGGAAG CCGGGCTGGG ATAAAAAAG- ---GCCAGGA
P939220      604 CTGGCTtgg- GTCACGGAAG CCGGGCTGGG ATAAAAAAGa aagGCCAGGA
                 ****** *** ********** ********** ********      *******
                 ******      ********* ********** ********      *******
                 ******      ********* ********** ********
                             ********* *********     *****

alignment position 851....... 861....... 871....... 881....... 891.......
P939218      744 TAAAGATAAT GTAGAGC-CC CACA------ --------TC ACGTTTCcgg
P939219      719 TAAAGTAAAC GGACCGC-CC TGCAAGTCCG GCTCGCCTTC TCATTTCAGG
P939220      653 GAAAGTAAAG GGACCGtgCC TGCAAGTCCT GATCTCCTTC ACGTTTCACG
                 ********** ******* ** ********** ********** **********
                 ********** ******  ** ********** ********** **********
                                    ** ****

alignment position 901....... 911....... 921....... 931....... 941.......
P939218      779 agctccttac agtgcactca caacgcatag gttcagatca gagcCCCTAG
P939219      768 ACATATTTCC TTGTGTCCCG ACACCT---- ---------- ----CCCTGA
P939220      703 ACACATTTCC TTGTGTCCAG ACACCT---- ---------- ----CCCAGA
                 ********** ********** ******                ******
                 ********** ********** ******                ******
                         ** ********** ******                ******

alignment position 951....... 961....... 971....... 981....... 991.......
P939218      829 GTTCAGATCT GGAACATTGA C---CTGCTG TGTGGTATTG GCCAAGTTAA
P939219      800 ATGCGGATCC GGAAATATTA CCCACTGCTG TGTATGACTG ACCAATTTAC
P939220      735 ATGTGAATCC GGAAATattg CCCACTGTTG TGTATTACTG GCCAATTTAT
                 ********** ********** ********** ********** **********
                 ********** ******     ********** ********** **********
                 ********** ******     ********** ********** **********
                                           ****** ********** **********
                                           ****** ********** **********

alignment position 1001...... 1011...... 1021...... 1031...... 1041......
P939218      876 TTAAACTTTc tgttcTTCAG TTTCCTTATC TGTAAAATGG Ggataacaat
P939219      850 TTAGCTTTTA agT--TTCAA TTTCCTTTTC TGTAGAATGG GAGAACGACC
P939220      785 TTAGCGTTTA caT--TTCAA TTTCCTTTTC TGTAGAATGG GAGAACTACC
                 **********   *  ***** ********** ********** **********
                 **********   *  ***** ********** ********** **********
                 **********   *  ***** ********** ********** **********
                 *********    *  ***** ********** ********** **********
                 *********       ***** ********** ********** *
```

```
                                   ** ***  ********** ********** *
                                   ** ***  ********** ********** *

alignment position  1051......  1061......  1071......  1081......  1091......
P939218        926  acctacttta  aggtttgTTA  TGAGGATTAC  ATTCTTTAAT  ATACATCAGG
P939219        898  TgTTACACAA  GGTGT--TTG  TAAGGATTAA  AATTTTAAAT  ATACATCAAA
P939220        833  TaTTACACAA  GGTGT--TTG  CGAGGATTAA  AATTTTAAAT  ATACgttAAA
                    *  ******** *****  ***  ********** ********** **********
                    *  ******** *****  ***  ********** ********** **********
                    *  ******** *****  ***  ********** ********** ****   ***
                    *  ******** *****  ***  ********** ********** ****   ***
                                       ***  ********** ********** ****   ***
                                                             ***  ****
                                                             ***  ****

alignment position  1101......  1111......  1121......  1131......  1141......
P939218        976  CCTTTAGCAC  ATGGTAAAAG  CTGAGT----  ----------  ----------
P939219        946  CCCTTAGCAC  GTGATAAAGG  CTCAGTAATT  TTTCTTTtct  ttttattttta
P939220        881  CCTTTAGCAC  ATGATAAAAG  CTCAGTTATT  TTATTTTat-  ----------
                    ********** **********  ********** ******
                    ********** **********  ********** ******
                    ********** **********  ********** ******
                    ********** **********  ******
                    ********** **********  ******
                    ********** **********  ******

alignment position  1151......  1161......  1171......  1181......  1191......
P939218       1002  ----------  ----------  ----------  ----------  ----------
P939219        996  tttatttatg  catttaTTTG  CACATTTGgt  -TTTTGCTAA  AAGccaCCAG
P939220        920  ----------  ------TTTG  CACATCTGtg  gTTTTGCTGA  AAGatgCCAA
                                      ****  ********    ********* ***    ****
                                                                         ****
                                                                         ****

alignment position  1201......  1211......  1221......  1231......  1241....
P939218       1002  ----------  ----------  ----------  ----------  --------
P939219       1045  GCAGTCAGGA  ACTCCTATGT  AGCCCCATTC  CACAGtgaag  g-------
P939220        954  GCAGTCAGGA  ACTCGTGTGT  AACCCCATTC  TACAGaaaag  agaagaat
                    ********** **********  ********** *****
                    ********** **********  ********** *****
                    ********** **********  ********** *****
```

Pairwise similarities:

For each pairwise alignment, the similarity (relative to the maximum similarity) and the number of identical nucleic acids (in % of shorter sequence) is given. Maximum values are underlined.

	P939219 (1085 bp)	P939220 (1001 bp)
**P939218 (1001 bp)**	0.264<br>46 %	0.245<br>39 %
**P939219 (1085 bp)**		<u>1.000</u><br><u>82 %</u>

Please note that the **similarity value 1.000** marks only the two **most similar** sequences, it does not necessarily mean that these sequences are identical.

**Extract alignment region**	
**Positions in alignment**	from: to:
**Output file**	dialign.seq

Extract aligned sequences

For comments, questions or bug reports, please contact support@genomatix.de

© Genomatix Software GmbH 1998-2005
- All rights reserved.

License Agreement

GEMS Launcher 4.1.1

Genomatix-Logo Logout Personal Messages GEMS-Logo

Genomatix_MainGEMSElDoradoGene2PromoterBiblioSphere
Frequently_asked_questionsYour_ResultsYour_SequencesYour_ProtocolHelp

GEMS Launcher Task: *DiAlign TF*: Multiple alignment plus TF sites
working on C4-COX17 (3 seq.)

[Alignment] [Pairwise_similarities]

DiAlign professional TF Release 3.1 December 2004 **Thu May 12 21:56:38 2005**

Solution parameters:

**Sequence file:**	C4-COX17 (3 seq.)
**Type of sequences:**	nucleotide sequences
**Threshold T:**	0.00
**'*' signs below alignment denote:**	diagonal similarity (max. similarity is represented by 10 '*' signs)
**Alignment output:**	complete alignment is shown<br># nucleic acids per line: 50
**TF output:**	common TF matches located in aligned regions (common to 3 (100.0 %) sequences)
**Family matches:**	yes
**MatInspector library:**	Matrix Family Library Version 5.0 (February 2005)
**Selected groups** (core/matrix sim):	• ALL user_defined.lib (0.75/Optimized)<br>• ALL vertebrates.lib (0.75/Optimized)

Aligned Sequences:

No.	Sequence Name	Sequence Description
1	P879435	P879435 sym=COX17|loc=Loc10063|taxid=9606|spec=Homo sapiens|chr=3|ctg=NT_005612|str=(-)|start=25890889|end=25891889|len=1001|tss=501|comm=COX17 homolog, cytochrome c oxidase assembly protein (yeast); (NM_005694/501/bronze;)
2	mCOX17	mCOX17 m5_dna range=chr16:38203020-38204074 5'pad=0 3'pad=0 revComp=FALSE strand=? repeatMasking=none
3	P879436	P879436 sym=Cox17|loc=Loc89786|taxid=10116|spec=Rattus norvegicus|chr=11|ctg=NW_047356|str=(-)|start=17580775|end=17581775|len=1001|tss=501|comm=cytochrome c oxidase, subunit XVII assembly protein homolog (yeast); (NM_053540/547/bronze;)

Alignment (DiAlign format):

Please note that only **upper-case** letters are considered to be **aligned**. For more information, please have a look at the user guide.

V$HESF V$ETSF U$NRF2 V$SP1F V$ZNFP

```
alignment position 1.........  11........  21........  31........  41........
P879435          1 ccaatagttg tggactcggt ggttc----- ---------- ----------
mCOX17           1 ---------- ---------- ---------- ---------- ----------
P879436          1 accaaatatc tgcggaacat atattaaacc agaaagaccg gaagaaagat

alignment position 51........  61........  71........  81........  91........
```

```
P879435         26 ---------- ---------- ---------- ---------- ----------
mCOX17           1 ---------- ---AGGGTGG AACCCGGATC TGCTACTGCT TGTCCAGTAC
P879436         51 cacaaaatgc cctAGGGTGG AACCTGGATC TGCCACTGGT TGTCCAGGAC
                                  ******* ********** ********** **********
                                  ******* ********** ********** **********
                                  ******* ********** ********** **********

alignment position 101....... 111....... 121....... 131....... 141.......
P879435         26 ---------- ---------- ---------- ---------- ----------
mCOX17          38 CaaaAGGTCA GCCCTGAAAA TACGAATACA GTTAGCATTA tacTGGACTG
P879436        101 CcagAGGTCA GCCATTAAAA TATGAATACA GATAGCATTA cagTGGACTG
                   *   ****** ********** ********** **********    *******
                   *   ****** ********** ********** **********    *******
                   *   ****** ********** ********** **********    *******

alignment position 151....... 161....... 171....... 181....... 191.......
P879435         26 ---------- ---------- ---------- ---------- ----------
mCOX17          88 AATGGGTCAT ATAGGAATAT ATGTATgcat gtatACATAT AAGCATGCAA
P879436        151 AACGGGTTAT AAAGGAATAT ATGTAT---- ----ACATAT ATGCATGCAA
                   ********** ********** ******         ****** **********
                   ********** ********** ******         ****** **********
                   ********** ********** ******         ****** **********
                                                        ****** **********

alignment position 201....... 211....... 221....... 231....... 241.......
P879435         26 ---------- ---------- ---------- ---------- ----------
mCOX17         138 TAACAATTAG GGAGGAAAAG GCCAtGAATT TGAAGGATAG TGGGGAATGG
P879436        193 TAACAATTAG GGAGGAAAAG GCCAcGAATT TGAAGGATAG TGGGGAACGG
                   ********** ********** **** ***** ********** **********
                   ********** ********** **** ***** ********** **********
                   ********** ********** **** ***** ********** **********
                   ********** ********** ****

alignment position 251....... 261....... 271....... 281....... 291.......
P879435         26 -TATGTCTTG CTATTCATTT TTAAATAGGT CAATGAAAGA Gaggaaaggc
mCOX17         188 GTATGTATAT GTAAGGATTT GTAGAAAGGA AAGGGAAAGG GAAAATGATG
P879436        243 GCGTGTATAT GTgAGGATTT GTAGAAAGGA AACAGAAAAG GGAAATGTTG
                   ********** ********** ********** ********** **********
                   ********** ** ******* ********** ********** **********
                   ********** ** ******* ********** ********** **********

alignment position 301....... 311....... 321....... 331....... 341.......
P879435         75 ctgcagtgtg tggggatcag gaagaaggcg gataaaagaa aagggggaaa
mCOX17         238 TAAttAAATT ACAATCTCAA AAACAA---- ---------- ----------
P879436        293 CAAtcAAATT ACAATCTCAA AAACAA---- ---------- ----------
                   ***  ***** ********** ******
                   ***  ***** ********** ******
                   ***  ***** ********** ******

alignment position 351....... 361....... 371....... 381....... 391.......
P879435        125 ctagtctgtt attcaaggga actggctttc acttatggtt tctcctgttt
mCOX17         264 ---------- ---------- ---------- ---------- ----------
P879436        319 ---------- ---------- ---------- ---------- ----------

alignment position 401....... 411....... 421....... 431....... 441.......
P879435        175 ggcttgAACA AATCATTTCA CCATCTTGGG CTTCCGTTTc gatattgggt
mCOX17         264 ------AACA AAAACAAAAA CCATTCTGGG TGACCTTTTA ACCT------
P879436        319 ------ACCA AATAGCAAAA CCAccctaga TGACCTTTTA ACCT------
                         **** ********** ********** ********** ****
                         **** ********** ***        ********** ****
                         **** ********** ***

alignment position 451....... 461....... 471....... 481....... 491.......
P879435        225 ggggaggagc gggtgttacg agatctctca gttgtcttgc tgctttcttt
mCOX17         302 ---------- ---------- ---------- ---------- ----------
P879436        357 ---------- ---------- ---------- ---------- ----------
```

```
alignment  position 501.......  511.......  521.......  531.......  541.......
P879435        275  ctt cgtgagc cct ccccaag tt gatt taac aaatactct at gacctagag
mCOX17         302  ---------- ---------- ---------- ---------- ----------
P879436        357  ---------- ---------- ---------- ---------- ----------

alignment  position 551.......  561.......  571.......  581.......  591.......
P879435        325  tcctagagtg taaataccac agtccaggtg agcgttcagc gggcctccTT
mCOX17         302  ---------- ---------- ---------- ---------- --------TT
P879436        357  ---------- ---------- ---------- ---------- --------TT
                                                                        **
                                                                        **

alignment  position 601.......  611.......  621.......  631.......  641.......
P879435        375  GCGCATGCGC TGAAGggggc accctctcgt taaggccgaa attttgccTC
mCOX17         304  GCGCCAGCGC TGACGCCACG CCCttggagc gaggccttgt accact--TC
P879436        359  TGGTCTGCGC TAAAGCCACG CCC------- ---------- ----------
                    ********** ********** ***                           **
                    ********** ********** ***

alignment  position 651.......  661.......  671.......  681.......  691.......
P879435        425  TTCTGCGCAC GCGCTCCTTt taaatt cccc acctgtcaga GAGAAGCAGG
mCOX17         352  TTTTGCGCAC GCGCTTCTTA GACTta CTTC CGTCCG---- GAGAGGCGGG
P879436        382  TTTTGTGTGC GCGCTCCTTA GGCTcg CTTC CGTCCG---- GAGAGGAGGG
                    ********** ********** ****   **** ******     **********
                    ********** ********          **** ******     **********
                                                 **** ******     **********
                                                                 **********

alignment  position 701.......  711.......  721.......  731.......  741.......
P879435        475  ACTTCCTGTA CTTTTTAGAG CGACTGCcGG AAGTGACTGC GGACGAATCG
mCOX17         398  ACTTCCTGTC CCCCTTCGAT TGACTGC-GG AAGTGGAGAC AGACGGCTCA
P879436        428  ACTTCCTGTC TGCGTTCGCT TGACTGC-GG AAGTGGATAC AGACAGCTCG
                    ********** ********** *******  ** ********** **********
                    ********** ********** *******  ** ********** **********
                    *********     ****** *******  ** ********** **********
                    *********     ****** *******

alignment  position 751.......  761.......  771.......  781.......  791.......
P879435        525  GCGTTTGCCG AG-GCTGGca tagatttggc tgtctcCGCT CATAGCTGCT
mCOX17         447  GGGTAGTCGG AG-TTTGGGA GCTTTGCGCG TGTG--CGCT CATAGTTGCT
P879436        477  GGTTGGTCTG AGtTTTGGGA GCTTTGCGCG TGTG--CGat cgttaCTGCT
                    ********** **  ******* ********** ****  **** **********
                    **            ******* ********** ****  **** **********
                    **                                      **** **********
                                                            **        *****
                                                                       ****
                                                                       ****

alignment  position 801.......  811.......  821.......  831.......  841.......
P879435        574  TTTGGCGCGA AAGATGCCGG GTCTGGTTGa ctcaaaCCCT GCCCCGCCTG
mCOX17         494  TTCGCCTGGA AAGATGCCGG GACTGGCgGC CGCTAGCCCT GCCCCGCCCG
P879436        525  TTCCTCTGGA AAGATGCCGG GACTGGCTGC CGCTAGCCCT GCCCCGCCCG
                    ********** ********** ********** ********** **********
                    ********** ********** ********** ********** **********
                    ********** ********** *******  ** ********** **********
                    ********** ********** *******  ** ********** **********
                    ********** ********** ******   ** ********** **********
                    ********** ********** ******   *        **** **********
                                                            **** **********
                                                            **** **********
                                                            **** **********

alignment  position 851.......  861.......  871.......  881.......  891.......
P879435        624  AGTCTCAGGA GAAGAAGCCg CTGAAGCCCT GCTGCGCTTG CCCGGAGACC
mCOX17         544  AGGCTCAGGA GAAGAAGCCa CTGAAGCCCT GCTGTGCCTG CCCGGAAACC
P879436        575  AGGCTCAGGA GAAGAAGCCt CTGAAGCCCT GCTGTGCCTG CCCGGAGACC
```

```
                         ********** *********  ********** ********** **********
                         ********** *********  ********** ********** **********
                         ********** *********  ********** ********** **********
                         ********** *********  ********** ********** **********
                         ********** *********  ********** ********** **********
                         ********** ********   ********** ********** **********
                         ********** ********   ********** ********** **********
                         ********** ********   ********** ********** **********
                         ********** ********   ********** ********** **********
                                               ********** ********** **********

alignment  position      901....... 911....... 921....... 931....... 941.......
P879435          674     AAGAAGGCGC gcGATGCGTG GTCAGTGCGC AGCCGGAGAG GGcgcggccg
mCOX17           594     AAGAAGGCGC GTGATGCGTG GTCAGTGCGT AGCCCGGGAG GGaagcCCGG
P879436          625     AAGAAGGCGC GTGATGCGTG GTCAGTGCGC AGCCGGGGAG GGgactCCGG
                         ********** ********** ********** ********** **    ****
                         ********** ********** ********** ********** **    ****
                         ********** ********** ********** ********** **
                         **********   ******** ********** ********** **
                         **********   ******** ********** ********** **
                         **********   ******** ********** ********** **
                         **********
                         **********
                         **********

alignment  position      951....... 961....... 971....... 981....... 991.......
P879435          724     acgagccccg ggccgcggcc tgtgcCTTCC GGTGCctctg ccctacggcc
mCOX17           644     G--------- ---------- -----CTTTA GCAGCTCGG- ----------
P879436          675     G--------- ---------- -----CTTCC GCTGCTTGG- ----------
                         *                          ***** *********
                         *                          ***** *********

alignment  position      1001...... 1011...... 1021...... 1031...... 1041......
P879435          774     tctgccctCT GCCTTCTGCC CTCTGCCCTC T--------- ----------
mCOX17           659     --------CT CCACTCTGCT CTCTGGgact ctggagatgc agggacccat
P879436          690     --------CT CCGTTCTACT CTCTGGCATC T--------- ----------
                                 ** ********** ********** *
                                 ** ********** ******

alignment  position      1051...... 1061...... 1071...... 1081...... 1091......
P879435          805     ---------- ---------- ---------- ---------G CCTTctg---
mCOX17           701     agttccgctg tcctctctgc tccctgatcc cgcaccccaG TCTCAGTTAG
P879436          713     ---------- ---------- ---------- ---------G TCTTAGTTAG
                                                                  * **********
                                                                  * ****

alignment  position      1101...... 1111...... 1121...... 1131...... 1141......
P879435          813     ---------- ---------- ---------- ---CCCTCTG CCCTCTTCCT
mCOX17           751     GGGTCAACGg AGAGGGCAAG AGGTTACATT GATCCCAAGG TTCTCGTCCT
P879436          724     GGGTCAACG- AGAGGGCAAG AGGTTAGATT GACCCCAAAG TTCggGTCCT
                         *********  ********** ********** ********** **********
                                    ********** ********** ********** ***  *****
                                    ********** ********** ********** ***  *****

alignment  position      1151...... 1161...... 1171...... 1181...... 1191......
P879435          830     TCCCCCCTTC CCCCActcct cctccctgtc tgcccccccc ccccgccccg
mCOX17           801     TCATTCCTTA CTACGATT-- ---------- ---------- ----------
P879436          773     TCATTCCTTG CTACAAAT-- ---------- ---------- ----------
                         ********** ********
                         ********** ********
                         ********** ********

alignment  position      1201...... 1211...... 1221...... 1231...... 1241......
P879435          880     ccccgccccg ccccattgcg gcgctctgga gataattatt ggGCCTTGTA
mCOX17           819     ---------- ---------- ---------- ---------- --GCATTTTA
P879436          791     ---------- ---------- ---------- ---------- --GCCTTTCA
```

```
                                                                      *********
                                                                      *********
                                                                      *********

alignment position  1251......  1261......  1271.....  1281.....  1291......
P879435        930  GGTTTCGTGGt cgggtctct gttgacttat cccgtagctt agtaccggga
mCOX17         827  GTCTTCTTAg ggatcaagacc cagatcaaat a gaagTGCGTA AAGATGCTTC
P879436        799  GGCTTCTTAGa --------- ---------- ----TGCATA AAGATGTTCC
                    **********                            ********************
                    *********                             ********************
                    *********                             ********************

alignment position  1301......  1311......  1321.....  1331.....  1341......
P879435        980  aacgatggag agagaggaga ac-------- ---------- ----------
mCOX17         877  TTGATTTAAA GGCATTTAAA ATaTGTGGAG ATTTGTTACC AGCCAATACT
P879436        826  TTGATTTAAA GGCACTTAAA ATgTGTGGAG ACTTGTTACC AGCCAGTACT
                    ********************** ******** ********************
                    ********************** ******** ********************
                    ********************** ******** ********************

alignment position  1351......  1361......  1371.....  1381.....  1391......
P879435       1002  ---------- ---------- ---------- ---------- ----------
mCOX17         927  ATACCACCAG CTgtg--ATA GATTTACTAA GACCTAGGTG TAAATtTTTC
P879436        876  ATGCCACCTG CTatggtATA GATTTACTAA GACCCAGGTG TAAATgTTTC
                    ************    ******************************  ****
                    ************    ******************************  ****
                    ************    ******************************  ****

alignment position  1401......  1411......  1421.....  1431.....  1441......
P879435       1002  ---------- ---------- ---------- ---------- ----------
mCOX17         975  AGGTAATCTT TAGTAATTTT TTTTTTTTTt ggtcGAAACG TGTGAATAGT
P879436        926  AGGTAATCTC TAGTAATTTT TTTTTCTTT- ----GAAACG TGTGAATAGT
                    *****************************      ****************
                    *****************************      ****************
                    *****************************      ****************
                                                       ****************

alignment position  1451......  1461......  1471.....  1481
P879435       1002  ---------- ---------- ---------- -
mCOX17        1025  AGAACACAGG ATACATTTCT GAAGggatac c
P879436        971  AGAACACAGG GTACATTTCT GAAGggatac c
                    ************************
                    ************************
                    ************************
                    ************************
```

Pairwise similarities:

For each pairwise alignment, the similarity (relative to the maximum similarity) and the number of identical nucleic acids (in % of shorter sequence) is given. Maximum values are underlined.

	mCOX17 (1055 bp)	P879436 (1001 bp)
**P879435 (1001 bp)**	0.198<br>30 %	0.180<br>24 %
**mCOX17 (1055 bp)**		<u>1.000</u><br><u>79 %</u>

Please note that the **similarity value 1.000** marks only the two **most similar** sequences, it does not necessarily mean that these sequences are identical.

Extract alignment region

**Positions in alignment**	from: to:
**Output file**	dialign.seq

Extract aligned sequences

For comments, questions or bug reports, please contact support@genomatix.de

© Genomatix Software GmbH 1998-2005
- All rights reserved.

License Agreement

GEMS Launcher 4.1.1

Genomatix-Logo Logout Personal Messages GEMS-Logo

Genomatix_MainGEMSElDoradoGene2PromoterBiblioSphere
Frequently_asked_questionsYour_ResultsYour_SequencesYour_ProtocolHelp

GEMS Launcher Task: *DiAlign TF*: Multiple alignment plus TF sites
working on C4-CYCS (3 seq.)

[Alignment] [Pairwise_similarities]

DiAlign professional TF Release 3.1 December 2004 **Fri May 13 03:44:13 2005**

Solution parameters:

**Sequence file:**	C4-CYCS (3 seq.)
**Type of sequences:**	nucleotide sequences
**Threshold T:**	0.00
**'*' signs below alignment denote**:	diagonal similarity (max. similarity is represented by 10 '*' signs)
**Alignment output:**	complete alignment is shown<br># nucleic acids per line: 50
**TF output:**	common TF matches located in aligned regions (common to 3 (100.0 %) sequences)
**Family matches:**	yes
**MatInspector library:**	Matrix Family Library Version 5.0 (February 2005)
**Selected groups** (core/matrix sim):	• ALL user_defined.lib (0.75/Optimized)<br>• ALL vertebrates.lib (0.75/Optimized)

Aligned Sequences:

No.	Sequence Name	Sequence Description
1	P882274	P882274 sym=CYCS|loc=Loc54205|taxid=9606|spec=Homo sapiens|chr=7|ctg=NT_007819|str=(-)|start=24460128|end=24461228|len=1101|tss=601|comm=cytochrome c, som (NM_018947/501/bronze;)
2	P882275	P882275 sym=Cycs|loc=Loc13063|taxid=10090|spec=Mus musculus|chr=6|ctg=NT_039343|str=(-)|start=2241526|end=2242696|len=1171|tss=601,644,669,670,671|comm=c c, somatic; (AK005336/504/gold;AK010651/572/gold;AK088098/574/gold;AK003168/547/gold;NM_007808/573/bronze;)
3	rCYCS	rCYCS n3_dna range=chr4:79103604-79104758 5'pad=0 3'pad=0 revComp=TRUE strand=? repeatMasking=none

Alignment (DiAlign format):

Please note that only **upper-case** letters are considered to be **aligned**. For more information, please have a look at the user guide.

V$RORA V$RARF V$PERO V$HNF4 V$CREB V$E4FF V$CEBP V$ZF5F V$ZFIA V$ECAT V$PCAT V$HIFF
V$GREF V$EREF U$EREF

alignment position		1	1 1	2 1	3 1	4 1 . . . .
**P882274**	1	- - - - - - - - - -	- - - - - - - - - -	- - - - - - - - - -	- - - - - - - - - -	- - - - - - - - - -
**P882275**	1	T C A G G C G A A T	C C T G C T T C T C	C C T T A A A G C T	T G C C T G C A C A	C C C T C C T C G T
**rCYCS**	1	T C A G G C A A A T	C C T G T T T C T T	C C T T A A A G C T	T G C C T G C A C A	C C C T C C T T G T

```
              ** ******* ***  *********  **********  **********  **********
              ** ******* ***  *********  **********  **********  **********
              ** ******* ***  *********  **********  **********  **********
              ** ******* ***  *********  **********  *********

alignment
position        51.......     61........  71........  81........  91........

P882274      1  ----------  ----------  ----------  ----------  ----------
P882275     51  TTTCTACAAA  CACTAGGGGA  AGAAAACAGG  gactctgggt  aaGATGTGAT
rCYCS       51  TTTTTCTACA  AACTAGGGGA  AGAAAACAGG  ----------  --GATAAGAT
                **********  **********  **********                ********
                **********  **********  **********
                **********  **********  **********

alignment
position        101.......    111.......  121.......  131.......  141.......

P882274      1  ----------  ----------  ----TTCCTC  AGTCTTGGGG  CGTCCAtcaa
P882275    101  CCTGTCTGCC  ACTTGTGCGt  -ACCTTCCCC  AGCTTTGAGG  CTCTCACTG-
rCYCS       89  CCCGTCTGCC  ACATGTACCg  cACCATCCCC  AGCTTTGAGG  CTTTCACTG-
                **********  *********    *********  **********  *********
                                         *********  **********  *********
                                         *********  **********  *********

alignment
position        151.......    161.......  171.......  181.......  191.......

P882274     27  gcGGAGAGCT  GGAAtgtgac  tgaaagTAAC  AGAGTATAGG  Tggaactaga
P882275    149  --GGAGAGCA  GGAAGATGGT  T-----TAAC  AGAGTAAAGG  T---------
rCYCS      138  --GGAGAACA  -GAAGATGGT  T-----TAAC  AGAGTGAAGG  T---------
                  ********  **********  *     ****  **********  *
                  ********   *********  *     ****  **********  *
                  ********   *********  *     ****  **********  *
                             *********  *     ****  **********  *

alignment
position        201.......    211.......  221.......  231.......  241.......

P882274     77  acaaggtCAC  GAGCTGGTGC  CCAactcgtg  taactcctgg  ttccaggctg
P882275    183  -------CAC  CAGCAGGCGT  CCActgcGAG  GCCAGTGCAG  TT--------
rCYCS      171  -------CAC  CAGCAt----  -------GAG  TCCGGTGCAG  TT--------
                       ***  **********  ***    ***  **********  **
                       ***  *****
                       ***  *****
                       ***  *****

alignment
position        251.......    261.......  271.......  281.......  291.......

P882274    127  tccctctagg  atgctgttgG  CGCCAGCCgt  ggtagtagGA  CAGAGTCTGG
P882275    218  ----------  ---------G  CGCCAGCCAG  GaagccttGA  CCAAGCCTGG
rCYCS      195  ----------  ---------A  CGCTAGTCAG  GgaatctaGA  CCAAGCCTGG
                                     *  **********  *       **  **********
                                                            **  **********
                                                            **  **********
                                                            **  **********
                                                            **  **********

alignment
position        301.......    311.......  321.......  331.......  341.......

P882274    177  GTAAAGGTCG  CCTGCTGACa  agatggtagc  taggagacct  ctaggtgcgc
P882275    249  GTAAAGGTCG  CCAGCACACC  GGCCGGCaGT  CTCAGCACTC  CCCGTGGCGG
rCYCS      226  GTAAAGGTCG  CCTTCAGACT  GGGCGGCgGT  CTTAGCACTA  CCCGTGGCGG
                **********  **********  *******  **  **********  **********
                **********  **********  *******  **  **********  **********
                **********  **********  *******  **  **********  **********
                **********  *********            **  **********  **********
                **********  *****

alignment
position        351.......    361.......  371.......  381.......  391.......

P882274    227  caaaattagg  gcgtcttttc  ctggaccggc  aaaacgccct  gctcaaagct
P882275    299  GAGGCCGAAG  GAGTCCTAgg  gccgggc---  ----------  ----------
```

```
rCYCS       276 GAGGCAGAAG GAGTCCTAag agccgg---- ---------- ----------
                ********** ********
                ********** ********
                ********** ********
                ********** ********

alignment
position        401....... 411....... 421....... 431....... 441.......

P882274     277 cattacatca ccacgcccaa gaacTGATAC TTTAGCAGGG CAACGCACCA
P882275     326 ---------- ---------- -----GTTAC CTGAGCCGAG CCACACCCGG
rCYCS       302 ---------- ---------- ----TGTTAC CTGAGCCCAG CCGCACCCAA
                                          ****** ********** **********
                                           ***** ********** **********
                                           ***** ********** **********

alignment
position        451....... 461....... 471....... 481....... 491.......

P882274     327 ACCAAGCGac tccgcc--CC ACCCTCCCCG CTCCTCCAGC ACACGCaagc
P882275     351 ATCCCAAGGG AGCatcggCC CCGCTACTTG C-CCTCGCCA TCTCGC----
rCYCS       328 ATCCCGAGGG AGCgaccaCC CCGCTACTCG CTCCTCCCCA ACACGC----
                ********** ***     ** ********** ********** ******
                ********** ***                     ******** ******
                ********** ***                     ******** ******

alignment
position        501....... 511....... 521....... 531....... 541.......

P882274     375 gcacagAGGC CCGCGAGTGA CGTCACctct cccgacacca cctcgttgtc
P882275     396 ------AGGC CAGAGGGTGA CGTCACgCAC GTCCACGCCT ----------
rCYCS       374 ------AGGC CGGAGGGTGA CGTCAtcCAC GTCCACGCCT ----------
                      **** ********** ****** *** **********
                      **** ********** *****  *** **********
                      **** ********** *****  *** **********
                                             *** **********

alignment
position        551....... 561....... 571....... 581....... 591.......

P882274     425 gTACGACCAA AGGCCTGTCG TAACGGCCGG GAAAA----G CCTCGAGAAG
P882275     430 -TACGTCCTA AGGCCTGTCG TAAGTGTCGG GCAAACGAGG CCTCTGAAGG
rCYCS       408 -TACGTCCAA GGGCCTGTCG TAAGTGTCGG GCAAACGAGG CCTCTAGAGG
                 ********* ********** ********** ********** **********
                 ********* ********** ********** ********** **********
                 ********* ********** ********** ********** **********
                 ********* ********** ********** ********** **********
                 ********* ********** ********** *****    * **********
                 ********* ********** ********** *****
                                         ******* *****

alignment
position        601....... 611....... 621....... 631....... 641.......

P882274     471 AAGGGCACGC ACgccGTACA CCCTAACATG CTCGCCAGCA TGCGCGCGCA
P882275     479 AAGGGCGCCC TCTgGGCACA TCCTACCATG CTAGCTCGCA TGCGCGCGCA
rCYCS       457 AAGGGCGCCC TCTcGGTACA ACCTACCATG CTAGCCCGCA TGCGCGCGCA
                ********** ***  ****** ********** ********** **********
                ********** *** ****** ********** ********** **********
                ********** *** ****** ********** ********** **********
                ********** ***  ***** ********** ********** **********
                ******          ***** ********** ********** **********
                                ***** ********** ********** **********
                                  *** ********** ********** **********

alignment
position        651....... 661....... 671....... 681....... 691.......

P882274     521 ggtccttggc gcaggcattg ggaCTGGAGC CAATGAGGTG CGGCGACGTT
P882275     529 Caaacctg-- --------TC ---CTGGAGC CAATGAGGTG CGGTTACGTC
rCYCS       507 Ccttgctagc gctcccaaTC ---CTGGAGC CAATGACATG CGGCTACGTC
                *                  **    ******* ********** **********
                *                  **    ******* ********** **********
                *                  **    ******* ********** **********
                                   **    ******* ********** **********
```

```
                                                  *******  **********  **********
                                                  *******  **********  **********
                                                  *******  **********  **********
                                                  *******  **********  **********

alignment position    701.......  711.......  721.......  731.......  741.......
P882274          571  ACTGCGTAGC  cgccagctcg  GCCGCACGTC  AGGGCGCGGG  AGCGCGGAGC
P882275          566  ACGGCGCAGC  agaCGGCGCT  ACCGCACGTC  GGGCCGCGGA  CGGGCGGAAC
rCYCS            554  ACGGCGCAGt  gccCGGCGCT  GCCGCACGTC  CGGCCGCGGG  AGGGCAGAAC
                      **********     *******  **********  **********  **********
                      **********     *******  **********  **********  **********
                      **********              **********  **********  **********
                      *********               **********  **********  **********
                      *********                *********  *********    *********
                      *********                *********  *********    *********
                      *********                                        *********
                      *********                                        *********

alignment position    751.......  761.......  771.......  781.......  791.......
P882274          621  GAGTTTGGTT  GcacttACAC  CGGTACTTAA  GCGCGGACCG  GCGTGTCCTT
P882275          616  AAGTGTGGTT  GCACCGACAC  CGGTACATAG  GCGCGGGCCG  GCGTGTCCTT
rCYCS            604  AAGTGTGGTT  GCATTGACAC  CGGTACATAG  GCGCGGGCCG  GCGTGTCCTT
                      **********  **********  **********  **********  **********
                      **********  **********  **********  **********  **********
                      **********  **********  **********  **********  **********
                      **********  **********  **********  **********  **********
                      **********  *     ****  **********  **********  **********
                      **********  *     ****  **********  **********  **********
                      **********        ****  **********  **********  **********
                      **********        ****  **********  **********  **********
                                        ****  **********  **********  **********
                                        ****  **********  **********  **********

alignment position    801.......  811.......  821.......  831.......  841.......
P882274          671  GGACTtagag  agtgGGGACG  TCCGGCTTCG  gagcgggagt  GTTCGTTGTG
P882275          666  GGGCTAGAGC  GC--GGGACG  TCTGTCTTCG  AGTCCGAAC-  GTTCGTGGTG
rCYCS            654  GGGCTAGAGA  GC--GGGACG  TCTccctaaG  AGTCTGATC-  CTTTGTGGTG
                      **********  **  ******  **********  *********   **********
                      **********  **  ******  ***      *  *********   **********
                      **********  **  ******  ***      *  *********   **********
                      **********  **  ******  ***
                      **********  **  ******  ***
                      *****
                      *****
                      *****
                      *****
                      *****

alignment position    851.......  861.......  871.......  881.......  891.......
P882274          721  ccagcgacta  aaaa------  ----------  --------GA  GGTGAGAGCG
P882275          713  TTGACCAGCC  CGGAACGGTG  AGCGCGCGAC  GGGGATGCGG  ACCGGGAACG
rCYCS            701  TTGACCAGCC  CGGACCGGTG  AGCGCGCGAC  GGGGACGCGG  GGCGGGAAGA
                      **********  **********  **********  **********  **********
                      **********  **********  **********  **********  **********
                      **********  **********  **********  **********  **********

alignment position    901.......  911.......  921.......  931.......  941.......
P882274          747  GGTCGCGGAG  GCCGCACCTG  GTTAGAg---  ---------G  CAGAGCTGTG
P882275          763  GGGCGAGGAG  GCCGGGGCAG  GCCTAACCTA  CAAAGCCATG  CAGAACTGTG
rCYCS            751  GGGCGAGGAG  ACCGAGGCAG  GCCTGACCTA  CAAAGACATG  CGGAACTGTG
                      **********  **********  **********  **********  **********
                      **********   *********  **********  **********  **********
                      **********   *********  **********  **********  **********
                                   ***                             *  **********
```

```
alignment position   951.......  961.......  971.......  981.......  991.......
P882274      785     Ggaggcgcgc  acttgcgagc  gaccgaaacc  caagcgggga  gcattcgagt
P882275      813     Gcac------  ----------  ----------  ----------  ----------
rCYCS        801     Gaggctc---  ----------  ----------  ----------  ----------
                     *
                     *
                     *
                     *

alignment position   1001......  1011......  1021......  1031......  1041......
P882274      835     ggagcccgcg  ctgggtggga  gggcggggag  tgaagaccct  ggactgtggt
P882275      817     ----------  ----------  ----------  ----------  ----------
rCYCS        808     ----------  ----------  ----------  ----------  ----------

alignment position   1051......  1061......  1071......  1081......  1091......
P882274      885     cagaccgagc  tgggcgagta  acggcttgag  gtgcggcgga  gccctaacta
P882275      817     ----------  ----------  ----------  ----------  ----------
rCYCS        808     ----------  ----------  ----------  ----------  ----------

alignment position   1101......  1111......  1121......  1131......  1141......
P882274      935     gggacaggta  tggtctcggt  caggGACTGG  AGGCGGCTTG  GATACAGATC
P882275      817     ----------  ----------  ----GGCTGG  AGGCGGCGTG  GGTGTAGGAC
rCYCS        808     ----------  ----------  ----------  ------CGTG  GGTGAAGGGC
                                                 ******  **********  **********
                                                               ****  **********
                                                               ****  **********

alignment position   1151......  1161......  1171......  1181......  1191......
P882274      985     CGAGGAggag  gcggcctctt  ccgtagtggt  tgctgaaggg  ctatggaaat
P882275      843     CGGGGCCCGG  AGTTCTAGAA  GGAACTcGCC  CGCCGCCCCT  TCT-------
rCYCS        822     TGGGGCCCAG  AGTTCTAGAG  GGAGCTtGCC  CGCCGCACCT  TCT-------
                     **********  **********  ****** ***  **********  ***
                     **********  **********  ****** ***  **********  ***
                     **********  **********  ****** ***  **********  ***
                                                    ***  **********  ***

alignment position   1201......  1211......  1221......  1231......  1241......
P882274     1035     gataggcaag  acttccctcc  tgGAAAGCCG  AAGCTtagag  cttcac----
P882275      886     ----------  ----------  --GAGAGCAG  AAGCTGCTGT  TCCTTcAGGA
rCYCS        865     ----------  ----------  --GAGAGCAG  AAGCTGCTGT  TTCTTaAGAA
                                               ********  **********  ***** ****
                                               ********  **********  *****
                                               ********  **********  *****
                                               ********  **********  *****

alignment position   1251......  1261......  1271......  1281......  1291......
P882274     1081     --------GT  TCTTCTTCAG  Aggcaaaa--  ----------  ----------
P882275      914     CTGGGCcaGT  TCTTTTCCTG  ATTAAGCATC  TGGGAGGGTG  GGTTTgtaaa
rCYCS        893     CTGGGCtgGT  TCTTTTTCTG  ATTAAGTTTC  TGGAAAGGTG  GGTTTcttca
                     ******  **  **********  **********  **********  *****
                             **  **********  **********  **********  *****
                             **  **********  **********  **********  *****

alignment position   1301......  1311......  1321......  1331......  1341......
P882274     1102     ----------  ----------  ----------  ----------  ----------
P882275      964     g---------  -------TCG  TAGTCACCCC  TGGGATCTC-  -AACGTGAGA
rCYCS        943     gtcccctttg  gatgaccTCG  TGGTTACCCC  TGGGATCTCa  gAACGCGAGA
```

```
                          *** ********** *********  *********
                          *** ********** *********  *********

alignment
position       1351......  1361......  1371......  1381......  1391......

P882274   1102 ----------  ----------  ----------  ----------  ----------
P882275    996 GTGAGACGGT  TCCTTCCCca  gtcctccctc  GCCTTCCCGT  GCCGGACTAA
rCYCS      993 GTGAGAAGGT  TCCTTCCCtt  ----------  GCCTTCCCGG  GCCGGACTAA
               **********  ********                **********  **********
               **********  ********                **********  **********

alignment
position       1401......  1411......  1421......  1431......  1441......

P882274   1102 ----------  ----------  ----------  ----------  ----------
P882275   1046 AGCAAGAGag  aagaGACTTA  ACCTCCTAAT  GAGACCGGAG  CAGGc---GG
rCYCS     1033 TGCAAGAGt-  ----GACTTA  ACCTCCAAAT  GAGACCAGAG  CAGGaggaGG
               ********        ******  **********  **********  ****    **
               ********        ******  **********  **********  ****
                               ******  **********  **********  ****

alignment
position       1451......  1461......  1471......  1481......  1491......

P882274   1102 ----------  ----------  ----------  ----------  ----------
P882275   1093 AAGCACTTAG  GATCaccccc  agcc------  ----------  ---TCCCTTA
rCYCS     1078 AAGCACTTAG  GATCttcagc  cccgagcccc  cattgatgta  tttTCCCTTA
               **********  ****                                   *******
                                                                  *******
                                                                  *******

alignment
position       1501......  1511......  1521......  1531......  1541....

P882274   1102 ----------  ----------  ----------  ----------  --------
P882275   1124 TCTTTGGAAG  TGACTTTAAt  cggcagcact  gacagcagtt  tagcattc
rCYCS     1128 TCTTTGGAAG  TGACTTTAAa  tgacagc---  ----------  --------
               **********  *********
               **********  *********
               **********  *********
```

Pairwise similarities:

For each pairwise alignment, the similarity (relative to the maximum similarity) and the number of identical nucleic acids (in % of shorter sequence) is given. Maximum values are underlined.

	**P882275 (1171 bp)**	**rCYCS (1154 bp)**
**P882274 (1101 bp)**	0.178<br>33 %	0.182<br>28 %
**P882275 (1171 bp)**		<u>1.000</u><br><u>79 %</u>

Please note that the **similarity value 1.000** marks only the two **most similar** sequences, it does not necessarily mean that these sequences are identical.

**Extract alignment region**	
**Positions in alignment**	from: to:
**Output file**	dialign.seq

Extract aligned sequences

For comments, questions or bug reports, please contact support@genomatix.de

© Genomatix Software GmbH 1998-2005
- All rights reserved.

License Agreement

GEMS Launcher 4.1.1

Genomatix-Logo Logout Personal Messages GEMS-Logo

Genomatix_MainGEMSElDoradoGene2PromoterBiblioSphere
Frequently_asked_questionsYour_ResultsYour_SequencesYour_ProtocolHelp

GEMS Launcher Task: *DiAlign TF*: Multiple alignment plus TF sites
working on C4-HCCS (3 seq.)

[Alignment] [Pairwise_similarities]

DiAlign professional TF Release 3.1 December 2004 **Fri May 13 00:43:02 2005**

Solution parameters:

**Sequence file:**	C4-HCCS (3 seq.)
**Type of sequences:**	nucleotide sequences
**Threshold T:**	0.00
**'*' signs below alignment denote:**	diagonal similarity (max. similarity is represented by 10 '*' signs)
**Alignment output:**	complete alignment is shown<br># nucleic acids per line: 50
**TF output:**	common TF matches located in aligned regions (common to 3 (100.0 %) sequences)
**Family matches:**	yes
**MatInspector library:**	Matrix Family Library Version 5.0 (February 2005)
**Selected groups** (core/matrix sim):	• ALL user_defined.lib (0.75/Optimized)<br>• ALL vertebrates.lib (0.75/Optimized)

Aligned Sequences:

No.	Sequence Name	Sequence Description
1	P928091	P928091 sym=HCCS|loc=Loc3052|taxid=9606|spec=Homo sapiens|chr=X|ctg=NT_011757|str=(+)|start=7095486|end=7096548|len=1063|tss=501,563|comm=holocytochrom c synthase (cytochrome c heme-lyase); (AK097815/501/gold;NM_005333/563/silver;)
2	P928092	P928092 sym=Hccs|loc=Loc15159|taxid=10090|spec=Mus musculus|chr=X|ctg=NT_083046|str=(-)|start=4043347|end=4044347|len=1001|tss=501|comm=holocytochrome c synthetase; (NM_008222/501/silver;)
3	rHCCS	rHCCS rn3_dna range=chrX:45670319-45671118 5'pad=0 3'pad=0 revComp=FALSE strand=? repeatMasking=non

Alignment (DiAlign format):

Please note that only **upper-case** letters are considered to be **aligned**. For more information, please have a look at the user guide.

V$STAT V$ETSF V$NFKB U$NRF2 V$SP1F

```
alignment position  1.........  11........  21........  31........  41........
P928091         1  ttattgccta  catccCTGAT  GGAAGAAAAT  GCTAAATTTC  TGTTGGAGGT
P928092         1  g---------  ----------  ----------  ----AAAAAC  CAAAAAAG--
rHCCS           1  cctat-----  -----CTGCT  GGTTTACCAT  ACACAAATAC  TATAAAAGCT
                                    *****  **********  **********  **********

alignment position 51.........  61........  71........  81........  91........
P928091        51  AAa-------  ----------  ----------  ----------  ----TGAAAA
```

```
P928092        16 ---------- ---------- ---------- ---------- ----------
rHCCS          41 TActttatat aatgtgaaat ataatacttt tattatatga aagcTGAAAA
                  **                                              ******

alignment position 101....... 111....... 121....... 131....... 141.......
P928091        60 TACAGACATT caaaatcaca tacccagtta agaagaattt agcagccaga
P928092        16 ---------- ---------- ---------- ---------- ----------
rHCCS          91 TTCAACGATT gcatgcacta ---------- ---------- ----------
                  **********

alignment position 151....... 161....... 171....... 181....... 191.......
P928091       110 acactgtctt gttcactttt ggatctccac caccgaagtt atgggCACCT
P928092        16 ---------- ---------- ---------- ---------- ----------
rHCCS         111 ---------- ---------- ---------- ---------- -----CACTC
                                                                   *****

alignment position 201....... 211....... 221....... 231....... 241.......
P928091       160 ACTAACGTTT CTTGAATCAT TGCTACAATT Aaaaccgata agattta---
P928092        16 ---------- ---------- ---------- ---------- ----------
rHCCS         116 ACAACTGTTA CTAGAGTCCT TGAAAGGACT Acgcacagaa gtttaagtgc
                  ********** ********** ********** *

alignment position 251....... 261....... 271....... 281....... 291.......
P928091       207 ---------- ---------- ---------- ---------- ----------
P928092        16 ---------- ---------- ---------- ---------- ----------
rHCCS         166 gtgtattgaa ttatagcact ttattccata aataagcaca aatatcactt

alignment position 301....... 311....... 321....... 331....... 341.......
P928091       207 ---------- ---------- ---------- ---------- ----------
P928092        16 --------AA AAAATACAAG TTGACCTTTC AAgAACCTAA GGAGATGACT
rHCCS         216 gttcgtcaAA AAAATACAAA TCGACCTTTC AAaAACCGAA GGAGATGACT
                          ** ********** ********** ** ******* **********
                          ** ********** ********** ** ******* **********
                                                      ******* **********

alignment position 351....... 361....... 371....... 381....... 391.......
P928091       207 ---AAAAAAA AACAAATTTA AAA-ACTCCT CCAAAGC-AC TCCTTCTGCC
P928092        58 TAGAAAAACA CAGCGACGTG ccttACCCCT CGGAAGCGAC ACCCcgcggt
rHCCS         266 GTGAAAAACA CAGCAACTTG ACA-ACTCCT CCAAAGCGAC TCCCTCTGCT
                  ********** ********** *** ****** ********** **********
                  ********** ********** *** ****** *******
                  ********** **********
                     ******* **********

alignment position 401....... 411....... 421....... 431....... 441.......
P928091       252 GCATAAagca tacatagtag agctcttcac tctagggaag cctctggagc
P928092       108 tccctaactt ctcaccctga gcggcttacc ggccggccgG CGGAGTTaa-
rHCCS         315 ACCTAAgtcc aacc------ ---------- ---------G CGGAGTTtct
                  ******                                   * *******

alignment position 451....... 461....... 471....... 481....... 491.......
P928091       302 agacaacctt ccctgccacc gccacattTT GAAAAAGGCT CCTGGCCTGG
P928092       157 ---------- ---------- ---------- -AAAACACCG CCTGGCCCGT
rHCCS         341 caaagagagc cgcagag--- --------TT GAAAACACCG CCCAGCCCTT
                                                ** ********** **********
                                                    ********* **********
                                                    ********* **********
                                                    ********* **********
                                                    ********* **********

alignment position 501....... 511....... 521....... 531....... 541.......
P928091       352 AGGGGCGGGC CCAGAAAGcg gcccccgca- ---------- ----------
P928092       176 AGGGGCGGAG ACGGAGAGAA Ggctcgggcg cctataggcc gcggagcgag
rHCCS         380 AGAGGCGGAG ACGGAGAGAA Ggcgg----- ---------- ----------
```

```
                      ********** ********** *
                      ********** ********** *
                      ********** ********** *
                      ********** ********** *
                      ********

alignment position    551....... 561....... 571....... 581....... 591.......
P928091           381 ---------- ---------- ---------- ---------- ----------
P928092           226 aaggggtgga ccgacccgcc cttaaacaca ccgccccgca cgtaggggcg
rHCCS             405 ---------- ---------- ---------- ---------- ----------

alignment position    601....... 611....... 621....... 631....... 641.......
P928091           381 ---------- ---------- GCAGTTACAG GCCGGGGCGA GGAGGGG-AC
P928092           276 gagacggaga gaaggctcGG GCGGCTCGAG GCCTCGGAGC GGAAGGGgGT
rHCCS             405 ---------- --------GG GCGGCTATTG GCCTCGGAGC GGAAGGG-GT
                                         ** ********** ********** ******* **
                                         ** ********** ********** ******* **
                                         ** ********** ********** *******

alignment position    651....... 661....... 671....... 681....... 691.......
P928091           410 GGAACCCGCT CTCCGTGATT GACggaaaat attaCTCTTC AGCCCGcctc
P928092           326 GGGAGCGATC CGCCCGACCT GACGAcc-GT TCCTCTCCTC AGCGCGagct
rHCCS             436 GGGACCGATC CTCCCTGACT GACAAgagGT TCCTTTCCTC AGCttgcggt
                      ********** ********** *****   ** ********** ******
                      ********** ********** ***

alignment position    701....... 711....... 721....... 731....... 741.......
P928091           460 ccgcgtg--- ---CCCGGAA GTCCCGCCTT CTAAAAAGGA AGTAGGGGTG
P928092           375 tgcctcgt-- GCTCCCGGAA GTCCCGCCTT CGGAACAGGA AGTAACGGCG
rHCCS             486 ttgtgtcagg GCTCCCGGAA GTCCCGCCTT CGGAAAAGGA AGTAACGGCt
                                 ********** ********** ********** **********
                                 ********** ********** ********** **********
                                 ********** ********** ********** **********
                                 ********** ********** ********** *********
                                 ********** ********** ********** *********
                                    ******* ********** ********** *********
                                    ******* ********** ********** *********
                                    ******* ********** ********** *********
                                    ******* ********** *  ******* ****
                                    ******* ********** *  ******* ****

alignment position    751....... 761....... 771....... 781....... 791.......
P928091           504 CGTGAGAACG CCGGCGGTGA CCGcagccac AGCGGTGACC GAGTGAGAGG
P928092           423 CCCGAGAACG CCGGCTGTGA CCGga-CGTT AGCGGTGACC CCGCGTGAGG
rHCCS             536 caCGAGAAAG CCGGCCGTGA CCcggtCGTT AGCGGTGACC CCGTGTGTGG
                      ********** ********** ***   **** ********** **********
                      ********** ********** ***   **** ********** **********
                      ********** ********** ***   **** ********** **********
                        ******** ********** **         ********** **********

alignment position    801....... 811....... 821....... 831....... 841.......
P928091           554 AAggcggcgg cggcggcggc ggcggcgtga agtcactgct gctcTGGGTT
P928092           472 AAAAACCGCG GGTGCagccg agcgac---- ---------- --------TT
rHCCS             586 AAAAGCGGCA GGTGCggttg gtggctgtgg ag-------- ----TGTGTT
                      ********** *****                                ******
                      ********** *****                                ******
                      ********** *****                                    **
                      **                                                  **
                                                                          **

alignment position    851....... 861....... 871....... 881....... 891.......
P928091           604 CGGGTTGGCG ACTGAAGGCG GTACCGGCCT CCCGGaac-- ----AGCCCG
P928092           500 CGGCTGGGCG GTTCGACGCG CTCGCGGCCG CGCCGcgcgg gacaAGTCGG
rHCCS             624 CGGCTTGGCG GTTCAACGCG GTCGCGGCCG CGCG------ ----------
                      ********** ********** ********** *****          ******
                      ********** ********** ********** ****
                      ********** ********** ********** ***
                      ********** ********** ********** ***
```

```
                        ********** ********** ********** ***
alignment position      901....... 911....... 921....... 931....... 941.......
P928091            648  GGGGAGGGCT TAGGTGCAGa agggcaggct GGCCGCGGCC GGTTTGGTCT
P928092            550  AGCGGAGACT TGGGGGCAG- ---------- GGCGGCGCCC GGGTTCGGCT
rHCCS              658  ---------- ---------- ---------- ---------- ----------
                        ********** *********             ********** **********
alignment position      951....... 961....... 971....... 981....... 991.......
P928091            698  GGGGACCACG ---------- ---------- ---------- ----------
P928092            589  GGAGCCGGCG gctgccgagt gagttcggct gggcaggttg acgcgctcgg
rHCCS              658  ---------- ---------- ---------- ---------- ----------
                        **********
alignment position      1001...... 1011...... 1021...... 1031...... 1041......
P928091            708  ---------- ---------- ---------- ---------- ----------
P928092            639  ggccgcgcgg cgcgGGACAG TCGGCGAGGT GACTTGGGGA CAGgACGGCG
rHCCS              658  ---------- ----GGACGG TTGGAGAGGA GACTCGGGGA CAG-ACGGTG
                                       ****** ********** ********** *** ******
                                       ****** ********** ********** *** ******
alignment position      1051...... 1061...... 1071...... 1081...... 1091......
P928091            708  ---------G GCTGGAGCAG GTGggcaagc gccgggacgg t---------
P928092            689  TCCGGGTCCG GCTGGAGCCG GTGCACTGTG CAGAccg--- --CGGTGCCG
rHCCS              693  TCCAGGTTAG GCTGGAGTCT GTGACCTGTG GACAcctcgg acCGGTAGAG
                        ********** ********** ********** ****         ********
                        ********** ********** ********** ****
alignment position      1101...... 1111...... 1121...... 1131...... 1141......
P928091            740  ---------- ---------G TGGGGAAGAC ACTTGGCagc tt--------
P928092            734  GTGGGACGCG CATAGACGGg aaGGCAACAC TGGTGGCTTC CTGCCCTTCG
rHCCS              743  GTGGGGCGCG CTTAGACAGG TGGGTAAGAT TTGTGGCTCC CTGCCCTTTG
                        ********** ********** ********** ********** **********
                                                ******** ********** **********
alignment position      1151...... 1161...... 1171...... 1181...... 1191......
P928091            763  ---------- -CTCGACCTC TGGTGTCCCT TCGTTTCTTC GAATTCCTAT
P928092            784  CCGCTccttg cCTCCCGTTT TGGTGACTTT TTTTTTTTTA ACCTTCCTAT
rHCCS              793  CCTCTtgt-- ---------- ---------- ---------- ----------
                        *****       ********* ********** ********** **********
                        *****
alignment position      1201...... 1211...... 1221...... 1231...... 1241......
P928091            802  Tcatgcgccg ca-----CCC TTCTCGGACC CCCAGCCCCT CGCTAaggct
P928092            834  Tttccctgag gcgcctgCCC TACTGGGAAC ACGGGTTCGA GGCTA-----
rHCCS              801  ---------- ---------- ---------- ---------- ----------
                        *                 *** ********** ********** *****
alignment position      1251...... 1261...... 1271...... 1281...... 1291......
P928091            847  ctggggatgg gggtcaagtc tagGCTCAAT GGACCTCCCC TCCTCCACCC
P928092            879  ---------- ---------- ---GTTCAGT GTATTTTCCC TTGGACCCCA
rHCCS              801  ---------- ---------- ---------- ---------- ----------
                                                 ******* ********** **********
alignment position      1301...... 1311...... 1321...... 1331...... 1341......
P928091            897  GGGTCACCAC TCCtcggaaa tcctcgcccc actcccgcct caggcctgta
P928092            906  CCATCAAGAC TACacccgtc ttcac----- ---------- ----------
rHCCS              801  ---------- ---------- ---------- ---------- ----------
                        ********** ***
alignment position      1351...... 1361...... 1371...... 1381...... 1391......
P928091            947  cctcctggct gtGACGTGGT Ttctcagcga ctgcctcacc tcagtcccaa
P928092            931  ---------- --GACGTGGT Tgcatcttac aagtttccag actctggc--
rHCCS              801  ---------- ---------- ---------- ---------- ----------
                                     ******** *
alignment position      1401...... 1411...... 1421...... 1431...... 1441......
```

```
P928091    997 aatcctcact gccttagata cat ACGTGCC CCCAGAGGTT GGCCTGGGTT
P928092    967 ---------- ---------- --- ACGGGTC CCCAAATGGT GGCATAGGCT
rHCCS      801 ---------- ---------- --- ------- ---------- ----------
                                         ******* ********** **********

alignment position 1451...... 1461...
P928091   1047 TCACAGTAtt tccctaa
P928092    994 TCAAGGTA-- -------
rHCCS      801 ---------- -------
               ********
```

Pairwise similarities:

For each pairwise alignment, the similarity (relative to the maximum similarity) and the number of identical nucleic acids (in % of shorter sequence) is given. Maximum values are underlined.

	P928092 (1001 bp)	rHCCS (800 bp)
**P928091 (1063 bp)**	0.260<br>26 %	0.282<br>33 %
**P928092 (1001 bp)**		<u>1.000</u><br><u>51 %</u>

Please note that the **similarity value 1.000** marks only the two **most similar** sequences, it does not necessarily mean that these sequences are identical.

**Extract alignment region**	
**Positions in alignment**	from: to:
**Output file**	dialign.seq

Extract aligned sequences

For comments, questions or bug reports, please contact support@genomatix.de

© Genomatix Software GmbH 1998-2005 - All rights reserved. License Agreement GEMS Launcher 4.1.1

Genomatix-Logo Logout Personal Messages GEMS-Logo

Genomatix_MainGEMSElDoradoGene2PromoterBiblioSphere
Frequently_asked_questionsYour_ResultsYour_SequencesYour_ProtocolHelp

GEMS Launcher Task: *DiAlign TF*: Multiple alignment plus TF sites
working on promoters.seq (3 seq.)

[Alignment] [Pairwise_similarities]

DiAlign professional TF Release 3.1 December 2004 **Tue May 3 21:44:27 2005**

Solution parameters:

**Sequence file:**	promoters.seq (3 seq.)
**Type of sequences:**	nucleotide sequences
**Threshold T:**	0.00
**'*' signs below alignment denote**:	diagonal similarity (max. similarity is represented by 10 '*' signs)
**Alignment output:**	complete alignment is shown<br># nucleic acids per line: 50
**TF output:**	common TF matches located in aligned regions (common to 3 (100.0 %) sequences)
**Family matches:**	yes
**MatInspector library:**	Matrix Family Library Version 5.0 (February 2005)
**Selected groups** (core/matrix sim):	• ALL user_defined.lib (0.75/Optimized)<br>• ALL vertebrates.lib (0.75/Optimized)

Aligned Sequences:

No.	Sequence Name	Sequence Description	Length
1	P881957	sym=SURF1|loc=Loc6834|taxid=9606|spec=Homo sapiens|chr=9| ctg=NT_035014|str=(-)|start=3000201|end=3000801|len=601| tss=501|comm=surfeit 1; (NM_003172/501/silver;)	601 bp
2	P881959	sym=Surf1|loc=Loc20930|taxid=10090|spec=Mus musculus|chr=2| ctg=NT_039206|str=(-)|start=4344877|end=4345492|len=616| tss=503,516| comm=surfeit gene 1; (AK002469/503/gold;NM_013677/516/bronze;)	616 bp
3	P881961	sym=Surf1|loc=Loc64463|taxid=10116|spec=Rattus norvegicus| chr=3|ctg=NW_047651|str=(-)|start=5813658|end=5814275| len=618|tss=501|comm=Surfeit 1; (NM_172068/501/bronze;)	618 bp

Alignment (DiAlign format):

Please note that only **upper-case** letters are considered to be **aligned**. For more information, please have a look at the user guide.

V$ZBPF V$EGRF V$OAZF V$ETSF U$NRF2

```
alignment position 1 . . . . . . . . .   1 1 . . . . . . . .   2 1 . . . . . . . .   3 1 . . . . . . . .   4 1 . . . . . . . .
P881957       1  t - - - - - - - - -   - - - - - - - - - -   - - - - - - - - - -   - - - - - - - - - -   - - - - - - - - - -
P881959       1  - - - - - - - - - -   - - - - - - - - - -   - - - - - - - - - -   - - - - - - T G G G   C A C A A T G T G T
P881961       1  g c a t t g g c c g   g c c a t c t a c g   t a c c g a t t c t   t t g t g c T G G G   C A C A A T G T G T
```

```
                                                     **** **********
                                                     **** **********
                                                     **** **********
                                                     **** **********

alignment position 51........ 61........ 71........ 81........ 91........
P881957          2 ---------- ---------- ---------- ---------- ----------
P881959         15 GGCTCGAAAG CTGCGTAATC GAAGttAGAG AAGGAACTTG ACAGCCGTTG
P881961         51 GGCTCGAACG CTGTGTAATC GAAGgcAGAG AAGGCACTTG AGAGCCGCTG
                   ********** ********** ****  **** ********** **********
                   ********** ********** ****  **** ********** **********
                   ********** ********** ****  **** ********** **********
                   ********** ********** ****  **** ********** **********

alignment position 101....... 111....... 121....... 131....... 141.......
P881957          2 -------TTG CCGCGGGTGT AGACCTGGAG CTCCGGCAGG CGGCAGGGCA
P881959         65 GTACTTCTTG CCGCGGGTGT ATTCCTGGAG CTCGGGCAGA CGGCAGGGCA
P881961        101 GTACTTCTTG CCGCTAGTGT AGACTTGGAG GTCCGGCAGA CGGCAGGGAA
                   ********** ********** ********** ********** **********
                   ********** ********** ********** ********** **********
                   ********** ********** ********** ********** **********
                   ********** ********** ********** ********** **********
                          *** ********** ********** ********** **********
                          *** ********** ********** ********** ******   *
                          *** ********** ********** ********** *******
                          *** ****            ***** ********** *******
                          *** ****            ***** ********** *******
                          *** ****            ***** ********** *******

alignment position 151....... 161....... 171....... 181....... 191.......
P881957         45 GCTCGTGACC TGTCAGGATG CACCTCACCT GCGGACGcgt gggcgggcag
P881959        115 GCTCATGGCC AGTTAGGGAG CAGCGAACCT GCGGATGAGC ATCTG-----
P881961        151 GCTCATGACC GGTTAGCGAG CAGCGTACCT GTGGATGAAC CGCTG-----
                   ********** ********** ********** ********** *****
                   ********** ********** ********** ********** *****
                   ********** ********** ********** ********** *****
                   ********** *****  *** ********** ********** *****
                   ********** *****  *** ********** *******
                   ********** *****  *** ********** ****

alignment position 201....... 211....... 221....... 231....... 241.......
P881957         95 gacGTCAGGA GGgccagggc tccgctccct ggtgccccct cctgccgcgc
P881959        160 ---GTCAGAA GGTGGtTCTT GCCAGCTCCG ---------- ----------
P881961        196 ---GTCAGAA GGTGGaTCTT GCCAGCTCCG ---------- ----------
                      ******* ***** **** **********
                      ******* ***** **** **********
                      ******* ***** **** **********
                      ******* ***** **** **********

alignment position 251....... 261....... 271....... 281....... 291.......
P881957        145 CCCCTTCCCT ctcctcccct gatcccgccg cgccctctcc cctgccctcc
P881959        187 CCCCTTCCCT GCA------- ---------- ---------- ----------
P881961        223 CCCCTTCCCT GCA------- ---------- ---------- ----------
                   ********** ***
                   ********** ***
                   ********** ***
                   ********** ***
                   **********

alignment position 301....... 311....... 321....... 331....... 341.......
P881957        195 cctctctcct gctctcccct ctcccctctc ccCTGCCCTC TCCTCT----
P881959        200 ---------- ---------- ---------- --CTGTCCTC ccaggtccca
P881961        236 ---------- ---------- ---------- --CTGCCCTC TCCTTC----
                                                      ******** ******
                                                      ********
                                                      ********
                                                      ********
                                                      ********

alignment position 351....... 361....... 371....... 381....... 391.......
```

```
P881957            241 ---------- ---------- -------CCC CTGCCTCCCC ctcagcccct
P881959            218 cgctttccag gcacaatact cccgggtCCC CCTCCTTTCT GGCCActgtC
P881961            250 ---------- ---------- -------CCC CTTCCTTTCC GGCCA----C
                                                    *** ********** *****    *
                                                    *** **********

alignment position     401....... 411....... 421....... 431....... 441.......
P881957            264 catcccaaCT GCGCCCTTCT CCGCCACTCC GTTCCCCTCC CGCGCTGcga
P881959            268 GGTC----CT CCGAGGTTCC GCTCCTCCCA GATCCCGTTC CTTCCTGGCC
P881961            269 GGTC----CT CCGAGGTCCC AATCCTTCCA GGTCCCGTTC CTTCCTGGCC
                       ****    ** ********** ********** ********** **********
                                                ******** ********** **********
                                                ******** ********** **********
                                                ******** ********** **********

alignment position     451....... 461....... 471....... 481....... 491.......
P881957            314 ---------- ---------- ---------A CCTTGCGGGC GTCCGTCTGG
P881959            314 CTACTCTTCC g-GTCACGGT TCTCTTCCGA CCTTGCGGGT GTTGGGCAGT
P881961            315 CTACTTTTCC ccGTCACGGT TCTCTTTCGA CCTTGTGGGT GTTGGGCAGC
                       **********   ******** ********** ********** **********
                       **********   ******** ********** ********** **********
                       **********   ******** ********** ********** **********
                       **********   ******** ********** ********** *********
                                                     * ********** *********
                                                     * ********** *********
                                                     * ********** *********

alignment position     501....... 511....... 521....... 531....... 541.......
P881957            335 AGCCGCAGGC TCGGGTGCTc cCGCAGAAAC GCCCGCACGT CGCCCGGCAA
P881959            363 AGTCTCAAGC TGGGGTGCTG GCGCAAGAAT GCAAGCACAT CAGAGGGTGG
P881961            365 AGTCTCAAGC TGGGGTGCTG ACGCAGAAAT GCAAGCACAT CAGAAGGTGG
                       ********** ********** ********** ********** **********
                       ********** ********** *****  *** ********** **********
                       ********** ********** *****  *** ********** **********
                       ********** *********         *** ********** **********
                       ********** *********         *** ********** **********
                                                    *** ********** **********

alignment position     551....... 561....... 571....... 581....... 591.......
P881957            385 CTCGCTCATG gccgacgcgc ccgCGGAGCA GCCCGGCGGA AGCCGCTCGC
P881959            413 TTCGTCCATG AT-------- ---CGGAGAT GCCCATCTGA CGCCGCAGCC
P881961            415 TTCGTCCATG AT-------- ---CGGAGac g-CCATCTGA CGCCACAGCC
                       ********** **            ******* ********** **********
                       ********** **            *****     ******** **********
                       ********** **            *****     ******** **********
                       ********** **            *****     ******** **********
                       ********** **            *****
                       **********               *****

alignment position     601....... 611....... 621....... 631....... 641.......
P881957            435 AGAACCTGGA GCCGGGGGCG Gggctctggg cgggactcag cggcgCCCCG
P881959            452 ACCACGTGAG ACAGAGCGCc GT-------- ---------- -----CCCGG
P881961            453 ACCACGTGAG GCGGAGCGCG GT-------- ---------- -----CCCGG
                       ********** ********** **                        *****
                       ********** *********  **                        *****
                       ********** *********  **                        *****
                       ********** *********  **                        *****
                                                                       *****

alignment position     651....... 661....... 671....... 681....... 691.......
P881957            485 GATGCAGATG CCTCCTGCGT CCCGGAAGCG CCCgcggggc cGGGTGCGAT
P881959            479 AAGGAAGCAG ATTTCTCGGT CCCGGAAGTG CTCAAAGAT- -GGCTGCTGT
P881961            480 AAGGAAGCAG GTTTCTCCGC CCCGGAAGTG tTCAAAGAT- -GGCTGCTGT
                       ********** ********** ********** ********    *********
                       ********** ********** **********  *******    *********
                       ********** ********** **********  *******    *********
                       ********** ********** **********  *******    *********
                       ********** ********** **********
```

```
alignment position   701.......  711.......  721.......  731.......  741.......
P881957         535  GGCGGCGGTG  GCTGCGTTGC  AGCTGGGGCT  Gcgggc----  ---------GG
P881959         527  GATGGCTTTG  GCTGTGCTGC  CGCGACGGAT  GACGCGGTGG  TCGCAATGGG
P881961         528  AATGGCTTTG  ACTGTCCTGC  gaagtCGGAT  AACGCGATGG  CCGCAGTGGG
                     **********  **********  **********  **********  **********
                     **********  **********       *****  **********  **********
                     **********  **********       *****  **********  **********
                     **********  **********

alignment position   751.......  761.......  771.......  781.......  791
P881957         573  CGGGGCTGGG  ACGGGTGAGC  GCCGGGTgc-  ----------  -
P881959         577  CCTACGCGGG  ACGGGTAAGC  GGGGGTTCAG  AGAGGATCCT  -
P881961         578  CCTGCGCGGG  GCCGGTAAGC  GGGGGTTCAG  AGAGGATCCT  g
                     **********  **********  **********  **********
                     **********     *******  **********  **********
                     **********     *******  **********  **********
                                    *******  **********  **********
```

Pairwise similarities:

For each pairwise alignment, the similarity (relative to the maximum similarity) and the number of identical nucleic acids (in % of shorter sequence) is given. Maximum values are underlined.

	P881959 (616 bp)	P881961 (618 bp)
P881957 (601 bp)	0.181 41 %	0.155 40 %
P881959 (616 bp)		1.000 81 %

Please note that the **similarity value 1.000** marks only the two **most similar** sequences, it does not necessarily mean that these sequences are identical.

Extract alignment region	
**Positions in alignment**	from: to:
**Output file**	dialign.seq

Extract aligned sequences

For comments, questions or bug reports, please contact support@genomatix.de

© Genomatix Software GmbH 1998-2005 - All rights reserved.

License Agreement

GEMS Launcher 4.1.1
