# Supplementary material for: Transcriptional co-expression and co-regulation of genes coding for components of the oxidative phosphorylation system
Source: BMC Genomics. 2008 Jan 14;9:18. doi: 10.1186/1471-2164-9-18 (PMC2268925; doi:10.1186/1471-2164-9-18)
Supplement: Additional file 8 — Phylogenetic footprinting of Complex V core promoters. Core Promoters of Complex V subunits/related genes identified by phylogenetic footprinting. The conserved regions are shown in color codes, which correspond to specific Transcription Factor Binding Sites (TFBS). [file 1471-2164-9-18-S8.pdf]

This Supplementary Material shows the Core Promoters of Complex V subunits/related genes identified by phylogenetic footprinting. The conserved regions are shown in color codes, which correspond to specific Transcription Factor Binding Sites (TFBS).

The description of the complete description of TFBS can be found in additional file 3 (in the “Matrix Family Library” Excell sheet).

Genomatix-Logo Logout Personal Messages GEMS-Logo
Logout Personal Messages

Genomatix_MainGEMSElDoradoGene2Promoter BiblioSphere
Frequently_asked_questions Your ResultsYour SequencesYour ProtocolHelp

GEMS Launcher Task: *DiAlign TF*: Multiple alignment plus TF sites working on promoters.seq (3 seq.)

[Alignment] [Pairwise_similarities]

DiAlign professional TF Release 3.1 December 2004 **Fri May 20 21:45:09 2005**

Solution parameters:

**Sequence file:**	promoters.seq (3 seq.)
**Type of sequences:**	nucleotide sequences
**Threshold T:**	0.00
**'*' signs below alignment denote:**	diagonal similarity (max. similarity is represented by 10 '*' signs)
**Alignment output:**	complete alignment is shown<br># nucleic acids per line: 50
**TF output:**	common TF matches located in aligned regions (common to 3 (100.0 %) sequences)
**Family matches:**	yes
**MatInspector library:**	Matrix Family Library Version 5.0 (February 2005)
**Selected groups** (core/matrix sim):	• ALL user_defined.lib (0.75/Optimized)<br>• ALL vertebrates.lib (0.75/Optimized)

Aligned Sequences:

No.	Sequence Name	Sequence Description	Length
1	P924542	sym=ATP5A1|loc=Loc498|taxid=9606|spec=Homo sapiens|chr=18|ctg=NT_010966|str=(-)|start=25166753|end=25167864|len=1112| tss=501,502,507,512|comm=ATP synthase, H+ transporting, mitochondrial F1 complex, alpha subunit, isoform 1, cardiac muscle; (AK092735/502/gold;AK129900/501/gold;AK129739/501/gold;AK098765/512/gold;NM_004046/507/bronze;)	1112 bp
2	P924549	sym=Atp5a1|loc=Loc11946|taxid=10090|spec=Mus musculus|chr=18|ctg=NT_039675|str=(+)|start=1236265|end=1237390| len=1126|tss=501,510,526|comm=ATP synthase, H+ transporting, mitochondrial F1 complex, alpha subunit, isoform 1; (AK043976/511/gold;AK098155/502/gold;AK076572/511/gold; NM_007505/527/silver;)	1126 bp
3	P924553	sym=Atp5a1|loc=Loc65262|taxid=10116|spec=Rattus norvegicus|chr=18|ctg=NW_047516|str=(+)|start=9660103|end=9661203| len=1101|tss=501|comm=mitochondrial H+-ATP synthase alpha subunit; (XM_341631/526/bronze;)	1101 bp

Alignment (DiAlign format):

Please note that only **upper-case** letters are considered to be **aligned**. For more information, please have a look at the user guide.

V$EBOX V$HIFF V$HAML V$RORA V$MYT1 V$SP1F V$ETSF U$NRF2 V$DEAF V$YY1F V$EREF U$EREF
V$TALE V$PAX5

```
alignment
position    1......... 11........ 21........ 31........ 41........

P924542   1 gactcagagg gtctgaagtg ggg------- ---------- ----------
P924549   1 tccacaagca tgCCTTGGTA CATATACATG CACACCACGC ATACactact
P924553   1 ---------- --CCTTGGCA CATACACATA CACAGCACAC ACACtaataa
                         ******** ********** ********** ****
                         ******** ********** ********** ****

alignment
position    51........ 61........ 71........ 81........ 91........

P924542  24 ---------- ---------- ---------- ---------- --CTAAGATC
P924549  51 actactacta attattatta TTTCAGTAAT TTCGGATAGT TCCCAGGACC
P924553  39 taataaacag ---------- TTTCAATAAT CTCAGAATGT TCTCAGGATC
                                  ********** ********** **********
                                  ********** ********** **********

alignment
position    101....... 111....... 121....... 131....... 141.......

P924542  32 TGCAGATTAA CGGGCTCCGT GGGAGgctcc ctgctggtgg ccagtcccgg
P924549 101 TGAGGACTAA AACCCTCTGG GTGGCTGCAC TGTGCAAGAG CTACCCCGgc
P924553  79 TGAGGACTAA AACCCTCTGG GTGGGTACAC TGTGCAAGAG TTTCCCCGac
            ********** ********** ********** ********** ********
            ********** ********** ********** ********** ********
                    ** ********** ********** ********** ********

alignment
position    151....... 161....... 171....... 181....... 191.......

P924542  82 ggtttagctg ttcactgttg ctactcgtgc taaACCACGG TCTAACACTA
P924549 151 ctgccacagc ---------- ---------- ---ACTGCGG TGAAACCCCA
P924553 129 ccggact--- ---------- ---------- ---------- ----------
                                             ******* **********

alignment
position    201....... 211....... 221....... 231....... 241.......

P924542 132 CCGACACTAT TAATCgccct ggtcctcaag acagccgcga CCCCAGACGC
P924549 178 CCAGCACGAT TAACCtaGCT CTTAGTCTAG GCAGCCCG-- CCCCAGGCAC
P924553 136 ---------- -------GCT CTTCATCTAT GCAGTTCG-- CCCTAGgtgg
            ********** *****  *** ********** ********   **********

alignment
position    251....... 261....... 271....... 281....... 291.......

P924542 182 G-TGAggccc tcatcctcta gcaccagatg gggcctggga actgctgctt
P924549 226 G-TGACGTTC AG-----GGA CGCGTCTCCT CT-------- ----------
P924553 167 gaTGAAGTTC AGttctaGGA CGCTTCCCCT CT-------- ----------
            *  ******* **     *** ********** **
                              *** ********** **
                              *** ********** **

alignment
position    301....... 311....... 321....... 331....... 341.......

P924542 231 agcaGATTCA GCTACCCACG TGGTCACCCG GGG--CAACC ACAAGGTCGA
P924549 252 ----GGTTCT GCTACTCACG TGGTCTCCCG -GG--CAACC ACAAGGTCGA
```

```
P924553 199 ----GGTTCT GCAACTCACG TGATCTCCCG GgcaaCAACC ACAAGGTCGA
                ****** ********** ********** ***  ***** **********
                ****** ********** **********  **  ***** **********
                ****** ********** *********       ***** **********
                ****** ********** *********       ***** **********
                  **** ********** *********       ***** **********
                                                  ***** **********
                                                  ***** **********

alignment
position    351....... 361....... 371....... 381....... 391.......

P924542 279 CTCAAAAgac CAAAATAAAT GTATAACGAA TGCCAGGACC AAAGCCGGAA
P924549 295 TTCAAAAAGA CCAAAAATTA AAATGTTGAG TACCAGGGAG GAGGCTGGAA
P924553 245 TTCAAAGAGA CCAAAAATTA AAATGATAAG TGCCAAGGAG GAGGCTGGAA
            ********** ********** ********** ********** **********
            ********** ********** *****   ** ********** **********
            ********** ********** *****   ** ********** **********
            ********** ********** *****
            *******
            *******
            ******

alignment
position    401....... 411....... 421....... 431....... 441.......

P924542 329 TATATCTAAT CATTCATTTT GAGGTTTAAA CTTAAAAATG TTACATAAAA
P924549 345 ACAATGTATT CGTTCATTTT TAGTTCTAAA CTTAAAAAAG AAAAAGAAAA
P924553 295 ACAATTGATT CGTTCATTTT TAGTCCTAAA CTTTAAAAAA AAAAAAAAAA
            ********** ********** ********** ********** **********
            ********** ********** ********** ********** **********
            ********** ********** ********** ********** **********
               ******* ********** ********** ********
               ******* ********** ********** ********
                         ******

alignment
position    451....... 461....... 471....... 481....... 491.......

P924542 379 AAataagtaa cctgcc---- ---------- --------AA AAAATGCAGA
P924549 395 Agttatag-- ---------- ---------- ----AAAAAA AAAAAAAAGA
P924553 345 AAaaagttgt taaaaaaaag aggcccatat cttaAAAAAA AAAAAAAAGA
            **                                ****** **********
            *                                 ****** **********
            *                                 ****** **********

alignment
position    501....... 511....... 521....... 531....... 541.......

P924542 407 GTGGCGGCGG GGGCGGGGGG Agaggtggtg aacgcGAGGG GCAGTACTTC
P924549 419 TTTGCGTCCG GAGGAGCAGG A--------- -----GAGGG GCGGTACTTC
P924553 395 TTTGCATCCG GAGGAGCTGG A--------- -----GAAGG GCGGTACTTC
            ********** ********** *               ***** **********
            ********** ******* ** *               ***** **********
            ********** ******* ** *               ***** **********
                                                  ***** **********
                                                  ***** **********
                                                           *******
                                                           *******
                                                           *******
                                                           *******
```

```
alignment
position        551....... 561....... 571....... 581....... 591.......

P924542  457    CGGGTCAGGT GGGCCGGCTG TCTTGACCTT CTTTGCGGCT CGGCCATTTT
P924549  455    CGGGTCAGGC GGGCTGGCTG CCTTGACCTT CCTTTGCGCT CGGCCATTTT
P924553  431    CGGGTCAGGT GGGCCGGCTG CCTTGACCTT CCTTCGCGCT CGGCCATTTT
                ********** ********** ********** ********** **********
                ********** ********** ********** ********** **********
                ********** ********** ********** ********** **********
                ********** ********** ********** ********** **********
                ********** ********** ********** ********** **********
                ********** ********** ********** ********** **********
                ********** ********** ********** ********** **********
                ********** **** ***** ********** *      *** **********
                ********** **** *****  ********* *
                                 *****  ********* *

alignment
position        601....... 611....... 621....... 631....... 641.......

P924542  507    GTCCCAGTCa GTCCGGAGGC TGCGGCTGCA GAAGtaccgc ctgcggagta
P924549  505    GTGCCAGTC- GTCCGGAGGC TGCGGCTGCA GACGGTCTCT CCgAGAAG--
P924553  481    GTGCCAGTC- GTCCGGAGGC TGCGGCTGCA GAAGATCTCT CCaAGAAG--
                *********  ********** ********** ********** ** *****
                *********  ********** ********** ********** ** *****
                *********  ********** ********** ********** ** *****
                *********  ********** ********** ****          *****
                *********  ********** ********** ****
                *********  ********** ********** ****
                *********  ********** ********** **
                *****

alignment
position        651....... 661....... 671....... 681....... 691.......

P924542  557    aCTGCAAAGA TGCTGTCCGT GCGCGTTGCT GCGGCCGTGG tCCGCGCCCT
P924549  552    -CTGCAAGGA TGCTGTCTGT GCGCGTCGCC GCGGCCGTGG CCCGTGCCCT
P924553  528    -CTGCAAGGA TGCTGTCCGT GCGCATCGCC GCGGCCGTGG CCCGTGCCCT
                 ********* ********** ********** ********** **********
                 ********* ********** ********** ********** **********
                 ********* ********** ********** ********** **********
                 ********* ********** ********** ********** **********
                 ********* ********** ********** ********** **********
                 ********* ********** ********** **********  *********
                 ********* ********** ********** **********  *********
                 ********* ********** ********** **********  *********
                 ********* ********** ********** **********  *********
                                                      *****  *********

alignment
position        701....... 711....... 721....... 731....... 741.......

P924542  607    TCCTCGGCGG GCCGGACTGG TGAGCACCGa aggccggcat gatgcaggcg
P924549  601    CCCTCGACGG GCGGGACTGG TGAGCACCGG GGCCTAG--- ----------
P924553  577    CCCTCGACGG GCGGGACTGG TGAGCGCCGG GGCCTAG--- ----------
                ********** ********** ********** *******
                ********** ********** ********** *******
                ********** ********** ********** *******
                ********** ********** *********
                ********** ********** *********
                ********** ********** *****  ***
                ********** ********** *****  ***
```

```
                    ********** ********** *****  ***
                    ********** ********** *****
                    ********** ********** *****

alignment position  751....... 761....... 771....... 781....... 791.......
P924542 657         gccgggtggg gctGCAGGGT GGTGGTGCGC CGG----CTC GGGcgctctc
P924549 638         ---------- ---GCAGGCT GGCGCGGGAG CGGagatCTC AGGTCTCTGC
P924553 614         ---------- ---GCAGGCT GGCGTGGGAG CGG----CTC AGGTCTCTGC
                                  ******* ********** ***    *** **********
                                  ******* ********** ***    *** **********
                                  ******* ********** ***

alignment position  801....... 811....... 821....... 831....... 841.......
P924542 703         tgcaggagg- ---------- --GCGAGGGG CTGTGGCGAA TGCCGCCATC
P924549 675         GGAGCGGAGC cggaGCGGTG CAGCGCGGGG CAGGGGCGGA CGGCGCCATC
P924553 647         GGATCGGAGC ----GCGGTG CAGTGCAGGA CTGGGGCGGA CGGCGCCATC
                    **********     ****** ********** ********** **********
                    **********              ******** ********** **********
                                                 *** ********** **********
                                                       ******** **********
                                                       ******** **********
                                                       ******** **********

alignment position  851....... 861....... 871....... 881....... 891.......
P924542 740         TTGCACCcgt ggcttctcCG GCTGGACAGA GCAGGCGACA CAGGTGCCCT
P924549 725         TTGCACCGTG G-------CT GCGGAGCAGC GCGGACGGCC GAGGGGCTTT
P924553 693         TTGCACCGTG G-------CT GCCAGGCAGC GCGGACGGCA AAGGGGCTCT
                    ********** *       ** ********** ********** **********
                    ********** *       ** **   ***** ********** **********
                    ********** *       ** **   ***** ********** **********
                    ********** *       ** **
                    *******               **
                    *******

alignment position  901....... 911....... 921....... 931....... 941.......
P924542 790         TTTGCtcgtc acctgcgcag aggcagaatg gtacagggca gacagttaac
P924549 768         GCTGCGGGCC TTCGGggcgc GCGCGGCGGG GCGGGGGTCT GGGACCTCGC
P924553 736         TTTGCAGGCC TTCGGt---- GCGCTGTGGG GCGTGGATCC GTGACCTCGC
                    ********** *****      ********** ********** **********
                    ********** *****      ********** ********** **********
                    ********** *****

alignment position  951....... 961....... 971....... 981....... 991.......
P924542 840         tcgatggtgt ccagagacag ggcctcAAGA TTCCTGTCTT CGGCTGACAC
P924549 818         CGTGGCtgag caagGGGGCT CTAGG-AGGG ATCTGGGCTT GGGGTGAGAC
P924553 782         CGTTGCgggc aaa-GGGGCT TTAGG-AAGG TTTCGATCTT GGGGTGAGGC
                    ******         ****** ***** **** ********** **********
                    ******         ****** ***** **** ********** **********
                                                      ********* **********

alignment position  1001...... 1011...... 1021...... 1031...... 1041......
P924542 890         CGGCCCTAGA agggggatct tGGGTGAAGG TCAGGGCTTG GGCGCTagct
```

```
P924549  867 CGGGCCTGGA ---------- --GGTGAAGG TCAGGGTGTG TGCCCTGCTC
P924553  830 CGGGGCTAa- ---------- -GGGTGAATG TCAGGGTTTG TGCACTGCTT
             **********             ********* ********** **********
             ****                    ******** ********** **********
             ****                    ******** ********** **********
                                     ******** ********** ******

alignment
position     1051...... 1061...... 1071...... 1081...... 1091......

P924542  940 ctccgaggcc tgttctgaat cggtgaggtt cctctctttc gaTTCTTAGC
P924549  905 GTCTGGCCTG CCcggaagct tg-------- ---------- ---TCCTGGC
P924553  868 GTCTGGCCTG CCctgtggct tg-------- ---------- --TTCCTGGC
             ********** **                                 ********
             ********** **                                  *******
             ********** **                                  *******
                                                            *******
                                                               ****

alignment
position     1101...... 1111...... 1121...... 1131...... 1141......

P924542  990 GTCGCGTCCC GCTGAAGGTC AGAaaactGC TGTCAGCtct tgttgaagct
P924549  934 GTCCGGCTCT GCTGAAGGTC AGAGGA--GC TGTCAGCcgt cttgtgccgg
P924553  898 GTCTGGCTCC GCTGAAGGTC AAAGGA--GC TGTCAGCagc cttctgc---
             ********** ********** ******  ** *******
             ********** ********** ******  ** *****
             ********** ********** ******  ** *****
             ********** ********** ******  ** *****
             ********** ********** *

alignment
position     1151...... 1161...... 1171...... 1181...... 1191......

P924542 1040 agtcagtg-- ------GAGT CTGACATTGT GTGAAGGTCA GCAGGCCc-A
P924549  982 aggaggaggA GGAGGCGAGC CTGACATTGT GTGAAGGTCA ACAGGCCGGA
P924553  943 ---------A GGAGGAGAGT CTGACATTGT ATGAAGGTCA ACAGGCCGGA
                      * ********** ********** ********** **********
                      * ********** ********** ********** **********
                      * ********** ********** ********** **********
                      * ********** ********** ********** **********
                              **** ********** ********** *******
                              **** ********** ********** *******
                              **** ********** ********** *******
                              **** ********** ********** *******

alignment
position     1201...... 1211...... 1221...... 1231...... 1241......

P924542 1081 GCATTTCACC TCTGCCTGGA GCTCAGttta ga-------- ----------
P924549 1032 GCAGTTCACC TCTGCTCCGA GGTCAATCCA AGGGAAAGGA ATAAATGAAA
P924553  984 GCATTTCACC TCTGCTCGGA GGTCGTTTCA AGGGAAAGGA ATAAATGAAA
             ********** ********** ********** ********** **********
             ********** ********** ********** ********** **********
             ********** ********** ******         ****** **********
             ********** ********** ****

alignment
position     1251...... 1261...... 1271...... 1281...... 1291......

P924542 1113 ---------- ---------- ---------- ---------- ----------
P924549 1082 GTGACTCTTG CATTGTGGGA TGGTttACCC ATTTGGAGCT Gaccc-----
P924553 1034 GTGACCCTTC AATTGTTGGA TGGTc-ACTC ACTTGGTGCT Gtatacggga
```

```
               * * * * * * * * * *  * * * * * * * * * *  * * * *     * * * *  * * * * * * * * * *  *
               * * * * * * * * * *  * * * * * * * * * *  * * * *
               * * * * * * * * * *  * * * * * * * * * *  * * * *
alignment
position       1 3 0 1 . . . . . .  1 3 1 1 . . . . .
P924542 1113   - - - - - - - - - -  - - - - - - - - - -
P924549 1127   - - - - - - - - - -  - - - - - - - - - -
P924553 1083   a a g g t c g c g a  a a a c a t c g c
```

Pairwise similarities:

For each pairwise alignment, the similarity (relative to the maximum similarity) and the number of identical nucleic acids (in % of shorter sequence) is given. Maximum values are underlined.

	P924549 (1126 bp)	P924553 (1101 bp)
P924542 (1112 bp)	0.341<br>43 %	0.337<br>43 %
P924549 (1126 bp)		1.000<br>76 %

Please note that the **similarity value 1.000** marks only the two **most similar** sequences, it does not necessarily mean that these sequences are identical.

Extract alignment region	
Positions in alignment	from: to:
Output file	dialign.seq

Extract aligned sequences

For comments, questions or bug reports, please contact support@genomatix.de

© Genomatix Software GmbH 1998-2005 - All rights reserved.

License Agreement

GEMS Launcher 4.1.1

Genomatix-Logo Logout Personal Messages GEMS-Logo
Logout Personal Messages

Genomatix_MainGEMSElDorado Gene2PromoterBiblioSphere
Frequently_asked_questionsYour ResultsYour SequencesYour_Protocol Help

GEMS Launcher Task: *DiAlign TF*: Multiple alignment plus TF sites working on promoters.seq (3 seq.)

[Alignment] [Pairwise_similarities]

DiAlign professional TF Release 3.1 December 2004 **Fri May 20 23:47:21 2005**

Solution parameters:

**Sequence file:**	promoters.seq (3 seq.)
**Type of sequences:**	nucleotide sequences
**Threshold T:**	0.00
**'*' signs below alignment denote:**	diagonal similarity (max. similarity is represented by 10 '*' signs)
**Alignment output:**	complete alignment is shown<br># nucleic acids per line: 50
**TF output:**	common TF matches located in aligned regions (common to 3 (100.0 %) sequences)
**Family matches:**	yes
**MatInspector library:**	Matrix Family Library Version 5.0 (February 2005)
**Selected groups** (core/matrix sim):	• ALL user_defined.lib (0.75/Optimized)<br>• ALL vertebrates.lib (0.75/Optimized)

Aligned Sequences:

No.	Sequence Name	Sequence Description	Length
1	P978027	sym=ATP5B|loc=Loc506|taxid=9606|spec=Homo sapiens|chr=12| ctg=NT_029419|str=(-)|start=19182563|end=19183563|len=1001| tss=501| comm=ATP synthase, H+ transporting, mitochondrial F1 complex, beta polypeptide; (NM_001686/523/bronze;)	1001 bp
2	P978029	sym=Atp5b|loc=Loc11947|taxid=10090|spec=Mus musculus|chr=10| ctg=NT_081856|str=(+)|start=5829087|end=5830098|len=1012| tss=501,502,512| comm=ATP synthase, H+ transporting mitochondrial F1 complex, beta subunit; (AK083276/541/gold;AK084009/552/gold;AK011035/541/gold; AK010314/542/gold;NM_016774/542/bronze;)	1012 bp
3	P978031	sym=Atp5b|loc=Loc171374|taxid=10116|spec=Rattus norvegicus| chr=7|ctg=NW_047773|str=(+)|start=1376032|end=1377032| len=1001|tss=501| comm=ATP synthase, H+ transporting, mitochondrial F1 complex, beta polypeptide; (XM_343136/501/bronze;)	1001 bp

Alignment (DiAlign format):

Please note that only **upper-case** letters are considered to be **aligned**. For more information, please have a look at the user guide.

V$EREF U$EREF V$E4FF V$ECAT V$HOXF V$NKXH V$HOMF V$MOKF V$DEAF V$ETSF V$DMTF V$RORA
V$RARF V$SF1F

```
alignment
position        1.........  11........  21........  31........  41........

P978027     1   cactacttcg  aga-------  ----------  ----------  ----------
P978029     1   ----------  -----CTTTC  GTTGCACGGA  TTTATATGAT  AAAGAgCCTC
P978031     1   cgtctatcga  cgtttCTTTC  GTTGCACAGA  TTTAAATGAT  AAAGAcCCTC
                                 *****  **********  **********  ***** ****
                                 *****  **********  **********  ***** ****
                                 *****  **********  **********  ***** ****

alignment
position        51........  61........  71........  81........  91........

P978027    14   ----------  ----------  ----------  ----------  ----------
P978029    36   CAGGTTTCGG  ACCTGCTGGG  CCGCCtGGAA  ACTTCCACCC  TCACTACTGT
P978031    51   CAGGTTTCCG  ACCTGCTGGG  CCGCCcGGAA  GCTTCCACCC  TCACTACTGT
                **********  **********  ***** ****  **********  **********
                **********  **********  ***** ****  **********  **********
                **********  **********  ***** ****  **********  **********

alignment
position        101.......  111.......  121.......  131.......  141.......

P978027    14   ----GAAAAA  CCTTATTTTG  --------GC  TCTAAAGAGG  GCTCCTGACT
P978029    86   CGAGGAAACC  CCACGTTTTG  --------GA  GCCACAGAAG  GTTCCTGACT
P978031   101   GGAGGAAACt  tcatgactcc  gccatgttGA  GCCACAGAAA  GCTCCTGCCT
                **********  **********          **  **********  **********
                *********                       **  **********  **********
                *********                       **  **********  **********
                    *****

alignment
position        151.......  161.......  171.......  181.......  191.......

P978027    52   Tcgccacgct  caccttcagt  ggtgcttcac  cgcactacca  cttccctaac
P978029   128   TTCAGGACGC  CGCCACTCaa  ccttaGC---  ----------  ----------
P978031   151   TTCGGGACGC  CAGGACTCca  tatctGC---  ----------  ----------
                **********  ********         **
                **********  ********         **
                **********  ********         **
                                             **

alignment
position        201.......  211.......  221.......  231.......  241.......

P978027   102   caccgcccct  atGGCTGTCA  CCTAgatcaa  ggacctatct  aaggagAAAG
P978029   155   ----------  --GGCTGTCA  CCTAACTC--  ----------  ------AAGG
P978031   178   ----------  --GGCTGTCA  CCCAACTC--  ----------  ------AAAG
                              ********  ********                    ****
                              ********  ********                    ****
                              ********  ********                    ****
                              ********  ********                    ****
                              ********  ****                          ****
                                                                    ****

alignment
position        251.......  261.......  271.......  281.......  291.......
```

```
P978027 152 CCCAAGGACA GGCAAAGACa ggccacgcac tttcaaca-- GGAACTCGGC
P978029 175 TCCAAGGACA GTCAAAGACg GCCTAGTGCA CAGGTTCAAC GGAACCCCGC
P978031 198 TCCAAGGACA GTCAAAGACc GCCTAGTGCA TAGGTTCAAC GGAACCCCGC
            ********** *********  ********** ********** **********
            ********** *********  ********** ********** **********
            ********** *********  ********** ********** **********
            ********** ********   ********** ********** **********
            ********** ********   ********** ********** **********
            ********** ********                         **********

alignment
position    301....... 311....... 321....... 331....... 341.......

P978027 200 CCCTTTCCTA -AACGTAGTT CCtctgatta ccctgtccaa cgGACGTCGC
P978029 225 CCCTTTATTT cAACGTAGTT CCGCCAAAAA CCt-CTCCTA CTGACGTTCC
P978031 248 CCCTTTATTT -AACGTAGTT CCGCCAAAAA CCcaCTCCTA CTGACGTTCC
            **********  ********* ********** **    ****** **********
            **********  ********* ********** **    ****** **********
            **********  ********* **               ****** **********
            **********  ********* **               ****** **********
            **********                                      ********
            **********                                      ********
                                                            ********
                                                            ********

alignment
position    351....... 361....... 371....... 381....... 391.......

P978027 249 TATGATCCAA TTAAGTTTTG AGGCTCAGAG tgacgccctc taacctcaca
P978029 274 CATGGGCCAA TTAGGTTTCG AGGCTTAGAG GGCGGCTCCT CCAATTA---
P978031 297 TATGGCCCAA TTAGGTTTCG AGGCTTAGAG GGCGGTTCCT TCAATTA---
            ********** ********** ********** ********** *******
            ********** ********** ********** ********** *******
            ********** ********** ********** ********** *******
            ********** ********** **********
            ********** ********** **********
            ********** ********** **********
            ********** ********** ****
            ********** ********** ****

alignment
position    401....... 411....... 421....... 431....... 441.......

P978027 299 ctgtccaatA AAAAAAGGAG TTTCAGATTA GCACCAGTTT TGACCAATGG
P978029 321 ---------A AAAAGAGAAT TTTCAGATTG GCACCAGTTT AGACCAATAG
P978031 344 ---------A AAAAAGGTAT CTTCAGATTG GCACCAGTTT TGACTAATAG
                     * ********** ********** ********** **********
                     * ********** ********** ********** **********
                     * ********** ********** ********** **********
                     * ********** ********** ********** **********
                     * ********** ********** ********** **********
                                  ********** ********** **********
                                  ********** ********** ********
                                        **** ********** ********
                                        **** ********** ********

alignment
position    451....... 461....... 471....... 481....... 491.......

P978027 349 ATGAGAAGat cagcgtg-TA TTAACCTGTA GAAGCAGCAc aGAGAATGAT
P978029 362 CTGATAAGCT CCGAGTttTT TTACCCTATA GAAGCGTTAG TGGTGATGAC
P978031 385 CTGATGAGCT CCGAGT--TT TTACCCTATA GGAGCGTCAG TGAAGATAAC
```

```
               ********** ******  ** ********** ********** **********
               ********** ******  ** ********** ********** **********
               ********** ******  ** ********** ********** **********
               ********** ******
               ********
               ********

alignment
position       501....... 511....... 521....... 531....... 541.......

P978027    398 GAGCAGCCAG TTCACCCAAT GGACCTGCCT ACTGCAGCGT AGGCCTCGCC
P978029    412 GAACAGCAAA ATCACCCAAT TACTGTGCCT ACGGCGGAGG TTGCCCCGCC
P978031    433 TAACATCAAG ATCATCCAAT GACTGTGCCT ATGGCGGAAG CTGCCCCGCC
               ********** ********** ********** ********** **********
               ********   ********** ********** ********** **********
               ********   ********** ********** ********** **********
                          ********** ********** ********** **********

alignment
position       551....... 561....... 571....... 581....... 591.......

P978027    448 TCAACGGCAG GAgagcaggc ggctgcggtt GCTGCAGCCT TCAGTCTCCA
P978029    462 CCAGCTGCAG GACCGGCGGA GAGGACC--- GCTTCGGCGC TCAGTCTCCA
P978031    483 TCGGCTGCAG GGCTGGCGCC CCGGACC--- GCTGGAGCGC TCAGTCTCCA
               ********** ********** *******    ********** **********
                ********* ********** *******    ********** **********
                ********* *                     ********** **********
                                                ********** **********
                                                ********** **********
                                                           **********
                                                           **********
                                                               ******
                                                               ******
                                                               ******

alignment
position       601....... 611....... 621....... 631....... 641.......

P978027    498 CCCGGACTAC GCCATGTTGG GGTTTGTGGG TCGGGTGGCC GCTGCTCCGG
P978029    509 CCCGGATTCC GCCATGTTGA GTCTTGTGGG GCGTGTGGCC TCGGCCTCGG
P978031    530 CCCGGACTCC GCCATGTTGA GTCTTGTGGG GCGTGTGGCC TCGGCCTCGG
               ********** ********** ********** ********** **********
               ********** ********** ********** ********** **********
               ********** ********** ********** ********** **********
               ********** ********** ********** ********** **********
               ********** ********** ********** ********** **********
               ********** ********** ********** ********** **********
               ********** ********** ********** ********** **********
               ********** ********** **********                   ***
               ********** *********      *******                  ***
               ********** *********      *******                  ***

alignment
position       651....... 661....... 671....... 681....... 691.......

P978027    548 CCTCCGGGGC CTTGCGGAGA CTCACCCCTT CAGCGTCGCT GCCCCCAGCT
P978029    559 CCTCCGGGGC CTTGCGGGGA CTCAGCCCTT CGGCGGCTCT GCCACAGGCG
P978031    580 CCTCCGGGGC CTTACGGGGA CTCAACCCTT TGGCGGCGCT GCCACAGGCG
               ********** ********** ********** ********** **********
               ********** ********** ********** ********** **********
               ********** ********** ********** ********** **********
               ********** ********** ********** ********** **********
```

```
            ********** ********** ********** ********** **********
            ********** ********** **** ***** ***** **** **********
            ********** ********** **** ***** *****
            ********** ********** **** ***** *****
            ********** *** ****** **** ***** *****
            ********** ***

alignment
position    701....... 711....... 721....... 731....... 741.......

P978027 598 CAGCTCTTAC TGCGGGCCGC TCCGACGGCG GTCCATCCTG GTAAGTGCTT
P978029 609 CAGCTTCTAC TGCGAGCTGC TCCCGCCGGG GTTCATCCTG GTAAGCGCCT
P978031 630 CAGCTTCTAC TACGAACCGC TCCCGCCGGG GTCCATCCTG GTAAGCGCCT
            ********** ********** ********** ********** **********
            ********** ********** ********** ********** **********
            ********** ********** ********** ********** **********
            ********** *****   ** ********** ********** **********
            ********** *****   ** ********** ********** **********
            ****   *** *****   ** ********** ********** **********
                   *** *****   ** ********** ********** **********
                               ** ***      * ********** *****

alignment
position    751....... 761....... 771....... 781....... 791.......

P978027 648 TTCTCTAGGA GCTAacgttc catttgcCG CCCCATGACC TTGAGCCGGG
P978029 659 TCCTGGAGGA GCTAgttcgg ttcTT---CT CCCCGTGACC TTGAGCTTGG
P978031 680 TCCTGGAGga ggggattcgg tt-TT---CT CCCCGTGACC TTGAGCCTGG
            ********** ****          **   ** ********** **********
            ********** ****          **   ** ********** **********
            ********                 **   ** ********** **********
            ********                      ** ********** **********
            ********                         ********** ******
            ********
            ********

alignment
position    801....... 811....... 821....... 831....... 841.......

P978027 698 Gaacgatggt agctcgggCC TAAGGGATCC GTggtgttaa aaggatgCCT
P978029 706 G--------- --------CC TATCGGATCC GTtCTCCATA AGGAT--TCC
P978031 726 G--------- --------CC TAGCAGATCT GTgCTCCATT AGGAT--CCT
            *                  ** ********** ** ******* *****  ***
            *                  ** ********** ** ******* *****  ***
            *                  ** ********** ** ******* *****  ***
            *

alignment
position    851....... 861....... 871....... 881....... 891.......

P978027 748 GGAGCCGCGT CTTGCTCTCT AATGGCTGga gaacaagaat gggacaccca
P978029 737 CGGGCAGCGG ACTACTGTCC AGCCGagGAG TGCAAGGACA GTCGCCCGTG
P978031 757 GGCGCAGCGA ACTACTGTCC AGCCGCAGAG TGCAAGGACA GTCGCCGGTG
            ********** ********** ********** ********** **********
            ********** ********** *****  *** ********** **********
            ********** ********** *****  *** ********** **********
                                         *** ********** **********

alignment
position    901....... 911....... 921....... 931....... 941.......

P978027 798 taGGAGGTTT CTTCGACCCA GCTCTGTCCC ATTTTGTata aagtcctttg
P978029 787 --GGAGCGTT GATATAACCA GCAGCGTCCC ATTTTGTG-- ----------
```

```
P978031 807 --GGAGCGTT GATCGAACCA GCTGTGTCCC ATTTTGTT-- ----------
              ******** ********** ********** ********
              ******** ********** ********** ********
              ******** ********** ********** *******
              ******** ***  ***** ********** *******
              ******** ***
              ******** ***

alignment
position    951.......  961.......  971.......  981.......  991.......

P978027 848 tgtgACGGAA AAGGTGCGGG CAGGTGCGTT GACCTTCCGG TTga-ATAGG
P978029 823 ----AGGCTA GAGTGGCCGG CAGCTGCCTC GACCTTGAGG TTTTGAGGGG
P978031 843 ----ACGTTA AAGTGGAGGG CAGCTGCCTT GACCTTGAGG TTCCTATGGG
                ****** ********** ********** ********** **********
                ****** ********** ********** ********** **********
                ****** ******  ** ********** ********** **********
                            *  ** ********** ********** **      **

alignment
position    1001......  1011......  1021......  1031......  1041......

P978027 897 GGTACTGAAC TCTCGAAAAA TGG-GGTCTT GTGCATGCGG ATGGGAAGGC
P978029 869 GATACCAAAC TCGAGAATAA TGGCGGTTCT GTGCACGAGG TCGGGAAAAC
P978031 889 GGTACTAAAC TCAAGAATAA TGGCGGTTTT GTGCACGAGG TTGGGAAAGC
            ********** ********** ********** ********** **********
            ********** ********** ********** ********** **********
            ********** ********** ********** ********** ******** *
            ********** ********** ********** ********** ******** *
                    ** ********** *** ****** ********** ******** *
                                      ****** ********** ******** *

alignment
position    1051......  1061......  1071......  1081......  1091......

P978027 946 TGTGCAGGCG CTTTATCGAT CGCTGCGCGG CCTTCCCtct tggacacgcg
P978029 919 AGTGCGGGTG GGTTCCCGAT TGCAGCGTGC CCTTCTCCAt gcctcctCCT
P978031 939 AGCGCGGGCG GGTTCCCGAT TGCAGCGTGG CCTTCTCCAc acggctcCCT
            ********** ********** ********** *********         ***
            ********** ********** ********** *********
            ********** ********** ********** *********
            ********** ********** ********** *********
            ********** ****
            **********

alignment
position    1101......  1111......  1121......  1131......  1141

P978027 996 ttccgt---- ---------- ---------- ---------- ----
P978029 969 ATCTTGCAGG AGActtagtc tgggctcagc taaccttccc tcgg
P978031 989 ATTTTGCAGG AGA------- ---------- ---------- ----
            ********** ***
```

Pairwise similarities:

For each pairwise alignment, the similarity (relative to the maximum similarity) and the number of identical nucleic acids (in % of shorter sequence) is given. Maximum values are underlined.

	P978029 (1012 bp)	P978031 (1001 bp)

**P978027 (1001 bp)**	0.291 49 %	0.309 50 %
**P978029 (1012 bp)**		1.000 83 %

Please note that the **similarity value 1.000** marks only the two **most similar** sequences, it does not necessarily mean that these sequences are identical.

**Extract alignment region**	
**Positions in alignment**	from: to:
**Output file**	dialign.seq

Extract aligned sequences

For comments, questions or bug reports, please contact support@genomatix.de

© Genomatix Software GmbH 1998-2005 - All rights reserved.

License Agreement

GEMS Launcher 4.1.1

Genomatix-Logo Logout Personal Messages GEMS-Logo
Logout Personal Messages

Genomatix_MainGEMSElDoradoGene2Promoter BiblioSphere
Frequently_asked_questions Your ResultsYour SequencesYour ProtocolHelp

GEMS Launcher Task: *DiAlign TF*: Multiple alignment plus TF sites working on promoters.seq (3 seq.)

[Alignment] [Pairwise_similarities]

DiAlign professional TF Release 3.1 December 2004 **Sat May 21 01:24:15 2005**

Solution parameters:

**Sequence file:**	promoters.seq (3 seq.)
**Type of sequences:**	nucleotide sequences
**Threshold T:**	0.00
**'*' signs below alignment denote:**	diagonal similarity (max. similarity is represented by 10 '*' signs)
**Alignment output:**	complete alignment is shown<br># nucleic acids per line: 50
**TF output:**	common TF matches located in aligned regions (common to 3 (100.0 %) sequences)
**Family matches:**	yes
**MatInspector library:**	Matrix Family Library Version 5.0 (February 2005)
**Selected groups** (core/matrix sim):	• ALL user_defined.lib (0.75/Optimized)<br>• ALL vertebrates.lib (0.75/Optimized)

Aligned Sequences:

No.	Sequence Name	Sequence Description	Length
1	P978102	sym=ATP5D|loc=Loc513|taxid=9606|spec=Homo sapiens|chr=19|ctg=NT_011255|str=(+)|start=1181249|end=1182249|len=1001| tss=501| comm=ATP synthase, H+ transporting, mitochondrial F1 complex, delta subunit; (NM_001687/530/silver;)	1001 bp
2	P978103	sym=Atp5d|loc=Loc66043|taxid=10090|spec=Mus musculus|chr=10|ctg=NT_039496|str=(+)|start=5994807|end=5995837|len=1031| tss=501,507,527,531| comm=ATP synthase, H+ transporting, mitochondrial F1 complex, delta subunit; (AK018011/508/gold;AK011374/532/gold;AK076064/528/gold; NM_025313/502/bronze;)	1031 bp
3	P978104	sym=Atp5d|loc=Loc245965|taxid=10116|spec=Rattus norvegicus|chr=7|ctg=NW_047773|str=(-)|start=11075406|end=11076406| len=1001|tss=501| comm=ATP synthase, H+ transporting, mitochondrial F1 complex, delta subunit; (NM_139106/563/silver;)	1001 bp

Alignment (DiAlign format):

Please note that only **upper-case** letters are considered to be **aligned**. For more information, please have a look at the user guide.

V$STAF V$NRF1 U$NRF1 V$WHZF V$AP4R V$NEUR V$RP58 V$EGRF

```
alignment
position     1......... 11........ 21........ 31........ 41........
P978102    1 gggtcaagtg attct---CC TGCCTTGGCC TCTCAAGgTG CTGGGATTAC
P978103    1 ctcaaactca gaaagccgCC TGCCTCTGCC TCCCAAG-TG CTGGGAATAA
P978104    1 tttacctatt tccagaatgc ---------- ---------- ----------
                                 ** ********** ******* ** **********

alignment
position     51........ 61........ 71........ 81........ 91........
P978102   48 AGGTTGGAGC CACCAcacct ggcccagagc cggaaacttg atgttttctt
P978103   50 AGGCACGCGC CACCActgcc catctatatt tgatgcactt ttggaaatag
P978104   21 ---------- ---------- ---------- ---------- ----------
             ********** *****

alignment
position     101....... 111....... 121....... 131....... 141.......
P978102   98 gatgtctatc cagggccctg catccaatga gcgccccaaa aatctttttt
P978103  100 gttgttccg- ---------- ---------- ---------- ----------
P978104   21 ---------- ---------- ---------- ---------- ----------

alignment
position     151....... 161....... 171....... 181....... 191.......
P978102  148 tttttttttg tacagacggg tcgggggtgg gggcgatcta cactattgcc
P978103  109 ---------- ---------- ---------- ---------- ----------
P978104   21 ---------- ---------- ---------- ---------- ----------

alignment
position     201....... 211....... 221....... 231....... 241.......
P978102  198 caggctggtt tccagctcct ggcctcaagt gatcctctgg cctcagcctc
P978103  109 ---------- ---------- ---------- ---------- ----------
P978104   21 ---------- ---------- ---------- ---------- ----------

alignment
position     251....... 261....... 271....... 281....... 291.......
P978102  248 ctaaAGCGCT GGTATTACAG TCGTGAGCCA CCACGCagcc gaatcttgaa
P978103  109 ----AGTGCG GGGATTGCAA GAGTGTGTCA CCGCAC---- -------TCT
P978104   21 ---------- -GGATTGCAA AAGTGCGTCA CCACACctga ctggtaaTCT
                 ****** ********** ********** ******            ***
                         ********* ********** ******            ***

alignment
position     301....... 311....... 321....... 331....... 341.......
P978102  298 tttcactgaa ---------- ---------- ---------- ----------
P978103  144 ACATTTTAAT TCTTCTGTtC CAACACGTTA CAGGTTTGCT TCTTCTGGGC
P978104   60 ACATTTTAAT TCTTCTGTcC CAACACGTTA CAGGTTTGCT TCTTTTGGGC
             ********** ********  * ********** ********** **********
             ********** ********  * ********** ********** **********
                                  * ********** ********** **********
                                  * ********** ********** **********

alignment
position     351....... 361....... 371....... 381....... 391.......
```

```
P978102 308 ---------- ---------- ---------- ---------- ----------
P978103 194 CTcgaatgc- ---------- ---------- --TTGGCACC TGATATCAAG
P978104 110 CTgaatgcgc gctaagtgcc tctctccaat ggTTGGACCC TGACATCAAA
            **                                  ******** **********
            **                                  ******** **********
            **
            **

alignment
position    401....... 411....... 421....... 431....... 441.......

P978102 308 ---------- ---------- ---------- ---------- ---GTTCGTT
P978103 221 CCCATATCAT TTCCGCGCAA ACattcccCT CCTGAGGGAA TATGTTCGTT
P978104 160 CCGGCATCTT TTCAGCTCAA ACgtact-CT CCGGAGGGAA GGTGTCCGTT
            ********** ********** **      ** ********** **********
            ********** ********** **      ** ********** **********

alignment
position    451....... 461....... 471....... 481....... 491.......

P978102 315 Ttactccacc tcccaggacg gtttcaccgc ccttatctga ccaccccctg
P978103 271 TGCAGAAaga accgcaTCCC AAGAACCCAG AGt--CGTTT GCTGTTAGAA
P978104 209 TGCAGAAcac gg----TCCT ATGGACCCAG GGcacCGTTT GCTGTTAGAA
            *******          **** ********** **   ***** **********
            *******                                ***** **********
                                                   ***** **********
                                                   ***** **********
                                                   ***** **********

alignment
position    501....... 511....... 521....... 531....... 541.......

P978102 365 gttctgccgc agcccgggcc gGAACAAAAC TGCAACCCCC AGAATACCTC
P978103 319 GGC------- ---------- -GAAGAAAAC TACAATTCCC AGAATGACCC
P978104 255 GGC------- ---------- -GAAGAAAAC TACAGTTCCC AGAATGATTC
            ***                    ********* ********** **********
            ***                    ********* ********** **********
            ***                    ********* ********** **********
            ***                    ********* ********** **********
            ***                    ********* ********** ***
                                   ********* ********** ***

alignment
position    551....... 561....... 571....... 581....... 591.......

P978102 415 ACGACGgggc actgcgtcgt caacgtcgcg tgcgc----- ----------
P978103 351 ACCCGGCATA GGAGGATCCC ACTTCTAGGC AtCACTGCAA GGGGTGGGGC
P978104 287 ACGAGGCATA GGAGGGTCCC ACTTCTAGGC AgCACTGCAA GGGGTGTGGC
            ********** ********** ********** *  ******** **********
            ********** ********** ********** *  ******** **********
            ********** ********** ********** *  ******** **********
            ******                             ******** **********

alignment
position    601....... 611....... 621....... 631....... 641.......

P978102 450 ---------- ---------- ---------- ---------- ----------
P978103 401 CAAGGGAAAG TATACCTCCC AGaATGCTCA GCGCGTACTG CATCGCGCAG
P978104 337 TAAGGCAAAG TATACCTCCC AGtATGCTCA GCGCGTTCTG CACCGCGCAG
            ********** ********** ** ******* ********** **********
            ********** ********** ** ******* ********** **********
            ********** ********** ** ******* ********** **********
            ********** ********** ** ******* ********** **********
```

```
alignment
position          651....... 661....... 671....... 681....... 691.......

P978102   450     CCTGCCGGGA GTCGTAGTTC GCGCTTGCGC TCTCGCCGGg ACTCCTCCTC
P978103   451     CCTTCGGAGA ATCctgt--- GCGCGTGCGT TCTTGTGGGA ACTGCGCCTC
P978104   387     CCTTCGGAGC ATCGCAGTGC GCGCGTGCGT TCTTGTGGGA ACTGCGCCTC
                  ********** ********** ********** ********** **********
                  ********** ***        ********** ********** **********
                  ********** ***        ********** ********** **********
                  ********** ***        ********** ********** **********
                  ********** ***        ********** ********** **********
                                        ********** *****      **********
                                                              **********
                                                                    ****
                                                                    ****

alignment
position          701....... 711....... 721....... 731....... 741.......

P978102   500     CCAGACGTCC CTGCGCGTCG TCCTCCTCGC CCTCCa---- ----------
P978103   498     CCAGAAGGCA CCGCGCGTCG TCCTTCTC-- CCTCCCTGAA GGCCGCCTCG
P978104   437     CCAGAAGACA CCGCGCGTCG TCCTCCTCGC CCTCCCTGAA GGCCGCCTCG
                  ********** ********** ********** ********** **********
                  ********** ********** ********** ********** **********
                  ********** ********** ********** *****
                  ********** ********** ********   *****
                  ********** ********** ********   *****
                  *******  ** ********** *******
                  *******
                  *******
                  *******

alignment
position          751....... 761....... 771....... 781....... 791.......

P978102   536     ---------- -----GGCCG CCCGCGccgc GCCGGAGTCC GCTGTCcgcc
P978103   546     CTtgCCCAGT GTGTCGGCCG CCCGCGAA-- GCTAGAGTCC ACTGACtTTT
P978104   487     CTatCCCTGT GTGTCTGCTG TCCGCTAA-- GCTAAAGTCC ACTGACgTTT
                  **  ****** ********** ********   ********** ****** ***
                  **  ****** ********** ********   ********** ****** ***
                      ****** ********** ********   ********** ****** ***
                                                                     ***

alignment
position          801....... 811....... 821....... 831....... 841.......

P978102   571     agctacccgc ttcctgccgc ccgCCGCTGC CATGCTGCCC GCCGCGCTGC
P978103   594     ---------- ---------- ---CCGCCAC CATGTTGCCC GCCTCACTGC
P978104   535     ---------- ---------- ---CCGCCAC CATGCTGCCC GCCGCATTGC
                                           ******* ********** **********
                                           ******* ********** **********
                                           ******* ********** **********
                                           ******* ********** **********
                                           ******* ********** **********
                                           ******* ********** **********
                                           ******* ********** **********

alignment
position          851....... 861....... 871....... 881....... 891.......

P978102   621     TCCGCCGCCC GGGaCTTGGC CGCCTCGTCC GCCACGCCCG TGCCTATGCC
P978103   621     TTCGTCACCC GGGCCTGCGC CGCCTGATGC TTCAGGCGCG TACATACGCC
```

```
P978104 562 TTCGTCACCC AGGTCTGCGC CGTCTGGTGC TCCAGGCGCG TACGTACGCC
            ********** ********** ********** ********** **********
            ********** ********** ********** ********** **********
            ********** ********** ********** ********** **********
            ********** ***     ** ********** ********** **********
            **********  **                                    ***
            **********  **
            **********

alignment
position    901....... 911....... 921....... 931....... 941.......

P978102 671 GAGGCCGCCG CCGCCCCGGC TGCCGCCTCT GGCCCCaacC AGATGTCCTT
P978103 671 GAGGCCGCCG CTGCACCTGC CCCCGCCGCC GGGCCCGGAC AGATGTCCTT
P978104 612 cAGGCCGCCG CCTCACCTGC CCCCGCCGCT GGGCCtGGAC AGATGTCCTT
            ********** ********** ********** ********** **********
            ********** ********** ********** ********** **********
             ********* ********** ********** ***** **** **********
             ********* ********** ********** ***** **** **********
             ********* ********** ********** ***** **** **********
             ********* ********** ********** *****    * **********
                                                      * **********
                                                      * **********
                                                      * **********
                                                      * **********

alignment
position    951....... 961....... 971....... 981....... 991.......

P978102 721 CACCTTCGCC TCTCCCACGC AGGTTCGggc gctgcgggtc gggaccctcc
P978103 721 CACCTTTGCC TCCCCGACGC AGGTACGaAC TCACA----- ----------
P978104 662 CACCTTCGCC TCCCCGACGC AGGTACG-AC TCACA----- ----------
            ********** ********** ******* ** *****
            ********** ********** ******* ** *****
            ********** ********** ******* ** *****
            ********** ********** *******
            ********** ********** *******
            ********** ********** *******
            ********** ********** ******
            ********** ********** ******
            ********** ********** ******
            ********** ********** ******

alignment
position    1001...... 1011...... 1021...... 1031...... 1041......

P978102 771 gtggccgccG CCCCCGGAGT Ccagggtccc cacc------ ----------
P978103 756 ---------T CCCCCAGACT CGTCAAGCCC CTGAtctgAC CCGTGGAAGA
P978104 696 ---------G CCCCCAGAGT CGTCAAGCCC CTGActttAC CCGTGGGAGA
                     * ********** ********** ****    ** **********
                     * ********** ********** ****    ** **********
                     * ********** ********** ****

alignment
position    1051...... 1061...... 1071...... 1081...... 1091......

P978102 805 ---------- ---------- ---------- ---------- ----------
P978103 797 GGTTCTCTTG TATTAGGTCa tttGTTTCAT CCCCATGGGG CCCTGTGAAC
P978104 737 GGGTCACTTG TATTGGGTCt cagGTTTCAC CCCCATGGGG CCGCGTGAAC
            ********** *********     ******* ********** **********
            ********** *********     ******* ********** **********
```

```
alignment position  1101...... 1111...... 1121...... 1131...... 1141......
P978102  805        ---------- ---------- ---------- ---------- ---CCCAGGG
P978103  847        CTCCCCCCac cccagacctc aCCCCTGGAT GGGGTCACCT GGACCCAGGG
P978104  787        CCCCTCCCca ggcctcg--- -CCCCTGGAT GGGGTCACCC GGACCCAGGG
                    ********              ********* ********** **********
                    ********              ********* ********** **********
                                          ********* ********** **********
                                          ********* ********** **********
                                                                  *******

alignment position  1151...... 1161...... 1171...... 1181...... 1191......
P978102  812        CGCGACCccc gcttccgggc ccccggaccc gcgcgccccg gaagccgagg
P978103  897        CTCGACCTCa --GTGTTCAC TGAAACTTCA TgctCCACCC CTAAGG----
P978104  833        CTCGACCTCt ctGTGTTCAC TGAAACTTCA TaccCCACCC CTAAGG----
                    **********   ******** ********** *   ****** ******
                    **********   ******** ********** *   ****** ******
                    **********                           ****** ******
                    **********                           ****** ******
                    *******

alignment position  1201...... 1211...... 1221...... 1231...... 1241......
P978102  862        ctactcagcc ctggaccctc ggccctgcct ctgctgcccg agccctgcgc
P978103  941        ---------- ---------- ---------- ---------- ----------
P978104  879        ---------- ---------- ---------- ---------- ----------

alignment position  1251...... 1261...... 1271...... 1281...... 1291......
P978102  912        tgccctcgtc ccgggcacct ccccgagata aGCGTCTCCT GGgtgccctc
P978103  941        ---------- ---------- ---------- -GCGTCACCT GGACATAAGA
P978104  879        ---------- ---------- ---------- -GCGTCACCT GGACCTAAGG
                                                      ********* **********
                                                      ********* **********
                                                      ********* **********
                                                      ********* **********

alignment position  1301...... 1311...... 1321...... 1331...... 1341......
P978102  962        cccgatctcc ctggccaaag cctgggtgtc gccggatcgt ----------
P978103  960        CTCCACCCTC TGGGAGGTGG ATCACCTATA TCTGGAACCC AGATTTTgcc
P978104  898        CTCCACCCTC TGGGGGTTGG ATCACCTATA CCTGGAAACC AGATTTTact
                    ********** ********** ********** ********** *******
                    ********** ********** ********** ********** *******
                    ********** ********** ********** ********** *******
                    *********

alignment position  1351...... 1361...... 1371...... 1381...... 1391......
P978102  1002       ---------- ---------- ---------- ---------- ----------
P978103  1010       tt-------- ---------- --CACAAACC CTTCTGTTTT gg--------
P978104  948        aatgtactgg tgtcacctgg acCACAAGTC CTGCTGTTTT tggggtgtcc
                                            ******** **********

alignment position  1401
```

P978102 1002 - - - -
P978103 1032 - - - -
P978104 998 t a g g

Pairwise similarities:

For each pairwise alignment, the similarity (relative to the maximum similarity) and the number of identical nucleic acids (in % of shorter sequence) is given. Maximum values are underlined.

	P978103 (1031 bp)	P978104 (1001 bp)
**P978102 (1001 bp)**	0.162<br>30 %	0.185<br>33 %
**P978103 (1031 bp)**		1.000<br>75 %

Please note that the **similarity value 1.000** marks only the two **most similar** sequences, it does not necessarily mean that these sequences are identical.

Extract alignment region	
**Positions in alignment**	from: to:
**Output file**	dialign.seq

Extract aligned sequences

For comments, questions or bug reports, please contact support@genomatix.de

© Genomatix Software GmbH 1998-2005 - All rights reserved. License Agreement GEMS Launcher 4.1.1

	Logout	Personal	Messages	
Genomatix-Logo	Logout	Personal	Messages	GEMS-Logo

Genomatix_MainGEMSElDoradoGene2Promoter BiblioSphere
Frequently_asked_questions Your ResultsYour SequencesYour ProtocolHelp

GEMS Launcher Task: *DiAlign TF*: Multiple alignment plus TF sites working on promoters.seq (3 seq.)

[Alignment] [Pairwise similarities]

DiAlign professional TF Release 3.1 December 2004 **Sat May 21 00:40:19 2005**

Solution parameters:

**Sequence file:**	promoters.seq (3 seq.)
**Type of sequences:**	nucleotide sequences
**Threshold T:**	0.00
**'*' signs below alignment denote:**	diagonal similarity (max. similarity is represented by 10 '*' signs)
**Alignment output:**	complete alignment is shown<br># nucleic acids per line: 50
**TF output:**	common TF matches located in aligned regions (common to 3 (100.0 %) sequences)
**Family matches:**	yes
**MatInspector library:**	Matrix Family Library Version 5.0 (February 2005)
**Selected groups** (core/matrix sim):	• ALL user_defined.lib (0.75/Optimized)<br>• ALL vertebrates.lib (0.75/Optimized)

Aligned Sequences:

No.	Sequence Name	Sequence Description	Length
1	P925014	sym=ATP5C1|loc=Loc509|taxid=9606|spec=Homo sapiens|chr=10|ctg=NT_077569|str=(+)|start=2192536|end=2193536|len=1001| tss=501| comm=ATP synthase, H+ transporting, mitochondrial F1 complex, gamma polypeptide 1; (NM_005174/589/silver;)	1001 bp
2	P925017	sym=Atp5c1|loc=Loc11949|taxid=10090|spec=Mus musculus|chr=2|ctg=NT_039202|str=(-)|start=7031016|end=7032041|len=1026| tss=501,508,526| comm=ATP synthase, H+ transporting, mitochondrial F1 complex, gamma polypeptide 1; (AK007063/547/gold;AK088164/572/gold;NM_020615/554/silver;)	1026 bp
3	P925020	sym=Atp5c1|loc=Loc116550|taxid=10116|spec=Rattus norvegicus|chr=17|ctg=NW_047492|str=(+)|start=33088545|end=33089545| len=1001|tss=501| comm=ATP synthase, H+ transporting, mitochondrial F1 complex, gamma polypeptide 1; (XM_341555/501/bronze;)	1001 bp

Alignment (DiAlign format):

Please note that only **upper-case** letters are considered to be **aligned**. For more information, please have a look at the user guide.

V$NKXH V$RP58 V$AP4R V$SF1F V$ETSF V$SP1F V$MAZF V$NRF1 U$NRF1 V$RORA V$GREF V$HAND

```
alignment
position         1.........  11........  21........  31........  41........

P925014     1    gactcgccca  ctg-------  ----------  ----------  ----------
P925017     1    gaccgaccgA  CCCCACCAGG  GAAGTCTAAA  TGTAAAACCT  CAGTGCTc--
P925020     1    c--------A  CCCCACCAGG  GAAGTCTAAA  TGTAAAACGT  CAATGCTgta
                          *  **********  **********  **********  *******
                          *  **********  **********  **********  *******
                          *  **********  **********  **********  *******
                          *  **********  **********  **********  *******

alignment
position         51........  61........  71........  81........  91........

P925014    14    ----------  ----------  ----------  ----------  ----------
P925017    49    ---AGCCTAG  CGATTGTAAG  CGatcagtga  GTGCTTGTCT  GCGACCACTT
P925020    43    cggAGCCTAG  CAAGTGGACG  TGtcagtgc-  GTGCTTGTCT  GGGATCACTT
                    *******  **********  **          **********  **********
                                                     **********  **********
                                                     **********  **********
                                                     **********  **********

alignment
position         101.......  111.......  121.......  131.......  141.......

P925014    14    ----------  ----------  ----------  ----------  CAGACCTCAC
P925017    96    CTCGTGGAGC  TGGAGGGCtt  c---------  ----------  ----------
P925020    92    CTCGGGGAGC  TGGAGGGCgg  cgctaagggc  tgtgaggaaa  CAGACCTCAC
                 **********  ********                            **********
                 **********  ********
                 **********  ********
                 **********  ********

alignment
position         151.......  161.......  171.......  181.......  191.......

P925014    24    AAGCCtcacg  acccggaccc  cgggagcccc  agccccgcgc  cctcagcccc
P925017   117    GCGCCGAGCT  G---------  ----------  ----------  ----------
P925020   142    GCGCCGAGCT  G---------  ----------  ----------  ----------
                 **********  *
                 **********  *
                 **********  *

alignment
position         201.......  211.......  221.......  231.......  241.......

P925014    74    gcgtccagga  cctgatcctg  gattgccagg  ggccctcctG  GGAAGACGGC
P925017   128    ----------  ----------  ----------  ---------G  GGAAGACTGC
P925020   153    ----------  ----------  ----------  ---------A  GGAAGACTGA
                                                              *  **********
                                                              *  **********
                                                              *  **********

alignment
position         251.......  261.......  271.......  281.......  291.......

P925014   124    GCCactccgg  gcc-------  ----------  ----------  ----------
P925017   139    GCCGGAGAGG  GCTCcaAGCC  GAGGGa----  ----------  ----------
P925020   164    GCCGGAGAGC  GCTCagAGCC  GAGGGtacca  gggtcgccgt  caggccccct c
```

```
            ********** ****  **** *****
            ********** ****
            ********** ****

alignment
position    301....... 311....... 321....... 331....... 341.......
P925014 137 ---------- ---------- -CACTCACCT CGTCCCGGCA CTGCTTCTG[illegible]
P925017 165 GGGGTCGCGG CCAGCCTGAG TCACTTACCT CGTCGCGGCA TTGCTTTTG[illegible]
P925020 214 GGGGTCGCGG CCACCCTGAG CCACTTACCT CGTCGCGGCA TTGTTTTTG[illegible]
            ********** ********** ********** ********** **********
            ********** ********** ********** ********** **********
            ********** ********** ********** ********** **********
            ********** ********** ********** ********** **********
                                   ********* ********** **********
                                   ********* ********** **********
                                   ********* ********** **********
                                   ********* ********** **********
                                                      * ******  ***
                                                                ***

alignment
position    351....... 361....... 371....... 381....... 391.......
P925014 166 CACATCTGGC AATACCAGCG TAGCTTCTGC AGCCCCTTGG ACTTGATCCT
P925017 215 CACATCTGGC AGTACCAGCG AAGCTTCTGg AGCCCTTTGG ACTTAATTCT
P925020 264 CACATCTGGC AGTACCAGCG AAGCTTCTGC AGCCCTTTAG ACTTAATTCT
            ********** ********** ********** ********** **********
            ********** ********** ********** ********** **********
            ********** ********** ********** ********** **********
            ********** ********** *********  ********** **********
            ********** ********** *********  ********** **********
            ********** ********** *********  ********** **********
            ********** ********** *********  ********** **********
            ********** ********** *********  ********** **********
            ********** * ******** *********
            ********** * ******** *********

alignment
position    401....... 411....... 421....... 431....... 441.......
P925014 216 GTTGGCGATA GCCTTGGGAG TAAGAAAATC CGACTTCCCC ATGGCGACCa
P925017 265 ATTGGCGATG GCCTTGGGGC TCAGAAAATC CGACTTGCCC ATGGCGACCC
P925020 314 ATTCGCGATG GCCTTGGGGC TCAGAAAATC TGACTTGCCC ATGGCGACCC
            ********** ********** ********** ********** **********
            ********** ********** ********** ********** **********
            ********** ********** ********** ********** **********
            ********** ********** ********** ********** **********
            ********** ********** ********** ********** **********
            ********** ********** ********** ********** *********
                       ********** ********** ********** *********
                       ********     ******** ********** *********
                           ****     ******** ********** *********
                           ****     ******** ********** *********

alignment
position    451....... 461....... 471....... 481....... 491.......
P925014 266 cggcagcgat cactttctgg accccagtac tcagcCAGCC GGAAACGGAA
P925017 315 TGAGcgcTAC AACTACAAAG ---------- -----CGGTC GGAAGCGGAA
P925020 364 TGAGag-TAC AACAGCGAAG ---------- -----CCGTC GGAAGCGGAA
```

```
                 ****   *** **********                       ***** **********
                 ****   *** **********                       ***** **********
                 ****                                        ***** **********
                 ****
                 ****

alignment
position         501....... 511....... 521....... 531....... 541.......

P925014  316     CCGGGCTGGC Ggcggtg--G GCGGGACAAA tcccagCTGA GAGGCGGAAC
P925017  350     CTGGctcaGC GCGGGGACAG GCGGGACCGA AA----TTGA CAGCAGGAAa
P925020  398     CTGGACCCGC GCGCGGATAG GCGGGACCAA AA----CTGA CAGCAGGAAC
                 ********** ********** ********** **    **** **********
                 ****    ** ********** ********** **    **** *********
                 ****    ** ********** ********** **    **** *********
                                                        **** *********

alignment
position         551....... 561....... 571....... 581....... 591.......

P925014  364     GAGGTGGGGC CTGGGAAGAC TGGGTTGCAA CGGTCGCGAG ACTcccctcg
P925017  396     TGGGTGGGGC CAGGGAACTC GCGGCTTCGG CGCTCACGAG ATTT------
P925020  444     TGGGTGGGGC CAGGGACCTC GCGGCTTCCG CGCTCACGAG ATTT------
                 ********** ********** ********** ********** ****
                 ********** ********** ********** ********** ****
                 ********** ********** ********** ********** ****
                 ********** ********** ********** **********
                 ********** ********** ********** **********
                   ******** ******

alignment
position         601....... 611....... 621....... 631....... 641.......

P925014  414     ccgtagcgcg aagaAACGAG ACTGAAGAAG AGAGCAAGGT GGGAGGGGcg
P925017  440     ---------- ----AGCGAG ACCGAGGACG CAGGCGGAGC GGGCCGGGgc
P925020  488     ---------- ----AGCGAG ACTGAGGGCG CTAGCGGAGC AGGCCGctgc
                                ****** ********** ********** ********
                                ****** ********** ********** ******
                                ****** ********** ********** ******

alignment
position         651....... 661....... 671....... 681....... 691.......

P925014  464     cgctgggGAG CTTCGGCGCA TGCGCGCTGA GGCCTGCCTG ACCGACCTTC
P925017  476     g-CCT--GAG CTCCCGCGCA CGCGCACTGA GTCCCGTCTG CCCGACCTTC
P925020  524     tcCCC--GAG CTCCCGCGCA CGCGCAGTGA GCCTCGTCTG CCCGACCTTC
                   ***  *** ********** ********** ********** **********
                   ***  *** ********** ********** ********** **********
                   ***  *** ********** ********** ********** **********
                        *** ********** ********** ********** **********
                                 ***** ********** ********** **********
                                 ***** ********** ********** **********
                                                          ** *******

alignment
position         701....... 711....... 721....... 731....... 741.......

P925014  514     Agcagggctg tggCTACCAT GTTCTCTCGC GCGGGTGTCG ctGGGCTGTC
P925017  523     AAGGCT---- ---CCACCAT GTTCTCGCGG GCGAGCGTTG TCGGGCTGTC
P925020  572     AAGGTT---- ---CTACCAT GTTCTCGCGG GCGAGTATTG TCGGGCTGTC
                 ******        ******* ********** ********** **********
                 ******        ******* ********** ********** **********
                 ******        ******* ********** *****  *** **********
```

```
            ******        *******  ********** *****  *** **********
            *             *******  ********** *****  ***   ********
            *             *******  ********** *****  *     ********
                                                           ********
                                                           ********
                                                           ********
                                                           ********

alignment
position    751....... 761....... 771....... 781....... 791.......

P925014 564 GGCCTGGACC TTGCAGCCGC AATGGTATGg cagcttgcgg gaagatcggc
P925017 566 GGCCTGCGCC GTGCAGCCGC AATGGTAgGA CCAC------ ----------
P925020 615 GGCCTGCTCC TTGCAGCCGC AATGGTATGA CTAC------ ----------
            ********** ********** ********** ****
            ********** ********** *********
            ********** ********** *********
            ********** ********** *********
            ********** ********** *******
            ********** ********** *******
            ********** ********** *******
            ********** ********** *******
            ********** ********** *******
            ********** ********** *******

alignment
position    801....... 811....... 821....... 831....... 841.......

P925014 614 aggACCGCAA GGGATGGAAG AGCTTGGGCA CGGGGCAGGG aggaggagat
P925017 600 ---ATCGGGA GGGATGCGGG GGCTCGGGCT TGCTGCGAGG CAGGAGGGAT
P925020 649 ---GCCGGGA GGCATGCCGG GGCTTGGGCT TGCTGCAAGA CAGGAGGGAT
               ******* ********** ********** ********** **********
                          ******* ********** ********** **********
                          ******* ********** ********** **********
                          ******* ********** **********

alignment
position    851....... 861....... 871....... 881....... 891.......

P925014 664 gggcgcgggg gcagatgtgg ggtcgcaggg cctagctggg cccctggccc
P925017 647 ---------- ---------- ---------- ---------- ----------
P925020 696 ---------- ---------- ---------- ---------- ----------

alignment
position    901....... 911....... 921....... 931....... 941.......

P925014 714 gtcggaggcg ccgggtagcg GGGGCTGCAg gaagcggctc tgaggtcg--
P925017 647 ---------- ---------- GGaGCTGCAT GCTCACTCCT GGCGGAAGAA
P925020 696 ---------- ---------- GGGGCTGCAT GCTCGCTCCT GGCGGAGGAA
                                  ********** ********** **********
                                  ** ******* ********** **********
                                  ** ******* ********** **********
                                     ******* ********** **********

alignment
position    951....... 961....... 971....... 981....... 991.......

P925014 762 ---------- ---------- ---------- -------GGC AAGGTGTGCg
P925017 677 GGCCCCAGCT Ca-------- ---------- -GGCCGCGGG GATGTCTGCA
P925020 726 GACCCCAGCT Cttgcacggc ccggccactc gGGCCGCGGC AAGGTCTGCA
```

```
            ********** *                              ********* **********
            ********** *                              ********* **********
            ********** *                              ********* **********
            ********** *

alignment
position    1001...... 1011...... 1021...... 1031...... 1041......

P925014 775 ca-------- ---------- -------GGG CCGGGCTCCG GCGGAGCAAC
P925017 708 GAGCTGGAAG GTTGCGTGCC ggacccctGG TAGGGGTCAA AGAGAACAAC
P925020 776 GAGCTGGAAG GTCGCGTGCC agtcccgGGG CTGGGGTCAA AGGGAACAAC
            ********** **********        *** ********** **********
            ********** **********              ******** **********
            ********** **********              ******** **********
                                               ******** **********

alignment
position    1051...... 1061...... 1071...... 1081...... 1091......

P925014 800 GTCCCGCCCG GTTCCCtgag gtcggag--G GCCACCTGGG ACCTCGGAC
P925017 758 GTCACGGTCT GATCCCCTGA GACTGGAACG GCCATCCGGG ACCTCGGAC
P925020 826 GTCACGGCCT GATCCgCTGG GACTGGAACG GCCACCTGGG ACCTCAGGAC
            ********** ********** ********** ********** **********
            ********** *****  *** ********** ********** **********
            ********** *****  *** ********** ********** **********
            ********** *****               * ********** **********
                                           * ********** **********

alignment
position    1101...... 1111...... 1121...... 1131...... 1141......

P925014 848 tgcggcctGA GTCCAAATTG ACGTCTGGGC GGTCTTGAGT TCCCTCAGCT
P925017 808 CCGgACGGGA TTCGGGTTGA GCTTTGGAGC CATCTGGGTT TCGATCAACT
P925020 876 CCGtACGAGA GTCGGATTGA GCGTTGGAGC CATCTGGGTT TCGATCAACT
            ***  ***** ********** ********** ********** **********
            ***  ***** ********** ********** ********** **********
            ***  ***** ********** ********** ********** **********
                                                        **********

alignment
position    1151...... 1161...... 1171...... 1181...... 1191......

P925014 898 aatcagcact tcctgagctt ccACTTCCAC TGCAAGCCAG Gcccccagt c
P925017 858 CCGT------ ---------- --ACTTCCGC TCCGTACCCG GGAGCACAg C
P925020 926 CCGT------ ---------- --ACTTCCGC TCCGTACTAG GGAGCACAt C
            ****                    ******** ********** ********  *
            ****                    ******** ********** ********  *
            ****                    ******** ********** ********  *
            ****                    ******** ********** ********  *

alignment
position    1201...... 1211...... 1221...... 1231...... 1241......

P925014 948 tc-------- ------AGGG CCTGCAAGGT GACGGGagcc tgagcaatag
P925017 890 TTGCAAAGTC TTTAGAAAGG CCCTGAAGGT GAAGGGGcga ----------
P925020 958 TTGCAAAGTC TTTAGAAAGG GCCTCAAGGT GAAGGGGaga tccg------
            ********** ********** ********** *******
            ********** ********** ********** *******
            ********** ********** ********** *******
            ********** ********** ********** *******

alignment
position    1251...... 1261...... 1271...... 1281...... 1291......
```

```
P925014  984 c c CAGGGGAA GGT TAAGT- -  - - - - - - - - - -  - - - - - - - - - -  - - - - - - - - - -
P925017  930 - - CCGGGGAA CGT TTGGT t g  t a a c t t t t g g  c t t t g a a g c c  c t g t g a a a c a
P925020 1002 - - - - - - - - - -  - - - - - - - - - -  - - - - - - - - - -  - - - - - - - - - -  - - - - - - - - - -
                 * * * * * * * *  * * * * * * * *

alignment
position         1 3 0 1 . . . . . .  1 3 1 1 . . . . . .  1 3 2 1 . . . . . .  1 3 3 1 . . . . . .  1 3 4 1 . . . . .

P925014 1002 - - - - - - - - - -  - - - - - - - - - -  - - - - - - - - - -  - - - - - - - - - -  - - - - - - - - - -
P925017  978 c t g c a c g g g g  g a g c a g c g t g  a c c c t t t a a g  g t g t g a t g g c  t t g c t t g a g
P925020 1002 - - - - - - - - - -  - - - - - - - - - -  - - - - - - - - - -  - - - - - - - - - -  - - - - - - - - - -
```

Pairwise similarities:

For each pairwise alignment, the similarity (relative to the maximum similarity) and the number of identical nucleic acids (in % of shorter sequence) is given. Maximum values are underlined.

	**P925017 (1026 bp)**	**P925020 (1001 bp)**
**P925014 (1001 bp)**	0.228<br>37 %	0.242<br>42 %
**P925017 (1026 bp)**		**1.000**<br>79 %

Please note that the **similarity value 1.000** marks only the two **most similar** sequences, it does not necessarily mean that these sequences are identical.

**Extract alignment region**	
**Positions in alignment**	from: to:
**Output file**	dialign.seq

Extract aligned sequences

For comments, questions or bug reports, please contact support@genomatix.de

© Genomatix Software GmbH 1998-2005 - All rights reserved. License Agreement GEMS Launcher 4.1.1

Genomatix-Logo Logout Personal Messages GEMS-Logo
Logout Personal Messages

Genomatix_MainGEMSElDorado Gene2PromoterBiblioSphere
Frequently_asked_questions Your ResultsYour SequencesYour ProtocolHelp

GEMS Launcher Task: *DiAlign TF*: Multiple alignment plus TF sites working on C5-ATP5E-HM.seq (2 seq.)

[Alignment] [Pairwise similarities]

DiAlign professional TF Release 3.1 December 2004 **Tue May 24 16:38:33 2005**

Solution parameters:

**Sequence file:**	C5-ATP5E-HM.seq (2 seq.)
**Type of sequences:**	nucleotide sequences
**Threshold T:**	0.00
**'*' signs below alignment denote:**	diagonal similarity (max. similarity is represented by 10 '*' signs)
**Alignment output:**	complete alignment is shown<br># nucleic acids per line: 50
**TF output:**	common TF matches located in aligned regions (common to 2 (100.0 %) sequences)
**Family matches:**	yes
**MatInspector library:**	Matrix Family Library Version 5.0 (February 2005)
**Selected groups** (core/matrix sim):	• ALL user_defined.lib (0.75/Optimized)<br>• ALL vertebrates.lib (0.75/Optimized)

Aligned Sequences:

No.	Sequence Name	Sequence Description	Len
1	P927229	P927229 sym=ATP5E|loc=Loc514|taxid=9606|spec=Homo sapiens|chr=20|ctg=NT_011362|str=(-)|start=22659786|end=22660796|len=1011|tss=501,511|comm=ATP synthase, H+ transporting, mitochondrial F1 complex, epsilon subunit; (AK026556/511/gold;NM_006886/501/bronze;)	101[illegible]
2	mATP5E	mATP5E m6_dna range=chr2:173888263-173889536 5'pad=0 3'pad=0 revComp=TRUE strand=? repeatMasking=none	127[illegible]

Alignment (DiAlign format):

Please note that only **upper-case** letters are considered to be **aligned**. For more information, please have a look at the user guide.

V$ECAT V$PCAT V$PBXC V$SP1F V$ETSF U$NRF2 V$AREB V$YY1F

alignment position 1 . . . . 11 . . . . 21 . . . . 31 . . . . 41 . . . .

```
P927229      1 gggggtcctc cactg----- ---------- ---------- ----------
mATP5E       1 tcaagttctt agcttcctac tttaaaggat tactcagttt aataattctt

alignment
position       51........ 61........ 71........ 81........ 91........
P927229     16 ---------- ---------- ---------- ---------- ----------
mATP5E      51 tggacatttg taaaagattt ttaaaaagta aaggaaaaat cgtttgacaa

alignment
position       101....... 111....... 121....... 131....... 141.......
P927229     16 ---------- ---------- ---------- ---------- ----------
mATP5E     101 gtggaatgaa tgttccagct aaacagagca ctacttaaca ttgttggaca

alignment
position       151....... 161....... 171....... 181....... 191.......
P927229     16 ---------- ---GGTTTCC AGGGTTTAg- ---------- ----------
mATP5E     151 aagtgtcagg tacGGTTTCT AGGAGTTAca cctgttctgg aagggtctga
                             ******* ********

alignment
position       201....... 211....... 221....... 231....... 241.......
P927229     32 ---------- ---------- ---------- ---------- ----------
mATP5E     201 tgccctgatg atgttttttt aagtatatac atttccactt tgcaatgtgg

alignment
position       251....... 261....... 271....... 281....... 291.......
P927229     32 ---------- ---------- ---------- ---------- ----------
mATP5E     251 ttccatcgtg aattctgcct gagtacaaat ggaatcctgg gcatgggtac

alignment
position       301....... 311....... 321....... 331....... 341.......
P927229     32 ---------- ---------- TGTCACTCCG AGaaaggggg tgccctaaga
mATP5E     301 atgcagacag gaacattagc TGTCACTCAG AGcggg---- ----------
                                     ********** **

alignment
position       351....... 361....... 371....... 381....... 391.......
P927229     62 gcctttctaa atctcagctc tcaggacacg cttgtctgtc cacagattta
mATP5E     337 ---------- ---------- ---------- ---------- ----------

alignment
position       401....... 411....... 421....... 431....... 441.......
P927229    112 aaaaagccgg cgccgtttcc ctgaagcggg cgcaaagcca ggccCTCGGG
mATP5E     337 ---------- ---------- ---------- ---------- ----CTCGAG
                                                               ******

alignment
position       451....... 461....... 471....... 481....... 491.......
P927229    162 GCGGCCGTGC CCGCgccgcc tc-------- ---------- -------GCG
mATP5E     343 GAGGCCCCGC CCCCtgcccg cagcacctgg aaggtgaccg gcatgcaGCG
               ********** ****                                 ***
```

```
alignment
position        501.......  511.......  521.......  531.......  541.......

P927229    187  TCCTCGCTTC  CCgaaagcac  gcggctcagt  ggtccttagc  gagcggggac
mATP5E     393  GCCTCGCTTT  CCcaggtgtc  tctacggcca  cagctt----  ----------
                **********  **

alignment
position        551.......  561.......  571.......  581.......  591.......

P927229    237  accccggcct  tcttgccccc  gtcgtccccg  ccggggtaat  acggaaggtc
mATP5E     429  ----------  ----------  ----------  ----------  ----------

alignment
position        601.......  611.......  621.......  631.......  641.......

P927229    287  gggggacaca  cgccCCGGGA  GCTCTTGGAG  CCAATAGGTG  GGCGCGGCCG
mATP5E     429  ----------  ----CCGGTA  GCCGCTCGGC  CAATGGGCAA  GGCGCGACCG
                                ******  **********  **********  **********

alignment
position        651.......  661.......  671.......  681.......  691.......

P927229    337  gccggcccgc  cccgggattg  gcacccgcag  tccagaccgc  GCGCGCCCAT
mATP5E     465  ----------  ----------  ----------  ----------  GCGCTCCTAT
                                                                **********
                                                                **********

alignment
position        701.......  711.......  721.......  731.......  741.......

P927229    387  TGGCTCCCGG  cgcccggggg  cttccgcggc  agggcagcag  gggccgtgct
mATP5E     475  TGGCTCCTGG  ttggcaaggg  c---------  ----------  ----------
                **********
                **********

alignment
position        751.......  761.......  771.......  781.......  791.......

P927229    437  caggggcggg  gccgGGTAGG  AGGCGGTGAT  TGGCAGGTCC  GCacgCCCCG
mATP5E     496  ----------  ----GGGATG  AGGCGCTGAC  TGGCAGGTCC  GCgtaCCCCA
                                ******  **********  **********  **   *****
                                ******  **********  **********  **   *****
                                ******  **********  **********  **   *****
                                ******  **********  **********  **   *****
                                                                     *****

alignment
position        801.......  811.......  821.......  831.......  841.......

P927229    487  CCTCTTCCGC  TTCTGTGGTC  TGATCTTCCT  GCGGCtgaac  cgcccggctg
mATP5E     532  CCCCTTCCGC  TACTCAGGCC  TGACCTTCCT  GCTGCcgggc  cggtttgagg
                **********  **********  **********  *****
                **********  **********  **********  *****
                **********  **********  **********  *****
                **********  **********  **********  *****
                **********  **********  **********  *****

alignment
position        851.......  861.......  871.......  881.......  891.......

P927229    537  agccgacatt  gccgGCGTCT  TGGCGATTCG  GCCCGACGAG  Ctccgctttc
mATP5E     582  ctactctgaa  ----GCGACC  CAGCGGTTCT  GCCCGACGCG  Cccgctcgag
                                ******  **********  **********  *
                                ******  **********  **********  *
```

```
alignment
position          901.......  911.......  921.......  931.......  941.......

P927229   587     gct ACAGCAT GGTGGCCTAC TGGAGACAGG CTGGACTCAG GTAggcccag
mATP5E    628     --- ACACCAT GGTGGCGTAC TGGCGACAGG CTGGACTCAG GTAcgcccag
                      ******* ********** ********** ********** ***
                      ******* ********** ********** ********** ***
                      ******* ********** ********** ********** ***
                      ******* ********** ********** ********** ***
                      ******* ********** ********** ********** ***
                      ******* ********** ********** ********** ***
                      ******* ********** ********** ********** ***
                      ******* ********** ********** ********** ***
                      ******* ********** ********** ********** ***
                      ******* ********** ********** ********** ***

alignment
position          951.......  961.......  971.......  981.......  991.......

P927229   637     gcctccagag GGAAGGGCTT CGCGAGCCGG CCCGCGCGCA TCCcggtgcc
mATP5E    675     aaagcgtt-- GGGAAGGCCT GGCCGGCCGT CACCAGGCCG TACgagac--
                             ********** ********** ********** ***

alignment
position          1001......  1011......  1021......  1031......  1041......

P927229   687     cagaggtcat gcaggacaca gagcagcggc cccacgcgtg accggggttg
mATP5E    721     ---------- ---------- ---------- ---------- ----------

alignment
position          1051......  1061......  1071......  1081......  1091......

P927229   737     GGCCAAGCCT CGggcgtcgC CGCCAAGATG GCGTCGCTGT GGGCAAAGCG
mATP5E    721     GGCCAAGCCA CGcggccacC CTCCAAGATG GCGTCGCCGT GGACAAGGCT
                  ********** **       * ********** ********** **********
                                      * ********** ********** **********
                                      * ********** ********** **********
                                      * ********** ********** **********
                                      * ********** ********** **********

alignment
position          1101......  1111......  1121......  1131......  1141......

P927229   787     CTcaggcggg cgcggcctcg acctcgcgtg ggagcgcgtc tgtgtcctgc
mATP5E    771     CTtcgcgggg tcatggggtc agggacccta aacagaggtg gctgctcctc
                  **
                  **
                  **
                  **
                  **

alignment
position          1151......  1161......  1171......  1181......  1191......

P927229   837     ctcgcttccc gacggtgcg- ---------- ---------- -------GCC
mATP5E    821     gggagaggta gcctacagta cctgcatgat cgctgtgacg gccaagtGCA
                                                                     ***

alignment
position          1201......  1211......  1221......  1231......  1241......

P927229   859     CAGCACCCac agagtgcccc t--------- ---------- ----------
mATP5E    871     CAGCACCCtt gacctggcca cgctgacact gaccttgctc acctgcgcag
                  ********
```

```
alignment
position          1251...... 1261...... 1271...... 1281...... 1291......
P927229    880 ---------- ---------- ---------- ---------- ----------
mATP5E     921 agggaggggc gcacaagttc attttcaagt gtgaccttag tgaccttttc

alignment
position          1301...... 1311...... 1321...... 1331...... 1341......
P927229    880 ---------- ---------- ---------- ---------- ----------
mATP5E     971 ccagtagtga aaaaaggtga aagccctcta acctgtggag cgccctgcag

alignment
position          1351...... 1361...... 1371...... 1381...... 1391......
P927229    880 ---------- ---------- ---------- ---------- ----------
mATP5E    1021 gggcccgtgg atgggtgcga gctgtgggcc taggagtggt ggtgcatggt

alignment
position          1401...... 1411...... 1421...... 1431...... 1441......
P927229    880 ---------- ---------- ---------- ---------- ----GGACAC
mATP5E    1071 ttccttgctc cagggagtgc atcttgttag tgtgctgggt ggagGAACAC
                                                               ******

alignment
position          1451...... 1461...... 1471...... 1481...... 1491......
P927229    886 CTTTGTGAAT GGCAGGTTtc tcgagagaga ggccag---- ----------
mATP5E    1121 CTTTCTGGGT ATCAGGCTga aggcccagtg ccccgacaga gttcaagcaa
               ********** ********

alignment
position          1501...... 1511...... 1521...... 1531...... 1541......
P927229    922 ---------- ---------- ---------- ---------- -CTCTTATTt
mATP5E    1171 agttgtgtag aacaaaagga cggtaggctc acagttgatg cCTCTTATTa
                                                           ********

alignment
position          1551...... 1561...... 1571...... 1581...... 1591......
P927229    931 agcaaacctt tttcttctag aatggtgctg gagccaggtg gtgaaagtag
mATP5E    1221 aactatctcc catggcgtcc atcaccttat tttcctgcgc ccagccgctt

alignment
position          1601...... 1611...... 1621...... 1631
P927229    981 tacagtcaca tccacctcgg gaaacaggag a
mATP5E    1271 gaag------ ---------- ---------- -
```

Pairwise similarities:

For each pairwise alignment, the similarity (relative to the maximum similarity) and the number of identical nucleic acids (in % of shorter sequence) is given. Maximum values are underlined.

	**mATP5E (1274 bp)**

**P927229 (1011 bp)**	1.000 26 %

Please note that the **similarity value 1.000** marks only the two **most similar** sequences, it does not necessarily mean that these sequences are identical.

**Extract alignment region**	
**Positions in alignment**	from: to:
**Output file**	dialign.seq

Extract aligned sequences

For comments, questions or bug reports, please contact support@genomatix.de

© Genomatix Software GmbH 1998-2005 - All rights reserved. License Agreement GEMS Launcher 4.1.1

Logout Personal Messages

GEMS Launcher Task: *DiAlign TF*: Multiple alignment plus TF sites working on C5-ATP5E.seq (3 seq.)

[Alignment] [Pairwise similarities]

DiAlign professional TF Release 3.1 December 2004 **Tue May 24 16:14:29 2005**

Solution parameters:

**Sequence file:**	C5-ATP5E.seq (3 seq.)
**Type of sequences:**	nucleotide sequences
**Threshold T:**	0.00
**'*' signs below alignment denote:**	diagonal similarity (max. similarity is represented by 10 '*' signs)
**Alignment output:**	complete alignment is shown<br># nucleic acids per line: 50
**TF output:**	common TF matches located in aligned regions (common to 3 (100.0 %) sequences)
**Family matches:**	yes
**MatInspector library:**	Matrix Family Library Version 5.0 (February 2005)
**Selected groups** (core/matrix sim):	• ALL user_defined.lib (0.75/Optimized)<br>• ALL vertebrates.lib (0.75/Optimized)

Aligned Sequences:

No.	Sequence Name	Sequence Description	Len
1	P927229	P927229 sym=ATP5E|loc=Loc514|taxid=9606|spec=Homo sapiens|chr=20|ctg=NT_011362|str=(-)|start=22659786|end=22660796|len=1011|tss=501,511|comm=ATP synthase, H+ transporting, mitochondrial F1 complex, epsilon subunit; (AK026556/511/gold;NM_006886/501/bronze;)	1011
2	mATP5E	mATP5E m6_dna range=chr2:173888263-173889536 5'pad=0 3'pad=0 revComp=TRUE strand=? repeatMasking=none	1274
3	rATP5E	rATP5E n3_dna range=chr14:83622419-83623508 5'pad=0 3'pad=0 revComp=FALSE strand=? repeatMasking=none	1090

Alignment (DiAlign format):

Please note that only **upper-case** letters are considered to be **aligned**. For more information, please have a look at the user guide.

V$ETSF V$SP1F

```
alignment position 1......... 11........ 21........ 31........ 41........
P927229          1 gggggtcctc cactg----- ---------- ---------- ----------
mATP5E           1 tcaagttctt agcttcctac tttaaaggat tactcagttt aataattctt
rATP5E           1 ccgc------ ---------- ---------- ---------- ----------

alignment position 51........ 61........ 71........ 81........ 91........
P927229         16 ---------- ---------- ---------- ---------- ----------
mATP5E          51 tggacaTTTG TAAAAGATTT TTAAAAAgta aaggaaaaat cgtttgacaa
rATP5E           5 ------TTTG CAAGAGAAAT TTGAAAAtag agccacagcc aagaccttcg
                         **** ********** *******

alignment position 101....... 111....... 121....... 131....... 141.......
P927229         16 ---------- ---------- ---------- ---------- ----------
mATP5E         101 gtggaatgaa tgttccagct aaa------- ---------- ----------
rATP5E          49 tttcagagag tgatttcata acaggagaag cttgagagag tcgactctta

alignment position 151....... 161....... 171....... 181....... 191.......
P927229         16 ---------- ---------- ---------- ---------- ----------
mATP5E         124 ---------- ---------- ---------- ---------- ----------
rATP5E          99 atctggccga gctgtttcca tgtgtttgac gaagttatta actcagcctg

alignment position 201....... 211....... 221....... 231....... 241.......
P927229         16 ---------- ---------- ---------- ---------- ----------
mATP5E         124 ---------- ---------- ---------- ---------- ----------
rATP5E         149 gtccagaggg ctaaaagtgt gtttgttggc tcatcctcct aatccaggtt

alignment position 251....... 261....... 271....... 281....... 291.......
P927229         16 ---------- ---------- ---------- ---------- ----------
mATP5E         124 ---------- -------CAG AGCACTACTT AACATTGTTG GACAAAGTGT
rATP5E         199 ctctggagac aagagtgCTG AACATTCCCT AGAGTTCTTG GACTATGTAA
                                      *** ********** ********** **********

alignment position 301....... 311....... 321....... 331....... 341.......
P927229         16 -------GGT TTCCAGGGTT TAGTGTCACT CCGAGaaagg gggtgcccta
mATP5E         157 CAGGTAcGGT TTCTAGGAGT TAcacctgtt ctggaagggt ctgatgccct
rATP5E         249 CAAGAAaac- -----GGGGT GTGTGTAACT CGGAGcgaat tgctaactta
                   ****** *** ********** ********** *****

alignment position 351....... 361....... 371....... 381....... 391.......
P927229         59 agagcctttc t--------- ---------- ---------- ----------
mATP5E         207 gatgatgttt ttTTAAGTAT Atacatttcc actttgcaat gtggttccat
rATP5E         293 ctgtaaactg --TTAAGTAT Aattaagaaa tacaagttaa tg--------
                                ******** *

alignment position 401....... 411....... 421....... 431....... 441.......
P927229         70 ---------- ---------- AAATCTCAGC TCTCAGGACA CGCTTGtctg
mATP5E         257 cgtgaattct gcCTGAGTAC AAATGGAATc ctgggcatgg gtacatgcag
rATP5E         333 ---------- --CTAAGTAC AAATGTAATG TACAAAGACA AGCTTGaaag
                                ******** ********** ********** ******

alignment position 451....... 461....... 471....... 481....... 491.......
P927229        100 tccacagatt ---------- ---------- ---------- ----------
```

```
mATP5E      307 acaggaacat tagctgtcac tcagagcggg ---------- ----------
rATP5E      371 gagagaccaa tttatatttc ccattatata ctcttttgtt gtcaaagtta

alignment position 501....... 511....... 521....... 531....... 541.......
P927229     110 ---------- ---------- ---------- ---------- ----------
mATP5E      337 ---------- ---------- ---------- ---------- ----------
rATP5E      421 aacctaaggt acataactca aaacaagttt agattaaact taaaatctgt

alignment position 551....... 561....... 571....... 581....... 591.......
P927229     110 ----TAAAAA AGCcggcgcc gtttccctga agcgggcgca aagccaggcc
mATP5E      337 ---------- ---------- ---------- ---------- ----------
rATP5E      471 ttgaTAAAAA AGCaatatcc g--------- ---------- ----------
                    ****** ***

alignment position 601....... 611....... 621....... 631....... 641.......
P927229     156 CTCGGGGCGG CCGTGCCCGC gccgcctc-- ---------- ----------
mATP5E      337 CTCGAGGAGG CCCCGCCCCC tgcccgcagc acctggaagg tgaccggcat
rATP5E      492 ---------- ---------- ---------- ---------- ----------
                ********** **********

alignment position 651....... 661....... 671....... 681....... 691.......
P927229     184 ---GCGTCCT CGCTTCCCga aagcacgcgg ctcagtggtc cttagcgagc
mATP5E      387 gcaGCGGCCT CGCTTTCCca ggtgtctcta cggccacagc tt--------
rATP5E      492 ---------- ---------- ---------- ---------- ----------
                   ******* ********

alignment position 701....... 711....... 721....... 731....... 741.......
P927229     231 ggggacaccc cggccttctt gcccccgtcg tccccgccgg ggtaatacgg
mATP5E      429 ---------- ---------- ---------- ---------- ----------
rATP5E      492 ---------- ---------- ---------- ---------- ----------

alignment position 751....... 761....... 771....... 781....... 791.......
P927229     281 aaggtcgggg gacacacgcc CCGGGAGCTC TTGGAGCCAA TAGGTGGGCG
mATP5E      429 ---------- ---------- CCGGTAGCCG CTCGGCCAAT GGGCAAGGCG
rATP5E      492 ---------- ---------- ---------- ---------- ----------
                                      ********** ********** **********

alignment position 801....... 811....... 821....... 831....... 841.......
P927229     331 CGGCCGgccg gcccgccccg ggattggcac ccgcagtcca gaccgcGCGC
mATP5E      459 CGACCG---- ---------- ---------- ---------- ------GCGC
rATP5E      492 ---------- ---------- ---------- ---------- ----------
                ******                                          ****

alignment position 851....... 861....... 871....... 881....... 891.......
P927229     381 GCCCATTGGC TCCCGGcgcc ggggggcttc cgcggcaggg cagcaggggc
mATP5E      469 TCCTATTGGC TCCTGGttgg caagggc--- ---------- ----------
rATP5E      492 ---------- ---------- ---------- ---------- ----------
                ********** ******

alignment position 901....... 911....... 921....... 931....... 941.......
P927229     431 cgtgctcagg ggcggggccg GGTAGGAGGC GGTGATTGGC AGGTCCGCac
mATP5E      496 ---------- ---------- GGGATGAGGC GCTGACTGGC AGGTCCGCgt
rATP5E      492 ---------- ---------- ---------- ---------- ----------
```

```
                                    ********** ********** ********
                                    ********** ********** ********

alignment position  951.......  961.......  971.......  981.......  991.......
P927229     481  gCCCCGCCTC  TTCCGCTTCT  GTGGTCTGAT  CTTCCTGCGG  CTGAACCGcc
mATP5E      526  aCCCCACCCC  TTCCGCTACT  CAGGCCTGAC  CTTCCTGCTG  CCGGGCCGGt
rATP5E      492  ----------  ----------  ----------  -TTCCTGCTG  CCGGGCCGGg
                   *********  **********  ********** ********** *********
                   *********  **********  ********** ********** *********
                                                       *********  *
                                                       *********  *

alignment position  1001......  1011......  1021......  1031......  1041......
P927229     531  cggctgagcc  gacattgccg  GCGTCTTGGC  GATTCGGCCC  GACGAGCTCC
mATP5E      576  ttgAGGCTAC  TCTGAA----  GCGACCCAGC  GGTTCTGCCC  GACGCGC-CC
rATP5E      511  ctcAGGCTAC  TCTGAA----  GCGACCCAGC  AGTTCTGCCT  GACACGCCCC
                     *******  ******      **********  **********  **********
                     *******  ******      **********  **********  *******  **
                     *******  ******      **********  **********  *******  **
                     *******  ******      **********  **********  *******  **
                                          **********  **********  *******  **

alignment position  1051......  1061......  1071......  1081......  1091......
P927229     581  GCTttcgctA  CAGCATGGTG  GCCTACTGGA  GACAGGCTGG  ACTCAGGTAg
mATP5E      621  GCTCGAG--A  CACCATGGTG  GCGTACTGGC  GACAGGCTGG  ACTCAGGTAC
rATP5E      557  GCTCGAG--A  CACCATGGTG  GCTTACTGGC  AACAGGCTGG  ACTCAGCTAC
                 *******   *  **********  **********  **********  **********
                 *******   *  **********  **********  **********  *********
                 *******   *  **********  **********  **********  *********
                 *******   *  **********  **********  **********  *********
                 ***       *  **********  **********  **********  *********
                           *  **********  **********  **********  *********
                           *  **********  **********  **********  *********
                           *  **********  **********  **********
                           *  **********  **********  **********
                           *  **********  **********  **********

alignment position  1101......  1111......  1121......  1131......  1141......
P927229     631  gcccaggcct  ccagagGGAA  GGGCTTCGCG  AGCCGGCCCG  CGCGCATCCc
mATP5E      669  GCCCAGaaag  cgtt--GGGA  AGGCCTGGCC  GGCCGTCACC  AGGCCGTACg
rATP5E      605  ATCCGGttct  cccagatctg  tgcaaaagca  gtgagggat-  ----------
                 ******             ****  **********  **********  *********

alignment position  1151......  1161......  1171......  1181......  1191......
P927229     681  ggtgcccaga  ggtcatgcag  gacacagagc  agcggcccca  cgcgtgaccg
mATP5E      717  agac------  ----------  ----------  ----------  ----------
rATP5E      644  ----------  ----------  ----------  ----------  ----------

alignment position  1201......  1211......  1221......  1231......  1241......
P927229     731  gggttgGGCC  AAGCCTCGgg  cgtcgCCGCC  AAGATGGCGT  CGCTGTGGGC
mATP5E      721  ------GGCC  AAGCCACGcg  gccacCCTCC  AAGATGGCGT  CGCCGTGGAC
rATP5E      644  ----------  ----------  ----------  ----------  ----------
                       ****  ********         *****  **********  **********
                                              *****  **********  **********

alignment position  1251......  1261......  1271......  1281......  1291......
P927229     781  AAAGCGCTca  ggcgggcgcg  gcctcgacct  cgcgtgggag  cgcgtctgtg
```

```
mATP5E         765 AAGGCTCTtc gcggggtcat ggggtcaggg accctaaaca gaggtggctg
rATP5E         644 ---------- ---------- ---------- ---------- ----------
                   ********
                   ********

alignment position 1301...... 1311...... 1321...... 1331...... 1341......
P927229        831 tcctgcctcg cttcccgacg gtgcg----- ---------- ----------
mATP5E         815 ctcctcggga gaggtagcct acagtacctg catgatcGCT GTGACGGCCA
rATP5E         644 ---------- ---------- ---------- -------GCC CTGAAGACCG
                                                           *** **********

alignment position 1351...... 1361...... 1371...... 1381...... 1391......
P927229        856 ---GCCCAGC ACCCac---- ---------- ---------- ----------
mATP5E         865 AGTGCACAGC ACCCttgacc tggccacgct gacactgacc ttgctcacct
rATP5E         657 AGTTCAAAGC gaacgctgag aagacttcgg gcagcagcat ----------
                   ********** ****

alignment position 1401...... 1411...... 1421...... 1431...... 1441......
P927229        869 ---------- ---------- ---------- ---AGAGTGC CCCTGGACAC
mATP5E         915 gcgcagaggg aggggcgcac aagttcattt tcaAGTGTGA CCTTAGTGAC
rATP5E         697 ---------- ---------- ---------- ---------- ----------
                                                      ******* **********

alignment position 1451...... 1461...... 1471...... 1481...... 1491......
P927229        886 CTTTgtgaat ggcaggt--- ---------- ---------- ----------
mATP5E         965 CTTTtcccag tagtgAAAAA AGGTGAAAGc cctctaacct ----------
rATP5E         697 ---------- -----AAAAA CAGTGAAAGt gaagaagcct gactgaagcc
                   ****            ***** *********

alignment position 1501...... 1511...... 1521...... 1531...... 1541......
P927229        903 ---------- ---------- ---------- ---------- ----------
mATP5E        1005 ----GTGGAG CGCCCTGCAG GGGCCCCGTG ATGGgtgcga gctgtgggcc
rATP5E         732 tgaaGTGCTG AGTCTTCCAG GTGAACCATG TGGGcacctg ttccggcaga
                       ****** ********** ********** ****

alignment position 1551...... 1561...... 1571...... 1581...... 1591......
P927229        903 ---------- ---------- -TTCTCGAGA GAGAGGCCAG CTCTTATTTA
mATP5E        1051 taggagtggt ggtgcatggt tTCCTTGCTC CAGGGAGTGC ATCTTGTTAG
rATP5E         782 tggagatcag ccgtacc--- -TCCTGGAGA CAGACGACCC ATCTTGTTGA
                                          ********* ********** **********

alignment position 1601...... 1611...... 1621...... 1631...... 1641......
P927229        932 GCAAaccttt ttcttctaga atggtgctgg aGCCAGGTGG TGAAAGTAGT
mATP5E        1101 TGTGCTG--- ---------- ---------- -Ggtggagga acacctttct
rATP5E         828 TGAATTG--- ---------- ---------- -GCCATGTCA ATAAATTAAC
                   *******                           ********* **********

alignment position 1651...... 1661...... 1671...... 1681...... 1691......
P927229        982 ACAGTcacat ccacctcggg aaacaggaga ---------- ----------
mATP5E        1127 gggtatca-- ---------- ---------- ---------- ----------
rATP5E         854 ACAGTtcact ctcggctgga aaaaaacaaa acaaaattca aaacattgaa
                   *****

alignment position 1701...... 1711...... 1721...... 1731...... 1741......
P927229       1012 ---------- ---------- ---------- ---------- ----------
mATP5E        1135 ---------- ---------- ---------- ---------- GGCTGAAGGC
rATP5E         904 tgcctgagta aaatgggtta aatctataat aggcttccat GGCAGAAGGC
```

```
                                                                              * * * * * * * * * *
alignment position 1751......  1761......  1771......  1781......  1791......
P927229     1012 ----------  ----------  ----------  ----------  ----------
mATP5E      1145 CCAGTgcccc  gac-------  ----------  ----------  ----------
rATP5E       954 CCAGTagttt  ccttagaggg  agcataaatc  atccaaccag  agtaagttgc
                 *****
alignment position 1801......  1811......  1821......  1831......  1841......
P927229     1012 ----------  ----------  ----------  ----------  ----------
mATP5E      1158 ----------  ----------  --AGAGTTCA  AGCAAAGTTG  tgtagaacaa
rATP5E      1004 atgttagcct  cattacagaa  taAGGGTTCA  TGGGAAGTAG  gacctgctag
                                           ********  **********
alignment position 1851......  1861......  1871......  1881......  1891......
P927229     1012 ----------  ----------  ----------  ----------  ----------
mATP5E      1186 aaggacggta  ggctcacagt  tgatgcctct  tattaaacta  tctcccatgg
rATP5E      1054 aggcctg---  ----------  ----------  ----------  ----------

alignment position 1901......  1911......  1921......  1931......  1941...
P927229     1012 ----------  ----------  ----------  ----------  --------
mATP5E      1236 cgtccatcac  cttatttTCC  TGCGCCCAgc  cgcttgaag-  --------
rATP5E      1061 ----------  -------TCC  TGCTCCCAct  atgtaggcca  aaacaat
                                    ***  ********
```

Pairwise similarities:

For each pairwise alignment, the similarity (relative to the maximum similarity) and the number of identical nucleic acids (in % of shorter sequence) is given. Maximum values are underlined.

	mATP5E (1274 bp)	rATP5E (1090 bp)
**P927229 (1011 bp)**	0.958<br>26 %	0.343<br>16 %
**mATP5E (1274 bp)**		1.000<br>22 %

Please note that the **similarity value 1.000** marks only the two **most similar** sequences, it does not necessarily mean that these sequences are identical.

Extract alignment region	
**Positions in alignment**	from: [ ] to: [ ]
**Output file**	dialign.seq

Extract aligned sequences

For comments, questions or bug reports, please contact support@genomatix.de

© Genomatix Software GmbH 1998-2005 - All rights reserved.

License Agreement

GEMS Launcher 4.1.1

Genomatix-Logo	Logout<br>Logout	Personal<br>Personal	Messages<br>Messages	GEMS-Logo

Genomatix_MainGEMSElDoradoGene2Promoter BiblioSphere
Frequently_asked_questions Your ResultsYour SequencesYour_ProtocolHelp

GEMS Launcher Task: *DiAlign TF*: Multiple alignment plus TF sites working on promoters.seq (3 seq.)

[Alignment] [Pairwise_similarities]

DiAlign professional TF Release 3.1 December 2004 **Tue May 24 16:57:02 2005**

Solution parameters:

**Sequence file:**	promoters.seq (3 seq.)
**Type of sequences:**	nucleotide sequences
**Threshold T:**	0.00
**'*' signs below alignment denote:**	diagonal similarity (max. similarity is represented by 10 '*' signs)
**Alignment output:**	complete alignment is shown<br># nucleic acids per line: 50
**TF output:**	common TF matches located in aligned regions (common to 3 (100.0 %) sequences)
**Family matches:**	yes
**MatInspector library:**	Matrix Family Library Version 5.0 (February 2005)
**Selected groups** (core/matrix sim):	• ALL user_defined.lib (0.75/Optimized)<br>• ALL vertebrates.lib (0.75/Optimized)

Aligned Sequences:

No.	Sequence Name	Sequence Description	Length
1	P978188	sym=ATP5F1|loc=Loc515|taxid=9606|spec=Homo sapiens|chr=1|ctg=NT_019273|str=(+)|start=8077683|end=8078683|len=1001| tss=501| comm=ATP synthase, H+ transporting, mitochondrial F0 complex, subunit b, isoform 1; (NM_001688/501/silver;)	1001 bp
2	P978189	sym=Atp5f1|loc=Loc11950|taxid=10090|spec=Mus musculus|chr=3|ctg=NT_039239|str=(-)|start=7786845|end=7787863|len=1019| tss=501,507,509,518,519| comm=ATP synthase, H+ transporting, mitochondrial F0 complex, subunit b, isoform 1; (AK011312/524/gold;AK002960/535/gold;AK019459/518/gold; AK081249/536/gold;NM_009725/526/bronze;)	1019 bp
3	P978190	sym=Atp5f1|loc=Loc171375|taxid=10116|spec=Rattus norvegicus|chr=2|ctg=NW_047627|str=(-)|start=9451549|end=9452549| len=1001|tss=501| comm=ATP synthase, H+ transporting, mitochondrial F0 complex, subunit b, isoform 1; (NM_134365/536/bronze;)	1001 bp

Alignment (DiAlign format):

Please note that only **upper-case** letters are considered to be **aligned**. For more information, please have a look at the user guide.

V$NFKB V$RREB V$AP2F V$PLAG V$ETSF V$EBOX V$HIFF V$CHRE V$ZBPF V$MAZF V$E2FF V$HAND V$YY1F V$GLIF

```
alignment
position        1.........  11........  21........  31........  41........

P978188    1  cagggtcagg  ataacatgca  gggcggggat  gggctgggag  ggtcagtctc
P978189    1  agcacCG---  ----------  ----------  ----------  ----------
P978190    1  gaag-CG---  ----------  ----------  ----------  ----------
                   **
                   **

alignment
position        51........  61........  71........  81........  91........

P978188   51  cGGGGAGGGG  CGCCCTGGGC  CGGGGTAAGG  GAGCTCCCAG  G-CCCGGGAT
P978189    8  -GGAGAGGGC  ACCCGAGACT  GGGGGTAAAG  GGGCTTCCAG  GcCCCGGGAT
P978190    7  -GGAGAGGGT  ACCCGTCACT  GGGTAAAGGG  GGACTTCCAG  G-CCCGGGAT
               *********  **********  **********  **********  * ********
               *********  **********  **********  **********  * ********
                                                     *******  * ********
                                                     *******  * ********
                                                     ******
                                                                ********
                                                                ********
                                                                ********
                                                                ********
                                                                ********

alignment
position        101.......  111.......  121.......  131.......  141.......

P978188  100  CTCGGCTCAC  CGGACCGGTA  CCGCGCAGCC  TCCAACTGCC  GTTCCATGCA
P978189   57  CTCGGCTCAC  CAGACCGGTA  CCGTGCAGCC  TCCAATTGAC  GTTCCATGCA
P978190   55  CTCGGCTCAC  CAGAGCGGTA  CCGTGCAGCT  TCCAACTGCC  GTTCCATGCA
              **********  **********  **********  **********  **********
              **********  **********  **********  **********  **********
              **********  **********  **********  **********  **********
              **********  **********  **********  **********  **********
              **********  **********  **********  **********  **********
              **********  **********  **********  **********  **********
              **********  **********  **********  **********  **********
              **********  **********  *** *****   **********  **********
              **********  **********  *** *****   **********  **********
              **********  **********  *** *****   **

alignment
position        151.......  161.......  171.......  181.......  191.......

P978188  150  GGCGGGCGCA  TTTGGGGGAA  GATTCCACTC  CCGGGCCGCC  GGGGGCACTA
P978189  107  TGCGGGCGCA  TTGGGGGGCA  GGTTCCACTC  GCGGGCCGCC  GGGGGCACGA
P978190  105  GGCGGGCGCA  TTGGGGGGCA  GGTTCCACTC  GCGGGCCGCC  GGGGGCACGA
              **********  **********  **********  **********  **********
              **********  **********  **********  **********  **********
              **********  **********  **********  **********  **********
              **********  **********  **********  **********  **********
              **********  **********  **********  **********  **********
              **********  ** *******  **********  **********  **********
              **********  ** *******  **********  **********  **********
              **********  ** *******  * ********  **********  **********
```

```
            ********** **     *** * ******** ********** **********

alignment
position    201....... 211....... 221....... 231....... 241.......

P978188 200 GGGGGGGTGG GGTTTCCTTC CGCATCTCCA CGGTTCCAAC TCCAACCTAC
P978189 157 GCGGAGGAGG GGtgTCCTTC CGCATCTCCA CGATTCCAAA TCTAACCTAC
P978190 155 GCGGAGGTGG GCTTTCCTTC CGCATCTCCA CGATTCCAAA TCTAACCTAC
            ********** ********** ********** ********** **********
            ********** ********** ********** ********** **********
            ********** **  ****** ********** ********** **********
            ********** **  ****** ********** ********** **********
            ********** **  ****** ********** ********** **********
            *******        ****** ********** ********** **********
            *******        ****** ********** ********** **********
            * *****        ****** ********** ** ******* **********
            * *****        ****** ********** ** ******* **********

alignment
position    251....... 261....... 271....... 281....... 291.......

P978188 250 ACTCAAACTG GACGCCGGCC GGAGACTCCG CTCCGGcagc aaACCCCACC
P978189 207 ACCCAGACTC GACGAGAGAC CCTGACTCGG CGCCGGAGGG ACcgCACACC
P978190 205 ACCCAAACTA GACGAGAtcc tgact----G CGCTGGAGGA ACACCACACC
            ********** ********** ********** ********** **********
            ********** ****                               ********
            ****                                          ********
            ****                                            ******
            ****                                            ******
            ****                                            ******
            **                                                ****
            **                                                ****
            **

alignment
position    301....... 311....... 321....... 331....... 341.......

P978188 300 TGGTGCACCT CTGAGCCTCC GCCCCTCTCC cgagggaacc gcaaCTCTAC
P978189 257 TGGTGTACCT CTGCGCAACC GCCCCTCTCC gagGGAATTC G---CTCTAC
P978190 251 TGGTGCACCT CTGCGCATCC GCCCCTCTCC a--GGGATTC G---CTCTAC
            ********** ********** **********    ******* *   ******
            ********** ********** **********    ******* *   ******
            ********** ********** **********    ******* *   ******
            ********** ********** **********                ******
            ********** ********** **********                ******
            ********** ********** **********                ******
            ********** ********** **********
            ********** ********** **********

alignment
position    351....... 361....... 371....... 381....... 391.......

P978188 350 TTCTCGCGAG AATTGCTTCT ATGGCTCCAT CCTGc-TTTC CGGCTGtcgc
P978189 304 TTCTCGCGAG AATTGTGTCT ACCCTTCCAG CCTGCTTTTC CGTCTCCTGT
P978190 296 ATCTCGCGAG AGTTGTGTCT ACaCTTCCAG CTCGCTTTTC CGTCTGCTAT
            ********** ********** ********** ********** **********
            ********** ********** ********** ********** **********
            ********** ********** **  ******* ****
            ********** ********** **  ******* ****
            ********** ********** **
            ********** ********** *
```

```
alignment
position         401.......  411.......  421.......  431.......  441.......

P978188   399    cctcatgcga  taggctctca  gcgttacttg  a---------  ----------
P978189   354    CCCCGCAGCG  GGA-------  -----TTTTG  TTTGTTTCCC  TCCCtcagcc
P978190   346    ACCCGCAACG  GGAtcttcgg  actttTTTTT  TTTTTTTCCC  GCCCc-----
                 **********  ***              *****  **********  ****
                 **********  ***

alignment
position         451.......  461.......  471.......  481.......  491.......

P978188   430    ----------  ------CTCT  TCTCGCGATA  atttttttta  aaaatctccc
P978189   392    ccacccattt  tTTCTCCTCT  TCTCGCGATA  GTGGGgaggg  ggcGGGGAAA
P978190   391    ----------  -TTTTCCTCT  TCTCGCGATA  GGGGGag---  ---GGGGAAA
                              *********  **********  *****          *******
                              *********  **********  *****          *******
                                   ****  **********

alignment
position         501.......  511.......  521.......  531.......  541.......

P978188   464    aaggaaagtt  gaaggaagag  tacaaaattt  tca----TCT  CGCGAGACTT
P978189   442    AAGCCGAGAA  CTTCCGAGAA  AAAGCGAGCt  GATCTCTTCT  CGCGAGATGT
P978190   424    AAAATCAGCA  CTTCCGAGAG  AAAGTGTGCa  GATCTCTTCT  CGCGAGATAT
                 **********  **********  *********   **********  **********
                 **********  **********  *********   **********  **********
                                                     **********  **********
                                                            ***  **********
                                                            ***  **********
                                                            ***  **********
                                                            ***  **********
                                                            ***  **********

alignment
position         551.......  561.......  571.......  581.......  591.......

P978188   510    GTGAGCGGCC  ATCTTGGTCC  TGCCCtgaca  gattctccta  TCGGGGTCAC
P978189   492    GCGAGCGGCC  ATCTTGCTCC  TGCCCCTGAC  AGGCTCGGG-  TCGGGGTCAC
P978190   474    GTGAGCGGCC  ATCTTGTTCC  TGCCCCTGAG  TGTCTCGGG-  TCGGGGTCAC
                 **********  **********  **********  *********   **********
                 **********  **********  **********  *********   **********
                 **********  **********  **********  *********   **********
                 **********  **********  *****                   **********
                 **********  **********  *****                   **********
                 **********  ****** ***  *****
                 **********  ****** ***  *****
                 **********  ****** ***  *****

alignment
position         601.......  611.......  621.......  631.......  641.......

P978188   560    AGGGACgcta  agattGCTAC  CTGGACTTTC  GTTGACCATG  CTGTCCGGC
P978189   541    AGGGACAcgg  agcCCGCTGT  CCCGCTCTTC  GCAGACAATG  CTGTCCGGC
P978190   523    AGGGACAtcg  gtaCCGCTGT  CTGGATCTTC  GCAGACAATG  CTGTCCGGC
                 *******        *******  **********  **********  **********
                 *******        *******  **********  **********  **********
                 *******        *******  **********  **********  **********
                 ******           *****  **********  **********  **********
                 ******                                     ***  **********
                                                            ***  **********
                                                            ***  **********
                                                                   ********
```

```
                                                          ********
                                                          ********

alignment
position    651....... 661....... 671....... 681....... 691.......

P978188 610 TGGTACTTTC CGCCGCCGCC ACAGCGGGTA AGgggtaTAG ACCCTGCTCT
P978189 591 TGGTGCTTTC TGCTGCCGCC ACAGCGGGTG AGAGACGTAG AGCCTAGGTT
P978190 573 TGGTACTTTC TGCCGCCGCC ACAGCGGGTA AGAGATGTAA ACCCGAAGCT
            ********** ********** ********** ********** **********
            ********** ********** ********** ********** **********
            ********** ********** ********** ********** ****
            ********** ********** ********** **     *** ****
            ********** ********** ********** **
            ********** ********** ********** **
            ********** ********** *******
            ***  ***** ********** *******
            ***  ***** ********** *******
            ***

alignment
position    701....... 711....... 721....... 731....... 741.......

P978188 660 GGACTATCAG AGTGATTGGA AAGAAGCgaa tctaggggtg acagggaggg
P978189 641 AGCGTAGCAG GGTGACTAGG AAGAAGC--- ---------- ----------
P978190 623 GGCATATCAG GATGACTGGG AAGAAGC--- ---------- ----------
            ********** ********** *******
            ********** ********** *******
             ********* ********** *******

alignment
position    751....... 761....... 771....... 781....... 791.......

P978188 710 gagaagggcg cagcgacaCG GGCCTGAGAG GCGAGTGGTC AGTGCAGTTC
P978189 668 ---------- --------CA GGCCGGGGAG GCGGGAAGCG GGTAGAGGAC
P978190 650 ---------- --------TA GGTCAGGagG GCGGAAAGTG CGTAAAGAAA
                               ** ********** ********** **********
                               ** *******  * ********** **********

alignment
position    801....... 811....... 821....... 831....... 841.......

P978188 760 GGAaggg--- ---------- ---------- ---------- ----------
P978189 700 GGACTGCGGC GTTTGGGCCc gagctcaccc acgggagcGA TCTGGAGAGC
P978190 682 GGACTGCAGT GTTTGGGCCt ---------- --------GA TCCGAAGAGT
            ********** *********                     ** **********
            ********** *********

alignment
position    851....... 861....... 871....... 881....... 891.......

P978188 767 --------AG CGTGGGGTCT TGAGGGACGG GAAAGAGGGG TACATAGGCt
P978189 750 TCCCGGGCCG CCCAGCATCT CAGGGCGCGG CAGAGACGGA TGCCAGGGCC
P978190 714 TCCCTGACAG TGTAGGATTT CGGAGGACCT CTGAGAAGGA TGCAGGGGCC
            ********** ********** ********** ********** **********

alignment
position    901....... 911....... 921....... 931....... 941.......

P978188 809 -TAGTGATAG AGCCTggcgg gggta-AGGA AAGGGCAAAC CAGATTTCat
P978189 800 Gacacccg-- ---------- -------GGA AGGTAGAAAC GCGATTTCTC
P978190 764 GTAGTGATCG CGACTcaccg agaccgAGGA AGGTAGAAAA TGGATTTCTC
```

```
                 ********** *****           **** ********** **********
                 ********** *****            *** ********** **********
                 ********** *****            *** ********** **********

alignment
position         951....... 961....... 971....... 981....... 991.......

P978188   857    -TTGACTTTG CTGACCTTCG CCTTGTCTAT CTGCAGCCCC ctcTCTGAAC
P978189   831    CTTGACTTTG CTAACCTCCG TCTTGTGTAT TTCCAGCCCC GTGTCTGAAC
P978190   814    CTTGACTTTG CTAACCTACG TCTTGTGTGT TTGCAGCCCC GTGTCTGAAC
                 ********** ********** ********** ********** **********
                 ********** ********** ********** ********** **********
                 ********** ********** ********** ********** **********
                  ********* ********** ********** ********** **********
                  ********* ******* ** ********** **********    *******
                  ********* ******* ** ********** **********    *******
                  ********* ******* ** ********** **********    *******
                                    ** ********** **********    *******

alignment
position         1001...... 1011...... 1021...... 1031...... 1041......

P978188   906    AATGCAGCCT TCCTAGGTCC AGGGTAAGTG Tgaggataat gctccctttc
P978189   881    AACGCGGCCG CCCTAGGTCC AGGGTGAGTG T--------- ----------
P978190   864    AACGCGGCCG TCCTGGGTCC AGGGTAAGTG T---------- ----------
                 ********** ********** ********** *
                 ********** ********** ********** *
                 ********** ********** ********** *
                 ********** ********** ********** *
                 ********** ********** ********** *
                 ********    ********* ********** *
                 ********    ********* ********** *
                 ********

alignment
position         1051...... 1061...... 1071...... 1081...... 1091......

P978188   956    gtctttgttt tCACTACCTT TTATTtcccg attcctgccc ccaccc----
P978189   912    ---------- -CACCACGTC TTATTCTCTT tcCATTTCCT AGCGTCAAGC
P978190   895    ---------- -CACCACATT TTATTCTCTT aaCATTTCTT ACCGTTAAGC
                             ********* **********   ******** **********
                             ********* **********   ******** **********
                             ********* **********   ******** **********

alignment
position         1101...... 1111...... 1121...... 1131...... 1141......

P978188   1002   ---------- ---------- ---------- ---------- ----------
P978189   951    CAAAAATTGC TGTTTTCCTT CAcgTTAGCC AGCTTGCATA CTCATTCGGA
P978190   934    CAGAAATTAC TGTTTTCCTT CActTTAGCA AGCTTACATC CCCATTCAGA
                 ********** ********** **  ****** ********** **********
                 ********** ********** **  ****** ********** **********
                 ********** ********** **

alignment
position         1151...... 1161.....

P978188   1002   ---------- ---------
P978189   1001   CGTTTGTTCT TCTAattgc
P978190   984    CATTCGTTCT TCTActtg-
                 ********** ****
                 ********** ****
```

Pairwise similarities:

For each pairwise alignment, the similarity (relative to the maximum similarity) and the number of identical nucleic acids (in % of shorter sequence) is given. Maximum values are underlined.

	P978189 (1019 bp)	P978190 (1001 bp)
P978188 (1001 bp)	0.500 50 %	0.557 53 %
P978189 (1019 bp)		1.000 78 %

Please note that the **similarity value 1.000** marks only the two **most similar** sequences, it does not necessarily mean that these sequences are identical.

Extract alignment region	
**Positions in alignment**	from: to:
**Output file**	dialign.seq

Extract aligned sequences

For comments, questions or bug reports, please contact support@genomatix.de

© Genomatix Software GmbH 1998-2005 - All rights reserved. License Agreement GEMS Launcher 4.1.1

Genomatix-Logo Logout Logout Personal Personal Messages Messages GEMS-Logo

Genomatix_MainGEMSElDoradoGene2PromoterBiblioSphere
Frequently_asked_questionsYour ResultsYour SequencesYour ProtocolHelp

GEMS Launcher Task: *DiAlign TF*: Multiple alignment plus TF sites working on promoters.seq (3 seq.)

[Alignment] [Pairwise_similarities]

DiAlign professional TF Release 3.1 December 2004 **Tue May 24 17:43:43 2005**

Solution parameters:

**Sequence file:**	promoters.seq (3 seq.)
**Type of sequences:**	nucleotide sequences
**Threshold T:**	0.00
**'*' signs below alignment denote:**	diagonal similarity (max. similarity is represented by 10 '*' signs)
**Alignment output:**	complete alignment is shown<br># nucleic acids per line: 50
**TF output:**	common TF matches located in aligned regions (common to 3 (100.0 %) sequences)
**Family matches:**	yes
**MatInspector library:**	Matrix Family Library Version 5.0 (February 2005)
**Selected groups** (core/matrix sim):	• ALL user_defined.lib (0.75/Optimized)<br>• ALL vertebrates.lib (0.75/Optimized)

Aligned Sequences:

No.	Sequence Name	Sequence Description	Length
1	P879005	sym=ATP5G1|loc=Loc516|taxid=9606|spec=Homo sapiens|chr=17|ctg=NT_010783|str=(+)|start=5622841|end=5623941|len=1101| tss=601| comm=ATP synthase, H+ transporting, mitochondrial F0 complex, subunit c (subunit 9), isoform 1; (NM_005175/501/bronze;)	1101 bp
2	P879006	sym=Atp5g1|loc=Loc11951|taxid=10090|spec=Mus musculus|chr=11|ctg=NT_039521|str=(-)|start=7475581|end=7476681| len=1101|tss=601| comm=ATP synthase, H+ transporting, mitochondrial F0 complex, subunit c (subunit 9), isoform 1; (NM_007506/562/bronze;)	1101 bp
3	P879007	sym=Atp5g1|loc=Loc29754|taxid=10116|spec=Rattus norvegicus|chr=10|ctg=NW_047338|str=(-)|start=167149|end=168249| len=1101|tss=601| comm=ATP synthase, H+ transporting, mitochondrial F0 complex, subunit c, isoform 1; (NM_017311/565/bronze;)	1101 bp

Alignment (DiAlign format):

Please note that only **upper-case** letters are considered to be **aligned**. For more information, please have a look at the user guide.

V$STAF	V$OCT1	V$AP1R	V$CREB	V$EGRF	V$SF1F	V$YY1F	V$ECAT	V$PAX6	V$NFKB	V$ETSF	V$PCAT
V$EBOX	V$HIFF	V$OAZF	V$INSM	V$ZF5F	V$HNF4	V$BARB	V$RXRF	U$EREF	V$MYOD	V$AREB	V$RREB
V$CLOX	V$GATA	V$ATBF	V$CART	V$PDX1	V$FAST	V$LHXF	V$HOMF	V$RBIT	V$HNF1	V$NKXH	V$IKRS
V$IRFF	V$TEAF	V$CEBP	V$RORA	V$HOXF							

```
alignment
position         1 . . . . . . . . .  1 1 . . . . . . . .  2 1 . . . . . . . .  3 1 . . . . . . . .  4 1 . . . . . . . .

P879005      1  t c c a t g a c t g  c t g a a c a c c g  g c t g a g g t c c  a c a a a a c t g c  g c t c a c c g c t
P879006      1  - - - - - - - - - -  - - - - - - - - - -  - - - - - - - - - -  - - - - - - - - - -  - - - - - - - - - -
P879007      1  - - - - - - - - - -  - - - - - - - - - -  - - - - - - - - - -  - - - - - - - - - -  - - - - - - - - - -

alignment
position         5 1 . . . . . . . .  6 1 . . . . . . . .  7 1 . . . . . . . .  8 1 . . . . . . . .  9 1 . . . . . . . .

P879005     51  t g g g g a c t t t  c C A G C C G G C C  T T t a c t c t c c  a t - - G G A A A G  C G G T C T T T C C
P879006      1  - - - - - - - - - -  - C A G C C T G C C  T T A A T A C T T G  G A C G A A A A T G  G G G g - T T T T T
P879007      1  - - - - - - - - - -  - - - - - C T G C C  T T A A T T C T T G  G T C A G A A A C G  G G G A T T T T T T
                                        * * * * * * * * *  * * * * * * * * * *  * * * * * * * * * *  * * * * * * * * * *
                                                                                                                * * * * *
                                                                                                                * * * * *
                                                                                                                * * * * *

alignment
position         1 0 1 . . . . . . .  1 1 1 . . . . . . .  1 2 1 . . . . . . .  1 3 1 . . . . . . .  1 4 1 . . . . . . .

P879005     99  A C A A A C C T C C  A C G G T G G G A A  T T A C T G C A A A  G G C A T C G G C T  C T C C C G G C C T
P879006     39  A C A A A T C A C G  A T G G T G G A A G  G T A C T G T A A A  G G C G T C G G C T  C T C C T T G C C A
P879007     36  A C A A A T C A C T  A T G G T G G A A T  G T A C T G T A A A  G G C G T C G G C T  C T C C C T G C C A
                * * * * * * * * * *  * * * * * * * * * *  * * * * * * * * * *  * * * * * * * * * *  * * * * * * * * * *
                * * * * * * * * * *  * * * * * * * * * *  * * * * * * * * * *  * * * * * * * * * *  * * * * * * * * * *
                * * * * * * * * * *  * * * * * * * * * *  * * * * * * * * * *  * * * * * * * * * *  * * * * * * * * * *
                * * * * * * * * * *  * * * * * * * * * *  * * * * * * * * * *  * * * * * * * * * *  * * * * * * * * * *
                                         * * * * * * * *  * * * * * * * * * *  * * * * * * * * * *  * * * * * * * * * *
                                                            * * * * * * * * *  * * * * * * * * * *  * * * * * * * * *

alignment
position         1 5 1 . . . . . . .  1 6 1 . . . . . . .  1 7 1 . . . . . . .  1 8 1 . . . . . . .  1 9 1 . . . . . . .

P879005    149  A G A A t - - - - A  T C T C G C G G G A  T C T C A G C T G T  C C T T G G G C C C  c c c c c c t c a g
P879006     89  G G A A G C A A C A  T C T C G C G G G A  T G T C G G T G G T  C C T T G A G T C C  T T G G A C A G G -
P879007     86  A G A A G C A A C G  T C T C G C G G G A  T G T C G G T G G T  C C T T A A G T C C  T C G G A C G G G -
                * * * * * * * * * *  * * * * * * * * * *  * * * * * * * * * *  * * * * * * * * * *  * * * * * * * * *
                * * * * * * * * * *  * * * * * * * * * *  * * * * * * * * * *  * * * * * * * * * *  * * * * * * * * *
                * * * * * * * * * *  * * * * * * * * * *  * * * * * * * * * *  * * * * * * * * * *  * * * * * * * * *
                * * * *           *  * * * * * * * * * *  * * * * * * * * * *  * * * * * * *
                * * * *

alignment
position         2 0 1 . . . . . . .  2 1 1 . . . . . . .  2 2 1 . . . . . . .  2 3 1 . . . . . . .  2 4 1 . . . . . . .

P879005    195  c g c a A C T A C C  A A T C C C A A C A  T G C A A C G C G C  C C C G T G G C C C  A A G C G C C C G A
P879006    138  - - - - A C T A C A  A G G C C C A A C A  T G C A A T G C G C  C C C A A G G T C C  A A G C G G C C G A
P879007    135  - - - - A C T A C A  A A G C C C A A T A  T G C A A T G C G C  T C C A A G G T C C  A A G C G G C C G A
                        * * * * * *  * * * * * * * * * *  * * * * * * * * * *  * * * * * * * * * *  * * * * * * * * * *
                        * * * * * *  * * * * * * * * * *  * * * * * * * * * *  * * * * * * * * * *  * * * * * * * * * *
                        * * * * * *  * * * * * * * * * *  * * * * * * * * * *  * * * * * * * * * *  * * * * * * * * * *
                        * * * * * *  * * * * * * * *   *  * * * * * * * * * *  * * * * * * * * * *  * * * * * * * * * *
                        * * * * * *  * * * * * * * *   *  * * * * * * * * * *  * * * * * * * * * *  * * * * * * * * * *
```

```
                           * ********** ***  ***** ****  ****

alignment position  251.......  261.......  271.......  281.......  291.......

P879005 245  ATGAAGGCTC  GGGGCCAAAG  TCATGTGTCT  GACTGCGTCA  CACGTCCCGT
P879006 184  ATGAAGGCCT  GGAGCCCTTA  TCACGTGTCA  GCTTGCGTCA  AGCGGCCGGT
P879007 181  ATGAAGACTC  GGAGCCCACG  TTACGTGTCC  GCCTGCGTCA  AGCGGGCGGT
             **********  **********  **********  **********  **********
             **********  **********  **********  **********  **********
             **********  **********  **********  **********  **********
             ******      ******          ******  ******
             ******
             ******

alignment position  301.......  311.......  321.......  331.......  341.......

P879005 295  CATCTCTCCC  ACGCCTTTTT  CTTCAGCGTA  CACCTTGGTC  GCCATCTTGT
P879006 234  TGTCACTCCC  ACGCTTTTTT  CTTCCGCTTG  CACCTTGGTC  GCCATTTTGT
P879007 231  aGTCACTCCC  ACGCTTTTTT  CTTCCGCTTG  CACCTTGGTC  GCCATTTTGT
             **********  **********  **********  **********  **********
              *********  **********  **********  **********  **********
              *********  **********  **********  **********  **********
              *********  **********  **********  **********  **********
              *********  **********  **********  **********  **********
                  *****  **********  **********  **********  **********
                  *****  **********  **********  **********  *****
                              *****  **********  **********  *****

alignment position  351.......  361.......  371.......  381.......  391.......

P879005 345  GcgcgAGGTT  ATGGAACCTC  TGAGTCCCGC  CAACTCTATG  GTCGAGCGTT
P879006 284  GTTGAAGGTT  GTAGAACCTC  TGAGTCTCGT  CGACTCTATG  GTCAAGCATT
P879007 281  GTTGAAGGCT  GTAGAACCTC  TGAGTCTCGT  CGACTCTATG  GTCAAGCATT
             **********  **********  **********  **********  **********
             **********  **********  **********  **********  **********
             **********  **********  **********  **********  **********
             **********  **********  **********  **********  **********
             *    *****  **********  **********  **********  **********
             *    *****  **********  **********  **********  **********
                            *******  **********  **********  *** ******
                            *******  **********  **********  ***

alignment position  401.......  411.......  421.......  431.......  441.......

P879005 395  TCAGGGATCG  GCCAATCGTA  ATCCAGATCT  CGTAGGAAAG  CCCCGCCTCT
P879006 334  TCGGGATTCG  ACCAATCGTA  ACCCCGAACT  CGGAAAAAGA  ACCCGCCTTT
P879007 331  TCGGGATTCG  ACCAATCGTA  ATCACGATCT  CGGAAAAAGA  CCCCGCCTTT
             **********  **********  **********  **********  **********
             **********  **********  **********  **********  **********
             **********  **********  **********  **********  **********
             **********  **********  *   ******  **********  **********
             **********  **********  *   ******  **********  **********
             **     ***  **********  *   ******  **           *********
             **     ***  **********  *                        ******  *
                                                              ******  *
                                                                      *

alignment position  451.......  461.......  471.......  481.......  491.......
```

```
P879005 445 ATCCGCATGG AGGCGGGAAT TGCCACGAAG CTCCTGTGGA GGGAGAGGAA
P879006 384 ATCCGCAAGG AGGCGGGAAT TGCCACGAAG CTCCTGTGGA GAGAGAGGAA
P879007 381 ATCCGCAAGG AGGCGGGAAT TGCCACGAAG CTCCCGTGGA GAGAGAGGAA
            ********** ********** ********** ********** **********
            ********** ********** ********** ********** **********
            ********** ********** ********** ********** **********
            ********** ********** ********** ********** **********
            ********** ********** ********** ********** **********
            ********** ********** ********** ********** **********
            ********** ********** ********** **** ***** **********
            ********** ********** ********** ****
            ********** ********** ********** *
                          ******* ********** *

alignment
position    501....... 511....... 521....... 531....... 541.......

P879005 495 GCAGCTGCGG AAAGCCAATA AGAgtgggga atc-GATGAC GTCAACCAAT
P879006 434 GAAGCTGAAG AAGACCAATA AGAAGGATGG AGTTGATGAC GCCAGCCAAT
P879007 431 GAACCTGAAA AAGACCAATA AGAAAGATGA AGTTGATGAC GCCAGCCAAT
            ********** ********** ********** ********** **********
            ********** ********** ********** ********** **********
            ********** ********** ********** ********** **********
            *** ****** ********** ***            ****** **********
            *** ****** ********** *              ****** **********
            *** ****** ********** *              ****** ****
            ***

alignment
position    551....... 561....... 571....... 581....... 591.......

P879005 544 GGGGACGCGG GGATATTACG GCCAATGAGA ATGGAGaagg tccaggACAC
P879006 484 TGGAACGTGG AGAGATAACG ACCAATGGGA GTGaAGGGAC TCCGA-ACAC
P879007 481 TGGAACGTGA GGAGATAACG GCCAATGGGA GTGGAGGGAC CCCGA-ACAC
            ********** ********** ********** ********** ***** ****
            ********** ********** ********** *** ****** ***** ****
            ********** ********** ********** *** ****** ***** ****
            ********** ********** ****                        ****
            ********** ********** ****                        ****

alignment
position    601....... 611....... 621....... 631....... 641.......

P879005 594 GTGGGTGGGG GAaGCTGAGC GCTGAGACCA AGGGCTAAAG CTGGGAGGTC
P879006 533 GTGGGTGGGG GA-GCTGAGC GCTCAGACCA AGGGCTAAAG CTGGGAGGTC
P879007 530 GTGGGTGGGG GA-GCTGAGC GCTGAGACCA AGGGCTAAAG CTGCGAGGTC
            ********** ** ******* ********** ********** **********
            ********** ** ******* ********** ********** **********
            ********** ** ******* ********** ********** **********
            ********** ** ******* ********** ********** **********
            ********** ** ******* ********** ********** **********
                          ******* ********** ********** **********
                          ******* ********** ********** **********
                          ******* *** ****** ********** **********
                          ******* *** ****** ********** **********
                          ******* *** ****** ********** **********

alignment
position    651....... 661....... 671....... 681....... 691.......

P879005 644 AGTctGTCAC CTTGAGCCGG GCGAGCGCTG TGGGCCAAGC AGGGGtTGCA
P879006 582 AGTCAGTCAC CTTGAGCAGG GAGTGTGCTG TGGGCCGAGC AGGGGCTGCA
```

```
P879007   579 AGTCAGTCAC CTTGAGCAGG GAGTGTGCTG TGGGCCGAGC AGGGGCTGCA
              ********** ********** ********** ********** **********
              ********** ********** ********** ********** **********
              ********** ********** ********** ********** **********
              ********** ********** ********** ********** **********
              ***  ***** ********** ********** ********** ***** ****
              ***  ***** ********** ********** ********** ***** ****
              ***  ***** ********** ********** ********** ***** ****
              ***  ***** ********** ********** ********** ***** ****
              ***                                               ****
              ***

alignment
position      701....... 711....... 721....... 731....... 741.......

P879005   694 GGGTAGTAGG AGTGCAGGTG ACTTGGGGCC GGGAGccagc aataggcagC
P879006   632 GGGGAGTGGG AGTGCAGGTG ACTTGGGGCT GGGAGACAGG GgTCCGCAAC
P879007   629 GGGGAGTGGG AGTGCAGGTG ACTTGGGGCC CGGAGACAGG G-TCCGCTAC
              ********** ********** ********** ********** * ********
              ********** ********** ********** ********** * ********
              ********** ********** ********** ********** *        *
              ********** ********** ********** *****               *
              ********** ********** ********** *****
              ********** ********** ********** *****
              ********** ********** ********** *****
              ********** ********** ********** *****
              ****

alignment
position      751....... 761....... 771....... 781....... 791.......

P879005   744 GTTGTGGGCT GGGCAGTGTG GAGGGGTATT TCCCCCCAGt gctgggagag
P879006   682 GCCGTGGGTT GTGCCACGTG GAGCACCATT TGCCTCCAGG GATAGGGAAC
P879007   678 GCCGTGGGCT GTGCCATGTG GAGCAGCATT TGCCTCCAGG GATAGGGAAC
              ********** ********** ********** ********** **********
              ********** ********** ********** ********** **********
              ********** ********** ***** **** ********** **********
              ********** ********** ***** **** *********
                                          **** *********

alignment
position      801....... 811....... 821....... 831....... 841.......

P879005   794 gctatggcaa tctagatatg GCCTTATCAA TGAATGTTAA TTACCAACGC
P879006   732 GAGGACTAgA GTCTTTAA-- GCCTTATCAA TTAATGTTAA TTACCAACGC
P879007   728 GAGGACTAaA GTCTTTAA-- GCCTTATCAA TTAATGTTAA TTACCAACGC
              ******** * ********   ********** ********** **********
              ******** * ********   ********** ********** **********
              ******** * ********   ********** ********** **********
                       * ********   ********** ********** **********
                                    ********** ********** **********
                                    ********** ********** **********
                                    ********** ********** **********
                                    ********** ********** **********
                                    ********** ********** **********
                                    ********** ********** **********

alignment
position      851....... 861....... 871....... 881....... 891.......

P879005   844 TAATGAACGA ttgactgcct CGGCTGGGGC GGGTcGGGGA CTGCTGTGTA
P879006   780 TAATGAGCGA TAAACGG--- CCGCTGGGGA GAGTAGGGGA CAGCAGTGTA
```

```
P879007  776     TAATGAGCGA TAAACGG--- CCGCTGGGGA GAGTAGGGGA CAGCTATGTA
                 ********** *******    ********** ********** **********
                 ********** *******    ********** ********** **********
                 ********** *******    ********** ********** **********
                 ********** *******    ********** ********** **********
                 **********                            ***** ****  ****
                 **********                            ***** ****  ****
                 **********                            ***** ****  ****
                 **********                            ***** ****  ****
                 **********
                 **********

alignment
position         901....... 911....... 921....... 931....... 941.......

P879005  894     AGATGGGCAG GATCCTGCGC GACacctgcC CAGTATCCGC CTCCAGTCTT
P879006  827     ACTTGGGCAG GATCCTGCAC GACGTCTG-C CAGTTTCTGC CTCCGGTCTT
P879007  823     ACATGGGCAG GATCCTGCAC GACGTCTG-C CAGTTTCTGC CTCCAGTCTT
                 ********** ********** ******** * ********** **********
                 ********** ********** ******** * ********** **********
                 ********** ********** ******** * ********** **********
                 ********** ********** ******** * ********** **********
                 ********** ********** ***      * ********** **********
                 ********** ********** ***      * ********** **********
                 ********** ********** ***      * ********** **********
                 ********** ********** ***

alignment
position         951....... 961....... 971....... 981....... 991.......

P879005  944     CCCGAGAGAC CATTCCCTTT CGCAACATTT CCTTCCTGGG GCTTGGGCac
P879006  876     CCCGGGAGAC CATTCCTTTT CGCAACATTT CCCTCTTGGG GCCTGAGCCA
P879007  872     CCCGGGAGAT CATTCCTTTT CGCAACGTTT CCCTCTTGGG GCCTGAGCCA
                 ********** ********** ********** ********** **********
                 ********** ********** ********** ********** **********
                 ********** ********** ********** ********** **********
                 ********** ********** ****** *** ********** **********
                 ********** ********** ****** *** ********** ********
                 *********  ****** *** ****** *** ********** ********
                 *********  ******            *** ********** ********

alignment
position         1001...... 1011...... 1021...... 1031...... 1041......

P879005  994     atTTCCTTGA CTTCCGATCT CCATGACTTT GCTTtgaaga ccca------
P879006  926     CCTCCTTTTA TTTCCGATCT CGGTTGCTGT CCTTCTGCAG ACCCGCTGCC
P879007  922     CCTCCTTTTA CTTCCGTTCT TGATTGCTGT CCTTCTGCAG ACCCGCTGCC
                 ********** ********** ********** ********** **********
                 ********** ******        ******* ********** **********
                 ********** ******        ******* ********** **********
                 ********** ******        ******* ****

alignment
position         1051...... 1061...... 1071...... 1081...... 1091......

P879005 1038     ---------- ---------- ---CTGCTCT GTCCCTCTGA CCTCTAATGC
P879006  976     CAGAgAGTGG TTGACCCCGG AATCTGCTCT GTCCTTCTGA CCTCTAATGC
P879007  972     CAGAaAGTGG TTGACCCCGG AATCTGCTCT GTCCTTCTGA CCTCTAATGC
                 **** ***** ********** ********** ********** **********
                 **** ***** ********** ********** ********** **********
                 **** ***** ********** ********** ********** **********
                      ***** ********** ********** ********** **********
```

```
                                                   * * * * * * *  * * * * * * * * * *  * * * * * * * * * *
                                                   * * * * * * *  * * * * * * * * * *  * * * * * * * * * *
                                                   * * * * * * *  * * * * * * * * * *  * * * * * * * * * *
                                                   * * * * * * *  * * * * * * * * * *  * * * * * * * * * *
                                                   * * * * * * *  * * * * * * * * * *  * * * * * * * * * *
                                                   * * * * * * *  * * * * * * * * * *  * * * * *

alignment
position      1 1 0 1 . . . . . .  1 1 1 1 . . . . . .  1 1 2 1 . . . . . .  1 1 3 1 . . . . . .  1 1 4 1 . . . . . .

P879005 1065  C C T C C T C A G C  T G G g g c a c g g  t g c a A G G A A G  A G C G T g g - - -  - - - - - - - - - -
P879006 1026  C C T C C T T A G C  T G G G G T A T - -  - - - - A G G A A G  A G G G T T G G G G  G - G A A C G A T T
P879007 1022  C C T C C T T A G T  T G G A G T A T - -  - - - - A G G A A G  A G G G T T G G G G  G a G A A C G A T T
              * * * * * * * * * *  * * * * * * * *             * * * * * *  * * * * * * * * * *  *   * * * * * * * *
              * * * * * * * * * *  * * * * * * * *             * * * * * *  * * * * * * * * * *  *   * * * * * * * *
              * * * * * * * * * *  * * * * * * * *             * * * * * *  * * * * * * * * * *  *   * * * * * * * *
              * * * * * * * * * *  * * *
              * * * * * * * * * *  * * *
              * * * * * * * * * *  * * *
              * * * * * * * * * *  * * *
              * * * * * * * * * *  * * *
              * * * * * * * * * *  * * *

alignment
position      1 1 5 1 . . . . . .  1 1 6 1 . . . . . .  1 1 7 1 . . . . . .  1 1 8 1 . .

P879005 1102  - - - - - - - - - -  - - - - - - - - - -  - - - - - - - - - -  - - - - - -
P879006 1069  G G A G G T G G A T  G G G G A C A A C A  G G A G G T T G C C  A G t - - -
P879007 1066  G G A G G T G G A T  G G G A A C G A C A  G G A G G T T G C C  A G t a t c
              * * * * * * * * * *  * * * * * * * * * *  * * * * * * * * * *  * *
              * * * * * * * * * *  * * * * * * * * * *  * * * * * * * * * *  * *
              * * * * * * * * * *  * * * * * * * * * *  * * * * * * * * * *  * *
```

Pairwise similarities:

For each pairwise alignment, the similarity (relative to the maximum similarity) and the number of identical nucleic acids (in % of shorter sequence) is given. Maximum values are underlined.

	P879006 (1101 bp)	P879007 (1101 bp)
**P879005 (1101 bp)**	0.443<br>64 %	0.442<br>62 %
**P879006 (1101 bp)**		1.000<br>92 %

Please note that the **similarity value 1.000** marks only the two **most similar** sequences, it does not necessarily mean that these sequences are identical.

Extract alignment region	
**Positions in alignment**	from: to:
**Output file**	dialign.seq

Extract aligned sequences

For comments, questions or bug reports, please contact support@genomatix.de

© Genomatix Software GmbH 1998-2005
- All rights reserved.

License Agreement

GEMS Launcher 4.1.1

Genomatix-Logo | Logout | Personal | Messages | GEMS-Logo
Logout | Personal | Messages

Genomatix_Main GEMS ElDorado Gene2Promoter BiblioSphere
Frequently_asked_questions Your Results Your Sequences Your Protocol Help

GEMS Launcher Task: *DiAlign TF*: Multiple alignment plus TF sites working on C5-ATP5G1-HMRC (4 seq.)

[Alignment] [Pairwise similarities]

DiAlign professional TF Release 3.1 December 2004 **Mon May 30 23:24:49 2005**

Solution parameters:

**Sequence file:**	C5-ATP5G1-HMRC (4 seq.)
**Type of sequences:**	nucleotide sequences
**Threshold T:**	0.00
**'*' signs below alignment denote:**	diagonal similarity (max. similarity is represented by 10 '*' signs)
**Alignment output:**	complete alignment is shown<br># nucleic acids per line: 50
**TF output:**	common TF matches located in aligned regions (common to 4 (100.0 %) sequences)
**Family matches:**	yes
**MatInspector library:**	Matrix Family Library Version 5.0 (February 2005)
**Selected groups** (core/matrix sim):	• ALL user_defined.lib (0.75/Optimized)<br>• ALL vertebrates.lib (0.75/Optimized)

Aligned Sequences:

No.	Sequence Name	Sequence Description	Length
1	P879005	P879005 sym=ATP5G1|loc=Loc516|taxid=9606|spec=Homo sapiens|chr=17|ctg=NT_010783|str=(+)|start=5622641|end=5623941|len=1301|tss=801|comm=ATP synthase, H+ transporting, mitochondrial F0 complex, subunit c (subunit 9), isoform 1; (NM_005175/501/bronze;)	1301 bp
2	P879006	P879006 sym=Atp5g1|loc=Loc11951|taxid=10090|spec=Mus musculus|chr=11|ctg=NT_039521|str=(-)|start=7475581|end=7476881|len=1301|tss=801|comm=ATP synthase, H+ transporting, mitochondrial F0 complex, subunit c (subunit 9), isoform 1; (NM_007506/562/bronze;)	1301 bp
3	P879007	P879007 sym=Atp5g1|loc=Loc29754|taxid=10116|spec=Rattus norvegicus|chr=10|ctg=NW_047338|str=(-)|start=167149|end=168449|len=1301|tss=801|comm=ATP synthase, H+ transporting, mitochondrial F0 complex, subunit c, isoform 1; (NM_017311/565/bronze;)	1301 bp
4	cATP5G1	cATP5G1 canFam1_dna range=chr9:17785192-17786691 5'pad=0 3'pad=0 revComp=FALSE strand=? repeatMasking=none	1500 bp

Alignment (DiAlign format):

Please note that only **upper-case** letters are considered to be **aligned**. For more information, please have a look at the user guide.

V$STAF V$EGRF V$SF1F V$YY1F V$ECAT V$PAX6 V$NFKB V$ETSF V$PCAT V$EBOX V$HIFF V$OAZF
V$EKLF V$BARB V$RXRF U$EREF V$MYOD V$AREB V$RREB V$CLOX V$ATBF V$CART V$FAST V$LHXF
V$HOMF V$RBIT V$HNF1 V$NKXH V$PDX1 V$IKRS V$TEAF V$RORA V$HOXF

```
alignment position    1.........  11........  21........  31........  41........
P879005      1  ttacaggcac gcgccacca- ---------- ---------C CATGCCCGG
P879006      1  a--------- ---------- ---------- ---------- --GAAGC---
P879007      1  acaattgtct ggacctacgt tggctttttg acctaaactc atGAAAC---
cATP5G1      1  ttctttaatt tctatttttc tttagcggac tgcctattcC TATCCTTTG
                                                        * *********
                                                            *****

alignment position    51........  61........  71........  81........  91........
P879005     31  TAATTTTTGT ATtcttagta gagacggggt ttcaccatgc tggTCAGGC
P879006      7  ---------- ---------- ---------- ---------- ---TTAGTC
P879007     48  ---------- ---------- ---------- ---------- ---TTAGTC
cATP5G1     51  TCGTTTTTCT ATaggagt-- ---------- ---------- ----------
                ********** **                                  *******
                                                               *******

alignment position    101.......  111.......  121.......  131.......  141.......
P879005     81  GATCTCGAAC TCCTGACCTC AGGTGATC-- ---CGCCCGC ctcggcctcc
P879006     14  GGCCTTGAAC TCCTGACTTT CCTGTCTCct aagCACCTGC C---------
P879007     55  GGCTTTGAAC TCCTGACCTT CCGGTCTC-- ---CACCTGC C---------
cATP5G1     69  ---------- ---------- ---------- ---------- ----------
                ********** ********** ********      ******* *
                ********** ********** ********      *******

alignment position    151.......  161.......  171.......  181.......  191.......
P879005    126  caAAGTGCTG GGATTACAGG CGTGAGCCAC TGCACCCGGC ccaacaaat
P879006     55  --AAGTGCTA AAATTTCGGG TACGAAaTAG CAAGCCTGGT TTTAATTGT
P879007     91  --AAGTACTA AGATTTCAGG TATGAACTAG CAAGCCTGGC TTTAATTGT
cATP5G1     69  ---------- ---------- ---------- ---------- ----ATTAT
                  ******** ********** ********** ********** **********

alignment position    201.......  211.......  221.......  231.......  241.......
P879005    176  ttttagaaag cc-------- ---------- ---------- ATCTGGATG
P879006    103  TTTTGTTGAG TTTTTTTTTT CCACCATTCA ACT------- -------TG
P879007    139  ---------- --TTGTTGTT TCAACATTCA ATT------- -------TG
cATP5G1     75  TTACCCTTAG TTGGTTTTTT TTACAATAAA Aaaaaaaaaa ATCTGGCTG
                ********** ********** ********** ***        **********

alignment position    251.......  261.......  271.......  281.......  291.......
P879005    198  TAGTCCATGA CTGCTGAACA CCGGCTGAGG tccacaaaac tgCGCTCAC
P879006    139  GAGTCCATGT TGACTGACTA CTGACAGAcc caCAAGAGCA --CGCTCAC
P879007    163  GAGT------ ---------- ---------- --CAAGAGCA --CGCTCAC
```

```
cATP5G1  125 TAGTCCAGGG CTGCTGAGCT CTGCCCGAGG ccaacaaggc gctcgtccac
             ********** ********** **********   ********    *******
                                                            *******

alignment
position     301....... 311....... 321....... 331....... 341.......

P879005  248 ------GCTT GGGGACTTTC CAGCCGGCCT TTACTCTCCA t--GGAAAG
P879006  187 ------ACTT AGAAGTTTTC CAGCCTGCCT TAATACTTGG ACGAAAATG
P879007  183 ------ACTT AGAAAATTTC CAACCTGCCT TAATTCTTGG TCAGAAACG
cATP5G1  175 tagcgaACTT TCCAGGCCAG CAGGCGGCCT TTATTCTCCA aggcggaag
                   **** ********** ********** ********** **********
                   **** ****   **** ********** **********

alignment
position     351....... 361....... 371....... 381....... 391.......

P879005  290 G-GTCTTTCC ACAAACCTCC ACGGTGGGAA TTACTGCAAA GGCATCGGC
P879006  231 G-GG-TTTTT ACAAATCACG ATGGTGGAAG GTACTGTAAA GGCGTCGGC
P879007  227 G-GATTTTTT ACAAATCACT ATGGTGGAAT GTACTGTAAA GGCGTCGGC
cATP5G1  225 aaGG-GTTCC ACGAATCAGG AgagcgcGAA TTGCTGCAAA GGCGTTGGC
             *  ******** ********** ********** ********** **********
                  ***** ********** ********** ********** **********
                                     ******** ********** **********
                                          *** ********** **********
                                              ********* **********
                                                ******* *****

alignment
position     401....... 411....... 421....... 431....... 441.......

P879005  339 CTCCCGGCCT A-----GAA- ---TATCTCG CGGGATCTCA GCTGTCCTT
P879006  279 CTCCTTGCCA G-----GAAG CAACATCTCG CGGGATGTCG GTGGTCCTT
P879007  276 CTCCCTGCCA A-----GAAG CAACGTCTCG CGGGATGTCG GTGGTCCTT
cATP5G1  274 CTCCCGGCCT caggagGAA- ---CGTCTCG CGGGATCTCA GCCCTCCTT
             ********** *     **** ********** ********** **********
             ********** *     **** ********** ********** **********
             ********** *     ***     ******* ********** **********
             **********       ***      ****** ********** **********
             *********

alignment
position     451....... 461....... 471....... 481....... 491.......

P879005  380 GGCCCccccc ctcagcgCAA CTACCAATCC CAACATGCAA CGCGCCCCGT
P879006  324 AGTCCTTGGA CA-----GGA CTACAAGGCC CAACATGCAA TGCGCCCCAA
P879007  321 AGTCCTCGGA CG-----GGA CTACAAAGCC CAATATGCAA TGCGCTCCAA
cATP5G1  320 GGGCCTCTGA CC-----CGA CTACAAATCC CGTCATCCAA CGTGCCTGAA
             ********** **      *** ********** ********** **********
             ********** **      *** ********** ********** **********
             *****              *** ********** ********** **********
             *****                         ** ***  ****** **********
                                           ** *    ****** **********
                                                                **

alignment
position     501....... 511....... 521....... 531....... 541.......

P879005  430 GGCCCAAGCG CCCGAATGAA GGCTCGGGGC CAAAGTCATG TGTCTGACT
P879006  369 GGTCCAAGCG GCCGAATGAA GGCCTGGAGC CCTTATCACG TGTCAGCTT
P879007  366 GGTCCAAGCG GCCGAATGAA GACTCGGAGC CCACGTTACG TGTCCGCCT
cATP5G1  365 GGCCCAAGCG CCCGAATGAA GGCGCAGGTC CAAGGTCATG TGTCTGTCT
```

```
            ********** ********** ********** ********** **********
            ********** ********** ********** ********** **********
            ********** ********** ********** ********** **********
            ********** ********** *     ***** ********** **********
            ********** ********** *                  *  ******  ***
            ********** ********** *
                  ***    *******

alignment
position    551....... 561....... 571....... 581....... 591.......

P879005 480 CGTCACACGT CCCGTCATCT CTCCCACGCC TTTTT----- ----------
P879006 419 CGTCAAGCGG CCGGTTGTCA CTCCCACGCT TTTTT----- ----------
P879007 416 CGTCAAGCGG GCGGTAGTCA CTCCCACGCT TTTTT----- ----------
cATP5G1 415 CGTAAGGCGT CCGGTCATCT CTCCCACGCC TTTTTttttt tttttttttt
            ********** ********** ********** *****
            ********** ********** ********** *****
            ********** ********** ********** *****
            ********** *****  **** ********** *****
            *                 **** ********** *****
                                   ********** *****
                                   *********
                                   *********

alignment
position    601....... 611....... 621....... 631....... 641.......

P879005 515 --CTTCAGCG TACACCTTGG TCGCCATCTT GTGCGCGAGG TTATGGAACC
P879006 454 --CTTCCGCT TGCACCTTGG TCGCCATTTT GTGTTGAAGG TTGTAGAACC
P879007 451 --CTTCCGCT TGCACCTTGG TCGCCATTTT GTGTTGAAGG CTGTAGAACC
cATP5G1 465 ttCTGCAGCT TCCACCTTGG CCGCCATTTT GTGCGGGAGG TTCTGGAACC
              ******** ********** ********** ********** **********
              ******** ********** ********** ********** **********
              ******** ********** ********** ********** **********
              ******** ********** ********** ********** **********
              ******** ********** ********** ***    *** **********
              ******** ********** *******           *** **********
              ******** ********** *******                    *****
                   *** ********** *******                    *****

alignment
position    651....... 661....... 671....... 681....... 691.......

P879005 563 TCTGAGTCCC GCCAACTCTA TGGTCGAGCG TTTCAGGGAT CGGCCAATCC
P879006 502 TCTGAGTCTC GTCGACTCTA TGGTCAAGCA TTTCGGGATT CGACCAATCC
P879007 499 TCTGAGTCTC GTCGACTCTA TGGTCAAGCA TTTCGGGATT CGACCAATCC
cATP5G1 515 TCTGAGCCCC GCCGACTCTA TGGTTGAGCA TTTCAGGGAT CGACCAATCC
            ********** ********** ********** ********** **********
            ********** ********** ********** ********** **********
            ********** ********** ********** ********** **********
            ********** ********** ********** ********** **********
            ********** ********** ********** ********** **********
            ********** ********** *****  **** ********** **  *******
            ********** ********** ****   **** ****
            ********** ********** ****

alignment
position    701....... 711....... 721....... 731....... 741.......

P879005 613 TAATCCAGAT CTCGTAGGAA AGCCCCGCCT CTATCCGCAT GGAGGCGGGA
P879006 552 TAACCCCGAA CTCGGAAAAA GAACCCGCCT TTATCCGCAA GGAGGCGGGA
P879007 549 TAATCACGAT CTCGGAAAAA GACCCCGCCT TTATCCGCAA GGAGGCGGGA
```

```
cATP5G1  565 GAAGCCAGAT GTCGGAGGAA AACCCCGCCT TCGCCCGCAG GGAGGCGGGA
             ********** ********** ********** ********** **********
             ********** ********** ********** ********** **********
             ********** ********** ********** ********** **********
             ********** ********** ********** ********** **********
             ***   **** ******  ** ********** ********** **********
             ***                      *******  ********* **********
                                      ******   ********* **********
                                                   ****** **********

alignment
position     751....... 761....... 771....... 781....... 791.......

P879005  663 ATTGCCACGA AGCTCCTGTG GAGGGAGAGG AAGCAGCTGC GGAAAGCCAA
P879006  602 ATTGCCACGA AGCTCCTGTG GAGAGAGAGG AAGAAGCTGA AGAAGACCAA
P879007  599 ATTGCCACGA AGCTCCCGTG GAGAGAGAGG AAGAACCTGA AAAAGACCAA
cATP5G1  615 ACTGCCACGA AGCTCCTGTG GAGAGAGAGG AAGAAGCTGC CGAAAGCCAG
             ********** ********** ********** ********** **********
             ********** ********** ********** ********** **********
             ********** ********** ********** ********** **********
             ********** ********** ********** ********** **********
             ********** ********** ********** *********   *********
             ********** ********** ********** *********
             ********** ********** ********** *****  ***
             * ******** ********** *** ****** *****  ***
               ******** ****** *** ***
               ******** ******

alignment
position     801....... 811....... 821....... 831....... 841.......

P879005  713 TAAGAgt gg- GGAATCGATG ACGTCAACCA ATGGGGACGC GGGGATATTA
P879006  652 TAAGAAGGAT GGAGTTGATG ACGCCAGCCA ATTGGAACGT GGAGAGATAA
P879007  649 TAAGAAAGAT GAAGTTGATG ACGCCAGCCA ATTGGAACGT GAGGAGATAA
cATP5G1  665 TAAGAAAGGA GGACTTGAGG ACGTTAACCA ATGAGGATGC GGGAGGATTC
             ********** ********** ********** ********** **********
             ********** ********** ********** ********** **********
             ********** ********** ********** ********** **********
             *****            **** ********** ********** ***  *****
             ***              **** ********** *

alignment
position     851....... 861....... 871....... 881....... 891.......

P879005  762 CGGCCAATGA GAATGGAGAA GGTCCAGGAC ACGTGGGT-G GGGgaAGCTC
P879006  702 CGACCAATGG GAGTGAAGGG ACTCCGA-AC ACGTGGGTGG GGG--AGCTC
P879007  699 CGGCCAATGG GAGTGGAGGG ACCCCGA-AC ACGTGGGTGG GGG--AGCTC
cATP5G1  715 CAGCCAATGA GAGTGGGGGG ATCCCGGGAC ACGTGGGT-G GGG--AGCGC
             ********** ********** ********** ********** ***  *****
             ********** ********** ******* ** ********** ***  *****
             ********** *****  *** ******* ** ******** * ***  *****
             ******                                    * ***  *****
               ****                                    * ***  *****
                                                              ****
                                                              ****
                                                              ****

alignment
position     901....... 911....... 921....... 931....... 941.......

P879005  811 AGCGCTGAGA CCAAGGGCTA AAGCTGGGAG GTGAGTCTGT CACCTTGAGC
P879006  749 AGCGCTCAGA CCAAGGGCTA AAGCTGGGAG GTGAGTCAGT CACCTTGAGC
```

```
P879007   746 AGCGCTGAGA CCAAGGGCTA AAGCTGCGAG GTGAGTCAGT CACCTTGAG
cATP5G1   762 AGCGCTGAGA CCTAGAGCTA AAGCcGGGAG GTGAGTCAGT CACCTTGAG
              ********** ********** ********** ********** **********
              ********** ********** ********** ********** **********
              ********** ********** ********** ********** **********
              ********** ********** ********** ********** **********
              ********** ********** ********** ********** **********
              ********** ********** ********** ********** **********
              ********** ********** **** ***** ******   ** **********
              ******  *** ********** **** ***** ******   ** **********
                     *** ********** **** ***** ******
                                         *** ******

alignment
position      951....... 961....... 971....... 981....... 991.......

P879005   861 CGGGCGAGCG CTGTGGGCCA AGCAGGGGTT GCAGGGTAGT AGGAGTGCAC
P879006   799 AGGGAGTGTG CTGTGGGCCG AGCAGGGGCT GCAGGGGAGT GGGAGTGCAC
P879007   796 AGGGAGTGTG CTGTGGGCCG AGCAGGGGCT GCAGGGGAGT GGGAGTGCAC
cATP5G1   812 CGGGCAAGCG CCGTGgtcgg attAGGGGCC GCGGGGCTGT GGGAGTGCAC
              ********** ********** ********** ********** **********
              ********** ********** ********** ********** **********
              ********** ********** ********** ********** **********
              ********** ********** ********** ********** **********
              ********** ********** ********** ********** **********
              ********** *****         ******* ********** **********
              ****                     *****  * ********** **********
              ****                     *****  * ********** **********
                                              * *******

alignment
position      1001...... 1011...... 1021...... 1031...... 1041......

P879005   911 GTGACTTGGG GCCGGGAGCC AGCAATAGGC AGGGTTGTGG GCTGGGCAGT
P879006   849 GTGACTTGGG GCTGGGAGAC AGGGGTCCGC AAGGCCGTGG GTTGTGCCAC
P879007   846 GTGACTTGGG GCCCGGAGAC AGGG-TCCGC TAGGCCGTGG GCTGTGCCAT
cATP5G1   862 GTGACTTGGG GCCGGAAGCC AGCGATCGGC AGGGGTGTGG GCTGGGCCGT
              ********** ********** ********** ********** **********
              ********** ********** ********** ********** **********
              ********** ********** **** ***** ********** **********
              ********** ********               ******** **********
              ********** ********
              ********** ***   **
              ********** ***
              ********** **

alignment
position      1051...... 1061...... 1071...... 1081...... 1091......

P879005   961 GTGGAGGGGT ATTTCCCCCC AGTGCTGGGA GAGGCtatgg caatctagaT
P879006   899 GTGGAGCACC ATTTGCCTCC AGGGATAGGG AAGGAGGACT AgAGTCTTTA
P879007   895 GTGGAGCAGC ATTTGCCTCC AGGGATAGGG AAGGAGGACT AaAGTCTTTA
cATP5G1   912 GTGG-GGGGC ATTTGCCCCC AGGGCCGGGA GAAGCgctag gtgcc----T
              ********** ********** ********** ********** * ********
              ********** ********** ********** ********** * ********
              **** ***  * ********** ********** *****        ********
              ****      * ********** **

alignment
position      1101...... 1111...... 1121...... 1131...... 1141......

P879005  1011 ATGGCCTTAT CAATGAATGT TAATTACCAA CGGTAATGAA CGATTGACTC
```

```
P879006   949 A--GCCTTAT CAATTAATGT TAATTACCAA CGGTAATGAG CGATAAACG
P879007   945 A--GCCTTAT CAATTAATGT TAATTACCAA CGGTAATGAG CGATAAACG
cATP5G1   957 ATATCCTTAA CAATTATTGT TAATTACCAA --GTAATGAG CGATTAGCT
              ********** ********** ********** ********** **********
              *  ******* ********** ********** ********** **********
              *  ******* ********** ********** ********** **********
                 ******* ********** ********** ********** ***
                 ******* ********** ********** ********** ***
                 ******* ********** ********** ********** ***
                 ******* ********** **********
                  ****** ********** **********

alignment
position      1151...... 1161...... 1171...... 1181...... 1191......

P879005  1061 cctCGGCTGG GGCGGGTCGG GGACTGCTGT GTAAGATGGG CAGGATCCT
P879006   997 ---CCGCTGG GGAGAGTAGG GGACAGCAGT GTAACTTGGG CAGGATCCT
P879007   993 ---CCGCTGG GGAGAGTAGG GGACAGCTAT GTAACATGGG CAGGATCCT
cATP5G1  1005 tccccaccga GGCGGGCCGA GGACAGCTGT GTAAAATGGG CAGCACTCT
                 ******* ********** ********** ********** **********
                 ******* ********** ********** ********** **********
                 ******* ********** ********** ********** **********
                                 ** ********** ********** **********
                                 ** ********** ********** **********
                                 ** *******  * ********** **********
                                    *******  * ********** ***

alignment
position      1201...... 1211...... 1221...... 1231...... 1241......

P879005  1111 CGCGACACCT GCCCAGTATC CGCCTCC--- AGTCTTCCCG AGAGACCAT
P879006  1044 CACGACGTCT G-CCAGTTTC TGCCTCC--- GGTCTTCCCG GGAGACCAT
P879007  1040 CACGACGTCT G-CCAGTTTC TGCCTCC--- AGTCTTCCCG GGAGATCAT
cATP5G1  1055 CCCGGCACCG ACCCAGTATC TGCCTCCtaa AGCCTTCCCG GGAGACCAT
              ********** ********** *******    ********** **********
              ********** * ******** *******    ********** **********
              ********** * ******** *******    ********** **********
              ******       ******** *******    ********** **********
              ******       ******** *******    ********** **********
              ****                                ******* **********
                                                  ******* ***** ****

alignment
position      1251...... 1261...... 1271...... 1281...... 1291......

P879005  1158 CCCTTTCGCA ACATTTCCTT CCTGGGGCTT GGGCacatTT CCTTGACTT
P879006  1090 CCTTTTCGCA ACATTTCCCT CTTGGGGCCT GAGCCACCTC CTTTTATTT
P879007  1086 CCTTTTCGCA ACGTTTCCCT CTTGGGGCCT GAGCCACCTC CTTTTACTT
cATP5G1  1105 CTCTTTAGCA ACATTTCCTT CCTGGGGTTT GGGCCTCCTT CCTCTGCTG
              ********** ********** ********** ********** **********
              ********** ********** ********** ********** **********
              ********** ********** ********** *****   ** **********
              ********** ********** ********** *****
              ********** ********** ********** *****
              ********** ** *****
              **     *** ** *****

alignment
position      1301...... 1311...... 1321...... 1331...... 1341......

P879005  1208 CGATCTCCAT GACTTTGCTT -TGAAGACCC a--------- ---------
P879006  1140 CGATCTCGGT TGCTGTCCTT CTGCAGACCC GCTGCGCAGA gAGTGGTTG
```

```
P879007 1136 CGTTCTTGAT TGCTGTCCTT CTGCAGACCC GCTGCGCAGA aAGTGGTTGA
cATP5G1 1155 AGATCTACGT GACCTTCCTT -TGCAGACCC ctttc----- ----------
             ********** ********** ********** **********  *********
             **       * ********** ********** **********  *********
             **       * **********                        *********

alignment
position     1351...... 1361...... 1371...... 1381...... 1391......
P879005 1238 ---------C TGCTCTGTCC CTCTGACCTC TAATGCCCTC CTCAGCTGGC
P879006 1190 CCCCGGAATC TGCTCTGTCC TTCTGACCTC TAATGCCCTC CTTAGCTGGC
P879007 1186 CCCCGGAATC TGCTCTGTCC TTCTGACCTC TAATGCCCTC CTTAGTTGGA
cATP5G1 1189 ---------- -GCGCTGTTC CTCTGACCTC TAATGCCCTC CTCAGCTGGC
             ********** ********** ********** ********** **********
             ********** ********** ********** ********** **********
             ********** ********** ********** ********** **********
                      * ********** ********** ********** **********
                      * ********** ********** ********** **********
                      * ********** ********** ********** *********
                         ********* ********** ********** *********
                         ********* ********** ********** *********
                         ********* ********** *  ******* *********

alignment
position     1401...... 1411...... 1421...... 1431...... 1441......
P879005 1279 GCACGGTGCA AGGAAGAGCG TGG------- ---------- ----------
P879006 1240 GTAT------ AGGAAGAGGG TTGGGGG-GA ACGATTGGA- GGTGGATGGC
P879007 1236 GTAT------ AGGAAGAGGG TTGGGGGaGA ACGATTGGA- GGTGGATGGC
cATP5G1 1228 GCACGGTGCA AGGAAGAACG TGGtcccggg agggcgggcg GGGTGAAGGC
             ********** ********** ******* ** *********  **********
             ********** ********** ******* ** *********  **********
             ****       ********** ***
             ****       ********** ***
             *

alignment
position     1451...... 1461...... 1471...... 1481...... 1491......
P879005 1302 ---------- ---------- ---------- ---------- ----------
P879006 1282 GACAACAGGA GGTTGCCAGT ---------- ---------- ----------
P879007 1279 AACGACAGGA GGTTGCCAGT Atc------- ---------- ----------
cATP5G1 1278 GACGCCCGAA GGTCGCCACT Aagcaaacga ctaggctgtg gatgaatctt
             ********** ********** *
             ********** *********

alignment
position     1501...... 1511...... 1521...... 1531...... 1541......
P879005 1302 ---------- ---------- ---------- ---------- ----------
P879006 1302 ---------- ---------- ---------- ---------- ----------
P879007 1302 ---------- ---------- ---------- ---------- ----------
cATP5G1 1328 tttttttttt tttttttttc cctctgcaga ctgagaaaat gcagaccacc

alignment
position     1551...... 1561...... 1571...... 1581...... 1591......
P879005 1302 ---------- ---------- ---------- ---------- ----------
P879006 1302 ---------- ---------- ---------- ---------- ----------
P879007 1302 ---------- ---------- ---------- ---------- ----------
cATP5G1 1378 ggggcattac tcattcctcc ggctctggta agctgcggct ccggggtgat
```

alignment position		1601......	1611......	1621......	1631......	1641......
**P879005**	1302	----------	----------	----------	----------	----------
**P879006**	1302	----------	----------	----------	----------	----------
**P879007**	1302	----------	----------	----------	----------	----------
**cATP5G1**	1428	ctgtagtgcc	aggtgtggga	cctgtcgatg	tttaatgaat	gaactgagag

alignment position		1651......	1661......	1671
**P879005**	1302	----------	----------	---
**P879006**	1302	----------	----------	---
**P879007**	1302	----------	----------	---
**cATP5G1**	1478	gagcctggca	tccagaggat	gtt

Pairwise similarities:

For each pairwise alignment, the similarity (relative to the maximum similarity) and the number of identical nucleic acids (in % of shorter sequence) is given. Maximum values are underlined.

	**P879006 (1301 bp)**	**P879007 (1301 bp)**	**cATP5G1 (1500 bp)**
**P879005 (1301 bp)**	0.411<br>58 %	0.415<br>59 %	0.473<br>63 %
**P879006 (1301 bp)**		**1.000**<br>**89 %**	0.311<br>57 %
**P879007 (1301 bp)**			0.304<br>53 %

Please note that the **similarity value 1.000** marks only the two **most similar** sequences, it does not necessarily mean that these sequences are identical.

**Extract alignment region**	
**Positions in alignment**	from: to:
**Output file**	dialign.seq

Extract aligned sequences

For comments, questions or bug reports, please contact support@genomatix.de

---

© Genomatix Software GmbH 1998-2005 - All rights reserved. License Agreement GEMS Launcher 4.1.1

Genomatix-Logo Logout Personal Messages GEMS-Logo
Logout Personal Messages

Genomatix_MainGEMSElDoradoGene2Promoter BiblioSphere
Frequently_asked_questions Your ResultsYour SequencesYour ProtocolHelp

GEMS Launcher Task: *DiAlign TF*: Multiple alignment plus TF sites working on promoters.seq (4 seq.)

[Alignment] [Pairwise similarities]

DiAlign professional TF Release 3.1 December 2004 **Tue May 24 21:11:02 2005**

Solution parameters:

**Sequence file:**	promoters.seq (4 seq.)
**Type of sequences:**	nucleotide sequences
**Threshold T:**	0.00
**'*' signs below alignment denote:**	diagonal similarity (max. similarity is represented by 10 '*' signs)
**Alignment output:**	complete alignment is shown<br># nucleic acids per line: 50
**TF output:**	common TF matches located in aligned regions (common to 4 (100.0 %) sequences)
**Family matches:**	yes
**MatInspector library:**	Matrix Family Library Version 5.0 (February 2005)
**Selected groups** (core/matrix sim):	• ALL user_defined.lib (0.75/Optimized)<br>• ALL vertebrates.lib (0.75/Optimized)

Aligned Sequences:

No.	Sequence Name	Sequence Description	Length
1	P879005	sym=ATP5G1|loc=Loc516|taxid=9606|spec=Homo sapiens|chr=17|ctg=NT_010783|str=(+)|start=5622941|end=5623941|len=1001| tss=501| comm=ATP synthase, H+ transporting, mitochondrial F0 complex, subunit c (subunit 9), isoform 1; (NM_005175/501/bronze;)	1001 bp
2	P879006	sym=Atp5g1|loc=Loc11951|taxid=10090|spec=Mus musculus|chr=11|ctg=NT_039521|str=(-)|start=7475581|end=7476581| len=1001|tss=501| comm=ATP synthase, H+ transporting, mitochondrial F0 complex, subunit c (subunit 9), isoform 1; (NM_007506/562/bronze;)	1001 bp
3	P879007	sym=Atp5g1|loc=Loc29754|taxid=10116|spec=Rattus norvegicus|chr=10|ctg=NW_047338|str=(-)|start=167149|end=168149| len=1001|tss=501| comm=ATP synthase, H+ transporting, mitochondrial F0 complex, subunit c, isoform 1; (NM_017311/565/bronze;)	1001 bp
4	P879008	sym=na|loc=Loc419992|taxid=9031|spec=Gallus gallus|chr=27|ctg=NW_060665|str=(-)|start=111543|end=112543|len=1001| tss=501|comm=similar to P1 subunit; (XM_418114/501/silver;)	1001 bp

Alignment (DiAlign format):

Please note that only **upper-case** letters are considered to be **aligned**. For more information, please have a look at the user guide.

V$EGRF V$CREB V$ECAT V$PCAT

```
alignment
position        1.........  11........  21........  31........  41........

P879005     1   aaacctcca-  -------CGG  TGGGAATTAC  TGCAAAGGCa  tcggctctcc
P879006     1   ----------  ----------  ----------  ----------  ----------
P879007     1   ----------  ----------  ----------  ----------  ----------
P879008     1   cgctgggccg  ccggctcCGC  TGGGCAGGGC  TGGACAGGCc  gtgcttcaac
                                   ***  **********  *********

alignment
position        51........  61........  71........  81........  91........

P879005    43   cggcctagaa  tatcTCGCGG  GATCTCAGCT  GTCCTTGGGC  CCCCcccctc
P879006     1   ----------  ----TCGCGG  GATGTCGGTG  GTCCTTGAGT  CCTT------
P879007     1   ----------  -------CGG  GATGTCGGTG  GTCCTTAAGT  CCTC------
P879008    51   aaaacacaag  gacatgtttc  agtgttcgaa  gcgatctttt  ttaggagcga
                                ******  **********  **********  ****
                                   ***  **********  **********  ****
                                   ***  **********  **********  ****
                                   ***  **********  **********  *

alignment
position        101.......  111.......  121.......  131.......  141.......

P879005    93   agcgca----  -------ACT  ACCAATCCCA  ACATGCAACG  CGCCCCGTGG
P879006    31   ----------  GGACAGGACT  ACAAGGCCCA  ACATGCAATG  CGCCCCAAGG
P879007    28   ----------  GGACGGGACT  ACAAAGCCCA  ATATGCAATG  CGCTCCAAGG
P879008   101   atgggaaaag  GGAGAGGCCG  ACACGGCggc  cgcaacagcc  tccggcgcac
                            **********  **********  **********  **********
                            **********  **********  **********  **********
                            **********  **********  **********  **********
                                   ***  ***  *****  **********  **********
                                   ***  ***  *****  **********  **********
                                   ***  ***  *****  **********  ******  **

alignment
position        151.......  161.......  171.......  181.......  191.......

P879005   132   CCCAAGCGCC  CGAATGAAGG  CTCGGGGCCA  AAGTCATGTG  TCTGACTGCG
P879006    71   TCCAAGCGGC  CGAATGAAGG  CCTGGAGCCC  TTATCACGTG  TCAGCTTGCG
P879007    68   TCCAAGCGGC  CGAATGAAGA  CTCGGAGCCC  ACGTTACGTG  TCCGCCTGCG
P879008   151   cggagagtac  cgtgagagga  cagctgagca  cag-------  ----------
                **********  **********  **********  **********  **********
                **********  **********  **********  **********  **********
                **********  **********  **********  **********  **********
                **********  *****          ******          ***  *********
                **********  *****
                *******  *  *****

alignment
position        201.......  211.......  221.......  231.......  241.......

P879005   182   TCACACGTCC  CGTCATCTCT  CCCACGCCTT  TTTCTTCAGC  GTACACCTTG
P879006   121   TCAAGCGGCC  GGTTGTCACT  CCCACGCTTT  TTTCTTCCGC  TTGCACCTTG
P879007   118   TCAAGCGGGC  GGTaGTCACT  CCCACGCTTT  TTTCTTCCGC  TTGCACCTTG
P879008   184   ----------  ----------  CCCACGCTgc  taccgccg--  ----------
```

```
                 ********** ********** ********** ********** **********
                 ********** *** ****** ********** ********** **********
                 ********** *** ****** ********** ********** **********
                                ****** ********** ********** **********
                                ****** ********** ********** **********
                                    ** ********** ********** **********
                                    ** ********** ********** **********
                                    ** ******* ** ********** **********

alignment
position         251....... 261....... 271....... 281....... 291.......

P879005  232     GTCGCCATCT TGTGcgcgAG GTTATGGAAC CTCTGAGTCC CGCCAACTCT
P879006  171     GTCGCCATTT TGTGTTGAAG GTTGTAGAAC CTCTGAGTCT CGTCGACTCT
P879007  168     GTCGCCATTT TGTGTTGAAG GCTGTAGAAC CTCTGAGTCT CGTCGACTCT
P879008  202     ---------- ---------- ---------- ---------C CACCACCTCT
                 ********** ********** ********** ********** **********
                 ********** ********** ********** ********** **********
                 ********** ********** ********** ********** **********
                 ********** ********** ********** ********** **********
                 ********** ****    ** ********** ********** **********
                 ********** ****    ** ********** ********** **********
                 ********                     **** ********** **********
                 ********                     **** ********** **********

alignment
position         301....... 311....... 321....... 331....... 341.......

P879005  282     ATGGTCGAGC ----GTTTCA GGGATCGGCC AATCGTAATC CAGATCTCGT
P879006  221     ATGGTCAAGC ----ATTTCG GGATTCGACC AATCGTAACC CCGAACTCGG
P879007  218     ATGGTCAAGC ----ATTTCG GGATTCGACC AATCGTAATC ACGATCTCGG
P879008  213     GTGGTCAcct ctatGGTTCG AGGAGCGGCC ccacgcgcct catggtcctc
                 **********     ****** ********** ********** **********
                 **********     ****** ********** ********** **********
                 **********     ****** ********** ********** **********
                 **********     ****** ********** ********    *********
                 **********     ****** ********** ********    *********
                 **********     *****      ****** ********    ********
                 ****** ***     *****      ****** ********
                 ******

alignment
position         351....... 361....... 371....... 381....... 391.......

P879005  328     AGGAAAGCCC CGCCTCTATC CGCATGGAGG CGGGAATTGC CACGAAGCTC
P879006  267     AAAAAGAACC CGCCTTTATC CGCAAGGAGG CGGGAATTGC CACGAAGCTC
P879007  264     AAAAAGACCC CGCCTTTATC CGCAAGGAGG CGGGAATTGC CACGAAGCTC
P879008  263     tgggaaacc- ---------- ---------- ---------- ----------
                 ********** ********** ********** ********** **********
                 ********** ********** ********** ********** **********
                 ********** ********** ********** ********** **********
                 ********** ********** ********** ********** **********
                 ********** ********** ********** ********** **********
                         ** ********** ********** ********** **********
                         ** ****  **** ********** ********** **********
                         ** ****  **** ********** ********** **********
                                  **** ********** ********** ********
                                                  ********** ********

alignment
position         401....... 411....... 421....... 431....... 441.......
```

```
P879005 378 CTGTGGAGGG AGAGGAAGCA GCTGCGGAAA GCCAATAAGA gtggggaaTC
P879006 317 CTGTGGAGAG AGAGGAAGAA GCTGAAGAAG ACCAATAAGA AGGATGGAGT
P879007 314 CCGTGGAGAG AGAGGAAGAA CCTGAAAAAG ACCAATAAGA AAGATGAAGT
P879008 272 ---------- ---------- ---------- ---------- --------TC
            ********** ********** ********** ********** **********
            ********** ********** ********** ********** **********
            ********** ********** ********** ********** **********
            ********** **********  ********* **********
            ********** **********  ********* ********
            ********** **********  ********* ********
            *  ******** **********
            *

alignment
position    451....... 461....... 471....... 481....... 491.......

P879005 428 -GATGACGTC AACCAATGGG GACGCGGGGA TATTA----- ----------
P879006 367 TGATGACGCC AGCCAATTGG AACGTGGAGA GATAA----- ----------
P879007 364 TGATGACGCC AGCCAATTGG AACGTGAGGA GATAA----- ----------
P879008 274 -CAGGCCGTC AGCCAATCGC AAAGCGCGTA GCGAAaataa ctaaccaatg
            ********** ********** ********** *****
            ********** ********** ********** *****
            ********** ********** ********** *****
             ********* ********** ********** *****
             ********* ********** ********** *****
             ********* ********** ********** *****

alignment
position    501....... 511....... 521....... 531....... 541.......

P879005 462 ---------- ---------- ---------- ---------- ----------
P879006 402 ---------- ---------- ---------- ---------- ----------
P879007 399 ---------- ---------- ---------- ---------- ----------
P879008 323 gaagctcgca tcatggcacg gttctcccgc ccccgtgctg ctatcccgga

alignment
position    551....... 561....... 571....... 581....... 591.......

P879005 462 ---------- ---------- -------CGG CCAATGAGAA TGGAGaaggt
P879006 402 ---------- ---------- -------CGA CCAATGGGAG TGaAGGGACT
P879007 399 ---------- ---------- -------CGG CCAATGGGAG TGGAGGGACC
P879008 373 tgctagtggg aggagcttgc tggactgCGG CCAATGGGga gcgcggatgt
                                         *** ********** **********
                                         *** ********** ** *******
                                         *** ********** ** *******
                                         *** ***
                                         *** ***
                                         *** ***

alignment
position    601....... 611....... 621....... 631....... 641.......

P879005 485 ccaggACACG T--------- ---------- ---------- ----------
P879006 425 CCGA-ACACG T--------- ---------- ---------- ----------
P879007 422 CCGA-ACACG T--------- ---------- ---------- ----------
P879008 423 tgtcccagag tgccgcgcgc cgcgcgcagc ggtagccaat cggcgcggag
            **** ***** *
            **** ***** *
            **** ***** *
                 ***** *
```

```
                 ***** *

alignment
position    651....... 661....... 671....... 681....... 691.......

P879005 496 ---------- ---------- --------GG GTGGGGGAaG CTGAGCGCTG
P879006 435 ---------- ---------- --------GG GTGGGGGA-G CTGAGCGCTC
P879007 432 ---------- ---------- --------GG GTGGGGGA-G CTGAGCGCTG
P879008 473 gacgctgggg gacacggcaa ggtgagcgGG GTGGGGGC-G Cgggcgcgct
                                             ** ******** * **********
                                             ** ******** * **********
                                             ** ******** * **********
                                             ** ******** * **********
                                             ** ******** * **********
                                                                  * **********
                                                                  * **********
                                                                  * *********
                                                                  * *********
                                                                  * *********

alignment
position    701....... 711....... 721....... 731....... 741.......

P879005 518 AGACCAAGGG CTAAAGCTGG GAGGTGAGTc tGTCACCTTG AGC--CGGGC
P879006 456 AGACCAAGGG CTAAAGCTGG GAGGTGAGTC AGTCACCTTG AGC--AGGGA
P879007 453 AGACCAAGGG CTAAAGCTGC GAGGTGAGTC AGTCACCTTG AGC--AGGGA
P879008 522 ctctcttcac agccgtcacc gtcgcagccc cgagaggaca ccgtgCAGGT
            ********** ********** ********** ********** ***  *****
            ********** ********** ********** ********** ***  *****
            ********** ********** ********** ********** ***  *****
            ********** ********** ********** ********** ***  *****
            ********** ********** *********   ********* ***  *****
            ********** ********** *********   ********* ***  *****
            ********** ********** *********   ********* ***  *****
            ********** ********** *********   ********* ***  *****
            ********** ********** *********
            ********** ********** *********

alignment
position    751....... 761....... 771....... 781....... 791.......

P879005 566 GAGCGCTGTG GGCCAAGCAG GGGTTGCAGG GTAGTAGGAG TGCAGGTGAC
P879006 504 GTGTGCTGTG GGCCGAGCAG GGGCTGCAGG GGAGTGGGAG TGCAGGTGAC
P879007 501 GTGTGCTGTG GGCCGAGCAG GGGCTGCAGG GGAGTGGGAG TGCAGGTGAC
P879008 572 GGGGTCTGCG TGCGGCCCCG GGGCTGtgag caggcaccgt ga--------
            ********** ********** ********** ********** **********
            ********** ********** ********** ********** **********
            ********** ********** ********** ********** **********
            ********** ********** ********** ********** **********
            ********** ********** *** ****** ********** **********
            ********** ********** *** ****** ********** **********
            ********** ********** *** ****** ********** **********
            ********** ********** *** ****** ********** **********
                                      ****** **

alignment
position    801....... 811....... 821....... 831....... 841.......

P879005 616 TTGGGGCCGG GAGccagcaa taggcagGGT TGTGGGCTGG GCAGTGTGGA
P879006 554 TTGGGGCTGG GAGACAGGGg TCCGCAAGGC CGTGGGTTGT GCCACGTGGA
P879007 551 TTGGGGCCCG GAGACAGGG- TCCGCTAGGC CGTGGGCTGT GCCATGTGGA
P879008 614 ---------- ---------- ---------- ---------- ----------
```

```
                      ********** *********  ********** ********** **********
                      ********** *********  ********** ********** **********
                      ********** *********         *** ********** **********
                      ********** ***               *** ********** **********
                      ********** ***
                      ********** ***
                      ********** ***
                      ********** ***

alignment position    851....... 861....... 871....... 881....... 891.......
P879005          666  GGGGTATTTC CCCCCAGtgc tgggagaggc tatggcaatc tagatatgGC
P879006          604  GCACCATTTG CCTCCAGGGA TAGGGAAGGA GGACTAgAGT CTTTAA--GC
P879007          600  GCAGCATTTG CCTCCAGGGA TAGGGAAGGA GGACTAaAGT CTTTAA--GC
P879008          614  ---------- ---------- ---------- ---------- ----------
                      ********** ********** ********** ****** *** ******  **
                      ********** ********** ********** ****** *** ******  **
                      *** ****** ********** ********** ****** *** ******  **
                      *** ****** *******                      *** ******  **
                          ****** *******                                  **
                                                                          **
                                                                          **
                                                                          **
                                                                          **
                                                                          **

alignment position    901....... 911....... 921....... 931....... 941.......
P879005          716  CTTATCAATG AATGTTAATT ACCAACGGTA ATGAACGAtt gactgcctCG
P879006          652  CTTATCAATT AATGTTAATT ACCAACGGTA ATGAGCGATA AACGG---CC
P879007          648  CTTATCAATT AATGTTAATT ACCAACGGTA ATGAGCGATA AACGG---CC
P879008          614  ---------- ---------- ---------- ---------- ----------
                      ********** ********** ********** ********** *****   **
                      ********** ********** ********** ********** *****   **
                      ********** ********** ********** ********** *****   **
                      ********** ********** ********** ********** *****   **
                      ********** ********** ********** ********
                      ********** ********** ********** ********
                      ********** ********** ********** ********
                      ********** ********** ********** ********
                      ********** ********** ********** ********
                      ********** ********** ********** ********

alignment position    951....... 961....... 971....... 981....... 991.......
P879005          766  GCTGGGGCGG GTcGGGGACT GCTGTGTAAG ATGGGCAGGA TCCTGCGCGA
P879006          699  GCTGGGGAGA GTAGGGGACA GCAGTGTAAC TTGGGCAGGA TCCTGCACGA
P879007          695  GCTGGGGAGA GTAGGGGACA GCTATGTAAC ATGGGCAGGA TCCTGCACGA
P879008          614  ---------- ---------- ---------- ---------- ----------
                      ********** ********** ********** ********** **********
                      ********** ********** ********** ********** **********
                      ********** ********** ********** ********** **********
                      ********** ********** ********** ********** **********
                                    ******* **  ****** ********** **********
                                    ******* **  ****** ********** **********
                                    ******* **  ****** ********** **********
                                    ******* **  ****** ********** **********
```

```
alignment position 1001...... 1011...... 1021...... 1031...... 1041......

P879005 816 CacctgcCCA GTATCCGCCT CCAGTCTTCC CGAGAGACCA TTCCCTTTCG
P879006 749 CGTCTG-CCA GTTTCTGCCT CCGGTCTTCC CGGGAGACCA TTCCTTTTCG
P879007 745 CGTCTG-CCA GTTTCTGCCT CCAGTCTTCC CGGGAGATCA TTCCTTTTCG
P879008 614 ---------- ---------- ---------- ---------- ----------
            ****** *** ********** ********** ********** **********
            ****** *** ********** ********** ********** **********
            ****** *** ********** ********** ********** **********
            ****** *** ********** ********** ********** **********
            *      *** ********** ********** ********** **********
            *      *** ********** ********** ******* ** **** *****
            *      *** ********** ********** ******* ** ****
            *

alignment position 1051...... 1061...... 1071...... 1081...... 1091......

P879005 866 CAACATTTCC TTCCTGGGGC TTGGGCacat TTCCTTGACT TCCGATCTCC
P879006 798 CAACATTTCC CTCTTGGGGC CTGAGCCACC TCCTTTTATT TCCGATCTCG
P879007 794 CAACGTTTCC CTCTTGGGGC CTGAGCCACC TCCTTTTACT TCCGTTCTTG
P879008 614 ---------- --CCTGGGGC TTGGccggtg gggtggtgcc tggccgggct
            ********** ********** ********** ********** **********
            ********** ********** ********** ********** ****
            ********** ********** ********** ********** ****
            **** ***** ********** ********** ********** ****
            **** ***** ********** ******
            **** ***** ********** ******
                 ***** ********** ******

alignment position 1101...... 1111...... 1121...... 1131...... 1141......

P879005 916 ATGACTTTGC TTTGAAG--- ---------- ---------- ------ACCC
P879006 848 GTTGCTGTCC TTCTGCAGAC CCGCTGCGCA GAgAGTGGTT GACCCCGGAA
P879007 844 ATTGCTGTCC TTCTGCAGAC CCGCTGCGCA GAaAGTGGTT GACCCCGGAA
P879008 652 gtacc----C TTCGATG--- ---------- ---------- ------GGAC
            ********** ********** ********** ** ******* **********
             ********* ********** ********** ** ******* **********
             ********* ********** ********** ** ******* **********
             ********* **                       ******* **********

alignment position 1151...... 1161...... 1171...... 1181...... 1191......

P879005 937 ACTGCTCTGT CCCTCT---- ---------- ---------- ----------
P879006 898 TCTGCTCTGT CCTTCT---- ---------- ---------- ----------
P879007 894 TCTGCTCTGT CCTTCT---- ---------- ---------- ----------
P879008 669 TCTGCTGTCC CCTGCTccgg ccgggactcg gttgtgctga ggtggttttg
            ********** ******
            ********** ******
            ********** ******
            ********** ******
             ********* ******
             ********* ******
             ********* ******
             ********* ******
             ********* ******
             ********* ******
```

```
alignment
position     1201...... 1211...... 1221...... 1231...... 1241......
P879005  953 ---------- ---------- ---------- ---------- ----------
P879006  914 ---------- ---------- ---------- ---------- ----------
P879007  910 ---------- ---------- ---------- ---------- ----------
P879008  719 gggtgtttag aatgaggaag gggagaaccg cggatctcct gatctgcagg

alignment
position     1251...... 1261...... 1271...... 1281...... 1291......
P879005  953 ---------- ---------- ---------- ---------- ----------
P879006  914 ---------- ---------- ---------- ---------- ----------
P879007  910 ---------- ---------- ---------- ---------- ----------
P879008  769 ggtgggccgc gaggcggatg tagcagcact ggggttccta attccttctt

alignment
position     1301...... 1311...... 1321...... 1331...... 1341......
P879005  953 ---------- ---------- ---------- -----GACCT CTAATGCCCT
P879006  914 ---------- ---------- ---------- -----GACCT CTAATGCCCT
P879007  910 ---------- ---------- ---------- -----GACCT CTAATGCCCT
P879008  819 ctcatgatta gtaataggta aagcaagatg cagacGGCTT CTTTGGCTCT
                                                  ***** **********
                                                  ***** **********
                                                  ***** **********
                                                  ***** **********
                                                  ***** **********
                                                  ***** **********
                                                  ***** **********
                                                  ***** **********
                                                  ***** **********
                                                  ***** **

alignment
position     1351...... 1361...... 1371...... 1381...... 1391......
P879005  968 CCTCAGCTGG ggcacggtgc aAGGA----- ----AGAGCG Tgg-------
P879006  929 CCTTAGCTGG GGTAT----- -AGGA----- ----AGAGGG TTGGGGG-GA
P879007  925 CCTTAGTTGG AGTAT----- -AGGA----- ----AGAGGG TTGGGGGaGA
P879008  869 CCTCAGCTct ccggctctgg taagaactct gcagAGAGGT GTAGTGG-TG
             ********** *****       ****          ****** ******* **
             ********** *****       ****          ****** ******* **
             ********** *****       ****          ****** ******* **
             **********
             **********
             **********
             **********
             **********
             **********

alignment
position     1401...... 1411...... 1421...... 1431...... 1441......
P879005 1002 ---------- ---------- ---------- ---------- ----------
P879006  963 ACGATTGGAG GTGGATGGGG ACAACAGGAG GTTGCCAGt- ----------
P879007  960 ACGATTGGAG GTGGATGGGA ACGACAGGAG GTTGCCAGta tc--------
P879008  918 CTTAGTGGGG CTGTGTGGct gggatcagga cgtgactctg ggcagccctg
```

```
                     * * * * * * * * * *  * * * * * * * * * *  * * * * * * * * * *  * * * * * * * *
                     * * * * * * * * * *  * * * * * * * * * *  * * * * * * * * * *  * * * * * * * *
                     * * * * * * * * * *  * * * * * * * * * *  * * * * * * * * * *  * * * * * * * *
alignment
position             1 4 5 1 . . . . . .  1 4 6 1 . . . . . .  1 4 7 1 . . . . . .  1 4 8 1
P879005 1002         - - - - - - - - - -  - - - - - - - - - -  - - - - - - - - - -  - - - -
P879006 1002         - - - - - - - - - -  - - - - - - - - - -  - - - - - - - - - -  - - - -
P879007 1002         - - - - - - - - - -  - - - - - - - - - -  - - - - - - - - - -  - - - -
P879008  968         g t t g a g a g g g  g g t c c c a t g a  g g c c a t t t t a  c a c a
```

Pairwise similarities:

For each pairwise alignment, the similarity (relative to the maximum similarity) and the number of identical nucleic acids (in % of shorter sequence) is given. Maximum values are underlined.

	P879006 (1001 bp)	P879007 (1001 bp)	P879008 (1001 bp)
P879005 (1001 bp)	0.458<br>65 %	0.450<br>63 %	0.009<br>16 %
P879006 (1001 bp)		1.000<br>92 %	0.005<br>14 %
P879007 (1001 bp)			0.007<br>14 %

Please note that the **similarity value 1.000** marks only the two **most similar** sequences, it does not necessarily mean that these sequences are identical.

Extract alignment region	
Positions in alignment	from: to:
Output file	dialign.seq

Extract aligned sequences

For comments, questions or bug reports, please contact support@genomatix.de

© Genomatix Software GmbH 1998-2005 - All rights reserved.

License Agreement

GEMS Launcher 4.1.1

GEMS Launcher Task: *DiAlign TF*: Multiple alignment plus TF sites working on C5-ATP5G2 (3 seq.)

[Alignment] [Pairwise_similarities]

DiAlign professional TF Release 3.1 December 2004 **Tue May 31 00:27:39 2005**

Solution parameters:

**Sequence file:**	C5-ATP5G2 (3 seq.)
**Type of sequences:**	nucleotide sequences
**Threshold T:**	0.00
**'*' signs below alignment denote:**	diagonal similarity (max. similarity is represented by 10 '*' signs)
**Alignment output:**	complete alignment is shown<br># nucleic acids per line: 50
**TF output:**	common TF matches located in aligned regions (common to 3 (100.0 %) sequences)
**Family matches:**	yes
**MatInspector library:**	Matrix Family Library Version 5.0 (February 2005)
**Selected groups** (core/matrix sim):	• ALL user_defined.lib (0.75/Optimized)<br>• ALL vertebrates.lib (0.75/Optimized)

Aligned Sequences:

No.	Sequence Name	Sequence Description	Length
1	P879011	P879011 sym=ATP5G2|loc=Loc517|taxid=9606|spec=Homo sapiens|chr=12|ctg=NT_029419|str=(-)|start=16212672|end=16213672|len=1001|tss=501|comm=ATP synthase, H+ transporting, mitochondrial F0 complex, subunit c (subunit 9), isoform 2; (AK130971/501/gold;)	1001 bp
2	mSTP5G2	mSTP5G2 m6_dna range=chr15:102731087-102731883 5'pad=0 3'pad=0 revComp=TRUE strand=? repeatMasking=none	797 bp
3	P879015	P879015 sym=Atp5g2|loc=Loc171082|taxid=10116|spec=Rattus norvegicus|chr=7|ctg=NW_047785|str=(-)|start=631146|end=632146|len=1001|comm=ATP synthase, H+ transporting, mitochondrial F0 complex, subunit c (subunit 9), isoform 2; (CompGen promoter)	1001 bp

Alignment (DiAlign format):

Please note that only **upper-case** letters are considered to be **aligned**. For more information, please have a look at the user guide.

V$NRF1 U$NRF1 V$ETSF U$NRF2 V$ZBPF V$EVI1

```
alignment
position         1.........  11........  21........  31........  41.........

P879011      1   agc-------  ----------  ----------  ----------  ----------
mSTP5G2      1   tt--------  ----------  ----------  ----------  ----------
P879015      1   cgggactcgt  gcccggaagc  ccccgcccac  cgccaccggg  ggagggcgg

alignment
position         51........  61........  71........  81........  91........

P879011      4   ----------  ----------  ----------  ----------  ----------
mSTP5G2      3   ----------  ----------  ----------  ----------  ----------
P879015     51   gcgaaagggc  actgaagcaa  agcaggggct  tggggagagc  ccctcgcgg

alignment
position         101.......  111.......  121.......  131.......  141.......

P879011      4   ----------  ----------  ----------  ----------  ----------
mSTP5G2      3   ----------  ----------  ----------  ----------  ----------
P879015    101   cggctgcaca  ttccagcttg  caccccctt  tccccagcaa  gtctgacag

alignment
position         151.......  161.......  171.......  181.......  191.......

P879011      4   ----------  ----------  ----------  ----------  ----------
mSTP5G2      3   ----------  ----------  ----------  ----------  ----------
P879015    151   ggtaaactgg  tcgcctgggg  tgaccttgca  cccctactac  aggcaataa

alignment
position         201.......  211.......  221.......  231.......  241.......

P879011      4   ----------  ----------  ----------  ----------  ----------
mSTP5G2      3   ----------  ----------  ----------  ----------  ----------
P879015    201   tgtggttatt  cctaattgat  cccaggactc  acaaaaggga  gatacaggc

alignment
position         251.......  261.......  271.......  281.......  291.......

P879011      4   ----------  ----------  ----------  ----------  ----------
mSTP5G2      3   ----------  ----------  ----------  ----------  ----------
P879015    251   ttgtttaccc  tcaagtttct  gactcgcaga  tgttggggtt  ttttttttgt

alignment
position         301.......  311.......  321.......  331.......  341.......

P879011      4   ----------  ----------  ----------  ----------  ----------
mSTP5G2      3   ----------  ----------  ----------  ----------  ----------
P879015    301   ttgtttgttt  gttttgtttt  gtttttttc  atgctctgct  catcagcga

alignment
position         351.......  361.......  371.......  381.......  391.......

P879011      4   ----------  ----------  ----------  ----AGCTGC  GGCATTTTG
mSTP5G2      3   ----------  ----------  ----------  ----------  ----------
P879015    351   atagggaaaa  caagaaataa  gttaaagccg  aaatAGCCGT  GACATTTTG-
                                                         ******  *********
```

Pairwise similarities:

For each pairwise alignment, the similarity (relative to the maximum similarity) and the number of identical nucleic acids (in % of shorter sequence) is given. Maximum values are underlined.

	mSTP5G2 (797 bp)	P879015 (1001 bp)
P879011 (1001 bp)	0.714<br>59 %	0.435<br>34 %
mSTP5G2 (797 bp)		1.000<br>54 %

Please note that the **similarity value 1.000** marks only the two **most similar** sequences, it does not necessarily mean that these sequences are identical.

Extract alignment region	
**Positions in alignment**	from: to:
**Output file**	dialign.seq

Extract aligned sequences

For comments, questions or bug reports, please contact support@genomatix.de

© Genomatix Software GmbH 1998-2005 - All rights reserved.

License Agreement

GEMS Launcher 4.1.1

Genomatix-Logo Logout Personal Messages GEMS-Logo

Logout Personal Messages

Genomatix_MainGEMSElDoradoGene2PromoterBiblioSphere
Frequently_asked_questionsYour ResultsYour SequencesYour ProtocolHelp

GEMS Launcher Task: *DiAlign TF*: Multiple alignment plus TF sites working on promoters.seq (3 seq.)

[Alignment] [Pairwise similarities]

DiAlign professional TF Release 3.1 December 2004 **Tue May 31 22:39:23 2005**

Solution parameters:

**Sequence file:**	promoters.seq (3 seq.)
**Type of sequences:**	nucleotide sequences
**Threshold T:**	0.00
**'*' signs below alignment denote:**	diagonal similarity (max. similarity is represented by 10 '*' signs)
**Alignment output:**	complete alignment is shown<br># nucleic acids per line: 50
**TF output:**	common TF matches located in aligned regions (common to 3 (100.0 %) sequences)
**Family matches:**	yes
**MatInspector library:**	Matrix Family Library Version 5.0 (February 2005)
**Selected groups** (core/matrix sim):	• ALL user_defined.lib (0.75/Optimized)<br>• ALL vertebrates.lib (0.75/Optimized)

Aligned Sequences:

No.	Sequence Name	Sequence Description	Length
1	P978262	sym=ATP5G3|loc=Loc518|taxid=9606|spec=Homo sapiens|chr=2|ctg=NT_005403|str=(-)|start=26255279|end=26256279|len=1001| tss=501| comm=ATP synthase, H+ transporting, mitochondrial F0 complex, subunit c (subunit 9) isoform 3; (NM_001689/501/bronze;)	1001 bp
2	P978265	sym=Atp5g3|loc=Loc228033|taxid=10090|spec=Mus musculus|chr=2|ctg=NT_039207|str=(-)|start=14746017|end=14747017| len=1001| comm=ATP synthase, H+ transporting, mitochondrial F0 complex, subunit c (subunit 9), isoform 3; (CompGen promoter)	1001 bp
3	P978269	sym=Atp5g3|loc=Loc114630|taxid=10116|spec=Rattus norvegicus|chr=3|ctg=NW_047656|str=(-)|start=2899390|end=2900390| len=1001| comm=ATP synthase, H+ transporting, mitochondrial F0 complex, subunit c (subunit 9) isoform 3; (CompGen promoter)	1001 bp

Alignment (DiAlign format):

Please note that only **upper-case** letters are considered to be **aligned**. For more information, please have a look at the user guide.

V$HOXF V$ECAT V$HOXH V$MYT1 V$XBBF V$RORA V$EREF U$EREF V$SF1F V$DMTF V$NRF1 U$NRF1 V$EGRF V$ZBPF V$HESF

```
alignment
position          1.........  11........  21........  31........  41........

P978262       1   gctttgctgg  ggaccctagt  ttcctgttaa  gtagtgaatg  acccttcttt
P978265       1   tacag-----  ----------  ----------  ----------  ----------
P978269       1   ttaaaaaa--  ----------  ----------  ----------  ----------

alignment
position          51........  61........  71........  81........  91........

P978262      51   cctcccgcca  gccacagtcc  TATTTCACAG  AGGAAGAGAA  TGACCAGCAA
P978265       6   ----------  ----------  ----------  ----------  -------CCA
P978269       9   ----------  ----------  TCTATAACAA  GGCCAGAAGT  TGAACAACCA
                                          **********  **********  **********
                                                                         ***
                                                                         ***

alignment
position          101.......  111.......  121.......  131.......  141.......

P978262     101   GTCAACT---  ----------  ----------  ----------  ----------
P978265       9   TTTATCTGAG  ACATTTCTGC  ATCCATCTGG  TTTGTTCaaA  GTTATCAGAA
P978269      39   CTTATCTGAG  ATATTTCTGC  ATTCATCTGG  TTTGTTC--A  GTTACCAGAA
                  **********  **********  **********  *******  *  **********
                  **********  **********  **********  *******
                  **********  **********  **********  *******

alignment
position          151.......  161.......  171.......  181.......  191.......

P978262     108   ----------  ----------  ----------  ----------  ----------
P978265      59   CTGGgtgctg  aatagATTTT  GAAAACTATT  CAGGCACTTT  TTtAGTCCGA
P978269      87   CTGGtgctga  -----ATTTT  GAAAAATATT  CAGGCACTTT  TT-AGTCCAA
                  ****             *****  **********  **********  ** *******
                                   *****  **********  **********  ** *******
                                                                     *******

alignment
position          201.......  211.......  221.......  231.......  241.......

P978262     108   ----------  ----------  ----------  ----------  ----------
P978265     109   AATGATGATA  CATTATTATC  TCTTAATTCT  cCCAGTTATA  ATGGTGGAAG
P978269     131   AATTATGATA  CATTATAATC  TCTTAATCCT  tCCAATTATA  ATGGTGGAAG
                  **********  **********  **********   *********  **********
                  **********  **********  **********   *********  **********
                  **********  **********  **********   *********  **********

alignment
position          251.......  261.......  271.......  281.......  291.......

P978262     108   ----------  ----------  ----------  ----------  ----------
P978265     159   GCAGCAGAAC  ACAGTggtAC  ATGGCTTTAA  TGTCAGCACT  TGGAAaACAG
P978269     181   GCAGCAGAAC  ACAGTagcAC  ATGGCTTTAA  TCGCAGCACT  TGCAAgACAG
                  **********  *****   **  **********  **********  ***** ****
                  **********  *****   **  **********  **********  ***** ****
                  **********  *****                                     ****

alignment
position          301.......  311.......  321.......  331.......  341.......
```

```
P978262  108 ---------- ---------- ---------- ----TCTACT ACAGAGAAGC
P978265  209 AGGCAGGAGG ATTTCTGTGA GTTGAAGGCT AGCCTagTCT ACAGAGTTGC
P978269  231 AGGCAGGAAG ATTTCTGTGA GTTGGAGGCT AGTCTGTTCT ACAGAGTTGC
             ********** ********** ********** ********** **********
             ********** ********** ********** *****   *** **********
             ********** ********** ********** *****   *** **********
                                                      *** **********

alignment
position     351....... 361....... 371....... 381....... 391.......

P978262  124 Agcaactgat a--------- ---------- ---------- ----------
P978265  259 AAAGCTCTTG AACCGTCATT TGTTCttccc aggaCTTCTA GGTAGGTCTT
P978269  281 AAAGCTCTTG AACCCTCATT TGTTCatttc agggCTTCTA GTCAGGTCTT
             ********** ********** *****          ****** **********
             ********** ********** *****          ****** **********
             ********** ********** *****
             ********** ********** *****

alignment
position     401....... 411....... 421....... 431....... 441.......

P978262  135 ---------- ---------- ---------- ---------- ----------
P978265  309 CTGAGAAAAG ATTTGTCGTC TTaaacaaaa gcaaacaaac aaacaaacaa
P978269  331 CTGAGGAAAT ATTTGCCGTC TT-------- ---------- ----------
             ********** ********** **
             ********** ********** **

alignment
position     451....... 461....... 471....... 481....... 491.......

P978262  135 ---------- ---------- ---------- ---------- ----------
P978265  359 aaaaccctgT TTTAGAGTCT TACAAAGAAA TAATCCCGCT GGAGTTGGTt
P978269  353 ---------T TTTAGGTTCT TATAAAGAAA TAATTCCGCT GGACCTGGTg
                      * ********** ********** ********** *********
                      * ********** ********** ********** *********

alignment
position     501....... 511....... 521....... 531....... 541.......

P978262  135 ---------- ---------- ---------- ---------- ----------
P978265  409 atgaaga--- --------AG CTAACTACTT GGAAGGGTGA AAGTGGACGA
P978269  394 gtgaagacct gaaatcctAG CTCGCTACTT GGAAAGCTAA AACTGGACGA
                                ** ********** ********** **********
                                ** ********** ********** **********

alignment
position     551....... 561....... 571....... 581....... 591.......

P978262  135 ---------- ---------- ---------- ---------- ----------
P978265  448 TCAAggttCT CCCTAACTTA CATACTGAGC TCAAGGCCTG CctGAGCAAC
P978269  444 TCAAagccCT CTCTAACTTA CATACTGAGC ACAAGGCCTG CtcGAGCAAC
             ****    ** ********** ********** ********** *   *******
             ****    ** ********** ********** ********** *   *******
                     ** ********** ********** ********** *

alignment
position     601....... 611....... 621....... 631....... 641.......

P978262  135 ---------- ------AAAG GCCAAATCtt acaaggtgcc aacgtg----
P978265  498 TCACCCTGTT TGAAATAAAA GCCAAATCAG GGTCGGCTTG TAGCTCCCTG
P978269  494 TCACCCTGTC TGAAATAAAA GtgAAAACAG GGTCGGCTTG CAGCTCTGTG
```

```
                ********** ********** ********** ********** **********
                ********** ********** *   ******* ********** **********

alignment
position        651....... 661....... 671....... 681....... 691.......

P978262    165  ---------- ---------- -----AAGAA AAAGGCCCAC TATCCtgcag
P978265    548  GGAGaacCCC TGCCTGGCAC GTGCAAGGAG CGAGGCTCAG TctgcgATAG
P978269    544  GTAGtatCCC TGCCTGGCAC GTGCAAGGAG AGAGGCTCAG TACCCaATAG
                ****   *** ********** ********** ********** *****  ****
                ****   *** ********** ********** ********** *      ****
                       *** ********** ********** ********** *

alignment
position        701....... 711....... 721....... 731....... 741.......

P978262    190  cCAACTCATA TCAAAAGTTA atctgtttag tcctctctgc cggggtgaat
P978265    598  -CGACAACAA CTAAAAATTA TCTGCACCGT TAGTaaCACT AACAAAGCCA
P978269    594  -CGACACAAA TTAAAATTTA TCTTCACCGT TAGTggCACT AACAATGCCA
                 ********* ********** ********** ****  **** **********
                 ********* ********** ********** ****  **** **********

alignment
position        751....... 761....... 771....... 781....... 791.......

P978262    240  ctacagtttt tctttTTGTC TCCCTCTTGC CCCATCACCa ----------
P978265    647  AGAG------ -----TCGTC TTCAGTGTG- --CTTTTCAT TTCACTGGCC
P978269    643  GGAG------ -----TTGTC TCCAGTGTGC TCCTTTTCCT TTCACTGGCT
                ****            ***** ********** ********** **********
                ****            ***** *********    ******** **********
                                                   ******** **********

alignment
position        801....... 811....... 821....... 831....... 841.......

P978262    280  ---------- -------GGT GGTTGTCGTT GTCCTTCCCG GCTAGTTgCC
P978265    683  GCCAGCCGGT CACGGAGGGG GCCTGTCATT GTCCTCCTCT GAGAGTTACC
P978269    682  GCCAGCCTGT TACGGAGGG- -CCTGTCATT GTCCTCCTCT GCTGGTTACC
                ********** ********** ********** ********** **********
                ********** *********   ********* ********** **********
                ********** *********   ********* ********** **********
                                          ******* ********** *******   **
                                                                       **
                                                                       **
                                                                       **
                                                                       **
                                                                       **

alignment
position        851....... 861....... 871....... 881....... 891.......

P978262    313  AATAAAGTTG TTACAAAGTG ACCTTGAGTG TCTTCCttgg tgcacccgaa
P978265    733  AATAAAGTTG TTACAAAGTG ACCTTGAGCG TCTTCC---- ----------
P978269    730  AATAAAGTTG TTACAAAGTG ACCTTGAGCG TCTTCC---- ----------
                ********** ********** ********** ******
                ********** ********** ********** ******
                ********** ********** ********** ******
                ********** ********** ********** ******
                ********** ********** ********** ******
                ********** ********** ********** ******
                ********** ********** ********** ******
                ********** ********** ********** ******
                ********** ********** ********** ******
```

```
                         ********** ********** ******

alignment position
             901....... 911....... 921....... 931....... 941.......
P978262  363 acCCCGCCTT CTTCATCCGG GTGCTGCGGC GCGAATAAGA GCCGGACCGC
P978265  769 --CCCGCCTT CTCCATCCGG GTGCTGCGGC GCGGATAAGA GCCGCACCGC
P978269  766 --CCCGCCTT CTCCATCCGG GTGCTGCGGC ACGGATAAGA GCCGCACCGC
               ******** ********** ********** ********** **********
               ******** ********** ********** ********** **********
               ******** ********** ********** ********** **********
               ******** ********** ********** ********** **********
               ******** ********** ********** ********** **********
               ******** ********** ********** ********** **   *****
               ******** ********** ********** ********** **   *****
               ******** ********** ********** *** ****** **
               ******** ********** ********** ***

alignment position
             951....... 961....... 971....... 981....... 991.......
P978262  413 GCTTGCGCAT TGAGTCCCAC TCCTTCGACC TCTGCCGCAG CCCGT---GC
P978265  817 GCTTGCGCgC CCAGCCCCAC TGCTTCGACC TCTGCCGCCG CCACT---GC
P978269  814 GCTTGCGCAC CCAGCCCCAC TGCTTCGACC TCTGCCGCCG CtgctgccGC
             ********** ********** ********** ********** *****   **
             ********** ********** ********** ********** *****   **
             ********** ********** ********** ********** *****   **
             ******** * ********** ********** ********** *       **
             *******  * ********** ********** ********** *       **
             *******         ***** ********** ********** *       **
             *******         ***** ********** *****
                             ***** ********** *****

alignment position
             1001...... 1011...... 1021...... 1031...... 1041......
P978262  460 CGCCGCCGCC tcct----GG GAAGGTAAGT GGAGGGCACA GGgagcccgg
P978265  864 CGCCGCCTCG GGAGGTAAGC GAAGGTAAAC GGAGGGCGCG GGgc------
P978269  864 CGCCGCCTCC GGAGGTAAGC GGAGGTAAAC GGAGGGCGcg ----------
             ********** ********** ********** ********** **
             ********** ********** ********** ********
             ********** ********** ********** ********
             **********         ** ********** ********
             *******
             *******

alignment position
             1051...... 1061...... 1071...... 1081...... 1091......
P978262  506 CCTGGCCAGG GCGGAGCGGc gcccagTAGG GACCCATTCA TTGTGCCGGC
P978265  908 CCGGGCCGGG GCGGAGCGGG TCT---CCCG GCCCTGTCCA CCGGGCTTGC
P978269  904 --GGGCCGGG GCGGAGCGGG TCT---TCGG GCCCTGTCCA CTAGACTTGT
             ********** ********** ***   **** ********** **********
               ******** ********** ***   **** ********** *
               ******** ********** ***   **** ********** *
                ******* *********

alignment position
             1101...... 1111...... 1121...... 1131...... 1141......
P978262  556 GCCTCACTGG gcacgGGGCC CAAGCTGACG GCGTGCACGG GAGCCTGCGG
P978265  955 GCTTCCCGGG CAC--GCGGC CACGCTGACC GCCTGCGCGG GGCCCCTCG-
P978269  949 GACTCCCGGG CAC--GCGGC CACGCCGACG GTCTGCGCGG GTCCCCTCGG
```

```
                   ********** ***  ***** ********** ********** **********
                      ******* ***  ***** ********** ********** *********
                      ******* ***  ***** ********** ********** *****
                                     *** ********** ********** *****
alignment position 1151...... 1161...... 1171...... 1181...... 1191......
P978262   606      GGCCtgggtg ggaagaacag gcccctggag ggcacttgac cttaagcctc
P978265  1002      ---------- ---------- ---------- ---------- ----------
P978269   997      GGCCg----- ---------- ---------- ---------- ----------
                   ****
alignment position 1201...... 1211...... 1221...... 1231...... 1241......
P978262   656      ttttcctccg cagagaggaa gcgggagagg agcccacgtc gcctgtcacc
P978265  1002      ---------- ---------- ---------- ---------- ----------
P978269  1002      ---------- ---------- ---------- ---------- ----------

alignment position 1251...... 1261...... 1271...... 1281...... 1291......
P978262   706      caatatctcc agccgcgcag tcccgaagag tgtaagatgt tcgcctgcgc
P978265  1002      ---------- ---------- ---------- ---------- ----------
P978269  1002      ---------- ---------- ---------- ---------- ----------

alignment position 1301...... 1311...... 1321...... 1331...... 1341......
P978262   756      caagctcgcc tgcaccccct ctctggtgcg tagcccaggc agggtcgggg
P978265  1002      ---------- ---------- ---------- ---------- ----------
P978269  1002      ---------- ---------- ---------- ---------- ----------

alignment position 1351...... 1361...... 1371...... 1381...... 1391......
P978262   806      ggattgaggg gtgggcgttt tggcctagag cgtccacgtt gttgaatggg
P978265  1002      ---------- ---------- ---------- ---------- ----------
P978269  1002      ---------- ---------- ---------- ---------- ----------

alignment position 1401...... 1411...... 1421...... 1431...... 1441......
P978262   856      gcaaccttcg ggcggggcac accgccctct cgggaacggc tttctcctta
P978265  1002      ---------- ---------- ---------- ---------- ----------
P978269  1002      ---------- ---------- ---------- ---------- ----------

alignment position 1451...... 1461...... 1471...... 1481...... 1491......
P978262   906      cctattaacc cattgccgtt ttcttcctaa tcttagggca tctcagccct
P978265  1002      ---------- ---------- ---------- ---------- ----------
P978269  1002      ---------- ---------- ---------- ---------- ----------

alignment position 1501...... 1511...... 1521...... 1531...... 1541..
P978262   956      ctccttgcca ctagggcgag gcagagcagc cgaggcctag ctgtca
P978265  1002      ---------- ---------- ---------- ---------- ------
```

P978269 1002 - - - - - - - - - - - - - - - - - - - - - - - - - - - - - - - - - - - - - - - - - - - - - - - - -

Pairwise similarities:

For each pairwise alignment, the similarity (relative to the maximum similarity) and the number of identical nucleic acids (in % of shorter sequence) is given. Maximum values are underlined.

	**P978265 (1001 bp)**	**P978269 (1001 bp)**
**P978262 (1001 bp)**	0.194<br>27 %	0.193<br>28 %
**P978265 (1001 bp)**		**1.000**<br>80 %

Please note that the **similarity value 1.000** marks only the two **most similar** sequences, it does not necessarily mean that these sequences are identical.

**Extract alignment region**	
**Positions in alignment**	from: to:
**Output file**	dialign.seq

Extract aligned sequences

For comments, questions or bug reports, please contact support@genomatix.de

© Genomatix Software GmbH 1998-2005 - All rights reserved. License Agreement GEMS Launcher 4.1.1

Genomatix-Logo Logout Personal Messages GEMS-Logo

Logout Personal Messages

Genomatix_MainGEMSElDorado Gene2PromoterBiblioSphere
Frequently_asked_questionsYour ResultsYour SequencesYour Protocol Help

GEMS Launcher Task: *DiAlign TF*: Multiple alignment plus TF sites working on C5-ATP5H (3 seq.)

[Alignment] [Pairwise similarities]

DiAlign professional TF Release 3.1 December 2004 **Tue May 31 23:42:02 2005**

Solution parameters:

**Sequence file:**	C5-ATP5H (3 seq.)
**Type of sequences:**	nucleotide sequences
**Threshold T:**	0.00
**'*' signs below alignment denote:**	diagonal similarity (max. similarity is represented by 10 '*' signs)
**Alignment output:**	complete alignment is shown<br># nucleic acids per line: 50
**TF output:**	common TF matches located in aligned regions (common to 3 (100.0 %) sequences)
**Family matches:**	yes
**MatInspector library:**	Matrix Family Library Version 5.0 (February 2005)
**Selected groups** (core/matrix sim):	• ALL user_defined.lib (0.75/Optimized)<br>• ALL vertebrates.lib (0.75/Optimized)

Aligned Sequences:

No.	Sequence Name	Sequence Description	Le[illegible]
1	P880080	P880080 sym=ATP5H|loc=Loc10476|taxid=9606|spec=Homo sapiens|chr=17|ctg=NT_010641|str=(-)|start=6968827|end=6969827|len=1001|tss=501|comm=ATP synthase, H+ transporting, mitochondrial F0 complex, subunit d; (NM_006356/501/silver;)	10[illegible]
2	P880083	P880083 sym=Atp5h|loc=Loc71679|taxid=10090|spec=Mus musculus|chr=11|ctg=NT_039521|str=(-)|start=26819896|end=26820902|len=1007|tss=501,507|comm=ATP synthase, H+ transporting, mitochondrial F0 complex, subunit d; (AK088617/507/gold;AK002376/501/gold;)	10[illegible]
3	rATP5H	rATP5H n3_dna range=chr10:105513408-105514236 5'pad=0 3'pad=0 revComp=TRUE strand=? repeatMasking=none	8[illegible]

Alignment (DiAlign format):

Please note that only **upper-case** letters are considered to be **aligned**. For more information, please have a look at the user guide.

V$PLAG V$EGRF V$MAZF V$ZBPF V$SP1F V$HEN1 V$YY1F V$AP2F V$ETSF U$NRF2 V$MOKF V$VMYB

```
alignment
position          1......... 11........ 21........ 31........ 41........

P880080      1 c--------- ---------- ---------- ---------- ---GCGCCGC
P880083      1 cacgaagtag gtaccgccca cgttgagccg gacccagcgg gcgGCCCCGC
rATP5H       1 ---------- ---------- ---------- ---------- ----------
                                                              *******
                                                              *******

alignment
position          51........ 61........ 71........ 81........ 91........

P880080      9 CGCCCCCTGC CCGCTCGGGT GGTCCGGGAC CcGGCTCCAG CGGCTGCGCG
P880083     51 CGCCTCCCGT CCGCTCCGGA GGTCCGGGCC CgGGCTCCAG CGGCTGCGCG
rATP5H       1 ---------- ---------- ---------- ---------- ----------
               ********** ********** ********** * ******** **********
               ********** ********** ********** * ******** **********

alignment
position          101....... 111....... 121....... 131....... 141.......

P880080     59 ACGGCGGCag ccgGGCGGCC GTGCCCGCGG GGCGTGGGGC CAGCCGGGCG
P880083    101 GCGGCGGCgg gctGGCGGCC GTGTCCGCGG GGTGTCGGGC CCGCAGGGCG
rATP5H       1 ---------- ---------- ---------- ---------- ----------
               ********      ******* ********** ********** **********
               ********      ******* ********** ********** **********

alignment
position          151....... 161....... 171....... 181....... 191.......

P880080    109 tGGGCTGGGG GGCCCGCGGA CTGGGCCACC CCCGTCGCCC AcccCACTCC
P880083    151 aGGGCTGGGG GGTCCGCGGC CCGAGCCGCC GCCGTCGCCC AtcgCACTGC
rATP5H       1 ---------- ---------- ---------- ---------- ----------
                ********* ********** ********** ********** *   ******
                ********* ********** ********** ********** *   ******

alignment
position          201....... 211....... 221....... 231....... 241.......

P880080    159 CGCCGCCCCC TCCCAGCCCC GCCATcGCCG GGTCCAGCTG CAGTTCCGCC
P880083    201 TGCCACCCCC TCCCAGCCCC GCCAcTGCCG GGTCCAGCTG CAGCTCCGCC
rATP5H       1 -GCCACCCCC TCCCAGCCCC GCCATTGCCG GGTCCAGCTG CAGCTCCGCC
               ********** ********** ********** ********** **********
               ********** ********** ********** ********** **********
                ********* ********** **** ***** ********** **********
                ********* ********** **** ***** ********** **********
                ********* ********** ****  **** ********** **********
                                           **** ********** **********
                                           **** ********** **********
                                           **** ********** **********
                                           **** ********** **********
                                           **** ********** **********

alignment
position          251....... 261....... 271....... 281....... 291.......

P880080    209 ATCTTGGACC GCCGCcgcca ccgctgcTGC CCGCGCGCAG CCGGGCCGGC
P880083    251 ATCTTGGGCC GCCGC----- -------TGC CCGCGCGCCG CCGGGCCGGC
rATP5H      50 ATCTTGGGCC GCCGC----- -------TGC CCGCCCGCCG CCGGGCCGGC
               ********** *****             *** ********** **********
               ********** *****             *** ********** **********
               ********** *****             *** ********** **********
               ********** *****             *** ********** **********
```

Pairwise similarities:

For each pairwise alignment, the similarity (relative to the maximum similarity) and the number of identical nucleic acids (in % of shorter sequence) is given. Maximum values are underlined.

	P880083 (1007 bp)	rATP5H (829 bp)
**P880080 (1001 bp)**	0.514<br>49 %	0.417<br>51 %
**P880083 (1007 bp)**		1.000<br>76 %

Please note that the **similarity value 1.000** marks only the two **most similar** sequences, it does not necessarily mean that these sequences are identical.

**Extract alignment region**	
**Positions in alignment**	from: to:
**Output file**	dialign.seq

Extract aligned sequences

For comments, questions or bug reports, please contact support@genomatix.de

© Genomatix Software GmbH 1998-2005 - All rights reserved. License Agreement GEMS Launcher 4.1.1

Genomatix-Logo Logout Personal Messages GEMS-Logo
Logout Personal Messages

Genomatix_MainGEMSElDoradoGene2PromoterBiblioSphere
Frequently_asked_questionsYour ResultsYour SequencesYour ProtocolHelp

GEMS Launcher Task: *DiAlign TF*: Multiple alignment plus TF sites working on promoters.seq (3 seq.)

[Alignment] [Pairwise similarities]

DiAlign professional TF Release 3.1 December 2004 **Wed Jun 1 22:20:06 2005**

Solution parameters:

**Sequence file:**	promoters.seq (3 seq.)
**Type of sequences:**	nucleotide sequences
**Threshold T:**	0.00
**'*' signs below alignment denote:**	diagonal similarity (max. similarity is represented by 10 '*' signs)
**Alignment output:**	complete alignment is shown<br># nucleic acids per line: 50
**TF output:**	common TF matches located in aligned regions (common to 3 (100.0 %) sequences)
**Family matches:**	yes
**MatInspector library:**	Matrix Family Library Version 5.0 (February 2005)
**Selected groups** (core/matrix sim):	• ALL user_defined.lib (0.75/Optimized)<br>• ALL vertebrates.lib (0.75/Optimized)

Aligned Sequences:

No.	Sequence Name	Sequence Description	Length
1	P937000	sym=ATP5I|loc=Loc521|taxid=9606|spec=Homo sapiens|chr=4|ctg=NT_037622|str=(-)|start=657542|end=658598|len=1057| tss=501,557| comm=ATP synthase, H+ transporting, mitochondrial F0 complex, subunit e; (AK000656/557/gold;NM_007100/501/silver;)	1057 bp
2	P937002	sym=Atp5k|loc=Loc11958|taxid=10090|spec=Mus musculus|chr=5|ctg=NT_039308|str=(-)|start=31053274|end=31054277|len=1004| tss=501,504| comm=ATP synthase, H+ transporting, mitochondrial F1F0 complex, subunit e; (AK011342/547/gold;NM_007507/544/bronze;)	1004 bp
3	P937003	sym=Atp5k|loc=Loc140608|taxid=10116|spec=Rattus norvegicus|chr=14|ctg=NW_047419|str=(+)|start=1519276|end=1520276| len=1001|tss=501| comm=ATP synthase, H+ transporting, mitochondrial F1F0 complex, subunit e; (NM_080481/547/bronze;)	1001 bp

Alignment (DiAlign format):

Please note that only **upper-case** letters are considered to be **aligned**. For more information, please have a look at the user guide.

V$NRF1 U$NRF1 V$ETSF U$NRF2 V$RORA V$EVI1 V$SF1F V$SP1F

```
alignment position 1.........  11........ 21........ 31........ 41........
P937000        1 aaggacctgc ccggggagc- ---------- ---------- ----------
P937002        1 tgaacattac ctgtaaagcc caatggaaat taggagcTCT GGGCGAGAAA
P937003        1 ---------- ---------- ---------- -------TCT GGGTGAGAAA
                                                        *** **********
                                                        *** **********
                                                        *** **********

alignment position 51........ 61........ 71........ 81........ 91........
P937000       20 ---------- ---------- ---------- ---------- ----------
P937002       51 ACACTGGTCG GAGGACATAT GTGTCCaatg ccggaaaTTG CAGGAAAGCC
P937003       14 ACACTGGCCA GAGGACATAT GCGTCCgagg ctggaagTTG CAGGAAAGCC
                 ********** ********** ******            *** **********
                 ********** ********** ******            *** **********
                 ********** ********** ******            *** **********
                                                         *** **********
                                                         *** **********

alignment position 101....... 111....... 121....... 131....... 141.......
P937000       20 ---------- ---------- ---------- ---------- --------CA
P937002      101 CTGGAAGGTT GCTGACCTCT CAGGAAGgGC CAATACAAGT TCTGTAATCT
P937003       64 CTGGAAGGTT GCTGACCTCT CAGGAAGaGC CAATACAAGT TCTGTAATCT
                 ********** ********** ******* ** ********** **********
                 ********** ********** ******* ** ********** **********
                 ********** ********** ******* ** ********** **********
                 ********** ********** *******
                 ********** ********** *******

alignment position 151....... 161....... 171....... 181....... 191.......
P937000       22 CCTGCACTCG GCTCACACTC CGCTCCCAGG ACCCCCaggt ccct------
P937002      151 CATGCCCaaa ---------- ---------- CCCACCGCAC CCAATGGGCC
P937003      114 CCTGCCCTGT CTCCCCCCTC CGCCCCCCCC CCCCCCGCAT CCATTGGGCC
                 ********** ********** ********** ********** **********
                 *******                          ********** **********
                 *******                          ******

alignment position 201....... 211....... 221....... 231....... 241.......
P937000       66 ---------- ---------- ---------- ---------- ----------
P937002      181 GCTGGAGTCG GCCAGGTGTg CTCGTCTGTT CCAAGAGATA AAGGGATTGT
P937003      164 GCCCCCGTCG GCCAGGTGTt CTTGTCTGTT CCAAGAGACG AAGGAATTGT
                 ********** *********  ********** ********** **********
                 ********** *********  ********** ********** **********
                                       ********** ********** **********

alignment position 251....... 261....... 271....... 281....... 291.......
P937000       66 -------GCC CAGGCCACCC TCGGGTGGGC GCGGTCCGCG GGGCTGGACc
P937002      231 TTGCGTGCTC CAGTCCACAA CAGGTGGGCG ACAGATTCCA GGGCTGGTCa
P937003      214 TTGCTTGGTC TAGTCCACAA CAGGTGGGCG ACGGATTCCA GGGCTTGTgg
                 ********** ********** ********** ********** *********
                 **********  ********* ********** ********** ********
                 **********  ********* ********** ********** ********
                             ********* ********** ********** ********

alignment position 301....... 311....... 321....... 331....... 341.......
P937000      109 cgcccg---- ---------- ---------- ---------- ----------
```

```
P937002    281 gTCATTACAC GCCGGTGCTG AAGTAACGAG GCTCTGTGTC aCCACCCTTT
P937003    264 tTCTTCACAC GTCGATGCGG AAGTAACGAG GCTCTGCGTC tCCACCCTTC
                *********  ********** ********** **********  *********
                *********  ********** ********** **********  *********
                *********  ********** ********** **********  *********

alignment position 351....... 361....... 371....... 381....... 391.......
P937000    115 ---AGCCGCC CTGACCTCAG GCCTCCGACG CTCCGCGCAA GAAgacagag
P937002    331 TGAAGCCAGC TTACACTAAC AGCCTAGAct tcctacct-G GAGAGTAGTA
P937003    314 TGAAGCCATC TTGCACTAAC AGCCCAGAGA CTTCGCACGG GAAAGCGGTA
               ********** ********** ********** ********** **********
               ********** ********** ********
               ********** ********** ********

alignment position 401....... 411....... 421....... 431....... 441.......
P937000    162 tgcgccccgg cccgcagtct ggggtgtgga cacacggcca ggacgcggac
P937002    380 ACCA------ ---------- ---------- ---------- ----------
P937003    364 ACCA------ ---------- ---------- ---------- ----------
               ****

alignment position 451....... 461....... 471....... 481....... 491.......
P937000    212 ggcggggcct cggcagcagc gcgggaaaac agcggacccc cagccctgcg
P937002    384 ---------- ---------- ---------- ---------- ----------
P937003    368 ---------- ---------- ---------- ---------- ----------

alignment position 501....... 511....... 521....... 531....... 541.......
P937000    262 gggaaaggaa accccccgcc cactcggcgc agatcccgcc tcacgcagca
P937002    384 ---------- ---------- ---------- ---------- ----------
P937003    368 ---------- ---------- ---------- ---------- ----------

alignment position 551....... 561....... 571....... 581....... 591.......
P937000    312 actgcgcagg cgcagccgat gatcctccgc gcactcccca gcctttgtgc
P937002    384 ---------- ---------- ---------- ---------- ----------
P937003    368 ---------- ---------- ---------- ---------- ----------

alignment position 601....... 611....... 621....... 631....... 641.......
P937000    362 gcgtgcgcct cacgtgacgt caccGCCGCG CGGGGGCGCG CAGTCACCCT
P937002    384 ---------- ---------- ----GCCccg TGGGAACGCT CGGCTATCCT
P937003    368 ---------- ---------- ----GCCTCT TGGGAACGCA CTGCTATCCT
                                         ****** ********** **********
                                                ********** **********
                                                ********** **********
                                                ********** **********
                                                 ********* **********

alignment position 651....... 661....... 671....... 681....... 691.......
P937000    412 TCGGTCGCCA GGGGGCgcgg ccccccgctc tctcgccgct gACGGCGCAT
P937002    410 CCAGTCGCTA GGGGGCAGCg ctttcaCT-- ---------- -GCGGCGCAT
P937003    394 CCAGTCGCTA GGGGGAAGCa tttgcgCT-- ---------- -ACGGCGCAT
               ********** *********       **              *********
               ********** *********       **              *********
               ********** *********       **              *********
               ********** *********                       *********
               ********** ****
```

```
alignment position 701....... 711....... 721....... 731....... 741.......
P937000    462 GCGtgcgccc agctccgcct ttgcctccaa ggct-TTGCT GGCTTgtgcg
P937002    447 GCGCCTTTCT GGCTTCAGGA GGCGGCTTtc attct---CT GTCTTCCGCC
P937003    431 GCGCTTTCCT GGCTTCAGGA GGCGTCTTgg cgttcTTGCT GGCTTCCGCC
               ********** ********** ********        ***** **********
               ********** ********** ********           ** **********
               ********** ********** ********           ** **********
               ***

alignment position 751....... 761....... 771....... 781....... 791.......
P937000    511 gcatcctgct ccgtctgcag GTTGTGCTTC CGGTGCGGAG GTCAGGGACA
P937002    494 TCA------- ---------- GTTGAGCTTC CGGCGCGAAG GTCACGGACA
P937003    481 CCA------- ---------- GGTGAGCTTC CGGCGCGGAG GTCACGGACA
               ***                   ********** ********** **********
               ***                   ********** ********** **********
               ***                   ********** ********** **********
                                     ********** ********** **********
                                     ********** ********** **********
                                       ******** ********** **********
                                       ******** ******* ** **********
                                       ******** ******* ** **********
                                                        ** **********
                                                        ** **********

alignment position 801....... 811....... 821....... 831....... 841.......
P937000    561 AGATGGTGCC aCCGGTGCAG GTCTCTCCGC TCATCAAGGT GAtccaggct
P937002    527 AAATGGTGCC CCCGGTTCAG GTCTCTCCAC TCATCAAGGT GAATCCTCAT
P937003    514 AAATGGTGCC CCCGGTTCAG GTCTCTCCGC TCATCAAGGT GAATCCCCTT
               ********** ********** ********** ********** **********
               ********** ********** ********** ********** **********
               ********** ********** ********** ********** **********
               ********** ********** ********** ********** **
               ********** ********** ********** ********** **
               **********  ********* ********** ********** **
               **********  ********* ******** * ********** **
               **********  ********* ******** * ********** **
               **********  ********* ********
               **********  ********* ********

alignment position 851....... 861....... 871....... 881....... 891.......
P937000    611 ttgcagc--- ---CGCCGCC CAGTGCTCTG GGCCCCCCCG TGTCCCGCCC
P937002    577 CCCCGTTGTT GCGCGGTGCT CAAAGCTCCT CTATGCCCCC TTTCCCCTCC
P937003    564 CACCGTCACT GCGCGGTGCT CACAGCCCTT GTATGCCCCC TTTCCCTTCC
               ********** ********** ********** ********** **********
               ********** ********** ********** ********** **********
               ********** ********** ********** ********** **********

alignment position 901....... 911....... 921....... 931....... 941.......
P937000    655 Cg-------- ---------- --CAGCTCCG ACCCCGCGGG CTGCGACCCC
P937002    627 CCTCCCGTGA CTTAGCATTG CCCACCCTTG GGACTGCTGG CTGTGGTCTc
P937003    614 CCTCCCCTGA CTTAGCATTG CCCACCCTCG AGACTGCCGG CTGTGGTCTC
               ********** ********** ********** ********** **********
               ********** ********** ********** ********** *********
               ********** ********** ********** ********** *********
                        * ********** ********** ********** *********

alignment position 951....... 961....... 971....... 981....... 991.......
P937000    685 Cggcccccgc ccccatgatc ctcgccccgt gaccctcgcc cggcgaccgg
```

```
P937002     677 g-AGCAATC- ---------- ---------- ---------- ----------
P937003     664 CaAGCAATC- ---------- ---------- ---------- ----------
                *  *******
                   *******
                   *******
                   *******

alignment position 1001...... 1011...... 1021...... 1031...... 1041......
P937000     735 acccccaCCC CTCCTGCGAC CTCTGCCCAG CGACCCCGCC CCCCCTCC--
P937002     685 -------CCG GCCCTGCAAC CCTTGCACAC ACACCCGCCC CGTACTCCCT
P937003     673 -------CCC GGCCTGCAAC CCTTGCACAC ACACCCGCCC attgCTCCCT
                       *** ********** ********** ********** **********
                       *** ********** ********** **********     ******
                       *** ********** ********** **********     ****
                       *** ********** ********** **********
                             ******** ********** **********

alignment position 1051...... 1061...... 1071...... 1081...... 1091......
P937000     783 ---------- TCCCGCGACC CCCGTCC-CG CGAGCCCCGT TTCGCGCCGc
P937002     728 CTCCCAGTAC TCCTCGATGT CCGACCCcTG CGATCCCCTT GTTCCCTCGG
P937003     716 TTCCCAGTCC TCCTCCATCT CCGACCC-TG CGATCCCCTT GTCCCACCGG
                ********** ********** ******* ** ********** **********
                ********** ********** ******* ** ********** **********
                           ********** ******* ** ********** **********

alignment position 1101...... 1111...... 1121...... 1131...... 1141......
P937000     822 ctctgaccaa cc-------- --TCTTTCGC AGCTCGGCCG CTACTCCGCC
P937002     778 TGTGCCTTCT ACTATAAtGT TTTCTTCTGC AGTTCGGCCG GTACTCCGCT
P937003     765 TGTTCCTTCT GCTCTAAcGT TTTGTTCTGC AGTTCGGCCG GTACTCCGCT
                ********** ******* ** ********** ********** **********
                ********** ******* ** ********** ********** **********
                ********** ******* ** ********** ********** **********
                                  ** ********** ********** **********
                                       ******** ********** **********
                                       ******** ********** **********
                                             ** ********** **********
                                             ** ********** **********

alignment position 1151...... 1161...... 1171...... 1181...... 1191......
P937000     862 CTGTTCCTCG GtgTGGCCTA CGGAGCCACG CGCTACAGTG AGTgaccgcg
P937002     828 CTGATCATCG GCATGGCATA CGGCGCCAAG CGCTACAGTG AGTTTGGGGA
P937003     815 CTGATCCTCG GCATGGCATA CGGCGCCAAG CGCTACAGTG AGTTTGGGGA
                ********** ********** ********** ********** **********
                ********** ********** ********** ********** ******
                ********** ********** ********** ********** ******
                ********** ********** ********** ********** ******
                ****** *** ********** ********** ********** ******
                ****** *** *  ******* ********** ********** ***
                ****** *** *  ******* ********** ********** ***
                ****** *** *  ******* ********** ********** ***
                       *** *  ******* ********** ********** ***

alignment position 1201...... 1211...... 1221...... 1231...... 1241......
P937000     912 ggcggggccg gggcggggt- ---------- ---------- ----CTGCTC
P937002     878 CCGGGAGAAC GGGATTCCGG Gtgaaag--- ---GCTGTGT GACACTGCTC
P937003     865 TTGGGAGGGC GGGATTCCGG Ggagagacac ctaGCTGTGT GACACTGCTC
```

```
                      ********** ********** *                     ******* **********
                                                                  ******* **********
                                                                  ******* **********

alignment position    1251...... 1261...... 1271...... 1281...... 1291......
P937000          937  GGcttcctg  tggagctcct gt-------- ---------- ----------
P937002          922  GAGCCTGCAG TTCCTTGGGA GGGagatgat gccaggccct AGAGGGGGGC
P937003          915  GGGCCAGCAC CTCCTCGGGA GGGaaataat tctaggcc-- AGAGGGGAGC
                      ********** ********** ***                   **********
                      ********** ********** ***                   **********
                      ********** ********** ***                   **********

alignment position    1301...... 1311...... 1321...... 1331...... 1341......
P937000          959  ---------- -GCTATTTGG AGAactccag ctctggaagg gggctttgga
P937002          972  AGGTGGGAAC AGCTATCTGG AGATTTTGGA gtc------- ----------
P937003          963  AGGTGGGGAC AGCTATCCGG AGATTCTGGA gtcggcttt- ----------
                      ********** ********** **********
                      ********** ********** **********
                      ********** ********** **********

alignment position    1351...... 1361...... 1371...... 1381...... 1391......
P937000          998  cgcatgcgag gctgcaggac cgtgtcctcc accgagcgtg accccgatag
P937002         1005  ---------- ---------- ---------- ---------- ----------
P937003         1002  ---------- ---------- ---------- ---------- ----------

alignment position    1401......
P937000         1048  tggaatcagc
P937002         1005  ----------
P937003         1002  ----------
```

Pairwise similarities:

For each pairwise alignment, the similarity (relative to the maximum similarity) and the number of identical nucleic acids (in % of shorter sequence) is given. Maximum values are underlined.

	**P937002 (1004 bp)**	**P937003 (1001 bp)**
**P937000 (1057 bp)**	0.151<br>25 %	0.168<br>33 %
**P937002 (1004 bp)**		<u>**1.000**</u><br><u>79 %</u>

Please note that the **similarity value 1.000** marks only the two **most similar** sequences, it does not necessarily mean that these sequences are identical.

**Extract alignment region**	
**Positions in alignment**	from: to:
**Output file**	dialign.seq

Extract aligned sequences

For comments, questions or bug reports, please contact support@genomatix.de

© Genomatix Software GmbH 1998-2005
- All rights reserved.

License Agreement

GEMS Launcher 4.1.1

Genomatix-Logo Logout Personal Messages GEMS-Logo
Logout Personal Messages

Genomatix_MainGEMSElDoradoGene2PromoterBiblioSphere
Frequently_asked_questionsYour ResultsYour SequencesYour ProtocolHelp

GEMS Launcher Task: *DiAlign TF*: Multiple alignment plus TF sites working on C5-ATP5J (3 seq.)

[Alignment] [Pairwise similarities]

DiAlign professional TF Release 3.1 December 2004 **Wed Jun 1 00:46:06 2005**

Solution parameters:

**Sequence file:**	C5-ATP5J (3 seq.)
**Type of sequences:**	nucleotide sequences
**Threshold T:**	0.00
**'*' signs below alignment denote:**	diagonal similarity (max. similarity is represented by 10 '*' signs)
**Alignment output:**	complete alignment is shown<br># nucleic acids per line: 50
**TF output:**	common TF matches located in aligned regions (common to 3 (100.0 %) sequences)
**Family matches:**	yes
**MatInspector library:**	Matrix Family Library Version 5.0 (February 2005)
**Selected groups** (core/matrix sim):	• ALL user_defined.lib (0.75/Optimized)<br>• ALL vertebrates.lib (0.75/Optimized)

Aligned Sequences:

No.	Sequence Name	Sequence Description	Le
1	P977923	P977923 sym=ATP5J|loc=Loc522|taxid=9606|spec=Homo sapiens|chr=21|ctg=NT_011512|str=(-)|start=12769355|end=12770355|len=1001|tss=501|comm=ATP synthase, H+ transporting, mitochondrial F0 complex, subunit F6; (NM_001685/544/silver;)	100
2	P977924	P977924 sym=Atp5j|loc=Loc11957|taxid=10090|spec=Mus musculus|chr=16|ctg=NT_039625|str=(-)|start=19651782|end=19652782|len=1001|tss=501|comm=ATP synthase, H+ transporting, mitochondrial F0 complex, subunit F; (NM_016755/596/silver;)	100
3	P977926	P977926 sym=Atp5j|loc=Loc94271|taxid=10116|spec=Rattus norvegicus|chr=11|ctg=NW_047354|str=(-)|start=24323700|end=24324800|len=1101|tss=1001|comm=ATP synthase, H+ transporting, mitochondrial F0 complex, subunit F6; (NM_053602/1194/silver;)	110

Alignment (DiAlign format):

Please note that only **upper-case** letters are considered to be **aligned**. For more information, please have a look at the user guide.

V$EGRF V$MAZF V$SP1F V$ZBPF V$PLAG V$PAX8 V$SNAP V$CLOX V$ETSF V$FKHD V$AP2F V$AHRR

V$NRF1 U$NRF1 V$ZF5F V$MZF1 V$VMYB V$NFKB U$NRF2 V$NOLF V$BARB V$PERO

```
alignment position   1.........  11........ 21........ 31........ 41........
P977923     1  gcgtgtcttc tagtacacaa aaaaatttcc aagtagacaa cccattgtta
P977924     1  ccaaccccca aaaaaggtcg gggggaggag tgggggaagt tggcaaggta
P977926     1  tgta------ ---------- ---------- ---------- ----------

alignment position   51........ 61........ 71........ 81........ 91........
P977923    51  tctccttggt ttacttcctt gcttaaaaac aaaaaaacaa acccagatgg
P977924    51  cagtaacttt cactgacatc ttcgggaaag acgcagaaag tcatcctatg
P977926     5  ---------- ---------- ---------- ---------- ----------

alignment position   101....... 111....... 121....... 131....... 141.......
P977923   101  ctaccatata cgctgataaa tgattctggt gtgtgttgtt ctataaagag
P977924   101  CATTAGAACG ATTGCACCGA TGCGACTCTa g-GACAGTTC CAGAAGGCA
P977926     5  CATTAGAATG ATGGCACCGG TGCGACTCTt gaGACAGTTC CAGAAAAGCA
               ********** ********** *********    ******** **********
               ********** ********** *********

alignment position   151....... 161....... 171....... 181....... 191.......
P977923   151  agcGGATTTG A-AATGAGTG AATCGCTGAG TCCCGCGATg AGACAGGGTC
P977924   150  Atcggtactg ctAGTGCGGG GATGGCTGAC TCGGGTGAC- AGACAGGTCT
P977926    55  ActGGGTTTG G-AGTGCGGG GATGGTCGAG TGCGGTGAT- AGGCAagcCT
               *  ******* * ******** ********** *********  **********
                                                                  **

alignment position   201....... 211....... 221....... 231....... 241.......
P977923   200  TGCATTTTGC tcttaaaatc tcgggggcgt caggaccaca tcccagctga
P977924   199  GGCATCTTCC CGAGAGACCC GGGAGCg--- ---------- ----------
P977926   103  GGCATCTTTC AGAGAGACCC GGGAGCt--- ---------- ----------
               ********** ********** ******
               ********** ********** ******

alignment position   251....... 261....... 271....... 281....... 291.......
P977923   250  gcaggctgag actgctgagg agtgcggacg ggcgaggagg ggacgcggaa
P977924   226  ---------- ---------- ---------- ---------- ----------
P977926   130  ---------- ---------- ---------- ---------- ----------

alignment position   301....... 311....... 321....... 331....... 341.......
P977923   300  aggagggagc ggcgacaggc gacccgtccg gcacgggtCC CGGCGGTGCC
P977924   226  ---------- ---------- ---------- --------CC CGGGGCTGTC
P977926   130  ---------- ---------- ---------- ---------C CGGGACTATC
                                                       ** **********
                                                        * **********

alignment position   351....... 361....... 371....... 381....... 391.......
```

```
P977923  350 CGGTCCCCCC GGGCTCCAGG CCgctcg--- ---------- ----------
P977924  238 CGCTCGGCCA GCGCGCCGAG CCTGGACCGc gcccgtGCGA CGGGCGACTC
P977926  141 CGCACCGTCA GCGCGCAGAG CCTGGACCGt gcctg-GCGA CGGGCGACTC
             ********** ********** *********        **** **********
             ********** ********** *********        **** **********
                                                    **** **********

alignment
position         401.......   411.......   421.......   431.......   441.......

P977923  377 ---------- ---------- ---------- ------CCCC CCGCCCCTCC
P977924  288 CGCCGCGTCC CGCAACGCGG CCCgGGCCAC GCCGCTCCCC GCGCCCCTCC
P977926  190 CGCAGCGTCC CGCAGCGCGG CCCcGGCCAC GCCGCTTCCC GCGCCCCTCC
             ********** ********** *** ****** ********** **********
             ********** ********** *** ****** ********** **********
             ********** ********** *** ****** ********** **********
                                              **** **********
                                               *** **********
                                               *** **********

alignment
position         451.......   461.......   471.......   481.......   491.......

P977923  391 CCTTCGCTCC CCGCACAG-C TCTCCCGCCC CTCCCCACCC GGCTCCCTCA
P977924  338 CCTCGGCTTC CCGCACAGCC TCTCCCGCCC CTCCCCACCA GGCTCCCTCA
P977926  240 CCTCGGCTCC CCGCACAGCC TCTCCCGCCC CTCCCCACCC GGCTCCCTCA
             ********** ********** ********** ********** **********
             ********** ********** ********** ********** **********
             ********** ********** ********** ********** **********
             ******** * ********** ********** ********** **********
             ******** * ******** * ********** ********** **********
             ******** * ******** * ********** ********** **********
                                 * ********** ********** **********
                                 * ********** ********** **********
                                 * ********** ********** **********
                                 * ********** ********** **********

alignment
position         501.......   511.......   521.......   531.......   541.......

P977923  440 AGTCTCCACG AACGGAAATG GGAGTCACAG CGCCGGGGA- CCAGCGGAAA
P977924  388 AGTCTCCACG AACGGAAATG GGAGTCGCAG CGCCGGGGAc CCAGCGGAAA
P977926  290 AGTCTCCACG AACGGAAATG GGAGTCACAG CGCCGGGGA- CCAGCGGAAA
             ********** ********** ********** *********  **********
             ********** ********** ********** *********  **********
             ********** ********** ********** *********  **********
             ********** ********** ********** *********  **********
             ********** ********** ********** *********  **********
             ********** ********** ********** *********  **********
             ********** ********** ********** *********  **********
             ********** ********** ********** *********  **********
             ********** ********** ********** *********  **********
             *********                                   **********

alignment
position         551.......   561.......   571.......   581.......   591.......

P977923  489 CAAAACACAG AGGCGGCCGC GGGGGGGCcc ttgtggGGGA AAAGGCCCCC
P977924  438 CAAAACACAG AGGCGGCCGC GGGGGGGgac ---------- --AAGCCCCC
P977926  339 CAAAACACAG AGGCGGCCGC GGGGGGGCtg cggc--GGGG GAAAGCCCCC
```

```
                  ********** ********** ********            **** **********
                  ********** ********** ********                   ********
                  ********** ********** ********                     ******
                  ********** ********** *******
                  ********** ********** *******
                  ********** ********** *******
                  ********** ********** *******
                  ********** ********** *******
                  ********** ********** *******

alignment
position          601....... 611....... 621....... 631....... 641.......

P977923   539     GAACGAGGCC GGCCCGCGAG GGCCCGCGCT CCCCTCCTCC CCGgcgGCCC
P977924   476     GAACCAGGCC G-CCCGCGCG GGCCCGCGCT CCCCGCCAGC CCGCG-TCCC
P977926   387     GAACCAGGCC GG-CCGCGCG GGCCCGCGCT CCCCGCCAGC CCGCA-GCCC
                  ********** ********** ********** ********** *****  ****
                  ********** *  ******* ********** ********** *****  ****
                  ********** *   ****** ********** ********** *****  ****
                                ******* ********** ********** ***    ****
                                ******* ********** ********** ***    ****

alignment
position          651....... 661....... 671....... 681....... 691.......

P977923   589     CA-CTCACCC ACTCCGGAAT CGGCCCTGTC GCCCCCGCCC GCaC-TCCAC
P977924   524     CAaCTCACCA GCTCCGGAAT TCGTCCCGTG GCCCTAGCCC GCGctTCCAC
P977926   435     CA-CTCACCA GCTCCGGAAT CCGCCCCGTG GCCCTCGCCC GCGC-TCCAC
                  **  ******* ********** ********** ********** ****  *****
                  **  ******* ********** ********** ********** ****  *****
                  **  ******* ********** ********** ********** ****  *****
                  **  ******* ********** ********** ********** **    *****
                  **  ******* ********** ********** *******          *****
                      ******* ********** ********** *******

alignment
position          701....... 711....... 721....... 731....... 741.......

P977923   637     AGCCGGCTGA GAACGGCAGC GGCGAGGGCT CCAcgtgCAG CGTCTCTCCC
P977924   574     AGCCGGCTGG GAACGGCGGC GGCGCGGGCT CCAGGTACAG CGCCTCTCCC
P977926   483     AGCCGGCTGG GAACGGCGGC GGCGCGGGCT CCAGGTACAG CGTCTCTCTC
                  ********** ********** ********** ********** **********
                  ********** ********** ********** ********** **********
                  ********** ********** ********** ********** **********
                  ********** ********** ********** ********** **********
                  ********** ********** ********** ***    *** **********
                  ********** ********** ********** ***    ***
                  ********** ********** ********** ***    ***
                  ********** ********** ********** ***

alignment
position          751....... 761....... 771....... 781....... 791.......

P977923   687     GGTAAGACCT GCAGCTCCCG CGACTAGaAG TGTGAGGTCC CAGGGCCCTT
P977924   624     GGCGAGCCGC GCCGCTCCCG CGAGTAGCAG GGTAAGGTCC CGGGGCCCTT
P977926   533     GGTAAGACGC GCCGGTCCCG CGAGTAGCAG TGTGAGGCCC CGGGGCCCTT
                  ********** ********** ********** ********** **********
                  ********** ********** ********** ********** **********
                  ********** ********** *******  ** ********** **********
                  ********** ********** *******  ** ********** **********
                  ********** ********** *******  ** ********** **********
                                                 **  ********* **********
```

```
                                                   ** *********  **********
alignment
position    801.......  811.......  821.......  831.......  841.......
P977923 737 CCCGCGGAAT  CGGCGCTgag  aagCCCCAAC  CCCCCTCCCG  CCTCCCAGGC
P977924 674 CCCGTGGAAT  CGGCGCTAAG  GAACCCCAGC  CCCTCTCCCG  CCTCCCAGGC
P977926 583 CCCGCGGAAT  CGGCGCTAAG  GAACCCCAGC  CCCTCTCCCG  CCTCCCAGGC
            **********  **********  **********  **********  **********
            **********  **********  **********  **********  **********
            **********  **********  **********  **********  **********
            **********  **********  **********  **********  **********
            **********  *******         *******  **********  **********
            **********  *******         *******  **********  **********
            **********  *******         *******  **********  **********
                             **         *******  **********  **********
                             **

alignment
position    851.......  861.......  871.......  881.......  891.......
P977923 787 ACCTTCGGGt  ccaggACTAC  AACTCCCGGC  AGGGTGCGCA  AAACG-ACCC
P977924 724 ACCCCCGGGC  CTGGAACTAC  AACTCCCAGC  AGGCTGCGCG  gcccgaAACC
P977926 633 ACCCCtGGGC  CTGGAACTAC  AACTCCCAGC  AGGCTGCGCG  ACACG-AACC
            **********  **********  **********  **********  ****  ****
            **********  **********  **********  **********  ****  ****
            *****  ***  **********  **********  **********
            *****  ***       *****  **********  **********
            *****  ***       *****  **********  **********
            *****            *****  **********  *********
            ***
            ***

alignment
position    901.......  911.......  921.......  931.......  941.......
P977923 836 CCCaaggCAA  CGGAGGCTCA  CGAGGTACAa  ggaagcacAA  CGCCCCA-AT
P977924 774 CCCGCGACGA  C-GGGGCTCA  CGAGGCACA-  --------AA  CGCCCCccAC
P977926 682 CCCGTGACAA  CGGGGGCTCG  CGAGGCACA-  --------AA  CGCCCCA-AC
            **********  **********  *********           **  *******  **
            ***            ********  *********           **  *******  **
                             ******  *********           **  ******

alignment
position    951.......  961.......  971.......  981.......  991.......
P977923 885 CCGCTTTG--  ----------  ----------  ----TTTTAC  acagtctcac
P977924 814 CCGCTTTGgt  tttttgtttt  gttttggttt  tggtTTTTAC  GCGCTCCTTT
P977926 722 CCGCTTTG--  ----------  ----------  ----TTTTAC  GCGCTCGTTT
            ********                                ******  **********
            ********                                ******

alignment
position    1001......  1011......  1021......  1031......  1041......
P977923 909 tttCCCTGCA  CCTTTATACA  CTTCGATAAC  CCTAACGAAC  AGGACGCTGA
P977924 864 gtaCCCCCAC  CCTACATCCA  CCTCCCCAAC  CCCAAGGATC  GGGACGCTGA
P977926 746 ta--CCCCAC  ATTACATCCA  CCTCCCCAAC  CCCAAGGATC  AGGACGTTGA
               *******  **********  **********  **********  **********
                ******  **********  **********  **********  **********
                ******  **********  **********  **********  **********
                             *****  **********  **********  **********
                                                                  *******
```

```
                                                              *******

alignment position  1051......  1061......  1071......  1081......  1091......
P977923   959  CTCGGAGGGG  AGCTTGAACT  AGGGGAAAGG  CCActcaaag  agc-------
P977924   914  CTCCGAAGGG  AGCTTGAACT  AGGGGACAGG  CCAgaatACG  CATGCGTACA
P977926   794  CTCCGTAGGG  AGCTTGAATT  AGGGGACAGG  CCAaagcACG  CATGCGTACA
               **********  **********  **********  ***    ***  **********
               **********  **********  **********  ***    ***  **********
               **********  **********  **********  ***
               **********  **********  **********  ***
               **********  **********  **********  ***
               *****  ***  **********  **********  ***
                      ***  **********  **********  ***

alignment position  1101......  1111......  1121......  1131......  1141......
P977923  1002  ----------  ----------  ----------  ----------  ----------
P977924   964  GTGCAGTGTc  TCCAAGAGGG  AAAAGGGGGT  GAAGTTAA--  ----------
P977926   844  GTGCAGTGTt  TCCAAGAGGG  AAAAGAGGGT  GAAGTTAAgt  gtaggaaaga
               *********   **********  **********  ********
               *********   **********  **********  ********

alignment position  1151......  1161......  1171......  1181......  1191......
P977923  1002  ----------  ----------  ----------  ----------  ----------
P977924  1002  ----------  ----------  ----------  ----------  ----------
P977926   894  tggcggagtc  ttagctgagg  gacccggaag  cacttaccct  ccacttcctc

alignment position  1201......  1211......  1221......  1231......  1241......
P977923  1002  ----------  ----------  ----------  ----------  ----------
P977924  1002  ----------  ----------  ----------  ----------  ----------
P977926   944  ctcctgtttg  gcttctgtct  cacctagagg  cgtccggtcg  cagactctct

alignment position  1251......  1261......  1271......  1281......  1291......
P977923  1002  ----------  ----------  ----------  ----------  ----------
P977924  1002  ----------  ----------  ----------  ----------  ----------
P977926   994  tcgaggcgct  tcctgtccgg  tgagcgtcga  acgactgaag  cggtggccca

alignment position  1301......  1311......  1321......  1331......  1341......
P977923  1002  ----------  ----------  ----------  ----------  ----------
P977924  1002  ----------  ----------  ----------  ----------  ----------
P977926  1044  tagtgcattg  cgatggcggg  taggcgtgtg  taggcggagc  cagggccgga

alignment position  1351....
P977923  1002  --------
P977924  1002  --------
P977926  1094  agtagaac
```

Pairwise similarities:

For each pairwise alignment, the similarity (relative to the maximum similarity) and the number of identical nucleic acids (in % of shorter sequence) is given. Maximum values are underlined.

	P977924 (1001 bp)	P977926 (1101 bp)
P977923 (1001 bp)	0.487<br>52 %	0.577<br>55 %
P977924 (1001 bp)		1.000<br>76 %

Please note that the **similarity value 1.000** marks only the two **most similar** sequences, it does not necessarily mean that these sequences are identical.

Extract alignment region	
Positions in alignment	from: to:
Output file	dialign.seq

Extract aligned sequences

For comments, questions or bug reports, please contact support@genomatix.de

© Genomatix Software GmbH 1998-2005 - All rights reserved. License Agreement GEMS Launcher 4.1.1

Logout Personal Messages

GEMS Launcher Task: *DiAlign TF*: Multiple alignment plus TF sites working on promoters.seq (3 seq.)

[Alignment] [Pairwise similarities]

DiAlign professional TF Release 3.1 December 2004 **Wed Jun 1 22:45:23 2005**

Solution parameters:

**Sequence file:**	promoters.seq (3 seq.)
**Type of sequences:**	nucleotide sequences
**Threshold T:**	0.00
**'*' signs below alignment denote:**	diagonal similarity (max. similarity is represented by 10 '*' signs)
**Alignment output:**	complete alignment is shown<br># nucleic acids per line: 50
**TF output:**	common TF matches located in aligned regions (common to 3 (100.0 %) sequences)
**Family matches:**	yes
**MatInspector library:**	Matrix Family Library Version 5.0 (February 2005)
**Selected groups** (core/matrix sim):	• ALL user_defined.lib (0.75/Optimized)<br>• ALL vertebrates.lib (0.75/Optimized)

Aligned Sequences:

No.	Sequence Name	Sequence Description	Length
1	P848677	sym=ATP5L|loc=Loc10632|taxid=9606|spec=Homo sapiens|chr=11| ctg=NT_033899|str=(+)|start=21834233|end=21835233|len=1001| tss=501| comm=ATP synthase, H+ transporting, mitochondrial F0 complex, subunit g; (NM_006476/501/bronze;)	1001 bp
2	P848678	sym=Atp5l|loc=Loc27425|taxid=10090|spec=Mus musculus|chr=9| ctg=NT_039473|str=(-)|start=4369214|end=4370255|len=1042| tss=501,502,507,510,511,513,516,517,520,527,532,535,542| comm=ATP synthase, H+ transporting, mitochondrial F0 complex, subunit g; (AK008321/510/gold;AK008251/516/gold;AK019320/514/gold; AK011046/538/gold;AK011038/538/gold;AK008183/516/gold; AK008087/530/gold;AK008169/505/gold;AK009612/523/gold; AK019252/516/gold;AK019350/516/gold;AK019343/545/gold; AK009471/516/gold;AK019192/516/gold;AK008002/545/gold; AK019274/516/gold;AK005321/505/gold;AK019355/516/gold; AK009465/516/gold;AK009806/516/gold;AK008012/519/gold; AK009468/516/gold;AK011099/513/gold;AK008030/519/gold; AK005175/535/gold;AK011842/545/gold;AK008036/505/gold; AK005380/504/gold;NM_013795/520/bronze;)	1042 bp

3	P848679	sym=MGC72942|loc=Loc300677|taxid=10116| spec=Rattus norvegicus|chr=8|ctg=NW_047799|str=(-)| start=17495578|end=17496578|len=1001|tss=501| comm=similar to CG6105-PA; (XM_217128/517/bronze;)	1001 bp

Alignment (DiAlign format):

Please note that only **upper-case** letters are considered to be **aligned**. For more information, please have a look at the user guide.

V$NRF1 U$NRF1 V$ETSF V$RORA V$EREF U$EREF V$COUP V$PAX5 V$DEAF V$ZF5F

```
alignment
position          1.........  11.........  21.........  31.........  41.........

P848677     1     ac--------  ----------  ----------  ----------  --------TG
P848678     1     ---GAAAACG  GAATTATaaa  AAGTCAATTA  AAGAAGTCGG  TGAATGGGTG
P848679     1     gagGAAAACG  GAATTATg--  AAGTCAATTA  AAGAAGTCGT  TGATTGGGTG
                     *******  *******     **********  **********  **********
                                          **********  **********  **********
                                          **********  **********  **********
                                          **********  **********  **********

alignment
position          51........  61........  71........  81........  91........

P848677     5     TCTCAAATAA  A---AAGGGC  TATGggttga  gcctggtgac  agcctgtgtg
P848678    48     AGTCACATAA  -TGTAACGCC  TATGT-----  ----------  ----------
P848679    49     ACTCACATAA  ATGTAAGGCC  TATGT-----  ----------  ----------
                  **********  **********  *****
                  **********   *********  *****
                  **********   *********  *****
                  **********

alignment
position          101.......  111.......  121.......  131.......  141.......

P848677    52     gtgattgctc  GGCCCCTAAC  TTTATATGTT  TGGCCTTGGA  CCctctCTAC
P848678    72     ----------  GGCCCCTTAA  AGCTCCTATT  TGACCTCAGA  CCTG--CTAT
P848679    74     ----------  GGCCCCTTAA  AGTTCTTATT  TGGTCTTGGC  CCTG--CTAT
                              **********  **********  **********  ****  ****
                              **********  **********  **          ****  ****
                              **********  **********  **
                              **********  **********  **

alignment
position          151.......  161.......  171.......  181.......  191.......

P848677   102     CTCTGTACAA  CGATCGTGTT  GGACTAGAAG  AATCTCAgcg  aatccttctc
P848678   110     GCCTTTAAAG  CGAACGTATA  GGCCCACAAG  CATCTTATGA  AGCCCTCCAG
P848679   112     GCCTTTAAGA  CGAACATATC  GACCCACAAG  CATCTTGTGA  AGCATTCCAG
                  **********  **********  **********  **********  **********
                  **********  ********      ********  **********  **********
                    ********  ********      ********  **********  **********
                                            ********  **********  **********

alignment
position          201.......  211.......  221.......  231.......  241.......

P848677   152     cagtactgac  ttcaaAGGTT  CTCAGAATCG  CGGCGAAAGC  ACCCTACGCA
P848678   160     CCTCGTTTTC  C----ATGTT  TTCTGAAAAG  CTATAAAACT  GCCGCAAGCT
```

```
P848679 162 CCTCGATTTC C----AtaTT TTCTGAAAAG CTACGAAACT GCCGCAAGCT
            ********** *    ***** ********** ********** **********
            ********** *    *  ** ********** ********** **********
            ********** *    *  ** ********** ********** **********
            ********** *    *  ** ********** ********** **********

alignment
position    251....... 261....... 271....... 281....... 291.......

P848677 202 AAatagcgga ggtcggcGCC AAGAAAAGCA CTGcaggttc aggggCGCATG
P848678 206 AATGTGGcGA TCAA---GCC AAGAAAACCA ATAA------ ----CGCATG
P848679 208 AATGTGGaGA TGAA---GCA AAGAAAATCA ATGA------ ----CGCATG
            ******* ** ****   *** ********** ****           ******
            ******* ** ****   *** ********** ****           ******
            ******* ** ****   *** ********** ****           ******
            *******                                         ******

alignment
position    301....... 311....... 321....... 331....... 341.......

P848677 252 CGCATTT-CC gctccttgcc ggttTTCCGG ACCTCTACGA GTGGCTGCCC
P848678 243 CGCATTTcCC CCACTTCACC GATCTTCCGG ACCAACATGA ATAGCTGTCC
P848679 245 CGCATTT-CC CCACTTCAAC GATCTTCCGG CCCACCATGA ATAGCTGTCC
            ******* ** ********** ********** ********** **********
            ******* ** ********** ********** ********** **********
            ******* ** ********** ********** ********** **********
            ******* ** ********** ********** ********** **********
                    **                ****** ********** **********
                                      ****** ********** *********

alignment
position    351....... 361....... 371....... 381....... 391.......

P848677 301 CACAAAATGC CTGCtTCTCT GCGGAATCCT ACTGTTCTTC ACATGCTCTC
P848678 293 CAAGTAATGC CTGC-TCCCT GAGGAATCTC AGGATTCTTC ACGTGTTATC
P848679 294 CAAGTTATGC CTGG-TTCCT GAGGAATCTC GTTATTCTCC ACGTGTTATC
            ********** **** ***** ********** ********** **********
            ********** **** ***** ********** ********** **********
            ********** **** ***** **********    ******* **********
            ********** ****    ** **********
            ********** ***

alignment
position    401....... 411....... 421....... 431....... 441.......

P848677 351 Taataccatc -TT-TTCATA TCCACTTTCT CTTCCATGTT GAAAAATTAA
P848678 342 GCGCCTCATT TTttTTTATA TCTAGGTTTT TACTTGgagT GAACAAAGTA
P848679 343 TTGCCTCATT TTA-TTTATA TCTACGTTTT TACTTGTGCT GAACAAAGTA
            ********** *** ****** ********** ********** **********
            ********** **  ****** ********** ******   * **********
            *                                         * **********
                                                      * **********
                                                      * **********

alignment
position    451....... 461....... 471....... 481....... 491.......

P848677 399 ATtgacaggc tggattctgc aaagatcttt ggacatttaa gtatcttcg-
P848678 392 AACTGACTAA TGAGATGCCA CAAACAgGGA ATCAATACCT ATATGTTTTG
P848679 392 ATCTGACTAA TGAGATGCCA CAAACAcGGA AGCAATGCCT ATATATTTTG
            ********** ********** ****** *** ********** **********
            ********** ********** ****** *** ********** **********
            ********** ********** ****** *** ********** **********
```

```
            * * * * * * * * * *   * * * * * * * * * *   * * * * * *
            * *

alignment
position    5 0 1 . . . . . . .   5 1 1 . . . . . . .   5 2 1 . . . . . . .   5 3 1 . . . . . . .   5 4 1 . . . . . . .

P848677 448 - A C C G G C G C G   A A A A G A G G C G   G C C T G A C C T T   G G A A G T G G G a   c - G G G G T C C T
P848678 442 A A C A G G C A C A   G A G A G G G G C A   G C C T G A C C T T   G G A A A T G G G G   A A G G G G T C C T
P848679 442 A A C A G G C A T A   G A G - G G G G C A   G C C T G A C C T T   A G A A G T G G G G   A A G G G G T C C T
            * * * * * * * * * *   * * * * * * * * * *   * * * * * * * * * *   * * * * * * * * * *   * * * * * * * * * *
            * * * * * * * * * *   * * * * * * * * * *   * * * * * * * * * *   * * * * * * * * * *   * * * * * * * * * *
            * * * * * * * * * *   * * *   * * * * * *   * * * * * * * * * *   * * * * * * * * * *   * * * * * * * * * *
              * * * * * * * * *   * * *   * * * * * *   * * * * * * * * * *   * * * * * * * * * *   * * * * * * * * * *
                                        * * * * * *   * * * * * * * * * *   * * * * * * * * *         * * * * * * * *
                                            * * * *   * * * * * * * * * *   * * * * * * * * *         * * * * * * * *
                                            * * * *   * * * * * * * * * *   * * * * * * * * *         * * * * * * * *

alignment
position    5 5 1 . . . . . . .   5 6 1 . . . . . . .   5 7 1 . . . . . . .   5 8 1 . . . . . . .   5 9 1 . . . . . . .

P848677 496 G C A G C G G G T C   C T T C C G G c G G   G T G A C A T T C A   G C C G G C G G T T   C G G G G C G A C G
P848678 492 G C C G C G G G T C   C T T C C G G - G G   G T G A C A T T C A   G C C G G C G G T T   C G G G G A G A C G
P848679 491 T C C G C G G G T C   C T T A C G G - G G   G T G A C A T T C A   G C C G C T G G T T   C G G G G A G A C G
            * * * * * * * * * *   * * * * * * *   * *   * * * * * * * * * *   * * * * * * * * * *   * * * * * * * * * *
            * * * * * * * * * *   * * * * * * *   * *   * * * * * * * * * *   * * * * * * * * * *   * * * * * * * * * *
            * * * * * * * * * *   * * * * * * *   * *   * * * * * * * * * *   * * * * * * * * * *   * * * * * * * * * *
            * * * * * * * * * *   * * * * * * *   * *   * * * * * * * * * *   * * * * * * * * * *   * * * * * * * * * *
              * * * * * * * * *   * * * * * * *   * *   * * * * * * * * * *   * * * * * * * * * *   * * * * * * * * * *
              * * * * * * * * *   * * * * * * *   * *   * * * * * * * * * *   * * * * * * * * * *   * * * * * * * * * *
              * * * * * * * * *   * * * * * * *   * *   * * * * * * * * * *   * * * *     * * * *   * * * * * * * * * *
                                                  * *   * * * * * * * * * *   * * * *     * * * *   * * * * * * * * * *
                                                  * *   * * * * * * * * * *   * * * *     * * * *   * * * * * * * * * *
                                                                                          * * * *   * * * * * * * * * *

alignment
position    6 0 1 . . . . . . .   6 1 1 . . . . . . .   6 2 1 . . . . . . .   6 3 1 . . . . . . .   6 4 1 . . . . . . .

P848677 546 G A C T C T C C a t   t c c a g a A C C A   T G G C C C A A T T   T G T C C G T A A C   C T T G T G G A G A
P848678 541 T A C C T T C C A C   C T T A G - A C C A   T G G C C A A G T T   C A T C C G T A A C   T T C G C G G A G A
P848679 540 A A C C T T C C A C   C T T A G - A C C A   T G G C C A A G T T   C A T C C G T A A C   C T C G C G G A C A
            * * * * * * * * * *   * * * * *   * * * *   * * * * * * * * * *   * * * * * * * * * *   * * * * * * * * * *
            * * * * * * * * * *   * * * * *   * * * *   * * * * * * * * * *   * * * * * * * * * *   * * * * * * * * * *
            * * * * * * * * * *   * * * * *   * * * *   * * * * * * * * * *   * * * * * * * * * *   * * * * * * * * * *
            * * * * * * * * * *   * * * * *   * * * *   * * * * * * * * * *   * * * * * * * * * *   * * * * * * * * * *
            * * * * * * * * * *   * * * * *   * * * *   * * * * * * * * * *   * * * * * * * * * *   * * * * * * * * * *
            * * * * * * * *                   * * * *   * * * * * * * * * *   * * * * * * * * * *   * * * * * * * * * *
            * * * * * * * *                   * * * *   * * * * * * * * * *   * * * * * * * * * *   * * * * * * * * * *
            * * *                             * * * *   * * * * * * *
            * * *
            * * *

alignment
position    6 5 1 . . . . . . .   6 6 1 . . . . . . .   6 7 1 . . . . . . .   6 8 1 . . . . . . .   6 9 1 . . . . . . .

P848677 596 A G a c c C C G G C   G C T G G T G A A C   G G T G A G C G C g   a t g t g a g a c c   g c c g a g g c g g
P848678 590 A G G C A C C G T C   G A T G G T A G C C   G G T G A G C G C A   G C C C G G G A G A   G g c T - - - - - -
P848679 589 A G G C A C C G T C   G A T G G T G G C G   G G T G A G C G C A   G C G C G G G A G A   G a a T - - - - - -
            * * * * * * * * * *   * * * * * * * * * *   * * * * * * * * * *   * * * * * * * * * *   *     *
            * * * * * * * * * *   * * * * * * * * * *   * * * * * * * * * *   * * * * * * * * * *   *     *
            * * * * * * * * * *   * * * * * *   * * *   * * * * * * * * *                                 *
            * * * * * * * * * *   * * * * * *                                                             *
            * *       * * * * *   * * * * * *
            * *
```

```
            **

alignment
position    701....... 711....... 721....... 731....... 741.......

P848677 646 gcacacgggc ggcagggagg cctgcgatgg CCTGAGAGGA AGAAGCGGGC
P848678 634 ---------- ---------- ---------- CGCGAGGGGA GGGATTGTGC
P848679 633 ---------- ---------- ---------- CGTGAGGGGA AGGAGTGTGC
                                             ********** **********
                                             ********** **********
                                             ********** **********
                                             ********** **********

alignment
position    751....... 761....... 771....... 781....... 791.......

P848677 696 TGCGaagacC CGAGGGGCCT GCGGGTGCCG GGGCGGgatg gcctgcaggt
P848678 654 TGCGGCA--C CAAGGGGCCg t-GGGCGTCG GGAGGGCttC CAGGAGGCCA
P848679 653 TGCGGCA--C CAAGGGGCCC GCGGGCGTCG GGAGGGCcaC CAGGAGGCAA
            *******  * ********** ********** *******  * **********
            *******  * *********    ******** ******   * **********
            *******  * *********                      * **********
            *******  * *********

alignment
position    801....... 811....... 821....... 831....... 841.......

P848677 746 ggagggagca ggaagcaggt tggcgactct tttctagcct tccaCTCGTC
P848678 701 CTGTAAGGCC GCAGC----- ---------- ---------- ----CTCGCC
P848679 701 CTGCAAAGCC CCAGT----- ---------- ---------- ----CTCGCC
            ********** *****                                ******
            ********** *****                                ******
            ********** *****                                ******

alignment
position    851....... 861....... 871....... 881....... 891.......

P848677 796 TGACCTGCag attgggttct ctacACAACC TAGAAGTTCC TGAATTCTAA
P848678 722 TGGCCTGCcc gcGGCTGCTT ----AGAGCT CACAAGCTGC AGGGTCCTAG
P848679 722 TGGCCTGtcc ctGGCTGCCT ----AGGGCG CACAAGCTGT TGGGCCCTAA
            ********     ********     ****** ********** **********
            *******      ********     ****** ********** **********
            *******

alignment
position    901....... 911....... 921....... 931....... 941.......

P848677 846 CCACGACTGG GAAGAGA--G GTAGTGTGaa ggaaGCCCGG CGTTGCCCGG
P848678 768 CTCGGCCGGG GAgcgagtgG GTCGTGTaac tc-TGCCCGG CCTCACTCCG
P848679 768 CCGGGCCTGT TAAGTGT--G GTCGTGTGgt tttTGCCCGG CCTCACTCCG
            ********** *******  * ********      ******* **********
            ******                                 ******* **********
                                                   ******* **********
                                                   ******* **********

alignment
position    951....... 961....... 971....... 981....... 991.......

P848677 894 CAGCTGCAAC CATTACGCCC CCTGagatcg ctaagagcag ggatctagag
P848678 817 CCGCGAACGC CGTGATGCCC CCTTACCGTG CCG------- ----------
P848679 816 CCGCGAACGC CATGACGCCC CCTTACCGTG CCG------- ----------
            ********** ********** ********** ***
            ********** * ******** ********** ***
            ********** * ******** ********** ***
```

```
                    ********** *  ******** ****

alignment position  1001...... 1011...... 1021...... 1031...... 1041......
P848677  944        gggatggttc tgttaGGAGA GCTAAGAAAA GGAAGgg--- ----------
P848678  850        ---------- -----GGAGA GTTGGGAAGG Gaggt----C GGTAACAGGG
P848679  849        ---------- -----GGAGA TCTAGGAAGG GGAGGacgaC GGTAACAGGG
                                    ***** ********** *****    * **********
                                    ***** ********** *        * **********
                                    ***** ********** *

alignment position  1051...... 1061...... 1071...... 1081...... 1091......
P848677  981        ---------- ---------- ---------- ---------- ----------
P848678  881        GAGAGagagG TCACACATCA GACCTCATAG CTGGTCTGCG TCTCAGCGAa
P848679  884        GAGAG----G TCACACATCA GACCTTATAG CTGATCTGGG TCTCAGTGAa
                    *****    * ********** ********** ********** *********
                    *****    * ********** ********** ********** *********
                             * ********** ********** ********** *********
                             * ********** ********** ********** *********

alignment position  1101...... 1111...... 1121...... 1131...... 1141......
P848677  981        ---------- ------CAGA AACGTGAAA- ---------- ------GTAA
P848678  931        gta-TTTGAA AGCCATCAGA AATTTTAAAg acgggtgaga gatcttTGAA
P848679  930        gttgTTAGAA AGTCACCAGA AATTTTAAA- ---------- ------TGAA
                        ****** ********** *********                 ****
                        ****** ********** *********                 ****
                                                                    ****
                                                                    ****

alignment position  1151...... 1161...... 1171...... 1181...... 1191......
P848677  998        Atca------ ---------- ---------- ---------- ----------
P848678  980        AGTTATGTTT GCTTACCATC CTGGTTAGAG CCTAGAgaat tctgcgtccg
P848679  963        AGTTATGTTT GCTTACCATC CTGGTCAGAT GCTAGAgaa- ----------
                    ********** ********** ********** ******
                    ********** ********** ********** ******
                    ********** ********** ********** ******
                    ********** ********** ********** ******

alignment position  1201...... 1211
P848677 1002        ---------- ---
P848678 1030        atgaagggaa ttt
P848679 1002        ---------- ---
```

Pairwise similarities:

For each pairwise alignment, the similarity (relative to the maximum similarity) and the number of identical nucleic acids (in % of shorter sequence) is given. Maximum values are underlined.

	**P848678** (1042 bp)	**P848679** (1001 bp)

**P848677 (1001 bp)**	0.175 42 %	0.166 42 %
**P848678 (1042 bp)**		1.000 84 %

Please note that the **similarity value 1.000** marks only the two **most similar** sequences, it does not necessarily mean that these sequences are identical.

**Extract alignment region**	
**Positions in alignment**	from: to:
**Output file**	dialign.seq

Extract aligned sequences

For comments, questions or bug reports, please contact support@genomatix.de

© Genomatix Software GmbH 1998-2005 - All rights reserved.

License Agreement

GEMS Launcher 4.1.1

Genomatix-Logo Logout Personal Messages GEMS-Logo
Logout Personal Messages

Genomatix_Main GEMS ElDorado Gene2Promoter BiblioSphere
Frequently_asked_questions Your Results Your Sequences Your_Protocol Help

GEMS Launcher Task: *DiAlign TF*: Multiple alignment plus TF sites working on promoters.seq (3 seq.)

[Alignment] [Pairwise_similarities]

DiAlign professional TF Release 3.1 December 2004 **Wed Jun 1 23:14:51 2005**

Solution parameters:

**Sequence file:**	promoters.seq (3 seq.)
**Type of sequences:**	nucleotide sequences
**Threshold T:**	0.00
**'*' signs below alignment denote:**	diagonal similarity (max. similarity is represented by 10 '*' signs)
**Alignment output:**	complete alignment is shown<br># nucleic acids per line: 50
**TF output:**	common TF matches located in aligned regions (common to 3 (100.0 %) sequences)
**Family matches:**	yes
**MatInspector library:**	Matrix Family Library Version 5.0 (February 2005)
**Selected groups** (core/matrix sim):	• ALL user_defined.lib (0.75/Optimized)<br>• ALL vertebrates.lib (0.75/Optimized)

Aligned Sequences:

No.	Sequence Name	Sequence Description	Length
1	P978665	sym=ATP5O|loc=Loc539|taxid=9606|spec=Homo sapiens|chr=21|ctg=NT_011512|str=(-)|start=20948942|end=20950528|len=1587| tss=501,587| comm=ATP synthase, H+ transporting, mitochondrial F1 complex, O subunit (oligomycin sensitivity conferring protein); (AK124187/587/gold;NM_001697/501/silver;)	1587 bp
2	P978667	sym=Atp5o|loc=Loc28080|taxid=10090|spec=Mus musculus|chr=16|ctg=NT_039625|str=(-)|start=26829146|end=26830647|len=1502| tss=501,502| comm=ATP synthase, H+ transporting, mitochondrial F1 complex, O subunit; (AK005309/520/gold;NM_138597/519/bronze;)	1502 bp
3	P978669	sym=Atp5o|loc=Loc192241|taxid=10116|spec=Rattus norvegicus|chr=11|ctg=NW_047354|str=(-)|start=31958531|end=31960031| len=1501|tss=501| comm=ATP synthase, H+ transporting, mitochondrial F1 complex, O subunit (oligomycin sensitivity conferring protein); (NM_138883/585/bronze;)	1501 bp

Alignment (DiAlign format):

Please note that only **upper-case** letters are considered to be **aligned**. For more information, please have a look at the user guide.

V$STAF V$NRF1 U$NRF1 V$RORA V$YY1F V$NEUR V$NFKB V$HOXF V$TBPF V$MEF2 V$FKHD V$PERO V$COUP

```
alignment
position        1.........  11........ 21........ 31........ 41........

P978665     1   cctatac--- --------CC ATCGCTATGG TCCTGAATAA AGTCTTCCTT
P978667     1   ataatataat aataa----- ---------- ---------A TCTTTGGGCT
P978669     1   acatcatctt tgattaaaCC CTGATTCTAG TTCTGGATTA ACTCTGTCCT
                                   ** ********** ********** **********

alignment
position        51........ 61........ 71........ 81........ 91........

P978665    40   ATCATGCttt aataagcatc actgaataat tctttattta acaatgggtA
P978667    27   GGAGCGAGCA GAGATCCAAA AACAAAAAtt aaatccccaa caaccacatg
P978669    51   GAAATGCACA CAGAGTCAAA ATCTAACA-- ---------- ---------A
                ********** ********** ********                     *

alignment
position        101....... 111....... 121....... 131....... 141.......

P978665    90   GTCATGGCCC AGGTAAGAGA GAcgtggtgt ccgctcctgg agctgcaagg
P978667    77   aaggctcaca accatctgta cagctacagt atactcatat acatgaagta
P978669    80   GTCAGGGCTA TGATAAGACT GAgaaggaaa gggaaaacta tca-------
                ********** ********** **

alignment
position        151....... 161....... 171....... 181....... 191.......

P978665   140   ctcctggggg cttttttgttt tg-------- ---------- ----------
P978667   127   aataaagaaa tctaaaaaaa gaaaaaaaga aaaagcacaa acacacctgt
P978669   123   ---------- ---------- ---------- ---------- ----------

alignment
position        201....... 211....... 221....... 231....... 241.......

P978665   162   ---------- ---------- ---------- ---------- ----------
P978667   177   caactgtcct atgtaatcaa aatatGGCCA GAAGAGCCAA Gaagtgcg--
P978669   123   ---------- ---------- -----GGCCA GAAGAGCCAA Gcaagacgtt
                                           ***** ********** *

alignment
position        251....... 261....... 271....... 281....... 291.......

P978665   162   ---------- --------TT TTGGTTTTG- -TCTTTGTAT GCAACGTGAC
P978667   225   ---CCCGGGT AGGTTCACTT TTGGTTTTT- -GTTTTAAGT AAAGCCTGAA
P978669   148   atcCCCTGGG AGATTCGCTT GTGGTTTTTt tGTTTTAAGT AAAGCCTGAA
                   ******* ********** *********   ********* **********
                                                  ********* **********
                                                  ********* **********
                                                  ********* **********

alignment
position        301....... 311....... 321....... 331....... 341.......

P978665   192   AAAAAGCCAt tgatgcaggc tttagcatgc tgggaaggaa gTGTAAAGAA
P978667   270   GCATTGCCAC AGAAGCACAC T-TGGCAGGT ---------- -TCAAGACGA
P978669   198   GCATTGTCAC AGAACCACAC TtTGACAGGT ---------- -TCTAGACAT
```

```
                     ********** ********** * ******** ********** *********
                     ********** ********** * ******** ********** *********
                     ********** ********** *
                     ********** ********** *

alignment position   351....... 361....... 371....... 381....... 391.......

P978665  242         GCCAGTGACG CTGGTAATAA CTGTGAAATG gaacaaatgc caccatattt
P978667  308         GTCAACGATG TTGACGATGC ATgTGCAAAG A--------- ----------
P978669  237         GGCAGAGATG TTGACGATGC ATATGCAAAG A--------- ----------
                     ********** ********** ********** *
                     ********** ********** **  ******* *
                                              ******* *

alignment position   401....... 411....... 421....... 431....... 441.......

P978665  292         ATCTAAATGT AAAttttaaa actggggaca gcaaatccat tctacagtga
P978667  339         GTTTAAATCT AAACCTAATT TCA------- ---------- ----------
P978669  268         ATCTAAATGT AAACCTAGTT TCA------- ---------- ----------
                     ********** ********** ***
                     ********** ********** ***
                     ********** ********** ***

alignment position   451....... 461....... 471....... 481....... 491.......

P978665  342         gcgccgcttg gccagtaaca atGACTTCAT TT-----TCa gatgcagtcg
P978667  362         ---------- ---------- --GACTTAAT TT-----TC- ----------
P978669  291         ---------- ---------- --GATTTAAT TttccgaTC- ----------
                                             ******** **     **
                                             ******** *      **
                                             ******** *

alignment position   501....... 511....... 521....... 531....... 541.......

P978665  387         aagccctgTG GCTCCACCCC GAGGGTCCAG CCttaccact g--CTAAGA
P978667  374         --------TG GTTCCGGTTC ACGAGTCCAG CCGGACCGCG GCAggtacGC
P978669  308         --------TT GTTCGGGTTC AAGAGTCTAG CGGGACCACG GCAcCTATGC
                             ** ********** ********** ********** *** ******
                             ** ********** ********** ********** ***     **
                                                                         **
                                                                         **
                                                                         **
                                                                         **

alignment position   551....... 561....... 571....... 581....... 591.......

P978665  435         ACTACAACTC CCAGCCCGAG GGAGGTctct gcgcctgcgg cctgacgccc
P978667  416         ACTACAACTC CCAGCATGCA AGAGGTTCTT ACCCACACtc ccgGGCTCCT
P978669  350         ACTACAACTC CCAGCGTGCA AGAGGTTCTT ACCCACACgc cccGGCTCCT
                     ********** ********** ********** ********      *******
                     ********** ********** ********** ********      *******
                     ********** ********** ********** ********      *******
                     ********** ********** ********** ********      *******
                     ********** ********** ********** ********
                     ********** ********** ******

alignment position   601....... 611....... 621....... 631....... 641.......
```

```
P978665  485 tACGCCTACA CTTCCCAGAG AACCTAGCGG TTACGCCAA- -CGCGCGCGT
P978667  466 GACGCGTACA TTTCCCAGAA AGCCGAGCGA TCACGCtggc gCGCGCGCGT
P978669  400 GGTGCGTTCA TTTCCCAGAA AACCGAGCGA TCACGCCGA- -CGCGCGCAT
             ********** ********** ********** *********   *********
             ********** ********** ********** *********   *********
             ********** ********** ********** *********   *********
             ********** ********** ********** ******      *********
              ********* ********** ********** ***         *********
                         ********* ********** ***         *********
                         ********* ********** ***

alignment
position     651....... 661....... 671....... 681....... 691.......

P978665  533 GCGCCCTTGC GCgtttc-TC TCTTCCCACT CGGGTTTGAC CTACAGCCGC
P978667  516 GCGCTCTGGC GCCAGTAGTC TCTTTTCATT TGGGTTTGAC CTACAGCCGC
P978669  448 GCGCTCTGGC ATCACTAGTC TCTT---ACT TGGGTTTGAC CTACAGCTGC
             ********** ********** ********** ********** **********
             ********** ********** ********** ********** **********
             ********** **      ** ********** ********** **********
             ***                ** ****   *** ********** **********
             ***                ** ****   *** ********** **********
             ***                          *** ********** **********
                                            * ********** **********
                                            * ********** **********
                                            * ********** **********
                                            * ********** **********

alignment
position     701....... 711....... 721....... 731....... 741.......

P978665  582 CCGGGAGAAG ATGGCTGCCC CAGCAGTGTC CGGGCTCTCC CGGCAGGTga
P978667  566 CCGGGAAAAG ATGGCCGCGC CTGCAGCGTC CGGACTGTCT CGACAGGTAC
P978669  495 CCGGGAAAAG ATGGCCGCAC CAGCAACATC CGTGCTGTCC CGACAGGTAC
             ********** ********** ********** ********** **********
             ********** ********** ********** ********** **********
             ********** ********** ********** ********** ********
             ********** ********  * ********** ********** *******
             ********** ********  * ********** ********** *******
             ********** ********
             ********** *****  **
             ********** *****
             ******  ** *****
             ******

alignment
position     751....... 761....... 771....... 781....... 791.......

P978665  632 gagaaaggtg gtcctgagag ccaGTGGGAG GATCCCTCCT GGGATGACCC
P978667  616 AAGCTG---- ---------- ---GCGGGAG GCTCCCTCCC AGGGTTGCGC
P978669  545 AAGCTGa--- ---------- ----CGGGAG GGTCCCTCCT GGGGTTCCCC
             ******                   ******* ********** **********
             ******                    ****** ********** **********
                                       ****** ********** **********
                                        ***** ********** **********

alignment
position     801....... 811....... 821....... 831....... 841.......

P978665  682 GTACCaaacc tcgagcacCA GGGTGCGATG CTTCGGCTGG GGaagtctgg
P978667  649 GTCCCCGGCT CCCAtCG-CC GCGTGTGAGG CCGTGGCTGA GGGGTCAGGC
P978669  578 GTCCCCGACT CCCAgCG-CT GCGTGTGAGG CTGTGGCTGA GGGGTCAAGC
```

```
            ********** ****  **   ** ********** ********** **********
            ********** ****  **   ** ********** ********** **********
            ********** ****  **   ** ********** ********** **********
            *****

alignment
position    851....... 861....... 871....... 881....... 891.......

P978665 732 cagtctt-CA AGGTTATGGG ACCTGGCGAG TGggagcgac tcaggacgac
P978667 698 AGCCCgcCCA GGGTCGTGGG GCCCGGCGAC TG-------- ----------
P978669 627 CGCCCa-CCA GGGTCGTGGG GCCCGGCCAT Tt-------- ----------
            *****   *** ********** ********** **
            *****   *** ********** *******
            *****

alignment
position    901....... 911....... 921....... 931....... 941.......

P978665 781 ccagcgtttg tccccgggga tgcctacggg gCAGTAGGCG CGGGGCTGcg
P978667 730 ---------- ---------- ---------- -CAGGCAGCG TGGGGCGGAT
P978669 658 ---------- ---------- ---------- -CAGGAAGCG TGGGGCTGAT
                                          ********* **********
                                          ********* **********

alignment
position    951....... 961....... 971....... 981....... 991.......

P978665 831 cgtgccgtaa ccttggcgtc ttccgagcac agcccctgga agctgctagc
P978667 749 CCGCGTTTCG TCCCC-GT-- ---------- ---------- ----------
P978669 677 TCGCGTTTTT TCCCCcGT-- ---------- ---------- ----------
            ********** *****  **
            ********** *****  **
                              **
                              **

alignment
position    1001...... 1011...... 1021...... 1031...... 1041......

P978665 881 ctgaggggcg caggagaggg ggtctgccct tcgctgaatt ccGGGGAAGC
P978667 766 ---------- ---------- ---------- ---------- --GGGGAGTC
P978669 695 ---------- ---------- ---------- ---------- --GGGGAGTC
                                                      ********
                                                      ********
                                                      ********
                                                      ********

alignment
position    1051...... 1061...... 1071...... 1081...... 1091......

P978665 931 GGCATGGACT agcccccaaa tcagctt--- ---------- ----------
P978667 774 GGCACGGATT TCTCTGTCTT GACCTTGACA tccaggactc acTGCATAGC
P978669 703 GGCACGAATT GCGCTATCTT GACCTTGACA tccaagagtc gaTGCATAGC
            ********** ********** **********              ********
            ********** ********** **********              ********
            ********** ********** **********              ********
            ********** ********** **********

alignment
position    1101...... 1111...... 1121...... 1131...... 1141......

P978665 958 ---------- ---------- ---------- ---------- -----CCTTT
P978667 824 GGAGCAGCTG GCAAGGGCAT GGGAGAGAGc acTGGGGAGA GTACATGCCT
P978669 753 TGAGCAGCTG GCAAAAGCAT GGGAGAGAGa atTGGGGAAA GCACACGTCT
```

```
             ********** ********** *********     ******** **********
             ********** ********** *********     ******** **********
             ********** ********** *********

alignment
position     1151...... 1161...... 1171...... 1181...... 1191......

P978665  963 GCCATTGTGG TAAAACCTGT CAGAGATCAC Ttactagtaa tgctaagGAA
P978667  874 GTCCTTGCTG GTAAACCTa- -AGGGCTGCC TGCTGCTTag gatt-TTGAC
P978669  803 AGCCTTGTTC GTAAACCTGC CAGGGCTAGC TGCTGCTTgg cagtaTTGAC
             ********** ********** ********** ********       *****
             ********** ********                             *****
                                                             *****
                                                             *****

alignment
position     1201...... 1211...... 1221...... 1231...... 1241......

P978665 1013 TATTTGTAAA ACACATTTCA CAGTTTTATA AATAAaatga ggccgaaaag
P978667  921 TGTTGGAAAA GCGCATCCCC TGTGTTTATA AAGAAggggg gAAGGGCTAA
P978669  853 TGTTCGTAAA GCGCATTCCA TGTGTTTATA AAGAAggaag aAAGGGTTAA
             ********** ********** ********** *****       *********
             ********** ********** ********** *****
             ********** ********** ********** *****
             ********** ********** ********** *****

alignment
position     1251...... 1261...... 1271...... 1281...... 1291......

P978665 1063 tttaaatgag TCTTTGGCTG ctCAGTCAGT AAGTGGTAGA A---TTGaga
P978667  971 ACgtCTCC-- CCTTTGGCTG G-CAGCTGGT GAGTGATGgc gagcTTGCAA
P978669  903 AC--CTCT-- TCTTTGGCTG G-CAGCCAGT AAGTGGTGAG A---TTGCAA
             **  ****   ********** *  ******** ********** *   ******
                        **********    ******** ********       ******
                                                              ******
                                                              ******
                                                              ***

alignment
position     1301...... 1311...... 1321...... 1331...... 1341......

P978665 1110 tttgaaccca cggaatcacc cagaagatcg agtttgaaaa aatgttatgc
P978667 1018 CAGGCCAAGT CCAGCAGAAC CTGGAATTGG GTGGaaaaaa atacgtgcct
P978669  945 CGGGCCAAGT CCAGCAGAAA CTGGAATTGG GTGGa----- ----------
             ********** ********** ********** ****
             ********** ********** ********** ****
             ********** ********** ********** ****
             ********** ********** ********** ****

alignment
position     1351...... 1361...... 1371...... 1381...... 1391......

P978665 1160 tgtttgtgct gcatctcacc atggtgcttt acatatcatg accccttcct
P978667 1068 gggTCATCTG CCATGATAAT TTTgtatcga cagccgtcat ggttttcatc
P978669  980 ---TCATCTG CCCTGATGAT TTTaattt-- ---------- ----------
                ******* ********** ***

alignment
position     1401...... 1411...... 1421...... 1431...... 1441......

P978665 1210 ggcatcttag GTAATTTTTT CATAAgcatt ---------- ---AAAAAGT
P978667 1118 tgcaccata- ---------- CAGAATTATT ACTAAGGCGT TAGAAAGtTT
P978669 1005 ---------- GTACTTAATT CATAATTTTT ATCAAGGCCT GAGAAAGCTT
```

```
                          ********** ********** ********** **********
                                                                  **
                                                                  **
                                                                  **

alignment position     1451...... 1461...... 1471...... 1481...... 1491......

P978665 1247           CTTAATCCTC ACAAGCAGTT TTGATAAAAC ACGTTCCAta gctggagtat
P978667 1157           TTTAACCCTC ATAGCCAGTT CCAATCGAGT ACATTCTAgt CTAGAGAGAC
P978669 1045           TTTAACCCTC ATAGCCAGTT CTAGTCGAGT ACAcc----- CTAGAGATGC
                       ********** ********** ********** ********   **********
                       ********** ********** ********** ***
                       ********** ********** ********** ***
                       ********** ********** ********** ***

alignment position     1501...... 1511...... 1521...... 1531...... 1541......

P978665 1297           ttgaaggtga cctgtgatCT GTGACCTGTG CGCTTGCA-G GTGATTTTTA
P978667 1207           TAGTATAtGA --------CT GTGACCTGTG CCCTCGCAAA ACGCTTTTTA
P978669 1090           GAGTATA-GA --------CA GTGACCTGTG CCCTTGAGAG GTGCTTTTTA
                       *******  **         ** ********** ********** **********
                               **          ** ********** ********** **********
                                           ** ********** *******

alignment position     1551...... 1561...... 1571...... 1581...... 1591......

P978665 1346           AACTGtgtat tccagctctg tcttttgatg ggcaggatag tgtctctcag
P978667 1249           AACTGgATTC C--------- ---------- ---------- ----------
P978669 1131           AAtggcATTC C--------- ---------- ---------- ----------
                       *****  ****  *
                       **     ****  *
                              ****  *
                              ****  *

alignment position     1601...... 1611...... 1621...... 1631...... 1641......

P978665 1396           aactaAGGCT TCTtgtcaga tcaaactctg cggcagtact caaaa-----
P978667 1260           -----AGGCT GCTCCAGTGA CAGACACAGT GCCTGaagcc tgGTGCTGTA
P978669 1142           -----AGGCT TCTCCAGTGA CAGACTCAGT GCCTG----- --GTGCTATA
                            ***** ********** ********** *****        ********
                            ***** ********** ********** *****        ********
                            ***** ********** ********** *****
                            ***** ********** ********** *****

alignment position     1651...... 1661...... 1671...... 1681...... 1691......

P978665 1441           --AATTCATC TTTATTTTCA GATAGCATTT GCATTCCgag gagggtacgt
P978667 1305           TCAAGAGATC TTTGGTCTTA GATAGCATTT CAGTTCCTAA GCAAGTAGGT
P978669 1180           TGAAAAGATC TTTGGTGTTT GATATCACTT CAATTCCTTA GCACATAGGT
                       ********** ********** ********** ********** **********
                       ********** ********** ********** ** ******* **********
                         ******** ********** ********** ** ****

alignment position     1701...... 1711...... 1721...... 1731...... 1741......

P978665 1489           ctgttgca-- ---------- ---------- -------AAT GATCTGCTCA
P978667 1355           TTCCTTGAAT TAAGACTGGA CTCatttcAT GTTCATTAAA GTTTTTCTCA
P978669 1230           TTACTCGAAT CAAGGCTGGC CTCctcttAT GTTCATTAAA GTTTTTCCCA
```

```
                   ********** ********** ***       ** ********** **********
                   ********** ********** ***       ** ********** **********
                                                   ** ********** **********

alignment position 1751...... 1761...... 1771...... 1781...... 1791......
P978665 1510       AGGGTCTGTT -AATAGAAAc gccaaaattt tggt------ ----------
P978667 1405       AAGGTGATGT -AATATAAAT CCTCCGCTTT CATAAAGCAT TctttccCAAA
P978669 1280       AAGGTGATag aAATATAAAT CCTCCACTTT GCTAAAGCAT TttcctCAAA
                   **********  ********* ********** ********** *     ****
                   ********    ********* ********** ********** *     ****
                   ********

alignment position 1801...... 1811...... 1821...... 1831...... 1841......
P978665 1543       ---------- ---TAAA--- ------TATT TTTGAAACAT GCACTCCTTC
P978667 1454       TGTGATAAAG CTCTTAA--- ------TTAT TTTGAAAAAT TCCACTTTGC
P978669 1330       TGTGATAAAG CTCTTgactt ttttttTTTT TTTTTTAAAT TCAATTTTGA
                   ********** *******          **** ********** **********
                   ********** *****            **** ********** **********

alignment position 1851...... 1861...... 1871...... 1881...... 1891......
P978665 1571       GAAGTTAGTG TTTTtct--- ---------- ---------- ----------
P978667 1495       GAAGTTAG-- ---------- ---------- ---------- ----------
P978669 1380       GAAGCTAGCA TTTTcttttct caactaccca gggaggaagg cagttgaaag
                   ********** ****
                   ********

alignment position 1901...... 1911...... 1921...... 1931...... 1941......
P978665 1588       ---------- ---------- ---------- ---------- ----------
P978667 1503       ---------- ---------- ---------- ---------- ----------
P978669 1430       acagtttctg aagcagtttg gggtgactct caatagggaa tgtataggta

alignment position 1951...... 1961...... 1971
P978665 1588       ---------- ---------- --
P978667 1503       ---------- ---------- --
P978669 1480       ataaggagca gttcccagtc at
```

Pairwise similarities:

For each pairwise alignment, the similarity (relative to the maximum similarity) and the number of identical nucleic acids (in % of shorter sequence) is given. Maximum values are underlined.

	P978667 (1502 bp)	P978669 (1501 bp)
P978665 (1587 bp)	0.150<br>31 %	0.152<br>31 %
P978667 (1502 bp)		1.000<br>67 %

Please note that the **similarity value 1.000** marks only the two **most similar** sequences, it does not necessarily mean that these sequences are identical.

**Extract alignment region**	
**Positions in alignment**	from: to:
**Output file**	dialign.seq

Extract aligned sequences

For comments, questions or bug reports, please contact support@genomatix.de

© Genomatix Software GmbH 1998-2005
- All rights reserved.

License Agreement

GEMS Launcher 4.1.1

Genomatix-Logo Logout Personal Messages GEMS-Logo
Logout Personal Messages

Genomatix_MainGEMSElDoradoGene2Promoter BiblioSphere
Frequently_asked_questions Your ResultsYour SequencesYour_ProtocolHelp

GEMS Launcher Task: *DiAlign TF*: Multiple alignment plus TF sites working on promoters.seq (3 seq.)

[Alignment] [Pairwise_similarities]

DiAlign professional TF Release 3.1 December 2004 **Fri Jun 3 22:12:33 2005**

Solution parameters:

**Sequence file:**	promoters.seq (3 seq.)
**Type of sequences:**	nucleotide sequences
**Threshold T:**	0.00
**'*' signs below alignment denote:**	diagonal similarity (max. similarity is represented by 10 '*' signs)
**Alignment output:**	complete alignment is shown<br># nucleic acids per line: 50
**TF output:**	common TF matches located in aligned regions (common to 3 (100.0 %) sequences)
**Family matches:**	yes
**MatInspector library:**	Matrix Family Library Version 5.0 (February 2005)
**Selected groups** (core/matrix sim):	• ALL user_defined.lib (0.75/Optimized)<br>• ALL vertebrates.lib (0.75/Optimized)

Aligned Sequences:

No.	Sequence Name	Sequence Description	Length
1	P973053	sym=ATP5S|loc=Loc27109|taxid=9606|spec=Homo sapiens|chr=14|ctg=NT_026437|str=(+)|start=31778252|end=31779652|len=1401| tss=601| comm=ATP synthase, H+ transporting, mitochondrial F0 complex, subunit s (factor B); (NM_015684/501/silver;)	1401 bp
2	P973055	sym=Atp5s|loc=Loc68055|taxid=10090|spec=Mus musculus|chr=12|ctg=NT_039551|str=(+)|start=14615676|end=14617088|len=1413| tss=601,613| comm=ATP synthase, H+ transporting, mitochondrial F0 complex, subunit s; (AK009141/501/gold;NM_026536/513/silver;)	1413 bp
3	P973058	sym=na|loc=Loc362749|taxid=10116|spec=Rattus norvegicus|chr=6|ctg=NW_047760|str=(+)|start=38712374|end=38713774| len=1401| comm=similar to mitochondrial ATP synthase regulatory component factor B; (CompGen promoter)	1401 bp

Alignment (DiAlign format):

Please note that only **upper-case** letters are considered to be **aligned**. For more information, please have a look at the user guide.

V$ZBPF V$SP1F V$EGRF V$MINI V$ECAT V$PCAT V$ZF5F V$GLIF

alignment position		1	1 1	2 1	3 1	4 1 . . . .
**P973053**	1	g a g c t a - - - -	- - - - - - - - - -	- - - - - - - - - -	- - - - - - - - - -	- - - - - - - - - -
**P973055**	1	t g - - - - - - - -	- - - - - - - - - -	- - - - - - - - - -	- - - - - - - - - -	- - - - - - - - - -
**P973058**	1	g a g g c a g t g g	t g a t a c a c a c	c t t t a a t c c c	a g c a a g g t g g	a g a c a a g a c a
alignment position		5 1	6 1	7 1	8 1	9 1 . . . .
**P973053**	7	- - - - - - - - - -	- - - - - - - - - -	- - - - - - - - - -	- - - - - - - - - -	- - - - - - - - - -
**P973055**	3	- - - - - - - - - -	- - - - - - - - - -	- - - - - - - - - -	- - - - - - - - - -	- - - - - - - - - -
**P973058**	51	c a g g a a a a t c	t t t g a g t t c a	a g g c c a g c c t	g g t t g a c a a g	a c a g c c a g g a
alignment position		1 0 1	1 1 1	1 2 1	1 3 1	1 4 1 . . . .
**P973053**	7	- - - - - - - - - -	- - - - - - - - - -	- - - - - - - - - -	- - - - - - - - - -	- - - - - - - - - -
**P973055**	3	- - - - - - - - - -	- - - - - - - - - -	- - - - - - - - - -	- - - - - - - - - -	- - - - - - - - - -
**P973058**	101	t a c a a a g a g a	g g c c c t a t g t	g g a a a g a c a g	t c a g a a a c a g	g c a t g a a g g g
alignment position		1 5 1	1 6 1	1 7 1	1 8 1	1 9 1 . . . .
**P973053**	7	- - - - - - - - - -	- - - - - - - - - -	- - - - - - - - - -	- - - - - - - - - -	- - - - - - - - - -
**P973055**	3	- - - - - - - - - -	- - - - - - - - - -	- - - - - - - - - -	- - - - - - - - - -	- - - - - - - - - -
**P973058**	151	g a a g g g a a a a	a g g g a g g a a g	a t g g a g a a g g	g a g g g g a g g g	g a g g g g a g g g
alignment position		2 0 1	2 1 1	2 2 1	2 3 1	2 4 1 . . . .
**P973053**	7	- - - - - - - - - -	- - - - - - - - - -	- - - - - - - - - -	- - - - - - - - - -	- - - - - - - - - -
**P973055**	3	- - - - - - - - - -	- - - - - - - - - -	- - - - - - - - - -	- - - - - - - - - -	- - - - - - - - - -
**P973058**	201	g g a g a g g a a g	a t t g g g a g a g	g t g g g g t g g c	a t g t g c c t t t	a a t t c c a g c g
alignment position		2 5 1	2 6 1	2 7 1	2 8 1	2 9 1 . . . .
**P973053**	7	- - - - - - - - - -	- - - - - - - - - -	- - - - - - - - - -	- - - - - - - - - -	- - - - - - - - - -
**P973055**	3	- - - - - - - - - -	- - - - - - - - - -	- - - - - - - - - -	- - - - - - - - - -	- - - - - - - - - -
**P973058**	251	c t t g g a a a g g	a g g g g c a g g t	t g a t c g c t t g	a g a c a t t g g a	g t c t g c c t g a
alignment position		3 0 1	3 1 1	3 2 1	3 3 1	3 4 1 . . . .
**P973053**	7	- - - - - - - - - -	- - - - - - - - - -	- - - - - - - - - -	- - - - - - - - - -	- - - - - - - - - -
**P973055**	3	- - - - - - - - - -	- - - - - - - - - -	- - - - - - - - - -	- - - - - - - - - -	- - - - - - - - - -
**P973058**	301	t c t a c a g g g c	t a a a t a g g g a	a a c c t a g t t t	c a a a g g a a a a	c a a a c c a c t a
alignment position		3 5 1	3 6 1	3 7 1	3 8 1	3 9 1 . . . .
**P973053**	7	- - - - - - - - - -	- - - - - - - - - -	- - - - - - - - - -	- - - - - - - - - -	- - - - - - - - - -
**P973055**	3	- - - - - - - - - -	- - - - - - - - - -	- - - - - - - - - -	- - - - - - - - - -	- - - - - - - - - -
**P973058**	351	c c a t t t a g c a	t g a c c a t t a g	a c t a a a a t t t	g a t g t g g c t t	a a a c a a a a g c

```
alignment position      401.......  411.......  421.......  431.......  441.......
P973053           7     ----------  ----------  ----------  ----------  ----------
P973055           3     ----------  ----------  ----------  ----------  ----------
P973058         401     aaggcgcccc  tctccagagt  ctttccacac  ctctatctgc  cactaaatcc

alignment position      451.......  461.......  471.......  481.......  491.......
P973053           7     ----------  ----------  ----------  ----------  ----------
P973055           3     ----------  ----------  ----------  ----------  ----------
P973058         451     attcgcacag  ctcaccttgc  ctcaaacaat  caaaaacttc  ccctgtaaag

alignment position      501.......  511.......  521.......  531.......  541.......
P973053           7     ----------  ----------  ----------  ----------  ----------
P973055           3     ----------  ----------  ----------  ----------  ----------
P973058         501     ctttcacaca  gaaaactgta  tttgccaagg  cagcccacgg  tggtcaactg

alignment position      551.......  561.......  571.......  581.......  591.......
P973053           7     ----------  ----------  ----------  ----------  ----------
P973055           3     ----------  ----------  ----------  ----------  ----------
P973058         551     agttgcttct  catgaggaca  actctaatgt  gtgcaaggaa  cttgacccac

alignment position      601.......  611.......  621.......  631.......  641.......
P973053           7     ----------  ----------  ----------  ----------  ----------
P973055           3     ----------  ----------  ----------  ----------  ----------
P973058         601     tgaagccaag  ttctggattt  tccaagcaac  acgggatgct  agctaagaaa

alignment position      651.......  661.......  671.......  681.......  691.......
P973053           7     ----------  ----------  ----------  ----------  ----------
P973055           3     ----------  ----------  ----------  ----------  --------AC
P973058         651     cagcatactt  atatttgcag  tatgctgggt  ctttttaacc  ttaaaaccAC
                                                                                **
                                                                                **
                                                                                **
                                                                                **
                                                                                **
                                                                                **

alignment position      701.......  711.......  721.......  731.......  741.......
P973053           7     ----------  ----------  ----------  ----------  ----------
P973055           5     CACCTAGGGG  CCAGACTAGG  ATCCTAGTCA  CCAGTCACcc  tttagAGTGG
P973058         701     CACCTAGCGG  CCAGACTAGG  ATCCTATTCA  CCACTCACcc  ac---AGTGG
                        **********  **********  **********  ********         *****
                        **********  **********  **********  ********         *****
                        **********  **********  **********  ********
                        **********  **********  **********  ********
```

```
                    ********** ********** ********** *******
                    ********** ********** ********** *******

alignment position  751....... 761....... 771....... 781....... 791.......

P973053        7    ---------- ---------- ---------- ---------- ---CTCCCGA
P973055       55    TTAAGAAATG GAGagg--AG TTGTGTATTC GGAGCCAGAT TAGCTCCGGA
P973058      748    TTAAGAAATA GAGgagatAG ATATGTATTC GGAGCCAGAT TGGCTCCTGA
                    ********** ***     ** ********** ********** **********
                    ********** ***     ** ********** ********** **********
                                       ** ********** ********** **********
                                       ** ********** ********** **********

alignment position  801....... 811....... 821....... 831....... 841.......

P973053       14    CCTCTACTCt atACACTGCC TTTCCTCCGG CCCGTAAATA CAACGAATGA
P973055      103    CCTCAACTCA --ACACTGCC TTTCCTATGG CGCACAAACG CAGTGGACGA
P973058      798    CTTCCACTCA --ACACTGCC TTTCCTATGT CCCACAAACA CTGTGGACGA
                    **********   ******** ********** ********** **********
                    **** *****   ******** ********** ********** **********
                    **** *****   ******** ********** ********** **********
                    **** *****   ******** ********** ********** **********
                         *****   ******** ********** ********** **********
                                 ******** ********** ********** **********
                                 ******** ********** ********** **********
                                                                       ***

alignment position  851....... 861....... 871....... 881....... 891.......

P973053       64    A-CCTGCAGG TTGGTAGCTC CTGGGATCCG AGGTTGGAGt gccacaggac
P973055      151    ACCCTGCGGG TCAGCAGCTC CTGGGATTCG AGGCTGGAcC ACAGAATCAG
P973058      846    ACCCTGCAGG TCAGCAGCTT CTGGAATTCG AGGCTGGAGC ACAGAATCAA
                    ********** ********** ********** ********** **********
                    ********** ********** ********** ********** **********
                    ********** ********** ********** ********** **********
                    ********** ********** ********** *******  * **********
                    ********** ********** ********** *******  * **********
                    ********** ********** ********** ******
                    *  ******* ********** ********** ******
                    *  ******* ********** ********** ******
                       ******* ********** ********** ******
                       ******* ********** ********** ******

alignment position  901....... 911....... 921....... 931....... 941.......

P973053      113    cccaacctgc tctcacCCCG GGACAGGGAA ATacgaacAG GGGC-----A
P973055      201    GACTTCCTCG ------CCGG GGACAGAGAC ATCGAgtcgg gggacacagA
P973058      896    GACTTCGTCG ------CTGT GGACAGAGAC ATCGAgg-AG GGAC-----A
                    **********       **** ********** *****   ** ****     *
                    **********       **** ********** *****               *
                    **********       **** ********** *****               *
                    **********       **** ********** *****
                    **********       **** ********** *****

alignment position  951....... 961....... 971....... 981....... 991.......

P973053      158    CAGGTCCACC ACCAGGTGCC TCCGCGAGGG GCAgc-AGCG CAGGCGGCGG
P973055      245    CAGGTTCAGT TGGAGAtcc- -CTGAGCGGA ACAGCaAGTG CAGGCCGCCG
```

```
P973058   934 CAGGTTCAGT TGGAGACACC TCTGAGGGGA GCAGCgAGTG CAGGCCGCCG
              ********** ********** ********** ***** **** **********
              ********** ******                      **** **********
              ********** ******                      **** **********
                                                     **** **********
                                                     **** **********
                                                     **** **********

alignment
position      1001...... 1011...... 1021...... 1031...... 1041......

P973053   207 ggaggaccag cggcCACTCA CCTGGTGCTG GCGCT---GC GGCTACCTCC
P973055   293 CCTCT----- ----CACTTA CCTGGTGCTG GAGCTtctGC GGCCACCCCC
P973058   984 CCTCT----- ----CACCTA CCTGGTACTG GAGCT---GC GACCACCCCT
              *****          ****** ********** *****   ** **********
              *****          ****** ********** *****   ** **********
              *****          ****** ********** *****   ** **********
              *****          ****** ********** *****   ** **********
              *****          ****** ********** *****   ** **********
              *****          ****** ********** *****   ** **********
                             ****** ********** *****   ** **********
                             ****** ********** *****
                             ****** ********** *****

alignment
position      1051...... 1061...... 1071...... 1081...... 1091......

P973053   254 ACACAGCGGT CTTGGCCTCC CAGACGCGAA CCCGCACGCC CCAGGGGAGC
P973055   334 GCACAGTAGC CCGGGCACCC CGGACGCCGG TCCACTTGCG CGGAGGAAGC
P973058  1022 GCAGAGTAGC CCGAGCACCC CGGACACCGG CCCGCTAGCG CGGAGGAAGC
              ********** ********** ********** ********** **********
              ********** ********** ********** ********** **********
              ********** ********** ***** **** ********** **********
              ********** ********** ***** **** ********** **********
              ********** ********** ***** *
              ******             ** ***** *
              ******

alignment
position      1101...... 1111...... 1121...... 1131...... 1141......

P973053   304 CACCGGCGAa aagcccgCGG GCCCGTCCGC AGGCACCAAC CAAATAACGC
P973055   384 CCCCAGCCAC GCagtacCGG GCCAGTCCGC AGACACCGCC TACGTAGCGC
P973058  1072 CCCCAGCAAC GCtgcaccca atCCGTCCGC AGACACCTCC CACATAACGC
              ********** **     *** ********** ********** **********
              ********** **     *** ********** ********** **********
              ********** **           ******** ********** **********
              ********** **           ******** ********** **********
                                        ****** ********** **********
                                        ****** ********** **********
                                        ****** ********** **********
                                        ****** ********** **********

alignment
position      1151...... 1161...... 1171...... 1181...... 1191......

P973053   354 AGCGctGGCA CCATCCCCTA CGCACGCTCC CCTCCCTCAG Cgctcagaag
P973055   434 AGGGTCGGCC ACATGGCCTG CGCCACCTCC CCTCCCTCCA CCCCAGCACc
P973058  1122 AGAGTCGGCC ACATGGTCTA CACCGCCTCC TCTTCCTCAA CCTTAGCAGt
              ********** ********** ********** ********** *********
              ********** ********** ********** ********** *
              ********** ********** ********** ********
```

```
             **********  ****
             ****    ****  ****
             ****          ***
             **
             **

alignment
position     1201.......  1211.......  1221......  1231......  1241.......

P973053  404 aagccacttg  acc--CTCCA  CGGCCGAGGA  CCCGCGCTCT  TTAGCCCCGC
P973055  484 cGCAACCTGA  ACGGACTCTA  AGGGTGAAGC  GCGCCGCTCT  CCAGCCCCGC
P973058 1172 gGCAGCCTGA  ACGGACTCTA  AGGGTGCAGC  GCGCCGCTCT  TCAGCCCCGC
              *********  **********  **********  **********  **********
              *********  **********  **********  **********  **********
              *********  **********  **********  **********  **********
              *********  **********  **********  *********    *********
              *********  **********  **********  *********    *********
              *********  **********  **********  *********    *********
                              *****  **********  *********    *********
                                                     ******

alignment
position     1251.......  1261.......  1271......  1281......  1291.......

P973053  452 CCCtCACGCG  GGGGCCAATG  AAgtggCGCG  CCTTCGCCCC  ATGTGGGAGA
P973055  534 CCC-CAGACG  AGAGCCAATG  AAACA-CGCG  CGTTTGCCTG  ACGTGGGCGT
P973058 1222 CCC-CAAGCG  AGAGCCAATG  AAACA--GTG  CTTTGGCACA  ACGTGGGCGT
             *** ******  **********  *****  ****  **********  **********
             *** ******  **********  *****                    **********
             *** ******  **********  *****                    **********
             *** ******  **********  *****                    **********
             *** ******  **********  *****                    **********
             ***                                              **********
             ***

alignment
position     1301.......  1311.......  1321......  1331......  1341......

P973053  502 GG-CTGCGGT  GGCCAATAGG  CGCGCGCGTC  G---------  ----GAGGGC
P973055  582 GGTCTCCGCT  GGCCAATGAG  CGAGCGCcgt  ggaaggaaaa  gaccGAGGGC
P973058 1269 GGCCTCCGCT  GGCCAATGGG  CGAGCGCGGT  G---------  ----GAGGGA
             **********  **********  **********  *               ******
             **********  **********  **********  *               ******
             **********  **********  **********  *               ******
             **********  **********  *******                     ******
             **********  **********  *******                     ******
             **********  **********  *******
                *******  **********  *******
                *******  **********  *******
                *******  **********  *******

alignment
position     1351.......  1361.......  1371......  1381......  1391.......

P973053  538 GAAGGGCCGG  CCAGGgtgcc  gca---GACG  CGGGGACGCt  ggctcgctcc
P973055  632 GAAGGGCCGG  CCAGG-----  --------CG  CAGGGACGCA  GC--------
P973058 1306 AAAGGtagag  ggcgacgggc  cggctcGACG  CAGGGACGCC  GC--------
             **********  *****             ****  **********  **
             **********  *****               **  **********  **
             **********  *****               **  **********  **
             *****                           **  **********  **
             *****
```

```
alignment
position       1401...... 1411...... 1421...... 1431...... 1441......

P973053   585  ctccctccct ccctccgACG CTGTGAGTA- GAGAAGCTAG GCCCCGAGCC
P973055   661  ---------- -------ACG CCGTGAGTAa GAGGAGCTAG GGCGAAGGCG
P973058  1348  ---------- -------ACG CCGTGAGTA- GAGGAACGAG GGCGGAGGCA
                                 *** *********  ********** **********
                                 *** *********  ********** **********
                                 *** *********  ********** **********
                                 *** *********  ********** **********
                                 *** *********  ********** **********
                                                ********** **********

alignment
position       1451...... 1461...... 1471...... 1481...... 1491......

P973053   634  GGGcgggact aGGGTGGTGG TTgtgttCTG CCCTCGCCCG TCGCCAACGC
P973055   694  GGGACC---- -GGGTGGTGG TTCCA--CTC TCCGGGACCT CCGCCCGCAC
P973058  1380  GGGACC---- -GGGTGGTGG TTCCAct--- ---------- ----------
               ******      ********* *****  *** ********** **********
               ******      ********* *****
               ******      ********* *****
               ******      ********* *****
               ******      ********* *****
               ***         ********* **

alignment
position       1501...... 1511...... 1521...... 1531...... 1541......

P973053   684  TGGGCTGACT GAAAACCtgg ccctttgggt cgcgcgtctt cgaccttcac
P973055   737  AGGCTTGACT GGACGCCggc gctgttccgc cagagaaggc tgggcatgtc
P973058  1402  ---------- ---------- ---------- ---------- ----------
               ********** *******

alignment
position       1551...... 1561...... 1571...... 1581...... 1591......

P973053   734  cgccatgcgg acatggggcc gtgggagaaa ctctgggcgg gattacggtg
P973055   787  ggggtgaccc ctggctc--- ---------- ---------- ----------
P973058  1402  ---------- ---------- ---------- ---------- ----------

alignment
position       1601...... 1611...... 1621...... 1631...... 1641......

P973053   784  aaaattcttg tttgggcggg ccctgcggct tctttCTTCC GGCTCGCCCT
P973055   804  ---------- ---------- ---------- -----CTGCG GCCTCTCCTT
P973058  1402  ---------- ---------- ---------- ---------- ----------
                                                     ***** **********

alignment
position       1651...... 1661...... 1671...... 1681...... 1691......

P973053   834  CGGCCCCGCC CCCgccccgc ccccgctcca gctccagccg ggtcctcgct
P973055   819  CGGCCACGCC TCCtggtcgg ttggcgacgc tgtggcccgg gagctgggga
P973058  1402  ---------- ---------- ---------- ---------- ----------
               ********** ***

alignment
position       1701...... 1711...... 1721...... 1731...... 1741......

P973053   884  cggccccgca cgcggcccgc ggtagacgga gggcctgggc acccaccgtc
P973055   869  ctaagccacc tcctgc---- ---------- ---------- ----------
P973058  1402  ---------- ---------- ---------- ---------- ----------
```

```
alignment
position          1751......  1761......  1771......  1781......  1791......

P973053   934     tccatcccgg  ccccagccca  gtcgagcgtc  cactagaagg  gCGCTCCCTG
P973055   885     ----------  ----------  ----------  ----------  -CGCTCCCTG
P973058  1402     ----------  ----------  ----------  ----------  ----------
                                                                   *********

alignment
position          1801......  1811......  1821......  1831......  1841......

P973053   984     TCcggcccta  atcctcgctc  ctcacggagg  ctctttgtca  cagcgaagaC
P973055   894     TCactaccct  g---------  ----------  ----------  ---------C
P973058  1402     ----------  ----------  ----------  ----------  ----------
                  **                                                       *

alignment
position          1851......  1861......  1871......  1881......  1891......

P973053  1034     TGACAGCCCG  CAGTCTTCTG  GACttCTTTT  ATGGGGCTCC  TGGcggtgct
P973055   906     TGATTGCCCG  CAGGTTTCTG  GGC--CCTTC  ATGGGGCTCC  TGGttggttg
P973058  1402     ----------  ----------  ----------  ----------  ----------
                  **********  **********  ***  *****  **********  ***

alignment
position          1901......  1911......  1921......  1931......  1941......

P973053  1084     aTTCATTCAG  TCATTGATTC  GTCTTGTAAA  TATGGAGCGC  ctgctctgta
P973055   954     -TTCATTCAA  CCACTGACTC  AGCTTGTAAA  TATCGGGCGC  tgagctaggc
P973058  1402     ----------  ----------  ----------  ----------  ----------
                   *********  **********  **********  **********
                   *********  **********  **********  **********
                   *********  **********  **********  **********

alignment
position          1951......  1961......  1971......  1981......  1991......

P973053  1134     ctcgacccgg  cactgggtac  cggaagaacc  AGACAGCTCG  GTTTTTGCCA
P973055  1003     gctggatgaa  agt-------  ----------  AGACGGCTCG  GTCTCTGTCG
P973058  1402     ----------  ----------  ----------  ----------  ----------
                                                      **********  **********

alignment
position          2001......  2011......  2021......  2031......  2041......

P973053  1184     CCATTTAtaa  gtctgtgtc-  ----------  ----------  ----------
P973055  1036     TCATTTAgtg  tggtttttcc  ggtttctatt  attgacttgt  atatgtgcct
P973058  1402     ----------  ----------  ----------  ----------  ----------
                  *******

alignment
position          2051......  2061......  2071......  2081......  2091......

P973053  1203     ----------  ----------  ----------  ----------  ----------
P973055  1086     atgcgagctt  attgcaacag  gtatgtgcag  gagttgtgag  accagaaag
P973058  1402     ----------  ----------  ----------  ----------  ----------

alignment
position          2101......  2111......  2121......  2131......  2141......

P973053  1203     ----------  ----------  ----------  ----------  ----------
P973055  1136     gggagtacag  gatcgcctgg  aactggaaat  tctgagctgc  catgtgggtg
```

```
P973058 1402 ---------- ---------- ---------- ---------- ----------

alignment
position     2151...... 2161...... 2171...... 2181...... 2191......
P973053 1203 ---------- ---------- ---------- ---------- ----------
P973055 1186 ctgggaatct aggccctcag gtaagaccag ttaacagtct taaccataga
P973058 1402 ---------- ---------- ---------- ---------- ----------

alignment
position     2201...... 2211...... 2221...... 2231...... 2241......
P973053 1203 ---------- ---------- CTTTTCTTGA GTACTGAACc gagatacagt
P973055 1236 gccatctctc tagccccaaa CTTTTCTTTA TTAATTTACa ----------
P973058 1402 ---------- ---------- ---------- ---------- ----------
                                  ********** *********
alignment
position     2251...... 2261...... 2271...... 2281...... 2291......
P973053 1233 tttaaatgtg ctgtgcggtc tctgagcagc gactcacctg tgcaggTCAC
P973055 1276 ---------- ---------- ---------- ---------- ------TCTC
P973058 1402 ---------- ---------- ---------- ---------- ----------
                                                                ****
alignment
position     2301...... 2311...... 2321...... 2331...... 2341......
P973053 1283 TTCACCTGAT TCCCTTT--- ---------- ---------- ----------
P973055 1280 TGCCCCAGCT TCCCCTTcct tctctcaggc cttcctgact aagccagctc
P973058 1402 ---------- ---------- ---------- ---------- ----------
             ********** *******
alignment
position     2351...... 2361...... 2371...... 2381...... 2391......
P973053 1300 ---------- ---------- ----ATGTAA AAATgagggc gatgggctgt
P973055 1330 agtcaacagg ttctccaggg agggATGAAA AAATtgaaaa gaaaaagaca
P973058 1402 ---------- ---------- ---------- ---------- ----------
                                      ****** ****
alignment
position     2401...... 2411...... 2421...... 2431...... 2441......
P973053 1326 gtaatctcta aaggaccttc taactcttta aaattgtcac tgtgccggtg
P973055 1380 attagacagt actattaaca taaccctagc ccgt------ ----------
P973058 1402 ---------- ---------- ---------- ---------- ----------

alignment
position     2451...... 2461...... 2471..
P973053 1376 ttgctaacac tcattctgta tgcttg
P973055 1414 ---------- ---------- ------
P973058 1402 ---------- ---------- ------
```

Pairwise similarities:

For each pairwise alignment, the similarity (relative to the maximum similarity) and the number of identical nucleic acids (in % of shorter sequence) is given. Maximum values are underlined.

	P973055 (1413 bp)	P973058 (1401 bp)
P973053 (1401 bp)	0.283 35 %	0.198 23 %
P973055 (1413 bp)		1.000 41 %

Please note that the **similarity value 1.000** marks only the two **most similar** sequences, it does not necessarily mean that these sequences are identical.

Extract alignment region	
**Positions in alignment**	from: to:
**Output file**	dialign.seq

Extract aligned sequences

For comments, questions or bug reports, please contact support@genomatix.de

© Genomatix Software GmbH 1998-2005 - All rights reserved. License Agreement GEMS Launcher 4.1.1

Genomatix-Logo Logout Personal Messages GEMS-Logo
Logout Personal Messages

Genomatix_MainGEMSElDorado Gene2PromoterBiblioSphere
Frequently_asked_questionsYour ResultsYour SequencesYour_Protocol Help

GEMS Launcher Task: *DiAlign TF*: Multiple alignment plus TF sites working on promoters.seq (3 seq.)

[Alignment] [Pairwise_similarities]

DiAlign professional TF Release 3.1 December 2004 **Thu Jun 2 01:38:57 2005**

Solution parameters:

**Sequence file:**	promoters.seq (3 seq.)
**Type of sequences:**	nucleotide sequences
**Threshold T:**	0.00
**'*' signs below alignment denote:**	diagonal similarity (max. similarity is represented by 10 '*' signs)
**Alignment output:**	complete alignment is shown<br># nucleic acids per line: 50
**TF output:**	common TF matches located in aligned regions (common to 3 (100.0 %) sequences)
**Family matches:**	yes
**MatInspector library:**	Matrix Family Library Version 5.0 (February 2005)
**Selected groups** (core/matrix sim):	• ALL user_defined.lib (0.75/Optimized)<br>• ALL vertebrates.lib (0.75/Optimized)

Aligned Sequences:

No.	Sequence Name	Sequence Description	Length
1	P870080	sym=ATPAF2|loc=Loc91647|taxid=9606|spec=Homo sapiens|chr=17|ctg=NT_010718|str=(-)|start=17539329|end=17540329|len=1001| tss=501| comm=ATP synthase mitochondrial F1 complex assembly factor 2; (NM_145691/511/silver;)	1001 bp
2	P870082	sym=Atpaf2|loc=Loc246782|taxid=10090|spec=Mus musculus|chr=11|ctg=NT_096135|str=(-)|start=25500075|end=25501113| len=1039|tss=501,539|comm=ATP synthase mitochondrial F1 complex assembly factor 2; (AK078500/501/gold;NM_145427/539/silver;)	1039 bp
3	P870084	sym=na|loc=Loc303190|taxid=10116|spec=Rattus norvegicus|chr=10|ctg=NW_047334|str=(-)|start=33521135|end=33522135| len=1001|tss=501| comm=similar to ATP synthase mitochondrial F1 complex assembly factor 2; (XM_220522/525/silver;)	1001 bp

Alignment (DiAlign format):

Please note that only **upper-case** letters are considered to be **aligned**. For more information, please have a look at the user guide.

V$RXRF V$ECAT V$NFKB V$NRF1 U$NRF1 V$ETSF V$EBOX V$HIFF V$STAF V$EGRF V$SP1F V$PAX3
V$COMP V$HOXC V$PBXC V$INSM V$BRAC V$YY1F V$RREB V$AREB V$IRFF

```
alignment
position       1.........  11........  21........  31........  41........

P870080     1  ccgccgccgc  gcgggagccg  aggagggtgg  cggggaggga  gaggccgggg
P870082     1  c---------  ----------  ----------  ----------  ----------
P870084     1  t---------  ----------  ----------  ----------  ----------

alignment
position       51........  61........  71........  81........  91........

P870080    51  cgcgcacgcg  cggggtgggg  gcgggagGGG  CGACCACCCG  CCCGCTGCCT
P870082     2  ----------  ----------  -------GGG  GAACCACCCG  CCCGCTGAGG
P870084     2  ----------  ----------  ----------  ----------  ----------
                                              ***  **********  **********
                                              ***  **********  **********

alignment
position       101.......  111.......  121.......  131.......  141.......

P870080   101  GCGGAGCAAG  CGCTCcggcC  GCCACCGGGA  CCCAGGGACC  TGCGAGCAGG
P870082    25  CCAGACCAAG  CGCTCgtgtt  GCCACCTGGA  CCCGGAGACC  TGCAAGCAGG
P870084     2  ----------  ---------C  GCCACCTGGA  CCCCGAGACC  TGCAAGCAGG
               **********  *****    *  **********  **********  **********
               **********  *****    *  **********  **********  **********
                                    *  **********  **********  **********
                                       **********  **********  **********
                                       **********  **********  **********
                                       **********  **********  **********
                                       **********  **********  **********
                                       **********  **********  **********
                                       **********  **********  **********

alignment
position       151.......  161.......  171.......  181.......  191.......

P870080   151  GCCTCCCAGT  CCTGAGCTGG  GTCCCCCTCC  CGACTTGCCC  TCGCG-----
P870082    75  GCCTCCCAGa  CCTGAGCTGG  GTCTCCCTTC  CCACTTGCCC  TCGCGGGtaC
P870084    33  GCCTCCCAGT  CCTGAGCTGG  GCTCCCCTTC  CCACTTGCCC  TCGCGGGctC
               **********  **********  **********  **********  *******  *
               **********  **********  **********  **********  *******  *
               **********  **********  **********  **********  *******
               *********   **********  **********  **********  *****
               *********   **********  **********  **********  *****
               *********   **********  **********  **********  *****
               *********   **********  **********  **********  *****
               *********
               *********

alignment
position       201.......  211.......  221.......  231.......  241.......

P870080   196  -CACACATAC  ACACACACAG  ACACTCA---  -CAGACACAC  A---------
P870082   125  ACACACACAC  ACACACACAC  ACACACA---  -CACACACAC  ACGCACGCAC
P870084    83  ACACATACAC  ACACACACAC  GCACACAtgc  aCACACACAC  GCGCACACAC
               **********  **********  *******      *********  **********
               **********  **********  *******      *********  **********
                *********  **********  *******      *********  **********
                *********  **********  *******      *********  *
```

```
                 *********  **********  *******     *********  *

alignment
position         251.......  261.......  271.......  281.......  291.......

P870080  232     ----------  ----------  ----------  ----------  ----------
P870082  171     GCACACACAC  ACACACGCGC  atacacactC  ACTCACTCAT  ACACACGCGC
P870084  133     TCACTCATAC  ACACACGCGC  g--------C  ACACACACAT  ACACACACAC
                 **********  **********           *  **********  **********
                 **********  **********           *  **********  **********
                 **********  **********

alignment
position         301.......  311.......  321.......  331.......  341.......

P870080  232     ----------  ----------  ----------  --------AC  ACACAAACAC
P870082  221     TCACACACAC  ACGCGCgcgc  t---------  ----------  ---CACACAC
P870084  175     ACACACACAC  TCGCGCacac  actcacacac  atacacacAC  ACACACACAC
                 **********  ******                          **  **********
                 **********  ******                          **  **********
                                                                    *******
                                                                    *******
                                                                    *******
                                                                    *******
                                                                       ****
                                                                       ****

alignment
position         351.......  361.......  371.......  381.......  391.......

P870080  244     ACACAGACAC  ACACACACCC  CGCCCCTTCC  GATTGGCTCA  GGCGAGCCTG
P870082  249     ACACAAACAC  ACACACACTC  TGCAGCTTCC  AATTGGCGCG  GGCGCGCTGG
P870084  225     ACACAAACAC  ACACACACTC  TACTGCTTCC  AATTGGCGCG  GACCCGCCGG
                 **********  **********  **********  **********  **********
                 **********  **********  **********  **********  **********
                 **********  **********  **********  **********  **********
                 **********  **********  **********  ******
                 **********  **********  **********  ******
                 **********  **********  **********  ***
                 **********  ********
                 **********  ********

alignment
position         401.......  411.......  421.......  431.......  441.......

P870080  294     GGgGGCGGGA  GCAGGCTCAG  GCGGCGCTTC  CGCCCATTGG  CGGgaggaga
P870082  299     AGAGGCGGGA  GCAAGCTCAC  GCGGCGCTTC  CGCCCATTGG  CGGACAGAGA
P870084  275     AGAGGCGGGA  GCAAGCTCAC  GCGGCGCTTC  CGCCCATTGG  CAGACAGAGA
                 **********  **********  **********  **********  **********
                 **********  **********  **********  **********  **********
                 **********  **********  **********  **********  **********
                    *******  **********  **********  **********  **********
                    *******  **********  **********  **********  ***
                    *******  **********  **********  **********  ***
                    *******  **********  **********  **********  ***
                    *******  **********  **********  **********  *
                    *******  **********  **********  **********  *
                                         **********  **********  *

alignment
position         451.......  461.......  471.......  481.......  491.......

P870080  344     aaaCACGTGG  CGCGGTGCGC  GAGCGGCCCG  CTGGGACTTG  TAGtctcctc
P870082  349     AAGCACGTGG  CGCAGAGCGC  GAACGGCCCT  CTGGGACTTG  TAGTTTTCTT
```

```
P870084 325 AAGCACGTGG TGCGGTGCGC GAATGGCCCT CTGGGACTTG TAGTTTTCTT
            ********** ********** ********** ********** **********
            ********** ********** ********** ********** **********
            ********** ********** ********** ********** **********
            ********** ********** ********** ********** **********
               ******* ********** ********** ********** ***
               ******* ********** ********** ********** ***
               *******       **** ********** ********** ***
               *******       **** ********** ********** ***
               *******       **** ********** ********** ***

alignment
position    501....... 511....... 521....... 531....... 541.......

P870080 394 gCCGAGGCGC GGCGTGGAGG GGGCGGGCGG AGgtgCGTTT GTGATGCTGA
P870082 399 CCCGTGGCGC GGCATGGAGG GGGCGTGGCG AGCA-CGTTT ATGATGCTGA
P870084 375 CCCGTaGCGC GGCATGGAGG GGGCGGGCGG AGCA-CGCTT GTGATGCTGA
            ********** ********** ********** **** ***** **********
            ********** ********** ********** **** ***** **********
            ***** **** ********** ********** **** ***** **********
            ***** **** ********** ********** **   ** ** **********
             **** **** ********** ********** **   ** ** **********
             **** **** ********** ********** **

alignment
position    551....... 561....... 571....... 581....... 591.......

P870080 444 TTGACTGGGG gtgtg----- -CGCAG-GAG GCGCGCGGCG GCGACGGCGC
P870082 448 TTGACGGGGG CGGGAGTGGC GCGCAGcGGG GCGCACGGCT GCGACGGCGC
P870084 424 TTGACGGGGG CGGGGGCGGT ACGCAG-GGG GCGCGTGGCT GTGACGGCGT
            ********** ********** ****** *** ********** **********
            ********** ********** ****** *** ********** **********
            ********** ********** ****** *** ********** **********
            **********             ***** *** ********** **********
            **********             ***** *** ********** **********
                                           * ********** **********
                                           * ********** **********
                                           * ********** **********

alignment
position    601....... 611....... 621....... 631....... 641.......

P870080 487 GCCGTAGGTG TCATGcGGAA GATGGCGGCG TCCAAGGGAG GTTGAGGGCT
P870082 498 GCCGTAGGTG TCATGAGGAA GATGGCGGCG TGCTGAGGAG GCTGAGGGCT
P870084 473 GCCGTAGGTG TCATGAcGAA GATGGCGGCG TGCTGAGGAG GCTGAGGGCT
            ********** ********** ********** ********** **********
            ********** ********** ********** ********** **********
            ********** ****** *** ********** ********** **********
            ********** *****  *** ********** ********** **********
            ********** *****  *** ********** ********** **********
            ********** *****  *** ********** ********** **********
            ********** **     *** ********** ********** **********
            ********** **

alignment
position    651....... 661....... 671....... 681....... 691.......

P870080 537 CTGAggaggc gtTGAGAGGT TTCCGTACAG CCCGACCGTG CGGCTTTGGG
P870082 548 ATGAACCCTT GGTGAGAGGT TTCCGTACAG AACCAGCGAG Ggg--TGTGG
P870084 523 TTGAACCCTT GGTGAGAGGT TTCCGTACGG AACCAGCGAG GCGTTTGGGG
```

```
               ********** ********** ********** ********** **********
               ********** ********** ********** ********** **********
               ********** ********** ********** ********** *    *****
               ********** ********** ********** ********** *
                ***         ******** ********** ********** *
                ***         ******** **********
                ***
                ***

alignment
position       701....... 711....... 721....... 731....... 741.......

P870080 587    GA-TCCCATC GCTCTgtgTT TCCTGCTCCA GGTTTCgcgt ctcgcaTCTT
P870082 596    GGTTCTCTTC GCTCT---TT TCCTGTACTC TGTTTCCACG CATGTGTCCA
P870084 573    GACTCTCTTC GCTCT---AT TCCTGTACT- -GTTTTCACG TATGGGTCCA
               ********** *****   ** ********** ********** **********
               ********** *****   ** *********   ********* **********
               **                                                ****

alignment
position       751....... 761....... 771....... 781....... 791.......

P870080 636    CCCAGACCCT CGGGCGCGAT GTGGAGGAGC TGCCtCCGGC TGCGGGACGG
P870082 643    CCCAGGTCCT TGGGCAGGAT GTGGCGAATC TACCCCCGGC TGAGGGACCG
P870084 618    CCCAAACCCT TGGGCATGAT GTGGCGAATC TACCCCCGGC TGAGGGACCG
               ********** ********** ********** ********** **********
               ********** ********** ********** ********** **********
               ********** ******  *** ********** ********** **********
                                 *** ********** ********** **********
                                 *** ********** ********** **********
                                 *** ********** *     ***** **********
                                                      ***** **********

alignment
position       801....... 811....... 821....... 831....... 841.......

P870080 686    GGGACGCCGT CTCCTGAATC GGCCggcgGG TGGCCCCAGC GCTTCTATGA
P870082 693    GTGGCGCGGT CTCTTGGACC GGCGTCTCAG TGACCCCACC GTCTCAGTAT
P870084 668    GTGGCGCGGT CTCTTGGACC GGCCTCTCGG TGACCCCACC GTTTCATTGT
               ********** ********** ********** ********** **********
               ********** ********** ********** ********** **********
               ********** ********** ********** ********** ******   *
               ********** ********** ****      ** ********** ******   *
               ********** ********** ****       * ********** ******
               ********** ********** ***
               *******

alignment
position       851....... 861....... 871....... 881....... 891.......

P870080 736    GTCCGGGGCC Aaccatcccg tCTCCAGCCC GGGCTTACGC CCCGCCGACA
P870082 743    GGCCGGGGCC AGCTCCGCAG CCTCCGGCCC GGGCCTATGT CCCGCCAACA
P870084 718    GGCCGGGGCC AGCTCTGAGG CCTCCAGCCC GGGCCTACGT CCCGCCAACA
               ********** ********** ********** ********** **********
               ********** ********** ********** ********** **********
               ********** ********** ********** ********** **********
               ********** *           ********* ********** **********
                                      ********* *******  ** **********
                                      ********* *******  ** **********

alignment
position       901....... 911....... 921....... 931....... 941.......

P870080 786    GGTAAGACCa tgacatgttc -----TTTTG GGTTGGGGCC GAGCTGGGGT
```

```
P870082 793 GGTGAGGCCC GGACataaTG TGACTTTTTG GTCAGGAGCC AAGTCGGGCT
P870084 768 GGTGAGGCCC GGACttc-TG TTACTTTTTG TTCAGGAGCC AAGTCGGGCT
            ********** ****    ** ********** ********** **********
            ********** ****    ** ********** ********** **********
            ********** ****    ** ********** ********** **********
            *********          ** ********** ********** **********
            *********
            *********

alignment
position    951....... 961....... 971....... 981....... 991.......

P870080 831 AAGGTGGgct tcgGGCTTCG GCTGTGCAAA GCGAAGTAAC TGAGGCAGGG
P870082 843 CACCTGGaGG AA-GGTTTCT GACGTGCAAT GCGGGGTGAC --AGGCAGGG
P870084 817 CACCTGGcGG AA-GGTTTCC GACGTGCAGT GCGGGGTGAC TGAGGCAGGG
            ******* ** ** ******* ********** ********** **********
            ******* ** ** ******* ********** ********** **********
            ******* ** ** ******* ********** ********** **********
            *******                               *****   ********
                                                  *****   ********
                                                          ********

alignment
position    1001...... 1011...... 1021...... 1031...... 1041......

P870080 881 TTCAGGCTCA TGAGGTTATG CAGgccagtc agGTCTTTTT ACAGATGAGA
P870082 890 TTCAGGCCTA TGCGAATATT TAGAGCAACA GAGTTCTGTT TCAATTTGAA
P870084 866 TTCAGGCCCA TGAGAATATT CAGAGTAATA GAacTCTGTT TCAGTTTGAA
            ********** ********** ********** ********** **********
            ********** ********** ********** **  ****** **********
            ********** ********** ********** **  ****** **********
            ********** ********** ***            ****** **********
            ********** ********** ***
            *******

alignment
position    1051...... 1061...... 1071...... 1081...... 1091......

P870080 931 AAGTGAAGCC CAGACAAGGG CAGac--GAC CCGAGAGGGA ACAGCGAGTC
P870082 940 AAATGACATC CAAACAAGTG CGaggGTGAC TTTAGGGGAC ACAGCGTGCC
P870084 916 AAATGACATC CAAACCAGTG CGGgaGTGAC TTTAGGGGAC ACAGCGTTTC
            ********** ********** ***  ***** ********** **********
            ********** ********** **   ***** ********** **********
            ********** ********** **   ***** ********** **********
            ********** ********** **

alignment
position    1101...... 1111...... 1121...... 1131...... 1141......

P870080 979 ACGCACgata cagtaattga tat------- ---------- ----------
P870082 990 CAGGACACGA GATATttggc tctTGAGGCT TTTCTCAatt tcctttattt
P870084 966 CAGGGTAGGA GATATttggc ---TGAGGCT TCTCTCAgt- ----------
            ********** *****         ******* *******
            ********** *****
            ********** *****
```

Pairwise similarities:

For each pairwise alignment, the similarity (relative to the maximum similarity) and the number of identical nucleic acids (in % of shorter sequence) is given. Maximum values are underlined.

	P870082 (1039 bp)	P870084 (1001 bp)
P870080 (1001 bp)	0.452 59 %	0.447 55 %
P870082 (1039 bp)		1.000 85 %

Please note that the **similarity value 1.000** marks only the two **most similar** sequences, it does not necessarily mean that these sequences are identical.

Extract alignment region	
Positions in alignment	from: to:
Output file	dialign.seq

Extract aligned sequences

For comments, questions or bug reports, please contact support@genomatix.de

© Genomatix Software GmbH 1998-2005 - All rights reserved.

License Agreement

GEMS Launcher 4.1.1

Genomatix-Logo Logout Personal Messages GEMS-Logo
Logout Personal Messages

Genomatix_MainGEMSElDoradoGene2Promoter BiblioSphere
Frequently_asked_questions Your ResultsYour SequencesYour ProtocolHelp

GEMS Launcher Task: *DiAlign TF*: Multiple alignment plus TF sites working on promoters.seq (3 seq.)

[Alignment] [Pairwise similarities]

DiAlign professional TF Release 3.1 December 2004 **Fri Jun 3 23:28:12 2005**

Solution parameters:

**Sequence file:**	promoters.seq (3 seq.)
**Type of sequences:**	nucleotide sequences
**Threshold T:**	0.00
**'*' signs below alignment denote:**	diagonal similarity (max. similarity is represented by 10 '*' signs)
**Alignment output:**	complete alignment is shown<br># nucleic acids per line: 50
**TF output:**	common TF matches located in aligned regions (common to 3 (100.0 %) sequences)
**Family matches:**	yes
**MatInspector library:**	Matrix Family Library Version 5.0 (February 2005)
**Selected groups** (core/matrix sim):	• ALL user_defined.lib (0.75/Optimized)<br>• ALL vertebrates.lib (0.75/Optimized)

Aligned Sequences:

No.	Sequence Name	Sequence Description	Length
1	P882240	sym=ATPIF1|loc=Loc93974|taxid=9606|spec=Homo sapiens|chr=1|ctg=NT_037485|str=(+)|start=2673320|end=2674374|len=1055| tss=501,555| comm=ATPase inhibitory factor 1; (AK000934/555/gold;NM_016311/501/bronze;NM_178190/501/bronze;NM_178191/501/bronze;)	1055 bp
2	P882241	sym=Atpi|loc=Loc11983|taxid=10090|spec=Mus musculus|chr=4|ctg=NT_078437|str=(+)|start=34008|end=35058|len=1051| tss=501,551| comm=ATPase inhibitor; (AK011886/551/gold;AK008346/551/gold;NM_007512/501/silver;)	1051 bp
3	P882242	sym=na|loc=Loc362620|taxid=10116|spec=Rattus norvegicus|chr=5|ctg=NW_047724|str=(+)|start=2519787|end=2520787| len=1001|tss=501| comm=similar to ATPase inhibitor, mitochondrial precursor; (XM_216338/513/silver;)	1001 bp

Alignment (DiAlign format):

Please note that only **upper-case** letters are considered to be **aligned**. For more information, please have a look at the user guide.

V$MAZF V$EGRF V$ZFIA V$NRSF V$DMTF V$ETSF U$NRF2 V$HNF4 V$SP1F

```
alignment
position    1......... 11........ 21........ 31........ 41........
P882240   1 aaagtgttgg gattacaggc gtgagccacc gcgcccagcc agaacactga
P882241   1 ---------- ---------- ---------- ---------- AGGTCAAAGA
P882242   1 aagcaggata aaaaaattgc tgacgacagg atgactagga AGGTCAAAGA
                                                        **********
                                                        **********
                                                        **********
                                                        **********
                                                        **********

alignment
position    51........ 61........ 71........ 81........ 91........
P882240  51 c--------- ---------- ---------- ---------- ----------
P882241  11 AGGGTTTCTC TGAGAAAGTG ACATTTAAGG tgagagaagg cACTGCAGTG
P882242  51 AGGGTTTCTC TGAGAAAGTG ACATTTAAGG tgagaggata gACTGCAGTG
            ********** ********** **********             *********
            ********** ********** **********             *********
            ********** ********** **********             *********
            ********** ********** **********             *********
            ********** ********** **********             *********

alignment
position    101....... 111....... 121....... 131....... 141.......
P882240  52 ---------- ---------- --ACTTCTCA TAACAAATGA ACGCGATTTa
P882241  61 GCAATATAAC CAAAGAACAA GAACACCTCT TAAGAGATAA ACACGATTTC
P882242 101 GCAATATAAC CAAAGAACAA GAACACCTC- -AAGAGATGA CTACGATTTC
            ********** ********** ********** ********** **********
            ********** ********** *********   ********* **********
            ********** ********** *********
            ********** ********** *********
            ********** ********** *********

alignment
position    151....... 161....... 171....... 181....... 191.......
P882240  80 tgtatatcta agccagggca caTTGGACGT CGGTATCCTG GACATCAGCA
P882241 111 ATATGTATAa acagagG--- --TTTGAGGT CGATATCCAG GCTGTCGGCA
P882242 149 ATATGTATAg aggtatG--- --TTGGAGGT CGATAACCAG ACTGTCGGCA
            *********        *     ******** ********** **********
            *********        *     ******** ********** **********
                             *     ******** ********** **********
                                   ******** ********** **********
                                        ***** ********** **********

alignment
position    201....... 211....... 221....... 231....... 241.......
P882240 130 TCCTGGACAC AGCT--CCCA AACCCCGAAT TCCCCCATTT TATTGATGct
P882241 156 TCCTAGGCAT GGCTACCACA AACTTTGTGT TTCCCCAATC TATTCATGTC
P882242 194 TCCTAAGCAc aaCTACCACA AACCTTTATT TCCCCAATaa tgt-----TC
            ********** ********** ********** ********** **********
            *********    **
            *********
            *********
            ****
```

```
alignment
position       251....... 261....... 271....... 281....... 291.......

P882240  178   agcaggccag ggaagggaaa acttcctttg ggcTTTGAAT AATAACGACT
P882241  206   CATAGGGCTT GCCATCgtGT ACAT------ ---TTTTAAT AAAATCGACC
P882242  239   CATAGGGATT GCCACCacGT ACAT------ ---TTTTAAT AAAATCGACC
               ********** ******  ** ****          ******* **********
                                 ** ****          ******* **********
                                 ** ****          ******* **********
                                                  ******* **********

alignment
position       301....... 311....... 321....... 331....... 341.......

P882240  228   TTAGAATTGA AATCGTGGGC TGGAGGTGGA CGCCACGCCT CATTCTGATG
P882241  247   TTTGGACGGA AATTGTGCaG TGGGAGTGAA CGAAATGCCT CATTCTAAAG
P882242  280   TTAAGACAAA AATTGTGCGG TGGAAATGAA CAAAATGCCT CATTTGAACG
               ********** ********** ********** ********** **********
               ********** ******** * ********** ********** **********
               ********** *******  * ********** ********** **********
               ********** *******         ***** ********** **********
                        * *******         ***** ********** ****

alignment
position       351....... 361....... 371....... 381....... 391.......

P882240  278   GGTCCAAGAG CCGAaatttc ggcttctggc acTCTCCTCA CTCAGCTCTC
P882241  297   AGACCAAAAG CCAAgattca ---------- --TCTTTTGA CTTCGCTCTG
P882242  330   AGACCAAAAa tctcacgggt tgggatga-- ---------- ----------
               ********** ****                    ******** **********
               ********** ****
               *********
               *********

alignment
position       401....... 411....... 421....... 431....... 441.......

P882240  328   TAAACTCCTC CATCATTcct ctttccagtc ctttggccct aagcccttgc
P882241  335   TAGACTCCTG GGTCATGatg cGTTAGAGCC TTGTTTATTA ACTGTTAACT
P882242  358   ---------- ---------- -GTCAGAGCC CTGTTTATTA GCTGTTTCCT
               ********** *******     ********* ********** **********
                                      ********* ********** **********

alignment
position       451....... 461....... 471....... 481....... 491.......

P882240  378   tagcggttgg ctAAGAAGAA ACCCGTCTCT TGCTCGGGAT TCGCTGtgag
P882241  385   A--------- --AGGGATAA ATCCGCCTCC CTCTCAACAA ACGCTGCAGA
P882242  387   A--------- --ATGCATAA Accaga---- CTCTCAACAA TCGCTACGGA
               *            ******** ********** ********** **********
               *            ******** *

alignment
position       501....... 511....... 521....... 531....... 541.......

P882240  428   ctccgggaag cccGCCT--- CCGCTCTCCC GTGATTCGCT ACTCCTGTGT
P882241  424   Tat-CATAA- ---GCCCcgc CCCCTCCCTC CTGATTCGCG GTTTCCGTGC
P882242  422   TtagCATAA- ---GCCC--- CGCCTCCCTT CTGATTCGCT GTTTCCCTGC
               *   *****     ****    ********** ********** **********
                                     ******** ********** **********
                                     ******** ********** **********
                                     ******** ********** **********
```

```
alignment position
            551....... 561....... 571....... 581....... 591.......
P882240 475 TCATCCGCCC -CTCCGTCGA CCAGATTGGG TGCT-TGGCC GTCCCTGCCA
P882241 469 TCGTCCCACC TCCCCGGCGT CTAGATTGGC AGTT-CAGCC GCCCTCGCAG
P882242 465 TCGTCCCgCC TCCCCGCCGT CTAGATTGGC GGTTcCAGCC GCCCTCGCAG
            ********** ********** ********** **** ***** **********
            ******* ** ********** ********** **** ***** **********
            ******* **              ******** **** ***** **********
            *******                               ***** **********
                                                  ***** **********
                                                  ***** **********

alignment position
            601....... 611....... 621....... 631....... 641.......
P882240 523 TTAGCGCGTA ACGAGAGACt gcttgctgCG GCAGAGACGC CAGAGGTGCA
P882241 518 TTAGCGCGCT AGGAGAGAAG CAgCCA--CG CCCGCAACGC GAGCTGAGCA
P882242 515 TTAGCGCGCT AGGAGAGAAG CAaCCA--CG CCCGCAACGC GAGCTGAGCA
            ********** ********** ** ***  ** ********** **********
            ********** ********** ** ***  ** ********** **********
            ********** ********** ** ***  ** ********** **********
            ********** ********** ** ***  ** ********** **********
            ********** ********** ** ***  ** ********** **********
            ********

alignment position
            651....... 661....... 671....... 681....... 691.......
P882240 573 GCTCCAGCAG CAATGGCAGT GACGGCGTTG GCGGCGCGGA CGTGGCTTGG
P882241 566 ACGCCGAAGA CAATGGCAGG CTCGGCGTTG GCAGTTCGGG CTCGGTTCGG
P882242 563 ACGCCGAAGA TAATGGCAGG CTCGGCGTTG GCGGTTCGGG CTCGGCTCGG
            ********** ********** ********** ********** **********
            ********** ********** ********** ********** **********
            ********** ********** ********** ********** **********
            ********** ********** ********** ********** **********
            ********** ********** ********** ********** **********
                        ********* ********** ********** **********

alignment position
            701....... 711....... 721....... 731....... 741.......
P882240 623 CGTGTGGGGC GTGAGGACCA TGCAAGCCCG AGGCTTCGGC TCGGATCAGG
P882241 616 TGTCTGGGGT ATGAAGGTCC TGCAAACCCG AGGCTTCGTC TCGGACTCGG
P882242 613 TGTCTGGGGT ATGAGGGTCC TGCAAACCCG AGGCTTCGGC TCGGACTCGG
            ********** ********** ********** ********** **********
            ********** ********** ********** ********** **********
            ********** ********** ********** ********** **********
            ********** ********** ********** ********** **********
            ********** ********** ********** ********** **********
                ****** ********** ********** ********** *****
                ****** ********** ********** ********** ****
                                  ********** ********** ****

alignment position
            751....... 761....... 771....... 781....... 791.......
P882240 673 TACGCTGCGG CAgtgtccgc tgctCCAGCC CCGCGGGCGG ATC--Ccatc
P882241 666 TAGGTGGCGG CACGGCCCAG CGC-CCATCG CGGGGcgcag gttctCTCTC
P882242 663 TAGGTGGCGG CACGACCTAC CGC-CCATCG CGGGGTGCGG GTC--CTCTT
```

```
            ********** ********** *** ****** ********** ***  *****
            ********** ********** *** ****** *****           *****
            ********** ********** *** ****** *****
            ********** ********** *** ****** *****
            **********

alignment
position    801....... 811....... 821....... 831....... 841.......

P882240 721 tttgctt--- CCGTACCTTT ACCAGTGTCC CTGTCTCTCT GCAGTCCGAG
P882241 715 CCTTGTTGTG CCTTTTCTTT TCACGTCCAC GACTCTCTTT GCAGTCGGAT
P882242 710 CCTTGTTGTG CCTTTTTTTT aat-GTCCAC GACTCTCTCT GCAGTCGGAG
            ********** ********** ********** ********** **********
            ********** **********     ****** ********** **********
                                      ****** ********** **********
                                      ****** ********** **********
                                                ******* *********

alignment
position    851....... 861....... 871....... 881....... 891.......

P882240 768 Aatgtcgacc gGGGCGCGGG CTCCATCCGG GAAGCCGGTG GGGCCTTCGG
P882241 765 AGCATGGATa CGGGCGCTGG CTCCATCCGA GAAGCTGGTG GAGCCTTCGG
P882242 759 AGCATGGATt CGGGCGCTGG CTCCATCCGA GAAGCTGGTG GGGCCTTCGG
            *********  ********** ********** ********** **********
            *********  ********** ********** ********** **********
            *********  ********** ********** ********** **********
            *********  ********** ********** ********** **********
                       ********** ********** ********** **********
                        ********* ********** ********** **********
                        ********* ********** ********** **********
                        ********* ********** ********** **********
                        ********* ********** ********** *  ********
                        ********* ********** ********** *  ********

alignment
position    901....... 911....... 921....... 931....... 941.......

P882240 818 AAAGAGAGAG CAGGCTGAAG AGGAACGATA TTTCCGGTGA GGCTCACCGG
P882241 815 AAAACGAGAA AAGGCTGAAG AGGATCGGTA CTTCCGGTGA GGCCCACCTG
P882242 809 gAAACGAGAG AAGGCTGAAG AGGATCGGTA CTTCCGGTGA GGCCCACCTG
            ********** ********** ********** ********** **********
            ********** ********** ********** ********** **********
            ********** ********** ********** ********** **********
             ********* ********** ********** ********** **********
             ********* ********** ********** ********** **********
             ********* ********** ********** ********** **********
             ********* ********** ********** ********** **********
                 ***** ********** **********  ********* *** ******
                 ***** ********** **********  ********* *** ******
                                              ********* ***

alignment
position    951....... 961....... 971....... 981....... 991.......

P882240 868 GTCCCAAGTC CAGCCCTGGA TCTCCCaatg gccttccaat ccttaaactg
P882241 865 GTCCCAAGTC CAGCCCCCAG TCGTCCTCCC TGTCCTTTCT ACGCTCTCTG
P882242 859 GTCCCAAGTC CAGCCCCCAA TCGCCCTCCC TGTCCTTTCT ACGCTCTCTG
            ********** ********** ********** ********** **********
            ********** ********** ********** ********** **********
            ********** ********** ********** ********** **********
            ********** ********** ********** ********** **********
```

```
                    ********** ********** ******     ********** **********
                    ********** ********** ******
                    ********** ******
                    ********** ******
                    ********** ******

alignment
position            1001...... 1011...... 1021...... 1031...... 1041......

P882240  918        ccaaTCGCCC CACCCGTTCC TACCTGGTGC CTTGGGCGCC CCATccccca
P882241  915        ----TCGCTC CACCCATCTT TACCTGGTCC CTTGGGGCGC CCATCTTCTG
P882242  909        ----TCGCTC CACCCATCTT TACCTGGTCC CCTGGGGCGC CCATCTTCTG
                        ****** ********** ********** ********** **********
                        ****** ********** ********** ********** **********
                        ****** ********** ********** ********** **********
                        ****** ********** ********** ********** **********
                        ****** ********** ********** ********** ****
                        ****** ********** ********** ********** ****
                        ****** ********** ********** ********** ****
                        ****** ********** *****

alignment
position            1051...... 1061...... 1071...... 1081...... 1091......

P882240  968        aCAGAACTCC CGGGCcccaa tccagtatac cctaaccctt gatgtcccga
P882241  961        GCAGAATTCC AAGGCtGATG AAGCCATCGG ---------- ----------
P882242  955        GCTGAATTCC AAGGCcGGTG AAGCCTTCCT ---------- ----------
                    ********** *****  **** **********
                    ********** *****
                    ********** *****
                    ********** *****

alignment
position            1101...... 1111...... 1121...... 1131...... 1141......

P882240  1018       ccGTTGCCAC GTATAGGGCA CTCCCAGTta cctgcaca-- ----------
P882241  991        --GTCGCCTC GTAGTCGAGA CTGCTAGTat cgcactgctg gcctcccagc
P882242  985        --GTTTCCAC GTAATCGAG- ---------- ---------- ----------
                      ******** ********** ********

alignment
position            1151...... 1161

P882240  1056       ---------- ---
P882241  1039       cagccttaag cct
P882242  1002       ---------- ---
```

Pairwise similarities:

For each pairwise alignment, the similarity (relative to the maximum similarity) and the number of identical nucleic acids (in % of shorter sequence) is given. Maximum values are underlined.

	P882241 (1051 bp)	P882242 (1001 bp)
P882240 (1055 bp)	0.225<br>44 %	0.219<br>43 %
P882241 (1051 bp)		<u>1.000</u><br><u>81 %</u>

Please note that the **similarity value 1.000** marks only the two **most similar** sequences, it does not necessarily mean that these sequences are identical.

**Extract alignment region**	
**Positions in alignment**	from: to:
**Output file**	dialign.seq

Extract aligned sequences

For comments, questions or bug reports, please contact support@genomatix.de

© Genomatix Software GmbH 1998-2005 - All rights reserved. License Agreement GEMS Launcher 4.1.1

Genomatix-Logo Logout Personal Messages GEMS-Logo
Logout Personal Messages

Genomatix_Main GEMS ElDorado Gene2Promoter BiblioSphere
Frequently_asked_questions Your Results Your Sequences Your Protocol Help

GEMS Launcher Task: *DiAlign TF*: Multiple alignment plus TF sites working on promoters.seq (3 seq.)

[Alignment] [Pairwise similarities]

DiAlign professional TF Release 3.1 December 2004 **Thu Jun 2 01:09:42 2005**

Solution parameters:

**Sequence file:**	promoters.seq (3 seq.)
**Type of sequences:**	nucleotide sequences
**Threshold T:**	0.00
**'*' signs below alignment denote:**	diagonal similarity (max. similarity is represented by 10 '*' signs)
**Alignment output:**	complete alignment is shown<br># nucleic acids per line: 50
**TF output:**	common TF matches located in aligned regions (common to 3 (100.0 %) sequences)
**Family matches:**	yes
**MatInspector library:**	Matrix Family Library Version 5.0 (February 2005)
**Selected groups** (core/matrix sim):	• ALL user_defined.lib (0.75/Optimized)<br>• ALL vertebrates.lib (0.75/Optimized)

Aligned Sequences:

No.	Sequence Name	Sequence Description	Length
1	P889644	sym=ATPAF1|loc=Loc64756|taxid=9606|spec=Homo sapiens|chr=1|ctg=NT_032977|str=(-)|start=952928|end=953928|len=1001| tss=501| comm=ATP synthase mitochondrial F1 complex assembly factor 1; (AK026004/527/gold;NM_022745/527/silver;)	1001 bp
2	P889645	sym=Atpaf1|loc=Loc230649|taxid=10090|spec=Mus musculus|chr=4|ctg=NT_039264|str=(+)|start=15907741|end=15908968| len=1228|tss=501,728| comm=ATP synthase mitochondrial F1 complex assembly factor 1; (AK032020/733/gold;NM_181040/506/silver;)	1228 bp
3	P889646	sym=na|loc=Loc313510|taxid=10116|spec=Rattus norvegicus|chr=5|ctg=NW_047718|str=(+)|start=7486974|end=7487974| len=1001|tss=501| comm=similar to ATP synthase mitochondrial F1 complex assembly factor 1; (XM_233402/501/silver;)	1001 bp

Alignment (DiAlign format):

Please note that only **upper-case** letters are considered to be **aligned**. For more information, please have a look at the user guide.

V$IRFF V$ZF5F V$STAF V$NOLF V$MOKF V$ETSF V$NRF1 U$NRF1 V$MYOD V$NEUR V$MTF1 V$ZBPF
V$CDEF V$IKRS V$EVI1 V$EGRF V$AREB V$EBOX

```
alignment
position       1.........   11........   21........   31........   41........

P889644    1 attactctcg ----------  ----------  ----------  ----------
P889645    1 ---GCATTGC GTTTTTAAAA AGCTTAGCTT TTAGGTGGAA CTTACTACTT
P889646    1 gagGCATTGC GTTTTTAAAA GGCTCAGCTC TTAGGTGGAA CTTACTACTT
                ******* ********** ********** ********** **********
                ******* ********** ********** ********** **********
                ******* ********** ********** ********** **********

alignment
position       51........   61........   71........   81........   91........

P889644   11 ---------- -----GCCTC AGTTTCCTTA TTTGTTAGAT GAGGATAATA
P889645   48 TAATGGTTCA CAGCCTGTTT AGCTTCTCCA TCTGTAAAAT GAGGTTAATA
P889646   51 TAATGGTTCG CAGCCGGCTT AGCTTCTCTG TCTGTAAAAT GAGGTTAATA
             ********** ********** ********** ********** **********
             ********** ********** ********** ********** **********
             ********** ********** ********   ********** **********
                               ** ********   ********** **********
                                              ********** **********
                                              ********** **********

alignment
position       101.......   111.......   121.......   131.......   141.......

P889644   46 -CTGCCACAC AGAGTATGAA GCCgctcagt g-TCCTGCAC ATAGCAACCt
P889645   98 TCTACTTCAT AGGGTATGAG GCCAATGGAA CCTTCTGCAA ATAGACACTC
P889646  101 TCTACTTCAT AGGGTATGAG GCCAATGGAA CCTTCTTCAA ATAGAAACCC
             ********** ********** ********** ********** **********
             ********** ********** ********** ********** **********
             ********** ********** ********** ********** **********
             ********** **********
             ********** **********

alignment
position       151.......   161.......   171.......   181.......   191.......

P889644   94 cagTAAAGAG AAGCTTgctg ctgtgattat aGTTGATGAA AAGACCTTCC
P889645  148 A--TAAAGAG AAGCTTCAGC TAT------- -GATGTTCAG ACATCCTTTC
P889646  151 A--TAAAGAG AAACTTCAGC TAT------- -GATGTTCAA ACATCCTTTC
             *  ******* ********** ***         ********* **********
             *  ******* **  ****** ***         ********* **********
             *  ******* **  ****** ***         ********* **********
                ******* **  ****** ***         ********* **********
                             ***

alignment
position       201.......   211.......   221.......   231.......   241.......

P889644  144 ATGCTGAAGG Acggactact acatcgagtg cctggcacTA CAGCGAGTGC
P889645  188 CAGCTAAAGA AACTGT---- ---------- --------TA CAGCTAGTGC
P889646  191 CAGCTAAAGA AACTGT---- ---------- --------TA CAGCTAGTGC
             ********** ******                     ** **********
             ********** ******                     ** **********
             ********** ******                     ** **********
             ********** ******                     ** **********
                          *****                     ** **********
```

```
alignment
position         251.......  261.......  271.......  281.......  291.......

P889644    194   CA--------  -------GGG  GCAAGCACCG  CGAGGACATG  GAGATCCgct
P889645    216   CAAGTAAATT  TAGAAAAGGG  GCAAGCCCTG  TGAGAACCCG  AAGATGCAAC
P889646    219   CAAGTAAATT  TAGAAAAGGG  GCAGGCCTGG  TGAGAACCCG  AAGATGCAAC
                 **********  **********  **********  **********  **********
                 **********  **********  ***      *  **********  **********
                 **********  **********  ***      *  **********  **********
                 **********  **********  ***      *  **********  **********
                 **********  **********  ***      *  **********  *****

alignment
position         301.......  311.......  321.......  331.......  341.......

P889644    229   ggctttagag  aacggact--  TGGCGCGCAG  AGGCTGCTGG  TCccagaggg
P889645    266   CCAGCTTTCC  AGAAG---CC  TGGCGCGCAG  AGGCTGCTGG  TCGGGGGTAC
P889646    269   CCAGCTTTCC  AGAAGaagCC  TGGCGCGCAG  AGGCTGCTGG  TCGGGGGTAC
                 **********  *****   **  **********  **********  **********
                 **********  *****   **  **********  **********  **********
                 **********  *****   **  **********  **********  **********
                 **********  *****   **  **********  **********  **********
                                     **  **********  **********  **********
                                         **********  **********  **
                                         **********  **********  **
                                         **********  **********  **

alignment
position         351.......  361.......  371.......  381.......  391.......

P889644    277   aggaagggcg  tTGAATTTGG  GCAGCCTCCC  TGGAGGAGGC  GGGCCgtggc
P889645    313   GGGGTGGGG-  -TGAATCCTG  GCAGACTCCC  GGGAGGAGAC  GCGCCACGAC
P889646    319   GGGGTGGGG-  -TGAATCCGG  GCAGACTCCC  GGGAGGAGAC  GCGCCACTAC
                 *********    *********  **********  **********  **********
                 *********    *********  **********  **********  **********
                 *********    *********  **********  **********  **********
                 *********    *********  **********  **********  **********
                 ********     *********  **********  **********  *****
                              *********  **********  **********  *****

alignment
position         401.......  411.......  421.......  431.......  441.......

P889644    327   atacacaggg  cggcgagagc  gcgcagtgcc  ggggcCAGAG  CGGcgcgccc
P889645    361   acccct----  ----------  ----------  ------AGAG  CGGGTGGGCA
P889646    367   accca-----  ----------  ----------  -----CAGAG  CGGGTGGGCT
                                                          *****  **********
                                                           ****  **********
                                                           ****  **********

alignment
position         451.......  461.......  471.......  481.......  491.......

P889644    377   cctcctcccc  gccccgcctc  gccgcta---  ----------  ----TCCCGC
P889645    381   GAACCGGGAG  CTGCCAGCCC  GAAGcgtcgt  TGTCCCTTCT  CCACTCCAGC
P889646    387   GAACCGAGAG  CCGCCAGCCC  TAAGagttgc  TGTCCCTGCT  CCACTCCAGC
                 **********  **********  ****        **********  **********
                 **********  **********  ****        **********  **********
                 **********  **********  ****        **********  **********
                                                     **********  **********
                                                                     ******
                                                                     ******
```

```
alignment
position            501.......  511.......  521.......  531.......  541.......

P889644  410        GCCCGGAACC  TTGGGCGCGC  GGAACCTCGG  TGCCGGAGCG  CGGGCGCGGC
P889645  431        ACCCGGAACC  TTGAGCGCGC  GGAACCTCGG  TGGCGGAACG  CGGGCCTCGC
P889646  437        ACCCGGAACC  TTGAGCACGC  GGAACTTCGG  TGGCGGAACT  CGGGCCTCGC
                    **********  **********  **********  **********  **********
                    **********  **********  **********  **********  **********
                    **********  **********  **********  **********  **********
                    **********  **********  **********  **********  **********
                    **********  **********  **********  **********  *
                    **********  ****** ***  **********  **********  *
                     *********  ****** ***  **********  **
                     *********  ****** ***  **********  **

alignment
position            551.......  561.......  571.......  581.......  591.......

P889644  460        GCACTCGGAG  CGGGATGGAG  CGGGCGCGCG  GGCCGCGGGC  CGAGGCCGAC
P889645  481        GGACcttgcg  ggaGATGGAG  CAGGCGCGCG  GGCCGGGGGC  CGAGGCTGAC
P889646  487        GGACTTAGAG  GGCGATGGAG  CAGGCGCGCG  GGCCGGGGGC  CGAGGCTGAC
                    **********  **********  **********  **********  **********
                    ****           *******  **********  **********  **********
                    ****           *******  **********  **********  **********
                    ****           *******  **********  **********  **********
                                   *******  **********  **********  **********
                                   *******  **********  **********  **********
                                   *******  **********  **********  **********
                                   *******  **********  **********  **********
                                     *****  **********  **********  **********
                                     *****  **********  **********  **********

alignment
position            601.......  611.......  621.......  631.......  641.......

P889644  510        GCGCCt---G  AGGAGGAGGA  GGAGGCGGGG  GCGGCCATGG  CTGCTGTggt
P889645  531        GCGcccGAGG  AGGAGGAGGG  GGAGTCGCAG  GCGGCCATGG  CGGCgGTA--
P889646  537        GCGCCaGAGG  AGGAGGAGGG  GGAGGCGCAG  GAGGCCATGG  CGGCTGTA--
                    ***** ****  **********  **********  **********  ********
                    ***** ****  **********  **********  **********  ********
                    ***** ****  **********  **********  **********  **** ***
                    ***      *  **********  **********  **********  **** ***
                    ***      *  **********  **********  **********  **** ***
                    ***      *  **********  **********  **********  **** **
                    ***      *  **********  **********  **********  **** **
                    ***
                    ***
                    ***

alignment
position            651.......  661.......  671.......  681.......  691.......

P889644  557        gGTGGCGGCT  GCGGGTGGCG  CGGGACCGGC  gGTCCTGCAG  GTGGCCGGTC
P889645  579        -GTGTCCGCT  GCGGGTGGAG  CGTGCCCCGC  AGTCCTGCAG  GTGGCGGGTC
P889646  585        -GTGTCCGCT  GCGGGTGGAG  CGTGCCCCGC  AGTCCTGCAG  GTGGCCGGCC
                     *********  **********  **********  **********  **********
                     *********  **********  **********  **********  **********
                     *********  **********  **********  **********  **********
                     *********  **********  **********  **********  **********
                     *********  **********  **********  **********  **********
                     *********  **********  **********   *********  **********
                                                         *********  **********
```

```
                                              *******
                                              *******

alignment
position    701....... 711....... 721....... 731....... 741.......

P889644 607 TCTACCGGGG CCTGTGCGCG GTGCGCAGCC GCGCCCTGGG CCTGGGGCTC
P889645 628 TCTACCGAGG CCTGTGTGCa GTGCGCAGCC GCGCCCTGGG GCTGGGGTTC
P889646 634 TCTACCGAGG CTTGTGTGCG GTGCGCAGCC GCGCCCTGGG GCTGGGGTTC
            ********** ********** ********** ********** **********
            ********** ********** ********** ********** **********
            ********** ********** ********** ********** **********
            ********** ********** ********** ********** **********
            ********** *********  ********** ********** **********
            ********** ******  ** ********** ********** **********
            ********** ******  ** ********** ********** **********
                               ** ********** ********** **********
                                  ********** ********** **********
                                  ********** ********** **********

alignment
position    751....... 761....... 771....... 781....... 791.......

P889644 657 GTGTCACCcG CGCAGCTGCG CGTCTTCCCA GTGCGCCCCG GCTCGGGCCg
P889645 678 GTGTCACCgG CACAGTTGCG CGTGTTCCCG GTGCGCCGGG GCTCGGGCCt
P889646 684 GTGTCACCtG CACAGTTGCG CGTGTTCCCG GTGCGCCGGG GCTCGGGCCt
            ******** * ********** ********** ********** *********
            ******** * ********** ********** ********** *********
            ******** * ********** ********** ********** *********
            ******** * ********** ********** ********** *********
            ******** * ********** ********** ********** *********
            ******** * ********** ********** ********** *********
            ******** * ********** ********** ********** *********
            *******  * ********** ********** ********** *********
            *******
            *******

alignment
position    801....... 811....... 821....... 831....... 841.......

P889644 707 GCCCGAGGGG GGCGCCGACA GCAGCGGGGT CGGggccgag gcCGAGCTCC
P889645 728 GCCCCCCGAG GGAGCAGACG GCAGTGGGGT CAG------- --CGAGCTGC
P889646 734 gtcctCCGGG GGAACAGACG GCGGTGGGGT CAG------- --CGAGCTGC
            ********** ********** ********** ***          ********
                 ***** ********** ********** ***          ********
                 ***** ********** ********** ***          ********
                 ***** ********** ********** **           ********
                                                          ********

alignment
position    851....... 861....... 871....... 881....... 891.......

P889644 757 AGGCCAACCC TTTCTACGAC CGCTACCGCG ACAAGATCCA GCTGCTGCGC
P889645 769 AAGCGAACCC CTTCTACGAC CGCTACCGgG ACAAGATCCA GCAGCTGCGC
P889646 775 AAactAACCC TTTCTACGAC CGCTACCGCG ACAAGATCCA GCAGCTGCGC
            ********** ********** ********** ********** **********
            ********** ********** ********** ********** **********
            **   ***** ********** ********** ********** **********
            **   ***** ********** ********** ********** **********
            **   ***** ********** ******** * ********** **********
                 ***** ********** ******** * ********** **********
                 ***** ********** ******** * ********** **********
                                           * ********** ** *******
```

```
                                                     * ********** **
                                                     * ********** **

alignment position  901.......  911.......  921.......  931.......  941.......

P889644  807 AGGTGCGGGC CTGGGTTGGC TGGAGGCGGG caggaggcgg gAGGAGGGGC
P889645  819 AGGTGCGGTC CTGGTCGGG- TGGGGGTGGG GACTC----- -ACGACCGGC
P889646  825 AGGTGCGGTC CTGGCCGGGT TGGGGGTGGG GACTC----- -GTGGCGTGC
             ********** ********** ********** *****       *********
             ********** ********** ********** *****       *********
             ********** *********  ********** *****       *********
             ********** *********  **********
             ********** *********
             ********** ****
             ********** ****
             ********** ****

alignment position  951.......  961.......  971.......  981.......  991.......

P889644  857 GGCCCGGGCT GCTGGGCCTT AGGACAGCCC TGTGAGGTgg gcaCTAGTAC
P889645  862 GGGCAGAGCT TCCGGcATGC ACTACTCTCC GGGACGGTGG G--CTTGCCC
P889646  869 GGGCAGAGCT TCCGGAATGC GCTACTGTCC TGCATGGTGG G--CTGGTAC
             ********** ********** ********** ********** *  *******
             ********** *****                               *******
             ********** *****

alignment position  1001......  1011......  1021......  1031......  1041......

P889644  907 TGTCCCAGCT CTTCAGATGA TGTag--CCA GGCTCCGCGA GGCTAGGTCA
P889645  910 TGGCCCTGCT ATACCAATAG TTTGTTTCCA AGTTCAGAGA GGCCAGGCCA
P889646  917 TGTCCCcaCT ATACCAATAG TTTGTTTCCA AGTTCAGAGA GACGAGGTCA
             ********** ********** ********** ********** **********
             ******   ** ********** ********** ********** **********
                      ** ********** ********** ********** **********
                      ** ********** ********** ********** *   ******
                                          *** ********** *   ******
                                          *** ********** *

alignment position  1051......  1061......  1071......  1081......  1091......

P889644  955 CTTGCCCAAG ATCACACagc taattggtgt cagagctggg agtctct---
P889645  960 CTTGCCTAAA GTCACATCTC TGCAGACTCT TTGGgtcttt ccacagtcgt
P889646  967 CTTGCCTAAG GTCACACCTC TGCAGAACCT TTGGa----- ----------
             ********** ********** ********** ****
             ********** ********** ********** ****
             ********** ********** ********** ****
             ********** *******
             ********** ******

alignment position  1101......  1111......  1121......  1131......  1141......

P889644 1002 ---------- ---------- ---------- ---------- ----------
P889645 1010 caaaacactc tttaaagccc tatgaagaag acagctcaga gaaagttgtc
P889646 1002 ---------- ---------- ---------- ---------- ----------

alignment position  1151......  1161......  1171......  1181......  1191......
```

```
P889644 1002 ---------- ---------- ---------- ---------- ----------
P889645 1060 ccctacttag gaagatgaaa atgaagacct tcagcgaagg catttgattt
P889646 1002 ---------- ---------- ---------- ---------- ----------

alignment
position     1201...... 1211...... 1221...... 1231...... 1241......
P889644 1002 ---------- ---------- ---------- ---------- ----------
P889645 1110 gctcaaggtc acatgttgag tcagcagata cctgcctgag agatcaagtt
P889646 1002 ---------- ---------- ---------- ---------- ----------

alignment
position     1251...... 1261...... 1271...... 1281...... 1291......
P889644 1002 ---------- ---------- ---------- ---------- ----------
P889645 1160 cacaggctct cccagcttag ggccctgact gcctggacaa ggagactggg
P889646 1002 ---------- ---------- ---------- ---------- ----------

alignment
position     1301...... 1311.....
P889644 1002 ---------- ---------
P889645 1210 attgtggcaa agactgata
P889646 1002 ---------- ---------
```

Pairwise similarities:

For each pairwise alignment, the similarity (relative to the maximum similarity) and the number of identical nucleic acids (in % of shorter sequence) is given. Maximum values are underlined.

	P889645 (1228 bp)	P889646 (1001 bp)
P889644 (1001 bp)	0.340<br>51 %	0.344<br>58 %
P889645 (1228 bp)		1.000<br>89 %

Please note that the **similarity value 1.000** marks only the two **most similar** sequences, it does not necessarily mean that these sequences are identical.

Extract alignment region	
Positions in alignment	from: to:
Output file	dialign.seq

Extract aligned sequences

For comments, questions or bug reports, please contact support@genomatix.de

© Genomatix Software GmbH 1998-2005 License Agreement GEMS Launcher 4.1.1

- All rights reserved.
